# Polycomb repressive complex 1 controls uterine decidualization

**DOI:** 10.1038/srep26061

**Published:** 2016-05-16

**Authors:** Fenghua Bian, Fei Gao, Andrey V. Kartashov, Anil G. Jegga, Artem Barski, Sanjoy K. Das

**Affiliations:** 1 Division of Reproductive Sciences, Cincinnati Children’s Hospital Medical Center, Cincinnati, OH 45229, USA; 2 Department of Pediatrics, University of Cincinnati College of Medicine, Cincinnati, OH 49267, USA; 3 Perinatal Institute, Cincinnati Children’s Hospital Medical Center, Cincinnati, OH 45229, USA; 4 Division of Allergy and Immunology, Cincinnati Children’s Hospital Medical Center, Cincinnati, OH 45229, USA; 5 Division of Human Genetics, Cincinnati Children’s Hospital Medical Center, Cincinnati, OH 45229, USA; 6 Biomedical Informatics, Cincinnati Children’s Hospital Medical Center, Cincinnati, OH 45229, USA

## Abstract

Uterine stromal cell decidualization is an essential part of the reproductive process. Decidual tissue development requires a highly regulated control of the extracellular tissue remodeling; however the mechanism of this regulation remains unknown. Through systematic expression studies, we detected that Cbx4/2, Rybp, and Ring1B [components of polycomb repressive complex 1 (PRC1)] are predominantly utilized in antimesometrial decidualization with polyploidy. Immunofluorescence analyses revealed that PRC1 members are co-localized with its functional histone modifier H2AK119ub1 (mono ubiquitination of histone-H2A at lysine-119) in polyploid cell. A potent small-molecule inhibitor of Ring1A/B E3-ubiquitin ligase or siRNA-mediated suppression of *Cbx4* caused inhibition of H2AK119ub1, in conjunction with perturbation of decidualization and polyploidy development, suggesting a role for Cbx4/Ring1B-containing PRC1 in these processes. Analyses of genetic signatures by RNA-seq studies showed that the inhibition of PRC1 function affects 238 genes (154 up and 84 down) during decidualization. Functional enrichment analyses identified that about 38% genes primarily involved in extracellular processes are specifically targeted by PRC1. Furthermore, ~15% of upregulated genes exhibited a significant overlap with the upregulated *Bmp2* null-induced genes in mice. Overall, Cbx4/Ring1B-containing PRC1 controls decidualization via regulation of extracellular gene remodeling functions and sheds new insights into underlying molecular mechanism(s) through transcriptional repression regulation.

Uterine stromal cells transform into morphologically and functionally distinct cells called decidual cells (decidualization). This event occurs in women during the secretory phase of the menstrual cycle as well as in pregnancy; in rodents, this process only occurs during pregnancy. The onset of decidualization following embryo implantation is essential for successful pregnancy^1^,^2^. A similar, although not identical, pattern of decidualization can be initiated by the application of artificial stimuli to a receptive pseudo-pregnant uterus, or one that has been appropriately primed by ovarian steroids^2^,^3^,^4^. Decidual polyploidization is a hallmark of terminally differentiated cells and has been well characterized in rodents^5^,^6^,^7^,^8^,^9^,^10^ and recently recognized in humans [Hirota Y and Dey SK (unpublished observations)]. These cells undergo an endoreduplication cycle to develop into giant mono- or bi-nuclear cells with multiple copies of chromosomes^5^,^6^,^7^,^8^,^9^,^10^ and possess increased mitochondrial activity^7^. The loss of decidual polyploidy in association with pregnancy failure by mid-gestation has been reported in *Dedd* null mice^11^, implicating polyploidy development is crucially involved with decidualization during the progression of early embryo implantation.

Polycomb group genes have been originally identified as developmental regulators of body segmentation through Hox gene repression in *Drosophila melanogaster*^12^,^13^; they are displayed in many biological processes in mammals, including stem cell maintenance and differentiation^14^,^15^, X-chromosome inactivation^16^, and control of cell fate, tissue development, and cancer^17^. Two polycomb group complexes, polycomb repressive complex- 1 (PRC1) and −2 (PRC2), predominantly control chromatin compaction^18^,^19^ and catalyze two post-translational histone modifications: H2AK119ub1 and H3K27me3 (tri-methylation of histone H3 at lysine-27) for gene transcription silencing, respectively^15^,^20^,^21^,^22^,^23^. The canonical silencing mechanism is mediated by H3K27me3, which recruits PRC1 on the chromatin via chromodomain containing protein (CBX) for repression function during cellular proliferation and differentiation events^15^,^20^. PRC1 consists of at least five protein families, including CBX (Cbx2/4/6/7/8), RING1/2 (Ring1A/B), PCGF (Bmi1, Mel18, and PCGF1/6), PHC (Phc1/2/3), and RYBP/YAF2^24^,^25^,^26^. The core RING1A/B proteins play a critical role to mark H2AK119ub1 via their E3 ubiquitin-protein ligase activity^16^,^22^.

Previously, genetic studies have provided evidence that the canonical PRC1 is essential for transcriptional repression^27^, and genome-wide recruitment analysis also revealed that PRC1 localizes to target promoters of developmental regulators^28^,^29^. Further studies provided evidence to suggest that PRC1 can directly regulate gene expression during cellular proliferation and differentiation, cell-fate determination, and tissue development^14^,^15^,^17^. Because these changes are required for successful progression of early pregnancy, our objective was to investigate the roles of PRC1 during the early embryonic-attachment and decidualization phases in mice. In this study, we observed that several PRC1 members (Cbx4, Cbx2, Ring1B, and Rybp) and related histone modifications (H2AK119ub1 and H3K27me3) were significantly upregulated during decidualization with polyploidy development. Based on small molecular inhibition of PRC1 or molecular suppression of *Cbx4*, we revealed perturbation of decidualization with reduced polyploidy development and pregnancy failure. Utilizing the RNA-seq studies following inhibition of PRC1, we identified PRC1 dependent genes that primarily implicate extracellular regulatory functions during decidual development. Furthermore, we revealed that 24 up genes after PRC1 inhibition were significantly overlapped with defective decidualization upregulated genes in Bmp2 knock-out mice, suggesting certain PRC1 suppressed genes are also controlled by BMP2-mediated downregulation during decidualization. Overall, PRC1 is a critical regulator of decidualization and polyploidy development through transcriptional repression regulation of extracellular genes.

## Results

### PRC1 members are differentially and spatiotemporally expressed in the periimplantation uteri

**Figure 1:**
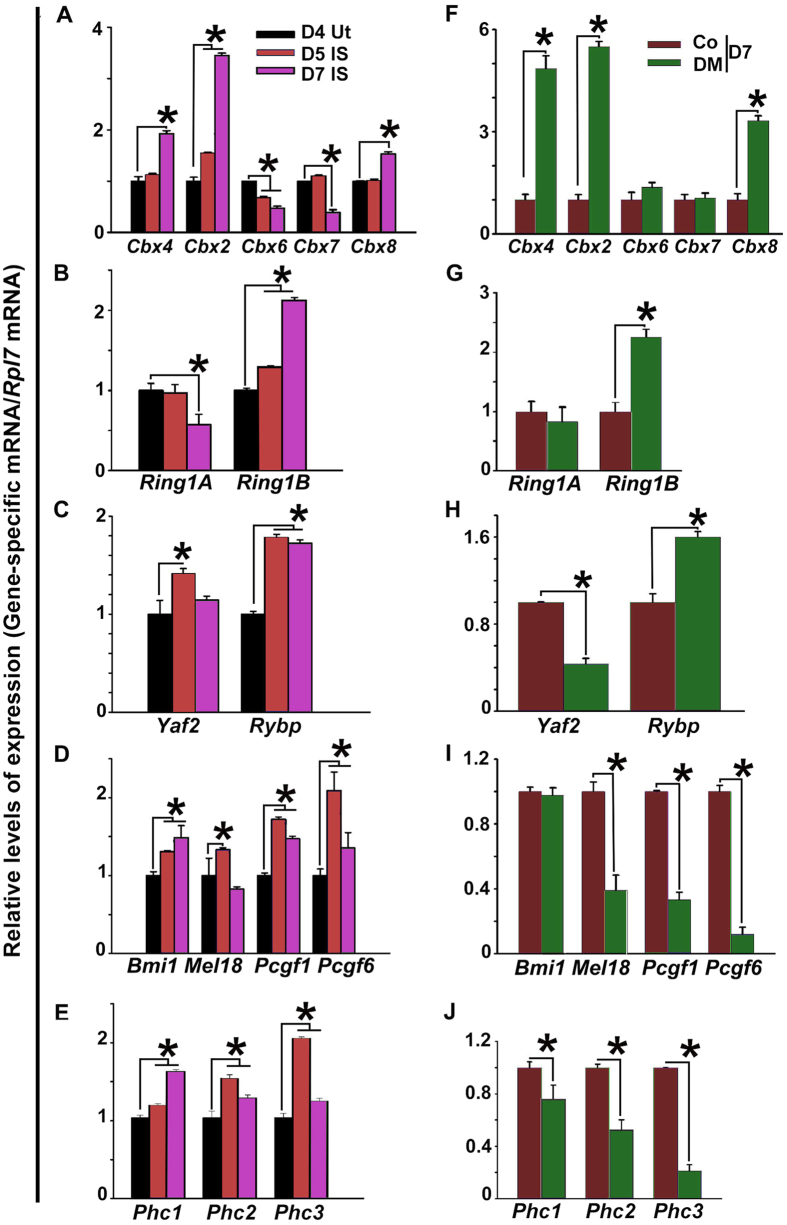
Analysis of uterine gene expression for five family members of PRC1 in early pregnancy. Quantitative RT-PCR: Total RNAs were extracted from uterine tissues collected during early pregnancy on D4 and IS on D5 and D7 (**A**–**E**), as well as, for a comparison, from vehicle-treated (control) and oil-induced uterine horns during experimental decidualization on D7 pseudopregnancy (**F**–**J**), and then subjected to real time RT-qPCR. The relative target gene expression was quantified after normalization against *Rpl7* mRNA levels. Ut, uterus; IS, implantation site; Co, control; DM, deciduoma. The error bars represent means ± SEM from three independent experiments. *Values are statistically different (P < 0.05).

In order to assess the role of PRC1 regulators in early pregnancy, we first analyzed expression of mRNAs for PRC1 members in the periimplantation uteri by RT-qPCR. Compared to day 4 levels only *Cbx2* or *Cbx6* was up or downregulated respectively at the implantation site (IS) on D5, whereas on D7-IS, *Cbx2, Cbx4,* and *Cbx8* were upregulated, but *Cbx6* and *Cbx7* were downregulated (Fig. 1A). In the case of RING1/2 family, only *Ring1B* was upregulated at the IS on both D5 and D7, while *Ring1A* was suppressed at D7-IS (Fig. 1B). For RYBP/YAF2 family members, both *Rybp* and *Yaf2* were upregulated at D5-IS, while only *Rybp* exhibited upregulation at D7-IS (Fig. 1C). In contrast, all members of PCGF and PHC, with an exception for *Mel18* on D7, were induced at the IS on both D5 and D7 (Fig. 1D,E, respectively).

These genes were also differentially regulated during experimental decidualization. For example, Cbx members, *Cbx2, Cbx4*, and *Cbx8*, but not *Cbx6* and *Cbx7*, were induced in the deciduoma (DM) as compared to the control (Co) (Fig. 1F). For RING1/2 and RYBP/YAF2 members, *Ring1B* and *Rybp* were induced, but *Yaf2* was downregulated in the DM as compared to Co (Fig. 1G,H, respectively). In contrast with PCGF and PHC members, all except *Bmi1* were downregulated in the DM as compared to Co (Fig. 1I,J, respectively).

**Figure 2:**
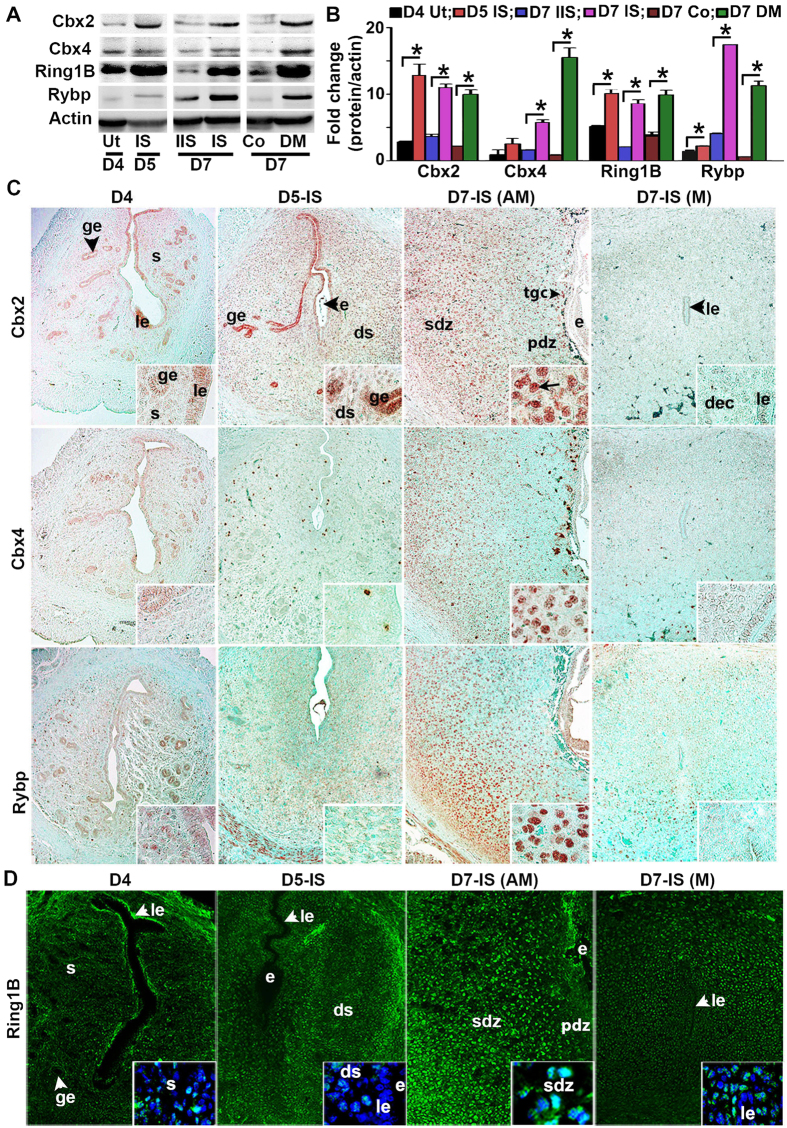
Analysis of uterine expression for Cbx2, Cbx4, Ring1B, and Rybp during early pregnancy at protein levels. (**A**) Western blot analysis. Total protein extracts of uterine tissues were collected during early pregnancy on D4, D5-IS, D7-IIS, and D7-IS. Experimentally induced deciduoma and control horns were also analyzed on D7 pseudopregnancy. Ut, uterus; IS, implantation site; IIS, interimplantation site; Co, control; DM, deciduoma. (**B**) Quantitation was achieved by direct analysis of the bands from (**A**). Fold changes in protein levels were normalized by Actin. *Values are statistically different (P < 0.05). (**C**) IHC localization of Cbx2, Cbx4, and Rybp expression. Representative uterine tissue sections on D4, D5-IS, and D7-IS in the M- and AM-pole locations are shown. Sections are shown at 100X and 400X (as insets). Red staining indicates the localization of immunoreaction. le, luminal epithelium; ge, glandular epithelium, s, stroma; ds, decidualizing stroma; pdz, primary decidual zone; sdz, secondary decidual zone; dec, decidual cells; e, embryo; tgc, trophoblast giant cells; M, mesometrial pole; AM, antimesometrial pole. Arrow indicates polyploid cells. (**D**) IF analysis of Ring1B. Blue and green staining indicates the localization of DAPI (for nuclear) and Ring1B, respectively. Sections are shown at 100X and 400X (as insets). These experiments were repeated at least three times with similar results.

Analysis of protein expression for implantation-induced PRC1 members (Cbx2, Cbx4, Ring1B, and Rybp) by western blotting revealed that these members were specifically induced at the IS on D5 and D7, as well as in DM on D7, with an exception for Cbx4 on D5-IS, when compared to corresponding controls (Fig. 2A,B). Next, spatiotemporal expression, was analyzed by immunohistochemistry (IHC) or immunofluorescence (IF) studies, as described in “Materials and Methods”. Cbx2, Cbx4, Rybp, and Ring1B were detected in luminal and glandular epithelial cell nuclei on D4, although a weak nuclear signal was also noted primarily in sub-luminal stromal cells (Fig. 2C,D). However, following the onset of implantation on D5, the expression of Cbx2 (Fig. 2C) and Ring1B (Fig. 2D) was observed throughout the endometrial stromal cell nuclei, while a strong expression for Cbx2 was also present in the luminal and glandular epithelial cell nuclei. In contrast, Cbx4 and Rybp staining did not reveal much expression in the endometrial cells, although a sub-set of stromal or muscle cells expressed Cbx4 or Rybp, respectively (Fig. 2C). On this day, embryos were also positive for Cbx2 and Rybp (Fig. 2C). However, at the IS on D7 (Fig. 2C,D), the expression for all these members was predominantly detected in the secondary decidual zone (sdz) cells with polyploidy, but primarily downregulated in the primary decidual zone (pdz). In the mesometrial (M) decidual bed, Cbx2, Cbx4, and Rybp were mostly undetected, although Ring1B exhibited weak signals (Fig. 2C,D). On this day, trophoblast giant cells and the embryo proper also had expression for all of the above members (Fig. 2C,D). In contrast, at the inter-implantation sites (IIS) on D7, these regulators were mostly absent with an exception for Rybp, which exhibited somewhat weak staining in epithelial and sub-epithelial stromal cells (Fig. S1A). No staining was noted in the antimesometrial (AM) location when D7-IS sections were incubated with normal rabbit or mouse IgG as controls (Fig. S1B). Overall, these results suggest that PRC1 members are differentially and spatiotemporally controlled during early pregnancy, with a striking up-regulation in polyploid decidual cells.

### H2AK119ub1 and H3K27me3 are colocalized with Cbx4/2 and Ring1B in the polyploid decidual cells

**Figure 3:**
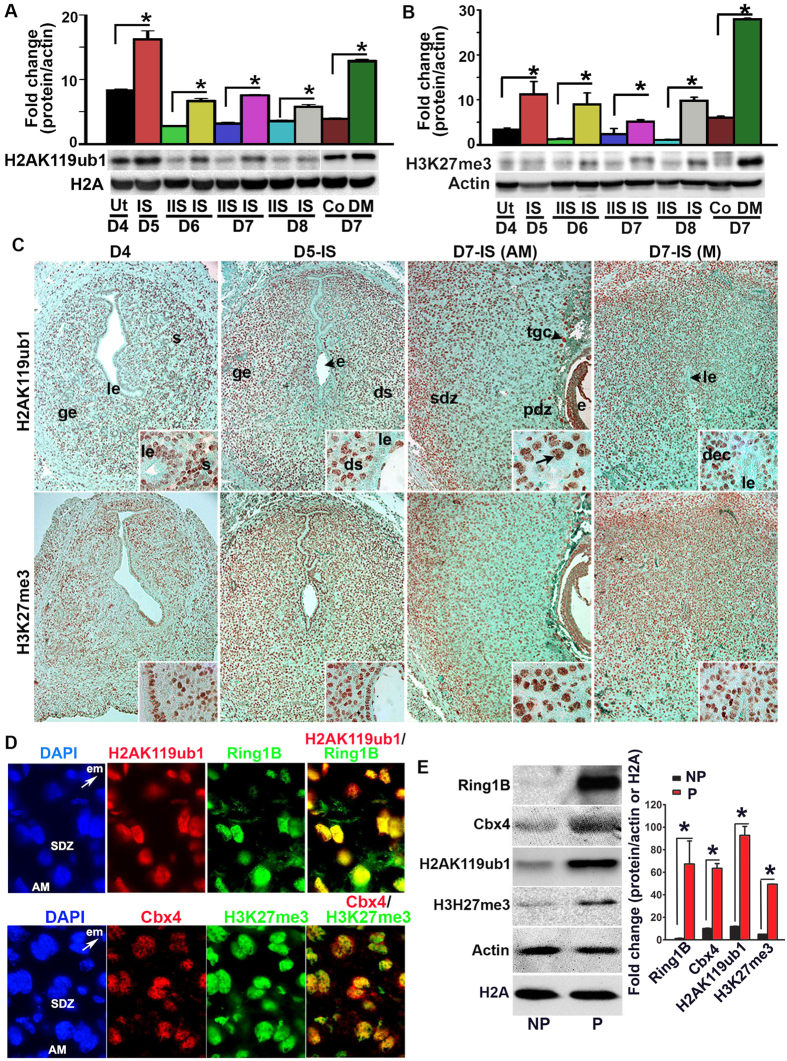
Analysis of uterine expression for H2AK119ub1 and H3K27me3 during early pregnancy. (**A**) Western blot analysis. Total protein extracts of uterine tissues were collected during early pregnancy on D4, D5-IS, and D6-8 IS and IIS. Experimentally induced deciduoma and control horns were also analyzed on D7 pseudopregnancy. Ut, uterus; IS, implantation site; IIS, interimplantation site; Co, control; DM, deciduoma. (**B**) Quantitation was achieved by direct analysis of the bands from (**A**). Fold changes were normalized by H2A. *Values are statistically different (P < 0.05). (**C**) IHC localization of H2AK119ub1 and H3K27me3. Representative uterine tissue sections on D4, D5-IS, and D7-IS in the M- and AM-pole locations are shown. Sections are shown at 100X and 400X (as insets). Red staining indicates the localization of immunoreaction. le, luminal epithelium; ge, glandular epithelium, s, stroma; ds, decidualizing stroma; pdz, primary decidual zone; sdz, secondary decidual zone; dec, decidual cells; e, embryo; tgc, trophoblast giant cells; M, mesometrial pole; AM, antimesometrial pole. Arrow indicates polyploid cells. (**D**) IF analysis of co-localized expression of H2AK119ub1 or Cbx4 together with Ring1B or H3K27me3 on D7-IS, respectively. Red (H2AK119ub1 or Cbx4) and green (Ring1B or H3K27me3) staining indicate immuno-localization of proteins. DAPI was used as nuclear stain. Arrow indicates direction of embryo (em) location. Pictures are shown at 400X. (**E**) Western blot analysis of Ring1B, Cbx4, H2AK119ub1, H3K27me3, Actin, H2A for non-polyploid (NP) vs. polyploid (P) decidual cells during experimental decidualization on D7. Quantitation (fold change) of Ring1B, Cbx4, and H3K27me3 was achieved by direct analysis of the bands against Actin, while that of H2AK119ub1 was done against H2A. *Values are statistically different (P < 0.05). These experiments were repeated at least three times with similar results.

Because functional activation of PRC1 leads to monoubiquitination of H2A on K119 and because this modification depends on H3K27me3 for canonical repressive mechanism^15^,^20^, we next measured uterine levels of H2AK119ub1 and H3K27me3 during early pregnancy. Based on western blot analyses, we detected both H2AK119ub1 and H3K27me3 on D4 (Fig. 3A,B). However, following implantation on D5, both were induced at the IS, and this up-regulation was maintained through D6-8 IS rather than their corresponding IISs (Fig. 3A,B). The analysis of deciduoma tissues revealed that both marks were upregulated as compared to control (Fig. 3A,B). We next analyzed cell specific expression of H2AK119ub1 and H3K27me3 by IHC studies. On D4, both were detected in all uterine cell types, although with a scattered distribution in muscle cells (Fig. 3C). This pattern of expression was somewhat maintained at D5-IS, although H2AK119ub1 was mostly undetected in epithelial cells (Fig. 3C). However, on D7-IS, both were distributed in sdz with polyploid cells, but the signal was undetected in pdz (Fig. 3C). In the M-pole, both were detected in decidual cells, but exhibited low or undetected expression in luminal epithelial cells (Fig. 3C). Trophoblast giant cells (tgc) and the embryo proper also expressed both on this day (Fig. 3C). At the D7-IIS, detectable levels were observed primarily in epithelial and stromal cells (Fig. S2A), yet no signals were revealed when D7-IS sections were incubated with normal mouse IgG as a control (Fig. S2B). We next examined the functional relationship between H2AK119ub1 and H3K27me3, in conjunction with PRC1 members, by dual IF studies at the D7-IS. Our analyses revealed a co-localization of H2AK119ub1 either with H3K27me3 (Fig. S3) or Ring1B (Fig. 3D), as well as co-localization of Cbx4 (Fig. 3D) or Cbx2 (Fig. S3) together with H3K27me3 in polyploid decidual cells. The expression of Ring1B, Cbx4, H2AK119ub1, and H3K27me3 was induced in isolated polyploid, as compared to non-polyploid decidual cells following an experimental decidualization on D7 (Fig. 3E). Overall, these results suggest that PRC1-dependent activation mark H2AK119ub1 is spatiotemporally and coordinately controlled with H3K27me3 in periimplantation uteri with particular emphasis in polyploid decidual cells.

### Inhibition of PRC1 minimally affects embryo-uterine attachment during early pregnancy

To examine the importance of the PRC1 function in early pregnancy, we applied a small-molecule inhibitor (PRT4165) of PRC1 in initial implantation events, specifically during the progression of embryo-uterine attachment and uterine decidualization. PRT4165, a known and potent inhibitor of H2AK119ub1, also inhibits Ring1A/B-containing PRC1 activity^30^. We first introduced the inhibitor during the preimplantation period on D3 and D4 (*schedule A*), as described in the “Materials and Methods”, to examine the embryo-uterine attachment reaction, monitored on D5 by blue-dye injection method^31^. Our analyses revealed that there were no significant alterations in the number of embryo attachment reactions and the weights of IS between the vehicle (control) or PRT4165 (4 or 8 mg/kg) treated groups (Fig. S4A,B). The analyses of IS morphology, proliferation status, and expression of implantation markers revealed no significant difference between the drug- and control-treated groups (Fig. S4C–F). Mice treated with the drug or control did not show any alteration in the number of pups. However, our analysis of H2AK119ub1 expression at the IS revealed its significant downregulation by PRT4165 as compared to control, suggesting PRC1 activity was indeed affected by the drug treatment (Fig. S4G). Overall, these results suggest that PRC1 plays a minimal role in early embryo-uterine attachment during implantation.

### Inhibition of PRC1 causes disruption of decidualization and polyploidy development during early pregnancy

**Figure 4:**
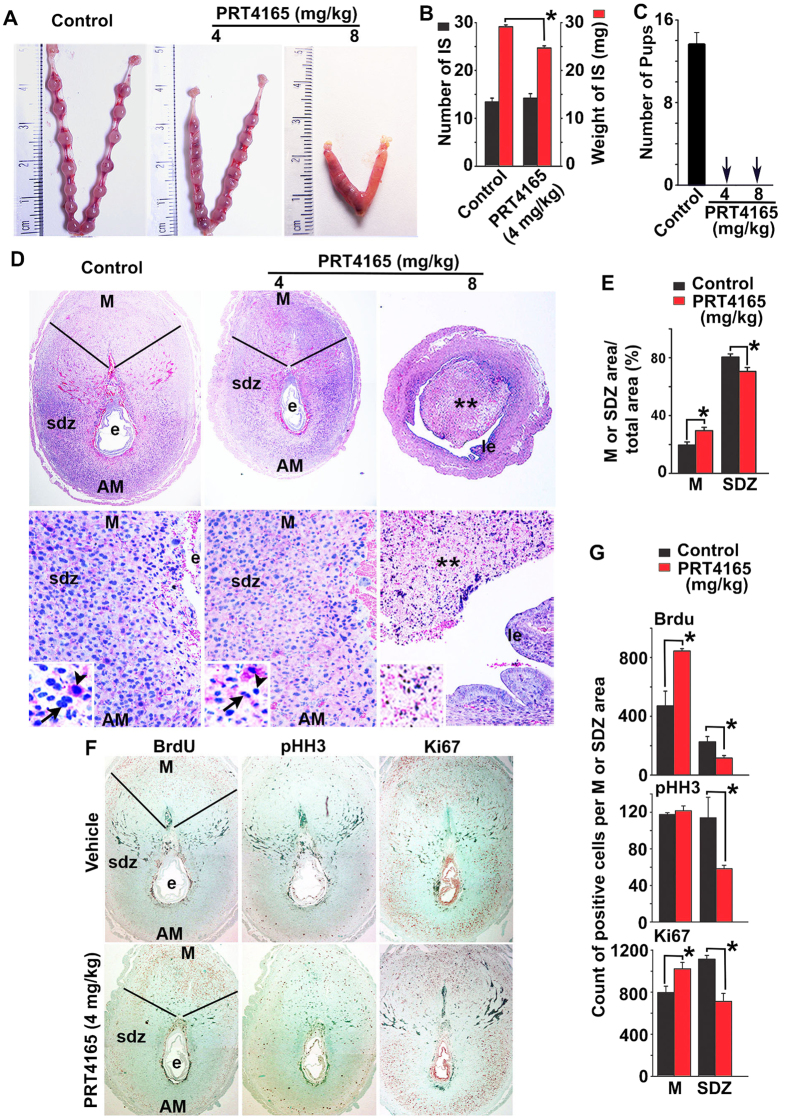
Inhibition of PRC1 affects decidual development in implantation. Mice were injected with PRC1 inhibitor (PRT4165 at 4 mg/kg and 8 mg/kg) or vehicle (control) on D5-D7 and analyzed on D8 (09:00 h) as described in Materials and Methods. (**A**) Representative photographs of uterine implantation sites are shown. (**B**) Analysis of the number or weight of IS reveals significant difference in weight without affecting the number. (**C**) Loss of pregnancy outcome after injection of PRT4165, as compared to control. (**D**) Histological analysis of IS after staining with hematoxylin and eosin. Representative pictures shown at 40X (upper panels), 100X (lower panels) and at 400X (as inset in lower panels). M, mesometrial pole; AM, anti-mesometrial pole; e, embryo; sdz, secondary decidual zone; le, luminal epithelium. Arrows and arrowheads represent mono- or bi-nucleated polyploid cells. Degenerated decidual tissue mass is shown by **. (**E**) Comparison of developmental area for M and sdz locations between PRT4165- and control-treated mice. *Significantly different (p < 0.05) for corresponding M or sdz locations between the groups. (**F**) IHC analysis of IS by BrdU, pHH3, and Ki67 between PRT4165- and control-treated mice. (**G**) Quantitative analyses of BrdU, pHH3, and Ki67 positively stained cells per area for M and sdz locations between the groups. *Significantly different (p < 0.05) for corresponding M or sdz locations between the groups. These experiments were repeated at least three times with similar results.

To analyze the role of PRC1 during the post-implantation period, we administered PRT4165 in mice during decidual progression on D5-7 (*schedule B*), as described in the “Materials and Methods”. Our analyses of implantation progression on D8-IS revealed a significant reduction in weight, but not in number, following addition of the drug at 4mg/kg, as compared to the control (Fig. 4A,B). In contrast, at a higher dose (8 mg/kg) of drug treatment, the inhibitory effect dramatically impacted the progression of decidualization (Fig. 4A); the presence of tissue debris (shown by **) within the lumen and the appearance of newly formed epithelial cell layer surrounding the lumen (Fig. 4D) suggests that a rapid regeneration of the endometrium follows the loss of decidualization. The drug treatment at both doses resulted in a complete loss of pups, as compared to the control (n = 6 for each group) (Fig. 4C), suggesting that PRC1 function during decidualization is critical to supporting successful pregnancy. The histological analyses of D8-IS revealed that compared to the control, the drug treatment at 4 mg/kg resulted in significant disruption of regional decidualization; the decidual bed in M-pole was bigger with a concomitant decrease in sdz (lateral and AM locations) (Fig. 4D,E). The above defects were not associated with any disturbances in ovarian E2 and P4 levels in serum or ovarian corpora luteal structures (Fig. S5A,B, respectively).

**Figure 5:**
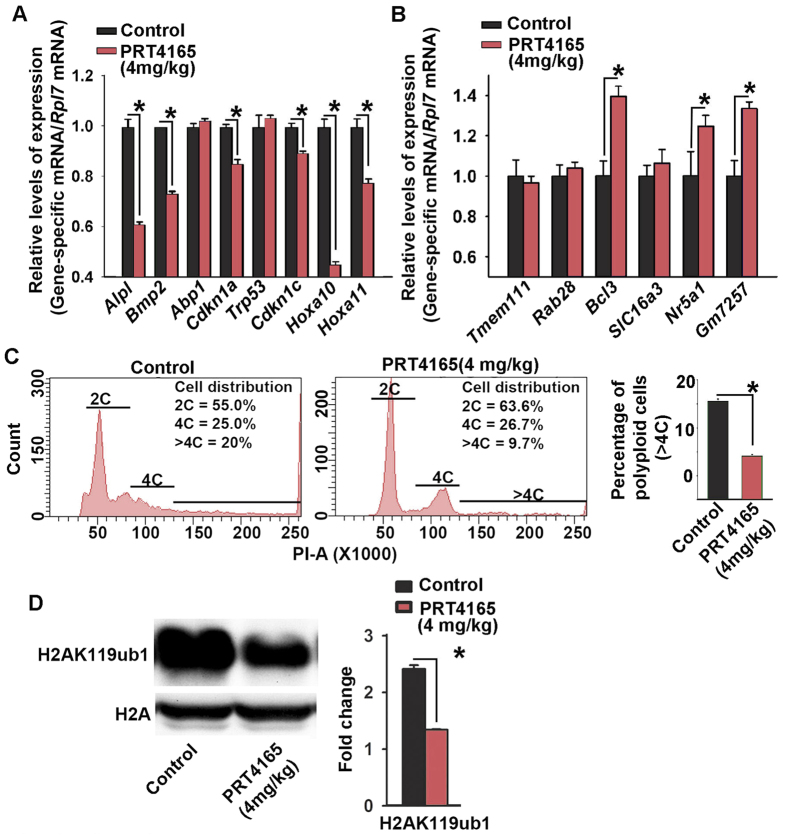
Inhibition of PRC1 causes alteration of gene regulation and polyploidy development during decidualization *in vivo*. Quantitative RT-PCR analyses of active genes (*Alpl, Bmp2, Abp1, Cdkn1a, Trp53, Cdkn1c, Hoxa10,* and *Hoxa11*) (**A**) and repressed genes (*Tmem111, Rab28, Bcl3, SlC16a3, Nr5a1,* and *Gm7257*) (**B**) during decidualization at the D8-IS after inhibition of PRC1 against control. (**C**) Flow cytometric analysis. Quantitative analyses of cell distribution (%) based on the DNA content are shown as insets for drug- or vehicle-treated groups. Quantitative analysis of polyploid cell count (%) without or with inhibition of PRC1 during decidualization. The error bars represent means ± SEM from three independent experiments. *Values are statistically different (P < 0.05). (**D**) Western-blot analysis reveals inhibition of PRC1, as judged by downregulation of H2AK119ub1, after addition of PRT4165 against control. Quantitation of H2AK119ub1 expression was achieved by direct analysis of the bands. Fold change was determined after normalization with H2A. *Values are statistically different (P < 0.05). These experiments were repeated at least three times with similar results.

Since successful decidualization requires coordinated progression of stromal cell proliferation and differentiation at the site of implantation, we first analyzed the aspects of proliferation at D8-IS, after the drug treatment at 4 mg/kg, as compared to the control. Both BrdU (S-phase marker) and Ki67 (G1-S-G2-M phase marker) positive cells were significantly increased in the M-pole region but decreased in sdz (Fig. 4F,G), compared to the control, while pHH3 (M-phase marker) positive cells downregulated in sdz, but remained the same in the M-pole region, suggesting an aberrant cell cycle progression during decidualization after PRC1 inhibition. We next analyzed markers of differentiation for stromal cell decidualization (*Alpl, Bmp2, Abp1, Cdkn1a, Trp53, Cdkn1c, Hoxa10,* and *Hoxa11*)^3^,^32^,^33^. Our results revealed that the presence of the drug was inhibitory for all except *Abp1* and *Trp53*, as compared to the control (Fig. 5A). However, when we analyzed regulation of repressed genes (*Tmem111, Rab28, Bcl3, SlC16a3, Nr5a1,* and *Gm7257*)^34^,^35^, we noted that *Bcl3, Nr5a1*, and *Gm7257* were specifically de-repressed after the drug treatment compared to the control (Fig. 5B). Analysis of polyploidy development revealed that the drug was able to suppress decidual polyploidy development (Fig. 5C). Western blot analysis demonstrated downregulation of H2AK119ub1 by PRT4165 as compared to the control, suggesting that the drug effectively inhibited PRC1 repressive function (Fig. 5D).

Next, to determine whether the above drug-mediated inhibitory effects on decidualization occur without involving an embryo, we examined effects of the inhibitor at 4 or 8 mg/kg during *in vivo* experimental decidualization, as described in the “Materials and Methods”. Our results showed that the drug treatment causes inhibition of deciduoma formation and polyploidy development, as judged by tissue morphology, weights, and nuclear staining intensity of polyploid cells, when compared with the control (Fig. S6A–C). Collectively, the above results suggest that PRC1 plays a major role in successful progression of decidualization and polyploidy development, through a strict regulation of gene expression.

### PRC1 controls stromal cell decidualization and polyploidy development *in vitro*

To examine the role of PRC1 during decidualization, the inhibitor PRT4165 was also used during stromal cells decidualization *in vitro*. Based on flow cytometric analyses of DNA content, we again found that polyploidization was significantly inhibited in a dose dependent manner by the presence of the inhibitor (25 and 50 μM), as compared to vehicle control (Fig. S7A,B). Quantitative analyses of expression by RT-PCR revealed that decidualization (*Prl8a2, Alpl, and Bmp2*) and polyploidy (*Abp1* and *Tdo2*) related genes were significantly downregulated by drug treatment compared to the vehicle (Fig. S7C). These results again suggest that PRC1 plays a critical role in stromal cell decidualization and polyploidy development *in vitro*.

### Cbx4-dependent PRC1 function is involved in stromal cell decidualization and polyploidy development *in vitro*

**Figure 6:**
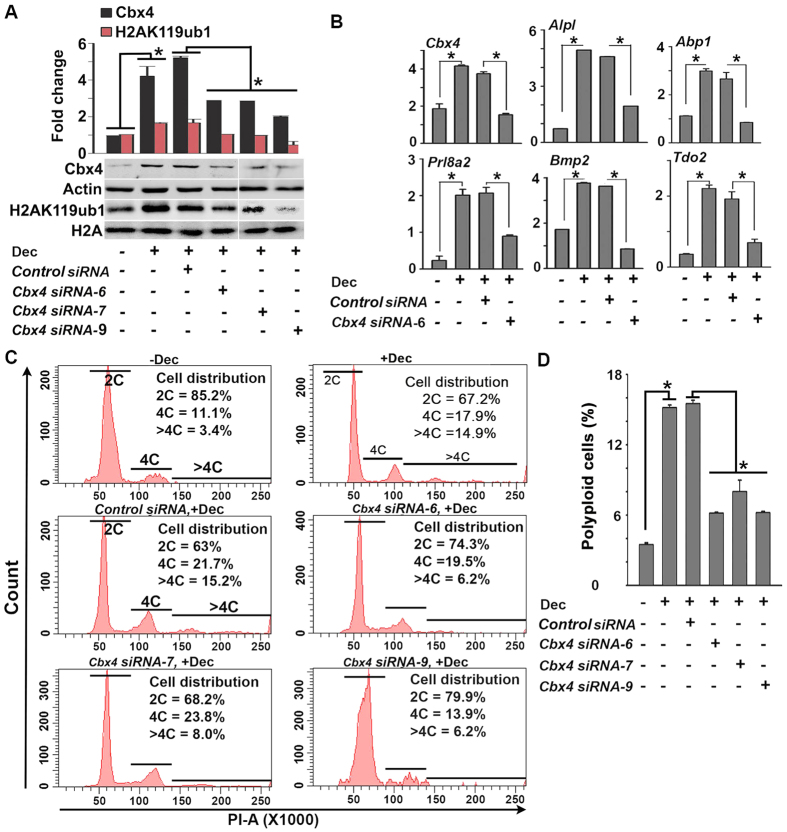
Loss of *Cbx4* by *siRNAs* causes inhibition of decidualization and polyploidization *in vitro*. Day 4 uterine stromal cells in the culture were transfected with the control *siRNA* prior to without (−) or with (+) decidualization (Dec), as described in Materials and Methods. Three independent *siRNAs* (siRNA-6, siRNA-7, and siRNA-9) for *Cbx4* were also transfected in separate cultures prior to decidualization. Cells were collected after 5 days of decidualization. (**A**) Western blot analysis. Total protein extracts were analyzed for the expression of Cbx4, H2AK119ub1, Actin, and H2A. Quantitation was achieved by direct analysis of the bands. Fold changes for Cbx4 or H2AK119ub1 were normalized by Actin or H2A, respectively. *Values are statistically different (P < 0.05). (**B**) Quantitative RT-PCR analyses of expression for *Cbx4, Prl8a2, Alpl, Bmp2, Abp1,* and *Tdo2* following suppression of *Cbx4* during decidualization. (**C**) Flow cytometric analysis. Quantitative analyses of cell distribution (%) based on the DNA content are shown as insets for each representative group. (**D**) Quantitative analysis of polyploid cell count (%) without or with inhibition of Cbx4 during decidualization. The error bars represent means ± SEM from three independent experiments. *Values are statistically different (P < 0.05).

The above regulation of PRC1 members and its associated histone modification marks in decidual polyploid cells has prompted us to examine PRC1 function in uterine stromal cell during decidualization. We first examined whether the knock-down of *Cbx4* by specific *siRNAs* affects *in vitro* decidualization. Western blotting showed downregulation of *Cbx4* expression during decidualization by three independent siRNAs as compared to control *siRNA* (Fig. 6A). This result was correlated with the inhibition of H2AK119ub1 levels, indicating perturbation of PRC1 functional activity. We next analyzed whether this PRC1 inhibitory condition affects decidualization and polyploidy development by RT-qPCR analyses of specific markers for these events. Our analysis revealed that decidualization (*Prl8a2, Alpl*, and *Bmp2*) and polyploidy (*Abp1* and *Tdo2*) related genes were significantly affected after *Cbx4* suppression as compared to control (Fig. 6B). Flow cytometric analyses of polyploidy development revealed that the above loss of *Cbx4* was also able to show significant abrogation of decidual polyploidy (Fig. 6C, D). Overall, these results suggest that PRC1 function, at least through Cbx4, is critically required for the progression of decidualization and polyploidy development *in vitro*.

### PRC1 regulates multiple molecular signaling networks during decidualization

**Figure 7:**
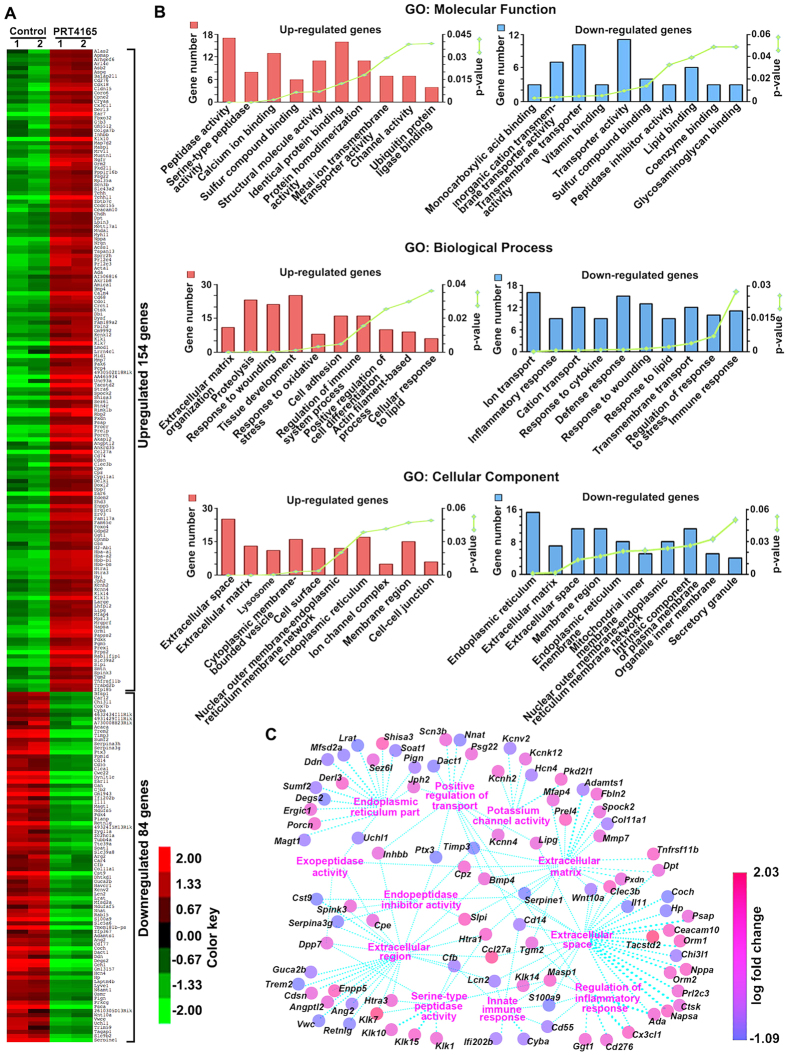
Analysis of global gene expression by RNAseq during decidualization after PRC1 inhibition in mice. (**A**) Heat map for differentially expressed genes. Mice were injected with PRT4165 (at 4 mg/kg) or vehicle (control) on D5-D7 and analyzed on D8 (09:00 h) as described in Materials and Methods. Duplicate samples were analyzed for each group. (**B**) Functional categorization of differentially expressed genes by ToppFun analysis. In each GO category, the 10 selected terms are shown with gene number and p-value. (**C**) A gene network generated by “Altanalyze” program for a total 92 (up- and down-regulated) genes associated with the selected 11 GO terms is shown.

To identify PRC1-mediated downstream pathways during decidualization, we performed RNA-seq analysis of gene expression in decidual tissues from D8-IS isolated from the animals treated with PRC1 inhibitor or control. Our analysis revealed that a total 238 genes (RPKM > 3; Fold changes > 1.5; *p* < 0.05) were differently expressed following inhibition of PRC1 during decidualization (Table S2). Among those genes, 154 genes were up-regulated and 84 genes down-regulated (Fig. 7A), which shows that a drastic alteration of gene expression results from the inhibition of PRC1 during decidualization. In order to validate the RNA-seq results, we analyzed the expressional changes by RT-qPCR for selected genes (Table S3). Consistent with our RNAseq data analysis, the expression of *Ccl27a, Klkl5, Papss2,* and *Rinklb* was upregulated, whereas that of *Coch, Cwc22, Degs2, Ear11, Lrat, Ptx3, and Wnt10a* was downregulated during decidualization following inhibition of PRC1 compared to control (Table S3).

**Figure 8:**
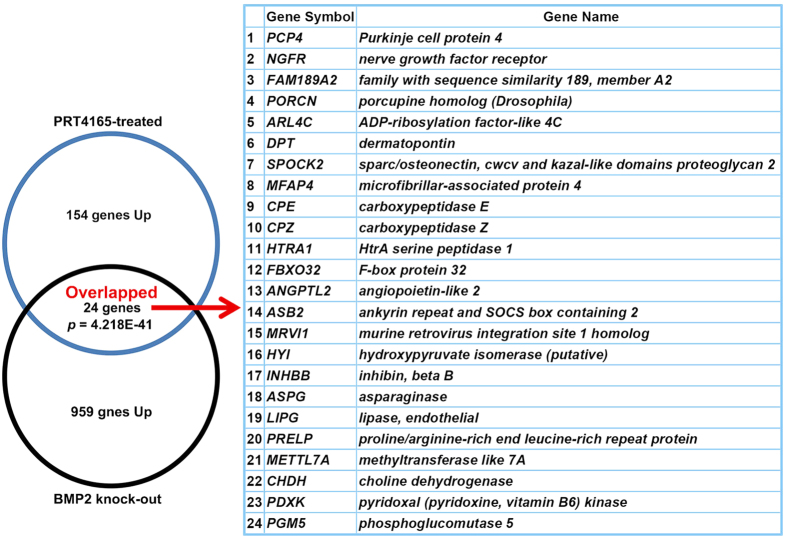
Comparison of genes upregulated by PRC1 inhibition with the genes up-regulated by *Bmp2* deletion during decidualization in mice. The Venn diagram shows the overlap between the two lists. A list of 24 overlapping genes is also shown.

To gain insights into the functions of differentially expressed genes, the functional enrichment analysis was carried out separately for the up- and down-regulated genes using the ToppFun program (Tables S4A and B, respectively). Selected over-represented Gene Ontology (GO)-terms are shown in Fig. 7B. Under GO: molecular function category, the upregulated genes were related to peptidase activity, identical protein binding, calcium ion binding, structural molecule activity, and protein dimerization activity (Fig. 7B and Table S4A), whereas downregulated genes were involved in transmembrane transporter activity, inorganic cation transmembrane transporter activity, lipid binding, and sulfur compound binding as major subcategories (Fig. 7B and Table S4B). Under GO: biological process category, the list of upregulated genes was enriched in genes involved in tissue development, proteolysis, response to wounding, cell adhesion, and regulation of immune system process (Fig. 7B and Table S4A), whereas downregulated genes were involved in ion transport, defense response, response to wounding, cation transport, and transmembrane transport (Fig. 7B and Table S4B). In regards to GO: cellular component, up-regulated genes encoded for proteins localizing to extracellular space, endoplasmic reticulum, cytoplasmic membrane-bounded vesicle, membrane region, and extracellular matrix (Fig. 7B and Table S4A), while that of down-regulated genes primarily revealed endoplasmic reticulum, extracellular space, membrane region, and intrinsic component of plasma membrane (Fig. 7B and Table S4B). These results are also summarized by the GO network in Fig. 7C that shows both up- and down-regulated genes (~38%) related to the terms “extracellular matrix”, “endopeptidase inhibitor activity”, “extracellular region”, “potassium channel activity”, “exopeptidase activity”, “serine-type peptidase activity”, “endoplasmic reticulum part”, “extracellular space”, “innate immune response”, “regulation of inflammatory response”, “positive regulation of transport”. Interestingly, our ToppFun analysis also revealed that a list of 154 upregulated genes was significantly (~15%, p = 4.218E-41) overlapped with the list of genes induced in decidualizing tissue by *Bmp2* knock-out in mice^36^ (Fig. 8, Table S4A).

Overall, these studies suggest that multiple molecular signaling networks that primarily involve endoplasmic reticulum part and extracellular functions, including matrix remodeling, peptidases activity, cell adhesion, transport/channel activity, and immune regulation genes are potentially targeted by PRC1 during decidualization.

## Discussion

Our studies provide comprehensive evidence to suggest that PRC1 plays a major role in successful progression of stromal cell decidualization, without affecting the early embryonic attachment. The highlight of the present investigation is that specific PRC1 subunits (Cbx2/4, Ring1B, and Rybp) exhibit distinct upregulation during decidualization with polyploidy. The functionally active PRC1 in polyploid decidual cells is revealed by detection of H2AK119ub1 via its co-localization together with PRC1 subunits or H3K27me3, an upstream canonical regulator of PRC1 function. Strikingly, inhibition of PRC1 function or perturbation of *Cbx4* expression resulted in decidual disruption with loss of polyploidy and pregnancy failure. Based on RNA-seq studies, we identified PRC1-dependent target genes that control multiple signaling networks primarily directed towards extracellular functions, including matrix remodeling, proteolytic function, cell adhesion, cellular transport, and immune regulation during decidual progression.

In the receptive uterus on D4 in mice, stromal cells experience proliferation, while epithelial cells follow differentiation, under the coordinated control of both ovarian estrogen and progesterone. Following the embryonic attachment, which occurs on D4 24:00 h, stromal cells at the site of implanting embryo exhibit rapid proliferation that persists until D5 morning. The first sign of stromal differentiation, formation of pdz, occurs in close proximity to the implantation chamber toward the AM-pole in the afternoon on D5^5^,^6^. PDZ is avascular and epithelioid in nature^37^; however, terminal differentiation of stroma with decidual polyploidy forms the secondary decidual zone (sdz), which occurs through continuous proliferation and differentiation next to the pdz on D6 through D8. SDZ develops both at the lateral and AM-pole locations of the decidual bed. In contrast to sdz development, stromal cells in the M-pole continue to proliferate and differentiate to form the non-polyploid decidual zone, a presumptive site for placentation.

Previously, studies based on biochemical purification or genome-wide localization have implicated that at least six various combinations of PRC1 subunits direct H2AK119ub1 marks^26^,^38^. In this regard, the presence of RYBP, in RING1A/B/PCGF-containing complex, appears to support RING1/2 toward H2AK119ub1, but also prevents incorporation of CBX/PHC in PRC1 core complex, indicating the inhibition of canonical acitivity^38^. Although the canonical activity depends on PRC1 recruitment via Cbx proteins downstream of PRC2-mediated H3K27me3 marks^15^,^20^, recent studies have also documented that H2AK119ub1 creates a binding site for Jarid2–Aebp2–containing PRC2 and promotes H3K27me3 on H2AK119ub1 nucleosomes^39^, thus suggesting a mutually interactive relationship. In contrast, studies have also shown that PRC1 can have independent functions via RYBP-containing PRC1 complex^26^,^40^,^41^. However, the specific composition and function of PRC1 variants in tissue biology remain poorly defined.

Here, we observed that a full complement of all five members of PRC1 was detected at the IS on D5 and D7, as compared to D4. The analyses of PRC1 composition, based on cell-specific expression revealed that at least Cbx2/4, Ring1B, and Rybp may be needed in uterine epithelial cells on D4. They also become predominantly localized in decidual polyploid cells at the D7-IS, suggesting that these regulators may participate not only in epithelial differentiation prior to implantation on D4, but also later in terminal differentiation for decidual polyploidy in the post-implantation uterus. The detection of H2AK119ub1 and H3K27me3 in epithelial and decidual polyploid cells suggests that the PRC1-mediated repressive function supports differentiation for these cell types. However, a weak expression in sub-luminal stroma on D4 or lack of detection in M-pole of the above PRC1 proteins on D7-IS, as opposed to a distinct wide-spread stromal/decidual expression of H2AK119ub1 and H3K27me3 on these days, suggests that these cells may utilize a different PRC1 complex for proliferation or differentiation functions, respectively. However, on D5-IS, throughout endometrial stromal expression of H2AK119ub1, H3K27me3, Cbx2, and Ring1B (with exceptions include Cbx4 and Rybp), suggests that the later members play limited roles during the initial decidual progression. The upregulation of Cbx2/4 specifically in polyploid decidual cells on D7 is interesting, since studies have shown that the cellular differentiation commitment is orchestrated by a combination of Cbx2 and Cbx4^42^. It has also been suggested that the presence of Cbx7 opposes differentiation progression^42^,^43^. Our observation of *Cbx7*’s downregulation at D7-IS may support the above notion.

The downregulation of *Phc1-3, Mel18, Pcgf1, and Pcgf6* in experimental decidualization, as opposed to their upregulation during implantation, and induced expression of H2AK119ub1 in isolated deciduomal polyploid cells, compared to that of non-polyploid cells, suggests that these PHCs and PCGFs play limited roles in PRC1-mediated functions during deciduomal polyploidization. In this regard, it is worth mentioning that differential gene regulation during implantation-induced decidualization vs. experimental decidualization has been reported^3^,^4^. However, the coordinated regulation of H3K27me3 and H2AK119ub1 in polyploid decidual or deciduomal cells suggests that canonical PRC1 silencing mechanism plays a major role during decidual polyploidization, although a different combination of PRC1 may be utilized.

To evaluate the role of PRC1 in early pregnancy, we observed that the inhibition of PRC1 was unable to show any aberration with the embryonic attachment, but instead exhibited disruption of decidual development and the failure of pregnancy. Specifically, we noted that PRC1 inhibition (at a lower dose of the drug) leads to abrogation of regional decidualization, primarily due to enhanced cell cycle activity for diploid cells in the M-pole and increased inhibition of cell cycle progression and polyploidy development in the sdz. These results indicate that the M-pole specific decidual bed (a presumptive site for placentation) may be developmentally interlinked with the sdz at the site of implantation. A similar conclusion has recently been shown by the genetic deletion of FoxM1, a regulator of cell cycle progression in mice^44^. The above decidualization defect was strongly correlated with dysregulation of expression for decidual genes (*Alp1, Bmp2, p21, p57, Hoxa10*, and *Hoxa11*)^5^,^6^,^7^,^34^,^45^,^46^,^47^, suggesting that the alteration of selective decidual genes is responsible for the disruption of normal decidual development. The inhibition of PRC1 also results in aberration with increased expression of downregulated genes (*Bcl3, Nr5a1*, and *Gm7257*)^34^,^48^ in the decidual bed, indicating PRC1-mediated regulation of both positive and negative genes is necessary for an appropriate control of uterine decidualization. It is also interesting to note that the loss of decidual growth by PRC1 inhibitor at a higher dose was rapidly restored with the epithelial cell lining surrounding the degenerated mass of decidua, suggesting that the inhibition of PRC1 is not detrimental to epithelial cell regeneration. Previously, studies have shown that decidual regression is coordinately balanced by the remarkable regeneration capacity of the endometrium^49^. In the regard, it is worth mentioning that the adult endometrial stem/progenitor cells, as likely contributors, have been identified in human and mouse endometrium^50^.

Our analysis of expression revealed that both Cbx4 and Cbx2 were detected with a similar expression pattern in the uterus during the periimplantation period, and since both can participate in the PRC1 complex, we just arbitrarily chosen *Cbx4* in our subsequent suppression studies to evaluate its role in decidualization. We specifically observed that the loss of *Cbx4* restricts decidual progression and polyploidy development, judged by the analyses of expression for markers of stromal cell differentiation (*Prl8a2, Alpl,* and *Bmp2*) and terminal differentiation for polyploidy (*Abp1* and *Tdo2*) and the developmental regulation of polyploidy by flow cytometric analysis. These results were also consistent with inhibition of H2AK119ub1, suggesting that this inhibition plays a central role in PRC1 function during successful decidualization and polyploidy development.

It has been well recognized that the decidual tissue development at the site of implantation critically controls invasion of trophoblast cells into the uterine endometrium^51^,^52^,^53^, which requires a highly regulated control of the extracellular tissue remodeling in decidual bed^54^,^55^,^56^; however the mechanism of this regulation remains unknown. We noted that the inhibition of PRC1 in the post-implantation uterus effectively altered 58 extracellular genes that are predominantly associated with including matrix remodeling, peptidases activity, cell adhesion, transport/channel activity, and immune regulation. During uterine decidualization and polyploidization, the strict control of immune gene expression has been shown^7^. Interestingly, after inhibition of PRC1 during decidualization in mice, we observed alteration of 28 immune regulatory genes. In addition, we also noted that PRC1 inhibition causes alteration of 32 genes associated with endoplasmic reticulum part. Interestingly, a network appears to suggest that many of these genes are connected to exopeptidase and transport regulatory activities, which further suggests that PRC1 regulates a program related to tissue remodeling. Overall, we believe that our identified PRC1-dependent gene networks will provide new molecular insights about the mechanism of PRC1 functions during decidualization.

Previously, studies have shown that BMP2, an exclusive marker of uterine stromal/decidual cells^33^, plays a critical role in decidualization in mice^36^ and humans^45^. Our study revealed that 24 PRT4165-dependent upregulated genes were also upregulated by BMP2 deletion during decidualization^36^, suggesting that PRC1 may be involved in suppression of these genes by BMP-dependent pathways. In this regard, it is worth mentioning that one of the genes d*ermatopontin* (*Dpt*), a regulator of extracellular matrix remodeling, shows predominant expression in the interimplantation region^57^. *Dpt* also displays aberrant up-regulation in defective decidualization either by PRC1 inhibition or loss of *Bmp2*, suggesting that PRC1 targets this gene for suppression during normal decidualization, as well as Bmp2-mediated mechanisms. The regulation of *Dpt* or other identified genes directly controlled by PRC1 during decidualization remains unknown and requires further ChIP-seq experiments.

In conclusion, we presented comprehensive evidence to suggest that uterine PRC1, uniquely with Ring1B/Cbx4-containing complex, plays a major role in the control of uterine stromal cell differentiation function, including the polyploidy development. Studies also provided new insights into underlying molecular mechanism(s) of decidualization through regulation of extracellular functions.

## Methods

### Animals, injections, and tissue collection

CD-1 mice were purchased from Charles River Laboratories (Wilmington, MA). Mice were housed in the animal care facility at Cincinnati Children’s Hospital Medical Center. All animal protocols were approved by the Institutional Animal Care and Use Committee (IACUC) (Approval number: IACUC2013-0059). The methods were carried out in accordance with the approved guidelines. Females (8–10 weeks old) were mated with fertile or vasectomized males to induce pregnancy or pseudopregnancy (D1 = vaginal plug), respectively. Uterine tissues were collected either as a whole on D4 (at 09:00 h) or after separation of IS and IIS on D5 through D8 (09:00 h). To stimulate experimentally-induced decidualization (deciduoma), sesame oil (25μl) was infused intraluminally in one uterine horn on D4 of pseudopregnancy; the contralateral horn was not injected to serve as the control^3^. Tissues were collected on D7 and D8 (09:00 h) of pseudopregnancy, flash frozen and kept at −80 °C for subsequent analysis or fixed in 10% formalin for paraffin blocks.

For studies with the inhibition of PRC1 functional activity *in vivo*, the injection of a small-molecule inhibitor PRT4165 (Millipore, Billerica, MA) in mice was followed by two schedules (A and B). In *schedule A*, PRT4165 [4 or 8 mg/kg in a 0.1 ml solution per mouse in sesame oil (Sigma, MO) containing 10% ethanol] or the vehicle (0.1 ml sesame oil per mouse containing 10% ethanol, as a control) was given twice (at 0900 and 1800 h on each day) during the pre-implantation period on D3 and D4, and the ISs were collected after using the blue dye injection method^31^ on D5 (0900 h). In *schedule B*, PRT4165 or the vehicle was injected twice (at 0900 and 1800 h on each day) at the same dosage specified in *schedule A* during the post-implantation period on D5 through D7, and the ISs were collected on D8 (0900 h). In case of experimental deciduoma formation, we also applied the vehicle (control) or PRT4165 (at 4 or 8 mg/kg) as indicated above on D5 through D7 of pseudopregnancy, and analysis was made on D8 (0900 h).

### Isolation of polyploid and non-polyploid decidual cells

Polyploid and non-polyploid decidual cells were isolated from deciduoma tissues on D7, as described^7^. Cells were stored at −80 °C until analysis.

### Antibodies

The affinity-purified rabbit polyclonal antibodies for Cbx4 (Cat# GTX109662) and Cbx2 (Cat# GTX117711) were purchased from GeneTex, (Irvine, CA). The affinity-purified rabbit polyclonal antibody for H2AK119ub1 (Cat# 4889) was purchased from Cell Signaling (Beverly, MA). The affinity-purified rabbit polyclonal antibodies for H2A (Cat# ab18255), BrdU (Cat# ab1893), and Rybp (Cat# ab5976) were purchased from Abcam Inc (Cambridge, MA). The purified mouse monoclonal antibodies for H3K27me3 (Cat# 05-1591), Ring1B (Cat# D193-3), and phosphorylated histone H3 (pHH3) (Cat#, MABE939) were purchased from Millipore Corp. (Billerica, MA). The affinity-purified rabbit polyclonal antibodies for actin (Cat#.sc-1615) and Ki67 (sc-7846) were purchased from Santa Cruz Biotechnology, Inc. (Santa Cruz, CA). Secondary antibodies for Cy2-conjugated donkey anti-rabbit (Cat# 711-225-152); Cy3-conjugated donkey anti-rabbit (Cat# 711-165-152) and donkey anti-mouse (Cat# 715-165-150); and peroxidase-conjugated goat anti-rabbit (Cat# 705-035-147) and donkey anti-goat (Cat# 705-035-147) were purchased from Jackson ImmunoResearch Laboratories, Inc. (West Grove, PA). Peroxidase-conjugated goat anti-rabbit secondary antibody (ready-to-use) (Cat# 50-235) for IHC and biotinylated antibody for BrdU (Cat# 93-3944) were obtained from Zymed Laboratories Inc. (San Francisco, CA).

### IHC and IF studies

PRC1 complex members were studied either by IHC using 10% formalin-fixed paraffin sections or by IF using frozen sections after fixation with 4% paraformaldehyde, as previously described^34^. Dilution of primary antibodies were as follows: rabbit anti-Cbx4 (1:100); rabbit anti-cbx2 (1:100); mouse anti-Ring1B (1:400); rabbit anti-Rybp (1:400); rabbit anti-H2AK119ub1 (1:1000); mouse anti-H3K27me3 (1:300); Ki67 (1:200); and sheep anti-BrdU (1:250).

### Western blotting

This procedure was followed as described^5^. Dilution of antibodies were as follows: Anti-Cbx4 (1:500); Anti-Cbx2 (1:500); Anti Ring1B (1:500); Anti-H2AK119ub1 (1:2000); Anti-H2A (1:1000); Anti-H3K27me3 (1:1000); and Actin (1:1000).

### *In vitro* stromal cell decidualization

Isolation and induction of primary uterine stromal cells for decidualization were followed as described^58^. In brief, mouse uterine stromal cells collected on day 4 of pseudopregnancy were cultured in presence of the growth medium [DMEM/F-12 (1:1), plus 10% charcoal stripped serum and antibiotic]. Following a brief attachment for 1 h, cells were washed thoroughly in HBSS and replenished with the growth medium for 24 h. Cells were then replaced with the decidualization medium [1 mM P4, 10 nM E2 and 0.5% HB-EGF in DMEM/F12, plus 1% charcoal stripped FBS] or with the above growth medium. Cells were washed and replenished every alternate day and collected after 5 days with or without decidualization.

### Inhibition of PRC1 during decidualization *in vitro*

Uterine stromal cells in the above culture were subjected to PRT4165 at 0, 25, 50, 100 μM concentrations during the progression of decidualization and cells were analyzed after 5 days of decidualization.

### Reverse transcription and quantitative real time-PCR

Total RNA (1 μg) was extracted from three independent cell preparations for each group and primed with random-hexamers in a volume of 20 μl and reverse transcribed into cDNA with MMLV reverse transcriptase (cat# M1701, Promega). The resulting cDNA was subjected to quantitative-PCR analysis. In brief, one-step RT-PCR was performed using Step One Plus real-time PCR system (Applied Biosystems, Grand Island, NY) and Fast SYBR Green Master Mix (cat# 4385610, Life Tech, Grand Island, NY). Holding stage at 95 °C for 20 s, 40 PCR cycling stage consisting of denaturation at 95 °C for 3 s, annealing and extension at 60 °C for 30 s, and melting curve stage consisting of denaturation at 95 °C for 15 s, annealing and extension at 60 °C for 1 min, and final termination at 95 °C for 15 s. Melting curves for all products showed single peaks. The relative target gene expression was quantified after normalization with *ribosomal protein l7* (*Rpl7*, housekeeping gene). The gene specific primers used for qPCR are indicated in Table S1.

### Flow cytometry analysis of DNA content

Cells in culture or isolated *in vivo* were fixed with pre-cold 70% ethanol, followed by RNase A (500 mg/mL) treatment for 30 min at 37 °C, and then stained with propidium iodide (PI, 50 mg/mL). DNA content was directly analyzed by flow cytometry (BD FACSCanto II) using the Cincinnati Children’s Core facility. A total of 50,000 to 100,000 cells were subjected for each analysis.

### *SiRNA-driven* perturbation of *cbx4* during *in vitro* decidualization

In the above primary culture model, prior to the initiation of decidualization, cell were subjected to transfection with *cbx4 siRNAs* (at 100 nM) using the lipofectamine 2000 reagent (Invitrogen) for 6 h, according to the Manufacturer’s instructions. We used three independent mouse specific *siRNAs* for *cbx4* and they were as follows: CAGGAAGAGCGGCAAGTATTA (*siRNA6*), AAGGTCCGAAGTTGAGGGAAA (*siRNA7*), and AAGGAGGCCTTTGGTGAGCAA (*siRNA9*) (Qiagen). In parallel studies, cells were also transfected with control *siRNAs* (Negative Control *siRNA*) (Qiagen). Cells were analyzed after 5 days of decidualization.

### RNA sequencing and data analysis

As described above in *schedule B*, 4 mg/kg PRT4165- or vehicle-treated ISs on D8 were used to collect separated decidual tissues for global gene expression analysis. Total RNAs were isolated using the Trizol reagent (Life Technologies) according to the Manufacturer’s instructions. The preparation of cDNA library and sequencing were performed at the Cincinnati Children’s Hospital Medical Center sequencing core. In brief, total RNA (150 to 500 ng) as determined by Qubit 3.0 Fluorometer (Life Technologies) was poly A selected and then reverse transcribed using the TruSeq stranded mRNA library preparation kit (Illumina). Each sample was fitted with one of 24 adapters containing a different 8 base molecular barcode for high level multiplexing. After 15 cycles of PCR amplification, completed libraries were sequenced on an Illumina HiSeq2500 in Rapid Mode, generating 20 million or more high quality 75 base long paired end reads per sample. RNA-seq data analysis was performed using BioWardrobe platform^59^ (http://biowardrobe.com). Briefly, reads were aligned to the mm10 genome with RNA-STAR^60^ provided with RefSeq annotation and RPKM values were calculated by BioWardrobe. Differentially expressed genes were identified with DESeq2^61^ using Padj < 0.05, fold change > 1.5 and RPKM > 3 in at least one condition. Heatmap was built using Cluster3 and JavaTreeView^62^,^63^. Functional enrichment analysis of the differentially expressed genes was performed using ToppFun (http://toppgene.cchmc.org)^64^ and AltAnalyze^65^ was used to build the network which was then manually curated using Cytoscape^66^. Raw data and RPKM values were deposited to GEO and accession number will be provided upon acceptance.

### Statistics

All experiments were performed at least three times and the results of a representative experiment are presented. Significant differences between experimental and control groups were analyzed using a Student’s t-test, and ANOVA test were used when there were more than two groups to compare. Differences of P < 0.05 were deemed to indicate statistically significant.

## Additional Information

**How to cite this article**: Bian, F. *et al*. Polycomb repressive complex 1 controls uterine decidualization. *Sci. Rep.* **6**, 26061; doi: 10.1038/srep26061 (2016).

## Supplementary Material

Supplementary Information
