# Supplementary material for: Polycomb repressive complex 1 controls uterine decidualization
Source: Sci Rep. 2016 May 16;6:26061. doi: 10.1038/srep26061 (PMC4867636; doi:10.1038/srep26061)
Supplement: Supplementary Information [file srep26061-s1.pdf]

SUPPLEMENTAL INFORMATION

Polycomb repressive complex 1 controls uterine decidualization

Short title: **PRC1 in decidualization**

Fenghua Bian[1,2], Fei Gao[1,2], Andrey V. Kartashov[3], Anil G. Jegga[4], Artem Barski[3] and Sanjoy K. Das[1,2]

[1]Division of Reproductive Sciences, Cincinnati Children's Hospital Medical Center, Cincinnati, OH 45229, USA; Department of Pediatrics, University of Cincinnati College of Medicine, Cincinnati, OH 49267, USA

[2] Perinatal Institute, Cincinnati Children's Hospital Medical Center, Cincinnati, OH 45229, USA

[3] Division of Allergy and Immunology, Division of Human Genetics, Cincinnati Children's Hospital Medical Center, Cincinnati, OH 45229, USA; Department of Pediatrics, University of Cincinnati College of Medicine, Cincinnati, OH 49267, USA

[4] Biomedical Informatics, Cincinnati Children's Hospital Medical Center, Cincinnati, OH 45229, USA; Department of Pediatrics, University of Cincinnati College of Medicine, Cincinnati, OH 49267, USA

Supplemental figure legends

Fig. S1. Immunohistochemical analyses of expression. (A) Expression of Cbx2, Cbx4, Rybp, and Ring1B at the D7-IIS. Pictures are shown at 100X and 400X (as insets). le, luminal epithelium; ge, glandular epithelium; s, stroma. (B) Immunological analyses after incubation with normal rabbit or mouse IgG as controls on D7-IS. Pictures are shown at 100X and 400X (as insets). e, embryo; sdz, secondary decidual zone.

Fig. S2. Immunohistochemical analyses of expression. (A) Expression of H2AK119ub1 and H3K27me3 at the D7-IIS. Pictures are shown at 100X and 400X (as insets). le, luminal epithelium; s, stroma. (B) Immunological analyses after incubation with normal mouse IgG as control on D7-IS. Pictures are shown at 100X and 400X (as insets). e, embryo; sdz, secondary decidual zone.

Fig. S3. Immunofluorescence analysis of co-localized expression on D7-IS. (A) Cbx2 (Red) together with H3K27me3 (green). (B) H2AK119ub1 (Red) together with H3K27me3 (green). DAPI was used for nuclear staining. AM, antimesometrial pole; sdz, second decidual zone. Arrow indicates direction of embryo (em) location. Pictures are shown at 400X.

Fig. S4. PRC1 inhibition has no effects on embryo-uterine attachment during early pregnancy. Mice were injected with PRC1 inhibitor (PRT4165 at 4mg/kg and 8mg/kg) or vehicle (control) on D3-D4 and analyzed on D5 (09:00h) as described in Materials and Methods. (A) Representative photographs of uterine implantation are shown after blue reaction. (B) Analysis of the number or weight of IS reveals no statistical difference. (C) Histological analysis of IS after staining with hematoxylin and eosin. (D) Immunostaining analysis of IS by Ki67 and phospho-histone H3 (pHH3). (E) Quantitative analysis of Ki67 and pHH3 positive staining cells in the endometrium as shown in D. (F) Quantitative RT-PCR analysis of expression for implantation related genes (*Hbegf, Lif,* and *Ptgs2*). (G) Western-blot analysis reveals inhibition of PRC1, as judged by downregulation of H2AK119ub1, after addition of PRT4165 against control. The error bars represent standard means ± SEM. "*", Values are statistically different ($P < 0.05$). These experiments were repeated at least three times with similar results.

Fig. S5. PRC1 inhibition has no effects on ovarian E2 and P4 levels in the serum or development of ovarian corpora luteal structures. Mice were injected with PRT4165 (at 4mg/kg and 8mg/kg) or vehicle (control) on D5-D7 and analyzed on D8 (09:00h) as described in Materials and Methods. (A) Analysis of E2 and P4 levels in the serum. (B) Histological analysis of the representative ovaries. Sections were stained with hematoxylin and eosin. Pictures are shown at 40X. CL, corpus luteum.

Fig. S6. Inhibition of PRC1 affects experimentally-induced decidualization. Mice in pseudopregnancy were injected with PRC1 inhibitor (PRT4165 at 4mg/kg and 8mg/kg) or vehicle (control) on D5-D7 and analyzed on D8 (09:00h) as described in Materials and Methods. (A) Representative photographs of deciduoma-induced uteri. B. Fold changes indicate comparison of weights between infused and noninfused uterine horns. The error bars represent standard errors from three independent experiments. *, Values are statistically different ($P < 0.05$) between PRT4165 vs. control. C. Histological analysis of deciduoma is shown by hematoxylin and eosin staining. Representative uterine sections are shown at 40X. High magnification pictures of SDZ areas are shown at 400X (insets). sdz, secondary decidual zone; M, mesometrial pole; AM, antimesometrial pole. These experiments were repeated at least three times with similar results.

Fig. S7. Inhibition of PRC1 affects decidual polyploidy development *in vitro*. Day 4 uterine stromal cells were treated with vehicle control or PRT4165 (at 25 and 50μM) in the culture during induction of decidualization (+Dec), as described in Materials and Methods. Cells were collected after 5 days of decidualization. Cells without induction of decidualization (-Dec) were served as decidualization control.

(A) Flow cytometric analysis. Quantitative analyses of cell distribution (%) based on the DNA content are shown as insets for each representative group. (B) Quantitative analysis of polyploid cell count (%) with or without inhibition of PRC1 during decidualization. The error bars represent means ± SEM from three independent experiments. *, Values are statistically different ($P < 0.05$). (C) Quantitative RT-PCR. Analysis of expression for *Prl8a2*, *Bmp2*, *Abp1*, *Alpl*, and *Tdo2* after inhibition of PRC1 against controls.

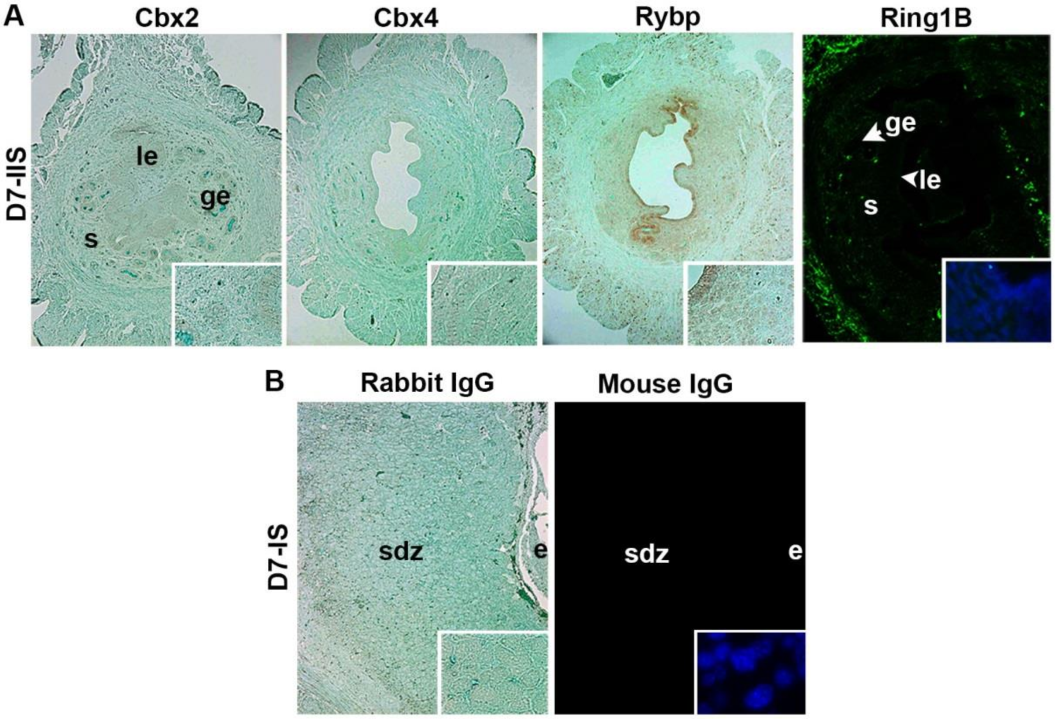

Fig. S1, Bian et al

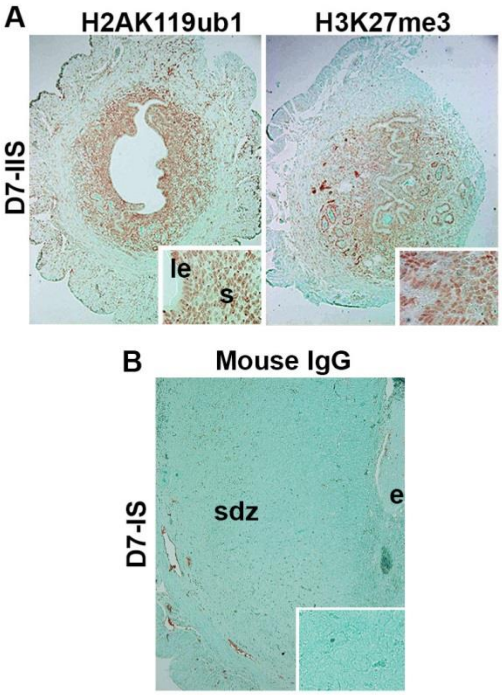

Fig. S2, Bian et al

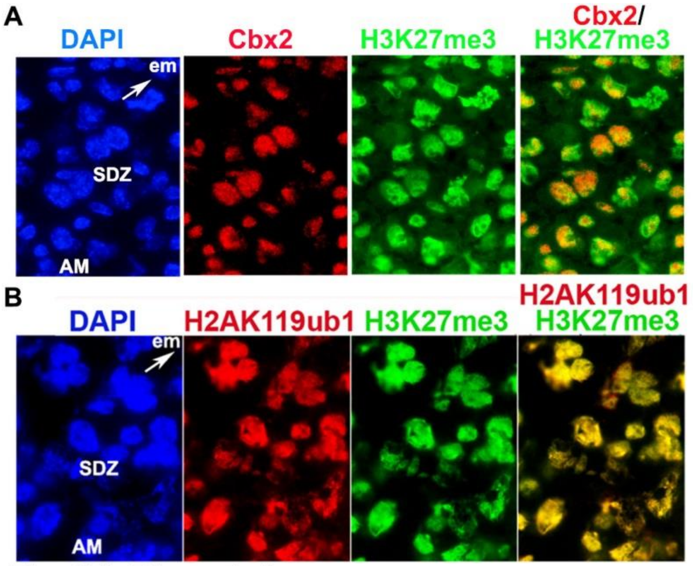

Fig. S3, Bian et al

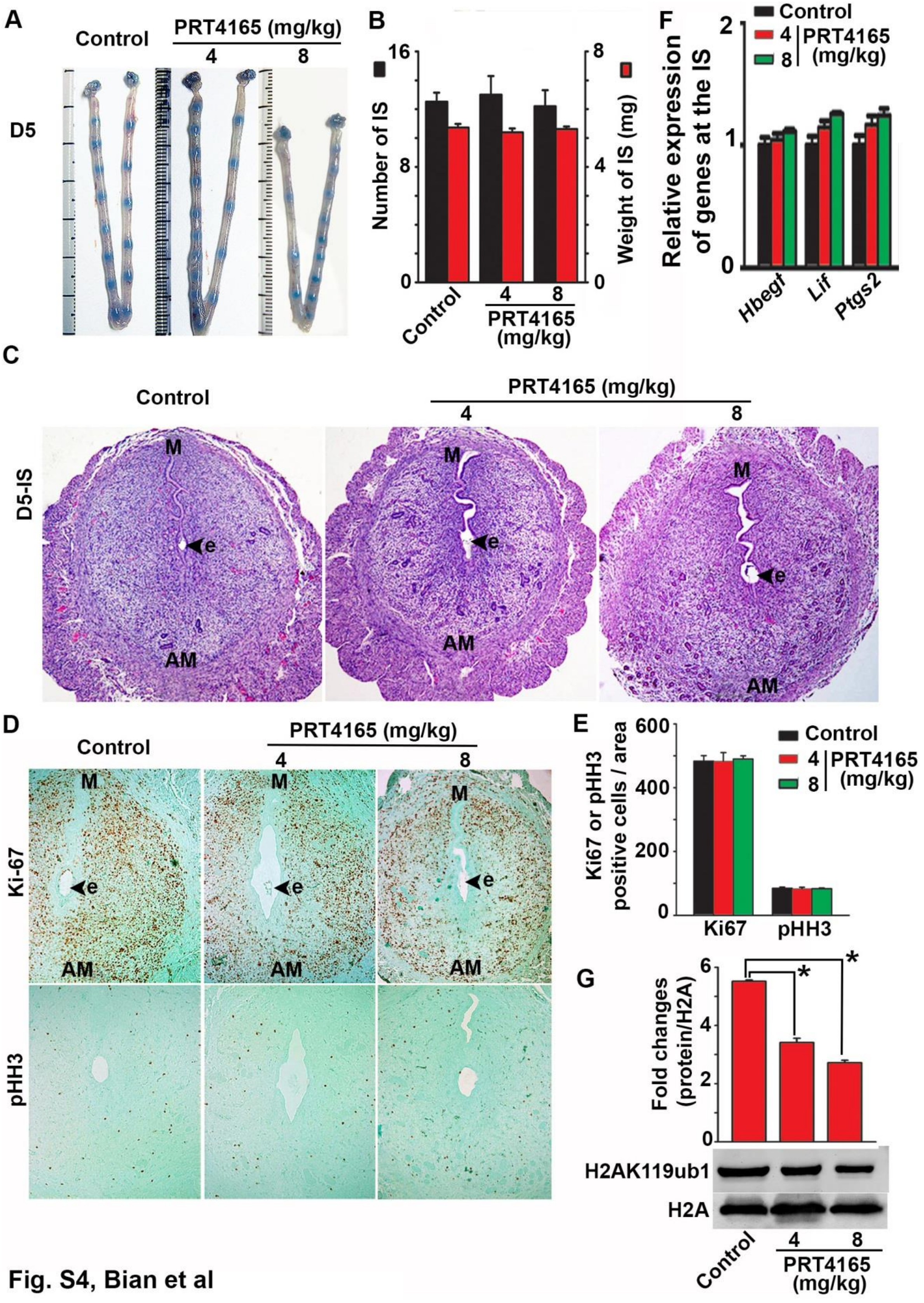

Fig. S4, Bian et al

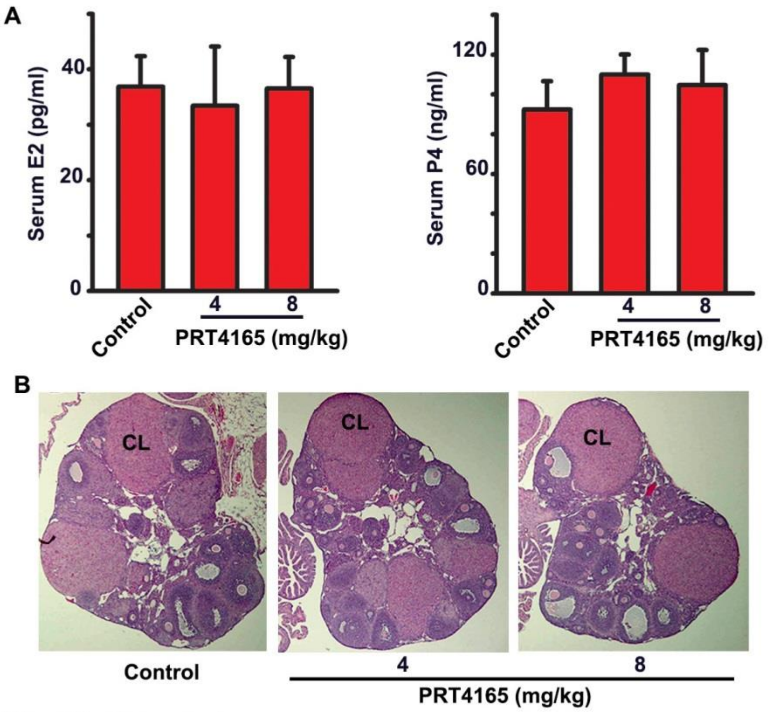

Fig. S5, Bian et al

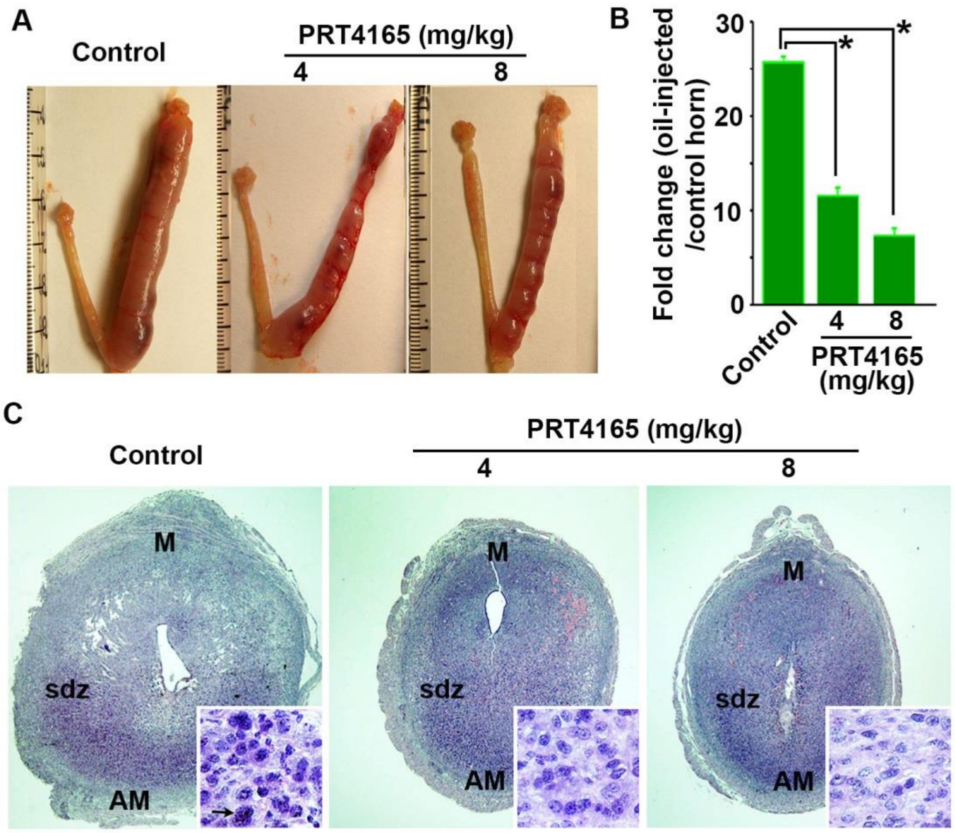

Fig. S6, Bian et al

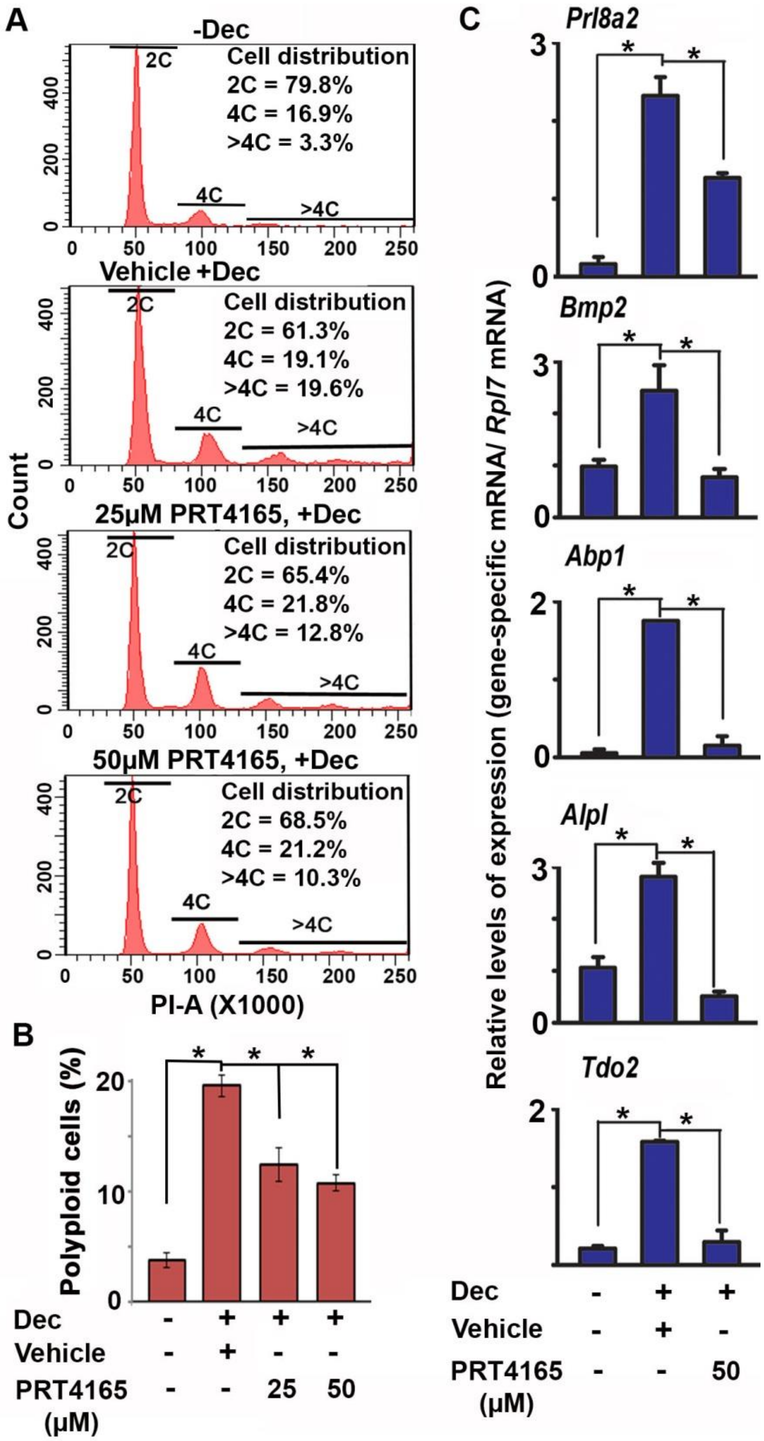

Fig. S7, Bian et al

Table S1. Sequence of primers used for RT-qPCR analyses.

Genes	Forward primers	Reverse primers
Cbx4	gcccttgagtgagttcaagc	gcgactccttcccactacac
Cbx2	gagccagagtcaggttcagg	ccttctgcgtcctcactttc
Cbx6	gccagaggagaacattctgg	ttgggtttaggtcccctctt
Cbx7	cgagcctcagggtataggaa	agagcttctcattgcccttg
Cbx8	tggagcccctctctgactaa	agaatccttggtccgtgttg
Ring1A	cctggacatgctgaagaaca	gcagtccgagcagaacctat
Ring1B	aggccccatccaactcttat	agtggcattgcctgaagtct
Bmi1	atgagtcaccagagggatgg	aagaggtggagggaacacct
Mel18	tgtgacgtccaggtccataa	tgtcctggagggttttgtct
Pcgf1	ggctgagttctggcaaagac	ggagctgtacatgctgtgga
Pcgf6	aggaggaggagatggaggag	tccagacgcagagagaagtg
Phc1	cccttacctgcagctcaaac	gcttctgtttggcttccttg
Phc2	gaaactctgcctccagcatc	tcctggatcacaaacccttc
Phc3	ccgcctttaactgtgtctcc	agggtgtggctggataagtg
Rybp	ggaaaagactcgctcctcct	cactggaggtcactgtggaa
Yaf2	gatgagggctactgggactg	catcgcacatcatgcactt
Bmp2	agatctgtaccgcaggcact	aagttcctccacggcttctt
Alpl	gcctgctctgtttcttcacc	gggcagcgtcagatgttaat
Abp1	gcgtgttgcctatgaggtcag	aaagcatccaggaaagtagcg
Tdo2	tgaaaggcctggaagaagaa	cgcttctcatcaaacaagca
Rpl7	gcagatgtaccgcactgagattc	acctttgggcttactccattgata
Slc16a3	ggtcagcgtctttttcaagg	ggtcctgtgccatagagcat
Rab28	gcagaaatcctgggaatcaa	gacaagggctcctggttgta
Tmem111	agtacctgaaccggcatctg	catgctggggccatatagtt
Bcl3	ctaccagggacctttgatgc	ggtgagtaggcaggttcagc
Gm7257	aatgtgcctcgctcataacc	gtctgcacagtcccagtgaa
Nr5a1	gccaggagttcgtctgtctc	ggctgtggttgttcaggaat
Hoxa10	cgcctagagatcagccgtag	tcaggaagcgaaaagacgtt
Hoxa11	tacctcccaatggtggtgat	gcacagcctctggagttttc
Cdkn1c	agagaactgcgcaggagaac	tctggccgttagcctctaaa
Trp53	tggaagactccagtgggaac	tcttctgtacggcggtctct
Cdkn1a	gttccttgccacttcttac	actgcttcactgtcatcc
Sfrp5	aaggattgtgggtctcatcg	gccttggatcttgctctttg
Cebpb	acttcagcccctacctggag	ggctcacgtaaccgtagtcg
Igf2	atgtcctgctctccacaacc	cagaagtcaggccaggtagc
Cxcl5	gtgcagtgggtttgagaaca	atatgctggaggaggtgtgg
Ccl20	cttgctttggcatgggtact	tgtacgagaggcaacagtcg
Rimklb	atggaaggatgcaaagcaac	atcaacaggtcaatgccaca
Prl8a2	agccagaaatcactgcca	tgatccatgcacccataaaa
Hbegf	ccacctcactccctttgtgt	aaagctccctgctcttcctc
Lif	attgtgcccttactgctgct	tgagctgtgccagttgattc
Ptgs2	tgcagaattgaaagccctct	ccccaaagatagcatctgga
Rimklb	atggaaggatgcaaagcaac	atcaacaggtcaatgccaca
Ccl27a	ttcggagccccggacat	tggcttgttggagacatcgg
Klk15	tggcttctccttgctttcgt	ccacaattaaaccggccacg
Papss2	aggctttatgcgggagaagg	atgactccatcacggggaac
Ear11	acatgggtctggagcaactt	tgctggatgtcaaaccaccg
Lrat	gctctcggatcagtccacag	gccaggcctgtgtagacaat
Ptx3	ggcacaaagtggaacccta	ccaccaccaacactagggac
Coch	gatcccacgcaggtccac	gacaggaatgggaaccgctc
Cwc22	cctctagtagtgcgagcgac	tagctgaggtagagctctgagtg
Degs2	acaccacgacttccccagta	ggttccagtcatccagtggc
Wnt10a	atggagaacatgcggcgtaa	ccctactgtgcggaactcag

Table S2A. List of up-regulated genes obtained by RNAseq analysis during decidualization on D8-IS, following the inhibition of PRC1 by PRT4165 in mice.
Total = 154 genes (filtered by RPKM >3; Padj <0.05; Fold change (PRT/Con) >1.5)

	Refseq_id	Gene_id	Chrom	txStart	txEnd	Strand	RPKM D8ISCon	RPKM D8ISPRT	LOGR	pvalue	padj	MaxRPKM	Fold change (PRT/Con)
1	NM_020047	Tacstd2	chr6	67534059	67535822	-	5.527243296	38.82996623	2.751259	6.89E-275	3.26E-271	38.82997	6.733045889
2	NM_026662	Prps2	chrX	167346333	167382699	-	40.90250079	117.7956632	2.049641	0.00E+00	0.00E+00	117.7957	4.14002856
3	NM_053111	Ear6	chr14	51853768	51854642	+	6.096319993	23.80173365	1.954032	7.83E-68	3.47E-65	23.80173	3.87455744
4	NM_011872	Klk7	chr7	43811444	43816359	+	5.063616753	25.23679988	1.90534	2.89E-79	1.57432E-76	25.2368	3.745972678
5	NM_017385	Ear7	chr14	51853998	51854465	+	16.49319998	57.05753136	1.898245	2.23E-66	9.29E-64	57.05753	3.727595179
6	NM_027664	Rimklb	chr6	122453609	122486305	-	8.477796714	38.69216665	1.884129	1.91E-212	4.52E-209	38.69217	3.691300374
7	NM_027762	Tchhl1	chr3	93468754	93471980	+	1.734480023	6.694516659	1.82987	8.20E-47	2.24E-44	6.694517	3.555051556
8	NM_010797,NM_183151	Mid1	chrX	169879622	169990797	+	4.012753626	11.60343639	1.721871	7.29E-110	6.08E-107	11.60344	3.298639738
9	NM_031185	Akap12	chr10	4266329	4359471	+	6.089183331	16.97993279	1.665789	2.11E-173	3.74E-170	16.97993	3.17287128
10	NM_001048179,NM_001164044,NM_001164045,NM_011336	Ccl27a	chr4	41772895	41774176	-	8.204076954	34.28704915	1.658815	2.35E-41	5.21E-39	34.28705	3.157570307
11	NR_028363	AA465934	chr11	83291699	83294637	+	0.792710324	7.139288048	1.60397	2.41E-22	2.09E-20	7.139288	3.039786734
12	NM_001033415	Shisa3	chr5	67607883	67613987	+	4.079860051	9.162206491	1.306758	2.40E-52	7.73E-50	9.162206	2.473849753
13	NM_033217	Ngfr	chr11	95568821	95587698	-	1.557393312	4.188209931	1.273031	2.65E-28	3.11E-26	4.18821	2.416688103
14	NM_001168620,NM_032003	Enpp5	chr17	44078848	44086561	+	1.959649007	4.572864274	1.209322	2.22E-21	1.78E-19	4.572864	2.31229021
15	NM_011171	Procr	chr2	155751217	155755478	+	66.39713415	138.0510686	1.196524	2.51E-150	3.56E-147	138.0511	2.291868292
16	NM_054077	Prelp	chr1	133910304	133921401	-	35.40763346	67.17159971	1.162794	5.57E-116	5.27E-113	67.1716	2.23890591
17	NM_020036	Calm4	chr13	3837757	3838671	+	4.92699337	12.38093344	1.154576	4.73E-19	3.24E-17	12.38093	2.226188396
18	NM_001039676	Slc39a2	chr14	51893610	51896745	+	8.303700129	17.16216628	1.118204	1.13E-46	3.03E-44	17.16217	2.170765544
19	NM_011414	Slpi	chr2	164354070	164356507	-	14.32743327	28.48206774	1.095791	2.76E-31	3.69E-29	28.48207	2.137301618
20	NM_001201470,NM_011864	Papss2	chr19	32620005	32667187	+	7.290506681	15.16336664	1.090289	1.78E-43	4.35E-41	15.16337	2.12916644
21	NM_008768	Orm1	chr4	63344566	63348162	+	1.672956626	5.279713392	1.078411	2.03E-11	6.90E-10	5.279713	2.111708329
22	NM_011606	Clec3b	chr9	123150946	123157432	+	6.565850099	11.6656402	1.051366	3.76E-13	1.55E-11	11.66564	2.072491148
23	NM_008437	Napsa	chr7	44572445	44586846	+	19.26353327	40.40209961	1.043065	2.48E-51	7.66E-49	40.4021	2.060600559
24	NM_172543	Fam117a	chr11	95337018	95381872	+	14.9483668	28.59666634	1.041226	1.11E-58	4.27E-56	28.59667	2.057976433
25	NM_009142	Cx3cl1	chr8	94772180	94782426	+	10.28335317	18.37166659	1.032053	2.61E-52	8.24E-50	18.37167	2.044932609
26	NM_001163098	Tchh	chr3	93442330	93449077	+	1.950859984	3.689353387	1.017521	3.26E-24	3.15E-22	3.689353	2.024437559
27	NM_008725	Nppa	chr4	148000746	148002067	+	3.544253349	8.860956828	1.013378	1.17E-12	4.58E-11	8.860957	2.018631577
28	NM_008764	Tnfrsf11b	chr15	54250619	54278484	-	21.01496633	37.07136663	1.00755	1.20E-69	5.47E-67	37.07137	2.010493885
29	NM_021719	Cldn15	chr5	136967869	136975845	+	4.183583339	7.37468346	0.998582	2.65E-15	1.35E-13	7.374683	1.998035417
30	NM_007675	Ceacam10	chr7	24777204	24784655	+	29.17983373	49.95736694	0.982076	3.69E-30	4.67E-28	49.95737	1.975305506
31	NM_001166206	Erv3	chr2	131853678	131859747	-	468.3306783	834.6779989	0.972135	3.83E-203	7.76E-200	834.678	1.961741249
32	NM_145379	Mrgprf	chr7	145300909	145309557	+	13.16950003	24.1260999	0.965641	2.86E-26	3.05E-24	24.1261	1.952930387
33	NM_001083955	Hba-a2	chr11	32296492	32297310	+	35.06522328	68.78483392	0.963265	1.20E-33	1.96E-31	68.78483	1.949717006
34	NM_008218	Hba-a1	chr11	32296489	32297303	+	35.31614829	69.27700889	0.963265	1.20E-33	1.96E-31	69.27701	1.949717006
35	NM_177782	Prex1	chr2	166566345	166713832	-	16.11926715	28.35736656	0.959169	8.84E-96	6.27E-93	28.35737	1.944189415
36	NM_011474	Sprr2h	chr3	92385685	92387319	+	14.92733383	27.53070005	0.949015	1.95E-21	1.59E-19	27.5307	1.930553814
37	NM_022029	Nrgn	chr9	37544493	37552745	-	3.910770019	7.676013629	0.947084	1.96E-13	8.38E-12	7.676014	1.927971992
38	NM_024440	Derl3	chr10	75893398	75895941	+	9.784983317	16.62534316	0.931898	2.28E-22	1.99E-20	16.62534	1.907784688
39	NM_001201391	Hbb-bs	chr7	103826523	103827928	-	52.21126684	98.54456584	0.929509	1.70E-46	4.39E-44	98.54457	1.904627345
40	NM_001278161	Hbb-b1	chr7	103826532	103827929	-	52.87323252	99.79406484	0.929509	1.70E-46	4.39E-44	99.79406	1.904627345
41	NM_174865	Klk15	chr7	43933771	43939590	+	1.073477666	4.399659952	0.919875	2.45E-08	5.61E-07	4.39966	1.891950975
42	NM_019564	Htra1	chr7	130936203	130985658	+	293.8063354	481.666331	0.91983	1.36E-157	2.14E-154	481.6663	1.89189296
43	NM_008555	Masp1	chr16	23451785	23520590	-	10.39828014	19.64683342	0.912778	4.04E-32	5.79E-30	19.64683	1.882667098
44	NM_001037999,NM_007830	Dbi	chr1	120113280	120121096	-	416.0871043	705.1212606	0.883843	5.09E-115	4.51E-112	705.1213	1.845283709
45	NM_011016	Orm2	chr4	63362449	63365877	+	1.91797332	4.304262618	0.883113	3.86E-08	8.60E-07	4.304263	1.844351095
46	NM_181422	Pkd2l1	chr19	44147637	44192442	-	4.292380015	8.155356725	0.880025	5.06E-22	4.32E-20	8.155357	1.840406686
47	NM_026346	Fbxo32	chr15	58175879	58214892	-	6.928113302	10.69583019	0.879173	2.01E-49	5.83E-47	10.69583	1.839320603
48	NM_016913,NM_023638,NM_145907,NM_145908	Porcn	chrX	8193850	8206525	-	6.742579291	15.42238005	0.878423	7.53E-20	5.57E-18	15.42238	1.838365317
49	NM_008795	Cdk18	chr1	132113547	132139685	-	6.370116711	11.1628933	0.871926	2.99E-20	2.29E-18	11.16289	1.830103948
50	NM_145356	Zbtb7c	chr18	75820178	76148564	+	6.140899976	9.829950015	0.871168	3.79E-29	4.59E-27	9.82995	1.829142937
51	NM_153107	Cpz	chr5	35502218	35525626	-	3.523993333	5.857133309	0.869516	1.43E-14	6.89E-13	5.857133	1.827050388
52	NM_001080813,NM_029423	Rab11fip1	chr8	27138773	27174646	-	3.693813284	6.480923216	0.868173	3.99E-28	4.53E-26	6.480923	1.825349458
53	NM_001085549	Trabd2b	chr4	114406724	114615098	+	3.939626614	7.326949914	0.859422	4.43E-32	6.23E-30	7.32695	1.814311337
54	NM_009853	Cd68	chr11	69664371	69666062	-	19.72989972	32.88833364	0.851045	8.96E-26	9.21E-24	32.88833	1.803807292
55	NM_009034	Rbp2	chr9	98490537	98509771	+	1.519089977	3.840509892	0.843949	3.09E-07	5.85E-06	3.84051	1.794956316
56	NM_029142	4930502E18Rik	chrX	53724831	53738222	+	11.88666662	22.67200025	0.838896	1.27E-17	7.86E-16	22.672	1.788680732
57	NM_008116	Ggt1	chr10	75573593	75586182	+	23.61266645	38.26629957	0.82513	3.50E-42	8.01E-40	38.2663	1.771694144
58	NM_053110	Gpnmb	chr6	49036518	49058182	+	186.7536672	299.0426636	0.818546	1.90E-128	1.92E-125	299.0427	1.763627935
59	NM_001081437,NM_007992	Fbln2	chr6	91212764	91272540	+	87.26499184	145.9793835	0.814802	1.28E-97	9.59E-95	145.9794	1.759056465
60	NM_013569	Kcnh2	chr5	24319589	24351604	-	1.805463314	3.069670041	0.813943	3.61E-12	1.34E-10	3.06967	1.758010159
61	NM_181390	Mustn1	chr14	30879257	30881610	+	17.95950063	27.60233307	0.811106	2.00E-21	1.62E-19	27.60233	1.754555865
62	NM_001272052,NM_007398	Ada	chr2	163726571	163750239	-	129.270635	200.7322985	0.811061	7.15E-94	4.83E-91	200.7323	1.754501597
63	NM_033037	Cdo1	chr18	46713205	46728342	-	5.919160048	10.86407661	0.808611	1.12E-13	4.93E-12	10.86408	1.751523733
64	NM_153507	Cpne2	chr8	94533028	94570529	+	3.271843354	5.720019976	0.805045	8.96E-12	3.17E-10	5.72002	1.747200675

	Refseq_id	Gene_id	Chrom	txStart	txEnd	Strand	RPKM D8ISCon	RPKM D8ISPRT	LOGR	pvalue	padj	MaxRPKM	Fold change (PRT/Con)
65	NM_001162475,NM_001162476,NM_001162479,NM_009291	Stra6	chr9	58134665	58153997	+	41.21353977	59.2091064	0.802518	3.10E-72	1.63E-69	59.20911	1.744143159
66	NM_199252	Unc93a	chr17	13108617	13131791	-	2.115853349	3.714533329	0.800679	6.46E-08	1.39E-06	3.714533	1.741920394
67	NM_080575	Acss1	chr2	150618111	150668240	-	29.26953316	50.26639938	0.793488	1.24E-55	4.52E-53	50.2664	1.733259891
68	NM_026601,NR_003946	Hyi	chr4	118359999	118362744	+	3.653113703	7.655693293	0.790855	2.52E-08	5.76E-07	7.655693	1.730099362
69	NM_001142539	Gm9992	chr17	7363712	7385305	-	11.28721333	19.1280098	0.775784	5.55E-21	4.37E-19	19.12801	1.712120516
70	NM_001081169	Aspg	chr12	112106683	112127573	+	55.53836695	87.62133153	0.775277	1.34E-58	5.02E-56	87.62133	1.711518652
71	NM_011118	Prl2c3	chr13	12790821	12800079	-	1.566356699	3.397016694	0.772425	1.61E-06	2.62E-05	3.397017	1.708138696
72	NM_011954	Prl2c4	chr13	12790818	12800058	-	1.600160658	3.470331599	0.772425	1.61E-06	2.62E-05	3.470332	1.708138696
73	NM_008791	Pcp4	chr16	96467606	96525793	+	51.13309987	79.97096507	0.771119	5.14E-20	3.83E-18	79.97097	1.706592409
74	NM_010639	Klk1	chr7	44225437	44229617	+	17.13300006	33.96789996	0.768811	1.93E-16	1.06E-14	33.9679	1.703864485
75	NM_001042615,NM_030127	Htra3	chr5	35661559	35679782	-	120.3467406	185.2084007	0.768721	1.76E-83	1.09E-80	185.2084	1.703758493
76	NR_002891	Gm5512	chr19	12905231	12906985	-	1.840397802	3.773915917	0.765259	4.92E-07	8.99E-06	3.773916	1.699675606
77	NR_015554,NR_038090	AI506816	chr5	23710017	23712667	-	10.40895357	14.71445157	0.76398	3.67E-23	3.28E-21	14.71445	1.698169118
78	NM_001109043,NM_009549	Zfp185	chrX	72987339	73031543	+	4.221596678	6.58476003	0.763201	5.11E-15	2.52E-13	6.58476	1.697252091
79	NM_001272041,NM_009606	Acta1	chr8	123891758	123894775	-	23.43482482	37.68388114	0.762225	1.64E-23	1.49E-21	37.68388	1.696104414
80	NM_001077694,NM_021469	Dysf	chr6	84019389	84211042	+	5.879138311	9.487102648	0.758909	6.13E-32	8.52E-30	9.487103	1.692210671
81	NM_201374	Ccdc155	chr7	45183676	45204892	-	11.53205331	18.38976701	0.747977	1.49E-19	1.05E-17	18.38977	1.679435654
82	NM_027334	Mettl7a1	chr15	100304817	100314348	+	10.41828346	15.71964391	0.746525	3.63E-19	2.50E-17	15.71964	1.677747021
83	NM_001033254,NM_001145854	Pak6	chr2	118677246	118698020	+	1.804239372	3.149507344	0.744104	2.19E-10	6.66E-09	3.149507	1.674933483
84	NM_001083917,NM_153522,NM_178227	Scn3b	chr9	40269216	40291618	+	7.524374483	13.19767454	0.74329	1.32E-23	1.21E-21	13.19767	1.673988471
85	NM_001130484,NM_001130485,NM_021338	Rpl35a	chr16	33056514	33060188	+	111.3624083	199.8143055	0.741619	5.90E-36	1.07E-33	199.8143	1.672050711
86	NM_175013	Pgm5	chr19	24678261	24861842	-	11.50220013	17.77260017	0.73757	1.00E-42	2.33E-40	17.7726	1.667364921
87	NM_174866	Klk14	chr7	43690418	43695535	+	1.581419984	3.387579918	0.736999	3.50E-06	5.34E-05	3.38758	1.666705225
88	NM_026170	Ergic1	chr17	26561512	26656933	+	27.10210037	41.91889954	0.724369	6.58E-43	1.58E-40	41.9189	1.652177792
89	NM_022982	Rtn4r	chr16	18127706	18152408	+	2.891266664	4.367123286	0.721382	2.95E-08	6.67E-07	4.367123	1.648761076
90	NM_001199283,NM_001199284,NM_173388	Slc43a2	chr11	75532112	75577572	+	5.591323761	8.651257927	0.720059	1.31E-27	1.46E-25	8.651258	1.647249433
91	NM_025359	Tspan13	chr12	36014555	36042478	-	12.16016674	18.57570012	0.718246	1.57E-16	8.72E-15	18.5757	1.645180772
92	NM_133983	Cd276	chr9	58524300	58540940	-	3.910726706	5.407540083	0.716846	2.36E-13	9.95E-12	5.40754	1.643585
93	NM_018789	Foxo4	chrX	101254528	101260873	+	25.65936661	36.38506635	0.714019	9.15E-43	2.16E-40	36.38507	1.640367845
94	NM_172589	Lhfpl2	chr13	94057796	94195409	+	8.035833677	12.38646666	0.70764	2.35E-20	1.81E-18	12.38647	1.633129956
95	NM_152801	Arhgef6	chrX	57231485	57338729	-	29.40803337	42.36790021	0.705037	4.43E-55	1.53E-52	42.3679	1.630186692
96	NM_001005421	Amica1	chr9	45079183	45108531	+	5.028093338	7.531693459	0.703518	1.03E-11	3.60E-10	7.531693	1.628470451
97	NM_001042605,NM_010545	Cd74	chr18	60803849	60812652	+	26.81494315	39.24891027	0.699072	7.29E-24	6.76E-22	39.24891	1.623460167
98	NM_008012	Akr1b8	chr6	34354164	34368454	+	12.48733362	19.18339984	0.698422	3.96E-14	1.82E-12	19.1834	1.622728482
99	NM_010826,NM_194464	Mrvi1	chr7	110868267	110982095	-	7.124556541	10.34092981	0.697651	3.64E-27	4.00E-25	10.34093	1.62186186
100	NM_019779	Cyp11a1	chr9	58015017	58027023	+	271.148997	376.3213298	0.69313	1.88E-83	1.11E-80	376.3213	1.616787396
101	NM_001159662,NM_153089	Ppp1r16b	chr2	158667135	158766334	+	3.422700087	4.698766589	0.692123	8.70E-16	4.57E-14	4.698767	1.61565943
102	NM_133712	Klk10	chr7	43781054	43785410	+	0.453342676	5.930229982	0.692061	6.51E-07	1.16E-05	5.93023	1.615589998
103	NM_001004152	Psg22	chr7	18718090	18727248	+	6.622243404	9.130380074	0.69145	3.89E-13	1.60E-11	9.13038	1.614905716
104	NM_001093749,NM_176993	Mpzl3	chr9	45055217	45074888	+	11.70460622	19.81789955	0.690651	2.09E-12	7.99E-11	19.8179	1.614012204
105	NM_009258	Spink3	chr18	43728069	43737237	-	112.7289988	193.9520009	0.690418	1.67E-21	1.38E-19	193.952	1.613751164
106	NM_007554	Bmp4	chr14	46383520	46390599	-	1.935726643	3.149316629	0.682701	1.46E-06	2.41E-05	3.149317	1.605141906
107	NM_001161775,NM_013607	Myh11	chr16	14194527	14291408	-	80.29789956	116.2442878	0.679982	1.13E-70	5.36E-68	116.2443	1.602120321
108	NM_001081124	Map7d2	chrX	159414578	159498947	+	15.1401666	30.03446643	0.676521	2.29E-32	3.39E-30	30.03447	1.598281302
109	NM_172134	Pdxk	chr10	78436747	78464948	-	12.60316658	17.72376664	0.676245	4.11E-31	5.45E-29	17.72377	1.597975717
110	NM_001136240,NM_172264,NM_175343	Chdh	chr14	30022633	30040466	+	4.254058689	6.369549602	0.675181	5.51905E-16	2.94253E-14	6.36955	1.596796676
111	NM_011923	Angptl2	chr2	33215961	33247714	+	8.399513086	12.0087533	0.673023	1.52E-15	7.82E-14	12.00875	1.594410357
112	NM_010687	Large	chr8	72814599	73352556	-	13.26769988	18.74353377	0.660126	4.87E-25	4.90E-23	18.74353	1.580220506
113	NM_052994	Spock2	chr10	60106257	60133913	+	8.184266408	11.1797967	0.65899	1.02E-19	7.38E-18	11.1798	1.578976738
114	NM_023049	Asb2	chr12	103321142	103356001	-	3.914010048	6.050500075	0.658827	2.67E-08	6.09E-07	6.0505	1.57879802
115	NM_001146120,NM_001146121,NM_001146122,NM_001146123,NM_001146124,NM_011179	Psap	chr10	60284461	60302600	+	4168.148556	5825.769377	0.657049	2.32E-88	1.50E-85	5825.769	1.57685376
116	NM_001160012,NM_008126	Gjb3	chr4	127325235	127330836	-	10.99880314	16.94190673	0.655554	2.08E-13	8.85E-12	16.94191	1.575220479

	Refseq_id	Gene_id	Chrom	txStart	txEnd	Strand	RPKM D8ISCon	RPKM D8ISPRT	LOGR	pvalue	padj	MaxRPKM	Fold change (PRT/Con)
117	NM_001008424	Cdsn	chr17	35552128	35557180	+	25.83063316	33.86670113	0.652505	8.99E-33	1.39E-30	33.8667	1.571895489
118	NM_010720	Lipg	chr18	74939322	74961263	-	155.9576619	221.6123352	0.645789	1.82E-71	9.21E-69	221.6123	1.564594684
119	NM_025833	Baiap2l1	chr5	144264525	144358112	-	2.566336632	4.356690089	0.644498	1.44E-07	2.94E-06	4.35669	1.563195308
120	NM_023608	Gdpd2	chrX	100729847	100738899	+	16.63589986	23.98329989	0.63915	9.72E-25	9.64E-23	23.9833	1.557411258
121	NM_001080708	Fam65c	chr2	167980786	168010593	-	22.89826647	35.94296646	0.638974	8.07E-33	1.26E-30	35.94297	1.557221184
122	NM_010810	Mmp7	chr9	7692111	7699416	+	10.34980679	15.50169341	0.632624	7.56E-09	1.88E-07	15.50169	1.550382292
123	NM_001199118,NM_022883	Lpin3	chr2	160888168	160906000	+	10.06986344	15.01260026	0.631833	9.16E-19	6.01E-17	15.0126	1.549532097
124	NM_001205076,NM_021566	Jph2	chr2	163336242	163397993	-	4.9104777	7.048145754	0.63179	1.15E-12	4.52E-11	7.048146	1.549486452
125	NM_177305	Arl4c	chr1	88698226	88702191	-	5.2714866	7.48401022	0.631421	8.24E-14	3.67E-12	7.48401	1.549089767
126	NM_001111051,NM_001111052,NM_001111053,NM_001195538,NM_001195539,NM_001195540,NM_019978	Dclk1	chr3	55461758	55539068	+	3.965543167	6.035296855	0.628162	3.55E-11	1.16E-09	6.035297	1.545595064
127	NM_001102446,NM_009653	Alas2	chrX	150547417	150570622	+	3.162460372	5.533199946	0.627843	4.96E-06	7.25E-05	5.5332	1.545253296
128	NM_028798	Crct1	chr3	93014205	93015686	-	10.98214308	15.37380648	0.627163	6.25E-08	1.35E-06	15.37381	1.544524539
129	NM_001253916,NM_001253917,NM_019982	Sez6l	chr5	112419151	112577198	-	12.54743989	17.99595658	0.625604	2.14E-29	2.62E-27	17.99596	1.54285663
130	NM_019759	Dpt	chr1	164796732	164824266	+	32.08090019	45.00450071	0.624369	4.51E-24	4.26E-22	45.0045	1.541536301
131	NM_001114174	Fam189a2	chr19	23972750	24031019	-	8.289526463	11.50336011	0.624088	1.28E-11	4.43E-10	11.50336	1.541236512
132	NM_008180	Gss	chr2	155563183	155592706	-	6.920223395	10.68720659	0.623156	1.40E-08	3.33E-07	10.68721	1.540240361
133	NM_001013019	Lrrn4cl	chr19	8850785	8853909	+	1.978856643	3.65471673	0.623053	1.97E-05	2.47E-04	3.654717	1.54013043
134	NM_029568	Mfap4	chr11	61485444	61488704	+	36.24956703	56.5500679	0.61666	3.90E-26	4.06E-24	56.55007	1.533320976
135	NM_001163510,NM_008433	Kcnn4	chr7	24370263	24385212	+	14.12978253	19.73623621	0.60463	4.64E-15	2.30E-13	19.73624	1.520588579
136	NM_181395	Pxdn	chr12	29938036	30017658	+	33.26430003	43.80966695	0.602859	2.60E-49	7.37E-47	43.80967	1.51872339
137	NM_053106	Lmod1	chr1	135324813	135368065	+	7.453499953	10.59132004	0.602277	1.09E-14	5.27E-13	10.59132	1.518111098
138	NM_139128,NM_139129,NM_139130	Coro6	chr11	77463913	77469501	+	4.842793306	9.03685538	0.601652	5.05E-07	9.20E-06	9.036855	1.517453688
139	NM_027977	Apmap	chr2	150583081	150608523	-	37.9336675	58.04526774	0.601536	2.75E-31	3.69E-29	58.04527	1.517331539
140	NM_199251	Kcnk12	chr17	87745821	87797994	-	4.944836617	6.240616798	0.600793	4.96E-08	1.08E-06	6.240617	1.516549901
141	NM_031843	Dpp7	chr2	25352290	25356332	-	11.60246658	15.71936703	0.598804	9.38E-11	2.95E-09	15.71937	1.51446039
142	NM_013494	Cpe	chr8	64592551	64693040	-	23.71776644	33.47923342	0.597663	8.22E-21	6.41E-19	33.47923	1.513263368
143	NM_020578	Ehd3	chr17	73804841	73832093	+	63.53429921	85.25	0.595582	5.28E-51	1.59E-48	85.25	1.511081741
144	NM_001141983,NM_027694	Golga7b	chr19	42255739	42270348	+	4.983453274	7.775960048	0.594091	1.18E-09	3.32E-08	7.77596	1.50952156
145	NM_013501	Cryaa	chr17	31677945	31681724	+	19.3192997	26.0661335	0.593501	7.05E-13	2.83E-11	26.06613	1.508904413
146	NM_145537	Edem2	chr2	155701673	155729475	-	60.06723277	82.28093338	0.592659	3.01E-39	6.19E-37	82.28093	1.508024075
147	NM_001170853	Mndal	chr1	173857220	173880187	-	3.615649939	5.62912337	0.591654	1.35E-06	2.26E-05	5.629123	1.506973369
148	NM_008381	Inhbb	chr1	119415465	119422248	-	6.1423467	8.094456514	0.591576	2.16E-13	9.15E-12	8.094457	1.506892073
149	NM_001081139	Ankrd35	chr3	96670131	96691034	+	7.093976657	10.35052999	0.590189	9.04E-16	4.71E-14	10.35053	1.50544441
150	NM_009373	Tgm2	chr2	158116405	158146392	-	117.0313314	157.1443354	0.588734	2.26E-61	9.14E-59	157.1443	1.503926516
151	NM_207105	H2-Ab1	chr17	34263227	34269418	+	10.44188341	14.27640597	0.58867	2.62E-09	7.09E-08	14.27641	1.503859946
152	NM_001029987	Doxl2	chr6	48975143	48978745	+	9.617739995	12.53962326	0.588413	1.41E-12	5.46E-11	12.53962	1.503591732
153	NM_001159284,NM_013870	Smtn	chr11	3517522	3539292	-	38.65653048	53.67711242	0.586659	2.38E-32	3.48E-30	53.67711	1.50176486
154	NM_007802	Ctsk	chr3	95499286	95509362	+	71.87073263	87.92606735	0.585828	1.05E-31	1.43E-29	87.92607	1.500900565

Table S2B. List of down-regulated genes obtained by RNAseq analysis during decidualization on D8-IS, following the inhibition of PRC1 by PRT4165 in mice.
Total = 84 genes (filtered by RPKM >3; Padj <0.05; Fold change (PRT/Con) >1.5)

	Refseq_id	Gene_id	Chrom	txStart	txEnd	Strand	RPKM D8ISCon	RPKM D8ISPRT	LOGR	pvalue	padj	MaxRPKM	Fold change (PRT/Con)
1	NM_030560,NM_172667	Cwc22	chr2	77895653	77946353	-	47.65317571	6.632063389	-2.974207	0	0	47.65318	7.858243544
2	NR_033520	Tmem181b-p	chr17	6439002	6450994	-	3.990670045	1.119038641	-2.321188	1.65906E-79	9.41154E-77	3.99067	4.9974348
3	NM_001171002,NM_027299	Degs2	chr12	108690222	108702306	-	31.13874876	6.932584845	-1.864374	8.6523E-100	6.81708E-97	31.13875	3.64109903
4	NM_053113	Ear11	chr14	51255262	51256112	-	102.7116674	29.80163256	-1.798482	1.2264E-136	1.4494E-133	102.7117	3.478541309
5	NM_028216	Psca	chr15	74714839	74717065	+	266.6583405	135.1041336	-1.68208	3.4487E-217	9.782E-214	266.6583	3.208903164
6	NM_001198835,NM_007728	Coch	chr12	51593341	51605773	+	118.2576718	36.6274849	-1.541654	5.7036E-136	6.2222E-133	118.2577	2.911280162
7	NM_008102	Gch1	chr14	47153895	47189402	-	13.50883357	3.909279982	-1.530533	1.65819E-60	6.53234E-58	13.50883	2.888925809
8	NM_009114	S100a9	chr3	90692633	90695691	-	29.36743355	10.51193968	-1.46601	5.29763E-31	6.89275E-29	29.36743	2.762567227
9	NM_011595	Timp3	chr10	86300412	86349505	+	163.1430003	64.47806803	-1.455715	5.6391E-240	1.9993E-236	163.143	2.742923904
10	NM_008987	Ptx3	chr3	66219887	66225806	+	27.78356679	9.332173347	-1.454433	3.255E-71	1.59181E-68	27.78357	2.740487796
11	NR_002928	Gm1943	chr8	109339800	109340908	-	4.020859957	0.590419665	-1.408794	3.89361E-18	2.48735E-16	4.02086	2.655151196
12	NM_009979	Cst9	chr2	148835147	148838737	+	5.31583341	3.356408	-1.397817	1.8785E-17	1.14831E-15	5.315833	2.635024831
13	NM_008871	Serpine1	chr5	137061506	137072272	-	46.80213292	21.08456675	-1.387574	1.3557E-144	1.7479E-141	46.80213	2.616384065
14	NM_023624	Lrat	chr3	82892582	82903974	-	7.135320187	2.388249954	-1.385014	3.34048E-55	1.18437E-52	7.13532	2.61174479
15	NM_001272078,NM_031254	Trem2	chr17	48346401	48352276	+	24.19478655	11.44614649	-1.282265	3.20274E-40	6.7793E-38	24.19479	2.432205218
16	NM_001127189	Gm13157	chr4	147753974	147809788	-	3.123549938	0.990109324	-1.175651	8.91869E-16	4.66734E-14	3.12355	2.258947656
17	NM_001145948,NM_153392	Ttc39a	chr4	109415647	109444745	+	7.720940466	3.077618311	-1.171848	4.13674E-27	4.51286E-25	7.72094	2.253001824
18	NM_178877	Slc9b2	chr3	135307700	135342767	+	3.006410042	1.150115987	-1.120039	1.26183E-13	5.52325E-12	3.00641	2.173528683
19	NM_008350	Il11	chr7	4772378	4778141	-	5.195606709	2.43105336	-1.063752	1.80885E-15	9.26103E-14	5.195607	2.090360889
20	NM_011102	Prkcg	chr7	3303532	3330696	+	6.006120046	2.916483323	-1.054576	2.02307E-21	1.63018E-19	6.00612	2.077107125
21	NM_009251	Serpina3g	chr12	104214544	104241934	+	8.961256663	4.219993353	-1.05143	1.0435E-19	7.47417E-18	8.961257	2.072582704
22	NM_181596	Retnlg	chr16	48872608	48874498	+	5.278006713	1.576400002	-1.013945	7.772E-10	2.25404E-08	5.278007	2.019426201
23	NM_008125	Gjb2	chr14	57098602	57104702	-	3.762769938	1.764410019	-1.013702	2.97822E-15	1.50847E-13	3.76277	2.019085998
24	NM_001081131	Dhtkd1	chr2	5898060	5942792	-	4.967869918	2.321350018	-1.005229	7.25739E-19	4.85492E-17	4.96787	2.007261556
25	NM_027913	Vwce	chr19	10634233	10665210	+	7.016483148	3.817733447	-0.995145	3.19917E-26	3.38586E-24	7.016483	1.993281069
26	NM_017370	Hp	chr8	109575128	109579172	-	9.831226667	4.228846629	-0.987023	8.88505E-17	5.06055E-15	9.831227	1.982090317
27	NM_001190466,NM_021532	Dact1	chr12	71309884	71320107	+	7.760429064	3.516935279	-0.985265	1.06353E-24	1.04742E-22	7.760429	1.979677257
28	NM_010923,NM_180960	Nnat	chr2	157560110	157562519	+	8.578743299	4.93467327	-0.971786	2.56242E-13	1.07198E-11	8.578743	1.961267452
29	NR_033450	Serpina3h	chr12	104247896	104254405	+	5.264666716	2.467016697	-0.96827	8.34198E-13	3.31389E-11	5.264667	1.95649275
30	NM_134050	Rab15	chr12	76797963	76822524	-	20.46530024	10.57029978	-0.967922	1.62419E-47	4.51653E-45	20.4653	1.956021806
31	NR_073205	4932415M13	chr17	53716509	53733843	+	4.877326647	2.936923345	-0.953917	3.6535E-18	2.34452E-16	4.877327	1.937125189
32	NM_025379	Cox7b	chrX	106015700	106022450	+	195.3903351	106.0431315	-0.940136	1.55964E-54	5.26638E-52	195.3903	1.918709621
33	NM_173181	Zc2hc1a	chr3	7503426	7553848	+	6.403150082	3.659849962	-0.89116	1.43836E-17	8.83064E-16	6.40315	1.854667328
34	NM_029662	Mfsd2a	chr4	122946851	122961188	-	26.29166667	15.03990014	-0.872378	1.01994E-35	1.83098E-33	26.29167	1.830677812
35	NM_009751	Bfsp1	chr2	143826528	143863173	-	3.060456594	1.471286615	-0.861234	5.82915E-09	1.48418E-07	3.060457	1.81659102
36	NM_001081121	4931429I11R	chr9	40894849	40964112	-	4.313899914	2.244286696	-0.858382	5.86513E-13	2.37655E-11	4.3139	1.813004305
37	NM_001166630	Dynlt1c	chr17	6648947	6655831	-	6.122316658	2.578645337	-0.856858	2.40049E-08	5.51763E-07	6.122317	1.811089655
38	NM_009230	Soat1	chr1	156428108	156474328	-	20.82063357	11.95483335	-0.845254	3.34752E-44	8.32887E-42	20.82063	1.796581324
39	NM_011670	Uchl1	chr5	66676121	66687234	+	12.91946634	6.394313335	-0.777787	9.60813E-12	3.37283E-10	12.91947	1.714498774
40	NM_026862	Cd177	chr7	24743983	24760311	-	6.870599906	4.422999938	-0.77559	9.87247E-14	4.37536E-12	6.8706	1.711890438
41	NM_172505	A730008H23	chr1	88264828	88277557	-	3.836889982	2.344866673	-0.774138	5.10011E-07	9.26116E-06	3.83689	1.710167825
42	NM_001166631,NM_001166632,NM_134248	Havcr1	chr11	46751168	46779578	+	4.850738143	3.132099703	-0.759497	9.31095E-09	2.28062E-07	4.850738	1.692900784
43	NM_133360	Acaca	chr11	84195438	84401651	+	4.750803312	3.170796712	-0.734436	8.8854E-23	7.77857E-21	4.750803	1.663747366
44	NM_009451	Tubb4a	chr17	57080066	57087782	-	6.240816752	3.897013346	-0.731383	1.40667E-10	4.38447E-09	6.240817	1.66022936
45	NM_001159331,NM_026366	N6amt1	chr16	87354185	87368649	+	11.81960682	6.920912802	-0.729178	1.2737E-13	5.55803E-12	11.81961	1.657694147
46	NM_001013741	Ddn	chr15	98803782	98807925	-	3.575970014	2.035850048	-0.719205	5.85964E-09	1.48927E-07	3.57597	1.646274778
47	NM_013743	Pdk4	chr6	5483351	5496278	-	8.055953185	4.45552667	-0.713456	2.56138E-15	1.30667E-13	8.055953	1.639727907
48	NM_178396	Car12	chr9	66713686	66766845	+	3.590490023	2.097246726	-0.71344	5.10449E-10	1.51131E-08	3.59049	1.639708874
49	NM_008491	Lcn2	chr2	32384637	32387739	-	37.46503385	28.77266693	-0.705447	8.93485E-19	5.89368E-17	37.46503	1.630649786
50	NM_001190409,NM_025952	Magt1	chrX	105968085	106011899	-	44.24493249	28.07442697	-0.693564	2.53922E-40	5.45625E-38	44.24493	1.617273351
51	NM_053247	Lyve1	chr7	110850607	110862953	-	33.07166672	21.06866582	-0.692302	4.29184E-32	6.08669E-30	33.07167	1.615859733
52	NM_007695	Chi3l1	chr1	134182404	134190031	+	3.472723405	2.14806668	-0.688604	2.01019E-06	3.19602E-05	3.472723	1.611722669
53	NM_009705	Arg2	chr12	79130788	79156301	+	4.850299994	2.76110665	-0.687334	4.32137E-06	6.41064E-05	4.8503	1.610304574
54	NM_183179	Kcnv2	chr19	27322619	27337179	+	3.734126647	2.408939918	-0.685222	8.6982E-11	2.75352E-09	3.734127	1.607949
55	NM_001177621,NM_001177622,NM_177870	Slc5a6	chr5	31036036	31048924	-	7.684082518	4.685419917	-0.678885	5.54333E-14	2.51168E-12	7.684083	1.600902041
56	NM_001135149,NM_001135150,NM_026228	Slc39a8	chr3	135825790	135888572	+	13.18777319	9.85278674	-0.675588	1.04184E-19	7.47417E-18	13.18777	1.597247872
57	NM_026445	Sumf2	chr5	129846990	129863951	+	20.60696665	14.30393346	-0.675365	2.98784E-19	2.067E-17	20.60697	1.597000523
58	NM_007449	Ang2	chr14	51195486	51195923	-	15.75599988	7.367883364	-0.670826	3.65249E-06	5.55193E-05	15.756	1.591984109
59	NM_145078	2610305D13l	chr4	147611936	147642508	-	3.174880028	1.033085336	-0.667194	1.21105E-05	0.000160665	3.17488	1.587981647
60	NM_147155	Tagap1	chr17	6954965	6961156	-	3.822519938	2.43211333	-0.66142	1.12196E-07	2.33089E-06	3.82252	1.581638447
61	NM_009899	Clca1	chr3	144730316	144760739	-	6.216623306	4.417476813	-0.660738	3.11163E-11	1.02626E-09	6.216623	1.580891536
62	NM_001167936	Zyg11a	chr4	108181934	108217922	-	7.528736591	4.340523402	-0.65981	1.19687E-08	2.88674E-07	7.528737	1.579875068
63	NM_010016	Cd55	chr1	130439030	130462740	-	61.13186518	48.55459976	-0.649719	1.40311E-33	2.26124E-31	61.13187	1.568863044
64	NM_033521	Laptm4b	chr15	34238026	34284295	+	75.94460042	59.76896795	-0.649449	5.25114E-39	1.0489E-36	75.9446	1.56856902
65	NM_001142706,NM_008198	Cfb	chr17	34856374	34862514	-	6.91568319	5.807686647	-0.648576	2.96312E-11	9.79557E-10	6.915683	1.567619865
66	NM_001145926,NM_001145927,NM_175696	Pianp	chr6	124998610	125003097	+	15.12538079	10.79055238	-0.647339	2.91906E-14	1.3708E-12	15.12538	1.566276398
67	NM_001030274	Ndufs5	chr4	123712710	123718186	-	16.2371664	10.19536686	-0.642761	1.06928E-06	1.82486E-05	16.23717	1.5613148
68	NM_016910	Ppm1d	chr11	85311254	85347071	+	11.18826675	7.52973334	-0.63732	1.14259E-13	5.0168E-12	11.18827	1.555437443
69	NM_025863	Trim59	chr3	69035294	69044742	-	22.4884669	15.42420038	-0.63327	1.76197E-11	6.00678E-10	22.48847	1.551076577
70	NM_011019	Osmr	chr15	6813577	6874313	-	25.44606654	17.94139989	-0.62609	2.87772E-34	4.85855E-32	25.44607	1.543376399
71	NM_009841	Cd14	chr18	36725067	36726654	-	16.72386646	11.99733353	-0.625845	7.26364E-12	2.60134E-10	16.72387	1.54311483
72	NM_001081192	Hcn4	chr9	58823512	58860955	+	26.63939985	17.69676717	-0.625775	3.87479E-30	4.86304E-28	26.6394	1.543039539
73	NM_007729	Col11a1	chr3	114030540	114220326	+	9.403656642	7.815993309	-0.620189	9.11002E-20	6.62556E-18	9.403657	1.537076829

	Refseq_id	Gene_id	Chrom	txStart	txEnd	Strand	RPKM D8ISCon	RPKM D8ISPRT	LOGR	pvalue	padj	MaxRPKM	Fold change (PRT/Con)
74	NM_175494	Zfp367	chr13	64133057	64153199	-	4.894506772	3.479233265	-0.616873	2.89245E-07	5.52839E-06	4.894507	1.533547864
75	NM_007806	Cyba	chr8	122424771	122432940	-	76.55629985	46.58926646	-0.612437	1.44436E-19	1.0242E-17	76.5563	1.528839134
76	NM_007607	Car4	chr11	84957754	84966054	+	11.4501667	7.675996621	-0.611079	5.41676E-08	1.17823E-06	11.45017	1.527401594
77	NM_001081151	Gan	chr8	117158135	117205186	+	9.156246821	6.477073193	-0.610581	1.26912E-10	3.96447E-09	9.156247	1.526874384
78	NM_013784	Pign	chr1	105518422	105663676	-	8.550390085	6.483526707	-0.608038	2.32058E-17	1.40045E-15	8.55039	1.524185441
79	NM_009621	Adamts1	chr16	85793828	85803115	-	71.51290131	47.07566706	-0.604366	1.04539E-52	3.44784E-50	71.5129	1.520310735
80	NM_008191	Guca2b	chr4	119656603	119658945	-	23.85046609	17.06593323	-0.599954	4.72461E-08	1.03402E-06	23.85047	1.515668093
81	NM_008327,NM_011940	Ifi202b	chr1	173962569	173982844	-	15.01661666	16.41421016	-0.592834	9.55104E-10	2.71449E-08	16.41421	1.508206553
82	NM_001080995	4632434I11R	chr7	92857527	92874232	-	3.123859962	2.422483325	-0.589585	1.72695E-06	2.78283E-05	3.12386	1.504813299
83	NM_027093	Ndufaf5	chr2	140170646	140205252	+	3.984139919	2.302339951	-0.585882	6.22846E-06	8.88652E-05	3.98414	1.500956825
84	NM_009518	Wnt10a	chr1	74792019	74804175	+	28.80039978	17.27460003	-0.585831	4.29919E-17	2.51947E-15	28.8004	1.500903364

Table S3. Validation of RNAseq data. The analysis of expressional changes for select genes was compared by RNAseq and RT-qPCR during *in vivo* decidualization on D8, following an inhibition of PRC1 by PRT4165 against the vehicle control.

Gene targets	Fold changes by RNAseq	Fold changes* by RT-qPCR	Expression status
Ccl27a	3.16	3.27±0.015	Up
Klk15	1.89	7.67±0.039	„
Papss2	2.13	2.34±0.055	„
Rimklb	3.69	28.90±0.035	„
Coch	2.91	2.94±0.074	Down
Cwc22	7.86	7.14±0.023	„
Degs2	3.64	3.70±0.01	„
Ear11	3.48	1.69±0.015	„
Lrat	2.61	1.75±0.012	„
Ptx3	2.74	1.92±0.01	„
Wnt10a	1.50	3.57±0.062	„

*, Significantly different (p<0.05)

Table S4A. Functional enrichment analysis of up-regulated genes by Toppfur

Category	ID	Name	Source	p-value	q-value Bonferroni	q-value FDR B&H	q-value FDR B&Y	Hit Count in Query List	Hit Count in Genome	Hit in Query List
GO: Molecular Function	GO:0008233	peptidase activity		0.00000246	0.00125	0.00125	0.00852	17	618	DPP7,KLK15,NAPSA,MASP1,KLK7,TRABD2B,HTRA1,KLK10,KLK14,CPE,CPZ,GGT1,SLPI,CAPN5,MMP7,KLK1,CTSK
GO: Molecular Function	GO:0070011	peptidase activity, acting on L-amino acid peptides		0.00000722	0.00367	0.00183	0.0125	16	599	DPP7,KLK15,NAPSA,MASP1,KLK7,TRABD2B,HTRA1,KLK10,KLK14,CPE,CPZ,GGT1,CAPN5,MMP7,KLK1,CTSK
GO: Molecular Function	GO:0004175	endopeptidase activity		0.0000298	0.0151	0.00504	0.0343	12	393	KLK15,NAPSA,MASP1,KLK7,TRABD2B,HTRA1,KLK10,KLK14,CAPN5,MMP7,KLK1,CTSK
GO: Molecular Function	GO:0008236	serine-type peptidase activity		0.0000472	0.024	0.0054	0.0368	8	178	DPP7,KLK15,MASP1,KLK7,HTRA1,KLK10,KLK14,KLK1
GO: Molecular Function	GO:0017171	serine hydrolase activity		0.0000532	0.027	0.0054	0.0368	8	181	DPP7,KLK15,MASP1,KLK7,HTRA1,KLK10,KLK14,KLK1
GO: Molecular Function	GO:0004252	serine-type endopeptidase activity		0.00015	0.0759	0.0113	0.0771	7	157	KLK15,MASP1,KLK7,HTRA1,KLK10,KLK14,KLK1
GO: Molecular Function	GO:0031720	haptoglobin binding		0.000156	0.0793	0.0113	0.0771	2	3	HBA2,HBB
GO: Molecular Function	GO:0005509	calcium ion binding		0.00163	0.825	0.0966	0.657	13	694	MASP1,PKD2L1,SPOCK2,CALML5,DYSF,EHD3,TGM2,TCHH,FBLN2,CCDC155,TCHHL1,EDEM2,CLEC3B
GO: Molecular Function	GO:0005539	glycosaminoglycan binding		0.00359	1.82	0.0966	0.657	6	202	SPOCK2,BMP4,LIPG,PRELP,CLEC3B,GPNMB
GO: Molecular Function	GO:0005344	oxygen transporter activity		0.00387	1.97	0.0966	0.657	2	13	HBA2,HBB
GO: Molecular Function	GO:0004601	peroxidase activity		0.00398	2.02	0.0966	0.657	3	44	PXDN,HBA2,HBB
GO: Molecular Function	GO:0016684	oxidoreductase activity, acting on peroxide as acceptor		0.00398	2.02	0.0966	0.657	3	44	PXDN,HBA2,HBB
GO: Molecular Function	GO:0016208	AMP binding		0.00449	2.28	0.0966	0.657	2	14	PRPS2,ACSS1
GO: Molecular Function	GO:0008201	heparin binding		0.00464	2.36	0.0966	0.657	5	149	BMP4,LIPG,PRELP,CLEC3B,GPNMB
GO: Molecular Function	GO:0016594	glycine binding		0.00516	2.62	0.0966	0.657	2	15	GSS,ALAS2
GO: Molecular Function	GO:1901681	sulfur compound binding		0.00656	3.33	0.0966	0.657	6	229	GSS,BMP4,LIPG,PRELP,CLEC3B,GPNMB
GO: Molecular Function	GO:0005198	structural molecule activity		0.00705	3.58	0.0966	0.657	11	641	RPL35A,MYH11,ACTA1,CLDN15,AKAP12,PGM5,SMTN,CRYAA,FBLN2,PXDN,PRELP
GO: Molecular Function	GO:0016843	amine-lyase activity		0.00731	3.71	0.0966	0.657	1	1	APMAP
GO: Molecular Function	GO:0036143	kringle domain binding		0.00731	3.71	0.0966	0.657	1	1	CLEC3B
GO: Molecular Function	GO:0016844	strictosidine synthase activity		0.00731	3.71	0.0966	0.657	1	1	APMAP
GO: Molecular Function	GO:0042658	MHC class II protein binding, via antigen binding groove		0.00731	3.71	0.0966	0.657	1	1	CD74
GO: Molecular Function	GO:0008903	hydroxypyruvate isomerase activity		0.00731	3.71	0.0966	0.657	1	1	HYI
GO: Molecular Function	GO:0046789	host cell surface receptor binding		0.00731	3.71	0.0966	0.657	1	1	INHBB
GO: Molecular Function	GO:0035718	macrophage migration inhibitory factor binding		0.00731	3.71	0.0966	0.657	1	1	CD74
GO: Molecular Function	GO:0031722	hemoglobin beta binding		0.00731	3.71	0.0966	0.657	1	1	HBB
GO: Molecular Function	GO:0008478	pyridoxal kinase activity		0.00731	3.71	0.0966	0.657	1	1	PDXK
GO: Molecular Function	GO:0070280	pyridoxal binding		0.00731	3.71	0.0966	0.657	1	1	PDXK
GO: Molecular Function	GO:0008812	choline dehydrogenase activity		0.00731	3.71	0.0966	0.657	1	1	CHDH
GO: Molecular Function	GO:0046812	host cell surface binding		0.00731	3.71	0.0966	0.657	1	1	INHBB
GO: Molecular Function	GO:0031403	lithium ion binding		0.00731	3.71	0.0966	0.657	1	1	PDXK
GO: Molecular Function	GO:0004363	glutathione synthase activity		0.00731	3.71	0.0966	0.657	1	1	GSS
GO: Molecular Function	GO:0022894	Intermediate conductance calcium-activated potassium channel activity		0.00731	3.71	0.0966	0.657	1	1	KCNN4
GO: Molecular Function	GO:0017172	cysteine dioxygenase activity		0.00731	3.71	0.0966	0.657	1	1	CDO1
GO: Molecular Function	GO:0002153	steroid receptor RNA activator RNA binding		0.00731	3.71	0.0966	0.657	1	1	THRA
GO: Molecular Function	GO:0047394	glycerophosphoinositol inositolphosphodiesterase activity		0.00731	3.71	0.0966	0.657	1	1	GDPD2
GO: Molecular Function	GO:0070678	preprotein binding		0.00731	3.71	0.0966	0.657	1	1	NGFR
GO: Molecular Function	GO:0072591	citrate-L-glutamate ligase activity		0.00731	3.71	0.0966	0.657	1	1	RIMKLB
GO: Molecular Function	GO:0070279	vitamin B6 binding		0.00731	3.71	0.0966	0.657	1	1	PDXK
GO: Molecular Function	GO:0015269	calcium-activated potassium channel activity		0.00741	3.77	0.0966	0.657	2	18	PKD2L1,KCNN4
GO: Molecular Function	GO:0008238	exopeptidase activity		0.00986	5.01	0.125	0.853	4	115	DPP7,CPE,CPZ,GGT1
GO: Molecular Function	GO:0042802	identical protein binding		0.0125	6.32	0.141	0.962	16	1190	PRPS2,MASP1,CDSN,INHBB,PKD2L1,CLDN15,AMICA1,PDXK,GSS,CRYAA,BMP4,THRA,CCDC155,KCNH2,MID1,CD74
GO: Molecular Function	GO:0004181	metallocarboxypeptidase activity		0.013	6.6	0.141	0.962	2	24	CPE,CPZ
GO: Molecular Function	GO:0016755	transferase activity, transferring amino-acyl groups		0.013	6.6	0.141	0.962	2	24	GGT1,TGM2
GO: Molecular Function	GO:0005267	potassium channel activity		0.0131	6.65	0.141	0.962	4	125	PKD2L1,KCNK12,KCNH2,KCNN4
GO: Molecular Function	GO:0005201	extracellular matrix structural constituent		0.0144	7.3	0.141	0.962	3	70	FBLN2,PXDN,PRELP
GO: Molecular Function	GO:0008386	cholesterol monooxygenase (side-chain-cleaving) activity		0.0145	7.35	0.141	0.962	1	2	CYP11A1
GO: Molecular Function	GO:0003870	5-aminolevulinate synthase activity		0.0145	7.35	0.141	0.962	1	2	ALAS2
GO: Molecular Function	GO:0072590	N-acetyl-L-aspartate-L-glutamate ligase activity		0.0145	7.35	0.141	0.962	1	2	RIMKLB
GO: Molecular Function	GO:0004067	asparaginase activity		0.0145	7.35	0.141	0.962	1	2	ASPG
GO: Molecular Function	GO:0004020	adenylylsulfate kinase activity		0.0145	7.35	0.141	0.962	1	2	PAPSS2
GO: Molecular Function	GO:0004781	sulfate adenylyltransferase (ATP) activity		0.0145	7.35	0.141	0.962	1	2	PAPSS2
GO: Molecular Function	GO:0045550	geranylgeranyl reductase activity		0.0145	7.35	0.141	0.962	1	2	AKR1B10
GO: Molecular Function	GO:0016209	antioxidant activity		0.0155	7.87	0.148	1	3	72	PXDN,HBA2,HBB
GO: Molecular Function	GO:0020037	heme binding		0.0161	8.18	0.151	1	4	133	CYP11A1,PXDN,HBA2,HBB
GO: Molecular Function	GO:0042803	protein homodimerization activity		0.018	9.13	0.166	1	11	734	PRPS2,MASP1,CDSN,INHBB,AMICA1,PDXK,GSS,BMP4,THRA,KCNH2,MID1
GO: Molecular Function	GO:0005261	cation channel activity		0.0193	9.78	0.166	1	6	290	JPH2,PKD2L1,KCNK12,KCNH2,KCNN4,SCN3B
GO: Molecular Function	GO:0005227	calcium activated cation channel activity		0.0199	10.1	0.166	1	2	30	PKD2L1,KCNN4
GO: Molecular Function	GO:0046906	tetrapyrrole binding		0.02	10.1	0.166	1	4	142	CYP11A1,PXDN,HBA2,HBB
GO: Molecular Function	GO:0015079	potassium ion transmembrane transporter activity		0.0214	10.9	0.166	1	4	145	PKD2L1,KCNK12,KCNH2,KCNN4
GO: Molecular Function	GO:0070840	dynein complex binding		0.0216	11	0.166	1	1	3	CCDC155
GO: Molecular Function	GO:0005152	interleukin-1 receptor antagonist activity		0.0216	11	0.166	1	1	3	PXDN
GO: Molecular Function	GO:0003987	acetate-CoA ligase activity		0.0216	11	0.166	1	1	3	ACSS1
GO: Molecular Function	GO:0047718	indanol dehydrogenase activity		0.0216	11	0.166	1	1	3	AKR1B10
GO: Molecular Function	GO:0031721	hemoglobin alpha binding		0.0216	11	0.166	1	1	3	HBB
GO: Molecular Function	GO:0004465	lipoprotein lipase activity		0.0216	11	0.166	1	1	3	LIPG
GO: Molecular Function	GO:0033040	sour taste receptor activity		0.0216	11	0.166	1	1	3	PKD2L1
GO: Molecular Function	GO:0016749	N-succinyltransferase activity		0.0225	11.4	0.168	1	2	32	PORCN,ALAS2
GO: Molecular Function	GO:0001540	beta-amyloid binding		0.0225	11.4	0.168	1	2	32	NGFR,CD74
GO: Molecular Function	GO:0016748	succinyltransferase activity		0.0238	12.1	0.176	1	2	33	PORCN,ALAS2
GO: Molecular Function	GO:0043531	ADP binding		0.0266	13.5	0.19	1	2	35	PRPS2,ACTA1
GO: Molecular Function	GO:0005272	sodium channel activity		0.0266	13.5	0.19	1	2	35	PKD2L1,SCN3B
GO: Molecular Function	GO:0016286	small conductance calcium-activated potassium channel activity		0.0287	14.6	0.19	1	1	4	KCNN4
GO: Molecular Function	GO:0004064	arylesterase activity		0.0287	14.6	0.19	1	1	4	APMAP
GO: Molecular Function	GO:1902282	voltage-gated potassium channel activity involved in ventricular cardiac muscle cell action potential repolarization		0.0287	14.6	0.19	1	1	4	KCNH2
GO: Molecular Function	GO:0005497	androgen binding		0.0287	14.6	0.19	1	1	4	TSPO
GO: Molecular Function	GO:0044213	intronic transcription regulatory region DNA binding		0.0287	14.6	0.19	1	1	4	THRA
GO: Molecular Function	GO:0008970	phosphatidylcholine 1-acylhydrolase activity		0.0287	14.6	0.19	1	1	4	LIPG
GO: Molecular Function	GO:0046873	metal ion transmembrane transporter activity		0.0294	14.9	0.19	1	7	408	SLC39A2,JPH2,PKD2L1,KCNK12,KCNH2,KCNN4,SCN3B
GO: Molecular Function	GO:0019825	oxygen binding		0.0295	15	0.19	1	2	37	HBA2,HBB
GO: Molecular Function	GO:0015485	cholesterol binding		0.031	15.8	0.194	1	2	38	CYP11A1,TSPO
GO: Molecular Function	GO:0004180	carboxypeptidase activity		0.031	15.8	0.194	1	2	38	CPE,CPZ

Category	ID	Name	Source	p-value	q-value Bonferroni	q-value FDR B&H	q-value FDR B&Y	Hit Count in Query List	Hit Count in Genome	Hit in Query List
GO: Molecular Function	GO:0022836	gated channel activity		0.0322	16.3	0.199	1	6	327	JPH2,PKD2L1,KCNK12,KCNH2,KCNN4,SCN3B
GO: Molecular Function	GO:0008235	metalloexopeptidase activity		0.0325	16.5	0.199	1	2	39	CPE,CPZ
GO: Molecular Function	GO:0022839	ion gated channel activity		0.0357	18.1	0.199	1	2	41	PKD2L1,KCNN4
GO: Molecular Function	GO:0008307	structural constituent of muscle		0.0357	18.1	0.199	1	2	41	MYH11,SMTN
GO: Molecular Function	GO:0042289	MHC class II protein binding		0.0358	18.2	0.199	1	1	5	CD74
GO: Molecular Function	GO:0003847	1-alkyl-2-acetylglycerophosphocholine esterase activity		0.0358	18.2	0.199	1	1	5	ASPG
GO: Molecular Function	GO:0004614	phosphoglucomutase activity		0.0358	18.2	0.199	1	1	5	PGM5
GO: Molecular Function	GO:0004749	ribose phosphate diphosphokinase activity		0.0358	18.2	0.199	1	1	5	PRPS2
GO: Molecular Function	GO:0086006	voltage-gated sodium channel activity involved in cardiac muscle cell action potential		0.0358	18.2	0.199	1	1	5	SCN3B
GO: Molecular Function	GO:0070324	thyroid hormone binding		0.0358	18.2	0.199	1	1	5	THRA
GO: Molecular Function	GO:0005506	iron ion binding		0.0361	18.3	0.199	1	4	171	CDO1,CYP11A1,HBA2,HBB
GO: Molecular Function	GO:0032934	sterol binding		0.0373	18.9	0.201	1	2	42	CYP11A1,TSPO
GO: Molecular Function	GO:0022803	passive transmembrane transporter activity		0.0383	19.4	0.201	1	7	432	JPH2,PKD2L1,KCNK12,GJB3,KCNH2,KCNN4,SCN3B
GO: Molecular Function	GO:0015267	channel activity		0.0383	19.4	0.201	1	7	432	JPH2,PKD2L1,KCNK12,GJB3,KCNH2,KCNN4,SCN3B
GO: Molecular Function	GO:0044389	ubiquitin-like protein ligase binding		0.0388	19.7	0.201	1	4	175	HLA-DQB1,KCNH2,MID1,NGFR
GO: Molecular Function	GO:0031625	ubiquitin protein ligase binding		0.0388	19.7	0.201	1	4	175	HLA-DQB1,KCNH2,MID1,NGFR
GO: Molecular Function	GO:0005516	calmodulin binding		0.0388	19.7	0.201	1	4	175	PCP4,MYH11,NRGN,KCNN4
GO: Molecular Function	GO:0048406	nerve growth factor binding		0.0428	21.7	0.21	1	1	6	NGFR
GO: Molecular Function	GO:0036374	glutathione hydrolase activity		0.0428	21.7	0.21	1	1	6	GGT1
GO: Molecular Function	GO:0016778	diphosphotransferase activity		0.0428	21.7	0.21	1	1	6	PRPS2
GO: Molecular Function	GO:0005168	neurotrophin TRKA receptor binding		0.0428	21.7	0.21	1	1	6	NGFR
GO: Molecular Function	GO:0008503	benzodiazepine receptor activity		0.0428	21.7	0.21	1	1	6	TSPO
GO: Molecular Function	GO:0008237	metallopeptidase activity		0.043	21.8	0.21	1	4	181	TRABD2B,CPE,CPZ,MMP7
GO: Molecular Function	GO:0070700	BMP receptor binding		0.0497	25.3	0.232	1	1	7	BMP4
GO: Molecular Function	GO:0005167	neurotrophin TRK receptor binding		0.0497	25.3	0.232	1	1	7	NGFR
GO: Molecular Function	GO:0070300	phosphatidic acid binding		0.0497	25.3	0.232	1	1	7	JPH2
GO: Molecular Function	GO:0086008	voltage-gated potassium channel activity involved in cardiac muscle cell action potential repolarization		0.0497	25.3	0.232	1	1	7	KCNH2
GO: Molecular Function	GO:0050897	cobalt ion binding		0.0497	25.3	0.232	1	1	7	CPE
GO: Biological Process	GO:0030198	extracellular matrix organization		0.000124	0.343	0.109	0.927	11	385	DPT,KLK7,MYH11,SPOCK2,BMP4,MFAP4,FBLN2,PXDN,MPZL3,MMP7,CTSK
GO: Biological Process	GO:0043062	extracellular structure organization		0.000127	0.351	0.109	0.927	11	386	DPT,KLK7,MYH11,SPOCK2,BMP4,MFAP4,FBLN2,PXDN,MPZL3,MMP7,CTSK
GO: Biological Process	GO:0042542	response to hydrogen peroxide		0.000149	0.412	0.109	0.927	6	109	CYP11A1,ADA,CRYAA,PXDN,HBA2,HBB
GO: Biological Process	GO:0006508	proteolysis		0.000171	0.473	0.109	0.927	23	1387	DPP7,KLK15,NAPSA,MASP1,KLK7,TRABD2B,HTRA1,KLK10,SPINK1,KLK14,CPE,CPZ,IFI16,GGT1,CRYAA,DERL3,SLPI,NGFR,CAPN5,MMP7,ASB2,KLK1,CTSK
GO: Biological Process	GO:1901566	organonitrogen compound biosynthetic process		0.00022	0.608	0.109	0.927	15	711	PRPS2,CDO1,NPPA,CHDH,PAPSS2,ADA,GGT1,AKAP12,PDXK,GSS,LPIN3,PRELP,TSPO,ALAS2,LARGE
GO: Biological Process	GO:0003012	muscle system process		0.000265	0.734	0.109	0.927	10	352	NPPA,MYH11,ACTA1,MRVI1,ADA,SMTN,KCNH2,LMOD1,FBXO32,SCN3B
GO: Biological Process	GO:0009611	response to wounding		0.0003	0.832	0.109	0.927	21	1255	MUSTN1,CDO1,MASP1,PSAP,PROCR,MRVI1,DYSF,IFI16,PAPSS2,ADA,AMICA1,GGT1,EHD3,ORM1,TGM2,ORM2,TSPO,NGFR,HBB,CX3CL1,CD276
GO: Biological Process	GO:0090191	negative regulation of branching involved in ureteric bud morphogenesis		0.000315	0.872	0.109	0.927	2	4	BMP4,TACSTD2
GO: Biological Process	GO:0002683	negative regulation of immune system process		0.000432	1.2	0.111	0.945	8	244	MASP1,HTRA1,HLA-DQB1,IFI16,ADA,BMP4,CD74,CD276
GO: Biological Process	GO:0002905	regulation of mature B cell apoptotic process		0.000522	1.45	0.111	0.945	2	5	ADA,CD74
GO: Biological Process	GO:0002906	negative regulation of mature B cell apoptotic process		0.000522	1.45	0.111	0.945	2	5	ADA,CD74
GO: Biological Process	GO:0002901	mature B cell apoptotic process		0.000522	1.45	0.111	0.945	2	5	ADA,CD74
GO: Biological Process	GO:0042744	hydrogen peroxide catabolic process		0.00053	1.47	0.111	0.945	3	22	PXDN,HBA2,HBB
GO: Biological Process	GO:0007618	mating		0.000562	1.56	0.111	0.945	4	52	CYP11A1,KLK14,ADA,THRA
GO: Biological Process	GO:0061156	pulmonary artery morphogenesis		0.000779	2.16	0.144	1	2	6	BMP4,STRA6
GO: Biological Process	GO:0048251	elastic fiber assembly		0.00109	3.01	0.156	1	2	7	MYH11,MFAP4
GO: Biological Process	GO:0050862	positive regulation of T cell receptor signaling pathway		0.00109	3.01	0.156	1	2	7	ADA,KCNN4
GO: Biological Process	GO:0072593	reactive oxygen species metabolic process		0.00111	3.08	0.156	1	6	159	PXDN,TSPO,NGFR,HBA2,HBB,PREX1
GO: Biological Process	GO:0009888	tissue development		0.00118	3.26	0.156	1	25	1794	MUSTN1,CDSN,KLK7,MYH11,PSAP,CYP11A1,ACTA1,JPH2,KLK14,CALML5,ADA,GGT1,CRYAA,BMP4,TGM2,TCHH,THRA,NGFR,FOXO4,CLEC3B,GPNMB,STRA6,ASB2,TACSTD2,CD276
GO: Biological Process	GO:0009628	response to abiotic stimulus		0.0013	3.61	0.156	1	17	1025	NPPA,INHBB,CYP11A1,ACTA1,PKD2L1,IFI16,ADA,GGT1,CRYAA,MFAP4,THRA,AKR1B10,TSPO,NGFR,ALAS2,FBXO32,STRA6
GO: Biological Process	GO:0044272	sulfur compound biosynthetic process		0.00135	3.73	0.156	1	6	165	CDO1,ACSS1,PAPSS2,GGT1,GSS,PRELP
GO: Biological Process	GO:0030593	neutrophil chemotaxis		0.00138	3.83	0.156	1	4	66	AMICA1,CD74,CX3CL1,PREX1
GO: Biological Process	GO:0014866	skeletal myofibril assembly		0.00144	3.99	0.156	1	2	8	MYH11,ACTA1
GO: Biological Process	GO:0006936	muscle contraction		0.00148	4.09	0.156	1	8	295	MYH11,ACTA1,MRVI1,ADA,SMTN,KCNH2,LMOD1,SCN3B
GO: Biological Process	GO:1990266	neutrophil migration		0.00154	4.28	0.156	1	4	68	AMICA1,CD74,CX3CL1,PREX1
GO: Biological Process	GO:0000302	response to reactive oxygen species		0.00157	4.34	0.156	1	6	170	CYP11A1,ADA,CRYAA,PXDN,HBA2,HBB
GO: Biological Process	GO:0048739	cardiac muscle fiber development		0.00184	5.11	0.156	1	2	9	MYH11,BMP4
GO: Biological Process	GO:0070244	negative regulation of thymocyte apoptotic process		0.00184	5.11	0.156	1	2	9	ADA,BMP4
GO: Biological Process	GO:0070229	negative regulation of lymphocyte apoptotic process		0.00193	5.35	0.156	1	3	34	ADA,BMP4,CD74
GO: Biological Process	GO:0003254	regulation of membrane depolarization		0.0021	5.82	0.156	1	3	35	TSPO,NGFR,SCN3B
GO: Biological Process	GO:0006534	cysteine metabolic process		0.00229	6.35	0.156	1	2	10	CDO1,GGT1
GO: Biological Process	GO:0071435	potassium ion export		0.00229	6.35	0.156	1	2	10	KCNH2,KCNN4
GO: Biological Process	GO:0009612	response to mechanical stimulus		0.00281	7.79	0.156	1	6	191	NPPA,INHBB,ACTA1,PKD2L1,GGT1,STRA6
GO: Biological Process	GO:0050868	negative regulation of T cell activation		0.00307	8.49	0.156	1	4	82	HLA-DQB1,BMP4,CD74,CD276
GO: Biological Process	GO:0071621	granulocyte chemotaxis		0.00307	8.49	0.156	1	4	82	AMICA1,CD74,CX3CL1,PREX1
GO: Biological Process	GO:0042487	regulation of odontogenesis of dentin-containing tooth		0.00333	9.23	0.156	1	2	12	BMP4,NGFR
GO: Biological Process	GO:0002903	negative regulation of B cell apoptotic process		0.00333	9.23	0.156	1	2	12	ADA,CD74
GO: Biological Process	GO:0002579	positive regulation of antigen processing and presentation		0.00333	9.23	0.156	1	2	12	HLA-DQB1,CD74
GO: Biological Process	GO:0033197	response to vitamin E		0.00333	9.23	0.156	1	2	12	CYP11A1,ADA
GO: Biological Process	GO:0051241	negative regulation of multicellular organismal process		0.00341	9.43	0.156	1	9	414	PROCR,CPE,MRVI1,ADA,BMP4,TSPO,NGFR,TACSTD2,CX3CL1
GO: Biological Process	GO:0006979	response to oxidative stress		0.00342	9.48	0.156	1	8	338	CYP11A1,ADA,GSS,CRYAA,PXDN,NGFR,HBA2,HBB
GO: Biological Process	GO:0051384	response to glucocorticoid		0.00345	9.55	0.156	1	5	138	CDO1,CYP11A1,CRYAA,BMP4,FBXO32
GO: Biological Process	GO:0010035	response to inorganic substance		0.00346	9.58	0.156	1	9	415	CYP11A1,ACTA1,ADA,GSS,CRYAA,PXDN,TSPO,HBA2,HBB
GO: Biological Process	GO:0044273	sulfur compound catabolic process		0.0038	10.5	0.156	1	3	43	CDO1,GGT1,PRELP
GO: Biological Process	GO:0060428	lung epithelium development		0.0038	10.5	0.156	1	3	43	BMP4,THRA,STRA6
GO: Biological Process	GO:0043171	peptide catabolic process		0.00392	10.9	0.156	1	2	13	CPE,GGT1
GO: Biological Process	GO:0050857	positive regulation of antigen receptor-mediated signaling pathway		0.00392	10.9	0.156	1	2	13	ADA,KCNN4

Category	ID	Name	Source	p-value	q-value Bonferroni	q-value FDR B&H	q-value FDR B&Y	Hit Count in Query List	Hit Count in Genome	Hit in Query List
GO: Biological Process	GO:0097530	granulocyte migration		0.00395	11	0.156	1	4	88	AMICA1,CD74,CX3CL1,PREX1
GO: Biological Process	GO:0042743	hydrogen peroxide metabolic process		0.00406	11.2	0.156	1	3	44	PXDN,HBA2,HBB
GO: Biological Process	GO:0043589	skin morphogenesis		0.00432	12	0.156	1	3	45	CDSN,KLK14,NGFR
GO: Biological Process	GO:0010721	negative regulation of cell development		0.00464	12.9	0.156	1	5	148	RTN4R,BMP4,TSPO,NGFR,TACSTD2
GO: Biological Process	GO:0031960	response to corticosteroid		0.00477	13.2	0.156	1	5	149	CDO1,CYP11A1,CRYAA,BMP4,FBXO32
GO: Biological Process	GO:2000241	regulation of reproductive process		0.00481	13.3	0.156	1	4	93	SPINK1,INHBB,ADA,BMP4
GO: Biological Process	GO:0043588	skin development		0.00483	13.4	0.156	1	8	358	CDSN,KLK7,KLK14,CALML5,BMP4,GJB3,TCHH,NGFR
GO: Biological Process	GO:0007155	cell adhesion		0.00489	13.5	0.156	1	16	1067	DPT,CDSN,SPOCK2,CLDN15,ADA,AMICA1,PGM5,TGM2,MFAP4,FBLN2,THRA,MPZL3,GPNMB,TACSTD2,CX3CL1,PREX1
GO: Biological Process	GO:0044057	regulation of system process		0.00491	13.6	0.156	1	9	438	INHBB,MRVI1,ADA,THRA,KCNH2,NGFR,FBXO32,CX3CL1,SCN3B
GO: Biological Process	GO:0015671	oxygen transport		0.00523	14.5	0.156	1	2	15	HBA2,HBB
GO: Biological Process	GO:0022610	biological adhesion		0.00534	14.8	0.156	1	16	1077	DPT,CDSN,SPOCK2,CLDN15,ADA,AMICA1,PGM5,TGM2,MFAP4,FBLN2,THRA,MPZL3,GPNMB,TACSTD2,CX3CL1,PREX1
GO: Biological Process	GO:0006939	smooth muscle contraction		0.00539	14.9	0.156	1	4	96	MYH11,MRVI1,ADA,SMTN
GO: Biological Process	GO:0042130	negative regulation of T cell proliferation		0.00582	16.1	0.156	1	3	50	HLA-DQB1,BMP4,CD276
GO: Biological Process	GO:0044711	single-organism biosynthetic process		0.00584	16.2	0.156	1	20	1491	PRPS2,CDO1,NPPA,CYP11A1,CHDH,ACSS1,PAPSS2,ADA,GGT1,AKAP12,PDXK,PGM5,GSS,LPIN3,BMP4,PRELP,TSPO,CD74,ALAS2,LARGE
GO: Biological Process	GO:0006750	glutathione biosynthetic process		0.00594	16.5	0.156	1	2	16	GGT1,GSS
GO: Biological Process	GO:0070243	regulation of thymocyte apoptotic process		0.00594	16.5	0.156	1	2	16	ADA,BMP4
GO: Biological Process	GO:0090036	regulation of protein kinase C signaling		0.00594	16.5	0.156	1	2	16	AKAP12,SEZ6L
GO: Biological Process	GO:2000107	negative regulation of leukocyte apoptotic process		0.00615	17	0.156	1	3	51	ADA,BMP4,CD74
GO: Biological Process	GO:0006805	xenobiotic metabolic process		0.00643	17.8	0.156	1	5	160	CYP11A1,ACSS1,PAPSS2,GGT1,GSS
GO: Biological Process	GO:0030282	bone mineralization		0.00644	17.8	0.156	1	4	101	BMP4,CLEC3B,GPNMB,CD276
GO: Biological Process	GO:0048286	lung alveolus development		0.00649	18	0.156	1	3	52	ADA,BMP4,STRA6
GO: Biological Process	GO:0071466	cellular response to xenobiotic stimulus		0.00659	18.3	0.156	1	5	161	CYP11A1,ACSS1,PAPSS2,GGT1,GSS
GO: Biological Process	GO:0051900	regulation of mitochondrial depolarization		0.0067	18.6	0.156	1	2	17	TSPO,NGFR
GO: Biological Process	GO:0019184	nonribosomal peptide biosynthetic process		0.0067	18.6	0.156	1	2	17	GGT1,GSS
GO: Biological Process	GO:0002577	regulation of antigen processing and presentation		0.0067	18.6	0.156	1	2	17	HLA-DQB1,CD74
GO: Biological Process	GO:0051150	regulation of smooth muscle cell differentiation		0.0067	18.6	0.156	1	2	17	BMP4,FOXO4
GO: Biological Process	GO:0042398	cellular modified amino acid biosynthetic process		0.00684	19	0.156	1	3	53	CHDH,GGT1,GSS
GO: Biological Process	GO:0046148	pigment biosynthetic process		0.00684	19	0.156	1	3	53	ADA,TSPO,ALAS2
GO: Biological Process	GO:0048545	response to steroid hormone		0.00695	19.3	0.156	1	8	381	CDO1,CYP11A1,ACTA1,CRYAA,BMP4,THRA,TSPO,FBXO32
GO: Biological Process	GO:0048872	homeostasis of number of cells		0.00734	20.3	0.156	1	6	233	ADA,BMP4,THRA,CD74,ALAS2,HBA2
GO: Biological Process	GO:0071893	BMP signaling pathway involved in nephric duct formation		0.00736	20.4	0.156	1	1	1	BMP4
GO: Biological Process	GO:0090172	microtubule cytoskeleton organization involved in homologous chromosome segregation		0.00736	20.4	0.156	1	1	1	CCDC155
GO: Biological Process	GO:0046090	deoxyadenosine metabolic process		0.00736	20.4	0.156	1	1	1	ADA
GO: Biological Process	GO:0048390	intermediate mesoderm morphogenesis		0.00736	20.4	0.156	1	1	1	BMP4
GO: Biological Process	GO:0048391	intermediate mesoderm formation		0.00736	20.4	0.156	1	1	1	BMP4
GO: Biological Process	GO:0032605	hepatocyte growth factor production		0.00736	20.4	0.156	1	1	1	INHBB
GO: Biological Process	GO:0061155	pulmonary artery endothelial tube morphogenesis		0.00736	20.4	0.156	1	1	1	BMP4
GO: Biological Process	GO:2000006	regulation of metanephric comma-shaped body morphogenesis		0.00736	20.4	0.156	1	1	1	BMP4
GO: Biological Process	GO:0061149	BMP signaling pathway involved in ureter morphogenesis		0.00736	20.4	0.156	1	1	1	BMP4
GO: Biological Process	GO:1902302	regulation of potassium ion export		0.00736	20.4	0.156	1	1	1	KCNH2
GO: Biological Process	GO:2000005	negative regulation of metanephric S-shaped body morphogenesis		0.00736	20.4	0.156	1	1	1	BMP4
GO: Biological Process	GO:0072096	negative regulation of branch elongation involved in ureteric bud branching		0.00736	20.4	0.156	1	1	1	BMP4
GO: Biological Process	GO:2000004	regulation of metanephric S-shaped body morphogenesis		0.00736	20.4	0.156	1	1	1	BMP4
GO: Biological Process	GO:0060994	regulation of transcription from RNA polymerase II promoter involved in kidney development		0.00736	20.4	0.156	1	1	1	BMP4
GO: Biological Process	GO:0090220	chromosome localization to nuclear envelope involved in homologous chromosome segregation		0.00736	20.4	0.156	1	1	1	CCDC155
GO: Biological Process	GO:0061216	regulation of transcription from RNA polymerase II promoter involved in mesonephros development		0.00736	20.4	0.156	1	1	1	BMP4
GO: Biological Process	GO:0048178	negative regulation of hepatocyte growth factor biosynthetic process		0.00736	20.4	0.156	1	1	1	INHBB
GO: Biological Process	GO:0072200	negative regulation of mesenchymal cell proliferation involved in ureter development		0.00736	20.4	0.156	1	1	1	BMP4
GO: Biological Process	GO:0002776	antimicrobial peptide secretion		0.00736	20.4	0.156	1	1	1	MMP7
GO: Biological Process	GO:0071938	vitamin A transport		0.00736	20.4	0.156	1	1	1	STRA6
GO: Biological Process	GO:0002779	antibacterial peptide secretion		0.00736	20.4	0.156	1	1	1	MMP7
GO: Biological Process	GO:0061151	BMP signaling pathway involved in renal system segmentation		0.00736	20.4	0.156	1	1	1	BMP4
GO: Biological Process	GO:2000137	negative regulation of cell proliferation involved in heart morphogenesis		0.00736	20.4	0.156	1	1	1	BMP4
GO: Biological Process	GO:0006751	glutathione catabolic process		0.00736	20.4	0.156	1	1	1	GGT1
GO: Biological Process	GO:0072101	specification of ureteric bud anterior/posterior symmetry by BMP signaling pathway		0.00736	20.4	0.156	1	1	1	BMP4
GO: Biological Process	GO:0072192	ureter epithelial cell differentiation		0.00736	20.4	0.156	1	1	1	BMP4
GO: Biological Process	GO:0061143	alveolar primary septum development		0.00736	20.4	0.156	1	1	1	STRA6
GO: Biological Process	GO:0002343	peripheral B cell selection		0.00736	20.4	0.156	1	1	1	HLA-DQB1
GO: Biological Process	GO:2000007	negative regulation of metanephric comma-shaped body morphogenesis		0.00736	20.4	0.156	1	1	1	BMP4
GO: Biological Process	GO:0048392	intermediate mesodermal cell differentiation		0.00736	20.4	0.156	1	1	1	BMP4
GO: Biological Process	GO:0055020	positive regulation of cardiac muscle fiber development		0.00736	20.4	0.156	1	1	1	BMP4
GO: Biological Process	GO:0055018	regulation of cardiac muscle fiber development		0.00736	20.4	0.156	1	1	1	BMP4
GO: Biological Process	GO:0072097	negative regulation of branch elongation involved in ureteric bud branching by BMP signaling pathway		0.00736	20.4	0.156	1	1	1	BMP4
GO: Biological Process	GO:1902303	negative regulation of potassium ion export		0.00736	20.4	0.156	1	1	1	KCNH2
GO: Biological Process	GO:0060407	negative regulation of penile erection		0.00736	20.4	0.156	1	1	1	ADA
GO: Biological Process	GO:0048175	hepatocyte growth factor biosynthetic process		0.00736	20.4	0.156	1	1	1	INHBB
GO: Biological Process	GO:0009443	pyridoxal 5'-phosphate salvage		0.00736	20.4	0.156	1	1	1	PDXK
GO: Biological Process	GO:0072099	anterior/posterior pattern specification involved in ureteric bud development		0.00736	20.4	0.156	1	1	1	BMP4
GO: Biological Process	GO:0061150	renal system segmentation		0.00736	20.4	0.156	1	1	1	BMP4
GO: Biological Process	GO:0060503	bud dilation involved in lung branching		0.00736	20.4	0.156	1	1	1	BMP4
GO: Biological Process	GO:0032646	regulation of hepatocyte growth factor production		0.00736	20.4	0.156	1	1	1	INHBB
GO: Biological Process	GO:0070684	seminal clot liquefaction		0.00736	20.4	0.156	1	1	1	KLK14
GO: Biological Process	GO:0060169	negative regulation of adenosine receptor signaling pathway		0.00736	20.4	0.156	1	1	1	ADA

Category	ID	Name	Source	p-value	q-value Bonferroni	q-value FDR B&H	q-value FDR B&Y	Hit Count in Query List	Hit Count in Genome	Hit in Query List
GO: Biological Process	GO:0046111	xanthine biosynthetic process		0.00736	20.4	0.156	1	1	1	ADA
GO: Biological Process	GO:0071939	vitamin A import		0.00736	20.4	0.156	1	1	1	STRA6
GO: Biological Process	GO:0072100	specification of ureteric bud anterior/posterior symmetry		0.00736	20.4	0.156	1	1	1	BMP4
GO: Biological Process	GO:0002344	B cell affinity maturation		0.00736	20.4	0.156	1	1	1	HLA-DQB1
GO: Biological Process	GO:0006157	deoxyadenosine catabolic process		0.00736	20.4	0.156	1	1	1	ADA
GO: Biological Process	GO:0051041	positive regulation of calcium-independent cell-cell adhesion		0.00736	20.4	0.156	1	1	1	CX3CL1
GO: Biological Process	GO:0035691	macrophage migration inhibitory factor signaling pathway		0.00736	20.4	0.156	1	1	1	CD74
GO: Biological Process	GO:0048176	regulation of hepatocyte growth factor biosynthetic process		0.00736	20.4	0.156	1	1	1	INHBB
GO: Biological Process	GO:0090194	negative regulation of glomerulus development		0.00736	20.4	0.156	1	1	1	BMP4
GO: Biological Process	GO:0071549	cellular response to dexamethasone stimulus		0.00751	20.8	0.156	1	2	18	BMP4,FBXO32
GO: Biological Process	GO:0085029	extracellular matrix assembly		0.00751	20.8	0.156	1	2	18	MYH11,MFAP4
GO: Biological Process	GO:0051882	mitochondrial depolarization		0.00751	20.8	0.156	1	2	18	TSPO,NGFR
GO: Biological Process	GO:0032456	endocytic recycling		0.00751	20.8	0.156	1	2	18	EHD3,ARL4C
GO: Biological Process	GO:0009410	response to xenobiotic stimulus		0.00767	21.2	0.159	1	5	167	CYP11A1,ACSS1,PAPSS2,GGT1,GSS
GO: Biological Process	GO:0030218	erythrocyte differentiation		0.00787	21.8	0.162	1	4	107	BMP4,THRA,ALAS2,HBA2
GO: Biological Process	GO:0031347	regulation of defense response		0.00827	22.9	0.165	1	10	562	MASP1,HTRA1,IFI16,ADA,GGT1,TGM2,CD74,CX3CL1,CTSK,CD276
GO: Biological Process	GO:0002902	regulation of B cell apoptotic process		0.00835	23.1	0.165	1	2	19	ADA,CD74
GO: Biological Process	GO:0086005	ventricular cardiac muscle cell action potential		0.00835	23.1	0.165	1	2	19	KCNH2,SCN3B
GO: Biological Process	GO:0034766	negative regulation of ion transmembrane transport		0.00835	23.1	0.165	1	2	19	SPINK1,KCNH2
GO: Biological Process	GO:0060688	regulation of morphogenesis of a branching structure		0.00837	23.2	0.165	1	3	57	CPE,BMP4,TACSTD2
GO: Biological Process	GO:0050768	negative regulation of neurogenesis		0.00839	23.2	0.165	1	4	109	RTN4R,BMP4,TSPO,NGFR
GO: Biological Process	GO:0008544	epidermis development		0.00861	23.9	0.168	1	7	316	CDSN,KLK7,KLK14,CALML5,BMP4,TCHH,NGFR
GO: Biological Process	GO:0070228	regulation of lymphocyte apoptotic process		0.00878	24.3	0.17	1	3	58	ADA,BMP4,CD74
GO: Biological Process	GO:0051051	negative regulation of transport		0.00893	24.7	0.171	1	8	398	SPINK1,INHBB,ADA,CRYAA,THRA,KCNH2,CD74,CX3CL1
GO: Biological Process	GO:0071214	cellular response to abiotic stimulus		0.00911	25.2	0.171	1	6	244	NPPA,PKD2L1,IFI16,GGT1,MFAP4,TSPO
GO: Biological Process	GO:0009991	response to extracellular stimulus		0.00918	25.4	0.171	1	8	400	INHBB,CYP11A1,ACTA1,IFI16,ADA,GSS,LIPG,TSPO
GO: Biological Process	GO:0008652	cellular amino acid biosynthetic process		0.00921	25.5	0.171	1	4	112	CDO1,CHDH,GGT1,GSS
GO: Biological Process	GO:0071548	response to dexamethasone		0.00923	25.6	0.171	1	2	20	BMP4,FBXO32
GO: Biological Process	GO:0010460	positive regulation of heart rate		0.00923	25.6	0.171	1	2	20	ADA,SCN3B
GO: Biological Process	GO:0045785	positive regulation of cell adhesion		0.00928	25.7	0.171	1	5	175	SPOCK2,TGM2,FBLN2,CX3CL1,PREX1
GO: Biological Process	GO:0051250	negative regulation of lymphocyte activation		0.0095	26.3	0.174	1	4	113	HLA-DQB1,BMP4,CD74,CD276
GO: Biological Process	GO:1901654	response to ketone		0.00979	27.1	0.178	1	4	114	CYP11A1,BMP4,TSPO,FBXO32
GO: Biological Process	GO:0050878	regulation of body fluid levels		0.0101	28	0.18	1	11	670	CDO1,PSAP,PROCR,MRVI1,PAPSS2,ADA,AMICA1,EHD3,KCNN4,HBB,CX3CL1
GO: Biological Process	GO:0034101	erythrocyte homeostasis		0.0104	28.8	0.18	1	4	116	BMP4,THRA,ALAS2,HBA2
GO: Biological Process	GO:0090257	regulation of muscle system process		0.0104	28.8	0.18	1	5	180	MRVI1,ADA,KCNH2,FBXO32,SCN3B
GO: Biological Process	GO:0050672	negative regulation of lymphocyte proliferation		0.0105	29.2	0.18	1	3	62	HLA-DQB1,BMP4,CD276
GO: Biological Process	GO:0032945	negative regulation of mononuclear cell proliferation		0.0105	29.2	0.18	1	3	62	HLA-DQB1,BMP4,CD276
GO: Biological Process	GO:0045669	positive regulation of osteoblast differentiation		0.011	30.5	0.18	1	3	63	IFI16,BMP4,CD276
GO: Biological Process	GO:0048661	positive regulation of smooth muscle cell proliferation		0.011	30.5	0.18	1	3	63	BMP4,TGM2,CX3CL1
GO: Biological Process	GO:0000097	sulfur amino acid biosynthetic process		0.0111	30.8	0.18	1	2	22	CDO1,GGT1
GO: Biological Process	GO:0070242	thymocyte apoptotic process		0.0111	30.8	0.18	1	2	22	ADA,BMP4
GO: Biological Process	GO:0050670	regulation of lymphocyte proliferation		0.0116	32.1	0.18	1	5	185	HLA-DQB1,ADA,BMP4,CD74,CD276
GO: Biological Process	GO:0032963	collagen metabolic process		0.0117	32.3	0.18	1	4	120	BMP4,MFAP4,MMP7,CTSK
GO: Biological Process	GO:0032944	regulation of mononuclear cell proliferation		0.0119	32.8	0.18	1	5	186	HLA-DQB1,ADA,BMP4,CD74,CD276
GO: Biological Process	GO:0070664	negative regulation of leukocyte proliferation		0.012	33.2	0.18	1	3	65	HLA-DQB1,BMP4,CD276
GO: Biological Process	GO:0048745	smooth muscle tissue development		0.0121	33.6	0.18	1	2	23	BMP4,STRA6
GO: Biological Process	GO:0006783	heme biosynthetic process		0.0121	33.6	0.18	1	2	23	TSPO,ALAS2
GO: Biological Process	GO:0015669	gas transport		0.0121	33.6	0.18	1	2	23	HBA2,HBB
GO: Biological Process	GO:0034763	negative regulation of transmembrane transport		0.0121	33.6	0.18	1	2	23	SPINK1,KCNH2
GO: Biological Process	GO:0070233	negative regulation of T cell apoptotic process		0.0121	33.6	0.18	1	2	23	ADA,BMP4
GO: Biological Process	GO:0086091	regulation of heart rate by cardiac conduction		0.0121	33.6	0.18	1	2	23	KCNH2,SCN3B
GO: Biological Process	GO:0006954	inflammatory response		0.0126	34.8	0.18	1	10	599	CDO1,MASP1,IFI16,ADA,GGT1,ORM1,TGM2,ORM2,CX3CL1,CD276
GO: Biological Process	GO:0048732	gland development		0.0127	35.2	0.18	1	8	424	CDO1,PSAP,ADA,BMP4,TGM2,THRA,TSPO,STRA6
GO: Biological Process	GO:0090189	regulation of branching involved in ureteric bud morphogenesis		0.0132	36.5	0.18	1	2	24	BMP4,TACSTD2
GO: Biological Process	GO:0001783	B cell apoptotic process		0.0132	36.5	0.18	1	2	24	ADA,CD74
GO: Biological Process	GO:0070663	regulation of leukocyte proliferation		0.0132	36.5	0.18	1	5	191	HLA-DQB1,ADA,BMP4,CD74,CD276
GO: Biological Process	GO:0031348	negative regulation of defense response		0.0134	37	0.18	1	4	125	HTRA1,IFI16,ADA,CD276
GO: Biological Process	GO:0030155	regulation of cell adhesion		0.0135	37.3	0.18	1	7	345	SPOCK2,ADA,TGM2,FBLN2,TACSTD2,CX3CL1,PREX1
GO: Biological Process	GO:0002821	positive regulation of adaptive immune response		0.0135	37.5	0.18	1	3	68	HLA-DQB1,ADA,CD74
GO: Biological Process	GO:0044259	multicellular organismal macromolecule metabolic process		0.0137	38.1	0.18	1	4	126	BMP4,MFAP4,MMP7,CTSK
GO: Biological Process	GO:0097529	myeloid leukocyte migration		0.0137	38.1	0.18	1	4	126	AMICA1,CD74,CX3CL1,PREX1
GO: Biological Process	GO:0006790	sulfur compound metabolic process		0.0139	38.4	0.18	1	7	347	CDO1,ACSS1,SPOCK2,PAPSS2,GGT1,GSS,PRELP
GO: Biological Process	GO:0050727	regulation of inflammatory response		0.014	38.7	0.18	1	6	268	MASP1,ADA,GGT1,TGM2,CX3CL1,CD276
GO: Biological Process	GO:0045581	negative regulation of T cell differentiation		0.0142	39.4	0.18	1	2	25	BMP4,CD74
GO: Biological Process	GO:0046864	isoprenoid transport		0.0146	40.3	0.18	1	1	2	STRA6
GO: Biological Process	GO:0019413	acetate biosynthetic process		0.0146	40.3	0.18	1	1	2	ACSS1
GO: Biological Process	GO:0008050	female courtship behavior		0.0146	40.3	0.18	1	1	2	THRA
GO: Biological Process	GO:0042823	pyridoxal phosphate biosynthetic process		0.0146	40.3	0.18	1	1	2	PDXK
GO: Biological Process	GO:0046124	purine deoxyribonucleoside catabolic process		0.0146	40.3	0.18	1	1	2	ADA
GO: Biological Process	GO:0034633	retinol transport		0.0146	40.3	0.18	1	1	2	STRA6
GO: Biological Process	GO:0051040	regulation of calcium-independent cell-cell adhesion		0.0146	40.3	0.18	1	1	2	CX3CL1
GO: Biological Process	GO:0018262	isopeptide cross-linking		0.0146	40.3	0.18	1	1	2	TGM2
GO: Biological Process	GO:0010983	positive regulation of high-density lipoprotein particle clearance		0.0146	40.3	0.18	1	1	2	LIPG
GO: Biological Process	GO:0043503	skeletal muscle fiber adaptation		0.0146	40.3	0.18	1	1	2	ACTA1
GO: Biological Process	GO:0061047	positive regulation of branching involved in lung morphogenesis		0.0146	40.3	0.18	1	1	2	BMP4
GO: Biological Process	GO:2000173	negative regulation of branching morphogenesis of a nerve		0.0146	40.3	0.18	1	1	2	CPE
GO: Biological Process	GO:0019452	L-cysteine catabolic process to taurine		0.0146	40.3	0.18	1	1	2	CDO1
GO: Biological Process	GO:0019285	glycine betaine biosynthetic process from choline		0.0146	40.3	0.18	1	1	2	CHDH
GO: Biological Process	GO:0017055	negative regulation of RNA polymerase II transcriptional preinitiation complex assembly		0.0146	40.3	0.18	1	1	2	THRA
GO: Biological Process	GO:0002314	germinal center B cell differentiation		0.0146	40.3	0.18	1	1	2	ADA
GO: Biological Process	GO:0018153	isopeptide cross-linking via N6-(L-isoglutamyl)-L-lysine		0.0146	40.3	0.18	1	1	2	TGM2
GO: Biological Process	GO:0070256	negative regulation of mucus secretion		0.0146	40.3	0.18	1	1	2	ADA

Category	ID	Name	Source	p-value	q-value Bonferroni	q-value FDR B&H	q-value FDR B&Y	Hit Count in Query List	Hit Count in Genome	Hit in Query List
GO: Biological Process	GO:0042488	positive regulation of odontogenesis of dentin-containing tooth		0.0146	40.3	0.18	1	1	2	NGFR
GO: Biological Process	GO:0042412	taurine biosynthetic process		0.0146	40.3	0.18	1	1	2	CDO1
GO: Biological Process	GO:0060427	lung connective tissue development		0.0146	40.3	0.18	1	1	2	GGT1
GO: Biological Process	GO:0031456	glycine betaine biosynthetic process		0.0146	40.3	0.18	1	1	2	CHDH
GO: Biological Process	GO:0042323	negative regulation of circadian sleep/wake cycle, non-REM sleep		0.0146	40.3	0.18	1	1	2	ADA
GO: Biological Process	GO:0046110	xanthine metabolic process		0.0146	40.3	0.18	1	1	2	ADA
GO: Biological Process	GO:0016488	farnesol catabolic process		0.0146	40.3	0.18	1	1	2	AKR1B10
GO: Biological Process	GO:0016487	farnesol metabolic process		0.0146	40.3	0.18	1	1	2	AKR1B10
GO: Biological Process	GO:0050902	leukocyte adhesive activation		0.0146	40.3	0.18	1	1	2	CX3CL1
GO: Biological Process	GO:0034230	enkephalin processing		0.0146	40.3	0.18	1	1	2	CPE
GO: Biological Process	GO:0016107	sesquiterpenoid catabolic process		0.0146	40.3	0.18	1	1	2	AKR1B10
GO: Biological Process	GO:0003064	regulation of heart rate by hormone		0.0146	40.3	0.18	1	1	2	KCNH2
GO: Biological Process	GO:0019542	propionate biosynthetic process		0.0146	40.3	0.18	1	1	2	ACSS1
GO: Biological Process	GO:0019427	acetyl-CoA biosynthetic process from acetate		0.0146	40.3	0.18	1	1	2	ACSS1
GO: Biological Process	GO:0010751	negative regulation of nitric oxide mediated signal transduction		0.0146	40.3	0.18	1	1	2	SPINK1
GO: Biological Process	GO:0046865	terpenoid transport		0.0146	40.3	0.18	1	1	2	STRA6
GO: Biological Process	GO:0010266	response to vitamin B1		0.0146	40.3	0.18	1	1	2	TSPO
GO: Biological Process	GO:0048389	intermediate mesoderm development		0.0146	40.3	0.18	1	1	2	BMP4
GO: Biological Process	GO:0031455	glycine betaine metabolic process		0.0146	40.3	0.18	1	1	2	CHDH
GO: Biological Process	GO:0061227	pattern specification involved in mesonephros development		0.0146	40.3	0.18	1	1	2	BMP4
GO: Biological Process	GO:0072098	anterior/posterior pattern specification involved in kidney development		0.0146	40.3	0.18	1	1	2	BMP4
GO: Biological Process	GO:0030322	stabilization of membrane potential		0.0146	40.3	0.18	1	1	2	KCNN4
GO: Biological Process	GO:0070301	cellular response to hydrogen peroxide		0.0146	40.5	0.18	1	3	70	PXDN,HBA2,HBB
GO: Biological Process	GO:0043500	muscle adaptation		0.0152	42.1	0.18	1	3	71	NPPA,ACTA1,FBXO32
GO: Biological Process	GO:0031214	biomineral tissue development		0.0153	42.2	0.18	1	4	130	BMP4,CLEC3B,GPNMB,CD276
GO: Biological Process	GO:0030325	adrenal gland development		0.0154	42.5	0.18	1	2	26	TSPO,STRA6
GO: Biological Process	GO:0002089	lens morphogenesis in camera-type eye		0.0154	42.5	0.18	1	2	26	CRYAA,BMP4
GO: Biological Process	GO:1900024	regulation of substrate adhesion-dependent cell spreading		0.0154	42.5	0.18	1	2	26	TACSTD2,PREX1
GO: Biological Process	GO:0042481	regulation of odontogenesis		0.0154	42.5	0.18	1	2	26	BMP4,NGFR
GO: Biological Process	GO:0050856	regulation of T cell receptor signaling pathway		0.0154	42.5	0.18	1	2	26	ADA,KCNN4
GO: Biological Process	GO:0033081	regulation of T cell differentiation in thymus		0.0154	42.5	0.18	1	2	26	ADA,BMP4
GO: Biological Process	GO:0007617	mating behavior		0.0154	42.5	0.18	1	2	26	CYP11A1,THRA
GO: Biological Process	GO:0016338	calcium-independent cell-cell adhesion via plasma membrane cell-adhesion molecules		0.0154	42.5	0.18	1	2	26	CLDN15,CX3CL1
GO: Biological Process	GO:0008015	blood circulation		0.0154	42.6	0.18	1	8	439	NPPA,MRVI1,ADA,THRA,KCNH2,HBB,CX3CL1,SCN3B
GO: Biological Process	GO:0030324	lung development		0.0155	42.9	0.18	1	5	199	ADA,GGT1,BMP4,THRA,STRA6
GO: Biological Process	GO:0002682	regulation of immune system process		0.0155	42.9	0.18	1	16	1212	MASP1,HTRA1,HLA-DQB1,IFI16,ADA,AMICA1,GGT1,BMP4,ORM1,ORM2,KCNN4,CD74,FOXO4,CX3CL1,CTSK,CD276
GO: Biological Process	GO:0002695	negative regulation of leukocyte activation		0.0156	43.3	0.181	1	4	131	HLA-DQB1,BMP4,CD74,CD276
GO: Biological Process	GO:0003013	circulatory system process		0.0158	43.7	0.182	1	8	441	NPPA,MRVI1,ADA,THRA,KCNH2,HBB,CX3CL1,SCN3B
GO: Biological Process	GO:0051899	membrane depolarization		0.016	44.4	0.184	1	4	132	KCNH2,TSPO,NGFR,SCN3B
GO: Biological Process	GO:0042440	pigment metabolic process		0.0164	45.3	0.185	1	3	73	ADA,TSPO,ALAS2
GO: Biological Process	GO:0003008	system process		0.0164	45.5	0.185	1	23	1978	NPPA,MYH11,INHBB,ACTA1,PKD2L1,PAK6,MRVI1,ADA,SMTN,CRYAA,BMP4,GJB3,THRA,KCNH2,NGFR,KCNN4,LMOD1,FBXO32,STRA6,HBB,TACSTD2,CX3CL1,SCN3B
GO: Biological Process	GO:1901685	glutathione derivative metabolic process		0.0165	45.7	0.185	1	2	27	GGT1,GSS
GO: Biological Process	GO:0045823	positive regulation of heart contraction		0.0165	45.7	0.185	1	2	27	ADA,SCN3B
GO: Biological Process	GO:1901687	glutathione derivative biosynthetic process		0.0165	45.7	0.185	1	2	27	GGT1,GSS
GO: Biological Process	GO:0061217	regulation of mesonephros development		0.0165	45.7	0.185	1	2	27	BMP4,TACSTD2
GO: Biological Process	GO:0030323	respiratory tube development		0.0167	46.4	0.187	1	5	203	ADA,GGT1,BMP4,THRA,STRA6
GO: Biological Process	GO:0070227	lymphocyte apoptotic process		0.017	47	0.189	1	3	74	ADA,BMP4,CD74
GO: Biological Process	GO:0042036	negative regulation of cytokine biosynthetic process		0.0177	49	0.192	1	2	28	INHBB,CD276
GO: Biological Process	GO:0086002	cardiac muscle cell action potential involved in contraction		0.0177	49	0.192	1	2	28	KCNH2,SCN3B
GO: Biological Process	GO:0070528	protein kinase C signaling		0.0177	49	0.192	1	2	28	AKAP12,SEZ6L
GO: Biological Process	GO:0043043	peptide biosynthetic process		0.0177	49	0.192	1	2	28	GGT1,GSS
GO: Biological Process	GO:0030224	monocyte differentiation		0.0177	49	0.192	1	2	28	IFI16,BMP4
GO: Biological Process	GO:0007620	copulation		0.0177	49	0.192	1	2	28	KLK14,ADA
GO: Biological Process	GO:0035270	endocrine system development		0.0181	50.2	0.192	1	4	137	BMP4,THRA,TSPO,STRA6
GO: Biological Process	GO:0007517	muscle organ development		0.0186	51.4	0.192	1	7	368	ACTA1,JPH2,SMTN,BMP4,FOXO4,STRA6,ASB2
GO: Biological Process	GO:0002262	myeloid cell homeostasis		0.0186	51.4	0.192	1	4	138	BMP4,THRA,ALAS2,HBA2
GO: Biological Process	GO:0006779	porphyrin-containing compound biosynthetic process		0.0189	52.4	0.192	1	2	29	TSPO,ALAS2
GO: Biological Process	GO:0097305	response to alcohol		0.0192	53.3	0.192	1	6	288	CDO1,INHBB,CYP11A1,BMP4,TSPO,FBXO32
GO: Biological Process	GO:0031667	response to nutrient levels		0.0193	53.5	0.192	1	7	371	INHBB,CYP11A1,IFI16,ADA,GSS,LIPG,TSPO
GO: Biological Process	GO:0050866	negative regulation of cell activation		0.0199	55.2	0.192	1	4	141	HLA-DQB1,BMP4,CD74,CD276
GO: Biological Process	GO:0010810	regulation of cell-substrate adhesion		0.0199	55.2	0.192	1	4	141	SPOCK2,FBLN2,TACSTD2,PREX1
GO: Biological Process	GO:0044236	multicellular organismal metabolic process		0.0199	55.2	0.192	1	4	141	BMP4,MFAP4,MMP7,CTSK
GO: Biological Process	GO:0002027	regulation of heart rate		0.0201	55.8	0.192	1	3	79	ADA,KCNH2,SCN3B
GO: Biological Process	GO:0048596	embryonic camera-type eye morphogenesis		0.0202	55.8	0.192	1	2	30	CRYAA,STRA6
GO: Biological Process	GO:0018149	peptide cross-linking		0.0202	55.8	0.192	1	2	30	SPOCK2,TGM2
GO: Biological Process	GO:0001503	ossification		0.0203	56.3	0.192	1	7	375	IFI16,BMP4,THRA,CLEC3B,GPNMB,CTSK,CD276
GO: Biological Process	GO:0006937	regulation of muscle contraction		0.0204	56.5	0.192	1	4	142	MRVI1,ADA,KCNH2,SCN3B
GO: Biological Process	GO:0033273	response to vitamin		0.0208	57.7	0.192	1	3	80	CYP11A1,ADA,TSPO
GO: Biological Process	GO:0033077	T cell differentiation in thymus		0.0208	57.7	0.192	1	3	80	ADA,BMP4,CD74
GO: Biological Process	GO:0048747	muscle fiber development		0.0208	57.7	0.192	1	3	80	MYH11,ACTA1,BMP4
GO: Biological Process	GO:0051188	cofactor biosynthetic process		0.0213	59.1	0.192	1	4	144	ACSS1,PDXK,TSPO,ALAS2
GO: Biological Process	GO:0061061	muscle structure development		0.0214	59.2	0.192	1	9	558	MYH11,ACTA1,JPH2,SMTN,BMP4,THRA,FOXO4,STRA6,ASB2
GO: Biological Process	GO:0014888	striated muscle adaptation		0.0215	59.4	0.192	1	2	31	NPPA,ACTA1
GO: Biological Process	GO:0051155	positive regulation of striated muscle cell differentiation		0.0215	59.4	0.192	1	2	31	BMP4,THRA
GO: Biological Process	GO:0097306	cellular response to alcohol		0.0215	59.6	0.192	1	3	81	INHBB,BMP4,FBXO32
GO: Biological Process	GO:0035993	deltoid tuberosity development		0.0218	60.3	0.192	1	1	3	BMP4
GO: Biological Process	GO:0002155	regulation of thyroid hormone mediated signaling pathway		0.0218	60.3	0.192	1	1	3	THRA
GO: Biological Process	GO:0010982	regulation of high-density lipoprotein particle clearance		0.0218	60.3	0.192	1	1	3	LIPG
GO: Biological Process	GO:0010159	specification of organ position		0.0218	60.3	0.192	1	1	3	BMP4
GO: Biological Process	GO:0007500	mesodermal cell fate determination		0.0218	60.3	0.192	1	1	3	BMP4
GO: Biological Process	GO:0000103	sulfate assimilation		0.0218	60.3	0.192	1	1	3	PAPSS2
GO: Biological Process	GO:0031179	peptide modification		0.0218	60.3	0.192	1	1	3	GGT1
GO: Biological Process	GO:0003133	endodermal-mesodermal cell signaling		0.0218	60.3	0.192	1	1	3	BMP4
GO: Biological Process	GO:2000143	negative regulation of DNA-templated transcription, initiation		0.0218	60.3	0.192	1	1	3	THRA
GO: Biological Process	GO:0072125	negative regulation of glomerular mesangial cell proliferation		0.0218	60.3	0.192	1	1	3	BMP4

Category	ID	Name	Source	p-value	q-value Bonferroni	q-value FDR B&H	q-value FDR B&Y	Hit Count in Query List	Hit Count in Genome	Hit in Query List
GO: Biological Process	GO:0030070	insulin processing		0.0218	60.3	0.192	1	1	3	CPE
GO: Biological Process	GO:0001581	detection of chemical stimulus involved in sensory perception of sour taste		0.0218	60.3	0.192	1	1	3	PKD2L1
GO: Biological Process	GO:0072190	ureter urothelium development		0.0218	60.3	0.192	1	1	3	BMP4
GO: Biological Process	GO:0002778	antibacterial peptide production		0.0218	60.3	0.192	1	1	3	MMP7
GO: Biological Process	GO:0072138	mesenchymal cell proliferation involved in ureteric bud development		0.0218	60.3	0.192	1	1	3	BMP4
GO: Biological Process	GO:0006154	adenosine catabolic process		0.0218	60.3	0.192	1	1	3	ADA
GO: Biological Process	GO:0010749	regulation of nitric oxide mediated signal transduction		0.0218	60.3	0.192	1	1	3	SPINK1
GO: Biological Process	GO:0060440	trachea formation		0.0218	60.3	0.192	1	1	3	BMP4
GO: Biological Process	GO:0046061	dATP catabolic process		0.0218	60.3	0.192	1	1	3	ADA
GO: Biological Process	GO:0002775	antimicrobial peptide production		0.0218	60.3	0.192	1	1	3	MMP7
GO: Biological Process	GO:0060197	cloacal septation		0.0218	60.3	0.192	1	1	3	BMP4
GO: Biological Process	GO:0033595	response to genistein		0.0218	60.3	0.192	1	1	3	CYP11A1
GO: Biological Process	GO:0060014	granulosa cell differentiation		0.0218	60.3	0.192	1	1	3	CYP11A1
GO: Biological Process	GO:0042822	pyridoxal phosphate metabolic process		0.0218	60.3	0.192	1	1	3	PDXK
GO: Biological Process	GO:0007619	courtship behavior		0.0218	60.3	0.192	1	1	3	THRA
GO: Biological Process	GO:0050756	fractalkine metabolic process		0.0218	60.3	0.192	1	1	3	CYP11A1
GO: Biological Process	GO:0006714	sesquiterpenoid metabolic process		0.0218	60.3	0.192	1	1	3	AKR1B10
GO: Biological Process	GO:0003130	BMP signaling pathway involved in heart induction		0.0218	60.3	0.192	1	1	3	BMP4
GO: Biological Process	GO:0060167	regulation of adenosine receptor signaling pathway		0.0218	60.3	0.192	1	1	3	ADA
GO: Biological Process	GO:0019541	propionate metabolic process		0.0218	60.3	0.192	1	1	3	ACSS1
GO: Biological Process	GO:0003134	endodermal-mesodermal cell signaling involved in heart induction		0.0218	60.3	0.192	1	1	3	BMP4
GO: Biological Process	GO:0046122	purine deoxyribonucleoside metabolic process		0.0218	60.3	0.192	1	1	3	ADA
GO: Biological Process	GO:0002339	B cell selection		0.0218	60.3	0.192	1	1	3	HLA-DQB1
GO: Biological Process	GO:0035696	monocyte extravasation		0.0218	60.3	0.192	1	1	3	AMICA1
GO: Biological Process	GO:0043585	nose morphogenesis		0.0218	60.3	0.192	1	1	3	STRA6
GO: Biological Process	GO:0030185	nitric oxide transport		0.0218	60.3	0.192	1	1	3	HBB
GO: Biological Process	GO:0010986	positive regulation of lipoprotein particle clearance		0.0218	60.3	0.192	1	1	3	LIPG
GO: Biological Process	GO:0001782	B cell homeostasis		0.0228	63.1	0.199	1	2	32	ADA,CD74
GO: Biological Process	GO:0015701	bicarbonate transport		0.0228	63.1	0.199	1	2	32	HBA2,HBB
GO: Biological Process	GO:0033014	tetrapyrrole biosynthetic process		0.0228	63.1	0.199	1	2	32	TSPO,ALAS2
GO: Biological Process	GO:0032941	secretion by tissue		0.0229	63.5	0.199	1	3	83	CDO1,ADA,KCNN4
GO: Biological Process	GO:2000106	regulation of leukocyte apoptotic process		0.0229	63.5	0.199	1	3	83	ADA,BMP4,CD74
GO: Biological Process	GO:0044283	small molecule biosynthetic process		0.0239	66.3	0.206	1	8	477	CDO1,CYP11A1,CHDH,ACSS1,GGT1,GSS,LPIN3,CD74
GO: Biological Process	GO:0051240	positive regulation of multicellular organismal process		0.0241	66.7	0.206	1	10	665	INHBB,IFI16,ADA,BMP4,LIPG,NGFR,CD74,CX3CL1,SCN3B,CD276
GO: Biological Process	GO:0086001	cardiac muscle cell action potential		0.0241	66.8	0.206	1	2	33	KCNH2,SCN3B
GO: Biological Process	GO:2000242	negative regulation of reproductive process		0.0241	66.8	0.206	1	2	33	ADA,BMP4
GO: Biological Process	GO:0030501	positive regulation of bone mineralization		0.0241	66.8	0.206	1	2	33	BMP4,CD276
GO: Biological Process	GO:0070252	actin-mediated cell contraction		0.0251	69.6	0.207	1	3	86	ACTA1,KCNH2,SCN3B
GO: Biological Process	GO:0060541	respiratory system development		0.0252	69.9	0.207	1	5	226	ADA,GGT1,BMP4,THRA,STRA6
GO: Biological Process	GO:0030595	leukocyte chemotaxis		0.0254	70.4	0.207	1	4	152	AMICA1,CD74,CX3CL1,PREX1
GO: Biological Process	GO:0045597	positive regulation of cell differentiation		0.0254	70.4	0.207	1	10	671	IFI16,ADA,BMP4,THRA,TSPO,NGFR,CD74,TACSTD2,PREX1,CD276
GO: Biological Process	GO:1901655	cellular response to ketone		0.0255	70.7	0.207	1	2	34	BMP4,FBXO32
GO: Biological Process	GO:0045123	cellular extravasation		0.0255	70.7	0.207	1	2	34	AMICA1,CX3CL1
GO: Biological Process	GO:0009725	response to hormone		0.0258	71.5	0.207	1	12	871	CDO1,NPPA,INHBB,CYP11A1,ACTA1,CRYAA,BMP4,THRA,TSPO,NGFR,FOXO4,FBXO32
GO: Biological Process	GO:0042391	regulation of membrane potential		0.0261	72.2	0.207	1	7	395	EHD3,KCNH2,TSPO,NGFR,KCNN4,CX3CL1,SCN3B
GO: Biological Process	GO:0010811	positive regulation of cell-substrate adhesion		0.0267	73.9	0.207	1	3	88	SPOCK2,FBLN2,PREX1
GO: Biological Process	GO:0071622	regulation of granulocyte chemotaxis		0.0269	74.6	0.207	1	2	35	CD74,CX3CL1
GO: Biological Process	GO:0045620	negative regulation of lymphocyte differentiation		0.0269	74.6	0.207	1	2	35	BMP4,CD74
GO: Biological Process	GO:0010712	regulation of collagen metabolic process		0.0269	74.6	0.207	1	2	35	BMP4,MFAP4
GO: Biological Process	GO:0070169	positive regulation of biomineral tissue development		0.0269	74.6	0.207	1	2	35	BMP4,CD276
GO: Biological Process	GO:0032371	regulation of sterol transport		0.0269	74.6	0.207	1	2	35	LIPG,TSPO
GO: Biological Process	GO:0014014	negative regulation of gliogenesis		0.0269	74.6	0.207	1	2	35	BMP4,TSPO
GO: Biological Process	GO:0048048	embryonic eye morphogenesis		0.0269	74.6	0.207	1	2	35	CRYAA,STRA6
GO: Biological Process	GO:0048730	epidermis morphogenesis		0.0269	74.6	0.207	1	2	35	KLK14,NGFR
GO: Biological Process	GO:0032374	regulation of cholesterol transport		0.0269	74.6	0.207	1	2	35	LIPG,TSPO
GO: Biological Process	GO:0016485	protein processing		0.0271	74.9	0.207	1	10	678	MASP1,SPINK1,CPE,CPZ,IFI16,GGT1,CRYAA,SLPI,NGFR,CLEC3B
GO: Biological Process	GO:0006952	defense response		0.0273	75.7	0.207	1	18	1515	CDO1,MASP1,HTRA1,INHBB,HLA-DQB1,IFI16,ADA,GGT1,ORM1,TGM2,ORM2,KCNN4,CD74,FOXO4,MMP7,CX3CL1,CTSK,CD276
GO: Biological Process	GO:0050880	regulation of blood vessel size		0.0276	76.5	0.207	1	4	156	NPPA,MRVI1,HBB,CX3CL1
GO: Biological Process	GO:0042060	wound healing		0.0278	76.9	0.207	1	10	681	MUSTN1,PSAP,PROCR,MRVI1,DYSF,PAPSS2,AMICA1,EHD3,HBB,CX3CL1
GO: Biological Process	GO:0035150	regulation of tube size		0.0282	78	0.207	1	4	157	NPPA,MRVI1,HBB,CX3CL1
GO: Biological Process	GO:0008016	regulation of heart contraction		0.0282	78	0.207	1	4	157	ADA,THRA,KCNH2,SCN3B
GO: Biological Process	GO:0042168	heme metabolic process		0.0284	78.6	0.207	1	2	36	TSPO,ALAS2
GO: Biological Process	GO:0044705	multi-organism reproductive behavior		0.0284	78.6	0.207	1	2	36	CYP11A1,THRA
GO: Biological Process	GO:0050854	regulation of antigen receptor-mediated signaling pathway		0.0284	78.6	0.207	1	2	36	ADA,KCNN4
GO: Biological Process	GO:0072095	regulation of branch elongation involved in ureteric bud branching		0.0289	80.1	0.207	1	1	4	BMP4
GO: Biological Process	GO:0061205	paramesonephric duct development		0.0289	80.1	0.207	1	1	4	STRA6
GO: Biological Process	GO:0002154	thyroid hormone mediated signaling pathway		0.0289	80.1	0.207	1	1	4	THRA
GO: Biological Process	GO:0035844	cloaca development		0.0289	80.1	0.207	1	1	4	BMP4
GO: Biological Process	GO:0046391	5-phosphoribose 1-diphosphate metabolic process		0.0289	80.1	0.207	1	1	4	PRPS2
GO: Biological Process	GO:0019344	cysteine biosynthetic process		0.0289	80.1	0.207	1	1	4	GGT1
GO: Biological Process	GO:0042816	vitamin B6 metabolic process		0.0289	80.1	0.207	1	1	4	PDXK
GO: Biological Process	GO:1900003	regulation of serine-type endopeptidase activity		0.0289	80.1	0.207	1	1	4	SPINK1
GO: Biological Process	GO:0061038	uterus morphogenesis		0.0289	80.1	0.207	1	1	4	STRA6
GO: Biological Process	GO:0051790	short-chain fatty acid biosynthetic process		0.0289	80.1	0.207	1	1	4	ACSS1
GO: Biological Process	GO:0046439	L-cysteine metabolic process		0.0289	80.1	0.207	1	1	4	CDO1
GO: Biological Process	GO:0071688	striated muscle myosin thick filament assembly		0.0289	80.1	0.207	1	1	4	MYH11
GO: Biological Process	GO:0045085	negative regulation of interleukin-2 biosynthetic process		0.0289	80.1	0.207	1	1	4	CD276
GO: Biological Process	GO:0060279	positive regulation of ovulation		0.0289	80.1	0.207	1	1	4	INHBB
GO: Biological Process	GO:0060686	negative regulation of prostatic bud formation		0.0289	80.1	0.207	1	1	4	BMP4
GO: Biological Process	GO:0072199	regulation of mesenchymal cell proliferation involved in ureter development		0.0289	80.1	0.207	1	1	4	BMP4
GO: Biological Process	GO:0072278	metanephric comma-shaped body morphogenesis		0.0289	80.1	0.207	1	1	4	BMP4
GO: Biological Process	GO:0033088	negative regulation of immature T cell proliferation in thymus		0.0289	80.1	0.207	1	1	4	BMP4
GO: Biological Process	GO:0030241	skeletal muscle myosin thick filament assembly		0.0289	80.1	0.207	1	1	4	MYH11
GO: Biological Process	GO:0046121	deoxyribonucleoside catabolic process		0.0289	80.1	0.207	1	1	4	ADA
GO: Biological Process	GO:0046103	inosine biosynthetic process		0.0289	80.1	0.207	1	1	4	ADA
GO: Biological Process	GO:0043103	hypoxanthine salvage		0.0289	80.1	0.207	1	1	4	ADA

Category	ID	Name	Source	p-value	q-value Bonferroni	q-value FDR B&H	q-value FDR B&Y	Hit Count in Query List	Hit Count in Genome	Hit in Query List
GO: Biological Process	GO:0072197	ureter morphogenesis		0.0289	80.1	0.207	1	1	4	BMP4
GO: Biological Process	GO:0072198	mesenchymal cell proliferation involved in ureter development		0.0289	80.1	0.207	1	1	4	BMP4
GO: Biological Process	GO:0019448	L-cysteine catabolic process		0.0289	80.1	0.207	1	1	4	CDO1
GO: Biological Process	GO:0061209	cell proliferation involved in mesonephros development		0.0289	80.1	0.207	1	1	4	BMP4
GO: Biological Process	GO:0045925	positive regulation of female receptivity		0.0289	80.1	0.207	1	1	4	THRA
GO: Biological Process	GO:0009093	cysteine catabolic process		0.0289	80.1	0.207	1	1	4	CDO1
GO: Biological Process	GO:0060992	response to fungicide		0.0289	80.1	0.207	1	1	4	CYP11A1
GO: Biological Process	GO:0033087	negative regulation of immature T cell proliferation		0.0289	80.1	0.207	1	1	4	BMP4
GO: Biological Process	GO:1900004	negative regulation of serine-type endopeptidase activity		0.0289	80.1	0.207	1	1	4	SPINK1
GO: Biological Process	GO:0051799	negative regulation of hair follicle development		0.0289	80.1	0.207	1	1	4	NGFR
GO: Biological Process	GO:0014878	response to electrical stimulus involved in regulation of muscle adaptation		0.0289	80.1	0.207	1	1	4	FBXO32
GO: Biological Process	GO:0018199	peptidyl-glutamine modification		0.0289	80.1	0.207	1	1	4	TGM2
GO: Biological Process	GO:0006015	5-phosphoribose 1-diphosphate biosynthetic process		0.0289	80.1	0.207	1	1	4	PRPS2
GO: Biological Process	GO:0006083	acetate metabolic process		0.0289	80.1	0.207	1	1	4	ACSS1
GO: Biological Process	GO:0016053	organic acid biosynthetic process		0.0294	81.5	0.209	1	6	318	CDO1,CHDH,ACSS1,GGT1,GSS,CD74
GO: Biological Process	GO:0046394	carboxylic acid biosynthetic process		0.0294	81.5	0.209	1	6	318	CDO1,CHDH,ACSS1,GGT1,GSS,CD74
GO: Biological Process	GO:0030029	actin filament-based process		0.0298	82.5	0.209	1	9	592	MYH11,ACTA1,CRYAA,BAIAP2L1,KCNH2,CORO6,TACSTD2,PREX1,SCN3B
GO: Biological Process	GO:0070232	regulation of T cell apoptotic process		0.0299	82.7	0.209	1	2	37	ADA,BMP4
GO: Biological Process	GO:0048566	embryonic digestive tract development		0.0299	82.7	0.209	1	2	37	ADA,STRA6
GO: Biological Process	GO:0009069	serine family amino acid metabolic process		0.0299	82.7	0.209	1	2	37	CDO1,GGT1
GO: Biological Process	GO:0071385	cellular response to glucocorticoid stimulus		0.0299	82.7	0.209	1	2	37	BMP4,FBXO32
GO: Biological Process	GO:0043094	cellular metabolic compound salvage		0.0299	82.7	0.209	1	2	37	ADA,PDXK
GO: Biological Process	GO:0019098	reproductive behavior		0.0314	86.9	0.217	1	2	38	CYP11A1,THRA
GO: Biological Process	GO:0030890	positive regulation of B cell proliferation		0.0314	86.9	0.217	1	2	38	ADA,CD74
GO: Biological Process	GO:0086004	regulation of cardiac muscle cell contraction		0.0314	86.9	0.217	1	2	38	KCNH2,SCN3B
GO: Biological Process	GO:0007589	body fluid secretion		0.0316	87.4	0.217	1	3	94	CDO1,ADA,KCNN4
GO: Biological Process	GO:0051604	protein maturation		0.0316	87.5	0.217	1	10	696	MASP1,SPINK1,CPE,CPZ,IFI16,GGT1,CRYAA,SLPI,NGFR,CLEC3B
GO: Biological Process	GO:0042592	homeostatic process		0.0321	88.8	0.217	1	17	1433	NAPSA,JPH2,ADA,BMP4,TGM2,THRA,LIPG,KCNH2,TSPO,NGFR,KCNN4,CD74,ALAS2,HBA2,CTSK,SCN3B,LARGE
GO: Biological Process	GO:0043900	regulation of multi-organism process		0.0322	89.2	0.217	1	6	325	HTRA1,SPINK1,INHBB,IFI16,ADA,SLPI
GO: Biological Process	GO:0060429	epithelium development		0.0323	89.4	0.217	1	14	1110	CDSN,KLK7,PSAP,CYP11A1,KLK14,CALML5,CRYAA,BMP4,TGM2,TCHH,THRA,NGFR,STRA6,TACSTD2
GO: Biological Process	GO:0071384	cellular response to corticosteroid stimulus		0.0329	91.2	0.217	1	2	39	BMP4,FBXO32
GO: Biological Process	GO:0071804	cellular potassium ion transport		0.033	91.3	0.217	1	4	165	PKD2L1,KCNK12,KCNH2,KCNN4
GO: Biological Process	GO:0071805	potassium ion transmembrane transport		0.033	91.3	0.217	1	4	165	PKD2L1,KCNK12,KCNH2,KCNN4
GO: Biological Process	GO:0006164	purine nucleotide biosynthetic process		0.034	94.3	0.217	1	5	245	PRPS2,NPPA,PAPSS2,ADA,AKAP12
GO: Biological Process	GO:0086010	membrane depolarization during action potential		0.0345	95.5	0.217	1	2	40	KCNH2,SCN3B
GO: Biological Process	GO:0051148	negative regulation of muscle cell differentiation		0.0345	95.5	0.217	1	2	40	BMP4,FOXO4
GO: Biological Process	GO:0086003	cardiac muscle cell contraction		0.0345	95.5	0.217	1	2	40	KCNH2,SCN3B
GO: Biological Process	GO:0042692	muscle cell differentiation		0.0348	96.3	0.217	1	6	331	MYH11,ACTA1,BMP4,THRA,FOXO4,ASB2
GO: Biological Process	GO:0046129	purine ribonucleoside biosynthetic process		0.036	99.7	0.217	1	3	99	PRPS2,PAPSS2,ADA
GO: Biological Process	GO:0042451	purine nucleoside biosynthetic process		0.036	99.7	0.217	1	3	99	PRPS2,PAPSS2,ADA
GO: Biological Process	GO:0060278	regulation of ovulation		0.036	99.7	0.217	1	1	5	INHBB
GO: Biological Process	GO:1902571	regulation of serine-type peptidase activity		0.036	99.7	0.217	1	1	5	SPINK1
GO: Biological Process	GO:0090185	negative regulation of kidney development		0.036	99.7	0.217	1	1	5	BMP4
GO: Biological Process	GO:0098598	learned vocalization behavior or vocal learning		0.036	99.7	0.217	1	1	5	STRA6
GO: Biological Process	GO:0002636	positive regulation of germinal center formation		0.036	99.7	0.217	1	1	5	ADA
GO: Biological Process	GO:0072103	glomerulus vasculature morphogenesis		0.036	99.7	0.217	1	1	5	BMP4
GO: Biological Process	GO:0046882	negative regulation of follicle-stimulating hormone secretion		0.036	99.7	0.217	1	1	5	INHBB
GO: Biological Process	GO:0070317	negative regulation of G0 to G1 transition		0.036	99.7	0.217	1	1	5	FOXO4
GO: Biological Process	GO:0006528	asparagine metabolic process		0.036	99.7	0.217	1	1	5	ASPG
GO: Biological Process	GO:0098597	observational learning		0.036	99.7	0.217	1	1	5	STRA6
GO: Biological Process	GO:2000343	positive regulation of chemokine (C-X-C motif) ligand 2 production		0.036	99.7	0.217	1	1	5	CD74
GO: Biological Process	GO:0046101	hypoxanthine biosynthetic process		0.036	99.7	0.217	1	1	5	ADA
GO: Biological Process	GO:0072179	nephric duct formation		0.036	99.7	0.217	1	1	5	BMP4
GO: Biological Process	GO:0086036	regulation of cardiac muscle cell membrane potential		0.036	99.7	0.217	1	1	5	EHD3
GO: Biological Process	GO:0097070	ductus arteriosus closure		0.036	99.7	0.217	1	1	5	STRA6
GO: Biological Process	GO:0060662	salivary gland cavitation		0.036	99.7	0.217	1	1	5	TGM2
GO: Biological Process	GO:0098596	imitative learning		0.036	99.7	0.217	1	1	5	STRA6
GO: Biological Process	GO:0032914	positive regulation of transforming growth factor beta1 production		0.036	99.7	0.217	1	1	5	CX3CL1
GO: Biological Process	GO:0045077	negative regulation of interferon-gamma biosynthetic process		0.036	99.7	0.217	1	1	5	CD276
GO: Biological Process	GO:0030240	skeletal muscle thin filament assembly		0.036	99.7	0.217	1	1	5	ACTA1
GO: Biological Process	GO:0009217	purine deoxyribonucleoside triphosphate catabolic process		0.036	99.7	0.217	1	1	5	ADA
GO: Biological Process	GO:0086070	SA node cell to atrial cardiac muscle cell communication		0.036	99.7	0.217	1	1	5	SCN3B
GO: Biological Process	GO:0060605	tube lumen cavitation		0.036	99.7	0.217	1	1	5	TGM2
GO: Biological Process	GO:0016115	terpenoid catabolic process		0.036	99.7	0.217	1	1	5	AKR1B10
GO: Biological Process	GO:0072104	glomerular capillary formation		0.036	99.7	0.217	1	1	5	BMP4
GO: Biological Process	GO:0042297	vocal learning		0.036	99.7	0.217	1	1	5	STRA6
GO: Biological Process	GO:2000172	regulation of branching morphogenesis of a nerve		0.036	99.7	0.217	1	1	5	CPE
GO: Biological Process	GO:1902572	negative regulation of serine-type peptidase activity		0.036	99.7	0.217	1	1	5	SPINK1
GO: Biological Process	GO:0061312	BMP signaling pathway involved in heart development		0.036	99.7	0.217	1	1	5	BMP4
GO: Biological Process	GO:0072193	ureter smooth muscle cell differentiation		0.036	99.7	0.217	1	1	5	BMP4
GO: Biological Process	GO:0035990	tendon cell differentiation		0.036	99.7	0.217	1	1	5	BMP4
GO: Biological Process	GO:0033591	response to L-ascorbic acid		0.036	99.7	0.217	1	1	5	CYP11A1
GO: Biological Process	GO:0035992	tendon formation		0.036	99.7	0.217	1	1	5	BMP4
GO: Biological Process	GO:1900028	negative regulation of ruffle assembly		0.036	99.7	0.217	1	1	5	TACSTD2
GO: Biological Process	GO:0010756	positive regulation of plasminogen activation		0.036	99.7	0.217	1	1	5	CLEC3B
GO: Biological Process	GO:0072191	ureter smooth muscle development		0.036	99.7	0.217	1	1	5	BMP4
GO: Biological Process	GO:0051902	negative regulation of mitochondrial depolarization		0.036	99.7	0.217	1	1	5	NGFR
GO: Biological Process	GO:0010739	positive regulation of protein kinase A signaling		0.036	99.7	0.217	1	1	5	AKAP12
GO: Biological Process	GO:0046102	inosine metabolic process		0.036	99.7	0.217	1	1	5	ADA
GO: Biological Process	GO:0018963	phthalate metabolic process		0.036	99.7	0.217	1	1	5	CYP11A1
GO: Biological Process	GO:0046881	positive regulation of follicle-stimulating hormone secretion		0.036	99.7	0.217	1	1	5	INHBB
GO: Biological Process	GO:0033594	response to hydroxyisoflavone		0.036	99.7	0.217	1	1	5	CYP11A1
GO: Biological Process	GO:1901723	negative regulation of cell proliferation involved in kidney development		0.036	99.7	0.217	1	1	5	BMP4

Category	ID	Name	Source	p-value	q-value Bonferroni	q-value FDR B&H	q-value FDR B&Y	Hit Count in Query List	Hit Count in Genome	Hit in Query List
GO: Biological Process	GO:0060681	branch elongation involved in ureteric bud branching		0.036	99.7	0.217	1	1	5	BMP4
GO: Biological Process	GO:0033085	negative regulation of T cell differentiation in thymus		0.036	99.7	0.217	1	1	5	BMP4
GO: Biological Process	GO:0071396	cellular response to lipid		0.0361	99.9	0.217	1	6	334	INHBB,CYP11A1,BMP4,THRA,TSPO,FBXO32
GO: Biological Process	GO:0061337	cardiac conduction		0.0361	99.9	0.217	1	2	41	KCNH2,SCN3B
GO: Biological Process	GO:0045071	negative regulation of viral genome replication		0.0361	99.9	0.217	1	2	41	IFI16,SLPI
GO: Biological Process	GO:0007584	response to nutrient		0.0368	102	0.221	1	4	171	CYP11A1,ADA,LIPG,TSPO
GO: Biological Process	GO:1903115	regulation of actin filament-based movement		0.0377	104	0.225	1	2	42	KCNH2,SCN3B
GO: Biological Process	GO:0042110	T cell activation		0.0378	105	0.225	1	7	428	HLA-DQB1,ADA,AMICA1,BMP4,CD74,PREX1,CD276
GO: Biological Process	GO:0048660	regulation of smooth muscle cell proliferation		0.0378	105	0.225	1	3	101	BMP4,TGM2,CX3CL1
GO: Biological Process	GO:0034599	cellular response to oxidative stress		0.0382	106	0.227	1	4	173	PXDN,NGFR,HBA2,HBB
GO: Biological Process	GO:0046686	response to cadmium ion		0.0394	109	0.23	1	2	43	CYP11A1,GSS
GO: Biological Process	GO:0031076	embryonic camera-type eye development		0.0394	109	0.23	1	2	43	CRYAA,STRA6
GO: Biological Process	GO:0034308	primary alcohol metabolic process		0.0394	109	0.23	1	2	43	ACSS1,AKR1B10
GO: Biological Process	GO:0051048	negative regulation of secretion		0.0396	110	0.23	1	4	175	INHBB,ADA,CD74,CX3CL1
GO: Biological Process	GO:0071887	leukocyte apoptotic process		0.0397	110	0.23	1	3	103	ADA,BMP4,CD74
GO: Biological Process	GO:0007596	blood coagulation		0.0404	112	0.23	1	8	529	PSAP,PROCR,MRVI1,PAPSS2,AMICA1,EHD3,HBB,CX3CL1
GO: Biological Process	GO:0072522	purine-containing compound biosynthetic process		0.0405	112	0.23	1	5	257	PRPS2,NPPA,PAPSS2,ADA,AKAP12
GO: Biological Process	GO:0048659	smooth muscle cell proliferation		0.0407	113	0.23	1	3	104	BMP4,TGM2,CX3CL1
GO: Biological Process	GO:0034614	cellular response to reactive oxygen species		0.0407	113	0.23	1	3	104	PXDN,HBA2,HBB
GO: Biological Process	GO:0043603	cellular amide metabolic process		0.041	114	0.23	1	4	177	ASPG,CPE,GGT1,GSS
GO: Biological Process	GO:0046651	lymphocyte proliferation		0.041	114	0.23	1	5	258	HLA-DQB1,ADA,BMP4,CD74,CD276
GO: Biological Process	GO:0050817	coagulation		0.0416	115	0.23	1	8	532	PSAP,PROCR,MRVI1,PAPSS2,AMICA1,EHD3,HBB,CX3CL1
GO: Biological Process	GO:0030048	actin filament-based movement		0.0417	116	0.23	1	3	105	ACTA1,KCNH2,SCN3B
GO: Biological Process	GO:0014902	myotube differentiation		0.0417	116	0.23	1	3	105	ACTA1,BMP4,THRA
GO: Biological Process	GO:0032943	mononuclear cell proliferation		0.0422	117	0.23	1	5	260	HLA-DQB1,ADA,BMP4,CD74,CD276
GO: Biological Process	GO:0007599	hemostasis		0.0423	117	0.23	1	8	534	PSAP,PROCR,MRVI1,PAPSS2,AMICA1,EHD3,HBB,CX3CL1
GO: Biological Process	GO:0003018	vascular process in circulatory system		0.0424	117	0.23	1	4	179	NPPA,MRVI1,HBB,CX3CL1
GO: Biological Process	GO:0044246	regulation of multicellular organismal metabolic process		0.0427	118	0.23	1	2	45	BMP4,MFAP4
GO: Biological Process	GO:0042771	intrinsic apoptotic signaling pathway in response to DNA damage by p53 class mediator		0.0427	118	0.23	1	2	45	IFI16,CD74
GO: Biological Process	GO:0002792	negative regulation of peptide secretion		0.0427	118	0.23	1	2	45	INHBB,CD74
GO: Biological Process	GO:0051881	regulation of mitochondrial membrane potential		0.0427	118	0.23	1	2	45	TSPO,NGFR
GO: Biological Process	GO:0006778	porphyrin-containing compound metabolic process		0.0427	118	0.23	1	2	45	TSPO,ALAS2
GO: Biological Process	GO:0007182	common-partner SMAD protein phosphorylation		0.043	119	0.23	1	1	6	BMP4
GO: Biological Process	GO:0072049	comma-shaped body morphogenesis		0.043	119	0.23	1	1	6	BMP4
GO: Biological Process	GO:0010940	positive regulation of necrotic cell death		0.043	119	0.23	1	1	6	TSPO
GO: Biological Process	GO:0046100	hypoxanthine metabolic process		0.043	119	0.23	1	1	6	ADA
GO: Biological Process	GO:0050428	3'-phosphoadenosine 5'-phosphosulfate biosynthetic process		0.043	119	0.23	1	1	6	PAPSS2
GO: Biological Process	GO:0002830	positive regulation of type 2 immune response		0.043	119	0.23	1	1	6	CD74
GO: Biological Process	GO:0032364	oxygen homeostasis		0.043	119	0.23	1	1	6	ALAS2
GO: Biological Process	GO:0043096	purine nucleobase salvage		0.043	119	0.23	1	1	6	ADA
GO: Biological Process	GO:0060449	bud elongation involved in lung branching		0.043	119	0.23	1	1	6	BMP4
GO: Biological Process	GO:0018894	dibenzo-p-dioxin metabolic process		0.043	119	0.23	1	1	6	CYP11A1
GO: Biological Process	GO:0071493	cellular response to UV-B		0.043	119	0.23	1	1	6	MFAP4
GO: Biological Process	GO:0072201	negative regulation of mesenchymal cell proliferation		0.043	119	0.23	1	1	6	BMP4
GO: Biological Process	GO:1901142	insulin metabolic process		0.043	119	0.23	1	1	6	CPE
GO: Biological Process	GO:0031034	myosin filament assembly		0.043	119	0.23	1	1	6	MYH11
GO: Biological Process	GO:0072672	neutrophil extravasation		0.043	119	0.23	1	1	6	AMICA1
GO: Biological Process	GO:0032278	positive regulation of gonadotropin secretion		0.043	119	0.23	1	1	6	INHBB
GO: Biological Process	GO:0070309	lens fiber cell morphogenesis		0.043	119	0.23	1	1	6	CRYAA
GO: Biological Process	GO:0002043	blood vessel endothelial cell proliferation involved in sprouting angiogenesis		0.043	119	0.23	1	1	6	BMP4
GO: Biological Process	GO:0055025	positive regulation of cardiac muscle tissue development		0.043	119	0.23	1	1	6	BMP4
GO: Biological Process	GO:0001957	intramembranous ossification		0.043	119	0.23	1	1	6	CTSK
GO: Biological Process	GO:0036072	direct ossification		0.043	119	0.23	1	1	6	CTSK
GO: Biological Process	GO:0034036	purine ribonucleoside bisphosphate biosynthetic process		0.043	119	0.23	1	1	6	PAPSS2
GO: Biological Process	GO:2001256	regulation of store-operated calcium entry		0.043	119	0.23	1	1	6	SPINK1
GO: Biological Process	GO:0050915	sensory perception of sour taste		0.043	119	0.23	1	1	6	PKD2L1
GO: Biological Process	GO:0018158	protein oxidation		0.043	119	0.23	1	1	6	TRABD2B
GO: Biological Process	GO:0072284	metanephric S-shaped body morphogenesis		0.043	119	0.23	1	1	6	BMP4
GO: Biological Process	GO:1900042	positive regulation of interleukin-2 secretion		0.043	119	0.23	1	1	6	CD276
GO: Biological Process	GO:0061046	regulation of branching involved in lung morphogenesis		0.043	119	0.23	1	1	6	BMP4
GO: Biological Process	GO:0046184	aldehyde biosynthetic process		0.043	119	0.23	1	1	6	PDXK
GO: Biological Process	GO:0018879	biphenyl metabolic process		0.043	119	0.23	1	1	6	CYP11A1
GO: Biological Process	GO:0006081	cellular aldehyde metabolic process		0.0445	123	0.237	1	2	46	PDXK,AKR1B10
GO: Biological Process	GO:0006518	peptide metabolic process		0.0447	124	0.238	1	3	108	CPE,GGT1,GSS
GO: Biological Process	GO:0051291	protein heterooligomerization		0.0457	127	0.239	1	3	109	KCNH2,HBA2,HBB
GO: Biological Process	GO:0071310	cellular response to organic substance		0.0459	127	0.239	1	20	1840	HTRA1,PSAP,INHBB,CYP11A1,HLA-DQB1,RTN4R,IFI16,BMP4,THRA,PXDN,DERL3,TSPO,NGFR,CD68,CD74,FOXO4,CLEC3B,FBXO32,CX3CL1,ARHGEF6
GO: Biological Process	GO:0060047	heart contraction		0.0461	128	0.239	1	4	184	ADA,THRA,KCNH2,SCN3B
GO: Biological Process	GO:0000096	sulfur amino acid metabolic process		0.0462	128	0.239	1	2	47	CDO1,GGT1
GO: Biological Process	GO:0033619	membrane protein proteolysis		0.0462	128	0.239	1	2	47	NAPSA,NGFR
GO: Biological Process	GO:0009581	detection of external stimulus		0.0469	130	0.239	1	4	185	PKD2L1,AKR1B10,NGFR,STRA6
GO: Biological Process	GO:0003015	heart process		0.0469	130	0.239	1	4	185	ADA,THRA,KCNH2,SCN3B
GO: Biological Process	GO:0050795	regulation of behavior		0.0476	132	0.239	1	4	186	ADA,CD74,STRA6,CX3CL1
GO: Biological Process	GO:0045580	regulation of T cell differentiation		0.0478	133	0.239	1	3	111	ADA,BMP4,CD74
GO: Biological Process	GO:0030239	myofibril assembly		0.048	133	0.239	1	2	48	MYH11,ACTA1
GO: Biological Process	GO:0045778	positive regulation of ossification		0.048	133	0.239	1	2	48	BMP4,CD276
GO: Biological Process	GO:0070661	leukocyte proliferation		0.0482	134	0.239	1	5	270	HLA-DQB1,ADA,BMP4,CD74,CD276
GO: Biological Process	GO:0050863	regulation of T cell activation		0.0482	134	0.239	1	5	270	HLA-DQB1,ADA,BMP4,CD74,CD276
GO: Biological Process	GO:0060538	skeletal muscle organ development		0.0484	134	0.239	1	4	187	ACTA1,BMP4,STRA6,ASB2
GO: Biological Process	GO:0051259	protein oligomerization		0.0487	135	0.239	1	7	453	PKD2L1,EHD3,CRYAA,TGM2,KCNH2,HBA2,HBB
GO: Biological Process	GO:0045667	regulation of osteoblast differentiation		0.0489	135	0.239	1	3	112	IFI16,BMP4,CD276
GO: Biological Process	GO:0009582	detection of abiotic stimulus		0.0492	136	0.239	1	4	188	PKD2L1,AKR1B10,NGFR,STRA6
GO: Biological Process	GO:0043604	amide biosynthetic process		0.0498	138	0.239	1	2	49	GGT1,GSS
GO: Biological Process	GO:0006953	acute-phase response		0.0498	138	0.239	1	2	49	ORM1,ORM2
GO: Biological Process	GO:0043901	negative regulation of multi-organism process		0.05	138	0.239	1	3	113	HTRA1,IFI16,SLPI
GO: Biological Process	GO:0048593	camera-type eye morphogenesis		0.05	138	0.239	1	3	113	CRYAA,BMP4,STRA6
GO: Biological Process	GO:0031345	negative regulation of cell projection organization		0.05	138	0.239	1	3	113	RTN4R,NGFR,TACSTD2

Category	ID	Name	Source	p-value	q-value Bonferroni	q-value FDR B&H	q-value FDR B&Y	Hit Count in Query List	Hit Count in Genome	Hit in Query List
GO: Cellular Component	GO:0005615	extracellular space		0.00000305	0.000815	0.000815	0.00503	25	1284	NAPSA,DPT,NPPA,MASP1,HTRA1,PSAP,SPINK1,ACTA1,KLK14,CPE,ADA,GGT1,BMP4,ORM1,ORM2,ANGPTL2,PXDN,LIPG,CLEC3B,MMP7,HBA2,HBB,TACSTD2,CX3CL1,CTSK
GO: Cellular Component	GO:0031012	extracellular matrix		0.00000918	0.00245	0.00123	0.00755	13	422	DPT,HTRA1,SPOCK2,CPZ,BMP4,TGM2,MFAP4,FBLN2,PXDN,PRELP,SLPI,CLEC3B,MMP7
GO: Cellular Component	GO:0005578	proteinaceous extracellular matrix		0.0000365	0.00976	0.00325	0.0201	11	348	DPT,SPOCK2,CPZ,BMP4,TGM2,MFAP4,FBLN2,PXDN,PRELP,CLEC3B,MMP7
GO: Cellular Component	GO:0031838	haptoglobin-hemoglobin complex		0.000293	0.0783	0.0196	0.121	2	4	HBA2,HBB
GO: Cellular Component	GO:0000323	lytic vacuole		0.000569	0.152	0.0253	0.156	11	477	DPP7,NAPSA,NPPA,PSAP,HLA-DQB1,ADA,PRELP,AKR1B10,CD68,CD74,CTSK
GO: Cellular Component	GO:0005764	lysosome		0.000569	0.152	0.0253	0.156	11	477	DPP7,NAPSA,NPPA,PSAP,HLA-DQB1,ADA,PRELP,AKR1B10,CD68,CD74,CTSK
GO: Cellular Component	GO:0030139	endocytic vesicle		0.000909	0.243	0.0347	0.214	7	218	HLA-DQB1,RAB11FIP1,EHD3,NGFR,CD74,HBA2,HBB
GO: Cellular Component	GO:0005773	vacuole		0.00136	0.363	0.0454	0.28	11	531	DPP7,NAPSA,NPPA,PSAP,HLA-DQB1,ADA,PRELP,AKR1B10,CD68,CD74,CTSK
GO: Cellular Component	GO:0043202	lysosomal lumen		0.00186	0.495	0.055	0.339	4	74	PSAP,PRELP,CD74,CTSK
GO: Cellular Component	GO:0005775	vacuolar lumen		0.00225	0.601	0.0553	0.341	4	78	PSAP,PRELP,CD74,CTSK
GO: Cellular Component	GO:0072562	blood microparticle		0.00245	0.653	0.0553	0.341	5	132	ACTA1,ORM1,ORM2,HBA2,HBB
GO: Cellular Component	GO:0044449	contractile fiber part		0.00249	0.664	0.0553	0.341	6	193	MYH11,ACTA1,JPH2,PGM5,FBXO32,SCN3B
GO: Cellular Component	GO:0005833	hemoglobin complex		0.00311	0.83	0.0631	0.389	2	12	HBA2,HBB
GO: Cellular Component	GO:0016023	cytoplasmic membrane-bounded vesicle		0.00331	0.883	0.0631	0.389	16	1062	DPP7,NAPSA,KLK7,MYH11,SLC39A2,HLA-DQB1,CPE,RAB11FIP1,DYSF,ADA,EHD3,NGFR,CD74,GPNMB,HBA2,HBB
GO: Cellular Component	GO:0043292	contractile fiber		0.00395	1.05	0.0657	0.405	6	212	MYH11,ACTA1,JPH2,PGM5,FBXO32,SCN3B
GO: Cellular Component	GO:0009986	cell surface		0.00397	1.06	0.0657	0.405	12	700	HLA-DQB1,APMAP,RTN4R,PKD2L1,ADA,GGT1,KCNH2,NGFR,CD74,CAPN5,CX3CL1,CD276
GO: Cellular Component	GO:0042599	lamellar body		0.00425	1.13	0.0657	0.405	2	14	NAPSA,KLK7
GO: Cellular Component	GO:0045334	clathrin-coated endocytic vesicle		0.00443	1.18	0.0657	0.405	3	47	HLA-DQB1,NGFR,CD74
GO: Cellular Component	GO:0042613	MHC class II protein complex		0.00555	1.48	0.0741	0.457	2	16	HLA-DQB1,CD74
GO: Cellular Component	GO:0071682	endocytic vesicle lumen		0.00555	1.48	0.0741	0.457	2	16	HBA2,HBB
GO: Cellular Component	GO:0031301	integral component of organelle membrane		0.00621	1.66	0.079	0.487	6	233	TRABD2B,HLA-DQB1,PORCN,DERL3,CD74,LARGE
GO: Cellular Component	GO:0035692	macrophage migration inhibitory factor receptor complex		0.0071	1.9	0.0793	0.489	1	1	CD74
GO: Cellular Component	GO:0035693	NOS2-CD74 complex		0.0071	1.9	0.0793	0.489	1	1	CD74
GO: Cellular Component	GO:0030018	Z disc		0.00743	1.98	0.0793	0.489	4	109	JPH2,PGM5,FBXO32,SCN3B
GO: Cellular Component	GO:0030176	integral component of endoplasmic reticulum membrane		0.00743	1.98	0.0793	0.489	4	109	HLA-DQB1,PORCN,DERL3,CD74
GO: Cellular Component	GO:0031410	cytoplasmic vesicle		0.00782	2.09	0.0803	0.495	16	1163	DPP7,NAPSA,KLK7,MYH11,SLC39A2,HLA-DQB1,CPE,RAB11FIP1,DYSF,ADA,EHD3,NGFR,CD74,GPNMB,HBA2,HBB
GO: Cellular Component	GO:0030017	sarcomere		0.00842	2.25	0.0832	0.513	5	177	ACTA1,JPH2,PGM5,FBXO32,SCN3B
GO: Cellular Component	GO:0031300	intrinsic component of organelle membrane		0.00881	2.35	0.084	0.518	6	251	TRABD2B,HLA-DQB1,PORCN,DERL3,CD74,LARGE
GO: Cellular Component	GO:0001725	stress fiber		0.00957	2.56	0.0869	0.536	3	62	MYH11,ACTA1,PGM5
GO: Cellular Component	GO:0031227	intrinsic component of endoplasmic reticulum membrane		0.00976	2.61	0.0869	0.536	4	118	HLA-DQB1,PORCN,DERL3,CD74
GO: Cellular Component	GO:0032432	actin filament bundle		0.0104	2.79	0.0873	0.538	3	64	MYH11,ACTA1,PGM5
GO: Cellular Component	GO:0098552	side of membrane		0.0105	2.79	0.0873	0.538	7	340	HLA-DQB1,ADA,GGT1,PGM5,NGFR,CD74,CD276
GO: Cellular Component	GO:0009897	external side of plasma membrane		0.0113	3.02	0.0908	0.56	6	265	HLA-DQB1,ADA,GGT1,NGFR,CD74,CD276
GO: Cellular Component	GO:0031674	I band		0.0116	3.09	0.0908	0.56	4	124	JPH2,PGM5,FBXO32,SCN3B
GO: Cellular Component	GO:0030016	myofibril		0.0135	3.6	0.103	0.634	5	199	ACTA1,JPH2,PGM5,FBXO32,SCN3B
GO: Cellular Component	GO:0042641	actomyosin		0.0143	3.83	0.106	0.656	3	72	MYH11,ACTA1,PGM5
GO: Cellular Component	GO:0042611	MHC protein complex		0.0154	4.12	0.11	0.681	2	27	HLA-DQB1,CD74
GO: Cellular Component	GO:0071556	integral component of lumenal side of endoplasmic reticulum membrane		0.0165	4.42	0.11	0.681	2	28	HLA-DQB1,CD74
GO: Cellular Component	GO:0098553	lumenal side of endoplasmic reticulum membrane		0.0165	4.42	0.11	0.681	2	28	HLA-DQB1,CD74
GO: Cellular Component	GO:0098576	lumenal side of membrane		0.0165	4.42	0.11	0.681	2	28	HLA-DQB1,CD74
GO: Cellular Component	GO:0060205	cytoplasmic membrane-bounded vesicle lumen		0.0184	4.9	0.12	0.737	3	79	ADA,HBA2,HBB
GO: Cellular Component	GO:0031983	vesicle lumen		0.0203	5.41	0.125	0.768	3	82	ADA,HBA2,HBB
GO: Cellular Component	GO:0042175	nuclear outer membrane-endoplasmic reticulum membrane network		0.0204	5.45	0.125	0.768	12	872	HLA-DQB1,JPH2,PORCN,MRVI1,LPIN3,ERGIC1,CCDC155,DERL3,EDEM2,CD74,SEZ6L,SHISA3
GO: Cellular Component	GO:0005914	spot adherens junction		0.021	5.61	0.125	0.768	1	3	PGM5
GO: Cellular Component	GO:0071953	elastic fiber		0.021	5.61	0.125	0.768	1	3	MFAP4
GO: Cellular Component	GO:0030658	transport vesicle membrane		0.0229	6.12	0.133	0.821	3	86	HLA-DQB1,CPE,CD74
GO: Cellular Component	GO:0005771	multivesicular body		0.0239	6.38	0.134	0.829	2	34	HLA-DQB1,CD74
GO: Cellular Component	GO:0043204	perikaryon		0.0243	6.5	0.134	0.829	3	88	CYP11A1,CPE,NGFR
GO: Cellular Component	GO:0034703	cation channel complex		0.0247	6.58	0.134	0.829	4	156	PKD2L1,KCNH2,KCNN4,SCN3B
GO: Cellular Component	GO:0002189	ribose phosphate diphosphokinase complex		0.0279	7.45	0.143	0.884	1	4	PRPS2
GO: Cellular Component	GO:0097209	epidermal lamellar body		0.0279	7.45	0.143	0.884	1	4	KLK7
GO: Cellular Component	GO:0036021	endolysosome lumen		0.0279	7.45	0.143	0.884	1	4	CTSK
GO: Cellular Component	GO:0030669	clathrin-coated endocytic vesicle membrane		0.0308	8.23	0.153	0.94	2	39	HLA-DQB1,CD74
GO: Cellular Component	GO:0032588	trans-Golgi network membrane		0.0308	8.23	0.153	0.94	2	39	HLA-DQB1,CD74
GO: Cellular Component	GO:0044433	cytoplasmic vesicle part		0.0333	8.88	0.161	0.996	8	527	HLA-DQB1,CPE,RAB11FIP1,DYSF,ADA,CD74,HBA2,HBB
GO: Cellular Component	GO:0030485	smooth muscle contractile fiber		0.0348	9.28	0.163	1	1	5	MYH11
GO: Cellular Component	GO:0044437	vacuolar part		0.0348	9.29	0.163	1	6	343	PSAP,HLA-DQB1,PRELP,CD68,CD74,CTSK
GO: Cellular Component	GO:0012507	ER to Golgi transport vesicle membrane		0.0354	9.44	0.163	1	2	42	HLA-DQB1,CD74
GO: Cellular Component	GO:0005783	endoplasmic reticulum		0.0383	10.2	0.171	1	17	1518	HLA-DQB1,RTN4R,JPH2,PORCN,PKD2L1,MRVI1,LPIN3,ERGIC1,TGM2,PXDN,DERL3,EDEM2,NGFR,CD74,METTL7A,SEZ6L,SHISA3
GO: Cellular Component	GO:0005789	endoplasmic reticulum membrane		0.0391	10.4	0.171	1	11	854	HLA-DQB1,JPH2,PORCN,MRVI1,LPIN3,ERGIC1,DERL3,EDEM2,CD74,SEZ6L,SHISA3
GO: Cellular Component	GO:0034702	ion channel complex		0.0414	11.1	0.171	1	5	268	PORCN,PKD2L1,KCNH2,KCNN4,SCN3B
GO: Cellular Component	GO:0031466	Cul5-RING ubiquitin ligase complex		0.0416	11.1	0.171	1	1	6	ASB2
GO: Cellular Component	GO:0019907	cyclin-dependent protein kinase activating kinase holoenzyme complex		0.0416	11.1	0.171	1	1	6	KDM5A
GO: Cellular Component	GO:0030314	junctional membrane complex		0.0416	11.1	0.171	1	1	6	JPH2
GO: Cellular Component	GO:0001652	granular component		0.0416	11.1	0.171	1	1	6	CLEC3B
GO: Cellular Component	GO:0016328	lateral plasma membrane		0.0468	12.5	0.187	1	2	49	CLDN15,TACSTD2
GO: Cellular Component	GO:0098589	membrane region		0.0468	12.5	0.187	1	15	1326	HLA-DQB1,JPH2,PORCN,PKD2L1,MRVI1,LPIN3,ERGIC1,CCDC155,DERL3,EDEM2,KCNN4,CD74,TACSTD2,SEZ6L,SHISA3
GO: Cellular Component	GO:0030134	ER to Golgi transport vesicle		0.0485	13	0.189	1	2	50	HLA-DQB1,CD74
GO: Cellular Component	GO:0005911	cell-cell junction		0.0488	13	0.189	1	6	373	CDSN,CLDN15,AMICA1,PGM5,GJB3,ARHGEF6

Category	ID	Name	Source	p-value	q-value Bonferroni	q-value FDR B&H	q-value FDR B&Y	Hit Count in Query List	Hit Count in Genome	Hit in Query List
Human Phenotype	HP:0001626	Abnormality of the cardiovascular system		0.0366	52.4	0.331	1	17	1274	NPPA,MASP1,RPL35A,MYH11,HTRA1,SPINK1,ACTA1,JPH2,PORCN,GJB3,KCNH2,MID1,ALAS2,CAPN5,STRA6,HBB,SCN3B
Human Phenotype	HP:0000489	Abnormality of globe location or size		0.0213	30.5	0.302	1	10	559	MASP1,RPL35A,PORCN,CRYAA,BMP4,PXDN,MID1,STRA6,CTSK,LARGE
Human Phenotype	HP:0000315	Abnormality of the orbital region		0.0244	34.9	0.304	1	10	571	MASP1,RPL35A,PORCN,CRYAA,BMP4,PXDN,MID1,STRA6,CTSK,LARGE
Human Phenotype	HP:0001903	Anemia		0.00117	1.67	0.152	1	9	307	RPL35A,PSAP,HLA-DQB1,ADA,GSS,ALAS2,HBA2,HBB,CTSK
Human Phenotype	HP:0010973	Abnormality of erythroid lineage cell		0.0023	3.29	0.194	1	9	338	RPL35A,PSAP,HLA-DQB1,ADA,GSS,ALAS2,HBA2,HBB,CTSK
Human Phenotype	HP:0001877	Abnormality of erythrocytes		0.0023	3.29	0.194	1	9	338	RPL35A,PSAP,HLA-DQB1,ADA,GSS,ALAS2,HBA2,HBB,CTSK
Human Phenotype	HP:0100763	Abnormality of the lymphatic system		0.00856	12.2	0.253	1	8	338	PSAP,PORCN,ADA,ALAS2,STRA6,HBA2,HBB,CTSK
Human Phenotype	HP:0001743	Abnormality of the spleen		0.00844	12.1	0.253	1	7	268	PSAP,ADA,ALAS2,STRA6,HBA2,HBB,CTSK
Human Phenotype	HP:0000481	Abnormality of the cornea		0.0245	35.1	0.304	1	7	330	PORCN,CRYAA,BMP4,GJB3,PXDN,TACSTD2,LARGE
Human Phenotype	HP:0010936	Abnormality of the lower urinary tract		0.0335	47.9	0.326	1	7	352	RPL35A,HTRA1,PSAP,CYP11A1,HLA-DQB1,BMP4,MID1
Human Phenotype	HP:0001143	Small eyes		0.000755	1.08	0.152	1	6	126	PORCN,CRYAA,BMP4,PXDN,STRA6,LARGE
Human Phenotype	HP:0000568	Microphthalmos		0.000755	1.08	0.152	1	6	126	PORCN,CRYAA,BMP4,PXDN,STRA6,LARGE
Human Phenotype	HP:0100887	Abnormality of globe size		0.00131	1.88	0.156	1	6	140	PORCN,CRYAA,BMP4,PXDN,STRA6,LARGE
Human Phenotype	HP:0010972	Anemia of inadequate production		0.000534	0.764	0.152	1	5	77	RPL35A,HLA-DQB1,ALAS2,HBA2,HBB
Human Phenotype	HP:0007957	Corneal opacity		0.00997	14.3	0.255	1	5	150	PORCN,BMP4,GJB3,PXDN,LARGE
Human Phenotype	HP:0000515	Corneal opacity		0.00997	14.3	0.255	1	5	150	PORCN,BMP4,GJB3,PXDN,LARGE
Human Phenotype	HP:0000501	Glaucoma		0.0111	15.9	0.274	1	5	154	MASP1,CRYAA,GJB3,PXDN,LARGE
Human Phenotype	HP:0001665	Abnormality of cardiac conduction		0.0185	26.5	0.279	1	5	175	NPPA,JPH2,KCNH2,HBB,SCN3B
Human Phenotype	HP:0000819	Diabetes mellitus		0.0281	40.2	0.312	1	5	195	SPINK1,HLA-DQB1,GJB3,ALAS2,HBB
Human Phenotype	HP:0001952	Abnormal glucose tolerance		0.0321	45.9	0.326	1	5	202	SPINK1,HLA-DQB1,GJB3,ALAS2,HBB
Human Phenotype	HP:0002115	Sparse or absent hair		0.0327	46.8	0.326	1	5	203	CDSN,HTRA1,HLA-DQB1,PORCN,GJB3
Human Phenotype	HP:0011122	Abnormality of skin physiology		0.0493	70.5	0.352	1	5	227	CDSN,HLA-DQB1,PORCN,PAPSS2,GJB3
Human Phenotype	HP:0001931	Hypochromic anemia		0.000029	0.0414	0.0414	0.325	4	21	HLA-DQB1,ALAS2,HBA2,HBB
Human Phenotype	HP:0001878	Hemolytic anemia		0.00861	12.3	0.253	1	4	92	ADA,GSS,HBA2,HBB
Human Phenotype	HP:0011895	Anemia due to reduced life span of red cells		0.00861	12.3	0.253	1	4	92	ADA,GSS,HBA2,HBB
Human Phenotype	HP:0001596	Alopecia		0.00997	14.3	0.255	1	4	96	HTRA1,HLA-DQB1,PORCN,GJB3
Human Phenotype	HP:0001231	Abnormality of the fingernails		0.0119	17	0.279	1	4	101	PORCN,BMP4,GJB3,CTSK
Human Phenotype	HP:0011400	Abnormal CNS myelination		0.0249	35.5	0.304	1	4	126	HTRA1,PSAP,HLA-DQB1,BMP4
Human Phenotype	HP:0000047	Hypospadias		0.0255	36.5	0.304	1	4	127	RPL35A,CYP11A1,BMP4,MID1
Human Phenotype	HP:0009381	Short finger		0.0289	41.3	0.315	1	4	132	MASP1,PORCN,BMP4,CTSK
Human Phenotype	HP:0000589	Coloboma		0.0333	47.6	0.326	1	4	138	PORCN,CRYAA,BMP4,LARGE
Human Phenotype	HP:0100627	Displacement of the external urethral meatus		0.0348	49.8	0.326	1	4	140	RPL35A,CYP11A1,BMP4,MID1
Human Phenotype	HP:0000204	Cleft upper lip		0.0381	54.4	0.336	1	4	144	MASP1,PORCN,MID1,LARGE
Human Phenotype	HP:0002520	Abnormal myelination		0.0423	60.6	0.336	1	4	149	HTRA1,PSAP,HLA-DQB1,BMP4
Human Phenotype	HP:0001120	Abnormality of corneal size		0.0488	69.8	0.352	1	4	156	CRYAA,BMP4,PXDN,LARGE
Human Phenotype	HP:0004840	Hypochromic microcytic anemia		0.0000755	0.108	0.054	0.423	3	10	ALAS2,HBA2,HBB
Human Phenotype	HP:0010472	Abnormality of the heme biosynthetic pathway		0.000489	0.699	0.152	1	3	18	ALAS2,HBA2,HBB
Human Phenotype	HP:0001935	Microcytic anemia		0.000782	1.12	0.152	1	3	21	ALAS2,HBA2,HBB
Human Phenotype	HP:0000528	Anophthalmia		0.00117	1.67	0.152	1	3	24	PORCN,BMP4,STRA6
Human Phenotype	HP:0003458	EMG: myopathic abnormalities		0.00226	3.23	0.194	1	3	30	ACTA1,DYSF,LARGE
Human Phenotype	HP:0001374	Congenital hip dislocation		0.00298	4.26	0.228	1	3	33	ACTA1,PORCN,THRA
Human Phenotype	HP:0001972	Macrocytic anemia		0.00353	5.05	0.23	1	3	35	RPL35A,HLA-DQB1,ALAS2
Human Phenotype	HP:0001539	Omphalocele		0.00593	8.48	0.253	1	3	42	MASP1,PORCN,THRA
Human Phenotype	HP:0007305	CNS demyelination		0.00965	13.8	0.255	1	3	50	HTRA1,PSAP,HLA-DQB1
Human Phenotype	HP:0000593	Abnormality of the anterior chamber		0.0132	18.8	0.279	1	3	56	MASP1,PXDN,LARGE
Human Phenotype	HP:0000482	Microcornea		0.0188	26.9	0.279	1	3	64	CRYAA,BMP4,PXDN
Human Phenotype	HP:0100688	Decreased corneal diameter		0.0188	26.9	0.279	1	3	64	CRYAA,BMP4,PXDN
Human Phenotype	HP:0000612	Iris coloboma		0.0257	36.7	0.304	1	3	72	PORCN,CRYAA,BMP4
Human Phenotype	HP:0000684	Delayed eruption of teeth		0.0266	38.1	0.304	1	3	73	PORCN,THRA,CTSK
Human Phenotype	HP:0001649	Tachycardia		0.0276	39.4	0.308	1	3	74	NPPA,JPH2,SCN3B
Human Phenotype	HP:0003381	Demyelination		0.0295	42.2	0.32	1	3	76	HTRA1,PSAP,HLA-DQB1
Human Phenotype	HP:0000020	Urinary incontinence		0.0305	43.7	0.326	1	3	77	HTRA1,PSAP,HLA-DQB1
Human Phenotype	HP:0007147	Urinary or fecal incontinence		0.0305	43.7	0.326	1	3	77	HTRA1,PSAP,HLA-DQB1
Human Phenotype	HP:0004378	Abnormality of the anus		0.0316	45.2	0.326	1	3	78	PORCN,MID1,LARGE
Human Phenotype	HP:0000126	Hydronephrosis		0.0348	49.7	0.326	1	3	81	MASP1,PORCN,STRA6
Human Phenotype	HP:0010946	Dilatation of the renal pelvis		0.0348	49.7	0.326	1	3	81	MASP1,PORCN,STRA6
Human Phenotype	HP:0010944	Abnormality of the renal pelvis		0.037	52.9	0.331	1	3	83	MASP1,PORCN,STRA6
Human Phenotype	HP:0002063	Rigidity		0.037	52.9	0.331	1	3	83	HTRA1,HLA-DQB1,ACTA1
Human Phenotype	HP:0012130	Abnormality of cells of the erythroid lineage		0.037	52.9	0.331	1	3	83	RPL35A,HBB,CTSK
Human Phenotype	HP:0002827	Hip dislocation		0.0393	56.2	0.336	1	3	85	ACTA1,PORCN,THRA
Human Phenotype	HP:0006292	Abnormality of dental eruption		0.0405	57.9	0.336	1	3	86	PORCN,THRA,CTSK
Human Phenotype	HP:0100326	Immunologic hypersensitivity		0.0405	57.9	0.336	1	3	86	CDSN,HLA-DQB1,ADA
Human Phenotype	HP:0000618	Blindness		0.0492	70.3	0.352	1	3	93	BMP4,CAPN5,LARGE
Human Phenotype	HP:0011907	Reduced alpha/beta synthesis ratio		0.00114	1.63	0.152	1	2	6	HBA2,HBB
Human Phenotype	HP:0001971	Hypersplenism		0.00114	1.63	0.152	1	2	6	HBA2,HBB
Human Phenotype	HP:0005560	Imbalanced hemoglobin synthesis		0.00159	2.27	0.162	1	2	7	HBA2,HBB
Human Phenotype	HP:0001930	Nonspherocytic hemolytic anemia		0.00159	2.27	0.162	1	2	7	HBA2,HBB
Human Phenotype	HP:0001891	Iron deficiency anemia		0.00334	4.78	0.228	1	2	10	HLA-DQB1,ALAS2
Human Phenotype	HP:0004443	Lambdoidal craniosynostosis		0.00334	4.78	0.228	1	2	10	MASP1,BMP4
Human Phenotype	HP:0003212	Increased IgE level		0.00334	4.78	0.228	1	2	10	CDSN,ADA
Human Phenotype	HP:0011902	Abnormal hemoglobin		0.00758	10.8	0.253	1	2	15	HBA2,HBB
Human Phenotype	HP:0000557	Buphthalmos		0.00862	12.3	0.253	1	2	16	PXDN,LARGE
Human Phenotype	HP:0000647	Sclerocornea		0.00971	13.9	0.255	1	2	17	BMP4,PXDN
Human Phenotype	HP:0001285	Spastic tetraparesis		0.0109	15.5	0.273	1	2	18	PSAP,GSS
Human Phenotype	HP:0001962	Palpitations		0.0121	17.3	0.279	1	2	19	JPH2,KCNH2
Human Phenotype	HP:0007700	Anterior segment dysgenesis		0.0133	19.1	0.279	1	2	20	PXDN,LARGE
Human Phenotype	HP:0012084	Abnormality of skeletal muscle fiber size		0.016	22.9	0.279	1	2	22	ACTA1,DYSF
Human Phenotype	HP:0004435	Hypergammaglobulinemia		0.0175	25	0.279	1	2	23	CDSN,ADA
Human Phenotype	HP:0002852	Hyperglobulinemia		0.0175	25	0.279	1	2	23	CDSN,ADA
Human Phenotype	HP:0002558	Supernumerary nipple		0.0175	25	0.279	1	2	23	MASP1,PORCN
Human Phenotype	HP:0040075	Hypopituitarism		0.019	27.1	0.279	1	2	24	BMP4,HBB
Human Phenotype	HP:0002518	Abnormality of the periventricular white matter		0.019	27.1	0.279	1	2	24	PSAP,LARGE
Human Phenotype	HP:0002570	Steatorrhea		0.019	27.1	0.279	1	2	24	SPINK1,HLA-DQB1
Human Phenotype	HP:0001081	Cholelithiasis		0.0205	29.3	0.293	1	2	25	ALAS2,HBB
Human Phenotype	HP:0006829	Severe muscular hypotonia		0.0205	29.3	0.293	1	2	25	BMP4,LARGE
Human Phenotype	HP:0001087	Congenital glaucoma		0.0205	29.3	0.293	1	2	25	PXDN,LARGE
Human Phenotype	HP:0002863	Myelodysplasia		0.0221	31.6	0.304	1	2	26	HBA2,HBB
Human Phenotype	HP:0002754	Osteomyelitis		0.0254	36.3	0.304	1	2	28	HBB,CTSK
Human Phenotype	HP:0000085	Horseshoe kidney		0.0271	38.8	0.305	1	2	29	PORCN,STRA6
Human Phenotype	HP:0002630	Fat malabsorption		0.0271	38.8	0.305	1	2	29	SPINK1,HLA-DQB1
Human Phenotype	HP:0012437	Abnormal gallbladder morphology		0.0289	41.3	0.315	1	2	30	ALAS2,HBB
Human Phenotype	HP:0010783	Erythema		0.0345	49.3	0.326	1	2	33	CDSN,GJB3
Human Phenotype	HP:0001131	Corneal dystrophy		0.0364	52.1	0.331	1	2	34	GJB3,TACSTD2
Human Phenotype	HP:0003298	Spina bifida occulta		0.0405	57.9	0.336	1	2	36	MASP1,PORCN
Human Phenotype	HP:0003678	Rapidly progressive		0.0425	60.8	0.336	1	2	37	HLA-DQB1,DYSF
Human Phenotype	HP:0002187	Intellectual disability, profound		0.0425	60.8	0.336	1	2	37	STRA6,LARGE
Human Phenotype	HP:0001798	Anonychia		0.0447	63.9	0.34	1	2	38	PORCN,CTSK
Human Phenotype	HP:0005110	Atrial fibrillation		0.0468	66.9	0.349	1	2	39	NPPA,SCN3B
Human Phenotype	HP:0005264	Abnormality of the gallbladder		0.0468	66.9	0.349	1	2	39	ALAS2,HBB
Human Phenotype	HP:0003114	Abnormal cardiological findings		0.0468	66.9	0.349	1	2	39	KCNH2,SCN3B
Human Phenotype	HP:0003115	Abnormal EKG		0.0468	66.9	0.349	1	2	39	KCNH2,SCN3B

Category	ID	Name	Source	p-value	q-value Bonferroni	q-value FDR B&H	q-value FDR B&Y	Hit Count in Query List	Hit Count in Genome	Hit in Query List
Human Phenotype	HP:0001692	Primary atrial arrhythmia		0.049	70.1	0.352	1	2	40	NPPA,SCN3B
Human Phenotype	HP:0006297	Hypoplasia of dental enamel		0.049	70.1	0.352	1	2	40	HLA-DQB1,PORCN
Human Phenotype	HP:0007204	Diffuse white matter abnormalities		0.00901	12.9	0.253	1	1	1	HTRA1
Human Phenotype	HP:0011000	Aplasia/Hypoplasia of the optic tract		0.00901	12.9	0.253	1	1	1	BMP4
Human Phenotype	HP:0012187	Increased erythrocyte protoporphyrin concentration		0.00901	12.9	0.253	1	1	1	ALAS2
Human Phenotype	HP:0003343	Glutathione synthetase deficiency		0.00901	12.9	0.253	1	1	1	GSS
Human Phenotype	HP:0007546	Linear hyperpigmentation		0.00901	12.9	0.253	1	1	1	PORCN
Human Phenotype	HP:0001996	Chronic metabolic acidosis		0.00901	12.9	0.253	1	1	1	GSS
Human Phenotype	HP:0011749	Adrenocorticotropic hormone excess		0.00901	12.9	0.253	1	1	1	CYP11A1
Human Phenotype	HP:0002622	Dissecting aortic aneurysm		0.00901	12.9	0.253	1	1	1	MYH11
Human Phenotype	HP:0006783	Posterior pharyngeal cleft		0.00901	12.9	0.253	1	1	1	MID1
Human Phenotype	HP:0005424	Absent specific antibody response		0.00901	12.9	0.253	1	1	1	ADA
Human Phenotype	HP:0030037	Bifid ureter		0.00901	12.9	0.253	1	1	1	PORCN
Human Phenotype	HP:0006608	Midclavicular hypoplasia		0.00901	12.9	0.253	1	1	1	PORCN
Human Phenotype	HP:0010999	Aplasia of the optic tract		0.00901	12.9	0.253	1	1	1	BMP4
Human Phenotype	HP:0200023	Priapism		0.00901	12.9	0.253	1	1	1	HBB
Human Phenotype	HP:0004931	Arteriosclerosis of small cerebral arteries		0.00901	12.9	0.253	1	1	1	HTRA1
Human Phenotype	HP:0008346	Increased red cell sickling tendency		0.00901	12.9	0.253	1	1	1	HBB
Human Phenotype	HP:0010740	Osteopathia striata		0.00901	12.9	0.253	1	1	1	PORCN
Human Phenotype	HP:0012180	Cystic medial necrosis		0.00901	12.9	0.253	1	1	1	MYH11
Human Phenotype	HP:0008205	Insulin-dependent but ketosis-resistant diabetes		0.00901	12.9	0.253	1	1	1	SPINK1
Human Phenotype	HP:0005524	Macrocytic hemolytic disease		0.00901	12.9	0.253	1	1	1	HBB
Human Phenotype	HP:0006638	Midclavicular aplasia		0.00901	12.9	0.253	1	1	1	PORCN
Human Phenotype	HP:0005311	Agenesis of pulmonary vessels		0.00901	12.9	0.253	1	1	1	STRA6
Human Phenotype	HP:0002678	Skull asymmetry		0.0178	25.4	0.279	1	1	2	MASP1
Human Phenotype	HP:0001817	Absent fingernail		0.0178	25.4	0.279	1	1	2	PORCN
Human Phenotype	HP:0011169	Generalized clonic seizures		0.0178	25.4	0.279	1	1	2	PSAP
Human Phenotype	HP:0100507	Folate deficiency		0.0178	25.4	0.279	1	1	2	HLA-DQB1
Human Phenotype	HP:0005944	Bilateral lung agenesis		0.0178	25.4	0.279	1	1	2	STRA6
Human Phenotype	HP:0005327	Loss of facial expression		0.0178	25.4	0.279	1	1	2	HLA-DQB1
Human Phenotype	HP:0005588	Patchy palmoplantar keratoderma		0.0178	25.4	0.279	1	1	2	GJB3
Human Phenotype	HP:0005511	Heinz body anemia		0.0178	25.4	0.279	1	1	2	HBB
Human Phenotype	HP:0003791	Deposits immunoreactive to beta-amyloid protein		0.0178	25.4	0.279	1	1	2	DYSF
Human Phenotype	HP:0007076	Extrapyramidal muscular rigidity		0.0178	25.4	0.279	1	1	2	HLA-DQB1
Human Phenotype	HP:0012191	B-cell lymphoma		0.0178	25.4	0.279	1	1	2	ADA
Human Phenotype	HP:0012559	Increased T3/T4 ratio		0.0178	25.4	0.279	1	1	2	THRA
Human Phenotype	HP:0001750	Single ventricle		0.0178	25.4	0.279	1	1	2	STRA6
Human Phenotype	HP:0002930	Thyroid hormone receptor defect		0.0178	25.4	0.279	1	1	2	THRA
Human Phenotype	HP:0012558	Abnormal T3/T4 ratio		0.0178	25.4	0.279	1	1	2	THRA
Human Phenotype	HP:0012335	Abnormality of folate metabolism		0.0178	25.4	0.279	1	1	2	HLA-DQB1
Human Phenotype	HP:0005156	Hypoplastic left atrium		0.0178	25.4	0.279	1	1	2	STRA6
Human Phenotype	HP:0007778	Neovascularization of peripheral and posterior retina		0.0178	25.4	0.279	1	1	2	CAPN5
Human Phenotype	HP:0006270	Hypoplastic spleen		0.0178	25.4	0.279	1	1	2	STRA6
Human Phenotype	HP:0009072	Decreased Achilles reflex		0.0178	25.4	0.279	1	1	2	DYSF
Human Phenotype	HP:0003258	Glyoxalase deficiency		0.0178	25.4	0.279	1	1	2	GSS
Human Phenotype	HP:0200099	Peripheral retinal pigmentation abnormalities		0.0178	25.4	0.279	1	1	2	GSS
Human Phenotype	HP:0005595	Generalized hyperkeratosis		0.0178	25.4	0.279	1	1	2	GJB3
Human Phenotype	HP:0010538	Small sella turcica		0.0178	25.4	0.279	1	1	2	BMP4
Human Phenotype	HP:0007658	Large hyperpigmented retinal spots		0.0178	25.4	0.279	1	1	2	CAPN5
Human Phenotype	HP:0004474	Persistent open anterior fontanelle		0.0266	38	0.304	1	1	3	CTSK
Human Phenotype	HP:0100512	Vitamin D deficiency		0.0266	38	0.304	1	1	3	HLA-DQB1
Human Phenotype	HP:0002448	Progressive encephalopathy		0.0266	38	0.304	1	1	3	HTRA1
Human Phenotype	HP:0001345	Psychotic mentation		0.0266	38	0.304	1	1	3	GSS
Human Phenotype	HP:0000134	Female hypogonadism		0.0266	38	0.304	1	1	3	BMP4
Human Phenotype	HP:0011906	Reduced beta/alpha synthesis ratio		0.0266	38	0.304	1	1	3	HBB
Human Phenotype	HP:0006216	Single interphalangeal crease of fifth finger		0.0266	38	0.304	1	1	3	MASP1
Human Phenotype	HP:0004727	Impaired renal concentrating ability		0.0266	38	0.304	1	1	3	HBB
Human Phenotype	HP:0001472	Familial predisposition		0.0266	38	0.304	1	1	3	BMP4
Human Phenotype	HP:0003419	Low back pain		0.0266	38	0.304	1	1	3	HTRA1
Human Phenotype	HP:0100502	Vitamin B12 deficiency		0.0266	38	0.304	1	1	3	HLA-DQB1
Human Phenotype	HP:0009600	Flexion contracture of thumb		0.0266	38	0.304	1	1	3	BMP4
Human Phenotype	HP:0005359	Aplasia of the thymus		0.0266	38	0.304	1	1	3	ADA
Human Phenotype	HP:0100511	Abnormality of vitamin D metabolism		0.0266	38	0.304	1	1	3	HLA-DQB1
Human Phenotype	HP:0004626	Lumbar scoliosis		0.0266	38	0.304	1	1	3	PAPSS2
Human Phenotype	HP:0007162	Diffuse demyelination of the cerebral white matter		0.0266	38	0.304	1	1	3	HTRA1
Human Phenotype	HP:0000537	Epicanthus inversus		0.0353	50.5	0.326	1	1	4	MASP1
Human Phenotype	HP:0005365	Severe B lymphocytopenia		0.0353	50.5	0.326	1	1	4	ADA
Human Phenotype	HP:0002487	Hyperkinesis		0.0353	50.5	0.326	1	1	4	PSAP
Human Phenotype	HP:0007068	Inferior vermis hypoplasia		0.0353	50.5	0.326	1	1	4	BMP4
Human Phenotype	HP:0009839	Osteolytic defects of the distal phalanges of the hand		0.0353	50.5	0.326	1	1	4	CTSK
Human Phenotype	HP:0002232	Patchy alopecia		0.0353	50.5	0.326	1	1	4	PORCN
Human Phenotype	HP:0012468	Chronic acidosis		0.0353	50.5	0.326	1	1	4	GSS
Human Phenotype	HP:0006280	Chronic pancreatitis		0.0353	50.5	0.326	1	1	4	SPINK1
Human Phenotype	HP:0004782	Hypotrichosis of the scalp		0.0353	50.5	0.326	1	1	4	CDSN
Human Phenotype	HP:0003191	Cleft ala nasi		0.0353	50.5	0.326	1	1	4	PORCN
Human Phenotype	HP:0009909	Uplifted earlobe		0.0353	50.5	0.326	1	1	4	BMP4
Human Phenotype	HP:0007834	Progressive cataract		0.0353	50.5	0.326	1	1	4	CRYAA
Human Phenotype	HP:0000087	Impaired renal function		0.0439	62.8	0.336	1	1	5	HBB
Human Phenotype	HP:0003304	Spondylolysis		0.0439	62.8	0.336	1	1	5	CTSK
Human Phenotype	HP:0007906	Increased intraocular pressure		0.0439	62.8	0.336	1	1	5	PXDN
Human Phenotype	HP:0005213	Pancreatic calcification		0.0439	62.8	0.336	1	1	5	SPINK1
Human Phenotype	HP:0001144	Orbital cyst		0.0439	62.8	0.336	1	1	5	BMP4
Human Phenotype	HP:0005206	Pancreatic pseudocyst		0.0439	62.8	0.336	1	1	5	SPINK1
Human Phenotype	HP:0006888	Meningoencephalocele		0.0439	62.8	0.336	1	1	5	LARGE
Human Phenotype	HP:0100831	Abnormality of vitamin K metabolism		0.0439	62.8	0.336	1	1	5	HLA-DQB1
Human Phenotype	HP:0004975	Erlenmeyer flask deformity of the femurs		0.0439	62.8	0.336	1	1	5	PSAP
Human Phenotype	HP:0012249	Abnormal ST segment		0.0439	62.8	0.336	1	1	5	SCN3B
Human Phenotype	HP:0000840	Adrenogenital syndrome		0.0439	62.8	0.336	1	1	5	CYP11A1
Human Phenotype	HP:0012133	Erythroid hypoplasia		0.0439	62.8	0.336	1	1	5	RPL35A
Human Phenotype	HP:0004935	Pulmonary artery atresia		0.0439	62.8	0.336	1	1	5	STRA6
Human Phenotype	HP:0012232	Shortened QT interval		0.0439	62.8	0.336	1	1	5	KCNH2
Human Phenotype	HP:0007901	Retinal malformation		0.0439	62.8	0.336	1	1	5	LARGE
Human Phenotype	HP:0004810	Congenital hypoplastic anemia		0.0439	62.8	0.336	1	1	5	HBB
Human Phenotype	HP:0011892	Vitamin K deficiency		0.0439	62.8	0.336	1	1	5	HLA-DQB1
Human Phenotype	HP:0012251	ST segment elevation		0.0439	62.8	0.336	1	1	5	SCN3B
Human Phenotype	HP:0007588	Reticular hyperpigmentation		0.0439	62.8	0.336	1	1	5	PORCN
Mouse Phenotype	MP:0001790	abnormal immune system physiology		0.0225	48.3	0.291	1	27	1975	PRPS2,PSAP,INHBB,CYP11A1,HLA-DQB1,PAPSS2,ADA,AKAP12,SMTN,TGM2,THRA,ANGPTL2,PXDN,MPZL3,SLPI,NGFR,KCNN4,CD68,CD74,GPNMB,MMP7,CX3CL1,CTSK,PREX1,ARHGEF6,CD276,LARGE
Mouse Phenotype	MP:0005369	muscle phenotype		0.00168	3.61	0.264	1	21	1140	NPPA,MYH11,PSAP,INHBB,CYP11A1,HLA-DQB1,ACTA1,JPH2,CPE,MRVI1,DYSF,AKAP12,SMTN,BMP4,THRA,MPZL3,KCNH2,NGFR,KCNN4,FBXO32,LARGE

Category	ID	Name	Source	p-value	q-value Bonferroni	q-value FDR B&H	q-value FDR B&Y	Hit Count in Query List	Hit Count in Genome	Hit in Query List
Mouse Phenotype	MP:0001819	abnormal immune cell physiology		0.022	47.3	0.291	1	19	1247	PRPS2,PSAP,CYP11A1,HLA-DQB1,ADA,TGM2,THRA,ANGPTL2,SLPI,KCNN4,CD68,CD74,GPNMB,MMP7,CX3CL1,CTSK,PREX1,ARHGEF6,CD276
Mouse Phenotype	MP:0002421	abnormal cell-mediated immunity		0.024	51.4	0.291	1	19	1258	PRPS2,PSAP,CYP11A1,HLA-DQB1,ADA,TGM2,THRA,ANGPTL2,SLPI,KCNN4,CD68,CD74,GPNMB,MMP7,CX3CL1,CTSK,PREX1,ARHGEF6,CD276
Mouse Phenotype	MP:0002420	abnormal adaptive immunity		0.0272	58.4	0.291	1	19	1275	PRPS2,PSAP,CYP11A1,HLA-DQB1,ADA,TGM2,THRA,ANGPTL2,SLPI,KCNN4,CD68,CD74,GPNMB,MMP7,CX3CL1,CTSK,PREX1,ARHGEF6,CD276
Mouse Phenotype	MP:0005381	digestive/alimentary phenotype		0.0109	23.3	0.286	1	18	1078	MYH11,PSAP,SPINK1,INHBB,HLA-DQB1,CLDN15,CPE,MRVI1,PAPSS2,ADA,SMTN,BMP4,THRA,NGFR,KCNN4,LHFPL2,MMP7,LARGE
Mouse Phenotype	MP:0002442	abnormal leukocyte physiology		0.0349	74.9	0.314	1	18	1221	PRPS2,PSAP,CYP11A1,HLA-DQB1,ADA,TGM2,THRA,ANGPTL2,SLPI,KCNN4,CD68,CD74,MMP7,CX3CL1,CTSK,PREX1,ARHGEF6,CD276
Mouse Phenotype	MP:0001732	postnatal growth retardation		0.00218	4.67	0.264	1	16	775	CDO1,MYH11,PSAP,SPINK1,CYP11A1,HLA-DQB1,ACSS1,PAPSS2,GGT1,SMTN,THRA,MPZL3,NGFR,STRA6,CTSK,LARGE
Mouse Phenotype	MP:0001731	abnormal postnatal growth		0.00293	6.29	0.264	1	16	798	CDO1,MYH11,PSAP,SPINK1,CYP11A1,HLA-DQB1,ACSS1,PAPSS2,GGT1,SMTN,THRA,MPZL3,NGFR,STRA6,CTSK,LARGE
Mouse Phenotype	MP:0002108	abnormal muscle morphology		0.00498	10.7	0.264	1	15	765	NPPA,MYH11,PSAP,INHBB,CYP11A1,HLA-DQB1,ACTA1,JPH2,DYSF,AKAP12,BMP4,MPZL3,NGFR,FBXO32,LARGE
Mouse Phenotype	MP:0000462	abnormal digestive system morphology		0.0108	23.1	0.286	1	15	833	MYH11,SPINK1,INHBB,CLDN15,MRVI1,PAPSS2,ADA,SMTN,BMP4,THRA,NGFR,KCNN4,LHFPL2,MMP7,LARGE
Mouse Phenotype	MP:0002452	abnormal professional antigen presenting cell physiology		0.0121	25.9	0.291	1	15	844	PRPS2,PSAP,HLA-DQB1,ADA,TGM2,THRA,ANGPTL2,SLPI,CD68,CD74,GPNMB,MMP7,CX3CL1,CTSK,ARHGEF6
Mouse Phenotype	MP:0002106	abnormal muscle physiology		0.0239	51.4	0.291	1	13	752	MYH11,PSAP,ACTA1,CPE,MRVI1,DYSF,AKAP12,SMTN,THRA,KCNH2,KCNN4,FBXO32,LARGE
Mouse Phenotype	MP:0002419	abnormal innate immunity		0.0113	24.3	0.289	1	11	532	PSAP,HLA-DQB1,TGM2,THRA,ANGPTL2,SLPI,CD68,MMP7,CX3CL1,CTSK,PREX1
Mouse Phenotype	MP:0008251	abnormal phagocyte morphology		0.0242	52	0.291	1	11	595	PSAP,KDM5A,ADA,GGT1,TGM2,THRA,NGFR,CD68,CX3CL1,CTSK,PREX1
Mouse Phenotype	MP:0003795	abnormal bone structure		0.031	66.4	0.305	1	11	618	CDO1,HTRA1,PAPSS2,GGT1,BMP4,THRA,MPZL3,CD68,CTSK,SCN3B,LARGE
Mouse Phenotype	MP:0002451	abnormal macrophage physiology		0.00192	4.11	0.264	1	10	357	PSAP,HLA-DQB1,TGM2,THRA,ANGPTL2,SLPI,CD68,MMP7,CX3CL1,CTSK
Mouse Phenotype	MP:0005253	abnormal eye physiology		0.00444	9.52	0.264	1	10	401	CDO1,INHBB,CRYAA,BMP4,PXDN,NGFR,LHFPL2,GPNMB,STRA6,LARGE
Mouse Phenotype	MP:0001921	reduced fertility		0.0206	44.2	0.291	1	10	505	INHBB,HLA-DQB1,CHDH,ACTA1,CPE,GGT1,BMP4,THRA,DCLK1,LARGE
Mouse Phenotype	MP:0001663	abnormal digestive system physiology		0.028	60.1	0.291	1	10	531	MYH11,PSAP,HLA-DQB1,CLDN15,CPE,MRVI1,SMTN,THRA,KCNN4,MMP7
Mouse Phenotype	MP:0004087	abnormal muscle fiber morphology		0.00351	7.54	0.264	1	9	325	MYH11,PSAP,HLA-DQB1,ACTA1,JPH2,DYSF,AKAP12,MPZL3,LARGE
Mouse Phenotype	MP:0002446	abnormal macrophage morphology		0.00708	15.2	0.264	1	9	362	PSAP,ADA,GGT1,TGM2,THRA,NGFR,CD68,CX3CL1,CTSK
Mouse Phenotype	MP:0005193	abnormal anterior eye segment morphology		0.015	32.2	0.291	1	9	409	DPT,PSAP,INHBB,GGT1,CRYAA,BMP4,PXDN,LHFPL2,GPNMB
Mouse Phenotype	MP:0005164	abnormal response to injury		0.0231	49.4	0.291	1	9	440	MYH11,RTN4R,TGM2,GJB3,SLPI,NGFR,KCNN4,MMP7,CX3CL1
Mouse Phenotype	MP:0008248	abnormal mononuclear phagocyte morphology		0.0319	68.3	0.311	1	9	466	PSAP,ADA,GGT1,TGM2,THRA,NGFR,CD68,CX3CL1,CTSK
Mouse Phenotype	MP:0004085	abnormal heartbeat		0.00113	2.42	0.264	1	8	221	NPPA,JPH2,CPE,MRVI1,TGM2,THRA,KCNH2,SCN3B
Mouse Phenotype	MP:0000249	abnormal blood vessel physiology		0.0176	37.7	0.291	1	8	350	MYH11,MRVI1,SMTN,BMP4,LIPG,NGFR,KCNN4,MMP7
Mouse Phenotype	MP:0001402	hypoactivity		0.0276	59.2	0.291	1	8	381	CDO1,PSAP,CYP11A1,RTN4R,ACTA1,CPE,PAK6,GGT1
Mouse Phenotype	MP:0001312	abnormal cornea morphology		0.00694	14.9	0.264	1	6	179	DPT,PSAP,INHBB,BMP4,PXDN,LHFPL2
Mouse Phenotype	MP:0020202	abnormal macrophage cell number		0.0143	30.6	0.291	1	6	209	ADA,GGT1,TGM2,THRA,CX3CL1,CTSK
Mouse Phenotype	MP:0000230	abnormal systemic arterial blood pressure		0.0146	31.2	0.291	1	6	210	NPPA,MYH11,MRVI1,SMTN,THRA,KCNN4
Mouse Phenotype	MP:0004174	abnormal spine curvature		0.0328	70.4	0.314	1	6	253	CDO1,PSAP,ACTA1,GGT1,CLEC3B,LARGE
Mouse Phenotype	MP:0004738	abnormal auditory brainstem response		0.0368	78.8	0.314	1	6	260	PSAP,BMP4,THRA,NGFR,LHFPL2,LARGE
Mouse Phenotype	MP:0001256	abnormal body length		0.0385	82.7	0.322	1	6	263	CDO1,CPE,PAPSS2,THRA,NGFR,STRA6
Mouse Phenotype	MP:0006335	abnormal hearing electrophysiology		0.0469	101	0.351	1	6	276	PSAP,BMP4,THRA,NGFR,LHFPL2,LARGE
Mouse Phenotype	MP:0006069	abnormal retinal neuronal layer morphology		0.0483	104	0.352	1	6	278	BMP4,PXDN,NGFR,LHFPL2,STRA6,LARGE
Mouse Phenotype	MP:0005425	increased macrophage cell number		0.00751	16.1	0.264	1	5	129	ADA,GGT1,TGM2,CX3CL1,CTSK
Mouse Phenotype	MP:0002376	abnormal dendritic cell physiology		0.0135	28.9	0.291	1	5	149	HLA-DQB1,ADA,CD68,CD74,GPNMB
Mouse Phenotype	MP:0000496	abnormal small intestine morphology		0.015	32.1	0.291	1	5	153	SPINK1,CLDN15,ADA,SMTN,THRA
Mouse Phenotype	MP:0000164	abnormal cartilage development		0.0162	34.7	0.291	1	5	156	PAPSS2,GGT1,BMP4,THRA,CLEC3B
Mouse Phenotype	MP:0005023	abnormal wound healing		0.0166	35.5	0.291	1	5	157	MYH11,GJB3,SLPI,NGFR,MMP7
Mouse Phenotype	MP:0000278	abnormal myocardial fiber morphology		0.0192	41.1	0.291	1	5	163	HLA-DQB1,JPH2,AKAP12,MPZL3,LARGE
Mouse Phenotype	MP:0006395	abnormal epiphyseal plate morphology		0.0201	43.1	0.291	1	5	165	PAPSS2,GGT1,THRA,CLEC3B,CTSK
Mouse Phenotype	MP:0003857	abnormal hindlimb zeugopod morphology		0.0241	51.7	0.291	1	5	173	GGT1,BMP4,THRA,PRELP,CTSK
Mouse Phenotype	MP:0000160	kyphosis		0.0252	54	0.291	1	5	175	CDO1,PSAP,GGT1,CLEC3B,LARGE
Mouse Phenotype	MP:0001629	abnormal heart rate		0.0252	54	0.291	1	5	175	NPPA,CPE,MRVI1,THRA,KCNH2
Mouse Phenotype	MP:0001513	limb grasping		0.0269	57.6	0.291	1	5	178	PSAP,DYSF,AKAP12,NGFR,LARGE
Mouse Phenotype	MP:0011082	abnormal gastrointestinal motility		0.00125	2.68	0.264	1	4	50	MYH11,PSAP,MRVI1,SMTN
Mouse Phenotype	MP:0001636	irregular heartbeat		0.00145	3.1	0.264	1	4	52	JPH2,TGM2,KCNH2,SCN3B
Mouse Phenotype	MP:0010875	increased bone volume		0.00217	4.66	0.264	1	4	58	HTRA1,THRA,CD68,CTSK
Mouse Phenotype	MP:0005468	abnormal thyroid hormone level		0.00231	4.96	0.264	1	4	59	CPE,GGT1,THRA,CTSK
Mouse Phenotype	MP:0002699	abnormal vitreous body morphology		0.0033	7.07	0.264	1	4	65	BMP4,PXDN,STRA6,LARGE
Mouse Phenotype	MP:0001158	abnormal prostate gland morphology		0.00388	8.33	0.264	1	4	68	PSAP,CYP11A1,AKAP12,BMP4
Mouse Phenotype	MP:0001314	corneal opacity		0.00551	11.8	0.264	1	4	75	INHBB,BMP4,PXDN,LHFPL2
Mouse Phenotype	MP:0002269	muscular atrophy		0.00855	18.4	0.264	1	4	85	PSAP,CYP11A1,ACTA1,FBXO32
Mouse Phenotype	MP:0005117	increased circulating pituitary hormone level		0.0125	26.9	0.291	1	4	95	INHBB,CYP11A1,CPE,THRA
Mouse Phenotype	MP:0001770	abnormal iron level		0.0125	26.9	0.291	1	4	95	SLC39A2,NGFR,KCNN4,ALAS2
Mouse Phenotype	MP:0000519	hydronephrosis		0.0125	26.9	0.291	1	4	95	MYH11,PSAP,PORCN,BMP4
Mouse Phenotype	MP:0005637	abnormal iron homeostasis		0.0149	31.9	0.291	1	4	100	SLC39A2,NGFR,KCNN4,ALAS2
Mouse Phenotype	MP:0005241	abnormal retinal ganglion layer morphology		0.0159	34.1	0.291	1	4	102	BMP4,PXDN,NGFR,LARGE
Mouse Phenotype	MP:0003973	increased pituitary hormone level		0.0192	41.3	0.291	1	4	108	INHBB,CYP11A1,CPE,THRA
Mouse Phenotype	MP:0010508	abnormal heart electrocardiography waveform feature		0.0198	42.5	0.291	1	4	109	HLA-DQB1,THRA,MMP7,SCN3B
Mouse Phenotype	MP:0002764	short tibia		0.021	45.1	0.291	1	4	111	GGT1,THRA,PRELP,CTSK
Mouse Phenotype	MP:0004194	abnormal kidney pelvis morphology		0.021	45.1	0.291	1	4	111	MYH11,PSAP,PORCN,BMP4

Category	ID	Name	Source	p-value	q-value Bonferroni	q-value FDR B&H	q-value FDR B&Y	Hit Count in Query List	Hit Count in Genome	Hit in Query List
Mouse Phenotype	MP:0011518	abnormal cell chemotaxis		0.0223	47.9	0.291	1	4	113	ANGPTL2,MMP7,CX3CL1,PREX1
Mouse Phenotype	MP:0010874	abnormal bone volume		0.025	53.6	0.291	1	4	117	HTRA1,THRA,CD68,CTSK
Mouse Phenotype	MP:0013330	abnormal male accessory sex gland morphology		0.0264	56.6	0.291	1	4	119	PSAP,CYP11A1,AKAP12,BMP4
Mouse Phenotype	MP:0003137	abnormal impulse conducting system conduction		0.0309	66.2	0.305	1	4	125	HLA-DQB1,THRA,MMP7,SCN3B
Mouse Phenotype	MP:0005595	abnormal vascular smooth muscle physiology		0.0316	67.9	0.31	1	4	126	MYH11,MRVI1,SMTN,KCNN4
Mouse Phenotype	MP:0005166	decreased susceptibility to injury		0.0366	78.6	0.314	1	4	132	TGM2,KCNN4,MMP7,CX3CL1
Mouse Phenotype	MP:0003448	altered tumor morphology		0.0375	80.4	0.314	1	4	133	TGM2,GPNMB,MMP7,DCLK1
Mouse Phenotype	MP:0005350	increased susceptibility to autoimmune disorder		0.042	90.2	0.344	1	4	138	HLA-DQB1,NGFR,CD74,CD276
Mouse Phenotype	MP:0001297	microphthalmia		0.0479	103	0.352	1	4	144	CRYAA,BMP4,PXDN,STRA6
Mouse Phenotype	MP:0000534	abnormal ureter morphology		0.0499	107	0.352	1	4	146	MYH11,PSAP,PORCN,BMP4
Mouse Phenotype	MP:0001163	abnormal prostate gland anterior lobe morphology		0.000511	1.1	0.264	1	3	17	CYP11A1,AKAP12,BMP4
Mouse Phenotype	MP:0004858	abnormal nervous system regeneration		0.000718	1.54	0.264	1	3	19	RTN4R,NGFR,LARGE
Mouse Phenotype	MP:0003289	abnormal intestinal peristalsis		0.000839	1.8	0.264	1	3	20	MYH11,PSAP,SMTN
Mouse Phenotype	MP:0004214	abnormal long bone diaphysis morphology		0.00112	2.4	0.264	1	3	22	BMP4,THRA,CTSK
Mouse Phenotype	MP:0000084	abnormal fontanelle morphology		0.00145	3.11	0.264	1	3	24	BMP4,THRA,CTSK
Mouse Phenotype	MP:0000479	abnormal enterocyte morphology		0.00279	5.98	0.264	1	3	30	CLDN15,SMTN,THRA
Mouse Phenotype	MP:0003271	abnormal duodenum morphology		0.00307	6.58	0.264	1	3	31	SPINK1,CLDN15,SMTN
Mouse Phenotype	MP:0006329	sensorineural hearing impairment		0.00336	7.21	0.264	1	3	32	PSAP,NGFR,LARGE
Mouse Phenotype	MP:0004740	sensorineural hearing loss		0.00336	7.21	0.264	1	3	32	PSAP,NGFR,LARGE
Mouse Phenotype	MP:0008511	thin retinal inner nuclear layer		0.004	8.58	0.264	1	3	34	BMP4,LHFPL2,LARGE
Mouse Phenotype	MP:0002455	abnormal dendritic cell antigen presentation		0.00435	9.32	0.264	1	3	35	HLA-DQB1,CD68,CD74
Mouse Phenotype	MP:0011996	abnormal retinal inner nuclear layer thickness		0.00435	9.32	0.264	1	3	35	BMP4,LHFPL2,LARGE
Mouse Phenotype	MP:0005300	abnormal corneal stroma morphology		0.00509	10.9	0.264	1	3	37	DPT,PSAP,PXDN
Mouse Phenotype	MP:0000740	impaired smooth muscle contractility		0.00509	10.9	0.264	1	3	37	MYH11,MRVI1,SMTN
Mouse Phenotype	MP:0009403	increased variability of skeletal muscle fiber size		0.00549	11.8	0.264	1	3	38	ACTA1,DYSF,LARGE
Mouse Phenotype	MP:0010868	increased bone trabecula number		0.00549	11.8	0.264	1	3	38	THRA,CD68,CTSK
Mouse Phenotype	MP:0011049	impaired adaptive thermogenesis		0.00591	12.7	0.264	1	3	39	ACSS1,CPE,THRA
Mouse Phenotype	MP:0001105	abnormal PNS glial cell morphology		0.00634	13.6	0.264	1	3	40	PSAP,NGFR,LARGE
Mouse Phenotype	MP:0001106	abnormal Schwann cell morphology		0.00634	13.6	0.264	1	3	40	PSAP,NGFR,LARGE
Mouse Phenotype	MP:0009347	increased trabecular bone thickness		0.00727	15.6	0.264	1	3	42	HTRA1,THRA,CTSK
Mouse Phenotype	MP:0003799	impaired macrophage chemotaxis		0.00828	17.8	0.264	1	3	44	ANGPTL2,MMP7,CX3CL1
Mouse Phenotype	MP:0000120	malocclusion		0.00828	17.8	0.264	1	3	44	CDO1,PAPSS2,CTSK
Mouse Phenotype	MP:0004874	abnormal timing of postnatal eyelid opening		0.0168	35.9	0.291	1	3	57	CDO1,INHBB,BMP4
Mouse Phenotype	MP:0003662	abnormal long bone epiphyseal plate proliferative zone		0.0175	37.6	0.291	1	3	58	PAPSS2,GGT1,THRA
Mouse Phenotype	MP:0004799	increased susceptibility to experimental autoimmune encephalomyelitis		0.0184	39.4	0.291	1	3	59	NGFR,CD74,CD276
Mouse Phenotype	MP:0005545	abnormal lens development		0.0184	39.4	0.291	1	3	59	CRYAA,BMP4,PXDN
Mouse Phenotype	MP:0004945	abnormal bone resorption		0.0192	41.2	0.291	1	3	60	THRA,CD68,CTSK
Mouse Phenotype	MP:0011019	abnormal adaptive thermogenesis		0.0192	41.2	0.291	1	3	60	ACSS1,CPE,THRA
Mouse Phenotype	MP:0010760	abnormal macrophage chemotaxis		0.0192	41.2	0.291	1	3	60	ANGPTL2,MMP7,CX3CL1
Mouse Phenotype	MP:0002212	abnormal secondary sex determination		0.0209	44.9	0.291	1	3	62	CYP11A1,GGT1,AKAP12
Mouse Phenotype	MP:0003733	abnormal retinal inner nuclear layer morphology		0.0228	48.8	0.291	1	3	64	BMP4,LHFPL2,LARGE
Mouse Phenotype	MP:0001541	abnormal osteoclast physiology		0.0256	55	0.291	1	3	67	THRA,CD68,CTSK
Mouse Phenotype	MP:0001835	abnormal antigen presentation		0.0266	57.1	0.291	1	3	68	HLA-DQB1,CD68,CD74
Mouse Phenotype	MP:0000747	muscle weakness		0.0266	57.1	0.291	1	3	68	PSAP,ACTA1,DYSF
Mouse Phenotype	MP:0000755	hindlimb paralysis		0.0354	76	0.314	1	3	76	PSAP,ACTA1,GGT1
Mouse Phenotype	MP:0000343	altered response to myocardial infarction		0.0391	83.8	0.322	1	3	79	TGM2,NGFR,MMP7
Mouse Phenotype	MP:0009345	abnormal trabecular bone thickness		0.0416	89.2	0.342	1	3	81	HTRA1,THRA,CTSK
Mouse Phenotype	MP:0004913	absent mandibular angle		0.000876	1.88	0.264	1	2	5	BMP4,CTSK
Mouse Phenotype	MP:0000578	ulcerated paws		0.00182	3.9	0.264	1	2	7	NGFR,CTSK
Mouse Phenotype	MP:0006200	vitreous body deposition		0.00182	3.9	0.264	1	2	7	PXDN,LARGE
Mouse Phenotype	MP:0009420	skeletal muscle endomysial fibrosis		0.00241	5.17	0.264	1	2	8	DYSF,LARGE
Mouse Phenotype	MP:0010964	increased compact bone volume		0.00308	6.6	0.264	1	2	9	THRA,CTSK
Mouse Phenotype	MP:0004220	abnormal peripheral nervous system regeneration		0.00382	8.2	0.264	1	2	10	NGFR,LARGE
Mouse Phenotype	MP:0003415	priapism		0.00382	8.2	0.264	1	2	10	ADA,GGT1
Mouse Phenotype	MP:0005480	increased circulating triiodothyronine level		0.00464	9.96	0.264	1	2	11	CPE,THRA
Mouse Phenotype	MP:0004596	abnormal mandibular angle morphology		0.00464	9.96	0.264	1	2	11	BMP4,CTSK
Mouse Phenotype	MP:0001107	decreased Schwann cell number		0.00464	9.96	0.264	1	2	11	NGFR,LARGE
Mouse Phenotype	MP:0008841	ruptured lens capsule		0.00554	11.9	0.264	1	2	12	CRYAA,PXDN
Mouse Phenotype	MP:0006226	iris hypoplasia		0.00554	11.9	0.264	1	2	12	BMP4,PXDN
Mouse Phenotype	MP:0009412	skeletal muscle fiber degeneration		0.00554	11.9	0.264	1	2	12	DYSF,LARGE
Mouse Phenotype	MP:0005474	increased triiodothyronine level		0.00554	11.9	0.264	1	2	12	CPE,THRA
Mouse Phenotype	MP:0003350	increased circulating levels of thyroid hormone		0.00651	14	0.264	1	2	13	CPE,THRA
Mouse Phenotype	MP:0003290	intestinal hypoperistalsis		0.00651	14	0.264	1	2	13	MYH11,SMTN
Mouse Phenotype	MP:0004002	abnormal jejunum morphology		0.00754	16.2	0.264	1	2	14	CLDN15,SMTN
Mouse Phenotype	MP:0001379	abnormal penile erection		0.00754	16.2	0.264	1	2	14	ADA,GGT1
Mouse Phenotype	MP:0010963	abnormal compact bone volume		0.00754	16.2	0.264	1	2	14	THRA,CTSK
Mouse Phenotype	MP:0006001	abnormal intestinal transit time		0.00865	18.6	0.264	1	2	15	MRVI1,SMTN
Mouse Phenotype	MP:0010755	abnormal heart right ventricle pressure		0.00865	18.6	0.264	1	2	15	NPPA,BMP4
Mouse Phenotype	MP:0004960	abnormal prostate gland weight		0.00983	21.1	0.267	1	2	16	PSAP,AKAP12
Mouse Phenotype	MP:0010966	abnormal compact bone area		0.0111	23.7	0.286	1	2	17	THRA,CTSK
Mouse Phenotype	MP:0009697	abnormal copulation		0.0111	23.7	0.286	1	2	17	ADA,GGT1
Mouse Phenotype	MP:0003236	abnormal lens capsule morphology		0.0124	26.5	0.291	1	2	18	CRYAA,PXDN
Mouse Phenotype	MP:0010254	nuclear cataracts		0.0137	29.5	0.291	1	2	19	GGT1,CRYAA
Mouse Phenotype	MP:0005257	abnormal intraocular pressure		0.0137	29.5	0.291	1	2	19	BMP4,GPNMB
Mouse Phenotype	MP:0003384	abnormal ventral body wall morphology		0.0152	32.6	0.291	1	2	20	PORCN,BMP4
Mouse Phenotype	MP:0009415	skeletal muscle degeneration		0.0152	32.6	0.291	1	2	20	DYSF,LARGE
Mouse Phenotype	MP:0005543	decreased cornea thickness		0.0152	32.6	0.291	1	2	20	DPT,BMP4
Mouse Phenotype	MP:0002769	abnormal vas deferens morphology		0.0167	35.8	0.291	1	2	21	CYP11A1,PORCN
Mouse Phenotype	MP:0009414	skeletal muscle fiber necrosis		0.0167	35.8	0.291	1	2	21	DYSF,LARGE
Mouse Phenotype	MP:0005042	abnormal level of surface class II molecules		0.0182	39.1	0.291	1	2	22	HLA-DQB1,CD74
Mouse Phenotype	MP:0003026	decreased vasoconstriction		0.0182	39.1	0.291	1	2	22	MYH11,SMTN
Mouse Phenotype	MP:0011961	abnormal cornea thickness		0.0182	39.1	0.291	1	2	22	DPT,BMP4
Mouse Phenotype	MP:0006398	increased long bone epiphyseal plate size		0.0182	39.1	0.291	1	2	22	THRA,CTSK
Mouse Phenotype	MP:0005198	abnormal aqueous drainage system morphology		0.0198	42.6	0.291	1	2	23	BMP4,PXDN
Mouse Phenotype	MP:0004434	abnormal cochlear outer hair cell physiology		0.0198	42.6	0.291	1	2	23	PSAP,THRA
Mouse Phenotype	MP:0004221	abnormal iridocorneal angle		0.0198	42.6	0.291	1	2	23	BMP4,PXDN
Mouse Phenotype	MP:0004696	abnormal thyroid follicle morphology		0.0198	42.6	0.291	1	2	23	THRA,CTSK
Mouse Phenotype	MP:0020280	increased creatine kinase level		0.0215	46.2	0.291	1	2	24	DYSF,LARGE
Mouse Phenotype	MP:0010090	increased circulating creatine kinase level		0.0215	46.2	0.291	1	2	24	DYSF,LARGE
Mouse Phenotype	MP:0005041	abnormal antigen presentation via MHC class II		0.0215	46.2	0.291	1	2	24	HLA-DQB1,CD74
Mouse Phenotype	MP:0004771	increased anti-single stranded DNA antibody level		0.0233	49.9	0.291	1	2	25	HLA-DQB1,CD276
Mouse Phenotype	MP:0000748	progressive muscle weakness		0.0233	49.9	0.291	1	2	25	PSAP,DYSF
Mouse Phenotype	MP:0009780	abnormal chondrocyte physiology		0.0233	49.9	0.291	1	2	25	PAPSS2,THRA
Mouse Phenotype	MP:0005476	abnormal circulating triiodothyronine level		0.0233	49.9	0.291	1	2	25	CPE,THRA
Mouse Phenotype	MP:0008828	abnormal lymph node cell ratio		0.0233	49.9	0.291	1	2	25	HLA-DQB1,CD74
Mouse Phenotype	MP:0010386	abnormal urinary bladder physiology		0.025	53.7	0.291	1	2	26	MYH11,PSAP
Mouse Phenotype	MP:0003237	abnormal lens epithelium morphology		0.025	53.7	0.291	1	2	26	CRYAA,PXDN
Mouse Phenotype	MP:0004263	abnormal limb posture		0.0269	57.6	0.291	1	2	27	CDO1,LARGE
Mouse Phenotype	MP:0002453	abnormal B lymphocyte antigen presentation		0.0269	57.6	0.291	1	2	27	HLA-DQB1,CD74
Mouse Phenotype	MP:0020279	abnormal creatine kinase level		0.0288	61.7	0.291	1	2	28	DYSF,LARGE

Category	ID	Name	Source	p-value	q-value Bonferroni	q-value FDR B&H	q-value FDR B&Y	Hit Count in Query List	Hit Count in Genome	Hit in Query List
Mouse Phenotype	MP:0003962	abnormal adrenaline level		0.0288	61.7	0.291	1	2	28	NPPA,CYP11A1
Mouse Phenotype	MP:0000065	abnormal bone marrow cavity morphology		0.0288	61.7	0.291	1	2	28	BMP4,CTSK
Mouse Phenotype	MP:0010089	abnormal circulating creatine kinase level		0.0288	61.7	0.291	1	2	28	DYSF,LARGE
Mouse Phenotype	MP:0009419	skeletal muscle fibrosis		0.0288	61.7	0.291	1	2	28	DYSF,LARGE
Mouse Phenotype	MP:0001290	delayed eyelid opening		0.0307	65.9	0.305	1	2	29	CDO1,INHBB
Mouse Phenotype	MP:0005472	abnormal triiodothyronine level		0.0307	65.9	0.305	1	2	29	CPE,THRA
Mouse Phenotype	MP:0004993	decreased bone resorption		0.0307	65.9	0.305	1	2	29	THRA,CD68
Mouse Phenotype	MP:0004876	decreased mean systemic arterial blood pressure		0.0307	65.9	0.305	1	2	29	MYH11,THRA
Mouse Phenotype	MP:0004432	abnormal cochlear hair cell physiology		0.0327	70.1	0.314	1	2	30	PSAP,THRA
Mouse Phenotype	MP:0004566	myocardial fiber degeneration		0.0327	70.1	0.314	1	2	30	HLA-DQB1,MPZL3
Mouse Phenotype	MP:0006396	decreased long bone epiphyseal plate size		0.0347	74.5	0.314	1	2	31	PAPSS2,GGT1
Mouse Phenotype	MP:0000752	dystrophic muscle		0.039	83.6	0.322	1	2	33	DYSF,LARGE
Mouse Phenotype	MP:0010878	increased trabecular bone volume		0.039	83.6	0.322	1	2	33	THRA,CTSK
Mouse Phenotype	MP:0008151	increased diameter of long bones		0.0434	93.1	0.35	1	2	35	THRA,CTSK
Mouse Phenotype	MP:0006143	increased systemic arterial diastolic blood pressure		0.0434	93.1	0.35	1	2	35	NPPA,KCNN4
Mouse Phenotype	MP:0002651	abnormal sciatic nerve morphology		0.0434	93.1	0.35	1	2	35	NGFR,LARGE
Mouse Phenotype	MP:0001792	impaired wound healing		0.0434	93.1	0.35	1	2	35	SLPI,MMP7
Mouse Phenotype	MP:0003852	skeletal muscle necrosis		0.0457	98	0.35	1	2	36	DYSF,LARGE
Mouse Phenotype	MP:0003966	abnormal adrenocorticotropin level		0.048	103	0.352	1	2	37	CYP11A1,CPE
Mouse Phenotype	MP:0000133	abnormal long bone metaphysis morphology		0.048	103	0.352	1	2	37	THRA,CTSK
Mouse Phenotype	MP:0004959	abnormal prostate gland size		0.048	103	0.352	1	2	37	PSAP,AKAP12
Mouse Phenotype	MP:0010539	decreased level of surface class II molecules		0.0096	20.6	0.264	1	1	1	HLA-DQB1
Mouse Phenotype	MP:0013796	abnormal duodenal goblet cell morphology		0.0096	20.6	0.264	1	1	1	CLDN15
Mouse Phenotype	MP:0006377	abnormal vestibulocollic reflex		0.0096	20.6	0.264	1	1	1	BMP4
Mouse Phenotype	MP:0004732	decreased circulating gastrin level		0.0096	20.6	0.264	1	1	1	CPE
Mouse Phenotype	MP:0009531	increased parotid gland size		0.0096	20.6	0.264	1	1	1	KCNN4
Mouse Phenotype	MP:0013730	glutathionuria		0.0096	20.6	0.264	1	1	1	GGT1
Mouse Phenotype	MP:0011641	abnormal pulmonary collagen fibril morphology		0.0096	20.6	0.264	1	1	1	CDO1
Mouse Phenotype	MP:0005520	decreased pancreas regeneration		0.0096	20.6	0.264	1	1	1	SPINK1
Mouse Phenotype	MP:0004006	impaired contractility of jejunal smooth muscle		0.0096	20.6	0.264	1	1	1	SMTN
Mouse Phenotype	MP:0003262	intestinal/bowel diverticulum		0.0096	20.6	0.264	1	1	1	SMTN
Mouse Phenotype	MP:0013798	abnormal jejunal goblet cell morphology		0.0096	20.6	0.264	1	1	1	CLDN15
Mouse Phenotype	MP:0010256	anterior cortical cataracts		0.0096	20.6	0.264	1	1	1	BMP4
Mouse Phenotype	MP:0010414	partial atrioventricular septal defect		0.0096	20.6	0.264	1	1	1	BMP4
Mouse Phenotype	MP:0008505	absent adrenal chromaffin cells		0.0096	20.6	0.264	1	1	1	CYP11A1
Mouse Phenotype	MP:0011851	abnormal embryonic cloaca development		0.0096	20.6	0.264	1	1	1	BMP4
Mouse Phenotype	MP:0012606	increased glutathione level		0.0096	20.6	0.264	1	1	1	GGT1
Mouse Phenotype	MP:0012603	abnormal glutathione level		0.0189	40.6	0.291	1	1	2	GGT1
Mouse Phenotype	MP:0011783	abnormal ureteral orifice morphology		0.0189	40.6	0.291	1	1	2	BMP4
Mouse Phenotype	MP:0005043	defective assembly of class II molecules		0.0189	40.6	0.291	1	1	2	CD74
Mouse Phenotype	MP:0006228	iris atrophy		0.0189	40.6	0.291	1	1	2	GPNMB
Mouse Phenotype	MP:0011882	enlarged duodenum		0.0189	40.6	0.291	1	1	2	CLDN15
Mouse Phenotype	MP:0012604	decreased glutathione level		0.0189	40.6	0.291	1	1	2	GGT1
Mouse Phenotype	MP:0004437	decreased cochlear outer hair cell electromotility		0.0189	40.6	0.291	1	1	2	THRA
Mouse Phenotype	MP:0009203	external male genitalia hypoplasia		0.0189	40.6	0.291	1	1	2	BMP4
Mouse Phenotype	MP:0004435	abnormal cochlear outer hair cell electromotility		0.0189	40.6	0.291	1	1	2	THRA
Mouse Phenotype	MP:0005518	abnormal pancreas regeneration		0.0189	40.6	0.291	1	1	2	SPINK1
Mouse Phenotype	MP:0004911	absent mandibular condyloid process		0.0189	40.6	0.291	1	1	2	BMP4
Mouse Phenotype	MP:0009483	enlarged ileum		0.0189	40.6	0.291	1	1	2	CLDN15
Mouse Phenotype	MP:0013794	abnormal intestinal villus goblet cell morphology		0.0189	40.6	0.291	1	1	2	CLDN15
Mouse Phenotype	MP:0009738	enlarged prostate gland anterior lobe		0.0189	40.6	0.291	1	1	2	AKAP12
Mouse Phenotype	MP:0003502	increased activity of thyroid		0.0189	40.6	0.291	1	1	2	THRA
Mouse Phenotype	MP:0010568	abnormal bulbus cordis morphology		0.0189	40.6	0.291	1	1	2	KCNH2
Mouse Phenotype	MP:0006250	abnormal line of Schwalbe morphology		0.0189	40.6	0.291	1	1	2	BMP4
Mouse Phenotype	MP:0004224	absent trabecular meshwork		0.0189	40.6	0.291	1	1	2	BMP4
Mouse Phenotype	MP:0003531	abnormal vagina development		0.0283	60.6	0.291	1	1	3	LHFPL2
Mouse Phenotype	MP:0013718	galactostasis		0.0283	60.6	0.291	1	1	3	INHBB
Mouse Phenotype	MP:0002770	absent bulbourethral gland		0.0283	60.6	0.291	1	1	3	CYP11A1
Mouse Phenotype	MP:0003285	gastric hypertrophy		0.0283	60.6	0.291	1	1	3	MRVI1
Mouse Phenotype	MP:0008796	increased lens fiber apoptosis		0.0283	60.6	0.291	1	1	3	CRYAA
Mouse Phenotype	MP:0008793	abnormal lens epithelium apoptosis		0.0283	60.6	0.291	1	1	3	CRYAA
Mouse Phenotype	MP:0013705	decreased megakaryocyte-erythroid progenitor cell number		0.0283	60.6	0.291	1	1	3	CD74
Mouse Phenotype	MP:0003094	abnormal cornea posterior stroma morphology		0.0283	60.6	0.291	1	1	3	DPT
Mouse Phenotype	MP:0009052	anal stenosis		0.0283	60.6	0.291	1	1	3	BMP4
Mouse Phenotype	MP:0003346	abnormal pectoral muscle morphology		0.0283	60.6	0.291	1	1	3	INHBB
Mouse Phenotype	MP:0009568	abnormal red blood cell deformability		0.0283	60.6	0.291	1	1	3	KCNN4
Mouse Phenotype	MP:0000741	impaired contractility of urinary bladder detrusor smooth muscle		0.0283	60.6	0.291	1	1	3	MYH11
Mouse Phenotype	MP:0008795	abnormal lens fiber apoptosis		0.0283	60.6	0.291	1	1	3	CRYAA
Mouse Phenotype	MP:0010713	corneal-lenticular stalk		0.0283	60.6	0.291	1	1	3	PXDN
Mouse Phenotype	MP:0011264	abnormal cardiac mesenchyme morphology		0.0283	60.6	0.291	1	1	3	BMP4
Mouse Phenotype	MP:0010255	cortical cataracts		0.0283	60.6	0.291	1	1	3	BMP4
Mouse Phenotype	MP:0012125	decreased bronchoconstrictive response		0.0283	60.6	0.291	1	1	3	MYH11
Mouse Phenotype	MP:0008794	increased lens epithelium apoptosis		0.0283	60.6	0.291	1	1	3	CRYAA
Mouse Phenotype	MP:0002213	true hermaphroditism		0.0283	60.6	0.291	1	1	3	PORCN
Mouse Phenotype	MP:0010455	aortopulmonary window		0.0283	60.6	0.291	1	1	3	BMP4
Mouse Phenotype	MP:0002771	absent prostate gland anterior lobe		0.0283	60.6	0.291	1	1	3	CYP11A1
Mouse Phenotype	MP:0008912	nervous		0.0283	60.6	0.291	1	1	3	GGT1
Mouse Phenotype	MP:0004797	increased anti-erythrocyte antigen antibody level		0.0283	60.6	0.291	1	1	3	HLA-DQB1
Mouse Phenotype	MP:0006258	abnormal circumvallate papillae morphology		0.0283	60.6	0.291	1	1	3	NGFR
Mouse Phenotype	MP:0010709	absent anterior chamber		0.0283	60.6	0.291	1	1	3	PXDN
Mouse Phenotype	MP:0003382	straub tail		0.0283	60.6	0.291	1	1	3	PSAP
Mouse Phenotype	MP:0000646	enlarged adrenocortical cells		0.0283	60.6	0.291	1	1	3	CYP11A1
Mouse Phenotype	MP:0001108	absent Schwann cells		0.0283	60.6	0.291	1	1	3	LARGE
Mouse Phenotype	MP:0011960	abnormal eye anterior chamber depth		0.0375	80.5	0.314	1	1	4	PXDN
Mouse Phenotype	MP:0013703	abnormal megakaryocyte-erythroid progenitor cell number		0.0375	80.5	0.314	1	1	4	CD74
Mouse Phenotype	MP:0004824	decreased susceptibility to experimental autoimmune myasthenia gravis		0.0375	80.5	0.314	1	1	4	HLA-DQB1
Mouse Phenotype	MP:0013792	abnormal small intestine goblet cell morphology		0.0375	80.5	0.314	1	1	4	CLDN15
Mouse Phenotype	MP:0010252	anterior subcapsular cataracts		0.0375	80.5	0.314	1	1	4	BMP4
Mouse Phenotype	MP:0002670	absent scrotum		0.0375	80.5	0.314	1	1	4	CYP11A1
Mouse Phenotype	MP:0004517	decreased vestibular hair cell stereocilia number		0.0375	80.5	0.314	1	1	4	BMP4
Mouse Phenotype	MP:0005302	neurogenic bladder		0.0375	80.5	0.314	1	1	4	PSAP
Mouse Phenotype	MP:0005081	abnormal dermis reticular layer morphology		0.0375	80.5	0.314	1	1	4	MPZL3
Mouse Phenotype	MP:0001136	dilated uterine cervix		0.0375	80.5	0.314	1	1	4	LHFPL2
Mouse Phenotype	MP:0006260	abnormal gustatory papillae taste bud morphology		0.0375	80.5	0.314	1	1	4	NGFR
Mouse Phenotype	MP:0011512	mesangial cell interposition		0.0375	80.5	0.314	1	1	4	EHD3
Mouse Phenotype	MP:0013283	failure of ventral body wall closure		0.0375	80.5	0.314	1	1	4	BMP4
Mouse Phenotype	MP:0004855	increased ovary weight		0.0375	80.5	0.314	1	1	4	INHBB
Mouse Phenotype	MP:0001002	abnormal taste bud morphology		0.0375	80.5	0.314	1	1	4	NGFR
Mouse Phenotype	MP:0003381	vitreal fibroplasia		0.0375	80.5	0.314	1	1	4	LARGE

Category	ID	Name	Source	p-value	q-value Bonferroni	q-value FDR B&H	q-value FDR B&Y	Hit Count in Query List	Hit Count in Genome	Hit in Query List
Mouse Phenotype	MP:0012511	dilated aortic sac		0.0375	80.5	0.314	1	1	4	KCNH2
Mouse Phenotype	MP:0003307	pyloric stenosis		0.0375	80.5	0.314	1	1	4	MRVI1
Mouse Phenotype	MP:0013614	abnormal areal bone mineral density		0.0375	80.5	0.314	1	1	4	THRA
Mouse Phenotype	MP:0005500	abnormal gustatory system morphology		0.0375	80.5	0.314	1	1	4	NGFR
Mouse Phenotype	MP:0013618	decreased areal bone mineral density		0.0375	80.5	0.314	1	1	4	THRA
Mouse Phenotype	MP:0003584	bifid ureter		0.0375	80.5	0.314	1	1	4	BMP4
Mouse Phenotype	MP:0012740	abnormal posterior primitive streak morphology		0.0375	80.5	0.314	1	1	4	BMP4
Mouse Phenotype	MP:0013702	abnormal megakaryocyte-erythroid progenitor cell morphology		0.0375	80.5	0.314	1	1	4	CD74
Mouse Phenotype	MP:0004005	impaired contractility of intestinal smooth muscle		0.0375	80.5	0.314	1	1	4	SMTN
Mouse Phenotype	MP:0006110	ventricular fibrillation		0.0375	80.5	0.314	1	1	4	TGM2
Mouse Phenotype	MP:0008293	abnormal adrenal gland zona glomerulosa morphology		0.0375	80.5	0.314	1	1	4	CYP11A1
Mouse Phenotype	MP:0013695	abnormal granulocyte monocyte progenitor cell number		0.0467	100	0.35	1	1	5	CD74
Mouse Phenotype	MP:0012437	increased Harderian gland tumor incidence		0.0467	100	0.35	1	1	5	FOXO4
Mouse Phenotype	MP:0001159	absent prostate gland		0.0467	100	0.35	1	1	5	CYP11A1
Mouse Phenotype	MP:0011488	abnormal ureterovesical junction morphology		0.0467	100	0.35	1	1	5	BMP4
Mouse Phenotype	MP:0010109	abnormal renal sodium reabsorbtion		0.0467	100	0.35	1	1	5	NPPA
Mouse Phenotype	MP:0009202	small external male genitalia		0.0467	100	0.35	1	1	5	BMP4
Mouse Phenotype	MP:0012510	abnormal aortic sac morphology		0.0467	100	0.35	1	1	5	KCNH2
Mouse Phenotype	MP:0003499	thyroid hypoplasia		0.0467	100	0.35	1	1	5	THRA
Mouse Phenotype	MP:0004226	absent Schlemm's canal		0.0467	100	0.35	1	1	5	BMP4
Mouse Phenotype	MP:0003622	ischuria		0.0467	100	0.35	1	1	5	MYH11
Mouse Phenotype	MP:0008837	increased transforming growth factor level		0.0467	100	0.35	1	1	5	SLPI
Mouse Phenotype	MP:0010399	decreased skeletal muscle glycogen level		0.0467	100	0.35	1	1	5	ACTA1
Mouse Phenotype	MP:0009255	degranulated pancreatic beta cells		0.0467	100	0.35	1	1	5	CPE
Mouse Phenotype	MP:0004899	absent squamosal bone		0.0467	100	0.35	1	1	5	BMP4
Mouse Phenotype	MP:0013697	decreased granulocyte monocyte progenitor cell number		0.0467	100	0.35	1	1	5	CD74
Mouse Phenotype	MP:0008162	increased diameter of tibia		0.0467	100	0.35	1	1	5	CTSK
Mouse Phenotype	MP:0011881	distended duodenum		0.0467	100	0.35	1	1	5	SMTN
Mouse Phenotype	MP:0013694	abnormal granulocyte monocyte progenitor cell morphology		0.0467	100	0.35	1	1	5	CD74
Mouse Phenotype	MP:0011455	absent glomerular endothelium fenestra		0.0467	100	0.35	1	1	5	EHD3
Domain	PF00089	Trypsin	Pfam	0.0000316	0.0257	0.00954	0.0695	7	118	KLK15,MASP1,KLK7,HTRA1,KLK10,KLK14,KLK1
Domain	IPR001254	Peptidase_S1_S6	InterPro	0.0000316	0.0257	0.00954	0.0695	7	118	KLK15,MASP1,KLK7,HTRA1,KLK10,KLK14,KLK1
Domain	IPR009003	Ser/Cys_Pept_Trypsin-like	InterPro	0.0000352	0.0286	0.00954	0.0695	7	120	KLK15,MASP1,KLK7,HTRA1,KLK10,KLK14,KLK1
Domain	IPR011992	EF-hand-like_dom	InterPro	0.00544	4.43	0.0862	0.627	7	280	PKD2L1,SPOCK2,CALML5,EHD3,TCHH,CCDC155,TCHHL1
Domain	G3DSA:1.10.238.10	EF-Hand_type	Gene3D	0.00544	4.43	0.0862	0.627	7	280	PKD2L1,SPOCK2,CALML5,EHD3,TCHH,CCDC155,TCHHL1
Domain	IPR018114	Peptidase_S1/S6_AS	InterPro	0.0000992	0.0807	0.0135	0.0985	6	98	KLK15,MASP1,KLK7,KLK10,KLK14,KLK1
Domain	PS00135	TRYPSIN_SER	PROSITE	0.000145	0.118	0.0135	0.0985	6	105	KLK15,MASP1,KLK7,KLK10,KLK14,KLK1
Domain	PS00134	TRYPSIN_HIS	PROSITE	0.000145	0.118	0.0135	0.0985	6	105	KLK15,MASP1,KLK7,KLK10,KLK14,KLK1
Domain	IPR001314	Peptidase_S1A	InterPro	0.000188	0.153	0.0135	0.0985	6	110	KLK15,MASP1,KLK7,KLK10,KLK14,KLK1
Domain	PS50240	TRYPSIN_DOM	PROSITE	0.000207	0.168	0.0135	0.0985	6	112	KLK15,MASP1,KLK7,KLK10,KLK14,KLK1
Domain	SM00020	Tryp_SPc	SMART	0.000228	0.185	0.0135	0.0985	6	114	KLK15,MASP1,KLK7,KLK10,KLK14,KLK1
Domain	PF00168	C2	Pfam	0.015	12.2	0.115	0.835	4	125	CPNE2,RAB11FIP1,DYSF,CAPN5
Domain	SM00239	C2	SMART	0.0189	15.4	0.115	0.839	4	134	CPNE2,RAB11FIP1,DYSF,CAPN5
Domain	IPR000008	C2_Ca-dep	InterPro	0.0203	16.5	0.115	0.839	4	137	CPNE2,RAB11FIP1,DYSF,CAPN5
Domain	PS50004	C2	PROSITE	0.0213	17.3	0.115	0.839	4	139	CPNE2,RAB11FIP1,DYSF,CAPN5
Domain	IPR008973	C2_Ca/lipid-bd_dom_CaLB	InterPro	0.0289	23.6	0.124	0.904	4	153	CPNE2,RAB11FIP1,DYSF,CAPN5
Domain	PF07686	V-set	Pfam	0.0487	39.6	0.144	1	4	181	AMICA1,MPZL3,SCN3B,CD276
Domain	IPR013106	Ig_V-set	InterPro	0.0487	39.6	0.144	1	4	181	AMICA1,MPZL3,SCN3B,CD276
Domain	PF00086	Thyroglobulin_1	Pfam	0.000266	0.217	0.0135	0.0985	3	17	SPOCK2,CD74,TACSTD2
Domain	SM00211	TY	SMART	0.000266	0.217	0.0135	0.0985	3	17	SPOCK2,CD74,TACSTD2
Domain	PS51162	THYROGLOBULIN_1_2	PROSITE	0.000266	0.217	0.0135	0.0985	3	17	SPOCK2,CD74,TACSTD2
Domain	PS00484	THYROGLOBULIN_1_1	PROSITE	0.000266	0.217	0.0135	0.0985	3	17	SPOCK2,CD74,TACSTD2
Domain	G3DSA:4.10.800.10	Thyroglobulin_1	Gene3D	0.000266	0.217	0.0135	0.0985	3	17	SPOCK2,CD74,TACSTD2
Domain	IPR000716	Thyroglobulin_1	InterPro	0.000266	0.217	0.0135	0.0985	3	17	SPOCK2,CD74,TACSTD2
Domain	SM00280	KAZAL	SMART	0.00476	3.87	0.0862	0.627	3	45	HTRA1,SPINK1,SPOCK2
Domain	IPR002350	Prot_inh_Kazal	InterPro	0.00476	3.87	0.0862	0.627	3	45	HTRA1,SPINK1,SPOCK2
Domain	PS51465	KAZAL_2	PROSITE	0.0057	4.64	0.0862	0.627	3	48	HTRA1,SPINK1,SPOCK2
Domain	PS50096	IQ	PROSITE	0.0317	25.8	0.126	0.915	3	91	PCP4,MYH11,NRGN
Domain	IPR000372	LRR-contain_N	InterPro	0.0373	30.3	0.129	0.936	3	97	RTN4R,PXDN,PRELP
Domain	SM00013	LRRNT	SMART	0.0373	30.3	0.129	0.936	3	97	RTN4R,PXDN,PRELP
Domain	IPR018029	C2_membr_targeting	InterPro	0.0454	37	0.139	1	3	105	CPNE2,RAB11FIP1,DYSF
Domain	IPR001500	A1A_glycop	InterPro	0.0000572	0.0466	0.0116	0.0847	2	2	ORM1,ORM2
Domain	IPR014766	CarboxyPept_regulatory_dom	InterPro	0.00419	3.41	0.0862	0.627	2	13	CPE,CPZ
Domain	G3DSA:2.60.40.1120	CarboxyPept_regulatory	Gene3D	0.00419	3.41	0.0862	0.627	2	13	CPE,CPZ
Domain	IPR008969	CarboxyPept-like_regulatory	InterPro	0.00635	5.17	0.0862	0.627	2	16	CPE,CPZ
Domain	PS00213	LIPOCALIN	PROSITE	0.00717	5.83	0.0862	0.627	2	17	ORM1,ORM2
Domain	SM00631	Zn_pept	SMART	0.00717	5.83	0.0862	0.627	2	17	CPE,CPZ
Domain	IPR002345	Lipocalin	InterPro	0.00802	6.53	0.0895	0.651	2	18	ORM1,ORM2
Domain	PS00133	CARBOXYPEPT_ZN_2	PROSITE	0.00892	7.26	0.0981	0.715	2	19	CPE,CPZ
Domain	PS00132	CARBOXYPEPT_ZN_1	PROSITE	0.00987	8.03	0.107	0.779	2	20	CPE,CPZ
Domain	PF07885	Ion_trans_2	Pfam	0.0109	8.83	0.115	0.835	2	21	KCNK12,KCNN4
Domain	IPR013099	Ion_trans_2	InterPro	0.0109	8.83	0.115	0.835	2	21	KCNK12,KCNN4
Domain	PF00246	Peptidase_M14	Pfam	0.0119	9.67	0.115	0.835	2	22	CPE,CPZ
Domain	IPR000834	Peptidase_M14	InterPro	0.0119	9.67	0.115	0.835	2	22	CPE,CPZ
Domain	IPR014715	Fibrinogen_a/b/g_C_2	InterPro	0.0141	11.4	0.115	0.835	2	24	MFAP4,ANGPTL2
Domain	G3DSA:4.10.530.10	Fibrinogen_a/b/g_C_2	Gene3D	0.0141	11.4	0.115	0.835	2	24	MFAP4,ANGPTL2
Domain	PS00139	THIOL_PROTEASE_CYS	PROSITE	0.0164	13.3	0.115	0.839	2	26	CAPN5,CTSK
Domain	PF01023	S_100	Pfam	0.0176	14.3	0.115	0.839	2	27	TCHH,TCHHL1
Domain	G3DSA:3.30.470.20	ATP_grasp_subdomain_2	Gene3D	0.0176	14.3	0.115	0.839	2	27	GSS,RIMKLB
Domain	IPR014716	Fibrinogen_a/b/g_C_1	InterPro	0.0176	14.3	0.115	0.839	2	27	MFAP4,ANGPTL2
Domain	PS00639	THIOL_PROTEASE_HIS	PROSITE	0.0176	14.3	0.115	0.839	2	27	CAPN5,CTSK
Domain	PF00147	Fibrinogen_C	Pfam	0.0176	14.3	0.115	0.839	2	27	MFAP4,ANGPTL2
Domain	PS00303	S100_CABP	PROSITE	0.0176	14.3	0.115	0.839	2	27	TCHH,TCHHL1
Domain	IPR013787	S100_Ca-bd_sub	InterPro	0.0176	14.3	0.115	0.839	2	27	TCHH,TCHHL1
Domain	G3DSA:3.90.215.10	Fibrinogen_a/b/g_C_1	Gene3D	0.0176	14.3	0.115	0.839	2	27	MFAP4,ANGPTL2
Domain	PS00640	THIOL_PROTEASE_ASN	PROSITE	0.0176	14.3	0.115	0.839	2	27	CAPN5,CTSK
Domain	IPR001111	TGF-b_N	InterPro	0.0189	15.4	0.115	0.839	2	28	INHBB,BMP4
Domain	PF00688	TGFb_propeptide	Pfam	0.0189	15.4	0.115	0.839	2	28	INHBB,BMP4
Domain	IPR000169	Pept_cys_AS	InterPro	0.0189	15.4	0.115	0.839	2	28	CAPN5,CTSK
Domain	SM00186	FBG	SMART	0.0202	16.4	0.115	0.839	2	29	MFAP4,ANGPTL2
Domain	IPR013816	ATP_grasp_subdomain_2	InterPro	0.0215	17.5	0.115	0.839	2	30	GSS,RIMKLB
Domain	IPR015615	TGF-beta-rel	InterPro	0.0229	18.6	0.116	0.842	2	31	INHBB,BMP4
Domain	IPR017948	TGFb_CS	InterPro	0.0229	18.6	0.116	0.842	2	31	INHBB,BMP4
Domain	IPR002181	Fibrinogen_a/b/g_C	InterPro	0.0243	19.8	0.121	0.877	2	32	MFAP4,ANGPTL2
Domain	PS00514	FIBRINOGEN_C_1	PROSITE	0.0243	19.8	0.121	0.877	2	32	MFAP4,ANGPTL2

Category	ID	Name	Source	p-value	q-value Bonferroni	q-value FDR B&H	q-value FDR B&Y	Hit Count in Query List	Hit Count in Genome	Hit in Query List
Domain	PS51406	FIBRINOGEN_C_2	PROSITE	0.0243	19.8	0.121	0.877	2	32	MFAP4,ANGPTL2
Domain	PF07648	Kazal_2	Pfam	0.0257	20.9	0.124	0.904	2	33	HTRA1,SPOCK2
Domain	IPR011497	Prot_Inh_Kazal_2	InterPro	0.0257	20.9	0.124	0.904	2	33	HTRA1,SPOCK2
Domain	PF00061	Lipocalin	Pfam	0.0272	22.1	0.124	0.904	2	34	ORM1,ORM2
Domain	IPR000566	Lipocln_cytosolic_FA-bd	InterPro	0.0272	22.1	0.124	0.904	2	34	ORM1,ORM2
Domain	PS51362	TGF_BETA_2	PROSITE	0.0302	24.6	0.125	0.91	2	36	INHBB,BMP4
Domain	IPR011038	Calycin-like	InterPro	0.0318	25.9	0.126	0.915	2	37	ORM1,ORM2
Domain	SM00204	TGFB	SMART	0.0318	25.9	0.126	0.915	2	37	INHBB,BMP4
Domain	IPR012674	Calycin	InterPro	0.0318	25.9	0.126	0.915	2	37	ORM1,ORM2
Domain	PF00019	TGF_beta	Pfam	0.0318	25.9	0.126	0.915	2	37	INHBB,BMP4
Domain	PS00250	TGF_BETA_1	PROSITE	0.0318	25.9	0.126	0.915	2	37	INHBB,BMP4
Domain	G3DSA:2.40.128.20	Calycin	Gene3D	0.0318	25.9	0.126	0.915	2	37	ORM1,ORM2
Domain	IPR011162	MHC_I/II-like_Ag-recog	InterPro	0.0318	25.9	0.126	0.915	2	37	HLA-DQB1,PROCR
Domain	IPR001839	TGF-b_C	InterPro	0.0318	25.9	0.126	0.915	2	37	INHBB,BMP4
Domain	IPR001331	GDS_CDC24_CS	InterPro	0.0351	28.5	0.129	0.936	2	39	PREX1,ARHGEF6
Domain	IPR002407	At__natriurtcpep	InterPro	0.00762	6.2	0.0862	0.627	1	1	NPPA
Domain	IPR008094	Claudin15	InterPro	0.00762	6.2	0.0862	0.627	1	1	CLDN15
Domain	IPR022189	Smoothelin	InterPro	0.00762	6.2	0.0862	0.627	1	1	SMTN
Domain	PF05995	CDO_I	Pfam	0.00762	6.2	0.0862	0.627	1	1	CDO1
Domain	IPR004141	Strictosidine_synthase	InterPro	0.00762	6.2	0.0862	0.627	1	1	APMAP
Domain	PS01105	RIBOSOMAL_L35AE	PROSITE	0.00762	6.2	0.0862	0.627	1	1	RPL35A
Domain	IPR015669	Endothetial_C_recpt	InterPro	0.00762	6.2	0.0862	0.627	1	1	PROCR
Domain	IPR018119	Strictosidine_synth_cons-reg	InterPro	0.00762	6.2	0.0862	0.627	1	1	APMAP
Domain	IPR022325	TNFR_16	InterPro	0.00762	6.2	0.0862	0.627	1	1	NGFR
Domain	IPR000172	GMC_OxRdtase_N	InterPro	0.00762	6.2	0.0862	0.627	1	1	CHDH
Domain	IPR001780	Ribosomal_L35Ae	InterPro	0.00762	6.2	0.0862	0.627	1	1	RPL35A
Domain	IPR002269	Connexin31	InterPro	0.00762	6.2	0.0862	0.627	1	1	GJB3
Domain	PF01247	Ribosomal_L35Ae	Pfam	0.00762	6.2	0.0862	0.627	1	1	RPL35A
Domain	IPR015644	Peptidase_C1A_cathepsin-K	InterPro	0.00762	6.2	0.0862	0.627	1	1	CTSK
Domain	IPR011988	MHC_II-assoc_invariant_trimer	InterPro	0.00762	6.2	0.0862	0.627	1	1	CD74
Domain	IPR012274	Alpha-crystallin_A	InterPro	0.00762	6.2	0.0862	0.627	1	1	CRYAA
Domain	PF00732	GMC_oxred_N	Pfam	0.00762	6.2	0.0862	0.627	1	1	CHDH
Domain	PF05199	GMC_oxred_C	Pfam	0.00762	6.2	0.0862	0.627	1	1	CHDH
Domain	G3DSA:1.10.870.10	MHCII_invariant	Gene3D	0.00762	6.2	0.0862	0.627	1	1	CD74
Domain	PF08831	MHCassoc_trimer	Pfam	0.00762	6.2	0.0862	0.627	1	1	CD74
Domain	IPR022339	MHC_II-assoc_invar_chain	InterPro	0.00762	6.2	0.0862	0.627	1	1	CD74
Domain	IPR012307	Xyl_isomerase_TIM-brl	InterPro	0.00762	6.2	0.0862	0.627	1	1	HYI
Domain	IPR014042	Glutathione_synthase_a-hlx_euk	InterPro	0.00762	6.2	0.0862	0.627	1	1	GSS
Domain	PF03199	GSH_synthase	Pfam	0.00762	6.2	0.0862	0.627	1	1	GSS
Domain	PS00624	GMC_OXRED_2	PROSITE	0.00762	6.2	0.0862	0.627	1	1	CHDH
Domain	G3DSA:1.10.1080.10	Glutathione_synthase_a-hlx_euk	Gene3D	0.00762	6.2	0.0862	0.627	1	1	GSS
Domain	IPR012132	GMC_OxRdtase	InterPro	0.00762	6.2	0.0862	0.627	1	1	CHDH
Domain	PD012670	Ribosomal_L35Ae	ProDom	0.00762	6.2	0.0862	0.627	1	1	RPL35A
Domain	IPR010300	Cys_dOase_I	InterPro	0.00762	6.2	0.0862	0.627	1	1	CDO1
Domain	G3DSA:3.30.1490.80	Glutathione_synthase_N_euk	Gene3D	0.00762	6.2	0.0862	0.627	1	1	GSS
Domain	G3DSA:3.40.50.1760	Glutathione_synth_subst-bd_euk	Gene3D	0.00762	6.2	0.0862	0.627	1	1	GSS
Domain	IPR013022	Xyl_isomerase-like_TIM-brl	InterPro	0.00762	6.2	0.0862	0.627	1	1	HYI
Domain	G3DSA:3.20.20.150	Xyl_isomerase-like_TIM-brl	Gene3D	0.00762	6.2	0.0862	0.627	1	1	HYI
Domain	IPR018266	Ribosomal_L35Ae_CS	InterPro	0.00762	6.2	0.0862	0.627	1	1	RPL35A
Domain	PF09307	MHC2-interact	Pfam	0.00762	6.2	0.0862	0.627	1	1	CD74
Domain	IPR007867	GMC_OxRtase_C	InterPro	0.00762	6.2	0.0862	0.627	1	1	CHDH
Domain	IPR005615	Glutathione_synthase_euk	InterPro	0.00762	6.2	0.0862	0.627	1	1	GSS
Domain	PF03917	GSH_synth_ATP	Pfam	0.00762	6.2	0.0862	0.627	1	1	GSS
Domain	PF03088	Str_synth	Pfam	0.00762	6.2	0.0862	0.627	1	1	APMAP
Domain	PF01261	AP_endonuc_2	Pfam	0.00762	6.2	0.0862	0.627	1	1	HYI
Domain	IPR004625	PyrdxlP_synth_PyrdxlKinase	InterPro	0.00762	6.2	0.0862	0.627	1	1	PDXK
Domain	IPR015386	MHC_II-assoc_invar/CLIP_MHC-bd	InterPro	0.00762	6.2	0.0862	0.627	1	1	CD74
Domain	IPR004887	Glutathione_synth_subst-bd_euk	InterPro	0.00762	6.2	0.0862	0.627	1	1	GSS
Domain	PF12510	Smoothelin	Pfam	0.00762	6.2	0.0862	0.627	1	1	SMTN
Domain	PS00623	GMC_OXRED_1	PROSITE	0.00762	6.2	0.0862	0.627	1	1	CHDH
Domain	IPR014049	Glutathione_synthase_N_euk	InterPro	0.00762	6.2	0.0862	0.627	1	1	GSS
Domain	IPR010891	GumN-prd	InterPro	0.0151	12.3	0.115	0.835	1	2	TRABD2B
Domain	IPR004307	TspO_MBR	InterPro	0.0151	12.3	0.115	0.835	1	2	TSPO
Domain	PF03832	WSK	Pfam	0.0151	12.3	0.115	0.835	1	2	AKAP12
Domain	IPR001573	Pkinase-A_anch_WSK-motif	InterPro	0.0151	12.3	0.115	0.835	1	2	AKAP12
Domain	IPR011904	Ac_CoA_lig	InterPro	0.0151	12.3	0.115	0.835	1	2	ACSS1
Domain	PF05781	MRVI1	Pfam	0.0151	12.3	0.115	0.835	1	2	MRVI1
Domain	IPR000381	Inhibin_betaB	InterPro	0.0151	12.3	0.115	0.835	1	2	INHBB
Domain	PF03073	TspO_MBR	Pfam	0.0151	12.3	0.115	0.835	1	2	TSPO
Domain	IPR004666	RimK_fam	InterPro	0.0151	12.3	0.115	0.835	1	2	RIMKLB
Domain	IPR008677	MRVI1	InterPro	0.0151	12.3	0.115	0.835	1	2	MRVI1
Domain	IPR017417	Pase-1_reg_su_16AB_euk	InterPro	0.0151	12.3	0.115	0.835	1	2	PPP1R16B
Domain	IPR005410	K_chnl_2pore_THIK	InterPro	0.0151	12.3	0.115	0.835	1	2	KCNK12
Domain	IPR002891	APS_kinase_C	InterPro	0.0151	12.3	0.115	0.835	1	2	PAPSS2
Domain	IPR021165	Saposin_chordata	InterPro	0.0151	12.3	0.115	0.835	1	2	PSAP
Domain	PF08443	RimK	Pfam	0.0151	12.3	0.115	0.835	1	2	RIMKLB
Domain	IPR002650	Sulphate_adenylyltransferase	InterPro	0.0151	12.3	0.115	0.835	1	2	PAPSS2
Domain	IPR002330	Lipo_Lipase	InterPro	0.0151	12.3	0.115	0.835	1	2	LIPG
Domain	IPR010961	4pyrrol_synth_NH2levulA_synth	InterPro	0.0151	12.3	0.115	0.835	1	2	ALAS2
Domain	IPR015118	5aminolev_synth_preseq	InterPro	0.0151	12.3	0.115	0.835	1	2	ALAS2
Domain	PF07446	GumN	Pfam	0.0151	12.3	0.115	0.835	1	2	TRABD2B
Domain	PF01747	ATP-sulfurylase	Pfam	0.0151	12.3	0.115	0.835	1	2	PAPSS2
Domain	PF09029	Preseq_ALAS	Pfam	0.0151	12.3	0.115	0.835	1	2	ALAS2
Domain	IPR006330	A_deaminase	InterPro	0.0151	12.3	0.115	0.835	1	2	ADA
Domain	PF01583	APS_kinase	Pfam	0.0151	12.3	0.115	0.835	1	2	PAPSS2
Domain	IPR013651	ATP-grasp_RimK-type	InterPro	0.0151	12.3	0.115	0.835	1	2	RIMKLB
Domain	IPR008758	Peptidase_S28	InterPro	0.0225	18.3	0.115	0.839	1	3	DPP7
Domain	IPR018134	LAMP_CS	InterPro	0.0225	18.3	0.115	0.839	1	3	CD68
Domain	PS51110	SAP_A	PROSITE	0.0225	18.3	0.115	0.839	1	3	PSAP
Domain	PS00263	NATRIURETIC_PEPTIDE	PROSITE	0.0225	18.3	0.115	0.839	1	3	NPPA
Domain	IPR003119	SapA	InterPro	0.0225	18.3	0.115	0.839	1	3	PSAP
Domain	SM00183	NAT_PEP	SMART	0.0225	18.3	0.115	0.839	1	3	NPPA
Domain	PF08165	FerA	Pfam	0.0225	18.3	0.115	0.839	1	3	DYSF
Domain	PF02199	SapA	Pfam	0.0225	18.3	0.115	0.839	1	3	PSAP
Domain	PF05184	SapB_1	Pfam	0.0225	18.3	0.115	0.839	1	3	PSAP
Domain	PF05978	DUF895	Pfam	0.0225	18.3	0.115	0.839	1	3	UNC93A
Domain	IPR003090	Alpha-crystallin_N	InterPro	0.0225	18.3	0.115	0.839	1	3	CRYAA
Domain	PF05577	Peptidase_S28	Pfam	0.0225	18.3	0.115	0.839	1	3	DPP7
Domain	IPR007856	SapB_1	InterPro	0.0225	18.3	0.115	0.839	1	3	PSAP
Domain	IPR003967	K_chnl_volt-dep_ERG	InterPro	0.0225	18.3	0.115	0.839	1	3	KCNH2
Domain	IPR000434	PKD_1	InterPro	0.0225	18.3	0.115	0.839	1	3	PKD2L1
Domain	IPR000663	Natr_peptide	InterPro	0.0225	18.3	0.115	0.839	1	3	NPPA
Domain	IPR012560	Ferlin_A-domain	InterPro	0.0225	18.3	0.115	0.839	1	3	DYSF
Domain	IPR010291	Ion_channel_UNC-93	InterPro	0.0225	18.3	0.115	0.839	1	3	UNC93A
Domain	IPR001728	ThyrH_rcpt	InterPro	0.0225	18.3	0.115	0.839	1	3	THRA

Category	ID	Name	Source	p-value	q-value Bonferroni	q-value FDR B&H	q-value FDR B&Y	Hit Count in Query List	Hit Count in Genome	Hit in Query List
Domain	IPR000842	PRib_PP_synth_CS	InterPro	0.0225	18.3	0.115	0.839	1	3	PRPS2
Domain	PS00114	PRPP_SYNTHASE	PROSITE	0.0225	18.3	0.115	0.839	1	3	PRPS2
Domain	PF04571	Lipin_N	Pfam	0.0225	18.3	0.115	0.839	1	3	LPIN3
Domain	PF00525	Crystallin	Pfam	0.0225	18.3	0.115	0.839	1	3	CRYAA
Domain	IPR007599	DER1	InterPro	0.0225	18.3	0.115	0.839	1	3	DERL3
Domain	IPR007651	Lipin_N	InterPro	0.0225	18.3	0.115	0.839	1	3	LPIN3
Domain	PF04511	DER1	Pfam	0.0225	18.3	0.115	0.839	1	3	DERL3
Domain	PF07970	DUF1692	Pfam	0.0225	18.3	0.115	0.839	1	3	ERGIC1
Domain	PS00311	LAMP_2	PROSITE	0.0225	18.3	0.115	0.839	1	3	CD68
Domain	IPR008373	Saposin	InterPro	0.0225	18.3	0.115	0.839	1	3	PSAP
Domain	IPR012936	DUF1692	InterPro	0.0225	18.3	0.115	0.839	1	3	ERGIC1
Domain	PF00212	ANP	Pfam	0.0225	18.3	0.115	0.839	1	3	NPPA
Domain	IPR020649	Ser/Thr_prot_kinase_DCLK	InterPro	0.0225	18.3	0.115	0.839	1	3	DCLK1
Domain	SM00693	DysFN	SMART	0.0225	18.3	0.115	0.839	1	3	DYSF
Domain	SM00162	SAPA	SMART	0.0225	18.3	0.115	0.839	1	3	PSAP
Domain	IPR019383	Golgin_A_7/ERF4	InterPro	0.0299	24.4	0.124	0.904	1	4	GOLGA7B
Domain	IPR015449	K_chnl_Ca-activ_SK	InterPro	0.0299	24.4	0.124	0.904	1	4	KCNN4
Domain	IPR017191	Junctophilin	InterPro	0.0299	24.4	0.124	0.904	1	4	JPH2
Domain	PF05672	MAP7	Pfam	0.0299	24.4	0.124	0.904	1	4	MAP7D2
Domain	IPR001940	Peptidase_S1C	InterPro	0.0299	24.4	0.124	0.904	1	4	HTRA1
Domain	PF03530	SK_channel	Pfam	0.0299	24.4	0.124	0.904	1	4	KCNN4
Domain	IPR004021	HIN200/IF120x	InterPro	0.0299	24.4	0.124	0.904	1	4	IFI16
Domain	PF00294	PfkB	Pfam	0.0299	24.4	0.124	0.904	1	4	PDXK
Domain	IPR011611	Carb/pur_kinase	InterPro	0.0299	24.4	0.124	0.904	1	4	PDXK
Domain	PF10256	Erf4	Pfam	0.0299	24.4	0.124	0.904	1	4	GOLGA7B
Domain	SM00694	DysFC	SMART	0.0299	24.4	0.124	0.904	1	4	DYSF
Domain	PF02880	PGM_PMM_III	Pfam	0.0299	24.4	0.124	0.904	1	4	PGM5
Domain	IPR002050	Env_polyprotein	InterPro	0.0299	24.4	0.124	0.904	1	4	ERV3-1
Domain	PS50834	HIN_200	PROSITE	0.0299	24.4	0.124	0.904	1	4	IFI16
Domain	IPR004178	CaM-bd_dom	InterPro	0.0299	24.4	0.124	0.904	1	4	KCNN4
Domain	IPR006650	A/AMP_deam_AS	InterPro	0.0299	24.4	0.124	0.904	1	4	ADA
Domain	PF08429	PLU-1	Pfam	0.0299	24.4	0.124	0.904	1	4	KDM5A
Domain	IPR016066	A-D-PHexomutase_CS	InterPro	0.0299	24.4	0.124	0.904	1	4	PGM5
Domain	IPR008097	Fractalkine	InterPro	0.0299	24.4	0.124	0.904	1	4	CX3CL1
Domain	PF02888	CaMBD	Pfam	0.0299	24.4	0.124	0.904	1	4	KCNN4
Domain	IPR005846	A-D-PHexomutase_a/b/a-III	InterPro	0.0299	24.4	0.124	0.904	1	4	PGM5
Domain	IPR008604	E-MAP-115	InterPro	0.0299	24.4	0.124	0.904	1	4	MAP7D2
Domain	IPR006614	Ferlin/Peroxisome_membrane	InterPro	0.0299	24.4	0.124	0.904	1	4	DYSF
Domain	IPR013637	PLU-1	InterPro	0.0299	24.4	0.124	0.904	1	4	KDM5A
Domain	PS51407	LAMP_3	PROSITE	0.0299	24.4	0.124	0.904	1	4	CD68
Domain	PF02760	HIN	Pfam	0.0299	24.4	0.124	0.904	1	4	IFI16
Domain	IPR011996	K_chnl_Ca-activ_SK_con	InterPro	0.0299	24.4	0.124	0.904	1	4	KCNN4
Domain	PF02928	zf-C5HC2	Pfam	0.0373	30.3	0.129	0.936	1	5	KDM5A
Domain	PF00408	PGM_PMM_IV	Pfam	0.0373	30.3	0.129	0.936	1	5	PGM5
Domain	PF10522	RII_binding_1	Pfam	0.0373	30.3	0.129	0.936	1	5	AKAP12
Domain	IPR003915	PKD_2	InterPro	0.0373	30.3	0.129	0.936	1	5	PKD2L1
Domain	PF01299	Lamp	Pfam	0.0373	30.3	0.129	0.936	1	5	CD68
Domain	IPR005946	PRib_PPkinase	InterPro	0.0373	30.3	0.129	0.936	1	5	PRPS2
Domain	PS51338	IMD	PROSITE	0.0373	30.3	0.129	0.936	1	5	BAIAP2L1
Domain	IPR018459	RII_binding_1	InterPro	0.0373	30.3	0.129	0.936	1	5	AKAP12
Domain	G3DSA:3.40.120.10	A-D-PHexomutase_a/b/a-I/II/III	Gene3D	0.0373	30.3	0.129	0.936	1	5	PGM5
Domain	IPR012561	Ferlin_B-domain	InterPro	0.0373	30.3	0.129	0.936	1	5	DYSF
Domain	IPR016055	A-D-PHexomutase_a/b/a-I/II/III	InterPro	0.0373	30.3	0.129	0.936	1	5	PGM5
Domain	PF03489	SapB_2	Pfam	0.0373	30.3	0.129	0.936	1	5	PSAP
Domain	PF02879	PGM_PMM_II	Pfam	0.0373	30.3	0.129	0.936	1	5	PGM5
Domain	IPR005845	A-D-PHexomutase_a/b/a-II	InterPro	0.0373	30.3	0.129	0.936	1	5	PGM5
Domain	IPR002337	Haemoglobin_b	InterPro	0.0373	30.3	0.129	0.936	1	5	HBB
Domain	PF02878	PGM_PMM_I	Pfam	0.0373	30.3	0.129	0.936	1	5	PGM5
Domain	IPR005844	A-D-PHexomutase_a/b/a-I	InterPro	0.0373	30.3	0.129	0.936	1	5	PGM5
Domain	IPR008138	SapB_2	InterPro	0.0373	30.3	0.129	0.936	1	5	PSAP
Domain	IPR004198	Znf_C5HC2	InterPro	0.0373	30.3	0.129	0.936	1	5	KDM5A
Domain	PF08150	FerB	Pfam	0.0373	30.3	0.129	0.936	1	5	DYSF
Domain	IPR005841	Alpha-D-phosphohexomutase	InterPro	0.0373	30.3	0.129	0.936	1	5	PGM5
Domain	PS00710	PGM_PMM	PROSITE	0.0373	30.3	0.129	0.936	1	5	PGM5
Domain	IPR002000	Lysosome-assoc_membr_glycop	InterPro	0.0373	30.3	0.129	0.936	1	5	CD68
Domain	IPR001239	Prot_inh_Kazal-m	InterPro	0.0373	30.3	0.129	0.936	1	5	SPINK1
Domain	IPR012968	FerIin-domain	InterPro	0.0373	30.3	0.129	0.936	1	5	DYSF
Domain	IPR005843	A-D-PHexomutase_C	InterPro	0.0373	30.3	0.129	0.936	1	5	PGM5
Domain	PF08151	FerI	Pfam	0.0373	30.3	0.129	0.936	1	5	DYSF
Domain	SM00775	LNS2	SMART	0.0445	36.3	0.137	0.996	1	6	LPIN3
Domain	SM00537	DCX	SMART	0.0445	36.3	0.137	0.996	1	6	DCLK1
Domain	IPR013209	LNS2	InterPro	0.0445	36.3	0.137	0.996	1	6	LPIN3
Domain	PF08235	LNS2	Pfam	0.0445	36.3	0.137	0.996	1	6	LPIN3
Domain	SM00741	SapB	SMART	0.0445	36.3	0.137	0.996	1	6	PSAP
Domain	IPR001365	A/AMP_deaminase	InterPro	0.0445	36.3	0.137	0.996	1	6	ADA
Domain	IPR015750	Ser/Thr_kinase_Pak-rel	InterPro	0.0445	36.3	0.137	0.996	1	6	PAK6
Domain	PF01849	NAC	Pfam	0.0445	36.3	0.137	0.996	1	6	BTF3P11
Domain	PF01821	ANATO	Pfam	0.0445	36.3	0.137	0.996	1	6	FBLN2
Domain	PF09457	RBD-FIP	Pfam	0.0445	36.3	0.137	0.996	1	6	RAB11FIP1
Domain	PS00435	PEROXIDASE_1	PROSITE	0.0445	36.3	0.137	0.996	1	6	PXDN
Domain	IPR019018	Rab11-bd_FIP_dom_C	InterPro	0.0445	36.3	0.137	0.996	1	6	RAB11FIP1
Domain	PF08397	IMD	Pfam	0.0445	36.3	0.137	0.996	1	6	BAIAP2L1
Domain	PS00436	PEROXIDASE_2	PROSITE	0.0445	36.3	0.137	0.996	1	6	PXDN
Domain	PS51151	NAC_AB	PROSITE	0.0445	36.3	0.137	0.996	1	6	BTF3P11
Domain	PS01178	ANAPHYLATOXIN_2	PROSITE	0.0445	36.3	0.137	0.996	1	6	FBLN2
Domain	IPR003990	Pancreatis_ac	InterPro	0.0445	36.3	0.137	0.996	1	6	CLEC3B
Domain	PS01177	ANAPHYLATOXIN_1	PROSITE	0.0445	36.3	0.137	0.996	1	6	FBLN2
Domain	IPR000020	Anaphylatoxin/fibulin	InterPro	0.0445	36.3	0.137	0.996	1	6	FBLN2
Domain	PF08953	DUF1899	Pfam	0.0445	36.3	0.137	0.996	1	6	CORO6
Domain	PS00599	AA_TRANSFER_CLASS_2	PROSITE	0.0445	36.3	0.137	0.996	1	6	ALAS2
Domain	PF00962	A_deaminase	Pfam	0.0445	36.3	0.137	0.996	1	6	ADA
Domain	G3DSA:1.10.225.10	Saposin_like	Gene3D	0.0445	36.3	0.137	0.996	1	6	PSAP
Domain	SM00104	ANATO	SMART	0.0445	36.3	0.137	0.996	1	6	FBLN2
Domain	PS00485	A_DEAMINASE	PROSITE	0.0445	36.3	0.137	0.996	1	6	ADA
Domain	IPR002715	Nas_poly-pep-assoc_cplx	InterPro	0.0445	36.3	0.137	0.996	1	6	BTF3P11
Domain	IPR000920	Myelin_P0	InterPro	0.0445	36.3	0.137	0.996	1	6	MPZL3
Domain	IPR015048	DUF1899	InterPro	0.0445	36.3	0.137	0.996	1	6	CORO6
Domain	IPR001917	Aminotrans_II_pyridoxalP_BS	InterPro	0.0445	36.3	0.137	0.996	1	6	ALAS2
Pathway	576262	Extracellular matrix organization	BioSystems: REACTOME	0.0132	7.54	0.228	1	6	264	KLK7,BMP4,MFAP4,FBLN2,MMP7,CTSK
Pathway	105698	Biological oxidations	BioSystems: REACTOME	0.00649	3.72	0.228	1	5	160	CYP11A1,ACSS1,PAPSS2,GGT1,GSS
Pathway	P00031	Inflammation mediated by chemokine and cytokine signaling pathway	PantherDB	0.0133	7.64	0.228	1	5	191	MYH11,ACTA1,PAK6,CX3CL1,PREX1
Pathway	99052	Lysosome	BioSystems: KEGG	0.0125	7.17	0.228	1	4	122	NAPSA,PSAP,CD68,CTSK
Pathway	M19553	Hemoglobin's Chaperone	MSigDB C2: BioCarta	0.000105	0.0601	0.0601	0.417	3	13	ALAS2,HBA2,HBB

Category	ID	Name	Source	p-value	q-value Bonferroni	q-value FDR B&H	q-value FDR B&Y	Hit Count in Query List	Hit Count in Genome	Hit in Query List
Pathway	730311	Molecules associated with elastic fibres	BioSystems: REACTOME	0.00136	0.78	0.18	1	3	30	BMP4,MFAP4,FBLN2
Pathway	730310	Elastic fibre formation	BioSystems: REACTOME	0.00337	1.93	0.193	1	3	41	BMP4,MFAP4,FBLN2
Pathway	82986	Glycerolipid metabolism	BioSystems: KEGG	0.00771	4.42	0.228	1	3	55	LPIN3,LIPG,AKR1B10
Pathway	138019	p75(NTR)-mediated signaling	BioSystems: Pathway Interaction Database	0.0102	5.87	0.228	1	3	61	RTN4R,NGFR,MMP7
Pathway	771599	Binding and Uptake of Ligands by Scavenger Receptors	BioSystems: REACTOME	0.0102	5.87	0.228	1	3	61	MASP1,HBA2,HBB
Pathway	698754	AGE/RAGE pathway	BioSystems: WikiPathways	0.0127	7.27	0.228	1	3	66	INHBB,FOXO4,MMP7
Pathway	P00016	Cytoskeletal regulation by Rho GTPase	PantherDB	0.0143	8.2	0.228	1	3	69	MYH11,ACTA1,PAK6
Pathway	M3270	Integrin Signaling Pathway	MSigDB C2: Signaling Transduction KE	0.0226	12.9	0.25	1	3	82	PAK6,ANGPTL2,ARHGEF6
Pathway	198825	Selenium Pathway	BioSystems: WikiPathways	0.0271	15.5	0.254	1	3	88	GGT1,HBA2,HBB
Pathway	576263	Degradation of the extracellular matrix	BioSystems: REACTOME	0.0279	16	0.254	1	3	89	KLK7,MMP7,CTSK
Pathway	105721	Phase II conjugation	BioSystems: REACTOME	0.0287	16.5	0.254	1	3	90	PAPSS2,GGT1,GSS
Pathway	645290	MHC class II antigen presentation	BioSystems: REACTOME	0.0404	23.2	0.283	1	3	103	HLA-DQB1,CD74,CTSK
Pathway	PW:0000165	taurine and hypotaurine metabolic	Pathway Ontology	0.0011	0.632	0.18	1	2	7	CDO1,GGT1
Pathway	645348	Uptake of Oxygen and Release of Carbon Dioxide by Erythrocytes	BioSystems: REACTOME	0.00233	1.34	0.18	1	2	10	HBA2,HBB
Pathway	645346	O2/CO2 exchange in erythrocytes	BioSystems: REACTOME	0.00233	1.34	0.18	1	2	10	HBA2,HBB
Pathway	645347	Uptake of Carbon Dioxide and Release of Oxygen by Erythrocytes	BioSystems: REACTOME	0.00233	1.34	0.18	1	2	10	HBA2,HBB
Pathway	82967	Taurine and hypotaurine metabolism	BioSystems: KEGG	0.00233	1.34	0.18	1	2	10	CDO1,GGT1
Pathway	PW:0000134	glutathione metabolic	Pathway Ontology	0.00283	1.62	0.18	1	2	11	GGT1,GSS
Pathway	105728	Glutathione synthesis and recycling	BioSystems: REACTOME	0.00283	1.62	0.18	1	2	11	GGT1,GSS
Pathway	MAP00480_Glutathione_metabolism	MAP00480 Glutathione metabolism	GenMAPP	0.0113	6.47	0.228	1	2	22	GGT1,GSS
Pathway	198824	Glutathione metabolism	BioSystems: WikiPathways	0.0123	7.05	0.228	1	2	23	GGT1,GSS
Pathway	161025	Smooth Muscle Contraction	BioSystems: REACTOME	0.0134	7.66	0.228	1	2	24	MYH11,LMOD1
Pathway	105727	Glutathione conjugation	BioSystems: REACTOME	0.0231	13.3	0.25	1	2	32	GGT1,GSS
Pathway	576264	Activation of Matrix Metalloproteinases	BioSystems: REACTOME	0.0231	13.3	0.25	1	2	32	MMP7,CTSK
Pathway	771600	Scavenging of Heme from Plasma	BioSystems: REACTOME	0.0245	14	0.254	1	2	33	HBA2,HBB
Pathway	194384	African trypanosomiasis	BioSystems: KEGG	0.0259	14.9	0.254	1	2	34	HBA2,HBB
Pathway	M16801	Genes related to regulation of the actin cytoskeleton	MSigDB C2: Signaling Gateway	0.0274	15.7	0.254	1	2	35	PAK6,ANGPTL2
Pathway	M6220	Agrin in Postsynaptic Differentiation	MSigDB C2: BioCarta	0.0288	16.5	0.254	1	2	36	ACTA1,PAK6
Pathway	M17400	ALK in cardiac myocytes	MSigDB C2: BioCarta	0.0303	17.4	0.259	1	2	37	NPPA,BMP4
Pathway	730309	Collagen degradation	BioSystems: REACTOME	0.0319	18.3	0.265	1	2	38	MMP7,CTSK
Pathway	82949	Glycine, serine and threonine metabolism	BioSystems: KEGG	0.0319	18.3	0.265	1	2	38	CHDH,ALAS2
Pathway	137972	Signaling events mediated by HDAC Class III	BioSystems: Pathway Interaction Database	0.0334	19.2	0.27	1	2	39	ACSS1,FOXO4
Pathway	169346	Regulation of RAC1 activity	BioSystems: Pathway Interaction Database	0.0334	19.2	0.27	1	2	39	PREX1,ARHGEF6
Pathway	82998	Pyruvate metabolism	BioSystems: KEGG	0.0366	21	0.28	1	2	41	ACSS1,AKR1B10
Pathway	138022	Class I PI3K signaling events	BioSystems: Pathway Interaction Database	0.0366	21	0.28	1	2	41	PREX1,ARHGEF6
Pathway	PW:0000031	purine metabolic	Pathway Ontology	0.04	22.9	0.283	1	2	43	PRPS2,ADA
Pathway	106444	NRAGE signals death through JNK	BioSystems: REACTOME	0.0434	24.9	0.283	1	2	45	NGFR,ARHGEF6
Pathway	M5193	Genes related to chemotaxis	MSigDB C2: Signaling Gateway	0.0434	24.9	0.283	1	2	45	PAK6,ANGPTL2
Pathway	83095	Type I diabetes mellitus	BioSystems: KEGG	0.047	26.9	0.296	1	2	47	HLA-DQB1,CPE
Pathway	138036	FoxO family signaling	BioSystems: Pathway Interaction Database	0.047	26.9	0.296	1	2	47	FOXO4,FBXO32
Pathway	142299	choline degradation I	BioSystems: BIOCYC	0.00744	4.26	0.228	1	1	1	CHDH
Pathway	SMP00143	5-Oxoprolinuria	SMPDB	0.00744	4.26	0.228	1	1	1	GSS
Pathway	SMP00371	Congenital Lipoid Adrenal Hyperplasia (CLAH) or Lipoid CAH	SMPDB	0.00744	4.26	0.228	1	1	1	CYP11A1
Pathway	SMP00337	Glutathione Synthetase Deficiency	SMPDB	0.00744	4.26	0.228	1	1	1	GSS
Pathway	SMP00144	Adenosine Deaminase Deficiency	SMPDB	0.00744	4.26	0.228	1	1	1	ADA
Pathway	782402	purine deoxyribonucleosides degradation	BioSystems: BIOCYC	0.00744	4.26	0.228	1	1	1	ADA
Pathway	142289	PRPP biosynthesis	BioSystems: BIOCYC	0.0147	8.43	0.228	1	1	2	PRPS2
Pathway	P02770	Pyridoxal phosphate salvage pathway	PantherDB	0.0147	8.43	0.228	1	1	2	PDXK
Pathway	142346	L-cysteine degradation I	BioSystems: BIOCYC	0.0147	8.43	0.228	1	1	2	CDO1
Pathway	142427	sulfate activation for sulfonation	BioSystems: BIOCYC	0.0147	8.43	0.228	1	1	2	PAPSS2
Pathway	P02778	Sulfate assimilation	PantherDB	0.0147	8.43	0.228	1	1	2	PAPSS2

Category	ID	Name	Source	p-value	q-value Bonferroni	q-value FDR B&H	q-value FDR B&Y	Hit Count in Query List	Hit Count in Genome	Hit in Query List
Pathway	142237	pyridoxal 5'-phosphate salvage pathway	BioSystems: BIOCYC	0.0147	8.43	0.228	1	1	2	PDXK
Pathway	142343	asparagine degradation I	BioSystems: BIOCYC	0.0147	8.43	0.228	1	1	2	ASPG
Pathway	545268	taurine biosynthesis	BioSystems: BIOCYC	0.0147	8.43	0.228	1	1	2	CDO1
Pathway	142432	purine ribonucleosides degradation to ribose-1-phosphate	BioSystems: BIOCYC	0.0147	8.43	0.228	1	1	2	ADA
Pathway	P02787	Vitamin B6 metabolism	PantherDB	0.022	12.6	0.25	1	1	3	PDXK
Pathway	413344	PRPP biosynthesis, ribose 5P => PRPP	BioSystems: KEGG	0.022	12.6	0.25	1	1	3	PRPS2
Pathway	PW:0000471	atrial natriuretic peptide signaling	Pathway Ontology	0.022	12.6	0.25	1	1	3	NPPA
Pathway	413393	Glutathione biosynthesis, glutamate => glutathione	BioSystems: KEGG	0.022	12.6	0.25	1	1	3	GSS
Pathway	547495	adenine and adenosine salvage III	BioSystems: BIOCYC	0.022	12.6	0.25	1	1	3	ADA
Pathway	106221	5-Phosphoribose 1-diphosphate biosynthesis	BioSystems: REACTOME	0.022	12.6	0.25	1	1	3	PRPS2
Pathway	413396	Steroid hormone biosynthesis, cholesterol => prognenolone => progesterone	BioSystems: KEGG	0.022	12.6	0.25	1	1	3	CYP11A1
Pathway	142314	methylglyoxal degradation III	BioSystems: BIOCYC	0.022	12.6	0.25	1	1	3	AKR1B10
Pathway	187206	Vitamins B6 activation to pyridoxal phosphate	BioSystems: REACTOME	0.022	12.6	0.25	1	1	3	PDXK
Pathway	106442	NFG and proNGF binds to p75NTR	BioSystems: REACTOME	0.022	12.6	0.25	1	1	3	NGFR
Pathway	P02722	Acetate utilization	PantherDB	0.022	12.6	0.25	1	1	3	ACSS1
Pathway	106451	Ceramide signalling	BioSystems: REACTOME	0.022	12.6	0.25	1	1	3	NGFR
Pathway	PW:0000078	cyanoamino acid metabolic	Pathway Ontology	0.022	12.6	0.25	1	1	3	GGT1
Pathway	106453	Axonal growth stimulation	BioSystems: REACTOME	0.0292	16.7	0.254	1	1	4	NGFR
Pathway	142231	tetrapyrrole biosynthesis	BioSystems: BIOCYC	0.0292	16.7	0.254	1	1	4	ALAS2
Pathway	MAP00430_Taurine_and_hypotaurine_metabolism	MAP00430 Taurine and hypotaurine metabolism	GenMAPP	0.0292	16.7	0.254	1	1	4	GGT1
Pathway	SMP00017	Vitamin B6 Metabolism	SMPDB	0.0292	16.7	0.254	1	1	4	PDXK
Pathway	730350	Antagonism of Activin by Follistatin	BioSystems: REACTOME	0.0292	16.7	0.254	1	1	4	INHBB
Pathway	MAP00460_Cyanoamino_acid_metabolism	MAP00460 Cyanoamino acid metabolism	GenMAPP	0.0292	16.7	0.254	1	1	4	GGT1
Pathway	SMP00021	Taurine and Hypotaurine Metabolism	SMPDB	0.0364	20.8	0.28	1	1	5	CDO1
Pathway	833818	Ficolins bind to repetitive carbohydrate structures on the target cell surface	BioSystems: REACTOME	0.0364	20.8	0.28	1	1	5	MASP1
Pathway	PW:0000480	protein C anticoagulant	Pathway Ontology	0.0435	24.9	0.283	1	1	6	PROCR
Pathway	83013	Vitamin B6 metabolism	BioSystems: KEGG	0.0435	24.9	0.283	1	1	6	PDXK
Pathway	MAP00450_Selenoamino_acid_metabolism	MAP00450 Selenoamino acid metabolism	GenMAPP	0.0435	24.9	0.283	1	1	6	GGT1
Pathway	106151	Pregnenolone biosynthesis	BioSystems: REACTOME	0.0435	24.9	0.283	1	1	6	CYP11A1
Pathway	187219	ER Quality Control Compartment (ERQC)	BioSystems: REACTOME	0.0435	24.9	0.283	1	1	6	EDEM2
Pathway	106447	p75NTR negatively regulates cell cycle via SC1	BioSystems: REACTOME	0.0435	24.9	0.283	1	1	6	NGFR
Pathway	SMP00041	Sulfate/Sulfite Metabolism	SMPDB	0.0435	24.9	0.283	1	1	6	PAPSS2
Pathway	161010	Transport and synthesis of PAPS	BioSystems: REACTOME	0.0435	24.9	0.283	1	1	6	PAPSS2
Pathway	106408	Lectin pathway of complement activation	BioSystems: REACTOME	0.0435	24.9	0.283	1	1	6	MASP1
Pubmed	23376485	Proteomic analysis of podocyte exosome-enriched fraction from normal human urine.	Pubmed	1.17E-14	1.73E-10	1.73E-10	1.76E-09	35	1715	DPP7,PRPS2,NAPSA,DPT,CDSN,TRABD2B,HTRA1,PSAP,ERV3-1,PROCR,APMAP,MRGPRF,KLK14,CPE,CPZ,CALML5,GGT1,PDXK,GSS,CRYAA,ORM1,MFAP4,FBLN2,ANGPTL2,AKR1B10,SLPI,CLEC3B,CAPN5,MMP7,HBA2,HBB,TACSTD2,KLK1,METTL7A,CD276
Pubmed	23533145	In-depth proteomic analyses of exosomes isolated from expressed prostatic secretions in urine.	Pubmed	3.44E-11	0.000000509	0.000000255	0.00000259	24	1053	DPP7,CPNE2,NAPSA,MYH11,PSAP,SPINK1,PROCR,RTN4R,ACTA1,CPE,DYSF,GGT1,GSS,ORM1,TGM2,ORM2,ANGPTL2,PXDN,BAIAP2L1,CLEC3B,CAPN5,MMP7,HBA2,HBB
Pubmed	16800724	A comprehensive nomenclature for serine proteases with homology to tissue kallikreins.	Pubmed	2.92E-09	0.0000431	0.0000144	0.000146	5	14	KLK15,KLK7,KLK10,KLK14,KLK1
Pubmed	16800723	Proceedings of the 1st International Symposium on Kallikreins, Lausanne, Switzerland, September 1-3, 2005.	Pubmed	4.36E-09	0.0000645	0.0000161	0.000164	5	15	KLK15,KLK7,KLK10,KLK14,KLK1
Pubmed	20424135	Blood biomarker levels to aid discovery of cancer-related single-nucleotide polymorphisms: kallikreins and prostate cancer.	GeneRIF	6.32E-09	0.0000934	0.0000187	0.00019	5	16	KLK15,KLK7,KLK10,KLK14,KLK1
Pubmed	19558318	Association between kallikrein-related peptidases (KLKs) and macroscopic indicators of semen analysis: their relation to sperm motility.	GeneRIF	7.27E-08	0.00108	0.000179	0.00182	4	10	KLK7,KLK10,KLK14,KLK1
Pubmed	15192120	Human tissue kallikreins: physiologic roles and applications in cancer.	Pubmed	0.00000017	0.00252	0.00036	0.00366	4	12	KLK15,KLK7,KLK10,KLK14
Pubmed	20551380	Proteomics characterization of extracellular space components in the human aorta.	Pubmed	0.000000325	0.00481	0.000505	0.00515	7	101	DPT,HTRA1,PSAP,MFAP4,PRELP,SLPI,CLEC3B
Pubmed	11054574	Sequencing and expression analysis of the serine protease gene cluster located in chromosome 19q13 region.	Pubmed	0.000000342	0.00505	0.000505	0.00515	4	14	KLK15,KLK7,KLK10,KLK1
Pubmed	15203212	Taxon-specific evolution of glandular kallikrein genes and identification of a progenitor of prostate-specific antigen.	Pubmed	0.000000342	0.00505	0.000505	0.00515	4	14	KLK15,KLK7,KLK10,KLK14
Pubmed	11780052	The DNA sequence and comparative analysis of human chromosome 20.	Pubmed	0.000000485	0.00717	0.000652	0.00663	13	516	PROCR,APMAP,ACSS1,JPH2,FAM65C,ADA,GSS,LPIN3,TGM2,EDEM2,SLPI,PPP1R16B,PREX1
Pubmed	12975309	The secreted protein discovery initiative (SPDI), a large-scale effort to identify novel human secreted and transmembrane proteins: a bioinformatics assessment.	Pubmed	0.00000208	0.0307	0.00256	0.0261	17	1005	APMAP,RTN4R,GDPD2,SPOCK2,AMICA1,ANGPTL2,LRRN4CL,MPZL3,LIPG,EDEM2,TSPAN13,ENPP5,GPNMB,STRA6,METTL7A,SEZ6L,CD276
Pubmed	12437987	Organization and evolution of the glandular kallikrein locus in Mus musculus.	Pubmed	0.00000454	0.0672	0.00515	0.0524	3	8	KLK15,KLK10,KLK14

Category	ID	Name	Source	p-value	q-value Bonferroni	q-value FDR B&H	q-value FDR B&Y	Hit Count in Query List	Hit Count in Genome	Hit in Query List
Pubmed	22261194	Proteomics analysis of cardiac extracellular matrix remodeling in a porcine model of ischemia/reperfusion injury.	Pubmed	0.00000931	0.138	0.00515	0.0524	5	64	DPT,PSAP,FBLN2,PRELP,CLEC3B
Pubmed	20301552	Anophthalmia/Microphthalmia Overview	Pubmed	0.0000148	0.219	0.00515	0.0524	4	34	PORCN,BMP4,PXDN,STRA6
Pubmed	19018521	Investigation of genetic variants of alpha-1 acid glycoprotein by ultra-performance liquid chromatography-mass spectrometry.	GeneRIF	0.0000192	0.283	0.00515	0.0524	2	2	ORM1,ORM2
Pubmed	16290938	Selective binding of coumarin enantiomers to human alpha1-acid glycoprotein genetic variants.	GeneRIF	0.0000192	0.283	0.00515	0.0524	2	2	ORM1,ORM2
Pubmed	8548834	Antiviral immune responses of mice lacking MHC class II or its associated invariant chain.	Pubmed	0.0000192	0.283	0.00515	0.0524	2	2	HLA-DQB1,CD74
Pubmed	14556985	Immunity to blood-stage murine malarial parasites is MHC class II dependent.	Pubmed	0.0000192	0.283	0.00515	0.0524	2	2	HLA-DQB1,CD74
Pubmed	15953414	MAG induces regulated intramembrane proteolysis of the p75 neurotrophin receptor to inhibit neurite outgrowth.	Pubmed	0.0000192	0.283	0.00515	0.0524	2	2	RTN4R,NGFR
Pubmed	17987628	Comparison of alpha-1-acid glycoprotein isoforms from healthy and cancer patients by capillary IEF.	GeneRIF	0.0000192	0.283	0.00515	0.0524	2	2	ORM1,ORM2
Pubmed	19479992	Neurons express hemoglobin alpha- and beta-chains in rat and human brains.	GeneRIF	0.0000192	0.283	0.00515	0.0524	2	2	HBA2,HBB
Pubmed	12480518	Structure of human alpha1-acid glycoprotein and its high-affinity binding site.	GeneRIF	0.0000192	0.283	0.00515	0.0524	2	2	ORM1,ORM2
Pubmed	11867743	Invariant chain induces B cell maturation in a process that is independent of its chaperonic activity.	Pubmed	0.0000192	0.283	0.00515	0.0524	2	2	HLA-DQB1,CD74
Pubmed	6726807	The crystal structure of human deoxyhaemoglobin at 1.74 A resolution.	Pubmed	0.0000192	0.283	0.00515	0.0524	2	2	HBA2,HBB
Pubmed	8980683	The crystal structure of horse deoxyhaemoglobin trapped in the high-affinity (R) state.	Pubmed	0.0000192	0.283	0.00515	0.0524	2	2	HBA2,HBB
Pubmed	2970990	Structure and characterisation of a duplicated human alpha 1 acid glycoprotein gene.	Pubmed	0.0000192	0.283	0.00515	0.0524	2	2	ORM1,ORM2
Pubmed	25408356	Structural heterogeneity of glycoform of alpha-1 Acid glycoprotein in alcoholic cirrhosis patients.	Pubmed	0.0000192	0.283	0.00515	0.0524	2	2	ORM1,ORM2
Pubmed	24389491	Distal promoter regions are responsible for differential regulation of human orosomucoid-1 and -2 gene expression and acute phase responses.	Pubmed	0.0000192	0.283	0.00515	0.0524	2	2	ORM1,ORM2
Pubmed	15647357	Genetic deletion of the Nogo receptor does not reduce neurite inhibition in vitro or promote corticospinal tract regeneration in vivo.	Pubmed	0.0000192	0.283	0.00515	0.0524	2	2	RTN4R,NGFR
Pubmed	20524398	Distinct DNA methylation profiles between adenocarcinoma and squamous cell carcinoma of human uterine cervix.	GeneRIF	0.0000192	0.283	0.00515	0.0524	2	2	RTN4R,PAK6
Pubmed	19342211	Antigen presentation in celiac disease.	GeneRIF	0.0000192	0.283	0.00515	0.0524	2	2	HLA-DQB1,TGM2
Pubmed	2822385	Structure and expression of the genes coding for human alpha 1-acid glycoprotein.	Pubmed	0.0000192	0.283	0.00515	0.0524	2	2	ORM1,ORM2
Pubmed	8765017	Lack of oral tolerance but oral priming for contact sensitivity to dinitrofluorobenzene in major histocompatibility complex class II-deficient mice and in CD4+ T cell-depleted mice.	Pubmed	0.0000192	0.283	0.00515	0.0524	2	2	HLA-DQB1,CD74
Pubmed	15253437	A new strategy for identification of N-glycosylated proteins and unambiguous assignment of their glycosylation sites using HILIC enrichment and partial deglycosylation.	Pubmed	0.0000192	0.283	0.00515	0.0524	2	2	ORM1,ORM2
Pubmed	4603214	The disulfide bonds of alpha1-acid glycoprotein.	Pubmed	0.0000192	0.283	0.00515	0.0524	2	2	ORM1,ORM2
Pubmed	7795416	alpha 1-Acid glycoprotein binds human immunodeficiency virus type 1 (HIV-1) envelope glycoprotein via N-linked glycans.	Pubmed	0.0000192	0.283	0.00515	0.0524	2	2	ORM1,ORM2
Pubmed	16418531	Coregulated human globin genes are frequently in spatial proximity when active.	GeneRIF	0.0000192	0.283	0.00515	0.0524	2	2	HBA2,HBB
Pubmed	18976018	Expression of the serine protease kallikrein 7 and its inhibitor antileukoprotease is decreased in prostate cancer.	GeneRIF	0.0000192	0.283	0.00515	0.0524	2	2	KLK7,SLPI
Pubmed	23224034	Parallel overexpression and clinical significance of kallikrein-related peptidases 7 and 14 (KLK7KLK14) in colon cancer.	Pubmed	0.0000192	0.283	0.00515	0.0524	2	2	KLK7,KLK14
Pubmed	8598039	The repertoire of T cells shaped by a single MHC/peptide ligand.	Pubmed	0.0000192	0.283	0.00515	0.0524	2	2	HLA-DQB1,CD74
Pubmed	4711474	Structure of 1 -acid glycoprotein. The complete amino acid sequence, multiple amino acid substitutions, and homology with the immunoglobulins.	Pubmed	0.0000192	0.283	0.00515	0.0524	2	2	ORM1,ORM2
Pubmed	12122004	Crystal structure of horse carbonmonoxyhemoglobin-bezafibrate complex at 1.55-A resolution. A novel allosteric binding site in R-state hemoglobin.	Pubmed	0.0000192	0.283	0.00515	0.0524	2	2	HBA2,HBB
Pubmed	12422217	P75 interacts with the Nogo receptor as a co-receptor for Nogo, MAG and OMgp.	GeneRIF	0.0000192	0.283	0.00515	0.0524	2	2	RTN4R,NGFR
Pubmed	18026953	Coinheritance of the different copy numbers of alpha-globin gene modifies severity of beta-thalassemia/Hb E disease.	GeneRIF	0.0000192	0.283	0.00515	0.0524	2	2	HBA2,HBB
Pubmed	21471216	Activation of bone morphogenetic protein 4 signaling leads to glomerulosclerosis that mimics diabetic nephropathy.	GeneRIF	0.0000192	0.283	0.00515	0.0524	2	2	BMP4,KCNH2
Pubmed	19846611	The spontaneous Ala147Thr amino acid substitution within the translocator protein influences pregnenolone production in lymphomonocytes of healthy individuals.	GeneRIF	0.0000192	0.283	0.00515	0.0524	2	2	CYP11A1,TSPO
Pubmed	19245215	Subunit-selective interrogation of CO recombination in carbonmonoxy hemoglobin by isotope-edited time-resolved resonance Raman spectroscopy.	GeneRIF	0.0000192	0.283	0.00515	0.0524	2	2	HBA2,HBB
Pubmed	20617306	Isoform differentiation of intact AGP from human serum by capillary electrophoresis-mass spectrometry.	GeneRIF	0.0000192	0.283	0.00515	0.0524	2	2	ORM1,ORM2
Pubmed	17920577	alpha/beta-Globin mRNA ratio determination by multiplex quantitative real-time reverse transcription-polymerase chain reaction as an indicator of globin gene function.	GeneRIF	0.0000192	0.283	0.00515	0.0524	2	2	HBA2,HBB
Pubmed	14972646	Altered kallikrein 7 and 10 concentrations in cerebrospinal fluid of patients with Alzheimer's disease and frontotemporal dementia.	GeneRIF	0.0000192	0.283	0.00515	0.0524	2	2	KLK7,KLK10
Pubmed	1567356	Analysis of the five glycosylation sites of human alpha 1-acid glycoprotein.	Pubmed	0.0000192	0.283	0.00515	0.0524	2	2	ORM1,ORM2

Category	ID	Name	Source	p-value	q-value Bonferroni	q-value FDR B&H	q-value FDR B&Y	Hit Count in Query List	Hit Count in Genome	Hit in Query List
Pubmed	18182498	Nogo receptor antagonizes p75NTR-dependent motor neuron death.	GeneRIF	0.0000192	0.283	0.00515	0.0524	2	2	RTN4R,NGFR
Pubmed	15492784	Expression of human kallikrein 7 (hK7/SCCE) and its inhibitor antileukoprotease (ALP/SLPI) in uterine endocervical glands and in cervical adenocarcinomas.	GeneRIF	0.0000192	0.283	0.00515	0.0524	2	2	KLK7,SLPI
Pubmed	17924658	Kinetic characterization and identification of the acylation and glycosylation sites of recombinant human gamma-glutamyltranspeptidase.	GeneRIF	0.0000192	0.283	0.00515	0.0524	2	2	GGT1,TGM2
Pubmed	22443383	Genome wide association study for plasma levels of natural anticoagulant inhibitors and protein C anticoagulant pathway: the MARTHA project.	GeneRIF	0.0000228	0.337	0.00603	0.0613	3	13	PROCR,GSS,EDEM2
Pubmed	16332269	Mouse models of ocular diseases.	Pubmed	0.0000232	0.344	0.00603	0.0613	4	38	GGT1,CRYAA,BMP4,GPNMB
Pubmed	19724895	Association of gene polymorphisms with chronic kidney disease in Japanese individuals.	GeneRIF	0.0000258	0.381	0.00658	0.067	4	39	ACSS1,AKAP12,ERGIC1,PXDN
Pubmed	15331780	Large-scale sequencing of the CD33-related Siglec gene cluster in five mammalian species reveals rapid evolution by multiple mechanisms.	Pubmed	0.0000289	0.428	0.00726	0.0739	3	14	KLK7,KLK10,KLK14
Pubmed	11336643	Human alpha1-acid glycoprotein binds to CCR5 expressed on the plasma membrane of human primary macrophages.	Pubmed	0.000057	0.844	0.00804	0.0818	2	3	ORM1,ORM2
Pubmed	15835899	Crystallographic evidence for a new ensemble of ligand-induced allosteric transitions in hemoglobin: the T-to-T(high) quaternary transitions.	Pubmed	0.000057	0.844	0.00804	0.0818	2	3	HBA2,HBB
Pubmed	14966521	LINGO-1 is a component of the Nogo-66 receptor/p75 signaling complex.	Pubmed	0.000057	0.844	0.00804	0.0818	2	3	RTN4R,NGFR
Pubmed	11201742	Identification of a receptor mediating Nogo-66 inhibition of axonal regeneration.	Pubmed	0.000057	0.844	0.00804	0.0818	2	3	RTN4R,NGFR
Pubmed	11724557	Control of heme reactivity by diffusion: structural basis and functional characterization in hemoglobin mutants.	Pubmed	0.000057	0.844	0.00804	0.0818	2	3	HBA2,HBB
Pubmed	12629221	Antigen presentation by keratinocytes directs autoimmune skin disease.	Pubmed	0.000057	0.844	0.00804	0.0818	2	3	HLA-DQB1,CD74
Pubmed	7492318	Evaluation of the extent of the binding site in human tissue kallikrein by synthetic substrates with sequences of human kininogen fragments.	Pubmed	0.000057	0.844	0.00804	0.0818	2	3	KLK1,CTSK
Pubmed	18643848	Molecular elements of the regulatory control of keratin filament modulator AHF/trichohyalin in the hair follicle.	GeneRIF	0.000057	0.844	0.00804	0.0818	2	3	BMP4,TCHH
Pubmed	11087837	Diversity of T cell repertoire shaped by a single peptide ligand is critically affected by its amino acid residue at a T cell receptor contact.	Pubmed	0.000057	0.844	0.00804	0.0818	2	3	HLA-DQB1,CD74
Pubmed	17470639	Molecular dissection of the myelin-associated glycoprotein receptor complex reveals cell type-specific mechanisms for neurite outgrowth inhibition.	Pubmed	0.000057	0.844	0.00804	0.0818	2	3	RTN4R,NGFR
Pubmed	25666623	LINGO-1 protein interacts with the p75 neurotrophin receptor in intracellular membrane compartments.	Pubmed	0.000057	0.844	0.00804	0.0818	2	3	RTN4R,NGFR
Pubmed	9256469	Functionality of major histocompatibility complex class II molecules in mice doubly deficient for invariant chain and H-2M complexes.	Pubmed	0.000057	0.844	0.00804	0.0818	2	3	HLA-DQB1,CD74
Pubmed	9441940	The use of electron tomography for structural analysis of disordered protein arrays.	Pubmed	0.000057	0.844	0.00804	0.0818	2	3	HBA2,HBB
Pubmed	17660536	Complex signatures of selection and gene conversion in the duplicated globin genes of house mice.	Pubmed	0.000057	0.844	0.00804	0.0818	2	3	HBA2,HBB
Pubmed	15541368	Junctophilin type 2 is associated with caveolin-3 and is down-regulated in the hypertrophic and dilated cardiomyopathies.	Pubmed	0.000057	0.844	0.00804	0.0818	2	3	NPPA,JPH2
Pubmed	24857170	Delineation of the molecular basis of borderline hemoglobin A2 in Chinese individuals.	Pubmed	0.000057	0.844	0.00804	0.0818	2	3	HBA2,HBB
Pubmed	20844138	Oligodendrocyte-myelin glycoprotein and Nogo negatively regulate activity-dependent synaptic plasticity.	GeneRIF	0.000057	0.844	0.00804	0.0818	2	3	RTN4R,NGFR
Pubmed	20036801	Alpha-hemoglobin stabilizing protein: molecular function and clinical correlation.	GeneRIF	0.000057	0.844	0.00804	0.0818	2	3	HBA2,HBB
Pubmed	8996239	T helper subset differentiation in the absence of invariant chain.	Pubmed	0.000057	0.844	0.00804	0.0818	2	3	HLA-DQB1,CD74
Pubmed	19843387	Alpha globin gene numbers: an important modifier of HbE/beta thalassemia.	GeneRIF	0.000057	0.844	0.00804	0.0818	2	3	HBA2,HBB
Pubmed	1512262	A third quaternary structure of human hemoglobin A at 1.7-A resolution.	Pubmed	0.000057	0.844	0.00804	0.0818	2	3	HBA2,HBB
Pubmed	12417617	Immunohistochemical localization of carboxypeptidases D, E, and Z in pituitary adenomas and normal human pituitary.	Pubmed	0.000057	0.844	0.00804	0.0818	2	3	CPE,CPZ
Pubmed	12360531	Allosteric effects of chloride ions at the intradimeric alpha1beta1 and alpha2beta2 interfaces of human hemoglobin.	GeneRIF	0.000057	0.844	0.00804	0.0818	2	3	HBA2,HBB
Pubmed	9455924	Effect of mannosylated derivatives on HIV-1 infection of macrophages and lymphocytes.	Pubmed	0.000057	0.844	0.00804	0.0818	2	3	ORM1,ORM2
Pubmed	23349856	Characterization of adult a- and ß-globin elevated by hydrogen peroxide in cervical cancer cells that play a cytoprotective role against oxidative insults.	GeneRIF	0.000057	0.844	0.00804	0.0818	2	3	HBA2,HBB
Pubmed	17869133	Nonself-antigens are the cognate specificities of Foxp3+ regulatory T cells.	Pubmed	0.000057	0.844	0.00804	0.0818	2	3	HLA-DQB1,CD74
Pubmed	22183073	Relationship between maternal hemoglobin concentration and neonatal birth weight.	GeneRIF	0.000057	0.844	0.00804	0.0818	2	3	HBA2,HBB
Pubmed	19383315	Androgens act synergistically to enhance estrogen-induced upregulation of human tissue kallikreins 10, 11, and 14 in breast cancer cells via a membrane bound androgen receptor.	GeneRIF	0.000057	0.844	0.00804	0.0818	2	3	KLK10,KLK14
Pubmed	9894000	Structural and functional properties of human hemoglobins reassembled after synthesis in Escherichia coli.	Pubmed	0.000057	0.844	0.00804	0.0818	2	3	HBA2,HBB
Pubmed	10390349	Modulation of ligand binding in engineered human hemoglobin distal pocket.	Pubmed	0.000057	0.844	0.00804	0.0818	2	3	HBA2,HBB
Pubmed	20080989	IRAG determines nitric oxide- and atrial natriuretic peptide-mediated smooth muscle relaxation.	GeneRIF	0.000057	0.844	0.00804	0.0818	2	3	NPPA,MRVI1
Pubmed	10930828	Genetically crosslinked hemoglobin: a structural study.	Pubmed	0.000057	0.844	0.00804	0.0818	2	3	HBA2,HBB

Category	ID	Name	Source	p-value	q-value Bonferroni	q-value FDR B&H	q-value FDR B&Y	Hit Count in Query List	Hit Count in Genome	Hit in Query List
Pubmed	1115799	Two new hemoglobins. Hemoglobin Alabama (beta39(C5)Gln leads to Lys) and hemoglobin Montgomery (alpha 48(CD 6) Leu leads to Arg).	Pubmed	0.000057	0.844	0.00804	0.0818	2	3	HBA2,HBB
Pubmed	12855551	Innate CD4+CD25+ regulatory T cells are required for oral tolerance and inhibition of CD8+ T cells mediating skin inflammation.	Pubmed	0.000057	0.844	0.00804	0.0818	2	3	HLA-DQB1,CD74
Pubmed	6644819	Structure of human oxyhaemoglobin at 2.1 A resolution.	Pubmed	0.000057	0.844	0.00804	0.0818	2	3	HBA2,HBB
Pubmed	9276160	Characterization of kininogenase activity of an acidic proteinase isolated from human kidney.	Pubmed	0.000057	0.844	0.00804	0.0818	2	3	KLK1,CTSK
Pubmed	18480170	Neurological deficits and glycosphingolipid accumulation in saposin B deficient mice.	GeneRIF	0.000057	0.844	0.00804	0.0818	2	3	PSAP,CD68
Pubmed	9830011	Crystal structure of deoxy-human hemoglobin beta6 Glu --> Trp. Implications for the structure and formation of the sickle cell fiber.	Pubmed	0.000057	0.844	0.00804	0.0818	2	3	HBA2,HBB
Pubmed	19421758	Elevated expressions of MMP7, TROP2, and survivin are associated with survival, disease recurrence, and liver metastasis of colon cancer.	GeneRIF	0.000057	0.844	0.00804	0.0818	2	3	MMP7,TACSTD2
Pubmed	25238043	The correlation of a-globin gene mutations and the XmnI polymorphism with clinical severity of Hb E/ß-thalassemia.	Pubmed	0.000057	0.844	0.00804	0.0818	2	3	HBA2,HBB
Pubmed	21703224	T-quaternary structure of oxy human adult hemoglobin in the presence of two allosteric effectors, L35 and IHP.	GeneRIF	0.000057	0.844	0.00804	0.0818	2	3	HBA2,HBB
Pubmed	24074341	Comparative haematological parameters of HbAA and HbAS genotype children infected with Plasmodium falciparum malaria in Yemen.	Pubmed	0.000057	0.844	0.00804	0.0818	2	3	HBA2,HBB
Pubmed	11160279	Alpha beta TCRs differ in the degree of their specificity for the positively selecting MHC/peptide ligand.	Pubmed	0.000057	0.844	0.00804	0.0818	2	3	HLA-DQB1,CD74
Pubmed	16094690	In vivo control of endosomal architecture by class II-associated invariant chain and cathepsin S.	GeneRIF	0.000057	0.844	0.00804	0.0818	2	3	HLA-DQB1,CD74
Pubmed	24635037	Capturing the hemoglobin allosteric transition in a single crystal form.	Pubmed	0.000057	0.844	0.00804	0.0818	2	3	HBA2,HBB
Pubmed	1552945	A human recombinant haemoglobin designed for use as a blood substitute.	Pubmed	0.000057	0.844	0.00804	0.0818	2	3	HBA2,HBB
Pubmed	15929941	Conditional mutagenesis of the murine serum response factor gene blocks cardiogenesis and the transcription of downstream gene targets.	GeneRIF	0.000064	0.947	0.00894	0.091	3	18	NPPA,ACTA1,BMP4
Pubmed	9432982	Invariant chain-independent function of H-2M in the formation of endogenous peptide-major histocompatibility complex class II complexes in vivo.	Pubmed	0.000114	1.68	0.0114	0.116	2	4	HLA-DQB1,CD74
Pubmed	17158887	A potential role for multiple tissue kallikrein serine proteases in epidermal desquamation.	GeneRIF	0.000114	1.68	0.0114	0.116	2	4	KLK14,KLK1
Pubmed	6683087	Separation of zeta (zeta) and various gamma (gamma) chains of human embryonic hemoglobin Portland I by reverse-phase high-performance liquid chromatography.	Pubmed	0.000114	1.68	0.0114	0.116	2	4	HBA2,HBB
Pubmed	18701653	TNF induction of atrogin-1/MAFbx mRNA depends on Foxo4 expression but not AKT-Foxo1/3 signaling.	GeneRIF	0.000114	1.68	0.0114	0.116	2	4	FOXO4,FBXO32
Pubmed	15140227	Degradation of corneodesmosome proteins by two serine proteases of the kallikrein family, SCTE/KLK5/hK5 and SCCE/KLK7/hK7.	GeneRIF	0.000114	1.68	0.0114	0.116	2	4	CDSN,KLK7
Pubmed	25071214	Epigenetic coordination of embryonic heart transcription by dynamically regulated long noncoding RNAs.	Pubmed	0.000114	1.68	0.0114	0.116	2	4	NPPA,ACTA1
Pubmed	10925256	Response of murine gamma delta T cells to the synthetic polypeptide poly-Glu50Tyr50.	Pubmed	0.000114	1.68	0.0114	0.116	2	4	HLA-DQB1,CD74
Pubmed	20301608	Alpha-Thalassemia	Pubmed	0.000114	1.68	0.0114	0.116	2	4	HBA2,HBB
Pubmed	8598040	Mice lacking H2-M complexes, enigmatic elements of the MHC class II peptide-loading pathway.	Pubmed	0.000114	1.68	0.0114	0.116	2	4	HLA-DQB1,CD74
Pubmed	12492488	Kininogen-derived peptides for investigating the putative vasoactive properties of human cathepsins K and L.	GeneRIF	0.000114	1.68	0.0114	0.116	2	4	KLK1,CTSK
Pubmed	11777953	Peptide specificity of thymic selection of CD4+CD25+ T cells.	Pubmed	0.000114	1.68	0.0114	0.116	2	4	HLA-DQB1,CD74
Pubmed	7985028	Reconstitution of an operational MHC class II compartment in nonantigen-presenting cells.	Pubmed	0.000114	1.68	0.0114	0.116	2	4	HLA-DQB1,CD74
Pubmed	9551884	A critical, invariant chain-independent role for H2-M in antigen presentation.	Pubmed	0.000114	1.68	0.0114	0.116	2	4	HLA-DQB1,CD74
Pubmed	11006094	Genomic organization of the human kallikrein gene family on chromosome 19q13.3-q13.4.	Pubmed	0.000114	1.68	0.0114	0.116	2	4	KLK15,KLK1
Pubmed	12119341	The developmentally regulated expression of Twisted gastrulation reveals a role for bone morphogenetic proteins in the control of T cell development.	GeneRIF	0.000114	1.68	0.0114	0.116	2	4	HLA-DQB1,BMP4
Pubmed	12021314	Cathepsin L regulates CD4+ T cell selection independently of its effect on invariant chain: a role in the generation of positively selecting peptide ligands.	GeneRIF	0.000114	1.68	0.0114	0.116	2	4	HLA-DQB1,CD74
Pubmed	19085836	Multiple kallikrein (KLK 5, 7, 8, and 10) expression in squamous cell carcinoma of the oral cavity.	GeneRIF	0.000114	1.68	0.0114	0.116	2	4	KLK7,KLK10
Pubmed	19657833	Description of two new alpha variants: Hb Canuts [alpha85(F6)Asp-->His (alpha1)] and Hb Ambroise Pare [alpha117(GH5)Phe-->Ile (alpha2)]; two new beta variants: Hb Beaujolais [beta84(EF8)Thr-->Asn] and Hb Monplaisir [beta147 (Tyr-Lys-Leu-Ala-Phe-Phe-Leu-Leu-Ser-Asn-Phe-Tyr-158-COOH)] and one new delta variant: Hb (A2)North Africa [delta59(E3)Lys-->Met].	GeneRIF	0.000114	1.68	0.0114	0.116	2	4	HBA2,HBB
Pubmed	17519359	Gene expression responses over 24 h to lengthening and shortening contractions in human muscle: major changes in CSRP3, MUSTN1, SIX1, and FBXO32.	GeneRIF	0.000114	1.68	0.0114	0.116	2	4	MUSTN1,FBXO32
Pubmed	19740759	Peroxidase activity of hemoglobin-haptoglobin complexes: covalent aggregation and oxidative stress in plasma and macrophages.	GeneRIF	0.000114	1.68	0.0114	0.116	2	4	HBA2,HBB
Pubmed	15084671	A proteomic analysis of human bile.	Pubmed	0.000119	1.77	0.0114	0.116	3	22	GGT1,ORM1,ORM2
Pubmed	15767668	GATA6 is essential for embryonic development of the liver but dispensable for early heart formation.	GeneRIF	0.000177	2.61	0.0114	0.116	3	25	NPPA,ACTA1,BMP4

Category	ID	Name	Source	p-value	q-value Bonferroni	q-value FDR B&H	q-value FDR B&Y	Hit Count in Query List	Hit Count in Genome	Hit in Query List
Pubmed	7679955	Mice lacking the MHC class II-associated invariant chain.	Pubmed	0.000189	2.8	0.0114	0.116	2	5	HLA-DQB1,CD74
Pubmed	15694321	A TNF receptor family member, TROY, is a coreceptor with Nogo receptor in mediating the inhibitory activity of myelin inhibitors.	GeneRIF	0.000189	2.8	0.0114	0.116	2	5	RTN4R,NGFR
Pubmed	17916326	Membrane interactions of hemoglobin variants, HbA, HbE, HbF and globin subunits of HbA: effects of aminophospholipids and cholesterol.	GeneRIF	0.000189	2.8	0.0114	0.116	2	5	HBA2,HBB
Pubmed	11729324	Regulation of cell survival by secreted proneurotrophins.	Pubmed	0.000189	2.8	0.0114	0.116	2	5	NGFR,MMP7
Pubmed	14764672	Mechanisms governing B cell developmental defects in invariant chain-deficient mice.	GeneRIF	0.000189	2.8	0.0114	0.116	2	5	HLA-DQB1,CD74
Pubmed	12426574	A p75(NTR) and Nogo receptor complex mediates repulsive signaling by myelin-associated glycoprotein.	Pubmed	0.000189	2.8	0.0114	0.116	2	5	RTN4R,NGFR
Pubmed	20881014	Hydroureternephrosis due to loss of Sox9-regulated smooth muscle cell differentiation of the ureteric mesenchyme.	GeneRIF	0.000199	2.94	0.0114	0.116	3	26	DPT,MYH11,BMP4
Pubmed	9403069	Mapping of eight testis-specific genes to mouse chromosomes.	Pubmed	0.000223	3.3	0.0114	0.116	3	27	CPE,AKAP12,CRYAA
Pubmed	20200332	Family-based analysis of candidate genes for polycystic ovary syndrome.	GeneRIF	0.000223	3.3	0.0114	0.116	3	27	INHBB,CYP11A1,CAPN5
Pubmed	17192395	Comparative gene expression profiling of in vitro differentiated megakaryocytes and erythroblasts identifies novel activatory and inhibitory platelet membrane proteins.	Pubmed	0.000245	3.62	0.0114	0.116	4	69	RTN4R,KCNH2,CD74,HBA2
Pubmed	12652657	Identification of cathepsin K as a novel marker of adiposity in white adipose tissue.	GeneRIF	0.000249	3.68	0.0114	0.116	3	28	PSAP,CPE,CTSK
Pubmed	20802025	Genome-wide association study identifies novel loci for plasma levels of protein C: the ARIC study.	GeneRIF	0.000283	4.18	0.0114	0.116	2	6	PROCR,EDEM2
Pubmed	1347473	Mapping of six DNA markers on mouse chromosome 17.	Pubmed	0.000283	4.18	0.0114	0.116	2	6	HLA-DQB1,CRYAA
Pubmed	16242130	Assembly of major histocompatibility complex class II subunits with invariant chain.	Pubmed	0.000283	4.18	0.0114	0.116	2	6	HLA-DQB1,CD74
Pubmed	12480535	Association of phospholamban with a cGMP kinase signaling complex.	Pubmed	0.000283	4.18	0.0114	0.116	2	6	ACTA1,MRVI1
Pubmed	17218603	The bone morphogenetic protein antagonist noggin regulates mammalian cardiac morphogenesis.	GeneRIF	0.000283	4.18	0.0114	0.116	2	6	NPPA,BMP4
Pubmed	6985477	The structure and evolution of the human beta-globin gene family.	Pubmed	0.000283	4.18	0.0114	0.116	2	6	HBA2,HBB
Pubmed	15800000	Fgf10 expression identifies parabronchial smooth muscle cell progenitors and is required for their entry into the smooth muscle cell lineage.	Pubmed	0.000283	4.18	0.0114	0.116	2	6	ACTA1,BMP4
Pubmed	17452335	Fukutin-related protein associates with the sarcolemmal dystrophin-glycoprotein complex.	GeneRIF	0.000283	4.18	0.0114	0.116	2	6	DYSF,LARGE
Pubmed	10675891	The new human kallikrein gene family: implications in carcinogenesis.	Pubmed	0.000283	4.18	0.0114	0.116	2	6	KLK15,KLK10
Pubmed	22516433	Proteomic analysis of microvesicles from plasma of healthy donors reveals high individual variability.	Pubmed	0.000287	4.25	0.0114	0.116	5	131	ACTA1,ORM1,ORM2,HBA2,HBB
Pubmed	10591208	The DNA sequence of human chromosome 22.	Pubmed	0.000357	5.28	0.0114	0.116	7	298	RTN4R,GGT1,SMTN,DERL3,TSPO,SEZ6L,LARGE
Pubmed	14973287	HtrA1 serine protease inhibits signaling mediated by Tgfbeta family proteins.	GeneRIF	0.000395	5.84	0.0114	0.116	2	7	HTRA1,BMP4
Pubmed	1774067	One subunit of the transcription factor NF-Y maps close to the major histocompatibility complex in murine and human chromosomes.	Pubmed	0.000395	5.84	0.0114	0.116	2	7	HLA-DQB1,CRYAA
Pubmed	18325335	Affixin activates Rac1 via betaPIX in C2C12 myoblast.	GeneRIF	0.000395	5.84	0.0114	0.116	2	7	DYSF,ARHGEF6
Pubmed	12951563	Myelin-associated inhibitors of axonal regeneration in the adult mammalian CNS.	Pubmed	0.000395	5.84	0.0114	0.116	2	7	RTN4R,NGFR
Pubmed	12634786	Selection of evolutionarily conserved mucosal-associated invariant T cells by MR1.	GeneRIF	0.000395	5.84	0.0114	0.116	2	7	HLA-DQB1,CD74
Pubmed	20013655	Signalling molecules involved in mouse bladder smooth muscle cellular differentiation.	Pubmed	0.000395	5.84	0.0114	0.116	2	7	MYH11,BMP4
Pubmed	7769664	The murine AIDS defective provirus acts as an insertional mutagen in its infected target B cells.	Pubmed	0.000395	5.84	0.0114	0.116	2	7	THRA,NGFR
Pubmed	10969073	Tissue-specific expression patterns and fine mapping of the human kallikrein (KLK) locus on proximal 19q13.4.	Pubmed	0.000395	5.84	0.0114	0.116	2	7	KLK10,KLK14
Pubmed	25774556	A Novel Population of Inner Cortical Cells in the Adrenal Gland That Displays Sexually Dimorphic Expression of Thyroid Hormone Receptor-ß1.	Pubmed	0.000395	5.84	0.0114	0.116	2	7	CYP11A1,THRA
Pubmed	16870172	Tbx5-dependent rheostatic control of cardiac gene expression and morphogenesis.	GeneRIF	0.000408	6.04	0.0114	0.116	3	33	CDO1,NPPA,FBXO32
Pubmed	20562862	A mouse knockout library for secreted and transmembrane proteins.	Pubmed	0.000478	7.07	0.0114	0.116	8	411	RTN4R,AMICA1,ANGPTL2,LRRN4CL,ENPP5,STRA6,METTL7A,SEZ6L
Pubmed	15042700	T-box transcription factor Tbx2 represses differentiation and formation of the cardiac chambers.	GeneRIF	0.000525	7.76	0.0114	0.116	2	8	NPPA,BMP4
Pubmed	15206957	Developmentally regulated expression of mouse HtrA3 and its role as an inhibitor of TGF-beta signaling.	GeneRIF	0.000525	7.76	0.0114	0.116	2	8	HTRA1,BMP4
Pubmed	15743826	Inactivation of the Sema5a gene results in embryonic lethality and defective remodeling of the cranial vascular system.	GeneRIF	0.000525	7.76	0.0114	0.116	2	8	NPPA,BMP4
Pubmed	15172687	Ablation of the CLP-1 gene leads to down-regulation of the HAND1 gene and abnormality of the left ventricle of the heart and fetal death.	GeneRIF	0.000525	7.76	0.0114	0.116	2	8	NPPA,ACTA1
Pubmed	12297046	Modulation of cardiac growth and development by HOP, an unusual homeodomain protein.	GeneRIF	0.000525	7.76	0.0114	0.116	2	8	NPPA,ACTA1
Pubmed	17335080	Roles of glial p75NTR in axonal regeneration.	Pubmed	0.000525	7.76	0.0114	0.116	2	8	RTN4R,NGFR
Pubmed	17490411	Urothelial sonic hedgehog signaling plays an important role in bladder smooth muscle formation.	GeneRIF	0.000525	7.76	0.0114	0.116	2	8	MYH11,BMP4
Pubmed	7555018	The multiple functions of hemoglobin.	Pubmed	0.000525	7.76	0.0114	0.116	2	8	HBA2,HBB
Pubmed	11847210	The leucine-rich repeat protein PRELP binds perlecan and collagens and may function as a basement membrane anchor.	Pubmed	0.000525	7.76	0.0114	0.116	2	8	FBLN2,PRELP
Pubmed	22406547	NgR1 and NgR3 are receptors for chondroitin sulfate proteoglycans.	GeneRIF	0.000525	7.76	0.0114	0.116	2	8	RTN4R,NGFR
Pubmed	20562099	Effects of regulator of G protein signaling 19 (RGS19) on heart development and function.	GeneRIF	0.000525	7.76	0.0114	0.116	2	8	NPPA,BMP4
Pubmed	12692556	The p75 receptor acts as a displacement factor that releases Rho from Rho-GDI.	Pubmed	0.000525	7.76	0.0114	0.116	2	8	RTN4R,NGFR

Category	ID	Name	Source	p-value	q-value Bonferroni	q-value FDR B&H	q-value FDR B&Y	Hit Count in Query List	Hit Count in Genome	Hit in Query List
Pubmed	23263863	GWAS of blood cell traits identifies novel associated loci and epistatic interactions in Caucasian and African-American children.	Pubmed	0.000574	8.49	0.0114	0.116	3	37	CDSN,IFI16,HBB
Pubmed	11092979	Mapping of melanoma modifier loci in RET transgenic mice.	Pubmed	0.000673	9.95	0.0114	0.116	2	9	INHBB,IFI16
Pubmed	12781691	Tissue interactions pattern the mesenchyme of the embryonic mouse lung.	GeneRIF	0.000673	9.95	0.0114	0.116	2	9	MYH11,BMP4
Pubmed	14659802	Cardia bifida, defective heart development and abnormal neural crest migration in embryos lacking hypoxia-inducible factor-1alpha.	Pubmed	0.000673	9.95	0.0114	0.116	2	9	NPPA,NGFR
Pubmed	20093372	Nogo-a regulates neural precursor migration in the embryonic mouse cortex.	GeneRIF	0.000673	9.95	0.0114	0.116	2	9	RTN4R,NGFR
Pubmed	20063299	Role of bone morphogenetic proteins on cochlear hair cell formation: analyses of Noggin and Bmp2 mutant mice.	GeneRIF	0.000673	9.95	0.0114	0.116	2	9	BMP4,NGFR
Pubmed	822353	3' non-coding region sequences in eukaryotic messenger RNA.	Pubmed	0.000673	9.95	0.0114	0.116	2	9	HBA2,HBB
Pubmed	14974682	Quantitative trait locus analysis of plasma cholesterol and triglyceride levels in KK x RR F2 mice.	Pubmed	0.000673	9.95	0.0114	0.116	2	9	CYP11A1,CPE
Pubmed	25139858	Nephric duct insertion requires EphA4/EphA7 signaling from the pericloacal mesenchyme.	Pubmed	0.000723	10.7	0.0114	0.116	3	40	DPT,MYH11,BMP4
Pubmed	11756498	Activation of Rac GTPase by p75 is necessary for c-jun N-terminal kinase-mediated apoptosis.	Pubmed	0.000723	10.7	0.0114	0.116	3	40	NGFR,PREX1,ARHGEF6
Pubmed	15181537	A novel mouse Chromosome 2 congenic strain with obesity phenotypes.	Pubmed	0.000839	12.4	0.0114	0.116	2	10	ACSS1,LPIN3
Pubmed	21858045	Transcriptomic analysis brings new insight into the biological role of the prion protein during mouse embryogenesis.	GeneRIF	0.000839	12.4	0.0114	0.116	2	10	NAPSA,SLPI
Pubmed	16374517	Inactivation of focal adhesion kinase in cardiomyocytes promotes eccentric cardiac hypertrophy and fibrosis in mice.	GeneRIF	0.000839	12.4	0.0114	0.116	2	10	NPPA,ACTA1
Pubmed	8973143	Genetics of obesity: advances from rodent studies.	Pubmed	0.000839	12.4	0.0114	0.116	2	10	CPE,ADA
Pubmed	18094026	The chromatin-remodeling enzyme BRG1 plays an essential role in primitive erythropoiesis and vascular development.	GeneRIF	0.000839	12.4	0.0114	0.116	2	10	ALAS2,HBA2
Pubmed	11854359	Dynamics of major histocompatibility complex class II compartments during B cell receptor-mediated cell activation.	Pubmed	0.000839	12.4	0.0114	0.116	2	10	HLA-DQB1,CD74
Pubmed	21068062	Patent ductus arteriosus in mice with smooth muscle-specific Jag1 deletion.	GeneRIF	0.000839	12.4	0.0114	0.116	2	10	MYH11,SMTN
Pubmed	22923619	Cyclin D2 rescues size and function of GATA4 haplo-insufficient hearts.	GeneRIF	0.000839	12.4	0.0114	0.116	2	10	NPPA,ACTA1
Pubmed	1978715	Localization of a mouse heat shock Hsp70 gene within the H-2 complex.	Pubmed	0.000839	12.4	0.0114	0.116	2	10	HLA-DQB1,CRYAA
Pubmed	20628624	Evaluation of candidate stromal epithelial cross-talk genes identifies association between risk of serous ovarian cancer and TERT, a cancer susceptibility "hot-spot".	GeneRIF	0.00099	14.7	0.0114	0.116	5	172	IFI16,BMP4,MFAP4,MMP7,CTSK
Pubmed	19661464	Tbx20 interacts with smads to confine tbx2 expression to the atrioventricular canal.	GeneRIF	0.00102	15.1	0.0114	0.116	2	11	NPPA,BMP4
Pubmed	22925353	A genome-wide association study of seasonal pattern mania identifies NF1A as a possible susceptibility gene for bipolar disorder.	GeneRIF	0.00102	15.1	0.0114	0.116	2	11	PGM5,FAM189A2
Pubmed	24068960	miR-1/133a clusters cooperatively specify the cardiomyogenic lineage by adjustment of myocardin levels during embryonic heart development.	Pubmed	0.00102	15.1	0.0114	0.116	2	11	NPPA,MYH11
Pubmed	12015300	Transcription factor AP-2gamma is essential in the extra-embryonic lineages for early postimplantation development.	GeneRIF	0.00102	15.1	0.0114	0.116	2	11	ADA,BMP4
Pubmed	8530045	Mouse chromosomal location of the CCAAT/enhancer binding proteins C/EBP beta (Cebpb), C/EBP delta (Cebpd), and CRP1 (Cebpe).	Pubmed	0.00102	15.1	0.0114	0.116	2	11	ADA,BMP4
Pubmed	14633990	NRSF regulates the fetal cardiac gene program and maintains normal cardiac structure and function.	GeneRIF	0.00102	15.1	0.0114	0.116	2	11	NPPA,ACTA1
Pubmed	14499647	TACE is required for fetal murine cardiac development and modeling.	Pubmed	0.00102	15.1	0.0114	0.116	2	11	NPPA,ACTA1
Pubmed	1672293	Genetic maps of mouse chromosome 17 including 12 new anonymous DNA loci and 25 anchor loci.	Pubmed	0.00102	15.1	0.0114	0.116	2	11	HLA-DQB1,CRYAA
Pubmed	18087045	Embryonic stromal clones reveal developmental regulators of definitive hematopoietic stem cells.	Pubmed	0.00102	15.1	0.0114	0.116	2	11	BMP4,NGFR
Pubmed	12376548	NRAGE, a p75 neurotrophin receptor-interacting protein, induces caspase activation and cell death through a JNK-dependent mitochondrial pathway.	Pubmed	0.00109	16.1	0.0114	0.116	3	46	NGFR,PREX1,ARHGEF6
Pubmed	12970439	Down-modulation of mature major histocompatibility complex class II and up-regulation of invariant chain cell surface expression are well-conserved functions of human and simian immunodeficiency virus nef alleles.	Pubmed	0.00122	18.1	0.0114	0.116	2	12	HLA-DQB1,CD74
Pubmed	11593029	HIV-1 Nef impairs MHC class II antigen presentation and surface expression.	Pubmed	0.00122	18.1	0.0114	0.116	2	12	HLA-DQB1,CD74
Pubmed	20152827	Emx2 and early hair cell development in the mouse inner ear.	GeneRIF	0.00122	18.1	0.0114	0.116	2	12	BMP4,NGFR
Pubmed	2649166	A review of the molecular genetics of the human alpha-globin gene cluster.	Pubmed	0.00122	18.1	0.0114	0.116	2	12	HBA2,HBB
Pubmed	11950896	Overexpression of Polycomb-group gene rae28 in cardiomyocytes does not complement abnormal cardiac morphogenesis in mice lacking rae28 but causes dilated cardiomyopathy.	GeneRIF	0.00122	18.1	0.0114	0.116	2	12	NPPA,ACTA1
Pubmed	20508144	A global role for KLF1 in erythropoiesis revealed by ChIP-seq in primary erythroid cells.	GeneRIF	0.00122	18.1	0.0114	0.116	2	12	TSPO,ALAS2
Pubmed	17164424	GATA and Nkx factors synergistically regulate tissue-specific gene expression and development in vivo.	Pubmed	0.00122	18.1	0.0114	0.116	2	12	NAPSA,BMP4
Pubmed	24624459	Pancreatitis Overview	Pubmed	0.00122	18.1	0.0114	0.116	2	12	SPINK1,GGT1
Pubmed	17468400	Essential roles of the bHLH transcription factor Hrt2 in repression of atrial gene expression and maintenance of postnatal cardiac function.	GeneRIF	0.00122	18.1	0.0114	0.116	2	12	NPPA,ACTA1

Category	ID	Name	Source	p-value	q-value Bonferroni	q-value FDR B&H	q-value FDR B&Y	Hit Count in Query List	Hit Count in Genome	Hit in Query List
Pubmed	18292603	Myocardial Pitx2 differentially regulates the left atrial identity and ventricular asymmetric remodeling programs.	GeneRIF	0.00122	18.1	0.0114	0.116	2	12	NPPA,BMP4
Pubmed	19538736	Foxl2 functions in sex determination and histogenesis throughout mouse ovary development.	GeneRIF	0.00123	18.2	0.0114	0.116	3	48	ZBTB7C,INHBB,CYP11A1
Pubmed	23791818	Fibronectin and integrin alpha 5 play requisite roles in cardiac morphogenesis.	Pubmed	0.00144	21.3	0.0114	0.116	2	13	NPPA,BMP4
Pubmed	7959737	Mapping of the syndecan genes in the mouse: linkage with members of the myc gene family.	Pubmed	0.00144	21.3	0.0114	0.116	2	13	NPPA,ADA
Pubmed	19540218	Lmx1a maintains proper neurogenic, sensory, and non-sensory domains in the mammalian inner ear.	GeneRIF	0.00144	21.3	0.0114	0.116	2	13	BMP4,NGFR
Pubmed	23946491	The transcription factor Foxf1 binds to serum response factor and myocardin to regulate gene transcription in visceral smooth muscle cells.	Pubmed	0.00144	21.3	0.0114	0.116	2	13	MYH11,BMP4
Pubmed	8406442	High-resolution comparative mapping of mouse chromosome 17.	Pubmed	0.00144	21.3	0.0114	0.116	2	13	HLA-DQB1,CRYAA
Pubmed	22260730	Transcriptional ontogeny of the developing liver.	Pubmed	0.00144	21.3	0.0114	0.116	2	13	SPINK1,ALAS2
Pubmed	19958090	Genetic determinants of warfarin dosing in the Han-Chinese population.	GeneRIF	0.00144	21.3	0.0114	0.116	2	13	ORM1,ORM2
Pubmed	19272526	The expression and crucial roles of BMP signaling in development of smooth muscle progenitor cells in the mouse embryonic gut.	Pubmed	0.00144	21.3	0.0114	0.116	2	13	BMP4,NGFR
Pubmed	23584254	Expression of the BMP receptor Alk3 in the second heart field is essential for development of the dorsal mesenchymal protrusion and atrioventricular septation.	GeneRIF	0.00144	21.3	0.0114	0.116	2	13	ACTA1,BMP4
Pubmed	17286962	Efficient capture of cardiogenesis-associated genes expressed in ES cells.	Pubmed	0.00144	21.3	0.0114	0.116	2	13	FBXO32,ASB2
Pubmed	21982860	A secreted protein microarray platform for extracellular protein interaction discovery.	Pubmed	0.00156	23	0.0114	0.116	3	52	AMICA1,MPZL3,NGFR
Pubmed	20110314	SIX1 acts synergistically with TBX18 in mediating ureteral smooth muscle formation.	GeneRIF	0.00168	24.8	0.0114	0.116	2	14	MYH11,BMP4
Pubmed	22847922	Regulation of the ovarian reserve by members of the transforming growth factor beta family.	Pubmed	0.00168	24.8	0.0114	0.116	2	14	INHBB,BMP4
Pubmed	17766470	Loss of mXinalpha, an intercalated disk protein, results in cardiac hypertrophy and cardiomyopathy with conduction defects.	Pubmed	0.00168	24.8	0.0114	0.116	2	14	NPPA,ACTA1
Pubmed	18448675	Cardiac developmental defects and eccentric right ventricular hypertrophy in cardiomyocyte focal adhesion kinase (FAK) conditional knockout mice.	GeneRIF	0.00168	24.8	0.0114	0.116	2	14	NPPA,ACTA1
Pubmed	22829776	A genome-wide association meta-analysis of circulating sex hormone-binding globulin reveals multiple Loci implicated in sex steroid hormone regulation.	Pubmed	0.00168	24.8	0.0114	0.116	2	14	BAIAP2L1,CD68
Pubmed	12438746	Mutagenic transgene insertion into a region of high gene density and multiple linkage disruptions on mouse chromosome 11.	GeneRIF	0.00168	24.8	0.0114	0.116	2	14	THRA,NGFR
Pubmed	1450512	Mouse microsatellites from a flow-sorted 4:6 Robertsonian chromosome.	Pubmed	0.00168	24.8	0.0114	0.116	2	14	NPPA,NGFR
Pubmed	21382375	Inositol 1,4,5-trisphosphate receptors are essential for the development of the second heart field.	GeneRIF	0.00168	24.8	0.0114	0.116	2	14	NPPA,BMP4
Pubmed	19056867	Large-scale proteomics and phosphoproteomics of urinary exosomes.	Pubmed	0.00176	26.1	0.0114	0.116	12	1016	NAPSA,PSAP,DYSF,PDXK,GSS,TGM2,ANGPTL2,BAIAP2L1,TSPO,CAPN5,HBB,KLK1
Pubmed	11471062	High-throughput sequence identification of gene coding variants within alcohol-related QTLs.	Pubmed	0.00193	28.5	0.0114	0.116	3	56	ADA,GSS,TSPO
Pubmed	19015276	microRNA-133a regulates cardiomyocyte proliferation and suppresses smooth muscle gene expression in the heart.	Pubmed	0.00193	28.5	0.0114	0.116	2	15	NPPA,KCNH2
Pubmed	21304516	Chromatin remodelling complex dosage modulates transcription factor function in heart development.	GeneRIF	0.00193	28.5	0.0114	0.116	2	15	NPPA,BMP4
Pubmed	22158707	Thymosin beta 4 is dispensable for murine cardiac development and function.	Pubmed	0.00193	28.5	0.0114	0.116	2	15	NPPA,ACTA1
Pubmed	15461802	A genome annotation-driven approach to cloning the human ORFeome.	Pubmed	0.00197	29.1	0.0114	0.116	6	295	RTN4R,GGT1,DERL3,TSPO,SEZ6L,LARGE
Pubmed	23760955	Porcn-dependent Wnt signaling is not required prior to mouse gastrulation.	GeneRIF	0.0022	32.5	0.0114	0.116	2	16	PORCN,BMP4
Pubmed	15944189	Comparative analysis of genes downstream of the Hoxd cluster in developing digits and external genitalia.	Pubmed	0.0022	32.5	0.0114	0.116	2	16	PAPSS2,STRA6
Pubmed	17048007	Association of warfarin dose with genes involved in its action and metabolism.	GeneRIF	0.0022	32.5	0.0114	0.116	2	16	ORM1,ORM2
Pubmed	15464586	GATA4 is a dosage-sensitive regulator of cardiac morphogenesis.	GeneRIF	0.0022	32.5	0.0114	0.116	2	16	NPPA,BMP4
Pubmed	24840128	A cardiomyocyte-specific Wdr1 knockout demonstrates essential functional roles for actin disassembly during myocardial growth and maintenance in mice.	Pubmed	0.0022	32.5	0.0114	0.116	2	16	NPPA,ACTA1
Pubmed	18778700	Dominant inhibition of lens placode formation in mice.	Pubmed	0.0022	32.5	0.0114	0.116	2	16	CRYAA,BMP4
Pubmed	15843409	Tbx20 dose-dependently regulates transcription factor networks required for mouse heart and motoneuron development.	GeneRIF	0.0022	32.5	0.0114	0.116	2	16	NPPA,BMP4
Pubmed	17959659	Human immunodeficiency virus type 1 Vpu protein interacts with CD74 and modulates major histocompatibility complex class II presentation.	GeneRIF	0.0022	32.5	0.0114	0.116	2	16	HLA-DQB1,CD74
Pubmed	17322895	Hdac2 regulates the cardiac hypertrophic response by modulating Gsk3 beta activity.	GeneRIF	0.0022	32.5	0.0114	0.116	2	16	NPPA,ACTA1
Pubmed	22747683	Genetic variants associated with breast size also influence breast cancer risk.	Pubmed	0.00235	34.7	0.0114	0.116	3	60	INHBB,LMOD1,DCLK1
Pubmed	1889815	The gene map of the Norway rat (Rattus norvegicus) and comparative mapping with mouse and man.	Pubmed	0.00235	34.7	0.0114	0.116	3	60	NPPA,CRYAA,THRA
Pubmed	18667074	Msx1 and Msx2 are required for endothelial-mesenchymal transformation of the atrioventricular cushions and patterning of the atrioventricular myocardium.	GeneRIF	0.00248	36.8	0.0114	0.116	2	17	NPPA,BMP4
Pubmed	18776146	Teashirt 3 is necessary for ureteral smooth muscle differentiation downstream of SHH and BMP4.	GeneRIF	0.00248	36.8	0.0114	0.116	2	17	MYH11,BMP4
Pubmed	24904546	The activity of Nef on HIV-1 infectivity.	Pubmed	0.00248	36.8	0.0114	0.116	2	17	HLA-DQB1,CD74

Category	ID	Name	Source	p-value	q-value Bonferroni	q-value FDR B&H	q-value FDR B&Y	Hit Count in Query List	Hit Count in Genome	Hit in Query List
Pubmed	15194762	Nef proteins from simian immunodeficiency virus-infected chimpanzees interact with p21-activated kinase 2 and modulate cell surface expression of various human receptors.	GeneRIF	0.00248	36.8	0.0114	0.116	2	17	HLA-DQB1,CD74
Pubmed	12702649	Abnormal epidermal differentiation and impaired epithelial-mesenchymal tissue interactions in mice lacking the retinoblastoma relatives p107 and p130.	GeneRIF	0.00248	36.8	0.0114	0.116	2	17	BMP4,NGFR
Pubmed	9876177	Abrogation of the Cripto gene in mouse leads to failure of postgastrulation morphogenesis and lack of differentiation of cardiomyocytes.	Pubmed	0.00248	36.8	0.0114	0.116	2	17	NPPA,MYH11
Pubmed	20300111	Jun and JunD-dependent functions in cell proliferation and stress response.	GeneRIF	0.00248	36.8	0.0114	0.116	2	17	CDO1,GSS
Pubmed	20347762	The Mediator complex protein Med31 is required for embryonic growth and cell proliferation during mammalian development.	GeneRIF	0.00248	36.8	0.0114	0.116	2	17	MUSTN1,ALAS2
Pubmed	19845895	Fine mapping of the CELIAC2 locus on chromosome 5q31-q33 in the Finnish and Hungarian populations.	GeneRIF	0.00248	36.8	0.0114	0.116	2	17	SPINK1,CD74
Pubmed	2567264	A comprehensive genetic map of murine chromosome 11 reveals extensive linkage conservation between mouse and human.	Pubmed	0.00248	36.8	0.0114	0.116	2	17	THRA,NGFR
Pubmed	15489335	Human ORFeome version 1.1: a platform for reverse proteomics.	Pubmed	0.00248	36.8	0.0114	0.116	2	17	CPZ,TACSTD2
Pubmed	23351706	Multiple essential roles for primary cilia in heart development.	Pubmed	0.00248	36.8	0.0114	0.116	2	17	BMP4,NGFR
Pubmed	12787561	Ten years on: mediation of cell death by the common neurotrophin receptor p75(NTR).	Pubmed	0.0027	39.9	0.0114	0.116	3	63	NGFR,PREX1,ARHGEF6
Pubmed	9716414	Activin is an essential early mesenchymal signal in tooth development that is required for patterning of the murine dentition.	Pubmed	0.00279	41.2	0.0114	0.116	2	18	INHBB,BMP4
Pubmed	25057208	LMO4 functions as a negative regulator of sensory organ formation in the mammalian cochlea.	Pubmed	0.00279	41.2	0.0114	0.116	2	18	BMP4,NGFR
Pubmed	19723499	T lymphocytes amplify the anabolic activity of parathyroid hormone through Wnt10b signaling.	Pubmed	0.00279	41.2	0.0114	0.116	2	18	HLA-DQB1,CD74
Pubmed	19592578	Regulation of cell surface protease matriptase by HAI2 is essential for placental development, neural tube closure and embryonic survival in mice.	GeneRIF	0.00279	41.2	0.0114	0.116	2	18	KLK15,KLK7
Pubmed	20301533	Congenital Diaphragmatic Hernia Overview	Pubmed	0.00279	41.2	0.0114	0.116	2	18	PORCN,STRA6
Pubmed	16341674	Transcriptome analysis of human gastric cancer.	Pubmed	0.00292	43.1	0.0114	0.116	9	672	CDK18,KLK10,RTN4R,BMP4,GJB3,DERL3,GPNMB,HYI,TACSTD2
Pubmed	20194497	TEAD-1 overexpression in the mouse heart promotes an age-dependent heart dysfunction.	GeneRIF	0.00311	45.9	0.0114	0.116	2	19	NPPA,ACTA1
Pubmed	24165912	Genome-wide association study identifies 8 novel loci associated with blood pressure responses to interventions in Han Chinese.	Pubmed	0.00311	45.9	0.0114	0.116	2	19	ARL4C,LARGE
Pubmed	21519551	Identification of genes concordantly expressed with Atoh1 during inner ear development.	Pubmed	0.00311	45.9	0.0114	0.116	2	19	LMOD1,FBXO32
Pubmed	15385160	Requirement for Mab21l2 during development of murine retina and ventral body wall.	GeneRIF	0.00311	45.9	0.0114	0.116	2	19	CRYAA,BMP4
Pubmed	18316483	Heart-specific ablation of Prkar1a causes failure of heart development and myxomagenesis.	GeneRIF	0.00311	45.9	0.0114	0.116	2	19	NPPA,ACTA1
Pubmed	23946445	Hedgehog signaling regulates prosensory cell properties during the basal-to-apical wave of hair cell differentiation in the mammalian cochlea.	Pubmed	0.00311	45.9	0.0114	0.116	2	19	BMP4,NGFR
Pubmed	23166209	Impact of ancestry and common genetic variants on QT interval in African Americans.	Pubmed	0.00311	45.9	0.0114	0.116	2	19	CLDN15,KCNH2
Pubmed	22673621	Identification of atrogin-1-targeted proteins during the myostatin-induced skeletal muscle wasting.	GeneRIF	0.00316	46.8	0.0114	0.116	4	137	ACTA1,FBLN2,FBXO32,HBA2
Pubmed	16985003	Large-scale analysis of ion channel gene expression in the mouse heart during perinatal development.	Pubmed	0.00316	46.8	0.0114	0.116	4	137	KCNK12,KCNH2,KCNN4,SCN3B
Pubmed	19598235	Genes related to sex steroids, neural growth, and social-emotional behavior are associated with autistic traits, empathy, and Asperger syndrome.	GeneRIF	0.00321	47.6	0.0114	0.116	3	67	CYP11A1,TSPO,NGFR
Pubmed	23251661	Novel genetic loci identified for the pathophysiology of childhood obesity in the Hispanic population.	Pubmed	0.00335	49.6	0.0114	0.116	10	819	CRCT1,TRABD2B,SPINK1,RTN4R,PKD2L1,PAK6,IFI16,BAIAP2L1,LIPG,DCLK1
Pubmed	21289070	DOT1L regulates dystrophin expression and is critical for cardiac function.	GeneRIF	0.00344	50.9	0.0114	0.116	2	20	NPPA,ACTA1
Pubmed	21979435	Transforming growth factor-ß signaling in myogenic cells regulates vascular morphogenesis, differentiation, and matrix synthesis.	GeneRIF	0.00344	50.9	0.0114	0.116	2	20	MYH11,SMTN
Pubmed	23326517	Insights into the genetic architecture of early stage age-related macular degeneration: a genome-wide association study meta-analysis.	Pubmed	0.00344	50.9	0.0114	0.116	2	20	HTRA1,INHBB
Pubmed	19948975	Integrative predictive model of coronary artery calcification in atherosclerosis.	GeneRIF	0.00345	51	0.0114	0.116	5	229	NPPA,HTRA1,ACTA1,LIPG,CAPN5
Pubmed	22008794	Integrated microarray and ChIP analysis identifies multiple Foxa2 dependent target genes in the notochord.	Pubmed	0.00351	51.9	0.0114	0.116	4	141	CDO1,HTRA1,SPINK1,TGM2
Pubmed	19851296	Assessment of a polymorphism of SDK1 with hypertension in Japanese Individuals.	GeneRIF	0.0036	53.2	0.0114	0.116	4	142	ACSS1,AKAP12,ERGIC1,PXDN
Pubmed	20198315	Association of genetic variants with hemorrhagic stroke in Japanese individuals.	GeneRIF	0.00369	54.5	0.0114	0.116	4	143	ACSS1,AKAP12,ERGIC1,PXDN
Pubmed	20696915	Severe anemia in the Nan mutant mouse caused by sequence-selective disruption of erythroid Kruppel-like factor.	GeneRIF	0.00379	56.1	0.0114	0.116	2	21	KCNN4,ALAS2
Pubmed	17107998	Heparan sulfate biosynthetic gene Ndst1 is required for FGF signaling in early lens development.	GeneRIF	0.00379	56.1	0.0114	0.116	2	21	CRYAA,BMP4
Pubmed	21900206	A directed protein interaction network for investigating intracellular signal transduction.	Pubmed	0.00394	58.3	0.0114	0.116	12	1122	CPNE2,ANKRD35,MASP1,RPL35A,PSAP,CHDH,PAK6,AKAP12,TGM2,NGFR,CLEC3B,ARHGEF6
Pubmed	18554416	Identification of transcripts with enriched expression in the developing and adult pancreas.	Pubmed	0.00402	59.5	0.0114	0.116	8	577	NPPA,SPINK1,CPZ,AKAP12,TGM2,FBLN2,ASB2,HBA2
Pubmed	14627618	Smooth muscle contraction and relaxation.	Pubmed	0.00416	61.5	0.0114	0.116	2	22	MYH11,LMOD1
Pubmed	15843407	T-box genes coordinate regional rates of proliferation and regional specification during cardiogenesis.	GeneRIF	0.00416	61.5	0.0114	0.116	2	22	NPPA,BMP4

Category	ID	Name	Source	p-value	q-value Bonferroni	q-value FDR B&H	q-value FDR B&Y	Hit Count in Query List	Hit Count in Genome	Hit in Query List
Pubmed	21693521	Msx genes define a population of mural cell precursors required for head blood vessel maturation.	GeneRIF	0.00416	61.5	0.0114	0.116	2	22	MYH11,BMP4
Pubmed	10021345	The cardiac homeobox gene Csx/Nkx2.5 lies genetically upstream of multiple genes essential for heart development.	Pubmed	0.00416	61.5	0.0114	0.116	2	22	NPPA,BMP4
Pubmed	21343882	Human neurotrophin receptor p75NTR defines differentiation-oriented skeletal muscle precursor cells: implications for muscle regeneration.	GeneRIF	0.0044	65.1	0.0114	0.116	1	1	NGFR
Pubmed	17961316	In vitro expression of beta-thalassaemia gene (IVS1-1G>C) reveals complete inactivation of the normal 5' splice site and alternative aberrant RNA splicing.	GeneRIF	0.0044	65.1	0.0114	0.116	1	1	HBB
Pubmed	23069627	Cathepsin K knockout mitigates high-fat diet-induced cardiac hypertrophy and contractile dysfunction.	GeneRIF	0.0044	65.1	0.0114	0.116	1	1	CTSK
Pubmed	22885985	Cytomegalovirus and tumor stress surveillance by binding of a human ¿d T cell antigen receptor to endothelial protein C receptor.	GeneRIF	0.0044	65.1	0.0114	0.116	1	1	PROCR
Pubmed	20479154	Selective molecular potassium channel blockade prevents atrial fibrillation.	GeneRIF	0.0044	65.1	0.0114	0.116	1	1	KCNH2
Pubmed	15615768	Onset and inheritance of abnormal epigenetic regulation in hematopoietic cells.	GeneRIF	0.0044	65.1	0.0114	0.116	1	1	HBB
Pubmed	8674818	The post-translational processing and intracellular sorting of carboxypeptidase H in the islets of Langerhans.	Pubmed	0.0044	65.1	0.0114	0.116	1	1	CPE
Pubmed	2026759	Cloning and sequencing of a novel 64-kDa autoantigen recognized by patients with autoimmune thyroid disease.	Pubmed	0.0044	65.1	0.0114	0.116	1	1	LMOD1
Pubmed	15452027	Atrial natriuretic peptide dose-dependently inhibits pressure overload-induced cardiac remodeling.	Pubmed	0.0044	65.1	0.0114	0.116	1	1	NPPA
Pubmed	2555693	An intronic 10-base-pair deletion in a class II A beta gene affects RNA processing.	Pubmed	0.0044	65.1	0.0114	0.116	1	1	HLA-DQB1
Pubmed	23029143	The schizophrenia-associated Kv11.1-3.1 isoform results in reduced current accumulation during repetitive brief depolarizations.	GeneRIF	0.0044	65.1	0.0114	0.116	1	1	KCNH2
Pubmed	17287605	ADA*2 allele of the adenosine deaminase gene may protect against coronary artery disease.	GeneRIF	0.0044	65.1	0.0114	0.116	1	1	ADA
Pubmed	20146992	Influence of significant weight loss on serum matrix metalloproteinase (MMP)-7 levels.	GeneRIF	0.0044	65.1	0.0114	0.116	1	1	MMP7
Pubmed	22220472	Physiopathological roles of human transglutaminase 2.	GeneRIF	0.0044	65.1	0.0114	0.116	1	1	TGM2
Pubmed	24453844	Raised serum adenosine deaminase level in nonobese type 2 diabetes mellitus.	Pubmed	0.0044	65.1	0.0114	0.116	1	1	ADA
Pubmed	24886657	The transcription factor FOXO4 is down-regulated and inhibits tumor proliferation and metastasis in gastric cancer.	Pubmed	0.0044	65.1	0.0114	0.116	1	1	FOXO4
Pubmed	1358880	A retinoic acid-inducible mRNA from human erythroleukemia cells encodes a novel tissue transglutaminase homologue.	Pubmed	0.0044	65.1	0.0114	0.116	1	1	TGM2
Pubmed	17158930	Distinctive signatures of histone methylation in transcribed coding and noncoding human beta-globin sequences.	GeneRIF	0.0044	65.1	0.0114	0.116	1	1	HBB
Pubmed	23997099	LQT2 nonsense mutations generate trafficking defective NH2-terminally truncated channels by the reinitiation of translation.	Pubmed	0.0044	65.1	0.0114	0.116	1	1	KCNH2
Pubmed	17442832	AKAP12 regulates human blood-retinal barrier formation by downregulation of hypoxia-inducible factor-1alpha.	GeneRIF	0.0044	65.1	0.0114	0.116	1	1	AKAP12
Pubmed	24759929	EHD3-dependent endosome pathway regulates cardiac membrane excitability and physiology.	Pubmed	0.0044	65.1	0.0114	0.116	1	1	EHD3
Pubmed	24526691	Role of endothelial cell-derived angptl2 in vascular inflammation leading to endothelial dysfunction and atherosclerosis progression.	Pubmed	0.0044	65.1	0.0114	0.116	1	1	ANGPTL2
Pubmed	17624316	Functional consequences of methionine oxidation of hERG potassium channels.	GeneRIF	0.0044	65.1	0.0114	0.116	1	1	KCNH2
Pubmed	18570253	Expression and regulation of HTRA1 during chick and early mouse development.	Pubmed	0.0044	65.1	0.0114	0.116	1	1	HTRA1
Pubmed	16996541	Mutation impact on dysferlin inferred from database analysis and computer-based structural predictions.	GeneRIF	0.0044	65.1	0.0114	0.116	1	1	DYSF
Pubmed	19755382	EKV mutant connexin 31 associated cell death is mediated by ER stress.	GeneRIF	0.0044	65.1	0.0114	0.116	1	1	GJB3
Pubmed	1517104	HB Prato [alpha 31(B12)Arg----Ser] in a Calabrian family.	Pubmed	0.0044	65.1	0.0114	0.116	1	1	HBA2
Pubmed	17825429	B7-H3 regulates the development of experimental allergic conjunctivitis in mice.	GeneRIF	0.0044	65.1	0.0114	0.116	1	1	CD276
Pubmed	17585114	KCa3.1 Ca2+ activated K+ channels regulate human airway smooth muscle proliferation.	GeneRIF	0.0044	65.1	0.0114	0.116	1	1	KCNN4
Pubmed	11822023	Evidence of genetic interaction between the beta-globin complex and chromosome 8q in the expression of fetal hemoglobin.	GeneRIF	0.0044	65.1	0.0114	0.116	1	1	HBB
Pubmed	25299965	b-Gamma-glutamyltransferase activity in human vulnerable carotid plaques.	Pubmed	0.0044	65.1	0.0114	0.116	1	1	GGT1
Pubmed	23724628	Endothelial cell protein C receptor (EPCR) is expressed by lung carcinoma and correlated with clinical parameters.	GeneRIF	0.0044	65.1	0.0114	0.116	1	1	PROCR
Pubmed	22394545	Functional characterization of naturally occurring transglutaminase 2 mutants implicated in early-onset type 2 diabetes.	GeneRIF	0.0044	65.1	0.0114	0.116	1	1	TGM2
Pubmed	9256258	Crystal structure of tetranectin, a trimeric plasminogen-binding protein with an alpha-helical coiled coil.	Pubmed	0.0044	65.1	0.0114	0.116	1	1	CLEC3B
Pubmed	19351586	Kinetic and structural studies of the role of the active site residue Asp235 of human pyridoxal kinase.	GeneRIF	0.0044	65.1	0.0114	0.116	1	1	PDXK
Pubmed	25501240	Correlation between DQB1 genetic polymorphism and genetic susceptibility in patients diagnosed with irritable bowel syndrome with diarrhea.	Pubmed	0.0044	65.1	0.0114	0.116	1	1	HLA-DQB1
Pubmed	7814155	nmb, a novel gene, is expressed in low-metastatic human melanoma cell lines and xenografts.	Pubmed	0.0044	65.1	0.0114	0.116	1	1	GPNMB
Pubmed	24146193	Specific expression of k63-linked ubiquitination of calmodulin-like protein 5 in breast cancer of premenopausal patients.	Pubmed	0.0044	65.1	0.0114	0.116	1	1	CALML5

Category	ID	Name	Source	p-value	q-value Bonferroni	q-value FDR B&H	q-value FDR B&Y	Hit Count in Query List	Hit Count in Genome	Hit in Query List
Pubmed	25282687	Lack of association between serum gamma-glutamyltransferase and carotid atherosclerosis: the Namwon Study.	Pubmed	0.0044	65.1	0.0114	0.116	1	1	GGT1
Pubmed	20042390	[Studies of effect of prosaposin on cell proliferation, cell apoptosis and its possible molecular mechanism].	GeneRIF	0.0044	65.1	0.0114	0.116	1	1	PSAP
Pubmed	3360326	Expression of human alpha 1-acid glycoprotein genes in cultured cells and in transgenic mice.	Pubmed	0.0044	65.1	0.0114	0.116	1	1	ORM1
Pubmed	23697984	Tissue kallikrein-modified mesenchymal stem cells provide enhanced protection against ischemic cardiac injury after myocardial infarction.	Pubmed	0.0044	65.1	0.0114	0.116	1	1	KLK1
Pubmed	19567873	Identification of the active form of endothelial lipase, a homodimer in a head-to-tail conformation.	GeneRIF	0.0044	65.1	0.0114	0.116	1	1	LIPG
Pubmed	11869935	DNAase I hypersensitive site 3' to the beta-globin gene cluster contains a TAA insertion specific for beta(S)-Benin haplotype.	GeneRIF	0.0044	65.1	0.0114	0.116	1	1	HBB
Pubmed	25864764	Atrial natriuretic Peptide expression in human articular cartilage.	Pubmed	0.0044	65.1	0.0114	0.116	1	1	NPPA
Pubmed	21556485	Translational research and therapeutic perspectives in dysferlinopathies.	GeneRIF	0.0044	65.1	0.0114	0.116	1	1	DYSF
Pubmed	21044581	Mutations within the S4-S5 linker alter voltage sensor constraints in hERG K+ channels.	GeneRIF	0.0044	65.1	0.0114	0.116	1	1	KCNH2
Pubmed	23806156	Bullous pemphigoid: etiology, pathogenesis, and inducing factors: facts and controversies.	Pubmed	0.0044	65.1	0.0114	0.116	1	1	HLA-DQB1
Pubmed	24321711	White matter abnormalities in 22q11.2 deletion syndrome: preliminary associations with the Nogo-66 receptor gene and symptoms of psychosis.	Pubmed	0.0044	65.1	0.0114	0.116	1	1	RTN4R
Pubmed	21614011	Identification of trichohyalin-like 1, an s100 fused-type protein selectively expressed in hair follicles.	GeneRIF	0.0044	65.1	0.0114	0.116	1	1	TCHHL1
Pubmed	19488929	Acid and bile salt up-regulate BMP4 expression in human esophageal epithelium cells.	GeneRIF	0.0044	65.1	0.0114	0.116	1	1	BMP4
Pubmed	1375242	Preferential use of a T cell receptor V beta gene by acetylcholine receptor reactive T cells from myasthenia gravis-susceptible mice.	Pubmed	0.0044	65.1	0.0114	0.116	1	1	HLA-DQB1
Pubmed	23281426	Voxel-based imaging of translocator protein 18 kDa (TSPO) in high-resolution PET.	GeneRIF	0.0044	65.1	0.0114	0.116	1	1	TSPO
Pubmed	23510199	The endothelial lipase protein is promising urinary biomarker for diagnosis of gastric cancer.	GeneRIF	0.0044	65.1	0.0114	0.116	1	1	LIPG
Pubmed	18489063	A novel H2A-E+ transgenic model susceptible to human but not mouse thyroglobulin-induced autoimmune thyroiditis: identification of mouse pathogenic epitopes.	Pubmed	0.0044	65.1	0.0114	0.116	1	1	HLA-DQB1
Pubmed	12568399	Cathepsin K mRNA and protein expression in prostate cancer progression.	GeneRIF	0.0044	65.1	0.0114	0.116	1	1	CTSK
Pubmed	11165490	Xgravin-like (Xgl), a novel putative a-kinase anchoring protein (AKAP) expressed during embryonic development in Xenopus.	Pubmed	0.0044	65.1	0.0114	0.116	1	1	AKAP12
Pubmed	8128229	Dependence on p75 for innervation of some sympathetic targets.	Pubmed	0.0044	65.1	0.0114	0.116	1	1	NGFR
Pubmed	21035106	MASP1 mutations in patients with facial, umbilical, coccygeal, and auditory findings of Carnevale, Malpuech, OSA, and Michels syndromes.	GeneRIF	0.0044	65.1	0.0114	0.116	1	1	MASP1
Pubmed	19471889	Exploration of senescence-associated genes by differential display reverse transcription polymerase chain reaction: prosaposin as a novel senescence-associated gene.	GeneRIF	0.0044	65.1	0.0114	0.116	1	1	PSAP
Pubmed	12973686	Co-expression of trypsin and tumour-associated trypsin inhibitor (TATI) in colorectal adenocarcinomas.	GeneRIF	0.0044	65.1	0.0114	0.116	1	1	SPINK1
Pubmed	17141155	Monounsaturated fatty acid modification of Wnt protein: its role in Wnt secretion.	Pubmed	0.0044	65.1	0.0114	0.116	1	1	PORCN
Pubmed	24973596	Atrial natriuretic peptide: an old hormone or a new cytokine?	Pubmed	0.0044	65.1	0.0114	0.116	1	1	NPPA
Pubmed	19668779	Characterization of novel KCNH2 mutations in type 2 long QT syndrome manifesting as seizures.	GeneRIF	0.0044	65.1	0.0114	0.116	1	1	KCNH2
Pubmed	24572971	No association between the HLA-DQB1*0501 variant and schizophrenia in a Chinese population.	Pubmed	0.0044	65.1	0.0114	0.116	1	1	HLA-DQB1
Pubmed	3006050	The nerve growth factor receptor gene is at human chromosome region 17q12-17q22, distal to the chromosome 17 breakpoint in acute leukemias.	Pubmed	0.0044	65.1	0.0114	0.116	1	1	NGFR
Pubmed	17213386	Transcriptional regulation of the cholesterol side chain cleavage cytochrome P450 gene (CYP11A1) revisited: binding of GATA, cyclic adenosine 3',5'-monophosphate response element-binding protein and activating protein (AP)-1 proteins to a distal novel cluster of cis-regulatory elements potentiates AP-2 and steroidogenic factor-1-dependent gene expression in the rodent placenta and ovary.	GeneRIF	0.0044	65.1	0.0114	0.116	1	1	CYP11A1
Pubmed	18565216	Altered fractalkine cleavage potentially promotes local inflammation in NOD salivary gland.	GeneRIF	0.0044	65.1	0.0114	0.116	1	1	CX3CL1
Pubmed	14630717	Aberrant maturation of astrocytes in thyroid hormone receptor alpha 1 knockout mice reveals an interplay between thyroid hormone receptor isoforms.	GeneRIF	0.0044	65.1	0.0114	0.116	1	1	THRA
Pubmed	18412162	Intercellular MHC transfer between thymic epithelial and dendritic cells.	Pubmed	0.0044	65.1	0.0114	0.116	1	1	HLA-DQB1
Pubmed	21094133	Transglutaminase 2 gene ablation protects against renal ischemic injury by blocking constant NF-¿B activation.	GeneRIF	0.0044	65.1	0.0114	0.116	1	1	TGM2
Pubmed	11591798	Adenosine-dependent airway inflammation and hyperresponsiveness in partially adenosine deaminase-deficient mice.	Pubmed	0.0044	65.1	0.0114	0.116	1	1	ADA
Pubmed	11939511	A novel beta0-thalassemia mutation at codon 55 (-A) and a rare 17 bp deletion at codons 126-131 in the Indian population.	GeneRIF	0.0044	65.1	0.0114	0.116	1	1	HBB
Pubmed	20301502	X-Linked Opitz G/BBB Syndrome	Pubmed	0.0044	65.1	0.0114	0.116	1	1	MID1

Category	ID	Name	Source	p-value	q-value Bonferroni	q-value FDR B&H	q-value FDR B&Y	Hit Count in Query List	Hit Count in Genome	Hit in Query List
Pubmed	23877403	Plasma endothelial protein C receptor influences innate immune response in ovarian cancer by decreasing the population of natural killer and TH17 helper cells.	Pubmed	0.0044	65.1	0.0114	0.116	1	1	PROCR
Pubmed	12151088	Ectopic bone formation by electroporatic transfer of bone morphogenetic protein-4 gene.	GeneRIF	0.0044	65.1	0.0114	0.116	1	1	BMP4
Pubmed	2560337	Deduced amino acid sequence from human phosphoribosylpyrophosphate synthetase subunit II cDNA.	Pubmed	0.0044	65.1	0.0114	0.116	1	1	PRPS2
Pubmed	16342940	Disulfide structure of the leucine-rich repeat C-terminal cap and C-terminal stalk region of Nogo-66 receptor.	GeneRIF	0.0044	65.1	0.0114	0.116	1	1	RTN4R
Pubmed	12730694	Transcription of antisense RNA leading to gene silencing and methylation as a novel cause of human genetic disease.	GeneRIF	0.0044	65.1	0.0114	0.116	1	1	HBA2
Pubmed	20798032	Fine-tuning of neuronal architecture requires two profilin isoforms.	Pubmed	0.0044	65.1	0.0114	0.116	1	1	NGFR
Pubmed	20864802	Transglutaminase-mediated oligomerization promotes osteoblast adhesive properties of osteopontin and bone sialoprotein.	GeneRIF	0.0044	65.1	0.0114	0.116	1	1	TGM2
Pubmed	19146825	Transglutaminases expression in human supraspinatus tendon ruptures and in mouse tendons.	Pubmed	0.0044	65.1	0.0114	0.116	1	1	TGM2
Pubmed	24191290	Tissue transglutaminase contributes to the pathogenesis of preeclampsia and stabilizes placental angiotensin receptor type 1 by ubiquitination-preventing isopeptide modification.	Pubmed	0.0044	65.1	0.0114	0.116	1	1	TGM2
Pubmed	22465688	Iloperidone (Fanapt®), a novel atypical antipsychotic, is a potent HERG blocker and delays cardiac ventricular repolarization at clinically relevant concentration.	GeneRIF	0.0044	65.1	0.0114	0.116	1	1	KCNH2
Pubmed	17763876	HERG1 currents in native K562 leukemic cells.	GeneRIF	0.0044	65.1	0.0114	0.116	1	1	KCNH2
Pubmed	20054820	Inhibition of p21-activated kinase 6 (PAK6) increases radiosensitivity of prostate cancer cells.	Pubmed	0.0044	65.1	0.0114	0.116	1	1	PAK6
Pubmed	3679210	Orosomucoid (ORM) typing by isoelectric focusing: evidence for gene duplication of ORM1 and genetic polymorphism of ORM2.	Pubmed	0.0044	65.1	0.0114	0.116	1	1	ORM2
Pubmed	12193960	Association between an atrial natriuretic peptide gene polymorphism and normal blood pressure in subjects of African ancestry.	GeneRIF	0.0044	65.1	0.0114	0.116	1	1	NPPA
Pubmed	25648353	Role of mutation and pharmacologic block of human KCNH2 in vasculogenesis and fetal mortality: partial rescue by transforming growth factor-ß.	Pubmed	0.0044	65.1	0.0114	0.116	1	1	KCNH2
Pubmed	15331667	Zinc metalloproteinase-mediated cleavage of the human Nogo-66 receptor.	GeneRIF	0.0044	65.1	0.0114	0.116	1	1	RTN4R
Pubmed	21099262	Physiological significance of SK4 channels in pancreatic ß-cell oscillations.	GeneRIF	0.0044	65.1	0.0114	0.116	1	1	KCNN4
Pubmed	18302245	Clinical variability of autosomal dominant cataract, microcornea and corneal opacity and novel mutation in the alpha A crystallin gene (CRYAA).	GeneRIF	0.0044	65.1	0.0114	0.116	1	1	CRYAA
Pubmed	17976575	Silencing of herg gene by shRNA inhibits SH-SY5Y cell growth in vitro and in vivo.	GeneRIF	0.0044	65.1	0.0114	0.116	1	1	KCNH2
Pubmed	11686350	A 23bp insertion in the endothelial protein C receptor (EPCR) gene impairs EPCR function.	GeneRIF	0.0044	65.1	0.0114	0.116	1	1	PROCR
Pubmed	15849356	Identification of a novel recognition sequence for fibronectin within the NH2-terminal beta-sandwich domain of tissue transglutaminase.	GeneRIF	0.0044	65.1	0.0114	0.116	1	1	TGM2
Pubmed	3048308	The precursor of sulfatide activator protein is processed to three different proteins.	Pubmed	0.0044	65.1	0.0114	0.116	1	1	PSAP
Pubmed	11939509	Hb Vila Real [beta36(C2)Pro-->His] in Italy: characterization of the amino acid substitution and the DNA mutation.	Pubmed	0.0044	65.1	0.0114	0.116	1	1	HBB
Pubmed	11156467	Effect of extracellular calcium on the gene expression of bone morphogenetic protein-2 and -4 of normal human bone cells.	Pubmed	0.0044	65.1	0.0114	0.116	1	1	BMP4
Pubmed	15866043	Anterograde axonal transport of BDNF and NT-3 by retinal ganglion cells: roles of neurotrophin receptors.	GeneRIF	0.0044	65.1	0.0114	0.116	1	1	NGFR
Pubmed	15618645	Inhibitory effects of alcohols on the activity of human matrix metalloproteinase 7 (matrilysin).	GeneRIF	0.0044	65.1	0.0114	0.116	1	1	MMP7
Pubmed	20849596	Increased serum levels of tumour-associated trypsin inhibitor independently predict a poor prognosis in colorectal cancer patients.	GeneRIF	0.0044	65.1	0.0114	0.116	1	1	SPINK1
Pubmed	17162989	A study of the diagnostic utility of HLA typing, CSF hypocretin-1 measurements, and MSLT testing for the diagnosis of narcolepsy in 163 Korean patients with unexplained excessive daytime sleepiness.	GeneRIF	0.0044	65.1	0.0114	0.116	1	1	HLA-DQB1
Pubmed	19798546	Association of HTRA1 promoter polymorphism with spinal disc degeneration in Japanese women.	GeneRIF	0.0044	65.1	0.0114	0.116	1	1	HTRA1
Pubmed	25263706	p28, a truncated form of TRa1 regulates mitochondrial physiology.	Pubmed	0.0044	65.1	0.0114	0.116	1	1	THRA
Pubmed	3356751	ERV3 human endogenous provirus mRNAs are expressed in normal and malignant tissues and cells, but not in choriocarcinoma tumor cells.	Pubmed	0.0044	65.1	0.0114	0.116	1	1	ERV3-1
Pubmed	21489024	Pharmacological inhibition of the hERG potassium channel is modulated by extracellular but not intracellular acidosis.	GeneRIF	0.0044	65.1	0.0114	0.116	1	1	KCNH2
Pubmed	2717620	Saposin A: second cerebrosidase activator protein.	Pubmed	0.0044	65.1	0.0114	0.116	1	1	PSAP
Pubmed	12453183	Gamma-glutamyl transpeptidase and its role in melanogenesis: redox reactions and regulation of tyrosinase.	GeneRIF	0.0044	65.1	0.0114	0.116	1	1	GGT1
Pubmed	19046947	Proteomic assessment of sympathetic ganglia from adult mice that possess null mutations of ExonIII or ExonIV in the p75 neurotrophin receptor gene.	GeneRIF	0.0044	65.1	0.0114	0.116	1	1	NGFR
Pubmed	2961612	Expression of pancreatic secretory trypsin inhibitor gene in neoplastic tissues.	Pubmed	0.0044	65.1	0.0114	0.116	1	1	SPINK1
Pubmed	25129277	The Anp genetic variant Rs5068 and circulating levels of natriuretic peptides in patients with chronic heart failure.	Pubmed	0.0044	65.1	0.0114	0.116	1	1	NPPA
Pubmed	2918909	A pseudo-exon in the functional human alpha A-crystallin gene.	Pubmed	0.0044	65.1	0.0114	0.116	1	1	CRYAA

Category	ID	Name	Source	p-value	q-value Bonferroni	q-value FDR B&H	q-value FDR B&Y	Hit Count in Query List	Hit Count in Genome	Hit in Query List
Pubmed	15897971	Saposin C stimulates growth and invasion, activates p42/44 and SAPK/JNK signaling pathways of MAPK and upregulates uPA/uPAR expression in prostate cancer and stromal cells.	GeneRIF	0.0044	65.1	0.0114	0.116	1	1	PSAP
Pubmed	19634686	Activated protein C ameliorates TNF-alpha-induced inflammatory response of endothelium via the endothelial protein C receptor.	GeneRIF	0.0044	65.1	0.0114	0.116	1	1	PROCR
Pubmed	9410900	HLA-DQB1 polymorphism determines incidence, onset, and severity of collagen-induced arthritis in transgenic mice. Implications in human rheumatoid arthritis.	Pubmed	0.0044	65.1	0.0114	0.116	1	1	HLA-DQB1
Pubmed	11086042	Altered selection of CD4+ T cells by class II MHC bound with dominant and low abundance self-peptides.	Pubmed	0.0044	65.1	0.0114	0.116	1	1	HLA-DQB1
Pubmed	21474444	Demonstration of physical proximity between the N terminus and the S4-S5 linker of the human ether-a-go-go-related gene (hERG) potassium channel.	GeneRIF	0.0044	65.1	0.0114	0.116	1	1	KCNH2
Pubmed	24793009	CYP11A1 microsatellite (tttta)n polymorphism in PCOS women from South India.	Pubmed	0.0044	65.1	0.0114	0.116	1	1	CYP11A1
Pubmed	19070294	Identification of the gene causing long QT syndrome in an Israeli family.	GeneRIF	0.0044	65.1	0.0114	0.116	1	1	KCNH2
Pubmed	12359768	Matrilysin (matrix metalloproteinase-7) selects for apoptosis-resistant mammary cells in vivo.	GeneRIF	0.0044	65.1	0.0114	0.116	1	1	MMP7
Pubmed	24002652	Polyamines transduce the nongenomic, androgen-induced calcium sensitization in intestinal smooth muscle.	Pubmed	0.0044	65.1	0.0114	0.116	1	1	TGM2
Pubmed	23345332	Proteasomal degradation of herpes simplex virus capsids in macrophages releases DNA to the cytosol for recognition by DNA sensors.	GeneRIF	0.0044	65.1	0.0114	0.116	1	1	IFI16
Pubmed	16368554	The role of transglutaminase-2 and its substrates in human diseases.	GeneRIF	0.0044	65.1	0.0114	0.116	1	1	TGM2
Pubmed	10862094	Survey of the coding region of the HERG gene in long QT syndrome reveals six novel mutations and an amino acid polymorphism with possible phenotypic effects.	Pubmed	0.0044	65.1	0.0114	0.116	1	1	KCNH2
Pubmed	2079435	Hb Zengcheng or alpha 2 beta(2)114(G16)Leu----Met.	Pubmed	0.0044	65.1	0.0114	0.116	1	1	HBB
Pubmed	3629259	Identification of a novel thyroid hormone receptor expressed in the mammalian central nervous system.	Pubmed	0.0044	65.1	0.0114	0.116	1	1	THRA
Pubmed	10862104	New missense mutation (G626V) in the predicted selectivity filter of the HERG channel associated with familial long QT syndrome.	Pubmed	0.0044	65.1	0.0114	0.116	1	1	KCNH2
Pubmed	11925859	[Correlation between expression of MMP-7 in gastric submucosal invasive carcinoma and lymph node micrometastasis].	GeneRIF	0.0044	65.1	0.0114	0.116	1	1	MMP7
Pubmed	16452662	Alzheimer precursor protein interaction with the Nogo-66 receptor reduces amyloid-beta plaque deposition.	Pubmed	0.0044	65.1	0.0114	0.116	1	1	RTN4R
Pubmed	23794283	GPNMB enhances bone regeneration by promoting angiogenesis and osteogenesis: potential role for tissue engineering bone.	Pubmed	0.0044	65.1	0.0114	0.116	1	1	GPNMB
Pubmed	17032915	Donor-derived thymic-dependent T cells cause chronic graft-versus-host disease.	Pubmed	0.0044	65.1	0.0114	0.116	1	1	HLA-DQB1
Pubmed	2430282	Early regions of JC virus and BK virus induce distinct and tissue-specific tumors in transgenic mice.	Pubmed	0.0044	65.1	0.0114	0.116	1	1	SPINK1
Pubmed	3008331	Gene transfer and molecular cloning of the human NGF receptor.	Pubmed	0.0044	65.1	0.0114	0.116	1	1	NGFR
Pubmed	22505514	Kallikrein-related peptidases in prostate, breast, and ovarian cancers: from pathobiology to clinical relevance.	Pubmed	0.0044	65.1	0.0114	0.116	1	1	KLK15
Pubmed	8034709	Cloning, expression, and characterization of stratum corneum chymotryptic enzyme. A skin-specific human serine proteinase.	Pubmed	0.0044	65.1	0.0114	0.116	1	1	KLK7
Pubmed	18796164	Somatic mutation analysis of MYH11 in breast and prostate cancer.	GeneRIF	0.0044	65.1	0.0114	0.116	1	1	MYH11
Pubmed	9765245	A K+ channel splice variant common in human heart lacks a C-terminal domain required for expression of rapidly activating delayed rectifier current.	Pubmed	0.0044	65.1	0.0114	0.116	1	1	KCNH2
Pubmed	21389974	Hematopoietic growth factor inducible neurokinin-1 (Gpnmb/Osteoactivin) is a biomarker of progressive renal injury across species.	GeneRIF	0.0044	65.1	0.0114	0.116	1	1	GPNMB
Pubmed	24470490	Aromatic-aromatic interactions between residues in KCa3.1 pore helix and S5 transmembrane segment control the channel gating process.	Pubmed	0.0044	65.1	0.0114	0.116	1	1	KCNN4
Pubmed	22699877	Association between polymorphisms of the CYP11A1 gene and polycystic ovary syndrome in Chinese women.	GeneRIF	0.0044	65.1	0.0114	0.116	1	1	CYP11A1
Pubmed	9000171	An intron 1 regulatory region from the murine adenosine deaminase gene can activate heterologous promoters for ubiquitous expression in transgenic mice.	Pubmed	0.0044	65.1	0.0114	0.116	1	1	ADA
Pubmed	22220479	Transglutaminase-mediated remodeling of the human erythrocyte membrane skeleton: relevance for erythrocyte diseases with shortened cell lifespan.	GeneRIF	0.0044	65.1	0.0114	0.116	1	1	TGM2
Pubmed	22350746	CD99-dependent expansion of myeloid-derived suppressor cells and attenuation of graft-versus-host disease.	Pubmed	0.0044	65.1	0.0114	0.116	1	1	HLA-DQB1
Pubmed	23626735	Identification of the control region of pancreatic expression of Bmp4 in vitro and in vivo.	Pubmed	0.0044	65.1	0.0114	0.116	1	1	BMP4
Pubmed	17145868	Matrilysin 1 influences colon carcinoma cell migration by cleavage of the laminin-5 beta3 chain.	GeneRIF	0.0044	65.1	0.0114	0.116	1	1	MMP7
Pubmed	18983539	Gpnmb is a melanoblast-expressed, MITF-dependent gene.	GeneRIF	0.0044	65.1	0.0114	0.116	1	1	GPNMB
Pubmed	22124116	hERG1a N-terminal eag domain-containing polypeptides regulate homomeric hERG1b and heteromeric hERG1a/hERG1b channels: a possible mechanism for long QT syndrome.	GeneRIF	0.0044	65.1	0.0114	0.116	1	1	KCNH2
Pubmed	18785187	Decreased muscle atrophy F-box (MAFbx) expression in regenerating muscle after muscle-damaging exercise.	GeneRIF	0.0044	65.1	0.0114	0.116	1	1	FBXO32

Category	ID	Name	Source	p-value	q-value Bonferroni	q-value FDR B&H	q-value FDR B&Y	Hit Count in Query List	Hit Count in Genome	Hit in Query List
Pubmed	2110056	A low-molecular-mass Kazal-type protease inhibitor isolated from rat hepatocytes is identical to rat pancreatic secretory trypsin inhibitor II. Purification and amino acid sequence.	Pubmed	0.0044	65.1	0.0114	0.116	1	1	SPINK1
Pubmed	22842973	Peroxidasin forms sulfilimine chemical bonds using hypohalous acids in tissue genesis.	Pubmed	0.0044	65.1	0.0114	0.116	1	1	PXDN
Pubmed	7911422	Assessment of alloreactive T cell subpopulations of aged mice in vivo. CD4+ but not CD8+ T cell-mediated rejection response declines with advanced age.	Pubmed	0.0044	65.1	0.0114	0.116	1	1	HLA-DQB1
Pubmed	23593394	Endothelial protein C receptor function in murine and human breast cancer development.	Pubmed	0.0044	65.1	0.0114	0.116	1	1	PROCR
Pubmed	19410023	Mustn1 is expressed during chondrogenesis and is necessary for chondrocyte proliferation and differentiation in vitro.	GeneRIF	0.0044	65.1	0.0114	0.116	1	1	MUSTN1
Pubmed	20817787	Group I p21-activated kinases regulate thyroid cancer cell migration and are overexpressed and activated in thyroid cancer invasion.	Pubmed	0.0044	65.1	0.0114	0.116	1	1	PAK6
Pubmed	21359089	Effects of the adenosine deaminase polymorphism and caffeine intake on sleep parameters in a large population sample.	GeneRIF	0.0044	65.1	0.0114	0.116	1	1	ADA
Pubmed	18174359	Mutation of mouse Cyp11a1 promoter caused tissue-specific reduction of gene expression and blunted stress response without affecting reproduction.	GeneRIF	0.0044	65.1	0.0114	0.116	1	1	CYP11A1
Pubmed	22508321	Tumour-associated trypsin inhibitor TATI is a prognostic marker in colorectal cancer.	GeneRIF	0.0044	65.1	0.0114	0.116	1	1	SPINK1
Pubmed	16331959	Temperature-induced molten globule-like state in human alpha1-acid glycoprotein: an infrared spectroscopic study.	GeneRIF	0.0044	65.1	0.0114	0.116	1	1	ORM2
Pubmed	12165562	Defective trafficking and cell death is characteristic of skin disease-associated connexin 31 mutations.	GeneRIF	0.0044	65.1	0.0114	0.116	1	1	GJB3
Pubmed	16803516	Association between HLA-DQB1 and cervical dysplasia in Vietnamese women.	GeneRIF	0.0044	65.1	0.0114	0.116	1	1	HLA-DQB1
Pubmed	24392001	The K+ channel KCa3.1 as a novel target for idiopathic pulmonary fibrosis.	Pubmed	0.0044	65.1	0.0114	0.116	1	1	KCNN4
Pubmed	15557215	Pattern of expression of HtrA1 during mouse development.	Pubmed	0.0044	65.1	0.0114	0.116	1	1	HTRA1
Pubmed	12757179	Distribution of HLA class I alleles differs in celiac disease patients according to age of onset.	GeneRIF	0.0044	65.1	0.0114	0.116	1	1	HLA-DQB1
Pubmed	20448140	A new amyloidosis caused by fibrillar aggregates of mutated corneodesmosin.	GeneRIF	0.0044	65.1	0.0114	0.116	1	1	CDSN
Pubmed	24957146	The endothelial protein C receptor enhances hemostasis of FVIIa administration in hemophilic mice in vivo.	Pubmed	0.0044	65.1	0.0114	0.116	1	1	PROCR
Pubmed	17520268	Thyroid hormone receptor alpha1 is a critical regulator for the expression of ion channels during final differentiation of outer hair cells.	GeneRIF	0.0044	65.1	0.0114	0.116	1	1	THRA
Pubmed	22719194	Role of ascites adenosine deaminase in differentiating between tuberculous peritonitis and peritoneal carcinomatosis.	GeneRIF	0.0044	65.1	0.0114	0.116	1	1	ADA
Pubmed	8031011	An Asp8Asn substitution results in the adenosine deaminase (ADA) genetic polymorphism (ADA 2 allozyme): occurrence on different chromosomal backgrounds and apparent intragenic crossover.	Pubmed	0.0044	65.1	0.0114	0.116	1	1	ADA
Pubmed	19632032	Transglutaminase 2 expression levels regulate sensitivity to cystamine plus TRAIL-mediated apoptosis.	GeneRIF	0.0044	65.1	0.0114	0.116	1	1	TGM2
Pubmed	17442483	AKAP12 induces apoptotic cell death in human fibrosarcoma cells by regulating CDKI-cyclin D1 and caspase-3 activity.	GeneRIF	0.0044	65.1	0.0114	0.116	1	1	AKAP12
Pubmed	2492095	Complete sequence of the murine invariant chain (Ii) gene.	Pubmed	0.0044	65.1	0.0114	0.116	1	1	CD74
Pubmed	1269836	Undersulfated chondroitin sulfate in the cartilage matrix of brachymorphic mice.	Pubmed	0.0044	65.1	0.0114	0.116	1	1	PAPSS2
Pubmed	15858067	Involvement of a Rac activator,P-Rex1, in neurotrophin-derived signaling and neuronal migration.	Pubmed	0.0044	65.1	0.0114	0.116	1	1	PREX1
Pubmed	9006104	Structural characterization by mass spectrometry o hemoglobin adducts formed after in vivo exposure to methyl bromide.	Pubmed	0.0044	65.1	0.0114	0.116	1	1	HBB
Pubmed	25123263	Matrix metalloproteinase-7 mRNA and protein expression in gastric carcinoma: a meta-analysis.	Pubmed	0.0044	65.1	0.0114	0.116	1	1	MMP7
Pubmed	21461854	Serum adenosine deaminase may predict disease activity in rheumatoid arthritis.	GeneRIF	0.0044	65.1	0.0114	0.116	1	1	ADA
Pubmed	12722832	Schwann cell apoptosis in the postnatal axotomized sciatic nerve is mediated via NGF through the low-affinity neurotrophin receptor.	GeneRIF	0.0044	65.1	0.0114	0.116	1	1	NGFR
Pubmed	15954900	Shedding of endothelial protein C receptor contributes to vasculopathy and renal injury in lupus: in vivo and in vitro evidence.	GeneRIF	0.0044	65.1	0.0114	0.116	1	1	PROCR
Pubmed	25527773	Knockdown of angiopoietin like-2 protects against angiotensin II-induced cerebral endothelial dysfunction in mice.	Pubmed	0.0044	65.1	0.0114	0.116	1	1	ANGPTL2
Pubmed	20085808	Oral cancer, cigarette smoke and mitochondrial 18kDa translocator protein (TSPO) - In vitro, in vivo, salivary analysis.	GeneRIF	0.0044	65.1	0.0114	0.116	1	1	TSPO
Pubmed	7493033	Opitz syndrome is genetically heterogeneous, with one locus on Xp22, and a second locus on 22q11.2.	Pubmed	0.0044	65.1	0.0114	0.116	1	1	MID1
Pubmed	16274630	B7-H3: another molecule marker for Mo-DCs?	GeneRIF	0.0044	65.1	0.0114	0.116	1	1	CD276
Pubmed	6328658	Sequencing the erbA gene of avian erythroblastosis virus reveals a new type of oncogene.	Pubmed	0.0044	65.1	0.0114	0.116	1	1	THRA
Pubmed	11566180	ATRA-regulated Asb-2 gene induced in differentiation of HL-60 leukemia cells.	Pubmed	0.0044	65.1	0.0114	0.116	1	1	ASB2
Pubmed	12798296	The Opitz syndrome gene MID1 is essential for establishing asymmetric gene expression in Hensen's node.	GeneRIF	0.0044	65.1	0.0114	0.116	1	1	MID1
Pubmed	12603095	The Dutch IVS-I-116 (A --> G) (alpha2) thalassemia mutation induces Hb H inclusion bodies when found in combination with the -alpha3.7 deletion defect.	Pubmed	0.0044	65.1	0.0114	0.116	1	1	HBA2

Category	ID	Name	Source	p-value	q-value Bonferroni	q-value FDR B&H	q-value FDR B&Y	Hit Count in Query List	Hit Count in Genome	Hit in Query List
Pubmed	9349239	Imbalance of expression of matrix metalloproteinases (MMPs) and tissue inhibitors of the matrix metalloproteinases (TIMPs) in human pancreatic carcinoma.	Pubmed	0.0044	65.1	0.0114	0.116	1	1	MMP7
Pubmed	17603629	p75 neurotrophin receptor suppresses the proliferation of human gastric cancer cells.	GeneRIF	0.0044	65.1	0.0114	0.116	1	1	NGFR
Pubmed	11912282	Regulation of natural antibiotic expression by inflammatory mediators and mimics of infection in human endometrial epithelial cells.	Pubmed	0.0044	65.1	0.0114	0.116	1	1	SLPI
Pubmed	24205828	Virus-encoded ectopic CD74 enhances poxvirus vaccine efficacy.	Pubmed	0.0044	65.1	0.0114	0.116	1	1	CD74
Pubmed	19330901	Association of 77 polymorphisms in 52 candidate genes with blood pressure progression and incident hypertension: the Women's Genome Health Study.	GeneRIF	0.0044	65.1	0.0114	0.116	1	1	NPPA
Pubmed	3718478	Haemoglobin binding with haptoglobin. Localization of the haptoglobin-binding sites on the beta-chain of human haemoglobin by synthetic overlapping peptides encompassing the entire chain.	Pubmed	0.0044	65.1	0.0114	0.116	1	1	HBB
Pubmed	12695533	Voltage-dependent profile of human ether-a-go-go-related gene channel block is influenced by a single residue in the S6 transmembrane domain.	GeneRIF	0.0044	65.1	0.0114	0.116	1	1	KCNH2
Pubmed	17157250	Histidine phosphorylation of the potassium channel KCa3.1 by nucleoside diphosphate kinase B is required for activation of KCa3.1 and CD4 T cells.	GeneRIF	0.0044	65.1	0.0114	0.116	1	1	KCNN4
Pubmed	19672693	Alternative RNA splicing in expression of the glutathione synthetase gene in human cells.	GeneRIF	0.0044	65.1	0.0114	0.116	1	1	GSS
Pubmed	12888625	Selective thyroid hormone receptor-beta activation: a strategy for reduction of weight, cholesterol, and lipoprotein (a) with reduced cardiovascular liability.	Pubmed	0.0044	65.1	0.0114	0.116	1	1	THRA
Pubmed	17602574	Characterization of the nitrosyl adduct of substrate-bound mouse cysteine dioxygenase by electron paramagnetic resonance: electronic structure of the active site and mechanistic implications.	GeneRIF	0.0044	65.1	0.0114	0.116	1	1	CDO1
Pubmed	12756220	Effects of pressure overload on extracellular matrix expression in the heart of the atrial natriuretic peptide-null mouse.	GeneRIF	0.0044	65.1	0.0114	0.116	1	1	NPPA
Pubmed	10614823	Mass spectrometric characterisation of post-translational modification and genetic variation in human tetranectin.	Pubmed	0.0044	65.1	0.0114	0.116	1	1	CLEC3B
Pubmed	23075452	Impact of endothelial lipase on cellular lipid composition.	GeneRIF	0.0044	65.1	0.0114	0.116	1	1	LIPG
Pubmed	25605802	BMP4 and BMP Antagonists Regulate Human White and Beige Adipogenesis.	Pubmed	0.0044	65.1	0.0114	0.116	1	1	BMP4
Pubmed	12630965	Mutation analysis of Connexin 31 (GJB3) in sporadic non-syndromic hearing impairment.	GeneRIF	0.0044	65.1	0.0114	0.116	1	1	GJB3
Pubmed	24123696	Brief report: Dclk1 deletion in tuft cells results in impaired epithelial repair after radiation injury.	Pubmed	0.0044	65.1	0.0114	0.116	1	1	DCLK1
Pubmed	11706124	Atrial natriuretic peptide gene G664A polymorphism and the risk of ischemic cerebrovascular disease.	GeneRIF	0.0044	65.1	0.0114	0.116	1	1	NPPA
Pubmed	23321458	Central nervous system expression and PET imaging of the translocator protein in relapsing-remitting experimental autoimmune encephalomyelitis.	GeneRIF	0.0044	65.1	0.0114	0.116	1	1	TSPO
Pubmed	15259020	Mice deficient in invariant-chain and MHC class II exhibit a normal mature B2 cell compartment.	Pubmed	0.0044	65.1	0.0114	0.116	1	1	CD74
Pubmed	9479000	The macrosialin promoter directs high levels of transcriptional activity in macrophages dependent on combinatorial interactions between PU.1 and c-Jun.	Pubmed	0.0044	65.1	0.0114	0.116	1	1	CD68
Pubmed	20548128	Adenosine deaminase activity in COPD patients and healthy subjects.	GeneRIF	0.0044	65.1	0.0114	0.116	1	1	ADA
Pubmed	11082042	Cathepsin K in thyroid epithelial cells: sequence, localization and possible function in extracellular proteolysis of thyroglobulin.	Pubmed	0.0044	65.1	0.0114	0.116	1	1	CTSK
Pubmed	12867537	The T111I mutation in the EL gene modulates the impact of dietary fat on the HDL profile in women.	GeneRIF	0.0044	65.1	0.0114	0.116	1	1	LIPG
Pubmed	12410803	Macrophages from high-risk HLA-DQB1*0201/*0302 type 1 diabetes mellitus patients are hypersensitive to lipopolysaccharide stimulation.	GeneRIF	0.0044	65.1	0.0114	0.116	1	1	HLA-DQB1
Pubmed	20375276	Ceramide modulates HERG potassium channel gating by translocation into lipid rafts.	GeneRIF	0.0044	65.1	0.0114	0.116	1	1	KCNH2
Pubmed	10318835	Cloning of a unique lipase from endothelial cells extends the lipase gene family.	Pubmed	0.0044	65.1	0.0114	0.116	1	1	LIPG
Pubmed	3442600	Complete amino-acid sequence and carbohydrate content of the naturally occurring glucosylceramide activator protein (A1 activator) absent from a new human Gaucher disease variant.	Pubmed	0.0044	65.1	0.0114	0.116	1	1	PSAP
Pubmed	11777929	Nerve growth factor-induced p75-mediated death of cultured hippocampal neurons is age-dependent and transduced through ceramide generated by neutral sphingomyelinase.	GeneRIF	0.0044	65.1	0.0114	0.116	1	1	NGFR
Pubmed	16537653	Polycystin-2 traffics to cilia independently of polycystin-1 by using an N-terminal RVxP motif.	Pubmed	0.0044	65.1	0.0114	0.116	1	1	PKD2L1
Pubmed	11733367	Cathepsin k is a critical protease in synovial fibroblast-mediated collagen degradation.	GeneRIF	0.0044	65.1	0.0114	0.116	1	1	CTSK
Pubmed	14980498	Cloning of murine early quiescence-1 gene: the murine counterpart of dermatopontin gene can induce and be induced by cell quiescence.	GeneRIF	0.0044	65.1	0.0114	0.116	1	1	DPT
Pubmed	24635948	Association of the endothelial protein C receptor (PROCR) rs867186-G allele with protection from severe malaria.	Pubmed	0.0044	65.1	0.0114	0.116	1	1	PROCR
Pubmed	3684612	Nucleotide sequence of cDNA encoding a novel human thyroid hormone receptor.	Pubmed	0.0044	65.1	0.0114	0.116	1	1	THRA
Pubmed	9209324	Human endogenous retroviruses: expression in various organs in vivo and its regulation in vitro.	Pubmed	0.0044	65.1	0.0114	0.116	1	1	ERV3-1
Pubmed	19139087	EHD3 regulates early-endosome-to-Golgi transport and preserves Golgi morphology.	GeneRIF	0.0044	65.1	0.0114	0.116	1	1	EHD3

Category	ID	Name	Source	p-value	q-value Bonferroni	q-value FDR B&H	q-value FDR B&Y	Hit Count in Query List	Hit Count in Genome	Hit in Query List
Pubmed	16446107	Three kinships with ALAS2 P520L (c. 1559 C --> T) mutation, two in association with severe iron overload, and one with sideroblastic anemia and severe iron overload.	GeneRIF	0.0044	65.1	0.0114	0.116	1	1	ALAS2
Pubmed	19249007	Mutations in BMP4 are associated with subepithelial, microform, and overt cleft lip.	GeneRIF	0.0044	65.1	0.0114	0.116	1	1	BMP4
Pubmed	3122219	Inhibins and activins: chemical properties and biological activity.	Pubmed	0.0044	65.1	0.0114	0.116	1	1	INHBB
Pubmed	8096464	Design of (omega-N-(O-acyl)hydroxy amid) aminodicarboxylic acid pyrrolidides as potent inhibitors of proline-specific peptidases.	Pubmed	0.0044	65.1	0.0114	0.116	1	1	DPP7
Pubmed	22923569	Cell biology. The unusual case of Porcupine.	Pubmed	0.0044	65.1	0.0114	0.116	1	1	PORCN
Pubmed	23765970	KLK1 A1789G gene polymorphism and the risk of coronary artery stenosis in the Chinese population.	Pubmed	0.0044	65.1	0.0114	0.116	1	1	KLK1
Pubmed	2934979	The pronatriodilatin gene is located on the distal short arm of human chromosome 1 and on mouse chromosome 4.	Pubmed	0.0044	65.1	0.0114	0.116	1	1	NPPA
Pubmed	20460154	Cell-autonomous alteration of dopaminergic transmission by wild type and mutant (DeltaE) TorsinA in transgenic mice.	Pubmed	0.0044	65.1	0.0114	0.116	1	1	NGFR
Pubmed	15033935	Atrial natriuretic peptide contributes to physiological control of lipid mobilization in humans.	GeneRIF	0.0044	65.1	0.0114	0.116	1	1	NPPA
Pubmed	9469614	Effects of facial nerve injury on mouse motoneurons lacking the p75 low-affinity neurotrophin receptor.	Pubmed	0.0044	65.1	0.0114	0.116	1	1	NGFR
Pubmed	24586593	Atrial natriuretic peptide and type 2 diabetes development--biomarker and genotype association study.	Pubmed	0.0044	65.1	0.0114	0.116	1	1	NPPA
Pubmed	24530480	Position of premature termination codons determines susceptibility of hERG mutations to nonsense-mediated mRNA decay in long QT syndrome.	Pubmed	0.0044	65.1	0.0114	0.116	1	1	KCNH2
Pubmed	16766578	Expression of intermediate-conductance, Ca2+-activated K+ channel (KCNN4) in H441 human distal airway epithelial cells.	GeneRIF	0.0044	65.1	0.0114	0.116	1	1	KCNN4
Pubmed	12664320	Dysferlin expression in tubular aggregates: their possible relationship to endoplasmic reticulum stress.	Pubmed	0.0044	65.1	0.0114	0.116	1	1	DYSF
Pubmed	23697974	Altered autophagy in the mice with a deficiency of saposin A and saposin B.	Pubmed	0.0044	65.1	0.0114	0.116	1	1	PSAP
Pubmed	24366185	Comparison of read-through effects of aminoglycosides and PTC124 on rescuing nonsense mutations of HERG gene associated with long QT syndrome.	Pubmed	0.0044	65.1	0.0114	0.116	1	1	KCNH2
Pubmed	9345904	PCP4 maps between D21S345 and P31P10SP6 on chromosome 21q22.2-->q22.3.	Pubmed	0.0044	65.1	0.0114	0.116	1	1	PCP4
Pubmed	12779276	Identification of alpha-thalassemia mutations in Iranian individuals with abnormal hematological indices and normal Hb A2.	GeneRIF	0.0044	65.1	0.0114	0.116	1	1	HBA2
Pubmed	22395501	Immunohistochemical expression of anti-CD68 antibody in atherosclerotic plaque.	GeneRIF	0.0044	65.1	0.0114	0.116	1	1	CD68
Pubmed	18798114	Mutation analysis of MYH11 in acute myeloid leukemia.	GeneRIF	0.0044	65.1	0.0114	0.116	1	1	MYH11
Pubmed	12034704	The crystal structure of the endothelial protein C receptor and a bound phospholipid.	Pubmed	0.0044	65.1	0.0114	0.116	1	1	PROCR
Pubmed	18308336	Universal metastability of sickle hemoglobin polymerization.	GeneRIF	0.0044	65.1	0.0114	0.116	1	1	HBB
Pubmed	19470753	Serine protease HtrA1 associates with microtubules and inhibits cell migration.	GeneRIF	0.0044	65.1	0.0114	0.116	1	1	HTRA1
Pubmed	22370591	Relationship between status of plasma atrial natriuretic peptide and heart rate variability in human subjects.	Pubmed	0.0044	65.1	0.0114	0.116	1	1	NPPA
Pubmed	17567581	Structural and functional characterization of a secreted hookworm Macrophage Migration Inhibitory Factor (MIF) that interacts with the human MIF receptor CD74.	GeneRIF	0.0044	65.1	0.0114	0.116	1	1	CD74
Pubmed	16721833	Natriuretic peptide Val7Met substitution and risk of coronary artery disease in Greek patients with familial hypercholesterolemia.	GeneRIF	0.0044	65.1	0.0114	0.116	1	1	NPPA
Pubmed	19851523	Patients with insulin-dependent diabetes or coronary heart disease following rehabilitation express serum fractalkine levels similar to those in healthy control subjects.	GeneRIF	0.0044	65.1	0.0114	0.116	1	1	CX3CL1
Pubmed	16434994	Expression of human Kallikrein 14 (KLK14) in breast cancer is associated with higher tumour grades and positive nodal status.	GeneRIF	0.0044	65.1	0.0114	0.116	1	1	KLK14
Pubmed	17110619	Identification of human oral keratinocyte stem/progenitor cells by neurotrophin receptor p75 and the role of neurotrophin/p75 signaling.	GeneRIF	0.0044	65.1	0.0114	0.116	1	1	NGFR
Pubmed	16199858	Genome architecture of the human beta-globin locus affects developmental regulation of gene expression.	GeneRIF	0.0044	65.1	0.0114	0.116	1	1	HBB
Pubmed	12360464	SPINK1/PSTI mutations are associated with tropical pancreatitis and type II diabetes mellitus in Bangladesh.	GeneRIF	0.0044	65.1	0.0114	0.116	1	1	SPINK1
Pubmed	9678511	Pathological study of mice with total deficiency of sphingolipid activator proteins (SAP knockout mice).	Pubmed	0.0044	65.1	0.0114	0.116	1	1	PSAP
Pubmed	10192396	A novel endothelial-derived lipase that modulates HDL metabolism.	Pubmed	0.0044	65.1	0.0114	0.116	1	1	LIPG
Pubmed	17244620	Both the N-terminal loop and wing W2 of the forkhead domain of transcription factor Foxo4 are important for DNA binding.	GeneRIF	0.0044	65.1	0.0114	0.116	1	1	FOXO4
Pubmed	24342995	Mechanisms with clinical implications for atrial fibrillation-associated remodeling: cathepsin K expression, regulation, and therapeutic target and biomarker.	Pubmed	0.0044	65.1	0.0114	0.116	1	1	CTSK
Pubmed	12082507	Foetal haemoglobin in normal healthy adults: relationship with polymorphic sequences cis to the beta globin gene.	GeneRIF	0.0044	65.1	0.0114	0.116	1	1	HBB
Pubmed	15232296	Expression of the sodium channel beta3 subunit in injured human sensory neurons.	Pubmed	0.0044	65.1	0.0114	0.116	1	1	SCN3B
Pubmed	23977080	Carboxypeptidase E/NFa1: a new neurotrophic factor against oxidative stress-induced apoptotic cell death mediated by ERK and PI3-K/AKT pathways.	Pubmed	0.0044	65.1	0.0114	0.116	1	1	CPE
Pubmed	19676133	A novel nonsynonymous variant of matrix metalloproteinase-7 confers risk of liver cirrhosis.	GeneRIF	0.0044	65.1	0.0114	0.116	1	1	MMP7

Category	ID	Name	Source	p-value	q-value Bonferroni	q-value FDR B&H	q-value FDR B&Y	Hit Count in Query List	Hit Count in Genome	Hit in Query List
Pubmed	22220477	Transglutaminase 2 dysfunctions in the development of autoimmune disorders: celiac disease and TG2-/- mouse.	GeneRIF	0.0044	65.1	0.0114	0.116	1	1	TGM2
Pubmed	20574037	Redefining dysferlinopathy phenotypes based on clinical findings and muscle imaging studies.	GeneRIF	0.0044	65.1	0.0114	0.116	1	1	DYSF
Pubmed	15691824	External GTP-bound transglutaminase 2 is a molecular switch for chondrocyte hypertrophic differentiation and calcification.	GeneRIF	0.0044	65.1	0.0114	0.116	1	1	TGM2
Pubmed	23055945	Calpain-5 mutations cause autoimmune uveitis, retinal neovascularization, and photoreceptor degeneration.	GeneRIF	0.0044	65.1	0.0114	0.116	1	1	CAPN5
Pubmed	12123595	Rac activation: P-Rex1 - a convergence point for PIP(3) and Gbetagamma?	GeneRIF	0.0044	65.1	0.0114	0.116	1	1	PREX1
Pubmed	11042591	Absence of p75(NTR) expression reduces nerve growth factor immunolocalization in cholinergic septal neurons.	Pubmed	0.0044	65.1	0.0114	0.116	1	1	NGFR
Pubmed	10987144	Human pyridoxal kinase: overexpression and properties of the recombinant enzyme.	Pubmed	0.0044	65.1	0.0114	0.116	1	1	PDXK
Pubmed	3501289	Primary structure of human pancreatic secretory trypsin inhibitor (PSTI) gene.	Pubmed	0.0044	65.1	0.0114	0.116	1	1	SPINK1
Pubmed	19332483	Loss of functional K+ channels encoded by ether-à-go-go-related genes in mouse myometrium prior to labour onset.	GeneRIF	0.0044	65.1	0.0114	0.116	1	1	KCNH2
Pubmed	23083999	Alterations of maternal plasma HTRA1 level in preeclampsia complicated by IUGR.	GeneRIF	0.0044	65.1	0.0114	0.116	1	1	HTRA1
Pubmed	10844560	S gene (Corneodesmosin) diversity and its relationship to psoriasis; high content of cSNP in the HLA-linked S gene.	Pubmed	0.0044	65.1	0.0114	0.116	1	1	CDSN
Pubmed	23853482	Transglutaminase 2 expression predicts progression free survival in non-small cell lung cancer patients treated with epidermal growth factor receptor tyrosine kinase inhibitor.	Pubmed	0.0044	65.1	0.0114	0.116	1	1	TGM2
Pubmed	20798489	Secondary erythrocytosis caused by hemoglobin Tak/(dß)0-thalassemia syndrome.	GeneRIF	0.0044	65.1	0.0114	0.116	1	1	HBB
Pubmed	20584617	p75NTR is an obligate signaling receptor required for cues that cause sympathetic neuron growth cone collapse.	GeneRIF	0.0044	65.1	0.0114	0.116	1	1	NGFR
Pubmed	23973696	Mutation E169K in junctophilin-2 causes atrial fibrillation due to impaired RyR2 stabilization.	Pubmed	0.0044	65.1	0.0114	0.116	1	1	JPH2
Pubmed	19320245	Perinatal zidovudine prophylaxis in HIV type-1-infected pregnant women with thalassaemia carriage in Thailand.	GeneRIF	0.0044	65.1	0.0114	0.116	1	1	HBB
Pubmed	22498419	Association between gamma-glutamyltransferase and coronary artery calcification.	Pubmed	0.0044	65.1	0.0114	0.116	1	1	GGT1
Pubmed	21801233	The ß-globin promoter -71 C>T mutation is a ß+ thalassemic allele.	GeneRIF	0.0044	65.1	0.0114	0.116	1	1	HBB
Pubmed	16764871	A microsatellite polymorphism (tttta)n in the promoter of the CYP11a gene in Chinese women with polycystic ovary syndrome.	GeneRIF	0.0044	65.1	0.0114	0.116	1	1	CYP11A1
Pubmed	24521862	Generation and analysis of serine protease inhibitor kazal type 3-cre driver mice.	Pubmed	0.0044	65.1	0.0114	0.116	1	1	SPINK1
Pubmed	21061147	Effect of methylglyoxal modification of human a-crystallin on the structure, stability and chaperone function.	GeneRIF	0.0044	65.1	0.0114	0.116	1	1	CRYAA
Pubmed	20113295	Characterization of a novel deletion causing beta-thalassemia major in an Afghan family.	GeneRIF	0.0044	65.1	0.0114	0.116	1	1	HBB
Pubmed	11691846	A short pseudoautosomal region in laboratory mice.	Pubmed	0.0044	65.1	0.0114	0.116	1	1	MID1
Pubmed	17075825	Increased serum levels of soluble fractalkine (CX3CL1) correlate with disease activity in rheumatoid vasculitis.	GeneRIF	0.0044	65.1	0.0114	0.116	1	1	CX3CL1
Pubmed	20544924	Novel diagnostic features of dysferlinopathies.	GeneRIF	0.0044	65.1	0.0114	0.116	1	1	DYSF
Pubmed	6434492	A silent hemoglobin variant detected by HPLC: hemoglobin City of Hope beta 69 (E13) Gly----Ser.	Pubmed	0.0044	65.1	0.0114	0.116	1	1	HBB
Pubmed	20180812	Matrix metalloproteinase-7 as a marker of metastasis and predictor of poor survival in bladder cancer.	GeneRIF	0.0044	65.1	0.0114	0.116	1	1	MMP7
Pubmed	22173492	Electrophysiological study of V535M hERG mutation of LQT2.	GeneRIF	0.0044	65.1	0.0114	0.116	1	1	KCNH2
Pubmed	10081983	Two hemoglobin variants with an alteration of the oxygen-linked chloride binding: Hb Antananarivo [alpha1(NA1)Val-->Gly] and Hb Barbizon [beta144(HC1)Lys-->Met].	Pubmed	0.0044	65.1	0.0114	0.116	1	1	HBA2
Pubmed	8921421	Comparison of thymocyte development in normal and invariant chain-deficient mice provides evidence that maturation-related changes in TCR and co-receptor levels play a critical role in cell fate.	Pubmed	0.0044	65.1	0.0114	0.116	1	1	CD74
Pubmed	20305663	Genetic variation in human HBB is associated with Plasmodium falciparum transmission.	GeneRIF	0.0044	65.1	0.0114	0.116	1	1	HBB
Pubmed	8043577	Solution conformation of an atrial natriuretic peptide variant selective for the type A receptor.	Pubmed	0.0044	65.1	0.0114	0.116	1	1	NPPA
Pubmed	22565177	Fortuitous description of hemoglobin Hope in a high-level Tunisian athlete: molecular diagnosis and origin.	GeneRIF	0.0044	65.1	0.0114	0.116	1	1	HBB
Pubmed	12356833	Enhanced C-terminal truncation of alphaA- and alphaB-crystallins in diabetic lenses.	Pubmed	0.0044	65.1	0.0114	0.116	1	1	CRYAA
Pubmed	20460579	Obese carboxypeptidase E knockout mice exhibit multiple defects in peptide hormone processing contributing to low bone mineral density.	GeneRIF	0.0044	65.1	0.0114	0.116	1	1	CPE
Pubmed	11827454	Expression of the mouse gap junction gene Gjb3 is regulated by distinct mechanisms in embryonic stem cells and keratinocytes.	GeneRIF	0.0044	65.1	0.0114	0.116	1	1	GJB3
Pubmed	24585442	A proinvasive role for the Ca(2+) -activated K(+) channel KCa3.1 in malignant glioma.	Pubmed	0.0044	65.1	0.0114	0.116	1	1	KCNN4
Pubmed	1917539	Hb Geelong [beta 139(H17)Asn----Asp].	Pubmed	0.0044	65.1	0.0114	0.116	1	1	HBB
Pubmed	12081494	Selective inhibition of the collagenolytic activity of human cathepsin K by altering its S2 subsite specificity.	GeneRIF	0.0044	65.1	0.0114	0.116	1	1	CTSK
Pubmed	11095909	Cataract development in gamma-glutamyl transpeptidase-deficient mice.	Pubmed	0.0044	65.1	0.0114	0.116	1	1	GGT1
Pubmed	14764704	Molecular characterization of human 4Ig-B7-H3, a member of the B7 family with four Ig-like domains.	Pubmed	0.0044	65.1	0.0114	0.116	1	1	CD276
Pubmed	16484589	Cell-surface CD74 initiates a signaling cascade leading to cell proliferation and survival.	Pubmed	0.0044	65.1	0.0114	0.116	1	1	CD74

Category	ID	Name	Source	p-value	q-value Bonferroni	q-value FDR B&H	q-value FDR B&Y	Hit Count in Query List	Hit Count in Genome	Hit in Query List
Pubmed	12082618	Expression of thyroid hormone receptor/erbA genes is altered in human breast cancer.	GeneRIF	0.0044	65.1	0.0114	0.116	1	1	THRA
Pubmed	23600595	A new ß(0)-thalassemia mutation (codon 102, AAC>ATCAC) in coexistence with a heterozygous P4.2 Nippon gene.	Pubmed	0.0044	65.1	0.0114	0.116	1	1	HBB
Pubmed	21221836	Intra-erythrocyte magnesium is associated with gamma-glutamyl transferase in obese children and adolescents.	GeneRIF	0.0044	65.1	0.0114	0.116	1	1	GGT1
Pubmed	19420749	Effect of claudin expression on paracellular permeability, migration and invasion of colonic cancer cells.	Pubmed	0.0044	65.1	0.0114	0.116	1	1	CLDN15
Pubmed	16025106	Activation of p75NTR by proBDNF facilitates hippocampal long-term depression.	GeneRIF	0.0044	65.1	0.0114	0.116	1	1	NGFR
Pubmed	17872380	The thyroid hormone receptor-alpha (TRalpha) gene encoding TRalpha1 controls deoxyribonuclei acid damage-induced tissue repair.	GeneRIF	0.0044	65.1	0.0114	0.116	1	1	THRA
Pubmed	20943106	Relative protection from ischaemic heart disease in beta-thalassaemia carriers.	GeneRIF	0.0044	65.1	0.0114	0.116	1	1	HBB
Pubmed	19563777	Aldo-keto reductase family 1 B10 protein detoxifies dietary and lipid-derived alpha, beta-unsaturated carbonyls at physiological levels.	GeneRIF	0.0044	65.1	0.0114	0.116	1	1	AKR1B10
Pubmed	21717271	Different doses of bone morphogenetic protein 4 promote the expression of early germ cell-specific gene in bone marrow mesenchymal stem cells.	GeneRIF	0.0044	65.1	0.0114	0.116	1	1	BMP4
Pubmed	24120603	Extramedullary haematopoiesis correlates with genotype and absence of cardiac iron overload in polytransfused adults with thalassaemia.	Pubmed	0.0044	65.1	0.0114	0.116	1	1	HBB
Pubmed	12054672	Dissection of promoter control modules that direct Bmp4 expression in the epithelium-derived components of hair follicles.	GeneRIF	0.0044	65.1	0.0114	0.116	1	1	BMP4
Pubmed	16132905	Is beta-thalassemia trait a protective factor against hypertension in young adults?	GeneRIF	0.0044	65.1	0.0114	0.116	1	1	HBB
Pubmed	23826747	The spectrum of ß-thalassemia mutations in Baghdad, Central Iraq.	Pubmed	0.0044	65.1	0.0114	0.116	1	1	HBB
Pubmed	23223129	Late-onset X-linked dominant protoporphyria: an etiology of photosensitivity in the elderly.	GeneRIF	0.0044	65.1	0.0114	0.116	1	1	ALAS2
Pubmed	25896020	Deficiency of cathepsin K prevents inflammation and bone erosion in rheumatoid arthritis and periodontitis and reveals its shared osteoimmune role.	Pubmed	0.0044	65.1	0.0114	0.116	1	1	CTSK
Pubmed	22170598	Bone morphogenetic protein-4 polymorphism and colorectal cancer risk: a meta analysis.	GeneRIF	0.0044	65.1	0.0114	0.116	1	1	BMP4
Pubmed	21277850	Tissue transglutaminase does not affect fibrotic matrix stability or regression of liver fibrosis in mice.	GeneRIF	0.0044	65.1	0.0114	0.116	1	1	TGM2
Pubmed	12102171	Atrial natriuretic peptide prohormone gene expression: hormones and diseases that upregulate its expression.	GeneRIF	0.0044	65.1	0.0114	0.116	1	1	NPPA
Pubmed	10833481	Constitutive and regulated secretion of secretory leukocyte proteinase inhibitor by human intestinal epithelial cells.	Pubmed	0.0044	65.1	0.0114	0.116	1	1	SLPI
Pubmed	21840883	Detrimental effect of fractalkine on myocardial ischaemia and heart failure.	GeneRIF	0.0044	65.1	0.0114	0.116	1	1	CX3CL1
Pubmed	23717683	Renal and urinary levels of endothelial protein C receptor correlate with acute renal allograft rejection.	Pubmed	0.0044	65.1	0.0114	0.116	1	1	PROCR
Pubmed	2686621	Cloning and expression of human salivary-gland kallikrein in Escherichia coli.	Pubmed	0.0044	65.1	0.0114	0.116	1	1	KLK1
Pubmed	15302166	Overexpression of HLA class I molecules on T cells among type 1 diabetes Brazilian patients.	GeneRIF	0.0044	65.1	0.0114	0.116	1	1	HLA-DQB1
Pubmed	23652531	Correlations between BMP-4 gene expression, heterotopic ossification and function after uncemented total hip replacement.	Pubmed	0.0044	65.1	0.0114	0.116	1	1	BMP4
Pubmed	25222044	ß-Thalassemia mutations in Western India: outcome of prenatal diagnosis in a hemoglobinopathies project.	Pubmed	0.0044	65.1	0.0114	0.116	1	1	HBB
Pubmed	20373350	Dysferlin overexpression in skeletal muscle produces a progressive myopathy.	GeneRIF	0.0044	65.1	0.0114	0.116	1	1	DYSF
Pubmed	24134756	Novel CTSK mutation resulting in an entire exon 2 skipping in a Thai girl with pycnodysostosis.	Pubmed	0.0044	65.1	0.0114	0.116	1	1	CTSK
Pubmed	8171392	Activation of apoptosis in early mouse embryos by 2'-deoxyadenosine exposure.	Pubmed	0.0044	65.1	0.0114	0.116	1	1	ADA
Pubmed	22028795	In silico analysis of single nucleotide polymorphism (SNPs) in human ß-globin gene.	GeneRIF	0.0044	65.1	0.0114	0.116	1	1	HBB
Pubmed	12062363	Characterization of a novel missense mutation E637K in the pore-S6 loop of HERG in a patient with long QT syndrome.	Pubmed	0.0044	65.1	0.0114	0.116	1	1	KCNH2
Pubmed	17230547	Cathepsin K and schizophrenia.	GeneRIF	0.0044	65.1	0.0114	0.116	1	1	CTSK
Pubmed	21868759	A role for bone morphogenetic protein-4 in lymph node vascular remodeling and primary tumor growth.	GeneRIF	0.0044	65.1	0.0114	0.116	1	1	BMP4
Pubmed	22363757	The potential role of ORM2 in the development of colorectal cancer.	GeneRIF	0.0044	65.1	0.0114	0.116	1	1	ORM2
Pubmed	8962141	Cell surface expression of mouse macrosialin and human CD68 and their role as macrophage receptors for oxidized low density lipoprotein.	Pubmed	0.0044	65.1	0.0114	0.116	1	1	CD68
Pubmed	19172259	Role of intracellular domains in the function of the herg potassium channel.	GeneRIF	0.0044	65.1	0.0114	0.116	1	1	KCNH2
Pubmed	2066109	The mechanism for a 33-nucleotide insertion in mRNA causing sphingolipid activator protein (SAP-1)-deficient metachromatic leukodystrophy.	Pubmed	0.0044	65.1	0.0114	0.116	1	1	PSAP
Pubmed	22484689	Inhibition of cathepsin K reduces cartilage degeneration in the anterior cruciate ligament transection rabbit and murine models of osteoarthritis.	Pubmed	0.0044	65.1	0.0114	0.116	1	1	CTSK
Pubmed	16920956	Murine Vbeta3+ and Vbeta7+ T cell subsets are specific targets for the HERV-K18 Env superantigen.	Pubmed	0.0044	65.1	0.0114	0.116	1	1	HLA-DQB1
Pubmed	23826326	A proline/arginine-rich end leucine-rich repeat protein (PRELP) variant is uniquely expressed in chronic lymphocytic leukemia cells.	Pubmed	0.0044	65.1	0.0114	0.116	1	1	PRELP
Pubmed	22348616	Role of translocator protein (18 kDa) in adult separation anxiety and attachment style in patients with depression.	GeneRIF	0.0044	65.1	0.0114	0.116	1	1	TSPO

Category	ID	Name	Source	p-value	q-value Bonferroni	q-value FDR B&H	q-value FDR B&Y	Hit Count in Query List	Hit Count in Genome	Hit in Query List
Pubmed	15699249	Novel mutation in the Per-Arnt-Sim domain of KCNH2 causes a malignant form of long-QT syndrome.	GeneRIF	0.0044	65.1	0.0114	0.116	1	1	KCNH2
Pubmed	10809227	Antiestrogens specifically up-regulate bone morphogenetic protein-4 promoter activity in human osteoblastic cells.	Pubmed	0.0044	65.1	0.0114	0.116	1	1	BMP4
Pubmed	24050898	The HLA class II allele DQB1 0309 is associated with dilated cardiomyopathy.	Pubmed	0.0044	65.1	0.0114	0.116	1	1	HLA-DQB1
Pubmed	11168679	The pathogenic activation of calpain: a marker and mediator of cellular toxicity and disease states.	Pubmed	0.0044	65.1	0.0114	0.116	1	1	CAPN5
Pubmed	17570372	Endothelial lipase is highly expressed in macrophages in advanced human atherosclerotic lesions.	GeneRIF	0.0044	65.1	0.0114	0.116	1	1	LIPG
Pubmed	24609271	Primary hepatocytes from mice lacking cysteine dioxygenase show increased cysteine concentrations and higher rates of metabolism of cysteine to hydrogen sulfide and thiosulfate.	Pubmed	0.0044	65.1	0.0114	0.116	1	1	CDO1
Pubmed	15389724	The sarcolemma in the Large(myd) mouse.	Pubmed	0.0044	65.1	0.0114	0.116	1	1	LARGE
Pubmed	20230792	Long bone osteoclasts display an augmented osteoclast phenotype compared to calvarial osteoclasts.	Pubmed	0.0044	65.1	0.0114	0.116	1	1	CTSK
Pubmed	21498529	The translocator protein.	GeneRIF	0.0044	65.1	0.0114	0.116	1	1	TSPO
Pubmed	1378867	Role of I-A molecules in early stages of B cell maturation.	Pubmed	0.0044	65.1	0.0114	0.116	1	1	HLA-DQB1
Pubmed	8645259	Genomic organization of the human bone morphogenetic protein-4 gene: molecular basis for multiple transcripts.	Pubmed	0.0044	65.1	0.0114	0.116	1	1	BMP4
Pubmed	20876384	Molecular determinants of human ether-à-go-go-related gene 1 (hERG1) K+ channel activation by NS1643.	GeneRIF	0.0044	65.1	0.0114	0.116	1	1	KCNH2
Pubmed	19413965	Interactions of H562 in the S5 helix with T618 and S621 in the pore helix are important determinants of hERG1 potassium channel structure and function.	GeneRIF	0.0044	65.1	0.0114	0.116	1	1	KCNH2
Pubmed	15522280	Role of a KCNH2 polymorphism (R1047 L) in dofetilide-induced Torsades de Pointes.	GeneRIF	0.0044	65.1	0.0114	0.116	1	1	KCNH2
Pubmed	18414926	Evidence of gene conversion in the evolutionary process of the codon 41/42 (-CTTT) mutation causing beta-thalassemia in southern China.	GeneRIF	0.0044	65.1	0.0114	0.116	1	1	HBB
Pubmed	11114299	Molecular cloning of a dendritic cell-associated transmembrane protein, DC-HIL, that promotes RGD-dependent adhesion of endothelial cells through recognition of heparan sulfate proteoglycans.	Pubmed	0.0044	65.1	0.0114	0.116	1	1	GPNMB
Pubmed	15858131	Postmortem molecular diagnosis of sickle beta thalassaemia.	Pubmed	0.0044	65.1	0.0114	0.116	1	1	HBB
Pubmed	25686905	Acquisition of doxorubicin resistance facilitates migrating and invasive potentials of gastric cancer MKN45 cells through up-regulating aldo-keto reductase 1B10.	Pubmed	0.0044	65.1	0.0114	0.116	1	1	AKR1B10
Pubmed	7070526	Globin structural mutant alpha 125Leu leads to Pro is a novel cause of alpha-thalassaemia.	Pubmed	0.0044	65.1	0.0114	0.116	1	1	HBA2
Pubmed	22916269	PAPSS2 promotes alkaline phosphates activity and mineralization of osteoblastic MC3T3-E1 cells by crosstalk and Smads signal pathways.	GeneRIF	0.0044	65.1	0.0114	0.116	1	1	PAPSS2
Pubmed	11158011	Possible human leukocyte antigen-mediated genetic interaction between type 1 and type 2 Diabetes.	GeneRIF	0.0044	65.1	0.0114	0.116	1	1	HLA-DQB1
Pubmed	3163150	Human urinary kallikrein--amino acid sequence and carbohydrate attachment sites.	Pubmed	0.0044	65.1	0.0114	0.116	1	1	KLK1
Pubmed	16144995	Dimethylarginine dimethylaminohydrolase overexpression suppresses graft coronary artery disease.	Pubmed	0.0044	65.1	0.0114	0.116	1	1	HLA-DQB1
Pubmed	14580149	Effect of hypoxia on the expression of fractalkine in human endothelial cells.	GeneRIF	0.0044	65.1	0.0114	0.116	1	1	CX3CL1
Pubmed	22877869	Cytochrome P450scc-dependent metabolism of 7-dehydrocholesterol in placenta and epidermal keratinocytes.	GeneRIF	0.0044	65.1	0.0114	0.116	1	1	CYP11A1
Pubmed	15661220	HLA-DQB1 and cervical cancer in Venezuelan women.	GeneRIF	0.0044	65.1	0.0114	0.116	1	1	HLA-DQB1
Pubmed	15652848	A novel missense mutation in a Japanese patient with gelatinous droplike corneal dystrophy.	GeneRIF	0.0044	65.1	0.0114	0.116	1	1	TACSTD2
Pubmed	18048364	Endoplasmic reticulum retention and rescue by heteromeric assembly regulate human ERG 1a/1b surface channel composition.	GeneRIF	0.0044	65.1	0.0114	0.116	1	1	KCNH2
Pubmed	17981573	Interferon-inducible IFI16 protein in human cancers and autoimmune diseases.	GeneRIF	0.0044	65.1	0.0114	0.116	1	1	IFI16
Pubmed	23875041	Overexpression of the endothelial protein C receptor is detrimental during pneumonia-derived gram-negative sepsis (Melioidosis).	Pubmed	0.0044	65.1	0.0114	0.116	1	1	PROCR
Pubmed	11231313	The level of prosaposin is decreased in the skin of patients with psoriasis vulgaris.	Pubmed	0.0044	65.1	0.0114	0.116	1	1	PSAP
Pubmed	15797245	Cathepsin X binds to cell surface heparan sulfate proteoglycans.	GeneRIF	0.0044	65.1	0.0114	0.116	1	1	CTSK
Pubmed	23376163	Identification and characterization of a 43 kDa actin protein involved in the DENV-2 binding and infection of ECV304 cells.	GeneRIF	0.0044	65.1	0.0114	0.116	1	1	ACTA1
Pubmed	15008265	Two new alpha chain variants: Hb Part-Dieu [alpha65(E14)Ala --> Thr (alpha2)] and Hb Decines-Charpieu [alpha69(E18)Ala --> Thr (alpha2)].	Pubmed	0.0044	65.1	0.0114	0.116	1	1	HBA2
Pubmed	12836053	Refined mapping of the Welander distal myopathy region on chromosome 2p13 positions the new candidate region telomeric of the DYSF locus.	GeneRIF	0.0044	65.1	0.0114	0.116	1	1	DYSF
Pubmed	8760210	Blood pressure and fluid-electrolyte balance in mice with reduced or absent ANP.	Pubmed	0.0044	65.1	0.0114	0.116	1	1	NPPA
Pubmed	15323426	[Expression of cytochrome P450scc in human early placenta].	GeneRIF	0.0044	65.1	0.0114	0.116	1	1	CYP11A1
Pubmed	10704052	Production of secretory leucocyte protease inhibitor (SLPI) in human pancreatic beta-cells.	Pubmed	0.0044	65.1	0.0114	0.116	1	1	SLPI
Pubmed	17190762	HLA-DQB1*0201 homozygosis predisposes to severe intestinal damage in celiac disease.	GeneRIF	0.0044	65.1	0.0114	0.116	1	1	HLA-DQB1
Pubmed	22569910	Cathepsin K preferentially solubilizes matured bone matrix.	GeneRIF	0.0044	65.1	0.0114	0.116	1	1	CTSK

Category	ID	Name	Source	p-value	q-value Bonferroni	q-value FDR B&H	q-value FDR B&Y	Hit Count in Query List	Hit Count in Genome	Hit in Query List
Pubmed	18462868	The inhibitory effect of p75 neurotrophin receptor on growth of human hepatocellular carcinoma cells.	GeneRIF	0.0044	65.1	0.0114	0.116	1	1	NGFR
Pubmed	16939368	A screening system for detecting genetic risk markers of type 1 diabetes in dried blood spots.	GeneRIF	0.0044	65.1	0.0114	0.116	1	1	HLA-DQB1
Pubmed	15978102	Autoantibodies against the endothelial receptor of protein C are associated with acute myocardial infarction in young women.	GeneRIF	0.0044	65.1	0.0114	0.116	1	1	PROCR
Pubmed	18420025	Secondary and tertiary structures of the transmembrane domains of the translocator protein TSPO determined by NMR. Stabilization of the TSPO tertiary fold upon ligand binding.	GeneRIF	0.0044	65.1	0.0114	0.116	1	1	TSPO
Pubmed	11962752	Tetranectin expression in gastric adenocarcinomas.	GeneRIF	0.0044	65.1	0.0114	0.116	1	1	CLEC3B
Pubmed	19385982	Hippocampal Nabeta3 expression in patients with temporal lobe epilepsy.	GeneRIF	0.0044	65.1	0.0114	0.116	1	1	SCN3B
Pubmed	24356113	IFI16 DNA sensor is required for death of lymphoid CD4 T cells abortively infected with HIV.	Pubmed	0.0044	65.1	0.0114	0.116	1	1	IFI16
Pubmed	24098680	Mice lacking the p43 mitochondrial T3 receptor become glucose intolerant and insulin resistant during aging.	Pubmed	0.0044	65.1	0.0114	0.116	1	1	THRA
Pubmed	19689997	Relation of soluble endothelial protein C receptor and cytokines after allogeneic hematopoietic stem cell transplantation.	GeneRIF	0.0044	65.1	0.0114	0.116	1	1	PROCR
Pubmed	12107443	Allelic and locus heterogeneity in autosomal recessive gelatinous drop-like corneal dystrophy.	GeneRIF	0.0044	65.1	0.0114	0.116	1	1	TACSTD2
Pubmed	18391202	Unregulated smooth-muscle myosin in human intestinal neoplasia.	GeneRIF	0.0044	65.1	0.0114	0.116	1	1	MYH11
Pubmed	17167402	A novel TACSTD2 gene mutation in a Turkish family with a gelatinous drop-like corneal dystrophy.	GeneRIF	0.0044	65.1	0.0114	0.116	1	1	TACSTD2
Pubmed	2896486	Renal gamma-glutamyl transpeptidases: structural and immunological studies.	Pubmed	0.0044	65.1	0.0114	0.116	1	1	GGT1
Pubmed	12959948	Time course of airway mechanics of the (+)insert myosin isoform knockout mouse.	Pubmed	0.0044	65.1	0.0114	0.116	1	1	MYH11
Pubmed	12408842	ProNGF induces p75-mediated death of oligodendrocytes following spinal cord injury.	Pubmed	0.0044	65.1	0.0114	0.116	1	1	NGFR
Pubmed	10858550	Cloning, expression and functional characterization of rat napsin.	Pubmed	0.0044	65.1	0.0114	0.116	1	1	NAPSA
Pubmed	19055866	Oxidative crosslinking, spectrin and membrane interactions of hemoglobin mixtures in HbEbeta-thalassemia.	GeneRIF	0.0044	65.1	0.0114	0.116	1	1	HBB
Pubmed	16522628	Mutation of a critical arginine in the GTP-binding site of transglutaminase 2 disinhibits intracellular cross-linking activity.	GeneRIF	0.0044	65.1	0.0114	0.116	1	1	TGM2
Pubmed	8811043	Tissue distribution and quantitation of a gene expressing a 64-kDa antigen associated with thyroid-associated ophthalmopathy.	Pubmed	0.0044	65.1	0.0114	0.116	1	1	LMOD1
Pubmed	10719280	Immunochemical characterization of assay for carboxyterminal telopeptide of human type I collagen: loss of antigenicity by treatment with cathepsin K.	Pubmed	0.0044	65.1	0.0114	0.116	1	1	CTSK
Pubmed	18501121	Association of human carboxypeptidase E exon5 gene polymorphisms with angiographical characteristics of coronary atherosclerosis in a Chinese population.	GeneRIF	0.0044	65.1	0.0114	0.116	1	1	CPE
Pubmed	9024663	A new class of membrane-bound chemokine with a CX3C motif.	Pubmed	0.0044	65.1	0.0114	0.116	1	1	CX3CL1
Pubmed	21820442	HIV Tat protein inhibits hERG K+ channels: a potential mechanism of HIV infection induced LQTs.	Pubmed	0.0044	65.1	0.0114	0.116	1	1	KCNH2
Pubmed	19048483	Regulation of human alpha-globin gene expression and alpha-thalassemia.	GeneRIF	0.0044	65.1	0.0114	0.116	1	1	HBA2
Pubmed	2013321	The organization of the gene for the human cerebroside sulfate activator protein.	Pubmed	0.0044	65.1	0.0114	0.116	1	1	PSAP
Pubmed	17582777	Analysis of alteration of p75NTR processing and signalling by PS2 mutation and gamma-secretase inhibition.	GeneRIF	0.0044	65.1	0.0114	0.116	1	1	NGFR
Pubmed	8810244	Requirement for invariant chain in B cell maturation and function.	Pubmed	0.0044	65.1	0.0114	0.116	1	1	CD74
Pubmed	24993425	Identification and functional characterization of the human ether-a-go-go-related gene Q738X mutant associated with hereditary long QT syndrome type 2.	Pubmed	0.0044	65.1	0.0114	0.116	1	1	KCNH2
Pubmed	11874483	Epidermal overexpression of stratum corneum chymotryptic enzyme in mice: a model for chronic itchy dermatitis.	Pubmed	0.0044	65.1	0.0114	0.116	1	1	KLK7
Pubmed	19750928	Narcolepsy with long sleep time: a specific entity?	GeneRIF	0.0044	65.1	0.0114	0.116	1	1	HLA-DQB1
Pubmed	17255476	A membrane receptor for retinol binding protein mediates cellular uptake of vitamin A.	Pubmed	0.0044	65.1	0.0114	0.116	1	1	STRA6
Pubmed	17509612	Mutations in JPH2-encoded junctophilin-2 associated with hypertrophic cardiomyopathy in humans.	GeneRIF	0.0044	65.1	0.0114	0.116	1	1	JPH2
Pubmed	6166590	Hemoglobin Brisbane: beta68 Leu replaced by His. A new high oxygen affinity variant.	Pubmed	0.0044	65.1	0.0114	0.116	1	1	HBB
Pubmed	15651049	1.70 A X-ray structure of human apo kallikrein 1: structural changes upon peptide inhibitor/substrate binding.	GeneRIF	0.0044	65.1	0.0114	0.116	1	1	KLK1
Pubmed	12569160	Endothelial lipase is a major determinant of HDL level.	GeneRIF	0.0044	65.1	0.0114	0.116	1	1	LIPG
Pubmed	24683193	Heterocomplex formation between MBL/ficolin/CL-11-associated serine protease-1 and -3 and MBL/ficolin/CL-11-associated protein-1.	Pubmed	0.0044	65.1	0.0114	0.116	1	1	MASP1
Pubmed	24368703	Inhibition of Notch signaling reduces the number of surviving Dclk1+ reserve crypt epithelial stem cells following radiation injury.	Pubmed	0.0044	65.1	0.0114	0.116	1	1	DCLK1
Pubmed	25531719	Overexpression of tumor-associated trypsin inhibitor (SPINK1/TATI) in hepatitis C-associated hepatocellular carcinoma: potential implications for viral hepatocarcinogenesis.	Pubmed	0.0044	65.1	0.0114	0.116	1	1	SPINK1
Pubmed	20082313	Dysferlin associates with the developing T-tubule system in rodent and human skeletal muscle.	GeneRIF	0.0044	65.1	0.0114	0.116	1	1	DYSF
Pubmed	25500991	Vascular function of the mesenteric artery isolated from thyroid hormone receptor-a knockout mice.	Pubmed	0.0044	65.1	0.0114	0.116	1	1	THRA

Category	ID	Name	Source	p-value	q-value Bonferroni	q-value FDR B&H	q-value FDR B&Y	Hit Count in Query List	Hit Count in Genome	Hit in Query List
Pubmed	23388709	Kaposi's sarcoma-associated herpesvirus latency in endothelial and B cells activates gamma interferon-inducible protein 16-mediated inflammasomes.	GeneRIF	0.0044	65.1	0.0114	0.116	1	1	IFI16
Pubmed	11043018	Genetic diversity of beta-thalassemia mutations in Pakistani population.	GeneRIF	0.0044	65.1	0.0114	0.116	1	1	HBB
Pubmed	20371597	Characterization of transglutaminase type II role in dendritic cell differentiation and function.	GeneRIF	0.0044	65.1	0.0114	0.116	1	1	TGM2
Pubmed	20543535	Phenotypic variability of the homozygous IVS3+2T>C mutation in the serine protease inhibitor Kazal type 1 (SPINK1) gene in patients with chronic pancreatitis.	GeneRIF	0.0044	65.1	0.0114	0.116	1	1	SPINK1
Pubmed	20129611	Plasma bilirubin and gamma-glutamyltransferase activity are inversely related in dyslipidemic patients with metabolic syndrome: relevance to oxidative stress.	GeneRIF	0.0044	65.1	0.0114	0.116	1	1	GGT1
Pubmed	2507633	The invariant chain is a phosphorylated subunit of class II molecules.	Pubmed	0.0044	65.1	0.0114	0.116	1	1	CD74
Pubmed	15365637	Activation of cardiac human ether-a-go-go related gene potassium currents is regulated by alpha(1A)-adrenoceptors.	GeneRIF	0.0044	65.1	0.0114	0.116	1	1	KCNH2
Pubmed	24351118	Severe a-thalassemia intermedia due to a compound heterozygosity for the highly unstable Hb Adana (HBA2: c.179G>A) and a novel codon 24 (HBA2: c.75T>A) mutation.	Pubmed	0.0044	65.1	0.0114	0.116	1	1	HBA2
Pubmed	25746564	Nuclear actin aggregation is a hallmark of anti-synthetase syndrome-induced dysimmune myopathy.	Pubmed	0.0044	65.1	0.0114	0.116	1	1	ACTA1
Pubmed	22368250	A possibility of a protein-bound water molecule as the ionizable group responsible for pKe at the alkaline side in human matrix metalloproteinase 7 activity.	GeneRIF	0.0044	65.1	0.0114	0.116	1	1	MMP7
Pubmed	19410631	Overexpression of the transcription factor Foxo4 is associated with rapid glucose clearance.	GeneRIF	0.0044	65.1	0.0114	0.116	1	1	FOXO4
Pubmed	24352879	A lack of secretory leukocyte protease inhibitor (SLPI) causes defects in granulocytic differentiation.	Pubmed	0.0044	65.1	0.0114	0.116	1	1	SLPI
Pubmed	12810961	Transglutaminase 2-/- mice reveal a phagocytosis-associated crosstalk between macrophages and apoptotic cells.	GeneRIF	0.0044	65.1	0.0114	0.116	1	1	TGM2
Pubmed	24223895	Zygotic Porcn paternal allele deletion in mice to model human focal dermal hypoplasia.	Pubmed	0.0044	65.1	0.0114	0.116	1	1	PORCN
Pubmed	18394312	CD 271 (P75 neurotrophin receptor).	GeneRIF	0.0044	65.1	0.0114	0.116	1	1	NGFR
Pubmed	9715271	The chimeric protein, PEBP2 beta/CBF beta-SMMHC, disorganizes cytoplasmic stress fibers and inhibits transcriptional activation.	Pubmed	0.0044	65.1	0.0114	0.116	1	1	MYH11
Pubmed	24762755	Factors influencing pleural adenosine deaminase level in patients with tuberculous pleurisy.	Pubmed	0.0044	65.1	0.0114	0.116	1	1	ADA
Pubmed	18391696	Correction of aberrant pre-mRNA splicing by antisense oligonucleotides in beta-thalassemia Egyptian patients with IVSI-110 mutation.	GeneRIF	0.0044	65.1	0.0114	0.116	1	1	HBB
Pubmed	19545604	Translocator protein (TSPO 18kDa) is expressed by neural stem and neuronal precursor cells.	GeneRIF	0.0044	65.1	0.0114	0.116	1	1	TSPO
Pubmed	19521708	Serum adenosine deaminase activity in gestational diabetes mellitus and normal pregnancy.	GeneRIF	0.0044	65.1	0.0114	0.116	1	1	ADA
Pubmed	21874302	The immune inhibitory receptor osteoactivin is upregulated in monocyte-derived dendritic cells by BCR-ABL tyrosine kinase inhibitors.	GeneRIF	0.0044	65.1	0.0114	0.116	1	1	GPNMB
Pubmed	8098731	Defective major histocompatibility complex class II assembly, transport, peptide acquisition, and CD4+ T cell selection in mice lacking invariant chain expression.	Pubmed	0.0044	65.1	0.0114	0.116	1	1	CD74
Pubmed	23085038	Transglutaminase 2 facilitates or ameliorates HIF signaling and ischemic cell death depending on its conformation and localization.	GeneRIF	0.0044	65.1	0.0114	0.116	1	1	TGM2
Pubmed	17254960	FoxOs in tumor suppression and stem cell maintenance.	Pubmed	0.0044	65.1	0.0114	0.116	1	1	FOXO4
Pubmed	19503809	Altered membrane structure and surface potential in homozygous hemoglobin C erythrocytes.	GeneRIF	0.0044	65.1	0.0114	0.116	1	1	HBB
Pubmed	17561806	Translocator protein (18 kDa) ligand PK 11195 induces transient mitochondrial Ca2+ release leading to transepithelial Cl- secretion in HT-29 human colon cancer cells.	GeneRIF	0.0044	65.1	0.0114	0.116	1	1	TSPO
Pubmed	20802163	Genetic variation in the glutathione synthesis pathway, air pollution, and children's lung function growth.	GeneRIF	0.0044	65.1	0.0114	0.116	1	1	GSS
Pubmed	9643675	Human pancreatic secretory trypsin inhibitor. Distribution, actions and possible role in mucosal integrity and repair.	Pubmed	0.0044	65.1	0.0114	0.116	1	1	SPINK1
Pubmed	19131591	Expression of CD74, the receptor for macrophage migration inhibitory factor, in non-small cell lung cancer.	GeneRIF	0.0044	65.1	0.0114	0.116	1	1	CD74
Pubmed	19562689	Mutations and polymorphisms of the skeletal muscle alpha-actin gene (ACTA1).	GeneRIF	0.0044	65.1	0.0114	0.116	1	1	ACTA1
Pubmed	12023766	Effect of menstrual status on antibacterial activity and secretory leukocyte protease inhibitor production by human uterine epithelial cells in culture.	GeneRIF	0.0044	65.1	0.0114	0.116	1	1	SLPI
Pubmed	17486367	The tetraspanin superfamily member NET-6 is a new tumor suppressor gene.	GeneRIF	0.0044	65.1	0.0114	0.116	1	1	TSPAN13
Pubmed	24930385	Expression and activity levels of matrix metalloproteinase-7 and in situ localization of caseinolytic activity in colorectal cancer.	Pubmed	0.0044	65.1	0.0114	0.116	1	1	MMP7
Pubmed	15317945	The immune regulatory protein B7-H3 promotes osteoblast differentiation and bone mineralization.	GeneRIF	0.0044	65.1	0.0114	0.116	1	1	CD276
Pubmed	17612588	Tissue transglutaminase (TG2) facilitates phosphatidylserine exposure and calpain activity in calcium-induced death of erythrocytes.	GeneRIF	0.0044	65.1	0.0114	0.116	1	1	TGM2
Pubmed	21123738	Calcium-activated K+ channels increase cell proliferation independent of K+ conductance.	GeneRIF	0.0044	65.1	0.0114	0.116	1	1	KCNN4

Category	ID	Name	Source	p-value	q-value Bonferroni	q-value FDR B&H	q-value FDR B&Y	Hit Count in Query List	Hit Count in Genome	Hit in Query List
Pubmed	19565042	Investigation of the SPINK1 N34S mutation in Romanian patients with alcoholic chronic pancreatitis. A clinical analysis based on the criteria of the M-ANNHEIM classification.	GeneRIF	0.0044	65.1	0.0114	0.116	1	1	SPINK1
Pubmed	8988543	Complete coding region of the new HLA-DQB1*0612 allele, obtained by RT-PCR.	Pubmed	0.0044	65.1	0.0114	0.116	1	1	HLA-DQB1
Pubmed	21219869	Enhanced inhibitory effect of acidosis on hERG potassium channels that incorporate the hERG1b isoform.	GeneRIF	0.0044	65.1	0.0114	0.116	1	1	KCNH2
Pubmed	8370464	Storage of saposins A and D in infantile neuronal ceroid-lipofuscinosis.	Pubmed	0.0044	65.1	0.0114	0.116	1	1	PSAP
Pubmed	20437615	CARASIL	Pubmed	0.0044	65.1	0.0114	0.116	1	1	HTRA1
Pubmed	19309688	PORCN mutations in focal dermal hypoplasia: coping with lethality.	GeneRIF	0.0044	65.1	0.0114	0.116	1	1	PORCN
Pubmed	21060151	Murine erythroid short-term radioprotection requires a BMP4-dependent, self-renewing population of stress erythroid progenitors.	GeneRIF	0.0044	65.1	0.0114	0.116	1	1	BMP4
Pubmed	3623975	Hb Olomouc or alpha 2 beta 2(86)(F2)Ala----Asp, a new high oxygen affinity variant.	Pubmed	0.0044	65.1	0.0114	0.116	1	1	HBB
Pubmed	22652528	Tissue transglutaminase (TG-2) modified amniotic membrane: a novel scaffold for biomedical applications.	GeneRIF	0.0044	65.1	0.0114	0.116	1	1	TGM2
Pubmed	23094635	Study of three families with Hb Agrinio [a29(B10)Leu¿Pro, CTG>CCG (a2)] in the Spanish population: three homozygous cases.	GeneRIF	0.0044	65.1	0.0114	0.116	1	1	HBA2
Pubmed	8690902	Phenotype and function of CD4+ T cells in mice lacking invariant chain.	Pubmed	0.0044	65.1	0.0114	0.116	1	1	CD74
Pubmed	393608	N-Terminal amino acid sequence of human urinary kallikrein homology with other serine proteases.	Pubmed	0.0044	65.1	0.0114	0.116	1	1	KLK1
Pubmed	21490315	Trafficking-deficient hERG K¿ channels linked to long QT syndrome are regulated by a microtubule-dependent quality control compartment in the ER.	GeneRIF	0.0044	65.1	0.0114	0.116	1	1	KCNH2
Pubmed	12595708	Purification and crystallization of the N-terminal domain from the human doublecortin-like kinase.	GeneRIF	0.0044	65.1	0.0114	0.116	1	1	DCLK1
Pubmed	8219185	A gene for a myosin peptide is disrupted by the inv(16)(p13q22) in acute nonlymphocytic leukemia M4Eo.	Pubmed	0.0044	65.1	0.0114	0.116	1	1	MYH11
Pubmed	12175552	Inhibition of cell death by ribosomal protein L35a.	GeneRIF	0.0044	65.1	0.0114	0.116	1	1	RPL35A
Pubmed	19278990	Transglutaminase-2 regulation by arecoline in gingival fibroblasts.	GeneRIF	0.0044	65.1	0.0114	0.116	1	1	TGM2
Pubmed	17114341	Inhibition of matrilysin expression by antisense or RNA interference decreases lysophosphatidic acid-induced epithelial ovarian cancer invasion.	GeneRIF	0.0044	65.1	0.0114	0.116	1	1	MMP7
Pubmed	24787022	B7-H3 was highly expressed in human primary hepatocellular carcinoma and promoted tumor progression.	Pubmed	0.0044	65.1	0.0114	0.116	1	1	CD276
Pubmed	10617859	Developmental patterns of zygotes from transgenic female mice with elevated tissue glutathione.	Pubmed	0.0044	65.1	0.0114	0.116	1	1	GSS
Pubmed	3399394	Organization and upstream DNA sequence of the mouse protease inhibitor gene.	Pubmed	0.0044	65.1	0.0114	0.116	1	1	SPINK1
Pubmed	16606933	Lariat branch point mutation in the dysferlin gene with mild limb-girdle muscular dystrophy.	GeneRIF	0.0044	65.1	0.0114	0.116	1	1	DYSF
Pubmed	23872121	Biochemical and preliminary X-ray characterization of the tumor-associated calcium signal transducer 2 (Trop2) ectodomain.	Pubmed	0.0044	65.1	0.0114	0.116	1	1	TACSTD2
Pubmed	21051573	Atrial natriuretic peptide protects against Staphylococcus aureus-induced lung injury and endothelial barrier dysfunction.	GeneRIF	0.0044	65.1	0.0114	0.116	1	1	NPPA
Pubmed	10533048	KIAA0369, doublecortin-like kinase, is expressed during brain development.	Pubmed	0.0044	65.1	0.0114	0.116	1	1	DCLK1
Pubmed	25250564	The association between HLA-DQB1 polymorphism and antituberculosis drug-induced liver injury: a Case-Control Study.	Pubmed	0.0044	65.1	0.0114	0.116	1	1	HLA-DQB1
Pubmed	20193381	[Study on the incidence of beta-Thalassemia and genotypes among children under 7 year-olds in Nanning, Liuzhou and Baise areas, Guangxi province].	GeneRIF	0.0044	65.1	0.0114	0.116	1	1	HBB
Pubmed	22700770	Epigenomic silencing of the BMP-4 gene in pituitary adenomas: a potential target for epidrug-induced re-expression.	GeneRIF	0.0044	65.1	0.0114	0.116	1	1	BMP4
Pubmed	22905972	CD74 expression is increased in high-grade, invasive urothelial carcinoma of the bladder.	GeneRIF	0.0044	65.1	0.0114	0.116	1	1	CD74
Pubmed	17259761	Association between an ANF Gene I/D dimorphism and left ventricular hypertrophy in a Gulf Arab population.	GeneRIF	0.0044	65.1	0.0114	0.116	1	1	NPPA
Pubmed	20454699	Identification and characterization of a novel TACSTD2 mutation in gelatinous drop-like corneal dystrophy.	GeneRIF	0.0044	65.1	0.0114	0.116	1	1	TACSTD2
Pubmed	7684766	A lipopolysaccharide-inducible macrophage gene (D3) is a new member of an interferon-inducible gene cluster and is selectively expressed in mononuclear phagocytes.	Pubmed	0.0044	65.1	0.0114	0.116	1	1	IFI16
Pubmed	18043741	Nogo Receptor 1 (RTN4R) as a candidate gene for schizophrenia: analysis using human and mouse genetic approaches.	GeneRIF	0.0044	65.1	0.0114	0.116	1	1	RTN4R
Pubmed	2111346	The IFN-gamma response of the murine invariant chain gene is mediated by a complex enhancer that includes several MHC class II consensus elements.	Pubmed	0.0044	65.1	0.0114	0.116	1	1	CD74
Pubmed	17095853	Hb Jambol: a new hyperunstable hemoglobin causing severe hemolytic anemia.	GeneRIF	0.0044	65.1	0.0114	0.116	1	1	HBB
Pubmed	22267845	Serum ¿-glutamyltransferase levels correlate with epicardial adipose tissue thickness in patients with coronary artery disease.	GeneRIF	0.0044	65.1	0.0114	0.116	1	1	GGT1
Pubmed	3038854	Gene structure of human cytochrome P-450(SCC), cholesterol desmolase.	Pubmed	0.0044	65.1	0.0114	0.116	1	1	CYP11A1
Pubmed	10790218	Analysis of the human KCNH2(HERG) gene: identification and characterization of a novel mutation Y667X associated with long QT syndrome and a non-pathological 9 bp insertion.	Pubmed	0.0044	65.1	0.0114	0.116	1	1	KCNH2

Category	ID	Name	Source	p-value	q-value Bonferroni	q-value FDR B&H	q-value FDR B&Y	Hit Count in Query List	Hit Count in Genome	Hit in Query List
Pubmed	22645299	The expression of translocator protein in human thyroid cancer and its role in the response of thyroid cancer cells to oxidative stress.	GeneRIF	0.0044	65.1	0.0114	0.116	1	1	TSPO
Pubmed	21562575	The histone demethylase JARID1A is associated with susceptibility to ankylosing spondylitis.	GeneRIF	0.0044	65.1	0.0114	0.116	1	1	KDM5A
Pubmed	20876535	InsP3R-associated cGMP kinase substrate determines inositol 1,4,5-trisphosphate receptor susceptibility to phosphoregulation by cyclic nucleotide-dependent kinases.	Pubmed	0.0044	65.1	0.0114	0.116	1	1	MRVI1
Pubmed	15296476	The role of peripheral benzodiazepine receptors on the function and survival of isolated human pancreatic islets.	GeneRIF	0.0044	65.1	0.0114	0.116	1	1	TSPO
Pubmed	21092609	[Study on the relationship between sclerosis rim and bone morphogenetic proteins of osteonecrosis of the femoral head].	GeneRIF	0.0044	65.1	0.0114	0.116	1	1	BMP4
Pubmed	16034138	Adenosine-dependent pulmonary fibrosis in adenosine deaminase-deficient mice.	Pubmed	0.0044	65.1	0.0114	0.116	1	1	ADA
Pubmed	19387015	Association of HTRA1 mutations and familial ischemic cerebral small-vessel disease.	GeneRIF	0.0044	65.1	0.0114	0.116	1	1	HTRA1
Pubmed	12799453	Nuclear matrix association of the human beta-globin locus utilizing a novel approach to quantitative real-time PCR.	GeneRIF	0.0044	65.1	0.0114	0.116	1	1	HBB
Pubmed	20686372	Kallikrein-related peptidase 10 (KLK10) expression and single nucleotide polymorphisms in ovarian cancer survival.	GeneRIF	0.0044	65.1	0.0114	0.116	1	1	KLK10
Pubmed	23577918	Application of multiplex ligation-dependent probe amplification to screen for ß-globin cluster deletions: detection of two novel deletions in a multi ethnic population.	Pubmed	0.0044	65.1	0.0114	0.116	1	1	HBB
Pubmed	10561600	Conformational stability of human erythrocyte transglutaminase. Patterns of thermal unfolding at acid and alkaline pH.	GeneRIF	0.0044	65.1	0.0114	0.116	1	1	TGM2
Pubmed	20022966	Nuclear localization of the p75 neurotrophin receptor intracellular domain.	GeneRIF	0.0044	65.1	0.0114	0.116	1	1	NGFR
Pubmed	19118660	Overexpression of cathepsin K accelerates the resorption cycle and osteoblast differentiation in vitro.	GeneRIF	0.0044	65.1	0.0114	0.116	1	1	CTSK
Pubmed	17950619	Purification and characterization of a recombinant human testican-2 expressed in baculovirus-infected Sf9 insect cells.	GeneRIF	0.0044	65.1	0.0114	0.116	1	1	SPOCK2
Pubmed	24099560	BMP4, a new prognostic factor for glioma.	Pubmed	0.0044	65.1	0.0114	0.116	1	1	BMP4
Pubmed	19759357	BMP4 regulates the hematopoietic stem cell niche.	GeneRIF	0.0044	65.1	0.0114	0.116	1	1	BMP4
Pubmed	24493577	Expression and localization of serine protease Htra1 in neuroblastoma: correlation with cellular differentiation grade.	Pubmed	0.0044	65.1	0.0114	0.116	1	1	HTRA1
Pubmed	21901792	First implication of STRA6 mutations in isolated anophthalmia, microphthalmia, and coloboma: a new dimension to the STRA6 phenotype.	GeneRIF	0.0044	65.1	0.0114	0.116	1	1	STRA6
Pubmed	19254853	Regional heterogeneity of beta-thalassemia mutations in the multi ethnic Indian population.	GeneRIF	0.0044	65.1	0.0114	0.116	1	1	HBB
Pubmed	11971871	Activation of the beta-like globin genes in transgenic mice is dependent on the presence of the beta-locus control region.	GeneRIF	0.0044	65.1	0.0114	0.116	1	1	HBB
Pubmed	19187775	Fasting-induced hypothermia and reduced energy production in mice lacking acetyl-CoA synthetase 2.	GeneRIF	0.0044	65.1	0.0114	0.116	1	1	ACSS1
Pubmed	12648278	HLA-DPB1*0401 is associated with dominant protection against type 1 diabetes in the general Saudi population and in subjects with a high-risk DR/DQ haplotype.	GeneRIF	0.0044	65.1	0.0114	0.116	1	1	HLA-DQB1
Pubmed	24050790	Identification of a novel benzoxazolone derivative as a selective, orally active 18 kDa translocator protein (TSPO) ligand.	Pubmed	0.0044	65.1	0.0114	0.116	1	1	TSPO
Pubmed	7510069	Diversity of endogenous epitopes bound to MHC class II molecules limited by invariant chain.	Pubmed	0.0044	65.1	0.0114	0.116	1	1	CD74
Pubmed	10233364	Unexpected pattern of beta-globin mutations in beta-thalassaemia patients from northern Portugal.	Pubmed	0.0044	65.1	0.0114	0.116	1	1	HBB
Pubmed	17353235	Combined saposin C and D deficiencies in mice lead to a neuronopathic phenotype, glucosylceramide and alpha-hydroxy ceramide accumulation, and altered prosaposin trafficking.	GeneRIF	0.0044	65.1	0.0114	0.116	1	1	PSAP
Pubmed	21670081	BMP4 promotes prostate tumor growth in bone through osteogenesis.	GeneRIF	0.0044	65.1	0.0114	0.116	1	1	BMP4
Pubmed	22400494	Potential association between frequent nonsynonymous variant of NPPA and cardioembolic stroke.	GeneRIF	0.0044	65.1	0.0114	0.116	1	1	NPPA
Pubmed	17502620	Photodynamic molecular beacon as an activatable photosensitizer based on protease-controlled singlet oxygen quenching and activation.	GeneRIF	0.0044	65.1	0.0114	0.116	1	1	MMP7
Pubmed	17149363	The cataract-causing mutation G98R in human alphaA-crystallin leads to folding defects and loss of chaperone activity.	GeneRIF	0.0044	65.1	0.0114	0.116	1	1	CRYAA
Pubmed	17546031	Deficiency of PORCN, a regulator of Wnt signaling, is associated with focal dermal hypoplasia.	GeneRIF	0.0044	65.1	0.0114	0.116	1	1	PORCN
Pubmed	21063096	60kDa lysophospholipase, a new Sgk1 molecular partner involved in the regulation of ENaC.	GeneRIF	0.0044	65.1	0.0114	0.116	1	1	ASPG
Pubmed	22558321	Choline dehydrogenase polymorphism rs12676 is a functional variation and is associated with changes in human sperm cell function.	GeneRIF	0.0044	65.1	0.0114	0.116	1	1	CHDH
Pubmed	21971542	Tissue transglutaminase antibodies in celiac disease: focus on the pediatric population.	GeneRIF	0.0044	65.1	0.0114	0.116	1	1	TGM2
Pubmed	11564864	Involvement of alpha-PAK-interacting exchange factor in the PAK1-c-Jun NH(2)-terminal kinase 1 activation and apoptosis induced by benzo[a]pyrene.	Pubmed	0.0044	65.1	0.0114	0.116	1	1	ARHGEF6
Pubmed	21683691	Valine 44 and valine 45 of human glutathione synthetase are key for subunit stability and negative cooperativity.	GeneRIF	0.0044	65.1	0.0114	0.116	1	1	GSS
Pubmed	11857779	Generation of a loxP flanked bmp4loxP-lacZ allele marked by conditional lacZ expression.	Pubmed	0.0044	65.1	0.0114	0.116	1	1	BMP4

Category	ID	Name	Source	p-value	q-value Bonferroni	q-value FDR B&H	q-value FDR B&Y	Hit Count in Query List	Hit Count in Genome	Hit in Query List
Pubmed	15039018	Modulation of Gardos channel activity by oxidants and oxygen tension: effects of 1-chloro-2,4-dinitrobenzene and phenazine methosulphate.	GeneRIF	0.0044	65.1	0.0114	0.116	1	1	KCNN4
Pubmed	11409652	Association study between the variants of the human ANP gene and essential hypertension.	GeneRIF	0.0044	65.1	0.0114	0.116	1	1	NPPA
Pubmed	20664972	Down-regulation of fractalkine inhibits the in vitro and in vivo angiogenesis of the hepatocellular carcinoma HepG2 cells.	GeneRIF	0.0044	65.1	0.0114	0.116	1	1	CX3CL1
Pubmed	3707969	Hb J-Antakya or alpha 2 beta (2)65(E9)Lys----Met in a Turkish family and Hb complutense or alpha 2 beta (2)127(H5)Gln----Glu in a Spanish family; correction of a previously published identification.	Pubmed	0.0044	65.1	0.0114	0.116	1	1	HBB
Pubmed	6994493	Development of a somatic mutation screening system using Hb mutants. IV. Successful detection of red cells containing the human frameshift mutants Hb Wayne and Hb Cranston using monospecific fluorescent antibodies.	Pubmed	0.0044	65.1	0.0114	0.116	1	1	HBA2
Pubmed	17499268	Spontaneous myocarditis mimicking human disease occurs in the presence of an appropriate MHC and non-MHC background in transgenic mice.	Pubmed	0.0044	65.1	0.0114	0.116	1	1	HLA-DQB1
Pubmed	20614481	CD74 is a survival receptor on colon epithelial cells.	GeneRIF	0.0044	65.1	0.0114	0.116	1	1	CD74
Pubmed	10821767	Bmp4 and Fgf10 play opposing roles during lung bud morphogenesis.	Pubmed	0.0044	65.1	0.0114	0.116	1	1	BMP4
Pubmed	19174314	Role of the R1135H KCNH2 mutation in Brugada syndrome.	GeneRIF	0.0044	65.1	0.0114	0.116	1	1	KCNH2
Pubmed	21799211	The effect of neurogranin on neural correlates of episodic memory encoding and retrieval.	GeneRIF	0.0044	65.1	0.0114	0.116	1	1	NRGN
Pubmed	18056261	Human kallikrein-related peptidase 14 (KLK14) is a new activator component of the KLK proteolytic cascade. Possible function in seminal plasma and skin.	GeneRIF	0.0044	65.1	0.0114	0.116	1	1	KLK14
Pubmed	23071671	Actin recruitment to the Chlamydia inclusion is spatiotemporally regulated by a mechanism that requires host and bacterial factors.	GeneRIF	0.0044	65.1	0.0114	0.116	1	1	ACTA1
Pubmed	15658186	Prevalence of the sickle cell gene in Yemen: a pilo study.	GeneRIF	0.0044	65.1	0.0114	0.116	1	1	HBB
Pubmed	19878304	Functional significance of five noncanonical Ca2+-binding sites of human transglutaminase 2 characterized by site-directed mutagenesis.	GeneRIF	0.0044	65.1	0.0114	0.116	1	1	TGM2
Pubmed	12960346	Identification of tissue transglutaminase as a novel molecule involved in human CD8+ T cell transendothelial migration.	GeneRIF	0.0044	65.1	0.0114	0.116	1	1	TGM2
Pubmed	19765650	Expression and significance of HERG (KCNH2) potassium channels in the regulation of MDA-MB-435S melanoma cell proliferation and migration.	GeneRIF	0.0044	65.1	0.0114	0.116	1	1	KCNH2
Pubmed	22031939	Important but differential roles for actin in trafficking of Epstein-Barr virus in B cells and epithelial cells.	GeneRIF	0.0044	65.1	0.0114	0.116	1	1	ACTA1
Pubmed	22273838	Unstable hemoglobin Rush [beta 101(G3) Glu>Gln, HBB:c.304G>C] in a Brazilian family with moderate hemolytic anemia.	GeneRIF	0.0044	65.1	0.0114	0.116	1	1	HBB
Pubmed	17454790	Homozygous 23-bp insertion of endothelial protein c receptor gene in a child with fatal sepsis.	GeneRIF	0.0044	65.1	0.0114	0.116	1	1	PROCR
Pubmed	10517660	Characterization of a novel missense mutation in the pore of HERG in a patient with long QT syndrome.	Pubmed	0.0044	65.1	0.0114	0.116	1	1	KCNH2
Pubmed	23474948	B7-H3 associated with tumor progression and epigenetic regulatory activity in cutaneous melanoma.	Pubmed	0.0044	65.1	0.0114	0.116	1	1	CD276
Pubmed	13999302	Four hemoglobins in each of three brothers. Genetic and biochemical significance.	Pubmed	0.0044	65.1	0.0114	0.116	1	1	HBA2
Pubmed	19553662	Angiopoietin 2 mediates the differentiation and migration of neural progenitor cells in the subventricular zone after stroke.	GeneRIF	0.0044	65.1	0.0114	0.116	1	1	ANGPTL2
Pubmed	24005896	A common polymorphism in the tissue kallikrein gene is associated with increased urinary excretions of calcium and sodium in Japanese volunteers.	Pubmed	0.0044	65.1	0.0114	0.116	1	1	KLK1
Pubmed	24391132	Noninvasive and quantitative live imaging reveals a potential stress-responsive enhancer in the failing heart.	Pubmed	0.0044	65.1	0.0114	0.116	1	1	NPPA
Pubmed	21123876	Structure of the human FOXO4-DBD-DNA complex at 1.9 Å resolution reveals new details of FOXO binding to the DNA.	GeneRIF	0.0044	65.1	0.0114	0.116	1	1	FOXO4
Pubmed	24088021	The role of basic amino acid surface clusters on the collagenase activity of cathepsin K.	Pubmed	0.0044	65.1	0.0114	0.116	1	1	CTSK
Pubmed	3022937	Expression and structure of the human NGF receptor.	Pubmed	0.0044	65.1	0.0114	0.116	1	1	NGFR
Pubmed	23790336	COX-2, MMP-7 expression in oral lichen planus and oral squamous cell carcinoma.	Pubmed	0.0044	65.1	0.0114	0.116	1	1	MMP7
Pubmed	18594946	Homocysteine-induced toxicity increases TG2 expression in Neuro2a cells.	GeneRIF	0.0044	65.1	0.0114	0.116	1	1	TGM2
Pubmed	18500535	Expression and intracellular localization of matrix metalloproteinases in intraductal papillary mucinous neoplasms of the pancreas.	GeneRIF	0.0044	65.1	0.0114	0.116	1	1	MMP7
Pubmed	22402880	Induction of human humoral immune responses in a novel HLA-DR-expressing transgenic NOD/Shi-scid/¿cnull mouse.	Pubmed	0.0044	65.1	0.0114	0.116	1	1	HLA-DQB1
Pubmed	8294454	Structure and expression of the human gene for the matrix metalloproteinase matrilysin.	Pubmed	0.0044	65.1	0.0114	0.116	1	1	MMP7
Pubmed	3610158	Confirmation of assignment of the human alpha 1-crystallin gene (CRYA1) to chromosome 21 with regional localization to q22.3.	Pubmed	0.0044	65.1	0.0114	0.116	1	1	CRYAA
Pubmed	9354791	Opitz G/BBB syndrome, a defect of midline development, is due to mutations in a new RING finger gene on Xp22.	Pubmed	0.0044	65.1	0.0114	0.116	1	1	MID1
Pubmed	18473248	Compound heterozygosity for Hb S [beta6(A3)GluVal, GAG-->GTG] and a new thalassemic mutation [beta132(H10)Lys-->term, AAA-->TAA] detected in a family from West Africa.	GeneRIF	0.0044	65.1	0.0114	0.116	1	1	HBB

Category	ID	Name	Source	p-value	q-value Bonferroni	q-value FDR B&H	q-value FDR B&Y	Hit Count in Query List	Hit Count in Genome	Hit in Query List
Pubmed	16916928	Stable stem cell commitment to the adipocyte lineage by inhibition of DNA methylation: role of the BMP-4 gene.	GeneRIF	0.0044	65.1	0.0114	0.116	1	1	BMP4
Pubmed	24963668	Role of the potassium channel KCa3.1 in diabetic nephropathy.	Pubmed	0.0044	65.1	0.0114	0.116	1	1	KCNN4
Pubmed	17851789	[Expression and clinical significance of costimulatory molecule B7-H3 mRNA and B7-H3 protein in gastric carcinoma].	GeneRIF	0.0044	65.1	0.0114	0.116	1	1	CD276
Pubmed	25330297	CD271 mediates stem cells to early progeny transition in human epidermis.	Pubmed	0.0044	65.1	0.0114	0.116	1	1	NGFR
Pubmed	15258566	AKAP12/Gravin is inactivated by epigenetic mechanism in human gastric carcinoma and shows growth suppressor activity.	GeneRIF	0.0044	65.1	0.0114	0.116	1	1	AKAP12
Pubmed	19060845	Cathepsin-k expression in pulmonary lymphangioleiomyomatosis.	GeneRIF	0.0044	65.1	0.0114	0.116	1	1	CTSK
Pubmed	20705078	Over-expression of dC-DCLK-short in mouse brain results in a more anxious behavioral phenotype.	GeneRIF	0.0044	65.1	0.0114	0.116	1	1	DCLK1
Pubmed	23288997	A novel mutation in CRYAA is associated with autosomal dominant suture cataracts in a Chinese family.	GeneRIF	0.0044	65.1	0.0114	0.116	1	1	CRYAA
Pubmed	20596026	Human melanoma-initiating cells express neural crest nerve growth factor receptor CD271.	GeneRIF	0.0044	65.1	0.0114	0.116	1	1	NGFR
Pubmed	24481864	Role of p75 neurotrophin receptor in stem cell biology: more than just a marker.	Pubmed	0.0044	65.1	0.0114	0.116	1	1	NGFR
Pubmed	21572087	Deletion of the murine scavenger receptor CD68.	GeneRIF	0.0044	65.1	0.0114	0.116	1	1	CD68
Pubmed	8585423	Cloning and complete coding sequence of a novel human cathepsin expressed in giant cells of osteoclastomas.	Pubmed	0.0044	65.1	0.0114	0.116	1	1	CTSK
Pubmed	25605781	A transient wave of BMP signaling in the retina is necessary for Müller glial differentiation.	Pubmed	0.0044	65.1	0.0114	0.116	1	1	BMP4
Pubmed	25183384	High prevalence of thalassemia in migrant populations in Guangdong Province, China.	Pubmed	0.0044	65.1	0.0114	0.116	1	1	HBB
Pubmed	9311716	Suppression of allograft responses induced by interleukin-6, which selectively modulates interferon-gamma but not interleukin-2 production.	Pubmed	0.0044	65.1	0.0114	0.116	1	1	HLA-DQB1
Pubmed	8931698	Cloning of the cDNA for a human homolog of the rat PEP-19 gene and mapping to chromosome 21q22.2-q22.3.	Pubmed	0.0044	65.1	0.0114	0.116	1	1	PCP4
Pubmed	15784962	Characterization of the signaling pathway downstream p75 neurotrophin receptor involved in beta-amyloid peptide-dependent cell death.	GeneRIF	0.0044	65.1	0.0114	0.116	1	1	NGFR
Pubmed	19428782	Cathepsin K generates enkephalin from beta-endorphin: a new mechanism with possible relevance for schizophrenia.	GeneRIF	0.0044	65.1	0.0114	0.116	1	1	CTSK
Pubmed	20465443	Autosomal dominant congenital nuclear cataracts caused by a CRYAA gene mutation.	GeneRIF	0.0044	65.1	0.0114	0.116	1	1	CRYAA
Pubmed	23105113	Mapping of the interaction site between sortilin and the p75 neurotrophin receptor reveals a regulatory role for the sortilin intracellular domain in p75 neurotrophin receptor shedding and apoptosis.	GeneRIF	0.0044	65.1	0.0114	0.116	1	1	NGFR
Pubmed	21173165	Human globin knock-in mice complete fetal-to-adult hemoglobin switching in postnatal development.	GeneRIF	0.0044	65.1	0.0114	0.116	1	1	HBB
Pubmed	13856	Hemoglobin Tarrant: alpha126(H9) Asp leads to Asn. A new hemoglobin variant in the alpha1beta1 contact region showing high oxygen affinity and reduced cooperativity.	Pubmed	0.0044	65.1	0.0114	0.116	1	1	HBA2
Pubmed	3018615	Comparison of benzodiazepine receptor binding in membranes from human or rat brain.	Pubmed	0.0044	65.1	0.0114	0.116	1	1	TSPO
Pubmed	15385559	Recruitment of transcription complexes to the beta-globin gene locus in vivo and in vitro.	GeneRIF	0.0044	65.1	0.0114	0.116	1	1	HBB
Pubmed	19351516	Scn3b knockout mice exhibit abnormal ventricular electrophysiological properties.	GeneRIF	0.0044	65.1	0.0114	0.116	1	1	SCN3B
Pubmed	23181747	Hb Iberia [a104(G11)Cys ¿ Arg,TGC>CGC (a2) (HBA2:c.313T>C)], a new a-thalassemic hemoglobin variant found in the Iberian Peninsula: report of six cases.	GeneRIF	0.0044	65.1	0.0114	0.116	1	1	HBA2
Pubmed	20699637	Channel triage: Emerging insights into the processing and quality control of hERG potassium channels by DnaJA proteins 1, 2 and 4.	GeneRIF	0.0044	65.1	0.0114	0.116	1	1	KCNH2
Pubmed	17720709	Nucleosome and transcription activator antagonism at human beta-globin locus control region DNase I hypersensitive sites.	GeneRIF	0.0044	65.1	0.0114	0.116	1	1	HBB
Pubmed	18252212	Mutations in BMP4 cause eye, brain, and digit developmental anomalies: overlap between the BMP4 and hedgehog signaling pathways.	GeneRIF	0.0044	65.1	0.0114	0.116	1	1	BMP4
Pubmed	24220096	Novel association between CD74 polymorphisms and hematologic toxicity in patients with NSCLC after platinum-based chemotherapy.	Pubmed	0.0044	65.1	0.0114	0.116	1	1	CD74
Pubmed	12579270	Expression of matrix metalloproteinases in human transitional cell carcinoma of the urinary bladder.	GeneRIF	0.0044	65.1	0.0114	0.116	1	1	MMP7
Pubmed	19544486	DC-induced CD8(+) T-cell response is inhibited by MHC class II-dependent DX5(+)CD4(+) Treg.	Pubmed	0.0044	65.1	0.0114	0.116	1	1	HLA-DQB1
Pubmed	15475579	HERG channel (dys)function revealed by dynamic action potential clamp technique.	GeneRIF	0.0044	65.1	0.0114	0.116	1	1	KCNH2
Pubmed	14506716	Late-onset distal muscular dystrophy affecting the posterior calves.	GeneRIF	0.0044	65.1	0.0114	0.116	1	1	DYSF
Pubmed	21797703	Molecular analysis of ß-thalassemia patients: first identification of mutations HBB:c.93-2A>G and HBB:c.114G>A in Brazil.	GeneRIF	0.0044	65.1	0.0114	0.116	1	1	HBB
Pubmed	9590438	Sympathetic and sensory innervation of the extracerebral vasculature: roles for p75NTR neuronal expression and nerve growth factor.	Pubmed	0.0044	65.1	0.0114	0.116	1	1	NGFR
Pubmed	25359863	Fractalkine promotes human monocyte survival via a reduction in oxidative stress.	Pubmed	0.0044	65.1	0.0114	0.116	1	1	CX3CL1
Pubmed	19903589	Analysis of linkage disequilibrium between the 5' and 3' haplotypes of the beta-globin gene cluster in Mexican afromestizos.	GeneRIF	0.0044	65.1	0.0114	0.116	1	1	HBB
Pubmed	9029710	Peripheral benzodiazepine receptor in cholesterol transport and steroidogenesis.	Pubmed	0.0044	65.1	0.0114	0.116	1	1	CYP11A1

Category	ID	Name	Source	p-value	q-value Bonferroni	q-value FDR B&H	q-value FDR B&Y	Hit Count in Query List	Hit Count in Genome	Hit in Query List
Pubmed	2464826	Alternative splicing of HLA-DQB transcripts and secretion of HLA-DQ beta-chain proteins: allelic polymorphism in splicing and polyadenylylation sites.	Pubmed	0.0044	65.1	0.0114	0.116	1	1	HLA-DQB1
Pubmed	24479710	Napsin A is a useful marker for metastatic adenocarcinomas of pulmonary origin.	Pubmed	0.0044	65.1	0.0114	0.116	1	1	NAPSA
Pubmed	20673767	Biological variations of MASP-3 and MAp44, two splice products of the MASP1 gene involved in regulation of the complement system.	GeneRIF	0.0044	65.1	0.0114	0.116	1	1	MASP1
Pubmed	11103812	A novel melanoma gene (MG50) encoding the interleukin 1 receptor antagonist and six epitopes recognized by human cytolytic T lymphocytes.	Pubmed	0.0044	65.1	0.0114	0.116	1	1	PXDN
Pubmed	17072861	Distinct expression and localization of serine protease HtrA1 in human endometrium and first-trimester placenta.	GeneRIF	0.0044	65.1	0.0114	0.116	1	1	HTRA1
Pubmed	2419119	Study of cholesterol side-chain cleavage (20,22 desmolase) deficiency causing congenital lipoid adrenal hyperplasia using bovine-sequence P450scc oligodeoxyribonucleotide probes.	Pubmed	0.0044	65.1	0.0114	0.116	1	1	CYP11A1
Pubmed	12093810	Gliadin T cell epitope selection by tissue transglutaminase in celiac disease. Role of enzyme specificity and pH influence on the transamidation versus deamidation process.	GeneRIF	0.0044	65.1	0.0114	0.116	1	1	TGM2
Pubmed	16480967	Plasma beta-globin DNA as a prognostic marker in chest pain patients.	GeneRIF	0.0044	65.1	0.0114	0.116	1	1	HBB
Pubmed	2563599	Regulation of the expression of some genes for enzymes of glutathione metabolism in hepatotoxicity and hepatocarcinogenesis.	Pubmed	0.0044	65.1	0.0114	0.116	1	1	GGT1
Pubmed	23720051	CX3CL1 promotes breast cancer via transactivation of the EGF pathway.	Pubmed	0.0044	65.1	0.0114	0.116	1	1	CX3CL1
Pubmed	25404341	Activity-regulating structural changes and autoantibody epitopes in transglutaminase 2 assessed by hydrogen/deuterium exchange.	Pubmed	0.0044	65.1	0.0114	0.116	1	1	TGM2
Pubmed	24044563	Associations between total serum GGT activity and metabolic risk: MESA.	Pubmed	0.0044	65.1	0.0114	0.116	1	1	GGT1
Pubmed	14749380	Capn5 is expressed in a subset of T cells and is dispensable for development.	GeneRIF	0.0044	65.1	0.0114	0.116	1	1	CAPN5
Pubmed	19950405	Smoothelin is a specific marker for smooth muscle neoplasms of the gastrointestinal tract.	GeneRIF	0.0044	65.1	0.0114	0.116	1	1	SMTN
Pubmed	17053109	A variant of the HTRA1 gene increases susceptibility to age-related macular degeneration.	GeneRIF	0.0044	65.1	0.0114	0.116	1	1	HTRA1
Pubmed	17526978	Endothelial lipase gene polymorphism is associated with acute myocardial infarction, independently of high-density lipoprotein-cholesterol levels.	GeneRIF	0.0044	65.1	0.0114	0.116	1	1	LIPG
Pubmed	17227582	Calpain-5 gene variants are associated with diastolic blood pressure and cholesterol levels.	GeneRIF	0.0044	65.1	0.0114	0.116	1	1	CAPN5
Pubmed	12640113	Retinoic acid repression of bone morphogenetic protein 4 in inner ear development.	Pubmed	0.0044	65.1	0.0114	0.116	1	1	BMP4
Pubmed	17568988	Association of the HTRA1 gene variant with age-related macular degeneration in the Japanese population.	GeneRIF	0.0044	65.1	0.0114	0.116	1	1	HTRA1
Pubmed	12063400	The mouse Arhgef6 gene: cDNA sequence, expression analysis, and chromosome assignment.	GeneRIF	0.0044	65.1	0.0114	0.116	1	1	ARHGEF6
Pubmed	11300344	Hb Tsukumi [beta117(G19)His-->Tyr] found in a Moroccan woman.	Pubmed	0.0044	65.1	0.0114	0.116	1	1	HBB
Pubmed	2498298	Structure of full-length cDNA coding for sulfatide activator, a Co-beta-glucosidase and two other homologous proteins: two alternate forms of the sulfatide activator.	Pubmed	0.0044	65.1	0.0114	0.116	1	1	PSAP
Pubmed	9139708	Mice with genetic gamma-glutamyl transpeptidase deficiency exhibit glutathionuria, severe growth failure, reduced life spans, and infertility.	Pubmed	0.0044	65.1	0.0114	0.116	1	1	GGT1
Pubmed	17053108	HTRA1 promoter polymorphism in wet age-related macular degeneration.	GeneRIF	0.0044	65.1	0.0114	0.116	1	1	HTRA1
Pubmed	12562924	The smooth muscle myosin seven amino acid heavy chain insert's kinetic role in the crossbridge cycle for mouse bladder.	Pubmed	0.0044	65.1	0.0114	0.116	1	1	MYH11
Pubmed	17439632	Autoantibodies against endothelial protein C receptor and the risk of a first deep vein thrombosis.	GeneRIF	0.0044	65.1	0.0114	0.116	1	1	PROCR
Pubmed	15749232	Serine protease inhibitor kazal type 1 mutations and pancreatitis.	GeneRIF	0.0044	65.1	0.0114	0.116	1	1	SPINK1
Pubmed	18460028	Alterations in the desquamation-related proteolytic cleavage of corneodesmosin and other corneodesmosomal proteins in psoriatic lesional epidermis.	Pubmed	0.0044	65.1	0.0114	0.116	1	1	CDSN
Pubmed	12887056	Cleavage site specificity of cathepsin K toward cartilage proteoglycans and protease complex formation.	GeneRIF	0.0044	65.1	0.0114	0.116	1	1	CTSK
Pubmed	17043648	Tissue transglutaminase expression promotes cell attachment, invasion and survival in breast cancer cells.	GeneRIF	0.0044	65.1	0.0114	0.116	1	1	TGM2
Pubmed	23966322	Misregulated progesterone secretion and impaired pregnancy in Cyp11a1 transgenic mice.	Pubmed	0.0044	65.1	0.0114	0.116	1	1	CYP11A1
Pubmed	22614534	Endothelial protein C receptor expressed by ovarian cancer cells as a possible biomarker of cancer onset.	GeneRIF	0.0044	65.1	0.0114	0.116	1	1	PROCR
Pubmed	10961988	Up-regulation of the IKCa1 potassium channel during T-cell activation. Molecular mechanism and functional consequences.	Pubmed	0.0044	65.1	0.0114	0.116	1	1	KCNN4
Pubmed	22183269	p75NTR is mainly responsible for Aß toxicity but not for its internalization: a primary study.	GeneRIF	0.0044	65.1	0.0114	0.116	1	1	NGFR
Pubmed	20144598	Mutation -538 T/C in bone morphogenetic protein 4 do not increase the risk in sickle-cell disease with orthopedic complications but strongly associated with increased LDH and uric acid level in Indian patients from Chhattisgarh and Jharkhand states.	GeneRIF	0.0044	65.1	0.0114	0.116	1	1	BMP4
Pubmed	8507180	Murine bone morphogenetic protein-4 gene: existence of multiple promoters and exons for the 5'-untranslated region.	Pubmed	0.0044	65.1	0.0114	0.116	1	1	BMP4

Category	ID	Name	Source	p-value	q-value Bonferroni	q-value FDR B&H	q-value FDR B&Y	Hit Count in Query List	Hit Count in Genome	Hit in Query List
Pubmed	18566344	Neurotrophin p75 receptor (p75NTR) promotes endothelial cell apoptosis and inhibits angiogenesis: implications for diabetes-induced impaired neovascularization in ischemic limb muscles.	GeneRIF	0.0044	65.1	0.0114	0.116	1	1	NGFR
Pubmed	13897827	Chemical studies of several varieties of Hb M.	Pubmed	0.0044	65.1	0.0114	0.116	1	1	HBB
Pubmed	1301199	A novel beta-globin structural mutant, Hb Brescia (beta 114 Leu-Pro), causing a severe beta-thalassemia intermedia phenotype.	Pubmed	0.0044	65.1	0.0114	0.116	1	1	HBB
Pubmed	12529763	Endothelial cell protein C receptor (EPCR) gene exon III, 23 bp insertion mutation in the Turkish pediatric thrombotic patients.	GeneRIF	0.0044	65.1	0.0114	0.116	1	1	PROCR
Pubmed	7238856	Hemoglobin Saint Mandé beta 102 (G4) asn replaced by tyr: a new low oxygen affinity variant.	Pubmed	0.0044	65.1	0.0114	0.116	1	1	HBB
Pubmed	17340203	The G22A polymorphism of the ADA gene and susceptibility to autism spectrum disorders.	GeneRIF	0.0044	65.1	0.0114	0.116	1	1	ADA
Pubmed	12969992	Fractalkine in vascular biology: from basic research to clinical disease.	GeneRIF	0.0044	65.1	0.0114	0.116	1	1	CX3CL1
Pubmed	8095045	Significance of Arg-107 and Glu-108 in the catalytic mechanism of human gamma-glutamyl transpeptidase. Identification by site-directed mutagenesis.	Pubmed	0.0044	65.1	0.0114	0.116	1	1	GGT1
Pubmed	12002684	Cellular transporters for zinc.	Pubmed	0.0044	65.1	0.0114	0.116	1	1	SLC39A2
Pubmed	24131739	Study of the association between the BMP4 gene and congenital anomalies of the kidney and urinary tract.	Pubmed	0.0044	65.1	0.0114	0.116	1	1	BMP4
Pubmed	10369661	Molecular basis of glutathione synthetase deficiency and a rare gene permutation event.	Pubmed	0.0044	65.1	0.0114	0.116	1	1	GSS
Pubmed	17310992	Blockage of intermediate-conductance-Ca(2+) -activated K(+) channels inhibits progression of human endometrial cancer.	GeneRIF	0.0044	65.1	0.0114	0.116	1	1	KCNN4
Pubmed	24979558	A novel alpha-thalassemia nonsense mutation in HBA2: C.382 A > T globin gene.	Pubmed	0.0044	65.1	0.0114	0.116	1	1	HBA2
Pubmed	11454986	Corneodesmosin expression in psoriasis vulgaris differs from normal skin and other inflammatory skin disorders.	Pubmed	0.0044	65.1	0.0114	0.116	1	1	CDSN
Pubmed	24036083	Electrocardiographic screening of 1-month-old infants for identifying prolonged QT intervals.	Pubmed	0.0044	65.1	0.0114	0.116	1	1	KCNH2
Pubmed	10722113	Hb Sallanches [alpha104(G11)Cys-->Tyr]: a rare alpha2-globin chain variant found in the homozygous state in three members of a Pakistani family.	Pubmed	0.0044	65.1	0.0114	0.116	1	1	HBA2
Pubmed	22876559	Influence of HLA DQ 2/8 genotypes in predisposing type 1 diabetes in siblings of a Saudi family with paternally inherited chromosomal translocations.	GeneRIF	0.0044	65.1	0.0114	0.116	1	1	HLA-DQB1
Pubmed	20655338	Acute inhibition of carboxypeptidase E expression in AtT-20 cells does not affect regulated secretion of ACTH.	GeneRIF	0.0044	65.1	0.0114	0.116	1	1	CPE
Pubmed	16111892	Ocular abnormalities in Large(myd) and Large(vls) mice, spontaneous models for muscle, eye, and brain diseases.	GeneRIF	0.0044	65.1	0.0114	0.116	1	1	LARGE
Pubmed	23085440	Impact of ß(S)-globin haplotypes on oxidative stress in patients with sickle cell anemia in steady state.	GeneRIF	0.0044	65.1	0.0114	0.116	1	1	HBB
Pubmed	23305733	Emergence, origin, and function of neutrophil-dendritic cell hybrids in experimentally induced inflammatory lesions in mice.	Pubmed	0.0044	65.1	0.0114	0.116	1	1	HLA-DQB1
Pubmed	12144065	A hemoglobin variant found during glycohemoglobin measurement, identified as Hb Toulon [alpha77(EF6)Pro-->His] by tandem mass spectrometry.	Pubmed	0.0044	65.1	0.0114	0.116	1	1	HBA2
Pubmed	15015603	Increased plasma levels of serine proteinase inhibitors in lung cancer patients.	GeneRIF	0.0044	65.1	0.0114	0.116	1	1	SLPI
Pubmed	18365875	BMP4 induces an epithelial-mesenchymal transition-like response in adult airway epithelial cells.	GeneRIF	0.0044	65.1	0.0114	0.116	1	1	BMP4
Pubmed	24692557	Fibulin 2, a tyrosine O-sulfated protein, is up-regulated following retinal detachment.	Pubmed	0.0044	65.1	0.0114	0.116	1	1	FBLN2
Pubmed	23824706	Celiac Disease-Specific TG2-Targeted Autoantibodies Inhibit Angiogenesis Ex Vivo and In Vivo in Mice by Interfering with Endothelial Cell Dynamics.	Pubmed	0.0044	65.1	0.0114	0.116	1	1	TGM2
Pubmed	15254015	Disruption of muscle membrane and phenotype divergence in two novel mouse models of dysferlin deficiency.	Pubmed	0.0044	65.1	0.0114	0.116	1	1	DYSF
Pubmed	8121239	Characterization and differentiation of peripheral-type benzodiazepine receptors in rat and human prostate.	Pubmed	0.0044	65.1	0.0114	0.116	1	1	TSPO
Pubmed	24657844	Human 60-kDa lysophospholipase contains an N-terminal L-asparaginase domain that is allosterically regulated by L-asparagine.	Pubmed	0.0044	65.1	0.0114	0.116	1	1	ASPG
Pubmed	12856083	Pancreatic NOD beta cells express MHC class II protein and the frequency of I-A(g7) mRNA-expressing beta cells strongly increases during progression to autoimmune diabetes.	GeneRIF	0.0044	65.1	0.0114	0.116	1	1	HLA-DQB1
Pubmed	23996702	The role of the antileukoprotease SLPI in smoking-induced human papillomavirus-independent head and neck squamous cell carcinomas.	Pubmed	0.0044	65.1	0.0114	0.116	1	1	SLPI
Pubmed	24218454	Temporal requirements for B cells in the establishment of CD4 T cell memory.	Pubmed	0.0044	65.1	0.0114	0.116	1	1	HLA-DQB1
Pubmed	23626764	CD271 defines a stem cell-like population in hypopharyngeal cancer.	Pubmed	0.0044	65.1	0.0114	0.116	1	1	NGFR
Pubmed	14752105	Cell type-specific activation of intracellular transglutaminase 2 by oxidative stress or ultraviolet irradiation: implications of transglutaminase 2 in age-related cataractogenesis.	GeneRIF	0.0044	65.1	0.0114	0.116	1	1	TGM2
Pubmed	21375584	Presence of SPINK-1 variant alters the course of chronic pancreatitis.	GeneRIF	0.0044	65.1	0.0114	0.116	1	1	SPINK1
Pubmed	15256071	Scanning of beta-globin gene for identification of beta-thalassemia mutation in Romanian population.	GeneRIF	0.0044	65.1	0.0114	0.116	1	1	HBB
Pubmed	9556067	Cathepsin K mRNA detection is restricted to osteoclasts during fetal mouse development.	Pubmed	0.0044	65.1	0.0114	0.116	1	1	CTSK
Pubmed	16990440	Polymorphisms in the HBB gene relate to individual cardiorespiratory adaptation in response to endurance training.	GeneRIF	0.0044	65.1	0.0114	0.116	1	1	HBB

Category	ID	Name	Source	p-value	q-value Bonferroni	q-value FDR B&H	q-value FDR B&Y	Hit Count in Query List	Hit Count in Genome	Hit in Query List
Pubmed	9858242	R411C mutation of the ALAS2 gene encodes a pyridoxine-responsive enzyme with low activity.	Pubmed	0.0044	65.1	0.0114	0.116	1	1	ALAS2
Pubmed	9731526	Dysferlin, a novel skeletal muscle gene, is mutated in Miyoshi myopathy and limb girdle muscular dystrophy.	Pubmed	0.0044	65.1	0.0114	0.116	1	1	DYSF
Pubmed	20130207	Silencing of Mustn1 inhibits myogenic fusion and differentiation.	GeneRIF	0.0044	65.1	0.0114	0.116	1	1	MUSTN1
Pubmed	16219026	Carboxypeptidase E is required for normal synaptic transmission from photoreceptors to the inner retina.	GeneRIF	0.0044	65.1	0.0114	0.116	1	1	CPE
Pubmed	8125973	Induction of peripheral-type benzodiazepine receptors during differentiation of mouse erythroleukemia cells. A possible involvement of these receptors in heme biosynthesis.	Pubmed	0.0044	65.1	0.0114	0.116	1	1	TSPO
Pubmed	23408932	Mac-1 directly binds to the endothelial protein C-receptor: a link between the protein C anticoagulant pathway and inflammation?	GeneRIF	0.0044	65.1	0.0114	0.116	1	1	PROCR
Pubmed	22972429	Expression of endothelial lipase correlates with the size of neointima in a murine model of vascular remodeling.	GeneRIF	0.0044	65.1	0.0114	0.116	1	1	LIPG
Pubmed	21344157	Expression of B7-H3 in hypopharyngeal squamous cell carcinoma as a predictive indicator for tumor metastasis and prognosis.	GeneRIF	0.0044	65.1	0.0114	0.116	1	1	CD276
Pubmed	12513847	[Association of the relationship between HLA-DQB1 alleles and major beta-thalassemia in 42 guangdong Chinese].	GeneRIF	0.0044	65.1	0.0114	0.116	1	1	HLA-DQB1
Pubmed	25289770	Protective roles of aldo-keto reductase 1B10 and autophagy against toxicity induced by p-quinone metabolites of tert-butylhydroquinone in lung cancer A549 cells.	Pubmed	0.0044	65.1	0.0114	0.116	1	1	AKR1B10
Pubmed	3853975	Human kidney kallikrein: cDNA cloning and sequence analysis.	Pubmed	0.0044	65.1	0.0114	0.116	1	1	KLK1
Pubmed	1958198	Isolation and characterization of prosaposin from human milk.	Pubmed	0.0044	65.1	0.0114	0.116	1	1	PSAP
Pubmed	19307991	CD40-activated apoptotic tumor cell-pulsed dendritic cell could potentially elicit antitumor immune response: involvement of up-regulation of B7-H3 expression.	GeneRIF	0.0044	65.1	0.0114	0.116	1	1	CD276
Pubmed	20549940	Physical and functional interaction between integrins and hERG1 channels in cancer cells.	GeneRIF	0.0044	65.1	0.0114	0.116	1	1	KCNH2
Pubmed	15746535	Tissue transglutaminase and celiac disease.	GeneRIF	0.0044	65.1	0.0114	0.116	1	1	TGM2
Pubmed	22064324	Bone morphogenic protein-4 induces endothelial cell apoptosis through oxidative stress-dependent p38MAPK and JNK pathway.	GeneRIF	0.0044	65.1	0.0114	0.116	1	1	BMP4
Pubmed	23598854	Expression of p75 neurotrophin receptor in desmoplastic trichoepithelioma, infiltrative basal cell carcinoma, and microcystic adnexal carcinoma.	Pubmed	0.0044	65.1	0.0114	0.116	1	1	NGFR
Pubmed	8519693	Inhibition of gamma-glutamyl transpeptidase activity at the surface of human myeloid cells is correlated with macrophage maturation and transforming growth factor beta production.	Pubmed	0.0044	65.1	0.0114	0.116	1	1	GGT1
Pubmed	6952222	Isolation and identification of a cDNA clone corresponding to an HLA-DR antigen beta chain.	Pubmed	0.0044	65.1	0.0114	0.116	1	1	HLA-DQB1
Pubmed	20198449	Activation of CD74 inhibits migration of human mesenchymal stem cells.	GeneRIF	0.0044	65.1	0.0114	0.116	1	1	CD74
Pubmed	19863546	Novel PORCN mutations in focal dermal hypoplasia.	GeneRIF	0.0044	65.1	0.0114	0.116	1	1	PORCN
Pubmed	23110108	The inhibition of KCa3.1 channels activity reduces cell motility in glioblastoma derived cancer stem cells.	GeneRIF	0.0044	65.1	0.0114	0.116	1	1	KCNN4
Pubmed	22625666	Hb S [ß6(A3)Glu¿Val, GAG>GTG] and ß-globin gene cluster haplotype distribution in Mauritania.	GeneRIF	0.0044	65.1	0.0114	0.116	1	1	HBB
Pubmed	24336891	Fractalkine promotes platelet activation and vascular dysfunction in congestive heart failure.	Pubmed	0.0044	65.1	0.0114	0.116	1	1	CX3CL1
Pubmed	9395522	Characterization and purification of human corneodesmosin, an epidermal basic glycoprotein associated with corneocyte-specific modified desmosomes.	Pubmed	0.0044	65.1	0.0114	0.116	1	1	CDSN
Pubmed	25370833	Angiopoietin-like protein 2 induces androgen-independent and malignant behavior in human prostate cancer cells.	Pubmed	0.0044	65.1	0.0114	0.116	1	1	ANGPTL2
Pubmed	1577484	Assignment of human erythroid delta-aminolevulinate synthase (ALAS2) to a distal subregion of band Xp11.21 by PCR analysis of somatic cell hybrids containing X; autosome translocations.	Pubmed	0.0044	65.1	0.0114	0.116	1	1	ALAS2
Pubmed	23449393	Stra6, a retinoic acid-responsive gene, participates in p53-induced apoptosis after DNA damage.	Pubmed	0.0044	65.1	0.0114	0.116	1	1	STRA6
Pubmed	12151390	New binding site on common molecular scaffold provides HERG channel specificity of scorpion toxin BeKm-1.	GeneRIF	0.0044	65.1	0.0114	0.116	1	1	KCNH2
Pubmed	22874716	TSPO-specific ligand vinpocetine exerts a neuroprotective effect by suppressing microglial inflammation.	GeneRIF	0.0044	65.1	0.0114	0.116	1	1	TSPO
Pubmed	12773623	The hSK4 (KCNN4) isoform is the Ca2+-activated K+ channel (Gardos channel) in human red blood cells.	GeneRIF	0.0044	65.1	0.0114	0.116	1	1	KCNN4
Pubmed	9511785	The long QT syndrome: ion channel diseases of the heart.	Pubmed	0.0044	65.1	0.0114	0.116	1	1	KCNH2
Pubmed	17668061	Cathepsin K null mice show reduced adiposity during the rapid accumulation of fat stores.	GeneRIF	0.0044	65.1	0.0114	0.116	1	1	CTSK
Pubmed	12230512	Targeted disruption of dermatopontin causes abnormal collagen fibrillogenesis.	GeneRIF	0.0044	65.1	0.0114	0.116	1	1	DPT
Pubmed	25132819	Inhibiting lung lining fluid glutathione metabolism with GGsTop as a novel treatment for asthma.	Pubmed	0.0044	65.1	0.0114	0.116	1	1	GGT1
Pubmed	17609251	Identification of two GTP-independent alternatively spliced forms of tissue transglutaminase in human leukocytes, vascular smooth muscle, and endothelial cells.	GeneRIF	0.0044	65.1	0.0114	0.116	1	1	TGM2
Pubmed	21606539	The relation of HLA genotype to hepatitis C viral load and markers of liver fibrosis in HIV-infected and HIV-uninfected women.	GeneRIF	0.0044	65.1	0.0114	0.116	1	1	HLA-DQB1

Category	ID	Name	Source	p-value	q-value Bonferroni	q-value FDR B&H	q-value FDR B&Y	Hit Count in Query List	Hit Count in Genome	Hit in Query List
Pubmed	19026638	Meta-analysis of the association of the HTRA1 polymorphisms with the risk of age-related macular degeneration.	GeneRIF	0.0044	65.1	0.0114	0.116	1	1	HTRA1
Pubmed	23500448	Distribution of beta-globin haplotypes among the tribes of southern Gujarat, India.	GeneRIF	0.0044	65.1	0.0114	0.116	1	1	HBB
Pubmed	22040987	[Clinical significance of serum NGF level and CD271 expression in patients with acute B lymphoid leukemia].	GeneRIF	0.0044	65.1	0.0114	0.116	1	1	NGFR
Pubmed	16378742	A structure-function study of MID1 mutations associated with a mild Opitz phenotype.	GeneRIF	0.0044	65.1	0.0114	0.116	1	1	MID1
Pubmed	16951226	The biological role of the low-affinity p75 neurotrophin receptor in esophageal squamous cell carcinoma.	GeneRIF	0.0044	65.1	0.0114	0.116	1	1	NGFR
Pubmed	11766880	Carboxypeptidases from A to z: implications in embryonic development and Wnt binding.	Pubmed	0.0044	65.1	0.0114	0.116	1	1	CPZ
Pubmed	12414983	Compromised influenza virus-specific CD8(+)-T-cell memory in CD4(+)-T-cell-deficient mice.	Pubmed	0.0044	65.1	0.0114	0.116	1	1	HLA-DQB1
Pubmed	11234777	Cloning of the mouse dysferlin gene and genomic characterization of the SJL-Dysf mutation.	Pubmed	0.0044	65.1	0.0114	0.116	1	1	DYSF
Pubmed	18155707	Substitution of aspartic acid at position 57 of the DQbeta1 affects relapse of autoimmune pancreatitis.	GeneRIF	0.0044	65.1	0.0114	0.116	1	1	HLA-DQB1
Pubmed	19434540	[Expression of TROP2 mRNA in left-sided and right-sided colon cancer and its clinical significance].	GeneRIF	0.0044	65.1	0.0114	0.116	1	1	TACSTD2
Pubmed	12439720	The androgen-regulated gene human kallikrein 15 (KLK15) is an independent and favourable prognostic marker for breast cancer.	GeneRIF	0.0044	65.1	0.0114	0.116	1	1	KLK15
Pubmed	3478817	Regional localization of the gene coding for sphingolipid activator protein SAP-1 on human chromosome 10.	Pubmed	0.0044	65.1	0.0114	0.116	1	1	PSAP
Pubmed	2634671	Hb N-Timone [alpha 2 beta 2(8)(A5)Lys----Glu]: a new fast-moving variant with normal stability and oxygen affinity.	Pubmed	0.0044	65.1	0.0114	0.116	1	1	HBB
Pubmed	16945536	Severe nemaline myopathy caused by mutations of the stop codon of the skeletal muscle alpha actin gene (ACTA1).	GeneRIF	0.0044	65.1	0.0114	0.116	1	1	ACTA1
Pubmed	23053022	Divergent results induced by different types of septic shock in transglutaminase 2 knockout mice.	GeneRIF	0.0044	65.1	0.0114	0.116	1	1	TGM2
Pubmed	19072833	p75 Neurotrophin receptor signaling regulates hepatic myofibroblast proliferation and apoptosis in recovery from rodent liver fibrosis.	GeneRIF	0.0044	65.1	0.0114	0.116	1	1	NGFR
Pubmed	11864972	Salt-inducible kinase represses cAMP-dependent protein kinase-mediated activation of human cholesterol side chain cleavage cytochrome P450 promoter through the CREB basic leucine zipper domain.	GeneRIF	0.0044	65.1	0.0114	0.116	1	1	CYP11A1
Pubmed	21899153	[Dementia: progress in diagnosis and treatment; Topics, V. Recent topics; 4. Detection of novel dementia-related genes; 2) Dysregulation of TGF-beta family signaling and hereditary cerebral small vessel disease: insight into molecular pathogenesis of CARASIL].	GeneRIF	0.0044	65.1	0.0114	0.116	1	1	HTRA1
Pubmed	24115033	Interleukin 6 stimulates endothelial binding and transport of high-density lipoprotein through induction of endothelial lipase.	Pubmed	0.0044	65.1	0.0114	0.116	1	1	LIPG
Pubmed	17654071	The molecular analysis of beta-thalassemia mutations in Lorestan Province, Iran.	GeneRIF	0.0044	65.1	0.0114	0.116	1	1	HBB
Pubmed	21641406	Secretory leukocyte protease inhibitor (SLPI) expression and tumor invasion in oral squamous cell carcinoma.	GeneRIF	0.0044	65.1	0.0114	0.116	1	1	SLPI
Pubmed	21099701	Familial pycnodysostosis: identification of a novel mutation in the CTSK gene (cathepsin K).	GeneRIF	0.0044	65.1	0.0114	0.116	1	1	CTSK
Pubmed	17696644	The p75 neurotrophin receptor is a central regulator of glioma invasion.	GeneRIF	0.0044	65.1	0.0114	0.116	1	1	NGFR
Pubmed	8806651	Heterodimerization preferences of thyroid hormone receptor alpha isoforms.	Pubmed	0.0044	65.1	0.0114	0.116	1	1	THRA
Pubmed	18197	Purification and some properties of gamma-glutamyltransferase from human liver.	Pubmed	0.0044	65.1	0.0114	0.116	1	1	GGT1
Pubmed	19125374	Effects of progesterone on total brain tissue adenosine deaminase activity in experimental epilepsy.	GeneRIF	0.0044	65.1	0.0114	0.116	1	1	ADA
Pubmed	22720081	Homologous recombination mediates functional recovery of dysferlin deficiency following AAV5 gene transfer.	GeneRIF	0.0044	65.1	0.0114	0.116	1	1	DYSF
Pubmed	18689502	p75 neurotrophin receptor regulates agonist-induced pulmonary vasoconstriction.	GeneRIF	0.0044	65.1	0.0114	0.116	1	1	NGFR
Pubmed	25443284	Reduction of p75 neurotrophin receptor ameliorates the cognitive deficits in a model of Alzheimer's disease.	Pubmed	0.0044	65.1	0.0114	0.116	1	1	NGFR
Pubmed	10849755	Cathepsin K expression in human lung.	Pubmed	0.0044	65.1	0.0114	0.116	1	1	CTSK
Pubmed	2868718	Molecular cloning of the sphingolipid activator protein-1 (SAP-1), the sulfatide sulfatase activator.	Pubmed	0.0044	65.1	0.0114	0.116	1	1	PSAP
Pubmed	16855103	Interaction of survival and death signaling in basal forebrain neurons: roles of neurotrophins and proneurotrophins.	Pubmed	0.0044	65.1	0.0114	0.116	1	1	NGFR
Pubmed	12144057	Beta-thalassemia intermedia from southern Iran: IVS-II-1 (G-->A) is the prevalent thalassemia intermedia allele.	Pubmed	0.0044	65.1	0.0114	0.116	1	1	HBB
Pubmed	20961458	The p75 neurotrophin receptor is expressed by adult mouse dentate progenitor cells and regulates neuronal and non-neuronal cell genesis.	GeneRIF	0.0044	65.1	0.0114	0.116	1	1	NGFR
Pubmed	20147632	Adenosine deaminase modulation of telomerase activity and replicative senescence in human CD8 T lymphocytes.	GeneRIF	0.0044	65.1	0.0114	0.116	1	1	ADA
Pubmed	22147010	Thyroid hormone receptor-a gene knockout mice are protected from diet-induced hepatic insulin resistance.	GeneRIF	0.0044	65.1	0.0114	0.116	1	1	THRA
Pubmed	17492940	The chondroitin sulfate form of invariant chain trimerizes with conventional invariant chain and these complexes are rapidly transported from the trans-Golgi network to the cell surface.	GeneRIF	0.0044	65.1	0.0114	0.116	1	1	CD74

Category	ID	Name	Source	p-value	q-value Bonferroni	q-value FDR B&H	q-value FDR B&Y	Hit Count in Query List	Hit Count in Genome	Hit in Query List
Pubmed	22935172	HtrA1 expression associated with the occurrence and development of esophageal cancer.	GeneRIF	0.0044	65.1	0.0114	0.116	1	1	HTRA1
Pubmed	19841742	BMP signaling mediates effects of exercise on hippocampal neurogenesis and cognition in mice.	GeneRIF	0.0044	65.1	0.0114	0.116	1	1	BMP4
Pubmed	22691496	Acetylation modulates cellular distribution and DNA sensing ability of interferon-inducible protein IFI16.	GeneRIF	0.0044	65.1	0.0114	0.116	1	1	IFI16
Pubmed	16800732	Human kallikrein 10, a predictive marker for breast cancer.	GeneRIF	0.0044	65.1	0.0114	0.116	1	1	KLK10
Pubmed	18182741	A novel SPINK1 gene mutation, c.206C>T, in a Thai patient with chronic alcoholic pancreatitis.	GeneRIF	0.0044	65.1	0.0114	0.116	1	1	SPINK1
Pubmed	15936242	Involvement of nuclear factor kappaB in up-regulation of aldose reductase gene expression by 12-O-tetradecanoylphorbol-13-acetate in HeLa cells.	GeneRIF	0.0044	65.1	0.0114	0.116	1	1	AKR1B10
Pubmed	15769477	Nuclear location-dependent role of peripheral benzodiazepine receptor (PBR) in hepatic tumoral cell lines proliferation.	GeneRIF	0.0044	65.1	0.0114	0.116	1	1	TSPO
Pubmed	11135002	TrkA-expressing trigeminal sensory neurons display both neurochemical and structural plasticity despite a loss of p75NTR function: responses to normal and elevated levels of nerve growth factor.	Pubmed	0.0044	65.1	0.0114	0.116	1	1	NGFR
Pubmed	2881947	Molecular cloning of a polymorphic DNA endonuclease fragment associates insulin-dependent diabetes mellitus with HLA-DQ.	Pubmed	0.0044	65.1	0.0114	0.116	1	1	HLA-DQB1
Pubmed	8307574	The human peripheral benzodiazepine receptor gene: cloning and characterization of alternative splicing in normal tissues and in a patient with congenital lipoid adrenal hyperplasia.	Pubmed	0.0044	65.1	0.0114	0.116	1	1	TSPO
Pubmed	10187793	Long QT syndrome-associated mutations in the Per-Arnt-Sim (PAS) domain of HERG potassium channels accelerate channel deactivation.	Pubmed	0.0044	65.1	0.0114	0.116	1	1	KCNH2
Pubmed	21191406	Inflammatory peeling skin syndrome caused by a mutation in CDSN encoding corneodesmosin.	GeneRIF	0.0044	65.1	0.0114	0.116	1	1	CDSN
Pubmed	12411602	The Opitz syndrome gene Mid1 is transcribed from a human endogenous retroviral promoter.	GeneRIF	0.0044	65.1	0.0114	0.116	1	1	MID1
Pubmed	21135103	Mechanistic insight into human ether-à-go-go-related gene (hERG) K+ channel deactivation gating from the solution structure of the EAG domain.	GeneRIF	0.0044	65.1	0.0114	0.116	1	1	KCNH2
Pubmed	1487420	Hb Graz or alpha 2 beta 2(2)(NA2)His-->Leu; a new beta chain variant observed in four families from southern Austria.	Pubmed	0.0044	65.1	0.0114	0.116	1	1	HBB
Pubmed	21860532	Is adenosine deaminase in pleural fluid a useful marker for differentiating tuberculosis from lung cancer or mesothelioma in Japan, a country with intermediate incidence of tuberculosis?	GeneRIF	0.0044	65.1	0.0114	0.116	1	1	ADA
Pubmed	18467695	Pharmacological blockade of A2A receptors prevents dermal fibrosis in a model of elevated tissue adenosine.	Pubmed	0.0044	65.1	0.0114	0.116	1	1	ADA
Pubmed	23723371	Endothelial lipase is a critical determinant of high-density lipoprotein-stimulated sphingosine 1-phosphate-dependent signaling in vascular endothelium.	GeneRIF	0.0044	65.1	0.0114	0.116	1	1	LIPG
Pubmed	20718862	Specific cleavage of the DNase-I binding loop dramatically decreases the thermal stability of actin.	GeneRIF	0.0044	65.1	0.0114	0.116	1	1	ACTA1
Pubmed	2320574	Characterization of a mutation in a family with saposin B deficiency: a glycosylation site defect.	Pubmed	0.0044	65.1	0.0114	0.116	1	1	PSAP
Pubmed	23869763	Effects of bone morphogenetic protein-4 (BMP-4) on adipocyte differentiation from mouse adipose-derived stem cells.	Pubmed	0.0044	65.1	0.0114	0.116	1	1	BMP4
Pubmed	16078082	Sequence variations in the transcriptional regulatory region and intron1 of HLA-DQB1 gene and their linkage in southern Chinese ethnic groups.	GeneRIF	0.0044	65.1	0.0114	0.116	1	1	HLA-DQB1
Pubmed	15694941	Association between serum gamma-glutamyltransferase and C-reactive protein.	GeneRIF	0.0044	65.1	0.0114	0.116	1	1	GGT1
Pubmed	17942544	Friend virus utilizes the BMP4-dependent stress erythropoiesis pathway to induce erythroleukemia.	GeneRIF	0.0044	65.1	0.0114	0.116	1	1	BMP4
Pubmed	22216176	Erythroid-specific expression of ß-globin from Sleeping Beauty-transduced human hematopoietic progenitor cells.	GeneRIF	0.0044	65.1	0.0114	0.116	1	1	HBB
Pubmed	22526679	Microbicidal activity of vascular peroxidase 1 in human plasma via generation of hypochlorous acid.	GeneRIF	0.0044	65.1	0.0114	0.116	1	1	PXDN
Pubmed	18344542	Chemical modulation of the chaperone function of human alphaA-crystallin.	GeneRIF	0.0044	65.1	0.0114	0.116	1	1	CRYAA
Pubmed	1268216	Haemoglobin Spanish Town alpha27 Glu replaced by Val (B8).	Pubmed	0.0044	65.1	0.0114	0.116	1	1	HBA2
Pubmed	22675570	Unspliced precursors of NMD-sensitive ß-globin transcripts exhibit decreased steady-state levels in erythroid cells.	GeneRIF	0.0044	65.1	0.0114	0.116	1	1	HBB
Pubmed	11495925	Tumor necrosis factor-alpha-converting enzyme (ADAM17) mediates the cleavage and shedding of fractalkine (CX3CL1).	Pubmed	0.0044	65.1	0.0114	0.116	1	1	CX3CL1
Pubmed	22912767	Silencing of GPNMB by siRNA inhibits the formation of melanosomes in melanocytes in a MITF-independent fashion.	GeneRIF	0.0044	65.1	0.0114	0.116	1	1	GPNMB
Pubmed	18987434	Molecular determinants of hERG channel block by terfenadine and cisapride.	GeneRIF	0.0044	65.1	0.0114	0.116	1	1	KCNH2
Pubmed	8144978	BUB/BnJ (H-2q) is a TCR deletion mutant mouse strain (TCR V beta a, KJ16-) that is susceptible to type II collagen-induced arthritis.	Pubmed	0.0044	65.1	0.0114	0.116	1	1	HLA-DQB1
Pubmed	6505702	Hemoglobin I mutation encoded at both alpha-globin loci on the same chromosome: concerted evolution in the human genome.	Pubmed	0.0044	65.1	0.0114	0.116	1	1	HBA2

Category	ID	Name	Source	p-value	q-value Bonferroni	q-value FDR B&H	q-value FDR B&Y	Hit Count in Query List	Hit Count in Genome	Hit in Query List
Pubmed	19729120	A human atrial natriuretic peptide gene mutation reveals a novel peptide with enhanced blood pressure-lowering, renal-enhancing, and aldosterone-suppressing actions.	GeneRIF	0.0044	65.1	0.0114	0.116	1	1	NPPA
Pubmed	23526947	Orosomucoid, a new biomarker in the association between obesity and periodontitis.	GeneRIF	0.0044	65.1	0.0114	0.116	1	1	ORM1
Pubmed	11812133	Micromechanics of isolated sickle cell hemoglobin fibers: bending moduli and persistence lengths.	GeneRIF	0.0044	65.1	0.0114	0.116	1	1	HBB
Pubmed	15181157	Molecular basis of slow activation of the human ether-a-go-go related gene potassium channel.	GeneRIF	0.0044	65.1	0.0114	0.116	1	1	KCNH2
Pubmed	9151756	The low-affinity neurotrophin receptor p75 regulates the function but not the selective survival of specific subpopulations of sensory neurons.	Pubmed	0.0044	65.1	0.0114	0.116	1	1	NGFR
Pubmed	12883099	High plasma levels of N-terminal pro-atrial natriuretic peptide associated with low anxiety in severe heart failure.	GeneRIF	0.0044	65.1	0.0114	0.116	1	1	NPPA
Pubmed	18692071	The crystal and molecular structures of a cathepsin K:chondroitin sulfate complex.	GeneRIF	0.0044	65.1	0.0114	0.116	1	1	CTSK
Pubmed	16142384	Expression of matrix metalloproteinases and tissue inhibitors of metalloproteinase in uterine endometrial carcinoma and a correlation between expression of matrix metalloproteinase-7 and prognosis.	GeneRIF	0.0044	65.1	0.0114	0.116	1	1	MMP7
Pubmed	10519135	Molecular and functional characterization of the small Ca(2+)-regulated K+ channel (rSK4) of colonic crypts.	Pubmed	0.0044	65.1	0.0114	0.116	1	1	KCNN4
Pubmed	22487487	Is the clinical malignant phenotype of prostate cancer a result of a highly proliferative immune-evasive B7-H3-expressing cell population?	GeneRIF	0.0044	65.1	0.0114	0.116	1	1	CD276
Pubmed	24831840	CD271 identifies functional human hepatic stellate cells, which localize in peri-sinusoidal and portal areas in liver after partial hepatectomy.	Pubmed	0.0044	65.1	0.0114	0.116	1	1	NGFR
Pubmed	21398278	Genetic dissection of the Gpnmb network in the eye.	GeneRIF	0.0044	65.1	0.0114	0.116	1	1	GPNMB
Pubmed	21787888	Thermodynamic analysis of ionizable groups involved in the catalytic mechanism of human matrix metalloproteinase 7 (MMP-7).	GeneRIF	0.0044	65.1	0.0114	0.116	1	1	MMP7
Pubmed	17219958	ANP T2238C, C-664G gene polymorphism and coronary heart diseasein chinese population.	GeneRIF	0.0044	65.1	0.0114	0.116	1	1	NPPA
Pubmed	15365094	Mechanism of block of hEag1 K+ channels by imipramine and astemizole.	GeneRIF	0.0044	65.1	0.0114	0.116	1	1	KCNH2
Pubmed	8299233	Homozygosity for a missense mutation (G20R) associated with neonatal onset adenosine deaminase-deficient severe combined immunodeficiency (ADA-SCID).	Pubmed	0.0044	65.1	0.0114	0.116	1	1	ADA
Pubmed	19071209	Studies on the protective role of lycopene against polychlorinated biphenyls (Aroclor 1254)-induced changes in StAR protein and cytochrome P450 scc enzyme expression on Leydig cells of adult rats.	GeneRIF	0.0044	65.1	0.0114	0.116	1	1	CYP11A1
Pubmed	19411705	A naturally occurring variant of endothelial lipase associated with elevated HDL exhibits impaired synthesis.	GeneRIF	0.0044	65.1	0.0114	0.116	1	1	LIPG
Pubmed	24478068	Protection from Clostridium difficile infection in CD4 T Cell- and polymeric immunoglobulin receptor-deficient mice.	Pubmed	0.0044	65.1	0.0114	0.116	1	1	HLA-DQB1
Pubmed	20662825	The action of a novel fluoroquinolone antibiotic agent antofloxacin hydrochloride on human-ether-a-go-go-related gene potassium channel.	GeneRIF	0.0044	65.1	0.0114	0.116	1	1	KCNH2
Pubmed	17114428	Loss of invariant chain protects nonobese diabetic mice against type 1 diabetes.	Pubmed	0.0044	65.1	0.0114	0.116	1	1	CD74
Pubmed	2895927	Use of synthetic oligonucleotides for genomic DNA dot hybridization to split the DQw3 haplotype.	Pubmed	0.0044	65.1	0.0114	0.116	1	1	HLA-DQB1
Pubmed	12393718	Absent phenotypic expression of X-linked sideroblastic anemia in one of 2 brothers with a novel ALAS2 mutation.	Pubmed	0.0044	65.1	0.0114	0.116	1	1	ALAS2
Pubmed	12431979	Cell cycle-dependent expression of HERG1 and HERG1B isoforms in tumor cells.	GeneRIF	0.0044	65.1	0.0114	0.116	1	1	KCNH2
Pubmed	20007578	Novel role for aldose reductase in mediating acute inflammatory responses in the lung.	GeneRIF	0.0044	65.1	0.0114	0.116	1	1	AKR1B10
Pubmed	21874011	Cathepsin K expression in the spectrum of perivascular epithelioid cell (PEC) lesions of the kidney.	GeneRIF	0.0044	65.1	0.0114	0.116	1	1	CTSK
Pubmed	18362022	Recurrent intrauterine fetal loss due to near absence of HERG: clinical and functional characterization of a homozygous nonsense HERG Q1070X mutation.	GeneRIF	0.0044	65.1	0.0114	0.116	1	1	KCNH2
Pubmed	22735390	A case of almost unilateral focal dermal hypoplasia resulting from a novel mutation in the PORCN gene.	GeneRIF	0.0044	65.1	0.0114	0.116	1	1	PORCN
Pubmed	2298916	Identification of a highly mobilizable subset of human neutrophil intracellular vesicles that contains tetranectin and latent alkaline phosphatase.	Pubmed	0.0044	65.1	0.0114	0.116	1	1	CLEC3B
Pubmed	15236405	Heterogeneity of nemaline myopathy cases with skeletal muscle alpha-actin gene mutations.	GeneRIF	0.0044	65.1	0.0114	0.116	1	1	ACTA1
Pubmed	16769794	Na+ permeation and block of hERG potassium channels.	GeneRIF	0.0044	65.1	0.0114	0.116	1	1	KCNH2
Pubmed	11017147	Secretory leukocyte protease inhibitor mediates non-redundant functions necessary for normal wound healing.	Pubmed	0.0044	65.1	0.0114	0.116	1	1	SLPI
Pubmed	2494138	No difference in the nucleotide sequence of the DQ beta beta 1 domain between narcoleptic and healthy individuals with DR2,Dw2.	Pubmed	0.0044	65.1	0.0114	0.116	1	1	HLA-DQB1
Pubmed	23940627	B7-H3 is overexpressed in patients suffering osteosarcoma and associated with tumor aggressiveness and metastasis.	Pubmed	0.0044	65.1	0.0114	0.116	1	1	CD276
Pubmed	19617705	Extracellular potassium dependency of block of HERG by quinidine and cisapride is primarily determined by the permeant ion and not by inactivation.	GeneRIF	0.0044	65.1	0.0114	0.116	1	1	KCNH2

Category	ID	Name	Source	p-value	q-value Bonferroni	q-value FDR B&H	q-value FDR B&Y	Hit Count in Query List	Hit Count in Genome	Hit in Query List
Pubmed	16059633	Differential effect of BMP4 on NIH/3T3 and C2C12 cells: implications for endochondral bone formation.	GeneRIF	0.0044	65.1	0.0114	0.116	1	1	BMP4
Pubmed	20507645	Two four-marker haplotypes on 7q36.1 region indicate that the potassium channel gene HERG1 (KCNH2, Kv11.1) is related to schizophrenia: a case control study.	GeneRIF	0.0044	65.1	0.0114	0.116	1	1	KCNH2
Pubmed	24089555	The viral ubiquitin ligase ICP0 is neither sufficient nor necessary for degradation of the cellular DNA sensor IFI16 during herpes simplex virus 1 infection.	Pubmed	0.0044	65.1	0.0114	0.116	1	1	IFI16
Pubmed	12176042	Expression of a connexin31 mutation causing erythrokeratodermia variabilis is lethal for HeLa cells.	GeneRIF	0.0044	65.1	0.0114	0.116	1	1	GJB3
Pubmed	24362517	Mitogen-activated protein kinase/I¿B kinase/NF-¿B-dependent and AP-1-independent CX3CL1 expression in intestinal epithelial cells stimulated with Clostridium difficile toxin A.	Pubmed	0.0044	65.1	0.0114	0.116	1	1	CX3CL1
Pubmed	8330979	Hb D-Neath or beta 121 (GH4) Glu-->Ala: a new member of the Hb D family.	Pubmed	0.0044	65.1	0.0114	0.116	1	1	HBB
Pubmed	11701474	Allele-specific regulation of matrix metalloproteinase-7 promoter activity is associated with coronary artery luminal dimensions among hypercholesterolemic patients.	GeneRIF	0.0044	65.1	0.0114	0.116	1	1	MMP7
Pubmed	23255486	Identification of a novel oligomerization disrupting mutation in CRY¿A associated with congenital cataract in a South Australian family.	GeneRIF	0.0044	65.1	0.0114	0.116	1	1	CRYAA
Pubmed	17044040	Prosaposin upregulates AR and PSA expression and activity in prostate cancer cells (LNCaP).	GeneRIF	0.0044	65.1	0.0114	0.116	1	1	PSAP
Pubmed	1929395	Purification and characterization of a trypsin inhibitor from mouse seminal vesicle secretion.	Pubmed	0.0044	65.1	0.0114	0.116	1	1	SPINK1
Pubmed	12052474	Evaluation of the atrial natriuretic peptide gene in stroke.	GeneRIF	0.0044	65.1	0.0114	0.116	1	1	NPPA
Pubmed	11274171	Targeted inactivation of Gh/tissue transglutaminase II.	Pubmed	0.0044	65.1	0.0114	0.116	1	1	TGM2
Pubmed	24522256	Fibulin-2 is involved in early extracellular matrix development of the outgrowing mouse mammary epithelium.	Pubmed	0.0044	65.1	0.0114	0.116	1	1	FBLN2
Pubmed	12680838	Low-affinity neurotrophin receptor with targeted mutation of exon 3 is capable of mediating the death of axotomized neurons.	Pubmed	0.0044	65.1	0.0114	0.116	1	1	NGFR
Pubmed	19152657	Impaired cutaneous wound healing in mice lacking tetranectin.	Pubmed	0.0044	65.1	0.0114	0.116	1	1	CLEC3B
Pubmed	20810886	Rab11 and its effector Rab coupling protein contribute to the trafficking of beta 1 integrins during axon growth in adult dorsal root ganglion neurons and PC12 cells.	GeneRIF	0.0044	65.1	0.0114	0.116	1	1	RAB11FIP1
Pubmed	11006246	A nonsense mutation (W9X) in CRYAA causes autosomal recessive cataract in an inbred Jewish Persian family.	Pubmed	0.0044	65.1	0.0114	0.116	1	1	CRYAA
Pubmed	18367588	IK1 channel activity contributes to cisplatin sensitivity of human epidermoid cancer cells.	GeneRIF	0.0044	65.1	0.0114	0.116	1	1	KCNN4
Pubmed	14573606	Mechanism of p21-activated kinase 6-mediated inhibition of androgen receptor signaling.	Pubmed	0.0044	65.1	0.0114	0.116	1	1	PAK6
Pubmed	18559421	Identification of a posttranslational mechanism for the regulation of hERG1 K+ channel expression and hERG1 current density in tumor cells.	GeneRIF	0.0044	65.1	0.0114	0.116	1	1	KCNH2
Pubmed	12638126	mRNA expression of the murine glycoprotein (transmembrane) nmb (Gpnmb) gene is linked to the developing retinal pigment epithelium and iris.	GeneRIF	0.0044	65.1	0.0114	0.116	1	1	GPNMB
Pubmed	25200161	[Single nucleotide polymorphisms of B7-H3 gene in blood cells of patients with rheumatoid arthritis].	Pubmed	0.0044	65.1	0.0114	0.116	1	1	CD276
Pubmed	12759437	Wound healing and expression of antimicrobial peptides/polypeptides in human keratinocytes, a consequence of common growth factors.	Pubmed	0.0044	65.1	0.0114	0.116	1	1	SLPI
Pubmed	8611626	A third human tissue transglutaminase homologue as a result of alternative gene transcripts.	Pubmed	0.0044	65.1	0.0114	0.116	1	1	TGM2
Pubmed	7827052	Sulfate-activating enzymes in normal and brachymorphic mice: evidence for a channeling defect.	Pubmed	0.0044	65.1	0.0114	0.116	1	1	PAPSS2
Pubmed	21931536	Natural killer cell lytic granule secretion occurs through a pervasive actin network at the immune synapse.	GeneRIF	0.0044	65.1	0.0114	0.116	1	1	ACTA1
Pubmed	3366116	The 2.5 A X-ray crystal structure of the acid-stable proteinase inhibitor from human mucous secretions analysed in its complex with bovine alpha-chymotrypsin.	Pubmed	0.0044	65.1	0.0114	0.116	1	1	SLPI
Pubmed	20837713	Mycobacterium tuberculosis synergizes with ATP to induce release of microvesicles and exosomes containing major histocompatibility complex class II molecules capable of antigen presentation.	Pubmed	0.0044	65.1	0.0114	0.116	1	1	HLA-DQB1
Pubmed	2890689	Molecular variation of human major histocompatibility complex DQw3 beta-chains.	Pubmed	0.0044	65.1	0.0114	0.116	1	1	HLA-DQB1
Pubmed	10940879	Gamma-glutamyltranspeptidase knockout mice as a model for understanding the consequences of diminished glutathione on T cell-dependent immune responses.	Pubmed	0.0044	65.1	0.0114	0.116	1	1	GGT1
Pubmed	10588683	Ancient origins of nitric oxide signaling in biological systems.	Pubmed	0.0044	65.1	0.0114	0.116	1	1	HBB
Pubmed	10386950	Molecular cloning of testican-2: defining a novel calcium-binding proteoglycan family expressed in brain.	Pubmed	0.0044	65.1	0.0114	0.116	1	1	SPOCK2
Pubmed	1968061	An alternatively processed mRNA specific for gamma-glutamyl transpeptidase in human tissues.	Pubmed	0.0044	65.1	0.0114	0.116	1	1	GGT1
Pubmed	20605361	The matrix metalloproteinase-7 polymorphism rs10895304 is associated with increased recurrence risk in patients with clinically localized prostate cancer.	GeneRIF	0.0044	65.1	0.0114	0.116	1	1	MMP7
Pubmed	17275231	The role of bone morphogenetic protein 4 in inner ear development and function.	GeneRIF	0.0044	65.1	0.0114	0.116	1	1	BMP4

Category	ID	Name	Source	p-value	q-value Bonferroni	q-value FDR B&H	q-value FDR B&Y	Hit Count in Query List	Hit Count in Genome	Hit in Query List
Pubmed	23673317	Tissue transglutaminase as a central mediator in inflammation-induced progression of breast cancer.	Pubmed	0.0044	65.1	0.0114	0.116	1	1	TGM2
Pubmed	24582280	The P75 neurotrophin receptor regulates proliferation of the human MG63 osteoblast cell line.	Pubmed	0.0044	65.1	0.0114	0.116	1	1	NGFR
Pubmed	17601882	A combined approach identifies a limited number of new thyroid hormone target genes in post-natal mouse cerebellum.	GeneRIF	0.0044	65.1	0.0114	0.116	1	1	THRA
Pubmed	6402712	The gene encoding the Ia antigen-associated invariant chain (Ii) is not linked to the H-2 complex.	Pubmed	0.0044	65.1	0.0114	0.116	1	1	CD74
Pubmed	25792458	Hypoxic induction of AKAP12 variant 2 shifts PKA-mediated protein phosphorylation to enhance migration and metastasis of melanoma cells.	Pubmed	0.0044	65.1	0.0114	0.116	1	1	AKAP12
Pubmed	25248717	Subtype-specific overexpression of the Rac-GEF P-REX1 in breast cancer is associated with promoter hypomethylation.	Pubmed	0.0044	65.1	0.0114	0.116	1	1	PREX1
Pubmed	20424129	Incorporation of a genetic factor into an epidemiologic model for prediction of individual risk of lung cancer: the Liverpool Lung Project.	GeneRIF	0.0044	65.1	0.0114	0.116	1	1	SEZ6L
Pubmed	6587773	Localization of the beta-globin gene by chromosomal in situ hybridization.	Pubmed	0.0044	65.1	0.0114	0.116	1	1	HBB
Pubmed	21168996	The 3'-UTR AACCins5874 in the stratum corneum chymotryptic enzyme gene (SCCE/KLK7), associated with atopic dermatitis; causes an increased mRNA expression without altering its stability.	GeneRIF	0.0044	65.1	0.0114	0.116	1	1	KLK7
Pubmed	18667446	Tissue transglutaminase protects epithelial ovarian cancer cells from cisplatin-induced apoptosis by promoting cell survival signaling.	GeneRIF	0.0044	65.1	0.0114	0.116	1	1	TGM2
Pubmed	2159298	The I-A beta b region (65-85) is a binding site for the superantigen, staphylococcal enterotoxin A.	Pubmed	0.0044	65.1	0.0114	0.116	1	1	HLA-DQB1
Pubmed	24282288	Secretory leukocyte protease inhibitor modulates urethane-induced lung carcinogenesis.	Pubmed	0.0044	65.1	0.0114	0.116	1	1	SLPI
Pubmed	19039244	Active and passive smoking and risk of narcolepsy in people with HLA DQB1*0602: a population-based case-control study.	GeneRIF	0.0044	65.1	0.0114	0.116	1	1	HLA-DQB1
Pubmed	18616963	The hERG potassium channel and hERG screening for drug-induced torsades de pointes.	Pubmed	0.0044	65.1	0.0114	0.116	1	1	KCNH2
Pubmed	16024538	Fecundability according to male serum inhibin B--a prospective study among first pregnancy planners.	GeneRIF	0.0044	65.1	0.0114	0.116	1	1	INHBB
Pubmed	24878022	ß-Globin gene sequencing of hemoglobin Austin revises the historically reported electrophoretic migration pattern.	Pubmed	0.0044	65.1	0.0114	0.116	1	1	HBB
Pubmed	4123689	Structure of sickled erythrocytes and of sickle-cell hemoglobin fibers.	Pubmed	0.0044	65.1	0.0114	0.116	1	1	HBB
Pubmed	15843175	Enzymatic profiling of human kallikrein 14 using phage-display substrate technology.	GeneRIF	0.0044	65.1	0.0114	0.116	1	1	KLK14
Pubmed	18992638	The presence of the CYP11A1 (TTTTA)6 allele increases the risk of biochemical relapse in organ confined and low-grade prostate cancer.	GeneRIF	0.0044	65.1	0.0114	0.116	1	1	CYP11A1
Pubmed	22562244	The Trop-2 signalling network in cancer growth.	GeneRIF	0.0044	65.1	0.0114	0.116	1	1	TACSTD2
Pubmed	15188376	Induction of arthritis in HLA-DR4-humanized and HLA-DQ8-humanized mice by human cartilage proteoglycan aggrecan but only in the presence of an appropriate (non-MHC) genetic background.	Pubmed	0.0044	65.1	0.0114	0.116	1	1	HLA-DQB1
Pubmed	19958186	A novel polymorphism causes a different restriction pattern by RsaI in the beta-globin gene cluster: application in prenatal diagnosis.	GeneRIF	0.0044	65.1	0.0114	0.116	1	1	HBB
Pubmed	12366792	A novel HLA-DQB1 allele, DQB1*05022, isolated from the Jing ethnic group in South-west China.	Pubmed	0.0044	65.1	0.0114	0.116	1	1	HLA-DQB1
Pubmed	17637845	Intergenic transcription, cell-cycle and the developmentally regulated epigenetic profile of the human beta-globin locus.	GeneRIF	0.0044	65.1	0.0114	0.116	1	1	HBB
Pubmed	3533531	Molecular cloning and expression of cDNA for human antileukoprotease from cervix uterus.	Pubmed	0.0044	65.1	0.0114	0.116	1	1	SLPI
Pubmed	24471820	Case report: prenatal diagnosis of Hb Hammersmith [ß42(CD1)Phe¿Ser; HBB: c.128T¿>¿C] in a family with an adult male patient.	Pubmed	0.0044	65.1	0.0114	0.116	1	1	HBB
Pubmed	2050125	Human erythroid 5-aminolevulinate synthase: promoter analysis and identification of an iron-responsive element in the mRNA.	Pubmed	0.0044	65.1	0.0114	0.116	1	1	ALAS2
Pubmed	19237091	Serum adenosine deaminase activities during acute attacks and attack-free periods of familial Mediterranean fever.	GeneRIF	0.0044	65.1	0.0114	0.116	1	1	ADA
Pubmed	16912123	Cathepsin K in adipocyte differentiation and its potential role in the pathogenesis of obesity.	GeneRIF	0.0044	65.1	0.0114	0.116	1	1	CTSK
Pubmed	9886843	The human RC3 gene homolog, NRGN contains a thyroid hormone-responsive element located in the first intron.	Pubmed	0.0044	65.1	0.0114	0.116	1	1	NRGN
Pubmed	20154589	Pitfalls in the use of smoothelin to identify muscularis propria invasion by urothelial carcinoma.	GeneRIF	0.0044	65.1	0.0114	0.116	1	1	SMTN
Pubmed	21315844	T-wave morphology abnormalities in benign, potent, and arrhythmogenic I(kr) inhibition.	GeneRIF	0.0044	65.1	0.0114	0.116	1	1	KCNH2
Pubmed	6417245	A gene mapping between the S and D regions of the H-2 complex influences resistance to Trichinella spiralis infections of mice.	Pubmed	0.0044	65.1	0.0114	0.116	1	1	HLA-DQB1
Pubmed	12556906	Lack of thyroid hormone receptor alpha1 is associated with selective alterations in behavior and hippocampal circuits.	GeneRIF	0.0044	65.1	0.0114	0.116	1	1	THRA
Pubmed	20300917	MMP-7 overexpression is an independent prognostic marker in gastric cancer.	GeneRIF	0.0044	65.1	0.0114	0.116	1	1	MMP7
Pubmed	15301976	HLA class II and antibody responses to circumsporozoite protein repeats of P. vivax (VK210, VK247 and P. vivax-like) in individuals naturally exposed to malaria.	GeneRIF	0.0044	65.1	0.0114	0.116	1	1	HLA-DQB1
Pubmed	18037420	Serum gamma-glutamyltransferase level and peripheral arterial disease.	GeneRIF	0.0044	65.1	0.0114	0.116	1	1	GGT1

Category	ID	Name	Source	p-value	q-value Bonferroni	q-value FDR B&H	q-value FDR B&Y	Hit Count in Query List	Hit Count in Genome	Hit in Query List
Pubmed	18405667	Extracellular transglutaminase 2 activates beta-catenin signaling in calcifying vascular smooth muscle cells.	Pubmed	0.0044	65.1	0.0114	0.116	1	1	TGM2
Pubmed	2384313	Direct demonstration of the HB Suan-Dok mutation in the alpha 2-globin gene by restriction analysis with Sma I.	Pubmed	0.0044	65.1	0.0114	0.116	1	1	HBA2
Pubmed	1284479	Identification of two new missense mutations (R156C and S291L) in two ADA- SCID patients unusual for response to therapy with partial exchange transfusions.	Pubmed	0.0044	65.1	0.0114	0.116	1	1	ADA
Pubmed	18240253	Cathepsin K deficiency partially inhibits, but does not prevent, bone destruction in human tumor necrosis factor-transgenic mice.	GeneRIF	0.0044	65.1	0.0114	0.116	1	1	CTSK
Pubmed	9892679	The human LARGE gene from 22q12.3-q13.1 is a new, distinct member of the glycosyltransferase gene family.	Pubmed	0.0044	65.1	0.0114	0.116	1	1	LARGE
Pubmed	22914608	Applications and limitations of immunohistochemical expression of "Napsin-A" in distinguishing lung adenocarcinoma from adenocarcinomas of other organs.	GeneRIF	0.0044	65.1	0.0114	0.116	1	1	NAPSA
Pubmed	10791557	Sickle hemoglobin (HbS) allele and sickle cell disease: a HuGE review.	GeneRIF	0.0044	65.1	0.0114	0.116	1	1	HBB
Pubmed	20353351	Hb Perpignan [beta136(H14)Gly-->Ser], a silent variant associated with normal hematology.	GeneRIF	0.0044	65.1	0.0114	0.116	1	1	HBB
Pubmed	21159840	Partial defect in the cholesterol side-chain cleavage enzyme P450scc (CYP11A1) resembling nonclassic congenital lipoid adrenal hyperplasia.	GeneRIF	0.0044	65.1	0.0114	0.116	1	1	CYP11A1
Pubmed	12545276	Duplication of the MID1 first exon in a patient with Opitz G/BBB syndrome.	GeneRIF	0.0044	65.1	0.0114	0.116	1	1	MID1
Pubmed	11683403	Transmission of high-risk HLA-DQB1 alleles in Chilean type 1 diabetic patients and their parents: stratification by the presence of ICA or GAD65 autoantibodies.	GeneRIF	0.0044	65.1	0.0114	0.116	1	1	HLA-DQB1
Pubmed	24838245	Calpain 5 is highly expressed in the central nervous system (CNS), carries dual nuclear localization signals, and is associated with nuclear promyelocytic leukemia protein bodies.	Pubmed	0.0044	65.1	0.0114	0.116	1	1	CAPN5
Pubmed	18294253	Analysis of beta globin mutations in the Indian population: presence of rare and novel mutations and region-wise heterogeneity.	GeneRIF	0.0044	65.1	0.0114	0.116	1	1	HBB
Pubmed	17300864	Aldose reductase regulates TNF-alpha-induced PGE2 production in human colon cancer cells.	GeneRIF	0.0044	65.1	0.0114	0.116	1	1	AKR1B10
Pubmed	21251161	B7-H3 expression in gastric cancer: a novel molecular blood marker for detecting circulating tumor cells.	GeneRIF	0.0044	65.1	0.0114	0.116	1	1	CD276
Pubmed	9296067	Genetics of type 1 diabetes.	Pubmed	0.0044	65.1	0.0114	0.116	1	1	HLA-DQB1
Pubmed	9704026	Human gap junction protein connexin31: molecular cloning and expression analysis.	Pubmed	0.0044	65.1	0.0114	0.116	1	1	GJB3
Pubmed	15845618	Temperature homeostasis in transgenic mice lacking thyroid hormone receptor-alpha gene products.	GeneRIF	0.0044	65.1	0.0114	0.116	1	1	THRA
Pubmed	10954746	Relative role of heme nitrosylation and beta-cysteine 93 nitrosation in the transport and metabolism of nitric oxide by hemoglobin in the human circulation.	Pubmed	0.0044	65.1	0.0114	0.116	1	1	HBB
Pubmed	12603093	Association of Hb G-Chinese [alpha30(B11)Glu --> Gln] with alpha-thalassemia-1 of the Thai type in a Taiwanese family.	Pubmed	0.0044	65.1	0.0114	0.116	1	1	HBA2
Pubmed	16720350	Changes in gene expression with increased transglutaminase 2 in a SH-SY5Y cell line.	GeneRIF	0.0044	65.1	0.0114	0.116	1	1	TGM2
Pubmed	22734587	The +1,506 (A>C) mutation in the 3' untranslated region affects ß-globin expression.	GeneRIF	0.0044	65.1	0.0114	0.116	1	1	HBB
Pubmed	18448157	MMP-7 (-181A>G) promoter polymorphisms and risk for cervical cancer.	GeneRIF	0.0044	65.1	0.0114	0.116	1	1	MMP7
Pubmed	20875798	Neurogranin in cerebrospinal fluid as a marker of synaptic degeneration in Alzheimer's disease.	GeneRIF	0.0044	65.1	0.0114	0.116	1	1	NRGN
Pubmed	7161106	Hemoglobin Palmerston North beta 23 (B5) Val replaced by Phe. A new variant identified in a patient with polycythemia.	Pubmed	0.0044	65.1	0.0114	0.116	1	1	HBB
Pubmed	15269824	Aberrant mucosal wound repair in the absence of secretory leukocyte protease inhibitor.	GeneRIF	0.0044	65.1	0.0114	0.116	1	1	SLPI
Pubmed	12581880	Sequences required for the transition from monomeric to homodimeric forms of thyroid hormone receptor alpha and v-erbA.	GeneRIF	0.0044	65.1	0.0114	0.116	1	1	THRA
Pubmed	3191134	Hb natal or alpha 2(minus Tyr-Arg) beta 2: a high oxygen affinity alpha chain variant with a deleted carboxy-terminus resulting from a TAC----TAA (Tyr----terminating codon) mutation in codon alpha 140.	Pubmed	0.0044	65.1	0.0114	0.116	1	1	HBA2
Pubmed	22220476	Tissue transglutaminase and its role in human cancer progression.	GeneRIF	0.0044	65.1	0.0114	0.116	1	1	TGM2
Pubmed	19695568	Serum gamma-glutamyl transferase and mortality in persons undergoing coronary angiography-The Ludwigshafen Risk and Cardiovascular Health Study.	GeneRIF	0.0044	65.1	0.0114	0.116	1	1	GGT1
Pubmed	17671125	Validation and characterization of human kallikrein 11 as a serum marker for diagnosis of ovarian carcinoma.	GeneRIF	0.0044	65.1	0.0114	0.116	1	1	KLK1
Pubmed	17440814	Tissue transglutaminase induces the release of apoptosis inducing factor and results in apoptotic death of pancreatic cancer cells.	GeneRIF	0.0044	65.1	0.0114	0.116	1	1	TGM2
Pubmed	18005432	Alternative polyadenylation signals and promoters act in concert to control tissue-specific expression of the Opitz Syndrome gene MID1.	GeneRIF	0.0044	65.1	0.0114	0.116	1	1	MID1
Pubmed	3024157	Human cholesterol side-chain cleavage enzyme, P450scc: cDNA cloning, assignment of the gene to chromosome 15, and expression in the placenta.	Pubmed	0.0044	65.1	0.0114	0.116	1	1	CYP11A1
Pubmed	7576232	Human cathepsin O2, a novel cysteine protease highly expressed in osteoclastomas and ovary molecular cloning, sequencing and tissue distribution.	Pubmed	0.0044	65.1	0.0114	0.116	1	1	CTSK
Pubmed	10856721	Intramitochondrial cholesterol transfer.	Pubmed	0.0044	65.1	0.0114	0.116	1	1	CYP11A1

Category	ID	Name	Source	p-value	q-value Bonferroni	q-value FDR B&H	q-value FDR B&Y	Hit Count in Query List	Hit Count in Genome	Hit in Query List
Pubmed	23337730	Varied clinical presentations of seven patients with mutations in CYP11A1 encoding the cholesterol side-chain cleavage enzyme, P450scc.	GeneRIF	0.0044	65.1	0.0114	0.116	1	1	CYP11A1
Pubmed	22629859	[Apoptosis modulation by PMA occurs independently of TsPO expression in squamous cell carcinoma cells].	GeneRIF	0.0044	65.1	0.0114	0.116	1	1	TSPO
Pubmed	20371614	Deletion of murine choline dehydrogenase results in diminished sperm motility.	GeneRIF	0.0044	65.1	0.0114	0.116	1	1	CHDH
Pubmed	21982044	Immunoexpression of B7-H3 in endometrial cancer: relation to tumor T-cell infiltration and prognosis.	GeneRIF	0.0044	65.1	0.0114	0.116	1	1	CD276
Pubmed	8449997	cDNA-derived molecular characteristics and antibodies to a new centrosome-associated and G2/M phase-prevalent protein.	Pubmed	0.0044	65.1	0.0114	0.116	1	1	PROCR
Pubmed	16131613	Anxiety, memory impairment, and locomotor dysfunction caused by a mutant thyroid hormone receptor alpha1 can be ameliorated by T3 treatment.	GeneRIF	0.0044	65.1	0.0114	0.116	1	1	THRA
Pubmed	17093390	Small leucine rich repeat proteoglycans (SLRPs) in the human sclera: identification of abundant levels of PRELP.	GeneRIF	0.0044	65.1	0.0114	0.116	1	1	PRELP
Pubmed	21364639	The anti-apoptotic function of human aA-crystallin is directly related to its chaperone activity.	GeneRIF	0.0044	65.1	0.0114	0.116	1	1	CRYAA
Pubmed	9718340	Opitz G/BBB syndrome in Xp22: mutations in the MID1 gene cluster in the carboxy-terminal domain.	Pubmed	0.0044	65.1	0.0114	0.116	1	1	MID1
Pubmed	19034038	Effects of common antitussive drugs on the hERG potassium channel current.	GeneRIF	0.0044	65.1	0.0114	0.116	1	1	KCNH2
Pubmed	18681786	Molecular basis for statin-induced muscle toxicity: implications and possibilities.	GeneRIF	0.0044	65.1	0.0114	0.116	1	1	FBXO32
Pubmed	11979071	Anetoderma: an altered balance between metalloproteinases and tissue inhibitors of metalloproteinases.	Pubmed	0.0044	65.1	0.0114	0.116	1	1	MMP7
Pubmed	8759518	Differential phosphorylation of alpha-A crystallin in human lens of different age.	Pubmed	0.0044	65.1	0.0114	0.116	1	1	CRYAA
Pubmed	22614334	Proteomics reveals drastic increase of extracellular matrix proteins collagen and dermatopontin in the aged mdx diaphragm model of Duchenne muscular dystrophy.	GeneRIF	0.0044	65.1	0.0114	0.116	1	1	DPT
Pubmed	17171344	A missense mutation (G604S) in the S5/pore region of HERG causes long QT syndrome in a Chinese family with a high incidence of sudden unexpected death.	GeneRIF	0.0044	65.1	0.0114	0.116	1	1	KCNH2
Pubmed	18754677	Structural and functional roles of deamidation and/or truncation of N- or C-termini in human alpha A-crystallin.	GeneRIF	0.0044	65.1	0.0114	0.116	1	1	CRYAA
Pubmed	22249105	Identification of essential sequences for cellular localization in the muscle-specific ubiquitin E3 ligase MAFbx/Atrogin 1.	GeneRIF	0.0044	65.1	0.0114	0.116	1	1	FBXO32
Pubmed	7923625	Smooth muscle myosin heavy chain exclusively marks the smooth muscle lineage during mouse embryogenesis.	Pubmed	0.0044	65.1	0.0114	0.116	1	1	MYH11
Pubmed	11169256	Identification of six novel polymorphisms in the human corneodesmosin gene.	Pubmed	0.0044	65.1	0.0114	0.116	1	1	CDSN
Pubmed	16531622	Mechanism of a hereditary cataract phenotype. Mutations in alphaA-crystallin activate substrate binding.	GeneRIF	0.0044	65.1	0.0114	0.116	1	1	CRYAA
Pubmed	9521756	High-resolution crystal structures of human hemoglobin with mutations at tryptophan 37beta: structural basis for a high-affinity T-state,.	Pubmed	0.0044	65.1	0.0114	0.116	1	1	HBB
Pubmed	23981445	Gestational hypertension in atrial natriuretic peptide knockout mice and the developmental origins of salt-sensitivity and cardiac hypertrophy.	Pubmed	0.0044	65.1	0.0114	0.116	1	1	NPPA
Pubmed	9532134	Islet cell autoimmunity in NOD mice transgenic to HLA-DQ8 and lacking I-Ag7.	Pubmed	0.0044	65.1	0.0114	0.116	1	1	HLA-DQB1
Pubmed	9255611	Hb Watts [alpha 74(EF3) or alpha 75(EF4)Asp-->0]: a shortened alpha chain variant due to the deletion of three nucleotides in exon 2 of the alpha 2-globin gene.	Pubmed	0.0044	65.1	0.0114	0.116	1	1	HBA2
Pubmed	21593056	Tyr219 of human matrix metalloproteinase 7 is not critical for catalytic activity, but is involved in the broad pH-dependence of the activity.	GeneRIF	0.0044	65.1	0.0114	0.116	1	1	MMP7
Pubmed	17453715	Autoimmunity in HLA-DQ8 transgenic mice expressing granulocyte/macrophage-colony stimulating factor in the beta cells of islets of Langerhans.	Pubmed	0.0044	65.1	0.0114	0.116	1	1	HLA-DQB1
Pubmed	18329265	Brain microglia express steroid-converting enzymes in the mouse.	Pubmed	0.0044	65.1	0.0114	0.116	1	1	TSPO
Pubmed	10196377	Identical mutation in patients with limb girdle muscular dystrophy type 2B or Miyoshi myopathy suggests a role for modifier gene(s).	Pubmed	0.0044	65.1	0.0114	0.116	1	1	DYSF
Pubmed	16946716	Role of cathepsin K in the turnover of the dermal extracellular matrix during scar formation.	GeneRIF	0.0044	65.1	0.0114	0.116	1	1	CTSK
Pubmed	21565221	Critical role for invariant chain in CD1d-mediated selection and maturation of Va14-invariant NKT cells.	GeneRIF	0.0044	65.1	0.0114	0.116	1	1	CD74
Pubmed	12453482	Nerve growth factor survival signaling in cultured hippocampal neurons is mediated through TrkA and requires the common neurotrophin receptor P75.	Pubmed	0.0044	65.1	0.0114	0.116	1	1	NGFR
Pubmed	24279989	Second-sphere interactions between the C93-Y157 cross-link and the substrate-bound Fe site influence the O2 coupling efficiency in mouse cysteine dioxygenase.	Pubmed	0.0044	65.1	0.0114	0.116	1	1	CDO1
Pubmed	3691763	Hb Linköping (beta 36 Pro----Thr): a new high oxygen affinity hemoglobin variant found in two families of Finnish origin.	Pubmed	0.0044	65.1	0.0114	0.116	1	1	HBB
Pubmed	7749374	Glandular kallikrein gene expression in the human uterus.	Pubmed	0.0044	65.1	0.0114	0.116	1	1	KLK1
Pubmed	21398580	The CX3C chemokine fractalkine mediates platelet adhesion via the von Willebrand receptor glycoprotein Ib.	GeneRIF	0.0044	65.1	0.0114	0.116	1	1	CX3CL1
Pubmed	16800735	The human kallikrein 10 promoter contains a functional retinoid response element.	GeneRIF	0.0044	65.1	0.0114	0.116	1	1	KLK10

Category	ID	Name	Source	p-value	q-value Bonferroni	q-value FDR B&H	q-value FDR B&Y	Hit Count in Query List	Hit Count in Genome	Hit in Query List
Pubmed	9144234	Intracellular misrouting and abnormal secretion of adrenocorticotropin and growth hormone in cpefat mice associated with a carboxypeptidase E mutation.	Pubmed	0.0044	65.1	0.0114	0.116	1	1	CPE
Pubmed	21349128	Atrial natriuretic peptide is an antiapoptotic factor for human granulosa luteinized cells with impact on the results of COH/IVF in women undergoing IVF program.	GeneRIF	0.0044	65.1	0.0114	0.116	1	1	NPPA
Pubmed	24096005	DCLK1 marks a morphologically distinct subpopulation of cells with stem cell properties in preinvasive pancreatic cancer.	Pubmed	0.0044	65.1	0.0114	0.116	1	1	DCLK1
Pubmed	21276088	High brain-natriuretic peptide level predicts cirrhotic cardiomyopathy in liver transplant patients.	GeneRIF	0.0044	65.1	0.0114	0.116	1	1	NPPA
Pubmed	17962462	A new mouse model of autoimmune ocular myasthenia gravis.	Pubmed	0.0044	65.1	0.0114	0.116	1	1	HLA-DQB1
Pubmed	16254462	Downregulation of human kallikrein 10 (KLK10/NES1) by CpG island hypermethylation in breast, ovarian and prostate cancers.	GeneRIF	0.0044	65.1	0.0114	0.116	1	1	KLK10
Pubmed	23159315	Failure of axonal transport induces a spatially coincident increase in astrocyte BDNF prior to synapse loss in a central target.	Pubmed	0.0044	65.1	0.0114	0.116	1	1	GPNMB
Pubmed	20524821	Phenotypic expression and origin of the rare beta-thalassemia splice site mutation HBB:c.315 + 1G>T.	GeneRIF	0.0044	65.1	0.0114	0.116	1	1	HBB
Pubmed	23332170	Fractalkine overexpression suppresses tau pathology in a mouse model of tauopathy.	Pubmed	0.0044	65.1	0.0114	0.116	1	1	CX3CL1
Pubmed	9893980	The crystal structure of human procathepsin K.	Pubmed	0.0044	65.1	0.0114	0.116	1	1	CTSK
Pubmed	11123904	Structural and functional changes in the alpha A-crystallin R116C mutant in hereditary cataracts.	Pubmed	0.0044	65.1	0.0114	0.116	1	1	CRYAA
Pubmed	16822871	Matrix metalloproteinase-7 activation of mouse paneth cell pro-alpha-defensins: SER43 down arrow ILE44 proteolysis enables membrane-disruptive activity.	GeneRIF	0.0044	65.1	0.0114	0.116	1	1	MMP7
Pubmed	2783588	Identification of a point mutation resulting in a heat-labile adenosine deaminase (ADA) in two unrelated children with partial ADA deficiency.	Pubmed	0.0044	65.1	0.0114	0.116	1	1	ADA
Pubmed	18555797	Aldosterone modifies hemostasis via upregulation of the protein-C receptor in human vascular endothelium.	GeneRIF	0.0044	65.1	0.0114	0.116	1	1	PROCR
Pubmed	18474442	Transglutaminase 2 activity promotes membrane resealing after mechanical damage in the lung cancer cell line A549.	GeneRIF	0.0044	65.1	0.0114	0.116	1	1	TGM2
Pubmed	12145347	Steroid deficiency syndromes in mice with targeted disruption of Cyp11a1.	GeneRIF	0.0044	65.1	0.0114	0.116	1	1	CYP11A1
Pubmed	10583485	Postnatal loss of Merkel cells, but not of slowly adapting mechanoreceptors in mice lacking the neurotrophin receptor p75.	Pubmed	0.0044	65.1	0.0114	0.116	1	1	NGFR
Pubmed	22052838	The solution structure of the S4-S5 linker of the hERG potassium channel.	GeneRIF	0.0044	65.1	0.0114	0.116	1	1	KCNH2
Pubmed	20705797	Smoking-induced upregulation of AKR1B10 expression in the airway epithelium of healthy individuals.	GeneRIF	0.0044	65.1	0.0114	0.116	1	1	AKR1B10
Pubmed	19387964	Correlation analysis of atrial natriuretic peptide concentration, echocardiographic left atrial and left ventricular dimensions, and renal function parameters in patients after permanent pacemaker implantation.	GeneRIF	0.0044	65.1	0.0114	0.116	1	1	NPPA
Pubmed	16393997	Aspartic acid homozygosity at codon 57 of HLA-DQ beta is associated with susceptibility to pulmonary tuberculosis in Cambodia.	GeneRIF	0.0044	65.1	0.0114	0.116	1	1	HLA-DQB1
Pubmed	3428272	A secretory protease inhibitor requires androgens for its expression in male sex accessory tissues but is expressed constitutively in pancreas.	Pubmed	0.0044	65.1	0.0114	0.116	1	1	SPINK1
Pubmed	9430637	Abnormal heart rate and body temperature in mice lacking thyroid hormone receptor alpha 1.	Pubmed	0.0044	65.1	0.0114	0.116	1	1	THRA
Pubmed	19052105	Deletion of SM-B, the high ATPase isoform of myosin, upregulates the PKC-mediated signal transduction pathway in murine urinary bladder smooth muscle.	Pubmed	0.0044	65.1	0.0114	0.116	1	1	MYH11
Pubmed	24590808	Induction of immunoregulatory CD271+ cells by metastatic tumor cells that express human endogenous retrovirus H.	Pubmed	0.0044	65.1	0.0114	0.116	1	1	NGFR
Pubmed	11003618	gamma-glutamyltranspeptidase-deficient knockout mice as a model to study the relationship between glutathione status, mitochondrial function, and cellular function.	Pubmed	0.0044	65.1	0.0114	0.116	1	1	GGT1
Pubmed	9748274	Identification of PKDL, a novel polycystic kidney disease 2-like gene whose murine homologue is deleted in mice with kidney and retinal defects.	Pubmed	0.0044	65.1	0.0114	0.116	1	1	PKD2L1
Pubmed	12374690	Overexpression of the peripheral benzodiazepine receptor is a relevant prognostic factor in stage III colorectal cancer.	GeneRIF	0.0044	65.1	0.0114	0.116	1	1	TSPO
Pubmed	15345707	Mutation in saposin D domain of sphingolipid activator protein gene causes urinary system defects and cerebellar Purkinje cell degeneration with accumulation of hydroxy fatty acid-containing ceramide in mouse.	GeneRIF	0.0044	65.1	0.0114	0.116	1	1	PSAP
Pubmed	9112393	Effect of carboxypeptidase E deficiency on progastrin processing and gastrin messenger ribonucleic acid expression in mice with the fat mutation.	Pubmed	0.0044	65.1	0.0114	0.116	1	1	CPE
Pubmed	18162604	A single amino acid difference between ether-a-go-go- related gene channel subtypes determines differential sensitivity to a small molecule activator.	GeneRIF	0.0044	65.1	0.0114	0.116	1	1	KCNH2
Pubmed	6589608	A human c-erbA oncogene homologue is closely proximal to the chromosome 17 breakpoint in acute promyelocytic leukemia.	Pubmed	0.0044	65.1	0.0114	0.116	1	1	THRA
Pubmed	11166164	Mild phenotype of nemaline myopathy with sleep hypoventilation due to a mutation in the skeletal muscle alpha-actin (ACTA1) gene.	Pubmed	0.0044	65.1	0.0114	0.116	1	1	ACTA1
Pubmed	10910890	alpha-thalassemia resulting from a negative chromosomal position effect.	Pubmed	0.0044	65.1	0.0114	0.116	1	1	HBA2

Category	ID	Name	Source	p-value	q-value Bonferroni	q-value FDR B&H	q-value FDR B&Y	Hit Count in Query List	Hit Count in Genome	Hit in Query List
Pubmed	21041690	Bone marrow-derived cells contribute to vascular inflammation but do not differentiate into smooth muscle cell lineages.	Pubmed	0.0044	65.1	0.0114	0.116	1	1	MYH11
Pubmed	7916904	Activation of autoreactive T-cell clones from NZB.H-2bm12 mice.	Pubmed	0.0044	65.1	0.0114	0.116	1	1	HLA-DQB1
Pubmed	16377621	The role of human HtrA1 in arthritic disease.	GeneRIF	0.0044	65.1	0.0114	0.116	1	1	HTRA1
Pubmed	18054208	Platelet 18 kDa Translocator Protein density is reduced in depressed patients with adult separation anxiety.	GeneRIF	0.0044	65.1	0.0114	0.116	1	1	TSPO
Pubmed	20833824	Increased proteolysis, myosin depletion, and atrophic AKT-FOXO signaling in human diaphragm disuse.	GeneRIF	0.0044	65.1	0.0114	0.116	1	1	FBXO32
Pubmed	10981873	Expression of MHC class II-associated invariant chain (Ii;CD74) in thymic epithelial neoplasms.	Pubmed	0.0044	65.1	0.0114	0.116	1	1	CD74
Pubmed	23608144	Sequence and structure-specific elements of HERG mRNA determine channel synthesis and trafficking efficiency.	Pubmed	0.0044	65.1	0.0114	0.116	1	1	KCNH2
Pubmed	24736806	Bone morphogenetic protein-4: a novel therapeutic target for pathological cardiac hypertrophy/heart failure.	Pubmed	0.0044	65.1	0.0114	0.116	1	1	BMP4
Pubmed	10469296	Molecular cloning and tissue expression of the murine analog to human stratum corneum chymotryptic enzyme.	Pubmed	0.0044	65.1	0.0114	0.116	1	1	KLK7
Pubmed	23672495	Interactions between voltage sensor and pore domains in a hERG K+ channel model from molecular simulations and the effects of a voltage sensor mutation.	Pubmed	0.0044	65.1	0.0114	0.116	1	1	KCNH2
Pubmed	19556882	Dipeptidyl peptidase 2 is an essential survival factor in the regulation of cell quiescence.	GeneRIF	0.0044	65.1	0.0114	0.116	1	1	DPP7
Pubmed	21075170	Recruitment of the oncoprotein v-ErbA to aggresomes.	GeneRIF	0.0044	65.1	0.0114	0.116	1	1	THRA
Pubmed	7759490	Human gamma-glutamyl transpeptidase mutants involving conserved aspartate residues and the unique cysteine residue of the light subunit.	Pubmed	0.0044	65.1	0.0114	0.116	1	1	GGT1
Pubmed	20215530	Glycoprotein nonmetastatic B is an independent prognostic indicator of recurrence and a novel therapeutic target in breast cancer.	GeneRIF	0.0044	65.1	0.0114	0.116	1	1	GPNMB
Pubmed	15558842	Mild phenotypes in a series of patients with Opitz GBBB syndrome with MID1 mutations.	Pubmed	0.0044	65.1	0.0114	0.116	1	1	MID1
Pubmed	1570328	Enzymatic defect in "X-linked" sideroblastic anemia: molecular evidence for erythroid delta-aminolevulinate synthase deficiency.	Pubmed	0.0044	65.1	0.0114	0.116	1	1	ALAS2
Pubmed	15890065	Cloning and characterization of the human glutathione synthetase 5'-flanking region.	GeneRIF	0.0044	65.1	0.0114	0.116	1	1	GSS
Pubmed	7592739	The primary structure of a basic leucine-rich repeat protein, PRELP, found in connective tissues.	Pubmed	0.0044	65.1	0.0114	0.116	1	1	PRELP
Pubmed	68958	Human beta-globin messenger RNA. III. Nucleotide sequences derived from complementary DNA.	Pubmed	0.0044	65.1	0.0114	0.116	1	1	HBB
Pubmed	22162062	Recovery from chronic spinal cord contusion after Nogo receptor intervention.	Pubmed	0.0044	65.1	0.0114	0.116	1	1	RTN4R
Pubmed	10051403	Genomic structure, chromosomal mapping, and expression pattern of human DCAMKL1 (KIAA0369), a homologue of DCX (XLIS).	Pubmed	0.0044	65.1	0.0114	0.116	1	1	DCLK1
Pubmed	25142202	Bone morphogenetic protein 4 inhibits liposaccharide-induced inflammation in the airway.	Pubmed	0.0044	65.1	0.0114	0.116	1	1	BMP4
Pubmed	14660481	Leukocytic cell sources of airway tissue kallikrein.	GeneRIF	0.0044	65.1	0.0114	0.116	1	1	KLK1
Pubmed	21762399	Multiplexed, ligation-dependent probe amplification for rapid and inexpensive HLA-DQB1 allelotyping.	GeneRIF	0.0044	65.1	0.0114	0.116	1	1	HLA-DQB1
Pubmed	11836160	Clinical phenotypes and molecular characterization of Hb H-Paksé disease.	GeneRIF	0.0044	65.1	0.0114	0.116	1	1	HBA2
Pubmed	11923404	Complete deletion of the neurotrophin receptor p75NTR leads to long-lasting increases in the number of basal forebrain cholinergic neurons.	Pubmed	0.0044	65.1	0.0114	0.116	1	1	NGFR
Pubmed	21450637	Higher aldosterone and lower N-terminal proatrial natriuretic peptide as biomarkers of salt sensitivity in the community.	GeneRIF	0.0044	65.1	0.0114	0.116	1	1	NPPA
Pubmed	24024878	Influence of endothelial cell protein C receptor on breast cancer development.	Pubmed	0.0044	65.1	0.0114	0.116	1	1	PROCR
Pubmed	8040308	Gene expression and secretion of atrial natriuretic peptide by murine macrophages.	Pubmed	0.0044	65.1	0.0114	0.116	1	1	NPPA
Pubmed	16156863	The frequency of the sickle allele in Jamaica has not declined over the last 22 years.	GeneRIF	0.0044	65.1	0.0114	0.116	1	1	HBB
Pubmed	18993085	Low ligand requirement for deletion and lack of synapses in positive selection enforce the gauntlet of thymic T cell maturation.	Pubmed	0.0044	65.1	0.0114	0.116	1	1	CD74
Pubmed	20680650	Hyponatremia is predictable in patients with aneurysmal subarachnoid hemorrhage--clinical significance of serum atrial natriuretic peptide.	GeneRIF	0.0044	65.1	0.0114	0.116	1	1	NPPA
Pubmed	22334565	Fatty liver index, gamma-glutamyltransferase, and early carotid plaques.	GeneRIF	0.0044	65.1	0.0114	0.116	1	1	GGT1
Pubmed	11517927	Calpain function in the modulation of signal transduction molecules.	Pubmed	0.0044	65.1	0.0114	0.116	1	1	CAPN5
Pubmed	16928223	Clinical significance of human kallikrein 10 gene expression in colorectal cancer and gastric cancer.	GeneRIF	0.0044	65.1	0.0114	0.116	1	1	KLK10
Pubmed	19464995	Kinetic studies of AKR1B10, human aldose reductase-like protein: endogenous substrates and inhibition by steroids.	GeneRIF	0.0044	65.1	0.0114	0.116	1	1	AKR1B10
Pubmed	15701626	Subunit exchange of polydisperse proteins: mass spectrometry reveals consequences of alphaA-crystallin truncation.	GeneRIF	0.0044	65.1	0.0114	0.116	1	1	CRYAA
Pubmed	19230568	Genetic variants of beta-globin gene in Thai malaria patients.	GeneRIF	0.0044	65.1	0.0114	0.116	1	1	HBB
Pubmed	2845979	Saposin D: a sphingomyelinase activator.	Pubmed	0.0044	65.1	0.0114	0.116	1	1	PSAP
Pubmed	2384314	Hb Matera [beta 55(D6)Met----Lys]: a new unstable hemoglobin variant in an Italian family.	Pubmed	0.0044	65.1	0.0114	0.116	1	1	HBB
Pubmed	17053165	Intervention of MAdCAM-1 or fractalkine alleviates graft-versus-host reaction associated intestinal injury while preserving graft-versus-tumor effects.	Pubmed	0.0044	65.1	0.0114	0.116	1	1	CX3CL1
Pubmed	24462143	Not all pathogenic mutations are pathogenic: KCNH2 mutations in two sisters with tetralogy of Fallot.	Pubmed	0.0044	65.1	0.0114	0.116	1	1	KCNH2

Category	ID	Name	Source	p-value	q-value Bonferroni	q-value FDR B&H	q-value FDR B&Y	Hit Count in Query List	Hit Count in Genome	Hit in Query List
Pubmed	20231425	Differing effects of HbS and HbC traits on uncomplicated falciparum malaria, anemia, and child growth.	GeneRIF	0.0044	65.1	0.0114	0.116	1	1	HBB
Pubmed	15837295	A structural screening approach to ketoamide-based inhibitors of cathepsin K.	GeneRIF	0.0044	65.1	0.0114	0.116	1	1	CTSK
Pubmed	15858026	Human immunodeficiency virus type 1 stimulates the expression and production of secretory leukocyte protease inhibitor (SLPI) in oral epithelial cells: a role for SLPI in innate mucosal immunity.	Pubmed	0.0044	65.1	0.0114	0.116	1	1	SLPI
Pubmed	9367857	Identification of a novel, tissue-specific calpain htra-3; a human homologue of the Caenorhabditis elegans sex determination gene.	Pubmed	0.0044	65.1	0.0114	0.116	1	1	CAPN5
Pubmed	21124911	The preferred substrates for transglutaminase 2 in a complex wheat gluten digest are Peptide fragments harboring celiac disease T-cell epitopes.	GeneRIF	0.0044	65.1	0.0114	0.116	1	1	TGM2
Pubmed	21938001	Support for association between the Ser205Leu polymorphism of p75(NTR) and major depressive disorder.	GeneRIF	0.0044	65.1	0.0114	0.116	1	1	NGFR
Pubmed	9669045	A common polygenic basis for quinine and PROP avoidance in mice.	Pubmed	0.0044	65.1	0.0114	0.116	1	1	CPE
Pubmed	24638068	The invariant chain p35 isoform promotes formation of nonameric complexes with MHC II molecules.	Pubmed	0.0044	65.1	0.0114	0.116	1	1	CD74
Pubmed	12581187	Distribution and frequency of beta-thalassemia mutations in northwestern and central Greece.	GeneRIF	0.0044	65.1	0.0114	0.116	1	1	HBB
Pubmed	15757863	Aging affects the retinoic acid and the triiodothyronine nuclear receptor mRNA expression in human peripheral blood mononuclear cells.	GeneRIF	0.0044	65.1	0.0114	0.116	1	1	THRA
Pubmed	11448102	Cloning, sequencing and expression of a cDNA encoding the mouse L35a ribosomal protein during differentiation of murine erythroleukemia (MEL) cells.	Pubmed	0.0044	65.1	0.0114	0.116	1	1	RPL35A
Pubmed	21872912	Functional KCa3.1 K+ channels are required for human fibrocyte migration.	GeneRIF	0.0044	65.1	0.0114	0.116	1	1	KCNN4
Pubmed	24489848	MASP-1 induces a unique cytokine pattern in endothelial cells: a novel link between complemen system and neutrophil granulocytes.	Pubmed	0.0044	65.1	0.0114	0.116	1	1	MASP1
Pubmed	18302529	Sex differences in adenosine deaminase activity of stroke patients.	GeneRIF	0.0044	65.1	0.0114	0.116	1	1	ADA
Pubmed	21571276	A single nucleotide polymorphism in exon 3 of the kallikrein 1 gene is associated with large but not small abdominal aortic aneurysm.	GeneRIF	0.0044	65.1	0.0114	0.116	1	1	KLK1
Pubmed	16116976	Role of the pentanucleotide (tttta)n polymorphisms of Cyp11alpha gene in the pathogenesis of hyperandrogenism in Chinese women with polycystic ovary syndrome.	GeneRIF	0.0044	65.1	0.0114	0.116	1	1	CYP11A1
Pubmed	17724207	Degradation of C-terminal truncated alpha A-crystallins by the ubiquitin-proteasome pathway.	Pubmed	0.0044	65.1	0.0114	0.116	1	1	CRYAA
Pubmed	67117	Defects in the cartilaginous growth plates of brachymorphic mice.	Pubmed	0.0044	65.1	0.0114	0.116	1	1	PAPSS2
Pubmed	17535305	Chronic exposure in vivo to thyrotropin receptor stimulating monoclonal antibodies sustains high thyroxine levels and thyroid hyperplasia in thyroid autoimmunity-prone HLA-DRB1*0301 transgenic mice.	Pubmed	0.0044	65.1	0.0114	0.116	1	1	HLA-DQB1
Pubmed	11600541	The first signs of beta-cell autoimmunity appear in infancy in genetically susceptible children from the general population: the Finnish Type 1 Diabetes Prediction and Prevention Study.	GeneRIF	0.0044	65.1	0.0114	0.116	1	1	HLA-DQB1
Pubmed	19915139	Nogo receptor 1 regulates formation of lasting memories.	GeneRIF	0.0044	65.1	0.0114	0.116	1	1	RTN4R
Pubmed	9147642	Association of the steroid synthesis gene CYP11a with polycystic ovary syndrome and hyperandrogenism.	Pubmed	0.0044	65.1	0.0114	0.116	1	1	CYP11A1
Pubmed	24429499	Differential expression of hERG1A and hERG1B genes in pediatric acute lymphoblastic leukemia identifies different prognostic subgroups.	Pubmed	0.0044	65.1	0.0114	0.116	1	1	KCNH2
Pubmed	15473257	Expression of the peripheral benzodiazepine receptor triggers thymocyte differentiation.	GeneRIF	0.0044	65.1	0.0114	0.116	1	1	TSPO
Pubmed	22306195	Genetic and functional analysis of the gene encoding neurogranin in schizophrenia.	GeneRIF	0.0044	65.1	0.0114	0.116	1	1	NRGN
Pubmed	21546760	Regulation of bmp4 expression in odontogenic mesenchyme: from simple to complex.	GeneRIF	0.0044	65.1	0.0114	0.116	1	1	BMP4
Pubmed	22461181	Functional genetic variation at the NRGN gene and schizophrenia: evidence from a gene-based case-control study and gene expression analysis.	GeneRIF	0.0044	65.1	0.0114	0.116	1	1	NRGN
Pubmed	21164287	Immunohistochemical staining for smoothelin in the duplicated versus the true muscularis mucosae of Barrett esophagus.	GeneRIF	0.0044	65.1	0.0114	0.116	1	1	SMTN
Pubmed	20709911	Unliganded thyroid hormone receptor alpha1 impairs adult hippocampal neurogenesis.	GeneRIF	0.0044	65.1	0.0114	0.116	1	1	THRA
Pubmed	24326630	Hepatic glucose uptake is increased in association with elevated serum ¿-glutamyl transpeptidase and triglyceride.	Pubmed	0.0044	65.1	0.0114	0.116	1	1	GGT1
Pubmed	18551196	Fever-induced QTc prolongation and ventricular arrhythmias in individuals with type 2 congenital long QT syndrome.	GeneRIF	0.0044	65.1	0.0114	0.116	1	1	KCNH2
Pubmed	3877508	Molecular cloning and nucleotide sequence of human pancreatic secretory trypsin inhibitor (PSTI) cDNA.	Pubmed	0.0044	65.1	0.0114	0.116	1	1	SPINK1
Pubmed	23792966	The x-ray crystal structure of mannose-binding lectin-associated serine proteinase-3 reveals the structural basis for enzyme inactivity associated with the Carnevale, Mingarelli, Malpuech, and Michels (3MC) syndrome.	Pubmed	0.0044	65.1	0.0114	0.116	1	1	MASP1
Pubmed	12366785	HLA-DRB1 and MHC class 1 chain-related A haplotypes in Basque families with celiac disease.	GeneRIF	0.0044	65.1	0.0114	0.116	1	1	HLA-DQB1
Pubmed	24931372	Insight into the molecular interaction between the cyclic nucleotide-binding homology domain and the eag domain of the hERG channel.	Pubmed	0.0044	65.1	0.0114	0.116	1	1	KCNH2

Category	ID	Name	Source	p-value	q-value Bonferroni	q-value FDR B&H	q-value FDR B&Y	Hit Count in Query List	Hit Count in Genome	Hit in Query List
Pubmed	21463954	High plasma fractalkine (CX3CL1) levels are associated with severe liver disease in HIV/HCV co-infected patients with HCV genotype 1.	GeneRIF	0.0044	65.1	0.0114	0.116	1	1	CX3CL1
Pubmed	8986645	Cathepsin K: isolation and characterization of the murine cDNA and genomic sequence, the homologue of the human pycnodysostosis gene.	Pubmed	0.0044	65.1	0.0114	0.116	1	1	CTSK
Pubmed	18483283	Association of gamma-glutamyltransferase and risk of cancer incidence in men: a prospective study.	GeneRIF	0.0044	65.1	0.0114	0.116	1	1	GGT1
Pubmed	16081633	Relationship between thyroid peroxidase T cell epitope restriction and antibody recognition of the autoantibody immunodominant region in human leukocyte antigen DR3 transgenic mice.	Pubmed	0.0044	65.1	0.0114	0.116	1	1	HLA-DQB1
Pubmed	17868276	A novel compound heterozygous dysferlin mutation in Miyoshi myopathy siblings responding to dantrolene.	GeneRIF	0.0044	65.1	0.0114	0.116	1	1	DYSF
Pubmed	17612748	Effect of heterozygous beta-thalassaemia trait on coronary atherosclerosis via coronary artery disease risk factors: a preliminary study.	GeneRIF	0.0044	65.1	0.0114	0.116	1	1	HBB
Pubmed	24913888	Mechanism of a novel missense mutation, p.V174M, of the human connexin31 (GJB3) in causing nonsyndromic hearing loss.	Pubmed	0.0044	65.1	0.0114	0.116	1	1	GJB3
Pubmed	20375326	Pathway-focused arrays reveal increased matrix metalloproteinase-7 (matrilysin) transcription in trachomatous trichiasis.	GeneRIF	0.0044	65.1	0.0114	0.116	1	1	MMP7
Pubmed	24640520	Coronary artery disease. A study of three polymorphic sites of adenosine deaminase gene.	Pubmed	0.0044	65.1	0.0114	0.116	1	1	ADA
Pubmed	8537364	A unique thyroid hormone response element in the human immunodeficiency virus type 1 long terminal repeat that overlaps the Sp1 binding sites.	Pubmed	0.0044	65.1	0.0114	0.116	1	1	THRA
Pubmed	20395516	Comprehensive and efficient HBB mutation analysis for detection of beta-hemoglobinopathies in a pan-ethnic population.	GeneRIF	0.0044	65.1	0.0114	0.116	1	1	HBB
Pubmed	24047895	Novel insights into eukaryotic ¿-glutamyltranspeptidase 1 from the crystal structure of the glutamate-bound human enzyme.	Pubmed	0.0044	65.1	0.0114	0.116	1	1	GGT1
Pubmed	17339605	IFI16 in human prostate cancer.	GeneRIF	0.0044	65.1	0.0114	0.116	1	1	IFI16
Pubmed	22736764	Modular dispensability of dysferlin C2 domains reveals rational design for mini-dysferlin molecules.	GeneRIF	0.0044	65.1	0.0114	0.116	1	1	DYSF
Pubmed	15885239	Spectrum of beta-thalassemia mutations in North Indian states: a beta-thalassemia trait with two mutations in cis.	GeneRIF	0.0044	65.1	0.0114	0.116	1	1	HBB
Pubmed	8827453	Effects of substitutions of the conserved histidine residues in human gamma-glutamyl transpeptidase.	Pubmed	0.0044	65.1	0.0114	0.116	1	1	GGT1
Pubmed	21741108	Atrial Natriuretic Peptide (ANP) in early pregnancy is associated with development of preeclampsia in type 1 diabetes.	GeneRIF	0.0044	65.1	0.0114	0.116	1	1	NPPA
Pubmed	2891746	Immunocytochemical localization of gamma-glutamyltranspeptidase during fetal development of mouse kidney.	Pubmed	0.0044	65.1	0.0114	0.116	1	1	GGT1
Pubmed	22067542	Hypertrophy and dietary tyrosine ameliorate the phenotypes of a mouse model of severe nemaline myopathy.	Pubmed	0.0044	65.1	0.0114	0.116	1	1	ACTA1
Pubmed	19013523	Modulation of tissue transglutaminase in tubular epithelial cells alters extracellular matrix levels: a potential mechanism of tissue scarring.	GeneRIF	0.0044	65.1	0.0114	0.116	1	1	TGM2
Pubmed	17898270	Four mutations (three novel, one founder) in TACSTD2 among Iranian GDLD patients.	GeneRIF	0.0044	65.1	0.0114	0.116	1	1	TACSTD2
Pubmed	20675377	The sodium channel {beta}3-subunit induces multiphasic gating in NaV1.3 and affects fast inactivation via distinct intracellular regions.	GeneRIF	0.0044	65.1	0.0114	0.116	1	1	SCN3B
Pubmed	24532136	In vitro effect of FGIN-1-27, a ligand to 18 kDa mitochondrial translocator protein, in human osteoblast-like cells.	Pubmed	0.0044	65.1	0.0114	0.116	1	1	TSPO
Pubmed	18761669	Neurotrophic signalling pathway triggered by prosaposin in PC12 cells occurs through lipid rafts.	GeneRIF	0.0044	65.1	0.0114	0.116	1	1	PSAP
Pubmed	23633213	Clinical phenotype of a new type of thyroid hormone resistance caused by a mutation of the TRa1 receptor: consequences of LT4 treatment.	Pubmed	0.0044	65.1	0.0114	0.116	1	1	THRA
Pubmed	26214904	[IDENTIFICATION OF MUTATION IVS1-5(G > C) OF THE ß-HEMOGLOBIN GENE (Hbß) BY RDBH-METHOD IN PATIENTS WITH ß-THALASSEMIA IN AZERBAIJAN].	Pubmed	0.0044	65.1	0.0114	0.116	1	1	HBB
Pubmed	20949628	Evaluation of germline BMP4 mutation as a cause of colorectal cancer.	GeneRIF	0.0044	65.1	0.0114	0.116	1	1	BMP4
Pubmed	9751800	Isolation and characterization of the human prosaposin promoter.	Pubmed	0.0044	65.1	0.0114	0.116	1	1	PSAP
Pubmed	20445115	An intergenic region between the tagSNP rs3793917 and rs11200638 in the HTRA1 gene indicates association with age-related macular degeneration.	GeneRIF	0.0044	65.1	0.0114	0.116	1	1	HTRA1
Pubmed	20944116	Human kallikrein 7 induces epithelial-mesenchymal transition-like changes in prostate carcinoma cells: a role in prostate cancer invasion and progression.	GeneRIF	0.0044	65.1	0.0114	0.116	1	1	KLK7
Pubmed	9452083	Mutation in the 5' noncoding region of the SRY gene in an XY sex-reversed patient.	Pubmed	0.0044	65.1	0.0114	0.116	1	1	SPINK1
Pubmed	15837121	Differential effects of endogenous brain-derived neurotrophic factor on the survival of axotomized sensory neurons in dorsal root ganglia: a possible role for the p75 neurotrophin receptor.	Pubmed	0.0044	65.1	0.0114	0.116	1	1	NGFR
Pubmed	22422195	High expression of CX3CL1 by tumor cells correlates with a good prognosis and increased tumor-infiltrating CD8+ T cells, natural killer cells, and dendritic cells in breast carcinoma.	GeneRIF	0.0044	65.1	0.0114	0.116	1	1	CX3CL1
Pubmed	16647913	Overexpression of kallikrein 10 (hK10) in uterine serous papillary carcinomas.	GeneRIF	0.0044	65.1	0.0114	0.116	1	1	KLK10

Category	ID	Name	Source	p-value	q-value Bonferroni	q-value FDR B&H	q-value FDR B&Y	Hit Count in Query List	Hit Count in Genome	Hit in Query List
Pubmed	9752000	An asymmetric deuterium labeling strategy to identify interprotomer and intraprotomer NOEs in oligomeric proteins.	Pubmed	0.0044	65.1	0.0114	0.116	1	1	CD74
Pubmed	19198000	A site-directed mutagenesis study of drug-binding selectivity in genetic variants of human alpha(1)-acid glycoprotein.	GeneRIF	0.0044	65.1	0.0114	0.116	1	1	ORM1
Pubmed	11422294	A novel putative transmembrane protein, IZP6, is expressed in neural cells during embryogenesis.	Pubmed	0.0044	65.1	0.0114	0.116	1	1	DERL3
Pubmed	3463531	Mapping through somatic cell hybrids and cDNA probes of protein C to chromosome 2, factor X to chromosome 13, and alpha 1-acid glycoprotein to chromosome 9.	Pubmed	0.0044	65.1	0.0114	0.116	1	1	ORM1
Pubmed	14572455	Regulation, cellular localization, and function of the p75 neurotrophin receptor (p75NTR) during the regeneration of facial motoneurons.	GeneRIF	0.0044	65.1	0.0114	0.116	1	1	NGFR
Pubmed	23460280	Endothelial lipase modulates pressure overload-induced heart failure through alternative pathway for fatty acid uptake.	GeneRIF	0.0044	65.1	0.0114	0.116	1	1	LIPG
Pubmed	20065949	Promoter hypermethylation of FBXO32, a novel TGF-beta/SMAD4 target gene and tumor suppressor, is associated with poor prognosis in human ovarian cancer.	GeneRIF	0.0044	65.1	0.0114	0.116	1	1	FBXO32
Pubmed	11015600	ANP in regulation of arterial pressure and fluid-electrolyte balance: lessons from genetic mouse models.	Pubmed	0.0044	65.1	0.0114	0.116	1	1	NPPA
Pubmed	21457553	A Kallikrein 15 (KLK15) single nucleotide polymorphism located close to a novel exon shows evidence of association with poor ovarian cancer survival.	GeneRIF	0.0044	65.1	0.0114	0.116	1	1	KLK15
Pubmed	7553002	Major histocompatibility class II peptide occupancy, antigen presentation, and CD4+ T cell function in mice lacking the p41 isoform of invariant chain.	Pubmed	0.0044	65.1	0.0114	0.116	1	1	CD74
Pubmed	20731657	Presence of tissue transglutaminase in granular endoplasmic reticulum is characteristic of melanized neurons in Parkinson's disease brain.	GeneRIF	0.0044	65.1	0.0114	0.116	1	1	TGM2
Pubmed	10518537	Regulation of nuclear translocation of forkhead transcription factor AFX by protein kinase B.	Pubmed	0.0044	65.1	0.0114	0.116	1	1	FOXO4
Pubmed	24572254	Dysferlin mediates the cytoprotective effects of TRAF2 following myocardial ischemia reperfusion injury.	Pubmed	0.0044	65.1	0.0114	0.116	1	1	DYSF
Pubmed	10332047	Novel genetic association between the corneodesmosin (MHC S) gene and susceptibility to psoriasis.	Pubmed	0.0044	65.1	0.0114	0.116	1	1	CDSN
Pubmed	24182909	Hemoglobin nonrecovery following acute myocardial infarction is a biomarker of poor outcome: a retrospective database study.	Pubmed	0.0044	65.1	0.0114	0.116	1	1	HBB
Pubmed	16263765	Downregulation of the HERG (KCNH2) K(+) channel by ceramide: evidence for ubiquitin-mediated lysosomal degradation.	GeneRIF	0.0044	65.1	0.0114	0.116	1	1	KCNH2
Pubmed	19886543	Cathepsin-k as a diagnostic marker in the identification of micro-granulomas in Crohn's disease.	GeneRIF	0.0044	65.1	0.0114	0.116	1	1	CTSK
Pubmed	17322565	Glycosylation of endothelial lipase at asparagine-116 reduces activity and the hydrolysis of native lipoproteins in vitro and in vivo.	GeneRIF	0.0044	65.1	0.0114	0.116	1	1	LIPG
Pubmed	16387637	Doublecortin-like kinase controls neurogenesis by regulating mitotic spindles and M phase progression.	GeneRIF	0.0044	65.1	0.0114	0.116	1	1	DCLK1
Pubmed	24425843	MMP7 expression in colorectal tumours of different stages.	Pubmed	0.0044	65.1	0.0114	0.116	1	1	MMP7
Pubmed	23580433	Downregulation of serine protease HTRA1 is associated with poor survival in breast cancer.	Pubmed	0.0044	65.1	0.0114	0.116	1	1	HTRA1
Pubmed	894324	Peripheral nerve changes induced by local application of bee venom.	Pubmed	0.0044	65.1	0.0114	0.116	1	1	CRYAA
Pubmed	16524878	The S4-S5 linker directly couples voltage sensor movement to the activation gate in the human ether-a'-go-go-related gene (hERG) K+ channel.	GeneRIF	0.0044	65.1	0.0114	0.116	1	1	KCNH2
Pubmed	18548257	The FVIIa-tissue factor complex induces the expression of MMP7 in LOVO cells in vitro.	GeneRIF	0.0044	65.1	0.0114	0.116	1	1	MMP7
Pubmed	21880796	A novel entity of clinically isolated adrenal insufficiency caused by a partially inactivating mutation of the gene encoding for P450 side chain cleavage enzyme (CYP11A1).	GeneRIF	0.0044	65.1	0.0114	0.116	1	1	CYP11A1
Pubmed	15147217	Conformational substates of the oxyheme centers in alpha and beta subunits of hemoglobin as disclosed by EPR and ENDOR studies of cryoreduced protein.	GeneRIF	0.0044	65.1	0.0114	0.116	1	1	HBB
Pubmed	24356998	HtrA1 downregulation induces cisplatin resistance in lung adenocarcinoma by promoting cancer stem cell-like properties.	Pubmed	0.0044	65.1	0.0114	0.116	1	1	HTRA1
Pubmed	17665502	A novel mutation of the beta-globin gene promoter (-102 C>A) and pitfalls in family screening.	GeneRIF	0.0044	65.1	0.0114	0.116	1	1	HBB
Pubmed	21469121	Th17 differentiation is the default program for DPP2-deficient T-cell differentiation.	GeneRIF	0.0044	65.1	0.0114	0.116	1	1	DPP7
Pubmed	7911253	Gh: a GTP-binding protein with transglutaminase activity and receptor signaling function.	Pubmed	0.0044	65.1	0.0114	0.116	1	1	TGM2
Pubmed	22782215	Tissue transglutaminase activity protects from cutaneous melanoma metastatic dissemination: an in vivo study.	GeneRIF	0.0044	65.1	0.0114	0.116	1	1	TGM2
Pubmed	24610422	Polymorphisms of pentanucleotide repeats (tttta)n in the promoter of CYP11A1 and their relationships to polycystic ovary syndrome (PCOS risk: a meta-analysis.	Pubmed	0.0044	65.1	0.0114	0.116	1	1	CYP11A1
Pubmed	11502818	Heterozygous mutation in the cholesterol side chain cleavage enzyme (p450scc) gene in a patient with 46,XY sex reversal and adrenal insufficiency.	Pubmed	0.0044	65.1	0.0114	0.116	1	1	CYP11A1
Pubmed	6576808	Association of human gamma chain with class II transplantation antigens during intracellular transport.	Pubmed	0.0044	65.1	0.0114	0.116	1	1	CD74
Pubmed	3384700	First observation of Hb Montgomery [alpha 48(CD6)Leu----Arg] in Tunisia.	Pubmed	0.0044	65.1	0.0114	0.116	1	1	HBA2

Category	ID	Name	Source	p-value	q-value Bonferroni	q-value FDR B&H	q-value FDR B&Y	Hit Count in Query List	Hit Count in Genome	Hit in Query List
Pubmed	21523319	Molecular characteristics of three hemoglobin variants observed in a Chinese population: Hb Ube 1 [ß98 (FG5) Val¿Met], Hb Ube-2 [a68 (E17) Asn¿Asp] and Hb Ube-4 [a116 (GH4) Glu¿Ala].	GeneRIF	0.0044	65.1	0.0114	0.116	1	1	HBB
Pubmed	24922571	The Nogo receptor NgR1 mediates infection by mammalian reovirus.	Pubmed	0.0044	65.1	0.0114	0.116	1	1	RTN4R
Pubmed	25015565	Histone demethylase RBP2 induced by Helicobactor Pylori CagA participates in the malignant transformation of gastric epithelial cells.	Pubmed	0.0044	65.1	0.0114	0.116	1	1	KDM5A
Pubmed	21077760	ß-globin gene haplotypes linked with the Hb D-Punjab [ß121(GH4)Glu¿Gln, GAA>CAA] mutation in eastern India.	GeneRIF	0.0044	65.1	0.0114	0.116	1	1	HBB
Pubmed	6095119	Nucleotide sequence of the gene encoding human atrial natriuretic factor precursor.	Pubmed	0.0044	65.1	0.0114	0.116	1	1	NPPA
Pubmed	9125171	Molecular cloning and expression of murine smooth muscle myosin heavy chains.	Pubmed	0.0044	65.1	0.0114	0.116	1	1	MYH11
Pubmed	12087468	Higher expression of human kallikrein 10 in breast cancer tissue predicts tamoxifen resistance.	GeneRIF	0.0044	65.1	0.0114	0.116	1	1	KLK10
Pubmed	8978768	Mapping of the metalloproteinase gene matrilysin (MMP7) to human chromosome 11q21-->q22.	Pubmed	0.0044	65.1	0.0114	0.116	1	1	MMP7
Pubmed	21052915	Chromosomal sharing in atypical cases of gelatinous drop-like corneal dystrophy.	GeneRIF	0.0044	65.1	0.0114	0.116	1	1	TACSTD2
Pubmed	26033354	Characterization of spontaneous air space enlargement in mice lacking microfibrillar-associated protein 4.	Pubmed	0.0044	65.1	0.0114	0.116	1	1	MFAP4
Pubmed	18328005	A novel Zip2 Gln/Arg/Leu codon 2 polymorphism is associated with carotid artery disease in aging.	GeneRIF	0.0044	65.1	0.0114	0.116	1	1	SLC39A2
Pubmed	10484477	Salt-sensitive hypertension in ANP knockout mice is prevented by AT1 receptor antagonist losartan.	Pubmed	0.0044	65.1	0.0114	0.116	1	1	NPPA
Pubmed	24780890	Serum gamma-glutamyl transferase: a novel biomarker for coronary artery disease.	Pubmed	0.0044	65.1	0.0114	0.116	1	1	GGT1
Pubmed	1634367	Hb Kodaira [beta 146(HC3)His----Gln]: a new beta chain variant with an amino acid substitution at the C-terminus.	Pubmed	0.0044	65.1	0.0114	0.116	1	1	HBB
Pubmed	22207728	Role for the guanine nucleotide exchange factor phosphatidylinositol-3,4,5-trisphosphate-dependent rac exchanger 1 in platelet secretion and aggregation.	GeneRIF	0.0044	65.1	0.0114	0.116	1	1	PREX1
Pubmed	8541228	Characterization of the promoter of the gene for a mouse vas deferens protein related to the aldo-keto reductase superfamily: effect of steroid hormones and phorbol esters.	Pubmed	0.0044	65.1	0.0114	0.116	1	1	AKR1B10
Pubmed	8486654	Macrosialin, a mouse macrophage-restricted glycoprotein, is a member of the lamp/lgp family.	Pubmed	0.0044	65.1	0.0114	0.116	1	1	CD68
Pubmed	17428496	Solution structure of the MID1 B-box2 CHC(D/C)C(2)H(2) zinc-binding domain: insights into an evolutionarily conserved RING fold.	GeneRIF	0.0044	65.1	0.0114	0.116	1	1	MID1
Pubmed	17312154	HLA transgenic mice provide evidence for a direct and dominant role of HLA class II variation in modulating the severity of streptococcal sepsis.	Pubmed	0.0044	65.1	0.0114	0.116	1	1	HLA-DQB1
Pubmed	10441162	Effect of the bm12 class II mutation on proliferative and cytokine responses of encephalitogenic T cells in myelin oligodendrocyte glycoprotein (MOG)-induced experimental autoimmune encephalomyelitis.	Pubmed	0.0044	65.1	0.0114	0.116	1	1	HLA-DQB1
Pubmed	24239457	Alternate splicing of dysferlin C2A confers Ca²¿-dependent and Ca²¿-independent binding for membrane repair.	Pubmed	0.0044	65.1	0.0114	0.116	1	1	DYSF
Pubmed	10475605	Gene structure of the P100 serine-protease component of the human Ra-reactive factor.	Pubmed	0.0044	65.1	0.0114	0.116	1	1	MASP1
Pubmed	15528049	Plasma membrane gamma-glutamyltransferase activity facilitates the uptake of vitamin C in melanoma cells.	GeneRIF	0.0044	65.1	0.0114	0.116	1	1	GGT1
Pubmed	21947419	SLC39A2 and FSIP1 polymorphisms as potential modifiers of arsenic-related bladder cancer.	Pubmed	0.0044	65.1	0.0114	0.116	1	1	SLC39A2
Pubmed	9143500	Structure, organization, and chromosomal mapping of the human neurogranin gene (NRGN).	Pubmed	0.0044	65.1	0.0114	0.116	1	1	NRGN
Pubmed	11759111	Secretory leukocyte protease inhibitor in punch biopsies from human colonic mucosa.	GeneRIF	0.0044	65.1	0.0114	0.116	1	1	SLPI
Pubmed	12609997	Molecular localization of the inhibitory arachidonic acid binding site to the pore of hIK1.	GeneRIF	0.0044	65.1	0.0114	0.116	1	1	KCNN4
Pubmed	10330043	Genetic disruption of atrial natriuretic peptide causes pulmonary hypertension in normoxic and hypoxic mice.	Pubmed	0.0044	65.1	0.0114	0.116	1	1	NPPA
Pubmed	24569994	A novel mechanism by which tissue transglutaminase activates signaling events that promote cell survival.	Pubmed	0.0044	65.1	0.0114	0.116	1	1	TGM2
Pubmed	12107223	Type-2 diabetes family history delays the onset of type-1 diabetes.	GeneRIF	0.0044	65.1	0.0114	0.116	1	1	HLA-DQB1
Pubmed	16043129	Studies of humoral immunity to preprohypocretin in human leukocyte antigen DQB1*0602-positive narcoleptic subjects with cataplexy.	GeneRIF	0.0044	65.1	0.0114	0.116	1	1	HLA-DQB1
Pubmed	23620432	Quantified KLK15 gene expression levels discriminate prostate cancer from benign tumors and constitute a novel independent predictor of disease progression.	GeneRIF	0.0044	65.1	0.0114	0.116	1	1	KLK15
Pubmed	23911203	Bmp4 expressed in preadipocytes is required for the onset of adipocyte differentiation.	Pubmed	0.0044	65.1	0.0114	0.116	1	1	BMP4
Pubmed	15304481	Cardiac IKr channels minimally comprise hERG 1a and 1b subunits.	GeneRIF	0.0044	65.1	0.0114	0.116	1	1	KCNH2
Pubmed	3770479	Molecular cloning and nucleotide sequence of human alpha 1 acid glycoprotein cDNA.	Pubmed	0.0044	65.1	0.0114	0.116	1	1	ORM1
Pubmed	24573298	Mir-592 regulates the induction and cell death-promoting activity of p75NTR in neuronal ischemic injury.	Pubmed	0.0044	65.1	0.0114	0.116	1	1	NGFR
Pubmed	19034039	Papaverine, a vasodilator, blocks the pore of the HERG channel at submicromolar concentration.	GeneRIF	0.0044	65.1	0.0114	0.116	1	1	KCNH2

Category	ID	Name	Source	p-value	q-value Bonferroni	q-value FDR B&H	q-value FDR B&Y	Hit Count in Query List	Hit Count in Genome	Hit in Query List
Pubmed	17957445	Molecular scanning of the human carboxypeptidase E gene for mutations in Chinese subjects with coronary atherosclerosis.	GeneRIF	0.0044	65.1	0.0114	0.116	1	1	CPE
Pubmed	20132547	Prosaposin down-modulation decreases metastatic prostate cancer cell adhesion, migration, and invasion.	GeneRIF	0.0044	65.1	0.0114	0.116	1	1	PSAP
Pubmed	12242267	Reduced apoptosis and increased lesion development in the flow-restricted carotid artery of p75(NTR)-null mutant mice.	Pubmed	0.0044	65.1	0.0114	0.116	1	1	NGFR
Pubmed	12217813	Beta- and alpha-globin genotypes in Albanian patients affected by beta-globin gene disorders.	GeneRIF	0.0044	65.1	0.0114	0.116	1	1	HBB
Pubmed	23730209	Extracellular tissue transglutaminase activates noncanonical NF-¿B signaling and promotes metastasis in ovarian cancer.	Pubmed	0.0044	65.1	0.0114	0.116	1	1	TGM2
Pubmed	9714015	Distinct, autosomal recessive form of spondyloepimetaphyseal dysplasia segregating in an inbred Pakistani kindred.	Pubmed	0.0044	65.1	0.0114	0.116	1	1	PAPSS2
Pubmed	18788756	Oxidation of PAH trans-dihydrodiols by human aldo-keto reductase AKR1B10.	GeneRIF	0.0044	65.1	0.0114	0.116	1	1	AKR1B10
Pubmed	20530040	Midregional pro-atrial natriuretic peptide and procalcitonin improve survival prediction in VAP.	GeneRIF	0.0044	65.1	0.0114	0.116	1	1	NPPA
Pubmed	15081124	Conservation and loss of the ERV3 open reading frame in primates.	GeneRIF	0.0044	65.1	0.0114	0.116	1	1	ERV3-1
Pubmed	12601245	Detection of a processed pseudogene of the human MBL-associated serine protease, MASP1.	GeneRIF	0.0044	65.1	0.0114	0.116	1	1	MASP1
Pubmed	9705231	Tetranectin is a novel marker for myogenesis during embryonic development, muscle regeneration, and muscle cell differentiation in vitro.	Pubmed	0.0044	65.1	0.0114	0.116	1	1	CLEC3B
Pubmed	19373584	Two new examples of Hb St. Etienne [beta 92(F8)HisGln] in association with venous thrombosis.	GeneRIF	0.0044	65.1	0.0114	0.116	1	1	HBB
Pubmed	18339318	Rapid genotyping of known mutations and polymorphisms in beta-globin gene based on the DHPLC profile patterns of homoduplexes and heteroduplexes.	GeneRIF	0.0044	65.1	0.0114	0.116	1	1	HBB
Pubmed	9467009	The mouse Mid1 gene: implications for the pathogenesis of Opitz syndrome and the evolution of the mammalian pseudoautosomal region.	Pubmed	0.0044	65.1	0.0114	0.116	1	1	MID1
Pubmed	12011155	Mutations in the pancreatic secretory trypsin inhibitor gene (PSTI/SPINK1) rather than the cationic trypsinogen gene (PRSS1) are significantly associated with tropical calcific pancreatitis.	GeneRIF	0.0044	65.1	0.0114	0.116	1	1	SPINK1
Pubmed	6137189	Glutathione.	Pubmed	0.0044	65.1	0.0114	0.116	1	1	GSS
Pubmed	12552124	Involvement of bone morphogenetic protein 4 (BMP-4) in pituitary prolactinoma pathogenesis through a Smad/estrogen receptor crosstalk.	GeneRIF	0.0044	65.1	0.0114	0.116	1	1	BMP4
Pubmed	22045060	Quaternary structural parameters of the congenital cataract causing mutants of aA-crystallin.	GeneRIF	0.0044	65.1	0.0114	0.116	1	1	CRYAA
Pubmed	8573994	Molecular role of sulfated glycoprotein-1 (SGP-1/prosaposin) in Sertoli cells.	Pubmed	0.0044	65.1	0.0114	0.116	1	1	PSAP
Pubmed	20120618	Does narcolepsy symptom severity vary according to HLA-DQB1*0602 allele status?	GeneRIF	0.0044	65.1	0.0114	0.116	1	1	HLA-DQB1
Pubmed	16914509	Role of CD4(+)CD25(+) T regulatory cells in IL-2-induced vascular leak.	Pubmed	0.0044	65.1	0.0114	0.116	1	1	HLA-DQB1
Pubmed	11817677	Novel distribution of the secretory leucocyte proteinase inhibitor in kidney.	GeneRIF	0.0044	65.1	0.0114	0.116	1	1	SLPI
Pubmed	9326665	A human intermediate conductance calcium-activated potassium channel.	Pubmed	0.0044	65.1	0.0114	0.116	1	1	KCNN4
Pubmed	6088736	Metabolism of vitamin B-6 by human liver.	Pubmed	0.0044	65.1	0.0114	0.116	1	1	PDXK
Pubmed	23478260	Age-related macular degeneration-associated silent polymorphisms in HtrA1 impair its ability to antagonize insulin-like growth factor 1.	GeneRIF	0.0044	65.1	0.0114	0.116	1	1	HTRA1
Pubmed	9893169	Carboxypeptidase E (CPE) deficiency in mice with the fat mutation have reduced stomach function.	Pubmed	0.0044	65.1	0.0114	0.116	1	1	CPE
Pubmed	24848360	In vivo selective expression of thyroid hormone receptor a1 in endothelial cells attenuates myocardial injury in experimental myocardial infarction in mice.	Pubmed	0.0044	65.1	0.0114	0.116	1	1	THRA
Pubmed	14985074	Volume overload results in exaggerated cardiac hypertrophy in the atrial natriuretic peptide knockout mouse.	Pubmed	0.0044	65.1	0.0114	0.116	1	1	NPPA
Pubmed	22228629	SPINK3 modulates mouse sperm physiology through the reduction of nitric oxide level independently of its trypsin inhibitory activity.	GeneRIF	0.0044	65.1	0.0114	0.116	1	1	SPINK1
Pubmed	17260973	Design, synthesis, and evaluation of gamma-phosphono diester analogues of glutamate as highly potent inhibitors and active site probes of gamma-glutamyl transpeptidase.	GeneRIF	0.0044	65.1	0.0114	0.116	1	1	GGT1
Pubmed	14662830	Cutting edge: CD4+ T cell help can be essential for primary CD8+ T cell responses in vivo.	Pubmed	0.0044	65.1	0.0114	0.116	1	1	HLA-DQB1
Pubmed	9462726	Cloning of the murine TROP2 gene: conservation of a PIP2-binding sequence in the cytoplasmic domain of TROP-2.	GeneRIF	0.0044	65.1	0.0114	0.116	1	1	TACSTD2
Pubmed	21377214	Lack of association of the RTN4R genetic variations with risk of schizophrenia and SPEM abnormality in a Korean population.	GeneRIF	0.0044	65.1	0.0114	0.116	1	1	RTN4R
Pubmed	12649567	N34S, a pancreatitis associated SPINK1 mutation, is not associated with sporadic pancreatic cancer.	GeneRIF	0.0044	65.1	0.0114	0.116	1	1	SPINK1
Pubmed	10235444	Nucleotide structure and characterization of the murine gene encoding the endothelial cell protein C receptor.	Pubmed	0.0044	65.1	0.0114	0.116	1	1	PROCR
Pubmed	19406468	Intermediate conductance Ca2+-activated potassium channel (KCa3.1) in the inner mitochondrial membrane of human colon cancer cells.	GeneRIF	0.0044	65.1	0.0114	0.116	1	1	KCNN4
Pubmed	19277198	F- and G-actin concentrations in lamellipodia of moving cells.	GeneRIF	0.0044	65.1	0.0114	0.116	1	1	ACTA1

Category	ID	Name	Source	p-value	q-value Bonferroni	q-value FDR B&H	q-value FDR B&Y	Hit Count in Query List	Hit Count in Genome	Hit in Query List
Pubmed	19066726	Expression of p75NTR in fetal brain and medulloblastomas: evidence of a precursor cell marker and its persistence in neoplasia.	GeneRIF	0.0044	65.1	0.0114	0.116	1	1	NGFR
Pubmed	20586184	[Downregulation of AKR1B10 gene expression in colorectal cancer].	GeneRIF	0.0044	65.1	0.0114	0.116	1	1	AKR1B10
Pubmed	22198767	A single conformational transglutaminase 2 epitope contributed by three domains is critical for celiac antibody binding and effects.	GeneRIF	0.0044	65.1	0.0114	0.116	1	1	TGM2
Pubmed	16436049	Gene order in human alpha-globin locus is required for their temporal specific expressions.	GeneRIF	0.0044	65.1	0.0114	0.116	1	1	HBA2
Pubmed	15878337	Carboxypeptidases cathepsins X and B display distinct protein profile in human cells and tissues.	GeneRIF	0.0044	65.1	0.0114	0.116	1	1	CTSK
Pubmed	11867708	Structural basis for the guanine nucleotide-binding activity of tissue transglutaminase and its regulation of transamidation activity.	Pubmed	0.0044	65.1	0.0114	0.116	1	1	TGM2
Pubmed	11309368	Connexin 31 (GJB3) is expressed in the peripheral and auditory nerves and causes neuropathy and hearing impairment.	Pubmed	0.0044	65.1	0.0114	0.116	1	1	GJB3
Pubmed	14614212	Embryonic lethal effect of expressing a dominant negative mutant human thyroid hormone receptor alpha1 in mice.	GeneRIF	0.0044	65.1	0.0114	0.116	1	1	THRA
Pubmed	18396043	Late onset in dysferlinopathy widens the clinical spectrum.	GeneRIF	0.0044	65.1	0.0114	0.116	1	1	DYSF
Pubmed	10517637	Polycystin-L is a calcium-regulated cation channel permeable to calcium ions.	Pubmed	0.0044	65.1	0.0114	0.116	1	1	PKD2L1
Pubmed	24040120	DCLK1 regulates pluripotency and angiogenic factors via microRNA-dependent mechanisms in pancreatic cancer.	Pubmed	0.0044	65.1	0.0114	0.116	1	1	DCLK1
Pubmed	25594860	PAPSS2 deficiency causes androgen excess via impaired DHEA sulfation--in vitro and in vivo studies in a family harboring two novel PAPSS2 mutations.	Pubmed	0.0044	65.1	0.0114	0.116	1	1	PAPSS2
Pubmed	20547678	Cysteine 723 in the C-linker segment confers oxidative inhibition of hERG1 potassium channels.	GeneRIF	0.0044	65.1	0.0114	0.116	1	1	KCNH2
Pubmed	7663503	Chewing the fat.	Pubmed	0.0044	65.1	0.0114	0.116	1	1	CPE
Pubmed	23410823	Profiling of lens protease involved in generation of aA-66-80 crystallin peptide using an internally quenched protease substrate.	GeneRIF	0.0044	65.1	0.0114	0.116	1	1	CRYAA
Pubmed	11067927	Vesicular localization and characterization of a novel post-proline-cleaving aminodipeptidase, quiescent cell proline dipeptidase.	Pubmed	0.0044	65.1	0.0114	0.116	1	1	DPP7
Pubmed	18291403	Role of cathepsin K in structural changes in brachiocephalic artery during progression of atherosclerosis in apoE-deficient mice.	GeneRIF	0.0044	65.1	0.0114	0.116	1	1	CTSK
Pubmed	9886021	Central neuronal loss and behavioral impairment in mice lacking neurotrophin receptor p75.	Pubmed	0.0044	65.1	0.0114	0.116	1	1	NGFR
Pubmed	23583036	Structure and T cell inhibition properties of B7 family member, B7-H3.	Pubmed	0.0044	65.1	0.0114	0.116	1	1	CD276
Pubmed	18222980	The non-synonymous coding IKr-channel variant KCNH2-K897T is associated with atrial fibrillation: results from a systematic candidate gene-based analysis of KCNH2 (HERG).	GeneRIF	0.0044	65.1	0.0114	0.116	1	1	KCNH2
Pubmed	20855509	Inhibition of T cells provides protection against early invasive pneumococcal disease.	Pubmed	0.0044	65.1	0.0114	0.116	1	1	HLA-DQB1
Pubmed	23122413	Tissue transglutaminase colocalizes with extracellular matrix proteins in cerebral amyloid angiopathy.	GeneRIF	0.0044	65.1	0.0114	0.116	1	1	TGM2
Pubmed	15128423	The C-->G transition in the alpha 2-globin gene of a normal alpha alpha-chromosome is responsible for the Hb G-Philadelphia variant in Sardinians.	GeneRIF	0.0044	65.1	0.0114	0.116	1	1	HBA2
Pubmed	12702148	Divergent effects of two sequence variants of GJB3 (G12D and R32W) on the function of connexin 31 in vitro.	GeneRIF	0.0044	65.1	0.0114	0.116	1	1	GJB3
Pubmed	21818567	TG2, a novel extracellular protein with multiple functions.	GeneRIF	0.0044	65.1	0.0114	0.116	1	1	TGM2
Pubmed	21339484	Disrupted junctional membrane complexes and hyperactive ryanodine receptors after acute junctophilin knockdown in mice.	GeneRIF	0.0044	65.1	0.0114	0.116	1	1	JPH2
Pubmed	3459184	Structure of the human Ia-associated invariant (gamma)-chain gene: identification of 5' sequences shared with major histocompatibility complex class II genes.	Pubmed	0.0044	65.1	0.0114	0.116	1	1	CD74
Pubmed	20064500	Identification of NPPA variants associated with atrial fibrillation in a Chinese GeneID population.	GeneRIF	0.0044	65.1	0.0114	0.116	1	1	NPPA
Pubmed	23038033	Gelatinous drop-like corneal dystrophy.	GeneRIF	0.0044	65.1	0.0114	0.116	1	1	TACSTD2
Pubmed	15140746	Isoform switching from SM-B to SM-A myosin results in decreased contractility and altered expression of thin filament regulatory proteins.	Pubmed	0.0044	65.1	0.0114	0.116	1	1	MYH11
Pubmed	16010686	Dysferlin mutations in LGMD2B, Miyoshi myopathy, and atypical dysferlinopathies.	GeneRIF	0.0044	65.1	0.0114	0.116	1	1	DYSF
Pubmed	23443026	HLA-DQB1*02 and DQB1*06:03P are associated with peanut allergy.	Pubmed	0.0044	65.1	0.0114	0.116	1	1	HLA-DQB1
Pubmed	6526653	Hemoglobin Shelby [beta 131(H9) Gln----Lys] a correction to the structure of hemoglobin Deaconess and hemoglobin Leslie.	Pubmed	0.0044	65.1	0.0114	0.116	1	1	HBB
Pubmed	22681639	Up-regulated aldo-keto reductase family 1 member B10 in chronic hepatitis C: association with serum alpha-fetoprotein and hepatocellular carcinoma.	GeneRIF	0.0044	65.1	0.0114	0.116	1	1	AKR1B10
Pubmed	23841699	Fibulin-2 deficiency attenuates angiotensin II-induced cardiac hypertrophy by reducing transforming growth factor-ß signalling.	Pubmed	0.0044	65.1	0.0114	0.116	1	1	FBLN2
Pubmed	8292032	Characterization of interactions of nitric oxide with human hemoglobin A by infrared spectroscopy.	Pubmed	0.0044	65.1	0.0114	0.116	1	1	HBB
Pubmed	17098827	Expression of cathepsin K is regulated by shear stress in cultured endothelial cells and is increased in endothelium in human atherosclerosis.	GeneRIF	0.0044	65.1	0.0114	0.116	1	1	CTSK
Pubmed	11734632	A targeted dominant negative mutation of the thyroid hormone alpha 1 receptor causes increased mortality, infertility, and dwarfism in mice.	Pubmed	0.0044	65.1	0.0114	0.116	1	1	THRA

Category	ID	Name	Source	p-value	q-value Bonferroni	q-value FDR B&H	q-value FDR B&Y	Hit Count in Query List	Hit Count in Genome	Hit in Query List
Pubmed	25425698	High plasma levels of soluble endothelial protein C receptor are associated with increased mortality among children with cerebral malaria in Benin.	Pubmed	0.0044	65.1	0.0114	0.116	1	1	PROCR
Pubmed	16133068	Lymphocyte adenosine deaminase activity in children with idiopathic nephrotic syndrome.	GeneRIF	0.0044	65.1	0.0114	0.116	1	1	ADA
Pubmed	15528269	Characterization of the cholesterol recognition amino acid consensus sequence of the peripheral-type benzodiazepine receptor.	GeneRIF	0.0044	65.1	0.0114	0.116	1	1	TSPO
Pubmed	15529278	Mapping of the major psoriasis-susceptibility locus (PSORS1) in a 70-Kb interval around the corneodesmosin gene (CDSN).	Pubmed	0.0044	65.1	0.0114	0.116	1	1	CDSN
Pubmed	18521724	Reversal of chaperone activity loss of glycated alphaA-crystallin by a crosslink breaker.	GeneRIF	0.0044	65.1	0.0114	0.116	1	1	CRYAA
Pubmed	2101840	Hb Jacksonville [alpha 2 beta 2(54)(D5)Val----Asp]: a new unstable variant found in a patient with hemolytic anemia.	Pubmed	0.0044	65.1	0.0114	0.116	1	1	HBB
Pubmed	12468440	Bleomycin-induced pulmonary fibrosis is attenuated in gamma-glutamyl transpeptidase-deficient mice.	Pubmed	0.0044	65.1	0.0114	0.116	1	1	GGT1
Pubmed	23977271	Retinal ganglion cell dendritic atrophy in DBA/2J glaucoma.	Pubmed	0.0044	65.1	0.0114	0.116	1	1	GPNMB
Pubmed	18835984	Interaction of the mycobacterial heparin-binding hemagglutinin with actin, as evidenced by single-molecule force spectroscopy.	Pubmed	0.0044	65.1	0.0114	0.116	1	1	ACTA1
Pubmed	15362860	Cell-surface-associated tissue transglutaminase is a target of MMP-2 proteolysis.	GeneRIF	0.0044	65.1	0.0114	0.116	1	1	TGM2
Pubmed	721609	Hemoglobin J Altgeld Gardens. A hemoglobin variant with a substitution of the proximal histidine of the beta-chain.	Pubmed	0.0044	65.1	0.0114	0.116	1	1	HBB
Pubmed	10580105	Napsin A, a member of the aspartic protease family, is abundantly expressed in normal lung and kidney tissue and is expressed in lung adenocarcinomas.	Pubmed	0.0044	65.1	0.0114	0.116	1	1	NAPSA
Pubmed	24656094	Aldoketoreductase family 1B10 (AKR1B10) as a biomarker to distinguish hepatocellular carcinoma from benign liver lesions.	Pubmed	0.0044	65.1	0.0114	0.116	1	1	AKR1B10
Pubmed	20156184	Production of active MMP7 in E. coli and its application for metalloproteinase inhibitors screening.	GeneRIF	0.0044	65.1	0.0114	0.116	1	1	MMP7
Pubmed	7558046	Fine mapping of the human bone morphogenetic protein-4 gene (BMP4) to chromosome 14q22-q23 by in situ hybridization.	Pubmed	0.0044	65.1	0.0114	0.116	1	1	BMP4
Pubmed	9845367	Crystal structure and functional analysis of the HERG potassium channel N terminus: a eukaryotic PAS domain.	Pubmed	0.0044	65.1	0.0114	0.116	1	1	KCNH2
Pubmed	2187469	Genetic control of autoimmunity in type 1 diabetes.	Pubmed	0.0044	65.1	0.0114	0.116	1	1	HLA-DQB1
Pubmed	6982931	Biosynthesis and glycosylation of the invariant chain associated with HLA-DR antigens.	Pubmed	0.0044	65.1	0.0114	0.116	1	1	CD74
Pubmed	22075726	Hemoglobins S and C interfere with actin remodeling in Plasmodium falciparum-infected erythrocytes.	GeneRIF	0.0044	65.1	0.0114	0.116	1	1	HBB
Pubmed	18664625	BMP4 regulation of human megakaryocytic differentiation is involved in thrombopoietin signaling.	GeneRIF	0.0044	65.1	0.0114	0.116	1	1	BMP4
Pubmed	2364091	Purification and characterization of recombinant human activin B.	Pubmed	0.0044	65.1	0.0114	0.116	1	1	INHBB
Pubmed	19715983	Blockade of T-lymphocyte KCa3.1 and Kv1.3 channels as novel immunosuppression strategy to prevent kidney allograft rejection.	GeneRIF	0.0044	65.1	0.0114	0.116	1	1	KCNN4
Pubmed	9449718	Major histocompatibility complex class II molecules can protect from diabetes by positively selecting T cells with additional specificities.	Pubmed	0.0044	65.1	0.0114	0.116	1	1	HLA-DQB1
Pubmed	22043020	Unmasking potential intracellular roles for dysferlin through improved immunolabeling methods.	GeneRIF	0.0044	65.1	0.0114	0.116	1	1	DYSF
Pubmed	24427339	The M2 polarization of macrophage induced by fractalkine in the endometriotic milieu enhances invasiveness of endometrial stromal cells.	Pubmed	0.0044	65.1	0.0114	0.116	1	1	CX3CL1
Pubmed	22285112	Distribution of HLA-DQB1 alleles in patients with Kleine-Levin syndrome.	GeneRIF	0.0044	65.1	0.0114	0.116	1	1	HLA-DQB1
Pubmed	25964566	Serum Angiopoietin-like Protein 2 Improves Preoperative Detection of Lymph Node Metastasis in Colorectal Cancer.	Pubmed	0.0044	65.1	0.0114	0.116	1	1	ANGPTL2
Pubmed	11323016	Matrix metalloproteinase deficiencies do not impair cell-associated fibrinolytic activity.	Pubmed	0.0044	65.1	0.0114	0.116	1	1	MMP7
Pubmed	11334427	The HLA-DPB1--associated component of the IDDM1 and its relationship to the major loci HLA-DQB1, -DQA1, and -DRB1.	Pubmed	0.0044	65.1	0.0114	0.116	1	1	HLA-DQB1
Pubmed	24599027	RNA sequencing analysis and atrial natriuretic peptide production in patients with dilated and ischemic cardiomyopathy.	Pubmed	0.0044	65.1	0.0114	0.116	1	1	NPPA
Pubmed	17914098	Activin and estrogen crosstalk regulates transcription in human breast cancer cells.	GeneRIF	0.0044	65.1	0.0114	0.116	1	1	INHBB
Pubmed	19567322	Temporal induction of secretory leukocyte protease inhibitor (SLPI) in odontoblasts by lipopolysaccharide and wound infection.	GeneRIF	0.0044	65.1	0.0114	0.116	1	1	SLPI
Pubmed	12355371	Salivary secretory leukocyte protease inhibitor is associated with reduced transmission of human immunodeficiency virus type 1 through breast milk.	Pubmed	0.0044	65.1	0.0114	0.116	1	1	SLPI
Pubmed	12616493	Human fractalkine mediates leukocyte adhesion but not capture under physiological shear conditions; a mechanism for selective monocyte recruitment.	GeneRIF	0.0044	65.1	0.0114	0.116	1	1	CX3CL1
Pubmed	22832923	Primary polydipsia, but not accumulated ceramide, causes lethal renal damage in saposin D-deficient mice.	Pubmed	0.0044	65.1	0.0114	0.116	1	1	PSAP
Pubmed	17140601	Association of the gene encoding neurogranin with schizophrenia in males.	GeneRIF	0.0044	65.1	0.0114	0.116	1	1	NRGN
Pubmed	15319819	Inhibin B in pubertal development and pubertal disorders.	GeneRIF	0.0044	65.1	0.0114	0.116	1	1	INHBB
Pubmed	10215920	The role of p75NTR in modulating neurotrophin survival effects in developing motoneurons.	Pubmed	0.0044	65.1	0.0114	0.116	1	1	NGFR

Category	ID	Name	Source	p-value	q-value Bonferroni	q-value FDR B&H	q-value FDR B&Y	Hit Count in Query List	Hit Count in Genome	Hit in Query List
Pubmed	16707602	Clinical significance of TROP2 expression in colorectal cancer.	GeneRIF	0.0044	65.1	0.0114	0.116	1	1	TACSTD2
Pubmed	22323332	ß-Globin polymorphisms in Amerindian populations from the Brazilian Amazon.	GeneRIF	0.0044	65.1	0.0114	0.116	1	1	HBB
Pubmed	14718386	Molecular cloning and characterization of Mustang, a novel nuclear protein expressed during skeletal development and regeneration.	Pubmed	0.0044	65.1	0.0114	0.116	1	1	MUSTN1
Pubmed	9922386	Genomic sequence comparison of the human and mouse adenosine deaminase gene regions.	Pubmed	0.0044	65.1	0.0114	0.116	1	1	ADA
Pubmed	12005165	Role of matrix metalloproteinase-7 in angiogenesis associated with age-related macular degeneration.	GeneRIF	0.0044	65.1	0.0114	0.116	1	1	MMP7
Pubmed	3672126	A novel thyroid hormone receptor encoded by a cDNA clone from a human testis library.	Pubmed	0.0044	65.1	0.0114	0.116	1	1	THRA
Pubmed	15481886	The 'hot-spot' of Hb E [beta26(B8)Glu-->Lys] in Southeast Asia: beta-globin anomalies in the Lao Theung population of southern Laos.	Pubmed	0.0044	65.1	0.0114	0.116	1	1	HBB
Pubmed	16153302	Tissue transglutaminase-induced alterations in extracellular matrix inhibit tumor invasion.	GeneRIF	0.0044	65.1	0.0114	0.116	1	1	TGM2
Pubmed	15696117	Matrilysin (MMP-7) is a major matrix metalloproteinase upregulated in biliary atresia-associated liver fibrosis.	GeneRIF	0.0044	65.1	0.0114	0.116	1	1	MMP7
Pubmed	2010218	Rapid typing of HLA-DQB1 DNA polymorphism using nonradioactive oligonucleotide probes and amplified DNA.	Pubmed	0.0044	65.1	0.0114	0.116	1	1	HLA-DQB1
Pubmed	16216915	Rab coupling protein (RCP): a novel target of progesterone action in primate endometrium.	GeneRIF	0.0044	65.1	0.0114	0.116	1	1	RAB11FIP1
Pubmed	24302765	Dysferlin stabilizes stress-induced Ca2+ signaling in the transverse tubule membrane.	Pubmed	0.0044	65.1	0.0114	0.116	1	1	DYSF
Pubmed	21536673	Rescue of aberrant gating by a genetically encoded PAS (Per-Arnt-Sim) domain in several long QT syndrome mutant human ether-á-go-go-related gene potassium channels.	GeneRIF	0.0044	65.1	0.0114	0.116	1	1	KCNH2
Pubmed	23524335	IgE production in CD40/CD40L cross-talk of B and mast cells and mediator release via TGase 2 in mouse allergic asthma.	Pubmed	0.0044	65.1	0.0114	0.116	1	1	TGM2
Pubmed	23014777	Fractalkine is an independent predictor of mortality in patients with advanced heart failure.	GeneRIF	0.0044	65.1	0.0114	0.116	1	1	CX3CL1
Pubmed	22547081	The 18 kDa translocator protein influences angiogenesis, as well as aggressiveness, adhesion, migration, and proliferation of glioblastoma cells.	GeneRIF	0.0044	65.1	0.0114	0.116	1	1	TSPO
Pubmed	15813858	Co-inheritance of the Hb Sun Prairie mutation with a point mutation at 5'-UTR in the eastern Indian population.	Pubmed	0.0044	65.1	0.0114	0.116	1	1	HBA2
Pubmed	12698189	Aspartic proteinase napsin is a useful marker for diagnosis of primary lung adenocarcinoma.	GeneRIF	0.0044	65.1	0.0114	0.116	1	1	NAPSA
Pubmed	17956658	Investigation of the MYH11 gene in sporadic patients with an isolated persistently patent arterial duct.	GeneRIF	0.0044	65.1	0.0114	0.116	1	1	MYH11
Pubmed	20347338	Genetic polymorphisms in MMP7 and reduced serum levels associate with the development of bronchiolitis obliterans syndrome after lung transplantation.	GeneRIF	0.0044	65.1	0.0114	0.116	1	1	MMP7
Pubmed	18598717	Interferon-inducible Ifi200-family genes in systemic lupus erythematosus.	GeneRIF	0.0044	65.1	0.0114	0.116	1	1	IFI16
Pubmed	15165637	p75-NGFR expression in the human prenatal pituitary gland.	GeneRIF	0.0044	65.1	0.0114	0.116	1	1	NGFR
Pubmed	22647618	Epilepsy as a pyridoxine-dependent condition: quantified urinary biomarkers for status evaluation and monitoring antiepileptic treatment.	GeneRIF	0.0044	65.1	0.0114	0.116	1	1	PDXK
Pubmed	17397052	Molecular analysis and characterization of nine novel CTSK mutations in twelve patients affected by pycnodysostosis. Mutation in brief #961. Online.	GeneRIF	0.0044	65.1	0.0114	0.116	1	1	CTSK
Pubmed	20157553	What determines the switch between atrophic and neovascular forms of age related macular degeneration? - the role of BMP4 induced senescence.	GeneRIF	0.0044	65.1	0.0114	0.116	1	1	BMP4
Pubmed	1490342	Report on the sequence of DQB1*0301 gene in ocular cicatricial pemphigoid patients.	Pubmed	0.0044	65.1	0.0114	0.116	1	1	HLA-DQB1
Pubmed	19320736	Gpnmb is a melanosome-associated glycoprotein that contributes to melanocyte/keratinocyte adhesion in a RGD-dependent fashion.	GeneRIF	0.0044	65.1	0.0114	0.116	1	1	GPNMB
Pubmed	843082	The primary structure of the human pancreatic secretory trypsin inhibitor. Amino acid sequence o the reduced S-aminoethylated protein.	Pubmed	0.0044	65.1	0.0114	0.116	1	1	SPINK1
Pubmed	18779867	Interaction of C-terminal truncated human alphaA-crystallins with target proteins.	GeneRIF	0.0044	65.1	0.0114	0.116	1	1	CRYAA
Pubmed	22209006	Comparison of intracellular zinc signals in nonadherent lymphocytes from young-adult and elderly donors: role of zinc transporters (Zip family) and proinflammatory cytokines.	Pubmed	0.0044	65.1	0.0114	0.116	1	1	SLC39A2
Pubmed	21656687	Combined genomic and phenotype screening reveals secretory factor SPINK1 as an invasion and survival factor associated with patient prognosis in breast cancer.	GeneRIF	0.0044	65.1	0.0114	0.116	1	1	SPINK1
Pubmed	25218786	Known and new hemoglobin A2 variants in Thailand and implication for ß-thalassemia screening.	Pubmed	0.0044	65.1	0.0114	0.116	1	1	HBA2
Pubmed	3560174	New assignment of the adenosine deaminase gene locus to chromosome 20q13 X 11 by study of a patient with interstitial deletion 20q.	Pubmed	0.0044	65.1	0.0114	0.116	1	1	ADA
Pubmed	21273788	Circulating endothelial cell protein C receptor: endothelial regulation and cumulative impact of gender and A3 haplotype.	GeneRIF	0.0044	65.1	0.0114	0.116	1	1	PROCR
Pubmed	12176134	Expression of the smoothelin gene is mediated by alternative promoters.	Pubmed	0.0044	65.1	0.0114	0.116	1	1	SMTN
Pubmed	20453667	Determinants of N-terminal proatrial natriuretic peptide plasma levels in a survey of adult male people from Southern Italy.	GeneRIF	0.0044	65.1	0.0114	0.116	1	1	NPPA
Pubmed	16883575	hERG1 channels in human esophagus: evidence for their aberrant expression in the malignant progression of Barrett's esophagus.	GeneRIF	0.0044	65.1	0.0114	0.116	1	1	KCNH2
Pubmed	8388689	The contribution of I-Abm12 to phenotypic and functional alterations among T-cell subsets in NZB mice.	Pubmed	0.0044	65.1	0.0114	0.116	1	1	HLA-DQB1

Category	ID	Name	Source	p-value	q-value Bonferroni	q-value FDR B&H	q-value FDR B&Y	Hit Count in Query List	Hit Count in Genome	Hit in Query List
Pubmed	22032123	[Analysis of HLA-DQB1 polymorphism for patients with allergic rhinitis of Uygur and Han people in Xinjiang].	GeneRIF	0.0044	65.1	0.0114	0.116	1	1	HLA-DQB1
Pubmed	17617617	A novel role for defensins in intestinal homeostasis: regulation of IL-1beta secretion.	Pubmed	0.0044	65.1	0.0114	0.116	1	1	MMP7
Pubmed	12152662	The G7763C endothelial protein C receptor (EPCR) gene mutation: prevalence and association with DVT in the Irish population.	GeneRIF	0.0044	65.1	0.0114	0.116	1	1	PROCR
Pubmed	11013236	Intron-exon swapping of transglutaminase mRNA and neuronal Tau aggregation in Alzheimer's disease.	Pubmed	0.0044	65.1	0.0114	0.116	1	1	TGM2
Pubmed	18322197	Lipopolysaccharide-activated IL-10-secreting dendritic cells suppress experimental autoimmune uveoretinitis by MHCII-dependent activation of CD62L-expressing regulatory T cells.	Pubmed	0.0044	65.1	0.0114	0.116	1	1	CD74
Pubmed	16798613	Claudins--key pieces in the tight junction puzzle.	Pubmed	0.0044	65.1	0.0114	0.116	1	1	CLDN15
Pubmed	15102777	CD4-T-lymphocyte interactions with pneumolysin and pneumococci suggest a crucial protective role in the host response to pneumococcal infection.	Pubmed	0.0044	65.1	0.0114	0.116	1	1	HLA-DQB1
Pubmed	21244222	Signaling via IRAG is essential for NO/cGMP-dependent inhibition of platelet activation.	Pubmed	0.0044	65.1	0.0114	0.116	1	1	MRVI1
Pubmed	11159808	Human 3'-phosphoadenosine 5'-phosphosulfate synthetase: radiochemical enzymatic assay, biochemical properties, and hepatic variation.	Pubmed	0.0044	65.1	0.0114	0.116	1	1	PAPSS2
Pubmed	12528117	Increased salivary gland tissue expression of Fas, Fas ligand, cytotoxic T lymphocyte-associated antigen 4, and programmed cell death 1 in primary Sjögren's syndrome.	Pubmed	0.0044	65.1	0.0114	0.116	1	1	ORM2
Pubmed	25374060	Downregulation of CPE regulates cell proliferation and chemosensitivity in pancreatic cancer.	Pubmed	0.0044	65.1	0.0114	0.116	1	1	CPE
Pubmed	16929298	The cells and logic for mammalian sour taste detection.	GeneRIF	0.0044	65.1	0.0114	0.116	1	1	PKD2L1
Pubmed	23838009	Receptor-mediated Ca2+ and PKC signaling triggers the loss of cortical PKA compartmentalization through the redistribution of gravin.	Pubmed	0.0044	65.1	0.0114	0.116	1	1	AKAP12
Pubmed	24015048	Single nucleotide deletion mutation of KCNH2 gene is responsible for LQT syndrome in a 3-generation Korean family.	Pubmed	0.0044	65.1	0.0114	0.116	1	1	KCNH2
Pubmed	4141628	Hereditary hypotrichosis simplex of the scalp. Evidence for autosomal dominant inheritance.	Pubmed	0.0044	65.1	0.0114	0.116	1	1	CDSN
Pubmed	21593678	Adenosine deaminase activity in patients with Crohn's disease: distinction between active and nonactive disease.	GeneRIF	0.0044	65.1	0.0114	0.116	1	1	ADA
Pubmed	11682484	ASB-2 inhibits growth and promotes commitment in myeloid leukemia cells.	Pubmed	0.0044	65.1	0.0114	0.116	1	1	ASB2
Pubmed	8550095	Evolutionary origins of retroposon lineages of Mhc class II Ab alleles.	Pubmed	0.0044	65.1	0.0114	0.116	1	1	HLA-DQB1
Pubmed	21771585	Aspartate 458 of human glutathione synthetase is important for cooperativity and active site structure.	GeneRIF	0.0044	65.1	0.0114	0.116	1	1	GSS
Pubmed	14649318	Mild Hb S-beta(+)-thalassemia with a deletion of five nucleotides at the polyadenylation site of the beta-globin gene.	GeneRIF	0.0044	65.1	0.0114	0.116	1	1	HBB
Pubmed	15909997	Transglutaminase activity is present in highly purified nonsynaptosomal mouse brain and liver mitochondria.	Pubmed	0.0044	65.1	0.0114	0.116	1	1	TGM2
Pubmed	10077590	The Opitz syndrome gene product, MID1, associates with microtubules.	Pubmed	0.0044	65.1	0.0114	0.116	1	1	MID1
Pubmed	25041028	Could targeting secretory leukocyte protease inhibitor be an effective therapeutic option to prevent infections in acute liver failure?	Pubmed	0.0044	65.1	0.0114	0.116	1	1	SLPI
Pubmed	23268325	Nerve growth factor receptor (NGFR): a potential marker for specific molecular subtypes of breast cancer.	GeneRIF	0.0044	65.1	0.0114	0.116	1	1	NGFR
Pubmed	10569726	Hb Toulon [alpha77(EF6)Pro-->His]: a new variant due to a mutation in the alpha2 gene found during measurement of glycated hemoglobin.	Pubmed	0.0044	65.1	0.0114	0.116	1	1	HBA2
Pubmed	15535137	In vivo and in vitro dysferlin expression in human muscle satellite cells.	GeneRIF	0.0044	65.1	0.0114	0.116	1	1	DYSF
Pubmed	18063690	Cell-specific activation of the human skeletal alpha-actin by androgens.	GeneRIF	0.0044	65.1	0.0114	0.116	1	1	ACTA1
Pubmed	12368090	Transglutaminase 2: an enigmatic enzyme with diverse functions.	Pubmed	0.0044	65.1	0.0114	0.116	1	1	TGM2
Pubmed	15565159	Autocatalytic RNA cleavage in the human beta-globin pre-mRNA promotes transcription termination.	GeneRIF	0.0044	65.1	0.0114	0.116	1	1	HBB
Pubmed	20696859	B7-H3 augments the inflammatory response and is associated with human sepsis.	GeneRIF	0.0044	65.1	0.0114	0.116	1	1	CD276
Pubmed	18081706	Beta-thalassemia intermedia due to two novel mutations in the promoter region of the beta-globin gene.	GeneRIF	0.0044	65.1	0.0114	0.116	1	1	HBB
Pubmed	23558685	Dysferlin regulates cell adhesion in human monocytes.	GeneRIF	0.0044	65.1	0.0114	0.116	1	1	DYSF
Pubmed	22393412	Pathogenic connexin-31 forms constitutively active hemichannels to promote necrotic cell death.	GeneRIF	0.0044	65.1	0.0114	0.116	1	1	GJB3
Pubmed	2989274	Characterization of the human melanoma nerve growth factor receptor.	Pubmed	0.0044	65.1	0.0114	0.116	1	1	NGFR
Pubmed	15314238	Identification of 4Ig-B7-H3 as a neuroblastoma-associated molecule that exerts a protective role from an NK cell-mediated lysis.	GeneRIF	0.0044	65.1	0.0114	0.116	1	1	CD276
Pubmed	19233165	Myofibril assembly visualized by imaging N-RAP, alpha-actinin, and actin in living cardiomyocytes.	Pubmed	0.0044	65.1	0.0114	0.116	1	1	ACTA1
Pubmed	17999653	Identification of a novel HLA-DQB1 allele, HLA-DQB1*0632.	GeneRIF	0.0044	65.1	0.0114	0.116	1	1	HLA-DQB1
Pubmed	22912785	Two variants in the fibulin2 gene are associated with lower systolic blood pressure and decreased risk of hypertension.	GeneRIF	0.0044	65.1	0.0114	0.116	1	1	FBLN2
Pubmed	3182793	Mutant human adenosine deaminase alleles and their expression by transfection into fibroblasts.	Pubmed	0.0044	65.1	0.0114	0.116	1	1	ADA

Category	ID	Name	Source	p-value	q-value Bonferroni	q-value FDR B&H	q-value FDR B&Y	Hit Count in Query List	Hit Count in Genome	Hit in Query List
Pubmed	18844446	S(1)' and S(2)' subsite specificities of human plasma kallikrein and tissue kallikrein 1 for the hydrolysis of peptides derived from the bradykinin domain of human kininogen.	GeneRIF	0.0044	65.1	0.0114	0.116	1	1	KLK1
Pubmed	3917973	Mutation at I-A beta chain prevents experimental autoimmune myasthenia gravis.	Pubmed	0.0044	65.1	0.0114	0.116	1	1	HLA-DQB1
Pubmed	19775947	[Diagnosis of hemoglobinopathies in newborns in Venezuela hospitals].	GeneRIF	0.0044	65.1	0.0114	0.116	1	1	HBB
Pubmed	8877792	Organization of the mouse cardiac natriuretic peptide locus encoding BNP and ANP.	Pubmed	0.0044	65.1	0.0114	0.116	1	1	NPPA
Pubmed	8179899	Low-affinity nerve growth factor receptor is expressed during testicular morphogenesis and in germ cells at specific stages of spermatogenesis.	Pubmed	0.0044	65.1	0.0114	0.116	1	1	NGFR
Pubmed	22217259	A new ß chain hemoglobin variant with increased oxygen affinity: Hb Santa Giusta Sardegna [ß93(F9)Cys¿Trp; HBB c.282T>G].	GeneRIF	0.0044	65.1	0.0114	0.116	1	1	HBB
Pubmed	21388362	HLA-DQB1*05:06, a novel HLA-DQB1*05 allele identified by sequence-based typing.	GeneRIF	0.0044	65.1	0.0114	0.116	1	1	HLA-DQB1
Pubmed	18173453	Expression of secretory leukocyte proteinase inhibitor in the submandibular glands of AIDS patients.	GeneRIF	0.0044	65.1	0.0114	0.116	1	1	SLPI
Pubmed	24826782	The stimulating effects of nitric oxide on intermediate conductance Ca²¿-activated K¿ channels in human dermal fibroblasts through PKG pathways but not the PKA pathways.	Pubmed	0.0044	65.1	0.0114	0.116	1	1	KCNN4
Pubmed	15662224	Induction of tissue kallikrein in human carotid atheroma does not lead to kallikrein-kinins pathway activation.	GeneRIF	0.0044	65.1	0.0114	0.116	1	1	KLK1
Pubmed	21487810	Expression analysis and clinical evaluation of kallikrein-related peptidase 10 (KLK10) in colorectal cancer.	GeneRIF	0.0044	65.1	0.0114	0.116	1	1	KLK10
Pubmed	23993723	Decreased incidence of EPCR 4678G/C SNP in multiple myeloma patients with thrombosis.	Pubmed	0.0044	65.1	0.0114	0.116	1	1	PROCR
Pubmed	15855348	Kallikrein gene delivery improves serum glucose and lipid profiles and cardiac function in streptozotocin-induced diabetic rats.	GeneRIF	0.0044	65.1	0.0114	0.116	1	1	KLK1
Pubmed	17412917	Regulatory function of CD4+CD25+ T cells from Class II MHC-deficient mice in contact hypersensitivity responses.	Pubmed	0.0044	65.1	0.0114	0.116	1	1	HLA-DQB1
Pubmed	18674600	Cholinergic neurons of mouse intrinsic cardiac ganglia contain noradrenergic enzymes, norepinephrine transporters, and the neurotrophin receptors tropomyosin-related kinase A and p75.	Pubmed	0.0044	65.1	0.0114	0.116	1	1	NGFR
Pubmed	20943460	Serine protease HtrA1 expression in human hepatocellular carcinoma.	GeneRIF	0.0044	65.1	0.0114	0.116	1	1	HTRA1
Pubmed	21865585	Truncated IRAG variants modulate cGMP-mediated inhibition of human colonic smooth muscle cell contraction.	GeneRIF	0.0044	65.1	0.0114	0.116	1	1	MRVI1
Pubmed	22292854	Human ether-a-go-go-related gene K+ channels regulate shedding of leukemia cell-derived microvesicles.	GeneRIF	0.0044	65.1	0.0114	0.116	1	1	KCNH2
Pubmed	20412337	Double-stranded RNA-specific adenosine deaminase (DSRAD) gene mutation in a Chinese family with dyschromatosis symmetrica hereditaria (DSH).	GeneRIF	0.0044	65.1	0.0114	0.116	1	1	ADA
Pubmed	15973763	A novel splice mutation of HERG in a Chinese family with long QT syndrome.	GeneRIF	0.0044	65.1	0.0114	0.116	1	1	KCNH2
Pubmed	15544850	Altered renal response to acute volume expansion in transgenic rats harboring the human tissue kallikrein gene.	GeneRIF	0.0044	65.1	0.0114	0.116	1	1	KLK1
Pubmed	19620787	RCP is a human breast cancer-promoting gene with Ras-activating function.	GeneRIF	0.0044	65.1	0.0114	0.116	1	1	RAB11FIP1
Pubmed	21832094	Antidepressant-induced ubiquitination and degradation of the cardiac potassium channel hERG.	GeneRIF	0.0044	65.1	0.0114	0.116	1	1	KCNH2
Pubmed	10457277	Functional characterization of two promoters in the human bone morphogenetic protein-4 gene.	Pubmed	0.0044	65.1	0.0114	0.116	1	1	BMP4
Pubmed	16754522	ADA polymorphisms and asthma: a study in the Chinese Han population.	GeneRIF	0.0044	65.1	0.0114	0.116	1	1	ADA
Pubmed	24406159	AKR1B10, a good prognostic indicator in gastric cancer.	Pubmed	0.0044	65.1	0.0114	0.116	1	1	AKR1B10
Pubmed	21548205	[Stromal cells inhibit Kallikrein 7 expression in the epithelial cells of benign prostate hyperplasia].	GeneRIF	0.0044	65.1	0.0114	0.116	1	1	KLK7
Pubmed	2993162	Murine invariant chain gene: chromosomal assignment and segregation in recombinant inbred strains.	Pubmed	0.0044	65.1	0.0114	0.116	1	1	CD74
Pubmed	23273495	The effect of HLA-DQB1 alleles on virologic breakthroughs during chronic hepatitis B treatment with genetically low barrier drugs.	Pubmed	0.0044	65.1	0.0114	0.116	1	1	HLA-DQB1
Pubmed	24576777	Expression and prognostic significance of p21-activated kinase 6 in hepatocellular carcinoma.	Pubmed	0.0044	65.1	0.0114	0.116	1	1	PAK6
Pubmed	16855288	Structural polymorphism exhibited by a quasipalindrome present in the locus control region (LCR) of the human beta-globin gene cluster.	GeneRIF	0.0044	65.1	0.0114	0.116	1	1	HBB
Pubmed	22083892	Tissue transglutaminase, inflammation, and cancer: how intimate is the relationship?	GeneRIF	0.0044	65.1	0.0114	0.116	1	1	TGM2
Pubmed	21034655	Different apoptotic reactions of dorsal root ganglion A- and B-cells after sciatic nerve axotomy: effect of p75 neurotrophin receptor.	GeneRIF	0.0044	65.1	0.0114	0.116	1	1	NGFR
Pubmed	19916908	Dermatopontin is expressed in human liver and is downregulated in hepatocellular carcinoma.	GeneRIF	0.0044	65.1	0.0114	0.116	1	1	DPT
Pubmed	19122960	Detection of beta-globin gene mutations among Kelantan Malay thalassaemia patients by polymerase chain reaction restriction fragment length polymorphism.	GeneRIF	0.0044	65.1	0.0114	0.116	1	1	HBB
Pubmed	23555723	Abnormal apoptosis of trophoblastic cells is related to the up-regulation of CYP11A gene in placenta of preeclampsia patients.	Pubmed	0.0044	65.1	0.0114	0.116	1	1	CYP11A1
Pubmed	24217247	Heat shock protein 90-a mediates aldo-keto reductase 1B10 (AKR1B10) protein secretion through secretory lysosomes.	Pubmed	0.0044	65.1	0.0114	0.116	1	1	AKR1B10
Pubmed	9272950	Genetically engineered mice demonstrate that adenosine deaminase is essential for early postimplantation development.	Pubmed	0.0044	65.1	0.0114	0.116	1	1	ADA
Pubmed	21122200	[Effects of lipid-modulation and antiplatelet treatment on the endothelial lipase expression].	GeneRIF	0.0044	65.1	0.0114	0.116	1	1	LIPG

Category	ID	Name	Source	p-value	q-value Bonferroni	q-value FDR B&H	q-value FDR B&Y	Hit Count in Query List	Hit Count in Genome	Hit in Query List
Pubmed	17182778	Subcutaneous Nogo receptor removes brain amyloid-beta and improves spatial memory in Alzheimer's transgenic mice.	GeneRIF	0.0044	65.1	0.0114	0.116	1	1	RTN4R
Pubmed	15485805	Endothelial lipase modulates monocyte adhesion to the vessel wall. A potential role in inflammation.	GeneRIF	0.0044	65.1	0.0114	0.116	1	1	LIPG
Pubmed	11715025	Loss of SM-B myosin affects muscle shortening velocity and maximal force development.	Pubmed	0.0044	65.1	0.0114	0.116	1	1	MYH11
Pubmed	22396785	Changes in channel trafficking and protein stability caused by LQT2 mutations in the PAS domain of the HERG channel.	GeneRIF	0.0044	65.1	0.0114	0.116	1	1	KCNH2
Pubmed	4850241	A new haemoglobin J from Turkey--Hb Ankara (beta10 (A7) Ala-Asp).	Pubmed	0.0044	65.1	0.0114	0.116	1	1	HBB
Pubmed	18806692	The 18-kDa translocator protein, formerly known as the peripheral-type benzodiazepine receptor, confers proapoptotic and antineoplastic effects in a human colorectal cancer cell line.	GeneRIF	0.0044	65.1	0.0114	0.116	1	1	TSPO
Pubmed	12039963	Collagenase activity of cathepsin K depends on complex formation with chondroitin sulfate.	GeneRIF	0.0044	65.1	0.0114	0.116	1	1	CTSK
Pubmed	24452365	Hb Famagusta--analysis of a novel d-globin chain variant [HBD:c.60C>A] in four families with diverse globin genotypes.	Pubmed	0.0044	65.1	0.0114	0.116	1	1	HBA2
Pubmed	18287336	Dynamics of single potassium channel proteins in the plasma membrane of migrating cells.	GeneRIF	0.0044	65.1	0.0114	0.116	1	1	KCNN4
Pubmed	10521459	Cell surface localization of tissue transglutaminase is dependent on a fibronectin-binding site in its N-terminal beta-sandwich domain.	Pubmed	0.0044	65.1	0.0114	0.116	1	1	TGM2
Pubmed	1581238	Hb H disease in a Turkish family resulting from the interaction of a deletional alpha-thalassaemia-1 and a newly discovered poly A mutation.	Pubmed	0.0044	65.1	0.0114	0.116	1	1	HBA2
Pubmed	22361821	A bimodal influence of thyroid hormone on cerebellum oligodendrocyte differentiation.	GeneRIF	0.0044	65.1	0.0114	0.116	1	1	THRA
Pubmed	19912255	Anti-transglutaminase antibodies in non-coeliac children suffering from infectious diseases.	GeneRIF	0.0044	65.1	0.0114	0.116	1	1	TGM2
Pubmed	25509351	[CD271 enrichment does not help isolating mesenchymal stromal cells from].	Pubmed	0.0044	65.1	0.0114	0.116	1	1	NGFR
Pubmed	12530664	Steroid deficiency syndromes in mice with targeted disruption of Cyp11a1.	GeneRIF	0.0044	65.1	0.0114	0.116	1	1	CYP11A1
Pubmed	12663458	A promoter mutation in the erythroid-specific 5-aminolevulinate synthase (ALAS2) gene causes X-linked sideroblastic anemia.	GeneRIF	0.0044	65.1	0.0114	0.116	1	1	ALAS2
Pubmed	19218287	Prominent role of KCa3.1 in endothelium-derived hyperpolarizing factor-type dilations and conducted responses in the microcirculation in vivo.	GeneRIF	0.0044	65.1	0.0114	0.116	1	1	KCNN4
Pubmed	8012357	A gene for autosomal recessive limb-girdle muscular dystrophy maps to chromosome 2p.	Pubmed	0.0044	65.1	0.0114	0.116	1	1	DYSF
Pubmed	25260909	Expression and integrity of dermatopontin in chronic cutaneous wounds: a crucial factor in impaired wound healing.	Pubmed	0.0044	65.1	0.0114	0.116	1	1	DPT
Pubmed	17310281	Binding of matrilysin-1 to human epithelial cells promotes its activity.	GeneRIF	0.0044	65.1	0.0114	0.116	1	1	MMP7
Pubmed	12719657	The regulation of bone resorption in tooth formation and eruption processes in mouse alveolar crest devoid of cathepsin k.	GeneRIF	0.0044	65.1	0.0114	0.116	1	1	CTSK
Pubmed	24698228	Cardio-reno-hepatic interactions in acute heart failure: the role of ¿-glutamyl transferase.	Pubmed	0.0044	65.1	0.0114	0.116	1	1	GGT1
Pubmed	20140874	[A novel mutation of the KCNH2 gene in a family with congenital long QT syndrome].	GeneRIF	0.0044	65.1	0.0114	0.116	1	1	KCNH2
Pubmed	23425204	Association in cis of the mutations +20 (C>T) in the 5' untranslated region and IVS-II-745 (C>G) on the ß-globin gene.	GeneRIF	0.0044	65.1	0.0114	0.116	1	1	HBB
Pubmed	12484634	Hb Manawatu [alpha 37(C2)Pro-->Leu]: a new mildly unstable mutation at an invariant proline residue.	Pubmed	0.0044	65.1	0.0114	0.116	1	1	HBA2
Pubmed	24654459	Assessment of the tumor-associated trypsin inhibitor (TATI) marker in patients with carcinoma of the uterine body 17 years after treatment.	Pubmed	0.0044	65.1	0.0114	0.116	1	1	SPINK1
Pubmed	8914737	Novel missense mutation in the cyclic nucleotide-binding domain of HERG causes long QT syndrome.	Pubmed	0.0044	65.1	0.0114	0.116	1	1	KCNH2
Pubmed	23841802	Mannan binding lectin-associated serine protease 1 is induced by hepatitis C virus infection and activates human hepatic stellate cells.	Pubmed	0.0044	65.1	0.0114	0.116	1	1	MASP1
Pubmed	22539971	DCLK1 variants are associated across schizophrenia and attention deficit/hyperactivity disorder.	GeneRIF	0.0044	65.1	0.0114	0.116	1	1	DCLK1
Pubmed	15075392	BMP4 supports self-renewal of embryonic stem cells by inhibiting mitogen-activated protein kinase pathways.	GeneRIF	0.0044	65.1	0.0114	0.116	1	1	BMP4
Pubmed	18593905	Frequent inactivation of a putative tumor suppressor, angiopoietin-like protein 2, in ovarian cancer.	GeneRIF	0.0044	65.1	0.0114	0.116	1	1	ANGPTL2
Pubmed	10727405	The heparin-binding site in tetranectin is located in the N-terminal region and binding does not involve the carbohydrate recognition domain.	Pubmed	0.0044	65.1	0.0114	0.116	1	1	CLEC3B
Pubmed	10518938	One single mRNA encodes the centrosomal protein CCD41 and the endothelial cell protein C receptor (EPCR).	Pubmed	0.0044	65.1	0.0114	0.116	1	1	PROCR
Pubmed	15769254	Rab coupling protein is selectively degraded by calpain in a Ca2+-dependent manner.	GeneRIF	0.0044	65.1	0.0114	0.116	1	1	RAB11FIP1
Pubmed	18049427	The role of osteoactivin-derived peptides in osteoblast differentiation.	GeneRIF	0.0044	65.1	0.0114	0.116	1	1	GPNMB
Pubmed	19205970	Molecular heterogeneity of beta-thalassemia in Algeria: how to face up to a major health problem.	GeneRIF	0.0044	65.1	0.0114	0.116	1	1	HBB
Pubmed	8525485	HLA class II region nucleotide sequences, 1995.	Pubmed	0.0044	65.1	0.0114	0.116	1	1	HLA-DQB1
Pubmed	21645211	AKR1B10 expression is associated with less aggressive hepatocellular carcinoma: a clinicopathological study of 168 cases.	GeneRIF	0.0044	65.1	0.0114	0.116	1	1	AKR1B10

Category	ID	Name	Source	p-value	q-value Bonferroni	q-value FDR B&H	q-value FDR B&Y	Hit Count in Query List	Hit Count in Genome	Hit in Query List
Pubmed	21840417	Novel participation of transglutaminase-2 through c-Jun N-terminal kinase activation in sphingosylphosphorylcholine-induced keratin reorganization of PANC-1 cells.	GeneRIF	0.0044	65.1	0.0114	0.116	1	1	TGM2
Pubmed	20416035	A retroviral mutagenesis screen identifies Cd74 as a common insertion site in murine B-lymphomas and reveals the existence of a novel IFNgamma-inducible Cd74 isoform.	GeneRIF	0.0044	65.1	0.0114	0.116	1	1	CD74
Pubmed	18081157	Evidence for association between genetic variants of p75 neurotrophin receptor (p75NTR) gene and antidepressant treatment response in Chinese major depressive disorder.	GeneRIF	0.0044	65.1	0.0114	0.116	1	1	NGFR
Pubmed	24471793	A new hemoglobin variant: Hb Henan [ß90(F6)Glu¿¿¿Gln; HBB: c.271G¿<¿C].	Pubmed	0.0044	65.1	0.0114	0.116	1	1	HBB
Pubmed	10921784	1H, 13C and 15N resonance assignments of the DNA binding domain of the human forkhead transcription factor AFX.	Pubmed	0.0044	65.1	0.0114	0.116	1	1	FOXO4
Pubmed	11739386	Corneodesmosin, a component of epidermal corneocyte desmosomes, displays homophilic adhesive properties.	Pubmed	0.0044	65.1	0.0114	0.116	1	1	CDSN
Pubmed	15754321	p75NTR-mediated signaling promotes the survival of myoblasts and influences muscle strength.	GeneRIF	0.0044	65.1	0.0114	0.116	1	1	NGFR
Pubmed	18617776	The SPINK1 N34S variant is associated with acute pancreatitis.	GeneRIF	0.0044	65.1	0.0114	0.116	1	1	SPINK1
Pubmed	20228172	Adaptations of the autonomous nervous system controlling heart rate are impaired by a mutant thyroid hormone receptor-alpha1.	GeneRIF	0.0044	65.1	0.0114	0.116	1	1	THRA
Pubmed	22935920	CD74: a potential novel target for triple-negative breast cancer.	GeneRIF	0.0044	65.1	0.0114	0.116	1	1	CD74
Pubmed	12668734	Nonmuscle Myosin motor of smooth muscle.	Pubmed	0.0044	65.1	0.0114	0.116	1	1	MYH11
Pubmed	18673094	Serum levels of adenosine deaminase and pregnancy-related hormones in hyperemesis gravidarum.	GeneRIF	0.0044	65.1	0.0114	0.116	1	1	ADA
Pubmed	12270761	Locally different role of atrial natriuretic peptide (ANP) in the pericardial fluid.	GeneRIF	0.0044	65.1	0.0114	0.116	1	1	NPPA
Pubmed	25057925	Regulation of endothelial cell inflammation and lung polymorphonuclear lymphocyte infiltration by transglutaminase 2.	Pubmed	0.0044	65.1	0.0114	0.116	1	1	TGM2
Pubmed	24886585	The associations between endothelial lipase 584C/T polymorphism and HDL-C level and coronary heart disease susceptibility: a meta-analysis.	Pubmed	0.0044	65.1	0.0114	0.116	1	1	LIPG
Pubmed	16797397	Loss of podocyte dysferlin expression is associated with minimal change nephropathy.	GeneRIF	0.0044	65.1	0.0114	0.116	1	1	DYSF
Pubmed	23841782	Functional evaluation of a novel tooth agenesis-associated bone morphogenetic protein 4 prodomain mutation.	Pubmed	0.0044	65.1	0.0114	0.116	1	1	BMP4
Pubmed	18539145	Atrial natriuretic peptide stability.	GeneRIF	0.0044	65.1	0.0114	0.116	1	1	NPPA
Pubmed	18052077	GTP-binding-defective forms of tissue transglutaminase trigger cell death.	GeneRIF	0.0044	65.1	0.0114	0.116	1	1	TGM2
Pubmed	10631115	Impaired feedback of gastric functions in carboxypeptidase E-deficient mice.	Pubmed	0.0044	65.1	0.0114	0.116	1	1	CPE
Pubmed	7558871	Hb Bibba or alpha 2 136(H19)Leu-->Pro beta 2 in a Caucasian family from Alabama.	Pubmed	0.0044	65.1	0.0114	0.116	1	1	HBA2
Pubmed	15905580	Transglutaminase type II is a key element in the regulation of the anti-inflammatory response elicited by apoptotic cell engulfment.	GeneRIF	0.0044	65.1	0.0114	0.116	1	1	TGM2
Pubmed	21310466	Quantitative assessment of AKAP12 promoter methylation in human prostate cancer using methylation-sensitive high-resolution melting: correlation with Gleason score.	GeneRIF	0.0044	65.1	0.0114	0.116	1	1	AKAP12
Pubmed	20434541	The small heat shock protein alphaA-crystallin is expressed in pancreas and acts as a negative regulator of carcinogenesis.	GeneRIF	0.0044	65.1	0.0114	0.116	1	1	CRYAA
Pubmed	15757857	Changes in serum inhibin B during normal male puberty.	GeneRIF	0.0044	65.1	0.0114	0.116	1	1	INHBB
Pubmed	23704086	Transglutaminase 2 inhibition found to induce p53 mediated apoptosis in renal cell carcinoma.	Pubmed	0.0044	65.1	0.0114	0.116	1	1	TGM2
Pubmed	17042915	Altering extracellular potassium concentration does not modulate drug block of human ether-a-go-go-related gene (hERG) channels.	GeneRIF	0.0044	65.1	0.0114	0.116	1	1	KCNH2
Pubmed	18490779	HLA-DQ6 (DQB1*0601)-restricted T cells protect against experimental autoimmune encephalomyelitis in HLA-DR3.DQ6 double-transgenic mice by generating anti-inflammatory IFN-gamma.	Pubmed	0.0044	65.1	0.0114	0.116	1	1	HLA-DQB1
Pubmed	12112311	Matrilysin (matrix metalloproteinase-7) expression in ulcerative colitis-related tumorigenesis.	GeneRIF	0.0044	65.1	0.0114	0.116	1	1	MMP7
Pubmed	8566783	Cloning of cDNA and genomic structure of the mouse gamma-glutamyl transpeptidase-encoding gene.	Pubmed	0.0044	65.1	0.0114	0.116	1	1	GGT1
Pubmed	25082449	Functional peptide of dermatopontin produces fibrinogen fibrils and modifies its biological activity.	Pubmed	0.0044	65.1	0.0114	0.116	1	1	DPT
Pubmed	22105615	Human myxomatous mitral valve prolapse: role of bone morphogenetic protein 4 in valvular interstitial cell activation.	GeneRIF	0.0044	65.1	0.0114	0.116	1	1	BMP4
Pubmed	24902367	Increased dysferlin expression in Duchenne muscular dystrophy.	Pubmed	0.0044	65.1	0.0114	0.116	1	1	DYSF
Pubmed	23333801	Perivascular adipose tissue-secreted angiopoietin-like protein 2 (Angptl2) accelerates neointimal hyperplasia after endovascular injury.	GeneRIF	0.0044	65.1	0.0114	0.116	1	1	ANGPTL2
Pubmed	11843482	Accelerated inactivation of voltage-dependent K+ outward current in cardiomyocytes from thyroid hormone receptor alpha1-deficient mice.	GeneRIF	0.0044	65.1	0.0114	0.116	1	1	THRA
Pubmed	18840928	Cathepsin K regulates adipocyte differentiation: possible involvement of type I collagen degradation.	GeneRIF	0.0044	65.1	0.0114	0.116	1	1	CTSK
Pubmed	22900900	A novel mutation in the HTRA1 gene identified in Chinese CARASIL pedigree.	GeneRIF	0.0044	65.1	0.0114	0.116	1	1	HTRA1
Pubmed	18771417	Association of bone morphogenetic protein 4 gene polymorphisms with nonsyndromic cleft lip with or without cleft palate in Chinese children.	GeneRIF	0.0044	65.1	0.0114	0.116	1	1	BMP4
Pubmed	21335488	Secretory leukocyte protease inhibitor plays an important role in the regulation of allergic asthma in mice.	GeneRIF	0.0044	65.1	0.0114	0.116	1	1	SLPI

Category	ID	Name	Source	p-value	q-value Bonferroni	q-value FDR B&H	q-value FDR B&Y	Hit Count in Query List	Hit Count in Genome	Hit in Query List
Pubmed	18663291	Spiral ganglion outgrowth and hearing development in p75-deficient mice.	GeneRIF	0.0044	65.1	0.0114	0.116	1	1	NGFR
Pubmed	17894837	Beta-globin gene cluster polymorphisms are strongly associated with severity of HbE/beta(0)-thalassemia.	GeneRIF	0.0044	65.1	0.0114	0.116	1	1	HBB
Pubmed	21365880	[Adenosine deaminase as costimulatory molecule and marker of cellular immunity].	GeneRIF	0.0044	65.1	0.0114	0.116	1	1	ADA
Pubmed	11355022	Analysis of the human pancreatic secretory trypsin inhibitor (PSTI) gene mutations in Japanese patients with chronic pancreatitis.	Pubmed	0.0044	65.1	0.0114	0.116	1	1	SPINK1
Pubmed	22246095	A/G Polymorphism of the MMP-7 Gene Promoter Region in Colorectal Cancer.	GeneRIF	0.0044	65.1	0.0114	0.116	1	1	MMP7
Pubmed	18951638	CX3CL1/fractalkine regulates branching and migration of monocyte-derived cells in the mouse olfactory epithelium.	GeneRIF	0.0044	65.1	0.0114	0.116	1	1	CX3CL1
Pubmed	9380751	hSK4, a member of a novel subfamily of calcium-activated potassium channels.	Pubmed	0.0044	65.1	0.0114	0.116	1	1	KCNN4
Pubmed	25065854	A novel HBA2 gene conversion in cis or trans: "a12 allele" in a Saudi population.	Pubmed	0.0044	65.1	0.0114	0.116	1	1	HBA2
Pubmed	17178901	A systematic assessment of common genetic variation in CYP11A and risk of breast cancer.	GeneRIF	0.0044	65.1	0.0114	0.116	1	1	CYP11A1
Pubmed	10998087	Geographical differences within Finland in the frequency of HLA-DQ genotypes associated with type 1 diabetes susceptibility. The Childhood Diabetes in Finland Study Group.	GeneRIF	0.0044	65.1	0.0114	0.116	1	1	HLA-DQB1
Pubmed	22042554	Down-regulation of matrix metalloproteinase-7 inhibits metastasis of human anaplastic thyroid cancer cell line.	GeneRIF	0.0044	65.1	0.0114	0.116	1	1	MMP7
Pubmed	19153557	Neural fractalkine expression is closely linked to pain and pancreatic neuritis in human chronic pancreatitis.	GeneRIF	0.0044	65.1	0.0114	0.116	1	1	CX3CL1
Pubmed	18292231	Border formation in a Bmp gradient reduced to single dissociated cells.	GeneRIF	0.0044	65.1	0.0114	0.116	1	1	BMP4
Pubmed	11008920	CYP1A1 I462V genetic polymorphism and lung cancer risk in a cohort of men in Shanghai, China.	GeneRIF	0.0044	65.1	0.0114	0.116	1	1	CYP11A1
Pubmed	16887237	Expression and p75 neurotrophin receptor dependence of cholesterol synthetic enzymes in adult mouse brain.	GeneRIF	0.0044	65.1	0.0114	0.116	1	1	NGFR
Pubmed	22266311	Mechanical stretch suppresses BMP4 induction of stem cell adipogenesis via upregulating ERK but not through downregulating Smad or p38.	GeneRIF	0.0044	65.1	0.0114	0.116	1	1	BMP4
Pubmed	20676023	Transglutaminase 2 inhibits apoptosis induced by calcium- overload through down-regulation of Bax.	GeneRIF	0.0044	65.1	0.0114	0.116	1	1	TGM2
Pubmed	7631155	Mechanisms by which the I-ABM12 mutation influences susceptibility to experimental myasthenia gravis: a study in homozygous and heterozygous mice.	Pubmed	0.0044	65.1	0.0114	0.116	1	1	HLA-DQB1
Pubmed	23951042	Cathepsin K is present in invasive oral tongue squamous cell carcinoma in vivo and in vitro.	Pubmed	0.0044	65.1	0.0114	0.116	1	1	CTSK
Pubmed	23307658	Changes in Spontaneous firing patterns of cerebellar Purkinje cells in p75 knockout mice.	Pubmed	0.0044	65.1	0.0114	0.116	1	1	NGFR
Pubmed	23314172	Detection of histone modifications at specific gene loci in single cells in histological sections.	Pubmed	0.0044	65.1	0.0114	0.116	1	1	MYH11
Pubmed	9126484	Linkage analysis of progressive hearing loss in five extended families maps the DFNA2 gene to a 1.25-Mb region on chromosome 1p.	Pubmed	0.0044	65.1	0.0114	0.116	1	1	GJB3
Pubmed	17293393	Probing the outer mouth structure of the HERG channel with peptide toxin footprinting and molecular modeling.	GeneRIF	0.0044	65.1	0.0114	0.116	1	1	KCNH2
Pubmed	17420287	VEGFR-1 (FLT-1), beta1 integrin, and hERG K+ channel for a macromolecular signaling complex in acute myeloid leukemia: role in cell migration and clinical outcome.	GeneRIF	0.0044	65.1	0.0114	0.116	1	1	KCNH2
Pubmed	19551681	HLA-DQB1 subtypes predict diabetic retinopathy in patients with type I diabetes mellitus.	GeneRIF	0.0044	65.1	0.0114	0.116	1	1	HLA-DQB1
Pubmed	19201861	Transglutaminase 2 is needed for the formation of an efficient phagocyte portal in macrophages engulfing apoptotic cells.	GeneRIF	0.0044	65.1	0.0114	0.116	1	1	TGM2
Pubmed	9642238	Identification and characterization of a conserved erythroid-specific enhancer located in intron 8 of the human 5-aminolevulinate synthase 2 gene.	Pubmed	0.0044	65.1	0.0114	0.116	1	1	ALAS2
Pubmed	7599635	Four new adenosine deaminase mutations, altering a zinc-binding histidine, two conserved alanines, and a 5' splice site.	Pubmed	0.0044	65.1	0.0114	0.116	1	1	ADA
Pubmed	2410423	Identification of functional murine adenosine deaminase cDNA clones by complementation in Escherichia coli.	Pubmed	0.0044	65.1	0.0114	0.116	1	1	ADA
Pubmed	9705284	cDNA cloning and functional characterization of the mouse Ca2+-gated K+ channel, mIK1. Roles in regulatory volume decrease and erythroid differentiation.	Pubmed	0.0044	65.1	0.0114	0.116	1	1	KCNN4
Pubmed	20644175	Dendritic cells continue to capture and present antigens after maturation in vivo.	Pubmed	0.0044	65.1	0.0114	0.116	1	1	HLA-DQB1
Pubmed	12860969	Rho activation patterns after spinal cord injury and the role of activated Rho in apoptosis in the central nervous system.	GeneRIF	0.0044	65.1	0.0114	0.116	1	1	NGFR
Pubmed	23672273	Hb AHVAZ [a83(F4)Leu¿Arg, CTG>CGG (a2); HBA2: c.251T>G],a new hemoglobin variant of the a2-globin gene.	Pubmed	0.0044	65.1	0.0114	0.116	1	1	HBA2
Pubmed	21113139	Fibrolamellar carcinomas are positive for CD68.	GeneRIF	0.0044	65.1	0.0114	0.116	1	1	CD68
Pubmed	23355199	A broad survey of cathepsin K immunoreactivity in human neoplasms.	GeneRIF	0.0044	65.1	0.0114	0.116	1	1	CTSK
Pubmed	11867764	Conserved tryptophan in the core domain of transglutaminase is essential for catalytic activity.	Pubmed	0.0044	65.1	0.0114	0.116	1	1	TGM2
Pubmed	12013553	Bone morphogenetic protein 4 is involved in cusp formation in molar tooth germ of mice.	GeneRIF	0.0044	65.1	0.0114	0.116	1	1	BMP4
Pubmed	8970733	The mammalian myosin heavy chain gene family.	Pubmed	0.0044	65.1	0.0114	0.116	1	1	MYH11
Pubmed	18793760	The propensity for deamidation and transamidation of peptides by transglutaminase 2 is dependent on substrate affinity and reaction conditions.	GeneRIF	0.0044	65.1	0.0114	0.116	1	1	TGM2

Category	ID	Name	Source	p-value	q-value Bonferroni	q-value FDR B&H	q-value FDR B&Y	Hit Count in Query List	Hit Count in Genome	Hit in Query List
Pubmed	7620186	The role of the erythroid-specific delta-aminolevulinate synthase gene expression in erythroid heme synthesis.	Pubmed	0.0044	65.1	0.0114	0.116	1	1	ALAS2
Pubmed	11170080	Bradycardia-induced long QT syndrome caused by a de novo missense mutation in the S2-S3 inner loop of HERG.	Pubmed	0.0044	65.1	0.0114	0.116	1	1	KCNH2
Pubmed	15882565	Degenerative knee joint disease in mice lacking 3'-phosphoadenosine 5'-phosphosulfate synthetase 2 (Papss2) activity: a putative model of human PAPSS2 deficiency-associated arthrosis.	Pubmed	0.0044	65.1	0.0114	0.116	1	1	PAPSS2
Pubmed	20872567	Elevated adenosine deaminase levels in celiac disease.	GeneRIF	0.0044	65.1	0.0114	0.116	1	1	ADA
Pubmed	23585521	KCa3.1 modulates neuroblast migration along the rostral migratory stream (RMS) in vivo.	Pubmed	0.0044	65.1	0.0114	0.116	1	1	KCNN4
Pubmed	15652345	Proliferative effects of apical, but not basal, matrix metalloproteinase-7 activity in polarized MDCK cells.	GeneRIF	0.0044	65.1	0.0114	0.116	1	1	MMP7
Pubmed	22739370	Oral vitamin A and retinoic acid supplementation stimulates antibody production and splenic Stra6 expression in tetanus toxoid-immunized mice.	GeneRIF	0.0044	65.1	0.0114	0.116	1	1	STRA6
Pubmed	10996323	Characterization of S818L mutation in HERG C-terminus in LQT2. Modification of activation-deactivation gating properties.	Pubmed	0.0044	65.1	0.0114	0.116	1	1	KCNH2
Pubmed	18473944	Building quantitative relationship between changed sequence and changed oxygen affinity in human hemoglobin beta-chain.	GeneRIF	0.0044	65.1	0.0114	0.116	1	1	HBB
Pubmed	11423178	Immune related genetic polymorphisms and schizophrenia among the Chinese.	GeneRIF	0.0044	65.1	0.0114	0.116	1	1	HLA-DQB1
Pubmed	23785517	Fibulin-2 is a driver of malignant progression in lung adenocarcinoma.	Pubmed	0.0044	65.1	0.0114	0.116	1	1	FBLN2
Pubmed	7646467	Sequencing and expression of a cDNA for human glutathione synthetase.	Pubmed	0.0044	65.1	0.0114	0.116	1	1	GSS
Pubmed	17965490	Can gamma-glutamyltransferase be an additional marker of arterial stiffness?	GeneRIF	0.0044	65.1	0.0114	0.116	1	1	GGT1
Pubmed	17973972	Presbyopia and heat: changes associated with aging of the human lens suggest a functional role for the small heat shock protein, alpha-crystallin, in maintaining lens flexibility.	GeneRIF	0.0044	65.1	0.0114	0.116	1	1	CRYAA
Pubmed	20943049	A study of Adenosine-Deaminase genetic polymorphism in rheumatoid arthritis.	GeneRIF	0.0044	65.1	0.0114	0.116	1	1	ADA
Pubmed	18358633	AKR1B10 in usual interstitial pneumonia: expression in squamous metaplasia in association with smoking and lung cancer.	GeneRIF	0.0044	65.1	0.0114	0.116	1	1	AKR1B10
Pubmed	23736297	Cardiac a-actin over-expression therapy in dominant ACTA1 disease.	Pubmed	0.0044	65.1	0.0114	0.116	1	1	ACTA1
Pubmed	12242335	Beta -Globin mRNA decay in erythroid cells: UG site-preferred endonucleolytic cleavage that is augmented by a premature termination codon.	GeneRIF	0.0044	65.1	0.0114	0.116	1	1	HBB
Pubmed	2025281	Secretion of sphingolipid hydrolase activator precursor, prosaposin.	Pubmed	0.0044	65.1	0.0114	0.116	1	1	PSAP
Pubmed	15545267	Regulation of P-Rex1 by phosphatidylinositol (3,4,5)-trisphosphate and Gbetagamma subunits.	GeneRIF	0.0044	65.1	0.0114	0.116	1	1	PREX1
Pubmed	23022675	RNA interference targeting E637K mutation rescues hERG channel currents and restores its kinetic properties.	GeneRIF	0.0044	65.1	0.0114	0.116	1	1	KCNH2
Pubmed	17363620	From T-tubule to sarcolemma: damage-induced dysferlin translocation in early myogenesis.	GeneRIF	0.0044	65.1	0.0114	0.116	1	1	DYSF
Pubmed	21362311	Relationship between endothelial cell protein C receptor gene 6936A/G polymorphisms and deep venous thrombosis.	GeneRIF	0.0044	65.1	0.0114	0.116	1	1	PROCR
Pubmed	3177365	Hemoglobin constant spring defined by specific oligonucleotide hybridization and hemoglobin D Punjab (beta 121----Gln) in a Batak Indonesian family.	Pubmed	0.0044	65.1	0.0114	0.116	1	1	HBA2
Pubmed	18495081	Influence of the HLA-DQB1*0201 allele on the immune response in a Schistosoma mansoni infection.	GeneRIF	0.0044	65.1	0.0114	0.116	1	1	HLA-DQB1
Pubmed	11985705	Modeling alternative binding registers of a minimal immunogenic peptide on two class II major histocompatibility complex (MHC II) molecules predicts polarized T-cell receptor (TCR) contact positions.	GeneRIF	0.0044	65.1	0.0114	0.116	1	1	HLA-DQB1
Pubmed	8083346	Molecular cloning of hamster brain and atrial natriuretic peptide cDNAs. Cardiomyopathic hamsters are useful models for brain and atrial natriuretic peptides.	Pubmed	0.0044	65.1	0.0114	0.116	1	1	NPPA
Pubmed	22326585	DQB1*06:02 allele-specific expression varies by allelic dosage, not narcolepsy status.	GeneRIF	0.0044	65.1	0.0114	0.116	1	1	HLA-DQB1
Pubmed	17370149	Association of HLA-DQB1 gene polymorphisms with outcomes of HBV infection in Chinese Han population.	GeneRIF	0.0044	65.1	0.0114	0.116	1	1	HLA-DQB1
Pubmed	9738450	An intronic promoter controls the expression of truncated human gamma-glutamyltransferase mRNAs.	Pubmed	0.0044	65.1	0.0114	0.116	1	1	GGT1
Pubmed	18678771	Smoothelin-B deficiency results in reduced arterial contractility, hypertension, and cardiac hypertrophy in mice.	GeneRIF	0.0044	65.1	0.0114	0.116	1	1	SMTN
Pubmed	18096051	Improved functional expression of recombinant human ether-a-go-go (hERG) K+ channels by cultivation at reduced temperature.	GeneRIF	0.0044	65.1	0.0114	0.116	1	1	KCNH2
Pubmed	12626667	Modulation of human ether-à-go-go-related K+ (HERG) channel inactivation by Cs+ and K+.	GeneRIF	0.0044	65.1	0.0114	0.116	1	1	KCNH2
Pubmed	15154080	Expression of a novel pyridoxal kinase mRNA splice variant, PKH-T, in human testis.	Pubmed	0.0044	65.1	0.0114	0.116	1	1	PDXK
Pubmed	19590037	Peroxidasin is secreted and incorporated into the extracellular matrix of myofibroblasts and fibrotic kidney.	GeneRIF	0.0044	65.1	0.0114	0.116	1	1	PXDN
Pubmed	12055244	Characterization of mouse and human B7-H3 genes.	GeneRIF	0.0044	65.1	0.0114	0.116	1	1	CD276
Pubmed	20625912	GGT levels in type II diabetic patients with acute coronary syndrome (does diabetes have any effect on GGT levels in acute coronary syndrome?).	GeneRIF	0.0044	65.1	0.0114	0.116	1	1	GGT1
Pubmed	23492185	Targeted inhibition of KCa3.1 channel attenuates airway inflammation and remodeling in allergic asthma.	GeneRIF	0.0044	65.1	0.0114	0.116	1	1	KCNN4

Category	ID	Name	Source	p-value	q-value Bonferroni	q-value FDR B&H	q-value FDR B&Y	Hit Count in Query List	Hit Count in Genome	Hit in Query List
Pubmed	21967524	In vitro characterization of the a-thalassemia point mutation HBA2:c.95+1G>A [IVS-I-1(G>A) (a2)].	GeneRIF	0.0044	65.1	0.0114	0.116	1	1	HBA2
Pubmed	20484958	Pooling-based genome-wide association study implicates gamma-glutamyltransferase 1 (GGT1) gene in pancreatic carcinogenesis.	GeneRIF	0.0044	65.1	0.0114	0.116	1	1	GGT1
Pubmed	1511740	The gene structure of tetranectin, a plasminogen binding protein.	Pubmed	0.0044	65.1	0.0114	0.116	1	1	CLEC3B
Pubmed	22281000	Birth order and narcolepsy risk among genetically susceptible individuals: a population-based case-control study.	GeneRIF	0.0044	65.1	0.0114	0.116	1	1	HLA-DQB1
Pubmed	21377455	The alarm secretory leukocyte protease inhibitor increases with progressive metabolic dysfunction.	GeneRIF	0.0044	65.1	0.0114	0.116	1	1	SLPI
Pubmed	18477484	Differential rate constants of racemization of aspartyl and asparaginyl residues in human alpha A-crystallin mutants.	GeneRIF	0.0044	65.1	0.0114	0.116	1	1	CRYAA
Pubmed	18683147	[The -384A >C polymorphism of endothelial lipase gene promoter region in Chinese healthy normolipidemic and endogenous hypertriglyceridemic subjects].	GeneRIF	0.0044	65.1	0.0114	0.116	1	1	LIPG
Pubmed	19608871	Matrilysin-1 mediates bronchiolization of alveoli, a potential premalignant change in lung cancer.	GeneRIF	0.0044	65.1	0.0114	0.116	1	1	MMP7
Pubmed	2850481	A constitutive promoter directs expression of the nerve growth factor receptor gene.	Pubmed	0.0044	65.1	0.0114	0.116	1	1	NGFR
Pubmed	21628128	Corneodesmosomes and corneodesmosin: from the stratum corneum cohesion to the pathophysiology of genodermatoses.	GeneRIF	0.0044	65.1	0.0114	0.116	1	1	CDSN
Pubmed	21503571	The level of secretory leukocyte protease inhibitor is decreased in metastatic head and neck squamous cell carcinoma.	GeneRIF	0.0044	65.1	0.0114	0.116	1	1	SLPI
Pubmed	17486499	A moderately severe alpha-thalassemia condition resulting from a combination of the alpha2 polyadenylation signal (AATAAA-->AATA- -) mutation and a 3.7 Kb alpha gene deletion in an Australian family.	GeneRIF	0.0044	65.1	0.0114	0.116	1	1	HBA2
Pubmed	10908614	Induction of postnatal schwann cell death by the low-affinity neurotrophin receptor in vitro and afte axotomy.	Pubmed	0.0044	65.1	0.0114	0.116	1	1	NGFR
Pubmed	21920426	Inhibitory effects of a bisbenzylisoquinline alkaloid dauricine on HERG potassium channels.	GeneRIF	0.0044	65.1	0.0114	0.116	1	1	KCNH2
Pubmed	12353077	Mice with a severe deficiency of the endothelial protein C receptor gene develop, survive, and reproduce normally, and do not present with enhanced arterial thrombosis after challenge.	GeneRIF	0.0044	65.1	0.0114	0.116	1	1	PROCR
Pubmed	19198021	Dynamic measurements of serum inhibin B and estradiol: a predictive evaluation of ovarian response to gonadotrophin stimulation in the early stage of IVF treatment.	GeneRIF	0.0044	65.1	0.0114	0.116	1	1	INHBB
Pubmed	12381379	Characterization and purification of adenosine deaminase 1 from human and chicken liver.	GeneRIF	0.0044	65.1	0.0114	0.116	1	1	ADA
Pubmed	22267596	Chromosome 20p11 gains are associated with liver specific metastasis in patients with colorectal cancer.	GeneRIF	0.0044	65.1	0.0114	0.116	1	1	APMAP
Pubmed	25698761	Spiral ganglion degeneration and hearing loss as a consequence of satellite cell death in saposin B-deficient mice.	Pubmed	0.0044	65.1	0.0114	0.116	1	1	PSAP
Pubmed	25719829	hERG1 channels drive tumour malignancy and may serve as prognostic factor in pancreatic ductal adenocarcinoma.	Pubmed	0.0044	65.1	0.0114	0.116	1	1	KCNH2
Pubmed	18347987	Tumor-associated protein SPIK/TATI suppresses serine protease dependent cell apoptosis.	GeneRIF	0.0044	65.1	0.0114	0.116	1	1	SPINK1
Pubmed	24421389	Downregulation of STRA6 in adipocytes and adipose stromovascular fraction in obesity and effects of adipocyte-specific STRA6 knockdown in vivo.	Pubmed	0.0044	65.1	0.0114	0.116	1	1	STRA6
Pubmed	21957202	Nascent HDL formation by hepatocytes is reduced by the concerted action of serum amyloid A and endothelial lipase.	GeneRIF	0.0044	65.1	0.0114	0.116	1	1	LIPG
Pubmed	17339197	A 65 bp duplication/insertion in exon II of the beta globin gene causing beta0-thalassemia.	GeneRIF	0.0044	65.1	0.0114	0.116	1	1	HBB
Pubmed	11186265	Hb Inkster [alpha85(F6)Asp-->Val] found in a caucasian male with polycythemia.	Pubmed	0.0044	65.1	0.0114	0.116	1	1	HBA2
Pubmed	1454804	Binding and transport of gangliosides by prosaposin.	Pubmed	0.0044	65.1	0.0114	0.116	1	1	PSAP
Pubmed	7515550	Localisation of secretory leucocyte proteinase inhibitor mRNA in nasal mucosa.	Pubmed	0.0044	65.1	0.0114	0.116	1	1	SLPI
Pubmed	2568315	Human gamma-glutamyl transpeptidase cDNA: comparison of hepatoma and kidney mRNA in the human and rat.	Pubmed	0.0044	65.1	0.0114	0.116	1	1	GGT1
Pubmed	24461013	Quantitation of the calcium and membrane binding properties of the C2 domains of dysferlin.	Pubmed	0.0044	65.1	0.0114	0.116	1	1	DYSF
Pubmed	15534371	Number of T reg cells that differentiate does not increase upon encounter of agonist ligand on thymic epithelial cells.	Pubmed	0.0044	65.1	0.0114	0.116	1	1	CD74
Pubmed	16335784	A potential role for the endothelin ETA receptor in salt-sensitive hypertension of the proANP gene-disrupted mouse.	GeneRIF	0.0044	65.1	0.0114	0.116	1	1	NPPA
Pubmed	24613362	Baseline gamma-glutamyl transferase levels strongly correlate with hepatocellular carcinoma development in non-cirrhotic patients with successful hepatitis C virus eradication.	Pubmed	0.0044	65.1	0.0114	0.116	1	1	GGT1
Pubmed	17292962	HtrA1 is a novel mast cell serine protease of mice and men.	GeneRIF	0.0044	65.1	0.0114	0.116	1	1	HTRA1
Pubmed	16765896	A structural and dynamic characterization of the EF-hand protein CLSP.	GeneRIF	0.0044	65.1	0.0114	0.116	1	1	CALML5
Pubmed	24056069	HIV-1 seroreversion in HIV-1-infected children: do genetic determinants play a role?	Pubmed	0.0044	65.1	0.0114	0.116	1	1	HLA-DQB1
Pubmed	17653040	Two novel mutations identified in two Chinese gelatinous drop-like corneal dystrophy families.	GeneRIF	0.0044	65.1	0.0114	0.116	1	1	TACSTD2
Pubmed	16529770	Solution structure of the RBCC/TRIM B-box1 domain of human MID1: B-box with a RING.	Pubmed	0.0044	65.1	0.0114	0.116	1	1	MID1
Pubmed	20348920	p75NTR-dependent, myelin-mediated axonal degeneration regulates neural connectivity in the adult brain.	GeneRIF	0.0044	65.1	0.0114	0.116	1	1	NGFR

Category	ID	Name	Source	p-value	q-value Bonferroni	q-value FDR B&H	q-value FDR B&Y	Hit Count in Query List	Hit Count in Genome	Hit in Query List
Pubmed	8977190	In vivo functions mediated by the p41 isoform of the MHC class II-associated invariant chain.	Pubmed	0.0044	65.1	0.0114	0.116	1	1	CD74
Pubmed	12437997	PRELP, collagen, and a theory of Hutchinson-Gilford progeria.	Pubmed	0.0044	65.1	0.0114	0.116	1	1	PRELP
Pubmed	25405919	Non-thalassemic phenotype associated with the -83 (G > A) mutation of the ß-globin gene promoter (HBB: c.-133G > A).	Pubmed	0.0044	65.1	0.0114	0.116	1	1	HBB
Pubmed	22692452	CX3CL1 expression in the conjunctiva is involved in immune cell trafficking during toxic ocular surface inflammation.	GeneRIF	0.0044	65.1	0.0114	0.116	1	1	CX3CL1
Pubmed	20450932	Crystal structure of human transglutaminase 2 in complex with adenosine triphosphate.	GeneRIF	0.0044	65.1	0.0114	0.116	1	1	TGM2
Pubmed	12392851	Modulation of insulitis and type 1 diabetes by transgenic HLA-DR3 and DQ8 in NOD mice lacking endogenous MHC class II.	Pubmed	0.0044	65.1	0.0114	0.116	1	1	HLA-DQB1
Pubmed	11304832	No association of two missense variations of the benzodiazepine receptor (peripheral) gene and mood disorders in a Japanese sample.	GeneRIF	0.0044	65.1	0.0114	0.116	1	1	TSPO
Pubmed	6688808	Molecular cloning of human adenosine deaminase gene sequences.	Pubmed	0.0044	65.1	0.0114	0.116	1	1	ADA
Pubmed	12454462	Structure of mutant human carbonmonoxyhemoglobin C (betaE6K) at 2.0 A resolution.	Pubmed	0.0044	65.1	0.0114	0.116	1	1	HBB
Pubmed	9470901	Expression of corneodesmosin in the granular laye and stratum corneum of normal and diseased epidermis.	Pubmed	0.0044	65.1	0.0114	0.116	1	1	CDSN
Pubmed	22364871	Cellular functions of tissue transglutaminase.	GeneRIF	0.0044	65.1	0.0114	0.116	1	1	TGM2
Pubmed	17094466	Analysis of HtrA1 serine protease expression in human lung cancer.	GeneRIF	0.0044	65.1	0.0114	0.116	1	1	HTRA1
Pubmed	22436018	Inhibition of SLPI ameliorates disease activity in experimental autoimmune encephalomyelitis.	GeneRIF	0.0044	65.1	0.0114	0.116	1	1	SLPI
Pubmed	12915603	Prognostic value of the human kallikrein gene 15 expression in ovarian cancer.	GeneRIF	0.0044	65.1	0.0114	0.116	1	1	KLK15
Pubmed	15737607	Carboxy-monopeptidase substrate specificity of human cathepsin X.	GeneRIF	0.0044	65.1	0.0114	0.116	1	1	CTSK
Pubmed	25415613	KCa3.1: a new player in progressive kidney disease.	Pubmed	0.0044	65.1	0.0114	0.116	1	1	KCNN4
Pubmed	21943334	The interaction of the second Kunitz-type domain (KD2) of TFPI-2 with a novel interaction partner, prosaposin, mediates the inhibition of the invasion and migration of human fibrosarcoma cells.	GeneRIF	0.0044	65.1	0.0114	0.116	1	1	PSAP
Pubmed	4561179	Isolation and partial characterization of the cyanogen bromide fragments of 1 -acid glycoprotein and the elucidation of the amino acid sequence of the carboxyl-terminal cyanogen bromide fragment.	Pubmed	0.0044	65.1	0.0114	0.116	1	1	ORM1
Pubmed	10400245	Differential loss of spinal sensory but not motor neurons in the p75NTR knockout mouse.	Pubmed	0.0044	65.1	0.0114	0.116	1	1	NGFR
Pubmed	17698237	Hepatic fat content-independent association of the serum level of gamma-glutamyltransferase with visceral adiposity, but not subcutaneous adiposity.	GeneRIF	0.0044	65.1	0.0114	0.116	1	1	GGT1
Pubmed	21739120	Association of tumor-associated trypsin inhibitor (TATI) expression with molecular markers, pathologic features and clinical outcomes of urothelial carcinoma of the urinary bladder.	GeneRIF	0.0044	65.1	0.0114	0.116	1	1	SPINK1
Pubmed	20529852	Liganded thyroid hormone receptor-alpha enhances proliferation of pancreatic beta-cells.	GeneRIF	0.0044	65.1	0.0114	0.116	1	1	THRA
Pubmed	19432809	CX3CL1/fractalkine shedding by human hepatic stellate cells: contribution to chronic inflammation in the liver.	GeneRIF	0.0044	65.1	0.0114	0.116	1	1	CX3CL1
Pubmed	17210673	Novel pycnodysostosis mouse model uncovers cathepsin K function as a potential regulator of osteoclast apoptosis and senescence.	GeneRIF	0.0044	65.1	0.0114	0.116	1	1	CTSK
Pubmed	19011095	Ablation of smooth muscle myosin heavy chain SM2 increases smooth muscle contraction and results in postnatal death in mice.	Pubmed	0.0044	65.1	0.0114	0.116	1	1	MYH11
Pubmed	17705262	Intranuclear rod myopathy: molecular pathogenesis and mechanisms of weakness.	GeneRIF	0.0044	65.1	0.0114	0.116	1	1	ACTA1
Pubmed	17699163	A novel role of the interferon-inducible protein IFI16 as inducer of proinflammatory molecules in endothelial cells.	GeneRIF	0.0044	65.1	0.0114	0.116	1	1	IFI16
Pubmed	21376840	Functional characterization of the LQT2-causing mutation R582C and the associated voltage-dependent fluorescence signal.	GeneRIF	0.0044	65.1	0.0114	0.116	1	1	KCNH2
Pubmed	18441445	Mutation site dependent variability of cardiac events in Japanese LQT2 form of congenital long-QT syndrome.	GeneRIF	0.0044	65.1	0.0114	0.116	1	1	KCNH2
Pubmed	22345152	Tumor cell-derived angiopoietin-like protein ANGPTL2 is a critical driver of metastasis.	GeneRIF	0.0044	65.1	0.0114	0.116	1	1	ANGPTL2
Pubmed	9861009	Virus-specific CD8(+) T cell numbers are maintained during gamma-herpesvirus reactivation in CD4-deficient mice.	Pubmed	0.0044	65.1	0.0114	0.116	1	1	HLA-DQB1
Pubmed	16979579	Tissue transglutaminase contributes to the formation of disulphide bridges in proteins of mitochondrial respiratory complexes.	GeneRIF	0.0044	65.1	0.0114	0.116	1	1	TGM2
Pubmed	22225906	Evidence that GTP-binding domain but not catalytic domain of transglutaminase 2 is essential for epithelial-to-mesenchymal transition in mammary epithelial cells.	GeneRIF	0.0044	65.1	0.0114	0.116	1	1	TGM2
Pubmed	9027587	Peripheral benzodiazepine receptors in isolated human pancreatic islets.	Pubmed	0.0044	65.1	0.0114	0.116	1	1	TSPO
Pubmed	6495650	ERV3, a full-length human endogenous provirus: chromosomal localization and evolutionary relationships.	Pubmed	0.0044	65.1	0.0114	0.116	1	1	ERV3-1
Pubmed	12821936	Neurotrophin receptor p75(NTR) characterizes human esophageal keratinocyte stem cells in vitro.	GeneRIF	0.0044	65.1	0.0114	0.116	1	1	NGFR
Pubmed	2271613	Substrates of cGMP kinase in vascular smooth muscle and their role in the relaxation process.	Pubmed	0.0044	65.1	0.0114	0.116	1	1	MRVI1
Pubmed	21525004	Progesterone impairs human ether-a-go-go-related gene (HERG) trafficking by disruption of intracellular cholesterol homeostasis.	GeneRIF	0.0044	65.1	0.0114	0.116	1	1	KCNH2
Pubmed	15294965	Murine B7-H3 is a negative regulator of T cells.	GeneRIF	0.0044	65.1	0.0114	0.116	1	1	CD276
Pubmed	10022432	Thyroid receptor alpha1 and alpha2 mutations in nonfunctioning pituitary tumors.	Pubmed	0.0044	65.1	0.0114	0.116	1	1	THRA

Category	ID	Name	Source	p-value	q-value Bonferroni	q-value FDR B&H	q-value FDR B&Y	Hit Count in Query List	Hit Count in Genome	Hit in Query List
Pubmed	8436293	Transgenic mice as a model to study the role of TGF-beta-related molecules in hair follicles.	Pubmed	0.0044	65.1	0.0114	0.116	1	1	BMP4
Pubmed	8641138	Assignment of the human prosaposin gene (PSAP) to 10q22.1 by fluorescence in situ hybridization. Giraffidae, okapi (Okapiajohnstoni), and giraffe (Giraffa camelopardalis): evidence for ancestral telomeres at the okapi polymorphic rob (4;26) fusion site.	Pubmed	0.0044	65.1	0.0114	0.116	1	1	PSAP
Pubmed	18360914	MID1 mutations in patients with X-linked Opitz G/BBB syndrome.	GeneRIF	0.0044	65.1	0.0114	0.116	1	1	MID1
Pubmed	17116873	Two isoforms of tissue transglutaminase mediate opposing cellular fates.	GeneRIF	0.0044	65.1	0.0114	0.116	1	1	TGM2
Pubmed	16877395	The role of dermatopontin in the stromal organization of the cornea.	Pubmed	0.0044	65.1	0.0114	0.116	1	1	DPT
Pubmed	9835221	Nerve growth factor evokes hyperalgesia in mice lacking the low-affinity neurotrophin receptor p75.	Pubmed	0.0044	65.1	0.0114	0.116	1	1	NGFR
Pubmed	23576428	Transglutaminase 2 expression in acute myeloid leukemia: association with adhesion molecule expression and leukemic blast motility.	Pubmed	0.0044	65.1	0.0114	0.116	1	1	TGM2
Pubmed	1522245	Molecular cloning and preliminary characterization of a novel cytoplasmic antigen recognized by myasthenia gravis sera.	Pubmed	0.0044	65.1	0.0114	0.116	1	1	AKAP12
Pubmed	15701642	Cleavage of p75 neurotrophin receptor by alpha-secretase and gamma-secretase requires specific receptor domains.	GeneRIF	0.0044	65.1	0.0114	0.116	1	1	NGFR
Pubmed	11215759	An association study between two missense variations of the benzodiazepine receptor (peripheral) gene and schizophrenia in a Japanese sample.	Pubmed	0.0044	65.1	0.0114	0.116	1	1	TSPO
Pubmed	22350792	Upregulation of bone morphogenetic protein 4 is associated with poor prognosis in patients with hepatocellular carcinoma.	GeneRIF	0.0044	65.1	0.0114	0.116	1	1	BMP4
Pubmed	17320163	The retinoblastoma binding protein RBP2 is an H3K4 demethylase.	GeneRIF	0.0044	65.1	0.0114	0.116	1	1	KDM5A
Pubmed	12364437	Natural history of beta-cell autoimmunity in young children with increased genetic susceptibility to type 1 diabetes recruited from the general population.	GeneRIF	0.0044	65.1	0.0114	0.116	1	1	HLA-DQB1
Pubmed	17597105	Aldo-keto reductase family 1 B10 gene silencing results in growth inhibition of colorectal cancer cells: Implication for cancer intervention.	GeneRIF	0.0044	65.1	0.0114	0.116	1	1	AKR1B10
Pubmed	19267908	Matrix metalloproteinase-7 facilitates immune access to the CNS in experimental autoimmune encephalomyelitis.	GeneRIF	0.0044	65.1	0.0114	0.116	1	1	MMP7
Pubmed	25043373	Gamma glutamyltransferase, alanine aminotransferase and risk of cancer: systematic review and meta-analysis.	Pubmed	0.0044	65.1	0.0114	0.116	1	1	GGT1
Pubmed	7736582	A mechanistic link between an inherited and an acquired cardiac arrhythmia: HERG encodes the IKr potassium channel.	Pubmed	0.0044	65.1	0.0114	0.116	1	1	KCNH2
Pubmed	24188875	Decreased inhibin B responses following recombinant human chorionic gonadotropin administration in normal women and women with polycystic ovary syndrome.	Pubmed	0.0044	65.1	0.0114	0.116	1	1	INHBB
Pubmed	15901484	Tetranectin in cerebrospinal fluid: biochemical characterisation and evidence of intrathecal synthesis or selective uptake into CSF.	Pubmed	0.0044	65.1	0.0114	0.116	1	1	CLEC3B
Pubmed	22631636	Expression and prognostic value of matrix metalloproteinase-7 in colorectal cancer.	GeneRIF	0.0044	65.1	0.0114	0.116	1	1	MMP7
Pubmed	10196186	Sphingolipid activator proteins are required for epidermal permeability barrier formation.	Pubmed	0.0044	65.1	0.0114	0.116	1	1	PSAP
Pubmed	3028473	Complete sequence and structure of the gene for human adenosine deaminase.	Pubmed	0.0044	65.1	0.0114	0.116	1	1	ADA
Pubmed	12615907	Increased susceptibility to LPS-induced endotoxin shock in secretory leukoprotease inhibitor (SLPI)-deficient mice.	GeneRIF	0.0044	65.1	0.0114	0.116	1	1	SLPI
Pubmed	21851971	Haplotypes of the endothelial protein C receptor gene and Behçet's disease.	GeneRIF	0.0044	65.1	0.0114	0.116	1	1	PROCR
Pubmed	19723636	Constitutive endocytosis of the chemokine CX3CL1 prevents its degradation by cell surface metalloproteases.	GeneRIF	0.0044	65.1	0.0114	0.116	1	1	CX3CL1
Pubmed	7816801	Expression of an active glycosylated human gamma-glutamyl transpeptidase mutant that lacks a membrane anchor domain.	Pubmed	0.0044	65.1	0.0114	0.116	1	1	GGT1
Pubmed	21050181	Role of p38 MAPK pathway in BMP4-mediated Smad-dependent premature senescence in lung cancer cells.	GeneRIF	0.0044	65.1	0.0114	0.116	1	1	BMP4
Pubmed	23356414	Microdeletion of exon 3 in the HBA2 gene associated with mild a-thalassemia trait.	GeneRIF	0.0044	65.1	0.0114	0.116	1	1	HBA2
Pubmed	19158083	BMP4 mediates oxidative stress-induced retinal pigment epithelial cell senescence and is overexpressed in age-related macular degeneration.	GeneRIF	0.0044	65.1	0.0114	0.116	1	1	BMP4
Pubmed	15584913	Developmental regulation of tissue transglutaminase in the mouse forebrain.	Pubmed	0.0044	65.1	0.0114	0.116	1	1	TGM2
Pubmed	19703146	Endogenous adenosine down-modulates mid-trimester intraamniotic tumor necrosis factor-alpha production.	GeneRIF	0.0044	65.1	0.0114	0.116	1	1	ADA
Pubmed	23000905	Reconsidering the diagnostic and prognostic utility of LN-2 for undifferentiated pleomorphic sarcoma and atypical fibroxanthoma.	GeneRIF	0.0044	65.1	0.0114	0.116	1	1	CD74
Pubmed	10036192	DCAMKL1, a brain-specific transmembrane protein on 13q12.3 that is similar to doublecortin (DCX).	Pubmed	0.0044	65.1	0.0114	0.116	1	1	DCLK1
Pubmed	17904186	HTRA1 variant increases risk to neovascular age-related macular degeneration in Chinese population.	GeneRIF	0.0044	65.1	0.0114	0.116	1	1	HTRA1
Pubmed	12687344	Association between polymorphisms of the atrial natriuretic peptide gene and proteinuria: a population-based study.	GeneRIF	0.0044	65.1	0.0114	0.116	1	1	NPPA
Pubmed	12601178	Endothelial lipase is a major genetic determinant for high-density lipoprotein concentration, structure, and metabolism.	GeneRIF	0.0044	65.1	0.0114	0.116	1	1	LIPG
Pubmed	22174694	Mining the LIPG allelic spectrum reveals the contribution of rare and common regulatory variants to HDL cholesterol.	GeneRIF	0.0044	65.1	0.0114	0.116	1	1	LIPG

Category	ID	Name	Source	p-value	q-value Bonferroni	q-value FDR B&H	q-value FDR B&Y	Hit Count in Query List	Hit Count in Genome	Hit in Query List
Pubmed	11731272	Characterization and expression of murine PRELP	GeneRIF	0.0044	65.1	0.0114	0.116	1	1	PRELP
Pubmed	3557128	Nucleotide sequence of mouse 5-aminolevulinic acid synthase cDNA and expression of its gene in hepatic and erythroid tissues.	Pubmed	0.0044	65.1	0.0114	0.116	1	1	ALAS2
Pubmed	25043946	Midline 1 directs lytic granule exocytosis and cytotoxicity of mouse killer T cells.	Pubmed	0.0044	65.1	0.0114	0.116	1	1	MID1
Pubmed	2825202	Nucleotide sequence of cloned cDNA for human sphingolipid activator protein 1 precursor.	Pubmed	0.0044	65.1	0.0114	0.116	1	1	PSAP
Pubmed	24023860	Polymorphisms in the MASP1 gene are associated with serum levels of MASP-1, MASP-3, and MAp44.	Pubmed	0.0044	65.1	0.0114	0.116	1	1	MASP1
Pubmed	20649959	Over-expression of adenosine deaminase in mouse podocytes does not reverse puromycin aminonucleoside resistance.	GeneRIF	0.0044	65.1	0.0114	0.116	1	1	ADA
Pubmed	14688321	Fully functional HLA B27-restricted CD4+ as well as CD8+ T cell responses in TCR transgenic mice.	Pubmed	0.0044	65.1	0.0114	0.116	1	1	HLA-DQB1
Pubmed	15072110	Actin-related myopathy without any missense mutation in the ACTA1 gene.	GeneRIF	0.0044	65.1	0.0114	0.116	1	1	ACTA1
Pubmed	16920206	The Rab11-FIP1/RCP gene codes for multiple protein transcripts related to the plasma membrane recycling system.	GeneRIF	0.0044	65.1	0.0114	0.116	1	1	RAB11FIP1
Pubmed	23350698	A small peptide mimetic of brain-derived neurotrophic factor promotes peripheral myelination.	Pubmed	0.0044	65.1	0.0114	0.116	1	1	NGFR
Pubmed	12670744	Diminished renal kallikrein responses to mineralocorticoid stimulation in African Americans: determinants of an intermediate phenotype for hypertension.	GeneRIF	0.0044	65.1	0.0114	0.116	1	1	KLK1
Pubmed	12514664	Association of the ScaI atrial natriuretic peptide gene polymorphism with nonfatal myocardial infarction and extent of coronary artery disease.	GeneRIF	0.0044	65.1	0.0114	0.116	1	1	NPPA
Pubmed	24616641	Frequency of CFTR, SPINK1, and cathepsin B gene mutation in North Indian population: connections between genetics and clinical data.	Pubmed	0.0044	65.1	0.0114	0.116	1	1	SPINK1
Pubmed	11950815	The N34S mutation of SPINK1 (PSTI) is associated with a familial pattern of idiopathic chronic pancreatitis but does not cause the disease.	GeneRIF	0.0044	65.1	0.0114	0.116	1	1	SPINK1
Pubmed	9354679	The plasmacytoma resistance gene, Pctr2, delays the onset of tumorigenesis and resides in the telomeric region of chromosome 4.	Pubmed	0.0044	65.1	0.0114	0.116	1	1	NPPA
Pubmed	1324502	Immunocytochemical localization of nerve growth factor receptor (NGFR) in human teeth.	Pubmed	0.0044	65.1	0.0114	0.116	1	1	NGFR
Pubmed	22318734	Proteasomal inhibition restores biological function of mis-sense mutated dysferlin in patient-derived muscle cells.	GeneRIF	0.0044	65.1	0.0114	0.116	1	1	DYSF
Pubmed	17525068	Effect of laparoscopic ovarian diathermy on circulating inhibin B in women with anovulatory polycystic ovary syndrome.	GeneRIF	0.0044	65.1	0.0114	0.116	1	1	INHBB
Pubmed	23952145	Acute chest syndrome is associated with single nucleotide polymorphism-defined beta globin cluster haplotype in children with sickle cell anaemia.	Pubmed	0.0044	65.1	0.0114	0.116	1	1	HBB
Pubmed	24283209	Chronic psychological stress activates BMP4-dependent extramedullary erythropoiesis.	Pubmed	0.0044	65.1	0.0114	0.116	1	1	BMP4
Pubmed	8592062	Elevated levels of the endogenous retrovirus ERV3 in human sebaceous glands.	Pubmed	0.0044	65.1	0.0114	0.116	1	1	ERV3-1
Pubmed	10790215	Identification of seven novel SNPS (five nucleotide and two amino acid substitutions) in the connexin31 (GJB3) gene.	Pubmed	0.0044	65.1	0.0114	0.116	1	1	GJB3
Pubmed	7499338	The mouse bone morphogenetic protein-4 gene. Analysis of promoter utilization in fetal rat calvarial osteoblasts and regulation by COUP-TFI orphan receptor.	Pubmed	0.0044	65.1	0.0114	0.116	1	1	BMP4
Pubmed	24498105	Humanized HLA-DR4 mice fed with the protozoan pathogen of oysters Perkinsus marinus (Dermo) do not develop noticeable pathology but elicit systemic immunity.	Pubmed	0.0044	65.1	0.0114	0.116	1	1	HLA-DQB1
Pubmed	8276405	Smooth muscle myosin heavy chain locus (MYH11) maps to 16p13.13-p13.12 and establishes a new region of conserved synteny between human 16p and mouse 16.	Pubmed	0.0044	65.1	0.0114	0.116	1	1	MYH11
Pubmed	15737197	Deficient contact hypersensitivity reaction in CD4-/- mice is because of impaired hapten-specific CD8+ T cell functions.	Pubmed	0.0044	65.1	0.0114	0.116	1	1	HLA-DQB1
Pubmed	18374455	Abeta(1-42) stimulates adult SVZ neurogenesis through the p75 neurotrophin receptor.	GeneRIF	0.0044	65.1	0.0114	0.116	1	1	NGFR
Pubmed	18381937	Tissue transglutaminase regulates focal adhesion kinase/AKT activation by modulating PTEN expression in pancreatic cancer cells.	GeneRIF	0.0044	65.1	0.0114	0.116	1	1	TGM2
Pubmed	7647473	Assignment of the gene encoding centrosome-associated protein CCD41 to mouse chromosome 2H.	Pubmed	0.0044	65.1	0.0114	0.116	1	1	PROCR
Pubmed	11939520	Unstable Hb Perth in a Taiwanese subject: a T-->C substitution at codon 32 of the beta-globin gene creates an MspI site.	Pubmed	0.0044	65.1	0.0114	0.116	1	1	HBB
Pubmed	18182448	Severe combined adrenal and gonadal deficiency caused by novel mutations in the cholesterol side chain cleavage enzyme, P450scc.	GeneRIF	0.0044	65.1	0.0114	0.116	1	1	CYP11A1
Pubmed	18188587	Facilitation of Ca2+-activated K+ channels (IKCa1) by mibefradil in B lymphocytes.	GeneRIF	0.0044	65.1	0.0114	0.116	1	1	KCNN4
Pubmed	21320609	Age and disease related changes in the translocator protein (TSPO) system in the human brain: positron emission tomography measurements with [11C]vinpocetine.	GeneRIF	0.0044	65.1	0.0114	0.116	1	1	TSPO
Pubmed	17718429	[Expression and clinical significance of matrilysin (MMP-7) in human rectal cancer].	GeneRIF	0.0044	65.1	0.0114	0.116	1	1	MMP7
Pubmed	16410462	Leukocyte transglutaminase 2 expression limits atherosclerotic lesion size.	GeneRIF	0.0044	65.1	0.0114	0.116	1	1	TGM2
Pubmed	25742143	The interferon-inducible DNA-sensor protein IFI16: a key player in the antiviral response.	Pubmed	0.0044	65.1	0.0114	0.116	1	1	IFI16

Category	ID	Name	Source	p-value	q-value Bonferroni	q-value FDR B&H	q-value FDR B&Y	Hit Count in Query List	Hit Count in Genome	Hit in Query List
Pubmed	15191543	Genetic association between an AACC insertion in the 3'UTR of the stratum corneum chymotryptic enzyme gene and atopic dermatitis.	GeneRIF	0.0044	65.1	0.0114	0.116	1	1	KLK7
Pubmed	19789311	Translocator protein blockade reduces prostate tumor growth.	GeneRIF	0.0044	65.1	0.0114	0.116	1	1	TSPO
Pubmed	15811552	Expression profiling with progression of dystrophic change in dysferlin-deficient mice (SJL).	Pubmed	0.0044	65.1	0.0114	0.116	1	1	DYSF
Pubmed	8104875	Complementary DNA sequence and chromosomal mapping of a human proteoglycan-binding cell-adhesion protein (dermatopontin).	Pubmed	0.0044	65.1	0.0114	0.116	1	1	DPT
Pubmed	22170009	Association of 2238T>C polymorphism of the atrial natriuretic peptide gene with coronary artery disease in Afro-Caribbeans with type 2 diabetes.	GeneRIF	0.0044	65.1	0.0114	0.116	1	1	NPPA
Pubmed	12505872	Physiological concentration of atrial natriuretic peptide induces endothelial regeneration in vitro.	GeneRIF	0.0044	65.1	0.0114	0.116	1	1	NPPA
Pubmed	10720488	Function of murine adenosine deaminase in the gastrointestinal tract.	Pubmed	0.0044	65.1	0.0114	0.116	1	1	ADA
Pubmed	17228174	Involvement of transglutaminase-2 in pathological changes in renal disease.	GeneRIF	0.0044	65.1	0.0114	0.116	1	1	TGM2
Pubmed	9089072	Characterization and developmental expression of a testis-specific adenosine deaminase mRNA in the mouse.	Pubmed	0.0044	65.1	0.0114	0.116	1	1	ADA
Pubmed	12242026	Nitric oxide potently inhibits the rate-limiting enzymatic step in steroidogenesis.	GeneRIF	0.0044	65.1	0.0114	0.116	1	1	CYP11A1
Pubmed	17510309	p75 neurotrophin receptor inhibits invasion and metastasis of gastric cancer.	GeneRIF	0.0044	65.1	0.0114	0.116	1	1	NGFR
Pubmed	20809785	Nogo receptor deletion and multimodal exercise improve distinct aspects of recovery in cervical spinal cord injury.	GeneRIF	0.0044	65.1	0.0114	0.116	1	1	RTN4R
Pubmed	9013890	Molecular cloning of a novel mouse aspartic protease-like protein that is expressed abundantly in the kidney.	Pubmed	0.0044	65.1	0.0114	0.116	1	1	NAPSA
Pubmed	14560310	Characterisation of the dysferlin skeletal muscle promoter.	Pubmed	0.0044	65.1	0.0114	0.116	1	1	DYSF
Pubmed	19931348	Regulated expression of endothelial lipase in atherosclerosis.	GeneRIF	0.0044	65.1	0.0114	0.116	1	1	LIPG
Pubmed	15531371	Developmental expression and function of Bmp4 in spermatogenesis and in maintaining epididymal integrity.	GeneRIF	0.0044	65.1	0.0114	0.116	1	1	BMP4
Pubmed	10486571	Nerve growth factor (NGF) exerts its pro-apoptotic effect via the P75NTR receptor in a cell cycle-dependent manner.	Pubmed	0.0044	65.1	0.0114	0.116	1	1	NGFR
Pubmed	18202319	Transglutaminase 2 is central to induction of the arterial calcification program by smooth muscle cells.	GeneRIF	0.0044	65.1	0.0114	0.116	1	1	TGM2
Pubmed	19065538	[Identification of a novel KCNH2 mutation in a family with congenital long QT syndrome and prediction of the secondary structure of its encoding protein].	GeneRIF	0.0044	65.1	0.0114	0.116	1	1	KCNH2
Pubmed	23250777	Kallikrein-related peptidase 10 expression in salivary gland tissues and tumours.	GeneRIF	0.0044	65.1	0.0114	0.116	1	1	KLK10
Pubmed	2391366	Invariant chain trimers are sequestered in the rough endoplasmic reticulum in the absence of association with HLA class II antigens.	Pubmed	0.0044	65.1	0.0114	0.116	1	1	CD74
Pubmed	23632887	ASB2a regulates migration of immature dendritic cells.	Pubmed	0.0044	65.1	0.0114	0.116	1	1	ASB2
Pubmed	8814310	Mouse cathepsin K: cDNA cloning and predominant expression of the gene in osteoclasts, and in some hypertrophying chondrocytes during mouse development.	Pubmed	0.0044	65.1	0.0114	0.116	1	1	CTSK
Pubmed	16767549	Fractalkine in rheumatoid arthritis and allied conditions.	GeneRIF	0.0044	65.1	0.0114	0.116	1	1	CX3CL1
Pubmed	18158327	A novel insertion pathway of mitochondrial outer membrane proteins with multiple transmembrane segments.	GeneRIF	0.0044	65.1	0.0114	0.116	1	1	TSPO
Pubmed	17151145	The Ca(2+)-activated K(+) channel KCa3.1 compartmentalizes in the immunological synapse of human T lymphocytes.	GeneRIF	0.0044	65.1	0.0114	0.116	1	1	KCNN4
Pubmed	20731658	Appearance of tissue transglutaminase in astrocytes in multiple sclerosis lesions: a role in cell adhesion and migration?	GeneRIF	0.0044	65.1	0.0114	0.116	1	1	TGM2
Pubmed	11416150	Genetic analysis reveals different functions for the products of the thyroid hormone receptor alpha locus.	Pubmed	0.0044	65.1	0.0114	0.116	1	1	THRA
Pubmed	23242015	B7-H3 overexpression in pancreatic cancer promotes tumor progression.	Pubmed	0.0044	65.1	0.0114	0.116	1	1	CD276
Pubmed	19672789	alphaPix interacts with Helicobacter pylori CagA to induce IL-8 expression in gastric epithelial cells.	GeneRIF	0.0044	65.1	0.0114	0.116	1	1	ARHGEF6
Pubmed	6234658	The structure of rat preproatrial natriuretic factor as defined by a complementary DNA clone.	Pubmed	0.0044	65.1	0.0114	0.116	1	1	NPPA
Pubmed	8589684	Three new adenosine deaminase mutations that define a splicing enhancer and cause severe and partial phenotypes: implications for evolution of a CpG hotspot and expression of a transduced ADA cDNA.	Pubmed	0.0044	65.1	0.0114	0.116	1	1	ADA
Pubmed	25227100	Selection of human p75NTR tag SNPs and its biological significance for clinical association studies.	Pubmed	0.0044	65.1	0.0114	0.116	1	1	NGFR
Pubmed	17975023	The microtubule-associated protein doublecortin-like regulates the transport of the glucocorticoid receptor in neuronal progenitor cells.	GeneRIF	0.0044	65.1	0.0114	0.116	1	1	DCLK1
Pubmed	67897	Complete 3' noncoding region sequences of rabbit and human beta-globin messenger RNAs.	Pubmed	0.0044	65.1	0.0114	0.116	1	1	HBB
Pubmed	9259319	Identification of transcripts initiated from an internal promoter in the c-erbA alpha locus that encode inhibitors of retinoic acid receptor-alpha and triiodothyronine receptor activities.	Pubmed	0.0044	65.1	0.0114	0.116	1	1	THRA
Pubmed	25070808	High frequency of DQB1*05 and absolute absence of DRB1*13 in muscle-specific tyrosine kinase positive myasthenia gravis.	Pubmed	0.0044	65.1	0.0114	0.116	1	1	HLA-DQB1
Pubmed	17610414	4IgB7-H3 is the major isoform expressed on immunocytes as well as malignant cells.	GeneRIF	0.0044	65.1	0.0114	0.116	1	1	CD276

Category	ID	Name	Source	p-value	q-value Bonferroni	q-value FDR B&H	q-value FDR B&Y	Hit Count in Query List	Hit Count in Genome	Hit in Query List
Pubmed	9537432	New member of aldose reductase family proteins overexpressed in human hepatocellular carcinoma.	Pubmed	0.0044	65.1	0.0114	0.116	1	1	AKR1B10
Pubmed	17971299	Down-regulation of surface fractalkine by RNA interference in B16 melanoma reduced tumor growth in mice.	GeneRIF	0.0044	65.1	0.0114	0.116	1	1	CX3CL1
Pubmed	23471968	Pore helices play a dynamic role as integrators of domain motion during Kv11.1 channel inactivation gating.	GeneRIF	0.0044	65.1	0.0114	0.116	1	1	KCNH2
Pubmed	17905306	Tracing changes in protonation: a prerequisite to factorize thermodynamic data of inhibitor binding to aldose reductase.	GeneRIF	0.0044	65.1	0.0114	0.116	1	1	AKR1B10
Pubmed	20515469	CDO1 promoter methylation is a biomarker for outcome prediction of anthracycline treated, estrogen receptor-positive, lymph node-positive breast cancer patients.	GeneRIF	0.0044	65.1	0.0114	0.116	1	1	CDO1
Pubmed	18931651	Active matrix metalloproteinase-7 is associated with invasion in buccal squamous cell carcinoma.	GeneRIF	0.0044	65.1	0.0114	0.116	1	1	MMP7
Pubmed	7553001	Reconstitution of invariant chain function in transgenic mice in vivo by individual p31 and p41 isoforms.	Pubmed	0.0044	65.1	0.0114	0.116	1	1	CD74
Pubmed	23967175	The 18-kDa translocator protein (TSPO) disrupts mammary epithelial morphogenesis and promotes breast cancer cell migration.	Pubmed	0.0044	65.1	0.0114	0.116	1	1	TSPO
Pubmed	14665693	Binding of Plasmodium falciparum-infected erythrocytes to the membrane-bound form of Fractalkine/CX3CL1.	GeneRIF	0.0044	65.1	0.0114	0.116	1	1	CX3CL1
Pubmed	21992849	Effect of low-dose human atrial natriuretic peptide on postoperative atrial fibrillation in patients undergoing pulmonary resection for lung cancer: a double-blind, placebo-controlled study.	GeneRIF	0.0044	65.1	0.0114	0.116	1	1	NPPA
Pubmed	11462236	Missense polymorphism in the human carboxypeptidase E gene alters enzymatic activity.	GeneRIF	0.0044	65.1	0.0114	0.116	1	1	CPE
Pubmed	8515813	Numerous candidate plasticity-related genes revealed by differential cDNA cloning.	Pubmed	0.0044	65.1	0.0114	0.116	1	1	DCLK1
Pubmed	22158048	The 14q22.2 colorectal cancer variant rs4444235 shows cis-acting regulation of BMP4.	GeneRIF	0.0044	65.1	0.0114	0.116	1	1	BMP4
Pubmed	3866610	Ia-associated invariant chain is fatty acylated before addition of sialic acid.	Pubmed	0.0044	65.1	0.0114	0.116	1	1	CD74
Pubmed	24623279	Re-trafficking of hERG reverses long QT syndrome 2 phenotype in human iPS-derived cardiomyocytes.	Pubmed	0.0044	65.1	0.0114	0.116	1	1	KCNH2
Pubmed	21182673	Detection of corneodesmosin on the surface of stratum corneum using atomic force microscopy.	GeneRIF	0.0044	65.1	0.0114	0.116	1	1	CDSN
Pubmed	24175906	Inhibition of transglutaminase 2, a novel target for pulmonary fibrosis, by two small electrophilic molecules.	Pubmed	0.0044	65.1	0.0114	0.116	1	1	TGM2
Pubmed	22394585	Altered responsiveness to TGF-ß results in reduced Papss2 expression and alterations in the biomechanical properties of mouse articular cartilage.	GeneRIF	0.0044	65.1	0.0114	0.116	1	1	PAPSS2
Pubmed	15015775	Neurotrophin effects on neuroblastoma cells: correlation with trk and p75NTR expression and influence of Trk receptor bodies.	GeneRIF	0.0044	65.1	0.0114	0.116	1	1	NGFR
Pubmed	19013100	Prevalence of narcolepsy in King County, Washington, USA.	GeneRIF	0.0044	65.1	0.0114	0.116	1	1	HLA-DQB1
Pubmed	11992124	A genomic screen for genes upregulated by demethylation and histone deacetylase inhibition in human colorectal cancer.	Pubmed	0.0044	65.1	0.0114	0.116	1	1	SEZ6L
Pubmed	12846902	Rapid, accurate genotyping of beta-thalassaemia mutations using a novel multiplex primer extension/denaturing high-performance liquid chromatography assay.	GeneRIF	0.0044	65.1	0.0114	0.116	1	1	HBB
Pubmed	25203114	NOX4 mediates BMP4-induced upregulation of TRPC1 and 6 protein expressions in distal pulmonary arterial smooth muscle cells.	Pubmed	0.0044	65.1	0.0114	0.116	1	1	BMP4
Pubmed	16150908	Thyroid hormones regulate fibroblast growth facto receptor signaling during chondrogenesis.	Pubmed	0.0044	65.1	0.0114	0.116	1	1	THRA
Pubmed	15249541	Comparison of HLA-DR1-restricted T cell response induced in HLA-DR1 transgenic mice deficient for murine MHC class II and HLA-DR1 transgenic mice expressing endogenous murine MHC class II molecules.	Pubmed	0.0044	65.1	0.0114	0.116	1	1	HLA-DQB1
Pubmed	16177035	Expression and functional phenotype of mouse ERG K+ channels in the inner ear: potential role in K+ regulation in the inner ear.	GeneRIF	0.0044	65.1	0.0114	0.116	1	1	KCNH2
Pubmed	19501051	hERG1a/1b heteromeric currents exhibit amplified attenuation of inactivation in variant 1 short QT syndrome.	GeneRIF	0.0044	65.1	0.0114	0.116	1	1	KCNH2
Pubmed	22253779	Impact of the genome wide supported NRGN gene on anterior cingulate morphology in schizophrenia	GeneRIF	0.0044	65.1	0.0114	0.116	1	1	NRGN
Pubmed	17577780	Gravin gene expression in acute leukaemias: clinical importance and review of the literature.	GeneRIF	0.0044	65.1	0.0114	0.116	1	1	AKAP12
Pubmed	18791274	Ligands of the mitochondrial 18 kDa translocator protein attenuate apoptosis of human glioblastoma cells exposed to erucylphosphohomocholine.	GeneRIF	0.0044	65.1	0.0114	0.116	1	1	TSPO
Pubmed	15452196	New insights on the voltage dependence of the KCa3.1 channel block by internal TBA.	GeneRIF	0.0044	65.1	0.0114	0.116	1	1	KCNN4
Pubmed	21112188	A neuropsychological investigation of the genome wide associated schizophrenia risk variant NRGN rs12807809.	GeneRIF	0.0044	65.1	0.0114	0.116	1	1	NRGN
Pubmed	22290289	Glycoprotein transmembrane nmb: an androgen-downregulated gene attenuates cell invasion and tumorigenesis in prostate carcinoma cells.	GeneRIF	0.0044	65.1	0.0114	0.116	1	1	GPNMB
Pubmed	20544921	Diaphragm displays early and progressive functional deficits in dysferlin-deficient mice.	GeneRIF	0.0044	65.1	0.0114	0.116	1	1	DYSF
Pubmed	24771064	TIKI2 suppresses growth of osteosarcoma by targeting Wnt/ß-catenin pathway.	Pubmed	0.0044	65.1	0.0114	0.116	1	1	TRABD2B
Pubmed	19479660	In vitro profiling against ion channels beyond hERG as an early indicator of cardiac risk.	GeneRIF	0.0044	65.1	0.0114	0.116	1	1	KCNH2

Category	ID	Name	Source	p-value	q-value Bonferroni	q-value FDR B&H	q-value FDR B&Y	Hit Count in Query List	Hit Count in Genome	Hit in Query List
Pubmed	21200088	[Association between polymorphism of rs3212855 and rs5515 of KLK1 gene with cerebral hemorrhage in Changsha Han population].	GeneRIF	0.0044	65.1	0.0114	0.116	1	1	KLK1
Pubmed	24324779	Enzyme-linked immunosorbent assay characterization of basal variation and heritability of systemic microfibrillar-associated protein 4.	Pubmed	0.0044	65.1	0.0114	0.116	1	1	MFAP4
Pubmed	10023778	Differentiating one smooth operator from another.	Pubmed	0.0044	65.1	0.0114	0.116	1	1	SMTN
Pubmed	24146918	Blocking KCa3.1 channels increases tumor cell killing by a subpopulation of human natural killer lymphocytes.	Pubmed	0.0044	65.1	0.0114	0.116	1	1	KCNN4
Pubmed	22103619	Serum BMP-4 levels in relation to arterial stiffness and carotid atherosclerosis in patients with Type 2 diabetes.	GeneRIF	0.0044	65.1	0.0114	0.116	1	1	BMP4
Pubmed	19934382	A stretch of 17 amino acids in the prosaposin C terminus is critical for its binding to sortilin and targeting to lysosomes.	GeneRIF	0.0044	65.1	0.0114	0.116	1	1	PSAP
Pubmed	11559852	Complete ablation of the neurotrophin receptor p75NTR causes defects both in the nervous and the vascular system.	Pubmed	0.0044	65.1	0.0114	0.116	1	1	NGFR
Pubmed	24958728	Chondroitin sulfate promotes activation of cathepsin K.	Pubmed	0.0044	65.1	0.0114	0.116	1	1	CTSK
Pubmed	24583226	TATI as a biomarker.	Pubmed	0.0044	65.1	0.0114	0.116	1	1	SPINK1
Pubmed	18272172	A splice site mutation in hERG leads to cryptic splicing in human long QT syndrome.	GeneRIF	0.0044	65.1	0.0114	0.116	1	1	KCNH2
Pubmed	8193381	Identification of a novel termination codon mutation (TAA-->TAT, Term-->Tyr) in the alpha 2 globin gene of a Laotian girl with hemoglobin H disease.	Pubmed	0.0044	65.1	0.0114	0.116	1	1	HBA2
Pubmed	16897606	No association between the genetic polymorphisms in the RTN4R gene and schizophrenia in the Chinese population.	GeneRIF	0.0044	65.1	0.0114	0.116	1	1	RTN4R
Pubmed	19690348	Characterization of Erg K+ channels in alpha- and beta-cells of mouse and human islets.	GeneRIF	0.0044	65.1	0.0114	0.116	1	1	KCNH2
Pubmed	25370943	Hepatocellular carcinomas promote tumor-associated macrophage M2-polarization via increased B7-H3 expression.	Pubmed	0.0044	65.1	0.0114	0.116	1	1	CD276
Pubmed	23010644	Fibroregulation of mesenchymal progenitor cells by BMP-4 after traumatic muscle injury.	GeneRIF	0.0044	65.1	0.0114	0.116	1	1	BMP4
Pubmed	23609438	The intermediate conductance calcium-activated potassium channel KCa3.1 regulates vascular smooth muscle cell proliferation via controlling calcium-dependent signaling.	GeneRIF	0.0044	65.1	0.0114	0.116	1	1	KCNN4
Pubmed	12460612	Increased p75(NTR) expression in hippocampal neurons containing hyperphosphorylated tau in Alzheimer patients.	GeneRIF	0.0044	65.1	0.0114	0.116	1	1	NGFR
Pubmed	17425679	Aldo-keto reductase family 1, member B10 in uterine carcinomas: a potential risk factor of recurrence after surgical therapy in cervical cancer.	GeneRIF	0.0044	65.1	0.0114	0.116	1	1	AKR1B10
Pubmed	23499583	Clinical significance of kallikrein-related peptidase (KLK10) mRNA expression in colorectal cancer.	Pubmed	0.0044	65.1	0.0114	0.116	1	1	KLK10
Pubmed	18717734	The p75 neurotrophin receptor regulates hippocampal neurogenesis and related behaviours.	GeneRIF	0.0044	65.1	0.0114	0.116	1	1	NGFR
Pubmed	7992842	Developmental and tissue-specific expression of prosaposin mRNA in murine tissues.	Pubmed	0.0044	65.1	0.0114	0.116	1	1	PSAP
Pubmed	3640338	Isolation and sequence of a human gene encoding a potent inhibitor of leukocyte proteases.	Pubmed	0.0044	65.1	0.0114	0.116	1	1	SLPI
Pubmed	24381307	Functional validation of a human CAPN5 exome variant by lentiviral transduction into mouse retina	Pubmed	0.0044	65.1	0.0114	0.116	1	1	CAPN5
Pubmed	12736144	Expression and function of KCNH2 (HERG) in the human jejunum.	GeneRIF	0.0044	65.1	0.0114	0.116	1	1	KCNH2
Pubmed	22573795	Expression of kallikrein-related peptidase 7 predicts poor prognosis in patients with unresectable pancreatic ductal adenocarcinoma.	GeneRIF	0.0044	65.1	0.0114	0.116	1	1	KLK7
Pubmed	17442697	Tailbud-derived mesenchyme promotes urinary tract segmentation via BMP4 signaling.	Pubmed	0.0044	65.1	0.0114	0.116	1	1	BMP4
Pubmed	24679481	[KCNH2 gene new mutation in a patient with prior diagnosis of epilepsy].	Pubmed	0.0044	65.1	0.0114	0.116	1	1	KCNH2
Pubmed	16227618	Dicer-dependent turnover of intergenic transcripts from the human beta-globin gene cluster.	GeneRIF	0.0044	65.1	0.0114	0.116	1	1	HBB
Pubmed	21918680	Re-expression of AKAP12 inhibits progression and metastasis potential of colorectal carcinoma in vivo and in vitro.	GeneRIF	0.0044	65.1	0.0114	0.116	1	1	AKAP12
Pubmed	24663349	FOXO4-knockdown suppresses oxidative stress-induced apoptosis of early pro-angiogenic cells and augments their neovascularization capacities in ischemic limbs.	Pubmed	0.0044	65.1	0.0114	0.116	1	1	FOXO4
Pubmed	19706538	Renal fibrosis is attenuated by targeted disruption of KCa3.1 potassium channels.	GeneRIF	0.0044	65.1	0.0114	0.116	1	1	KCNN4
Pubmed	20437185	Transglutaminase 2 silencing reduced the beta-amyloid-effects on the activation of human THP-1 cells.	GeneRIF	0.0044	65.1	0.0114	0.116	1	1	TGM2
Pubmed	17242183	FoxO4 regulates tumor necrosis factor alpha-directed smooth muscle cell migration by activating matrix metalloproteinase 9 gene transcription.	Pubmed	0.0044	65.1	0.0114	0.116	1	1	FOXO4
Pubmed	21034624	T allele at site 6007 of bone morphogenetic protein 4 gene increases genetic susceptibility to ossification of the posterior longitudinal ligament in male Chinese Han population.	GeneRIF	0.0044	65.1	0.0114	0.116	1	1	BMP4
Pubmed	10355635	Identification of SLPI (secretory leukocyte protease inhibitor) in human mast cells using immunohistochemistry and in situ hybridisation.	Pubmed	0.0044	65.1	0.0114	0.116	1	1	SLPI
Pubmed	17560524	Identification of prosaposin as a novel interaction partner for Rhox5.	GeneRIF	0.0044	65.1	0.0114	0.116	1	1	PSAP
Pubmed	14555966	The promoter of the mouse tissue transglutaminase gene directs tissue-specific, retinoid-regulated and apoptosis-linked expression.	Pubmed	0.0044	65.1	0.0114	0.116	1	1	TGM2

Category	ID	Name	Source	p-value	q-value Bonferroni	q-value FDR B&H	q-value FDR B&Y	Hit Count in Query List	Hit Count in Genome	Hit in Query List
Pubmed	25222042	Characterization of Hb Calvino (HBB: c.406G¿>¿A): a new silent ß-globin gene variant found in coexistence with a-thalassemia in a family of African origin.	Pubmed	0.0044	65.1	0.0114	0.116	1	1	HBB
Pubmed	14599844	Characterization of HLA DR2 and DQ8 transgenic mouse with a new engineered mouse class II deletion, which lacks all endogenous class II genes.	Pubmed	0.0044	65.1	0.0114	0.116	1	1	HLA-DQB1
Pubmed	16049332	The immunomodulatory proteins B7-DC, B7-H2, and B7-H3 are differentially expressed across gestation in the human placenta.	Pubmed	0.0044	65.1	0.0114	0.116	1	1	CD276
Pubmed	22458599	Pcp4l1 contains an auto-inhibitory element that prevents its IQ motif from binding to calmodulin.	Pubmed	0.0044	65.1	0.0114	0.116	1	1	PCP4
Pubmed	3060147	Human mucus proteinase inhibitor (human MPI). Human seminal inhibitor I (HUSI-I), antileukoprotease (ALP), secretory leukocyte protease inhibitor (SLPI).	Pubmed	0.0044	65.1	0.0114	0.116	1	1	SLPI
Pubmed	12183616	Neuroscience. It takes more than two to Nogo.	Pubmed	0.0044	65.1	0.0114	0.116	1	1	RTN4R
Pubmed	12683950	Inhibition of staurosporine-induced apoptosis of endothelial cells by activated protein C requires protease-activated receptor-1 and endothelial cell protein C receptor.	Pubmed	0.0044	65.1	0.0114	0.116	1	1	PROCR
Pubmed	18306167	Dysferlin-deficient muscular dystrophy features amyloidosis.	GeneRIF	0.0044	65.1	0.0114	0.116	1	1	DYSF
Pubmed	8642277	The cytoplasmic domain of CD4 promotes the development of CD4 lineage T cells.	Pubmed	0.0044	65.1	0.0114	0.116	1	1	HLA-DQB1
Pubmed	21621530	Severe impairment of cerebellum development in mice expressing a dominant-negative mutation inactivating thyroid hormone receptor alpha1 isoform.	GeneRIF	0.0044	65.1	0.0114	0.116	1	1	THRA
Pubmed	9757090	Structure of the C-type lectin carbohydrate recognition domain of human tetranectin.	Pubmed	0.0044	65.1	0.0114	0.116	1	1	CLEC3B
Pubmed	23041462	Purification and structural characterization of the voltage-sensor domain of the hERG potassium channel.	GeneRIF	0.0044	65.1	0.0114	0.116	1	1	KCNH2
Pubmed	2388839	cDNA encoding the human homologue of rat ribosomal protein L35a.	Pubmed	0.0044	65.1	0.0114	0.116	1	1	RPL35A
Pubmed	17698854	Membrane translocation of P-Rex1 is mediated by G protein betagamma subunits and phosphoinositide 3-kinase.	GeneRIF	0.0044	65.1	0.0114	0.116	1	1	PREX1
Pubmed	7600951	p75-deficient embryonic dorsal root sensory and neonatal sympathetic neurons display a decreased sensitivity to NGF.	Pubmed	0.0044	65.1	0.0114	0.116	1	1	NGFR
Pubmed	14649319	A new alpha2-globin gene [codon 90 (AAG --> TAG)] nonsense mutation.	GeneRIF	0.0044	65.1	0.0114	0.116	1	1	HBA2
Pubmed	7721091	Comparison of repetitive elements in the third intron of human and rodent mitochondrial benzodiazepine receptor-encoding genes.	Pubmed	0.0044	65.1	0.0114	0.116	1	1	TSPO
Pubmed	16505581	Heterogeneity in expression and subcellular localization of tight junction proteins, claudin-10 and -15, examined by RT-PCR and immunofluorescence microscopy.	Pubmed	0.0044	65.1	0.0114	0.116	1	1	CLDN15
Pubmed	19655169	The mechanism of transglutaminase 2 inhibition with glucosamine: implications of a possible anti-inflammatory effect through transglutaminase inhibition.	GeneRIF	0.0044	65.1	0.0114	0.116	1	1	TGM2
Pubmed	22349828	Upregulation of Trop-2 quantitatively stimulates human cancer growth.	GeneRIF	0.0044	65.1	0.0114	0.116	1	1	TACSTD2
Pubmed	16083722	Autophagic cell death of pancreatic acinar cells in serine protease inhibitor Kazal type 3-deficient mice.	Pubmed	0.0044	65.1	0.0114	0.116	1	1	SPINK1
Pubmed	16511838	Evidence that variation in the peripheral benzodiazepine receptor (PBR) gene influences susceptibility to panic disorder.	GeneRIF	0.0044	65.1	0.0114	0.116	1	1	TSPO
Pubmed	9693050	Human calcium-activated potassium channel gene KCNN4 maps to chromosome 19q13.2 in the region deleted in diamond-blackfan anemia.	Pubmed	0.0044	65.1	0.0114	0.116	1	1	KCNN4
Pubmed	23399492	Novel and recurrent PORCN gene mutations in almost unilateral and typical focal dermal hypoplasia patients.	Pubmed	0.0044	65.1	0.0114	0.116	1	1	PORCN
Pubmed	14675563	Tumour-associated trypsin inhibitor, carcinoembryonic antigen and acute-phase reactant proteins CRP and alpha1-antitrypsin in patients with gastrointestinal malignancies.	GeneRIF	0.0044	65.1	0.0114	0.116	1	1	SPINK1
Pubmed	20153760	A mutant thyroid hormone receptor alpha1 alters hippocampal circuitry and reduces seizure susceptibility in mice.	GeneRIF	0.0044	65.1	0.0114	0.116	1	1	THRA
Pubmed	10844062	Gelatino-lattice corneal dystrophy: clinical features and mutational analysis.	Pubmed	0.0044	65.1	0.0114	0.116	1	1	TACSTD2
Pubmed	21958735	Functional characterization of the human translocator protein (18kDa) gene promoter in human breast cancer cell lines.	GeneRIF	0.0044	65.1	0.0114	0.116	1	1	TSPO
Pubmed	15765151	Arterial and renal consequences of partial genetic deficiency in tissue kallikrein activity in humans.	GeneRIF	0.0044	65.1	0.0114	0.116	1	1	KLK1
Pubmed	20557680	Reverse hybridization StripAssay detection of beta-thalassemia mutations in northeast Egypt.	GeneRIF	0.0044	65.1	0.0114	0.116	1	1	HBB
Pubmed	20506259	Temporal and spatial expression of osteoactivin during fracture repair.	GeneRIF	0.0044	65.1	0.0114	0.116	1	1	GPNMB
Pubmed	12563028	Stretch-activated atrial natriuretic peptide secretion in atria with heat shock protein 70 overexpression.	GeneRIF	0.0044	65.1	0.0114	0.116	1	1	NPPA
Pubmed	12016106	CX(3)C chemokine fractalkine in pulmonary arterial hypertension.	GeneRIF	0.0044	65.1	0.0114	0.116	1	1	CX3CL1
Pubmed	12736685	Defective membrane repair in dysferlin-deficient muscular dystrophy.	GeneRIF	0.0044	65.1	0.0114	0.116	1	1	DYSF
Pubmed	3422739	Identification of the glycosaminoglycan-attachment site of mouse invariant-chain proteoglycan core protein by site-directed mutagenesis.	Pubmed	0.0044	65.1	0.0114	0.116	1	1	CD74
Pubmed	12889141	[Neurogranin: a brain-specific protein].	Pubmed	0.0044	65.1	0.0114	0.116	1	1	NRGN
Pubmed	20053996	A novel mannose-binding lectin/ficolin-associated protein is highly expressed in heart and skeletal muscle tissues and inhibits complement activation.	GeneRIF	0.0044	65.1	0.0114	0.116	1	1	MASP1
Pubmed	16307662	A non-sense mutation in the corneodesmosin gene in a Mexican family with hypotrichosis simplex of the scalp.	Pubmed	0.0044	65.1	0.0114	0.116	1	1	CDSN

Category	ID	Name	Source	p-value	q-value Bonferroni	q-value FDR B&H	q-value FDR B&Y	Hit Count in Query List	Hit Count in Genome	Hit in Query List
Pubmed	24559233	Genetic analysis of BMP4 gene in Chinese Han female population with premature ovarian insufficiency.	Pubmed	0.0044	65.1	0.0114	0.116	1	1	BMP4
Pubmed	21087085	Murine aldo-keto reductase family 1 subfamily B: identification of AKR1B8 as an ortholog of human AKR1B10.	GeneRIF	0.0044	65.1	0.0114	0.116	1	1	AKR1B10
Pubmed	6687721	Hemoglobin saitama or beta 117 (G19) His leads to Pro, a new variant causing hemolytic disease.	Pubmed	0.0044	65.1	0.0114	0.116	1	1	HBB
Pubmed	25792561	Functional significance of SPINK1 promoter variants in chronic pancreatitis.	Pubmed	0.0044	65.1	0.0114	0.116	1	1	SPINK1
Pubmed	18340552	Matrilysin inhibits proliferation and modulates sensitivity of lung cancer cells to FasL-mediated apoptosis.	GeneRIF	0.0044	65.1	0.0114	0.116	1	1	MMP7
Pubmed	10825574	Regulation of the dual function tissue transglutaminase/Galpha(h) during murine neuromuscular development: gene and enzyme isoform expression.	Pubmed	0.0044	65.1	0.0114	0.116	1	1	TGM2
Pubmed	23103358	Measurement of the phospholipase activity of endothelial lipase in mouse plasma.	Pubmed	0.0044	65.1	0.0114	0.116	1	1	LIPG
Pubmed	3001652	Structure of the human gene encoding the invariant gamma-chain of class II histocompatibility antigens.	Pubmed	0.0044	65.1	0.0114	0.116	1	1	CD74
Pubmed	24367117	Cooperative assembly of IFI16 filaments on dsDNA provides insights into host defense strategy.	Pubmed	0.0044	65.1	0.0114	0.116	1	1	IFI16
Pubmed	22970163	aA-Crystallin-derived mini-chaperone modulates stability and function of cataract causing aAG98R-crystallin.	GeneRIF	0.0044	65.1	0.0114	0.116	1	1	CRYAA
Pubmed	12032167	Characterization of the lipolytic activity of endothelial lipase.	Pubmed	0.0044	65.1	0.0114	0.116	1	1	LIPG
Pubmed	23263862	Molecular and functional analysis of the C-terminal region of human erythroid-specific 5-aminolevulinic synthase associated with X-linked dominant protoporphyria (XLDPP).	GeneRIF	0.0044	65.1	0.0114	0.116	1	1	ALAS2
Pubmed	15599663	Molecular defects in the beta-globin gene identified in different ethnic groups/populations during prenatal diagnosis for beta-thalassemia: a Malaysian experience.	GeneRIF	0.0044	65.1	0.0114	0.116	1	1	HBB
Pubmed	24386483	Effects of the neurogranin variant rs12807809 on thalamocortical morphology in schizophrenia.	Pubmed	0.0044	65.1	0.0114	0.116	1	1	NRGN
Pubmed	12242667	The HtrA1 serine protease is down-regulated during human melanoma progression and represses growth of metastatic melanoma cells.	Pubmed	0.0044	65.1	0.0114	0.116	1	1	HTRA1
Pubmed	21420434	Transglutaminase 2 null macrophages respond to lipopolysaccharide stimulation by elevated proinflammatory cytokine production due to an enhanced avß3 integrin-induced Src tyrosine kinase signaling.	GeneRIF	0.0044	65.1	0.0114	0.116	1	1	TGM2
Pubmed	24277854	Targeting Wnt-driven cancer through the inhibition of Porcupine by LGK974.	Pubmed	0.0044	65.1	0.0114	0.116	1	1	PORCN
Pubmed	24403568	PCP4: a regulator of aldosterone synthesis in human adrenocortical tissues.	Pubmed	0.0044	65.1	0.0114	0.116	1	1	PCP4
Pubmed	23276939	TGM2 and implications for human disease: role of alternative splicing.	GeneRIF	0.0044	65.1	0.0114	0.116	1	1	TGM2
Pubmed	3242555	Complete amino-acid sequence of the naturally occurring A2 activator protein for enzymic sphingomyelin degradation: identity to the sulfatide activator protein (SAP-1).	Pubmed	0.0044	65.1	0.0114	0.116	1	1	PSAP
Pubmed	21216834	Junctophilin-2 expression silencing causes cardiocyte hypertrophy and abnormal intracellular calcium-handling.	GeneRIF	0.0044	65.1	0.0114	0.116	1	1	JPH2
Pubmed	19327232	Elevated serum levels of soluble CX3CL1 in patients with microscopic polyangiitis.	GeneRIF	0.0044	65.1	0.0114	0.116	1	1	CX3CL1
Pubmed	25304119	R368X mutation in MID1 among recurrent mutations in patients with X-linked Opitz G/BBB syndrome.	Pubmed	0.0044	65.1	0.0114	0.116	1	1	MID1
Pubmed	18209470	Cooperative interactions between R531 and acidic residues in the voltage sensing module of hERG1 channels.	GeneRIF	0.0044	65.1	0.0114	0.116	1	1	KCNH2
Pubmed	18473247	THE Hb S/beta+ -thalassemia phenotype demonstrates that the IVS-I (-2) (A>C) mutation is a mild beta-thalassemia allele.	GeneRIF	0.0044	65.1	0.0114	0.116	1	1	HBB
Pubmed	19958200	alpha-Thalassemia caused by two novel splice mutations of the alpha2-globin gene: IVS-I-1 (G>A and G>T).	GeneRIF	0.0044	65.1	0.0114	0.116	1	1	HBA2
Pubmed	18325042	Three novel mutations in the PORCN gene underlying focal dermal hypoplasia.	GeneRIF	0.0044	65.1	0.0114	0.116	1	1	PORCN
Pubmed	21681431	Expression of Nogo-66 receptor in human astrocytoma is correlated with tumor malignancy.	GeneRIF	0.0044	65.1	0.0114	0.116	1	1	RTN4R
Pubmed	7751801	[Subtraction cloning of growth arrest inducible genes in normal human epithelial cells].	Pubmed	0.0044	65.1	0.0114	0.116	1	1	SLC39A2
Pubmed	17415055	Polymorphisms in the 3' untranslated region of the fractalkine (CX3CL1) gene and the risk of HIV-1 infection and disease progression.	GeneRIF	0.0044	65.1	0.0114	0.116	1	1	CX3CL1
Pubmed	9718316	Isotypic variants of the interferon-inducible transcriptional repressor IFI 16 arise through differential mRNA splicing.	Pubmed	0.0044	65.1	0.0114	0.116	1	1	IFI16
Pubmed	20501432	Identification and functional characterization of the intermediate-conductance Ca(2+)-activated K(+) channel (IK-1) in biliary epithelium.	GeneRIF	0.0044	65.1	0.0114	0.116	1	1	KCNN4
Pubmed	159937	B6.C-H-2bm12. A new H-2 mutation in the I region in the mouse.	Pubmed	0.0044	65.1	0.0114	0.116	1	1	HLA-DQB1
Pubmed	8510562	Synthesis of N-(4'-pyridoxyl)sphingosine and its uptake and metabolism by isolated cells.	Pubmed	0.0044	65.1	0.0114	0.116	1	1	PDXK
Pubmed	24880079	XMD8-92 inhibits pancreatic tumor xenograft growth via a DCLK1-dependent mechanism.	Pubmed	0.0044	65.1	0.0114	0.116	1	1	DCLK1
Pubmed	8999942	Diverse genetic regulatory motifs required for murine adenosine deaminase gene expression in the placenta.	Pubmed	0.0044	65.1	0.0114	0.116	1	1	ADA
Pubmed	12975322	An essential role of Bmp4 in the atrioventricular septation of the mouse heart.	GeneRIF	0.0044	65.1	0.0114	0.116	1	1	BMP4
Pubmed	1654492	Peripheral-type benzodiazepine receptors in human cerebral cortex, kidney, and colon.	Pubmed	0.0044	65.1	0.0114	0.116	1	1	TSPO
Pubmed	12794303	Knockout of p75NTR does not alter the viability of striatal neurons following a metabolic or excitotoxic injury.	GeneRIF	0.0044	65.1	0.0114	0.116	1	1	NGFR

Category	ID	Name	Source	p-value	q-value Bonferroni	q-value FDR B&H	q-value FDR B&Y	Hit Count in Query List	Hit Count in Genome	Hit in Query List
Pubmed	20942907	Differential expression of a BMP4 reporter allele in anterior fungiform versus posterior circumvallate taste buds of mice.	GeneRIF	0.0044	65.1	0.0114	0.116	1	1	BMP4
Pubmed	21757823	Myocardial protective effect of human atrial natriuretic Peptide in cardiac surgery. -hANP Shot? in clinical safety trial-.	GeneRIF	0.0044	65.1	0.0114	0.116	1	1	NPPA
Pubmed	9244445	Assignment of the human gene for smoothelin (SMTN) to chromosome 22q12 by fluorescence in situ hybridization and radiation hybrid mapping.	Pubmed	0.0044	65.1	0.0114	0.116	1	1	SMTN
Pubmed	7579580	Cloning and sequence of bone morphogenetic protein 4 (BMP-4) from a human placental cDNA library.	Pubmed	0.0044	65.1	0.0114	0.116	1	1	BMP4
Pubmed	21991369	Genetic ablation of CD68 results in mice with increased bone and dysfunctional osteoclasts.	GeneRIF	0.0044	65.1	0.0114	0.116	1	1	CD68
Pubmed	20347569	Evaluation of cerebrospinal fluid adenosine deaminase activity in HIV-seropositive subjects and its association with lactate dehydrogenase and protein levels.	GeneRIF	0.0044	65.1	0.0114	0.116	1	1	ADA
Pubmed	14707062	Invariant chain and the MHC class II cytoplasmic domains regulate localization of MHC class II molecules to lipid rafts in tumor cell-based vaccines.	GeneRIF	0.0044	65.1	0.0114	0.116	1	1	CD74
Pubmed	10850529	Intracoronary serum smooth muscle myosin heavy chain levels following PTCA may predict restenosis.	Pubmed	0.0044	65.1	0.0114	0.116	1	1	MYH11
Pubmed	17823114	Activation gating of hERG potassium channels: S6 glycines are not required as gating hinges.	GeneRIF	0.0044	65.1	0.0114	0.116	1	1	KCNH2
Pubmed	21757944	A 13-bp deletion in the 3' untranslated region of the ß-globin gene causes ß-thalassemia major in compound heterozygosity with IVSII-1 mutation.	GeneRIF	0.0044	65.1	0.0114	0.116	1	1	HBB
Pubmed	18688035	Tissue transglutaminase contributes to interstitial renal fibrosis by favoring accumulation of fibrillar collagen through TGF-beta activation and cell infiltration.	GeneRIF	0.0044	65.1	0.0114	0.116	1	1	TGM2
Pubmed	15980664	Mutations in the serine protease inhibitor Kazal Type 1 (SPINK1) gene in Japanese patients with pancreatitis.	GeneRIF	0.0044	65.1	0.0114	0.116	1	1	SPINK1
Pubmed	12925527	IFI16 as a negative regulator in the regulation of p53 and p21(Waf1).	GeneRIF	0.0044	65.1	0.0114	0.116	1	1	IFI16
Pubmed	14729678	Nuclear export of the oncoprotein v-ErbA is mediated by acquisition of a viral nuclear export sequence.	GeneRIF	0.0044	65.1	0.0114	0.116	1	1	THRA
Pubmed	22366952	High abundance of plasma cells secreting transglutaminase 2-specific IgA autoantibodies with limited somatic hypermutation in celiac disease intestinal lesions.	GeneRIF	0.0044	65.1	0.0114	0.116	1	1	TGM2
Pubmed	3864916	Invariant chain is the core protein of the Ia-associated chondroitin sulfate proteoglycan.	Pubmed	0.0044	65.1	0.0114	0.116	1	1	CD74
Pubmed	21647148	Risk variant for schizophrenia in the neurogranin gene impacts on hippocampus activation during contextual fear conditioning.	GeneRIF	0.0044	65.1	0.0114	0.116	1	1	NRGN
Pubmed	2477366	Interferons as gene activators. Indications for repeated gene duplication during the evolution of a cluster of interferon-activatable genes on murine chromosome 1.	Pubmed	0.0044	65.1	0.0114	0.116	1	1	IFI16
Pubmed	12515291	Intestinal permeability to mannitol and lactulose in children with type 1 diabetes with the HLA-DQB1*02 allele.	GeneRIF	0.0044	65.1	0.0114	0.116	1	1	HLA-DQB1
Pubmed	23334847	The E3 ubiquitin ligase midline 1 promotes allergen and rhinovirus-induced asthma by inhibiting protein phosphatase 2A activity.	GeneRIF	0.0044	65.1	0.0114	0.116	1	1	MID1
Pubmed	23606517	Mouse pyrin and HIN domain family member 1 (pyhin1) protein positively regulates LPS-induced IFN-ß and NO production in macrophages.	Pubmed	0.0044	65.1	0.0114	0.116	1	1	IFI16
Pubmed	3557994	Hemoglobin La Desirade alpha A2 beta 2 129 (H7) Ala----Val: a new unstable hemoglobin.	Pubmed	0.0044	65.1	0.0114	0.116	1	1	HBB
Pubmed	15265002	Cathepsin K: a cysteine protease with unique kinin degrading properties.	Pubmed	0.0044	65.1	0.0114	0.116	1	1	CTSK
Pubmed	11441124	Acetylcholine receptor peptide recognition in HLA DR3-transgenic mice: in vivo responses correlate with MHC-peptide binding.	Pubmed	0.0044	65.1	0.0114	0.116	1	1	HLA-DQB1
Pubmed	24193405	Mg²¿ modulation of the single-channel properties of KCa3.1 in human erythroleukemia cells.	Pubmed	0.0044	65.1	0.0114	0.116	1	1	KCNN4
Pubmed	8993389	The biosynthetic processing and secretion of endogenous carboxypeptidase H in mouse pituitary cells.	Pubmed	0.0044	65.1	0.0114	0.116	1	1	CPE
Pubmed	10234569	Characterization of mouse cathepsin K gene, the gene promoter, and the gene expression.	Pubmed	0.0044	65.1	0.0114	0.116	1	1	CTSK
Pubmed	18941465	Smooth-muscle myosin mutations in hereditary non-polyposis colorectal cancer syndrome.	GeneRIF	0.0044	65.1	0.0114	0.116	1	1	MYH11
Pubmed	20149358	Serum inhibin-b in fertile men is strongly correlated with low but not high sperm counts: a coordinated study of 1,797 European and US men.	GeneRIF	0.0044	65.1	0.0114	0.116	1	1	INHBB
Pubmed	21134591	[Peeling skin disease: the responsibility of corneodesmosin].	GeneRIF	0.0044	65.1	0.0114	0.116	1	1	CDSN
Pubmed	16243036	P-Rex1 is a primary Rac2 guanine nucleotide exchange factor in mouse neutrophils.	GeneRIF	0.0044	65.1	0.0114	0.116	1	1	PREX1
Pubmed	23470493	Allele-specific RNA interference rescues the long-QT syndrome phenotype in human-induced pluripotency stem cell cardiomyocytes.	Pubmed	0.0044	65.1	0.0114	0.116	1	1	KCNH2
Pubmed	23355200	Napsin A expression in primary mucin-producing adenocarcinomas of the lung: an immunohistochemical study.	GeneRIF	0.0044	65.1	0.0114	0.116	1	1	NAPSA
Pubmed	23398055	Normal Hb A2 ß-thalassemia trait: frameshift mutation (HBB: c.187_251dup) in cis with the Hb A2' d-globin gene missense mutation (HBD: c.49G>C).	Pubmed	0.0044	65.1	0.0114	0.116	1	1	HBB
Pubmed	15648549	Intracerebroventricular administration of atrial natriuretic peptide prevents increase of plasma ADH, aldosterone and corticosterone levels in restrained conscious dehydrated rabbits.	GeneRIF	0.0044	65.1	0.0114	0.116	1	1	NPPA
Pubmed	15923193	Cross-species sequence analysis reveals multiple charged residue-rich domains that regulate nuclear/cytoplasmic partitioning and membrane localization of a kinase anchoring protein 12 (SSeCKS/Gravin).	GeneRIF	0.0044	65.1	0.0114	0.116	1	1	AKAP12

Category	ID	Name	Source	p-value	q-value Bonferroni	q-value FDR B&H	q-value FDR B&Y	Hit Count in Query List	Hit Count in Genome	Hit in Query List
Pubmed	17475886	Gpnmb is induced in macrophages by IFN-gamma and lipopolysaccharide and acts as a feedback regulator of proinflammatory responses.	GeneRIF	0.0044	65.1	0.0114	0.116	1	1	GPNMB
Pubmed	10671522	Carboxypeptidase Z is present in the regulated secretory pathway and extracellular matrix in cultured cells and in human tissues.	Pubmed	0.0044	65.1	0.0114	0.116	1	1	CPZ
Pubmed	12766166	Bone morphogenic protein 4 produced in endothelial cells by oscillatory shear stress stimulates an inflammatory response.	GeneRIF	0.0044	65.1	0.0114	0.116	1	1	BMP4
Pubmed	22216983	Identification of the p. R116H mutation in a Chinese family with novel variable cataract phenotype: evidence for a mutational hot spot in aA-crystallin gene.	GeneRIF	0.0044	65.1	0.0114	0.116	1	1	CRYAA
Pubmed	9458354	Expression of human endogenous retrovirus ERV3 (HERV-R) mRNA in normal and neoplastic tissues.	Pubmed	0.0044	65.1	0.0114	0.116	1	1	ERV3-1
Pubmed	8175905	A novel phosphoglucomutase-related protein is concentrated in adherens junctions of muscle and nonmuscle cells.	Pubmed	0.0044	65.1	0.0114	0.116	1	1	PGM5
Pubmed	2121367	MHC class II-associated invariant chain contains a sorting signal for endosomal compartments.	Pubmed	0.0044	65.1	0.0114	0.116	1	1	CD74
Pubmed	11062177	Interindividual variation in CYP1A1 expression in breast tissue and the role of genetic polymorphism.	GeneRIF	0.0044	65.1	0.0114	0.116	1	1	CYP11A1
Pubmed	20398728	Pituitary tumors: cell type-specific roles for BMP-4.	Pubmed	0.0044	65.1	0.0114	0.116	1	1	BMP4
Pubmed	11247893	Parallel expression of macrophage metalloelastase (MMP-12) in duodenal and skin lesions of patients with dermatitis herpetiformis.	Pubmed	0.0044	65.1	0.0114	0.116	1	1	MMP7
Pubmed	24682206	Insights into behavioral vulnerability to differential sleep pressure and circadian phase from a functional ADA polymorphism.	Pubmed	0.0044	65.1	0.0114	0.116	1	1	ADA
Pubmed	21128249	Carcinogen-induced squamous papillomas and oncogenic progression in the absence of the SSeCKS/AKAP12 metastasis suppressor correlate with FAK upregulation.	GeneRIF	0.0044	65.1	0.0114	0.116	1	1	AKAP12
Pubmed	19644414	Relationship between haplotypes of KCNN4 gene and susceptibility to human vascular diseases in Japanese.	GeneRIF	0.0044	65.1	0.0114	0.116	1	1	KCNN4
Pubmed	22216005	Epstein-Barr virus evades CD4+ T cell responses in lytic cycle through BZLF1-mediated downregulation of CD74 and the cooperation of vBcl-2.	GeneRIF	0.0044	65.1	0.0114	0.116	1	1	CD74
Pubmed	8449522	Assignment of the human carboxypeptidase E (CPE) gene to chromosome 4.	Pubmed	0.0044	65.1	0.0114	0.116	1	1	CPE
Pubmed	24391722	To investigate the necessity of STRA6 upregulation in T cells during T cell immune responses.	Pubmed	0.0044	65.1	0.0114	0.116	1	1	STRA6
Pubmed	15664014	Impaired bone resorption in cathepsin K-deficient mice is partially compensated for by enhanced osteoclastogenesis and increased expression of other proteases via an increased RANKL/OPG ratio.	Pubmed	0.0044	65.1	0.0114	0.116	1	1	CTSK
Pubmed	18555216	Osteoactivin, an anabolic factor that regulates osteoblast differentiation and function.	GeneRIF	0.0044	65.1	0.0114	0.116	1	1	GPNMB
Pubmed	10727467	Invariant chain controls H2-M proteolysis in mouse splenocytes and dendritic cells.	Pubmed	0.0044	65.1	0.0114	0.116	1	1	CD74
Pubmed	17118749	BMP stimulation of alkaline phosphatase activity in pluripotent mouse C2C12 cells is inhibited by dermatopontin, one of the most abundant low molecular weight proteins in demineralized bone matrix.	GeneRIF	0.0044	65.1	0.0114	0.116	1	1	DPT
Pubmed	11248802	Secretion of microbicidal alpha-defensins by intestinal Paneth cells in response to bacteria.	Pubmed	0.0044	65.1	0.0114	0.116	1	1	MMP7
Pubmed	11300346	Prenatal diagnosis of beta-thalassemia major by high-performance liquid chromatography analysis of hemoglobins in fetal blood samples.	GeneRIF	0.0044	65.1	0.0114	0.116	1	1	HBB
Pubmed	9765596	Sequence and expression levels in human tissues of a new member of the aldo-keto reductase family.	Pubmed	0.0044	65.1	0.0114	0.116	1	1	AKR1B10
Pubmed	10625055	P75 nerve growth factor receptor is important for retrograde transport of neurotrophins in adult cholinergic basal forebrain neurons.	Pubmed	0.0044	65.1	0.0114	0.116	1	1	NGFR
Pubmed	20615646	Activation of transglutaminase type 2 for aortic wall protection in a rat abdominal aortic aneurysm formation.	GeneRIF	0.0044	65.1	0.0114	0.116	1	1	TGM2
Pubmed	24306026	Ehd3, a regulator of vesicular trafficking, is silenced in gliomas and functions as a tumor suppressor by controlling cell cycle arrest and apoptosis.	Pubmed	0.0044	65.1	0.0114	0.116	1	1	EHD3
Pubmed	1423520	Differentiation of human skeletal muscle cells in culture: maturation as indicated by titin and desmin striation.	Pubmed	0.0044	65.1	0.0114	0.116	1	1	ACTA1
Pubmed	23717413	Glucose homeostasis in mice is transglutaminase 2 independent.	Pubmed	0.0044	65.1	0.0114	0.116	1	1	TGM2
Pubmed	19618018	The relationship between the type of mutation in the globin gene and the type and severity of sickle/beta-thalassemia disease in Jordanian patients.	GeneRIF	0.0044	65.1	0.0114	0.116	1	1	HBB
Pubmed	25036006	Shisa3 is associated with prolonged survival through promoting ß-catenin degradation in lung cancer.	Pubmed	0.0044	65.1	0.0114	0.116	1	1	SHISA3
Pubmed	11602353	Murine cysteine dioxygenase gene: structural organization, tissue-specific expression and promoter identification.	GeneRIF	0.0044	65.1	0.0114	0.116	1	1	CDO1
Pubmed	21551009	Hypoxic exercise training causes erythrocyte senescence and rheological dysfunction by depressed Gardos channel activity.	GeneRIF	0.0044	65.1	0.0114	0.116	1	1	KCNN4
Pubmed	24166472	KCa3.1 mediates activation of fibroblasts in diabetic renal interstitial fibrosis.	Pubmed	0.0044	65.1	0.0114	0.116	1	1	KCNN4
Pubmed	23139156	Channel sialic acids limit hERG channel activity during the ventricular action potential.	GeneRIF	0.0044	65.1	0.0114	0.116	1	1	KCNH2
Pubmed	21820048	Role of VPO1, a newly identified heme-containing peroxidase, in ox-LDL induced endothelial cell apoptosis.	GeneRIF	0.0044	65.1	0.0114	0.116	1	1	PXDN
Pubmed	6230082	Purification and complete amino acid sequence of alpha-human atrial natriuretic polypeptide (alpha-hANP).	Pubmed	0.0044	65.1	0.0114	0.116	1	1	NPPA

Category	ID	Name	Source	p-value	q-value Bonferroni	q-value FDR B&H	q-value FDR B&Y	Hit Count in Query List	Hit Count in Genome	Hit in Query List
Pubmed	12573711	Expression of the low affinity neurotrophin receptor p75 in spinal motoneurons in a transgenic mouse model for amyotrophic lateral sclerosis.	GeneRIF	0.0044	65.1	0.0114	0.116	1	1	NGFR
Pubmed	12031592	A novel mutation in exon 5 of the ALAS2 gene results in X-linked sideroblastic anemia.	GeneRIF	0.0044	65.1	0.0114	0.116	1	1	ALAS2
Pubmed	18071703	Identification of beta thalassemia mutations in South Brazilians.	GeneRIF	0.0044	65.1	0.0114	0.116	1	1	HBB
Pubmed	10474818	gamma-Glutamyl transferase (GGT) deficiency in the GGTenu1 mouse results from a single point mutation that leads to a stop codon in the first coding exon of GGT mRNA.	Pubmed	0.0044	65.1	0.0114	0.116	1	1	GGT1
Pubmed	16766195	The regulation of tau phosphorylation by PCTAIRE 3: implications for the pathogenesis of Alzheimer's disease.	GeneRIF	0.0044	65.1	0.0114	0.116	1	1	CDK18
Pubmed	24606930	Proline scan of the HERG channel S6 helix reveals the location of the intracellular pore gate.	Pubmed	0.0044	65.1	0.0114	0.116	1	1	KCNH2
Pubmed	3840930	The nucleotide sequence of the env gene from the human provirus ERV3 and isolation and characterization of an ERV3-specific cDNA.	Pubmed	0.0044	65.1	0.0114	0.116	1	1	ERV3-1
Pubmed	21333566	The association between intragenic SNP haplotypes and mutations of the beta globin gene in a Turkish population.	GeneRIF	0.0044	65.1	0.0114	0.116	1	1	HBB
Pubmed	16488412	Null mutations for exon III and exon IV of the p75 neurotrophin receptor gene enhance sympathetic sprouting in response to elevated levels of nerve growth factor in transgenic mice.	Pubmed	0.0044	65.1	0.0114	0.116	1	1	NGFR
Pubmed	23681073	Napsin A expression in anaplastic, poorly differentiated, and micropapillary pattern thyroid carcinomas.	GeneRIF	0.0044	65.1	0.0114	0.116	1	1	NAPSA
Pubmed	24274186	Hb Ullevaal [ß78(EF2)Leu¿Val; HBB: c.235C>G], a new hemoglobin variant interfering with Hb A1c measurement using a cation exchange high performance liquid chromatography method.	Pubmed	0.0044	65.1	0.0114	0.116	1	1	HBB
Pubmed	25692548	Cathepsin K knockout alleviates aging-induced cardiac dysfunction.	Pubmed	0.0044	65.1	0.0114	0.116	1	1	CTSK
Pubmed	2884330	Tissue-specific expression of human provirus ERV3 mRNA in human placenta: two of the three ERV3 mRNAs contain human cellular sequences.	Pubmed	0.0044	65.1	0.0114	0.116	1	1	ERV3-1
Pubmed	24424413	T cell immunodominance is dictated by the positively selecting self-peptide.	Pubmed	0.0044	65.1	0.0114	0.116	1	1	CD74
Pubmed	18952077	Invariant chain+ N2a neuroblastoma cells stably expressing the class II MHC transactivator CIITA fail to stimulate anti-tumor immunity.	GeneRIF	0.0044	65.1	0.0114	0.116	1	1	CD74
Pubmed	15863355	Glycosylation of site-specific glycans of alpha1-acid glycoprotein and alterations in acute and chronic inflammation.	GeneRIF	0.0044	65.1	0.0114	0.116	1	1	ORM2
Pubmed	11771768	A potential role for the p75 low-affinity neurotrophin receptor in spinal motor neuron degeneration in murine and human amyotrophic lateral sclerosis.	GeneRIF	0.0044	65.1	0.0114	0.116	1	1	NGFR
Pubmed	17227580	Phenotypes of myopathy-related actin mutants in differentiated C2C12 myotubes.	GeneRIF	0.0044	65.1	0.0114	0.116	1	1	ACTA1
Pubmed	20148757	Intensive therapies for mantle cell lymphoma: time for a disease-specific approach?	Pubmed	0.0044	65.1	0.0114	0.116	1	1	SLC39A2
Pubmed	19950207	BMP4 regulates vascular progenitor development in human embryonic stem cells through a Smad-dependent pathway.	GeneRIF	0.0044	65.1	0.0114	0.116	1	1	BMP4
Pubmed	17332365	Fractalkine (CX3CL1)- and interleukin-2-enriched neuroblastoma microenvironment induces eradication of metastases mediated by T cells and natural killer cells.	GeneRIF	0.0044	65.1	0.0114	0.116	1	1	CX3CL1
Pubmed	25898755	[Immunization witha synthetic fragment 155-164 of neurotrophin receptor p75 prevents memory loss and decreases beta-amyloid level in mice with experimentally induced Alzheimer's disease].	Pubmed	0.0044	65.1	0.0114	0.116	1	1	NGFR
Pubmed	14585970	A human globin enhancer causes both discrete and widespread alterations in chromatin structure.	GeneRIF	0.0044	65.1	0.0114	0.116	1	1	HBB
Pubmed	24794504	No association of IFI16 (interferon-inducible protein 16) variants with susceptibility to multiple sclerosis.	Pubmed	0.0044	65.1	0.0114	0.116	1	1	IFI16
Pubmed	23785173	Influence of TSPO genotype on 11C-PBR28 standardized uptake values.	Pubmed	0.0044	65.1	0.0114	0.116	1	1	TSPO
Pubmed	22363992	[Pancreatic secretory trypsin inhibitor gene N34S mutation in patients with idiopathic chronic pancreatitis].	GeneRIF	0.0044	65.1	0.0114	0.116	1	1	SPINK1
Pubmed	14990577	Function of conserved residues of human glutathione synthetase: implications for the ATP-grasp enzymes.	GeneRIF	0.0044	65.1	0.0114	0.116	1	1	GSS
Pubmed	16103715	Mutations associated with beta-thalassemia intermedia in Kuwait.	GeneRIF	0.0044	65.1	0.0114	0.116	1	1	HBB
Pubmed	19146958	Inhibition of p75(NTR) in glia potentiates TrkA-mediated survival of injured retinal ganglion cells.	Pubmed	0.0044	65.1	0.0114	0.116	1	1	NGFR
Pubmed	22104430	Evaluation of cerebrospinal fluid adenosine deaminase activity for the differential diagnosis of tuberculous and nontuberculous meningitis.	GeneRIF	0.0044	65.1	0.0114	0.116	1	1	ADA
Pubmed	27075	Effects of sodium nitroprusside and other smooth muscle relaxants on cyclic GMP formation in smooth muscle and platelets.	Pubmed	0.0044	65.1	0.0114	0.116	1	1	TSPAN13
Pubmed	21674241	BMP-4 expression has prognostic significance in advanced serous ovarian carcinoma and is affected by cisplatin in OVCAR-3 cells.	GeneRIF	0.0044	65.1	0.0114	0.116	1	1	BMP4
Pubmed	18497708	Administration of exogenous fractalkine, a CX3C chemokine, is capable of modulating inflammatory response in cecal ligation and puncture-induced sepsis.	GeneRIF	0.0044	65.1	0.0114	0.116	1	1	CX3CL1
Pubmed	24460721	BMP4 promotes vascularization of human adipose stromal cells and endothelial cells in vitro and in vivo.	Pubmed	0.0044	65.1	0.0114	0.116	1	1	BMP4
Pubmed	20414589	Lower frequency of the low activity adenosine deaminase allelic variant (ADA1*2) in schizophrenic patients.	GeneRIF	0.0044	65.1	0.0114	0.116	1	1	ADA

Category	ID	Name	Source	p-value	q-value Bonferroni	q-value FDR B&H	q-value FDR B&Y	Hit Count in Query List	Hit Count in Genome	Hit in Query List
Pubmed	11843884	Molecular analysis of beta-thalassaemia patients in a high incidence area of southern Italy.	GeneRIF	0.0044	65.1	0.0114	0.116	1	1	HBB
Pubmed	22654577	Role of hemoglobin and serum iron in oral submucous fibrosis: a clinical study.	GeneRIF	0.0044	65.1	0.0114	0.116	1	1	HBB
Pubmed	10395666	I-Ag7-mediated antigen presentation by B lymphocytes is critical in overcoming a checkpoint in T cell tolerance to islet beta cells of nonobese diabetic mice.	Pubmed	0.0044	65.1	0.0114	0.116	1	1	HLA-DQB1
Pubmed	23690979	Nuclear DNA sensor IFI16 as circulating protein in autoimmune diseases is a signal of damage that impairs endothelial cells through high-affinity membrane binding.	Pubmed	0.0044	65.1	0.0114	0.116	1	1	IFI16
Pubmed	21816820	Structures and oxygen affinities of crystalline human hemoglobin C (ß6 Glu->Lys) in the R and R2 quaternary structures.	GeneRIF	0.0044	65.1	0.0114	0.116	1	1	HBB
Pubmed	14762600	[Determination of autoantibody for myelin antigens in the serum of patients HLA-DQB1*0602 with multiple sclerosis].	GeneRIF	0.0044	65.1	0.0114	0.116	1	1	HLA-DQB1
Pubmed	21994736	Actin in herpesvirus infection.	GeneRIF	0.0044	65.1	0.0114	0.116	1	1	ACTA1
Pubmed	15347667	Physiological roles of the intermediate conductance, Ca2+-activated potassium channel Kcnn4.	GeneRIF	0.0044	65.1	0.0114	0.116	1	1	KCNN4
Pubmed	16428778	Intranasal exposure to bacterial superantigens induces airway inflammation in HLA class II transgenic mice.	Pubmed	0.0044	65.1	0.0114	0.116	1	1	HLA-DQB1
Pubmed	25635829	Identification of a key cholesterol binding enhancement motif in translocator protein 18 kDa.	Pubmed	0.0044	65.1	0.0114	0.116	1	1	TSPO
Pubmed	20035815	Rapid genetic screening for major human leukocyte antigen risk haplotypes in patients with type 1 diabetes from Northeastern Brazil.	GeneRIF	0.0044	65.1	0.0114	0.116	1	1	HLA-DQB1
Pubmed	6857757	Hemoglobin Rio Grande [beta 8 (A5) Lys leads to Thr] a new variant found in a Mexican-American family.	Pubmed	0.0044	65.1	0.0114	0.116	1	1	HBB
Pubmed	12392704	Secretory leukocyte protease inhibitor: inhibition o human immunodeficiency virus-1 infection of monocytic THP-1 cells by a newly cloned protein.	Pubmed	0.0044	65.1	0.0114	0.116	1	1	SLPI
Pubmed	23562717	Update meta-analysis on MMP-7 -181A>G polymorphism and cancer risk: evidence from 25 studies.	GeneRIF	0.0044	65.1	0.0114	0.116	1	1	MMP7
Pubmed	20157294	Relationship of strain-dependent susceptibility to experimentally induced acute pancreatitis with regulation of Prss1 and Spink3 expression.	GeneRIF	0.0044	65.1	0.0114	0.116	1	1	SPINK1
Pubmed	9351446	Electrophysiological characterization of an alternatively processed ERG K+ channel in mouse and human hearts.	Pubmed	0.0044	65.1	0.0114	0.116	1	1	KCNH2
Pubmed	15056289	Astrocytic production of nerve growth factor in motor neuron apoptosis: implications for amyotrophic lateral sclerosis.	Pubmed	0.0044	65.1	0.0114	0.116	1	1	NGFR
Pubmed	11473121	Cleavage of doublecortin-like kinase by calpain releases an active kinase fragment from a microtubule anchorage domain.	GeneRIF	0.0044	65.1	0.0114	0.116	1	1	DCLK1
Pubmed	12120224	SPINK1 mutations are associated with multiple phenotypes.	GeneRIF	0.0044	65.1	0.0114	0.116	1	1	SPINK1
Pubmed	22966065	The p35 human invariant chain in transgenic mice restores mature B cells in the absence of endogenous CD74.	GeneRIF	0.0044	65.1	0.0114	0.116	1	1	CD74
Pubmed	3920321	One gene encodes two distinct Ia-associated invariant chains.	Pubmed	0.0044	65.1	0.0114	0.116	1	1	CD74
Pubmed	16614359	Castration prevents suppression of MHC class II (Ia) expression on macrophages after trauma-hemorrhage.	GeneRIF	0.0044	65.1	0.0114	0.116	1	1	HLA-DQB1
Pubmed	1515698	Effect of cholesterol and its autooxidation derivatives on endocytosis and dipeptidyl peptidases of aortic endothelial cells.	Pubmed	0.0044	65.1	0.0114	0.116	1	1	DPP7
Pubmed	20558140	Functional dominant-negative mutation of sodium channel subunit gene SCN3B associated with atrial fibrillation in a Chinese GeneID population.	GeneRIF	0.0044	65.1	0.0114	0.116	1	1	SCN3B
Pubmed	19027045	HLA-DQ and risk gradient for celiac disease.	GeneRIF	0.0044	65.1	0.0114	0.116	1	1	HLA-DQB1
Pubmed	12507777	Suppressed clinical experimental autoimmune myasthenia gravis in bm12 mice is linked to reduced intracellular calcium mobilization and IL-10 and IFN-gamma release by acetylcholine receptor-specific T cells.	Pubmed	0.0044	65.1	0.0114	0.116	1	1	HLA-DQB1
Pubmed	19247486	Inter-MAR association contributes to transcriptionally active looping events in human beta-globin gene cluster.	GeneRIF	0.0044	65.1	0.0114	0.116	1	1	HBB
Pubmed	15621116	Quantitative gene expression analysis of fractalkine using laser microdissection in biopsies from kidney allografts with acute rejection.	GeneRIF	0.0044	65.1	0.0114	0.116	1	1	CX3CL1
Pubmed	19326120	[Amyloidosis in muscular dystrophy].	GeneRIF	0.0044	65.1	0.0114	0.116	1	1	DYSF
Pubmed	18786358	p75(NTR) mediates ephrin-A reverse signaling required for axon repulsion and mapping.	Pubmed	0.0044	65.1	0.0114	0.116	1	1	NGFR
Pubmed	24582965	Histone demethylase RBP2 is critical for breast cancer progression and metastasis.	Pubmed	0.0044	65.1	0.0114	0.116	1	1	KDM5A
Pubmed	10862717	Identification and molecular-genetic characterization of a LAMP/CD68-like protein from Caenorhabditis elegans.	Pubmed	0.0044	65.1	0.0114	0.116	1	1	CD68
Pubmed	22723003	The association between endothelial lipase -384A/C gene polymorphism and acute coronary syndrome in a Chinese population.	GeneRIF	0.0044	65.1	0.0114	0.116	1	1	LIPG
Pubmed	19917686	MAp44, a human protein associated with pattern recognition molecules of the complement system and regulating the lectin pathway of complement activation.	GeneRIF	0.0044	65.1	0.0114	0.116	1	1	MASP1
Pubmed	15012590	Obesity and diabetes in transgenic mice expressing proSAAS.	Pubmed	0.0044	65.1	0.0114	0.116	1	1	CPE
Pubmed	22266633	Association between ¿-glutamyltransferase and prehypertension.	GeneRIF	0.0044	65.1	0.0114	0.116	1	1	GGT1
Pubmed	25389337	The lung-restricted marker napsin A is highly expressed in clear cell carcinomas of the ovary.	Pubmed	0.0044	65.1	0.0114	0.116	1	1	NAPSA
Pubmed	11451415	Hidden variables: unstable Abeta chain genes encoding antigen recognition structures in tumor survivors.	Pubmed	0.0044	65.1	0.0114	0.116	1	1	HLA-DQB1

Category	ID	Name	Source	p-value	q-value Bonferroni	q-value FDR B&H	q-value FDR B&Y	Hit Count in Query List	Hit Count in Genome	Hit in Query List
Pubmed	20097192	The amiodarone derivative KB130015 activates hERG1 potassium channels via a novel mechanism.	GeneRIF	0.0044	65.1	0.0114	0.116	1	1	KCNH2
Pubmed	17654072	Distribution of beta-thalassemia mutations in the northern provinces of Iran.	GeneRIF	0.0044	65.1	0.0114	0.116	1	1	HBB
Pubmed	22587817	Structure-activity relationships for the binding of polymyxins with human a-1-acid glycoprotein.	GeneRIF	0.0044	65.1	0.0114	0.116	1	1	ORM1
Pubmed	15496411	Multiple promoters direct expression of three AKAP12 isoforms with distinct subcellular and tissue distribution profiles.	GeneRIF	0.0044	65.1	0.0114	0.116	1	1	AKAP12
Pubmed	11850175	Derivation in culture of primordial germ cells from cells of the mouse epiblast: phenotypic induction and growth control by Bmp4 signalling.	GeneRIF	0.0044	65.1	0.0114	0.116	1	1	BMP4
Pubmed	11883932	Osmotic resistance of high-density erythrocytes in transglutaminase 2-deficient mice.	GeneRIF	0.0044	65.1	0.0114	0.116	1	1	TGM2
Pubmed	8107717	X-linked pyridoxine-responsive sideroblastic anemia due to a Thr388-to-Ser substitution in erythroid 5-aminolevulinate synthase.	Pubmed	0.0044	65.1	0.0114	0.116	1	1	ALAS2
Pubmed	21109023	Functional effects of a missense mutation in HERG associated with type 2 long QT syndrome.	GeneRIF	0.0044	65.1	0.0114	0.116	1	1	KCNH2
Pubmed	21115960	A missense HTRA1 mutation expands CARASIL syndrome to the Caucasian population.	GeneRIF	0.0044	65.1	0.0114	0.116	1	1	HTRA1
Pubmed	858816	Defective endochondral ossification in brachymorphic mice.	Pubmed	0.0044	65.1	0.0114	0.116	1	1	PAPSS2
Pubmed	22560623	Myogenesis in dysferlin-deficient myoblasts is inhibited by an intrinsic inflammatory response.	GeneRIF	0.0044	65.1	0.0114	0.116	1	1	DYSF
Pubmed	24006921	Upregulation of CPE promotes cell proliferation and tumorigenicity in colorectal cancer.	Pubmed	0.0044	65.1	0.0114	0.116	1	1	CPE
Pubmed	17584649	[Cloning of human PAK6 cDNA, preparation of anti-PAK6 polyclonal antibody and PAK6 expression in prostate cancer].	GeneRIF	0.0044	65.1	0.0114	0.116	1	1	PAK6
Pubmed	21945129	HIV-mediated up-regulation of invariant chain (CD74) correlates with generalized immune activation in HIV+ subjects.	GeneRIF	0.0044	65.1	0.0114	0.116	1	1	CD74
Pubmed	9622483	Complexes of adenosine deaminase with two potent inhibitors: X-ray structures in four independent molecules at pH of maximum activity.	Pubmed	0.0044	65.1	0.0114	0.116	1	1	ADA
Pubmed	1235495	Institutionalization, deinstitutionalization and the adversary process.	Pubmed	0.0044	65.1	0.0114	0.116	1	1	LARGE
Pubmed	19800993	A cysteine-type carboxypeptidase, cathepsin X, generates peptide receptor agonists.	GeneRIF	0.0044	65.1	0.0114	0.116	1	1	CTSK
Pubmed	23761265	The mERG1a channel modulates skeletal muscle MuRF1, but not MAFbx, expression.	Pubmed	0.0044	65.1	0.0114	0.116	1	1	KCNH2
Pubmed	20943077	[Effects of AKR1B10 gene silence on the growth and gene expression of HCC cell line MHCC97H].	GeneRIF	0.0044	65.1	0.0114	0.116	1	1	AKR1B10
Pubmed	12055082	Claudins create charge-selective channels in the paracellular pathway between epithelial cells.	Pubmed	0.0044	65.1	0.0114	0.116	1	1	CLDN15
Pubmed	17133428	The study of sequence configuration and functiona impact of the (AC)n(AT)xTy motif in human beta-globin gene promoter.	GeneRIF	0.0044	65.1	0.0114	0.116	1	1	HBB
Pubmed	15339840	Distribution of dehydration rates generated by maximal Gardos-channel activation in normal and sickle red blood cells.	GeneRIF	0.0044	65.1	0.0114	0.116	1	1	KCNN4
Pubmed	7584048	Prediction of the coding sequences of unidentified human genes. II. The coding sequences of 40 new genes (KIAA0041-KIAA0080) deduced by analysis of cDNA clones from human cell line KG-1 (supplement).	Pubmed	0.0044	65.1	0.0114	0.116	1	1	ARHGEF6
Pubmed	11408388	Polymorphisms in the hANP (human atrial natriuretic peptide) gene, albuminuria, and hypertension.	GeneRIF	0.0044	65.1	0.0114	0.116	1	1	NPPA
Pubmed	15548330	Saposin C promotes survival and prevents apoptosis via PI3K/Akt-dependent pathway in prostate cancer cells.	GeneRIF	0.0044	65.1	0.0114	0.116	1	1	PSAP
Pubmed	23547709	The Wnt signalling pathway is upregulated in an in vitro model of acquired tamoxifen resistant breast cancer.	Pubmed	0.0044	65.1	0.0114	0.116	1	1	PORCN
Pubmed	19723494	Angiopoietin-like protein 2 promotes chronic adipose tissue inflammation and obesity-related systemic insulin resistance.	GeneRIF	0.0044	65.1	0.0114	0.116	1	1	ANGPTL2
Pubmed	14724254	Suppression of p75NTR does not promote regeneration of injured spinal cord in mice.	GeneRIF	0.0044	65.1	0.0114	0.116	1	1	NGFR
Pubmed	15682454	B7-H3 promotes acute and chronic allograft rejection.	Pubmed	0.0044	65.1	0.0114	0.116	1	1	CD276
Pubmed	21079765	Proteomic analysis of the dysferlin protein comple: unveils its importance for sarcolemmal maintenance and integrity.	GeneRIF	0.0044	65.1	0.0114	0.116	1	1	DYSF
Pubmed	25155404	The spectrum of ß-thalassemia mutations in Hatay, Turkey: reporting three new mutations.	Pubmed	0.0044	65.1	0.0114	0.116	1	1	HBB
Pubmed	20036025	Identification and expression analysis of the aldo-ketoreductase1-B10 gene in primary malignant liver tumours.	GeneRIF	0.0044	65.1	0.0114	0.116	1	1	AKR1B10
Pubmed	10688874	beta 3: an additional auxiliary subunit of the voltage-sensitive sodium channel that modulates channel gating with distinct kinetics.	Pubmed	0.0044	65.1	0.0114	0.116	1	1	SCN3B
Pubmed	17219436	MMP-7 (matrilysin) expression in human brain tumors.	GeneRIF	0.0044	65.1	0.0114	0.116	1	1	MMP7
Pubmed	18955490	Identification of amino acid residues of matrix metalloproteinase-7 essential for binding to cholesterol sulfate.	GeneRIF	0.0044	65.1	0.0114	0.116	1	1	MMP7
Pubmed	10692254	The cellular mechanism by which the human endogenous retrovirus ERV-3 env gene affects proliferation and differentiation in a human placental trophoblast model, BeWo.	Pubmed	0.0044	65.1	0.0114	0.116	1	1	ERV3-1
Pubmed	23635481	Association of functional kallikrein-1 promoter polymorphisms and acute kidney injury: a case-control and longitudinal cohort study.	Pubmed	0.0044	65.1	0.0114	0.116	1	1	KLK1
Pubmed	19459043	Enhanced expression of alpha1-acid glycoprotein and fucosylation in hepatitis B patients provides an insight into pathogenesis.	GeneRIF	0.0044	65.1	0.0114	0.116	1	1	ORM1

Category	ID	Name	Source	p-value	q-value Bonferroni	q-value FDR B&H	q-value FDR B&Y	Hit Count in Query List	Hit Count in Genome	Hit in Query List
Pubmed	20730570	Effect of bone morphogenetic protein 4 in the human brain glioma cell line U251.	GeneRIF	0.0044	65.1	0.0114	0.116	1	1	BMP4
Pubmed	23739325	Severe malaria is associated with parasite binding to endothelial protein C receptor.	GeneRIF	0.0044	65.1	0.0114	0.116	1	1	PROCR
Pubmed	10508519	Mutations in the skeletal muscle alpha-actin gene in patients with actin myopathy and nemaline myopathy.	Pubmed	0.0044	65.1	0.0114	0.116	1	1	ACTA1
Pubmed	19376791	A label-free quantitative proteomics strategy to identify E3 ubiquitin ligase substrates targeted to proteasome degradation.	Pubmed	0.0044	65.1	0.0114	0.116	1	1	ASB2
Pubmed	18593908	Loss of the SSeCKS/Gravin/AKAP12 gene results in prostatic hyperplasia.	GeneRIF	0.0044	65.1	0.0114	0.116	1	1	AKAP12
Pubmed	18307388	Frequencies of promoter pentanucleotide (TTTTA)n of CYP11A gene in European and North African populations.	GeneRIF	0.0044	65.1	0.0114	0.116	1	1	CYP11A1
Pubmed	2302219	Detection of a point mutation in sphingolipid activator protein-1 mRNA in patients with a variant form of metachromatic leukodystrophy.	Pubmed	0.0044	65.1	0.0114	0.116	1	1	PSAP
Pubmed	3357890	Characterization of a thyroid hormone receptor expressed in human kidney and other tissues.	Pubmed	0.0044	65.1	0.0114	0.116	1	1	THRA
Pubmed	19596982	Cutting edge: Limiting MHC class II expression to dendritic cells alters the ability to develop Th2-dependent allergic airway inflammation.	Pubmed	0.0044	65.1	0.0114	0.116	1	1	HLA-DQB1
Pubmed	11175339	Identification of a 428-kb homozygously deleted region disrupting the SEZ6L gene at 22q12.1 in a lung cancer cell line.	Pubmed	0.0044	65.1	0.0114	0.116	1	1	SEZ6L
Pubmed	24166784	X-linked sideroblastic anemia due to ALAS2 intron 1 enhancer element GATA-binding site mutations.	Pubmed	0.0044	65.1	0.0114	0.116	1	1	ALAS2
Pubmed	3758227	Isolation and partial characterization of the human alpha A-crystallin gene.	Pubmed	0.0044	65.1	0.0114	0.116	1	1	CRYAA
Pubmed	16494848	Regulation of matrilysin expression in endothelium by fibroblast growth factor-2.	GeneRIF	0.0044	65.1	0.0114	0.116	1	1	MMP7
Pubmed	25451226	Identification of the WNT1 residues required for palmitoylation by Porcupine.	Pubmed	0.0044	65.1	0.0114	0.116	1	1	PORCN
Pubmed	17120019	Activation of hEAG1 potassium channels by arachidonic acid.	GeneRIF	0.0044	65.1	0.0114	0.116	1	1	KCNH2
Pubmed	12845557	Expression of thyroid hormone receptor isoform alpha1 in pancreatic islets.	GeneRIF	0.0044	65.1	0.0114	0.116	1	1	THRA
Pubmed	24974304	CD74 indicates microglial activation in experimental diabetic retinopathy and exogenous methylglyoxal mimics the response in normoglycemic retina.	Pubmed	0.0044	65.1	0.0114	0.116	1	1	CD74
Pubmed	15846783	Nerve growth factor-mediated collateral sprouting of central sensory axons into deafferentated regions of the dorsal horn is enhanced in the absence of the p75 neurotrophin receptor.	Pubmed	0.0044	65.1	0.0114	0.116	1	1	NGFR
Pubmed	17083122	Mice lacking the p75 receptor fail to acquire a normal complement of taste buds and geniculate ganglion neurons by adulthood.	Pubmed	0.0044	65.1	0.0114	0.116	1	1	NGFR
Pubmed	1921150	Analysis of the cDNA sequence encoding MHC-A beta in tubular epithelium from mouse kidney.	Pubmed	0.0044	65.1	0.0114	0.116	1	1	HLA-DQB1
Pubmed	24936060	Peripheral benzodiazepine receptor/translocator protein global knock-out mice are viable with no effects on steroid hormone biosynthesis.	Pubmed	0.0044	65.1	0.0114	0.116	1	1	TSPO
Pubmed	15964556	Matrix metalloproteinase-7 degrades all insulin-like growth factor binding proteins and facilitates insulin-like growth factor bioavailability.	GeneRIF	0.0044	65.1	0.0114	0.116	1	1	MMP7
Pubmed	20709912	The melanoma-associated transmembrane glycoprotein Gpnmb controls trafficking of cellular debris for degradation and is essential for tissue repair.	GeneRIF	0.0044	65.1	0.0114	0.116	1	1	GPNMB
Pubmed	24401906	The endothelial protein C receptor impairs the antibacterial response in murine pneumococcal pneumonia and sepsis.	Pubmed	0.0044	65.1	0.0114	0.116	1	1	PROCR
Pubmed	15797722	Disruption of the mouse Large gene in the enr and myd mutants results in nerve, muscle, and neuromuscular junction defects.	Pubmed	0.0044	65.1	0.0114	0.116	1	1	LARGE
Pubmed	22052794	A new haplotype in BMP4 implicated in ossification of the posterior longitudinal ligament (OPLL) in a Chinese population.	GeneRIF	0.0044	65.1	0.0114	0.116	1	1	BMP4
Pubmed	18063838	Lung lining fluid glutathione attenuates IL-13-induced asthma.	Pubmed	0.0044	65.1	0.0114	0.116	1	1	GGT1
Pubmed	15727248	Reliable and high-throughput mutation screening for beta-thalassemia by a single-base extension/fluorescence polarization assay.	GeneRIF	0.0044	65.1	0.0114	0.116	1	1	HBB
Pubmed	23000601	Association of serum gamma-glutamyltransferase with arterial stiffness in established coronary artery disease.	GeneRIF	0.0044	65.1	0.0114	0.116	1	1	GGT1
Pubmed	16476739	Binding of active matrilysin to cell surface cholesterol sulfate is essential for its membrane-associated proteolytic action and induction of homotypic cell adhesion.	GeneRIF	0.0044	65.1	0.0114	0.116	1	1	MMP7
Pubmed	11316948	Identification and characterization of novel smoothelin isoforms in vascular smooth muscle.	Pubmed	0.0044	65.1	0.0114	0.116	1	1	SMTN
Pubmed	19401418	Enhanced glucose tolerance by SK4 channel inhibition in pancreatic beta-cells.	GeneRIF	0.0044	65.1	0.0114	0.116	1	1	KCNN4
Pubmed	23185377	Dysferlin-peptides reallocate mutated dysferlin thereby restoring function.	GeneRIF	0.0044	65.1	0.0114	0.116	1	1	DYSF
Pubmed	7506762	Autosomal recessive neuromuscular disorder in a transgenic line of mice.	Pubmed	0.0044	65.1	0.0114	0.116	1	1	LARGE
Pubmed	15276478	Atrial natriuretic peptide modulates the hyperpolarization-activated current (If) in human atrial myocytes.	Pubmed	0.0044	65.1	0.0114	0.116	1	1	NPPA
Pubmed	19041722	Recombinant expression of biologically active murine soluble EPCR.	GeneRIF	0.0044	65.1	0.0114	0.116	1	1	PROCR
Pubmed	19397368	Coexpression of human alpha- and circularly permuted beta-globins yields a hemoglobin with normal R state but modified T state properties.	GeneRIF	0.0044	65.1	0.0114	0.116	1	1	HBB
Pubmed	25057196	Cocaine abuse in humans is not associated with increased microglial activation: an 18-kDa translocator protein positron emission tomography imaging study with [11C]PBR28.	Pubmed	0.0044	65.1	0.0114	0.116	1	1	TSPO

Category	ID	Name	Source	p-value	q-value Bonferroni	q-value FDR B&H	q-value FDR B&Y	Hit Count in Query List	Hit Count in Genome	Hit in Query List
Pubmed	17984371	Natriuretic peptide precursor a gene polymorphisms and risk of blood pressure progression and incident hypertension.	GeneRIF	0.0044	65.1	0.0114	0.116	1	1	NPPA
Pubmed	23321671	Osteoclast-specific cathepsin K deletion stimulates S1P-dependent bone formation.	GeneRIF	0.0044	65.1	0.0114	0.116	1	1	CTSK
Pubmed	9546365	Accelerated methylmercury elimination in gamma-glutamyl transpeptidase-deficient mice.	Pubmed	0.0044	65.1	0.0114	0.116	1	1	GGT1
Pubmed	16087766	Identification of a novel founder mutation in the DYSF gene causing clinical variability in the Spanish population.	GeneRIF	0.0044	65.1	0.0114	0.116	1	1	DYSF
Pubmed	22215634	Matrilysin (MMP-7) inhibition of BMP-7 induced renal tubular branching morphogenesis suggests a role in the pathogenesis of human renal dysplasia.	GeneRIF	0.0044	65.1	0.0114	0.116	1	1	MMP7
Pubmed	12878587	Binding of the thyroid hormone receptor to a negative element in the basal growth hormone promoter is associated with histone acetylation.	GeneRIF	0.0044	65.1	0.0114	0.116	1	1	THRA
Pubmed	9022042	The mouse prosaposin locus: promoter organization.	Pubmed	0.0044	65.1	0.0114	0.116	1	1	PSAP
Pubmed	16640876	[Association of soluble human leukocyte antigen DQB1 with recurrent spontaneous abortion].	GeneRIF	0.0044	65.1	0.0114	0.116	1	1	HLA-DQB1
Pubmed	23746248	[Expression and clinical significance of soluble B7 H3 in sera of patients with primary hepatocellular carcinoma].	GeneRIF	0.0044	65.1	0.0114	0.116	1	1	CD276
Pubmed	18757851	Endothelial protein C receptor polymorphisms and risk of myocardial infarction.	GeneRIF	0.0044	65.1	0.0114	0.116	1	1	PROCR
Pubmed	24593816	Innate DNA sensing is impaired in HIV patients and IFI16 expression correlates with chronic immune activation.	Pubmed	0.0044	65.1	0.0114	0.116	1	1	IFI16
Pubmed	14741399	Functional differences between two DCLK splice variants.	GeneRIF	0.0044	65.1	0.0114	0.116	1	1	DCLK1
Pubmed	18593567	Functional studies on three novel HCNH2 mutations in Taiwan: identification of distinct mechanisms of channel defect and dissociation between glycosylation defect and assembly defect.	GeneRIF	0.0044	65.1	0.0114	0.116	1	1	KCNH2
Pubmed	11823440	Direct measurement of the male recombination fraction in the human beta-globin hot spot.	GeneRIF	0.0044	65.1	0.0114	0.116	1	1	HBB
Pubmed	3346101	Absence of expression of a human endogenous retrovirus is correlated with choriocarcinoma.	Pubmed	0.0044	65.1	0.0114	0.116	1	1	ERV3-1
Pubmed	6324166	cDNA clone for the human invariant gamma chain of class II histocompatibility antigens and its implications for the protein structure.	Pubmed	0.0044	65.1	0.0114	0.116	1	1	CD74
Pubmed	19328785	Myelin regulates immune cell adhesion and motility.	GeneRIF	0.0044	65.1	0.0114	0.116	1	1	RTN4R
Pubmed	23817628	Induction and translocation of tissue transglutaminase isoforms increased phosphorylation in retinoic acid treated erythroleukemia cells.	Pubmed	0.0044	65.1	0.0114	0.116	1	1	TGM2
Pubmed	8587608	The inward rectification mechanism of the HERG cardiac potassium channel.	Pubmed	0.0044	65.1	0.0114	0.116	1	1	KCNH2
Pubmed	19675095	Evolution of duplicated beta-globin genes and the structural basis of hemoglobin isoform differentiation in Mus.	Pubmed	0.0044	65.1	0.0114	0.116	1	1	HBB
Pubmed	8764136	Identification of a novel serine protease-like gene, the expression of which is down-regulated during breast cancer progression.	Pubmed	0.0044	65.1	0.0114	0.116	1	1	KLK10
Pubmed	16723117	Template switching within exons 3 and 4 of KV11.1 (HERG) gives rise to a 5' truncated cDNA.	GeneRIF	0.0044	65.1	0.0114	0.116	1	1	KCNH2
Pubmed	3597771	Molecular basis for nondeletion alpha-thalassemia in American blacks. Alpha 2(116GAG----UAG).	Pubmed	0.0044	65.1	0.0114	0.116	1	1	HBA2
Pubmed	23466867	Molecular mechanism of transglutaminase-2 in corneal epithelial migration and adhesion.	GeneRIF	0.0044	65.1	0.0114	0.116	1	1	TGM2
Pubmed	17951401	Osteoactivin promotes breast cancer metastasis to bone.	GeneRIF	0.0044	65.1	0.0114	0.116	1	1	GPNMB
Pubmed	19218742	Insufficient membrane fusion in dysferlin-deficient muscle fibers after heavy-ion irradiation.	GeneRIF	0.0044	65.1	0.0114	0.116	1	1	DYSF
Pubmed	11960641	Mice deficient in fractalkine are less susceptible to cerebral ischemia-reperfusion injury.	GeneRIF	0.0044	65.1	0.0114	0.116	1	1	CX3CL1
Pubmed	21060055	Genotype at polymorphism rs11200638 and HTRA1 expression level.	GeneRIF	0.0044	65.1	0.0114	0.116	1	1	HTRA1
Pubmed	19158403	L-Thyroxine vs. 3,5,3'-triiodo-L-thyronine and cell proliferation: activation of mitogen-activated protein kinase and phosphatidylinositol 3-kinase.	Pubmed	0.0044	65.1	0.0114	0.116	1	1	THRA
Pubmed	22966088	Angiopoietin-like protein 2 and risk of type 2 diabetes in a general Japanese population: the Hisayama study.	GeneRIF	0.0044	65.1	0.0114	0.116	1	1	ANGPTL2
Pubmed	18427598	Human HtrA1 in the archived eyes with age-related macular degeneration.	GeneRIF	0.0044	65.1	0.0114	0.116	1	1	HTRA1
Pubmed	15248236	Antileukoproteinase: modulation of neutrophil function and therapeutic effects on anti-type II collagen antibody-induced arthritis.	GeneRIF	0.0044	65.1	0.0114	0.116	1	1	SLPI
Pubmed	20222126	Overexpression of translocator protein in inflammatory bowel disease: potential diagnostic and treatment value.	GeneRIF	0.0044	65.1	0.0114	0.116	1	1	TSPO
Pubmed	12621127	Clinical, genetic, and biophysical characterization of a homozygous HERG mutation causing severe neonatal long QT syndrome.	Pubmed	0.0044	65.1	0.0114	0.116	1	1	KCNH2
Pubmed	19910509	Atrial natriuretic peptide reduces vascular leakage and choroidal neovascularization.	Pubmed	0.0044	65.1	0.0114	0.116	1	1	NPPA
Pubmed	14729471	The interferon-inducible IFI16 gene inhibits tube morphogenesis and proliferation of primary, but not HPV16 E6/E7-immortalized human endothelial cells.	GeneRIF	0.0044	65.1	0.0114	0.116	1	1	IFI16
Pubmed	9454638	Sympathetic and sensory axons invade the brains of nerve growth factor transgenic mice in the absence of p75NTR expression.	Pubmed	0.0044	65.1	0.0114	0.116	1	1	NGFR
Pubmed	1826098	Hydrolysis of intact and Cys-Phe-cleaved human atrial natriuretic peptide in vitro by human tissue kallikrein.	Pubmed	0.0044	65.1	0.0114	0.116	1	1	NPPA
Pubmed	19215240	HERG-F463L potassium channels linked to long QT syndrome reduce I(Kr) current by a trafficking-deficient mechanism.	GeneRIF	0.0044	65.1	0.0114	0.116	1	1	KCNH2

Category	ID	Name	Source	p-value	q-value Bonferroni	q-value FDR B&H	q-value FDR B&Y	Hit Count in Query List	Hit Count in Genome	Hit in Query List
Pubmed	15086560	A 4.2 kb upstream region of the human corneodesmosin gene directs site-specific expression in hair follicles and hyperkeratotic epidermis of transgenic mice.	Pubmed	0.0044	65.1	0.0114	0.116	1	1	CDSN
Pubmed	24325597	Chimeric hERG channels containing a tetramerization domain are functional and stable.	Pubmed	0.0044	65.1	0.0114	0.116	1	1	KCNH2
Pubmed	15965951	Subcellular distribution of calcium-sensitive potassium channels (IK1) in migrating cells.	GeneRIF	0.0044	65.1	0.0114	0.116	1	1	KCNN4
Pubmed	12097502	Localization of Nogo-A and Nogo-66 receptor proteins at sites of axon-myelin and synaptic contact.	GeneRIF	0.0044	65.1	0.0114	0.116	1	1	RTN4R
Pubmed	18604550	Soluble endothelial protein C receptor levels in Behçet patients with and without ocular involvement.	GeneRIF	0.0044	65.1	0.0114	0.116	1	1	PROCR
Pubmed	1696926	Identical 3250-bp deletion between two AluI repeats in the ADA genes of unrelated ADA-SCID patients.	Pubmed	0.0044	65.1	0.0114	0.116	1	1	ADA
Pubmed	17301974	Large-scale population genetic analysis for hemoglobinopathies reveals different mutation spectra in central Greece compared to the rest of the country.	GeneRIF	0.0044	65.1	0.0114	0.116	1	1	HBB
Pubmed	24307198	Mechanism of hERG channel block by the psychoactive indole alkaloid ibogaine.	Pubmed	0.0044	65.1	0.0114	0.116	1	1	KCNH2
Pubmed	24417241	Allosteric effects of erythromycin pretreatment on thioridazine block of hERG potassium channels.	Pubmed	0.0044	65.1	0.0114	0.116	1	1	KCNH2
Pubmed	16423057	Role of matrix metalloproteinase-7 in the modulation of a Chlamydia trachomatis infection.	Pubmed	0.0044	65.1	0.0114	0.116	1	1	MMP7
Pubmed	8756078	Nondeletional alpha-thalassemia: first description of alpha Hph alpha and alpha Nco alpha mutations in a Spanish population.	Pubmed	0.0044	65.1	0.0114	0.116	1	1	HBA2
Pubmed	20404051	Association of gamma-glutamyltransferase with incidence of type 2 diabetes in Japan.	GeneRIF	0.0044	65.1	0.0114	0.116	1	1	GGT1
Pubmed	18283336	Caveolin-1-mediated expression and secretion of kallikrein 6 in colon cancer cells.	GeneRIF	0.0044	65.1	0.0114	0.116	1	1	KLK1
Pubmed	22582805	Changes in the level of serum high-temperature requirement A1 (HtrA1) during pregnancy and its relationship to preeclampsia.	GeneRIF	0.0044	65.1	0.0114	0.116	1	1	HTRA1
Pubmed	20332224	Kallikrein-related peptidase 7 promotes multicellular aggregation via the alpha(5)beta(1) integrin pathway and paclitaxel chemoresistance in serous epithelial ovarian carcinoma.	Pubmed	0.0044	65.1	0.0114	0.116	1	1	KLK7
Pubmed	15039315	Salivary secretory leukocyte protease inhibitor and oral candidiasis in human immunodeficiency virus type 1-infected persons.	GeneRIF	0.0044	65.1	0.0114	0.116	1	1	SLPI
Pubmed	15716318	Corneodesmosin polymorphisms in psoriatic arthritis.	Pubmed	0.0044	65.1	0.0114	0.116	1	1	CDSN
Pubmed	15853909	Identification of a novel HLA-DQB1 allele, DQB1*0314, by sequence-based typing in the Korean population.	GeneRIF	0.0044	65.1	0.0114	0.116	1	1	HLA-DQB1
Pubmed	17220280	Impaired adipogenesis caused by a mutated thyroid hormone alpha1 receptor.	GeneRIF	0.0044	65.1	0.0114	0.116	1	1	THRA
Pubmed	18794722	The 22G>A polymorphism in the adenosine deaminase gene impairs catalytic function but does not affect reactive hyperaemia in humans in vivo.	GeneRIF	0.0044	65.1	0.0114	0.116	1	1	ADA
Pubmed	14514721	Protein overload induces fractalkine upregulation in proximal tubular cells through nuclear factor kappaB- and p38 mitogen-activated protein kinase-dependent pathways.	GeneRIF	0.0044	65.1	0.0114	0.116	1	1	CX3CL1
Pubmed	18715564	Serum gamma-glutamyltransferase is not associated to peripheral arterial disease in type 2 diabetic patients: Cross-sectional findings.	GeneRIF	0.0044	65.1	0.0114	0.116	1	1	GGT1
Pubmed	19545895	Placental dysferlin expression is reduced in severe preeclampsia.	GeneRIF	0.0044	65.1	0.0114	0.116	1	1	DYSF
Pubmed	21234558	Quantitative prediction of the arrhythmogenic effects of de novo hERG mutations in computational models of human ventricular tissues.	GeneRIF	0.0044	65.1	0.0114	0.116	1	1	KCNH2
Pubmed	7916636	Molecular cloning and sequence analysis of the monkey and human tissue kallikrein genes.	Pubmed	0.0044	65.1	0.0114	0.116	1	1	KLK1
Pubmed	12820696	High-risk genotype for type 1 diabetes: a new simple microtiter plate-based ELOSA assay.	GeneRIF	0.0044	65.1	0.0114	0.116	1	1	HLA-DQB1
Pubmed	24260365	Fluvastatin interferes with hepatitis C virus replication via microtubule bundling and a doublecortin-like kinase-mediated mechanism.	Pubmed	0.0044	65.1	0.0114	0.116	1	1	DCLK1
Pubmed	18070922	Fibulin-2 is dispensable for mouse development and elastic fiber formation.	GeneRIF	0.0044	65.1	0.0114	0.116	1	1	FBLN2
Pubmed	7731963	Disruption of the adenosine deaminase gene causes hepatocellular impairment and perinatal lethality in mice.	Pubmed	0.0044	65.1	0.0114	0.116	1	1	ADA
Pubmed	19668118	Ala147Thr substitution in translocator protein is associated with adult separation anxiety in patients with depression.	GeneRIF	0.0044	65.1	0.0114	0.116	1	1	TSPO
Pubmed	12358858	Complete characterization of the DQB1 first exon polymorphism.	GeneRIF	0.0044	65.1	0.0114	0.116	1	1	HLA-DQB1
Pubmed	12855405	Abnormal alveolar development associated with elevated adenine nucleosides.	Pubmed	0.0044	65.1	0.0114	0.116	1	1	ADA
Pubmed	17009310	Transglutaminase 2 limits murine peritoneal acute gout-like inflammation by regulating macrophage clearance of apoptotic neutrophils.	GeneRIF	0.0044	65.1	0.0114	0.116	1	1	TGM2
Pubmed	2009	Biochemical and electrophoretic studies of erythrocyte pyridoxine kinase in white and black Americans.	Pubmed	0.0044	65.1	0.0114	0.116	1	1	PDXK
Pubmed	12629213	Sequences in the (A)gamma-delta intergenic region are not required for stage-specific regulation of the human beta-globin gene locus.	GeneRIF	0.0044	65.1	0.0114	0.116	1	1	HBB
Pubmed	11869041	The role of tissue transglutaminase (transglutaminase type II) for the intestinal manifestations of murine semi-allogenic graft-versus-host disease.	GeneRIF	0.0044	65.1	0.0114	0.116	1	1	TGM2
Pubmed	22963604	A haplotype of the SMTN gene associated with myocardial infarction in Japanese women.	GeneRIF	0.0044	65.1	0.0114	0.116	1	1	SMTN
Pubmed	2727382	Lysosomal heterogeneity of dipeptidyl peptidase II active on collagen-related peptides.	Pubmed	0.0044	65.1	0.0114	0.116	1	1	DPP7

Category	ID	Name	Source	p-value	q-value Bonferroni	q-value FDR B&H	q-value FDR B&Y	Hit Count in Query List	Hit Count in Genome	Hit in Query List
Pubmed	16165280	Interneurons transiently express the ERG K+ channels during development of mouse spinal networks in vitro.	Pubmed	0.0044	65.1	0.0114	0.116	1	1	KCNH2
Pubmed	11804203	(AC)n dinucleotide repeat polymorphism in 5' beta globin gene in native and Mestizo Mexican populations.	GeneRIF	0.0044	65.1	0.0114	0.116	1	1	HBB
Pubmed	1726096	Detection of the Hb Quong Sze mutation in a Chinese family by selective amplification of the alpha 2-globin gene and restriction map analysis with Msp I.	Pubmed	0.0044	65.1	0.0114	0.116	1	1	HBA2
Pubmed	10694575	Postexposure vaccination massively increases the prevalence of gamma-herpesvirus-specific CD8+ T cells but confers minimal survival advantage on CD4-deficient mice.	Pubmed	0.0044	65.1	0.0114	0.116	1	1	HLA-DQB1
Pubmed	24876405	p204-initiated innate antiviral response in mouse Leydig cells.	Pubmed	0.0044	65.1	0.0114	0.116	1	1	IFI16
Pubmed	18823996	The 1.8-A crystal structure of alpha1-acid glycoprotein (Orosomucoid) solved by UV RIP reveals the broad drug-binding activity of this human plasma lipocalin.	GeneRIF	0.0044	65.1	0.0114	0.116	1	1	ORM1
Pubmed	12718853	Structure of the Nogo receptor ectodomain: a recognition module implicated in myelin inhibition.	Pubmed	0.0044	65.1	0.0114	0.116	1	1	RTN4R
Pubmed	8755578	Growth retardation and cysteine deficiency in gamma-glutamyl transpeptidase-deficient mice.	Pubmed	0.0044	65.1	0.0114	0.116	1	1	GGT1
Pubmed	14769912	Crystal structure of HLA-DQ0602 that protects against type 1 diabetes and confers strong susceptibility to narcolepsy.	GeneRIF	0.0044	65.1	0.0114	0.116	1	1	HLA-DQB1
Pubmed	17825554	Dysferlinopathy in the Jews of the Caucasus: a frequent mutation in the dysferlin gene.	GeneRIF	0.0044	65.1	0.0114	0.116	1	1	DYSF
Pubmed	25480631	Increase of serum fractalkine and fractalkine gene expression levels in sickle cell disease patients.	Pubmed	0.0044	65.1	0.0114	0.116	1	1	CX3CL1
Pubmed	14570980	Autoimmune cardiomyopathy and heart block develop spontaneously in HLA-DQ8 transgenic IAbeta knockout NOD mice.	GeneRIF	0.0044	65.1	0.0114	0.116	1	1	HLA-DQB1
Pubmed	21029002	The relationship of gamma-glutamyltransferase to aortic elastic properties in young patients with prehypertension.	GeneRIF	0.0044	65.1	0.0114	0.116	1	1	GGT1
Pubmed	22460790	p75 neurotrophin receptor regulates glucose homeostasis and insulin sensitivity.	GeneRIF	0.0044	65.1	0.0114	0.116	1	1	NGFR
Pubmed	20154340	Efficient recovery of dysferlin deficiency by dual adeno-associated vector-mediated gene transfer.	Pubmed	0.0044	65.1	0.0114	0.116	1	1	DYSF
Pubmed	16704963	The genetic basis of human erythrocyte pyridoxal kinase activity variation.	GeneRIF	0.0044	65.1	0.0114	0.116	1	1	PDXK
Pubmed	7733886	Molecular cloning, purification and in situ localization of human colon kallikrein.	Pubmed	0.0044	65.1	0.0114	0.116	1	1	KLK1
Pubmed	1531041	The T cell receptor/CD3 complex is composed of at least two autonomous transduction modules.	Pubmed	0.0044	65.1	0.0114	0.116	1	1	CD74
Pubmed	16127690	Expression pattern of the ether-a-go-go-related (ERG) family proteins in the adult mouse central nervous system: evidence for coassembly of different subunits.	GeneRIF	0.0044	65.1	0.0114	0.116	1	1	KCNH2
Pubmed	9000000	Gravin, an autoantigen recognized by serum from myasthenia gravis patients, is a kinase scaffold protein.	Pubmed	0.0044	65.1	0.0114	0.116	1	1	AKAP12
Pubmed	16368448	Association between human atrial natriuretic peptide Val7Met polymorphism and baseline blood pressure, plasma trough irbesartan concentrations, and the antihypertensive efficacy o irbesartan in rural Chinese patients with essential hypertension.	GeneRIF	0.0044	65.1	0.0114	0.116	1	1	NPPA
Pubmed	17822686	Endothelial lipase enhances low density lipoprotein binding and cell association in THP-1 macrophages.	GeneRIF	0.0044	65.1	0.0114	0.116	1	1	LIPG
Pubmed	22623621	Connexin 31 (GJB3) deficiency in mouse trophoblast stem cells alters giant cell differentiation and leads to loss of oxygen sensing.	GeneRIF	0.0044	65.1	0.0114	0.116	1	1	GJB3
Pubmed	230515	Defect in 3'-phosphoadenosine 5'-phosphosulfate formation in brachymorphic mice.	Pubmed	0.0044	65.1	0.0114	0.116	1	1	PAPSS2
Pubmed	19654002	Hypoxia inhibits maturation and trafficking of hERG K(+) channel protein: Role of Hsp90 and ROS.	GeneRIF	0.0044	65.1	0.0114	0.116	1	1	KCNH2
Pubmed	10399321	Molecular cloning of a novel human PAPS synthetase which is differentially expressed in metastatic and non-metastatic colon carcinoma cells.	Pubmed	0.0044	65.1	0.0114	0.116	1	1	PAPSS2
Pubmed	11985060	[Study of genotype frequencies of ANP 664G/A polymorphism in normal subjects and CVD patients, and its association with plasma ANP levels].	GeneRIF	0.0044	65.1	0.0114	0.116	1	1	NPPA
Pubmed	22715361	Levels of soluble endothelial protein C receptor are associated with CD4+ changes in Maraviroc-treated HIV-infected patients.	Pubmed	0.0044	65.1	0.0114	0.116	1	1	PROCR
Pubmed	21285511	An N-terminal truncated carboxypeptidase E splice isoform induces tumor growth and is a biomarker for predicting future metastasis in human cancers.	GeneRIF	0.0044	65.1	0.0114	0.116	1	1	CPE
Pubmed	3754244	Hb Himeji or beta 140 (H18) Ala----Asp. A slightly unstable hemoglobin with increased beta N-terminal glycation.	Pubmed	0.0044	65.1	0.0114	0.116	1	1	HBB
Pubmed	12833403	X-linked Opitz syndrome: novel mutations in the MID1 gene and redefinition of the clinical spectrum.	GeneRIF	0.0044	65.1	0.0114	0.116	1	1	MID1
Pubmed	8662840	Induced and spontaneous mutations at Ser202 of carboxypeptidase E. Effect on enzyme expression, activity, and intracellular routing.	Pubmed	0.0044	65.1	0.0114	0.116	1	1	CPE
Pubmed	24828664	TGM2: a cell surface marker in esophageal adenocarcinomas.	Pubmed	0.0044	65.1	0.0114	0.116	1	1	TGM2
Pubmed	12356573	HLA-DQB1*0201: a marker for good prognosis in British and Dutch patients with sarcoidosis.	GeneRIF	0.0044	65.1	0.0114	0.116	1	1	HLA-DQB1
Pubmed	16281908	p75NTR independent oligodendrocyte death in cuprizone-induced demyelination in C57BL/6 mice.	GeneRIF	0.0044	65.1	0.0114	0.116	1	1	NGFR

Category	ID	Name	Source	p-value	q-value Bonferroni	q-value FDR B&H	q-value FDR B&Y	Hit Count in Query List	Hit Count in Genome	Hit in Query List
Pubmed	22089883	Expression of transglutaminase-2 isoforms in normal human tissues and cancer cell lines: dysregulation of alternative splicing in cancer.	GeneRIF	0.0044	65.1	0.0114	0.116	1	1	TGM2
Pubmed	9209323	Tissue and differentiation specific expression on the endogenous retrovirus ERV3 (HERV-R) in normal human tissues and during induced monocytic differentiation in the U-937 cell line.	Pubmed	0.0044	65.1	0.0114	0.116	1	1	ERV3-1
Pubmed	21145773	Association of endothelial lipase Thr111Ile polymorphism with lipid metabolism and microvascular complications in type 2 diabetic patients.	GeneRIF	0.0044	65.1	0.0114	0.116	1	1	LIPG
Pubmed	19578768	Angiogenesis in cervical cancer is mediated by HeLa metabolites through endothelial cell tissue kallikrein.	GeneRIF	0.0044	65.1	0.0114	0.116	1	1	KLK1
Pubmed	18560234	Adenosine deaminase genetic polymorphism and coronary artery disease.	GeneRIF	0.0044	65.1	0.0114	0.116	1	1	ADA
Pubmed	24041948	Atrial natriuretic peptide genetic variant rs5065 and risk for cardiovascular disease in the general community: a 9-year follow-up study.	Pubmed	0.0044	65.1	0.0114	0.116	1	1	NPPA
Pubmed	16112212	Suppression of macrophage responses to bacterial lipopolysaccharide (LPS) by secretory leukocyte protease inhibitor (SLPI) is independent of its anti-protease function.	GeneRIF	0.0044	65.1	0.0114	0.116	1	1	SLPI
Pubmed	19430464	Haematopoietic malignancies caused by dysregulation of a chromatin-binding PHD finger.	GeneRIF	0.0044	65.1	0.0114	0.116	1	1	KDM5A
Pubmed	21914860	Mitochondrial T3 receptor p43 regulates insulin secretion and glucose homeostasis.	GeneRIF	0.0044	65.1	0.0114	0.116	1	1	THRA
Pubmed	2881279	Mouse skeletal alpha actin has limited restriction fragment length polymorphism and is not a member of the human 1q-mouse distal chromosome 1 syntatic group.	Pubmed	0.0044	65.1	0.0114	0.116	1	1	ACTA1
Pubmed	17444953	A novel mannose-binding lectin-associated serine protease 1/3 gene variant.	GeneRIF	0.0044	65.1	0.0114	0.116	1	1	MASP1
Pubmed	25092313	Overexpression of junctophilin-2 does not enhance baseline function but attenuates heart failure development after cardiac stress.	Pubmed	0.0044	65.1	0.0114	0.116	1	1	JPH2
Pubmed	22343981	Functional analysis of eight missense mutations in the SPINK1 gene.	GeneRIF	0.0044	65.1	0.0114	0.116	1	1	SPINK1
Pubmed	22999863	Induced expression of B7-H3 on the lung cancer cells and macrophages suppresses T-cell mediating anti-tumor immune response.	GeneRIF	0.0044	65.1	0.0114	0.116	1	1	CD276
Pubmed	1978610	Purification and properties of gamma-glutamyl transpeptidase from human testis.	Pubmed	0.0044	65.1	0.0114	0.116	1	1	GGT1
Pubmed	22297152	Two common mutations (p.Gln832X and c.663+1G>C) account for about a third of the DYSF mutations in Korean patients with dysferlinopathy.	GeneRIF	0.0044	65.1	0.0114	0.116	1	1	DYSF
Pubmed	16251351	Phosphatidylinositol 3-phosphate indirectly activates KCa3.1 via 14 amino acids in the carboxy terminus of KCa3.1.	GeneRIF	0.0044	65.1	0.0114	0.116	1	1	KCNN4
Pubmed	17900621	Mixed oligomer formation between human alphaA crystallin and its cataract-causing G98R mutant: structural, stability and functional differences.	GeneRIF	0.0044	65.1	0.0114	0.116	1	1	CRYAA
Pubmed	9714291	Human ether-a-gogo related gene (HERG) K+ channels as pharmacological targets: present and future implications.	Pubmed	0.0044	65.1	0.0114	0.116	1	1	KCNH2
Pubmed	23630167	Frequent inactivation of cysteine dioxygenase type 1 contributes to survival of breast cancer cells and resistance to anthracyclines.	Pubmed	0.0044	65.1	0.0114	0.116	1	1	CDO1
Pubmed	20068074	Secretory leukocyte protease inhibitor antagonizes paclitaxel in ovarian cancer cells.	GeneRIF	0.0044	65.1	0.0114	0.116	1	1	SLPI
Pubmed	20431028	Tumor progression stage and anatomical site regulate tumor-associated macrophage and bone marrow-derived monocyte polarization.	Pubmed	0.0044	65.1	0.0114	0.116	1	1	CD68
Pubmed	18550819	Carboxypeptidase E mediates palmitate-induced beta-cell ER stress and apoptosis.	GeneRIF	0.0044	65.1	0.0114	0.116	1	1	CPE
Pubmed	23026773	A novel regulatory pathway for autoimmune disease: binding of partial MHC class II constructs to monocytes reduces CD74 expression and induces both specific and bystander T-cell tolerance.	GeneRIF	0.0044	65.1	0.0114	0.116	1	1	CD74
Pubmed	18192489	Ly49 C/I-dependent NKT cell-derived IL-10 is required for corneal graft survival and peripheral tolerance.	Pubmed	0.0044	65.1	0.0114	0.116	1	1	HLA-DQB1
Pubmed	22689013	Human invariant chain isoform p35 restores thymic selection and antigen presentation in CD74-deficient mice.	GeneRIF	0.0044	65.1	0.0114	0.116	1	1	CD74
Pubmed	17587614	Thalassemia intermedia associated with the Hb Constant Spring EE Bart's disease in pregnancy: a molecular and hematological analysis.	GeneRIF	0.0044	65.1	0.0114	0.116	1	1	HBA2
Pubmed	16507768	Human skeletal muscle atrophy in amyotrophic lateral sclerosis reveals a reduction in Akt and an increase in atrogin-1.	GeneRIF	0.0044	65.1	0.0114	0.116	1	1	FBXO32
Pubmed	21355047	Mutant huntingtin causes defective actin remodeling during stress: defining a new role for transglutaminase 2 in neurodegenerative disease.	GeneRIF	0.0044	65.1	0.0114	0.116	1	1	TGM2
Pubmed	9348180	Paradoxical action of activin A on folliculogenesis in immature and adult mice.	Pubmed	0.0044	65.1	0.0114	0.116	1	1	INHBB
Pubmed	22942358	Major histocompatibility complex class II+ invariant chain negative breast cancer cells present unique peptides that activate tumor-specific T cells from breast cancer patients.	GeneRIF	0.0044	65.1	0.0114	0.116	1	1	CD74
Pubmed	23517369	Variable presentation of HB H disease due to homozygosity for the rare polyadenylation signal A T(Indian) (AATAAA>AATA- -) mutation in four Indian families.	Pubmed	0.0044	65.1	0.0114	0.116	1	1	HBA2
Pubmed	11154980	Influence of alpha-thalassemia trait on the prevalence and severity of anemia in pregnancy among women in Kuwait.	GeneRIF	0.0044	65.1	0.0114	0.116	1	1	HBA2
Pubmed	17243920	Increased adenosine deaminase in hydatidiform mole.	GeneRIF	0.0044	65.1	0.0114	0.116	1	1	ADA
Pubmed	11035058	Divergent roles for CD4+ T cells in the priming and effector/memory phases of adoptive immunotherapy.	Pubmed	0.0044	65.1	0.0114	0.116	1	1	HLA-DQB1

Category	ID	Name	Source	p-value	q-value Bonferroni	q-value FDR B&H	q-value FDR B&Y	Hit Count in Query List	Hit Count in Genome	Hit in Query List
Pubmed	20739659	Tissue transglutaminase contributes to the all-trans retinoic acid-induced differentiation syndrome phenotype in the NB4 model of acute promyelocytic leukemia.	GeneRIF	0.0044	65.1	0.0114	0.116	1	1	TGM2
Pubmed	19039956	Chlamydia trachomatis and Chlamydophila abortus induce the expression of secretory leukocyte protease inhibitor in cells of the human female reproductive tract.	GeneRIF	0.0044	65.1	0.0114	0.116	1	1	SLPI
Pubmed	16650820	The expression of inhibin beta B is high in human adipocytes, reduced by weight loss, and correlates to factors implicated in metabolic disease.	GeneRIF	0.0044	65.1	0.0114	0.116	1	1	INHBB
Pubmed	19373586	beta-Thalassemia mutations in the Iranian Kurdish population of Kurdistan and West Azerbaijan provinces.	GeneRIF	0.0044	65.1	0.0114	0.116	1	1	HBB
Pubmed	14744775	herg1 gene and HERG1 protein are overexpressed in colorectal cancers and regulate cell invasion of tumor cells.	GeneRIF	0.0044	65.1	0.0114	0.116	1	1	KCNH2
Pubmed	21112636	The production of secretory leukocyte protease inhibitor by dendritic cells.	GeneRIF	0.0044	65.1	0.0114	0.116	1	1	SLPI
Pubmed	12764110	Delayed systemic Nogo-66 receptor antagonist promotes recovery from spinal cord injury.	GeneRIF	0.0044	65.1	0.0114	0.116	1	1	RTN4R
Pubmed	22038702	Is HbA2 level a reliable diagnostic measurement for ß-thalassemia trait in people with iron deficiency?	GeneRIF	0.0044	65.1	0.0114	0.116	1	1	HBA2
Pubmed	21567078	Up-regulation of Foxo4 mediated by hepatitis B virus X protein confers resistance to oxidative stress-induced cell death.	GeneRIF	0.0044	65.1	0.0114	0.116	1	1	FOXO4
Pubmed	10880769	The identification of novel therapeutic targets for the treatment of malignant brain tumors.	Pubmed	0.0044	65.1	0.0114	0.116	1	1	RPL35A
Pubmed	21496384	Isoenzymes of adenosine deaminase and metalloproteinases as biomarkers in in vitro fertilization and embryo transfer.	GeneRIF	0.0044	65.1	0.0114	0.116	1	1	ADA
Pubmed	15017003	De novo crypt formation and juvenile polyposis on BMP inhibition in mouse intestine.	GeneRIF	0.0044	65.1	0.0114	0.116	1	1	BMP4
Pubmed	12779273	A new unstable alpha2-globin gene variant: Hb Chartres [alpha33(B14)Phe --> Ser].	Pubmed	0.0044	65.1	0.0114	0.116	1	1	HBA2
Pubmed	8707825	Smoothelin, a novel cytoskeletal protein specific for smooth muscle cells.	Pubmed	0.0044	65.1	0.0114	0.116	1	1	SMTN
Pubmed	10048321	Crystal structure of wild-type human procathepsin K.	Pubmed	0.0044	65.1	0.0114	0.116	1	1	CTSK
Pubmed	15782101	Pancreatic secretory trypsin inhibitor (SPINK1) gene mutations in patients with acute pancreatitis.	GeneRIF	0.0044	65.1	0.0114	0.116	1	1	SPINK1
Pubmed	15569046	Altered patterns of the interferon-inducible gene IFI16 expression in head and neck squamous cell carcinoma: immunohistochemical study including correlation with retinoblastoma protein, human papillomavirus infection and proliferation index.	GeneRIF	0.0044	65.1	0.0114	0.116	1	1	IFI16
Pubmed	18385288	Endogenous heparan sulfate and heparin modulate bone morphogenetic protein-4 signaling and activity.	GeneRIF	0.0044	65.1	0.0114	0.116	1	1	BMP4
Pubmed	21777220	A novel mutation in CDSN causes peeling skin disease in a patient from Morocco.	Pubmed	0.0044	65.1	0.0114	0.116	1	1	CDSN
Pubmed	14674747	Solution structure of human saposin C: pH-dependent interaction with phospholipid vesicles.	GeneRIF	0.0044	65.1	0.0114	0.116	1	1	PSAP
Pubmed	15199098	Crosslinking and G-protein functions of transglutaminase 2 contribute differentially to fibroblast wound healing responses.	GeneRIF	0.0044	65.1	0.0114	0.116	1	1	TGM2
Pubmed	20619102	[Expression of endothelial protein C receptor in tumor cell lines and It's significance].	GeneRIF	0.0044	65.1	0.0114	0.116	1	1	PROCR
Pubmed	1925539	Atomic structure of adenosine deaminase complexed with a transition-state analog: understanding catalysis and immunodeficiency mutations.	Pubmed	0.0044	65.1	0.0114	0.116	1	1	ADA
Pubmed	17296897	New phenotype associated with an Arg116Cys mutation in the CRYAA gene: nuclear cataract, iris coloboma, and microphthalmia.	GeneRIF	0.0044	65.1	0.0114	0.116	1	1	CRYAA
Pubmed	15126571	Large-scale analysis of the relationship between CYP11A promoter variation, polycystic ovarian syndrome, and serum testosterone.	GeneRIF	0.0044	65.1	0.0114	0.116	1	1	CYP11A1
Pubmed	20830473	Histopathology of aseptic necrosis of the femoral head in sickle cell disease.	GeneRIF	0.0044	65.1	0.0114	0.116	1	1	HBB
Pubmed	19279140	Context-dependent functional substitution of alpha-skeletal actin by gamma-cytoplasmic actin.	Pubmed	0.0044	65.1	0.0114	0.116	1	1	ACTA1
Pubmed	18191158	Protriptyline block of the human ether-à-go-go-related gene (HERG) K+ channel.	GeneRIF	0.0044	65.1	0.0114	0.116	1	1	KCNH2
Pubmed	11233253	[Analysis of HLA-DQB1 polymorphism by PCR-SSO in Yichu of Yunnan Province].	GeneRIF	0.0044	65.1	0.0114	0.116	1	1	HLA-DQB1
Pubmed	22378763	Transcriptional regulation of translocator protein (Tspo) via a SINE B2-mediated natural antisense transcript in MA-10 Leydig cells.	GeneRIF	0.0044	65.1	0.0114	0.116	1	1	TSPO
Pubmed	22198221	Propofol neurotoxicity is mediated by p75 neurotrophin receptor activation.	GeneRIF	0.0044	65.1	0.0114	0.116	1	1	NGFR
Pubmed	16466947	Novel and Mediterranean beta thalassemia mutations in the indigenous Northern Ireland population.	GeneRIF	0.0044	65.1	0.0114	0.116	1	1	HBB
Pubmed	23940126	An adult female with resistance to thyroid hormone mediated by defective thyroid hormone receptor a.	Pubmed	0.0044	65.1	0.0114	0.116	1	1	THRA
Pubmed	11327387	Autoantibodies detectable in the sera of silicosis patients. The relationship between the anti-topoisomerase I antibody response and HLA-DQB1*0402 allele in Japanese silicosis patients.	GeneRIF	0.0044	65.1	0.0114	0.116	1	1	HLA-DQB1
Pubmed	17402746	The role of Ets transcription factors in the basal transcription of the translocator protein (18 kDa).	GeneRIF	0.0044	65.1	0.0114	0.116	1	1	TSPO
Pubmed	16460673	The p75(NTR) tumor suppressor induces cell cycle arrest facilitating caspase mediated apoptosis in prostate tumor cells.	GeneRIF	0.0044	65.1	0.0114	0.116	1	1	NGFR
Pubmed	12944424	Carboxypeptidase Z (CPZ) modulates Wnt signaling and regulates the development of skeletal elements in the chicken.	Pubmed	0.0044	65.1	0.0114	0.116	1	1	CPZ
Pubmed	25283374	DCLK1 is detectable in plasma of patients with Barrett's esophagus and esophageal adenocarcinoma.	Pubmed	0.0044	65.1	0.0114	0.116	1	1	DCLK1

Category	ID	Name	Source	p-value	q-value Bonferroni	q-value FDR B&H	q-value FDR B&Y	Hit Count in Query List	Hit Count in Genome	Hit in Query List
Pubmed	15883381	The neurotrophin receptor p75NTR modulates long-term depression and regulates the expression of AMPA receptor subunits in the hippocampus.	GeneRIF	0.0044	65.1	0.0114	0.116	1	1	NGFR
Pubmed	16867155	Microfibril-associated protein 4 binds to surfactant protein A (SP-A) and colocalizes with SP-A in the extracellular matrix of the lung.	GeneRIF	0.0044	65.1	0.0114	0.116	1	1	MFAP4
Pubmed	25347256	Hb Youngstown [ß101(G3)Glu¿¿¿Ala; HBB: c.305A > C]: An unstable hemoglobin variant causing severe hemolytic anemia.	Pubmed	0.0044	65.1	0.0114	0.116	1	1	HBB
Pubmed	12609765	Hematopoietic growth factor inducible neurokinin-1 type: a transmembrane protein that is similar to neurokinin 1 interacts with substance P.	Pubmed	0.0044	65.1	0.0114	0.116	1	1	GPNMB
Pubmed	16000639	(+)Insert smooth muscle myosin heavy chain (SM-B) isoform expression in human tissues.	Pubmed	0.0044	65.1	0.0114	0.116	1	1	MYH11
Pubmed	24202302	Inhibition of bone morphogenic protein 4 restores endothelial function in db/db diabetic mice.	Pubmed	0.0044	65.1	0.0114	0.116	1	1	BMP4
Pubmed	1612590	Structure and evolution of the human prosaposin chromosomal gene.	Pubmed	0.0044	65.1	0.0114	0.116	1	1	PSAP
Pubmed	17996867	Cluster specific regulation pattern of upstream regulatory elements in human alpha- and beta-globin gene clusters.	GeneRIF	0.0044	65.1	0.0114	0.116	1	1	HBA2
Pubmed	21182082	CD4+ T-cell immunity to the Burkholderia pseudomallei ABC transporter LolC in melioidosis.	Pubmed	0.0044	65.1	0.0114	0.116	1	1	HLA-DQB1
Pubmed	24098564	The impact of genome-wide supported schizophrenia risk variants in the neurogranin gene on brain structure and function.	Pubmed	0.0044	65.1	0.0114	0.116	1	1	NRGN
Pubmed	21940052	Compartmentalized secretory leukocyte protease inhibitor expression and hormone responses along the reproductive tract of postmenopausal women.	GeneRIF	0.0044	65.1	0.0114	0.116	1	1	SLPI
Pubmed	23473737	An NMR investigation of the structure, function and role of the hERG channel selectivity filter in the long QT syndrome.	GeneRIF	0.0044	65.1	0.0114	0.116	1	1	KCNH2
Pubmed	10508505	Dysferlin deletion in SJL mice (SJL-Dysf) defines a natural model for limb girdle muscular dystrophy 2B.	Pubmed	0.0044	65.1	0.0114	0.116	1	1	DYSF
Pubmed	18443268	The functional properties of a truncated form of endothelial cell protein C receptor generated by alternative splicing.	GeneRIF	0.0044	65.1	0.0114	0.116	1	1	PROCR
Pubmed	21532395	Mouse auditory organ development required bone morphogenetic protein signaling.	GeneRIF	0.0044	65.1	0.0114	0.116	1	1	BMP4
Pubmed	19236779	[Correlation between gene polymorphism of endothelial protein C receptor and cerebral infarction in Chinese Han population of Hubei province].	GeneRIF	0.0044	65.1	0.0114	0.116	1	1	PROCR
Pubmed	2308943	Allelic diversification at the class II DQB locus of the mammalian major histocompatibility complex.	Pubmed	0.0044	65.1	0.0114	0.116	1	1	HLA-DQB1
Pubmed	15741796	Interplay of platelet polymorphisms, risk factors, and von [corrected] Willebrand factor [corrected] in determining collagen-adenosine diphosphate PFA-100 results in patients with coronary artery disease.	GeneRIF	0.0044	65.1	0.0114	0.116	1	1	HBB
Pubmed	21302026	Marked relationship between matrix metalloproteinase 7 and brain atrophy in HIV infection.	GeneRIF	0.0044	65.1	0.0114	0.116	1	1	MMP7
Pubmed	19172215	Ablation of cathepsin k activity in the young mouse causes hypermineralization of long bone and growth plates.	GeneRIF	0.0044	65.1	0.0114	0.116	1	1	CTSK
Pubmed	11027547	The rearrangement of the human alpha(1)-acid glycoprotein/orosomucoid gene: evidence for tandemly triplicated genes consisting of two AGP1 and one AGP2.	Pubmed	0.0044	65.1	0.0114	0.116	1	1	ORM1
Pubmed	21989057	Dysregulation of Rho GTPases in the aPix/Arhgef6 mouse model of X-linked intellectual disability is paralleled by impaired structural and synaptic plasticity and cognitive deficits.	GeneRIF	0.0044	65.1	0.0114	0.116	1	1	ARHGEF6
Pubmed	11707591	Bone morphogenetic protein 4 in the extraembryonic mesoderm is required for allantois development and the localization and survival of primordial germ cells in the mouse.	Pubmed	0.0044	65.1	0.0114	0.116	1	1	BMP4
Pubmed	23799939	A genetic variant of the atrial natriuretic peptide gene is associated with left ventricular hypertrophy in a non-diabetic population--the Malmö preventive project study.	Pubmed	0.0044	65.1	0.0114	0.116	1	1	NPPA
Pubmed	11927634	Role of p75 neurotrophin receptor in the neurotoxicity by beta-amyloid peptides and synergistic effect of inflammatory cytokines.	GeneRIF	0.0044	65.1	0.0114	0.116	1	1	NGFR
Pubmed	23905909	Fasting serum long-acting natriuretic peptide correlates with the ankle brachial index in renal transplant recipients.	Pubmed	0.0044	65.1	0.0114	0.116	1	1	NPPA
Pubmed	20007614	Analysis of matrix metalloproteinase-7 expression in eutopic and ectopic endometrium samples from patients with different forms of endometriosis.	GeneRIF	0.0044	65.1	0.0114	0.116	1	1	MMP7
Pubmed	17470662	Age decline in the activity of the Ca2+-sensitive K+ channel of human red blood cells.	GeneRIF	0.0044	65.1	0.0114	0.116	1	1	KCNN4
Pubmed	19955852	Transglutaminase 2 is involved in autophagosome maturation.	GeneRIF	0.0044	65.1	0.0114	0.116	1	1	TGM2
Pubmed	19596049	The voltage-gated Na+ channel beta3 subunit does not mediate trans homophilic cell adhesion or associate with the cell adhesion molecule contactin.	GeneRIF	0.0044	65.1	0.0114	0.116	1	1	SCN3B
Pubmed	25202021	Transglutaminase II/microRNA-218/-181a loop regulates positive feedback relationship between allergic inflammation and tumor metastasis.	Pubmed	0.0044	65.1	0.0114	0.116	1	1	TGM2
Pubmed	11939518	Dominant beta-thalassemia due to a newly identified frameshift mutation in exon 3 (codon 113, GTG-->Tg).	Pubmed	0.0044	65.1	0.0114	0.116	1	1	HBB
Pubmed	7374785	Evidence for mutation in an I-A gene.	Pubmed	0.0044	65.1	0.0114	0.116	1	1	HLA-DQB1
Pubmed	7798325	A potential role for tetranectin in mineralization during osteogenesis.	Pubmed	0.0044	65.1	0.0114	0.116	1	1	CLEC3B

Category	ID	Name	Source	p-value	q-value Bonferroni	q-value FDR B&H	q-value FDR B&Y	Hit Count in Query List	Hit Count in Genome	Hit in Query List
Pubmed	17160997	Severe beta(0) thalassemia/hemoglobin E disease caused by de novo 22-base pair duplication in the paternal allele of beta globin gene.	GeneRIF	0.0044	65.1	0.0114	0.116	1	1	HBB
Pubmed	11742196	Germline mutations in TGM2 do not contribute to coeliac disease susceptibility in the Swedish population.	GeneRIF	0.0044	65.1	0.0114	0.116	1	1	TGM2
Pubmed	19375943	HTRA1 promoter polymorphism and risk of age-related macular degeneration: a meta-analysis.	GeneRIF	0.0044	65.1	0.0114	0.116	1	1	HTRA1
Pubmed	21935437	Molecular determinants of interactions between the N-terminal domain and the transmembrane core that modulate hERG K+ channel gating.	GeneRIF	0.0044	65.1	0.0114	0.116	1	1	KCNH2
Pubmed	21309041	Sideroblastic anemia: molecular analysis of the ALAS2 gene in a series of 29 probands and functional studies of 10 missense mutations.	GeneRIF	0.0044	65.1	0.0114	0.116	1	1	ALAS2
Pubmed	24291354	A possible role of transglutaminase 2 in the nucleus of INS-1E and of cells of human pancreatic islets.	Pubmed	0.0044	65.1	0.0114	0.116	1	1	TGM2
Pubmed	21349832	Structural insights into differences in drug-binding selectivity between two forms of human alpha1-acid glycoprotein genetic variants, the A and F1*S forms.	GeneRIF	0.0044	65.1	0.0114	0.116	1	1	ORM1
Pubmed	18697831	P-Rex1 - a multidomain protein that regulates neurite differentiation.	GeneRIF	0.0044	65.1	0.0114	0.116	1	1	PREX1
Pubmed	10958697	MyoD-dependent induction during myoblast differentiation of p204, a protein also inducible by interferon.	Pubmed	0.0044	65.1	0.0114	0.116	1	1	IFI16
Pubmed	18425362	Secretory leukoprotease inhibitor inhibits cell growth through apoptotic pathway on ovarian cancer.	GeneRIF	0.0044	65.1	0.0114	0.116	1	1	SLPI
Pubmed	17897792	AP-1 stimulates the cathepsin K promoter in RAW 264.7 cells.	GeneRIF	0.0044	65.1	0.0114	0.116	1	1	CTSK
Pubmed	21441900	Role and predictive strength of transglutaminase type 2 expression in premalignant lesions of the cervix.	GeneRIF	0.0044	65.1	0.0114	0.116	1	1	TGM2
Pubmed	23689398	A novel mutation in two families with pycnodysostosis.	Pubmed	0.0044	65.1	0.0114	0.116	1	1	CTSK
Pubmed	19301206	The susceptibility to experimental autoimmune encephalomyelitis is not related to dysferlin-deficiency.	GeneRIF	0.0044	65.1	0.0114	0.116	1	1	DYSF
Pubmed	24936057	ZIP2 protein, a zinc transporter, is associated with keratinocyte differentiation.	Pubmed	0.0044	65.1	0.0114	0.116	1	1	SLC39A2
Pubmed	20547769	Redox regulation of transglutaminase 2 activity.	GeneRIF	0.0044	65.1	0.0114	0.116	1	1	TGM2
Pubmed	11416151	Functional interference between thyroid hormone receptor alpha (TRalpha) and natural truncated TRDeltaalpha isoforms in the control of intestine development.	Pubmed	0.0044	65.1	0.0114	0.116	1	1	THRA
Pubmed	24067108	CD68 tumor-associated macrophage marker is not prognostic of clinical outcome in classical Hodgkin lymphoma.	Pubmed	0.0044	65.1	0.0114	0.116	1	1	CD68
Pubmed	23791568	Complex rearrangement of the exon 6 genomic region among Opitz G/BBB Syndrome MID1 alterations.	Pubmed	0.0044	65.1	0.0114	0.116	1	1	MID1
Pubmed	20530577	The p75NTR intracellular domain generated by neurotrophin-induced receptor cleavage potentiates Trk signaling.	GeneRIF	0.0044	65.1	0.0114	0.116	1	1	NGFR
Pubmed	20110462	Transgenic expression of pancreatic secretory trypsin inhibitor-1 rescues SPINK3-deficient mice and restores a normal pancreatic phenotype.	GeneRIF	0.0044	65.1	0.0114	0.116	1	1	SPINK1
Pubmed	1412415	Molecular analysis of novel HLA-DR2.DQwl haplotypes in Asian Indians.	Pubmed	0.0044	65.1	0.0114	0.116	1	1	HLA-DQB1
Pubmed	17666595	Symptoms associated with menopausal transition and reproductive hormones in midlife women.	GeneRIF	0.0044	65.1	0.0114	0.116	1	1	INHBB
Pubmed	25417860	Angiopoietin-like-2: a multifaceted protein with physiological and pathophysiological properties.	Pubmed	0.0044	65.1	0.0114	0.116	1	1	ANGPTL2
Pubmed	15826870	Differential turnover of cortical and trabecular bone in transgenic mice overexpressing cathepsin K.	GeneRIF	0.0044	65.1	0.0114	0.116	1	1	CTSK
Pubmed	16678175	CLIP-region mediated interaction of Invariant chain with MHC class I molecules.	GeneRIF	0.0044	65.1	0.0114	0.116	1	1	CD74
Pubmed	12191993	Transient state kinetic investigation of 5-aminolevulinate synthase reaction mechanism.	GeneRIF	0.0044	65.1	0.0114	0.116	1	1	ALAS2
Pubmed	11997174	Steroidogenic enzyme gene expression in the human brain.	Pubmed	0.0044	65.1	0.0114	0.116	1	1	CYP11A1
Pubmed	4944483	Haemoglobin Constant Spring--a chain termination mutant?	Pubmed	0.0044	65.1	0.0114	0.116	1	1	HBA2
Pubmed	8301666	Atrial natriuretic factor blocks the high-threshold Ca2+ current and increases K+ current in fetal single ventricular cells.	Pubmed	0.0044	65.1	0.0114	0.116	1	1	NPPA
Pubmed	9888422	Immunolocalization and gene expression of matrilysin during corneal wound healing.	Pubmed	0.0044	65.1	0.0114	0.116	1	1	MMP7
Pubmed	6629824	Hemoglobin Maputo: a new beta-chain variant (alpha 2 beta 2 47 (CD6) Asp replaced by Tyr) in combination with hemoglobin S, identified by high performance liquid chromatography (HPLC).	Pubmed	0.0044	65.1	0.0114	0.116	1	1	HBB
Pubmed	18800027	Urinary fractalkine is a marker of acute rejection.	GeneRIF	0.0044	65.1	0.0114	0.116	1	1	CX3CL1
Pubmed	1691151	Expression of the gene coding for human prostate-specific antigen and related hGK-1 in benign and malignant tumors of the human prostate.	Pubmed	0.0044	65.1	0.0114	0.116	1	1	KLK1
Pubmed	22434102	Association between HLA-DQB1 gene and patients with acute lymphoblastic leukemia (ALL).	GeneRIF	0.0044	65.1	0.0114	0.116	1	1	HLA-DQB1
Pubmed	12920025	Developmental stage-specific epigenetic control of human beta-globin gene expression is potentiated in hematopoietic progenitor cells prior to their transcriptional activation.	GeneRIF	0.0044	65.1	0.0114	0.116	1	1	HBB
Pubmed	24837365	Matrix metalloproteinase 7 restrains Helicobacter pylori-induced gastric inflammation and premalignant lesions in the stomach by altering macrophage polarization.	Pubmed	0.0044	65.1	0.0114	0.116	1	1	MMP7
Pubmed	9348219	Beta-cell lines derived from transgenic Cpe(fat)/Cpe(fat) mice are defective in carboxypeptidase E and proinsulin processing.	Pubmed	0.0044	65.1	0.0114	0.116	1	1	CPE

Category	ID	Name	Source	p-value	q-value Bonferroni	q-value FDR B&H	q-value FDR B&Y	Hit Count in Query List	Hit Count in Genome	Hit in Query List
Pubmed	23261412	Contribution of putative genetic factors and candidate gene variants to inter-individual variation of circulating fractalkine (CX3CL1) levels in a large UK twins' sample.	GeneRIF	0.0044	65.1	0.0114	0.116	1	1	CX3CL1
Pubmed	19503744	An alphaA-crystallin gene mutation, Arg12Cys, causing inherited cataract-microcornea exhibits an altered heat-shock response.	GeneRIF	0.0044	65.1	0.0114	0.116	1	1	CRYAA
Pubmed	23524483	High hERG1 expression in advanced melanoma.	Pubmed	0.0044	65.1	0.0114	0.116	1	1	KCNH2
Pubmed	2399911	Hemoglobin Dhonburi alpha 2 beta 2 126 (H4) Val----Gly: a new unstable beta variant producing a beta-thalassemia intermedia phenotype in association with beta zero-thalassemia.	Pubmed	0.0044	65.1	0.0114	0.116	1	1	HBB
Pubmed	19923419	Extensive mononuclear infiltration and myogenesis characterize recovery of dysferlin-null skeletal muscle from contraction-induced injuries.	GeneRIF	0.0044	65.1	0.0114	0.116	1	1	DYSF
Pubmed	11570724	A novel splice acceptor site mutation of the alpha2-globin gene causing alpha-thalassemia.	Pubmed	0.0044	65.1	0.0114	0.116	1	1	HBA2
Pubmed	22761798	Down-regulation of HtrA1 activates the epithelial-mesenchymal transition and ATM DNA damage response pathways.	GeneRIF	0.0044	65.1	0.0114	0.116	1	1	HTRA1
Pubmed	18023162	Endothelium specific matrilysin (MMP-7) expression in human cancers.	GeneRIF	0.0044	65.1	0.0114	0.116	1	1	MMP7
Pubmed	23268413	[Enhancement of kallikrein-related peptidase 10 expression attenuates proliferation and invasiveness of human tongue cancer cells in vitro].	GeneRIF	0.0044	65.1	0.0114	0.116	1	1	KLK10
Pubmed	18648013	Common MMP-7 polymorphisms and breast cancer susceptibility: a multistage study of association and functionality.	GeneRIF	0.0044	65.1	0.0114	0.116	1	1	MMP7
Pubmed	20018890	SSeCKS/Gravin/AKAP12 inhibits cancer cell invasiveness and chemotaxis by suppressing a protein kinase C- Raf/MEK/ERK pathway.	GeneRIF	0.0044	65.1	0.0114	0.116	1	1	AKAP12
Pubmed	21610753	Assessing the pathological relevance of SPINK1 promoter variants.	GeneRIF	0.0044	65.1	0.0114	0.116	1	1	SPINK1
Pubmed	21350874	Serum adenosine deaminase activity in patients with systemic lupus erythematosus: a study based on ADA1 and ADA2 isoenzymes pattern.	GeneRIF	0.0044	65.1	0.0114	0.116	1	1	ADA
Pubmed	10227420	Experimental autoimmune encephalomyelitis induced in B6.C-H-2bm12 mice by myelin oligodendrocyte glycoprotein: effect of MHC class II mutation on immunodominant epitope selection and fine epitope specificity of encephalitogenic T cells.	Pubmed	0.0044	65.1	0.0114	0.116	1	1	HLA-DQB1
Pubmed	7839143	Genetic decreases in atrial natriuretic peptide and salt-sensitive hypertension.	Pubmed	0.0044	65.1	0.0114	0.116	1	1	NPPA
Pubmed	22856365	The effect of the neurogranin schizophrenia risk variant rs12807809 on brain structure and function.	GeneRIF	0.0044	65.1	0.0114	0.116	1	1	NRGN
Pubmed	17706674	Endothelial Bmp4 is induced during arterial remodeling: effects on smooth muscle cell migration and proliferation.	GeneRIF	0.0044	65.1	0.0114	0.116	1	1	BMP4
Pubmed	15300636	[The associations of HLA-DQB1 gene with onset age and autoantibodies in type 1 diabetes].	GeneRIF	0.0044	65.1	0.0114	0.116	1	1	HLA-DQB1
Pubmed	23881209	Endothelial protein C receptor polymorphisms and risk of severe sepsis in critically ill patients.	Pubmed	0.0044	65.1	0.0114	0.116	1	1	PROCR
Pubmed	23121329	Haplotype of smoothelin gene associated with essential hypertension.	Pubmed	0.0044	65.1	0.0114	0.116	1	1	SMTN
Pubmed	11238527	Luteinizing hormone receptor, steroidogenesis acute regulatory protein, and steroidogenic enzyme messenger ribonucleic acids are overexpressed in thecal and granulosa cells from polycystic ovaries.	Pubmed	0.0044	65.1	0.0114	0.116	1	1	CYP11A1
Pubmed	11169248	Identification of a new DQB1 allele that appears to have been generated by an interallelic sequence exchange.	Pubmed	0.0044	65.1	0.0114	0.116	1	1	HLA-DQB1
Pubmed	18976909	Genotype-phenotype correlations in ACTA1 mutations that cause congenital myopathies.	GeneRIF	0.0044	65.1	0.0114	0.116	1	1	ACTA1
Pubmed	21170050	Mapping the sequence of conformational changes underlying selectivity filter gating in the K(v)11.1 potassium channel.	GeneRIF	0.0044	65.1	0.0114	0.116	1	1	KCNH2
Pubmed	23899883	BMP-4 polymorphisms in the susceptibility of cervical spondylotic myelopathy and its outcome after anterior cervical corpectomy and fusion.	Pubmed	0.0044	65.1	0.0114	0.116	1	1	BMP4
Pubmed	19949665	Inhibition of the human ether-a-go-go-related gene (HERG) K+ channels by Lindera erythrocarpa.	GeneRIF	0.0044	65.1	0.0114	0.116	1	1	KCNH2
Pubmed	14722925	Two novel severe mutations in the pancreatic secretory trypsin inhibitor gene (SPINK1) cause familial and/or hereditary pancreatitis.	GeneRIF	0.0044	65.1	0.0114	0.116	1	1	SPINK1
Pubmed	23912490	Overexpression of Aldo-keto reductase family 1 B10 protein in ductal carcinoma in situ of the breast correlates with HER2 positivity.	Pubmed	0.0044	65.1	0.0114	0.116	1	1	AKR1B10
Pubmed	20035626	Expression of the costimulatory molecule B7-H3 is associated with prolonged survival in human pancreatic cancer.	GeneRIF	0.0044	65.1	0.0114	0.116	1	1	CD276
Pubmed	12821676	Osmotic swelling induces p75 neurotrophin receptor (p75NTR) expression via nitric oxide.	GeneRIF	0.0044	65.1	0.0114	0.116	1	1	NGFR
Pubmed	12185203	Human kallikrein 10 expression in normal tissues by immunohistochemistry.	Pubmed	0.0044	65.1	0.0114	0.116	1	1	KLK10
Pubmed	12488357	Trafficking of mutant carboxypeptidase E to secretory granules in a beta-cell line derived from Cpe(fat)/Cpe(fat) mice.	Pubmed	0.0044	65.1	0.0114	0.116	1	1	CPE
Pubmed	24997812	Association between EHD3 gene and the cognitive function of patients with major depressive disorder.	Pubmed	0.0044	65.1	0.0114	0.116	1	1	EHD3
Pubmed	22921425	Transglutaminase activity regulates atherosclerotic plaque composition at locations exposed to oscillatory shear stress.	GeneRIF	0.0044	65.1	0.0114	0.116	1	1	TGM2
Pubmed	11701663	Cardiac steroidogenesis in the normal and failing heart.	Pubmed	0.0044	65.1	0.0114	0.116	1	1	CYP11A1

Category	ID	Name	Source	p-value	q-value Bonferroni	q-value FDR B&H	q-value FDR B&Y	Hit Count in Query List	Hit Count in Genome	Hit in Query List
Pubmed	24487939	Association between the atrial natriuretic peptide rs5065 gene polymorphism and the presence and severity of coronary artery disease in an Iranian population.	Pubmed	0.0044	65.1	0.0114	0.116	1	1	NPPA
Pubmed	20114049	Cooperation between the thyroid hormone receptor TRalpha1 and the WNT pathway in the induction of intestinal tumorigenesis.	GeneRIF	0.0044	65.1	0.0114	0.116	1	1	THRA
Pubmed	24055799	Up-regulation of KCa3.1 promotes human airway smooth muscle cell phenotypic modulation.	Pubmed	0.0044	65.1	0.0114	0.116	1	1	KCNN4
Pubmed	10392451	Gamma-glutamyl transpeptidase gene organization and expression: a comparative analysis in rat, mouse, pig and human species.	Pubmed	0.0044	65.1	0.0114	0.116	1	1	GGT1
Pubmed	1326278	The human "peripheral-type" benzodiazepine receptor: regional mapping of the gene and characterization of the receptor expressed from cDNA.	Pubmed	0.0044	65.1	0.0114	0.116	1	1	TSPO
Pubmed	19586929	Phenotype and genotype in 17 patients with Goltz-Gorlin syndrome.	GeneRIF	0.0044	65.1	0.0114	0.116	1	1	PORCN
Pubmed	9344681	Localization of the gene for a serine protease with IGF-binding domain (PRSS11) to human chromosome 10q25.3-q26.2.	Pubmed	0.0044	65.1	0.0114	0.116	1	1	HTRA1
Pubmed	11212211	Tissue distribution and functional expression of the human voltage-gated sodium channel beta3 subunit.	Pubmed	0.0044	65.1	0.0114	0.116	1	1	SCN3B
Pubmed	15184894	LARGE can functionally bypass alpha-dystroglycan glycosylation defects in distinct congenital muscular dystrophies.	Pubmed	0.0044	65.1	0.0114	0.116	1	1	LARGE
Pubmed	15767753	Molecular heterogeneity of beta-thalassemia in the United Arab Emirates.	GeneRIF	0.0044	65.1	0.0114	0.116	1	1	HBB
Pubmed	9237107	Both invariant chain isoforms Ii31 and Ii41 promote class II antigen presentation.	Pubmed	0.0044	65.1	0.0114	0.116	1	1	CD74
Pubmed	18570185	Absence of carboxypeptidase E leads to adult hippocampal neuronal degeneration and memory deficits.	GeneRIF	0.0044	65.1	0.0114	0.116	1	1	CPE
Pubmed	11010966	Molecular cloning of the human kallikrein 15 gene (KLK15). Up-regulation in prostate cancer.	Pubmed	0.0044	65.1	0.0114	0.116	1	1	KLK15
Pubmed	25666037	Differences in Kallikrein-related peptidase mRNA sequences and KLK6 enzyme activity between mouse strains.	Pubmed	0.0044	65.1	0.0114	0.116	1	1	KLK15
Pubmed	18174247	The transglutaminase 2 gene (TGM2), a potential molecular marker for chemotherapeutic drug sensitivity, is epigenetically silenced in breast cancer.	GeneRIF	0.0044	65.1	0.0114	0.116	1	1	TGM2
Pubmed	18443145	DNA replication timing of the human beta-globin domain is controlled by histone modification at the origin.	GeneRIF	0.0044	65.1	0.0114	0.116	1	1	HBB
Pubmed	24673969	Sleep-disordered breathing is associated with elevated human atrial natriuretic peptide levels in a Japanese urban population: the Suita study.	Pubmed	0.0044	65.1	0.0114	0.116	1	1	NPPA
Pubmed	24325651	High-titre circulating tissue transglutaminase-2 antibodies predict small bowel villous atrophy, but decision cut-off limits must be locally validated.	Pubmed	0.0044	65.1	0.0114	0.116	1	1	TGM2
Pubmed	23519124	Secreted calmodulin-like skin protein inhibits neuronal death in cell-based Alzheimer's disease models via the heterotrimeric Humanin receptor.	GeneRIF	0.0044	65.1	0.0114	0.116	1	1	CALML5
Pubmed	22771765	Mouse TSPO in a lipid environment interacting with a functionalized monolayer.	GeneRIF	0.0044	65.1	0.0114	0.116	1	1	TSPO
Pubmed	23747913	Electroporation as a strategy to promote HtrA1 gene uptake and chemotherapy efficacy in a mouse model of mesothelioma.	Pubmed	0.0044	65.1	0.0114	0.116	1	1	HTRA1
Pubmed	17568390	Functional analysis of pancreatitis-associated missense mutations in the pancreatic secretory trypsin inhibitor (SPINK1) gene.	GeneRIF	0.0044	65.1	0.0114	0.116	1	1	SPINK1
Pubmed	17937925	Recessive congenital total cataract with microcornea and heterozygote carrier signs caused by a novel missense CRYAA mutation (R54C).	GeneRIF	0.0044	65.1	0.0114	0.116	1	1	CRYAA
Pubmed	17135237	An insight into the mechanism of human cysteine dioxygenase. Key roles of the thioether-bonded tyrosine-cysteine cofactor.	Pubmed	0.0044	65.1	0.0114	0.116	1	1	CDO1
Pubmed	25775533	Atrial natriuretic peptide prevents cancer metastasis through vascular endothelial cells.	Pubmed	0.0044	65.1	0.0114	0.116	1	1	NPPA
Pubmed	25170811	The adenosine deaminase gene polymorphism is associated with chronic heart failure risk in Chinese.	Pubmed	0.0044	65.1	0.0114	0.116	1	1	ADA
Pubmed	21666108	IRAG and novel PKG targeting in the cardiovascular system.	Pubmed	0.0044	65.1	0.0114	0.116	1	1	MRVI1
Pubmed	2842863	Coding of two sphingolipid activator proteins (SAP-1 and SAP-2) by same genetic locus.	Pubmed	0.0044	65.1	0.0114	0.116	1	1	PSAP
Pubmed	22988594	hERG K(+) channels: structure, function, and clinical significance.	GeneRIF	0.0044	65.1	0.0114	0.116	1	1	KCNH2
Pubmed	19720020	Transfer of rolf S3-S4 linker to HERG eliminates activation gating but spares inactivation.	GeneRIF	0.0044	65.1	0.0114	0.116	1	1	KCNH2
Pubmed	12055102	TIMAP, a novel CAAX box protein regulated by TGF-beta1 and expressed in endothelial cells.	GeneRIF	0.0044	65.1	0.0114	0.116	1	1	PPP1R16B
Pubmed	7794273	Primary substrate specificity of recombinant human stratum corneum chymotryptic enzyme.	Pubmed	0.0044	65.1	0.0114	0.116	1	1	KLK7
Pubmed	22595807	Atrial natriuretic peptide: a possible mediator involved in dexamethasone's inhibition of cell proliferation in multiple myeloma.	GeneRIF	0.0044	65.1	0.0114	0.116	1	1	NPPA
Pubmed	23586372	Silent ß-thalassemia mutations at -101 (C>T) and -71 (C>T) and their coinheritance with the sickle cell mutation in Bahrain.	Pubmed	0.0044	65.1	0.0114	0.116	1	1	HBB
Pubmed	20872971	Prospective study of MMP7 serum levels in the diagnosis of cholangiocarcinoma.	GeneRIF	0.0044	65.1	0.0114	0.116	1	1	MMP7
Pubmed	11590192	Effects of intracellular zinc depletion on metallothionein and ZIP2 transporter expression and apoptosis.	Pubmed	0.0044	65.1	0.0114	0.116	1	1	SLC39A2
Pubmed	9367898	Transcriptional regulation of the mBMP-4 gene through an E-box in the 5'-flanking promoter region involving USF.	Pubmed	0.0044	65.1	0.0114	0.116	1	1	BMP4
Pubmed	16581794	Thyroid hormone receptor alpha1 directly controls transcription of the beta-catenin gene in intestinal epithelial cells.	GeneRIF	0.0044	65.1	0.0114	0.116	1	1	THRA

Category	ID	Name	Source	p-value	q-value Bonferroni	q-value FDR B&H	q-value FDR B&Y	Hit Count in Query List	Hit Count in Genome	Hit in Query List
Pubmed	20424634	Two binding sites for [3H]PBR28 in human brain: implications for TSPO PET imaging of neuroinflammation.	GeneRIF	0.0044	65.1	0.0114	0.116	1	1	TSPO
Pubmed	22698685	Tissue transglutaminase overexpression does not modify the disease phenotype of the R6/2 mouse model of Huntington's disease.	GeneRIF	0.0044	65.1	0.0114	0.116	1	1	TGM2
Pubmed	21186333	Association of serum bone morphogenetic protein 4 levels with obesity and metabolic syndrome in non-diabetic individuals.	GeneRIF	0.0044	65.1	0.0114	0.116	1	1	BMP4
Pubmed	24923741	BMP4 promotes SSEA-1(+) hUC-MSC differentiation into male germ-like cells in vitro.	Pubmed	0.0044	65.1	0.0114	0.116	1	1	BMP4
Pubmed	20056821	Effects of bone morphogenetic protein (BMP) on adrenocorticotropin production by pituitary corticotrope cells: involvement of up-regulation of BMP receptor signaling by somatostatin analogs.	GeneRIF	0.0044	65.1	0.0114	0.116	1	1	BMP4
Pubmed	21907835	TIMAP protects endothelial barrier from LPS-induced vascular leakage and is down-regulated by LPS.	GeneRIF	0.0044	65.1	0.0114	0.116	1	1	PPP1R16B
Pubmed	20486775	Bone morphogenetic protein 4 mediates human embryonic germ cell derivation.	GeneRIF	0.0044	65.1	0.0114	0.116	1	1	BMP4
Pubmed	19598279	Overexpression of Zip-2 mRNA in the leukocytes of asthmatic infants.	Pubmed	0.0044	65.1	0.0114	0.116	1	1	SLC39A2
Pubmed	23369704	Circulating cathepsin K as a potential novel biomarker of coronary artery disease.	Pubmed	0.0044	65.1	0.0114	0.116	1	1	CTSK
Pubmed	18084248	TROP2: a novel prognostic marker in squamous cell carcinoma of the oral cavity.	GeneRIF	0.0044	65.1	0.0114	0.116	1	1	TACSTD2
Pubmed	1917537	A new unstable and low oxygen affinity hemoglobin variant: Hb Stanmore [beta 111(G13)Val----Ala].	Pubmed	0.0044	65.1	0.0114	0.116	1	1	HBB
Pubmed	3132422	Localization of the HLA class II-associated invariant chain gene to human chromosome band 5q32.	Pubmed	0.0044	65.1	0.0114	0.116	1	1	CD74
Pubmed	24628083	Lack of transglutaminase 2 diminished T-cell responses in mice.	Pubmed	0.0044	65.1	0.0114	0.116	1	1	TGM2
Pubmed	12144064	Double heterozygosity for Hb Pyrgos [beta83(EF7)Gly-->Asp] and Hb E [beta26(B8)Glu-->Lys] found in association with alpha-thalassemia.	Pubmed	0.0044	65.1	0.0114	0.116	1	1	HBB
Pubmed	15086562	Homo-oligomerization of human corneodesmosin is mediated by its N-terminal glycine loop domain.	GeneRIF	0.0044	65.1	0.0114	0.116	1	1	CDSN
Pubmed	23975544	AKR1B10 is induced by hyperglycaemia and lipopolysaccharide in patients with diabetic nephropathy.	Pubmed	0.0044	65.1	0.0114	0.116	1	1	AKR1B10
Pubmed	3020711	Assignment of human nerve growth factor receptor gene to chromosome 17 and regulation of receptor expression in somatic cell hybrids.	Pubmed	0.0044	65.1	0.0114	0.116	1	1	NGFR
Pubmed	17145820	Expression of matrix metalloproteinase-7 on cancer cells and tissue endothelial cells in renal cell carcinoma: prognostic implications and clinical significance for invasion and metastasis.	GeneRIF	0.0044	65.1	0.0114	0.116	1	1	MMP7
Pubmed	15054879	Microsatellite polymorphism of steroid hormone synthesis gene CYP11A1 is associated with advanced prostate cancer.	GeneRIF	0.0044	65.1	0.0114	0.116	1	1	CYP11A1
Pubmed	18325919	Human tissue kallikrein 7, a novel biomarker for advanced ovarian carcinoma using a novel in situ quantitative method of protein expression.	GeneRIF	0.0044	65.1	0.0114	0.116	1	1	KLK7
Pubmed	24265122	Osteoactivin induces transdifferentiation of C2C12 myoblasts into osteoblasts.	Pubmed	0.0044	65.1	0.0114	0.116	1	1	GPNMB
Pubmed	9021161	Localization of the beta-nerve growth factor gene (NGFB) to bovine chromosome 3.	Pubmed	0.0044	65.1	0.0114	0.116	1	1	PSAP
Pubmed	465367	Oxidative damage to neutrophils in glutathione synthetase deficiency.	Pubmed	0.0044	65.1	0.0114	0.116	1	1	GSS
Pubmed	10878663	Cathepsin K gene mutations and 1q21 haplotypes in at patients with pycnodysostosis in an outbred population.	Pubmed	0.0044	65.1	0.0114	0.116	1	1	CTSK
Pubmed	22220471	Structure and regulation of type 2 transglutaminase in relation to its physiological functions and pathological roles.	GeneRIF	0.0044	65.1	0.0114	0.116	1	1	TGM2
Pubmed	8452534	Demonstration of adenosine deaminase activity in human fibroblast lysosomes.	Pubmed	0.0044	65.1	0.0114	0.116	1	1	ADA
Pubmed	2022497	A rare HLA DQB allele sequenced from patients with systemic lupus erythematosus.	Pubmed	0.0044	65.1	0.0114	0.116	1	1	HLA-DQB1
Pubmed	17785623	Bone morphogenic protein antagonists are coexpressed with bone morphogenic protein 4 in endothelial cells exposed to unstable flow in vitro in mouse aortas and in human coronary arteries: role of bone morphogenic protein antagonists in inflammation and atherosclerosis.	GeneRIF	0.0044	65.1	0.0114	0.116	1	1	BMP4
Pubmed	20353353	Compound heterozygous Hb Tak/Hb E causes secondary erythrocytosis in a Thai family.	GeneRIF	0.0044	65.1	0.0114	0.116	1	1	HBB
Pubmed	10406956	Mouse fibulin-2 gene. Complete exon-intron organization and promoter characterization.	Pubmed	0.0044	65.1	0.0114	0.116	1	1	FBLN2
Pubmed	17721311	A missense mutation in the M1S1 gene found in a turkish patient with gelatinous droplike corneal dystrophy.	GeneRIF	0.0044	65.1	0.0114	0.116	1	1	TACSTD2
Pubmed	2207678	The effect of antimitotic agents on the intraneuronal distribution of lysosomes.	Pubmed	0.0044	65.1	0.0114	0.116	1	1	DPP7
Pubmed	22132073	Association between Prostinogen (KLK15) genetic variants and prostate cancer risk and aggressiveness in Australia and a meta-analysis of GWAS data.	GeneRIF	0.0044	65.1	0.0114	0.116	1	1	KLK15
Pubmed	15020232	Mitosis increases levels of secretory leukocyte protease inhibitor in keratinocytes.	GeneRIF	0.0044	65.1	0.0114	0.116	1	1	SLPI
Pubmed	21496174	Patient with syncope and LQTS carrying a mutation in the PAS domain of the hERG1 channel.	GeneRIF	0.0044	65.1	0.0114	0.116	1	1	KCNH2
Pubmed	3462719	Isolation, properties, and complete amino acid sequence of human secretory leukocyte protease inhibitor, a potent inhibitor of leukocyte elastase.	Pubmed	0.0044	65.1	0.0114	0.116	1	1	SLPI
Pubmed	23918145	Modulation of p75(NTR) prevents diabetes- and proNGF-induced retinal inflammation and blood-retina barrier breakdown in mice and rats.	Pubmed	0.0044	65.1	0.0114	0.116	1	1	NGFR

Category	ID	Name	Source	p-value	q-value Bonferroni	q-value FDR B&H	q-value FDR B&Y	Hit Count in Query List	Hit Count in Genome	Hit in Query List
Pubmed	25418316	Voltage sensor gating charge transfer in a hERG potassium channel model.	Pubmed	0.0044	65.1	0.0114	0.116	1	1	KCNH2
Pubmed	20099768	Estimated incidence of sickle-cell disease in Aruba and St. Maarten suggests cost-effectiveness of a universal screening programme for St. Maarten.	GeneRIF	0.0044	65.1	0.0114	0.116	1	1	HBB
Pubmed	12638124	mPPP1R16B is a novel mouse protein phosphatase 1 targeting subunit whose mRNA is located in cell bodies and dendrites of neurons in four distinct regions of the brain.	GeneRIF	0.0044	65.1	0.0114	0.116	1	1	PPP1R16B
Pubmed	8245130	Structure and expression of fibulin-2, a novel extracellular matrix protein with multiple EGF-like repeats and consensus motifs for calcium binding.	Pubmed	0.0044	65.1	0.0114	0.116	1	1	FBLN2
Pubmed	15808842	Impaired Ca2+ handling and contraction in cardiomyocytes from mice with a dominant negative thyroid hormone receptor alpha1.	GeneRIF	0.0044	65.1	0.0114	0.116	1	1	THRA
Pubmed	15312129	British Society for Matrix Biology meeting. Manchester, 5-6 april 2004. Abstracts.	Pubmed	0.0044	65.1	0.0114	0.116	1	1	DPT
Pubmed	25448018	Specificity studies on Kallikrein-related peptidase 7 (KLK7) and effects of osmolytes and glycosaminoglycans on its peptidase activity.	Pubmed	0.0044	65.1	0.0114	0.116	1	1	KLK7
Pubmed	17096035	[Evaluation of coverage by a neonatal screening program for hemoglobinopathies in the Recôncavo region of Bahia, Brazil].	GeneRIF	0.0044	65.1	0.0114	0.116	1	1	HBB
Pubmed	16783473	HLA-DQB1 genotypes, islet antibodies and beta cell function in the classification of recent-onset diabetes among young adults in the nationwide Diabetes Incidence Study in Sweden.	GeneRIF	0.0044	65.1	0.0114	0.116	1	1	HLA-DQB1
Pubmed	12906861	Duplication of primate and rodent B7-H3 immunoglobulin V- and C-like domains: divergent history of functional redundancy and exon loss.	Pubmed	0.0044	65.1	0.0114	0.116	1	1	CD276
Pubmed	19026999	Adenosine deaminase in patients with primary immunodeficiency syndromes: the analysis of serum ADA1 and ADA2 activities.	GeneRIF	0.0044	65.1	0.0114	0.116	1	1	ADA
Pubmed	15638380	Macrophage/histiocytic antigen CD68 expression in neoplastic and reactive lymph nodes.	GeneRIF	0.0044	65.1	0.0114	0.116	1	1	CD68
Pubmed	23696273	Focal dermal hypoplasia (Goltz-Gorlin syndrome): a new case with a novel variant in the PORCN gene (c.1250T>C:p.F417S) and unusual spinal anomaly.	Pubmed	0.0044	65.1	0.0114	0.116	1	1	PORCN
Pubmed	11484084	Prevalence of coeliac disease in siblings of patients with Type I diabetes is related to the prevalence of DQB1*02 allele.	GeneRIF	0.0044	65.1	0.0114	0.116	1	1	HLA-DQB1
Pubmed	20096949	Serum MMP-7 is increased in diabetic renal disease and diabetic diastolic dysfunction.	GeneRIF	0.0044	65.1	0.0114	0.116	1	1	MMP7
Pubmed	22968319	A genetic polymorphism for translocator protein 18 kDa affects both in vitro and in vivo radioligand binding in human brain to this putative biomarker of neuroinflammation.	GeneRIF	0.0044	65.1	0.0114	0.116	1	1	TSPO
Pubmed	15743835	The exon 8-containing prosaposin gene splice variant is dispensable for mouse development, lysosomal function, and secretion.	GeneRIF	0.0044	65.1	0.0114	0.116	1	1	PSAP
Pubmed	8660701	A single mouse glutathione synthetase gene encodes six mRNAs with different 5' ends.	Pubmed	0.0044	65.1	0.0114	0.116	1	1	GSS
Pubmed	21303860	Mouse models of dominant ACTA1 disease recapitulate human disease and provide insight into therapies.	Pubmed	0.0044	65.1	0.0114	0.116	1	1	ACTA1
Pubmed	20206312	New roles and mechanism of action of BMP4 in postnatal tooth cytodifferentiation.	GeneRIF	0.0044	65.1	0.0114	0.116	1	1	BMP4
Pubmed	24198334	Nuclear interferon-inducible protein 16 promotes silencing of herpesviral and transfected DNA.	Pubmed	0.0044	65.1	0.0114	0.116	1	1	IFI16
Pubmed	668922	Hemoglobin Presbyterian: beta108 (G10) asparagine leads to lysine, A hemoglobin variant with low oxygen affinity.	Pubmed	0.0044	65.1	0.0114	0.116	1	1	HBB
Pubmed	1846035	Nerve growth factor binding domain of the nerve growth factor receptor.	Pubmed	0.0044	65.1	0.0114	0.116	1	1	NGFR
Pubmed	14714110	Clinical and electrophysiological characterization of a novel mutation R863X in HERG C-terminus associated with long QT syndrome.	GeneRIF	0.0044	65.1	0.0114	0.116	1	1	KCNH2
Pubmed	16741156	Flow-dependent remodeling of small arteries in mice deficient for tissue-type transglutaminase: possible compensation by macrophage-derived factor XIII.	Pubmed	0.0044	65.1	0.0114	0.116	1	1	TGM2
Pubmed	15673289	Exogenous BMP-4 amplifies asymmetric ureteric branching in the developing mouse kidney in vitro.	GeneRIF	0.0044	65.1	0.0114	0.116	1	1	BMP4
Pubmed	16146727	Role of protein modifications mediated by transglutaminase 2 in human viral diseases.	GeneRIF	0.0044	65.1	0.0114	0.116	1	1	TGM2
Pubmed	12208132	Prosaposin: threshold rescue and analysis of the "neuritogenic" region in transgenic mice.	Pubmed	0.0044	65.1	0.0114	0.116	1	1	PSAP
Pubmed	1917530	A new abnormal variant, Hb Yahata or beta 112(G14)Cys----Tyr, found in a Japanese: structural confirmation by DNA sequencing of the beta-globin gene.	Pubmed	0.0044	65.1	0.0114	0.116	1	1	HBB
Pubmed	14668215	Expression and role of the ether-à-go-go-related (MERG1A) potassium-channel protein during preimplantation mouse development.	GeneRIF	0.0044	65.1	0.0114	0.116	1	1	KCNH2
Pubmed	20691621	Hemoglobin H disease due to a de novo mutation at the a2-globin gene and an inherited common a-thalassemia deletion found in a Chinese boy.	GeneRIF	0.0044	65.1	0.0114	0.116	1	1	HBA2
Pubmed	17554258	Advanced glycation end products decrease mesangial cell MMP-7: a role in matrix accumulation in diabetic nephropathy?	GeneRIF	0.0044	65.1	0.0114	0.116	1	1	MMP7
Pubmed	11756414	The R116C mutation in alpha A-crystallin diminishes its protective ability against stress-induced lens epithelial cell apoptosis.	GeneRIF	0.0044	65.1	0.0114	0.116	1	1	CRYAA
Pubmed	15468953	The expression of src-suppressed C kinase substrate (SSeCKS) and uptake of exogenous particles in endothelial and reticular cells.	GeneRIF	0.0044	65.1	0.0114	0.116	1	1	AKAP12
Pubmed	17981541	Role of IFI16 in DNA damage and checkpoint.	GeneRIF	0.0044	65.1	0.0114	0.116	1	1	IFI16
Pubmed	12829173	A common polymorphism in KCNH2 (HERG) hastens cardiac repolarization.	GeneRIF	0.0044	65.1	0.0114	0.116	1	1	KCNH2

Category	ID	Name	Source	p-value	q-value Bonferroni	q-value FDR B&H	q-value FDR B&Y	Hit Count in Query List	Hit Count in Genome	Hit in Query List
Pubmed	16981266	Association of rare SPINK1 gene mutation with another base substitution in chronic pancreatitis patients.	GeneRIF	0.0044	65.1	0.0114	0.116	1	1	SPINK1
Pubmed	23843146	SPINK1 expression is tightly linked to 6q15- and 5q21-deleted ERG-fusion negative prostate cancers but unrelated to PSA recurrence.	Pubmed	0.0044	65.1	0.0114	0.116	1	1	SPINK1
Pubmed	20576937	T-tubule remodeling during transition from hypertrophy to heart failure.	Pubmed	0.0044	65.1	0.0114	0.116	1	1	JPH2
Pubmed	16024047	Background and gender effects on survival in the TgN(SOD1-G93A)1Gur mouse model of ALS.	Pubmed	0.0044	65.1	0.0114	0.116	1	1	DYSF
Pubmed	23002075	Hypoxia-induced endothelial CX3CL1 triggers lung smooth muscle cell phenotypic switching and proliferative expansion.	GeneRIF	0.0044	65.1	0.0114	0.116	1	1	CX3CL1
Pubmed	12231242	Nerve growth factor alters p75 neurotrophin receptor-induced effects in mouse facial motoneurons following axotomy.	GeneRIF	0.0044	65.1	0.0114	0.116	1	1	NGFR
Pubmed	16956587	Absence of the basilar pons in mice lacking a functional Large glycosyltransferase gene suggests a defect in pontine neuron migration.	Pubmed	0.0044	65.1	0.0114	0.116	1	1	LARGE
Pubmed	18461170	5'HS5 of the human beta-globin locus control region is dispensable for the formation of the beta-globin active chromatin hub.	GeneRIF	0.0044	65.1	0.0114	0.116	1	1	HBB
Pubmed	24493799	Monovalent engagement of the BCR activates ovalbumin-specific transnuclear B cells.	Pubmed	0.0044	65.1	0.0114	0.116	1	1	HLA-DQB1
Pubmed	12808021	Stimulation of MMP-7 (matrilysin) by Helicobacter pylori in human gastric epithelial cells: role in epithelial cell migration.	GeneRIF	0.0044	65.1	0.0114	0.116	1	1	MMP7
Pubmed	24523531	Coronin 6 regulates acetylcholine receptor clustering through modulating receptor anchorage to actin cytoskeleton.	Pubmed	0.0044	65.1	0.0114	0.116	1	1	CORO6
Pubmed	11791875	Hb Gerland [alpha55(E4)Val-->Ala (alpha2)]: a new neutral alpha chain variant involving the alpha2 gene.	Pubmed	0.0044	65.1	0.0114	0.116	1	1	HBA2
Pubmed	21814043	The p75NTR extracellular domain: a potential molecule regulating the solubility and removal of amyloid-ß.	GeneRIF	0.0044	65.1	0.0114	0.116	1	1	NGFR
Pubmed	22170235	Immunohistochemical location of the p75 neurotrophin receptor (p75NTR) in oral leukoplakia and oral squamous cell carcinoma.	GeneRIF	0.0044	65.1	0.0114	0.116	1	1	NGFR
Pubmed	22570378	Properties of subependymal cerebrospinal fluid contacting neurones in the dorsal vagal complex of the mouse brainstem.	Pubmed	0.0044	65.1	0.0114	0.116	1	1	PKD2L1
Pubmed	21164259	P450 side-chain cleavage deficiency--a rare cause of congenital adrenal hyperplasia.	GeneRIF	0.0044	65.1	0.0114	0.116	1	1	CYP11A1
Pubmed	20397631	Spectroscopic and computational characterization of substrate-bound mouse cysteine dioxygenase: nature of the ferrous and ferric cysteine adducts and mechanistic implications.	GeneRIF	0.0044	65.1	0.0114	0.116	1	1	CDO1
Pubmed	1540659	Structure of the EF corner favors deamidation of asparaginyl residues in hemoglobin: the example of Hb La Roche-sur-Yon [beta 81 (EF5) Leu----His].	Pubmed	0.0044	65.1	0.0114	0.116	1	1	HBB
Pubmed	12865307	Identification of a putative autocrine bone morphogenetic protein-signaling pathway in human ovarian surface epithelium and ovarian cancer cells.	GeneRIF	0.0044	65.1	0.0114	0.116	1	1	BMP4
Pubmed	9843411	Crystal structure of the S-nitroso form of liganded human hemoglobin.	Pubmed	0.0044	65.1	0.0114	0.116	1	1	HBB
Pubmed	20056603	Elevated expression of Paneth cell CRS4C in ileitis-prone SAMP1/YitFc mice: regional distribution, subcellular localization, and mechanism of action.	Pubmed	0.0044	65.1	0.0114	0.116	1	1	MMP7
Pubmed	20605794	Cholesterol sulfate alters substrate preference of matrix metalloproteinase-7 and promotes degradations of pericellular laminin-332 and fibronectin.	GeneRIF	0.0044	65.1	0.0114	0.116	1	1	MMP7
Pubmed	10187819	Regulation of forestomach-specific expression of the murine adenosine deaminase gene.	Pubmed	0.0044	65.1	0.0114	0.116	1	1	ADA
Pubmed	23613869	Combined MRI and ^{31}P-MRS investigations of the ACTA1(H40Y) mouse model of nemaline myopathy show impaired muscle function and altered energy metabolism.	Pubmed	0.0044	65.1	0.0114	0.116	1	1	ACTA1
Pubmed	17376219	Varicocele sclerotherapy improves serum inhibin B levels and seminal parameters.	GeneRIF	0.0044	65.1	0.0114	0.116	1	1	INHBB
Pubmed	16980590	Endothelial lipase is associated with inflammation in humans.	GeneRIF	0.0044	65.1	0.0114	0.116	1	1	LIPG
Pubmed	11884394	Alternative splice variants of doublecortin-like kinase are differentially expressed and have different kinase activities.	GeneRIF	0.0044	65.1	0.0114	0.116	1	1	DCLK1
Pubmed	20443131	Expression of CD74 in high grade gliomas: a potential role in temozolomide resistance.	GeneRIF	0.0044	65.1	0.0114	0.116	1	1	CD74
Pubmed	22952909	Adenosine deaminase polymorphism affects sleep EEG spectral power in a large epidemiological sample.	GeneRIF	0.0044	65.1	0.0114	0.116	1	1	ADA
Pubmed	23941696	Identification of a specific one amino acid change in recombinant human transglutaminase 2 that regulates its activity and calcium sensitivity.	Pubmed	0.0044	65.1	0.0114	0.116	1	1	TGM2
Pubmed	20353347	Extended molecular spectrum of beta- and alpha-thalassemia in Oman.	GeneRIF	0.0044	65.1	0.0114	0.116	1	1	HBB
Pubmed	18697196	Clinical and molecular studies of patients with characteristics of Opitz G/BBB syndrome shows a novel MID1 mutation.	GeneRIF	0.0044	65.1	0.0114	0.116	1	1	MID1
Pubmed	21935707	Transglutaminase 2 interaction with small heat shock proteins mediate cell survival upon excitotoxic stress.	GeneRIF	0.0044	65.1	0.0114	0.116	1	1	TGM2
Pubmed	22749579	Immunolocalization of DCAMKL-1, a putative intestinal stem cell marker, in normal colonic tissue.	GeneRIF	0.0044	65.1	0.0114	0.116	1	1	DCLK1
Pubmed	7604285	HERG, a human inward rectifier in the voltage-gated potassium channel family.	Pubmed	0.0044	65.1	0.0114	0.116	1	1	KCNH2
Pubmed	8146865	Characterization of a novel DQB1 (DQB1*0609) allele by PCR amplification with sequence-specific primers (PCR-SSP) and nucleotide sequencing.	Pubmed	0.0044	65.1	0.0114	0.116	1	1	HLA-DQB1
Pubmed	21901120	The redox state of transglutaminase 2 controls arterial remodeling.	GeneRIF	0.0044	65.1	0.0114	0.116	1	1	TGM2

Category	ID	Name	Source	p-value	q-value Bonferroni	q-value FDR B&H	q-value FDR B&Y	Hit Count in Query List	Hit Count in Genome	Hit in Query List
Pubmed	16519950	Deficient p75 low-affinity neurotrophin receptor expression does alter the composition of cellular infiltrate in experimental autoimmune encephalomyelitis in C57BL/6 mice.	Pubmed	0.0044	65.1	0.0114	0.116	1	1	NGFR
Pubmed	2770711	Ecto-enzyme activity of human erythrocyte adenosine deaminase.	Pubmed	0.0044	65.1	0.0114	0.116	1	1	ADA
Pubmed	15966860	A TrkA-to-p75NTR molecular switch activates amyloid beta-peptide generation during aging.	GeneRIF	0.0044	65.1	0.0114	0.116	1	1	NGFR
Pubmed	23589888	SK4 Ca2+ activated K+ channel is a critical player in cardiac pacemaker derived from human embryonic stem cells.	GeneRIF	0.0044	65.1	0.0114	0.116	1	1	KCNN4
Pubmed	11186256	Beta-thalassemia mutations and haplotype analysis in Lebanon.	GeneRIF	0.0044	65.1	0.0114	0.116	1	1	HBB
Pubmed	3458521	High resolution chromosome analysis of constitutional and acquired t(15;17) maps c-erbA to subband 17q11.2.	Pubmed	0.0044	65.1	0.0114	0.116	1	1	THRA
Pubmed	22194042	Tissue-specific responses to loss of transglutaminase 2.	GeneRIF	0.0044	65.1	0.0114	0.116	1	1	TGM2
Pubmed	2079431	Hb Sun Prairie or alpha(2)130(H13)Ala----Pro beta 2; second observation in an Indian adult.	Pubmed	0.0044	65.1	0.0114	0.116	1	1	HBA2
Pubmed	21734186	Implication of microRNAs in atrial natriuretic peptide and nitric oxide signaling in vascular smooth muscle cells.	GeneRIF	0.0044	65.1	0.0114	0.116	1	1	NPPA
Pubmed	18056715	Nitrite reductase activity of hemoglobin S (sickle) provides insight into contributions of heme redox potential versus ligand affinity.	GeneRIF	0.0044	65.1	0.0114	0.116	1	1	HBB
Pubmed	20375921	GPNMB expression in uveal melanoma: a potential for targeted therapy.	GeneRIF	0.0044	65.1	0.0114	0.116	1	1	GPNMB
Pubmed	10200571	Mapping and sequencing of the murine 'tissue' transglutaminase (Tgm2) gene: absence of mutations in MRLlpr/lpr mice.	Pubmed	0.0044	65.1	0.0114	0.116	1	1	TGM2
Pubmed	8566789	Identification of a murine homolog of the human adenosine deaminase thymic enhancer.	Pubmed	0.0044	65.1	0.0114	0.116	1	1	ADA
Pubmed	17253944	Bone morphogenetic protein 4 contributes to the maintenance of primitive cord blood hematopoietic progenitors in an ex vivo stroma-noncontact co-culture system.	GeneRIF	0.0044	65.1	0.0114	0.116	1	1	BMP4
Pubmed	3635530	N-terminal amino acid sequence of human urinary prokallikrein.	Pubmed	0.0044	65.1	0.0114	0.116	1	1	KLK1
Pubmed	11776299	Prevalence of a 23bp insertion in exon 3 of the endothelial cell protein C receptor gene in venous thrombophilia.	GeneRIF	0.0044	65.1	0.0114	0.116	1	1	PROCR
Pubmed	2349106	Nucleotide sequence of the murine thyroid hormone receptor (alpha-1) cDNA.	Pubmed	0.0044	65.1	0.0114	0.116	1	1	THRA
Pubmed	9415050	Carboxypeptidase E and obesity in the mouse.	Pubmed	0.0044	65.1	0.0114	0.116	1	1	CPE
Pubmed	10450861	Clinical, biochemical, and molecular characterization of patients with glutathione synthetase deficiency.	Pubmed	0.0044	65.1	0.0114	0.116	1	1	GSS
Pubmed	15607699	3' haplotypes of the beta-globin gene in beta(S)-chromosomes of Mexican individuals.	GeneRIF	0.0044	65.1	0.0114	0.116	1	1	HBB
Pubmed	12126634	Coexpression of susceptible and resistant HLA class II transgenes in murine experimental autoimmune thyroiditis: DQ8 molecules downregulate DR3-mediated thyroiditis.	Pubmed	0.0044	65.1	0.0114	0.116	1	1	HLA-DQB1
Pubmed	14688214	Immunohistochemical assessment of the peripheral benzodiazepine receptor in human tissues.	Pubmed	0.0044	65.1	0.0114	0.116	1	1	TSPO
Pubmed	19907127	Regional expression of prosaposin in the wild-type and saposin D-deficient mouse brain detected by an anti-mouse prosaposin-specific antibody.	GeneRIF	0.0044	65.1	0.0114	0.116	1	1	PSAP
Pubmed	10965129	Structure and chromosome location of Smtn, the mouse smoothelin gene.	Pubmed	0.0044	65.1	0.0114	0.116	1	1	SMTN
Pubmed	23045690	Critical period for acoustic preference in mice.	Pubmed	0.0044	65.1	0.0114	0.116	1	1	RTN4R
Pubmed	19244476	State-dependent block of HERG potassium channels by R-roscovitine: implications for cancer therapy.	GeneRIF	0.0044	65.1	0.0114	0.116	1	1	KCNH2
Pubmed	8894685	Full genetic rescue of adenosine deaminase-deficient mice through introduction of the human gene.	Pubmed	0.0044	65.1	0.0114	0.116	1	1	ADA
Pubmed	893142	Hemoglobin Mizuho or beta 68 (E 12) leucine leads to proline, a new unstable variant associated with severe hemolytic anemia.	Pubmed	0.0044	65.1	0.0114	0.116	1	1	HBB
Pubmed	24816726	Trop2 gene: a novel target for cervical cancer treatment.	Pubmed	0.0044	65.1	0.0114	0.116	1	1	TACSTD2
Pubmed	11833656	Predominant neuronal B-cell loss in L5 DRG of p75 receptor-deficient mice.	Pubmed	0.0044	65.1	0.0114	0.116	1	1	NGFR
Pubmed	22355577	CD74 deficiency ameliorates Pseudomonas aeruginosa-induced ocular infection.	Pubmed	0.0044	65.1	0.0114	0.116	1	1	CD74
Pubmed	22526274	Do genetic variants in the SPINK1 gene affect the level of serum PSTI?	GeneRIF	0.0044	65.1	0.0114	0.116	1	1	SPINK1
Pubmed	21843890	Comparisons of inhibin B versus antimüllerian hormone in poor ovarian responders undergoing in vitro fertilization.	GeneRIF	0.0044	65.1	0.0114	0.116	1	1	INHBB
Pubmed	17438108	HLA-DQB1*02-restricted HPV-16 E7 peptide-specific CD4+ T-cell immune responses correlate with regression of HPV-16-associated high-grade squamous intraepithelial lesions.	GeneRIF	0.0044	65.1	0.0114	0.116	1	1	HLA-DQB1
Pubmed	12421833	Unexpected down-regulation of the hIK1 Ca2+-activated K+ channel by its opener 1-ethyl-2-benzimidazolinone in HaCaT keratinocytes. Inverse effects on cell growth and proliferation.	GeneRIF	0.0044	65.1	0.0114	0.116	1	1	KCNN4
Pubmed	12161514	Compound heterozygous mutations in the cholesterol side-chain cleavage enzyme gene (CYP11A) cause congenital adrenal insufficiency in humans.	Pubmed	0.0044	65.1	0.0114	0.116	1	1	CYP11A1
Pubmed	10384121	BALB/c invariant chain mutant mice display relatively efficient maturation of CD4+ T cells in the periphery and secondary proliferative responses elicited upon peptide challenge.	Pubmed	0.0044	65.1	0.0114	0.116	1	1	CD74
Pubmed	10049358	Bmp4 is required for the generation of primordial germ cells in the mouse embryo.	Pubmed	0.0044	65.1	0.0114	0.116	1	1	BMP4
Pubmed	16556651	Effect of S5P alpha-helix charge mutants on inactivation of hERG K+ channels.	GeneRIF	0.0044	65.1	0.0114	0.116	1	1	KCNH2
Pubmed	25278057	In vivo fluorescence reflectance imaging of protease activity in a mouse model of post-traumatic osteoarthritis.	Pubmed	0.0044	65.1	0.0114	0.116	1	1	CTSK

Category	ID	Name	Source	p-value	q-value Bonferroni	q-value FDR B&H	q-value FDR B&Y	Hit Count in Query List	Hit Count in Genome	Hit in Query List
Pubmed	12190955	Differential regulation of atrial and brain natriuretic peptides and its implications for the management of left ventricular volume overload.	Pubmed	0.0044	65.1	0.0114	0.116	1	1	NPPA
Pubmed	22833548	Gene therapy/bone marrow transplantation in ADA-deficient mice: roles of enzyme-replacement therapy and cytoreduction.	Pubmed	0.0044	65.1	0.0114	0.116	1	1	ADA
Pubmed	20155713	Human kallikrein 14 (KLK14) expression in salivary gland tumors.	GeneRIF	0.0044	65.1	0.0114	0.116	1	1	KLK14
Pubmed	22140512	Congenital cataract causing mutants of aA-crystallin/sHSP form aggregates and aggresomes degraded through ubiquitin-proteasome pathway.	GeneRIF	0.0044	65.1	0.0114	0.116	1	1	CRYAA
Pubmed	11337747	Characterization of ARHGEF6, a guanine nucleotide exchange factor for Rho GTPases and a candidate gene for X-linked mental retardation: mutation screening in Börjeson-Forssman-Lehmann syndrome and MRX27.	Pubmed	0.0044	65.1	0.0114	0.116	1	1	ARHGEF6
Pubmed	19760322	Characterization of the dwg mutations: dwg and dwg(Bayer) are new mutant alleles of the Ggt1 gene.	GeneRIF	0.0044	65.1	0.0114	0.116	1	1	GGT1
Pubmed	12955718	Rapid detection of beta-globin gene (HBB) mutations coupling heteroduplex and primer-extension analysis by DHPLC.	GeneRIF	0.0044	65.1	0.0114	0.116	1	1	HBB
Pubmed	7818555	Molecular cloning of human cDNA for cathepsin K: novel cysteine proteinase predominantly expressed in bone.	Pubmed	0.0044	65.1	0.0114	0.116	1	1	CTSK
Pubmed	24080366	The regulatory role of the adrenergic agonists phenylephrine and isoproterenol on fetal hemoglobin expression and erythroid differentiation.	Pubmed	0.0044	65.1	0.0114	0.116	1	1	HBB
Pubmed	21992268	Single turnover of substrate-bound ferric cysteine dioxygenase with superoxide anion: enzymatic reactivation, product formation, and a transient intermediate.	GeneRIF	0.0044	65.1	0.0114	0.116	1	1	CDO1
Pubmed	25010402	Glycoprotein nonmetastatic melanoma protein B (GPNMB) as a novel neuroprotective factor in cerebral ischemia-reperfusion injury.	Pubmed	0.0044	65.1	0.0114	0.116	1	1	GPNMB
Pubmed	24475283	The extracellular domain of neurotrophin receptor p75 as a candidate biomarker for amyotrophic lateral sclerosis.	Pubmed	0.0044	65.1	0.0114	0.116	1	1	NGFR
Pubmed	6629822	Hemoglobin Quin-Hai, beta 78 (EF2) Leu replaced by Arg, a new abnormal hemoglobin found in Guangdong, China.	Pubmed	0.0044	65.1	0.0114	0.116	1	1	HBB
Pubmed	15557198	Inhibition of fractalkine ameliorates murine collagen-induced arthritis.	GeneRIF	0.0044	65.1	0.0114	0.116	1	1	CX3CL1
Pubmed	18924235	BMP4 is required in the anterior heart field and its derivatives for endocardial cushion remodeling, outflow tract septation, and semilunar valve development.	GeneRIF	0.0044	65.1	0.0114	0.116	1	1	BMP4
Pubmed	12104097	Relationship between plasma malondialdehyde levels and adenosine deaminase activities in preeclampsia.	GeneRIF	0.0044	65.1	0.0114	0.116	1	1	ADA
Pubmed	22272574	Influence of MHCIITA rs3087456 and rs4774 polymorphisms in the susceptibility to cardiovascular disease of patients with rheumatoid arthritis.	GeneRIF	0.0044	65.1	0.0114	0.116	1	1	HLA-DQB1
Pubmed	2886427	Structural and functional variability among DQ beta alleles of DR2 subtypes.	Pubmed	0.0044	65.1	0.0114	0.116	1	1	HLA-DQB1
Pubmed	24477458	Transglutaminase 2 reprogramming of glucose metabolism in mammary epithelial cells via activation of inflammatory signaling pathways.	Pubmed	0.0044	65.1	0.0114	0.116	1	1	TGM2
Pubmed	21768354	Loss of CD4 T-cell-dependent tolerance to proteins with modified amino acids.	Pubmed	0.0044	65.1	0.0114	0.116	1	1	HLA-DQB1
Pubmed	17300216	Nucleic acids potentiate Factor VII-activating protease (FSAP)-mediated cleavage of platelet-derived growth factor-BB and inhibition of vascular smooth muscle cell proliferation.	GeneRIF	0.0044	65.1	0.0114	0.116	1	1	PSAP
Pubmed	17882010	Cathepsin K predicts femoral neck bone mineral density change in nonosteoporotic peri- and early postmenopausal women.	GeneRIF	0.0044	65.1	0.0114	0.116	1	1	CTSK
Pubmed	23300672	Action potential clamp and pharmacology of the variant 1 Short QT Syndrome T618I hERG K¿ channel.	GeneRIF	0.0044	65.1	0.0114	0.116	1	1	KCNH2
Pubmed	22188018	Rab11 is phosphorylated by classical and novel protein kinase C isoenzymes upon sustained phorbol ester activation.	GeneRIF	0.0044	65.1	0.0114	0.116	1	1	RAB11FIP1
Pubmed	15591902	A molecular epidemiologic study of thalassemia using newborns' cord blood in a multiracial Asian population in Singapore: results and recommendations for a population screening program.	GeneRIF	0.0044	65.1	0.0114	0.116	1	1	HBB
Pubmed	23472198	BMP4 is a peripherally-derived factor for motor neurons and attenuates glutamate-induced excitotoxicity in vitro.	Pubmed	0.0044	65.1	0.0114	0.116	1	1	BMP4
Pubmed	25131137	Characterisation of tissue transglutaminase-reactive T cells from patients with coeliac disease and healthy controls.	Pubmed	0.0044	65.1	0.0114	0.116	1	1	TGM2
Pubmed	15153712	Molecular analysis of beta-thalassemia and sickle cell anemia in Antalya.	GeneRIF	0.0044	65.1	0.0114	0.116	1	1	HBB
Pubmed	1590788	The primary structure of mouse saposin.	Pubmed	0.0044	65.1	0.0114	0.116	1	1	PSAP
Pubmed	17565724	Sickle liver disease--an unusual presentation in a compound heterozygote for HbS and a novel beta-thalassemia mutation.	GeneRIF	0.0044	65.1	0.0114	0.116	1	1	HBB
Pubmed	22906265	Levels of the oxidative stress marker ¿-glutamyltranspeptidase at different stages of nonalcoholic fatty liver disease.	GeneRIF	0.0044	65.1	0.0114	0.116	1	1	GGT1
Pubmed	24731748	High class II-associated invariant chain peptide expression on residual leukemic cells is associated with increased relapse risk in acute myeloid leukemia.	Pubmed	0.0044	65.1	0.0114	0.116	1	1	CD74
Pubmed	11773860	Human 3'-phosphoadenosine 5'-phosphosulfate synthetase 2 (PAPSS2) pharmacogenetics: gene resequencing, genetic polymorphisms and functional characterization of variant allozymes.	Pubmed	0.0044	65.1	0.0114	0.116	1	1	PAPSS2
Pubmed	11857806	A conditional null allele of the major histocompatibility IA-beta chain gene.	Pubmed	0.0044	65.1	0.0114	0.116	1	1	HLA-DQB1
Pubmed	6646217	Alpha-thalassaemia caused by a polyadenylation signal mutation.	Pubmed	0.0044	65.1	0.0114	0.116	1	1	HBA2

Category	ID	Name	Source	p-value	q-value Bonferroni	q-value FDR B&H	q-value FDR B&Y	Hit Count in Query List	Hit Count in Genome	Hit in Query List
Pubmed	10620608	In vivo evidence for the contribution of human histocompatibility leukocyte antigen (HLA)-DQ molecules to the development of diabetes.	Pubmed	0.0044	65.1	0.0114	0.116	1	1	HLA-DQB1
Pubmed	8474455	Localization of a bidirectional DNA replication origin in the native locus and in episomally amplified murine adenosine deaminase loci.	Pubmed	0.0044	65.1	0.0114	0.116	1	1	ADA
Pubmed	21922519	The p75 neurotrophin receptor has nonapoptotic antineurotrophic actions in the basal forebrain.	GeneRIF	0.0044	65.1	0.0114	0.116	1	1	NGFR
Pubmed	11766078	Autoantibody to gravin is expressed more strongly in younger and nonthymomatous patients with myasthenia gravis.	Pubmed	0.0044	65.1	0.0114	0.116	1	1	AKAP12
Pubmed	19958187	Molecular characterization of a novel 55.1 kb (G)gamma((A)gammadeltabeta)(0)-thalassemia deletion in two Canadian families.	GeneRIF	0.0044	65.1	0.0114	0.116	1	1	HBB
Pubmed	1371116	Simultaneous deficiency of sphingolipid activator proteins 1 and 2 is caused by a mutation in the initiation codon of their common gene.	Pubmed	0.0044	65.1	0.0114	0.116	1	1	PSAP
Pubmed	19221801	Identification and characterisation of human dysferlin transcript variants: implications for dysferlin mutational screening and isoforms.	GeneRIF	0.0044	65.1	0.0114	0.116	1	1	DYSF
Pubmed	10982753	SPINK1/PSTI polymorphisms act as disease modifiers in familial and idiopathic chronic pancreatitis.	Pubmed	0.0044	65.1	0.0114	0.116	1	1	SPINK1
Pubmed	19702001	Reactive oxygen species-mediated effects on vascular remodeling induced by human atrial natriuretic peptide T2238C molecular variant in endothelial cells in vitro.	GeneRIF	0.0044	65.1	0.0114	0.116	1	1	NPPA
Pubmed	12867159	Structural requirements for lysosomal targeting of the prosaposin precursor protein.	Pubmed	0.0044	65.1	0.0114	0.116	1	1	PSAP
Pubmed	22898041	A new ß°-thalassemia frameshift mutation [ß 48 (-T)] in a Uruguayan family.	GeneRIF	0.0044	65.1	0.0114	0.116	1	1	HBB
Pubmed	17516459	Blockade of HERG K+ channel by an antihistamine drug brompheniramine requires the channel binding within the S6 residue Y652 and F656.	GeneRIF	0.0044	65.1	0.0114	0.116	1	1	KCNH2
Pubmed	10861239	Kinetic properties of missense mutations in patients with glutathione synthetase deficiency.	Pubmed	0.0044	65.1	0.0114	0.116	1	1	GSS
Pubmed	20727023	Cathepsin K expression: a useful marker for the differential diagnosis of dermatofibroma and dermatofibrosarcoma protuberans.	GeneRIF	0.0044	65.1	0.0114	0.116	1	1	CTSK
Pubmed	22996270	Enhancement of membrane B7-H3 costimulatory molecule but reduction of its soluble form in multiple sclerosis.	GeneRIF	0.0044	65.1	0.0114	0.116	1	1	CD276
Pubmed	17897828	Limb-girdle muscular dystrophy: diagnostic evaluation, frequency and clues to pathogenesis.	GeneRIF	0.0044	65.1	0.0114	0.116	1	1	DYSF
Pubmed	12853460	Trichohyalin mechanically strengthens the hair follicle: multiple cross-bridging roles in the inner root shealth.	Pubmed	0.0044	65.1	0.0114	0.116	1	1	TCHH
Pubmed	21622153	Identification of protein-protein interactions of human HtrA1.	GeneRIF	0.0044	65.1	0.0114	0.116	1	1	HTRA1
Pubmed	21777565	The hERG K(+) channel S4 domain L532P mutation: characterization at 37°C.	GeneRIF	0.0044	65.1	0.0114	0.116	1	1	KCNH2
Pubmed	23824674	Clinical and radiographic features of the autosomal recessive form of brachyolmia caused by PAPSS2 mutations.	Pubmed	0.0044	65.1	0.0114	0.116	1	1	PAPSS2
Pubmed	15857675	Tissue transglutaminase during mouse central nervous system development: lack of alternative RNA processing and implications for its role(s) in murine models of neurotrauma and neurodegeneration.	GeneRIF	0.0044	65.1	0.0114	0.116	1	1	TGM2
Pubmed	23096761	Association between serum ¿-glutamyltransferase and pulmonary dysfunction.	GeneRIF	0.0044	65.1	0.0114	0.116	1	1	GGT1
Pubmed	18642328	Increased PAK6 expression in prostate cancer and identification of PAK6 associated proteins.	GeneRIF	0.0044	65.1	0.0114	0.116	1	1	PAK6
Pubmed	10666717	Polymorphism and divergence in the beta-globin replication origin initiation region.	Pubmed	0.0044	65.1	0.0114	0.116	1	1	HBB
Pubmed	19720059	p75 neurotrophin receptor is involved in proliferation of undifferentiated mouse embryonic stem cells.	GeneRIF	0.0044	65.1	0.0114	0.116	1	1	NGFR
Pubmed	21093326	Hb Stanleyville II [alpha 78(EF7) Asn¿Lys] occurrence in combination with Hb Constant Spring.	GeneRIF	0.0044	65.1	0.0114	0.116	1	1	HBA2
Pubmed	19269710	Diagnosis value of serum B7-H3 expression in non small cell lung cancer.	GeneRIF	0.0044	65.1	0.0114	0.116	1	1	CD276
Pubmed	7751914	Elements in the 5' flanking sequences of the mouse low-affinity NGF receptor gene direct appropriate CNS, but not PNS, expression in transgenic mice.	Pubmed	0.0044	65.1	0.0114	0.116	1	1	NGFR
Pubmed	15752564	Can tissue transglutaminase be a marker of idiopathic inflammatory myopathies?	GeneRIF	0.0044	65.1	0.0114	0.116	1	1	TGM2
Pubmed	23590708	BMP4, a strong better prognosis predictor, has a subtype preference and cell development association in gliomas.	Pubmed	0.0044	65.1	0.0114	0.116	1	1	BMP4
Pubmed	12127686	Thyroid hormone receptor alpha 1 (c-erb A alpha 1) suppressed transforming phenotype of nasopharyngeal carcinoma cell line.	GeneRIF	0.0044	65.1	0.0114	0.116	1	1	THRA
Pubmed	12506066	Corneal neovascularization after excimer keratectomy wounds in matrilysin-deficient mice.	Pubmed	0.0044	65.1	0.0114	0.116	1	1	MMP7
Pubmed	17546030	Mutations in X-linked PORCN, a putative regulator of Wnt signaling, cause focal dermal hypoplasia.	GeneRIF	0.0044	65.1	0.0114	0.116	1	1	PORCN
Pubmed	14645229	Regulation of collagenase activities of human cathepsins by glycosaminoglycans.	GeneRIF	0.0044	65.1	0.0114	0.116	1	1	CTSK
Pubmed	20651391	An indication for correlation between the serum ADA level and gastric cancer risk.	GeneRIF	0.0044	65.1	0.0114	0.116	1	1	ADA
Pubmed	18547995	KCa3.1 potassium channels are critical for cAMP-dependent chloride secretion and cyst growth in autosomal-dominant polycystic kidney disease.	GeneRIF	0.0044	65.1	0.0114	0.116	1	1	KCNN4
Pubmed	12381271	The human homologue of unc-93 maps to chromosome 6q27 - characterisation and analysis in sporadic epithelial ovarian cancer.	GeneRIF	0.0044	65.1	0.0114	0.116	1	1	UNC93A
Pubmed	16960130	Bone morphogenetic protein signaling inhibits hair follicle anagen induction by restricting epithelial stem/progenitor cell activation and expansion.	GeneRIF	0.0044	65.1	0.0114	0.116	1	1	BMP4

Category	ID	Name	Source	p-value	q-value Bonferroni	q-value FDR B&H	q-value FDR B&Y	Hit Count in Query List	Hit Count in Genome	Hit in Query List
Pubmed	17981596	The biological role of interferon-inducible P204 protein in the development of the mononuclear phagocyte system.	GeneRIF	0.0044	65.1	0.0114	0.116	1	1	IFI16
Pubmed	14510697	New TCR transgenic model for tracking allospecific CD4 T-cell activation and tolerance in vivo.	Pubmed	0.0044	65.1	0.0114	0.116	1	1	HLA-DQB1
Pubmed	23470150	Identification of the first mutation in a BRE motif of the ß-globin gene and its inheritance with two other a-globin gene mutations in a Lebanese family.	Pubmed	0.0044	65.1	0.0114	0.116	1	1	HBB
Pubmed	15529011	The chemokine fractalkine in patients with severe traumatic brain injury and a mouse model of closed head injury.	GeneRIF	0.0044	65.1	0.0114	0.116	1	1	CX3CL1
Pubmed	18368130	Cathepsin K in melanoma invasion.	GeneRIF	0.0044	65.1	0.0114	0.116	1	1	CTSK
Pubmed	24646840	Methylated cysteine dioxygenase-1 gene promoter in the serum is a potential biomarker for hepatitis B virus-related hepatocellular carcinoma.	Pubmed	0.0044	65.1	0.0114	0.116	1	1	CDO1
Pubmed	22207443	P75 nerve growth factor receptor as a useful marker to distinguish spindle cell melanoma from other spindle cell neoplasms of sun-damaged skin.	GeneRIF	0.0044	65.1	0.0114	0.116	1	1	NGFR
Pubmed	22036762	The modifying effect of Xmn1-HBG2 on thalassemic phenotype is associated with its linked elements in the beta globin locus control region, including the palindromic site at 5'HS4.	GeneRIF	0.0044	65.1	0.0114	0.116	1	1	HBB
Pubmed	20352165	Blocking endothelial protein C receptor (EPCR) accelerates thrombus development in vivo.	GeneRIF	0.0044	65.1	0.0114	0.116	1	1	PROCR
Pubmed	8227344	Novel splicing, missense, and deletion mutations in seven adenosine deaminase-deficient patients with late/delayed onset of combined immunodeficiency disease. Contribution of genotype to phenotype.	Pubmed	0.0044	65.1	0.0114	0.116	1	1	ADA
Pubmed	22786767	Secretory leukocyte proteinase inhibitor-competent DNA deposits are potent stimulators of plasmacytoid dendritic cells: implication for psoriasis.	GeneRIF	0.0044	65.1	0.0114	0.116	1	1	SLPI
Pubmed	1818763	Hydroxyapatite formation in the presence of proteoglycans of reduced sulfate content: studies in the brachymorphic mouse.	Pubmed	0.0044	65.1	0.0114	0.116	1	1	PAPSS2
Pubmed	24074973	Genetic modifier of the QTc interval associated with early-onset atrial fibrillation.	Pubmed	0.0044	65.1	0.0114	0.116	1	1	KCNH2
Pubmed	22100229	Loss of fibulin-2 protects against progressive ventricular dysfunction after myocardial infarction.	GeneRIF	0.0044	65.1	0.0114	0.116	1	1	FBLN2
Pubmed	8946175	Fibulin-2: genetic mapping and exclusion as a candidate gene in Marfan syndrome type 2.	Pubmed	0.0044	65.1	0.0114	0.116	1	1	FBLN2
Pubmed	20872251	Midregional pro-atrial natriuretic peptide: a novel marker of myocardial fibrosis in patients with hypertrophic cardiomyopathy.	GeneRIF	0.0044	65.1	0.0114	0.116	1	1	NPPA
Pubmed	24552590	Identification of a new hormone-binding site on the surface of thyroid hormone receptor.	Pubmed	0.0044	65.1	0.0114	0.116	1	1	THRA
Pubmed	23516275	Calpains, cleaved mini-dysferlinC72, and L-type channels underpin calcium-dependent muscle membrane repair.	GeneRIF	0.0044	65.1	0.0114	0.116	1	1	DYSF
Pubmed	22125836	[Expression and clinical significance of FOX04 in colorectal cancer].	GeneRIF	0.0044	65.1	0.0114	0.116	1	1	FOXO4
Pubmed	9050929	Human orosomucoid polymorphism: molecular basis of the three common ORM1 alleles, ORM1*F1, ORM1*F2, and ORM1*S.	Pubmed	0.0044	65.1	0.0114	0.116	1	1	ORM1
Pubmed	19239248	A quantitative analysis of transglutaminase 2-mediated deamidation of gluten peptides: implications for the T-cell response in celiac disease.	GeneRIF	0.0044	65.1	0.0114	0.116	1	1	TGM2
Pubmed	16689864	Influence of calcium on the proteolytic degradation of the calmodulin-like skin protein (calmodulin-like protein 5) in psoriatic epidermis.	GeneRIF	0.0044	65.1	0.0114	0.116	1	1	CALML5
Pubmed	18791070	The human ERG1 channel polymorphism, K897T, creates a phosphorylation site that inhibits channel activity.	GeneRIF	0.0044	65.1	0.0114	0.116	1	1	KCNH2
Pubmed	2525038	Atrial natriuretic factor inhibits proliferation of vascular smooth muscle cells stimulated by platelet-derived growth factor.	Pubmed	0.0044	65.1	0.0114	0.116	1	1	NPPA
Pubmed	24145649	Frequent expression of napsin A in clear cell carcinoma of the endometrium: potential diagnostic utility.	Pubmed	0.0044	65.1	0.0114	0.116	1	1	NAPSA
Pubmed	15727901	Hb Montfermeil [beta 130(H8) Tyr-->Cys]: suggests a key role for the interaction between helix A and H in oxygen affinity of the hemoglobin molecule.	GeneRIF	0.0044	65.1	0.0114	0.116	1	1	HBB
Pubmed	23870673	The steroidogenic enzyme Cyp11a1 is essential for development of peanut-induced intestinal anaphylaxis.	Pubmed	0.0044	65.1	0.0114	0.116	1	1	CYP11A1
Pubmed	12810822	Seminolipid and its precursor/degradative product, galactosylalkylacylglycerol, in the testis of saposin A- and prosaposin-deficient mice.	GeneRIF	0.0044	65.1	0.0114	0.116	1	1	PSAP
Pubmed	24854852	Angiographic features of transgenic mice with increased expression of human serine protease HTRA1 in retinal pigment epithelium.	Pubmed	0.0044	65.1	0.0114	0.116	1	1	HTRA1
Pubmed	3838976	A new unstable, high oxygen affinity hemoglobin: Hb Nagoya or beta 97 (FG4) His----Pro.	Pubmed	0.0044	65.1	0.0114	0.116	1	1	HBB
Pubmed	17051151	Surface expression of MHC class II in dendritic cells is controlled by regulated ubiquitination.	Pubmed	0.0044	65.1	0.0114	0.116	1	1	HLA-DQB1
Pubmed	16763214	Tissue-transglutaminase contributes to neutrophil granulocyte differentiation and functions.	GeneRIF	0.0044	65.1	0.0114	0.116	1	1	TGM2
Pubmed	19615036	Low-affinity nerve growth factor receptor (P75 NGFR) as a marker of perineural invasion in malignant melanomas.	GeneRIF	0.0044	65.1	0.0114	0.116	1	1	NGFR
Pubmed	7673713	Characteristics of the influenza virus-specific CD8+ T cell response in mice homozygous for disruption of the H-2lAb gene.	Pubmed	0.0044	65.1	0.0114	0.116	1	1	HLA-DQB1
Pubmed	20692240	Role of p75NTR in NMDAR-mediated excitotoxic brain injury in neonatal mice.	GeneRIF	0.0044	65.1	0.0114	0.116	1	1	NGFR

Category	ID	Name	Source	p-value	q-value Bonferroni	q-value FDR B&H	q-value FDR B&Y	Hit Count in Query List	Hit Count in Genome	Hit in Query List
Pubmed	17982048	Secretory leukoprotease inhibitor in mucosal lymph node dendritic cells regulates the threshold for mucosal tolerance.	GeneRIF	0.0044	65.1	0.0114	0.116	1	1	SLPI
Pubmed	24129163	Pro-inflammatory effects of matrix metalloproteinase 7 in acute inflammation.	Pubmed	0.0044	65.1	0.0114	0.116	1	1	MMP7
Pubmed	16396936	Specific haplotypes of the CALPAIN-5 gene are associated with polycystic ovary syndrome.	GeneRIF	0.0044	65.1	0.0114	0.116	1	1	CAPN5
Pubmed	16868661	Immunohistochemical expression of peripheral benzodiazepine receptors in human astrocytomas and its correlation with grade of malignancy, proliferation, apoptosis and survival.	GeneRIF	0.0044	65.1	0.0114	0.116	1	1	TSPO
Pubmed	9843921	Structure of the murine secretory leukoprotease inhibitor (Slpi) gene and chromosomal localization of the human and murine SLPI genes.	Pubmed	0.0044	65.1	0.0114	0.116	1	1	SLPI
Pubmed	23656990	Distinct underlying mechanisms of limb and respiratory muscle fiber weaknesses in nemaline myopathy.	GeneRIF	0.0044	65.1	0.0114	0.116	1	1	ACTA1
Pubmed	22161836	Proteome changes induced by overexpression of the p75 neurotrophin receptor (p75NTR) in breast cancer cells.	GeneRIF	0.0044	65.1	0.0114	0.116	1	1	NGFR
Pubmed	22472607	Translocator protein (18¿kDa) polymorphism (rs6971) explains in-vivo brain binding affinity of the PET radioligand [(18)F]-FEPPA.	GeneRIF	0.0044	65.1	0.0114	0.116	1	1	TSPO
Pubmed	24760034	AKAP12 mediates barrier functions of fibrotic scars during CNS repair.	Pubmed	0.0044	65.1	0.0114	0.116	1	1	AKAP12
Pubmed	20636795	Smoothelin immunohistochemistry is a useful adjunct for assessing muscularis propria invasion in bladder carcinoma.	GeneRIF	0.0044	65.1	0.0114	0.116	1	1	SMTN
Pubmed	25156503	Nitric oxide confers cisplatin resistance in human lung cancer cells through upregulation of aldo-keto reductase 1B10 and proteasome.	Pubmed	0.0044	65.1	0.0114	0.116	1	1	AKR1B10
Pubmed	16919618	Peripheral benzodiazepine receptors on platelets of fibromyalgic patients.	GeneRIF	0.0044	65.1	0.0114	0.116	1	1	TSPO
Pubmed	20079887	Effects of congenital cataract mutation R116H on alphaA-crystallin structure, function and stability.	GeneRIF	0.0044	65.1	0.0114	0.116	1	1	CRYAA
Pubmed	19958201	Detection of a rare mutation in an Iranian family: codons 37/38/39 (7 bp deletion).	GeneRIF	0.0044	65.1	0.0114	0.116	1	1	HBB
Pubmed	11309303	Cloning of a new member of the human kallikrein gene family, KLK14, which is down-regulated in different malignancies.	Pubmed	0.0044	65.1	0.0114	0.116	1	1	KLK14
Pubmed	22899288	Bone morphogenetic protein 4 expression in multiple normal and tumor tissues reveals its importance beyond development.	GeneRIF	0.0044	65.1	0.0114	0.116	1	1	BMP4
Pubmed	23188502	Pharmacological inhibition of the Wnt acyltransferase PORCN prevents growth of WNT-driven mammary cancer.	Pubmed	0.0044	65.1	0.0114	0.116	1	1	PORCN
Pubmed	17766369	Crystal Structure of human pyridoxal kinase: structural basis of M(+) and M(2+) activation.	GeneRIF	0.0044	65.1	0.0114	0.116	1	1	PDXK
Pubmed	9604001	Changes of gene expression by lysophosphatidylcholine in vascular endothelial cells: 12 up-regulated distinct genes including 5 cell growth-related, 3 thrombosis-related, and 4 others.	Pubmed	0.0044	65.1	0.0114	0.116	1	1	AKAP12
Pubmed	3485543	The acid-stable proteinase inhibitor of human mucous secretions (HUSI-I, antileukoprotease). Complete amino acid sequence as revealed by protein and cDNA sequencing and structural homology to whey proteins and Red Sea turtle proteinase inhibitor.	Pubmed	0.0044	65.1	0.0114	0.116	1	1	SLPI
Pubmed	24043713	Human ERV3-1 env protein expression in various human tissues and tumours.	Pubmed	0.0044	65.1	0.0114	0.116	1	1	ERV3-1
Pubmed	21514153	Extramuscular manifestations in children with severe congenital myopathy due to ACTA1 gene mutations.	GeneRIF	0.0044	65.1	0.0114	0.116	1	1	ACTA1
Pubmed	19157587	A family of hereditary long QT syndrome caused by Q738X HERG mutation.	GeneRIF	0.0044	65.1	0.0114	0.116	1	1	KCNH2
Pubmed	12408967	Widely spaced alternative promoters, conserved between human and rodent, control expression of the Opitz syndrome gene MID1.	GeneRIF	0.0044	65.1	0.0114	0.116	1	1	MID1
Pubmed	19540319	Classification and nomenclature of endogenous retroviral sequences (ERVs): problems and recommendations.	Pubmed	0.0044	65.1	0.0114	0.116	1	1	ERV3-1
Pubmed	19305991	Structure of the pore-helix of the hERG K(+) channel.	GeneRIF	0.0044	65.1	0.0114	0.116	1	1	KCNH2
Pubmed	19921697	Analysis of human kallikrein 7 expression as a potential biomarker in cervical neoplasia.	GeneRIF	0.0044	65.1	0.0114	0.116	1	1	KLK7
Pubmed	22728374	Axonal regeneration induced by blockade of glial inhibitors coupled with activation of intrinsic neuronal growth pathways.	GeneRIF	0.0044	65.1	0.0114	0.116	1	1	RTN4R
Pubmed	10974542	The KLK7 (PRSS6) gene, encoding for the stratum corneum chymotryptic enzyme is a new member of the human kallikrein gene family - genomic characterization, mapping, tissue expression and hormonal regulation.	Pubmed	0.0044	65.1	0.0114	0.116	1	1	KLK7
Pubmed	22739755	Fractalkine--a local inflammatory marker aggravating platelet activation at the vulnerable plaque.	GeneRIF	0.0044	65.1	0.0114	0.116	1	1	CX3CL1
Pubmed	19864383	Hepatitis B and hepatitis C virus replication upregulates serine protease inhibitor Kazal, resulting in cellular resistance to serine protease-dependent apoptosis.	GeneRIF	0.0044	65.1	0.0114	0.116	1	1	SPINK1
Pubmed	18540978	Nerve growth factor receptor (p75 NTR) and pattern of invasion predict poor prognosis in oral squamous cell carcinoma.	GeneRIF	0.0044	65.1	0.0114	0.116	1	1	NGFR
Pubmed	17681820	Co-inheritance of a novel deletion of the entire SPINK1 gene with a CFTR missense mutation (L997F) in a family with chronic pancreatitis.	GeneRIF	0.0044	65.1	0.0114	0.116	1	1	SPINK1
Pubmed	7202842	Brachymorphic mice (bm/bm): a generalized biochemical defect expressed primarily cartilage.	Pubmed	0.0044	65.1	0.0114	0.116	1	1	PAPSS2
Pubmed	23459091	hERG channel function: beyond long QT.	Pubmed	0.0044	65.1	0.0114	0.116	1	1	KCNH2
Pubmed	15590635	AKAP12alpha, an atypical serum response factor-dependent target gene.	GeneRIF	0.0044	65.1	0.0114	0.116	1	1	AKAP12
Pubmed	17959786	Refining our understanding of NgR1 function during myelin inhibition.	GeneRIF	0.0044	65.1	0.0114	0.116	1	1	RTN4R

Category	ID	Name	Source	p-value	q-value Bonferroni	q-value FDR B&H	q-value FDR B&Y	Hit Count in Query List	Hit Count in Genome	Hit in Query List
Pubmed	24519935	p75 neurotrophin receptor cleavage by a- and ¿-secretases is required for neurotrophin-mediated proliferation of brain tumor-initiating cells.	Pubmed	0.0044	65.1	0.0114	0.116	1	1	NGFR
Pubmed	19958194	Hb Sallanches [alpha104(G11)Cys-->Tyr, TGC>TAC] occurs frequently on the Indian subcontinent.	GeneRIF	0.0044	65.1	0.0114	0.116	1	1	HBA2
Pubmed	18855978	Suppression of gastric cancer growth by baculovirus vector-mediated transfer of normal epithelial cell specific-1 gene.	GeneRIF	0.0044	65.1	0.0114	0.116	1	1	KLK10
Pubmed	19553003	In vivo growth inhibition of head and neck squamous cell carcinoma by the Interferon-inducible gene IFI16.	GeneRIF	0.0044	65.1	0.0114	0.116	1	1	IFI16
Pubmed	22037454	Proteomic identification of dysferlin-interacting protein complexes in human vascular endothelium.	GeneRIF	0.0044	65.1	0.0114	0.116	1	1	DYSF
Pubmed	11531782	Increased deposition of fibulin-2 in solar elastosis and its colocalization with elastic fibres.	Pubmed	0.0044	65.1	0.0114	0.116	1	1	FBLN2
Pubmed	25179834	In ferrochelatase-deficient protoporphyria patients, ALAS2 expression is enhanced and erythrocytic protoporphyrin concentration correlates with iron availability.	Pubmed	0.0044	65.1	0.0114	0.116	1	1	ALAS2
Pubmed	14967485	Epitopes recognized by CBV4 responding T cells: effect of type 1 diabetes and associated HLA-DR-DQ haplotypes.	GeneRIF	0.0044	65.1	0.0114	0.116	1	1	HLA-DQB1
Pubmed	15181368	Altered biosynthesis of neuropeptide processing enzyme carboxypeptidase E after brain ischemia: molecular mechanism and implication.	GeneRIF	0.0044	65.1	0.0114	0.116	1	1	CPE
Pubmed	9354586	Mechanisms of the inhibition of human erythrocyte pyridoxal kinase by drugs.	Pubmed	0.0044	65.1	0.0114	0.116	1	1	PDXK
Pubmed	22190649	Postnatal growth and DNA methylation are associated with differential gene expression of the TACSTD2 gene and childhood fat mass.	GeneRIF	0.0044	65.1	0.0114	0.116	1	1	TACSTD2
Pubmed	19309282	Dysferlinopathy in Chile: evidence of two novel mutations in the first reported cases.	GeneRIF	0.0044	65.1	0.0114	0.116	1	1	DYSF
Pubmed	12958188	Synergistic cooperation between the AP-1 and LEF-1 transcription factors in activation of the matrilysin promoter by the src oncogene: implications in cellular invasion.	GeneRIF	0.0044	65.1	0.0114	0.116	1	1	MMP7
Pubmed	23551888	FOXO4 is necessary for neural differentiation of human embryonic stem cells.	GeneRIF	0.0044	65.1	0.0114	0.116	1	1	FOXO4
Pubmed	14512969	Cell death triggered by a novel mutation in the alphaA-crystallin gene underlies autosomal dominant cataract linked to chromosome 21q.	Pubmed	0.0044	65.1	0.0114	0.116	1	1	CRYAA
Pubmed	2666327	Human urinary kallikrein. Complete amino acid sequence and sites of glycosylation.	Pubmed	0.0044	65.1	0.0114	0.116	1	1	KLK1
Pubmed	20465523	Machine learning algorithms for the prediction of hERG and CYP450 binding in drug development.	GeneRIF	0.0044	65.1	0.0114	0.116	1	1	KCNH2
Pubmed	23437895	Sistani population: a different spectrum of ß-thalassemia mutations from other ethnic groups of Iran.	GeneRIF	0.0044	65.1	0.0114	0.116	1	1	HBB
Pubmed	16492780	Structure and mechanism of mouse cysteine dioxygenase.	GeneRIF	0.0044	65.1	0.0114	0.116	1	1	CDO1
Pubmed	9214621	Dimer model for the microfibrillar protein fibulin-2 and identification of the connecting disulfide bridge.	Pubmed	0.0044	65.1	0.0114	0.116	1	1	FBLN2
Pubmed	891976	Haemoglobin North Shore-Caracas beta 134 (H12) valine replaced by glutamic acid.	Pubmed	0.0044	65.1	0.0114	0.116	1	1	HBB
Pubmed	22473715	B7-H3 expression in colorectal cancer: nuclear localization strongly predicts poor outcome in colon cancer.	GeneRIF	0.0044	65.1	0.0114	0.116	1	1	CD276
Pubmed	20301551	Sickle Cell Disease	Pubmed	0.0044	65.1	0.0114	0.116	1	1	HBB
Pubmed	11604516	Mice with a targeted deletion of the tetranectin gene exhibit a spinal deformity.	Pubmed	0.0044	65.1	0.0114	0.116	1	1	CLEC3B
Pubmed	17716721	Neuroimmune crosstalk in asthma: dual role of the neurotrophin receptor p75NTR.	GeneRIF	0.0044	65.1	0.0114	0.116	1	1	NGFR
Pubmed	23506283	A novel GFP reporter mouse reveals Mustn1 expression in adult regenerating skeletal muscle, activated satellite cells and differentiating myoblasts.	Pubmed	0.0044	65.1	0.0114	0.116	1	1	MUSTN1
Pubmed	22257696	Role of the purinergic system in patients with cervical intraepithelial neoplasia and uterine cancer.	GeneRIF	0.0044	65.1	0.0114	0.116	1	1	ADA
Pubmed	25051500	Carboxypeptidase E modulates intestinal immune homeostasis and protects against experimental colitis in mice.	Pubmed	0.0044	65.1	0.0114	0.116	1	1	CPE
Pubmed	7142173	Purification and characterization of a tumor-associated trypsin inhibitor from the urine of a patient with ovarian cancer.	Pubmed	0.0044	65.1	0.0114	0.116	1	1	SPINK1
Pubmed	19696195	Full-length sequence of mouse acupuncture-induced 1-L (aig1l) gene including its transcriptional start site.	Pubmed	0.0044	65.1	0.0114	0.116	1	1	SEZ6L
Pubmed	18573919	Characterization of the humoral immune response to islet antigen 2 in children with newly diagnosed type 1 diabetes.	GeneRIF	0.0044	65.1	0.0114	0.116	1	1	HLA-DQB1
Pubmed	25632122	Plasticity of intact rubral projections mediates spontaneous recovery of function after corticospinal tract injury.	Pubmed	0.0044	65.1	0.0114	0.116	1	1	RTN4R
Pubmed	14729908	InsP3R-associated cGMP kinase substrate (IRAG) is essential for nitric oxide-induced inhibition of calcium signaling in human colonic smooth muscle.	GeneRIF	0.0044	65.1	0.0114	0.116	1	1	MRVI1
Pubmed	12393745	Hypoxic up-regulation of erythroid 5-aminolevulinate synthase.	GeneRIF	0.0044	65.1	0.0114	0.116	1	1	ALAS2
Pubmed	14642006	Validation of dHPLC for molecular diagnosis of beta-thalassemia in Southern Italy.	GeneRIF	0.0044	65.1	0.0114	0.116	1	1	HBB
Pubmed	14769019	Urea-dependent unfolding of murine adenosine deaminase: sequential destabilization as measured by 19F NMR.	GeneRIF	0.0044	65.1	0.0114	0.116	1	1	ADA
Pubmed	18322212	Potent antimycobacterial activity of mouse secretory leukocyte protease inhibitor.	GeneRIF	0.0044	65.1	0.0114	0.116	1	1	SLPI
Pubmed	10735633	Long QT syndrome with a high mortality rate caused by a novel G572R missense mutation in KCNH2.	Pubmed	0.0044	65.1	0.0114	0.116	1	1	KCNH2
Pubmed	22536427	Effects of MASP-1 of the complement system on activation of coagulation factors and plasma clot formation.	GeneRIF	0.0044	65.1	0.0114	0.116	1	1	MASP1

Category	ID	Name	Source	p-value	q-value Bonferroni	q-value FDR B&H	q-value FDR B&Y	Hit Count in Query List	Hit Count in Genome	Hit in Query List
Pubmed	8988541	A new HLA-DQB1*0306 allele sharing motifs from DQB1*03032 and DQB1*04 sequences.	Pubmed	0.0044	65.1	0.0114	0.116	1	1	HLA-DQB1
Pubmed	19054061	The human orthologue of murine Mpzl3 with predicted adhesive and immune functions is a potential candidate gene for immune-related hereditary hair loss.	GeneRIF	0.0044	65.1	0.0114	0.116	1	1	MPZL3
Pubmed	18202161	Identification of pro-MMP-7 as a serum marker for renal cell carcinoma by use of proteomic analysis.	GeneRIF	0.0044	65.1	0.0114	0.116	1	1	MMP7
Pubmed	23940017	A small molecule p75(NTR) ligand protects neurogenesis after traumatic brain injury.	Pubmed	0.0044	65.1	0.0114	0.116	1	1	NGFR
Pubmed	8977104	Primary structure of a putative serine protease specific for IGF-binding proteins.	Pubmed	0.0044	65.1	0.0114	0.116	1	1	HTRA1
Pubmed	1910207	Depletion of CD4+ T cells in major histocompatibility complex class II-deficient mice.	Pubmed	0.0044	65.1	0.0114	0.116	1	1	HLA-DQB1
Pubmed	2852071	Differential expression of the nerve growth factor receptor gene in multiple brain areas.	Pubmed	0.0044	65.1	0.0114	0.116	1	1	NGFR
Pubmed	18690846	The immunoregulatory protein human B7H3 is a tumor-associated antigen that regulates tumor cell migration and invasion.	GeneRIF	0.0044	65.1	0.0114	0.116	1	1	CD276
Pubmed	15905889	Relationship between the regulatory region polymorphism of human tissue kallikrein gene and essential hypertension.	GeneRIF	0.0044	65.1	0.0114	0.116	1	1	KLK1
Pubmed	11939508	Hb O-Tibesti [beta121(GH4)Glu-->Lys; beta11(A8)Val-->Ile], a hemoglobin variant carrying in the same beta chain the substitutions of Hb O-Arab and Hb Hamilton, found in combination with Hb S [beta6(A3)Glu-->Val].	Pubmed	0.0044	65.1	0.0114	0.116	1	1	HBB
Pubmed	12871285	Specificity of coagulation factor signaling.	Pubmed	0.0044	65.1	0.0114	0.116	1	1	PROCR
Pubmed	23066321	Adenosine deaminase activity in tuberculous peritonitis among patients with underlying liver cirrhosis.	GeneRIF	0.0044	65.1	0.0114	0.116	1	1	ADA
Pubmed	17009118	Role of matrix metalloproteinase-7 in colorectal adenomas.	GeneRIF	0.0044	65.1	0.0114	0.116	1	1	MMP7
Pubmed	19467228	Regulation of endothelial protein C receptor shedding by cytokines is mediated through differential activation of MAP kinase signaling pathways.	GeneRIF	0.0044	65.1	0.0114	0.116	1	1	PROCR
Pubmed	18250473	Transglutaminase type II is involved in the pathogenesis of endotoxic shock.	GeneRIF	0.0044	65.1	0.0114	0.116	1	1	TGM2
Pubmed	18292521	Interactions of T cells with fibroblast-like synoviocytes: role of the B7 family costimulatory ligand B7-H3.	GeneRIF	0.0044	65.1	0.0114	0.116	1	1	CD276
Pubmed	16356476	Differential gene expression profiling between wild-type and ALAS2-null erythroblasts: identification of novel heme-regulated genes.	GeneRIF	0.0044	65.1	0.0114	0.116	1	1	ALAS2
Pubmed	20931521	[Effect of bone morphogenetic protein 4 on glioma stem cell proliferation and apoptosis in vitro].	GeneRIF	0.0044	65.1	0.0114	0.116	1	1	BMP4
Pubmed	24244862	FER-1/Dysferlin promotes cholinergic signaling at the neuromuscular junction in C. elegans and mice.	Pubmed	0.0044	65.1	0.0114	0.116	1	1	DYSF
Pubmed	24254562	The relationship between serum tumor-associated trypsin inhibitor levels and clinicopathological parameters in patients with gastric cancer.	Pubmed	0.0044	65.1	0.0114	0.116	1	1	SPINK1
Pubmed	22406195	Differential regulation of EHD3 in human and mammalian heart failure.	GeneRIF	0.0044	65.1	0.0114	0.116	1	1	EHD3
Pubmed	25545021	Functional role of the KCa3.1 potassium channel in synovial fibroblasts from rheumatoid arthritis patients.	Pubmed	0.0044	65.1	0.0114	0.116	1	1	KCNN4
Pubmed	17944232	[Influence of ORM1 polymorphism on serum concentration of free nortriptyline].	GeneRIF	0.0044	65.1	0.0114	0.116	1	1	ORM1
Pubmed	21345794	Involvement of dominant-negative spliced variants of the intermediate conductance Ca2+-activated K+ channel, K(Ca)3.1, in immune function of lymphoid cells.	GeneRIF	0.0044	65.1	0.0114	0.116	1	1	KCNN4
Pubmed	9845323	Neuronal expression of fractalkine in the presence and absence of inflammation.	Pubmed	0.0044	65.1	0.0114	0.116	1	1	CX3CL1
Pubmed	12217699	Sickle hemoglobin fibers: mechanisms of depolymerization.	GeneRIF	0.0044	65.1	0.0114	0.116	1	1	HBB
Pubmed	24997584	Serum adenosine deaminase activity in cutaneous anthrax.	Pubmed	0.0044	65.1	0.0114	0.116	1	1	ADA
Pubmed	17580193	Atrial natriuretic peptide in diabetic and nondiabetic patients with and without heart transplantation.	GeneRIF	0.0044	65.1	0.0114	0.116	1	1	NPPA
Pubmed	24372652	Inflammatory peeling skin syndrome caused by homozygous genomic deletion in the PSORS1 region encompassing the CDSN gene.	Pubmed	0.0044	65.1	0.0114	0.116	1	1	CDSN
Pubmed	22156377	Tetrahydrocurcumin extends life span and inhibits the oxidative stress response by regulating the FOXO forkhead transcription factor.	GeneRIF	0.0044	65.1	0.0114	0.116	1	1	FOXO4
Pubmed	24695734	Role of the cytoplasmic N-terminal Cap and Per-Arnt-Sim (PAS) domain in trafficking and stabilization of Kv11.1 channels.	Pubmed	0.0044	65.1	0.0114	0.116	1	1	KCNH2
Pubmed	17895524	Plasma levels of cell-free apoptotic DNA ladders and gamma-glutamyltranspeptidase (GGT) in diabetic children.	GeneRIF	0.0044	65.1	0.0114	0.116	1	1	GGT1
Pubmed	17171363	Development of autoimmunity to transglutaminase C in children of patients with type 1 diabetes: relationship to islet autoantibodies and infant feeding.	GeneRIF	0.0044	65.1	0.0114	0.116	1	1	TGM2
Pubmed	17804757	Seizure 6-like (SEZ6L) gene and risk for lung cancer.	GeneRIF	0.0044	65.1	0.0114	0.116	1	1	SEZ6L
Pubmed	17708801	[Expression of BMP-4 in stromal cells in vitro derived from human aorta-gonad-mesonephros region].	GeneRIF	0.0044	65.1	0.0114	0.116	1	1	BMP4
Pubmed	16449978	Implications of increased tissue transglutaminase (TG2) expression in drug-resistant breast cancer (MCF-7) cells.	GeneRIF	0.0044	65.1	0.0114	0.116	1	1	TGM2
Pubmed	21077989	Secretory leukocyte protease inhibitor inhibits neutrophil apoptosis.	GeneRIF	0.0044	65.1	0.0114	0.116	1	1	SLPI
Pubmed	16085752	Initiation of DNA replication at the human beta-globin 3' enhancer.	GeneRIF	0.0044	65.1	0.0114	0.116	1	1	HBB
Pubmed	18562675	The role of thyroid hormone receptor DNA binding in negative thyroid hormone-mediated gene transcription.	GeneRIF	0.0044	65.1	0.0114	0.116	1	1	THRA

Category	ID	Name	Source	p-value	q-value Bonferroni	q-value FDR B&H	q-value FDR B&Y	Hit Count in Query List	Hit Count in Genome	Hit in Query List
Pubmed	11248807	Epitope affinity for MHC class I determines helper requirement for CTL priming.	Pubmed	0.0044	65.1	0.0114	0.116	1	1	HLA-DQB1
Pubmed	22216449	Statistical evaluation of CZE-UV and CZE-ESI-MS data of intact a-1-acid glycoprotein isoforms for their use as potential biomarkers in bladder cancer.	GeneRIF	0.0044	65.1	0.0114	0.116	1	1	ORM1
Pubmed	22536365	Targeted overexpression of osteoactivin in cells of osteoclastic lineage promotes osteoclastic resorption and bone loss in mice.	GeneRIF	0.0044	65.1	0.0114	0.116	1	1	GPNMB
Pubmed	12528814	Transglutaminase cross-links in intranuclear inclusions in Huntington disease.	GeneRIF	0.0044	65.1	0.0114	0.116	1	1	TGM2
Pubmed	18220417	Structure of the MID1 tandem B-boxes reveals an interaction reminiscent of intermolecular ring heterodimers.	GeneRIF	0.0044	65.1	0.0114	0.116	1	1	MID1
Pubmed	9497919	Human cysteine dioxygenase type I (CDO-I; EC 1.13.11.20): 5' flanking region and intron-exon structure of the gene.	Pubmed	0.0044	65.1	0.0114	0.116	1	1	CDO1
Pubmed	25155936	Individualizing fetal hemoglobin augmenting therapy for ß-type hemoglobinopathies patients.	Pubmed	0.0044	65.1	0.0114	0.116	1	1	HBB
Pubmed	18179407	Significant increase in the expression of matrix metalloproteinase 7 in primary CNS lymphoma.	GeneRIF	0.0044	65.1	0.0114	0.116	1	1	MMP7
Pubmed	20543198	Evaluation of non-synonymous NPPA single nucleotide polymorphisms in atrial fibrillation.	GeneRIF	0.0044	65.1	0.0114	0.116	1	1	NPPA
Pubmed	9642285	The human interferon-inducible protein, IFI 16, is a repressor of transcription.	Pubmed	0.0044	65.1	0.0114	0.116	1	1	IFI16
Pubmed	2937057	Inhibition of splicing but not cleavage at the 5' splice site by truncating human beta-globin pre-mRNA.	Pubmed	0.0044	65.1	0.0114	0.116	1	1	HBB
Pubmed	12569156	Endothelial lipase: direct evidence for a role in HDL metabolism.	GeneRIF	0.0044	65.1	0.0114	0.116	1	1	LIPG
Pubmed	1849407	Regulated expression of cytochrome P-450scc (cholesterol-side-chain cleavage enzyme) in cultured cell lines detected by antibody against bacterially expressed human protein.	Pubmed	0.0044	65.1	0.0114	0.116	1	1	CYP11A1
Pubmed	21326312	Hypospadias associated with hypertelorism, the mildest phenotype of Opitz syndrome.	GeneRIF	0.0044	65.1	0.0114	0.116	1	1	MID1
Pubmed	24709677	Clinical, pathologic, and mutational spectrum of dystroglycanopathy caused by LARGE mutations.	Pubmed	0.0044	65.1	0.0114	0.116	1	1	LARGE
Pubmed	23105112	The effects of transmembrane sequence and dimerization on cleavage of the p75 neurotrophin receptor by ¿-secretase.	GeneRIF	0.0044	65.1	0.0114	0.116	1	1	NGFR
Pubmed	17202853	Spatio-temporal expression and regulation of dermatopontin in the early pregnant mouse uterus.	GeneRIF	0.0044	65.1	0.0114	0.116	1	1	DPT
Pubmed	24391771	Genome-wide and differential proteomic analysis of hepatitis B virus and aflatoxin B1 related hepatocellular carcinoma in Guangxi, China.	Pubmed	0.0044	65.1	0.0114	0.116	1	1	AKR1B10
Pubmed	23342274	Soluble endothelial protein C receptor (sEPCR) is likely a biomarker of cancer-associated hypercoagulability in human hematologic malignancies.	Pubmed	0.0044	65.1	0.0114	0.116	1	1	PROCR
Pubmed	10793007	A gene for hypotrichosis simplex of the scalp maps to chromosome 6p21.3.	Pubmed	0.0044	65.1	0.0114	0.116	1	1	CDSN
Pubmed	15956966	Decreased expression of haemoglobin beta (HBB) gene in anaplastic thyroid cancer and recovery of its expression inhibits cell growth.	GeneRIF	0.0044	65.1	0.0114	0.116	1	1	HBB
Pubmed	24163367	Silencing of the tandem pore domain halothane-inhibited K+ channel 2 (THIK2) relies on combined intracellular retention and low intrinsic activity at the plasma membrane.	Pubmed	0.0044	65.1	0.0114	0.116	1	1	KCNK12
Pubmed	1613792	Three-dimensional structure of a recombinant variant of human pancreatic secretory trypsin inhibitor (Kazal type).	Pubmed	0.0044	65.1	0.0114	0.116	1	1	SPINK1
Pubmed	15300249	CD4+ T cells are required for the maintenance, not programming, of memory CD8+ T cells after acute infection.	Pubmed	0.0044	65.1	0.0114	0.116	1	1	HLA-DQB1
Pubmed	7589422	Transforming growth factor-beta 1 downregulates dexamethasone-induced tetranectin gene expression during the in vitro mineralization of the human osteoblastic cell line SV-HFO.	Pubmed	0.0044	65.1	0.0114	0.116	1	1	CLEC3B
Pubmed	21245144	The common modification in alphaA-crystallin in the lens, N101D, is associated with increased opacity in a mouse model.	GeneRIF	0.0044	65.1	0.0114	0.116	1	1	CRYAA
Pubmed	12779272	Origin of Hb A2' (Hb B2) [delta16(A13)Gly --> Arg (GGC --> CGC)].	GeneRIF	0.0044	65.1	0.0114	0.116	1	1	HBA2
Pubmed	11823488	Preferential Th1 immune response in invariant chain-deficient mice.	GeneRIF	0.0044	65.1	0.0114	0.116	1	1	CD74
Pubmed	16753017	Mice lacking cathepsin K maintain bone remodeling but develop bone fragility despite high bone mass.	GeneRIF	0.0044	65.1	0.0114	0.116	1	1	CTSK
Pubmed	25898735	[Intron 2 of human beta-globin in 3'-untranslated region enhances expression of chimeric genes].	Pubmed	0.0044	65.1	0.0114	0.116	1	1	HBB
Pubmed	18497543	High expression of KLK14 in prostatic adenocarcinoma is associated with elevated risk of prostate-specific antigen relapse.	GeneRIF	0.0044	65.1	0.0114	0.116	1	1	KLK14
Pubmed	9701626	The human bone morphogenetic protein 4 (BMP-4) gene: molecular structure and transcriptional regulation.	Pubmed	0.0044	65.1	0.0114	0.116	1	1	BMP4
Pubmed	12220575	Functional modulation of human brain Nav1.3 sodium channels, expressed in mammalian cells, by auxiliary beta 1, beta 2 and beta 3 subunits.	Pubmed	0.0044	65.1	0.0114	0.116	1	1	SCN3B
Pubmed	19958196	Hb Alperton [beta135(H13)Ala-->Val] shows decreased oxygen affinity.	GeneRIF	0.0044	65.1	0.0114	0.116	1	1	HBB
Pubmed	23941573	Expression of codon optimized human bone morphogenetic protein 4 in Pichia pastoris.	Pubmed	0.0044	65.1	0.0114	0.116	1	1	BMP4
Pubmed	17898543	Small artery remodeling and erythrocyte deformability in L-NAME-induced hypertension: role of transglutaminases.	GeneRIF	0.0044	65.1	0.0114	0.116	1	1	TGM2
Pubmed	22260316	Molecular analysis of the ß-globin gene cluster haplotypes in a Sudanese population with sickle cell anaemia.	GeneRIF	0.0044	65.1	0.0114	0.116	1	1	HBB
Pubmed	25165859	Rabies Virus Hijacks and accelerates the p75NTR retrograde axonal transport machinery.	Pubmed	0.0044	65.1	0.0114	0.116	1	1	NGFR

Category	ID	Name	Source	p-value	q-value Bonferroni	q-value FDR B&H	q-value FDR B&Y	Hit Count in Query List	Hit Count in Genome	Hit in Query List
Pubmed	23543478	Gravin gene expression in acute myeloid leukemia	Pubmed	0.0044	65.1	0.0114	0.116	1	1	AKAP12
Pubmed	2060627	Mutation in the sphingolipid activator protein 2 in a patient with a variant of Gaucher disease.	Pubmed	0.0044	65.1	0.0114	0.116	1	1	PSAP
Pubmed	20716166	Smoothelin is a specific and robust marker for distinction of muscularis propria and muscularis mucosae in the gastrointestinal tract.	GeneRIF	0.0044	65.1	0.0114	0.116	1	1	SMTN
Pubmed	16470532	Identification of the four most common beta-globin gene mutations in Greek beta-thalassemic patients and carriers by PCR-SSCP: advantages and limitations of the method.	GeneRIF	0.0044	65.1	0.0114	0.116	1	1	HBB
Pubmed	20364105	Quantitative assessment of AKAP12 promoter methylation in colorectal cancer using methylation-sensitive high resolution melting: Correlation with Duke's stage.	GeneRIF	0.0044	65.1	0.0114	0.116	1	1	AKAP12
Pubmed	10090398	In vitro and in vivo expression analysis of the interferon-inducible 203 gene.	Pubmed	0.0044	65.1	0.0114	0.116	1	1	IFI16
Pubmed	22509316	PORCN moonlights in a Wnt-independent pathway that regulates cancer cell proliferation.	GeneRIF	0.0044	65.1	0.0114	0.116	1	1	PORCN
Pubmed	20836741	Adenosine deaminase levels in premature infants with respiratory distress syndrome and bronchopulmonary dysplasia.	GeneRIF	0.0044	65.1	0.0114	0.116	1	1	ADA
Pubmed	24813308	Combined effect of serum gamma-glutamyltransferase and uric acid on Framingham risk score.	Pubmed	0.0044	65.1	0.0114	0.116	1	1	GGT1
Pubmed	21794126	Cathepsin K deficiency in mice induces structural and metabolic changes in the central nervous system that are associated with learning and memory deficits.	GeneRIF	0.0044	65.1	0.0114	0.116	1	1	CTSK
Pubmed	12927778	Characterization of nucleotide pyrophosphatase-5 as an oligomannosidic glycoprotein in rat brain.	Pubmed	0.0044	65.1	0.0114	0.116	1	1	ENPP5
Pubmed	22139411	Transglutaminase 2 as a biomarker of osteoarthritis: an update.	GeneRIF	0.0044	65.1	0.0114	0.116	1	1	TGM2
Pubmed	19412172	A primate-specific, brain isoform of KCNH2 affects cortical physiology, cognition, neuronal repolarization and risk of schizophrenia.	GeneRIF	0.0044	65.1	0.0114	0.116	1	1	KCNH2
Pubmed	21698135	Recurrent chromosome 16p13.1 duplications are a risk factor for aortic dissections.	GeneRIF	0.0044	65.1	0.0114	0.116	1	1	MYH11
Pubmed	25535830	PK11195 effect on steroidogenesis is not mediated through the translocator protein (TSPO).	Pubmed	0.0044	65.1	0.0114	0.116	1	1	TSPO
Pubmed	16604509	[With genechip technique to investigate HLA-DQB1 gene polymorphisms in south and north regions of China].	GeneRIF	0.0044	65.1	0.0114	0.116	1	1	HLA-DQB1
Pubmed	19380810	Direct hematological toxicity and illegitimate chromosomal recombination caused by the systemic activation of CreERT2.	Pubmed	0.0044	65.1	0.0114	0.116	1	1	BMP4
Pubmed	12034837	Evidence that DNase I hypersensitive site 5 of the human beta-globin locus control region functions as a chromosomal insulator in transgenic mice.	GeneRIF	0.0044	65.1	0.0114	0.116	1	1	HBB
Pubmed	16103744	Identification of new splice variants and differential expression of the human kallikrein 10 gene, a candidate cancer biomarker.	GeneRIF	0.0044	65.1	0.0114	0.116	1	1	KLK10
Pubmed	19416837	p75 reduces beta-amyloid-induced sympathetic innervation deficits in an Alzheimer's disease mouse model.	GeneRIF	0.0044	65.1	0.0114	0.116	1	1	NGFR
Pubmed	15167969	Human neutrophil defensins and secretory leukocyte proteinase inhibitor in squamous metaplastic epithelium of bronchial airways.	GeneRIF	0.0044	65.1	0.0114	0.116	1	1	SLPI
Pubmed	15994821	ERV3 and related sequences in humans: structure and RNA expression.	Pubmed	0.0044	65.1	0.0114	0.116	1	1	ERV3-1
Pubmed	16265378	Short QT syndrome: mechanisms, diagnosis and treatment.	GeneRIF	0.0044	65.1	0.0114	0.116	1	1	KCNH2
Pubmed	25938801	Association of a Novel ACTA1 Mutation With a Dominant Progressive Scapuloperoneal Myopathy in an Extended Family.	Pubmed	0.0044	65.1	0.0114	0.116	1	1	ACTA1
Pubmed	23768730	Adenosine deaminase activity in cerebrospinal fluid and serum for the diagnosis of tuberculous meningitis.	Pubmed	0.0044	65.1	0.0114	0.116	1	1	ADA
Pubmed	20814674	Detection and quantification of remote microglial activation in rodent models of focal ischaemia using the TSPO radioligand CLINDE.	GeneRIF	0.0044	65.1	0.0114	0.116	1	1	TSPO
Pubmed	18752444	Sickle cell trait is associated with a delayed onset of malaria: implications for time-to-event analysis in clinical studies of malaria.	GeneRIF	0.0044	65.1	0.0114	0.116	1	1	HBB
Pubmed	16609006	Glycoprotein nonmetastatic melanoma protein B, a potential molecular therapeutic target in patients with glioblastoma multiforme.	GeneRIF	0.0044	65.1	0.0114	0.116	1	1	GPNMB
Pubmed	20347720	Distinct role of the intracellular C-terminus for subcellular expression, shedding and function of the murine transmembrane chemokine CX3CL1.	GeneRIF	0.0044	65.1	0.0114	0.116	1	1	CX3CL1
Pubmed	18781376	Effect of sibutramine HCl on cardiac hERG K+ channel.	GeneRIF	0.0044	65.1	0.0114	0.116	1	1	KCNH2
Pubmed	8361346	Structure and regulation of the gene encoding the neuron-specific protein PEP-19.	Pubmed	0.0044	65.1	0.0114	0.116	1	1	PCP4
Pubmed	23152936	Hkat, a novel nutritionally regulated transmembrane protein in adipose tissues.	GeneRIF	0.0044	65.1	0.0114	0.116	1	1	TRABD2B
Pubmed	9315882	Absence of p75NTR causes increased basal forebrain cholinergic neuron size, choline acetyltransferase activity, and target innervation.	Pubmed	0.0044	65.1	0.0114	0.116	1	1	NGFR
Pubmed	8240317	A new member of the C1s family of complement proteins found in a bactericidal factor, Ra-reactive factor, in human serum.	Pubmed	0.0044	65.1	0.0114	0.116	1	1	MASP1
Pubmed	24863408	Enhanced transglutaminase 2 expression in response to stress-related catecholamines in macrophages.	Pubmed	0.0044	65.1	0.0114	0.116	1	1	TGM2
Pubmed	20712009	Intermediate conductance Ca2+ activated K+ channels are expressed and functional in breast adenocarcinomas: correlation with tumour grade and metastasis status.	GeneRIF	0.0044	65.1	0.0114	0.116	1	1	KCNN4
Pubmed	21458573	Increased expression of the beta3 subunit of voltage-gated Na+ channels in the spinal cord of the SOD1G93A mouse.	GeneRIF	0.0044	65.1	0.0114	0.116	1	1	SCN3B

Category	ID	Name	Source	p-value	q-value Bonferroni	q-value FDR B&H	q-value FDR B&Y	Hit Count in Query List	Hit Count in Genome	Hit in Query List
Pubmed	18823373	Predictive value of MMP-7 expression for response to chemotherapy and survival in patients with non-small cell lung cancer.	GeneRIF	0.0044	65.1	0.0114	0.116	1	1	MMP7
Pubmed	16877565	Fractalkine stimulates angiogenesis by activating the Raf-1/MEK/ERK- and PI3K/Akt/eNOS-dependent signal pathways.	GeneRIF	0.0044	65.1	0.0114	0.116	1	1	CX3CL1
Pubmed	23275345	Autosomal recessive atrial dilated cardiomyopathy with standstill evolution associated with mutation of Natriuretic Peptide Precursor A.	GeneRIF	0.0044	65.1	0.0114	0.116	1	1	NPPA
Pubmed	22228370	Is the SPINK1 variant p.N34S overrepresented in patients with acute pancreatitis?	GeneRIF	0.0044	65.1	0.0114	0.116	1	1	SPINK1
Pubmed	17695544	High gene expression of matrix metalloproteinase-7 is associated with early stages of oral cancer.	GeneRIF	0.0044	65.1	0.0114	0.116	1	1	MMP7
Pubmed	9467006	Autosomal dominant congenital cataract associated with a missense mutation in the human alpha crystallin gene CRYAA.	Pubmed	0.0044	65.1	0.0114	0.116	1	1	CRYAA
Pubmed	9809976	The role for NES1 serine protease as a novel tumor suppressor.	Pubmed	0.0044	65.1	0.0114	0.116	1	1	KLK10
Pubmed	14975928	Molecular and functional characterization of common polymorphisms in HERG (KCNH2) potassium channels.	GeneRIF	0.0044	65.1	0.0114	0.116	1	1	KCNH2
Pubmed	22169612	[Gene mapping and mutation detection in a family with congenital anterior polar cataract].	GeneRIF	0.0044	65.1	0.0114	0.116	1	1	CRYAA
Pubmed	8811317	Hb J-Europa [beta 62(E6)Ala-->Asp]: normal oxygen binding properties in a new variant involving a residue located distal to the heme.	Pubmed	0.0044	65.1	0.0114	0.116	1	1	HBB
Pubmed	19819978	Residues K128, 132, and 134 in the thyroid hormone receptor-alpha are essential for receptor acetylation and activity.	GeneRIF	0.0044	65.1	0.0114	0.116	1	1	THRA
Pubmed	19270724	Intermediate-conductance Ca2+-activated K+ channels (IKCa1) regulate human prostate cancer cell proliferation through a close control of calcium entry.	GeneRIF	0.0044	65.1	0.0114	0.116	1	1	KCNN4
Pubmed	23076164	Effect of serum gamma-glutamyl transferase levels on myocardial perfusion and long-term prognosis after primary angioplasty in patients with acute ST-elevation myocardial infarction.	Pubmed	0.0044	65.1	0.0114	0.116	1	1	TGM2
Pubmed	18042732	2-[2-(3,4-dichloro-phenyl)-2,3-dihydro-1H-isoindol-5-ylamino]-nicotinic acid (PD-307243) causes instantaneous current through human ether-a-go-go-related gene potassium channels.	GeneRIF	0.0044	65.1	0.0114	0.116	1	1	KCNH2
Pubmed	11890201	Carboxyl-terminal modification alters the subunit interactions and assembly pathways of normal and sickle hemoglobins.	GeneRIF	0.0044	65.1	0.0114	0.116	1	1	HBB
Pubmed	24152195	Elevated transglutaminase 2 activity is associated with hypoxia-induced experimental pulmonary hypertension in mice.	Pubmed	0.0044	65.1	0.0114	0.116	1	1	TGM2
Pubmed	12484636	Hb Rampa [alpha 95(G2)pro-->Ser (alpha 2)] in a family of European ancestry: DNA analysis confirms the CCG-->TCG mutation at codon 95 of the alpha 2-globin gene; clinical and laboratory features.	Pubmed	0.0044	65.1	0.0114	0.116	1	1	HBA2
Pubmed	12809673	Linkage disequilibrium analysis of the human adenosine deaminase (ada) gene provides evidence for a lack of correlation between hot spots of equal and unequal homologous recombination.	GeneRIF	0.0044	65.1	0.0114	0.116	1	1	ADA
Pubmed	16184579	Three novel polymorphisms in the beta globin gene.	GeneRIF	0.0044	65.1	0.0114	0.116	1	1	HBB
Pubmed	23860812	Critical roles of junctophilin-2 in T-tubule and excitation-contraction coupling maturation during postnatal development.	Pubmed	0.0044	65.1	0.0114	0.116	1	1	JPH2
Pubmed	20382119	Activin B inhibits lipolysis in 3T3-L1 adipocytes.	Pubmed	0.0044	65.1	0.0114	0.116	1	1	INHBB
Pubmed	19901882	Pleural effusion adenosine deaminase (ADA) level and occult tuberculous pleurisy.	GeneRIF	0.0044	65.1	0.0114	0.116	1	1	ADA
Pubmed	11778661	An erythroid-specific chromatin opening element reorganizes beta-globin promoter chromatin structure and augments gene expression.	GeneRIF	0.0044	65.1	0.0114	0.116	1	1	HBB
Pubmed	20504958	Distinct functions of BMP4 during different stages of mouse ES cell neural commitment.	GeneRIF	0.0044	65.1	0.0114	0.116	1	1	BMP4
Pubmed	9895333	Posttranslational deamidation of proteins: the case of hemoglobin J Sardegna [alpha50(CD8)His-->Asn-->Asp].	Pubmed	0.0044	65.1	0.0114	0.116	1	1	HBA2
Pubmed	11429284	Gene trap insertion reveals two open reading frames in the mouse SSeCKS gene: the form predominantly detected in the nervous system is suppressed by the insertion while the other, specific of the testis, remains expressed.	Pubmed	0.0044	65.1	0.0114	0.116	1	1	AKAP12
Pubmed	14738432	Newborn screening for sickle cell disease in Brazil: the Campinas experience.	GeneRIF	0.0044	65.1	0.0114	0.116	1	1	HBB
Pubmed	23376073	Trichohyalin-like 1 protein, a member of fused S100 proteins, is expressed in normal and pathologic human skin.	GeneRIF	0.0044	65.1	0.0114	0.116	1	1	TCHHL1
Pubmed	21916995	Identification of the putative intestinal stem cell marker doublecortin and CaM kinase-like-1 in Barrett's esophagus and esophageal adenocarcinoma.	GeneRIF	0.0044	65.1	0.0114	0.116	1	1	DCLK1
Pubmed	7915400	Positive selection of CD4+ T cells by TCR ligation without aggregation even in the absence of MHC.	Pubmed	0.0044	65.1	0.0114	0.116	1	1	HLA-DQB1
Pubmed	20336610	Bmp4-directed nuclear cyan fluorescent protein provides a tool for live imaging and reveals cellular resolution of Bmp4 expression patterns during embryogenesis.	GeneRIF	0.0044	65.1	0.0114	0.116	1	1	BMP4
Pubmed	18212314	Pharmacogenetic association of the NPPA T2238C genetic variant with cardiovascular disease outcomes in patients with hypertension.	GeneRIF	0.0044	65.1	0.0114	0.116	1	1	NPPA
Pubmed	16837459	Bone morphogenetic protein-4, a novel modulator of melanogenesis.	GeneRIF	0.0044	65.1	0.0114	0.116	1	1	BMP4
Pubmed	3010705	Decreased adenosine deaminase (ADA) and 5'nucleotidase (5NT) activity in peripheral blood T cells in Hodgkin disease.	Pubmed	0.0044	65.1	0.0114	0.116	1	1	ADA

Category	ID	Name	Source	p-value	q-value Bonferroni	q-value FDR B&H	q-value FDR B&Y	Hit Count in Query List	Hit Count in Genome	Hit in Query List
Pubmed	12427763	Cloning and functional characterization of the smooth muscle ether-a-go-go-related gene K+ channel. Potential role of a conserved amino acid substitution in the S4 region.	GeneRIF	0.0044	65.1	0.0114	0.116	1	1	KCNH2
Pubmed	24116970	Alu-mediated large deletion of the CDSN gene as a cause of peeling skin disease.	Pubmed	0.0044	65.1	0.0114	0.116	1	1	CDSN
Pubmed	5309548	A study of recombination between the H-2 (histocompatibility) locus and loci closely linked with it in the house mouse.	Pubmed	0.0044	65.1	0.0114	0.116	1	1	THRA
Pubmed	24436369	Functional analysis of two haplotypes of the human endothelial protein C receptor gene.	Pubmed	0.0044	65.1	0.0114	0.116	1	1	PROCR
Pubmed	16883535	[HLA-DQB1 allele polymorphism and clinical characteristics of 15 familial myasthenia gravis cases in north China].	GeneRIF	0.0044	65.1	0.0114	0.116	1	1	HLA-DQB1
Pubmed	2387582	Structural and functional analysis of the murine adenosine deaminase gene.	Pubmed	0.0044	65.1	0.0114	0.116	1	1	ADA
Pubmed	19034806	A novel nonsense mutation Y652X in the S6/pore region of human ether-go-go gene found in a long QT syndrome family.	GeneRIF	0.0044	65.1	0.0114	0.116	1	1	KCNH2
Pubmed	20944073	Langerhans cells are not required for graft-versus-host disease.	Pubmed	0.0044	65.1	0.0114	0.116	1	1	HLA-DQB1
Pubmed	11913965	Kinetic peculiarities of human tissue kallikrein: 1--substrate activation in the catalyzed hydrolysis of H-D-valyl-L-leucyl-L-arginine 4-nitroanilide and H-D-valyl-L-leucyl-L-lysine 4-nitroanilide; 2--substrate inhibition in the catalyzed hydrolysis of N alpha-p-tosyl-L-arginine methyl ester.	GeneRIF	0.0044	65.1	0.0114	0.116	1	1	KLK1
Pubmed	17622582	A point mutation in the activation function 2 domain of thyroid hormone receptor alpha1 expressed after CRE-mediated recombination partially recapitulates hypothyroidism.	GeneRIF	0.0044	65.1	0.0114	0.116	1	1	THRA
Pubmed	12963695	Matrilysin-dependent elastolysis by human macrophages.	GeneRIF	0.0044	65.1	0.0114	0.116	1	1	MMP7
Pubmed	17251434	ERG conductance expression modulates the excitability of ventral horn GABAergic interneurons that control rhythmic oscillations in the developing mouse spinal cord.	GeneRIF	0.0044	65.1	0.0114	0.116	1	1	KCNH2
Pubmed	10587579	Mutations in connexin31 underlie recessive as well as dominant non-syndromic hearing loss.	Pubmed	0.0044	65.1	0.0114	0.116	1	1	GJB3
Pubmed	24725450	Conformational changes and translocation of tissue-transglutaminase to the plasma membranes: role in cancer cell migration.	Pubmed	0.0044	65.1	0.0114	0.116	1	1	TGM2
Pubmed	18036543	P75 nerve growth factor receptor is expressed in regenerating human nerve grafts.	GeneRIF	0.0044	65.1	0.0114	0.116	1	1	NGFR
Pubmed	15001552	Tissue transglutaminase catalyzes the formation of alpha-synuclein crosslinks in Parkinson's disease.	GeneRIF	0.0044	65.1	0.0114	0.116	1	1	TGM2
Pubmed	16039280	Tissue-specific expression pattern of human endothelial lipase in transgenic mice.	GeneRIF	0.0044	65.1	0.0114	0.116	1	1	LIPG
Pubmed	14204459	[A NEW HEMOGLOBIN I VARIANT: HBI-INTERLAKEN].	Pubmed	0.0044	65.1	0.0114	0.116	1	1	HBA2
Pubmed	12842094	Misexpression of mouse porcupine isoforms modulates the differentiation of P19 embryonic carcinoma cells.	Pubmed	0.0044	65.1	0.0114	0.116	1	1	PORCN
Pubmed	11570721	The beta-thalassemia mutation spectrum in the Iranian population.	GeneRIF	0.0044	65.1	0.0114	0.116	1	1	HBB
Pubmed	4442	Studies of human kidney gamma-glutamyl transpeptidase. Purification and structural, kinetic and immunological properties.	Pubmed	0.0044	65.1	0.0114	0.116	1	1	GGT1
Pubmed	23538006	Transglutaminase type 2 in human abdominal aortic aneurysm is a potential factor in the stabilization of extracellular matrix.	GeneRIF	0.0044	65.1	0.0114	0.116	1	1	TGM2
Pubmed	24300714	Molecular and cellular analysis of three novel alpha2-globin gene promoter mutations [HBA2: c.-59C>T], [HBA2: c.-81C>A] and [HBA2: c.-91G>A] reveal varying patterns of transcriptional and translational activities.	Pubmed	0.0044	65.1	0.0114	0.116	1	1	HBA2
Pubmed	11718469	Fluorescence polarization assay and SDS-PAGE confirms matrilysin degrades fibronectin and collagen IV whereas gelatinase A degrades collagen IV but not fibronectin.	Pubmed	0.0044	65.1	0.0114	0.116	1	1	MMP7
Pubmed	24862151	Role of the protein C receptor in cancer progression.	Pubmed	0.0044	65.1	0.0114	0.116	1	1	PROCR
Pubmed	21057706	Quantitative expression analysis and study of the novel human kallikrein-related peptidase 14 gene (KLK14) in malignant and benign breast tissues.	GeneRIF	0.0044	65.1	0.0114	0.116	1	1	KLK14
Pubmed	15577839	HLA-DQB1 *03 in allergic fungal sinusitis and other chronic hypertrophic rhinosinusitis disorders.	GeneRIF	0.0044	65.1	0.0114	0.116	1	1	HLA-DQB1
Pubmed	10681599	Metalloproteolytic release of endothelial cell protein C receptor.	Pubmed	0.0044	65.1	0.0114	0.116	1	1	PROCR
Pubmed	16386375	Radiation-induced upregulation of gamma-glutamyltransferase in colon carcinoma cells is mediated through the Ras signal transduction pathway.	GeneRIF	0.0044	65.1	0.0114	0.116	1	1	GGT1
Pubmed	17389030	HLA-DQB1*0317 is a novel allele with an unusual DR-DQ haplotype.	GeneRIF	0.0044	65.1	0.0114	0.116	1	1	HLA-DQB1
Pubmed	8938428	A nonsense mutation in the cathepsin K gene observed in a family with pycnodysostosis.	Pubmed	0.0044	65.1	0.0114	0.116	1	1	CTSK
Pubmed	21248746	Congenital muscular dystrophy type 1D (MDC1D) due to a large intragenic insertion/deletion, involving intron 10 of the LARGE gene.	GeneRIF	0.0044	65.1	0.0114	0.116	1	1	LARGE
Pubmed	17444521	Drug block of the hERG potassium channel: insight from modeling.	GeneRIF	0.0044	65.1	0.0114	0.116	1	1	KCNH2
Pubmed	22033905	Association of -619C/T polymorphism in CDSN gene and psoriasis risk: a meta-analysis.	GeneRIF	0.0044	65.1	0.0114	0.116	1	1	CDSN
Pubmed	15854524	[Proliferation of type II collagen specific T cell response and antibody formation in rheumatoid arthritis and their relations to HLA-DR4].	GeneRIF	0.0044	65.1	0.0114	0.116	1	1	HLA-DQB1
Pubmed	23519732	Dysferlin aggregation in limb-girdle muscular dystrophy type 2B/Miyoshi Myopathy necessitates mutational screen for diagnosis [corrected].	GeneRIF	0.0044	65.1	0.0114	0.116	1	1	DYSF
Pubmed	18552411	Association of HLA-DQB1 allelic sequence variation with susceptibility to systemic lupus erythematosus.	GeneRIF	0.0044	65.1	0.0114	0.116	1	1	HLA-DQB1

Category	ID	Name	Source	p-value	q-value Bonferroni	q-value FDR B&H	q-value FDR B&Y	Hit Count in Query List	Hit Count in Genome	Hit in Query List
Pubmed	25149537	CD271 is a functional and targetable marker of tumor-initiating cells in head and neck squamous cell carcinoma.	Pubmed	0.0044	65.1	0.0114	0.116	1	1	NGFR
Pubmed	23644223	Variants at chromosome 10q26 locus and the expression of HTRA1 in the retina.	GeneRIF	0.0044	65.1	0.0114	0.116	1	1	HTRA1
Pubmed	8163018	Multiple functions of pro-parts of aspartic proteinase zymogens.	Pubmed	0.0044	65.1	0.0114	0.116	1	1	NAPSA
Pubmed	23942012	Bipolar Disorder is associated with the rs6971 polymorphism in the gene encoding 18 kDa Translocator Protein (TSPO).	Pubmed	0.0044	65.1	0.0114	0.116	1	1	TSPO
Pubmed	17212715	A novel HLA-DQB1*06 allele, DQB1*0628, found through routine sequence-based HLA typing and confirmation of DQB1*060302.	Pubmed	0.0044	65.1	0.0114	0.116	1	1	HLA-DQB1
Pubmed	15520409	Missense mutations of ACTA1 cause dominant congenital myopathy with cores.	GeneRIF	0.0044	65.1	0.0114	0.116	1	1	ACTA1
Pubmed	22460808	Role of HERG1 potassium channel in both malignant transformation and disease progression in head and neck carcinomas.	GeneRIF	0.0044	65.1	0.0114	0.116	1	1	KCNH2
Pubmed	19333781	Abnormal hemoglobins among Kurdish population of Western Iran: hematological and molecular features.	GeneRIF	0.0044	65.1	0.0114	0.116	1	1	HBB
Pubmed	21328455	Associations of saposin C, Src, and androgen receptor upregulate the expression and function of androgen receptor in human prostate cancer cells.	GeneRIF	0.0044	65.1	0.0114	0.116	1	1	PSAP
Pubmed	6203042	Cloning and sequence analysis of cDNA encoding a precursor for human atrial natriuretic polypeptide.	Pubmed	0.0044	65.1	0.0114	0.116	1	1	NPPA
Pubmed	14627823	Hairpin-duplex equilibrium reflected in the A-->B transition in an undecamer quasi-palindrome present in the locus control region of the human beta-globin gene cluster.	GeneRIF	0.0044	65.1	0.0114	0.116	1	1	HBB
Pubmed	19616527	The expression of fucose isoforms of amniotic and plasma alpha-1-acid glycoprotein derived from 2nd and 3rd trimester normal pregnancies.	GeneRIF	0.0044	65.1	0.0114	0.116	1	1	ORM1
Pubmed	19205976	Diversity of the 5' beta-globin haplotype of four beta-thalassemia mutations in the Mexican population.	GeneRIF	0.0044	65.1	0.0114	0.116	1	1	HBB
Pubmed	17110383	Expression and functional characterization of the cancer-related serine protease, human tissue kallikrein 14.	GeneRIF	0.0044	65.1	0.0114	0.116	1	1	KLK14
Pubmed	826083	Hb Helsinki: a variant with a high oxygen affinity and a substitution at a 2,3-DPG binding site (beta82[EF6] Lys replaced by Met).	Pubmed	0.0044	65.1	0.0114	0.116	1	1	HBB
Pubmed	20133899	Extracellular K+ is a prerequisite for the function and plasma membrane stability of HERG channels.	GeneRIF	0.0044	65.1	0.0114	0.116	1	1	KCNH2
Pubmed	20947828	A major role for HERG in determining frequency of reentry in neonatal rat ventricular myocyte monolayer.	GeneRIF	0.0044	65.1	0.0114	0.116	1	1	KCNH2
Pubmed	22701669	An evolutionarily conserved enhancer regulates Bmp4 expression in developing incisor and limb bud.	GeneRIF	0.0044	65.1	0.0114	0.116	1	1	BMP4
Pubmed	11795900	In vivo reversal of glutathione deficiency and susceptibility to in vivo dexamethasone-induced apoptosis by N-acetylcysteine and L-2-oxothiazolidine-4-carboxylic acid, but not ascorbic acid, in thymocytes from gamma-glutamyltranspeptidase-deficient knockout mice.	Pubmed	0.0044	65.1	0.0114	0.116	1	1	GGT1
Pubmed	22047080	MMP7 -181G allele is a low-penetrant risk factor for cancer development in East Asians.	GeneRIF	0.0044	65.1	0.0114	0.116	1	1	MMP7
Pubmed	22086212	The side chain of glutamine 13 is the acyl-donor amino acid modified by type 2 transglutaminase in subunit T of the native rabbit skeletal muscle troponin complex.	GeneRIF	0.0044	65.1	0.0114	0.116	1	1	TGM2
Pubmed	17405911	Interaction between the Helicobacter pylori CagA and alpha-Pix in gastric epithelial AGS cells.	GeneRIF	0.0044	65.1	0.0114	0.116	1	1	ARHGEF6
Pubmed	16254903	Beta-globin cluster haplotypes in normal individuals and beta(0)39-thalassemia carriers from Sardinia, Italy.	GeneRIF	0.0044	65.1	0.0114	0.116	1	1	HBB
Pubmed	10637279	Tetracycline-controllable selection of CD4(+) T cells: half-life and survival signals in the absence of major histocompatibility complex class II molecules.	Pubmed	0.0044	65.1	0.0114	0.116	1	1	HLA-DQB1
Pubmed	23761630	The neutral, hydrophobic isoleucine at position I521 in the extracellular S4 domain of hERG contributes to channel gating equilibrium.	Pubmed	0.0044	65.1	0.0114	0.116	1	1	KCNH2
Pubmed	25279554	Structural requirements for the collagenase and elastase activity of cathepsin K and its selective inhibition by an exosite inhibitor.	Pubmed	0.0044	65.1	0.0114	0.116	1	1	CTSK
Pubmed	19346129	Aberrant development of neuromuscular junctions in glycosylation-defective Large(myd) mice.	GeneRIF	0.0044	65.1	0.0114	0.116	1	1	LARGE
Pubmed	23899126	Loss-of-function KCNH2 mutation in a family with long QT syndrome, epilepsy, and sudden death.	Pubmed	0.0044	65.1	0.0114	0.116	1	1	KCNH2
Pubmed	16121195	Crystal structure of 5-aminolevulinate synthase, the first enzyme of heme biosynthesis, and its link to XLSA in humans.	GeneRIF	0.0044	65.1	0.0114	0.116	1	1	ALAS2
Pubmed	3839456	Adenosine deaminase: characterization and expression of a gene with a remarkable promoter.	Pubmed	0.0044	65.1	0.0114	0.116	1	1	ADA
Pubmed	20850749	Aortic valve disease and gamma-glutamyltransferase: accumulation in tissue and relationships with calcific degeneration.	GeneRIF	0.0044	65.1	0.0114	0.116	1	1	GGT1
Pubmed	6505687	The long terminal repeat sequences of a novel human endogenous retrovirus.	Pubmed	0.0044	65.1	0.0114	0.116	1	1	ERV3-1
Pubmed	12061780	GTP is required to stabilize and display transamidation activity of transglutaminase 2.	GeneRIF	0.0044	65.1	0.0114	0.116	1	1	TGM2
Pubmed	21107115	B7-h3 ligand expression by primary breast cancer and associated with regional nodal metastasis.	GeneRIF	0.0044	65.1	0.0114	0.116	1	1	CD276
Pubmed	8877771	A mutation in HERG associated with notched T waves in long QT syndrome.	Pubmed	0.0044	65.1	0.0114	0.116	1	1	KCNH2
Pubmed	15481895	An alpha-thalassemia phenotype in a Dutch Hindustani, caused by a new point mutation that creates an alternative splice donor site in the first exon of the alpha2-globin gene.	Pubmed	0.0044	65.1	0.0114	0.116	1	1	HBA2

Category	ID	Name	Source	p-value	q-value Bonferroni	q-value FDR B&H	q-value FDR B&Y	Hit Count in Query List	Hit Count in Genome	Hit in Query List
Pubmed	23936770	The glycosylation of AGP and its associations with the binding to methadone.	Pubmed	0.0044	65.1	0.0114	0.116	1	1	ORM1
Pubmed	2900635	In vitro translation and processing of human hepatoma cell (Hep G2) gamma-glutamyl transpeptidase.	Pubmed	0.0044	65.1	0.0114	0.116	1	1	GGT1
Pubmed	23131169	Focal dermal hypoplasia due to a novel mutation in a boy with Klinefelter syndrome.	Pubmed	0.0044	65.1	0.0114	0.116	1	1	PORCN
Pubmed	22267731	PRELP protein inhibits the formation of the complement membrane attack complex.	GeneRIF	0.0044	65.1	0.0114	0.116	1	1	PRELP
Pubmed	10550614	Smoothelin in adult and developing human arteries and myocardium.	Pubmed	0.0044	65.1	0.0114	0.116	1	1	SMTN
Pubmed	23240012	Adenosine deaminase enhances the immunogenicity of human dendritic cells from healthy and HIV-infected individuals.	GeneRIF	0.0044	65.1	0.0114	0.116	1	1	ADA
Pubmed	9887386	Fibulin-2 exhibits high degree of variability, but no structural changes concordant with abdominal aortic aneurysms.	Pubmed	0.0044	65.1	0.0114	0.116	1	1	FBLN2
Pubmed	12709369	Rapid detection of beta-globin gene mutations and polymorphisms by temporal temperature gradient gel electrophoresis.	GeneRIF	0.0044	65.1	0.0114	0.116	1	1	HBB
Pubmed	22633841	a1-Acid glycoprotein induced effects in rat brain microvessel endothelial cells.	GeneRIF	0.0044	65.1	0.0114	0.116	1	1	ORM1
Pubmed	17347581	Enhanced expression of ANGPTL2 in the microvascular lesions of diabetic glomerulopathy.	GeneRIF	0.0044	65.1	0.0114	0.116	1	1	ANGPTL2
Pubmed	21692766	Genetic association of HLA-DQB1 and HLA-DRB1 polymorphisms with alopecia areata in the Italian population.	GeneRIF	0.0044	65.1	0.0114	0.116	1	1	HLA-DQB1
Pubmed	20141580	Paternal endothelial protein C receptor 219Gly variant as a mild and limited risk factor for deep vein thrombosis during pregnancy.	GeneRIF	0.0044	65.1	0.0114	0.116	1	1	PROCR
Pubmed	19027346	p204, a p200 family protein, as a multifunctional regulator of cell proliferation and differentiation.	GeneRIF	0.0044	65.1	0.0114	0.116	1	1	IFI16
Pubmed	17545692	N-Glycosylation regulates endothelial lipase-mediated phospholipid hydrolysis in apoE- and apoA-I-containing high density lipoproteins.	GeneRIF	0.0044	65.1	0.0114	0.116	1	1	LIPG
Pubmed	21472682	Expression of smoothelin and smooth muscle actin in the skin.	GeneRIF	0.0044	65.1	0.0114	0.116	1	1	SMTN
Pubmed	19796257	Scn3b knockout mice exhibit abnormal sino-atrial and cardiac conduction properties.	GeneRIF	0.0044	65.1	0.0114	0.116	1	1	SCN3B
Pubmed	24914940	Chondrocytes play a major role in the stimulation of bone growth by thyroid hormone.	Pubmed	0.0044	65.1	0.0114	0.116	1	1	THRA
Pubmed	18984536	A new C-terminal hERG mutation A915fs+47X associated with symptomatic LQT2 and auditory-trigger syncope.	GeneRIF	0.0044	65.1	0.0114	0.116	1	1	KCNH2
Pubmed	20800579	The role of the glycine triad in human glutathione synthetase.	GeneRIF	0.0044	65.1	0.0114	0.116	1	1	GSS
Pubmed	14679179	Auricular chondritis in NOD.DQ8.Abetao (Ag7-/-) transgenic mice resembles human relapsing polychondritis.	Pubmed	0.0044	65.1	0.0114	0.116	1	1	HLA-DQB1
Pubmed	17239714	Long QT syndrome in adults.	GeneRIF	0.0044	65.1	0.0114	0.116	1	1	KCNH2
Pubmed	17699431	Partial human genetic deficiency in tissue kallikrein activity and renal calcium handling.	GeneRIF	0.0044	65.1	0.0114	0.116	1	1	KLK1
Pubmed	17768381	Exclusion of TACSTD2 in an Iranian GDLD pedigree.	GeneRIF	0.0044	65.1	0.0114	0.116	1	1	TACSTD2
Pubmed	16550488	Interactions between charged residues in the transmembrane segments of the voltage-sensing domain in the hERG channel.	GeneRIF	0.0044	65.1	0.0114	0.116	1	1	KCNH2
Pubmed	19210757	Localization of the low-affinity nerve growth facto receptor p75 in human limbal epithelial cells.	GeneRIF	0.0044	65.1	0.0114	0.116	1	1	NGFR
Pubmed	22893068	Association of pattern dystrophy with an HTRA1 single-nucleotide polymorphism.	GeneRIF	0.0044	65.1	0.0114	0.116	1	1	HTRA1
Pubmed	22667341	Macrophage, mast cell and T lymphocyte infiltrations are independent predictive biomarkers of primary refractoriness or early relapse in classical Hodgkin lymphoma.	GeneRIF	0.0044	65.1	0.0114	0.116	1	1	CD68
Pubmed	1507231	Structure-function relationships in the low-affinity mutant haemoglobin Aalborg (Gly74 (E18)beta----Arg).	Pubmed	0.0044	65.1	0.0114	0.116	1	1	HBB
Pubmed	25128474	Lack of angiopoietin-like-2 expression limits the metabolic stress induced by a high-fat diet and maintains endothelial function in mice.	Pubmed	0.0044	65.1	0.0114	0.116	1	1	ANGPTL2
Pubmed	19560465	FoxO4 inhibits NF-kappaB and protects mice against colonic injury and inflammation.	GeneRIF	0.0044	65.1	0.0114	0.116	1	1	FOXO4
Pubmed	23612897	Therapeutic cardiac-targeted delivery of miR-1 reverses pressure overload-induced cardiac hypertrophy and attenuates pathological remodeling.	GeneRIF	0.0044	65.1	0.0114	0.116	1	1	FBLN2
Pubmed	18721140	Matrilysin (matrix metalloprotease-7) cleaves membrane-bound annexin II and enhances binding of tissue-type plasminogen activator to cancer cell surfaces.	GeneRIF	0.0044	65.1	0.0114	0.116	1	1	MMP7
Pubmed	22223806	Dystroglycan function requires xylosyl- and glucuronyltransferase activities of LARGE.	GeneRIF	0.0044	65.1	0.0114	0.116	1	1	LARGE
Pubmed	23325003	The potassium channel KCa3.1 as new therapeutic target for the prevention of obliterative airway disease.	GeneRIF	0.0044	65.1	0.0114	0.116	1	1	KCNN4
Pubmed	15795925	Molecular genetic confirmatory testing from newborn screening samples for the common African-American, Asian Indian, Southeast Asian, and Chinese beta-thalassemia mutations.	GeneRIF	0.0044	65.1	0.0114	0.116	1	1	HBB
Pubmed	22288963	Napsin A, a new marker for lung adenocarcinoma, is complementary and more sensitive and specific than thyroid transcription factor 1 in the differential diagnosis of primary pulmonary carcinoma: evaluation of 1674 cases by tissue microarray.	GeneRIF	0.0044	65.1	0.0114	0.116	1	1	NAPSA
Pubmed	21978521	Roles of p75(NTR), long-term depression, and cholinergic transmission in anxiety and acute stress coping.	GeneRIF	0.0044	65.1	0.0114	0.116	1	1	NGFR
Pubmed	28899	Cytoplasmic gamma-glutamyltransferase: isolation, product formation and physiological role.	Pubmed	0.0044	65.1	0.0114	0.116	1	1	GGT1
Pubmed	19806250	The A6936G polymorphism of the endothelial protein C receptor gene is associated with the risk of unexplained foetal loss in Mediterranean European couples.	GeneRIF	0.0044	65.1	0.0114	0.116	1	1	PROCR

Category	ID	Name	Source	p-value	q-value Bonferroni	q-value FDR B&H	q-value FDR B&Y	Hit Count in Query List	Hit Count in Genome	Hit in Query List
Pubmed	23160984	Serum adenosine deaminase and total immunoglobulin G correlate with markers of immune activation and inversely with CD4 counts in asymptomatic, treatment-naive HIV infection.	GeneRIF	0.0044	65.1	0.0114	0.116	1	1	ADA
Pubmed	19111905	Rod-cone dystrophy with maculopathy in genetic glutathione synthetase deficiency: a morphologic and electrophysiologic study.	GeneRIF	0.0044	65.1	0.0114	0.116	1	1	GSS
Pubmed	8242080	A homozygous 5 base-pair deletion in exon 10 of the adenosine deaminase (ADA) gene in a child with severe combined immunodeficiency and very low levels of ADA mRNA and protein.	Pubmed	0.0044	65.1	0.0114	0.116	1	1	ADA
Pubmed	19254589	Alteration of secretory leukocyte protease inhibitor in human myometrium during labor.	GeneRIF	0.0044	65.1	0.0114	0.116	1	1	SLPI
Pubmed	11279026	Refined characterization of corneodesmosin proteolysis during terminal differentiation of human epidermis and its relationship to desquamation.	Pubmed	0.0044	65.1	0.0114	0.116	1	1	CDSN
Pubmed	16798639	A new alpha chain hemoglobin variant: Hb Al-Hammadi Riyadh [alpha75(EF4)Asp-->Val (alpha2)].	GeneRIF	0.0044	65.1	0.0114	0.116	1	1	HBA2
Pubmed	23295001	Lifestyle-related biomarkers and endometrial cancer survival: elevated gamma-glutamyltransferase as an important risk factor.	GeneRIF	0.0044	65.1	0.0114	0.116	1	1	GGT1
Pubmed	22167620	Bone morphogenetic protein 4 (BMP4) is required for migration and invasion of breast cancer.	GeneRIF	0.0044	65.1	0.0114	0.116	1	1	BMP4
Pubmed	10078545	Major DQ8-restricted T-cell epitopes for human GAD65 mapped using human CD4, DQA1*0301, DQB1*0302 transgenic IA(null) NOD mice.	Pubmed	0.0044	65.1	0.0114	0.116	1	1	HLA-DQB1
Pubmed	18080843	Association of the mutation for the human carboxypeptidase E gene exon 4 with the severity of coronary artery atherosclerosis.	GeneRIF	0.0044	65.1	0.0114	0.116	1	1	CPE
Pubmed	17927564	Human heart LIM protein activates atrial-natriuretic-factor gene expression by interacting with the cardiac-restricted transcription factor Nkx2.5.	GeneRIF	0.0044	65.1	0.0114	0.116	1	1	NPPA
Pubmed	19088442	A highly conserved alanine in the S6 domain of the hERG1 K+ channel is required for normal gating.	GeneRIF	0.0044	65.1	0.0114	0.116	1	1	KCNH2
Pubmed	21864386	Effects of radiation therapy on tissue and serum concentrations of tumour associated trypsin inhibitor and their prognostic significance in rectal cancer patients.	GeneRIF	0.0044	65.1	0.0114	0.116	1	1	SPINK1
Pubmed	24044460	BMP4 is involved in the chemoresistance of myeloid leukemia cells through regulating autophagy-apoptosis balance.	Pubmed	0.0044	65.1	0.0114	0.116	1	1	BMP4
Pubmed	12085005	Indirect alloreactivity and chronic rejection.	Pubmed	0.0044	65.1	0.0114	0.116	1	1	HLA-DQB1
Pubmed	20304962	Synoviocyte-derived angiopoietin-like protein 2 contributes to synovial chronic inflammation in rheumatoid arthritis.	GeneRIF	0.0044	65.1	0.0114	0.116	1	1	ANGPTL2
Pubmed	20639625	Hemoglobin H disease in Guangxi province, Southern China: clinical review of 357 patients.	GeneRIF	0.0044	65.1	0.0114	0.116	1	1	HBA2
Pubmed	12858357	Differential expression of the human kallikrein gene 14 (KLK14) in normal and cancerous prostatic tissues.	GeneRIF	0.0044	65.1	0.0114	0.116	1	1	KLK14
Pubmed	16156939	Neonatal screening for sickle cell disorders in Ouagadougou, Burkina Faso: a pilot study.	GeneRIF	0.0044	65.1	0.0114	0.116	1	1	HBB
Pubmed	17357149	p75(NTR) gene and suicide attempts in young adults with a history of childhood-onset mood disorder.	GeneRIF	0.0044	65.1	0.0114	0.116	1	1	NGFR
Pubmed	15358678	The carboxypeptidase E knockout mouse exhibits endocrinological and behavioral deficits.	GeneRIF	0.0044	65.1	0.0114	0.116	1	1	CPE
Pubmed	15302598	Connexin31-deficient trophoblast stem cells: a model to analyze the role of gap junction communication in mouse placental development.	GeneRIF	0.0044	65.1	0.0114	0.116	1	1	GJB3
Pubmed	19277062	Goltz-Gorlin (focal dermal hypoplasia) and the microphthalmia with linear skin defects (MLS) syndrome: no evidence of genetic overlap.	GeneRIF	0.0044	65.1	0.0114	0.116	1	1	PORCN
Pubmed	23260665	Genetic dissection of neurotrophin signaling through the p75 neurotrophin receptor.	GeneRIF	0.0044	65.1	0.0114	0.116	1	1	NGFR
Pubmed	19041213	Patterns of expression and function of the p75(NGFR) protein in pancreatic cancer cells and tumours.	GeneRIF	0.0044	65.1	0.0114	0.116	1	1	NGFR
Pubmed	11821706	Molecular cloning and functional characterization of the upstream rat atrial natriuretic peptide promoter.	GeneRIF	0.0044	65.1	0.0114	0.116	1	1	NPPA
Pubmed	10562467	Preparation of the cerebroside sulfate activator (CSAct or saposin B) from human urine.	Pubmed	0.0044	65.1	0.0114	0.116	1	1	PSAP
Pubmed	22392896	Protein sequences involved in the mitochondrial import of the 3,5,3'-L-triiodothyronine receptor p43.	GeneRIF	0.0044	65.1	0.0114	0.116	1	1	THRA
Pubmed	19127788	Hemoglobin E detection using PCR with confronting two-pair primers.	GeneRIF	0.0044	65.1	0.0114	0.116	1	1	HBB
Pubmed	23442027	Proline/arginine-rich end leucine-rich repeat protein converts stem cells to ligament tissue and Zn(II) influences its nuclear expression.	Pubmed	0.0044	65.1	0.0114	0.116	1	1	PRELP
Pubmed	17845721	Role of human HGFIN/nmb in breast cancer.	GeneRIF	0.0044	65.1	0.0114	0.116	1	1	GPNMB
Pubmed	3348976	Elevated red cell adenosine deaminase activity: a marker of disordered erythropoiesis in Diamond-Blackfan anaemia and other haematologic diseases.	Pubmed	0.0044	65.1	0.0114	0.116	1	1	ADA
Pubmed	16822554	Translocator protein (18kDa): new nomenclature for the peripheral-type benzodiazepine receptor based on its structure and molecular function.	Pubmed	0.0044	65.1	0.0114	0.116	1	1	TSPO
Pubmed	23797957	First reported case of compound heterozygosity for HbA2-Yialousa (HBD: c.82 G>C) and HbA2-Wrens (HBD: c.295 G>A) in Oman.	Pubmed	0.0044	65.1	0.0114	0.116	1	1	HBA2
Pubmed	17561962	Saposin B mobilizes lipids from cholesterol-poor and bis(monoacylglycero)phosphate-rich membranes at acidic pH. Unglycosylated patient variant saposin B lacks lipid-extraction capacity.	GeneRIF	0.0044	65.1	0.0114	0.116	1	1	PSAP

Category	ID	Name	Source	p-value	q-value Bonferroni	q-value FDR B&H	q-value FDR B&Y	Hit Count in Query List	Hit Count in Genome	Hit in Query List
Pubmed	25294915	Elevated serum angiopoietin-like protein 2 correlates with the metastatic properties of colorectal cancer: a serum biomarker for early diagnosis and recurrence.	Pubmed	0.0044	65.1	0.0114	0.116	1	1	ANGPTL2
Pubmed	20535123	Private dysferlin exon skipping mutation (c.5492G>A) with a founder effect reveals further alternative splicing involving exons 49-51.	GeneRIF	0.0044	65.1	0.0114	0.116	1	1	DYSF
Pubmed	20691173	Serum gamma-glutamyltransferase fractions in myotonic dystrophy type I: differences with health subjects and patients with liver disease.	GeneRIF	0.0044	65.1	0.0114	0.116	1	1	GGT1
Pubmed	23254335	DYSF mutation analysis in a group of Chinese patients with dysferlinopathy.	Pubmed	0.0044	65.1	0.0114	0.116	1	1	DYSF
Pubmed	6097248	Cloning of genomic DNA for human atrial natriuretic factor.	Pubmed	0.0044	65.1	0.0114	0.116	1	1	NPPA
Pubmed	17964555	Pro-NGF, sortilin, and p75NTR: potential mediators of injury-induced apoptosis in the mouse dorsal root ganglion.	GeneRIF	0.0044	65.1	0.0114	0.116	1	1	NGFR
Pubmed	15556610	Overexpression of transglutaminase 2 accelerates the erythroid differentiation of human chronic myelogenous leukemia K562 cell line through PI3K/Akt signaling pathway.	GeneRIF	0.0044	65.1	0.0114	0.116	1	1	TGM2
Pubmed	12378589	Nogo-receptor gene activity: cellular localization and developmental regulation of mRNA in mice and humans.	GeneRIF	0.0044	65.1	0.0114	0.116	1	1	RTN4R
Pubmed	23029319	Nemaline myopathy-related skeletal muscle a-actin (ACTA1) mutation, Asp286Gly, prevents proper strong myosin binding and triggers muscle weakness.	GeneRIF	0.0044	65.1	0.0114	0.116	1	1	ACTA1
Pubmed	12054736	Dimerization of v-erbA on inverted repeats.	GeneRIF	0.0044	65.1	0.0114	0.116	1	1	THRA
Pubmed	15810949	High tissue expression of tumour-associated trypsin inhibitor (TATI) associates with a more favourable prognosis in gastric cancer.	GeneRIF	0.0044	65.1	0.0114	0.116	1	1	SPINK1
Pubmed	17430976	Characterisation of IRTKS, a novel IRSp53/MIM family actin regulator with distinct filament bundling properties.	GeneRIF	0.0044	65.1	0.0114	0.116	1	1	BAIAP2L1
Pubmed	18513186	Localization and characterization of the novel protein encoded by C20orf3.	GeneRIF	0.0044	65.1	0.0114	0.116	1	1	APMAP
Pubmed	20533271	[Association study on single nucleotide polymorphisms in HTRA1 gene and rheumatoid arthritis].	GeneRIF	0.0044	65.1	0.0114	0.116	1	1	HTRA1
Pubmed	1686097	Mutation of tryptophan-21 in mouse nerve growth factor (NGF) affects binding to the fast NGF receptor but not induction of neurites on PC12 cells.	Pubmed	0.0044	65.1	0.0114	0.116	1	1	NGFR
Pubmed	14747475	Molecular characterization of covalent complexes between tissue transglutaminase and gliadin peptides.	GeneRIF	0.0044	65.1	0.0114	0.116	1	1	TGM2
Pubmed	17564253	Beta-globin gene cluster haplotype frequencies in Khalkhs and Buryats of Mongolia.	GeneRIF	0.0044	65.1	0.0114	0.116	1	1	HBB
Pubmed	11731613	Ablation of TRalpha2 and a concomitant overexpression of alpha1 yields a mixed hypo- and hyperthyroid phenotype in mice.	Pubmed	0.0044	65.1	0.0114	0.116	1	1	THRA
Pubmed	11950817	Mutations in serine protease inhibitor Kazal type 1 are strongly associated with chronic pancreatitis.	GeneRIF	0.0044	65.1	0.0114	0.116	1	1	SPINK1
Pubmed	24204727	C-terminal ß9-strand of the cyclic nucleotide-binding homology domain stabilizes activated states of Kv11.1 channels.	Pubmed	0.0044	65.1	0.0114	0.116	1	1	KCNH2
Pubmed	1618849	5' flanking sequences of the murine adenosine deaminase gene direct expression of a reporter gene to specific prenatal and postnatal tissues in transgenic mice.	Pubmed	0.0044	65.1	0.0114	0.116	1	1	ADA
Pubmed	22220475	Transglutaminase 2 at the crossroads between cell death and survival.	GeneRIF	0.0044	65.1	0.0114	0.116	1	1	TGM2
Pubmed	10899903	Metabolic consequences of adenosine deaminase deficiency in mice are associated with defects in alveogenesis, pulmonary inflammation, and airway obstruction.	Pubmed	0.0044	65.1	0.0114	0.116	1	1	ADA
Pubmed	15198986	Multiple interactions between regulatory regions are required to stabilize an active chromatin hub.	GeneRIF	0.0044	65.1	0.0114	0.116	1	1	HBB
Pubmed	20406103	Mutation analysis of the HBB gene in selected Bangladeshi beta-thalassemic individuals: presence of rare mutations.	GeneRIF	0.0044	65.1	0.0114	0.116	1	1	HBB
Pubmed	2538352	Molecular cloning and sequencing of human cDNA for phosphoribosyl pyrophosphate synthetase subunit II.	Pubmed	0.0044	65.1	0.0114	0.116	1	1	PRPS2
Pubmed	10397730	Structural and functional implications of the intron/exon organization of the human endothelial cell protein C/activated protein C receptor (EPCR) gene: comparison with the structure of CD1/major histocompatibility complex alpha1 and alpha2 domains.	Pubmed	0.0044	65.1	0.0114	0.116	1	1	PROCR
Pubmed	24085483	Role of tissue transglutaminase and effect of cantharidinate in human colorectal cancer.	Pubmed	0.0044	65.1	0.0114	0.116	1	1	TGM2
Pubmed	15657052	Mouse testican-2. Expression, glycosylation, and effects on neurite outgrowth.	GeneRIF	0.0044	65.1	0.0114	0.116	1	1	SPOCK2
Pubmed	22112832	Human labour is associated with decreased cytoplasmic FoxO4.	GeneRIF	0.0044	65.1	0.0114	0.116	1	1	FOXO4
Pubmed	24328742	Murine b7-h3 is a co-stimulatory molecule for T cell activation.	Pubmed	0.0044	65.1	0.0114	0.116	1	1	CD276
Pubmed	25808416	Transglutaminase 2 interactions with extracellular matrix proteins as probed with celiac disease autoantibodies.	Pubmed	0.0044	65.1	0.0114	0.116	1	1	TGM2
Pubmed	21612679	[CRYAA gene mutation study in a family with autosomal dominant congenital cataract combined with microcornea].	GeneRIF	0.0044	65.1	0.0114	0.116	1	1	CRYAA
Pubmed	19205971	Molecular characterization of alpha-thalassemia in the Dohuk region of Iraq.	GeneRIF	0.0044	65.1	0.0114	0.116	1	1	HBA2
Pubmed	21844952	[Expression of glycoprotein non-metastatic melanoma protein B in prostate cancer and its significance].	GeneRIF	0.0044	65.1	0.0114	0.116	1	1	GPNMB
Pubmed	18510947	Biliverdin is the endogenous ligand of human serum alpha1-acid glycoprotein.	GeneRIF	0.0044	65.1	0.0114	0.116	1	1	ORM1
Pubmed	22417142	Is MMP-7 gene polymorphism a possible risk factor for chronic obstructive pulmonary disease in Turkish patients.	GeneRIF	0.0044	65.1	0.0114	0.116	1	1	MMP7
Pubmed	24727157	Transglutaminase 2 on the surface of dendritic cells is proposed to be involved in dendritic cell-T cell interaction.	Pubmed	0.0044	65.1	0.0114	0.116	1	1	TGM2

Category	ID	Name	Source	p-value	q-value Bonferroni	q-value FDR B&H	q-value FDR B&Y	Hit Count in Query List	Hit Count in Genome	Hit in Query List
Pubmed	18430612	Regulation of cardiac innervation and function via the p75 neurotrophin receptor.	GeneRIF	0.0044	65.1	0.0114	0.116	1	1	NGFR
Pubmed	23966323	Potential new mechanisms of placental damage in celiac disease: anti-transglutaminase antibodies impair human endometrial angiogenesis.	Pubmed	0.0044	65.1	0.0114	0.116	1	1	TGM2
Pubmed	19269866	Identification and molecular characterization of four new large deletions in the beta-globin gene cluster.	GeneRIF	0.0044	65.1	0.0114	0.116	1	1	HBB
Pubmed	18235968	Sequence change in the HS2-LCR and Ggamma-globin gene promoter region of sickle cell anemia patients.	GeneRIF	0.0044	65.1	0.0114	0.116	1	1	HBB
Pubmed	10870886	Hb Campinas [alpha26(B7)Ala -->Val]: a novel, electrophoretically silent, variant.	Pubmed	0.0044	65.1	0.0114	0.116	1	1	HBA2
Pubmed	23903071	Influence of the NRGN gene on intellectual ability in schizophrenia.	Pubmed	0.0044	65.1	0.0114	0.116	1	1	NRGN
Pubmed	6119797	Cortisone-induced cleft palate in the brachymorphic mouse.	Pubmed	0.0044	65.1	0.0114	0.116	1	1	PAPSS2
Pubmed	21672517	Atrial natriuretic peptide regulates lipid mobilization and oxygen consumption in human adipocytes by activating AMPK.	GeneRIF	0.0044	65.1	0.0114	0.116	1	1	NPPA
Pubmed	23770721	Increased numbers and suppressive activity of regulatory CD25(+)CD4(+) T lymphocytes in the absence of CD4 engagement by MHC class II molecules.	Pubmed	0.0044	65.1	0.0114	0.116	1	1	HLA-DQB1
Pubmed	12023969	Secretory leukocyte protease inhibitor mediates proliferation of human endometrial epithelial cells by positive and negative regulation of growth-associated genes.	GeneRIF	0.0044	65.1	0.0114	0.116	1	1	SLPI
Pubmed	3004571	Nucleotide sequence of cloned cDNA for human pancreatic kallikrein.	Pubmed	0.0044	65.1	0.0114	0.116	1	1	KLK1
Pubmed	23332128	Oxidative stress and pathology in muscular dystrophies: focus on protein thiol oxidation and dysferlinopathies.	Pubmed	0.0044	65.1	0.0114	0.116	1	1	DYSF
Pubmed	17132725	Involvement of the fractalkine pathway in the pathogenesis of childhood hemolytic uremic syndrome.	GeneRIF	0.0044	65.1	0.0114	0.116	1	1	CX3CL1
Pubmed	21807134	High abundance of CD271(+) multipotential stromal cells (MSCs) in intramedullary cavities of long bones.	GeneRIF	0.0044	65.1	0.0114	0.116	1	1	NGFR
Pubmed	19506770	Prevalence of the HLA-DQB1*0602 allele in narcolepsy and idiopathic hypersomnia patients seen at a sleep disorders outpatient unit in São Paulo.	GeneRIF	0.0044	65.1	0.0114	0.116	1	1	HLA-DQB1
Pubmed	24223695	Vitamin A transport and the transmembrane pore in the cell-surface receptor for plasma retinol binding protein.	Pubmed	0.0044	65.1	0.0114	0.116	1	1	STRA6
Pubmed	18292351	BMP4 is dispensable for skeletogenesis and fracture-healing in the limb.	GeneRIF	0.0044	65.1	0.0114	0.116	1	1	BMP4
Pubmed	17445142	Association between haemoglobin variants S and C and Mycobacterium ulcerans disease (Buruli ulcer): a case-control study in Benin.	GeneRIF	0.0044	65.1	0.0114	0.116	1	1	HBB
Pubmed	19116240	A novel homozygous mutation in CYP11A1 gene is associated with late-onset adrenal insufficiency and hypospadias in a 46,XY patient.	GeneRIF	0.0044	65.1	0.0114	0.116	1	1	CYP11A1
Pubmed	17446841	N34S mutation in the SPINK1 gene is not associated with alternative splicing.	GeneRIF	0.0044	65.1	0.0114	0.116	1	1	SPINK1
Pubmed	17589844	Molecular characterization and origins of Hb Constant Spring and Hb Paksé in Southeast Asian populations.	GeneRIF	0.0044	65.1	0.0114	0.116	1	1	HBA2
Pubmed	23128494	B7-H3 expression in ductal and lobular breast cancer and its association with IL-10.	Pubmed	0.0044	65.1	0.0114	0.116	1	1	CD276
Pubmed	18086265	Evaluating the role of HLA-DQ polymorphisms or immune response to bacterial superantigens using transgenic mice.	Pubmed	0.0044	65.1	0.0114	0.116	1	1	HLA-DQB1
Pubmed	22981786	Characterization of three new deletions in the ß-globin gene cluster during a screening survey in two French urban areas.	GeneRIF	0.0044	65.1	0.0114	0.116	1	1	HBB
Pubmed	12138199	Mice lacking skeletal muscle actin show reduced muscle strength and growth deficits and die during the neonatal period.	GeneRIF	0.0044	65.1	0.0114	0.116	1	1	ACTA1
Pubmed	22888000	Precise regulation of porcupine activity is required for physiological Wnt signaling.	GeneRIF	0.0044	65.1	0.0114	0.116	1	1	PORCN
Pubmed	7693620	Hb Hradec Kralove (Hb HK) or alpha 2 beta 2 115(G17)Ala-->Asp, a severely unstable hemoglobin variant resulting in a dominant beta-thalassemia trait in a Czech family.	Pubmed	0.0044	65.1	0.0114	0.116	1	1	HBB
Pubmed	17062081	The CX3C-chemokine fractalkine (CX3CL1) is detectable in serum of patients affected by active pityriasis rosea.	GeneRIF	0.0044	65.1	0.0114	0.116	1	1	CX3CL1
Pubmed	19875504	Dysferlin deficiency and the development of cardiomyopathy in a mouse model of limb-girdle muscular dystrophy 2B.	GeneRIF	0.0044	65.1	0.0114	0.116	1	1	DYSF
Pubmed	16189298	Peripheral benzodiazepine receptor: characterization in human T-lymphoma Jurkat cells.	Pubmed	0.0044	65.1	0.0114	0.116	1	1	TSPO
Pubmed	15930031	The functional polymorphism in the matrix metalloproteinase-7 promoter increases susceptibility to esophageal squamous cell carcinoma, gastric cardiac adenocarcinoma and non-small cell lung carcinoma.	GeneRIF	0.0044	65.1	0.0114	0.116	1	1	MMP7
Pubmed	21978377	Molecular basis of ß-thalassemia in Karnataka, India.	GeneRIF	0.0044	65.1	0.0114	0.116	1	1	HBB
Pubmed	10854329	Smooth-muscle contraction without smooth-muscle myosin.	Pubmed	0.0044	65.1	0.0114	0.116	1	1	MYH11
Pubmed	23204517	The calmodulin regulator protein, PEP-19, sensitizes ATP-induced Ca2+ release.	GeneRIF	0.0044	65.1	0.0114	0.116	1	1	PCP4
Pubmed	24074001	A R54L mutation of CRYAA associated with autosomal dominant nuclear cataracts in a Chinese family.	Pubmed	0.0044	65.1	0.0114	0.116	1	1	CRYAA
Pubmed	25920768	Myofiber damage precedes macrophage infiltration after in vivo injury in dysferlin-deficient a/j mouse skeletal muscle.	Pubmed	0.0044	65.1	0.0114	0.116	1	1	DYSF
Pubmed	22347398	The thermal structural transition of alpha-crystallin modulates subunit interactions and increases protein solubility.	GeneRIF	0.0044	65.1	0.0114	0.116	1	1	CRYAA
Pubmed	23852859	Overexpression of CPE-¿N predicts poor prognosis in colorectal cancer patients.	Pubmed	0.0044	65.1	0.0114	0.116	1	1	CPE

Category	ID	Name	Source	p-value	q-value Bonferroni	q-value FDR B&H	q-value FDR B&Y	Hit Count in Query List	Hit Count in Genome	Hit in Query List
Pubmed	20959412	Adenosine deaminase potentiates the generation of effector, memory, and regulatory CD4+ T cells.	GeneRIF	0.0044	65.1	0.0114	0.116	1	1	ADA
Pubmed	21228923	Enhanced expression of CD74 in gastrointestinal cancers and benign tissues.	GeneRIF	0.0044	65.1	0.0114	0.116	1	1	CD74
Pubmed	23602322	Serum Angptl2 levels are independently associated with albuminuria in type 2 diabetes.	Pubmed	0.0044	65.1	0.0114	0.116	1	1	ANGPTL2
Pubmed	11914751	A retroviral long terminal repeat adjacent to the HLA DQB1 gene (DQ-LTR13) modifies Type I diabetes susceptibility on high risk DQ haplotypes.	GeneRIF	0.0044	65.1	0.0114	0.116	1	1	HLA-DQB1
Pubmed	21997537	Retinoids produced by macrophages engulfing apoptotic cells contribute to the appearance of transglutaminase 2 in apoptotic thymocytes.	GeneRIF	0.0044	65.1	0.0114	0.116	1	1	TGM2
Pubmed	6112969	gamma-Glutamyl transpeptidase from WI-38 fibroblasts: purification and active site modification studies.	Pubmed	0.0044	65.1	0.0114	0.116	1	1	GGT1
Pubmed	25370867	Molecular characterization of a ß-thalassemia intermedia patient presenting inferior vena cava thrombosis: interaction of the ß-globin erythroid Krüppel-like factor binding site mutation with Hb E and a(+)-thalassemia.	Pubmed	0.0044	65.1	0.0114	0.116	1	1	HBB
Pubmed	23248151	Bone morphogenetic protein-4 mediates cardiac hypertrophy, apoptosis, and fibrosis in experimentally pathological cardiac hypertrophy.	GeneRIF	0.0044	65.1	0.0114	0.116	1	1	BMP4
Pubmed	12702499	Cytokines and BMP-4 promote hematopoietic differentiation of human embryonic stem cells.	GeneRIF	0.0044	65.1	0.0114	0.116	1	1	BMP4
Pubmed	11354825	Autism: evidence of association with adenosine deaminase genetic polymorphism.	GeneRIF	0.0044	65.1	0.0114	0.116	1	1	ADA
Pubmed	9028530	Localization of cathepsin K in human osteoclasts by in situ hybridization and immunohistochemistry.	Pubmed	0.0044	65.1	0.0114	0.116	1	1	CTSK
Pubmed	17473989	Regulation of zinc transporters by dietary zinc supplement in breast cancer.	Pubmed	0.0044	65.1	0.0114	0.116	1	1	SLC39A2
Pubmed	24663824	Involvement of CD74 in head and neck squamous cell carcinomas.	Pubmed	0.0044	65.1	0.0114	0.116	1	1	CD74
Pubmed	19350120	Clinical significance of kallikrein-related peptidase 7 (KLK7) in colorectal cancer.	GeneRIF	0.0044	65.1	0.0114	0.116	1	1	KLK7
Pubmed	19358841	Inhibition of cathepsin K reduces bone erosion, cartilage degradation and inflammation evoked by collagen-induced arthritis in mice.	GeneRIF	0.0044	65.1	0.0114	0.116	1	1	CTSK
Pubmed	17353352	Structural determinants of the closed KCa3.1 channel pore in relation to channel gating: results from a substituted cysteine accessibility analysis.	GeneRIF	0.0044	65.1	0.0114	0.116	1	1	KCNN4
Pubmed	18979635	Regulation of cathepsin K activity by hydrogen peroxide.	GeneRIF	0.0044	65.1	0.0114	0.116	1	1	CTSK
Pubmed	12507826	Prevalence and HLA association of GAD65 antibodies in Hungarian schoolchildren.	GeneRIF	0.0044	65.1	0.0114	0.116	1	1	HLA-DQB1
Pubmed	7907422	Molecular and population genetic analysis of allelic sequence diversity at the human beta-globin locus.	Pubmed	0.0044	65.1	0.0114	0.116	1	1	HBB
Pubmed	20536386	Activation of the plasma kallikrein-kinin system on human lung epithelial cells.	GeneRIF	0.0044	65.1	0.0114	0.116	1	1	KLK1
Pubmed	2479939	Synthesis of alpha 1-acid glycoprotein by the human prostate.	Pubmed	0.0044	65.1	0.0114	0.116	1	1	ORM1
Pubmed	15147324	TrkA and mitogen-activated protein kinase phosphorylation are enhanced in sympathetic neurons lacking functional p75 neurotrophin receptor expression.	Pubmed	0.0044	65.1	0.0114	0.116	1	1	NGFR
Pubmed	20717931	Hyperosmolarity-mediated mitochondrial dysfunction requires Transglutaminase-2 in human corneal epithelial cells.	GeneRIF	0.0044	65.1	0.0114	0.116	1	1	TGM2
Pubmed	12810688	Fractalkine preferentially mediates arrest and migration of CD16+ monocytes.	GeneRIF	0.0044	65.1	0.0114	0.116	1	1	CX3CL1
Pubmed	21904642	Chopper is prodeath regardless of the effect of p75ICD on sensitivity to oxidative stress.	GeneRIF	0.0044	65.1	0.0114	0.116	1	1	NGFR
Pubmed	15958618	Aberrant expression of novel and previously described cell membrane markers in human breast cancer cell lines and tumors.	GeneRIF	0.0044	65.1	0.0114	0.116	1	1	TSPAN13
Pubmed	18317448	Autocrine induction of invasion and metastasis by tumor-associated trypsin inhibitor in human colon cancer cells.	GeneRIF	0.0044	65.1	0.0114	0.116	1	1	SPINK1
Pubmed	23623979	HtrA1 is induced by oxidative stress and enhances cell senescence through p38 MAPK pathway.	Pubmed	0.0044	65.1	0.0114	0.116	1	1	HTRA1
Pubmed	18198	Partial purification and some properties of gamma-glutamyl transpeptidase from human bile.	Pubmed	0.0044	65.1	0.0114	0.116	1	1	GGT1
Pubmed	17064362	Transglutaminase 2 ablation leads to defective function of mitochondrial respiratory complex I affecting neuronal vulnerability in experimental models of extrapyramidal disorders.	Pubmed	0.0044	65.1	0.0114	0.116	1	1	TGM2
Pubmed	24594996	CD74: a new prognostic factor for patients with malignant pleural mesothelioma.	Pubmed	0.0044	65.1	0.0114	0.116	1	1	CD74
Pubmed	24194851	Characterization of a soluble B7-H3 (sB7-H3) spliced from the intron and analysis of sB7-H3 in the sera of patients with hepatocellular carcinoma.	Pubmed	0.0044	65.1	0.0114	0.116	1	1	CD276
Pubmed	12832399	Opposing roles of Ras/Raf oncogenes and the MEK1/ERK signaling module in regulation of expression and adhesive function of surface transglutaminase.	GeneRIF	0.0044	65.1	0.0114	0.116	1	1	TGM2
Pubmed	15591037	Molecular basis of bone morphogenetic protein-4 inhibitory action on progesterone secretion by ovine granulosa cells.	GeneRIF	0.0044	65.1	0.0114	0.116	1	1	BMP4
Pubmed	20026756	Stronger evidence for replication of NPPA using genome-wide genotyping data.	GeneRIF	0.0044	65.1	0.0114	0.116	1	1	NPPA
Pubmed	25461595	BMP4 increases the expression of TRPC and basal [Ca2+]i via the p38MAPK and ERK1/2 pathways independent of BMPRII in PASMCs.	Pubmed	0.0044	65.1	0.0114	0.116	1	1	BMP4
Pubmed	20623140	Role of secretory protease inhibitor SPINK3 in mouse uterus during early pregnancy.	GeneRIF	0.0044	65.1	0.0114	0.116	1	1	SPINK1
Pubmed	18032650	Non-cell-autonomous regulation of GABAergic neuron development by neurotrophins and the p75 receptor.	GeneRIF	0.0044	65.1	0.0114	0.116	1	1	NGFR
Pubmed	22868908	Angiopoietin-like protein 2 mediates endotoxin-induced acute inflammation in the eye.	GeneRIF	0.0044	65.1	0.0114	0.116	1	1	ANGPTL2

Category	ID	Name	Source	p-value	q-value Bonferroni	q-value FDR B&H	q-value FDR B&Y	Hit Count in Query List	Hit Count in Genome	Hit in Query List
Pubmed	11587070	Characterization of genomic rearrangements of the alpha1-acid glycoprotein/orosomucoid gene in Ghanaians.	GeneRIF	0.0044	65.1	0.0114	0.116	1	1	ORM2
Pubmed	7645262	Abundance of an endogenous retroviral envelope protein in placental trophoblasts suggests a biological function.	Pubmed	0.0044	65.1	0.0114	0.116	1	1	ERV3-1
Pubmed	18498386	Identification of beta-globin gene mutations in Thailand using an automated fluorescence-based DNA sequencer.	GeneRIF	0.0044	65.1	0.0114	0.116	1	1	HBB
Pubmed	12438557	Developmental changes in opioid peptides and their receptors in Cpe(fat)/Cpe(fat) mice lacking peptide processing enzyme carboxypeptidase E.	Pubmed	0.0044	65.1	0.0114	0.116	1	1	CPE
Pubmed	8718696	A novel intrachromosomal rearrangement in the beta-globin gene found in an African-American family.	Pubmed	0.0044	65.1	0.0114	0.116	1	1	HBB
Pubmed	11352573	Identification and characterization of KLK14, a novel kallikrein serine protease gene located on human chromosome 19q13.4 and expressed in prostate and skeletal muscle.	Pubmed	0.0044	65.1	0.0114	0.116	1	1	KLK14
Pubmed	2050393	Full length beta chain cDNAs of DQw9 and DQw8 molecules encode proteins that differ only at amino acid 57.	Pubmed	0.0044	65.1	0.0114	0.116	1	1	HLA-DQB1
Pubmed	22698853	The function of BMP4 during neurogenesis in the adult hippocampus in Alzheimer's disease.	GeneRIF	0.0044	65.1	0.0114	0.116	1	1	BMP4
Pubmed	19495974	Prognostic significance of hERG1 expression in gastric cancer.	GeneRIF	0.0044	65.1	0.0114	0.116	1	1	KCNH2
Pubmed	486536	A new abnormal human hemoglobin: Hb Prato (alpha 2 31 (B12) Arg leads to Ser beta 2).	Pubmed	0.0044	65.1	0.0114	0.116	1	1	HBA2
Pubmed	3384708	Hb Las Palmas or alpha 2 beta 2(49)(CD8)Ser----Phe, a mildly unstable hemoglobin variant.	Pubmed	0.0044	65.1	0.0114	0.116	1	1	HBB
Pubmed	24091414	Dysferlin is a newly identified binding partner of AßPP and it co-aggregates with amyloid-ß42 within sporadic inclusion-body myositis (s-IBM) muscle fibers.	Pubmed	0.0044	65.1	0.0114	0.116	1	1	DYSF
Pubmed	19194656	Cathepsin X-mediated beta2 integrin activation results in nanotube outgrowth.	GeneRIF	0.0044	65.1	0.0114	0.116	1	1	CTSK
Pubmed	1440860	Determination of tolerance to self E alpha peptides by clonal elimination of H-2E reactive T cells and antigen presentation by H-2A molecules.	Pubmed	0.0044	65.1	0.0114	0.116	1	1	HLA-DQB1
Pubmed	15585642	Prognostic significance of tissue transglutaminase in drug resistant and metastatic breast cancer.	GeneRIF	0.0044	65.1	0.0114	0.116	1	1	TGM2
Pubmed	6586420	The complete sequence of the mRNA for the HLA-DR-associated invariant chain reveals a polypeptide with an unusual transmembrane polarity.	Pubmed	0.0044	65.1	0.0114	0.116	1	1	CD74
Pubmed	3838797	Transient expression of human adenosine deaminase cDNAs: identification of a nonfunctional clone resulting from a single amino acid substitution.	Pubmed	0.0044	65.1	0.0114	0.116	1	1	ADA
Pubmed	14529320	A one-step real-time PCR assay for rapid prenatal diagnosis of sickle cell disease and detection of maternal contamination.	GeneRIF	0.0044	65.1	0.0114	0.116	1	1	HBB
Pubmed	12205028	Role of transglutaminase 2 in glucose tolerance: knockout mice studies and a putative mutation in a MODY patient.	GeneRIF	0.0044	65.1	0.0114	0.116	1	1	TGM2
Pubmed	14975585	Deficient p75 low-affinity neurotrophin receptor expression exacerbates experimental allergic encephalomyelitis in C57/BL6 mice.	Pubmed	0.0044	65.1	0.0114	0.116	1	1	NGFR
Pubmed	18823758	Dipeptidyl peptidase II is not a marker for progression in melanoma.	GeneRIF	0.0044	65.1	0.0114	0.116	1	1	DPP7
Pubmed	4053691	High-resolution chromosomal localization of the beta-gene of the human beta-globin gene complex by in situ hybridization.	Pubmed	0.0044	65.1	0.0114	0.116	1	1	HBB
Pubmed	7371221	Prenatal detection of a probable heterozygote for ADA deficiency and severe combined immunodeficiency disease using a microradioassay.	Pubmed	0.0044	65.1	0.0114	0.116	1	1	ADA
Pubmed	17218472	Increased gastric expression of MMP-7 in hypergastrinemia and significance for epithelial-mesenchymal signaling.	Pubmed	0.0044	65.1	0.0114	0.116	1	1	MMP7
Pubmed	25139714	B7-H3 expression in colorectal cancer: associations with clinicopathological parameters and patient outcome.	Pubmed	0.0044	65.1	0.0114	0.116	1	1	CD276
Pubmed	19775691	Cathepsin K gene disruption does not affect murine aneurysm formation.	GeneRIF	0.0044	65.1	0.0114	0.116	1	1	CTSK
Pubmed	23103500	High-throughput screening of drug-binding dynamics to HERG improves early drug safety assessment.	GeneRIF	0.0044	65.1	0.0114	0.116	1	1	KCNH2
Pubmed	10221600	Adult carboxypeptidase E-deficient fat/fat mice have a near-total depletion of brain CCK 8 accompanied by a massive accumulation of glycine and arginine extended CCK: identification of CCK 8 Gly as the immediate precursor of CCK 8 in rodent brain.	Pubmed	0.0044	65.1	0.0114	0.116	1	1	CPE
Pubmed	11239517	The HLA-DQB alleles and amino acid variants of the vitamin D-binding protein in diabetic patients in Alsace.	GeneRIF	0.0044	65.1	0.0114	0.116	1	1	HLA-DQB1
Pubmed	16877501	Cooperative sequence modules determine replication initiation sites at the human beta-globin locus.	GeneRIF	0.0044	65.1	0.0114	0.116	1	1	HBB
Pubmed	25093680	Heterodimerization of p45-p75 modulates p75 signaling: structural basis and mechanism of action.	Pubmed	0.0044	65.1	0.0114	0.116	1	1	NGFR
Pubmed	22177089	Fractalkine (CX3CL1) levels in patients with Behçet's disease and neuro-Behçet's disease.	GeneRIF	0.0044	65.1	0.0114	0.116	1	1	CX3CL1
Pubmed	12941700	HS5 of the human beta-globin locus control region: a developmental stage-specific border in erythroid cells.	GeneRIF	0.0044	65.1	0.0114	0.116	1	1	HBB
Pubmed	8943885	An IVS1-116 (A-->G) acceptor splice site mutation in the alpha 2 globin gene causing alpha + thalassaemia in two Dutch families.	Pubmed	0.0044	65.1	0.0114	0.116	1	1	HBA2
Pubmed	17617604	The antiviral CD8+ T cell response is differentially dependent on CD4+ T cell help over the course of persistent infection.	Pubmed	0.0044	65.1	0.0114	0.116	1	1	HLA-DQB1
Pubmed	12822871	Limited contribution of the SPINK1 N34S mutation to the risk and severity of alcoholic chronic pancreatitis: a report from the United States.	GeneRIF	0.0044	65.1	0.0114	0.116	1	1	SPINK1

Category	ID	Name	Source	p-value	q-value Bonferroni	q-value FDR B&H	q-value FDR B&Y	Hit Count in Query List	Hit Count in Genome	Hit in Query List
Pubmed	17762170	Sphingolipid metabolite ceramide causes metabolic perturbation contributing to HERG K+ channel dysfunction.	GeneRIF	0.0044	65.1	0.0114	0.116	1	1	KCNH2
Pubmed	15187116	Host dendritic cells alone are sufficient to initiate acute graft-versus-host disease.	Pubmed	0.0044	65.1	0.0114	0.116	1	1	HLA-DQB1
Pubmed	10902817	Thyroid hormone receptor gene expression in first trimester human fetal brain.	Pubmed	0.0044	65.1	0.0114	0.116	1	1	THRA
Pubmed	18584285	Transglutaminase-2 differently regulates cartilage destruction and osteophyte formation in a surgical model of osteoarthritis.	GeneRIF	0.0044	65.1	0.0114	0.116	1	1	TGM2
Pubmed	11733520	Activation of Paneth cell alpha-defensins in mouse small intestine.	Pubmed	0.0044	65.1	0.0114	0.116	1	1	MMP7
Pubmed	15793780	[Rapid detection of three common deletional alpha thalassemias in Chinese by single-tube multiplex PCR].	GeneRIF	0.0044	65.1	0.0114	0.116	1	1	HBB
Pubmed	23725125	Current evidence on associations between the MMP-7 (-181A>G) polymorphism and digestive system cancer risk.	Pubmed	0.0044	65.1	0.0114	0.116	1	1	MMP7
Pubmed	21194265	Co-inheritance of hemoglobin D and ß-thalassemia traits in three Iranian families: clinical relevance.	GeneRIF	0.0044	65.1	0.0114	0.116	1	1	HBB
Pubmed	20956206	Modulation of calcium-activated potassium channels induces cardiogenesis of pluripotent stem cells and enrichment of pacemaker-like cells.	GeneRIF	0.0044	65.1	0.0114	0.116	1	1	KCNN4
Pubmed	24386436	hERG potassium channel blockage by scorpion toxin BmKKx2 enhances erythroid differentiation of human leukemia cells K562.	Pubmed	0.0044	65.1	0.0114	0.116	1	1	KCNH2
Pubmed	15019984	Cloning and expression analysis of two novel PCTAIRE 3 transcripts from human brain.	GeneRIF	0.0044	65.1	0.0114	0.116	1	1	CDK18
Pubmed	8433367	Solution structure of a variant of human pancreatic secretory trypsin inhibitor determined by nuclear magnetic resonance spectroscopy.	Pubmed	0.0044	65.1	0.0114	0.116	1	1	SPINK1
Pubmed	21585341	Aldo-keto reductase family 1, member B10 is secreted through a lysosome-mediated non-classical pathway.	GeneRIF	0.0044	65.1	0.0114	0.116	1	1	AKR1B10
Pubmed	10323344	Identification of a novel DQB1 allele DBQ1*0616	Pubmed	0.0044	65.1	0.0114	0.116	1	1	HLA-DQB1
Pubmed	12576428	Alpha1 acid glycoprotein binds to imatinib (STI571) and substantially alters its pharmacokinetics in chronic myeloid leukemia patients.	GeneRIF	0.0044	65.1	0.0114	0.116	1	1	ORM1
Pubmed	15523695	Matrilysin (MMP-7) promotes invasion of ovarian cancer cells by activation of progelatinase.	GeneRIF	0.0044	65.1	0.0114	0.116	1	1	MMP7
Pubmed	15315795	Impact of beta globin gene mutations on the clinical phenotype of beta thalassemia in India.	GeneRIF	0.0044	65.1	0.0114	0.116	1	1	HBB
Pubmed	21552534	aA-crystallin peptide SDRDKFVIFLDVKHF accumulating in aging lens impairs the function of a-crystallin and induces lens protein aggregation.	GeneRIF	0.0044	65.1	0.0114	0.116	1	1	CRYAA
Pubmed	12493744	Trafficking of the Ca2+-activated K+ channel, hIK1, is dependent upon a C-terminal leucine zipper.	GeneRIF	0.0044	65.1	0.0114	0.116	1	1	KCNN4
Pubmed	14672349	Deletion mutants of BMP folding variants act as BMP antagonists and are efficient inhibitors for heterotopic ossification.	GeneRIF	0.0044	65.1	0.0114	0.116	1	1	BMP4
Pubmed	10580106	Human napsin A: expression, immunochemical detection, and tissue localization.	Pubmed	0.0044	65.1	0.0114	0.116	1	1	NAPSA
Pubmed	20488664	The proapoptotic activity of the Interferon-inducible gene IFI16 provides new insights into its etiopathogenetic role in autoimmunity.	GeneRIF	0.0044	65.1	0.0114	0.116	1	1	IFI16
Pubmed	18664521	Species differences between human and rat in the substrate specificity of cathepsin K.	GeneRIF	0.0044	65.1	0.0114	0.116	1	1	CTSK
Pubmed	24415158	Osteoactivin promotes osteoblast adhesion through HSPG and avß1 integrin.	Pubmed	0.0044	65.1	0.0114	0.116	1	1	GPNMB
Pubmed	20466052	The p75 neurotrophin receptor protects primary auditory neurons against acoustic trauma in mice.	GeneRIF	0.0044	65.1	0.0114	0.116	1	1	NGFR
Pubmed	19127454	HLA-DQB1* polymorphism and associations with dilated cardiomyopathy, inflammatory dilated cardiomyopathy and myocarditis.	GeneRIF	0.0044	65.1	0.0114	0.116	1	1	HLA-DQB1
Pubmed	15961727	Constitutive and inducible expression of b7 family of ligands by human airway epithelial cells.	Pubmed	0.0044	65.1	0.0114	0.116	1	1	CD276
Pubmed	12482850	Structural determinants of procryptdin recognition and cleavage by matrix metalloproteinase-7.	GeneRIF	0.0044	65.1	0.0114	0.116	1	1	MMP7
Pubmed	17570215	Bone morphogenetic protein 4 expressed in esophagitis induces a columnar phenotype in esophageal squamous cells.	GeneRIF	0.0044	65.1	0.0114	0.116	1	1	BMP4
Pubmed	23085087	Expression, purification and kinetic characterisation of human tissue transglutaminase.	GeneRIF	0.0044	65.1	0.0114	0.116	1	1	TGM2
Pubmed	16391898	No association between the microsatellite polymorphism (TTTTA)n in the promoter of the CYP11A gene and ovarian hyperstimulation syndrome.	GeneRIF	0.0044	65.1	0.0114	0.116	1	1	CYP11A1
Pubmed	25054912	Role of microRNA-150 and glycoprotein nonmetastatic melanoma protein B in angiogenesis during hyperoxia-induced neonatal lung injury.	Pubmed	0.0044	65.1	0.0114	0.116	1	1	GPNMB
Pubmed	19199799	Why do cysteine dioxygenase enzymes contain a 3-His ligand motif rather than a 2His/1Asp motif like most nonheme dioxygenases?	GeneRIF	0.0044	65.1	0.0114	0.116	1	1	CDO1
Pubmed	21300760	Pharmacokinetics of hedgehog pathway inhibitor vismodegib (GDC-0449) in patients with locally advanced or metastatic solid tumors: the role of alpha-1-acid glycoprotein binding.	GeneRIF	0.0044	65.1	0.0114	0.116	1	1	ORM1
Pubmed	12351521	The expression of secretory leukocyte protease inhibitor (SLPI) in the fallopian tube: SLPI protects the acrosome reaction of sperm from inhibitory effects of elastase.	GeneRIF	0.0044	65.1	0.0114	0.116	1	1	SLPI
Pubmed	14753734	Characterization of common genetic variants in cathepsin K and testing for association with bone mineral density in a large cohort of perimenopausal women from Scotland.	GeneRIF	0.0044	65.1	0.0114	0.116	1	1	CTSK
Pubmed	18670869	VDAC activation by the 18 kDa translocator protein (TSPO), implications for apoptosis.	GeneRIF	0.0044	65.1	0.0114	0.116	1	1	TSPO

Category	ID	Name	Source	p-value	q-value Bonferroni	q-value FDR B&H	q-value FDR B&Y	Hit Count in Query List	Hit Count in Genome	Hit in Query List
Pubmed	10406958	Structural and membrane-binding properties of saposin D.	Pubmed	0.0044	65.1	0.0114	0.116	1	1	PSAP
Pubmed	2464855	Hypomethylation and ADA gene expression in mouse CAK cells.	Pubmed	0.0044	65.1	0.0114	0.116	1	1	ADA
Pubmed	18294255	A polymorphism in the NPPA gene associates with asthma.	GeneRIF	0.0044	65.1	0.0114	0.116	1	1	NPPA
Pubmed	6546794	Sequence of human adenosine deaminase cDNA including the coding region and a small intron.	Pubmed	0.0044	65.1	0.0114	0.116	1	1	ADA
Pubmed	11939512	A novel beta-thalassemia mutation in an Asian Indian.	Pubmed	0.0044	65.1	0.0114	0.116	1	1	HBB
Pubmed	20686692	Altered actin centripetal retrograde flow in physically restricted immunological synapses.	GeneRIF	0.0044	65.1	0.0114	0.116	1	1	ACTA1
Pubmed	16848606	Simple model of sickle hemogloblin.	GeneRIF	0.0044	65.1	0.0114	0.116	1	1	HBB
Pubmed	14523183	Plasma concentrations of aminoterminal pro atrial natriuretic peptide and aminoterminal pro brain natriuretic peptide in healthy neonates: marked and rapid increase after birth.	Pubmed	0.0044	65.1	0.0114	0.116	1	1	NPPA
Pubmed	22965143	A critical role for phosphatidylinositol (3,4,5)-trisphosphate-dependent Rac exchanger 1 in endothelial junction disruption and vascular hyperpermeability.	GeneRIF	0.0044	65.1	0.0114	0.116	1	1	PREX1
Pubmed	22159897	Differences in metabolite burden of di(2-ethylhexyl)phthalate in pregnant and postpartum dams and their offspring in relation to drug-metabolizing enzymes in mice.	GeneRIF	0.0044	65.1	0.0114	0.116	1	1	LIPG
Pubmed	11180632	Structure of the asparagine-linked sugar chains of porcine kidney and human urine cerebroside sulfate activator protein.	Pubmed	0.0044	65.1	0.0114	0.116	1	1	PSAP
Pubmed	3631077	Genetic studies of low-abundance human plasma proteins. V. Evidence for a second orosomucoid structural locus (ORM2) expressed in plasma.	Pubmed	0.0044	65.1	0.0114	0.116	1	1	ORM2
Pubmed	11287620	Generation and analysis of mice lacking the chemokine fractalkine.	Pubmed	0.0044	65.1	0.0114	0.116	1	1	CX3CL1
Pubmed	19234087	PKA phosphorylation of HERG protein regulates the rate of channel synthesis.	GeneRIF	0.0044	65.1	0.0114	0.116	1	1	KCNH2
Pubmed	11723121	Localization of the transglutaminase cross-linking site in SVS III, a novel glycoprotein secreted from mouse seminal vesicle.	Pubmed	0.0044	65.1	0.0114	0.116	1	1	TGM2
Pubmed	12593854	A HERG current sustains a cardiac-type action potential in neuroblastoma S cells.	GeneRIF	0.0044	65.1	0.0114	0.116	1	1	KCNH2
Pubmed	3023820	The complete sequence of the mouse skeletal alpha actin gene reveals several conserved and inverted repeat sequences outside of the protein-coding region.	Pubmed	0.0044	65.1	0.0114	0.116	1	1	ACTA1
Pubmed	22382775	Transglutaminase 2 transamidation activity during first-phase insulin secretion: natural substrates in INS-1E.	GeneRIF	0.0044	65.1	0.0114	0.116	1	1	TGM2
Pubmed	19088204	Trop2 identifies a subpopulation of murine and human prostate basal cells with stem cell characteristics.	GeneRIF	0.0044	65.1	0.0114	0.116	1	1	TACSTD2
Pubmed	25060179	Relationship of serum MMP-7 levels for colorectal cancer: a meta-analysis.	Pubmed	0.0044	65.1	0.0114	0.116	1	1	MMP7
Pubmed	24454878	Dysferlin and other non-red cell proteins accumulate in the red cell membrane of Diamond-Blackfan Anemia patients.	Pubmed	0.0044	65.1	0.0114	0.116	1	1	DYSF
Pubmed	10470726	Corneodesmosin (MHC S) gene in guttate psoriasis.	Pubmed	0.0044	65.1	0.0114	0.116	1	1	CDSN
Pubmed	21246534	Uterine serous papillary carcinomas overexpress human trophoblast-cell-surface marker (Trop-2) and are highly sensitive to immunotherapy with hRS7, a humanized anti-Trop-2 monoclonal antibody.	GeneRIF	0.0044	65.1	0.0114	0.116	1	1	TACSTD2
Pubmed	12897745	Endothelial protein C receptor-dependent inhibition of human eosinophil chemotaxis by protein C.	GeneRIF	0.0044	65.1	0.0114	0.116	1	1	PROCR
Pubmed	15748811	Metalloproteinase-7 contributes to joint destruction in Staphylococcus aureus induced arthritis.	GeneRIF	0.0044	65.1	0.0114	0.116	1	1	MMP7
Pubmed	23895900	Matrix metalloproteinase7 -181A/G polymorphism is associated with increased cancer risk among high-quality studies: Evidence from a meta-analysis.	Pubmed	0.0044	65.1	0.0114	0.116	1	1	MMP7
Pubmed	19149873	Sickle cell trait (HbAS) and stunting in children below two years of age in an area of high malaria transmission.	GeneRIF	0.0044	65.1	0.0114	0.116	1	1	HBB
Pubmed	22861376	Structures of apo and product-bound human L-asparaginase: insights into the mechanism of autoproteolysis and substrate hydrolysis.	GeneRIF	0.0044	65.1	0.0114	0.116	1	1	ASPG
Pubmed	10619458	Early postnatal Müller cell death leads to retinal but not optic nerve degeneration in NSE-Hu-Bcl-2 transgenic mice.	Pubmed	0.0044	65.1	0.0114	0.116	1	1	NGFR
Pubmed	19562471	Tissue transglutaminase can be involved in airway inflammation of toluene diisocyanate-induced occupational asthma.	GeneRIF	0.0044	65.1	0.0114	0.116	1	1	TGM2
Pubmed	10794857	Cholinergic medial septum neurons do not degenerate in aged 129/Sv control or p75(NGFR)-/ mice.	Pubmed	0.0044	65.1	0.0114	0.116	1	1	NGFR
Pubmed	16425277	Polymorphic amino acids at codons 9 and 37 of HLA-DQB1 alleles may confer susceptibility to cervical cancer among Chinese women.	GeneRIF	0.0044	65.1	0.0114	0.116	1	1	HLA-DQB1
Pubmed	12439719	Quantitative analysis of human kallikrein gene 14 expression in breast tumours indicates association with poor prognosis.	GeneRIF	0.0044	65.1	0.0114	0.116	1	1	KLK14
Pubmed	14722738	Hydroxyurea in the treatment of major beta-thalassemia and importance of genetic screening.	GeneRIF	0.0044	65.1	0.0114	0.116	1	1	HBB
Pubmed	17709632	Common genetic variation in KCNH2 is associated with QT interval duration: the Framingham Heart Study.	GeneRIF	0.0044	65.1	0.0114	0.116	1	1	KCNH2
Pubmed	18504605	Characterization of hERG1a and hERG1b potassium channels-a possible role for hERG1b in the I (Kr) current.	GeneRIF	0.0044	65.1	0.0114	0.116	1	1	KCNH2
Pubmed	16823039	Crystal structures of saposins A and C.	GeneRIF	0.0044	65.1	0.0114	0.116	1	1	PSAP
Pubmed	10491211	Mutations of CTSK result in pycnodysostosis via a reduction in cathepsin K protein.	Pubmed	0.0044	65.1	0.0114	0.116	1	1	CTSK
Pubmed	21718816	The effects of the cathepsin K inhibitor odanacatib on osteoclastic bone resorption and vesicular trafficking.	Pubmed	0.0044	65.1	0.0114	0.116	1	1	CTSK

Category	ID	Name	Source	p-value	q-value Bonferroni	q-value FDR B&H	q-value FDR B&Y	Hit Count in Query List	Hit Count in Genome	Hit in Query List
Pubmed	21823154	Plasma tissue kallikrein level is negatively associated with incident and recurrent stroke: a multicenter case-control study in China.	GeneRIF	0.0044	65.1	0.0114	0.116	1	1	KLK1
Pubmed	21242518	Presentation of acquired peptide-MHC class II ligands by CD4+ regulatory T cells or helper cells differentially regulates antigen-specific CD4+ T cell response.	GeneRIF	0.0044	65.1	0.0114	0.116	1	1	HLA-DQB1
Pubmed	19037656	Disruption of the Gardos channel (KCa3.1) in mice causes subtle erythrocyte macrocytosis and progressive splenomegaly.	GeneRIF	0.0044	65.1	0.0114	0.116	1	1	KCNN4
Pubmed	16970880	Association of adenosine deaminase polymorphism with mild mental retardation.	GeneRIF	0.0044	65.1	0.0114	0.116	1	1	ADA
Pubmed	11978834	A novel role for p75NTR in subplate growth cone complexity and visual thalamocortical innervation.	Pubmed	0.0044	65.1	0.0114	0.116	1	1	NGFR
Pubmed	8146069	Expression of bone morphogenetic protein messenger RNAs by normal rat and human prostate and prostate cancer cells.	Pubmed	0.0044	65.1	0.0114	0.116	1	1	BMP4
Pubmed	18694993	Tumor cell and tumor vasculature expression of B7-H3 predict survival in clear cell renal cell carcinoma.	GeneRIF	0.0044	65.1	0.0114	0.116	1	1	CD276
Pubmed	17272306	BMP4 induces EMT and Rho GTPase activation in human ovarian cancer cells.	GeneRIF	0.0044	65.1	0.0114	0.116	1	1	BMP4
Pubmed	24606995	Mutations in Danish patients with long QT syndrome and the identification of a large founder family with p.F29L in KCNH2.	Pubmed	0.0044	65.1	0.0114	0.116	1	1	KCNH2
Pubmed	6671902	The first example of a deletion in the human alpha chain: hemoglobin Boyle Heights or alpha 2 6 (A4) Asp----to O beta 2.	Pubmed	0.0044	65.1	0.0114	0.116	1	1	HBA2
Pubmed	19088443	Biophysical characterization of the short QT mutation hERG-N588K reveals a mixed gain-and loss-of-function.	GeneRIF	0.0044	65.1	0.0114	0.116	1	1	KCNH2
Pubmed	19253956	Characterization of lipid binding specificities of dysferlin C2 domains reveals novel interactions with phosphoinositides.	GeneRIF	0.0044	65.1	0.0114	0.116	1	1	DYSF
Pubmed	20056711	Glycoprotein nonmetastatic melanoma protein b, a melanocytic cell marker, is a melanosome-specific and proteolytically released protein.	GeneRIF	0.0044	65.1	0.0114	0.116	1	1	GPNMB
Pubmed	24313005	[Sibling cases of severe infantile form of nemaline myopathy with ACTA1-gene mutation].	Pubmed	0.0044	65.1	0.0114	0.116	1	1	ACTA1
Pubmed	10427470	Large number of polymorphic nucleotides and a termination codon in the env gene of the endogenous human retrovirus ERV3.	Pubmed	0.0044	65.1	0.0114	0.116	1	1	ERV3-1
Pubmed	25224959	BMP4 inhibits breast cancer metastasis by blocking myeloid-derived suppressor cell activity.	Pubmed	0.0044	65.1	0.0114	0.116	1	1	BMP4
Pubmed	7835708	Cloning of a cDNA encoding murine tetranectin.	Pubmed	0.0044	65.1	0.0114	0.116	1	1	CLEC3B
Pubmed	1783368	[Integration and expression of human endogenous retrovirus].	Pubmed	0.0044	65.1	0.0114	0.116	1	1	ERV3-1
Pubmed	15468086	Actin mutations are one cause of congenital fibre type disproportion.	GeneRIF	0.0044	65.1	0.0114	0.116	1	1	ACTA1
Pubmed	21893365	Stimulation of B7-H3 (CD276) directs the differentiation of human marrow stromal cells to osteoblasts.	GeneRIF	0.0044	65.1	0.0114	0.116	1	1	CD276
Pubmed	11727832	Tissue kallikrein KLK1 is expressed de novo in endothelial cells and mediates relaxation of human umbilical veins.	GeneRIF	0.0044	65.1	0.0114	0.116	1	1	KLK1
Pubmed	18768527	Controlling alpha-globin: a review of alpha-globin expression and its impact on beta-thalassemia.	GeneRIF	0.0044	65.1	0.0114	0.116	1	1	HBA2
Pubmed	12187509	SPINK1 is a susceptibility gene for fibrocalculous pancreatic diabetes in subjects from the Indian subcontinent.	GeneRIF	0.0044	65.1	0.0114	0.116	1	1	SPINK1
Pubmed	20066577	A dispensable role for P450(scc) in the overproduction of aldosterone in aldosterone-producing adenoma and idiopathic hyperaldosteronism in patients with primary aldosteronism.	GeneRIF	0.0044	65.1	0.0114	0.116	1	1	CYP11A1
Pubmed	21777419	Role of tissue transglutaminase 2 in the acquisition of a mesenchymal-like phenotype in highly invasive A431 tumor cells.	GeneRIF	0.0044	65.1	0.0114	0.116	1	1	TGM2
Pubmed	23327998	Atrial natriuretic peptide suppresses Th17 development through regulation of cGMP-dependent protein kinase and PI3K-Akt signaling pathways.	GeneRIF	0.0044	65.1	0.0114	0.116	1	1	NPPA
Pubmed	16251447	The p75 neurotrophin receptor negatively modulates dendrite complexity and spine density in hippocampal neurons.	Pubmed	0.0044	65.1	0.0114	0.116	1	1	NGFR
Pubmed	20491643	Cigarette smoke decreases salivary 18 kDa translocator protein binding affinity--in association with oxidative stress.	GeneRIF	0.0044	65.1	0.0114	0.116	1	1	TSPO
Pubmed	7636234	Protection from T helper cell-mediated graft-versus host disease by the presence of an MHC class I alloantigen is associated with perturbation of MHC class II-restricted responses by class I-derived peptides.	Pubmed	0.0044	65.1	0.0114	0.116	1	1	HLA-DQB1
Pubmed	8418471	Tetranectin: a novel secretory protein from human monocytes.	Pubmed	0.0044	65.1	0.0114	0.116	1	1	CLEC3B
Pubmed	22236693	Genetic polymorphisms of nerve growth factor receptor (NGFR) and the risk of Alzheimer's disease.	GeneRIF	0.0044	65.1	0.0114	0.116	1	1	NGFR
Pubmed	17486497	Two new beta0-thalassemic mutations: a deletion (-CC) at codon 142 or overlapping codons 142-143, and an insertion (+T) at codon 45 or overlapping codons 44-45/45-46 of the beta-globin gene.	GeneRIF	0.0044	65.1	0.0114	0.116	1	1	HBB
Pubmed	8640859	Lack of MMTV superantigen presentation in MHC class II-deficient mice.	Pubmed	0.0044	65.1	0.0114	0.116	1	1	HLA-DQB1
Pubmed	20452215	Drosophila indirect flight muscle specific Act88F actin mutants as a model system for studying congenital myopathies of the human ACTA1 skeletal muscle actin gene.	GeneRIF	0.0044	65.1	0.0114	0.116	1	1	ACTA1
Pubmed	11804200	Polymorphism in the upstream regulatory region of the HLA-DQB1 gene in liver graft recipients.	GeneRIF	0.0044	65.1	0.0114	0.116	1	1	HLA-DQB1
Pubmed	18775317	Robust stability of the embryonic axial pattern requires a secreted scaffold for chordin degradation.	Pubmed	0.0044	65.1	0.0114	0.116	1	1	BMP4

Category	ID	Name	Source	p-value	q-value Bonferroni	q-value FDR B&H	q-value FDR B&Y	Hit Count in Query List	Hit Count in Genome	Hit in Query List
Pubmed	19002712	Participation of HERG channel cytoplasmic structures on regulation by the G protein-coupled TRH receptor.	GeneRIF	0.0044	65.1	0.0114	0.116	1	1	KCNH2
Pubmed	24643083	Determination of [(11)C]PBR28 binding potential in vivo: a first human TSPO blocking study.	Pubmed	0.0044	65.1	0.0114	0.116	1	1	TSPO
Pubmed	12939639	Mouse B7-H3 induces antitumor immunity.	Pubmed	0.0044	65.1	0.0114	0.116	1	1	CD276
Pubmed	18241860	Combined functional and positional gene information for the identification of susceptibility variants in celiac disease.	GeneRIF	0.0044	65.1	0.0114	0.116	1	1	HLA-DQB1
Pubmed	14657015	Protein kinase A regulates AKAP250 (gravin) scaffold binding to the beta2-adrenergic receptor.	GeneRIF	0.0044	65.1	0.0114	0.116	1	1	AKAP12
Pubmed	24375797	Transglutaminase 2-mediated serotonylation in pulmonary hypertension.	Pubmed	0.0044	65.1	0.0114	0.116	1	1	TGM2
Pubmed	16843677	The localization, trafficking and retrograde transport of BDNF bound to p75NTR in sympathetic neurons.	Pubmed	0.0044	65.1	0.0114	0.116	1	1	NGFR
Pubmed	15963762	Humoral autoimmunity in mice overexpressing B cell surface CD19: vital role for MHC class II.	Pubmed	0.0044	65.1	0.0114	0.116	1	1	HLA-DQB1
Pubmed	23060305	Smoothelin expression in the gastrointestinal tract: implication in colonic inertia.	Pubmed	0.0044	65.1	0.0114	0.116	1	1	SMTN
Pubmed	7959953	Genomic organization of IFI16, an interferon-inducible gene whose expression is associated with human myeloid cell differentiation: correlation of predicted protein domains with exon organization.	Pubmed	0.0044	65.1	0.0114	0.116	1	1	IFI16
Pubmed	17852802	Potassium channel KCNH2 K897T polymorphism and cardiac repolarization during exercise test: The Finnish Cardiovascular Study.	GeneRIF	0.0044	65.1	0.0114	0.116	1	1	KCNH2
Pubmed	8526877	Tissue-specific expression of two aldose reductase-like genes in mice: abundant expression of mouse vas deferens protein and fibroblast growth factor-regulated protein in the adrenal gland.	Pubmed	0.0044	65.1	0.0114	0.116	1	1	AKR1B10
Pubmed	12790766	Inhibin B, follicle stimulating hormone, luteinizing hormone, and estradiol and their relationship to the regulation of follicle development in girls during childhood and puberty.	Pubmed	0.0044	65.1	0.0114	0.116	1	1	INHBB
Pubmed	12527383	Differential incorporation of biotinylated polyamines by transglutaminase 2.	GeneRIF	0.0044	65.1	0.0114	0.116	1	1	TGM2
Pubmed	9361033	An adenosine deaminase (ADA) allele contains two newly identified deleterious mutations (Y97C and L106V) that interact to abolish enzyme activity.	Pubmed	0.0044	65.1	0.0114	0.116	1	1	ADA
Pubmed	14973143	Impairment of HERG K(+) channel function by tumor necrosis factor-alpha: role of reactive oxygen species as a mediator.	GeneRIF	0.0044	65.1	0.0114	0.116	1	1	KCNH2
Pubmed	23281611	Identification and molecular characterization of a novel 55-kb deletion recurrent in southern Italy: the Italian (G) ¿((A) ¿dß)°-thalassemia.	GeneRIF	0.0044	65.1	0.0114	0.116	1	1	HBB
Pubmed	6542248	Nucleotide sequences of the human and mouse atrial natriuretic factor genes.	Pubmed	0.0044	65.1	0.0114	0.116	1	1	NPPA
Pubmed	18511517	Cleavage of type II collagen by cathepsin K in human osteoarthritic cartilage.	GeneRIF	0.0044	65.1	0.0114	0.116	1	1	CTSK
Pubmed	22013116	Gluten-specific T cells cross-react between HLA-DQ8 and the HLA-DQ2a/DQ8ß transdimer.	GeneRIF	0.0044	65.1	0.0114	0.116	1	1	HLA-DQB1
Pubmed	6952552	Evolutionary relationship between HLA-DR antigen beta-chains, HLA-A, B, C antigen subunits and immunoglobulin chains.	Pubmed	0.0044	65.1	0.0114	0.116	1	1	HLA-DQB1
Pubmed	7908614	Congenital myopathy with fiber type disproportion: a family with a chromosomal translocation t(10;17) may indicate candidate gene regions.	Pubmed	0.0044	65.1	0.0114	0.116	1	1	ACTA1
Pubmed	9019408	Carboxypeptidase E is a regulated secretory pathway sorting receptor: genetic obliteration leads to endocrine disorders in Cpe(fat) mice.	Pubmed	0.0044	65.1	0.0114	0.116	1	1	CPE
Pubmed	7338468	Hemoglobin Miyashiro (beta 23[B5] val substituting for gly) an electrophoretically silent variant discovered by the isopropanol test.	Pubmed	0.0044	65.1	0.0114	0.116	1	1	HBB
Pubmed	19230186	[Immunogenetic aspects of the course and treatment of pulmonary tuberculosis].	GeneRIF	0.0044	65.1	0.0114	0.116	1	1	HLA-DQB1
Pubmed	12581188	Beta-globin gene cluster haplotypes and HbF levels are not the only modulators of sickle cell disease in Lebanon.	GeneRIF	0.0044	65.1	0.0114	0.116	1	1	HBB
Pubmed	24604282	Isolation of neural progenitor cells from the human adult subventricular zone based on expression of the cell surface marker CD271.	Pubmed	0.0044	65.1	0.0114	0.116	1	1	NGFR
Pubmed	21617844	Increased expression of aA-crystallin in human diabetic eye.	GeneRIF	0.0044	65.1	0.0114	0.116	1	1	CRYAA
Pubmed	21398523	Dermatopontin interacts with fibronectin, promotes fibronectin fibril formation, and enhances cell adhesion.	GeneRIF	0.0044	65.1	0.0114	0.116	1	1	DPT
Pubmed	18841163	Endothelial protein C receptor-assisted transport of activated protein C across the mouse blood-brain barrier.	GeneRIF	0.0044	65.1	0.0114	0.116	1	1	PROCR
Pubmed	24400123	Matrix metalloproteinase 7 is associated with symptomatic lesions and adverse events in patients with carotid atherosclerosis.	Pubmed	0.0044	65.1	0.0114	0.116	1	1	MMP7
Pubmed	10508587	Evolutionary rate of a gene affected by chromosomal position.	Pubmed	0.0044	65.1	0.0114	0.116	1	1	MID1
Pubmed	19574736	Mechanistic details of BK channel inhibition by the intermediate conductance, Ca2+-activated K channel.	GeneRIF	0.0044	65.1	0.0114	0.116	1	1	KCNN4
Pubmed	17406098	Single-nucleotide polymorphisms in the B7H3 gene are not associated with human autoimmune myasthenia gravis.	GeneRIF	0.0044	65.1	0.0114	0.116	1	1	CD276
Pubmed	12456713	Multiple connexins contribute to intercellular communication in the Xenopus embryo.	Pubmed	0.0044	65.1	0.0114	0.116	1	1	GJB3
Pubmed	22236996	Resistance to HSV-1 infection in the epithelium resides with the novel innate sensor, IFI-16.	GeneRIF	0.0044	65.1	0.0114	0.116	1	1	IFI16
Pubmed	11352721	Solution structure of the DNA binding domain of the human forkhead transcription factor AFX (FOXO4).	Pubmed	0.0044	65.1	0.0114	0.116	1	1	FOXO4
Pubmed	7898318	Localization of RC3 (neurogranin) in rat brain subcellular fractions.	Pubmed	0.0044	65.1	0.0114	0.116	1	1	NRGN

Category	ID	Name	Source	p-value	q-value Bonferroni	q-value FDR B&H	q-value FDR B&Y	Hit Count in Query List	Hit Count in Genome	Hit in Query List
Pubmed	22967270	Inhibition of Nogo-66 receptor 1 enhances recovery of cognitive function after traumatic brain injury in mice.	GeneRIF	0.0044	65.1	0.0114	0.116	1	1	RTN4R
Pubmed	9215686	Missense mutations in the human glutathione synthetase gene result in severe metabolic acidosis, 5-oxoprolinuria, hemolytic anemia and neurological dysfunction.	Pubmed	0.0044	65.1	0.0114	0.116	1	1	GSS
Pubmed	11854117	Increased risk of arrhythmic events in long-QT syndrome with mutations in the pore region of the human ether-a-go-go-related gene potassium channel.	GeneRIF	0.0044	65.1	0.0114	0.116	1	1	KCNH2
Pubmed	19295543	Genetic association of HLA DQB1 with CD4+CD25+(high) T-cell apoptosis in type 1 diabetes.	GeneRIF	0.0044	65.1	0.0114	0.116	1	1	HLA-DQB1
Pubmed	16107560	CD74 is a member of the regulated intramembrane proteolysis-processed protein family.	GeneRIF	0.0044	65.1	0.0114	0.116	1	1	CD74
Pubmed	10640747	Neuronal fractalkine expression in HIV-1 encephalitis: roles for macrophage recruitment and neuroprotection in the central nervous system.	Pubmed	0.0044	65.1	0.0114	0.116	1	1	CX3CL1
Pubmed	22322207	Correlation of serum CX3CL1 level with disease activity in adult-onset Still's disease and significant involvement in hemophagocytic syndrome.	GeneRIF	0.0044	65.1	0.0114	0.116	1	1	CX3CL1
Pubmed	4852224	Hb J Sicilia: beta 65 (E9) Lys-Asn, a beta homologue of Hb Zambia.	Pubmed	0.0044	65.1	0.0114	0.116	1	1	HBB
Pubmed	10773105	Growth parameters in the epiphyseal cartilage of brachymorphic (bm/bm) mice.	Pubmed	0.0044	65.1	0.0114	0.116	1	1	PAPSS2
Pubmed	18690032	Mutational analysis of block and facilitation of HERG current by a class III anti-arrhythmic agent, nifekalant.	GeneRIF	0.0044	65.1	0.0114	0.116	1	1	KCNH2
Pubmed	18182238	Protective actions of human tissue kallikrein gene in transgenic rat hearts.	GeneRIF	0.0044	65.1	0.0114	0.116	1	1	KLK1
Pubmed	21784485	B7-H3 over expression in prostate cancer promotes tumor cell progression.	GeneRIF	0.0044	65.1	0.0114	0.116	1	1	CD276
Pubmed	11186266	Hb Clinico-Madrid [alpha90(FG2)Lys-->Arg]: a new hemoglobin mutation in the alpha2-globin gene.	Pubmed	0.0044	65.1	0.0114	0.116	1	1	HBA2
Pubmed	22863596	[Expression of B7-H3 costimulatory molecule in peripheral blood of myasthenia gravis patients].	GeneRIF	0.0044	65.1	0.0114	0.116	1	1	CD276
Pubmed	14506941	Reduced p75NTR expression delays disease onset only in female mice of a transgenic model of familial amyotrophic lateral sclerosis.	GeneRIF	0.0044	65.1	0.0114	0.116	1	1	NGFR
Pubmed	15481894	A novel alpha-thalassemia nonsense mutation in codon 23 of the alpha2-globin gene (GAG-->TAG) in a Tunisian family.	Pubmed	0.0044	65.1	0.0114	0.116	1	1	HBA2
Pubmed	19693293	A novel TACSTD2 mutation identified in two Chinese brothers with gelatinous drop-like corneal dystrophy.	GeneRIF	0.0044	65.1	0.0114	0.116	1	1	TACSTD2
Pubmed	18281513	Identification of Trop-2 as an oncogene and an attractive therapeutic target in colon cancers.	GeneRIF	0.0044	65.1	0.0114	0.116	1	1	TACSTD2
Pubmed	2320430	Sequence of a murine cDNA, pcp-4, that encodes the homolog of the rat brain-specific antigen PEP-19.	Pubmed	0.0044	65.1	0.0114	0.116	1	1	PCP4
Pubmed	21098279	Recognition of tandem PxxP motifs as a unique Src homology 3-binding mode triggers pathogen-driven actin assembly.	GeneRIF	0.0044	65.1	0.0114	0.116	1	1	BAIAP2L1
Pubmed	25814530	B7-H3 expression in donor T cells and host cells negatively regulates acute graft-versus-host disease lethality.	Pubmed	0.0044	65.1	0.0114	0.116	1	1	CD276
Pubmed	24660172	Frequencies of HLA-DQ2 and HLA-DQ8 haplotypes in Czech and Slovak coeliac patients and the healthy population.	Pubmed	0.0044	65.1	0.0114	0.116	1	1	HLA-DQB1
Pubmed	19500648	Axonal regeneration of optic nerve after crush in Nogo66 receptor knockout mice.	GeneRIF	0.0044	65.1	0.0114	0.116	1	1	RTN4R
Pubmed	20237149	Thromboxane prostanoid receptor stimulation induces shedding of the transmembrane chemokine CX3CL1 yet enhances CX3CL1-dependent leukocyte adhesion.	GeneRIF	0.0044	65.1	0.0114	0.116	1	1	CX3CL1
Pubmed	16428763	The Helicobacter pylori urease B subunit binds to CD74 on gastric epithelial cells and induces NF-kappaB activation and interleukin-8 production.	GeneRIF	0.0044	65.1	0.0114	0.116	1	1	CD74
Pubmed	15650263	Secretory leukocyte protease inhibitor in cancer development.	GeneRIF	0.0044	65.1	0.0114	0.116	1	1	SLPI
Pubmed	22042794	Angiopoietin-like protein 2 is an important facilitator of inflammatory carcinogenesis and metastasis.	GeneRIF	0.0044	65.1	0.0114	0.116	1	1	ANGPTL2
Pubmed	17641055	Adenosine deamination sustains dendritic cell activation in inflammation.	Pubmed	0.0044	65.1	0.0114	0.116	1	1	ADA
Pubmed	18428016	Hemoglobin variants, hematological parameters and beta-globin gene cluster haplotypes in an isolated Amerindian group from the Orinoco River Delta.	GeneRIF	0.0044	65.1	0.0114	0.116	1	1	HBB
Pubmed	1988759	Hemoglobin Columbia Missouri or alpha 2[88 (F9) Ala----Val]beta 2: a new high-oxygen-affinity hemoglobin that causes erythrocytosis.	Pubmed	0.0044	65.1	0.0114	0.116	1	1	HBA2
Pubmed	21551320	Expression of Trop2 cell surface glycoprotein in normal and tumor tissues: potential implications as a cancer therapeutic target.	GeneRIF	0.0044	65.1	0.0114	0.116	1	1	TACSTD2
Pubmed	12651621	Transglutaminase type II plays a protective role in hepatic injury.	GeneRIF	0.0044	65.1	0.0114	0.116	1	1	TGM2
Pubmed	23877317	TGM2 inhibition attenuates ID1 expression in CD44-high glioma-initiating cells.	Pubmed	0.0044	65.1	0.0114	0.116	1	1	TGM2
Pubmed	24067471	Association of HLA-DQB1*0501 with scleroderma and its clinical features in Chinese population.	Pubmed	0.0044	65.1	0.0114	0.116	1	1	HLA-DQB1
Pubmed	15211519	Exploring structural features of the interaction between the scorpion toxinCnErg1 and ERG K+ channels.	GeneRIF	0.0044	65.1	0.0114	0.116	1	1	KCNH2
Pubmed	20580289	Novel large deletions in the human alpha-globin gene cluster: Clarifying the HS-40 long-range regulatory role in the native chromosome environment.	GeneRIF	0.0044	65.1	0.0114	0.116	1	1	HBA2
Pubmed	11901813	Human tissue kallikrein gene family: a rich source of novel disease biomarkers.	Pubmed	0.0044	65.1	0.0114	0.116	1	1	KLK15
Pubmed	24778599	Biological functionalities of transglutaminase 2 and the possibility of its compensation by other members of the transglutaminase family.	Pubmed	0.0044	65.1	0.0114	0.116	1	1	TGM2

Category	ID	Name	Source	p-value	q-value Bonferroni	q-value FDR B&H	q-value FDR B&Y	Hit Count in Query List	Hit Count in Genome	Hit in Query List
Pubmed	1062801	Hemoglobin Wayne: a frameshift mutation detected in human hemoglobin alpha chains.	Pubmed	0.0044	65.1	0.0114	0.116	1	1	HBA2
Pubmed	25110884	Increased secretory leukocyte protease inhibitor (SLPI) production by highly metastatic mouse breast cancer cells.	Pubmed	0.0044	65.1	0.0114	0.116	1	1	SLPI
Pubmed	25406832	Positron emission tomography and functional characterization of a complete PBR/TSPO knockout.	Pubmed	0.0044	65.1	0.0114	0.116	1	1	TSPO
Pubmed	17183611	Anti-OX40 stimulation in vivo enhances CD8+ memory T cell survival and significantly increases recall responses.	Pubmed	0.0044	65.1	0.0114	0.116	1	1	HLA-DQB1
Pubmed	7686953	Trichohyalin: a structural protein of hair, tongue, nail, and epidermis.	Pubmed	0.0044	65.1	0.0114	0.116	1	1	TCHH
Pubmed	19592662	Diminished intracellular invariant chain expression after vaccinia virus infection.	GeneRIF	0.0044	65.1	0.0114	0.116	1	1	CD74
Pubmed	8258301	Assignment of the human skeletal muscle alpha actin gene (ACTA1) to 1q42 by fluorescence in situ hybridisation.	Pubmed	0.0044	65.1	0.0114	0.116	1	1	ACTA1
Pubmed	22161019	Imatinib has the potential to exert its antileukemia effects by down-regulating hERG1 K+ channels in chronic myelogenous leukemia.	GeneRIF	0.0044	65.1	0.0114	0.116	1	1	KCNH2
Pubmed	23136848	The human leucocyte antigen DQB1*0602 allele is associated with electroencephelograph differences in individuals with obstructive sleep apnoea syndrome.	GeneRIF	0.0044	65.1	0.0114	0.116	1	1	HLA-DQB1
Pubmed	15342690	Role of N-linked glycosylation in the secretion and activity of endothelial lipase.	GeneRIF	0.0044	65.1	0.0114	0.116	1	1	LIPG
Pubmed	25016529	Elevation of human ERV3-1 env protein expression in colorectal cancer.	Pubmed	0.0044	65.1	0.0114	0.116	1	1	ERV3-1
Pubmed	24279918	Calmodulin-like skin protein level increases in the differentiated epidermal layers in atopic dermatitis.	Pubmed	0.0044	65.1	0.0114	0.116	1	1	CALML5
Pubmed	17600367	The neurotrophin receptor p75NTR in Schwann cells is implicated in remyelination and motor recovery after peripheral nerve injury.	Pubmed	0.0044	65.1	0.0114	0.116	1	1	NGFR
Pubmed	12730457	Regulation of zinc metabolism and genomic outcomes.	Pubmed	0.0044	65.1	0.0114	0.116	1	1	SLC39A2
Pubmed	9843486	Structure of a trimeric domain of the MHC class II-associated chaperonin and targeting protein Ii.	Pubmed	0.0044	65.1	0.0114	0.116	1	1	CD74
Pubmed	24634127	Lack of association between a common polymorphism of the endothelial lipase gene and early-onset coronary artery disease in a Chinese Han population.	Pubmed	0.0044	65.1	0.0114	0.116	1	1	LIPG
Pubmed	20816669	MHC class II-associated invariant chain (Ii) modulates dendritic cells-derived microvesicles (DCMV)-mediated activation of microglia.	GeneRIF	0.0044	65.1	0.0114	0.116	1	1	CD74
Pubmed	16762919	Regulation of AKAP-membrane interactions by calcium.	GeneRIF	0.0044	65.1	0.0114	0.116	1	1	AKAP12
Pubmed	15305334	Prosaptide TX14A stimulates growth, migration, and invasion and activates the Raf-MEK-ERK-RSK-Elk-1 signaling pathway in prostate cancer cells.	GeneRIF	0.0044	65.1	0.0114	0.116	1	1	PSAP
Pubmed	18441507	The association between an increased level of gamma-glutamyl transferase and systolic blood pressure in diabetic subjects.	GeneRIF	0.0044	65.1	0.0114	0.116	1	1	GGT1
Pubmed	22556334	Macrophage-derived angiopoietin-like protein 2 accelerates development of abdominal aortic aneurysm.	GeneRIF	0.0044	65.1	0.0114	0.116	1	1	ANGPTL2
Pubmed	20938172	ß-globin gene cluster haplotypes in a cohort of 221 children with sickle cell anemia or Sß°-thalassemia and their association with clinical and hematological features.	GeneRIF	0.0044	65.1	0.0114	0.116	1	1	HBB
Pubmed	12727021	Human keratinocytes express fractalkine/CX3CL1.	GeneRIF	0.0044	65.1	0.0114	0.116	1	1	CX3CL1
Pubmed	17525707	Atrial natriuretic peptide (ANP) gene promoter variant and increased susceptibility to early development of hypertension in humans.	GeneRIF	0.0044	65.1	0.0114	0.116	1	1	NPPA
Pubmed	16417576	Altered neuropeptide processing in prefrontal cortex of Cpe (fat/fat) mice: implications for neuropeptide discovery.	Pubmed	0.0044	65.1	0.0114	0.116	1	1	CPE
Pubmed	23977274	Multimodal MRI and (31)P-MRS investigations of the ACTA1(Asp286Gly) mouse model of nemaline myopathy provide evidence of impaired in vivo muscle function, altered muscle structure and disturbed energy metabolism.	Pubmed	0.0044	65.1	0.0114	0.116	1	1	ACTA1
Pubmed	19851043	Imaging of peripheral-type benzodiazepine receptor in tumor: carbon ion irradiation reduced the uptake of a positron emission tomography ligand [11C]DAC in tumor.	GeneRIF	0.0044	65.1	0.0114	0.116	1	1	TSPO
Pubmed	22050847	Imaging brain microglial activation using positron emission tomography and translocator protein-specific radioligands.	GeneRIF	0.0044	65.1	0.0114	0.116	1	1	TSPO
Pubmed	20333377	Clinical significance and regulation of the costimulatory molecule B7-H3 in human colorectal carcinoma.	GeneRIF	0.0044	65.1	0.0114	0.116	1	1	CD276
Pubmed	18477014	Pan-neurotrophin receptor p75NTR expression is strongly induced in lesional atopic mast cells.	GeneRIF	0.0044	65.1	0.0114	0.116	1	1	NGFR
Pubmed	21177251	The role of monoubiquitination in endocytic degradation of human ether-a-go-go-related gene (hERG) channels under low K+ conditions.	GeneRIF	0.0044	65.1	0.0114	0.116	1	1	KCNH2
Pubmed	10200993	Mesangial cells from diabetic NOD mice constitutively express increased density of atrial natriuretic peptide C receptors.	Pubmed	0.0044	65.1	0.0114	0.116	1	1	NPPA
Pubmed	21829356	Structural basis of chemokine sequestration by CrmD, a poxvirus-encoded tumor necrosis factor receptor.	Pubmed	0.0044	65.1	0.0114	0.116	1	1	CX3CL1
Pubmed	19141362	Atrial natriuretic peptide polymorphisms, hydrochlorothiazide and urinary potassium excretion.	GeneRIF	0.0044	65.1	0.0114	0.116	1	1	NPPA
Pubmed	16427282	Autosomal dominant nemaline myopathy with intranuclear rods due to mutation of the skeletal muscle ACTA1 gene: clinical and pathological variability within a kindred.	GeneRIF	0.0044	65.1	0.0114	0.116	1	1	ACTA1
Pubmed	10520227	Localization of the human 64kD autoantigen D1 to myofibrils in a subset of extraocular muscle fibers.	GeneRIF	0.0044	65.1	0.0114	0.116	1	1	LMOD1

Category	ID	Name	Source	p-value	q-value Bonferroni	q-value FDR B&H	q-value FDR B&Y	Hit Count in Query List	Hit Count in Genome	Hit in Query List
Pubmed	12639246	Increased intrathecal release of soluble fractalkine in HIV-infected patients.	GeneRIF	0.0044	65.1	0.0114	0.116	1	1	CX3CL1
Pubmed	21072679	BMP-4 suppresses progesterone production by inhibiting histone H3 acetylation of StAR in bovine granulosa cells in vitro.	GeneRIF	0.0044	65.1	0.0114	0.116	1	1	BMP4
Pubmed	23746069	A novel nonsense mutation in the CDSN gene underlying hypotrichosis simplex of the scalp in a Chinese family.	Pubmed	0.0044	65.1	0.0114	0.116	1	1	CDSN
Pubmed	22414869	Modafinil inhibits K(Ca)3.1 currents and muscle contraction via a cAMP-dependent mechanism.	GeneRIF	0.0044	65.1	0.0114	0.116	1	1	KCNN4
Pubmed	22618232	Preferential binding of IFI16 protein to cruciform structure and superhelical DNA.	GeneRIF	0.0044	65.1	0.0114	0.116	1	1	IFI16
Pubmed	23979406	Increased expression of Trop2 correlates with poor survival in extranodal NK/T cell lymphoma, nasal type.	Pubmed	0.0044	65.1	0.0114	0.116	1	1	TACSTD2
Pubmed	15120823	Postmortem molecular screening in unexplained sudden death.	GeneRIF	0.0044	65.1	0.0114	0.116	1	1	KCNH2
Pubmed	24767306	Cathepsin K analysis in a pycnodysostosis cohort: demographic, genotypic and phenotypic features.	Pubmed	0.0044	65.1	0.0114	0.116	1	1	CTSK
Pubmed	22854970	Crystal structure and functional characterization of the complement regulator mannose-binding lectin (MBL)/ficolin-associated protein-1 (MAP-1).	GeneRIF	0.0044	65.1	0.0114	0.116	1	1	MASP1
Pubmed	15481888	Molecular characterization of Hb Val de Marne [alpha133(H16)Ser-->Arg; AGC-->AGA; (alpha2)] in a Chinese family.	Pubmed	0.0044	65.1	0.0114	0.116	1	1	HBA2
Pubmed	19395425	Orosomucoid influences the response to antidepressants in major depressive disorder.	GeneRIF	0.0044	65.1	0.0114	0.116	1	1	ORM1
Pubmed	16338459	Tissue transglutaminase expression and activity in placenta.	GeneRIF	0.0044	65.1	0.0114	0.116	1	1	TGM2
Pubmed	18266765	Cryptic splicing sites are differentially utilized in vivo.	GeneRIF	0.0044	65.1	0.0114	0.116	1	1	HBB
Pubmed	8672487	Probing the functional role of two conserved active site aspartates in mouse adenosine deaminase.	Pubmed	0.0044	65.1	0.0114	0.116	1	1	ADA
Pubmed	22764740	A novel missense mutation causing a G487R substitution in the S2-S3 loop of human ether-à-go-go-related gene channel.	GeneRIF	0.0044	65.1	0.0114	0.116	1	1	KCNH2
Pubmed	21640091	Analysis of serum adenosine deaminase (ADA) and ADA1 and ADA2 isoenzyme activities in HIV positive and HIV-HBV co-infected patients.	GeneRIF	0.0044	65.1	0.0114	0.116	1	1	ADA
Pubmed	6151297	Morphological studies of the epiphyseal growth zone in the brachymorphic (bm/bm) mouse.	Pubmed	0.0044	65.1	0.0114	0.116	1	1	PAPSS2
Pubmed	23152408	Effects of cathepsin K deficiency on intercellular junction proteins, luminal mucus layers, and extracellular matrix constituents in the mouse colon.	GeneRIF	0.0044	65.1	0.0114	0.116	1	1	CTSK
Pubmed	24403225	Characterization of hERG1 channel role in mouse colorectal carcinogenesis.	Pubmed	0.0044	65.1	0.0114	0.116	1	1	KCNH2
Pubmed	19380136	Physical inactivity interacts with an endothelial lipase polymorphism to modulate high density lipoprotein cholesterol in the GOLDN study.	GeneRIF	0.0044	65.1	0.0114	0.116	1	1	LIPG
Pubmed	635569	The glycosylation of hemoglobin: relevance to diabetes mellitus.	Pubmed	0.0044	65.1	0.0114	0.116	1	1	HBB
Pubmed	22511748	Rare, nonsynonymous variant in the smooth muscle-specific isoform of myosin heavy chain, MYH11, R247C, alters force generation in the aorta and phenotype of smooth muscle cells.	GeneRIF	0.0044	65.1	0.0114	0.116	1	1	MYH11
Pubmed	20407432	KCNN4 gene variant is associated with ileal Crohn's Disease in the Australian and New Zealand population.	GeneRIF	0.0044	65.1	0.0114	0.116	1	1	KCNN4
Pubmed	21357734	hERG potassium channel gating is mediated by N- and C-terminal region interactions.	GeneRIF	0.0044	65.1	0.0114	0.116	1	1	KCNH2
Pubmed	17436019	Intragenic deletion in the LARGE gene causes Walker-Warburg syndrome.	Pubmed	0.0044	65.1	0.0114	0.116	1	1	LARGE
Pubmed	12869545	A thyroid hormone receptor alpha gene mutation (P398H) is associated with visceral adiposity and impaired catecholamine-stimulated lipolysis in mice.	GeneRIF	0.0044	65.1	0.0114	0.116	1	1	THRA
Pubmed	10598905	Secretory leukocyte protease inhibitor (SLPI): oxidation of SLPI does not explain its variable anti-HIV activity.	Pubmed	0.0044	65.1	0.0114	0.116	1	1	SLPI
Pubmed	17909180	Chymotryptic specificity determinants in the 1.0 A structure of the zinc-inhibited human tissue kallikrein 7.	GeneRIF	0.0044	65.1	0.0114	0.116	1	1	KLK7
Pubmed	19816200	Bone morphogenetic protein 4 and its receptors are expressed in the leptomeninges and meningiomas and signal via the Smad pathway.	GeneRIF	0.0044	65.1	0.0114	0.116	1	1	BMP4
Pubmed	18632639	Clinical and biological significance of tissue transglutaminase in ovarian carcinoma.	GeneRIF	0.0044	65.1	0.0114	0.116	1	1	TGM2
Pubmed	23777246	Bone marrow transplantation in dysferlin-deficient mice results in a mild functional improvement.	Pubmed	0.0044	65.1	0.0114	0.116	1	1	DYSF
Pubmed	17181544	Carrier frequency of a nonsense mutation in the adenosine deaminase (ADA) gene implies a high incidence of ADA-deficient severe combined immunodeficiency (SCID) in Somalia and a single, common haplotype indicates common ancestry.	GeneRIF	0.0044	65.1	0.0114	0.116	1	1	ADA
Pubmed	23349064	Inhibin B in plasma samples from male volunteer panel selected for health but not fertility: sources of variability.	GeneRIF	0.0044	65.1	0.0114	0.116	1	1	INHBB
Pubmed	11170993	Secretory leukocyte protease inhibitor in vaginal fluids and perinatal human immunodeficiency virus type 1 transmission.	Pubmed	0.0044	65.1	0.0114	0.116	1	1	SLPI
Pubmed	24086649	Overexpression of TROP2 predicts poor prognosis of patients with cervical cancer and promotes the proliferation and invasion of cervical cancer cells by regulating ERK signaling pathway.	Pubmed	0.0044	65.1	0.0114	0.116	1	1	TACSTD2
Pubmed	24603819	Transglutaminase 2: a new player in bronchopulmonary dysplasia?	Pubmed	0.0044	65.1	0.0114	0.116	1	1	TGM2
Pubmed	8718692	Identification of a new high oxygen affinity hemoglobin variant: Hb Aurora [beta 139(H17) Asn-->Tyr].	Pubmed	0.0044	65.1	0.0114	0.116	1	1	HBB

Category	ID	Name	Source	p-value	q-value Bonferroni	q-value FDR B&H	q-value FDR B&Y	Hit Count in Query List	Hit Count in Genome	Hit in Query List
Pubmed	18941196	Anterior pituitary progenitor cells express costimulatory molecule 4Ig-B7-H3.	GeneRIF	0.0044	65.1	0.0114	0.116	1	1	CD276
Pubmed	16146723	Tissue transglutaminase: from biological glue to cell survival cues.	GeneRIF	0.0044	65.1	0.0114	0.116	1	1	TGM2
Pubmed	24349233	Localization of microfibrillar-associated protein 4 (MFAP4) in human tissues: clinical evaluation of serum MFAP4 and its association with various cardiovascular conditions.	Pubmed	0.0044	65.1	0.0114	0.116	1	1	MFAP4
Pubmed	18561261	The TGM2 gene is associated with schizophrenia in a British population.	GeneRIF	0.0044	65.1	0.0114	0.116	1	1	TGM2
Pubmed	23001131	Transglutaminase 2 exacerbates experimental autoimmune encephalomyelitis through positive regulation of encephalitogenic T cell differentiatio and inflammation.	Pubmed	0.0044	65.1	0.0114	0.116	1	1	TGM2
Pubmed	19493611	Novel DYSF mutations in Thai patients with distal myopathy.	GeneRIF	0.0044	65.1	0.0114	0.116	1	1	DYSF
Pubmed	25532697	Structural analysis of sulindac as an inhibitor of aldose reductase and AKR1B10.	Pubmed	0.0044	65.1	0.0114	0.116	1	1	AKR1B10
Pubmed	17849044	Polymorphisms in the endothelial protein C receptor gene and thrombophilia.	GeneRIF	0.0044	65.1	0.0114	0.116	1	1	PROCR
Pubmed	24453475	Identification of proteins that interact with alpha A crystallin using a human proteome microarray.	Pubmed	0.0044	65.1	0.0114	0.116	1	1	CRYAA
Pubmed	11779849	An mRNA splice variant of the AFX gene with altered transcriptional activity.	GeneRIF	0.0044	65.1	0.0114	0.116	1	1	FOXO4
Pubmed	8554069	Analysis of a splice-site mutation in the sap-precursor gene of a patient with metachromatic leukodystrophy.	Pubmed	0.0044	65.1	0.0114	0.116	1	1	PSAP
Pubmed	22955423	Molecular dynamics simulations reveal structural instability of human trypsin inhibitor upon D50E and Y54H mutations.	GeneRIF	0.0044	65.1	0.0114	0.116	1	1	SPINK1
Pubmed	23275124	CD68 expression in aortocoronary saphenous vein bypass grafts.	Pubmed	0.0044	65.1	0.0114	0.116	1	1	CD68
Pubmed	19147803	Pancreatic secretory trypsin inhibitor is a major motogenic and protective factor in human breast milk.	GeneRIF	0.0044	65.1	0.0114	0.116	1	1	SPINK1
Pubmed	19338743	Cathepsin K: a therapeutic target for bone diseases.	GeneRIF	0.0044	65.1	0.0114	0.116	1	1	CTSK
Pubmed	1807098	[Adenosine deaminase. A pluridisciplinary enzyme].	Pubmed	0.0044	65.1	0.0114	0.116	1	1	ADA
Pubmed	20558759	Membrane blebbing as an assessment of functional rescue of dysferlin-deficient human myotubes via nonsense suppression.	GeneRIF	0.0044	65.1	0.0114	0.116	1	1	DYSF
Pubmed	23135699	SMB myosin heavy chain knockout enhances tonic contraction and reduces the rate of force generatior in ileum and stomach antrum.	Pubmed	0.0044	65.1	0.0114	0.116	1	1	MYH11
Pubmed	1354271	Tetranectin, a plasminogen kringle 4-binding protein. Cloning and gene expression pattern in human colon cancer.	Pubmed	0.0044	65.1	0.0114	0.116	1	1	CLEC3B
Pubmed	20642332	Identification of the linkage of a 1.357 KB beta-globin gene deletion and A gamma-globin gene triplication in a Chinese family.	GeneRIF	0.0044	65.1	0.0114	0.116	1	1	HBB
Pubmed	3654265	A new unstable and low oxygen affinity hemoglobin variant: Hb J-Auckland [beta 25(B7)Gly----Asp].	Pubmed	0.0044	65.1	0.0114	0.116	1	1	HBB
Pubmed	15666592	The ScaI gene polymorphism of the atrial natriuretic factor and essential arterial hypertension in childhood.	GeneRIF	0.0044	65.1	0.0114	0.116	1	1	NPPA
Pubmed	13129853	Reversal of charge selectivity in cation or anion-selective epithelial lines by expression of different claudins.	Pubmed	0.0044	65.1	0.0114	0.116	1	1	CLDN15
Pubmed	17524563	Glucocorticoid modulation of atrial natriuretic peptide, oxytocin, vasopressin and Fos expression in response to osmotic, angiotensinergic and cholinergic stimulation.	GeneRIF	0.0044	65.1	0.0114	0.116	1	1	NPPA
Pubmed	20618995	Effects of rituximab in two patients with dysferlin-deficient muscular dystrophy.	GeneRIF	0.0044	65.1	0.0114	0.116	1	1	DYSF
Pubmed	22432041	Genome-wide association scan identifies a risk locus for preeclampsia on 2q14, near the inhibin, beta B gene.	GeneRIF	0.0044	65.1	0.0114	0.116	1	1	INHBB
Pubmed	8688429	Active site of 5-aminolevulinate synthase resides at the subunit interface. Evidence from in vivo heterodimer formation.	Pubmed	0.0044	65.1	0.0114	0.116	1	1	ALAS2
Pubmed	19912309	alpha-thalassaemia masked by beta gene defects and a new polyadenylation site mutation on the alpha2-globin gene.	GeneRIF	0.0044	65.1	0.0114	0.116	1	1	HBA2
Pubmed	16409859	[Microsatellite polymorphism of (tttta) n in the promoter of CYP11a gene in Chinese women with polycystic ovary syndrome].	GeneRIF	0.0044	65.1	0.0114	0.116	1	1	CYP11A1
Pubmed	24001019	Human junctophilin-2 undergoes a structural rearrangement upon binding PtdIns(3,4,5)P3 and the S101R mutation identified in hypertrophic cardiomyopathy obviates this response.	Pubmed	0.0044	65.1	0.0114	0.116	1	1	JPH2
Pubmed	20544339	HERG1 channelopathies.	GeneRIF	0.0044	65.1	0.0114	0.116	1	1	KCNH2
Pubmed	10998642	Smooth muscle myosin heavy chain isoforms and their role in muscle physiology.	Pubmed	0.0044	65.1	0.0114	0.116	1	1	MYH11
Pubmed	16584192	Effect of site-directed mutagenesis of methylglyoxal-modifiable arginine residues on the structure and chaperone function of human alphaA crystallin.	GeneRIF	0.0044	65.1	0.0114	0.116	1	1	CRYAA
Pubmed	16258000	Partially adenosine deaminase-deficient mice develop pulmonary fibrosis in association with adenosine elevations.	Pubmed	0.0044	65.1	0.0114	0.116	1	1	ADA
Pubmed	18654885	Hb Montreal II: a novel elongated beta-globin variant caused by a frameshift mutation [beta142 (-C)].	Pubmed	0.0044	65.1	0.0114	0.116	1	1	HBB
Pubmed	20473942	Elevated serum matrix metalloproteinase 7 levels predict poor prognosis after radical prostatectomy.	GeneRIF	0.0044	65.1	0.0114	0.116	1	1	MMP7
Pubmed	17828519	Dysfunction of dysferlin-deficient hearts.	GeneRIF	0.0044	65.1	0.0114	0.116	1	1	DYSF
Pubmed	10956626	Hypoxia reduces atrial natriuretic peptide clearance receptor gene expression in ANP knockout mice.	Pubmed	0.0044	65.1	0.0114	0.116	1	1	NPPA
Pubmed	11139392	Cloning and functional expression of rat kidney dipeptidyl peptidase II.	Pubmed	0.0044	65.1	0.0114	0.116	1	1	DPP7
Pubmed	15532024	Mutations of the Nogo-66 receptor (RTN4R) gene in schizophrenia.	GeneRIF	0.0044	65.1	0.0114	0.116	1	1	RTN4R
Pubmed	25237067	Holo-retinol-binding protein and its receptor STRA6 drive oncogenic transformation.	Pubmed	0.0044	65.1	0.0114	0.116	1	1	STRA6

Category	ID	Name	Source	p-value	q-value Bonferroni	q-value FDR B&H	q-value FDR B&Y	Hit Count in Query List	Hit Count in Genome	Hit in Query List
Pubmed	22319575	Activation and inhibition of transglutaminase 2 in mice.	GeneRIF	0.0044	65.1	0.0114	0.116	1	1	TGM2
Pubmed	12874244	Murine macrophages produce secretory leukocyte protease inhibitor during clearance of apoptotic cells: implications for resolution of the inflammatory response.	GeneRIF	0.0044	65.1	0.0114	0.116	1	1	SLPI
Pubmed	9042565	Analysis of cutaneous sensory neurons in transgenic mice lacking the low affinity neurotrophin receptor p75.	Pubmed	0.0044	65.1	0.0114	0.116	1	1	NGFR
Pubmed	9273702	Basal forebrain neuronal loss in mice lacking neurotrophin receptor p75.	Pubmed	0.0044	65.1	0.0114	0.116	1	1	NGFR
Pubmed	12100035	Fractalkine expression on human renal tubular epithelial cells: potential role in mononuclear cell adhesion.	GeneRIF	0.0044	65.1	0.0114	0.116	1	1	CX3CL1
Pubmed	11173530	Purification, molecular cloning, and immunohistochemical localization of dipeptidyl peptidase II from the rat kidney and its identity with quiescent cell proline dipeptidase.	Pubmed	0.0044	65.1	0.0114	0.116	1	1	DPP7
Pubmed	20473768	Expression of the GA733 gene family and its relationship to prognosis in pulmonary adenocarcinoma.	GeneRIF	0.0044	65.1	0.0114	0.116	1	1	TACSTD2
Pubmed	23399772	Thyroid hormone receptor alpha gene variants increase the risk of developing obesity and show gene-diet interactions.	Pubmed	0.0044	65.1	0.0114	0.116	1	1	THRA
Pubmed	22130737	Tissue transglutaminase: a new target to reverse cancer drug resistance.	GeneRIF	0.0044	65.1	0.0114	0.116	1	1	TGM2
Pubmed	19301169	Selection of CD271(+) cells and human AB serum allows a large expansion of mesenchymal stromal cells from human bone marrow.	GeneRIF	0.0044	65.1	0.0114	0.116	1	1	NGFR
Pubmed	22183397	Expression of the integrin coreceptor transglutaminase-2 in the RPE in vivo and in culture.	GeneRIF	0.0044	65.1	0.0114	0.116	1	1	TGM2
Pubmed	15481897	The beta-globin gene cluster haplotypes in sickle cell anemia patients from Northeast Brazil: a clinical and molecular view.	GeneRIF	0.0044	65.1	0.0114	0.116	1	1	HBB
Pubmed	19490267	Not all hERG pore domain mutations have a severe phenotype: G584S has an inactivation gating defect with mild phenotype compared to G572S, which has a dominant negative trafficking defect and a severe phenotype.	GeneRIF	0.0044	65.1	0.0114	0.116	1	1	KCNH2
Pubmed	18667508	Herpes simplex virus downregulates secretory leukocyte protease inhibitor: a novel immune evasion mechanism.	GeneRIF	0.0044	65.1	0.0114	0.116	1	1	SLPI
Pubmed	7579524	Growth factors in development: the role of TGF-beta related polypeptide signalling molecules in embryogenesis.	Pubmed	0.0044	65.1	0.0114	0.116	1	1	BMP4
Pubmed	12929572	Differential expression of tissue transglutaminase protein in mouse ovarian follicle.	GeneRIF	0.0044	65.1	0.0114	0.116	1	1	TGM2
Pubmed	24535794	Mutation in the HTRA1 gene in a patient with degenerated spine as a component of CARASIL syndrome.	Pubmed	0.0044	65.1	0.0114	0.116	1	1	HTRA1
Pubmed	9346885	Proinsulin targeting to the regulated pathway is not impaired in carboxypeptidase E-deficient Cpefat/Cpefat mice.	Pubmed	0.0044	65.1	0.0114	0.116	1	1	CPE
Pubmed	19819870	Functional roles of human kallikrein-related peptidases.	Pubmed	0.0044	65.1	0.0114	0.116	1	1	KLK15
Pubmed	21707759	Elevated MMP-7 levels in patients with systemic sclerosis: correlation with pulmonary involvement.	GeneRIF	0.0044	65.1	0.0114	0.116	1	1	MMP7
Pubmed	22251481	The endothelial protein C receptor (PROCR) Ser219Gly variant and risk of common thrombotic disorders: a HuGE review and meta-analysis of evidence from observational studies.	GeneRIF	0.0044	65.1	0.0114	0.116	1	1	PROCR
Pubmed	20813964	aB-crystallin is elevated in highly infiltrative apoptosis-resistant glioblastoma cells.	GeneRIF	0.0044	65.1	0.0114	0.116	1	1	CRYAA
Pubmed	3620699	An alpha-globin gene initiation codon mutation in a black family with HbH disease.	Pubmed	0.0044	65.1	0.0114	0.116	1	1	HBA2
Pubmed	15226407	Myopathy mutations in alpha-skeletal-muscle actin cause a range of molecular defects.	GeneRIF	0.0044	65.1	0.0114	0.116	1	1	ACTA1
Pubmed	24670995	AAV-mediated expression of human PRELP inhibits complement activation, choroidal neovascularization and deposition of membrane attack complex in mice.	Pubmed	0.0044	65.1	0.0114	0.116	1	1	PRELP
Pubmed	14514946	Analysis of DQB1 allele frequencies in pulmonary tuberculosis: preliminary report.	GeneRIF	0.0044	65.1	0.0114	0.116	1	1	HLA-DQB1
Pubmed	21672310	AKR1B10 is associated with smoking and smoking-related non-small-cell lung cancer.	GeneRIF	0.0044	65.1	0.0114	0.116	1	1	AKR1B10
Pubmed	12142119	Association between HERG K897T polymorphism and QT interval in middle-aged Finnish women.	GeneRIF	0.0044	65.1	0.0114	0.116	1	1	KCNH2
Pubmed	19181662	Differential Processing of {alpha}- and {beta}-Defensin Precursors by Matrix Metalloproteinase-7 (MMP-7).	GeneRIF	0.0044	65.1	0.0114	0.116	1	1	MMP7
Pubmed	23896945	Macrophage migration inhibitory factor receptor CD74 mediates alphavirus-induced arthritis and myositis in murine models of alphavirus infection.	Pubmed	0.0044	65.1	0.0114	0.116	1	1	CD74
Pubmed	6198240	Isolation of cDNA clones for human adenosine deaminase.	Pubmed	0.0044	65.1	0.0114	0.116	1	1	ADA
Pubmed	11309366	A novel mutation in the coding region of the prosaposin gene leads to a complete deficiency of prosaposin and saposins, and is associated with a complex sphingolipidosis dominated by lactosylceramide accumulation.	Pubmed	0.0044	65.1	0.0114	0.116	1	1	PSAP
Pubmed	15725655	Nobiletin, a citrus flavonoid, down-regulates matrix metalloproteinase-7 (matrilysin) expression in HT-29 human colorectal cancer cells.	GeneRIF	0.0044	65.1	0.0114	0.116	1	1	MMP7
Pubmed	19066892	The 4-bp deletion (-AAAC) in the 5' untranslated region of the beta-globin gene: a simple polymorphism?	GeneRIF	0.0044	65.1	0.0114	0.116	1	1	HBB
Pubmed	20004623	Takotsubo cardiomyopathy and congenital long QT syndrome in a patient with a novel duplication in the Per-Arnt-Sim (PAS) domain of hERG1.	GeneRIF	0.0044	65.1	0.0114	0.116	1	1	KCNH2
Pubmed	3086866	Sequence analysis and structure-function correlations of murine q, k, u, s, and f haplotype I-A beta cDNA clones.	Pubmed	0.0044	65.1	0.0114	0.116	1	1	HLA-DQB1

Category	ID	Name	Source	p-value	q-value Bonferroni	q-value FDR B&H	q-value FDR B&Y	Hit Count in Query List	Hit Count in Genome	Hit in Query List
Pubmed	18312620	Secretion of celiac disease autoantibodies after in vitro gliadin challenge is dependent on small-bowel mucosal transglutaminase 2-specific IgA deposits.	GeneRIF	0.0044	65.1	0.0114	0.116	1	1	TGM2
Pubmed	7835394	A partial cDNA sequence corrects the human alpha A-crystallin primary structure.	Pubmed	0.0044	65.1	0.0114	0.116	1	1	CRYAA
Pubmed	24312257	The Ca²¿-activated K¿ channel KCa3.1 as a potential new target for the prevention of allograft vasculopathy.	Pubmed	0.0044	65.1	0.0114	0.116	1	1	KCNN4
Pubmed	10886328	SEK1/MKK4, c-Jun and NFKappaB are differentially activated in forebrain neurons during postnatal development and injury in both control and p75NGFR-deficient mice.	Pubmed	0.0044	65.1	0.0114	0.116	1	1	NGFR
Pubmed	10928776	Epithelial barrier function and ultrastructure of gelatinous drop-like corneal dystrophy.	Pubmed	0.0044	65.1	0.0114	0.116	1	1	TACSTD2
Pubmed	2253219	Purification and characterization of extracellular matrix-degrading metalloproteinase, matrin (pump-1), secreted from human rectal carcinoma cell line.	Pubmed	0.0044	65.1	0.0114	0.116	1	1	MMP7
Pubmed	6324001	Nucleotide sequence of mutant I-A beta bm12 gene is evidence for genetic exchange between mouse immune response genes.	Pubmed	0.0044	65.1	0.0114	0.116	1	1	HLA-DQB1
Pubmed	19287092	Loss-of-function variants in endothelial lipase are a cause of elevated HDL cholesterol in humans.	GeneRIF	0.0044	65.1	0.0114	0.116	1	1	LIPG
Pubmed	15248222	Dichotomous effects of complete versus partial class II major histocompatibility complex deficiency on circulating autoantibody levels in autoimmune-prone mice.	Pubmed	0.0044	65.1	0.0114	0.116	1	1	HLA-DQB1
Pubmed	12355495	Mice lacking endogenous major histocompatibility complex class II develop arthritis resembling psoriatic arthritis at an advanced age.	Pubmed	0.0044	65.1	0.0114	0.116	1	1	HLA-DQB1
Pubmed	6254664	The nucleotide sequence of the human beta-globin gene.	Pubmed	0.0044	65.1	0.0114	0.116	1	1	HBB
Pubmed	24905200	An RNA editor, adenosine deaminase acting on double-stranded RNA (ADAR1).	Pubmed	0.0044	65.1	0.0114	0.116	1	1	ADA
Pubmed	18207215	Further mapping of 10q26 supports strong association of HTRA1 polymorphisms with age-related macular degeneration.	GeneRIF	0.0044	65.1	0.0114	0.116	1	1	HTRA1
Pubmed	15367892	Acute pancreatitis in the early stages of pregnancy associated with a PSTI gene mutation.	GeneRIF	0.0044	65.1	0.0114	0.116	1	1	SPINK1
Pubmed	21938679	Inhibin beta B: a useful tumor marker in uterine endometrioid adenocarcinomas?	GeneRIF	0.0044	65.1	0.0114	0.116	1	1	INHBB
Pubmed	11481435	Identification of peptides from brain and pituitary of Cpe(fat)/Cpe(fat) mice.	Pubmed	0.0044	65.1	0.0114	0.116	1	1	CPE
Pubmed	22187944	Potential role of soluble B7-H3 in liver immunopathogenesis during chronic HBV infection.	GeneRIF	0.0044	65.1	0.0114	0.116	1	1	CD276
Pubmed	21222374	Prevalence of hemoglobin alpha-chain gene deletion in neonates in North of Iran.	GeneRIF	0.0044	65.1	0.0114	0.116	1	1	HBA2
Pubmed	21798344	Vascular peroxidase-1 is rapidly secreted, circulates in plasma, and supports dityrosine cross-linking reactions.	GeneRIF	0.0044	65.1	0.0114	0.116	1	1	PXDN
Pubmed	19122164	Atrial natriuretic peptide increases inflammation, infarct size, and mortality after experimental coronary occlusion.	Pubmed	0.0044	65.1	0.0114	0.116	1	1	NPPA
Pubmed	15331434	Hyplip2, a new gene for combined hyperlipidemia and increased atherosclerosis.	Pubmed	0.0044	65.1	0.0114	0.116	1	1	FBXO32
Pubmed	8036737	Regulation of the human immunodeficiency virus type 1 long terminal repeat: interactions of thyroid hormone receptor with retinoid-X receptor, nuclear factor kappa B, Sp1, and Tat.	Pubmed	0.0044	65.1	0.0114	0.116	1	1	THRA
Pubmed	22127435	In vitro mitochondrial effects of PK 11195, a synthetic translocator protein 18 kDa (TSPO) ligand, in human osteoblast-like cells.	GeneRIF	0.0044	65.1	0.0114	0.116	1	1	TSPO
Pubmed	12913006	Regulated intramembrane proteolysis of the p75 neurotrophin receptor modulates its association with the TrkA receptor.	GeneRIF	0.0044	65.1	0.0114	0.116	1	1	NGFR
Pubmed	21044991	Use of a Mendelian randomization approach to assess the causal relation of gamma-Glutamyltransferase with blood pressure and serum insulin levels.	GeneRIF	0.0044	65.1	0.0114	0.116	1	1	GGT1
Pubmed	20056222	Evaluation of translocator protein quantification as a tool for characterising macrophage burden in human carotid atherosclerosis.	GeneRIF	0.0044	65.1	0.0114	0.116	1	1	TSPO
Pubmed	3155690	Ca2+ capacity and uptake rate in skinned fibers of myodystrophic muscle.	Pubmed	0.0044	65.1	0.0114	0.116	1	1	LARGE
Pubmed	24629698	Correlation of deregulated like-acetylglucosaminyl transferase and aberrant a-dystroglycan expression with human tongue cancer metastasis.	Pubmed	0.0044	65.1	0.0114	0.116	1	1	LARGE
Pubmed	7912692	The human tissue transglutaminase gene maps on chromosome 20q12 by in situ fluorescence hybridization.	Pubmed	0.0044	65.1	0.0114	0.116	1	1	TGM2
Pubmed	21465474	Dynamic redistribution of calcium sensitive potassium channels (hK(Ca)3.1) in migrating cells.	GeneRIF	0.0044	65.1	0.0114	0.116	1	1	KCNN4
Pubmed	16099913	Stable trimerization of recombinant rabies virus glycoprotein ectodomain is required for interaction with the p75NTR receptor.	GeneRIF	0.0044	65.1	0.0114	0.116	1	1	NGFR
Pubmed	21573751	Comparison of protein behavior between wild-type and G601S hERG in living cells by fluorescence correlation spectroscopy.	GeneRIF	0.0044	65.1	0.0114	0.116	1	1	KCNH2
Pubmed	2432402	Molecular cloning of the murine adenosine deaminase gene from a genetically enriched source: identification and characterization of the promoter region.	Pubmed	0.0044	65.1	0.0114	0.116	1	1	ADA
Pubmed	24350253	Association of genetic variants of BMP4 with type 2 diabetes mellitus and clinical traits in a Chinese Han population.	Pubmed	0.0044	65.1	0.0114	0.116	1	1	BMP4
Pubmed	20238298	Role of HTRA1, a serine protease, in the progression of articular cartilage degeneration.	GeneRIF	0.0044	65.1	0.0114	0.116	1	1	HTRA1
Pubmed	16336202	SLPI and elafin: one glove, many fingers.	Pubmed	0.0044	65.1	0.0114	0.116	1	1	SLPI
Pubmed	21075280	HbE/ß-thalassemia: basis of marked clinical diversity.	GeneRIF	0.0044	65.1	0.0114	0.116	1	1	HBB
Pubmed	6808517	Enkephalin convertase: purification and characterization of a specific enkephalin-synthesizing carboxypeptidase localized to adrenal chromaffin granules.	Pubmed	0.0044	65.1	0.0114	0.116	1	1	CPE

Category	ID	Name	Source	p-value	q-value Bonferroni	q-value FDR B&H	q-value FDR B&Y	Hit Count in Query List	Hit Count in Genome	Hit in Query List
Pubmed	11575428	Cellular distribution of napsin (kidney-derived aspartic protease-like protein, KAP) mRNA in the kidney, lung and lymphatic organs of adult and developing mice.	Pubmed	0.0044	65.1	0.0114	0.116	1	1	NAPSA
Pubmed	23673478	The effects of endothelial lipase gene (LIPG) variants on inflammation marker levels and atherosclerosis development.	Pubmed	0.0044	65.1	0.0114	0.116	1	1	LIPG
Pubmed	16204895	High-resolution neutron protein crystallography with radically small crystal volumes: application of perdeuteration to human aldose reductase.	GeneRIF	0.0044	65.1	0.0114	0.116	1	1	AKR1B10
Pubmed	12090587	Association between the HLA DQB1*0301 gene and human papillomavirus infection in high-grade cervical intraepithelial neoplasia.	GeneRIF	0.0044	65.1	0.0114	0.116	1	1	HLA-DQB1
Pubmed	10398311	Hb Iraq-Halabja beta10 (A7) Ala-->Val (GCC-->GTC): a new beta-chain silent variant in a family with multiple Hb disorders.	Pubmed	0.0044	65.1	0.0114	0.116	1	1	HBB
Pubmed	21077767	Molecular screening of the Hbs Constant Spring (codon 142, TAA>CAA, a2) and Paksé (codon 142, TAA>TAT, a2) mutations in Thailand.	GeneRIF	0.0044	65.1	0.0114	0.116	1	1	HBA2
Pubmed	23065995	Diabetic ketoacidosis at diagnosis: role of family history and class II HLA genotypes.	GeneRIF	0.0044	65.1	0.0114	0.116	1	1	HLA-DQB1
Pubmed	21817099	Cathepsin K deficiency reduces elastase perfusion-induced abdominal aortic aneurysms in mice.	GeneRIF	0.0044	65.1	0.0114	0.116	1	1	CTSK
Pubmed	17371258	The role of secretory leucoprotease inhibitor in the resolution of inflammatory responses.	GeneRIF	0.0044	65.1	0.0114	0.116	1	1	SLPI
Pubmed	11480785	Genetic heterogeneity of beta-thalassemia at Cukurova in southern Turkey.	GeneRIF	0.0044	65.1	0.0114	0.116	1	1	HBB
Pubmed	24563071	Angiopoietin-like protein 2 regulates endothelial colony forming cell vasculogenesis.	Pubmed	0.0044	65.1	0.0114	0.116	1	1	ANGPTL2
Pubmed	8921412	Exon structure of the gene encoding the human mannose-binding protein-associated serine protease light chain: comparison with complement C1r and C1s genes.	Pubmed	0.0044	65.1	0.0114	0.116	1	1	MASP1
Pubmed	25444857	Steady-state substrate specificity and O2-coupling efficiency of mouse cysteine dioxygenase.	Pubmed	0.0044	65.1	0.0114	0.116	1	1	CDO1
Pubmed	23527199	Global gene expression profiling reveals SPINK1 as a potential hepatocellular carcinoma marker.	GeneRIF	0.0044	65.1	0.0114	0.116	1	1	SPINK1
Pubmed	1716694	Characterization of a novel human papillomavirus DNA in the cervical carcinoma cell line ME180.	Pubmed	0.0044	65.1	0.0114	0.116	1	1	ZBTB7C
Pubmed	21195129	Regulation of steroid production: analysis of Cyp11a1 promoter.	GeneRIF	0.0044	65.1	0.0114	0.116	1	1	CYP11A1
Pubmed	16826431	Tissue array-based expression of transglutaminase-2 in human breast and ovarian cancer.	GeneRIF	0.0044	65.1	0.0114	0.116	1	1	TGM2
Pubmed	24154981	Screening for cardiac HERG potassium channel interacting proteins using the yeast two-hybrid technique.	Pubmed	0.0044	65.1	0.0114	0.116	1	1	KCNH2
Pubmed	21844367	Increased expression of multifunctional serine protease, HTRA1, in retinal pigment epithelium induces polypoidal choroidal vasculopathy in mice.	GeneRIF	0.0044	65.1	0.0114	0.116	1	1	HTRA1
Pubmed	19290524	A novel mutation of -50 (G-->A) in the direct repeat element of the beta-globin gene identified in a patient with severe beta-thalassemia.	GeneRIF	0.0044	65.1	0.0114	0.116	1	1	HBB
Pubmed	24075519	Cyst fluid SPINK1 may help to differentiate benign and potentially malignant cystic pancreatic lesions.	Pubmed	0.0044	65.1	0.0114	0.116	1	1	SPINK1
Pubmed	17056066	Cooperativeness of the higher chromatin structure of the beta-globin locus revealed by the deletion mutations of DNase I hypersensitive site 3 of the LCR.	GeneRIF	0.0044	65.1	0.0114	0.116	1	1	HBB
Pubmed	11867617	Analysis of tissue transglutaminase function in the migration of Swiss 3T3 fibroblasts: the active-state conformation of the enzyme does not affect cell motility but is important for its secretion.	GeneRIF	0.0044	65.1	0.0114	0.116	1	1	TGM2
Pubmed	15355514	Rab coupling protein associates with phagosomes and regulates recycling from the phagosomal compartment.	GeneRIF	0.0044	65.1	0.0114	0.116	1	1	RAB11FIP1
Pubmed	19888199	SPINK1 N34S is strongly associated with recurrent acute pancreatitis but is not a risk factor for the first or sentinel acute pancreatitis event.	GeneRIF	0.0044	65.1	0.0114	0.116	1	1	SPINK1
Pubmed	15102692	Comprehensive analysis of matrix metalloproteinase and tissue inhibitor expression in pancreatic cancer: increased expression of matrix metalloproteinase-7 predicts poor survival.	GeneRIF	0.0044	65.1	0.0114	0.116	1	1	MMP7
Pubmed	24685690	Lipid accumulation in dysferlin-deficient muscles.	Pubmed	0.0044	65.1	0.0114	0.116	1	1	DYSF
Pubmed	19034330	Gammaglutamyltransferase in ESRD as a predictor of all-cause and cardiovascular mortality: another facet of oxidative stress burden.	GeneRIF	0.0044	65.1	0.0114	0.116	1	1	GGT1
Pubmed	20228126	Silencing of the microtubule-associated proteins doublecortin-like and doublecortin-like kinase-long induces apoptosis in neuroblastoma cells.	GeneRIF	0.0044	65.1	0.0114	0.116	1	1	DCLK1
Pubmed	22498646	Reduction of cytotoxic p-quinone metabolites of tert-butylhydroquinone by human aldo-keto reductase (AKR) 1B10.	GeneRIF	0.0044	65.1	0.0114	0.116	1	1	AKR1B10
Pubmed	11060308	Glutathiolation of proteins by glutathione disulfide S-oxide derived from S-nitrosoglutathione. Modifications of rat brain neurogranin/RC3 and neuromodulin/GAP-43.	Pubmed	0.0044	65.1	0.0114	0.116	1	1	NRGN
Pubmed	12638941	Glutathione synthetase deficiency: is gamma-glutamylcysteine accumulation a way to cope with oxidative stress in cells with insufficient levels of glutathione?	GeneRIF	0.0044	65.1	0.0114	0.116	1	1	GSS
Pubmed	11939510	The beta+-IVS-I-6 (T-->C) mutation accounts for half of the thalassemia chromosomes in the Palestinian populations of the mountain regions.	GeneRIF	0.0044	65.1	0.0114	0.116	1	1	HBB
Pubmed	23235792	[Beta globin haplotypes in hemoglobin S carriers in Colombia].	GeneRIF	0.0044	65.1	0.0114	0.116	1	1	HBB

Category	ID	Name	Source	p-value	q-value Bonferroni	q-value FDR B&H	q-value FDR B&Y	Hit Count in Query List	Hit Count in Genome	Hit in Query List
Pubmed	15713129	High frequency of partial DIIIa and DAR alleles found in sickle cell disease patients suggests increased risk of alloimmunization to RhD.	GeneRIF	0.0044	65.1	0.0114	0.116	1	1	HBB
Pubmed	12556916	HLA and genetic susceptibility to sleepwalking.	GeneRIF	0.0044	65.1	0.0114	0.116	1	1	HLA-DQB1
Pubmed	16011830	The N588K-HERG K+ channel mutation in the 'short QT syndrome': mechanism of gain-in-function determined at 37 degrees C.	GeneRIF	0.0044	65.1	0.0114	0.116	1	1	KCNH2
Pubmed	8745430	HB Hinwil or beta 38(C4)Thr-->Asn: a new beta chain variant detected in a Swiss family.	Pubmed	0.0044	65.1	0.0114	0.116	1	1	HBB
Pubmed	16831915	The regulation of cathepsin K gene expression.	GeneRIF	0.0044	65.1	0.0114	0.116	1	1	CTSK
Pubmed	22424154	Are hepatic steatosis and carotid intima media thickness associated in obese patients with normal or slightly elevated gamma-glutamyl-transferase?	GeneRIF	0.0044	65.1	0.0114	0.116	1	1	GGT1
Pubmed	7684189	Human smooth muscle myosin heavy chain gene mapped to chromosomal region 16q12.	Pubmed	0.0044	65.1	0.0114	0.116	1	1	MYH11
Pubmed	3158606	Molecular studies of the atrial natriuretic factor gene.	Pubmed	0.0044	65.1	0.0114	0.116	1	1	NPPA
Pubmed	12036680	Two brothers with gelatinous drop-like dystrophy at different stages of the disease: role of mutational analysis.	GeneRIF	0.0044	65.1	0.0114	0.116	1	1	TACSTD2
Pubmed	10548568	Enhanced secretory leukocyte protease inhibitor in human immunodeficiency virus type 1-infected patients.	Pubmed	0.0044	65.1	0.0114	0.116	1	1	SLPI
Pubmed	19668260	Adenosine deaminase enhances T-cell response elicited by dendritic cells loaded with inactivated HIV.	GeneRIF	0.0044	65.1	0.0114	0.116	1	1	ADA
Pubmed	2109324	RTA, a candidate G protein-coupled receptor: cloning, sequencing, and tissue distribution.	Pubmed	0.0044	65.1	0.0114	0.116	1	1	MRGPRF
Pubmed	24976126	p75NTR is highly expressed in vestibular schwannomas and promotes cell survival by activating nuclear transcription factor ¿B.	Pubmed	0.0044	65.1	0.0114	0.116	1	1	NGFR
Pubmed	22281496	The role of angiopoietin-like protein 2 in pathogenesis of dermatomyositis.	GeneRIF	0.0044	65.1	0.0114	0.116	1	1	ANGPTL2
Pubmed	23702783	Angiopoietin-like protein 2 is a potent hemangiogenic and lymphangiogenic factor in corneal inflammation.	GeneRIF	0.0044	65.1	0.0114	0.116	1	1	ANGPTL2
Pubmed	7759315	Surface adenosine deaminase. A novel B-cell marker in chronic lymphocytic leukemia.	Pubmed	0.0044	65.1	0.0114	0.116	1	1	ADA
Pubmed	21521327	Serum gamma-glutamyltransferase is associated with arterial stiffness in healthy individuals.	GeneRIF	0.0044	65.1	0.0114	0.116	1	1	GGT1
Pubmed	10839371	Modification of glial-neuronal cell interactions prevents photoreceptor apoptosis during light-induced retinal degeneration.	Pubmed	0.0044	65.1	0.0114	0.116	1	1	NGFR
Pubmed	23136988	Association of soluble endothelial protein C receptor plasma levels and PROCR rs867186 with cardiovascular risk factors and cardiovascular events in coronary artery disease patients: the Athero Gene study.	GeneRIF	0.0044	65.1	0.0114	0.116	1	1	PROCR
Pubmed	11703343	Characterization of protein C receptor expression in monocytes.	Pubmed	0.0044	65.1	0.0114	0.116	1	1	PROCR
Pubmed	19305425	Upregulation of PIP3-dependent Rac exchanger 1 (P-Rex1) promotes prostate cancer metastasis.	GeneRIF	0.0044	65.1	0.0114	0.116	1	1	PREX1
Pubmed	25295538	PCP4 regulates Purkinje cell excitability and cardiac rhythmicity.	Pubmed	0.0044	65.1	0.0114	0.116	1	1	PCP4
Pubmed	21798427	A genetic variant of the atrial natriuretic peptide gene is associated with cardiometabolic protection in the general community.	GeneRIF	0.0044	65.1	0.0114	0.116	1	1	NPPA
Pubmed	11726558	Dramatic phenotypic improvement during pregnancy in a genetic leukodystrophy: estrogen appears to be a critical factor.	Pubmed	0.0044	65.1	0.0114	0.116	1	1	PSAP
Pubmed	23718075	[TSPO ligand PK11195 and MAPK inhibitor UO126 modulate tspo expression level].	GeneRIF	0.0044	65.1	0.0114	0.116	1	1	TSPO
Pubmed	24654911	Correlations of ANP genetic polymorphisms and serum levels with ischemic stroke risk: a meta-analysis.	Pubmed	0.0044	65.1	0.0114	0.116	1	1	NPPA
Pubmed	23392713	Inhibitory effects of blockage of intermediate conductance Ca(2+)-activated K (+) channels on proliferation of hepatocellular carcinoma cells.	Pubmed	0.0044	65.1	0.0114	0.116	1	1	KCNN4
Pubmed	22848423	A molecular switch driving inactivation in the cardiac K+ channel HERG.	GeneRIF	0.0044	65.1	0.0114	0.116	1	1	KCNH2
Pubmed	16835374	Ex vivo gene therapy with lentiviral vectors rescues adenosine deaminase (ADA)-deficient mice and corrects their immune and metabolic defects.	Pubmed	0.0044	65.1	0.0114	0.116	1	1	ADA
Pubmed	15365620	The role of the p75 neurotrophin receptor in the morphology of dorsal root ganglion cells in streptozotocin diabetic mice: effects of sciatic nerve crush.	GeneRIF	0.0044	65.1	0.0114	0.116	1	1	NGFR
Pubmed	21029690	[Association between HPV infection and HLA-DQB1 alleles polymorphism in the cervical carcinogenesis in Uyghur women in southern Xinjiang].	GeneRIF	0.0044	65.1	0.0114	0.116	1	1	HLA-DQB1
Pubmed	15013397	Tertiary structure of human alpha1-acid glycoprotein (orosomucoid). Straightforward fluorescence experiments revealing the presence of a binding pocket.	GeneRIF	0.0044	65.1	0.0114	0.116	1	1	ORM1
Pubmed	21034280	Rapid identification of common ß-thalassemia mutations in the Chinese population using duplex or triplex amplicon genotyping by high-resolution melting analysis.	GeneRIF	0.0044	65.1	0.0114	0.116	1	1	HBB
Pubmed	16023652	Association of endothelial lipase gene (LIPG) haplotypes with high-density lipoprotein cholesterol subfractions and apolipoprotein AI plasma levels in Japanese Americans.	GeneRIF	0.0044	65.1	0.0114	0.116	1	1	LIPG
Pubmed	24751878	Anti-inflammatory activity of bone morphogenetic protein signaling pathways in stomachs of mice.	Pubmed	0.0044	65.1	0.0114	0.116	1	1	BMP4
Pubmed	16259007	The multigenic structure of the MHC locus contributes to positive selection efficiency: a role for MHC class II gene-specific restriction.	Pubmed	0.0044	65.1	0.0114	0.116	1	1	HLA-DQB1
Pubmed	24397957	Translocator protein (18 kDa) (TSPO) is expressed in reactive retinal microglia and modulates microglial inflammation and phagocytosis.	Pubmed	0.0044	65.1	0.0114	0.116	1	1	TSPO

Category	ID	Name	Source	p-value	q-value Bonferroni	q-value FDR B&H	q-value FDR B&Y	Hit Count in Query List	Hit Count in Genome	Hit in Query List
Pubmed	15763343	Expression of osteoactivin in rat and human liver and isolated rat liver cells.	GeneRIF	0.0044	65.1	0.0114	0.116	1	1	GPNMB
Pubmed	23588617	Delayed fracture healing in tetranectin-deficient mice.	Pubmed	0.0044	65.1	0.0114	0.116	1	1	CLEC3B
Pubmed	19360007	Subcutaneous fat shows higher thyroid hormone receptor-alpha1 gene expression than omental fat.	GeneRIF	0.0044	65.1	0.0114	0.116	1	1	THRA
Pubmed	7237554	The adult beta-globin genes of the "single" type mouse C57BL.	Pubmed	0.0044	65.1	0.0114	0.116	1	1	HBB
Pubmed	18422740	Plasma matrix metalloproteinase-7 as a metastatic marker and survival predictor in patients with renal cell carcinomas.	GeneRIF	0.0044	65.1	0.0114	0.116	1	1	MMP7
Pubmed	17492429	Levels of three distinct p75 neurotrophin receptor forms found in human plasma are altered in type 2 diabetic patients.	GeneRIF	0.0044	65.1	0.0114	0.116	1	1	NGFR
Pubmed	8622652	Regulation of AP-1 and NFAT transcription factors during thymic selection of T cells.	Pubmed	0.0044	65.1	0.0114	0.116	1	1	HLA-DQB1
Pubmed	19136169	Novel mutation (H402R) in the S1 domain of KCNH2-encoded gene associated with long QT syndrome in a Spanish family.	GeneRIF	0.0044	65.1	0.0114	0.116	1	1	KCNH2
Pubmed	8334382	Murine prosaposin: expression in the reproductive system of a gene implicated in human genetic diseases.	Pubmed	0.0044	65.1	0.0114	0.116	1	1	PSAP
Pubmed	11724775	Modulation of mouse Paneth cell alpha-defensin secretion by mIKCa1, a Ca2+-activated, intermediate conductance potassium channel.	Pubmed	0.0044	65.1	0.0114	0.116	1	1	KCNN4
Pubmed	17327371	Overexpression of cysteine dioxygenase reduces intracellular cysteine and glutathione pools in HepG2/C3A cells.	GeneRIF	0.0044	65.1	0.0114	0.116	1	1	CDO1
Pubmed	21142793	CD271/p75(NTR) inhibits the differentiation of mesenchymal stem cells into osteogenic, adipogenic, chondrogenic, and myogenic lineages.	GeneRIF	0.0044	65.1	0.0114	0.116	1	1	NGFR
Pubmed	17878632	Detection of mRNAs of GA733 genes by RT-PCR in exfoliated cells of pleural and peritoneal effusions and its clinical values.	GeneRIF	0.0044	65.1	0.0114	0.116	1	1	TACSTD2
Pubmed	10607586	The metalloproteinase matrilysin proteolytically generates active soluble Fas ligand and potentiates epithelial cell apoptosis.	Pubmed	0.0044	65.1	0.0114	0.116	1	1	MMP7
Pubmed	21981819	Antisense oligonucleotide targeting matrix metalloproteinase-7 (MMP-7) changes the ultrastructure of human A549 lung adenocarcinoma cells.	GeneRIF	0.0044	65.1	0.0114	0.116	1	1	MMP7
Pubmed	12619129	Bone morphogenetic protein 4 (BMP4): a regulator of capsule chondrogenesis in the developing mouse inner ear.	GeneRIF	0.0044	65.1	0.0114	0.116	1	1	BMP4
Pubmed	11186269	Second report of Hb Toulon [alpha77(EF6)Pro-->His] in a Canadian family of Italian descent.	Pubmed	0.0044	65.1	0.0114	0.116	1	1	HBA2
Pubmed	16444274	Mutations in myosin heavy chain 11 cause a syndrome associating thoracic aortic aneurysm/aortic dissection and patent ductus arteriosus.	GeneRIF	0.0044	65.1	0.0114	0.116	1	1	MYH11
Pubmed	1378736	Gamma-glutamyltransferase: nucleotide sequence of the human pancreatic cDNA. Evidence for a ubiquitous gamma-glutamyltransferase polypeptide in human tissues.	Pubmed	0.0044	65.1	0.0114	0.116	1	1	GGT1
Pubmed	22198009	A word of caution regarding napsin A expression in squamous cell carcinomas of the lung.	GeneRIF	0.0044	65.1	0.0114	0.116	1	1	NAPSA
Pubmed	11296153	Association between HLA-DQB1 alleles and type 1 diabetes in a case-parents study conducted in Santiago, Chile.	GeneRIF	0.0044	65.1	0.0114	0.116	1	1	HLA-DQB1
Pubmed	15611141	Kallikrein/kinin protects against myocardial apoptosis after ischemia/reperfusion via Akt-glycogen synthase kinase-3 and Akt-Bad.14-3-3 signaling pathways.	GeneRIF	0.0044	65.1	0.0114	0.116	1	1	KLK1
Pubmed	12014716	Mutations of the serine protease inhibitor, Kazal type 1 gene, in patients with idiopathic chronic pancreatitis.	GeneRIF	0.0044	65.1	0.0114	0.116	1	1	SPINK1
Pubmed	992050	Haemoglobin caribbean beta91 (F7) Leu replaced by Arg: a mildly haemoglobin with a low oxygen affinity.	Pubmed	0.0044	65.1	0.0114	0.116	1	1	HBB
Pubmed	18381568	Endometrial inhibin/activin beta-B subunit expression is related to decidualization and is reduced in tubal ectopic pregnancy.	GeneRIF	0.0044	65.1	0.0114	0.116	1	1	INHBB
Pubmed	9583739	T cell responses in EAMG-susceptible and non-susceptible mouse strains after immunization with overlapping peptides encompassing the extracellular part of Torpedo californica acetylcholine receptor alpha chain. Implication to role in myasthenia gravis of autoimmune T-cell responses against receptor degradation products.	Pubmed	0.0044	65.1	0.0114	0.116	1	1	HLA-DQB1
Pubmed	24748164	¿-Glutamyltranspeptidase is a prognostic marker of survival and recurrence in radiofrequency-ablation treatment of hepatocellular carcinoma.	Pubmed	0.0044	65.1	0.0114	0.116	1	1	GGT1
Pubmed	21304968	Cytosolic guanine nucledotide binding deficient form of transglutaminase 2 (R580a) potentiates cell death in oxygen glucose deprivation.	GeneRIF	0.0044	65.1	0.0114	0.116	1	1	TGM2
Pubmed	22238156	Increased in vivo expression of an inflammatory marker in temporal lobe epilepsy.	GeneRIF	0.0044	65.1	0.0114	0.116	1	1	TSPO
Pubmed	17575134	Haplotype analysis of CYP11A1 identifies promoter variants associated with breast cancer risk.	GeneRIF	0.0044	65.1	0.0114	0.116	1	1	CYP11A1
Pubmed	10364477	The endothelial cell protein C receptor (EPCR) functions as a primary receptor for protein C activation on endothelial cells in arteries, veins, and capillaries.	Pubmed	0.0044	65.1	0.0114	0.116	1	1	PROCR
Pubmed	12933806	Novel membrane protein containing glycerophosphodiester phosphodiesterase motif is transiently expressed during osteoblast differentiation.	GeneRIF	0.0044	65.1	0.0114	0.116	1	1	GDPD2
Pubmed	8678932	Overexpression of an osteogenic morphogen in fibrodysplasia ossificans progressiva.	Pubmed	0.0044	65.1	0.0114	0.116	1	1	BMP4
Pubmed	10537232	Effect of saline infusion on kidney and collecting duct function in atrial natriuretic peptide (ANP) gene "knockout" mice.	Pubmed	0.0044	65.1	0.0114	0.116	1	1	NPPA

Category	ID	Name	Source	p-value	q-value Bonferroni	q-value FDR B&H	q-value FDR B&Y	Hit Count in Query List	Hit Count in Genome	Hit in Query List
Pubmed	20067463	Expression analysis and study of the KLK15 mRNA splice variants in prostate cancer and benign prostatic hyperplasia.	GeneRIF	0.0044	65.1	0.0114	0.116	1	1	KLK15
Pubmed	1487423	A Canadian family with Hb Wayne; characterization by HPLC and DNA sequencing.	Pubmed	0.0044	65.1	0.0114	0.116	1	1	HBA2
Pubmed	24690996	BMP4 enhances foam cell formation by BMPR-2/Smad1/5/8 signaling.	Pubmed	0.0044	65.1	0.0114	0.116	1	1	BMP4
Pubmed	15812165	Down-regulation of secretory leukocyte protease inhibitor expression in gastric mucosa is a general phenomenon in Helicobacter pylori-related gastroduodenal diseases.	GeneRIF	0.0044	65.1	0.0114	0.116	1	1	SLPI
Pubmed	18562134	ADA genetic polymorphism and the effect of smoking on neonatal bilirubinemia and developmental parameters.	GeneRIF	0.0044	65.1	0.0114	0.116	1	1	ADA
Pubmed	18098313	Therapeutic RNA silencing of Cys-X3-Cys chemokine ligand 1 gene prevents mice from adenovirus vector-induced acute liver injury.	GeneRIF	0.0044	65.1	0.0114	0.116	1	1	CX3CL1
Pubmed	17408647	Transglutaminase 2 regulates mallory body inclusion formation and injury-associated liver enlargement.	Pubmed	0.0044	65.1	0.0114	0.116	1	1	TGM2
Pubmed	23915319	The spectrum of ß-thalassemia mutations in Kermanshah Province in West Iran and its association with hematological parameters.	Pubmed	0.0044	65.1	0.0114	0.116	1	1	HBB
Pubmed	11782463	Sickle hemoglobin polymer stability probed by triple and quadruple mutant hybrids.	GeneRIF	0.0044	65.1	0.0114	0.116	1	1	HBB
Pubmed	21881051	Site-specific gene correction of a point mutation in human iPS cells derived from an adult patient with sickle cell disease.	GeneRIF	0.0044	65.1	0.0114	0.116	1	1	HBB
Pubmed	11857746	Reliability of DHPLC in mutational screening of beta-globin (HBB) alleles.	GeneRIF	0.0044	65.1	0.0114	0.116	1	1	HBB
Pubmed	19205978	Homozygosity for a rare beta 0-thalassemia mutation [frameshift codons 25/26 (+T)] causes beta-thalassemia intermedia in an Iranian family.	GeneRIF	0.0044	65.1	0.0114	0.116	1	1	HBB
Pubmed	15572367	Peptidomics of Cpe fat/fat mouse hypothalamus: effect of food deprivation and exercise on peptide levels.	GeneRIF	0.0044	65.1	0.0114	0.116	1	1	CPE
Pubmed	24744376	Crystal structure of a claudin provides insight into the architecture of tight junctions.	Pubmed	0.0044	65.1	0.0114	0.116	1	1	CLDN15
Pubmed	10536674	Chronic regulation of arterial blood pressure in ANP transgenic and knockout mice: role of cardiovascular sympathetic tone.	Pubmed	0.0044	65.1	0.0114	0.116	1	1	NPPA
Pubmed	22146835	Inflammatory peeling skin syndrome caused a novel mutation in CDSN.	Pubmed	0.0044	65.1	0.0114	0.116	1	1	CDSN
Pubmed	19317743	A real-time polymerase chain reaction assay for the rapid identification of the autoimmune disease-associated allele HLA-DQB1*0602.	GeneRIF	0.0044	65.1	0.0114	0.116	1	1	HLA-DQB1
Pubmed	17118062	Ureteric branching morphogenesis in BMP4 heterozygous mutant mice.	Pubmed	0.0044	65.1	0.0114	0.116	1	1	BMP4
Pubmed	11030761	New mutations in MID1 provide support for loss of function as the cause of X-linked Opitz syndrome.	Pubmed	0.0044	65.1	0.0114	0.116	1	1	MID1
Pubmed	8423399	Human trichohyalin gene is clustered with the genes for other epidermal structural proteins and calcium-binding proteins at chromosomal locus 1q21.	Pubmed	0.0044	65.1	0.0114	0.116	1	1	TCHH
Pubmed	7578036	1.7 A structure of FR-1, a fibroblast growth factor-induced member of the aldo-keto reductase family, complexed with coenzyme and inhibitor.	Pubmed	0.0044	65.1	0.0114	0.116	1	1	AKR1B10
Pubmed	8940036	Carboxypeptidase E activity is deficient in mice with the fat mutation. Effect on peptide processing	Pubmed	0.0044	65.1	0.0114	0.116	1	1	CPE
Pubmed	20691404	Loss of corneodesmosin leads to severe skin barrier defect, pruritus, and atopy: unraveling the peeling skin disease.	GeneRIF	0.0044	65.1	0.0114	0.116	1	1	CDSN
Pubmed	22193359	Crystal structure and characterization of coiled-coi domain of the transient receptor potential channel PKD2L1.	GeneRIF	0.0044	65.1	0.0114	0.116	1	1	PKD2L1
Pubmed	2869484	Molecular cloning and nucleotide sequence of rat kidney gamma-glutamyl transpeptidase cDNA.	Pubmed	0.0044	65.1	0.0114	0.116	1	1	GGT1
Pubmed	20299617	Onset of obesity in carboxypeptidase E-deficient mice and effect on airway responsiveness and pulmonary responses to ozone.	GeneRIF	0.0044	65.1	0.0114	0.116	1	1	CPE
Pubmed	2019586	Sulfatide activator protein. Alternative splicing that generates three mRNAs and a newly found mutation responsible for a clinical disease.	Pubmed	0.0044	65.1	0.0114	0.116	1	1	PSAP
Pubmed	15658192	A new highly unstable alpha chain variant causing alpha(+)-thalassemia: Hb Zurich Albisrieden [alpha59(E8)Gly-->Arg (alpha2)].	Pubmed	0.0044	65.1	0.0114	0.116	1	1	HBA2
Pubmed	20300660	Cystic fibrosis is a risk factor for celiac disease.	GeneRIF	0.0044	65.1	0.0114	0.116	1	1	HLA-DQB1
Pubmed	24311462	Haploinsufficiency of BMP4 gene may be the underlying cause of Frías syndrome.	Pubmed	0.0044	65.1	0.0114	0.116	1	1	BMP4
Pubmed	19915493	Screening detects a high proportion of celiac disease in young HLA-genotyped children.	GeneRIF	0.0044	65.1	0.0114	0.116	1	1	HLA-DQB1
Pubmed	22036807	Endothelial protein C receptor 1651C/G polymorphism and soluble endothelial protein C receptor levels in women with idiopathic recurrent miscarriage.	GeneRIF	0.0044	65.1	0.0114	0.116	1	1	PROCR
Pubmed	9342213	Characterisation of the promoter which regulates expression of a phosphoglucomutase-related protein, a component of the dystrophin/utrophin cytoskeleton predominantly expressed in smooth muscle.	Pubmed	0.0044	65.1	0.0114	0.116	1	1	PGM5
Pubmed	10627276	Apparent up-regulation of stimulatory G-protein alpha subunits in the pregnant human myometrium is mimicked by elevated smoothelin expression.	Pubmed	0.0044	65.1	0.0114	0.116	1	1	SMTN
Pubmed	6954511	Complete amino acid sequence of an HLA-DR antigen-like beta chain as predicted from the nucleotide sequence: similarities with immunoglobulins and HLA-A, -B, and -C antigens.	Pubmed	0.0044	65.1	0.0114	0.116	1	1	HLA-DQB1
Pubmed	2265255	Molecular basis for alpha-thalassemia associated with the structural mutant hemoglobin Suan-Dok (alpha 2 109leu----arg)	Pubmed	0.0044	65.1	0.0114	0.116	1	1	HBA2

Category	ID	Name	Source	p-value	q-value Bonferroni	q-value FDR B&H	q-value FDR B&Y	Hit Count in Query List	Hit Count in Genome	Hit in Query List
Pubmed	1138922	Haemoglobin Camperdown beta104(G6) arginine leads to serine.	Pubmed	0.0044	65.1	0.0114	0.116	1	1	HBB
Pubmed	19424205	Breaking the gene barrier in schizophrenia.	GeneRIF	0.0044	65.1	0.0114	0.116	1	1	KCNH2
Pubmed	21468611	Molecular and clinical characteristics of hemoglobin Ottawa detected in a Chinese population.	GeneRIF	0.0044	65.1	0.0114	0.116	1	1	HBA2
Pubmed	12445813	Phosphate depletion enhances bone morphogenetic protein-4 gene expression in a cultured mouse marrow stromal cell line ST2.	GeneRIF	0.0044	65.1	0.0114	0.116	1	1	BMP4
Pubmed	8825653	The gene encoding human glutathione synthetase (GSS) maps to the long arm of chromosome 20 at band 11.2.	Pubmed	0.0044	65.1	0.0114	0.116	1	1	GSS
Pubmed	11703436	Analysis of the association between diabetic nephropathy and polymorphisms in the aldose reductase gene in Type 1 and Type 2 diabetes mellitus.	GeneRIF	0.0044	65.1	0.0114	0.116	1	1	AKR1B10
Pubmed	23868985	Establishment of a human corneal epithelial cell line lacking the functional TACSTD2 gene as an in vitro model for gelatinous drop-like dystrophy.	Pubmed	0.0044	65.1	0.0114	0.116	1	1	TACSTD2
Pubmed	25170120	Induction of fetal hemoglobin through enhanced translation efficiency of ¿-globin mRNA.	Pubmed	0.0044	65.1	0.0114	0.116	1	1	HBB
Pubmed	12824004	Fractalkine/CX3CL1 production by human aortic smooth muscle cells impairs monocyte procoagulant and inflammatory responses.	GeneRIF	0.0044	65.1	0.0114	0.116	1	1	CX3CL1
Pubmed	18192845	Altered regulation of renal interstitial hydrostatic pressure and the renal renin-angiotensin system in the absence of atrial natriuretic peptide.	GeneRIF	0.0044	65.1	0.0114	0.116	1	1	NPPA
Pubmed	25753202	The role of microRNA-1274a in the tumorigenesis of gastric cancer: accelerating cancer cell proliferation and migration via directly targeting FOXO4.	Pubmed	0.0044	65.1	0.0114	0.116	1	1	FOXO4
Pubmed	19120309	Carboxypeptidase-H autoantibodies differentiate a more latent subset of autoimmune diabetes from phenotypic type 2 diabetes among Chinese adults.	GeneRIF	0.0044	65.1	0.0114	0.116	1	1	CPE
Pubmed	24632971	Circulating human fractalkine is decreased post-operatively after orthopedic and coronary bypass surgery.	Pubmed	0.0044	65.1	0.0114	0.116	1	1	CX3CL1
Pubmed	17634244	The ORF49 protein of murine gammaherpesvirus 68 cooperates with RTA in regulating virus replication.	GeneRIF	0.0044	65.1	0.0114	0.116	1	1	MRGPRF
Pubmed	22710722	Adhesion-mediated cytoskeletal remodeling is controlled by the direct scaffolding of Src from FAK complexes to lipid rafts by SSeCKS/AKAP12.	GeneRIF	0.0044	65.1	0.0114	0.116	1	1	AKAP12
Pubmed	17706365	The p75 neurotrophin receptor is essential for neuronal cell survival and improvement of functional recovery after spinal cord injury.	GeneRIF	0.0044	65.1	0.0114	0.116	1	1	NGFR
Pubmed	9177350	Neurotactin, a membrane-anchored chemokine upregulated in brain inflammation.	Pubmed	0.0044	65.1	0.0114	0.116	1	1	CX3CL1
Pubmed	15469878	Conservation of expression and alternative splicing in the prosaposin gene.	GeneRIF	0.0044	65.1	0.0114	0.116	1	1	PSAP
Pubmed	18461503	Nemaline myopathy with exclusively intranuclear rods and a novel mutation in ACTA1 (Q139H).	GeneRIF	0.0044	65.1	0.0114	0.116	1	1	ACTA1
Pubmed	8330972	Hb Karlskoga or alpha 2 beta (2)21(B3) Asp-->His: a new slow-moving variant found in Sweden.	Pubmed	0.0044	65.1	0.0114	0.116	1	1	HBB
Pubmed	22916308	Filamins but not Janus kinases are substrates of the ASB2a cullin-ring E3 ubiquitin ligase in hematopoietic cells.	GeneRIF	0.0044	65.1	0.0114	0.116	1	1	ASB2
Pubmed	8330974	Hb Alesha or alpha 2 beta (2)67(E11)Val-->Met: a new unstable hemoglobin variant identified through sequencing of amplified DNA.	Pubmed	0.0044	65.1	0.0114	0.116	1	1	HBB
Pubmed	3038530	Primary structure of the gene for the murine Ia antigen-associated invariant chains (Ii). An alternatively spliced exon encodes a cysteine-rich domain highly homologous to a repetitive sequence of thyroglobulin.	Pubmed	0.0044	65.1	0.0114	0.116	1	1	CD74
Pubmed	15023533	Exchange of serine residues 263 and 266 reduces the function of mouse gap junction protein connexin31 and exhibits a dominant-negative effect on the wild-type protein in HeLa cells.	GeneRIF	0.0044	65.1	0.0114	0.116	1	1	GJB3
Pubmed	16080200	Amplification and overexpression of prosaposin in prostate cancer.	GeneRIF	0.0044	65.1	0.0114	0.116	1	1	PSAP
Pubmed	22876326	Isoform-specific dominant-negative effects associated with hERG1 G628S mutation in long QT syndrome.	GeneRIF	0.0044	65.1	0.0114	0.116	1	1	KCNH2
Pubmed	20450755	[Polymorphism of CYP11A1 gene in Chinese patients with polycystic ovarian syndrome].	GeneRIF	0.0044	65.1	0.0114	0.116	1	1	CYP11A1
Pubmed	11736906	HLA-DQB1 alleles in Italian patients with mucous membrane pemphigoid predominantly affecting the oral cavity.	GeneRIF	0.0044	65.1	0.0114	0.116	1	1	HLA-DQB1
Pubmed	7965120	Hemoglobin augmentation of interleukin-1 beta-induced production of nitric oxide in smooth-muscle cells.	Pubmed	0.0044	65.1	0.0114	0.116	1	1	HBB
Pubmed	22110769	Dysferlin forms a dimer mediated by the C2 domains and the transmembrane domain in vitro and in living cells.	GeneRIF	0.0044	65.1	0.0114	0.116	1	1	DYSF
Pubmed	23692058	Inhibin B levels in hypothyroid males.	Pubmed	0.0044	65.1	0.0114	0.116	1	1	INHBB
Pubmed	15648546	Peripheral type benzodiazepine receptor in human parathyroid glands: up-regulation in adenoma.	GeneRIF	0.0044	65.1	0.0114	0.116	1	1	TSPO
Pubmed	21413144	Generation of mice with a conditional allele for the p75(NTR) neurotrophin receptor gene.	Pubmed	0.0044	65.1	0.0114	0.116	1	1	NGFR
Pubmed	7523374	Type VI RNA is the major gamma-glutamyl transpeptidase RNA in the mouse small intestine.	Pubmed	0.0044	65.1	0.0114	0.116	1	1	GGT1
Pubmed	10500303	Peptides, enzymes and obesity: new insights from a 'dead' enzyme.	Pubmed	0.0044	65.1	0.0114	0.116	1	1	CPE
Pubmed	11407979	Calmodulin-like skin protein: a new marker of keratinocyte differentiation.	Pubmed	0.0044	65.1	0.0114	0.116	1	1	CALML5
Pubmed	23973664	The Orosomucoid 1 protein is involved in the vitamin D - mediated macrophage de-activation process.	Pubmed	0.0044	65.1	0.0114	0.116	1	1	ORM1

Category	ID	Name	Source	p-value	q-value Bonferroni	q-value FDR B&H	q-value FDR B&Y	Hit Count in Query List	Hit Count in Genome	Hit in Query List
Pubmed	22580281	A422T mutation in HERG potassium channel retained in ER is rescurable by pharmacologic or molecular chaperones.	GeneRIF	0.0044	65.1	0.0114	0.116	1	1	KCNH2
Pubmed	14595541	Absence of association between SPINK1 trypsin inhibitor mutations and Type 1 or 2 diabetes mellitus in India and Germany.	GeneRIF	0.0044	65.1	0.0114	0.116	1	1	SPINK1
Pubmed	12366786	Corneodesmosin DNA polymorphisms in MHC haplotypes and Japanese patients with psoriasis.	GeneRIF	0.0044	65.1	0.0114	0.116	1	1	CDSN
Pubmed	9267038	Pinealocyte ultrastructure in mutant mice that lack sympathetic innervation to the pineal gland.	Pubmed	0.0044	65.1	0.0114	0.116	1	1	NGFR
Pubmed	15778703	Blockade of HERG cardiac K+ current by antifungal drug miconazole.	GeneRIF	0.0044	65.1	0.0114	0.116	1	1	KCNH2
Pubmed	24377640	Negative association of the HLA-DQB1*02 allele with breast cancer development among Jordanians.	Pubmed	0.0044	65.1	0.0114	0.116	1	1	HLA-DQB1
Pubmed	10029606	Four new mutations in the erythroid-specific 5-aminolevulinate synthase (ALAS2) gene causing X-linked sideroblastic anemia: increased pyridoxine responsiveness after removal of iron overload by phlebotomy and coinheritance of hereditary hemochromatosis.	Pubmed	0.0044	65.1	0.0114	0.116	1	1	ALAS2
Pubmed	20621031	Role of endothelial lipase in atherosclerosis.	GeneRIF	0.0044	65.1	0.0114	0.116	1	1	LIPG
Pubmed	11924532	Sequencing and chromosomal assignment of the rat endothelial-derived lipase gene (Lipg).	Pubmed	0.0044	65.1	0.0114	0.116	1	1	LIPG
Pubmed	23089702	d0-Thalassemia in cis of ßKnossos globin gene: first homozygous description in thalassemia intermedia Libyans and first combination with codon 39 (C¿T) in thalassemia intermedia Tunisian patients.	GeneRIF	0.0044	65.1	0.0114	0.116	1	1	HBB
Pubmed	25422423	Structural basis of collagen fiber degradation by cathepsin K.	Pubmed	0.0044	65.1	0.0114	0.116	1	1	CTSK
Pubmed	8268805	Heme biosynthesis in mammalian systems: evidence of a Schiff base linkage between the pyridoxal 5'-phosphate cofactor and a lysine residue in 5-aminolevulinate synthase.	Pubmed	0.0044	65.1	0.0114	0.116	1	1	ALAS2
Pubmed	15052692	Metabolic syndrome is directly associated with gamma glutamyl transpeptidase elevation in Japanese women.	GeneRIF	0.0044	65.1	0.0114	0.116	1	1	GGT1
Pubmed	24168369	Concentration-dependent bimodal effect of specific 18 kDa translocator protein (TSPO) ligands on cell death processes induced by ammonium chloride: potential implications for neuropathological effects due to hyperammonemia.	Pubmed	0.0044	65.1	0.0114	0.116	1	1	TSPO
Pubmed	24058567	Transglutaminase 2 overexpression in tumor stroma identifies invasive ductal carcinomas of breast at high risk of recurrence.	Pubmed	0.0044	65.1	0.0114	0.116	1	1	TGM2
Pubmed	16773658	BMP induction of cardiogenesis in P19 cells requires prior cell-cell interaction(s).	GeneRIF	0.0044	65.1	0.0114	0.116	1	1	BMP4
Pubmed	7890676	Molecular cloning and expression of murine and bovine endothelial cell protein C/activated protein C receptor (EPCR). The structural and functional conservation in human, bovine, and murine EPCR.	Pubmed	0.0044	65.1	0.0114	0.116	1	1	PROCR
Pubmed	19026664	Deletion of p75NTR impairs regeneration of peripheral nerves in mice.	GeneRIF	0.0044	65.1	0.0114	0.116	1	1	NGFR
Pubmed	12754508	Hypotrichosis simplex of the scalp is associated with nonsense mutations in CDSN encoding corneodesmosin.	GeneRIF	0.0044	65.1	0.0114	0.116	1	1	CDSN
Pubmed	16731063	Identification and characterization of the Mustang promoter: regulation by AP-1 during myogenic differentiation.	Pubmed	0.0044	65.1	0.0114	0.116	1	1	MUSTN1
Pubmed	7889573	A molecular basis for cardiac arrhythmia: HERG mutations cause long QT syndrome.	Pubmed	0.0044	65.1	0.0114	0.116	1	1	KCNH2
Pubmed	24586431	Tissue kallikrein mediates pro-inflammatory pathways and activation of protease-activated receptor-4 in proximal tubular epithelial cells.	Pubmed	0.0044	65.1	0.0114	0.116	1	1	KLK1
Pubmed	17043407	X-linked Opitz G/BBB syndrome: identification of a novel mutation and prenatal diagnosis in a Korean family.	GeneRIF	0.0044	65.1	0.0114	0.116	1	1	MID1
Pubmed	3839802	Identification of a point mutation in the adenosine deaminase gene responsible for immunodeficiency.	Pubmed	0.0044	65.1	0.0114	0.116	1	1	ADA
Pubmed	23249718	[The expression level and clinical significance of MMP-7 protein in peripheral blood in the patients with lung cancer].	Pubmed	0.0044	65.1	0.0114	0.116	1	1	MMP7
Pubmed	23350603	The cysteine dioxgenase knockout mouse: altered cysteine metabolism in nonhepatic tissues leads to excess H2S/HS(-) production and evidence of pancreatic and lung toxicity.	Pubmed	0.0044	65.1	0.0114	0.116	1	1	CDO1
Pubmed	19176112	[Single nucleotide polymorphisms of promoter of human leukocyte antigen-DQB1 alleles in Chinese Han patients with Vogt-Koyanagi-Harada syndrome].	GeneRIF	0.0044	65.1	0.0114	0.116	1	1	HLA-DQB1
Pubmed	24784578	Rapid actin-cytoskeleton-dependent recruitment of plasma membrane-derived dysferlin at wounds is critical for muscle membrane repair.	Pubmed	0.0044	65.1	0.0114	0.116	1	1	DYSF
Pubmed	17510972	Effects of IFI16 overexpression on the growth and doxorubicin sensitivity of head and neck squamous cell carcinoma-derived cell lines.	GeneRIF	0.0044	65.1	0.0114	0.116	1	1	IFI16
Pubmed	16002559	Augmented responses to ozone in obese carboxypeptidase E-deficient mice.	Pubmed	0.0044	65.1	0.0114	0.116	1	1	CPE
Pubmed	19213032	Two novel STRA6 mutations in a patient with anophthalmia and diaphragmatic eventration.	GeneRIF	0.0044	65.1	0.0114	0.116	1	1	STRA6
Pubmed	25523132	Trop2: from development to disease.	Pubmed	0.0044	65.1	0.0114	0.116	1	1	TACSTD2
Pubmed	22681493	A missense mutation in the GJB3 gene responsible for erythrokeratodermia variabilis in a Chinese family.	GeneRIF	0.0044	65.1	0.0114	0.116	1	1	GJB3
Pubmed	21055387	NMR solution structure of the N-terminal domain of hERG and its interaction with the S4-S5 linker.	GeneRIF	0.0044	65.1	0.0114	0.116	1	1	KCNH2
Pubmed	24100324	Structure of fully liganded Hb ¿2ß2s trapped in a tense conformation.	Pubmed	0.0044	65.1	0.0114	0.116	1	1	HBA2
Pubmed	23077045	The soluble isoform of CX3CL1 is necessary for neuroprotection in a mouse model of Parkinson's disease.	Pubmed	0.0044	65.1	0.0114	0.116	1	1	CX3CL1

Category	ID	Name	Source	p-value	q-value Bonferroni	q-value FDR B&H	q-value FDR B&Y	Hit Count in Query List	Hit Count in Genome	Hit in Query List
Pubmed	2578891	Keratinocyte-specific transglutaminase of cultured human epidermal cells: relation to cross-linked envelope formation and terminal differentiation.	Pubmed	0.0044	65.1	0.0114	0.116	1	1	TGM2
Pubmed	18720384	Oversulfated chondroitin sulfate-E binds to BMP-4 and enhances osteoblast differentiation.	GeneRIF	0.0044	65.1	0.0114	0.116	1	1	BMP4
Pubmed	15201665	Tissue transglutaminase: a mediator and predictor of chronic allograft nephropathy?	GeneRIF	0.0044	65.1	0.0114	0.116	1	1	TGM2
Pubmed	20074642	Characterization of P-Rex1 for its role in fMet-Leu-Phe-induced superoxide production in reconstituted COS(phox) cells.	GeneRIF	0.0044	65.1	0.0114	0.116	1	1	PREX1
Pubmed	11186260	Hb Nikaia [alpha20(B1)His-Asp]: a new variant of the alpha2 gene.	Pubmed	0.0044	65.1	0.0114	0.116	1	1	HBA2
Pubmed	15333584	A synonymous SNP of the corneodesmosin gene leads to increased mRNA stability and demonstrates association with psoriasis across diverse ethnic groups.	GeneRIF	0.0044	65.1	0.0114	0.116	1	1	CDSN
Pubmed	8114671	In vitro reconstitution of a functional peripheral-type benzodiazepine receptor from mouse Leydig tumor cells.	Pubmed	0.0044	65.1	0.0114	0.116	1	1	TSPO
Pubmed	19892732	PD-118057 contacts the pore helix of hERG1 channels to attenuate inactivation and enhance K+ conductance.	GeneRIF	0.0044	65.1	0.0114	0.116	1	1	KCNH2
Pubmed	7697546	Allelic differences affecting invariant chain dependency of MHC class II subunit assembly.	Pubmed	0.0044	65.1	0.0114	0.116	1	1	CD74
Pubmed	20606865	Association of G>A transition in exon-1 of alpha crystallin gene in age-related cataracts.	GeneRIF	0.0044	65.1	0.0114	0.116	1	1	CRYAA
Pubmed	15764155	Mutations N34S and P55S of the SPINK1 gene in patients with chronic pancreatitis or pancreatic cancer and in healthy subjects: a report from Finland.	GeneRIF	0.0044	65.1	0.0114	0.116	1	1	SPINK1
Pubmed	19595763	A novel mutation (F71L) in alphaA-crystallin with defective chaperone-like function associated with age-related cataract.	GeneRIF	0.0044	65.1	0.0114	0.116	1	1	CRYAA
Pubmed	9033587	Crystal structure of human cathepsin K complexed with a potent inhibitor.	Pubmed	0.0044	65.1	0.0114	0.116	1	1	CTSK
Pubmed	23090949	Spatial gradients in action potential duration created by regional magnetofection of hERG are a substrate for wavebreak and turbulent propagation in cardiomyocyte monolayers.	GeneRIF	0.0044	65.1	0.0114	0.116	1	1	KCNH2
Pubmed	15481884	Molecular basis of beta-thalassemia in the population of Tunisia.	GeneRIF	0.0044	65.1	0.0114	0.116	1	1	HBB
Pubmed	1567857	High-resolution X-ray study of deoxyhemoglobin Rothschild 37 beta Trp----Arg: a mutation that creates an intersubunit chloride-binding site.	Pubmed	0.0044	65.1	0.0114	0.116	1	1	HBB
Pubmed	10403572	Physiologic and endocrinologic characterization of male sex-biased diabetes in C57BLKS/J mice congenic for the fat mutation at the carboxypeptidease E locus.	Pubmed	0.0044	65.1	0.0114	0.116	1	1	CPE
Pubmed	15692663	Hemoglobinopathies in newborns from Salvador, Bahia, Northeast Brazil.	GeneRIF	0.0044	65.1	0.0114	0.116	1	1	HBB
Pubmed	23192962	Drug-binding energetics of human a-1-acid glycoprotein assessed by isothermal titration calorimetry and molecular docking simulations.	GeneRIF	0.0044	65.1	0.0114	0.116	1	1	ORM1
Pubmed	21209087	Novel androstenetriol interacts with the mitochondrial translocator protein and controls steroidogenesis.	GeneRIF	0.0044	65.1	0.0114	0.116	1	1	TSPO
Pubmed	22964610	Early LQT2 nonsense mutation generates N-terminally truncated hERG channels with altered gating properties by the reinitiation of translation.	GeneRIF	0.0044	65.1	0.0114	0.116	1	1	KCNH2
Pubmed	14627645	Thyroid hormone induces cerebellar Purkinje cell dendritic development via the thyroid hormone receptor alpha1.	GeneRIF	0.0044	65.1	0.0114	0.116	1	1	THRA
Pubmed	17486493	Three new beta-globin gene promoter mutations identified through newborn screening.	GeneRIF	0.0044	65.1	0.0114	0.116	1	1	HBB
Pubmed	17519411	Hemoglobin variants and disease manifestations in severe falciparum malaria.	GeneRIF	0.0044	65.1	0.0114	0.116	1	1	HBB
Pubmed	4531009	Sites of acetylation of sickle cell hemoglobin by aspirin.	Pubmed	0.0044	65.1	0.0114	0.116	1	1	HBB
Pubmed	17715390	Tissue-specific histone modification and transcription factor binding in alpha globin gene expression.	GeneRIF	0.0044	65.1	0.0114	0.116	1	1	HBA2
Pubmed	9143491	Genomic organization and chromosome localization of the human cathepsin K gene (CTSK).	Pubmed	0.0044	65.1	0.0114	0.116	1	1	CTSK
Pubmed	17010111	Identification of plant cytoskeleton-interacting proteins by screening for actin stress fiber association in mammalian fibroblasts.	Pubmed	0.0044	65.1	0.0114	0.116	1	1	ACTA1
Pubmed	25271992	Blood typing profile of a school-aged population o a North Togo township.	Pubmed	0.0044	65.1	0.0114	0.116	1	1	HBB
Pubmed	25500521	a-Thalassemia with Haemoglobin Adana mutation: prenatal diagnosis.	Pubmed	0.0044	65.1	0.0114	0.116	1	1	HBA2
Pubmed	21348942	The histone demethylase JARID1A regulates progesterone receptor expression.	GeneRIF	0.0044	65.1	0.0114	0.116	1	1	KDM5A
Pubmed	12021132	Molecular modeling of eluted peptides from DQ6 molecules (DQB1*0602 and DQB1*0604) negatively and positively associated with type 1 diabetes.	GeneRIF	0.0044	65.1	0.0114	0.116	1	1	HLA-DQB1
Pubmed	18184876	Apparent intermediate K conductance channel hyposmotic activation in human lens epithelial cells.	GeneRIF	0.0044	65.1	0.0114	0.116	1	1	KCNN4
Pubmed	19863187	Cultured human cells release soluble gamma-glutamyltransferase complexes corresponding to the plasma b-GGT.	GeneRIF	0.0044	65.1	0.0114	0.116	1	1	GGT1
Pubmed	18345496	Minimization of potential hERG liability in histamine H3 receptor antagonists.	GeneRIF	0.0044	65.1	0.0114	0.116	1	1	KCNH2
Pubmed	24139879	Soluble B7-H3 elevations in hospitalized children with Mycoplasma pneumoniae pneumonia.	Pubmed	0.0044	65.1	0.0114	0.116	1	1	CD276
Pubmed	17470426	Role of Scarf and its binding target proteins in epidermal calcium homeostasis.	GeneRIF	0.0044	65.1	0.0114	0.116	1	1	CALML5
Pubmed	10937555	Rapid detection of M1S1 mutations by the protein truncation test.	Pubmed	0.0044	65.1	0.0114	0.116	1	1	TACSTD2
Pubmed	24409061	Smoothelin, a new marker to determine the origin of liver fibrogenic cells.	Pubmed	0.0044	65.1	0.0114	0.116	1	1	SMTN

Category	ID	Name	Source	p-value	q-value Bonferroni	q-value FDR B&H	q-value FDR B&Y	Hit Count in Query List	Hit Count in Genome	Hit in Query List
Pubmed	22681753	Protective effects of transgenic human endothelial protein C receptor expression in murine models of transplantation.	GeneRIF	0.0044	65.1	0.0114	0.116	1	1	PROCR
Pubmed	10092762	Chronic modulation of the TCR repertoire in the lymphoid periphery.	Pubmed	0.0044	65.1	0.0114	0.116	1	1	HLA-DQB1
Pubmed	21797702	Molecular basis of ß-thalassemia in the western province of Saudi Arabia: identification of rare ß-thalassemia mutations.	GeneRIF	0.0044	65.1	0.0114	0.116	1	1	HBB
Pubmed	23100416	Metalloproteinases and their associated genes contribute to the functional integrity and noise-induced damage in the cochlear sensory epithelium.	Pubmed	0.0044	65.1	0.0114	0.116	1	1	MMP7
Pubmed	11463606	Sensory nerves promote ozone-induced lung inflammation in mice.	Pubmed	0.0044	65.1	0.0114	0.116	1	1	NGFR
Pubmed	12612061	Selective knockout of mouse ERG1 B potassium channel eliminates I(Kr) in adult ventricular myocytes and elicits episodes of abrupt sinus bradycardia.	GeneRIF	0.0044	65.1	0.0114	0.116	1	1	KCNH2
Pubmed	24269275	Exome sequencing identifies CTSK mutations in patients originally diagnosed as intermediate osteopetrosis.	Pubmed	0.0044	65.1	0.0114	0.116	1	1	CTSK
Pubmed	21543648	The Ia.2 epitope defines a subset of lipid raft-resident MHC class II molecules crucial to effective antigen presentation.	Pubmed	0.0044	65.1	0.0114	0.116	1	1	HLA-DQB1
Pubmed	25070563	The role of strong electrostatic interactions at the dimer interface of human glutathione synthetase.	Pubmed	0.0044	65.1	0.0114	0.116	1	1	GSS
Pubmed	16467651	The functional HERG variant 897T is associated with Conn's adenoma.	GeneRIF	0.0044	65.1	0.0114	0.116	1	1	KCNH2
Pubmed	18276916	Distinct regulation of developmental and heart disease-induced atrial natriuretic factor expression by two separate distal sequences.	GeneRIF	0.0044	65.1	0.0114	0.116	1	1	NPPA
Pubmed	11436557	[Effect of genotype and social stress on cAMP-and substrate-dependent mechanisms of regulating hormonal function of testis in mice].	Pubmed	0.0044	65.1	0.0114	0.116	1	1	BMP4
Pubmed	24685135	Polarized expression of p75(NTR) specifies axons during development and adult neurogenesis.	Pubmed	0.0044	65.1	0.0114	0.116	1	1	NGFR
Pubmed	24368139	Rho-kinase limits BMP-4-stimulated osteocalcin synthesis in osteoblasts: regulation of the p38 MAP kinase pathway.	Pubmed	0.0044	65.1	0.0114	0.116	1	1	BMP4
Pubmed	24481335	Endoplasmic reticulum stress activates transglutaminase 2 leading to protein aggregation.	Pubmed	0.0044	65.1	0.0114	0.116	1	1	TGM2
Pubmed	20455895	Autoimmunity predominates in a large South African cohort with Addison's disease of mainly European descent despite long-standing disease and is associated with HLA DQB*0201.	GeneRIF	0.0044	65.1	0.0114	0.116	1	1	HLA-DQB1
Pubmed	19661345	Prognostic impact of CD68 and kallikrein 6 in human glioma.	GeneRIF	0.0044	65.1	0.0114	0.116	1	1	CD68
Pubmed	19154415	Overexpression of protease inhibitor-dead secretory leukocyte protease inhibitor causes more aggressive ovarian cancer in vitro and in vivo.	GeneRIF	0.0044	65.1	0.0114	0.116	1	1	SLPI
Pubmed	14584048	Effects of peripheral benzodiazepine receptor ligands on proliferation and differentiation of human mesenchymal stem cells.	GeneRIF	0.0044	65.1	0.0114	0.116	1	1	TSPO
Pubmed	18654889	An unusually frequent beta-thalassemia mutation in an Iranian Province.	GeneRIF	0.0044	65.1	0.0114	0.116	1	1	HBB
Pubmed	9266755	Structural analysis of the mouse prosaposin (SGP-1) gene reveals the presence of an exon that is alternatively spliced in transcribed mRNAs.	Pubmed	0.0044	65.1	0.0114	0.116	1	1	PSAP
Pubmed	16555252	Loss of p75 neurotrophin receptor expression accompanies malignant progression to human and murine retinoblastoma.	GeneRIF	0.0044	65.1	0.0114	0.116	1	1	NGFR
Pubmed	22326684	The function of tissue transglutaminase in celiac disease.	GeneRIF	0.0044	65.1	0.0114	0.116	1	1	TGM2
Pubmed	23134353	L539 fs/47, a truncated mutation of human ether-a-go-go-related gene (hERG), decreases hERG ion channel currents in HEK 293 cells.	GeneRIF	0.0044	65.1	0.0114	0.116	1	1	KCNH2
Pubmed	23263658	Identification of a novel cell-penetrating peptide from human phosphatidate phosphatase LPIN3.	Pubmed	0.0044	65.1	0.0114	0.116	1	1	LPIN3
Pubmed	11300351	Identification of Hb Villejuif [beta123(H1)Thr-->Ile] in Southern Italy.	Pubmed	0.0044	65.1	0.0114	0.116	1	1	HBB
Pubmed	7592575	Tissue-specific rescue suggests that placental adenosine deaminase is important for fetal development in mice.	Pubmed	0.0044	65.1	0.0114	0.116	1	1	ADA
Pubmed	11687514	A novel mutation in the M1S1 gene responsible for gelatinous droplike corneal dystrophy.	Pubmed	0.0044	65.1	0.0114	0.116	1	1	TACSTD2
Pubmed	24939590	Novel mutations in PXDN cause microphthalmia and anterior segment dysgenesis.	Pubmed	0.0044	65.1	0.0114	0.116	1	1	PXDN
Pubmed	1497627	Molecular characterization of a low-molecular-mass matrix metalloproteinase secreted by glomerular mesangial cells as PUMP-1.	Pubmed	0.0044	65.1	0.0114	0.116	1	1	MMP7
Pubmed	1316514	Morphologic analysis of enteric lesions in conventional and streptomycin-treated inbred C3H/HeN mice infected with Serpulina (Treponema) hyodysenteriae.	Pubmed	0.0044	65.1	0.0114	0.116	1	1	CPE
Pubmed	15460447	Tissue transglutaminase is not involved in the aggregate formation of stably expressed alpha-synuclein in SH-SY5Y human neuroblastoma cells.	GeneRIF	0.0044	65.1	0.0114	0.116	1	1	TGM2
Pubmed	22194990	Comparison of dysferlin expression in human skeletal muscle with that in monocytes for the diagnosis of dysferlin myopathy.	GeneRIF	0.0044	65.1	0.0114	0.116	1	1	DYSF
Pubmed	19114089	Attenuation of opioid analgesic tolerance in p75 neurotrophin receptor null mutant mice.	GeneRIF	0.0044	65.1	0.0114	0.116	1	1	NGFR
Pubmed	20547545	I-Ag7 is subject to post-translational chaperoning by CLIP.	GeneRIF	0.0044	65.1	0.0114	0.116	1	1	CD74
Pubmed	11317658	Feasibility of genetic and immunological prediction of type I diabetes in a population-based birth cohort.	GeneRIF	0.0044	65.1	0.0114	0.116	1	1	HLA-DQB1
Pubmed	16042135	[HLA DQB1 genotyping in latent autoimmune diabetes of adults (LADA)].	GeneRIF	0.0044	65.1	0.0114	0.116	1	1	HLA-DQB1

Category	ID	Name	Source	p-value	q-value Bonferroni	q-value FDR B&H	q-value FDR B&Y	Hit Count in Query List	Hit Count in Genome	Hit in Query List
Pubmed	9878261	Identification of PKD2L, a human PKD2-related gene: tissue-specific expression and mapping to chromosome 10q25.	Pubmed	0.0044	65.1	0.0114	0.116	1	1	PKD2L1
Pubmed	21232998	Analyzing 5'HS3 and 5'HS4 LCR core regions and NF-E2 in Iranian thalassemia intermedia patients with normal or carrier status for beta-globin mutations.	GeneRIF	0.0044	65.1	0.0114	0.116	1	1	HBB
Pubmed	11224528	B7-H3: a costimulatory molecule for T cell activation and IFN-gamma production.	Pubmed	0.0044	65.1	0.0114	0.116	1	1	CD276
Pubmed	23468018	Role of BMP signaling in pancreatic progenitor differentiation from human embryonic stem cells.	Pubmed	0.0044	65.1	0.0114	0.116	1	1	BMP4
Pubmed	19839778	Association between bone morphogenetic protein 4 gene polymorphisms with nonsyndromic cleft lip with or without cleft palate in a chilean population.	GeneRIF	0.0044	65.1	0.0114	0.116	1	1	BMP4
Pubmed	17531263	A single hERG mutation underlying a spectrum of acquired and congenital long QT syndrome phenotypes.	GeneRIF	0.0044	65.1	0.0114	0.116	1	1	KCNH2
Pubmed	22120592	Acetylation of aA-crystallin in the human lens: effects on structure and chaperone function.	GeneRIF	0.0044	65.1	0.0114	0.116	1	1	CRYAA
Pubmed	19488752	A novel beta-globin mutation (HBB:c.107A>G; or codon 35 beta (A-->G)) at alpha-beta chain interfaces.	GeneRIF	0.0044	65.1	0.0114	0.116	1	1	HBB
Pubmed	21125972	Elevated serum gamma-glutamyl transferase activity in patients with cardiac syndrome X and its relationship with carotid intima media thickness.	GeneRIF	0.0044	65.1	0.0114	0.116	1	1	GGT1
Pubmed	22968129	Incomplete segregation of MYH11 variants with thoracic aortic aneurysms and dissections and patent ductus arteriosus.	Pubmed	0.0044	65.1	0.0114	0.116	1	1	MYH11
Pubmed	21129482	Transglutaminase 2 regulates early chondrogenesis and glycosaminoglycan synthesis.	GeneRIF	0.0044	65.1	0.0114	0.116	1	1	TGM2
Pubmed	23080515	Role of translocator protein in melanoma growth and progression.	GeneRIF	0.0044	65.1	0.0114	0.116	1	1	TSPO
Pubmed	9363777	Molecular cloning and expression of an interferon-inducible protein encoded by gene 203 from the gene 200 cluster.	Pubmed	0.0044	65.1	0.0114	0.116	1	1	IFI16
Pubmed	24594926	Assessment of Nogo-66 receptor 1 function in vivo after spinal cord injury.	Pubmed	0.0044	65.1	0.0114	0.116	1	1	RTN4R
Pubmed	18923241	Increased tissue transglutaminase expression in human atherosclerotic coronary arteries.	GeneRIF	0.0044	65.1	0.0114	0.116	1	1	TGM2
Pubmed	16515559	Matrix metalloproteinase-7 disrupts dendritic spines in hippocampal neurons through NMDA receptor activation.	GeneRIF	0.0044	65.1	0.0114	0.116	1	1	MMP7
Pubmed	10561547	Biochemical evidence that Escherichia coli hyi (orf b0508, gip) gene encodes hydroxypyruvate isomerase.	Pubmed	0.0044	65.1	0.0114	0.116	1	1	HYI
Pubmed	17387733	The pathogenesis of ACTA1-related congenital fiber type disproportion.	GeneRIF	0.0044	65.1	0.0114	0.116	1	1	ACTA1
Pubmed	18688858	Overexpression of a secretory leukocyte protease inhibitor in human gastric cancer.	GeneRIF	0.0044	65.1	0.0114	0.116	1	1	SLPI
Pubmed	19556751	A pilot beta-thalassaemia screening program in the Albanian population for a health planning program.	GeneRIF	0.0044	65.1	0.0114	0.116	1	1	HBB
Pubmed	9828198	Developmental profile of a caltrin-like protease inhibitor, P12, in mouse seminal vesicle and characterization of its binding sites on sperm surface.	Pubmed	0.0044	65.1	0.0114	0.116	1	1	SPINK1
Pubmed	12209010	Position of aromatic residues in the S6 domain, not inactivation, dictates cisapride sensitivity of HERG and eag potassium channels.	GeneRIF	0.0044	65.1	0.0114	0.116	1	1	KCNH2
Pubmed	17981725	Multiple splicing results in at least two p203 proteins that are expressed in the liver and down-regulated during liver regeneration.	GeneRIF	0.0044	65.1	0.0114	0.116	1	1	IFI16
Pubmed	17693551	Structural basis of action for a human ether-a-go-go-related gene 1 potassium channel activator.	GeneRIF	0.0044	65.1	0.0114	0.116	1	1	KCNH2
Pubmed	15477515	Phenotypic features and genetic findings in 2 chinese families with Miyoshi distal myopathy.	GeneRIF	0.0044	65.1	0.0114	0.116	1	1	DYSF
Pubmed	7903404	Nerve growth factor receptor expression in fetal, mature, and diseased human kidneys.	Pubmed	0.0044	65.1	0.0114	0.116	1	1	NGFR
Pubmed	3384710	Hemoglobin Brest [beta 127 (H5)Gln----Lys] a new unstable human hemoglobin variant located at the alpha 1 beta 1 interface with specific electrophoretic behavior.	Pubmed	0.0044	65.1	0.0114	0.116	1	1	HBB
Pubmed	22253730	Mutation of Rubie, a novel long non-coding RNA located upstream of Bmp4, causes vestibular malformation in mice.	GeneRIF	0.0044	65.1	0.0114	0.116	1	1	BMP4
Pubmed	20230396	Epidemiology of beta-thalassaemia in Western India: mapping the frequencies and mutations in sub-regions of Maharashtra and Gujarat.	GeneRIF	0.0044	65.1	0.0114	0.116	1	1	HBB
Pubmed	7691243	Long-term in vivo expression of a murine adenosine deaminase gene in rhesus monkey hematopoietic cells of multiple lineages after retroviral mediated gene transfer into CD34+ bone marrow cells.	Pubmed	0.0044	65.1	0.0114	0.116	1	1	ADA
Pubmed	9543632	Quantitation of asparagine-101 deamidation from alpha-A crystallin during aging of the human lens.	Pubmed	0.0044	65.1	0.0114	0.116	1	1	CRYAA
Pubmed	20489165	Decreased S-nitrosylation of tissue transglutaminase contributes to age-related increases in vascular stiffness.	GeneRIF	0.0044	65.1	0.0114	0.116	1	1	TGM2
Pubmed	20175216	A mutation in the saposin C domain of the sphingolipid activator protein (Prosaposin) gene causes neurodegenerative disease in mice.	GeneRIF	0.0044	65.1	0.0114	0.116	1	1	PSAP
Pubmed	12030366	Gamma-glutamyl transpeptidase activity mediates NF-kappaB activation through lipid peroxidation in human leukemia U937 cells.	GeneRIF	0.0044	65.1	0.0114	0.116	1	1	GGT1
Pubmed	22554054	Biochemical and functional characterisation of aPIX, a specific regulator of axonal and dendritic branching in hippocampal neurons.	GeneRIF	0.0044	65.1	0.0114	0.116	1	1	ARHGEF6
Pubmed	12887413	Distribution of central sensory axons in transgenic mice overexpressing nerve growth factor and lacking functional p75 neurotrophin receptor expression.	Pubmed	0.0044	65.1	0.0114	0.116	1	1	NGFR
Pubmed	20054848	Identification and molecular characterization of the --CAMPANIA deletion, a novel alpha (0) -thalassemic defect, in two unrelated Italian families [corrected].	GeneRIF	0.0044	65.1	0.0114	0.116	1	1	HBA2

Category	ID	Name	Source	p-value	q-value Bonferroni	q-value FDR B&H	q-value FDR B&Y	Hit Count in Query List	Hit Count in Genome	Hit in Query List
Pubmed	12354768	A novel mutation (T65P) in the PAS domain of the human potassium channel HERG results in the long QT syndrome by trafficking deficiency.	GeneRIF	0.0044	65.1	0.0114	0.116	1	1	KCNH2
Pubmed	18587533	Tissue transgluaminase 2 expression in meningiomas.	GeneRIF	0.0044	65.1	0.0114	0.116	1	1	TGM2
Pubmed	11741197	Molecular analysis of the beta-globin gene cluster in the Niokholo Mandenka population reveals a recent origin of the beta(S) Senegal mutation.	GeneRIF	0.0044	65.1	0.0114	0.116	1	1	HBB
Pubmed	9771894	Altered processing of procholecystokinin in carboxypeptidase E-deficient fat mice: differential synthesis in neurons and endocrine cells.	Pubmed	0.0044	65.1	0.0114	0.116	1	1	CPE
Pubmed	16735993	A novel fan-shaped cataract-microcornea syndrome caused by a mutation of CRYAA in an Indian family.	GeneRIF	0.0044	65.1	0.0114	0.116	1	1	CRYAA
Pubmed	24590570	Altered hematopoietic stem cell and osteoclast precursor frequency in cathepsin K null mice.	Pubmed	0.0044	65.1	0.0114	0.116	1	1	CTSK
Pubmed	18257074	Beta-globin gene cluster haplotypes and alpha-thalassemia in sickle cell disease patients from Trinidad.	GeneRIF	0.0044	65.1	0.0114	0.116	1	1	HBB
Pubmed	11804364	Prenatal diagnosis of haemoglobinopathies.	GeneRIF	0.0044	65.1	0.0114	0.116	1	1	HBB
Pubmed	23398480	Identification of thyroid hormone response elements in vivo using mice expressing a tagged thyroid hormone receptor a1.	Pubmed	0.0044	65.1	0.0114	0.116	1	1	THRA
Pubmed	7945328	Possible involvement of GTP-binding proteins in 1 alpha,25-dihydroxyvitamin D3 induction of tissue transglutaminase in mouse peritoneal macrophages.	Pubmed	0.0044	65.1	0.0114	0.116	1	1	TGM2
Pubmed	9079649	Exon 6 is essential for invariant chain trimerization and induction of large endosomal structures.	Pubmed	0.0044	65.1	0.0114	0.116	1	1	CD74
Pubmed	20559678	Requirements for leukocyte transmigration via the transmembrane chemokine CX3CL1.	GeneRIF	0.0044	65.1	0.0114	0.116	1	1	CX3CL1
Pubmed	12572894	Part of CD68+ macrophages in the clearence of apoptotic bodies in human metanephros.	Pubmed	0.0044	65.1	0.0114	0.116	1	1	CD68
Pubmed	17182551	Genetic manipulation of CD74 in mouse strains of different backgrounds can result in opposite responses to central nervous system injury.	GeneRIF	0.0044	65.1	0.0114	0.116	1	1	CD74
Pubmed	10458770	Fractalkine, a CX3C chemokine, is expressed by dendritic cells and is up-regulated upon dendritic cell maturation.	Pubmed	0.0044	65.1	0.0114	0.116	1	1	CX3CL1
Pubmed	18629540	BMP4 signaling regulates formation of Hertwig's epithelial root sheath during tooth root development.	GeneRIF	0.0044	65.1	0.0114	0.116	1	1	BMP4
Pubmed	9525678	Physiological knockout of the envelope gene of the single-copy ERV-3 human endogenous retrovirus in a fraction of the Caucasian population.	Pubmed	0.0044	65.1	0.0114	0.116	1	1	ERV3-1
Pubmed	20979053	Down-regulation of tumor suppressor A kinase anchor protein 12 in human hepatocarcinogenesis by epigenetic mechanisms.	GeneRIF	0.0044	65.1	0.0114	0.116	1	1	AKAP12
Pubmed	24402571	Sex differences in translocator protein 18 kDa (TSPO) in the heart: implications for imaging myocardial inflammation.	Pubmed	0.0044	65.1	0.0114	0.116	1	1	TSPO
Pubmed	20181291	The molecular basis of beta-thalassemia intermedia in southern China: genotypic heterogeneity and phenotypic diversity.	GeneRIF	0.0044	65.1	0.0114	0.116	1	1	HBB
Pubmed	22311109	Cardiac and vascular atrogin-1 mRNA expression is not associated with dexamethasone efficacy in the monocrotaline model of pulmonary hypertension.	GeneRIF	0.0044	65.1	0.0114	0.116	1	1	FBXO32
Pubmed	22156835	Napsin A expression in lung and kidney neoplasia: a review and update.	GeneRIF	0.0044	65.1	0.0114	0.116	1	1	NAPSA
Pubmed	19469903	Expression and regulation of cathepsin K in skin fibroblasts.	GeneRIF	0.0044	65.1	0.0114	0.116	1	1	CTSK
Pubmed	12810527	Reversible skeletal abnormalities in gamma-glutamyl transpeptidase-deficient mice.	GeneRIF	0.0044	65.1	0.0114	0.116	1	1	GGT1
Pubmed	11939519	Identification of two new beta-thalassemia splice mutations: IVS-I-1 (G-->C) and IVS-I (-2) (A-->C).	Pubmed	0.0044	65.1	0.0114	0.116	1	1	HBB
Pubmed	9225964	Two newly identified mutations (Thr233Ile and Leu152Met) in partially adenosine deaminase-deficient (ADA-) individuals that result in differing biochemical and metabolic phenotypes.	Pubmed	0.0044	65.1	0.0114	0.116	1	1	ADA
Pubmed	9409787	Cloning of the murine tetranectin gene and 5'-flanking region.	Pubmed	0.0044	65.1	0.0114	0.116	1	1	CLEC3B
Pubmed	19423618	Increased CD74 expression in human atherosclerotic plaques: contribution to inflammatory responses in vascular cells.	GeneRIF	0.0044	65.1	0.0114	0.116	1	1	CD74
Pubmed	18928603	[Analysis of single nucleotide polymorphism in the human beta-globin gene of patients with minor beta-thalassemia from Wenzhou].	GeneRIF	0.0044	65.1	0.0114	0.116	1	1	HBB
Pubmed	19785773	MMP7 expression regulated by endocrine therapy in ERbeta-positive colon cancer cells.	GeneRIF	0.0044	65.1	0.0114	0.116	1	1	MMP7
Pubmed	21881323	Low-dose atrial natriuretic peptide for chronic kidney disease in coronary surgery.	GeneRIF	0.0044	65.1	0.0114	0.116	1	1	NPPA
Pubmed	22451718	Transglutaminase is essential for IgA nephropathy development acting through IgA receptors.	GeneRIF	0.0044	65.1	0.0114	0.116	1	1	TGM2
Pubmed	19670825	Hypertrophic gene expression induced by chronic stretch of excised mouse heart muscle.	GeneRIF	0.0044	65.1	0.0114	0.116	1	1	NPPA
Pubmed	22198799	Carboplatin-gemcitabine combination chemotherapy upregulates AKR1B10 expression in bladder cancer.	GeneRIF	0.0044	65.1	0.0114	0.116	1	1	AKR1B10
Pubmed	4639022	Hemoglobin Istanbul: substitution of glutamine for histidine in a proximal histidine (F8(92)).	Pubmed	0.0044	65.1	0.0114	0.116	1	1	HBB
Pubmed	21721952	Cathepsin K deficiency aggravates lung injury in hyperoxia-exposed newborn mice.	GeneRIF	0.0044	65.1	0.0114	0.116	1	1	CTSK
Pubmed	18591626	Defective mRNA levels are responsible for a beta-thalassemia phenotype associated with Hb Federico II, a novel hemoglobin variant [beta-106 (G8) Leu->Val].	GeneRIF	0.0044	65.1	0.0114	0.116	1	1	HBA2
Pubmed	12514744	Genetics analysis of mouse mutations Abnormal feet and tail and rough coat, which cause developmental abnormalities and alopecia.	Pubmed	0.0044	65.1	0.0114	0.116	1	1	MPZL3

Category	ID	Name	Source	p-value	q-value Bonferroni	q-value FDR B&H	q-value FDR B&Y	Hit Count in Query List	Hit Count in Genome	Hit in Query List
Pubmed	17938585	Expression and significance of HERG protein in gastric cancer.	GeneRIF	0.0044	65.1	0.0114	0.116	1	1	KCNH2
Pubmed	20712994	Phosphatidylinositol-4,5-bisphosphate (PIP(2)) stabilizes the open pore conformation of the Kv11.1 (hERG) channel.	GeneRIF	0.0044	65.1	0.0114	0.116	1	1	KCNH2
Pubmed	8037836	The contribution of H-2bm12 and non H-2 background genes on murine lupus in NZB.H-2bm12/b mice.	Pubmed	0.0044	65.1	0.0114	0.116	1	1	HLA-DQB1
Pubmed	19168574	Targeted inactivation of endothelial lipase attenuates lung allergic inflammation through raising plasma HDL level and inhibiting eosinophil infiltration.	GeneRIF	0.0044	65.1	0.0114	0.116	1	1	LIPG
Pubmed	16175509	The beta -globin recombinational hotspot reduces the effects of strong selection around HbC, a recently arisen mutation providing resistance to malaria.	Pubmed	0.0044	65.1	0.0114	0.116	1	1	HBB
Pubmed	16552049	Antibody-independent, interleukin-17A-mediated, cross-serotype immunity to pneumococci in mice immunized intranasally with the cell wall polysaccharide.	Pubmed	0.0044	65.1	0.0114	0.116	1	1	HLA-DQB1
Pubmed	25521803	Nonspecific reactivity of polyclonal napsin a antibody in mucinous adenocarcinomas of various sites: a word of caution.	Pubmed	0.0044	65.1	0.0114	0.116	1	1	NAPSA
Pubmed	23720728	Constitutive interferon-inducible protein 16-inflammasome activation during Epstein-Barr virus latency I, II, and III in B and epithelial cells.	GeneRIF	0.0044	65.1	0.0114	0.116	1	1	IFI16
Pubmed	25246042	Differential expression of leukocyte receptors in disseminated intravascular coagulation: prognostic value of low protein C receptor expression.	Pubmed	0.0044	65.1	0.0114	0.116	1	1	PROCR
Pubmed	9544837	Multiple different missense mutations in the pore region of HERG in patients with long QT syndrome.	Pubmed	0.0044	65.1	0.0114	0.116	1	1	KCNH2
Pubmed	23388637	BMP4-mediated brown fat-like changes in white adipose tissue alter glucose and energy homeostasis.	GeneRIF	0.0044	65.1	0.0114	0.116	1	1	BMP4
Pubmed	12885765	Identification of a COOH-terminal segment involved in maturation and stability of human ether-a-go-go-related gene potassium channels.	GeneRIF	0.0044	65.1	0.0114	0.116	1	1	KCNH2
Pubmed	16618111	Invariant chain transmembrane domain trimerization: a step in MHC class II assembly.	GeneRIF	0.0044	65.1	0.0114	0.116	1	1	CD74
Pubmed	25327250	Identification of multipotent mammary stem cells by protein C receptor expression.	Pubmed	0.0044	65.1	0.0114	0.116	1	1	PROCR
Pubmed	22315051	Prognostic significance of gamma-glutamyltransferase in patients with endometrial cancer: a multi-centre trial.	GeneRIF	0.0044	65.1	0.0114	0.116	1	1	GGT1
Pubmed	17761919	Association of kallikrein gene polymorphisms with intracranial aneurysms.	GeneRIF	0.0044	65.1	0.0114	0.116	1	1	KLK1
Pubmed	14649317	Hb Castilla [beta32(B14)Leu --> Arg] caused by a de novo mutation.	GeneRIF	0.0044	65.1	0.0114	0.116	1	1	HBB
Pubmed	16893505	Soluble mouse B7-H3 down-regulates dendritic cell stimulatory capacity to allogenic T cell proliferation and production of IL-2 and IFN-gamma.	GeneRIF	0.0044	65.1	0.0114	0.116	1	1	CD276
Pubmed	2110563	Location of the protease-inhibitory region of secretory leukocyte protease inhibitor.	Pubmed	0.0044	65.1	0.0114	0.116	1	1	SLPI
Pubmed	10770945	The crystal structure of the chemokine domain of fractalkine shows a novel quaternary arrangement.	Pubmed	0.0044	65.1	0.0114	0.116	1	1	CX3CL1
Pubmed	20466371	Lack of association between common genetic variation in endothelial lipase (LIPG) and the risk for CAD and DVT.	GeneRIF	0.0044	65.1	0.0114	0.116	1	1	LIPG
Pubmed	25453103	hERG 1b is critical for human cardiac repolarization.	Pubmed	0.0044	65.1	0.0114	0.116	1	1	KCNH2
Pubmed	21984378	Nitric oxide regulates tissue transglutaminase localization and function in the vasculature.	GeneRIF	0.0044	65.1	0.0114	0.116	1	1	TGM2
Pubmed	23118027	The immunoglobulin domain of the sodium channel β3 subunit contains a surface-localized disulfide bond that is required for homophilic binding.	GeneRIF	0.0044	65.1	0.0114	0.116	1	1	SCN3B
Pubmed	22414809	Extrahepatic tissues compensate for loss of hepatic taurine synthesis in mice with liver-specific knockout of cysteine dioxygenase.	GeneRIF	0.0044	65.1	0.0114	0.116	1	1	CDO1
Pubmed	12969973	Novel role of the membrane-bound chemokine fractalkine in platelet activation and adhesion.	GeneRIF	0.0044	65.1	0.0114	0.116	1	1	CX3CL1
Pubmed	25686748	Histone demethylase KDM5A is regulated by its reader domain through a positive-feedback mechanism.	Pubmed	0.0044	65.1	0.0114	0.116	1	1	KDM5A
Pubmed	11999881	Anti-adenosine deaminase antibodies in lupus erythematosus.	Pubmed	0.0044	65.1	0.0114	0.116	1	1	ADA
Pubmed	17592028	Estrogen protects against the development of salt-induced cardiac hypertrophy in heterozygous proANP gene-disrupted mice.	Pubmed	0.0044	65.1	0.0114	0.116	1	1	NPPA
Pubmed	20585586	Hypoxia regulates BMP4 expression in the murine spleen during the recovery from acute anemia.	GeneRIF	0.0044	65.1	0.0114	0.116	1	1	BMP4
Pubmed	17554076	Dysferlin is expressed in human placenta but does not associate with caveolin.	GeneRIF	0.0044	65.1	0.0114	0.116	1	1	DYSF
Pubmed	18798254	Genetic polymorphisms in the MMP-7 gene and breast cancer survival.	GeneRIF	0.0044	65.1	0.0114	0.116	1	1	MMP7
Pubmed	18386042	Expression pattern of serine protease inhibitor kazal type 3 (Spink3) during mouse embryonic development.	GeneRIF	0.0044	65.1	0.0114	0.116	1	1	SPINK1
Pubmed	19463	Human kidney gamma-glutamyl transpeptidase. Catalytic properties, subunit structure, and localization of the gamma-glutamyl binding site on the light subunit.	Pubmed	0.0044	65.1	0.0114	0.116	1	1	GGT1
Pubmed	429365	The structure of hemoglobin Indianapolis [beta112(G14) arginine]. An unstable variant detectable only by isotopic labeling.	Pubmed	0.0044	65.1	0.0114	0.116	1	1	HBB
Pubmed	18634834	FRET with multiply labeled HERG K(+) channels as a reporter of the in vivo coarse architecture of the cytoplasmic domains.	GeneRIF	0.0044	65.1	0.0114	0.116	1	1	KCNH2
Pubmed	21368222	Therapeutic targeting of SPINK1-positive prostate cancer.	GeneRIF	0.0044	65.1	0.0114	0.116	1	1	SPINK1
Pubmed	20336466	Age, beta thalassaemia trait, and iron-deficient anaemia significantly affect reticulocyte indices in pre-school children.	GeneRIF	0.0044	65.1	0.0114	0.116	1	1	HBB

Category	ID	Name	Source	p-value	q-value Bonferroni	q-value FDR B&H	q-value FDR B&Y	Hit Count in Query List	Hit Count in Genome	Hit in Query List
Pubmed	21830119	Transglutaminase 2 is secreted from smooth muscle cells by transamidation-dependent microparticle formation.	GeneRIF	0.0044	65.1	0.0114	0.116	1	1	TGM2
Pubmed	9561193	The aspartic proteinases. An historical overview.	Pubmed	0.0044	65.1	0.0114	0.116	1	1	NAPSA
Pubmed	17409237	The doublecortin-expressing population in the developing and adult brain contains multipotential precursors in addition to neuronal-lineage cells.	GeneRIF	0.0044	65.1	0.0114	0.116	1	1	DCLK1
Pubmed	24374318	Investigating the interactions of the 18kDa translocator protein and its ligand PK11195 in planar lipid bilayers.	Pubmed	0.0044	65.1	0.0114	0.116	1	1	TSPO
Pubmed	23222475	Muscle-specific expression of LARGE restores neuromuscular transmission deficits in dystrophic LARGE(myd) mice.	GeneRIF	0.0044	65.1	0.0114	0.116	1	1	LARGE
Pubmed	1684954	Human glandular Kallikrein genes: genetic and physical mapping of the KLK1 locus using a highly polymorphic microsatellite PCR marker.	Pubmed	0.0044	65.1	0.0114	0.116	1	1	KLK1
Pubmed	14678801	Molecular analysis of LGMD-2B and MM patients: identification of novel DYSF mutations and possible founder effect in the Italian population.	Pubmed	0.0044	65.1	0.0114	0.116	1	1	DYSF
Pubmed	16896923	Identification and characterization of a novel human dysferlin transcript: dysferlin_v1.	GeneRIF	0.0044	65.1	0.0114	0.116	1	1	DYSF
Pubmed	20508610	Prognostic value of minimal residual disease by real-time quantitative PCR in acute myeloid leukemia with CBFB-MYH11 rearrangement: the French experience.	GeneRIF	0.0044	65.1	0.0114	0.116	1	1	MYH11
Pubmed	11911961	The influence of alpha1-acid glycoprotein (orosomucoid) and its glycoforms on the function of human thyrocytes and CHO cells transfected with the human TSH receptor.	GeneRIF	0.0044	65.1	0.0114	0.116	1	1	ORM1
Pubmed	25216694	CAPN5 gene silencing by short hairpin RNA interference.	Pubmed	0.0044	65.1	0.0114	0.116	1	1	CAPN5
Pubmed	2929915	The nasocardiac reflex.	Pubmed	0.0044	65.1	0.0114	0.116	1	1	BMP4
Pubmed	17188332	Increased expression of the Nogo receptor in the hippocampus and its relation to the neuropathology in Alzheimer's disease.	GeneRIF	0.0044	65.1	0.0114	0.116	1	1	RTN4R
Pubmed	817940	The amino acid sequence of the A chain of human alpha-crystallin.	Pubmed	0.0044	65.1	0.0114	0.116	1	1	CRYAA
Pubmed	15381106	Transglutaminase 2 induces nitric oxide synthesis in BV-2 microglia.	GeneRIF	0.0044	65.1	0.0114	0.116	1	1	TGM2
Pubmed	17917825	Fractalkine: an important candidate for directing periglomerular leukocyte accumulation in irradiated mouse kidneys.	GeneRIF	0.0044	65.1	0.0114	0.116	1	1	CX3CL1
Pubmed	22706116	[p75NTR receptor--role in cell growth and apoptosis].	GeneRIF	0.0044	65.1	0.0114	0.116	1	1	NGFR
Pubmed	8703060	Pycnodysostosis, a lysosomal disease caused by cathepsin K deficiency.	Pubmed	0.0044	65.1	0.0114	0.116	1	1	CTSK
Pubmed	18195689	The aspartic protease napsin A suppresses tumor growth independent of its catalytic activity.	GeneRIF	0.0044	65.1	0.0114	0.116	1	1	NAPSA
Pubmed	11877258	Successful treatment of murine beta-thalassemia intermedia by transfer of the human beta-globin gene.	GeneRIF	0.0044	65.1	0.0114	0.116	1	1	HBB
Pubmed	22891158	The potential of GPNMB as novel neuroprotective factor in amyotrophic lateral sclerosis.	GeneRIF	0.0044	65.1	0.0114	0.116	1	1	GPNMB
Pubmed	17881306	Hb Santa Clara (beta 97His-->Asn), a human haemoglobin variant: functional characterization and structure modelling.	GeneRIF	0.0044	65.1	0.0114	0.116	1	1	HBB
Pubmed	17379283	HLA-DQB1 polymorphisms and risk for cervical cancer: a case-control study in a southern Chinese population.	GeneRIF	0.0044	65.1	0.0114	0.116	1	1	HLA-DQB1
Pubmed	16474003	Molecular determinants of HERG channel block.	GeneRIF	0.0044	65.1	0.0114	0.116	1	1	KCNH2
Pubmed	18189308	p75NTR expression in rat urinary bladder sensory neurons and spinal cord with cyclophosphamide-induced cystitis.	GeneRIF	0.0044	65.1	0.0114	0.116	1	1	NGFR
Pubmed	23755258	Identification of subunit-subunit interaction sites in aA-WT crystallin and mutant aA-G98R crystallin using isotope-labeled cross-linker and mass spectrometry.	Pubmed	0.0044	65.1	0.0114	0.116	1	1	CRYAA
Pubmed	14631123	Increase in transglutaminase 2 in idiopathic inflammatory myopathies.	GeneRIF	0.0044	65.1	0.0114	0.116	1	1	TGM2
Pubmed	21722074	Association of a functional polymorphism in the MMP7 gene promoter with susceptibility to vulnerable carotid plaque in a Han Chinese population.	GeneRIF	0.0044	65.1	0.0114	0.116	1	1	MMP7
Pubmed	20047904	Beneficial effects of secretory leukocyte protease inhibitor after spinal cord injury.	GeneRIF	0.0044	65.1	0.0114	0.116	1	1	SLPI
Pubmed	12732097	Human aldose reductase and human small intestine aldose reductase are efficient retinal reductases: consequences for retinoid metabolism.	GeneRIF	0.0044	65.1	0.0114	0.116	1	1	AKR1B10
Pubmed	14517167	Dose-dependent acceleration of high-density lipoprotein catabolism by endothelial lipase.	GeneRIF	0.0044	65.1	0.0114	0.116	1	1	LIPG
Pubmed	22005466	Ion channels in the regulation of platelet migration.	Pubmed	0.0044	65.1	0.0114	0.116	1	1	KCNN4
Pubmed	22198379	Complete transglutaminase 2 ablation results in reduced stroke volumes and astrocytes that exhibit increased survival in response to ischemia.	GeneRIF	0.0044	65.1	0.0114	0.116	1	1	TGM2
Pubmed	18725411	Functional adhesiveness of the CX3CL1 chemokine requires its aggregation. Role of the transmembrane domain.	GeneRIF	0.0044	65.1	0.0114	0.116	1	1	CX3CL1
Pubmed	7472477	Impaired peripheral nerve regeneration in a mutant strain of mice (Enr) with a Schwann cell defect.	Pubmed	0.0044	65.1	0.0114	0.116	1	1	LARGE
Pubmed	10602992	The human polycystic kidney disease 2-like (PKDL) gene: exon/intron structure and evidence for a novel splicing mechanism.	Pubmed	0.0044	65.1	0.0114	0.116	1	1	PKD2L1
Pubmed	16146777	Tissue transglutaminase (TG2)--a wound response enzyme.	GeneRIF	0.0044	65.1	0.0114	0.116	1	1	TGM2
Pubmed	24152733	Ubiquitination-dependent quality control of hERG K+ channel with acquired and inherited conformational defect at the plasma membrane.	Pubmed	0.0044	65.1	0.0114	0.116	1	1	KCNH2
Pubmed	21250885	ß+-Thalassemia trait due to a novel mutation in the ß-globin gene promoter: -26 (A>C) [HBB c.-76A>C].	GeneRIF	0.0044	65.1	0.0114	0.116	1	1	HBB

Category	ID	Name	Source	p-value	q-value Bonferroni	q-value FDR B&H	q-value FDR B&Y	Hit Count in Query List	Hit Count in Genome	Hit in Query List
Pubmed	7749372	Molecular cloning and characterization of a novel kallikrein transcript in colon and its distribution in human tissues.	Pubmed	0.0044	65.1	0.0114	0.116	1	1	KLK1
Pubmed	21824644	Elevated soluble endothelial cell protein C receptor (sEPCR) levels in women with preeclampsia: a marker of endothelial activation/damage?	GeneRIF	0.0044	65.1	0.0114	0.116	1	1	PROCR
Pubmed	12019212	A mutation in GJB3 is associated with recessive erythrokeratodermia variabilis (EKV) and leads to defective trafficking of the connexin 31 protein.	GeneRIF	0.0044	65.1	0.0114	0.116	1	1	GJB3
Pubmed	25130438	Lumisterol is metabolized by CYP11A1: discovery of a new pathway.	Pubmed	0.0044	65.1	0.0114	0.116	1	1	CYP11A1
Pubmed	2474547	Adenosine deaminase gene expression. Tissue-dependent regulation of transcriptional elongation.	Pubmed	0.0044	65.1	0.0114	0.116	1	1	ADA
Pubmed	8776585	Targeted disruption of the mouse sphingolipid activator protein gene: a complex phenotype, including severe leukodystrophy and wide-spread storage of multiple sphingolipids.	Pubmed	0.0044	65.1	0.0114	0.116	1	1	PSAP
Pubmed	12231529	Expansion of microsatellite in the thyroid hormone receptor-alpha1 gene linked to increased receptor expression and less aggressive thyroid cancer.	GeneRIF	0.0044	65.1	0.0114	0.116	1	1	THRA
Pubmed	24191930	Napsin A as a marker of clear cell ovarian carcinoma.	Pubmed	0.0044	65.1	0.0114	0.116	1	1	NAPSA
Pubmed	22508050	Comparative effects of liensinine and neferine on the human ether-a-go-go-related gene potassium channel and pharmacological activity analysis.	GeneRIF	0.0044	65.1	0.0114	0.116	1	1	KCNH2
Pubmed	18364512	Matrix metalloproteinase-7 and the 20S proteasome contribute to cellular senescence.	GeneRIF	0.0044	65.1	0.0114	0.116	1	1	MMP7
Pubmed	10213453	Nerve growth factor-induced growth of sympathetic axons into the optic tract of mature mice is enhanced by an absence of p75NTR expression.	Pubmed	0.0044	65.1	0.0114	0.116	1	1	NGFR
Pubmed	20590444	A study of three polymorphic sites of ADA gene in colon cancer.	GeneRIF	0.0044	65.1	0.0114	0.116	1	1	ADA
Pubmed	11884478	A 320-kilobase artificial chromosome encoding the human HLA DR3-DQ2 MHC haplotype confers HLA restriction in transgenic mice.	Pubmed	0.0044	65.1	0.0114	0.116	1	1	HLA-DQB1
Pubmed	8397411	Cell surface HLA-DR-invariant chain complexes are targeted to endosomes by rapid internalization.	Pubmed	0.0044	65.1	0.0114	0.116	1	1	CD74
Pubmed	15198992	Evidence for a dominant-negative effect in ACTA1 nemaline myopathy caused by abnormal folding, aggregation and altered polymerization of mutant actin isoforms.	Pubmed	0.0044	65.1	0.0114	0.116	1	1	ACTA1
Pubmed	18178159	T cell precursor frequency differentially affects CTL responses under different immune conditions.	Pubmed	0.0044	65.1	0.0114	0.116	1	1	HLA-DQB1
Pubmed	23033319	Association of the smoothelin (SMTN) gene with cerebral infarction in men: a haplotype-based case-control study.	GeneRIF	0.0044	65.1	0.0114	0.116	1	1	SMTN
Pubmed	25745166	Structural biology. Structural basis for chemokine recognition and activation of a viral G protein-coupled receptor.	Pubmed	0.0044	65.1	0.0114	0.116	1	1	CX3CL1
Pubmed	23065828	Soluble ST2 is regulated by p75 neurotrophin receptor and predicts mortality in diabetic patients with critical limb ischemia.	GeneRIF	0.0044	65.1	0.0114	0.116	1	1	NGFR
Pubmed	22943051	The spectrum of a- and ß-thalassemia mutations in Yunnan Province of Southwestern China.	GeneRIF	0.0044	65.1	0.0114	0.116	1	1	HBB
Pubmed	19726881	Extracellular K+ concentration controls cell surface density of IKr in rabbit hearts and of the HERG channel in human cell lines.	GeneRIF	0.0044	65.1	0.0114	0.116	1	1	KCNH2
Pubmed	24315351	Atrial natriuretic peptide as a predictor of atrial fibrillation in a male population study. The Study of Men Born in 1913 and 1923.	Pubmed	0.0044	65.1	0.0114	0.116	1	1	NPPA
Pubmed	16109069	Newborn HLA-DR,DQ genotype screening: age- and ethnicity-specific type 1 diabetes risk estimates.	GeneRIF	0.0044	65.1	0.0114	0.116	1	1	HLA-DQB1
Pubmed	16129698	Transgenic activation of the kallikrein-kinin system inhibits intramyocardial inflammation, endothelial dysfunction and oxidative stress in experimental diabetic cardiomyopathy.	GeneRIF	0.0044	65.1	0.0114	0.116	1	1	KLK1
Pubmed	14754884	Role of the NH2 terminus in the assembly and trafficking of the intermediate conductance Ca2+-activated K+ channel hIK1.	GeneRIF	0.0044	65.1	0.0114	0.116	1	1	KCNN4
Pubmed	7529552	Cloning and characterization of the t(X;11) breakpoint from a leukemic cell line identify a new member of the forkhead gene family.	Pubmed	0.0044	65.1	0.0114	0.116	1	1	FOXO4
Pubmed	16337236	Human osteoblasts produce cathepsin K.	GeneRIF	0.0044	65.1	0.0114	0.116	1	1	CTSK
Pubmed	20110394	Ventilatory chemoresponsiveness, narcolepsy-cataplexy and human leukocyte antigen DQB1*0602 status.	GeneRIF	0.0044	65.1	0.0114	0.116	1	1	HLA-DQB1
Pubmed	9769324	Matrilysin expression and function in airway epithelium.	Pubmed	0.0044	65.1	0.0114	0.116	1	1	MMP7
Pubmed	11843288	Triple heterozygosity of a hemoglobin variant: hemoglobin Pyrgos with other hemoglobinopathies.	GeneRIF	0.0044	65.1	0.0114	0.116	1	1	HBB
Pubmed	19926013	Genotype-phenotype aspects of type 2 long QT syndrome.	GeneRIF	0.0044	65.1	0.0114	0.116	1	1	KCNH2
Pubmed	12144061	Four new variants of the alpha2-globin gene without clinical or hematologic effects: Hb Park Ridge [alpha9(alpha7)Asn-->Lys (alpha2)], Hb Norton [alpha72(EF1)His-->Asp (alpha2)], Hb Lombard [alpha103(G10)His-->Tyr (alpha2)], and Hb San Antonio [A113(GH2)Leu-->Arg (A2)].	Pubmed	0.0044	65.1	0.0114	0.116	1	1	HBA2
Pubmed	21725047	Myeloid dysplasia and bone marrow hypocellularity in adenosine deaminase-deficient severe combined immune deficiency.	GeneRIF	0.0044	65.1	0.0114	0.116	1	1	ADA
Pubmed	19148481	Gene expression profiling reveals overexpression of TSPAN13 in prostate cancer.	GeneRIF	0.0044	65.1	0.0114	0.116	1	1	TSPAN13
Pubmed	24859602	Interferon-inducible protein 205 (p205) plays a role in adipogenic differentiation of mouse adipose-derived stem cells.	Pubmed	0.0044	65.1	0.0114	0.116	1	1	IFI16

Category	ID	Name	Source	p-value	q-value Bonferroni	q-value FDR B&H	q-value FDR B&Y	Hit Count in Query List	Hit Count in Genome	Hit in Query List
Pubmed	14985432	A pro-apoptotic fragment of the p75 neurotrophin receptor is expressed in p75NTRExonIV null mice.	GeneRIF	0.0044	65.1	0.0114	0.116	1	1	NGFR
Pubmed	11358845	Overexpression of the retinoic acid-responsive gene Stra6 in human cancers and its synergistic induction by Wnt-1 and retinoic acid.	Pubmed	0.0044	65.1	0.0114	0.116	1	1	STRA6
Pubmed	25846370	Tetranectin positive expression in tumour tissue leads to longer survival in Danish women with ovarian cancer. Results from the 'Malova' ovarian cancer study.	Pubmed	0.0044	65.1	0.0114	0.116	1	1	CLEC3B
Pubmed	10856700	A large "footprint" at the boundary of the human beta-globin locus control region hypersensitive site 2.	GeneRIF	0.0044	65.1	0.0114	0.116	1	1	HBB
Pubmed	19427062	Study of thyroid hormone receptor alpha gene polymorphisms on Alzheimer's disease.	GeneRIF	0.0044	65.1	0.0114	0.116	1	1	THRA
Pubmed	21075288	Hemoglobin gene therapy for ß-thalassemia.	GeneRIF	0.0044	65.1	0.0114	0.116	1	1	HBB
Pubmed	22237629	Antigen-specific CD4(+) T cells regulate function of myeloid-derived suppressor cells in cancer via retrograde MHC class II signaling.	Pubmed	0.0044	65.1	0.0114	0.116	1	1	HLA-DQB1
Pubmed	9777695	All or none peripheral tolerance induction in H-Y antigen-specific TCR transgenic mice.	Pubmed	0.0044	65.1	0.0114	0.116	1	1	HLA-DQB1
Pubmed	23861840	The serine protease domain of MASP-3: enzymatic properties and crystal structure in complex with ecotin.	Pubmed	0.0044	65.1	0.0114	0.116	1	1	MASP1
Pubmed	17070838	Chronic angiotensin II stimulation in the heart produces an acquired long QT syndrome associated with IK1 potassium current downregulation.	GeneRIF	0.0044	65.1	0.0114	0.116	1	1	KCNN4
Pubmed	20480728	A study of three polymorphic sites of the ADA gene in children with type 1 diabetes mellitus.	GeneRIF	0.0044	65.1	0.0114	0.116	1	1	ADA
Pubmed	9852107	Human HtrA, an evolutionarily conserved serine protease identified as a differentially expressed gene product in osteoarthritic cartilage.	Pubmed	0.0044	65.1	0.0114	0.116	1	1	HTRA1
Pubmed	18681782	The serine protease HtrA1 is a novel prognostic factor for human mesothelioma.	GeneRIF	0.0044	65.1	0.0114	0.116	1	1	HTRA1
Pubmed	17167097	Progressive deafness and altered cochlear innervation in knock-out mice lacking prosaposin.	GeneRIF	0.0044	65.1	0.0114	0.116	1	1	PSAP
Pubmed	15231497	Molecular analysis of PIP2 regulation of HERG and IKr.	GeneRIF	0.0044	65.1	0.0114	0.116	1	1	KCNH2
Pubmed	20090360	Association between serum gamma-glutamyltransferase and chronic kidney disease among US adults.	GeneRIF	0.0044	65.1	0.0114	0.116	1	1	GGT1
Pubmed	25303326	Joint testing of genotypic and gene-environment interaction identified novel association for BMP4 with non-syndromic CL/P in an Asian population using data from an International Cleft Consortium.	Pubmed	0.0044	65.1	0.0114	0.116	1	1	BMP4
Pubmed	16157452	Macrosialin increases during normal brain aging are attenuated by caloric restriction.	GeneRIF	0.0044	65.1	0.0114	0.116	1	1	CD68
Pubmed	1385301	Characterization of the human kallikrein locus.	Pubmed	0.0044	65.1	0.0114	0.116	1	1	KLK1
Pubmed	16409383	The brachymorph mouse and the developmental-genetic basis for canalization and morphological integration.	Pubmed	0.0044	65.1	0.0114	0.116	1	1	PAPSS2
Pubmed	9783701	Localisation of a gene for non-specific X linked mental retardation (MRX46) to Xq25-q26.	Pubmed	0.0044	65.1	0.0114	0.116	1	1	ARHGEF6
Pubmed	10751614	Accumulation of DNA damage in the organs of mice deficient in gamma-glutamyltranspeptidase.	Pubmed	0.0044	65.1	0.0114	0.116	1	1	GGT1
Pubmed	12126629	A structure for the trimeric MHC class II-associated invariant chain transmembrane domain.	GeneRIF	0.0044	65.1	0.0114	0.116	1	1	CD74
Pubmed	23831066	Extracellular cleavage of the p75 neurotrophin receptor is implicated in its pro-survival effect in breast cancer cells.	Pubmed	0.0044	65.1	0.0114	0.116	1	1	NGFR
Pubmed	11734895	The evolutionary history of prosaposin: two successive tandem-duplication events gave rise to the four saposin domains in vertebrates.	GeneRIF	0.0044	65.1	0.0114	0.116	1	1	PSAP
Pubmed	19386899	Converging evidence for the Nogo-66 receptor gene in schizophrenia.	GeneRIF	0.0044	65.1	0.0114	0.116	1	1	RTN4R
Pubmed	11814462	Influence of the ORM1 phenotypes on serum unbound concentration and protein binding of quinidine.	GeneRIF	0.0044	65.1	0.0114	0.116	1	1	ORM1
Pubmed	25477223	Blood-brain barrier KCa3.1 channels: evidence for a role in brain Na uptake and edema in ischemic stroke.	Pubmed	0.0044	65.1	0.0114	0.116	1	1	KCNN4
Pubmed	17605936	[Polymorphism of HLA-DQB1 alleles in Chinese Han patients with Vogt-Koyanagi-Harada syndrome].	GeneRIF	0.0044	65.1	0.0114	0.116	1	1	HLA-DQB1
Pubmed	2907498	The primary structure of human gamma-glutamyl transpeptidase.	Pubmed	0.0044	65.1	0.0114	0.116	1	1	GGT1
Pubmed	24852509	Association of endothelial lipase Thr111Ile polymorphism with proliferative retinopathy in type 2 diabetes patients.	Pubmed	0.0044	65.1	0.0114	0.116	1	1	LIPG
Pubmed	6547996	mRNA sequence for human cardiodilatin-atrial natriuretic factor precursor and regulation of precursor mRNA in rat atria.	Pubmed	0.0044	65.1	0.0114	0.116	1	1	NPPA
Pubmed	9712893	Expression cloning of human corneodesmosin proves its identity with the product of the S gene and allows improved characterization of its processing during keratinocyte differentiation.	Pubmed	0.0044	65.1	0.0114	0.116	1	1	CDSN
Pubmed	11849458	Association of the tissue kallikrein gene promoter with ESRD and hypertension.	GeneRIF	0.0044	65.1	0.0114	0.116	1	1	KLK1
Pubmed	23981618	A novel deletion-frameshift mutation in the S1 region of HERG gene in a Chinese family with long QT syndrome.	Pubmed	0.0044	65.1	0.0114	0.116	1	1	KCNH2
Pubmed	21943122	Transglutaminase 2 as an independent prognostic marker for survival of patients with non-adenocarcinoma subtype of non-small cell lung cancer.	GeneRIF	0.0044	65.1	0.0114	0.116	1	1	TGM2
Pubmed	12894224	Role of IFI 16, a member of the interferon-inducible p200-protein family, in prostate epithelial cellular senescence.	GeneRIF	0.0044	65.1	0.0114	0.116	1	1	IFI16
Pubmed	21541270	Two novel mutations of TACSTD2 found in three Japanese gelatinous drop-like corneal dystrophy families with their aberrant subcellular localization.	GeneRIF	0.0044	65.1	0.0114	0.116	1	1	TACSTD2

Category	ID	Name	Source	p-value	q-value Bonferroni	q-value FDR B&H	q-value FDR B&Y	Hit Count in Query List	Hit Count in Genome	Hit in Query List
Pubmed	23446636	Tissue-specific effects of saposin A and saposin B on glycosphingolipid degradation in mutant mice.	Pubmed	0.0044	65.1	0.0114	0.116	1	1	PSAP
Pubmed	25009214	Effect of antigen sensitization and challenge on oscillatory mechanics of the lung and pulmonary inflammation in obese carboxypeptidase E-deficient mice.	Pubmed	0.0044	65.1	0.0114	0.116	1	1	CPE
Pubmed	24607927	The gender-specific association of EHD3 polymorphisms with major depressive disorder.	Pubmed	0.0044	65.1	0.0114	0.116	1	1	EHD3
Pubmed	18648506	Splice variants of the forkhead box protein AFX exhibit dominant negative activity and inhibit AFXalpha-mediated tumor cell apoptosis.	GeneRIF	0.0044	65.1	0.0114	0.116	1	1	FOXO4
Pubmed	24158513	Globotriaosylceramide induces lysosomal degradation of endothelial KCa3.1 in fabry disease.	Pubmed	0.0044	65.1	0.0114	0.116	1	1	KCNN4
Pubmed	15609277	beta-globin gene cluster haplotypes associated with beta-thalassemia on Corsica island.	GeneRIF	0.0044	65.1	0.0114	0.116	1	1	HBB
Pubmed	9629496	An alpha-thalassemic hemoglobinopathy: homozygosity for the HB Agrinio alpha 2-globin chain variant.	Pubmed	0.0044	65.1	0.0114	0.116	1	1	HBA2
Pubmed	14676148	Sudden death associated with short-QT syndrome linked to mutations in HERG.	GeneRIF	0.0044	65.1	0.0114	0.116	1	1	KCNH2
Pubmed	21086906	Cathepsin-K is a sensitive immunohistochemical marker for detection of micro-granulomas in hypersensitivity pneumonitis.	GeneRIF	0.0044	65.1	0.0114	0.116	1	1	CTSK
Pubmed	15768558	Two new hemoglobin variants: Hb Brem-sur-Mer [beta9(A6)Ser-->Tyr] and Hb Passy [alpha81(F2)Ser-->Pro (alpha2)].	Pubmed	0.0044	65.1	0.0114	0.116	1	1	HBA2
Pubmed	19914800	Analysis of interactions between genetic variants of BMP4 and environmental factors with nonsyndromic cleft lip with or without cleft palate susceptibility.	GeneRIF	0.0044	65.1	0.0114	0.116	1	1	BMP4
Pubmed	23204795	Pharmacogenetic influence of LOC387715/HTRA1 on the efficacy of bevacizumab treatment for age-related macular degeneration in a Korean population.	Pubmed	0.0044	65.1	0.0114	0.116	1	1	HTRA1
Pubmed	25238142	Loss of Trop2 causes ErbB3 activation through a neuregulin-1-dependent mechanism in the mesenchymal subtype of HNSCC.	Pubmed	0.0044	65.1	0.0114	0.116	1	1	TACSTD2
Pubmed	21252495	New mutation in erythroid-specific delta-aminolevulinate synthase as the cause of X-linked sideroblastic anemia responsive to pyridoxine.	GeneRIF	0.0044	65.1	0.0114	0.116	1	1	ALAS2
Pubmed	6090454	Human adenosine deaminase. cDNA and complete primary amino acid sequence.	Pubmed	0.0044	65.1	0.0114	0.116	1	1	ADA
Pubmed	15373622	Frequency of HLA-DQB*06 in Caucasian,African American, and Mexican American patients with a positive direct antiglobulin test.	GeneRIF	0.0044	65.1	0.0114	0.116	1	1	HLA-DQB1
Pubmed	23101664	Microsatellite and single nucleotide polymorphisms in the ß-globin locus control region-hypersensitive Site 2: SPECIFICITY of Tunisian ßs chromosomes.	GeneRIF	0.0044	65.1	0.0114	0.116	1	1	HBB
Pubmed	16769909	Matrix metalloproteinase-7 affects connexin-43 levels, electrical conduction, and survival after myocardial infarction.	GeneRIF	0.0044	65.1	0.0114	0.116	1	1	MMP7
Pubmed	2464749	Characterization of a third human thyroid hormone receptor coexpressed with other thyroid hormone receptors in several tissues.	Pubmed	0.0044	65.1	0.0114	0.116	1	1	THRA
Pubmed	22658978	Transglutaminase 2 modulates antigen-specific antibody response by suppressing Blimp-1 and AID expression of B cells in mice.	GeneRIF	0.0044	65.1	0.0114	0.116	1	1	TGM2
Pubmed	24718052	Expression of murine 5-aminolevulinate synthase variants causes protoporphyrin IX accumulation and light-induced mammalian cell death.	Pubmed	0.0044	65.1	0.0114	0.116	1	1	ALAS2
Pubmed	21797701	Comparison between capillary electrophoresis and high performance liquid chromatography for detection and quantification of Hb constant spring [Hb CS; a142, Term¿Gln (TAA>CAA IN a2)].	GeneRIF	0.0044	65.1	0.0114	0.116	1	1	HBA2
Pubmed	20533273	[Association of single nucleotide polymorphisms of kallikrein 1 gene with cerebral hemorrhage in Changsha Han Chinese].	GeneRIF	0.0044	65.1	0.0114	0.116	1	1	KLK1
Pubmed	9627126	Immunobiological studies on experimental visceral Leishmaniasis. V. The I-A(Bm12) mutation specifies resistance to infection.	Pubmed	0.0044	65.1	0.0114	0.116	1	1	HLA-DQB1
Pubmed	12815711	Fractalkine protein localization and gene expression in mouse brain.	GeneRIF	0.0044	65.1	0.0114	0.116	1	1	CX3CL1
Pubmed	18199971	Cataract-causing alphaAG98R mutant shows substrate-dependent chaperone activity.	GeneRIF	0.0044	65.1	0.0114	0.116	1	1	CRYAA
Pubmed	20053725	A conserved lysine in the thyroid hormone recepto alpha1 DNA-binding domain, mutated in hepatocellular carcinoma, serves as a sensor for transcriptional regulation.	GeneRIF	0.0044	65.1	0.0114	0.116	1	1	THRA
Pubmed	23739531	Angiopoietin-like protein 2 is associated with chronic kidney disease in a general Japanese population: the Hisayama Study.	Pubmed	0.0044	65.1	0.0114	0.116	1	1	ANGPTL2
Pubmed	15828882	Short QT syndrome and atrial fibrillation caused by mutation in KCNH2.	GeneRIF	0.0044	65.1	0.0114	0.116	1	1	KCNH2
Pubmed	8323276	Isolation, characterization, and proteolysis of human prosaposin, the precursor of saposins (sphingolipid activator proteins).	Pubmed	0.0044	65.1	0.0114	0.116	1	1	PSAP
Pubmed	17525091	Missense mutations in pancreatic secretory trypsin inhibitor (SPINK1) cause intracellular retention and degradation.	GeneRIF	0.0044	65.1	0.0114	0.116	1	1	SPINK1
Pubmed	22493362	The relationship between secretory leukocyte protease inhibitor expression and Epstein-Barr virus status among patients with nasopharyngeal carcinoma.	GeneRIF	0.0044	65.1	0.0114	0.116	1	1	SLPI
Pubmed	15265642	Differences in spatial learning comparing transgenic p75 knockout, New Zealand Black, C57BL/6, and Swiss Webster mice.	Pubmed	0.0044	65.1	0.0114	0.116	1	1	NGFR
Pubmed	17907166	Fractalkine mediates T cell-dependent proliferation of synovial fibroblasts in rheumatoid arthritis.	GeneRIF	0.0044	65.1	0.0114	0.116	1	1	CX3CL1
Pubmed	20575011	A theoretical study on the catalytic mechanism of Mus musculus adenosine deaminase.	GeneRIF	0.0044	65.1	0.0114	0.116	1	1	ADA

Category	ID	Name	Source	p-value	q-value Bonferroni	q-value FDR B&H	q-value FDR B&Y	Hit Count in Query List	Hit Count in Genome	Hit in Query List
Pubmed	2373702	Identification of transcription stop sites at the 5' and 3' ends of the murine adenosine deaminase gene.	Pubmed	0.0044	65.1	0.0114	0.116	1	1	ADA
Pubmed	14512171	Muscle inflammation and MHC class I up-regulation in muscular dystrophy with lack of dysferlin: an immunopathological study.	GeneRIF	0.0044	65.1	0.0114	0.116	1	1	DYSF
Pubmed	21671471	B7-H3 contributes to the metastatic capacity of melanoma cells by modulation of known metastasis-associated genes.	GeneRIF	0.0044	65.1	0.0114	0.116	1	1	CD276
Pubmed	7818278	Evidence for a role of corneodesmosin, a protein which may serve to modify desmosomes during cornification, in stratum corneum cell cohesion and desquamation.	Pubmed	0.0044	65.1	0.0114	0.116	1	1	CDSN
Pubmed	19958185	Multiplex ligation-dependent probe amplification identification of 17 different beta-globin gene deletions (including four novel mutations) in the UK population.	GeneRIF	0.0044	65.1	0.0114	0.116	1	1	HBB
Pubmed	18392622	Molecular mechanism of beta-thalassaemia caused by 22-bp duplication.	GeneRIF	0.0044	65.1	0.0114	0.116	1	1	HBB
Pubmed	12769506	Exposure-disease continuum for 2-chloro-2'-deoxyadenosine, a prototype ocular teratogen. 3. Intervention with PK11195.	GeneRIF	0.0044	65.1	0.0114	0.116	1	1	TSPO
Pubmed	15777683	Bone mass effects of a BMP4 gene polymorphism in postmenopausal women.	GeneRIF	0.0044	65.1	0.0114	0.116	1	1	BMP4
Pubmed	12779274	A new hemoglobin variant, Hb Fukui [alpha139(HC1)Lys --> Asn (AAA --> AAC) (alpha2)].	Pubmed	0.0044	65.1	0.0114	0.116	1	1	HBA2
Pubmed	10570964	Organization and chromosomal localization of the human endothelial protein C receptor gene.	Pubmed	0.0044	65.1	0.0114	0.116	1	1	PROCR
Pubmed	24844923	Frequency of SPINK1 N34S mutation in acute and recurrent acute pancreatitis.	Pubmed	0.0044	65.1	0.0114	0.116	1	1	SPINK1
Pubmed	8432561	Alpha 1-acid glycoprotein expression in human leukocytes: possible correlation between alpha 1-acid glycoprotein and inflammatory cytokines in rheumatoid arthritis.	Pubmed	0.0044	65.1	0.0114	0.116	1	1	ORM1
Pubmed	23957618	Identification of the first nonsense CDSN mutation with expression of a truncated protein causing peeling skin syndrome type B.	Pubmed	0.0044	65.1	0.0114	0.116	1	1	CDSN
Pubmed	25299872	Role of adenosine deaminase and the influence of age on the diagnosis of pleural tuberculosis.	Pubmed	0.0044	65.1	0.0114	0.116	1	1	ADA
Pubmed	21512179	Association with serum gamma-glutamyltransferase levels and alcohol consumption on stroke and coronary artery disease the Suita study.	GeneRIF	0.0044	65.1	0.0114	0.116	1	1	GGT1
Pubmed	2710139	Expression of three forms of thyroid hormone receptor in human tissues.	Pubmed	0.0044	65.1	0.0114	0.116	1	1	THRA
Pubmed	16600635	Crystal structure of human pyridoxal kinase.	Pubmed	0.0044	65.1	0.0114	0.116	1	1	PDXK
Pubmed	17203517	Role of the HLA-DQ locus in the development of chronic gastritis and gastric carcinoma in Mexican patients.	GeneRIF	0.0044	65.1	0.0114	0.116	1	1	HLA-DQB1
Pubmed	23705025	Identification of antithrombin-modulating genes. Role of LARGE, a gene encoding a bifunctional glycosyltransferase, in the secretion of proteins?	Pubmed	0.0044	65.1	0.0114	0.116	1	1	LARGE
Pubmed	10818106	Identification of a novel sequence involved in lysosomal sorting of the sphingolipid activator protein prosaposin.	Pubmed	0.0044	65.1	0.0114	0.116	1	1	PSAP
Pubmed	15921164	Spectrum of beta-globin gene mutations among thalassemia patients in the West Bank region of Palestine.	GeneRIF	0.0044	65.1	0.0114	0.116	1	1	HBB
Pubmed	24794518	Homozygous deletion of six genes including corneodesmosin on chromosome 6p21.3 is associated with generalized peeling skin disease.	Pubmed	0.0044	65.1	0.0114	0.116	1	1	CDSN
Pubmed	22638758	Hb Etobicoke mutation in a hybrid HBA212 allele [HBA212 84 (F5) Ser>Arg; HBA212:c.255C>G].	GeneRIF	0.0044	65.1	0.0114	0.116	1	1	HBA2
Pubmed	8035838	Linkage of autosomal dominant hearing loss to the short arm of chromosome 1 in two families.	Pubmed	0.0044	65.1	0.0114	0.116	1	1	GJB3
Pubmed	22734501	The IVS-II-837 (T>G) appears to be a relatively common 'rare' ß-globin gene mutation in ß-thalassemia patients in Karnataka State, South India.	GeneRIF	0.0044	65.1	0.0114	0.116	1	1	HBB
Pubmed	19525461	Quantitative analysis of peripheral benzodiazepine receptor in the human brain using PET with (11)C-AC-5216.	GeneRIF	0.0044	65.1	0.0114	0.116	1	1	TSPO
Pubmed	2358682	The BM12 mutation and autoantibodies to dsDNA in NZB.H-2bm12 mice.	Pubmed	0.0044	65.1	0.0114	0.116	1	1	HLA-DQB1
Pubmed	22005198	FoxO4 inhibits atherosclerosis through its function in bone marrow derived cells.	GeneRIF	0.0044	65.1	0.0114	0.116	1	1	FOXO4
Pubmed	22943743	a-thalassemia trait caused by frameshift mutations in exon 2 of the a2-globin gene: HBA2:c.131delT and HBA2:c.143delA.	GeneRIF	0.0044	65.1	0.0114	0.116	1	1	HBA2
Pubmed	22988836	BMP4 can generate primordial germ cells from bone-marrow-derived pluripotent stem cells.	GeneRIF	0.0044	65.1	0.0114	0.116	1	1	BMP4
Pubmed	24438169	Crystal structures of the human Dysferlin inner DysF domain.	Pubmed	0.0044	65.1	0.0114	0.116	1	1	DYSF
Pubmed	18036564	Intrinsic selectivity in binding of matrix metalloproteinase-7 to differently charged lipid membranes.	GeneRIF	0.0044	65.1	0.0114	0.116	1	1	MMP7
Pubmed	21654857	Association between a thyroid hormone receptor-a gene polymorphism and blood pressure but not with coronary heart disease risk.	GeneRIF	0.0044	65.1	0.0114	0.116	1	1	THRA
Pubmed	10577279	A novel mutation of the erythroid-specific gamma-Aminolevulinate synthase gene in a patient with non-inherited pyridoxine-responsive sideroblastic anemia.	Pubmed	0.0044	65.1	0.0114	0.116	1	1	ALAS2
Pubmed	8942668	Site-directed mutagenesis of histidine 238 in mouse adenosine deaminase: substitution of histidine 238 does not impede hydroxylate formation.	Pubmed	0.0044	65.1	0.0114	0.116	1	1	ADA
Pubmed	19027781	Human ether-a-go-go related gene (hERG) K+ channels: function and dysfunction.	GeneRIF	0.0044	65.1	0.0114	0.116	1	1	KCNH2
Pubmed	25018622	Comparison of effect of gamma ray irradiation on wild-type and N-terminal mutants of aA-crystallin.	Pubmed	0.0044	65.1	0.0114	0.116	1	1	CRYAA
Pubmed	16564506	Progressive hearing loss in mice carrying a mutation in the p75 gene.	GeneRIF	0.0044	65.1	0.0114	0.116	1	1	NGFR

Category	ID	Name	Source	p-value	q-value Bonferroni	q-value FDR B&H	q-value FDR B&Y	Hit Count in Query List	Hit Count in Genome	Hit in Query List
Pubmed	11791870	Hb Dartmouth [alpha66(E15)Leu-->Pro (alpha2)(CTG-->CCG)]: a novel alpha2-globin gene mutation associated with severe neonatal anemia when inherited in trans with Southeast Asian alpha-thalassemia-1.	Pubmed	0.0044	65.1	0.0114	0.116	1	1	HBA2
Pubmed	22079192	Intrathecally synthesized IgG in multiple sclerosis cerebrospinal fluid recognizes identical epitopes over time.	Pubmed	0.0044	65.1	0.0114	0.116	1	1	SLC39A2
Pubmed	18606701	Fractalkine and CX3CR1 mediate leukocyte capture by endothelium in response to Shiga toxin.	GeneRIF	0.0044	65.1	0.0114	0.116	1	1	CX3CL1
Pubmed	18828016	Effect of invariant chain on major histocompatibility complex class I molecule expression and stability on human breast tumor cell lines.	GeneRIF	0.0044	65.1	0.0114	0.116	1	1	CD74
Pubmed	15147902	ADP-ribosylation factor (ARF)-like 7 (ARL7) is induced by cholesterol loading and participates in apolipoprotein AI-dependent cholesterol export.	Pubmed	0.0044	65.1	0.0114	0.116	1	1	ARL4C
Pubmed	22908695	Association of elevated a1-acid glycoprotein (AGP) and the prevalence of anemia in Nicaraguan preschool children.	GeneRIF	0.0044	65.1	0.0114	0.116	1	1	ORM1
Pubmed	21226238	Role of transglutaminase 2 in apoptosis induced by hydrogen peroxide in human chondrocytes.	GeneRIF	0.0044	65.1	0.0114	0.116	1	1	TGM2
Pubmed	24006262	The chemokine CX3CL1 promotes trafficking of dendritic cells through inflamed lymphatics.	Pubmed	0.0044	65.1	0.0114	0.116	1	1	CX3CL1
Pubmed	23017645	The contribution of the SPINK1 c.194+2T>C mutation to the clinical course of idiopathic chronic pancreatitis in Chinese patients.	GeneRIF	0.0044	65.1	0.0114	0.116	1	1	SPINK1
Pubmed	2898948	Structure and chromosomal localization of the human renal kallikrein gene.	Pubmed	0.0044	65.1	0.0114	0.116	1	1	KLK1
Pubmed	24527386	Wnt secretion from epithelial cells and subepithelial myofibroblasts is not required in the mouse intestinal stem cell niche in vivo.	Pubmed	0.0044	65.1	0.0114	0.116	1	1	PORCN
Pubmed	10072583	Assignment of the human skeletal muscle [FC12]a actin gene (ACTA1) to chromosome 1q42.13-->q42.2 by radiation hybrid mapping.	Pubmed	0.0044	65.1	0.0114	0.116	1	1	ACTA1
Pubmed	1814856	Hb Coimbra or alpha 2 beta (2)99(G1)Asp----Glu, a newly discovered highoxygen affinity variant.	Pubmed	0.0044	65.1	0.0114	0.116	1	1	HBB
Pubmed	20488756	Coeliac disease autoantibodies mediate significant inhibition of tissue transglutaminase.	GeneRIF	0.0044	65.1	0.0114	0.116	1	1	TGM2
Pubmed	11762979	Tissue transglutaminase expression in HIV-infected cells: an enzyme with an antiviral effect?	Pubmed	0.0044	65.1	0.0114	0.116	1	1	TGM2
Pubmed	24220659	An N-terminal pro-atrial natriuretic peptide (NT-proANP) 'aggregation-prone' segment involved in isolated atrial amyloidosis.	Pubmed	0.0044	65.1	0.0114	0.116	1	1	NPPA
Pubmed	22923476	C-peptide prevents hyperglycemia-induced endothelial apoptosis through inhibition of reactive oxygen species-mediated transglutaminase 2 activation.	GeneRIF	0.0044	65.1	0.0114	0.116	1	1	TGM2
Pubmed	18795907	Severe psychomotor and metabolic damages caused by a mutant thyroid hormone receptor alpha 1 in mice: can patients with a similar mutation be found and treated?	GeneRIF	0.0044	65.1	0.0114	0.116	1	1	THRA
Pubmed	8024008	ANG II receptor blockade prevents ventricular hypertrophy and ANF gene expression with pressure overload in mice.	Pubmed	0.0044	65.1	0.0114	0.116	1	1	NPPA
Pubmed	24993941	Simultaneous rather than ordered cleavage of two sites within the BMP4 prodomain leads to loss of ligand in mice.	Pubmed	0.0044	65.1	0.0114	0.116	1	1	BMP4
Pubmed	17392380	Expression and function of COOH-terminal myosin heavy chain isoforms in mouse smooth muscle.	Pubmed	0.0044	65.1	0.0114	0.116	1	1	MYH11
Pubmed	12225953	Gamma-glutamyl transferase deficiency results in lung oxidant stress in normoxia.	GeneRIF	0.0044	65.1	0.0114	0.116	1	1	GGT1
Pubmed	12884003	Relationship between serum HDL-C levels and common genetic variants of the endothelial lipase gene in Japanese school-aged children.	GeneRIF	0.0044	65.1	0.0114	0.116	1	1	LIPG
Pubmed	15993670	Expression of mouse HtrA1 serine protease in normal bone and cartilage and its upregulation in joint cartilage damaged by experimental arthritis.	GeneRIF	0.0044	65.1	0.0114	0.116	1	1	HTRA1
Pubmed	12144532	Erythrocytes as targets for gamma-glutamyltranspeptidase initiated pro-oxidant reaction.	Pubmed	0.0044	65.1	0.0114	0.116	1	1	GGT1
Pubmed	20473923	KLK15 is a prognostic marker for progression-free survival in patients with radical prostatectomy.	GeneRIF	0.0044	65.1	0.0114	0.116	1	1	KLK15
Pubmed	1155453	Hemoglobin Koya Dora: high frequency of a chain termination mutant.	Pubmed	0.0044	65.1	0.0114	0.116	1	1	HBA2
Pubmed	19557432	A predicted functional single-nucleotide polymorphism of bone morphogenetic protein-4 gene affects mRNA expression and shows a significant association with cutaneous melanoma in Southern Italian population.	GeneRIF	0.0044	65.1	0.0114	0.116	1	1	BMP4
Pubmed	15528018	Serine protease inhibitor Kazal type 1 mutations and pancreatitis.	Pubmed	0.0044	65.1	0.0114	0.116	1	1	SPINK1
Pubmed	21042280	P-Rex1 participates in Neuregulin-ErbB signal transduction and its expression correlates with patient outcome in breast cancer.	GeneRIF	0.0044	65.1	0.0114	0.116	1	1	PREX1
Pubmed	12614764	A novel mutation of M1S1 gene found in a Vietnamese patient with gelatinous droplike corneal dystrophy.	GeneRIF	0.0044	65.1	0.0114	0.116	1	1	TACSTD2
Pubmed	12532447	The analysis of gamma-glutamyl transpeptidase gene in different type liver tissues.	GeneRIF	0.0044	65.1	0.0114	0.116	1	1	GGT1
Pubmed	10848509	Increased central AT(1)-receptor activation, not systemic vasopressin, sustains hypertension in ANP knockout mice.	Pubmed	0.0044	65.1	0.0114	0.116	1	1	NPPA
Pubmed	17482290	Elevated renal norepinephrine in proANP gene-disrupted mice is associated with increased tyrosine hydroxylase expression in sympathetic ganglia.	GeneRIF	0.0044	65.1	0.0114	0.116	1	1	NPPA
Pubmed	18987455	Association between serum gamma-glutamyl transferase level and prehypertension among community-dwelling men.	GeneRIF	0.0044	65.1	0.0114	0.116	1	1	GGT1

Category	ID	Name	Source	p-value	q-value Bonferroni	q-value FDR B&H	q-value FDR B&Y	Hit Count in Query List	Hit Count in Genome	Hit in Query List
Pubmed	24921172	Successful long-term management of iris flocculi and miosis in a patient with a strong family history of thoracic aortic aneurysms and dissections associated with an MYH11 mutation.	Pubmed	0.0044	65.1	0.0114	0.116	1	1	MYH11
Pubmed	20301480	Dysferlinopathy	Pubmed	0.0044	65.1	0.0114	0.116	1	1	DYSF
Pubmed	8635248	Structure and characterization of the 5'-flanking region of the mouse smooth muscle myosin heavy chain (SM1/2) gene.	Pubmed	0.0044	65.1	0.0114	0.116	1	1	MYH11
Pubmed	8195006	Hb Footscray or alpha 133(H16) Ser-->Arg: a new hemoglobin variant.	Pubmed	0.0044	65.1	0.0114	0.116	1	1	HBA2
Pubmed	23529168	Cathepsin K knockout alleviates pressure overload-induced cardiac hypertrophy.	GeneRIF	0.0044	65.1	0.0114	0.116	1	1	CTSK
Pubmed	15496208	Characterization of a novel HLA-DQB1 allele, DQB1*020102.	Pubmed	0.0044	65.1	0.0114	0.116	1	1	HLA-DQB1
Pubmed	12966029	Mutations in the human LARGE gene cause MDC1D, a novel form of congenital muscular dystrophy with severe mental retardation and abnormal glycosylation of alpha-dystroglycan.	Pubmed	0.0044	65.1	0.0114	0.116	1	1	LARGE
Pubmed	17560885	A novel mutation in human ether-a-go-go-related gene, alanine to proline at position 490, found in a large family with autosomal dominant long QT syndrome.	GeneRIF	0.0044	65.1	0.0114	0.116	1	1	KCNH2
Pubmed	19958198	Complex interaction of Hb E [beta26(B8)Glu-->Lys], Hb Korle-Bu [beta73(E17)Asp-->Asn] and a deletional alpha-thalassemia-1 in pregnancy.	GeneRIF	0.0044	65.1	0.0114	0.116	1	1	HBB
Pubmed	21679467	Renal kallikrein excretion and epigenetics in human acute kidney injury: expression, mechanisms and consequences.	GeneRIF	0.0044	65.1	0.0114	0.116	1	1	KLK1
Pubmed	9190932	Intermediate TCR cells in mouse lung: their effector function to induce pneumonitis in mice with autoimmune-like graft-versus-host disease.	Pubmed	0.0044	65.1	0.0114	0.116	1	1	HLA-DQB1
Pubmed	21743496	Anti-angiogenic and tumor-suppressive roles of candidate tumor-suppressor gene, Fibulin-2, in nasopharyngeal carcinoma.	GeneRIF	0.0044	65.1	0.0114	0.116	1	1	FBLN2
Pubmed	9407042	A novel gene, hKCa4, encodes the calcium-activated potassium channel in human T lymphocytes.	Pubmed	0.0044	65.1	0.0114	0.116	1	1	KCNN4
Pubmed	24814875	Targeting mitochondrial 18 kDa translocator protein (TSPO) regulates macrophage cholesterol efflux and lipid phenotype.	Pubmed	0.0044	65.1	0.0114	0.116	1	1	TSPO
Pubmed	17576861	Nonsense mutations in hERG cause a decrease in mutant mRNA transcripts by nonsense-mediated mRNA decay in human long-QT syndrome.	GeneRIF	0.0044	65.1	0.0114	0.116	1	1	KCNH2
Pubmed	12560090	Relevance of the proximal domain in the amino-terminus of HERG channels for regulation by a phospholipase C-coupled hormone receptor.	GeneRIF	0.0044	65.1	0.0114	0.116	1	1	KCNH2
Pubmed	23249638	Influence of platelet ¿-glutamyltransferase on oxidative stress and apoptosis in the presence of holo-transferrin.	GeneRIF	0.0044	65.1	0.0114	0.116	1	1	GGT1
Pubmed	18845279	Bone density, strength, and formation in adult cathepsin K (-/-) mice.	GeneRIF	0.0044	65.1	0.0114	0.116	1	1	CTSK
Pubmed	20857305	Functional significance of the intermediate conductance Ca2+-activated K+ channel for the short-term survival of injured erythrocytes.	GeneRIF	0.0044	65.1	0.0114	0.116	1	1	KCNN4
Pubmed	23368878	Hb Plasencia [a125(H8)Leu¿Arg (a2)] is a frequent cause of a+-thalassemia in the Portuguese population.	GeneRIF	0.0044	65.1	0.0114	0.116	1	1	HBA2
Pubmed	16506891	Evaluation of the rubidium efflux assay for preclinical identification of HERG blockade.	GeneRIF	0.0044	65.1	0.0114	0.116	1	1	KCNH2
Pubmed	10682309	A non-glycosylated and functionally deficient mutant (N215H) of the sphingolipid activator protein B (SAP-B) in a novel case of metachromatic leukodystrophy (MLD).	Pubmed	0.0044	65.1	0.0114	0.116	1	1	PSAP
Pubmed	12655507	No further loss of dorsal root ganglion cells after axotomy in p75 neurotrophin receptor knockout mice.	GeneRIF	0.0044	65.1	0.0114	0.116	1	1	NGFR
Pubmed	24838345	Genetic and epigenetic determinants of low dysferlin expression in monocytes.	Pubmed	0.0044	65.1	0.0114	0.116	1	1	DYSF
Pubmed	11931653	Transcriptional regulation of human 3'-phosphoadenosine 5'-phosphosulphate synthase 2.	GeneRIF	0.0044	65.1	0.0114	0.116	1	1	PAPSS2
Pubmed	18357489	Differential diagnosis of tuberculous and malignant pleural effusions: what is the role of adenosine deaminase?	GeneRIF	0.0044	65.1	0.0114	0.116	1	1	ADA
Pubmed	10505613	Improved survival of injured sciatic nerve Schwann cells in mice lacking the p75 receptor.	Pubmed	0.0044	65.1	0.0114	0.116	1	1	NGFR
Pubmed	15060158	The human beta-globin replication initiation region consists of two modular independent replicators.	GeneRIF	0.0044	65.1	0.0114	0.116	1	1	HBB
Pubmed	12394345	Characterization of beta-globin haplotypes using blood spots from a population-based cohort of newborns with homozygous HbS.	GeneRIF	0.0044	65.1	0.0114	0.116	1	1	HBB
Pubmed	22987366	Potential therapeutic target and independent prognostic marker of TROP2 in laryngeal squamous cell carcinoma.	Pubmed	0.0044	65.1	0.0114	0.116	1	1	TACSTD2
Pubmed	17986713	Protective effect of an endothelial lipase gene variant on coronary artery disease in a Chinese population.	GeneRIF	0.0044	65.1	0.0114	0.116	1	1	LIPG
Pubmed	11891331	Deletion of the thyroid hormone receptor alpha 1 prevents the structural alterations of the cerebellum induced by hypothyroidism.	GeneRIF	0.0044	65.1	0.0114	0.116	1	1	THRA
Pubmed	19411665	The T111I variant in the endothelial lipase gene and risk of coronary heart disease in three independent populations.	GeneRIF	0.0044	65.1	0.0114	0.116	1	1	LIPG
Pubmed	16113566	Plasma atrial natriuretic peptide in fetuses with cardiac disease.	GeneRIF	0.0044	65.1	0.0114	0.116	1	1	NPPA
Pubmed	21907015	Homozygous mutations in PXDN cause congenital cataract, corneal opacity, and developmental glaucoma.	GeneRIF	0.0044	65.1	0.0114	0.116	1	1	PXDN
Pubmed	12609609	PCP4 is highly expressed in ectoderm and particularly in neuroectoderm derivatives during mouse embryogenesis.	Pubmed	0.0044	65.1	0.0114	0.116	1	1	PCP4
Pubmed	20080610	Inhibition of the K+ channel KCa3.1 ameliorates T cell-mediated colitis.	GeneRIF	0.0044	65.1	0.0114	0.116	1	1	KCNN4

Category	ID	Name	Source	p-value	q-value Bonferroni	q-value FDR B&H	q-value FDR B&Y	Hit Count in Query List	Hit Count in Genome	Hit in Query List
Pubmed	12859424	Pressure-independent enhancement of cardiac hypertrophy in atrial natriuretic peptide-deficient mice.	Pubmed	0.0044	65.1	0.0114	0.116	1	1	NPPA
Pubmed	21621584	Importance of pH and disulfide bridges on the structural and binding properties of human a1-acid glycoprotein.	GeneRIF	0.0044	65.1	0.0114	0.116	1	1	ORM1
Pubmed	17516066	A novel mutation of -73(A-->T) in the CCAAT box of the beta-globin gene identified in a patient with the mild beta-thalassemia intermedia.	GeneRIF	0.0044	65.1	0.0114	0.116	1	1	HBB
Pubmed	24338389	Novel association analysis between HLA-DQB1 polymorphisms and rectal cancer based on a cross-validation design.	Pubmed	0.0044	65.1	0.0114	0.116	1	1	HLA-DQB1
Pubmed	16227340	Specific serine proteases selectively damage KCNH2 (hERG1) potassium channels and I(Kr).	Pubmed	0.0044	65.1	0.0114	0.116	1	1	KCNH2
Pubmed	23028699	Cysteine dioxygenase 1 is a tumor suppressor gene silenced by promoter methylation in multiple human cancers.	GeneRIF	0.0044	65.1	0.0114	0.116	1	1	CDO1
Pubmed	8954791	The gene organization, chromosome location, and expression of a 55-kDa matrix protein (PRELP) of human articular cartilage.	Pubmed	0.0044	65.1	0.0114	0.116	1	1	PRELP
Pubmed	8786106	The human L35a ribosomal protein (RPL35A) gene is located at chromosome band 3q29-qter.	Pubmed	0.0044	65.1	0.0114	0.116	1	1	RPL35A
Pubmed	19324319	Aminoglycoside antibiotics restore functional expression of truncated HERG channels produced by nonsense mutations.	GeneRIF	0.0044	65.1	0.0114	0.116	1	1	KCNH2
Pubmed	22347476	Mutations in human aA-crystallin/sHSP affect subunit exchange interaction with aB-crystallin.	GeneRIF	0.0044	65.1	0.0114	0.116	1	1	CRYAA
Pubmed	15149287	Human leukocyte antigen-DQB1 alleles are not associated with schizophrenia in Kuwaiti Arabs.	GeneRIF	0.0044	65.1	0.0114	0.116	1	1	HLA-DQB1
Pubmed	19654412	Mndal, a new interferon-inducible family member, is highly polymorphic, suppresses cell growth, and may modify plasmacytoma susceptibility.	GeneRIF	0.0044	65.1	0.0114	0.116	1	1	IFI16
Pubmed	20306731	Adenosine deaminase activity in subjects with normal pregnancy, pregnancy induced hypertension and pre-eclampsia.	GeneRIF	0.0044	65.1	0.0114	0.116	1	1	ADA
Pubmed	12831462	Coagulation and inflammation.	Pubmed	0.0044	65.1	0.0114	0.116	1	1	PROCR
Pubmed	10799472	Secretory leukocyte protease inhibitor inhibits infection of monocytes and lymphocytes with human immunodeficiency virus type 1 but does no interfere with transcytosis of cell-associated virus across tight epithelial barriers.	Pubmed	0.0044	65.1	0.0114	0.116	1	1	SLPI
Pubmed	12354792	Skeletal, cardiac and tongue muscle pathology, defective retinal transmission, and neuronal migration defects in the Large(myd) mouse defines a natural model for glycosylation-deficient muscle eye - brain disorders.	Pubmed	0.0044	65.1	0.0114	0.116	1	1	LARGE
Pubmed	15921688	EPCR Ser219Gly: elevated sEPCR, prothrombin F1+2, risk for coronary heart disease, and increased sEPCR shedding in vitro.	GeneRIF	0.0044	65.1	0.0114	0.116	1	1	PROCR
Pubmed	19674475	A mutation in CTSK gene in an autosomal recessive pycnodysostosis family of Pakistani origin.	GeneRIF	0.0044	65.1	0.0114	0.116	1	1	CTSK
Pubmed	24610445	Transglutaminase 2 inhibitor abrogates renal cell carcinoma in xenograft models.	Pubmed	0.0044	65.1	0.0114	0.116	1	1	TGM2
Pubmed	11486027	Structural and functional analysis of an mRNP complex that mediates the high stability of human beta-globin mRNA.	Pubmed	0.0044	65.1	0.0114	0.116	1	1	HBB
Pubmed	22437349	Evaluation and prognostic significance of human tissue kallikrein-related peptidase 10 (KLK10) in colorectal cancer.	GeneRIF	0.0044	65.1	0.0114	0.116	1	1	KLK10
Pubmed	21658164	Abnormal expression of dysferlin in skeletal muscle and monocytes supports primary dysferlinopathy in patients with one mutated allele.	GeneRIF	0.0044	65.1	0.0114	0.116	1	1	DYSF
Pubmed	24692541	Regulation of the mitochondrial permeability transition pore by the outer membrane does not involve the peripheral benzodiazepine receptor (Translocator Protein of 18 kDa (TSPO)).	Pubmed	0.0044	65.1	0.0114	0.116	1	1	TSPO
Pubmed	7730378	Structural analysis of saposin C and B. Complete localization of disulfide bridges.	Pubmed	0.0044	65.1	0.0114	0.116	1	1	PSAP
Pubmed	9197538	Analysis of an expression profile of genes in the human adipose tissue.	Pubmed	0.0044	65.1	0.0114	0.116	1	1	CPE
Pubmed	18327081	Genetic and dietary control of plasma tissue kallikrein secretion and urinary kinins exretion in man.	GeneRIF	0.0044	65.1	0.0114	0.116	1	1	KLK1
Pubmed	2550050	Pump-1 cDNA codes for a protein with characteristics similar to those of classical collagenase family members.	Pubmed	0.0044	65.1	0.0114	0.116	1	1	MMP7
Pubmed	24965595	Further observations on associations between the ADA gene and past malaria morbidity in Sardinia.	Pubmed	0.0044	65.1	0.0114	0.116	1	1	ADA
Pubmed	18414673	Pathways to injury in chronic pancreatitis: decoding the role of the high-risk SPINK1 N34S haplotype using meta-analysis.	GeneRIF	0.0044	65.1	0.0114	0.116	1	1	SPINK1
Pubmed	22751291	The N34S mutation of the SPINK1 gene and alcoholic chronic pancreatitis.	GeneRIF	0.0044	65.1	0.0114	0.116	1	1	SPINK1
Pubmed	1541632	Interferon action: nucleolar and nucleoplasmic localization of the interferon-inducible 72-kD protein that is encoded by the Ifi 204 gene from the gene 200 cluster.	Pubmed	0.0044	65.1	0.0114	0.116	1	1	IFI16
Pubmed	24847890	Hypomethylation of the MMP7 promoter and increased expression of MMP7 distinguishes the basal-like breast cancer subtype from other triple-negative tumors.	Pubmed	0.0044	65.1	0.0114	0.116	1	1	MMP7
Pubmed	17275179	Human tissue kallikreins: the cancer biomarker family.	Pubmed	0.0044	65.1	0.0114	0.116	1	1	KLK7
Pubmed	18535205	Abnormalities of the large ribosomal subunit protein, Rpl35a, in Diamond-Blackfan anemia.	GeneRIF	0.0044	65.1	0.0114	0.116	1	1	RPL35A
Pubmed	17397971	[Adenosine deaminase is useful for the diagnosis of peritoneal tuberculosis in patients with end-stage renal failure].	GeneRIF	0.0044	65.1	0.0114	0.116	1	1	ADA
Pubmed	22920719	Polymorphisms in seizure 6-like gene are associated with bipolar disorder I: evidence of gene × gender interaction.	Pubmed	0.0044	65.1	0.0114	0.116	1	1	SEZ6L

Category	ID	Name	Source	p-value	q-value Bonferroni	q-value FDR B&H	q-value FDR B&Y	Hit Count in Query List	Hit Count in Genome	Hit in Query List
Pubmed	12405608	Hypocretin (orexin) levels in cerebrospinal fluid of patients with narcolepsy: relationship to cataplexy and HLA DQB1*0602 status.	GeneRIF	0.0044	65.1	0.0114	0.116	1	1	HLA-DQB1
Pubmed	23019195	BMP4 is a novel paracrine inhibitor of liver regeneration.	GeneRIF	0.0044	65.1	0.0114	0.116	1	1	BMP4
Pubmed	1431214	Mapping of the trichohyalin gene: co-localization with the profilaggrin, involucrin, and loricrin genes.	Pubmed	0.0044	65.1	0.0114	0.116	1	1	TCHH
Pubmed	22418245	Napsin A is an independent prognostic factor in surgically resected adenocarcinoma of the lung.	GeneRIF	0.0044	65.1	0.0114	0.116	1	1	NAPSA
Pubmed	25387045	Oxidative uncoupling in cysteine dioxygenase is gated by a proton-sensitive intermediate.	Pubmed	0.0044	65.1	0.0114	0.116	1	1	CDO1
Pubmed	9396465	Human protein C receptor is present primarily on endothelium of large blood vessels: implications for the control of the protein C pathway.	Pubmed	0.0044	65.1	0.0114	0.116	1	1	PROCR
Pubmed	11872662	Genetic risk determines the emergence of diabetes-associated autoantibodies in young children.	GeneRIF	0.0044	65.1	0.0114	0.116	1	1	HLA-DQB1
Pubmed	6256358	Mouse actin messenger RNAs. Construction and characterization of a recombinant plasmid molecule containing a complementary DNA transcript of mouse alpha-actin mRNA.	Pubmed	0.0044	65.1	0.0114	0.116	1	1	ACTA1
Pubmed	24928497	Increased fracture callus mineralization and strength in cathepsin K knockout mice.	Pubmed	0.0044	65.1	0.0114	0.116	1	1	CTSK
Pubmed	24846539	The autolysis of human HtrA1 is governed by the redox state of its N-terminal domain.	Pubmed	0.0044	65.1	0.0114	0.116	1	1	HTRA1
Pubmed	18073349	Alternative mRNA is favored by the A3 haplotype of the EPCR gene PROCR and generates a novel soluble form of EPCR in plasma.	GeneRIF	0.0044	65.1	0.0114	0.116	1	1	PROCR
Pubmed	23935018	Identification of a novel erythroid-specific enhancer for the ALAS2 gene and its loss-of-function mutation which is associated with congenital sideroblastic anemia.	Pubmed	0.0044	65.1	0.0114	0.116	1	1	ALAS2
Pubmed	1939222	Biosynthesis of delta-aminolevulinic acid and the regulation of heme formation by immature erythroid cells in man.	Pubmed	0.0044	65.1	0.0114	0.116	1	1	ALAS2
Pubmed	22326583	Severe vestibular dysfunction and altered vestibular innervation in mice lacking prosaposin.	GeneRIF	0.0044	65.1	0.0114	0.116	1	1	PSAP
Pubmed	22109994	Depletion of the p43 mitochondrial T3 receptor in mice affects skeletal muscle development and activity.	Pubmed	0.0044	65.1	0.0114	0.116	1	1	THRA
Pubmed	16077902	Intracellular distribution, assembly and effect of disease-associated connexin 31 mutants in HeLa cells.	GeneRIF	0.0044	65.1	0.0114	0.116	1	1	GJB3
Pubmed	1051893	Nursing in transition.	Pubmed	0.0044	65.1	0.0114	0.116	1	1	PROCR
Pubmed	25412720	Transfusion-dependent thalassemia in Northern Sarawak: a molecular study to identify different genotypes in the multi-ethnic groups and the importance of genomic sequencing in unstudied populations.	Pubmed	0.0044	65.1	0.0114	0.116	1	1	HBB
Pubmed	1518371	The physiological consequences of glutathione variations.	Pubmed	0.0044	65.1	0.0114	0.116	1	1	GSS
Pubmed	24653034	Structure of the mitochondrial translocator protein in complex with a diagnostic ligand.	Pubmed	0.0044	65.1	0.0114	0.116	1	1	TSPO
Pubmed	24954318	Increased serum cathepsin K in patients with coronary artery disease.	Pubmed	0.0044	65.1	0.0114	0.116	1	1	CTSK
Pubmed	19046820	Epigenetic regulation and gene expression of vasopressin and atrial natriuretic peptide in alcohol withdrawal.	GeneRIF	0.0044	65.1	0.0114	0.116	1	1	NPPA
Pubmed	21220118	Molecular insights into ¿d T cell costimulation by an anti-JAML antibody.	GeneRIF	0.0044	65.1	0.0114	0.116	1	1	AMICA1
Pubmed	24768817	Elucidation of the specific function of the conserved threonine triad responsible for human L-asparaginase autocleavage and substrate hydrolysis.	Pubmed	0.0044	65.1	0.0114	0.116	1	1	ASPG
Pubmed	9380773	Melatonin high-affinity binding to alpha-1-acid glycoprotein in human serum.	Pubmed	0.0044	65.1	0.0114	0.116	1	1	ORM1
Pubmed	22705794	CTCF-mediated transcriptional regulation through cell type-specific chromosome organization in the ß-globin locus.	GeneRIF	0.0044	65.1	0.0114	0.116	1	1	HBB
Pubmed	15504325	Nogo-66 receptor prevents raphespinal and rubrospinal axon regeneration and limits functional recovery from spinal cord injury.	Pubmed	0.0044	65.1	0.0114	0.116	1	1	RTN4R
Pubmed	12510003	Expression, purification, crystallization, and preliminary X-ray analysis of recombinant human saposin B.	Pubmed	0.0044	65.1	0.0114	0.116	1	1	PSAP
Pubmed	17698709	Phenotypic study in 40 patients with dysferlin gene mutations: high frequency of atypical phenotypes.	GeneRIF	0.0044	65.1	0.0114	0.116	1	1	DYSF
Pubmed	20490473	Characterization of the rapidly activating delayed rectifier potassium current, I (Kr), in HL-1 mouse atrial myocytes.	GeneRIF	0.0044	65.1	0.0114	0.116	1	1	KCNH2
Pubmed	20484222	Saposin C mutations in Gaucher disease patients resulting in lysosomal lipid accumulation, saposin C deficiency, but normal prosaposin processing and sorting.	GeneRIF	0.0044	65.1	0.0114	0.116	1	1	PSAP
Pubmed	19918800	Novel localization of low affinity NGF receptor (p75) in the stroma of prostate cancer and possible implication in neoplastic invasion: an immunohistochemical and ultracytochemical study.	GeneRIF	0.0044	65.1	0.0114	0.116	1	1	NGFR
Pubmed	8676084	HLA-DR4-IE chimeric class II transgenic, murine class II-deficient mice are susceptible to experimental allergic encephalomyelitis.	Pubmed	0.0044	65.1	0.0114	0.116	1	1	HLA-DQB1
Pubmed	15948210	Scanning the beta-globin gene for mutations in large populations by denaturing capillary and gel electrophoresis.	GeneRIF	0.0044	65.1	0.0114	0.116	1	1	HBB
Pubmed	15297163	The serine protease stratum corneum chymotryptic enzyme (kallikrein 7) is highly overexpressed in squamous cervical cancer cells.	GeneRIF	0.0044	65.1	0.0114	0.116	1	1	KLK7
Pubmed	21391350	[(TTTTA), polymorphism in the promoter of the CYP11A1 gene in the pathogenesis of polycystic ovary syndrome].	GeneRIF	0.0044	65.1	0.0114	0.116	1	1	CYP11A1

Category	ID	Name	Source	p-value	q-value Bonferroni	q-value FDR B&H	q-value FDR B&Y	Hit Count in Query List	Hit Count in Genome	Hit in Query List
Pubmed	17728005	Overexpression of matrix metalloproteinase-7 (MMP-7) correlates with tumor proliferation, and a poor prognosis in non-small cell lung cancer.	GeneRIF	0.0044	65.1	0.0114	0.116	1	1	MMP7
Pubmed	23710940	HLA class II allele polymorphism in an outbreak of chikungunya fever in Middle Andaman, India.	Pubmed	0.0044	65.1	0.0114	0.116	1	1	HLA-DQB1
Pubmed	23831272	A model for intracellular actin waves explored by nonlinear local perturbation analysis.	Pubmed	0.0044	65.1	0.0114	0.116	1	1	ACTA1
Pubmed	10477574	A novel apoptotic pathway in quiescent lymphocytes identified by inhibition of a post-proline cleaving aminodipeptidase: a candidate target protease, quiescent cell proline dipeptidase.	Pubmed	0.0044	65.1	0.0114	0.116	1	1	DPP7
Pubmed	18196575	The p75 NTR neurotrophin receptor is a tumor suppressor in human and murine retinoblastoma development.	GeneRIF	0.0044	65.1	0.0114	0.116	1	1	NGFR
Pubmed	6407114	Murine I-A beta chain polymorphism: nucleotide sequences of three allelic I-A beta genes.	Pubmed	0.0044	65.1	0.0114	0.116	1	1	HLA-DQB1
Pubmed	12125807	Pycnodysostosis: role and regulation of cathepsin K in osteoclast function and human disease.	GeneRIF	0.0044	65.1	0.0114	0.116	1	1	CTSK
Pubmed	23300742	CD271¿ subpopulation of pancreatic stellate cells correlates with prognosis of pancreatic cancer and is regulated by interaction with cancer cells.	GeneRIF	0.0044	65.1	0.0114	0.116	1	1	NGFR
Pubmed	20880200	CX3CL1 expression induced by Candida albicans in oral fibroblasts.	GeneRIF	0.0044	65.1	0.0114	0.116	1	1	CX3CL1
Pubmed	12581867	Tissue kallikrein is synthesized and secreted by human vascular endothelial cells.	GeneRIF	0.0044	65.1	0.0114	0.116	1	1	KLK1
Pubmed	6526272	Cloning of human adenosine deaminase cDNA and expression in mouse cells.	Pubmed	0.0044	65.1	0.0114	0.116	1	1	ADA
Pubmed	12970338	The novel murine Ca2+-binding protein, Scarf, is differentially expressed during epidermal differentiation.	Pubmed	0.0044	65.1	0.0114	0.116	1	1	CALML5
Pubmed	2962900	Localized expression of the atrial natriuretic factor gene during cardiac embryogenesis.	Pubmed	0.0044	65.1	0.0114	0.116	1	1	NPPA
Pubmed	20444940	Transient expression of interferon-inducible p204 in the early stage is required for adipogenesis in 3T3-L1 cells.	GeneRIF	0.0044	65.1	0.0114	0.116	1	1	IFI16
Pubmed	24403586	The mitochondrial translocator protein, TSPO, inhibits HIV-1 envelope glycoprotein biosynthesis via the endoplasmic reticulum-associated protein degradation pathway.	Pubmed	0.0044	65.1	0.0114	0.116	1	1	TSPO
Pubmed	23571109	Epigenome-wide ovarian cancer analysis identifies a methylation profile differentiating clear-cell histology with epigenetic silencing of the HERG K+ channel.	Pubmed	0.0044	65.1	0.0114	0.116	1	1	KCNH2
Pubmed	17287450	Symptomatic dysferlin gene mutation carriers: characterization of two cases.	GeneRIF	0.0044	65.1	0.0114	0.116	1	1	DYSF
Pubmed	17189614	Bone morphogenetic protein-4 abrogates lumen formation by mammary epithelial cells and promotes invasive growth.	GeneRIF	0.0044	65.1	0.0114	0.116	1	1	BMP4
Pubmed	2981770	The gene encoding the Ia-associated invariant chain is located on chromosome 18 in the mouse.	Pubmed	0.0044	65.1	0.0114	0.116	1	1	CD74
Pubmed	2409529	Structure of the human alpha 1-acid glycoprotein gene: sequence homology with other human acute phase protein genes.	Pubmed	0.0044	65.1	0.0114	0.116	1	1	ORM1
Pubmed	11723075	Diabetes-associated HLA-DQ genes and birth weight.	GeneRIF	0.0044	65.1	0.0114	0.116	1	1	HLA-DQB1
Pubmed	24304973	Follicular lymphoma-protective HLA class II variants correlate with increased HLA-DQB1 protein expression.	Pubmed	0.0044	65.1	0.0114	0.116	1	1	HLA-DQB1
Pubmed	23010577	A novel mutation in the transmembrane nonpore region of the KCNH2 gene causes severe clinical manifestations of long QT syndrome.	GeneRIF	0.0044	65.1	0.0114	0.116	1	1	KCNH2
Pubmed	22298777	Characterization of heparin-binding site of tissue transglutaminase: its importance in cell surface targeting, matrix deposition, and cell signaling.	GeneRIF	0.0044	65.1	0.0114	0.116	1	1	TGM2
Pubmed	17296316	Silencing invariant chain of DCs enhances Th1 response using small interfering RNA.	GeneRIF	0.0044	65.1	0.0114	0.116	1	1	CD74
Pubmed	9180259	Induction of glutathione synthetase by 1,10-phenanthroline.	Pubmed	0.0044	65.1	0.0114	0.116	1	1	GSS
Pubmed	17105964	Detection of aberrant association of DM with MHC class II subunits in the absence of invariant chain.	Pubmed	0.0044	65.1	0.0114	0.116	1	1	CD74
Pubmed	5794113	Hemoglobin L Ferrara in a Jewish family associated with a hemolytic state in the propositus.	Pubmed	0.0044	65.1	0.0114	0.116	1	1	HBA2
Pubmed	20711995	The roles of BMP and IL-3 signaling pathways in the control of hematopoietic stem cells in the mouse embryo.	GeneRIF	0.0044	65.1	0.0114	0.116	1	1	BMP4
Pubmed	15837528	1,25-dihydroxyvitamin D inhibits human ANP gene promoter activity.	GeneRIF	0.0044	65.1	0.0114	0.116	1	1	NPPA
Pubmed	11722794	Haploinsufficient Bmp4 ocular phenotypes include anterior segment dysgenesis with elevated intraocular pressure.	GeneRIF	0.0044	65.1	0.0114	0.116	1	1	BMP4
Pubmed	20033322	TGM2 is a novel marker for prognosis and therapeutic target in colorectal cancer.	GeneRIF	0.0044	65.1	0.0114	0.116	1	1	TGM2
Pubmed	17331981	Two endoplasmic reticulum-associated degradation (ERAD) systems for the novel variant of the mutant dysferlin: ubiquitin/proteasome ERAD(I) and autophagy/lysosome ERAD(II).	GeneRIF	0.0044	65.1	0.0114	0.116	1	1	DYSF
Pubmed	24920668	Unstable reaction intermediates and hysteresis during the catalytic cycle of 5-aminolevulinate synthase: implications from using pseudo and alternate substrates and a promiscuous enzyme variant.	Pubmed	0.0044	65.1	0.0114	0.116	1	1	ALAS2
Pubmed	16956593	Association between the functional polymorphism in the matrix metalloproteinase-7 promoter and susceptibility to adult astrocytoma.	GeneRIF	0.0044	65.1	0.0114	0.116	1	1	MMP7
Pubmed	21633537	Specific HLA-DQB1 alleles associated with risk for development of hepatocellular carcinoma: a meta-analysis.	GeneRIF	0.0044	65.1	0.0114	0.116	1	1	HLA-DQB1

Category	ID	Name	Source	p-value	q-value Bonferroni	q-value FDR B&H	q-value FDR B&Y	Hit Count in Query List	Hit Count in Genome	Hit in Query List
Pubmed	7338469	A new unstable hemoglobin, Hb Yokohama beta 31 (B13)Leu substituting for Pro, causing hemolytic anemia.	Pubmed	0.0044	65.1	0.0114	0.116	1	1	HBB
Pubmed	19346882	Spontaneous mutation of hemoglobin Lufkin in a white boy.	GeneRIF	0.0044	65.1	0.0114	0.116	1	1	HBB
Pubmed	11583587	Identification of a novel integral plasma membrane protein induced during adipocyte differentiation.	Pubmed	0.0044	65.1	0.0114	0.116	1	1	APMAP
Pubmed	19490382	Genetic polymorphism of KCNH2 confers predisposition of acquired atrial fibrillation in Chinese.	GeneRIF	0.0044	65.1	0.0114	0.116	1	1	KCNH2
Pubmed	21196314	TNF-alpha mediated NF-kappaB activation is constantly extended by transglutaminase 2.	GeneRIF	0.0044	65.1	0.0114	0.116	1	1	TGM2
Pubmed	12021266	Defective human Ether-à-go-go-related gene trafficking linked to an endoplasmic reticulum retention signal in the C terminus.	GeneRIF	0.0044	65.1	0.0114	0.116	1	1	KCNH2
Pubmed	23390935	Detection of Hb Constant Spring [a142, Term¿Gln TAA>CAA (a2)] in heterozygotes combined with ß-thalassemia.	GeneRIF	0.0044	65.1	0.0114	0.116	1	1	HBA2
Pubmed	11698419	Recipient MHC class II expression is required to achieve long-term survival of murine cardiac allografts after costimulatory blockade.	Pubmed	0.0044	65.1	0.0114	0.116	1	1	HLA-DQB1
Pubmed	10233760	Dermatopontin expression is decreased in hypertrophic scar and systemic sclerosis skin fibroblasts and is regulated by transforming growth factor-beta1, interleukin-4, and matrix collagen.	Pubmed	0.0044	65.1	0.0114	0.116	1	1	DPT
Pubmed	19929428	Exclusion of mutations in the dysferlin alternative exons 1 of DYSF-v1, 5a, and 40a in a cohort of 26 patients.	GeneRIF	0.0044	65.1	0.0114	0.116	1	1	DYSF
Pubmed	8415725	Identification in the HLA class I region of a gene expressed late in keratinocyte differentiation.	Pubmed	0.0044	65.1	0.0114	0.116	1	1	CDSN
Pubmed	19287144	Copy number variations in chronic pancreatitis.	Pubmed	0.0044	65.1	0.0114	0.116	1	1	SPINK1
Pubmed	7524679	Human cysteine dioxygenase type I: primary structure derived from base sequencing of cDNA.	Pubmed	0.0044	65.1	0.0114	0.116	1	1	CDO1
Pubmed	19862354	Synergistic effects of metal ion and the pre-senile cataract-causing G98R alphaA-crystallin: self-aggregation propensities and chaperone activity.	GeneRIF	0.0044	65.1	0.0114	0.116	1	1	CRYAA
Pubmed	17238043	Differential roles of the SPINK1 gene mutations in alcoholic and nonalcoholic chronic pancreatitis.	GeneRIF	0.0044	65.1	0.0114	0.116	1	1	SPINK1
Pubmed	18052980	A potential role for calcium / calmodulin-dependent protein kinase-related peptide in neuronal apoptosis: in vivo and in vitro evidence.	Pubmed	0.0044	65.1	0.0114	0.116	1	1	DCLK1
Pubmed	7959781	Assignment of a human melanoma associated gene MG50 (D2S448) to chromosome 2p25.3 by fluorescence in situ hybridization.	Pubmed	0.0044	65.1	0.0114	0.116	1	1	PXDN
Pubmed	2894075	A newly characterized HLA DQ beta allele associated with pemphigus vulgaris.	Pubmed	0.0044	65.1	0.0114	0.116	1	1	HLA-DQB1
Pubmed	19146799	A review of the role of atrial natriuretic peptide gene polymorphisms in hypertension and its sequelae.	GeneRIF	0.0044	65.1	0.0114	0.116	1	1	NPPA
Pubmed	25453044	In vivo quantification of cerebral translocator protein binding in humans using 6-chloro-2-(4'-123I-iodophenyl)-3-(N,N-diethyl)-imidazo[1,2-a]pyridine-3-acetamide SPECT.	Pubmed	0.0044	65.1	0.0114	0.116	1	1	TSPO
Pubmed	17095717	Gamma glutamyl transferase and metabolic syndrome, cardiovascular disease, and mortality risk: the Framingham Heart Study.	GeneRIF	0.0044	65.1	0.0114	0.116	1	1	GGT1
Pubmed	7173395	Structural study of hemoglobin Knossos, beta 27 (B9) Ala leads to Ser. A new abnormal hemoglobin present as a silent beta-thalassemia.	Pubmed	0.0044	65.1	0.0114	0.116	1	1	HBB
Pubmed	9766636	The IFN-inducible nucleoprotein IFI 16 is expressed in cells of the monocyte lineage, but is rapidly and markedly down-regulated in other myeloid precursor populations.	Pubmed	0.0044	65.1	0.0114	0.116	1	1	IFI16
Pubmed	9446639	Deficient heme and globin synthesis in embryonic stem cells lacking the erythroid-specific delta-aminolevulinate synthase gene.	Pubmed	0.0044	65.1	0.0114	0.116	1	1	ALAS2
Pubmed	24696729	Cathepsin K in the immunohistochemical diagnosis of melanocytic lesions.	Pubmed	0.0044	65.1	0.0114	0.116	1	1	CTSK
Pubmed	18948633	Role of junctional adhesion molecule-like protein in mediating monocyte transendothelial migration.	Pubmed	0.0044	65.1	0.0114	0.116	1	1	AMICA1
Pubmed	11111119	Premarital screening of beta-thalassemia trait in the province of Denizli, Turkey.	GeneRIF	0.0044	65.1	0.0114	0.116	1	1	HBB
Pubmed	11940314	[The value of gamma-glutamyl transpeptidase mRNA typing in monitoring carcinogensis of heptocytes].	GeneRIF	0.0044	65.1	0.0114	0.116	1	1	GGT1
Pubmed	9175734	A novel mechanism generating short deletion/insertions following slippage is suggested by a mutation in the human alpha2-globin gene.	Pubmed	0.0044	65.1	0.0114	0.116	1	1	HBA2
Pubmed	2513289	Hb Hinsdale [beta 139 (H17)Asn----Lys]: a variant in the central cavity showing reduced affinity for oxygen and 2,3-diphosphoglycerate.	Pubmed	0.0044	65.1	0.0114	0.116	1	1	HBB
Pubmed	6410508	Nucleotide sequence of a light chain gene of the mouse I-A subregion: A beta d.	Pubmed	0.0044	65.1	0.0114	0.116	1	1	HLA-DQB1
Pubmed	21228320	Dual-color quantum dot detection of a heterotetrameric potassium channel (hKCa3.1).	GeneRIF	0.0044	65.1	0.0114	0.116	1	1	KCNN4
Pubmed	20660661	Modification of hERG1 channel gating by Cd2+.	GeneRIF	0.0044	65.1	0.0114	0.116	1	1	KCNH2
Pubmed	6256275	Niemann-Pick disease, Type C: evidence for the deficiency of an activating factor stimulating sphingomyelin and glucocerebroside degradation.	Pubmed	0.0044	65.1	0.0114	0.116	1	1	PSAP
Pubmed	8995352	The human delta1261 mutation of the HERG potassium channel results in a truncated protein that contains a subunit interaction domain and decreases the channel expression.	Pubmed	0.0044	65.1	0.0114	0.116	1	1	KCNH2
Pubmed	12387450	Abnormal accumulation of tTGase products in muscle and erythrocytes of chorea-acanthocytosis patients.	GeneRIF	0.0044	65.1	0.0114	0.116	1	1	TGM2
Pubmed	6327293	Structure of the murine Ia-associated invariant (Ii) chain as deduced from a cDNA clone.	Pubmed	0.0044	65.1	0.0114	0.116	1	1	CD74

Category	ID	Name	Source	p-value	q-value Bonferroni	q-value FDR B&H	q-value FDR B&Y	Hit Count in Query List	Hit Count in Genome	Hit in Query List
Pubmed	12393610	Aberrant iron accumulation and oxidized status of erythroid-specific delta-aminolevulinate synthase (ALAS2)-deficient definitive erythroblasts.	GeneRIF	0.0044	65.1	0.0114	0.116	1	1	ALAS2
Pubmed	24917237	The involvement of the HLA-DQB1 alleles in the risk and the severity of Iranian coeliac disease patients.	Pubmed	0.0044	65.1	0.0114	0.116	1	1	HLA-DQB1
Pubmed	19819973	Neurogenin 3-specific dipeptidyl peptidase-2 deficiency causes impaired glucose tolerance, insulin resistance, and visceral obesity.	GeneRIF	0.0044	65.1	0.0114	0.116	1	1	DPP7
Pubmed	18055214	Palmitoylation of the C-terminal fragment of p75(NTR) regulates death signaling and is required for subsequent cleavage by gamma-secretase.	GeneRIF	0.0044	65.1	0.0114	0.116	1	1	NGFR
Pubmed	23268354	The small SLC43 family: facilitator system l amino acid transporters and the orphan EEG1.	Pubmed	0.0044	65.1	0.0114	0.116	1	1	SLC43A2
Pubmed	3036998	Four Ia invariant chain forms derive from a single gene by alternate splicing and alternate initiation of transcription/translation.	Pubmed	0.0044	65.1	0.0114	0.116	1	1	CD74
Pubmed	25260157	The expression of NPPA splice variants during mouse cardiac development.	Pubmed	0.0044	65.1	0.0114	0.116	1	1	NPPA
Pubmed	18386051	The G604S-hERG mutation alters the biophysical properties and exerts a dominant-negative effect on expression of hERG channels in HEK293 cells.	GeneRIF	0.0044	65.1	0.0114	0.116	1	1	KCNH2
Pubmed	23227248	Serum ¿-glutamyl transferase levels, insulin resistance and liver fibrosis in patients with chronic liver diseases.	GeneRIF	0.0044	65.1	0.0114	0.116	1	1	GGT1
Pubmed	7947320	Mice homozygous for a null mutation of activin beta B are viable and fertile.	Pubmed	0.0044	65.1	0.0114	0.116	1	1	INHBB
Pubmed	21796630	Dermatopontin: a potential predictor for metastasis of human oral cancer.	GeneRIF	0.0044	65.1	0.0114	0.116	1	1	DPT
Pubmed	17206463	Progressive retinal dystrophy in two sisters with glutathione synthetase (GS) deficiency.	GeneRIF	0.0044	65.1	0.0114	0.116	1	1	GSS
Pubmed	19564340	MASP-1, a promiscuous complement protease: structure of its catalytic region reveals the basis of its broad specificity.	GeneRIF	0.0044	65.1	0.0114	0.116	1	1	MASP1
Pubmed	12764037	Doublecortin functions at the extremities of growing neuronal processes.	Pubmed	0.0044	65.1	0.0114	0.116	1	1	DCLK1
Pubmed	16716198	Transgenic rescue of erythroid 5-aminolevulinate synthase-deficient mice results in the formation of ring sideroblasts and siderocytes.	Pubmed	0.0044	65.1	0.0114	0.116	1	1	ALAS2
Pubmed	12732717	Secretory leukocyte protease inhibitor promotes the tumorigenic and metastatic potential of cancer cells.	GeneRIF	0.0044	65.1	0.0114	0.116	1	1	SLPI
Pubmed	17129617	Behavioral inhibition and impaired spatial learning and memory in hypothyroid mice lacking thyroid hormone receptor alpha.	Pubmed	0.0044	65.1	0.0114	0.116	1	1	THRA
Pubmed	22613764	Mechanisms underlying the protein-kinase mediated regulation of the HERG potassium channel synthesis.	GeneRIF	0.0044	65.1	0.0114	0.116	1	1	KCNH2
Pubmed	3009181	Purification and characterization of a novel, oligomeric, plasminogen kringle 4 binding protein from human plasma: tetranectin.	Pubmed	0.0044	65.1	0.0114	0.116	1	1	CLEC3B
Pubmed	21219849	Characterization of histone H3K27 modifications in the ß-globin locus.	GeneRIF	0.0044	65.1	0.0114	0.116	1	1	HBB
Pubmed	14576048	A haplotype of the EPCR gene is associated with increased plasma levels of sEPCR and is a candidate risk factor for thrombosis.	GeneRIF	0.0044	65.1	0.0114	0.116	1	1	PROCR
Pubmed	11198284	Distal anterior compartment myopathy: a dysferlin mutation causing a new muscular dystrophy phenotype.	Pubmed	0.0044	65.1	0.0114	0.116	1	1	DYSF
Pubmed	22981931	Diagnostic power of the mid-regional pro-atrial natriuretic peptide for heart failure patients with dyspnea: a meta-analysis.	GeneRIF	0.0044	65.1	0.0114	0.116	1	1	NPPA
Pubmed	23722541	Histone demethylase RBP2 promotes lung tumorigenesis and cancer metastasis.	Pubmed	0.0044	65.1	0.0114	0.116	1	1	KDM5A
Pubmed	19525468	Quantification of translocator protein (18 kDa) in the human brain with PET and a novel radioligand, (18)F-PBR06.	GeneRIF	0.0044	65.1	0.0114	0.116	1	1	TSPO
Pubmed	18815587	Impaired recovery of dysferlin-null skeletal muscle after contraction-induced injury in vivo.	GeneRIF	0.0044	65.1	0.0114	0.116	1	1	DYSF
Pubmed	10377177	Short ragweed allergen induces eosinophilic lung disease in HLA-DQ transgenic mice.	Pubmed	0.0044	65.1	0.0114	0.116	1	1	HLA-DQB1
Pubmed	16322094	Characterization of a novel thyroid hormone receptor alpha variant involved in the regulation of myoblast differentiation.	GeneRIF	0.0044	65.1	0.0114	0.116	1	1	THRA
Pubmed	21836138	Identification of SPOCK2 as a susceptibility gene for bronchopulmonary dysplasia.	GeneRIF	0.0044	65.1	0.0114	0.116	1	1	SPOCK2
Pubmed	21135701	Pharmacological activation of Kv11.1 in transgenic long QT-1 rabbits.	GeneRIF	0.0044	65.1	0.0114	0.116	1	1	KCNH2
Pubmed	18776039	Physiological properties of hERG 1a/1b heteromeric currents and a hERG 1b-specific mutation associated with Long-QT syndrome.	GeneRIF	0.0044	65.1	0.0114	0.116	1	1	KCNH2
Pubmed	21625439	Cleavage of kininogen and subsequent bradykinin release by the complement component: mannose-binding lectin-associated serine protease (MASP)-1.	GeneRIF	0.0044	65.1	0.0114	0.116	1	1	MASP1
Pubmed	24352382	Small bowel transglutaminase 2-specific IgA deposits in dermatitis herpetiformis.	Pubmed	0.0044	65.1	0.0114	0.116	1	1	TGM2
Pubmed	22903127	Neuronal overexpression of Nogo receptor 1 in APPswe/PSEN1(¿E9) mice impairs spatial cognition tasks without influencing plaque formation.	GeneRIF	0.0044	65.1	0.0114	0.116	1	1	RTN4R
Pubmed	9499372	[Structure and chromosomal localization of human neurogranin gene].	Pubmed	0.0044	65.1	0.0114	0.116	1	1	NRGN
Pubmed	24291518	Developmental molecular and functional cerebellar alterations induced by PCP4/PEP19 overexpression: implications for Down syndrome.	Pubmed	0.0044	65.1	0.0114	0.116	1	1	PCP4
Pubmed	9113392	Smoothelin expression characteristics: development of a smooth muscle cell in vitro system and identification of a vascular variant.	Pubmed	0.0044	65.1	0.0114	0.116	1	1	SMTN
Pubmed	2897347	Molecular analysis of DQ beta 3.1 genes.	Pubmed	0.0044	65.1	0.0114	0.116	1	1	HLA-DQB1
Pubmed	6490612	Initiation codon mutation as a cause of alpha thalassemia.	Pubmed	0.0044	65.1	0.0114	0.116	1	1	HBA2
Pubmed	20544292	Clinical significance of human kallikrein7 gene expression in colorectal cancer.	GeneRIF	0.0044	65.1	0.0114	0.116	1	1	KLK7

Category	ID	Name	Source	p-value	q-value Bonferroni	q-value FDR B&H	q-value FDR B&Y	Hit Count in Query List	Hit Count in Genome	Hit in Query List
Pubmed	18326752	Expression of ZnT and ZIP zinc transporters in the human RPE and their regulation by neurotrophic factors.	Pubmed	0.0044	65.1	0.0114	0.116	1	1	SLC39A2
Pubmed	23462328	Variation in the ¿-glutamyltransferase 1 gene and risk of chronic pancreatitis.	Pubmed	0.0044	65.1	0.0114	0.116	1	1	GGT1
Pubmed	22042851	Natural killer (NK)-dendritic cell interactions generate MHC class II-dressed NK cells that regulate CD4+ T cells.	Pubmed	0.0044	65.1	0.0114	0.116	1	1	HLA-DQB1
Pubmed	20045866	Pitavastatin decreases the expression of endothelial lipase both in vitro and in vivo.	GeneRIF	0.0044	65.1	0.0114	0.116	1	1	LIPG
Pubmed	18452160	Intracellular localization of the tumor suppressor HtrA1/Prss11 and its association with HPV16 E6 and E7 proteins.	GeneRIF	0.0044	65.1	0.0114	0.116	1	1	HTRA1
Pubmed	7935490	Thyroid hormone receptor-alpha inhibits retinoic acid-responsive gene expression and modulates retinoic acid-stimulated neural differentiation in mouse embryonic stem cells.	Pubmed	0.0044	65.1	0.0114	0.116	1	1	THRA
Pubmed	15809719	Involvement of matrix metalloproteinase-7 in invasion-metastasis through induction of cell dissociation in pancreatic cancer.	GeneRIF	0.0044	65.1	0.0114	0.116	1	1	MMP7
Pubmed	9434796	How subtle differences in MHC class II affect the severity of experimental myasthenia gravis.	Pubmed	0.0044	65.1	0.0114	0.116	1	1	HLA-DQB1
Pubmed	23850810	The neuroinflammation marker translocator protein is not elevated in individuals with mild-to-moderate depression: a [¹¹C]PBR28 PET study.	Pubmed	0.0044	65.1	0.0114	0.116	1	1	TSPO
Pubmed	12589760	Crystal structure of MHC class II I-Ab in complex with a human CLIP peptide: prediction of an I-Ab peptide-binding motif.	GeneRIF	0.0044	65.1	0.0114	0.116	1	1	HLA-DQB1
Pubmed	16244363	Long QT syndrome-associated I593R mutation in HERG potassium channel activates ER stress pathways.	GeneRIF	0.0044	65.1	0.0114	0.116	1	1	KCNH2
Pubmed	21937640	Hepatitis C virus-induced cancer stem cell-like signatures in cell culture and murine tumor xenografts.	GeneRIF	0.0044	65.1	0.0114	0.116	1	1	DCLK1
Pubmed	20516044	KLK1 gene polymorphisms are not associated with lupus nephritis in a Chinese Han population.	GeneRIF	0.0044	65.1	0.0114	0.116	1	1	KLK1
Pubmed	14988284	Geographical variation in risk HLA-DQB1 genotypes for type 1 diabetes and signs of beta-cell autoimmunity in a high-incidence country.	GeneRIF	0.0044	65.1	0.0114	0.116	1	1	HLA-DQB1
Pubmed	23407879	Voltage dependence of the Ca(2+)-activated K(+) channel K(Ca)3.1 in human erythroleukemia cells.	GeneRIF	0.0044	65.1	0.0114	0.116	1	1	KCNN4
Pubmed	20137368	Atrial natriuretic peptide gene polymorphism is not associated with hypertrophic cardiomyopathy.	GeneRIF	0.0044	65.1	0.0114	0.116	1	1	NPPA
Pubmed	12569179	Fractalkine (FRK) levels in amniotic fluid and its production during pregnancy.	GeneRIF	0.0044	65.1	0.0114	0.116	1	1	CX3CL1
Pubmed	9610724	Human Trop-2 is a tumor-associated calcium signal transducer.	Pubmed	0.0044	65.1	0.0114	0.116	1	1	TACSTD2
Pubmed	9804553	Transformation of tooth type induced by inhibition of BMP signaling.	Pubmed	0.0044	65.1	0.0114	0.116	1	1	BMP4
Pubmed	22614344	Lack of association of the G22A polymorphism of the ADA gene in patients with ankylosing spondylitis.	GeneRIF	0.0044	65.1	0.0114	0.116	1	1	ADA
Pubmed	15572050	Novel C-terminus frameshift mutation, 1122fs/147, of HERG in LQT2: additional amino acids generated by frameshift cause accelerated inactivation.	GeneRIF	0.0044	65.1	0.0114	0.116	1	1	KCNH2
Pubmed	18087047	Structural basis for the high all-trans-retinaldehyde reductase activity of the tumor marker AKR1B10.	GeneRIF	0.0044	65.1	0.0114	0.116	1	1	AKR1B10
Pubmed	23600400	Synaptic dysfunction in human immunodeficiency virus type-1-positive subjects: inflammation or impaired neuronal plasticity?	GeneRIF	0.0044	65.1	0.0114	0.116	1	1	NGFR
Pubmed	22956240	Translocator protein 18 kDa (TSPO) expression in multiple sclerosis patients.	GeneRIF	0.0044	65.1	0.0114	0.116	1	1	TSPO
Pubmed	12603094	Hb Prato [alpha31(B12)Arg --> Ser (A2)] and alpha-thalassemia in a Taiwanese.	Pubmed	0.0044	65.1	0.0114	0.116	1	1	HBA2
Pubmed	16800737	Kallikrein-related peptidase 14 may be a major contributor to trypsin-like proteolytic activity in human stratum corneum.	GeneRIF	0.0044	65.1	0.0114	0.116	1	1	KLK14
Pubmed	24903600	Pericellular proteolysis by matrix metalloproteinase-7 is differentially modulated by cholesterol sulfate, sulfatide, and cardiolipin.	Pubmed	0.0044	65.1	0.0114	0.116	1	1	MMP7
Pubmed	16294209	Matrix changes induced by transglutaminase 2 lead to inhibition of angiogenesis and tumor growth.	Pubmed	0.0044	65.1	0.0114	0.116	1	1	TGM2
Pubmed	9857182	Low-affinity nerve-growth factor receptor (P75NTR) can serve as a receptor for rabies virus.	Pubmed	0.0044	65.1	0.0114	0.116	1	1	NGFR
Pubmed	16407273	Phosphorylation of histones by tissue transglutaminase.	GeneRIF	0.0044	65.1	0.0114	0.116	1	1	TGM2
Pubmed	22269113	The carboxyl-terminal region of erythroid-specific 5-aminolevulinate synthase acts as an intrinsic modifier for its catalytic activity and protein stability.	GeneRIF	0.0044	65.1	0.0114	0.116	1	1	ALAS2
Pubmed	22222635	Overexpression and oncogenic function of aldo-keto reductase family 1B10 (AKR1B10) in pancreatic carcinoma.	GeneRIF	0.0044	65.1	0.0114	0.116	1	1	AKR1B10
Pubmed	16893387	Analysis of HLA-DQB1 gene polymorphisms in asymptomatic HBV carriers and chronic hepatitis B patients in the Chinese Han population.	GeneRIF	0.0044	65.1	0.0114	0.116	1	1	HLA-DQB1
Pubmed	19362342	Knockdown of p21-activated kinase 6 inhibits prostate cancer growth and enhances chemosensitivity to docetaxel.	Pubmed	0.0044	65.1	0.0114	0.116	1	1	PAK6
Pubmed	24519927	Microtubule-mediated defects in junctophilin-2 trafficking contribute to myocyte transverse-tubule remodeling and Ca2+ handling dysfunction in heart failure.	Pubmed	0.0044	65.1	0.0114	0.116	1	1	JPH2
Pubmed	22517356	Proton block of the pore underlies the inhibition of hERG cardiac K+ channels during acidosis.	GeneRIF	0.0044	65.1	0.0114	0.116	1	1	KCNH2
Pubmed	20060709	Trop-2 overexpression as an independent marker for poor overall survival in ovarian carcinoma patients.	GeneRIF	0.0044	65.1	0.0114	0.116	1	1	TACSTD2
Pubmed	23982886	Lipopolysaccharide increases the incidence of collagen-induced arthritis in mice through induction of protease HTRA-1 expression.	Pubmed	0.0044	65.1	0.0114	0.116	1	1	HTRA1

Category	ID	Name	Source	p-value	q-value Bonferroni	q-value FDR B&H	q-value FDR B&Y	Hit Count in Query List	Hit Count in Genome	Hit in Query List
Pubmed	7919352	Hereditary overexpression of adenosine deaminase in erythrocytes: studies in erythroid cell lines and transgenic mice.	Pubmed	0.0044	65.1	0.0114	0.116	1	1	ADA
Pubmed	15780970	Selenium binding to human hemoglobin via selenotrisulfide.	Pubmed	0.0044	65.1	0.0114	0.116	1	1	HBB
Pubmed	15866044	Transgenic inhibition of Nogo-66 receptor function allows axonal sprouting and improved locomotion after spinal injury.	Pubmed	0.0044	65.1	0.0114	0.116	1	1	RTN4R
Pubmed	23594188	Role of the K(Ca)3.1 K+ channel in auricular lymph node CD4+ T-lymphocyte function of the delayed-type hypersensitivity model.	Pubmed	0.0044	65.1	0.0114	0.116	1	1	KCNN4
Pubmed	17284525	DC-HIL is a negative regulator of T lymphocyte activation.	GeneRIF	0.0044	65.1	0.0114	0.116	1	1	GPNMB
Pubmed	19020808	Role of cysteine residues in the enhancement of chaperone function in methylglyoxal-modified human alpha A-crystallin.	GeneRIF	0.0044	65.1	0.0114	0.116	1	1	CRYAA
Pubmed	14691584	Expression analysis of the human kallikrein 7 (KLK7) in breast tumors: a new potential biomarker for prognosis of breast carcinoma.	GeneRIF	0.0044	65.1	0.0114	0.116	1	1	KLK7
Pubmed	12456652	Normal timing of oligodendrocyte development depends on thyroid hormone receptor alpha 1 (TRalpha1).	Pubmed	0.0044	65.1	0.0114	0.116	1	1	THRA
Pubmed	11939514	Hb Canterbury [beta112(G14)Cys-->Phe]: a new, mildly unstable variant.	Pubmed	0.0044	65.1	0.0114	0.116	1	1	HBB
Pubmed	23243261	Clinical features and a mutation with late onset of limb girdle muscular dystrophy 2B.	GeneRIF	0.0044	65.1	0.0114	0.116	1	1	DYSF
Pubmed	8929222	[Cloning cDNA for human neurogranin].	Pubmed	0.0044	65.1	0.0114	0.116	1	1	NRGN
Pubmed	20037138	Effects of FoxO4 overexpression on cholesterol biosynthesis, triacylglycerol accumulation, and glucose uptake.	GeneRIF	0.0044	65.1	0.0114	0.116	1	1	FOXO4
Pubmed	24227782	Selective inhibition of KCa3.1 channels mediates adenosine regulation of the motility of human T cells.	Pubmed	0.0044	65.1	0.0114	0.116	1	1	KCNN4
Pubmed	21172654	Identification of the Rac-GEF P-Rex1 as an essential mediator of ErbB signaling in breast cancer.	GeneRIF	0.0044	65.1	0.0114	0.116	1	1	PREX1
Pubmed	20048070	Matrix metalloproteinase-7 and premalignant host responses in Helicobacter pylori-infected mice.	GeneRIF	0.0044	65.1	0.0114	0.116	1	1	MMP7
Pubmed	19553472	The low-affinity receptor for neurotrophins p75NTR plays a key role for satellite cell function in muscle repair acting via RhoA.	GeneRIF	0.0044	65.1	0.0114	0.116	1	1	NGFR
Pubmed	18273814	A/G Gln20Arg (exon 1) and G/A Val156Met (exon 5) polymorphisms of the human orosomucoid 1 gene in Mexico.	GeneRIF	0.0044	65.1	0.0114	0.116	1	1	ORM1
Pubmed	6238331	Molecular cloning and characterization of DNA sequences encoding rat and human atrial natriuretic factors.	Pubmed	0.0044	65.1	0.0114	0.116	1	1	NPPA
Pubmed	24138859	Molecular dynamics simulations of scorpion toxin recognition by the Ca(2+)-activated potassium channel KCa3.1.	Pubmed	0.0044	65.1	0.0114	0.116	1	1	KCNN4
Pubmed	19594366	Identification of different genomic deletions and one duplication in the dysferlin gene using multiplex ligation-dependent probe amplification and genomic quantitative PCR.	GeneRIF	0.0044	65.1	0.0114	0.116	1	1	DYSF
Pubmed	18728523	HLA-DQB1*02 dose effect on RIA anti-tissue transglutaminase autoantibody levels and clinicopathological expressivity of celiac disease.	GeneRIF	0.0044	65.1	0.0114	0.116	1	1	HLA-DQB1
Pubmed	9761252	Hemoglobin Rambam (beta69[E13]Gly-->Asp), a pitfall in the assessment of diabetic control: characterization by electrospray mass spectrometry and HPLC.	Pubmed	0.0044	65.1	0.0114	0.116	1	1	HBB
Pubmed	22394569	Serum level of soluble CX3CL1/fractalkine is elevated in patients with polymyositis and dermatomyositis, which is correlated with disease activity.	GeneRIF	0.0044	65.1	0.0114	0.116	1	1	CX3CL1
Pubmed	11897800	Expression of invariant chain (CD 74) and major histocompatibility complex (MHC) class II antigens in the human fetus.	GeneRIF	0.0044	65.1	0.0114	0.116	1	1	CD74
Pubmed	15464980	Molecular cloning and characterization of rat and human calpain-5.	GeneRIF	0.0044	65.1	0.0114	0.116	1	1	CAPN5
Pubmed	19029296	Small GTPase determinants for the Golgi processing and plasmalemmal expression of human ether-a-go-go related (hERG) K+ channels.	GeneRIF	0.0044	65.1	0.0114	0.116	1	1	KCNH2
Pubmed	25030421	Aberrant host defense against Leishmania major in the absence of SLPI.	Pubmed	0.0044	65.1	0.0114	0.116	1	1	SLPI
Pubmed	19649259	Host responses to intestinal microbial antigens in gluten-sensitive mice.	Pubmed	0.0044	65.1	0.0114	0.116	1	1	HLA-DQB1
Pubmed	16115946	Targeting matrilysin and its impact on tumor growth in vivo: the potential implications in breast cancer therapy.	GeneRIF	0.0044	65.1	0.0114	0.116	1	1	MMP7
Pubmed	17082482	The CX3C chemokine fractalkine induces vascular dysfunction by generation of superoxide anions.	GeneRIF	0.0044	65.1	0.0114	0.116	1	1	CX3CL1
Pubmed	23393392	Organ-specific protection against lipopolysaccharide-induced vascular leak is dependent on the endothelial protein C receptor.	GeneRIF	0.0044	65.1	0.0114	0.116	1	1	PROCR
Pubmed	3937824	Hemoglobin North Chicago (beta 36 [C2] proline--serine): a new high affinity hemoglobin.	Pubmed	0.0044	65.1	0.0114	0.116	1	1	HBB
Pubmed	17498734	Cardiac IK1 underlies early action potential shortening during hypoxia in the mouse heart.	Pubmed	0.0044	65.1	0.0114	0.116	1	1	KCNN4
Pubmed	19958184	Multi centric origin of Hb D-Punjab [beta121(GH4)Glu-->Gln, GAA>CAA].	GeneRIF	0.0044	65.1	0.0114	0.116	1	1	HBB
Pubmed	15991024	Diabetes-associated HLA genotypes affect birthweight in the general population.	GeneRIF	0.0044	65.1	0.0114	0.116	1	1	HLA-DQB1
Pubmed	9665396	Light and electron microscopic demonstration of the p75 nerve growth factor receptor in normal human cutaneous nerve fibers: new vistas.	Pubmed	0.0044	65.1	0.0114	0.116	1	1	NGFR
Pubmed	15576837	Expression of the endothelial lipase gene in murine embryos and reproductive organs.	GeneRIF	0.0044	65.1	0.0114	0.116	1	1	LIPG
Pubmed	18690018	An NH2-terminal multi-basic RKR motif is required for the ATP-dependent regulation of hIK1.	GeneRIF	0.0044	65.1	0.0114	0.116	1	1	KCNN4
Pubmed	15159300	Population-based case-control study of CYP11A gene polymorphism and breast cancer risk.	GeneRIF	0.0044	65.1	0.0114	0.116	1	1	CYP11A1

Category	ID	Name	Source	p-value	q-value Bonferroni	q-value FDR B&H	q-value FDR B&Y	Hit Count in Query List	Hit Count in Genome	Hit in Query List
Pubmed	21746810	Epithelial transglutaminase 2 is needed for T cell interleukin-17 production and subsequent pulmonary inflammation and fibrosis in bleomycin treated mice.	GeneRIF	0.0044	65.1	0.0114	0.116	1	1	TGM2
Pubmed	11872939	Study of the role of the low-affinity neurotrophin receptor p75 in naturally occurring cell death during development of the rat retina.	GeneRIF	0.0044	65.1	0.0114	0.116	1	1	NGFR
Pubmed	2101838	Low quantities of Hb Boyle Heights or alpha 2(6)(A4)Asp----O beta 2 observed in three members of a Caucasian family.	Pubmed	0.0044	65.1	0.0114	0.116	1	1	HBA2
Pubmed	19406877	The evolutionarily conserved residue A653 plays a key role in HERG channel closing.	GeneRIF	0.0044	65.1	0.0114	0.116	1	1	KCNH2
Pubmed	20046882	Diverse hematological malignancies including hodgkin-like lymphomas develop in chimeric MHC class II transgenic mice.	Pubmed	0.0044	65.1	0.0114	0.116	1	1	HLA-DQB1
Pubmed	12405606	Search for neuron-specific and nonneuron-specific antibodies in narcoleptic patients with and without HLA DQB1*0602.	GeneRIF	0.0044	65.1	0.0114	0.116	1	1	HLA-DQB1
Pubmed	11939521	Hb G-Chinese: a G-->C substitution at codon 30 of the alpha2-globin gene creates a PstI cutting site.	Pubmed	0.0044	65.1	0.0114	0.116	1	1	HBA2
Pubmed	10605006	Effects of T3R alpha 1 and T3R alpha 2 gene deletion on T and B lymphocyte development.	Pubmed	0.0044	65.1	0.0114	0.116	1	1	THRA
Pubmed	12807967	Haplotype analysis of distantly related populations implicates corneodesmosin in psoriasis susceptibility.	Pubmed	0.0044	65.1	0.0114	0.116	1	1	CDSN
Pubmed	1847678	Molecular cloning and chromosomal localization of a human peripheral-type benzodiazepine receptor.	Pubmed	0.0044	65.1	0.0114	0.116	1	1	TSPO
Pubmed	22875505	[Clinical investigation of a Chinese family with hypotrichosis simplex of the scalp and mutational analysis of CDSN gene].	GeneRIF	0.0044	65.1	0.0114	0.116	1	1	CDSN
Pubmed	2879242	The c-erb-A protein is a high-affinity receptor for thyroid hormone.	Pubmed	0.0044	65.1	0.0114	0.116	1	1	THRA
Pubmed	20580649	Increased depressive behaviour in mice harboring the mutant thyroid hormone receptor alpha 1.	GeneRIF	0.0044	65.1	0.0114	0.116	1	1	THRA
Pubmed	15469449	Dysferlin mutation analysis in a group of Italian patients with limb-girdle muscular dystrophy and Miyoshi myopathy.	GeneRIF	0.0044	65.1	0.0114	0.116	1	1	DYSF
Pubmed	18092889	Transglutaminase 2 undergoes a large conformational change upon activation.	GeneRIF	0.0044	65.1	0.0114	0.116	1	1	TGM2
Pubmed	7759119	Localization of the genes for the 100-kDa complement-activating components of Ra-reactive factor (CRARF and Crarf) to human 3q27-q28 and mouse 16B2-B3.	Pubmed	0.0044	65.1	0.0114	0.116	1	1	MASP1
Pubmed	14655281	[Role of aspartic acid at position 57 of the HLA-DQ beta chain in sporadic and familial forms of selective IgA deficiency].	GeneRIF	0.0044	65.1	0.0114	0.116	1	1	HLA-DQB1
Pubmed	11927011	Prognostic significance of matrix metalloproteinase-7 (MMP-7) expression at the invasive front in gastric carcinoma.	GeneRIF	0.0044	65.1	0.0114	0.116	1	1	MMP7
Pubmed	21700912	Transglutaminase 2 and its role in pulmonary fibrosis.	GeneRIF	0.0044	65.1	0.0114	0.116	1	1	TGM2
Pubmed	23033485	Identification and characterization of a compound that protects cardiac tissue from human Ether-à-go-go-related gene (hERG)-related drug-induced arrhythmias.	GeneRIF	0.0044	65.1	0.0114	0.116	1	1	KCNH2
Pubmed	21389146	Mechanism of escape from nonsense-mediated mRNA decay of human beta-globin transcripts with nonsense mutations in the first exon.	GeneRIF	0.0044	65.1	0.0114	0.116	1	1	HBB
Pubmed	20305575	A novel missense mutation in cathepsin K (CTSK) gene in a consanguineous Pakistani family with pycnodysostosis.	GeneRIF	0.0044	65.1	0.0114	0.116	1	1	CTSK
Pubmed	24799129	The nerve growth factor receptor CD271 is crucial to maintain tumorigenicity and stem-like properties of melanoma cells.	Pubmed	0.0044	65.1	0.0114	0.116	1	1	NGFR
Pubmed	20301656	Adenosine Deaminase Deficiency	Pubmed	0.0044	65.1	0.0114	0.116	1	1	ADA
Pubmed	3017948	Interferons as gene activators. Cloning of the 5' terminus and the control segment of an interferon activated gene.	Pubmed	0.0044	65.1	0.0114	0.116	1	1	IFI16
Pubmed	11820370	Identification of cis-DNA regions controlling Bmp4 expression during tooth morphogenesis in vivo.	Pubmed	0.0044	65.1	0.0114	0.116	1	1	BMP4
Pubmed	14754911	Synthesis and metabolism of leukotrienes in gamma-glutamyl transpeptidase deficiency.	GeneRIF	0.0044	65.1	0.0114	0.116	1	1	GGT1
Pubmed	17275810	Bone morphogenetic protein-4 is overexpressed in colonic adenocarcinomas and promotes migration and invasion of HCT116 cells.	GeneRIF	0.0044	65.1	0.0114	0.116	1	1	BMP4
Pubmed	18694524	Comparison of the mismatch-specific endonuclease method and denaturing high-performance liquid chromatography for the identification of HBB gene mutations.	GeneRIF	0.0044	65.1	0.0114	0.116	1	1	HBB
Pubmed	22574522	Characterization of 6-mercaptopurine binding site on human alpha1-acid glycoprotein (orosomucoid) using molecular docking.	GeneRIF	0.0044	65.1	0.0114	0.116	1	1	ORM1
Pubmed	18799080	Increased serum fractalkine in systemic sclerosis. Down-regulation by prostaglandin E1.	GeneRIF	0.0044	65.1	0.0114	0.116	1	1	CX3CL1
Pubmed	12198548	T-cell engagement of dendritic cells rapidly rearranges MHC class II transport.	Pubmed	0.0044	65.1	0.0114	0.116	1	1	HLA-DQB1
Pubmed	18235007	Tissue transglutaminase expression and activity in normal and glaucomatous human trabecular meshwork cells and tissues.	GeneRIF	0.0044	65.1	0.0114	0.116	1	1	TGM2
Pubmed	16781300	The vulvovaginal gingival syndrome: a severe subgroup of lichen planus with characteristic clinical features and a novel association with the class II HLA DQB1*0201 allele.	GeneRIF	0.0044	65.1	0.0114	0.116	1	1	HLA-DQB1
Pubmed	15848710	In the search of potential human islet stem cells: is tetranectin showing us the way?	GeneRIF	0.0044	65.1	0.0114	0.116	1	1	CLEC3B
Pubmed	23864605	Pharmacological correction of long QT-linked mutations in KCNH2 (hERG) increases the trafficking of Kv11.1 channels stored in the transitional endoplasmic reticulum.	Pubmed	0.0044	65.1	0.0114	0.116	1	1	KCNH2
Pubmed	7928378	A second case of Hb Hanamaki [alpha 2 139(HC1)Lys->Glu beta 2] in an American family with erythrocytosis.	Pubmed	0.0044	65.1	0.0114	0.116	1	1	HBA2

Category	ID	Name	Source	p-value	q-value Bonferroni	q-value FDR B&H	q-value FDR B&Y	Hit Count in Query List	Hit Count in Genome	Hit in Query List
Pubmed	23258229	Matrix metalloproteinase-7 coordinates airway epithelial injury response and differentiation of ciliated cells.	GeneRIF	0.0044	65.1	0.0114	0.116	1	1	MMP7
Pubmed	22042694	FTY720 increases CD74 expression and sensitizes mantle cell lymphoma cells to milatuzumab-mediated cell death.	GeneRIF	0.0044	65.1	0.0114	0.116	1	1	CD74
Pubmed	16103716	Alpha-2-globin gene polyadenylation (AATAAA-->AATAAG) mutation in hemoglobin H disease among Kuwaitis.	GeneRIF	0.0044	65.1	0.0114	0.116	1	1	HBA2
Pubmed	20353593	Genetic modifiers of Hb E/beta0 thalassemia identified by a two-stage genome-wide association study.	GeneRIF	0.0044	65.1	0.0114	0.116	1	1	HBB
Pubmed	24459672	Site-specific distribution of CD68-positive microglial cells in the brains of human midterm fetuses: a topographical relationship with growing axons.	Pubmed	0.0044	65.1	0.0114	0.116	1	1	CD68
Pubmed	23614625	Interaction of Hb adana (HBA2: c.179G>A) with deletional and nondeletional a(+)-thalassemia mutations: diverse hematological and clinical features.	Pubmed	0.0044	65.1	0.0114	0.116	1	1	HBA2
Pubmed	24488599	Diagnostic overview of blood-based dysferlin protein assay for dysferlinopathies.	Pubmed	0.0044	65.1	0.0114	0.116	1	1	DYSF
Pubmed	23975178	Clonal analysis of multipotent stromal cells derived from CD271+ bone marrow mononuclear cells: functional heterogeneity and different mechanisms of allosuppression.	Pubmed	0.0044	65.1	0.0114	0.116	1	1	NGFR
Pubmed	20097443	Keratins regulate yolk sac hematopoiesis and vasculogenesis through reduced BMP-4 signaling.	GeneRIF	0.0044	65.1	0.0114	0.116	1	1	BMP4
Pubmed	19783326	No association between high temperature requirement 1 (HTRA1) gene polymorphisms and Alzheimer's disease.	GeneRIF	0.0044	65.1	0.0114	0.116	1	1	HTRA1
Pubmed	19014391	Peptidomics of Cpe(fat/fat) mouse brain regions: implications for neuropeptide processing.	Pubmed	0.0044	65.1	0.0114	0.116	1	1	CPE
Pubmed	22100990	Transfection by eukaryotic expression vector pcDNA3-HERG inhibits the cultured neonatal rabbit ventricular myocyte hypertrophy induced by phenylephrine.	GeneRIF	0.0044	65.1	0.0114	0.116	1	1	KCNH2
Pubmed	12063277	Role of glycosylation in cell surface expression and stability of HERG potassium channels.	Pubmed	0.0044	65.1	0.0114	0.116	1	1	KCNH2
Pubmed	21646052	Altered norepinephrine content and ventricular function in p75NTR-/- mice after myocardial infarction.	GeneRIF	0.0044	65.1	0.0114	0.116	1	1	NGFR
Pubmed	25015117	Different associations between obesity and impaired fasting glucose depending on serum gamma-glutamyltransferase levels within normal range: a cross-sectional study.	Pubmed	0.0044	65.1	0.0114	0.116	1	1	TGM2
Pubmed	11597766	Multiple transcripts generated by the DCAMKL gene are expressed in the rat hippocampus.	GeneRIF	0.0044	65.1	0.0114	0.116	1	1	DCLK1
Pubmed	3399404	Multiple sequences encoding potential thyroid hormone receptors isolated from mouse skeletal muscle cDNA libraries.	Pubmed	0.0044	65.1	0.0114	0.116	1	1	THRA
Pubmed	14607905	CD8alpha+ and CD11b+ dendritic cell-restricted MHC class II controls Th1 CD4+ T cell immunity.	Pubmed	0.0044	65.1	0.0114	0.116	1	1	HLA-DQB1
Pubmed	21896673	Genome-wide association study of HPV seropositivity.	Pubmed	0.0044	65.1	0.0114	0.116	1	1	HLA-DQB1
Pubmed	23215760	Incidence of the Hb E [ß26(B8)Glu¿Lys, GAG>AAG] variant in Totos, one of the smallest primitive tribes in the world.	GeneRIF	0.0044	65.1	0.0114	0.116	1	1	HBB
Pubmed	8602627	Hemoglobin Debrousse (beta 96[FG3]Leu-->Pro): a new unstable hemoglobin with twofold increased oxygen affinity.	Pubmed	0.0044	65.1	0.0114	0.116	1	1	HBB
Pubmed	9342357	A gene spans the pseudoautosomal boundary in mice.	Pubmed	0.0044	65.1	0.0114	0.116	1	1	MID1
Pubmed	17584847	Abolition of Ca2+-mediated intestinal anion secretion and increased stool dehydration in mice lacking the intermediate conductance Ca2+-dependent K+ channel Kcnn4.	GeneRIF	0.0044	65.1	0.0114	0.116	1	1	KCNN4
Pubmed	17292492	N-terminal pro-atrial natriuretic peptide and exercise prescription in patients with myocardial infarction.	GeneRIF	0.0044	65.1	0.0114	0.116	1	1	NPPA
Pubmed	25209703	Characterization of distinct sub-cellular location of transglutaminase type II: changes in intracellular distribution in physiological and pathological states.	Pubmed	0.0044	65.1	0.0114	0.116	1	1	TGM2
Pubmed	18715980	SPINKL, a Kazal-type serine protease inhibitor-like protein purified from mouse seminal vesicle fluid, is able to inhibit sperm capacitation.	Pubmed	0.0044	65.1	0.0114	0.116	1	1	SPINK1
Pubmed	19061996	Early follicle development alters the relationship between antral follicle counts and inhibin B and follicle-stimulating hormone levels on cycle day 3.	GeneRIF	0.0044	65.1	0.0114	0.116	1	1	INHBB
Pubmed	12110289	Cysteine dioxygenase: modulation of expression in human cell lines by cytokines and control of sulphate production.	Pubmed	0.0044	65.1	0.0114	0.116	1	1	CDO1
Pubmed	3110276	Structure-function analysis of the Abm12 beta mutation using site-directed mutagenesis and DNA-mediated gene transfer.	Pubmed	0.0044	65.1	0.0114	0.116	1	1	HLA-DQB1
Pubmed	17594537	Transcriptional regulation of human CYP11A1 in gonads and adrenals.	GeneRIF	0.0044	65.1	0.0114	0.116	1	1	CYP11A1
Pubmed	3744871	A new electrophoretically-silent hemoglobin variant: hemoglobin Kofu or alpha 2 beta 2 84 (EF8) Thr----Ile.	Pubmed	0.0044	65.1	0.0114	0.116	1	1	HBB
Pubmed	23715724	Genetic ablation of myelin protein zero-like 3 in mice increases energy expenditure, improves glycemic control, and reduces hepatic lipid synthesis.	GeneRIF	0.0044	65.1	0.0114	0.116	1	1	MPZL3
Pubmed	16100390	Osteoactivin upregulates expression of MMP-3 and MMP-9 in fibroblasts infiltrated into denervated skeletal muscle in mice.	GeneRIF	0.0044	65.1	0.0114	0.116	1	1	GPNMB
Pubmed	23686108	Up-regulation of Slc39A2(Zip2) mRNA in peripheral blood mononuclear cells from patients with pulmonary tuberculosis.	Pubmed	0.0044	65.1	0.0114	0.116	1	1	SLC39A2
Pubmed	10843886	Inducible polymorphic ventricular tachyarrhythmias in a transgenic mouse model with a long Q-T phenotype.	Pubmed	0.0044	65.1	0.0114	0.116	1	1	KCNH2

Category	ID	Name	Source	p-value	q-value Bonferroni	q-value FDR B&H	q-value FDR B&Y	Hit Count in Query List	Hit Count in Genome	Hit in Query List
Pubmed	17503335	Matthew-Wood syndrome is caused by truncating mutations in the retinol-binding protein receptor gene STRA6.	GeneRIF	0.0044	65.1	0.0114	0.116	1	1	STRA6
Pubmed	12865230	Molecular epidemiological study of alpha- and beta-thalassemia in Sihui city.	GeneRIF	0.0044	65.1	0.0114	0.116	1	1	HBB
Pubmed	16285941	Unexpected role of surface transglutaminase type II in celiac disease.	GeneRIF	0.0044	65.1	0.0114	0.116	1	1	TGM2
Pubmed	7579699	The metalloproteinase matrilysin is preferentially expressed by epithelial cells in a tissue-restricted pattern in the mouse.	Pubmed	0.0044	65.1	0.0114	0.116	1	1	MMP7
Pubmed	9586666	Colorectal carcinoma invasion inhibition by CO17-1A/GA733 antigen and its murine homologue.	Pubmed	0.0044	65.1	0.0114	0.116	1	1	TACSTD2
Pubmed	24465594	Angiopoietin-like protein 2 induced by mechanical stress accelerates degeneration and hypertrophy of the ligamentum flavum in lumbar spinal canal stenosis.	Pubmed	0.0044	65.1	0.0114	0.116	1	1	ANGPTL2
Pubmed	17928338	The rabies virus glycoprotein receptor p75NTR is not essential for rabies virus infection.	GeneRIF	0.0044	65.1	0.0114	0.116	1	1	NGFR
Pubmed	17688704	Classical sickle beta-globin haplotypes exhibit a high degree of long-range haplotype similarity in African and Afro-Caribbean populations.	GeneRIF	0.0044	65.1	0.0114	0.116	1	1	HBB
Pubmed	18759082	Beta-thalassemia mutations in western India.	GeneRIF	0.0044	65.1	0.0114	0.116	1	1	HBB
Pubmed	24439080	Pulmonary alveolar proteinosis in adenosine deaminase-deficient mice.	Pubmed	0.0044	65.1	0.0114	0.116	1	1	ADA
Pubmed	23712470	HtrA1 in human urothelial bladder cancer: a secreted protein and a potential novel biomarker.	Pubmed	0.0044	65.1	0.0114	0.116	1	1	HTRA1
Pubmed	8420838	Complete characterization of the expressed immune response genes in Biozzi AB/H mice: structural and functional identity between AB/H and NOD A region molecules.	Pubmed	0.0044	65.1	0.0114	0.116	1	1	HLA-DQB1
Pubmed	7626220	The nucleotide sequence, structure, and preliminary studies on the transcriptional regulation of the bovine alpha skeletal actin gene.	Pubmed	0.0044	65.1	0.0114	0.116	1	1	ACTA1
Pubmed	24510064	Bone morphogenetic protein-4 induces upregulation of Cav3.1 Ca^2¿ channels in HL-1 atrial myocytes.	Pubmed	0.0044	65.1	0.0114	0.116	1	1	BMP4
Pubmed	10777534	Characterization and regulation of the 5'-flanking region of the murine endothelial protein C receptor gene.	Pubmed	0.0044	65.1	0.0114	0.116	1	1	PROCR
Pubmed	16728349	Inhibin B levels in peripheral vein do not correlate with inhibin B levels in the spermatic vein in adolescents with varicocele.	GeneRIF	0.0044	65.1	0.0114	0.116	1	1	INHBB
Pubmed	22075385	Uterine and ovarian carcinosarcomas overexpressing Trop-2 are sensitive to hRS7, a humanized anti-Trop-2 antibody.	GeneRIF	0.0044	65.1	0.0114	0.116	1	1	TACSTD2
Pubmed	17654503	Increased gamma-globin gene expression in beta-thalassemia intermedia patients correlates with a mutation in 3'HS1.	GeneRIF	0.0044	65.1	0.0114	0.116	1	1	HBB
Pubmed	12034504	Molecular cloning and initial characterization of the MG61/PORC gene, the human homologue of the Drosophila segment polarity gene Porcupine.	Pubmed	0.0044	65.1	0.0114	0.116	1	1	PORCN
Pubmed	19084402	A new phenotype of dysferlinopathy with congenital onset.	GeneRIF	0.0044	65.1	0.0114	0.116	1	1	DYSF
Pubmed	11101006	Development of an in vitro model for cysteine dioxygenase expression in the brain.	Pubmed	0.0044	65.1	0.0114	0.116	1	1	CDO1
Pubmed	11468312	Genomic organization of the dysferlin gene and novel mutations in Miyoshi myopathy.	Pubmed	0.0044	65.1	0.0114	0.116	1	1	DYSF
Pubmed	12618908	Extensive polymorphism and different evolutionar patterns of intron 2 were identified in the HLA-DQB1 gene.	GeneRIF	0.0044	65.1	0.0114	0.116	1	1	HLA-DQB1
Pubmed	22535375	Integrated epigenomics identifies BMP4 as a modulator of cisplatin sensitivity in gastric cancer.	GeneRIF	0.0044	65.1	0.0114	0.116	1	1	BMP4
Pubmed	21800356	Sideroblastic anemia, iron overload, and ALAS2 R452S in African-American males: phenotype and genotype features of five unrelated patients.	GeneRIF	0.0044	65.1	0.0114	0.116	1	1	ALAS2
Pubmed	21178583	NGFR (p75) expression in cutaneous scars; Further evidence for a potential pitfall in evaluation of reexcision scars of cutaneous neoplasms, in particular desmoplastic melanoma.	GeneRIF	0.0044	65.1	0.0114	0.116	1	1	NGFR
Pubmed	8635257	Missense mutation in the pore region of HERG causes familial long QT syndrome.	Pubmed	0.0044	65.1	0.0114	0.116	1	1	KCNH2
Pubmed	24939843	A role for the p75 neurotrophin receptor in axonal degeneration and apoptosis induced by oxidative stress.	Pubmed	0.0044	65.1	0.0114	0.116	1	1	NGFR
Pubmed	11705853	Analysis of normal epithelial cell specific-1 (NES1)/kallikrein 10 mRNA expression by in situ hybridization, a novel marker for breast cancer.	GeneRIF	0.0044	65.1	0.0114	0.116	1	1	KLK10
Pubmed	19300392	Evaluation of association between the CYP11alpha promoter pentannucleotide (TTTTA)n polymorphism and polycystic ovarian syndrome among Han Chinese women.	GeneRIF	0.0044	65.1	0.0114	0.116	1	1	CYP11A1
Pubmed	20036632	An investigation of the occurrence and properties of the mitochondrial intermediate-conductance Ca2+-activated K+ channel mtKCa3.1.	GeneRIF	0.0044	65.1	0.0114	0.116	1	1	KCNN4
Pubmed	16261636	Glycosylation site analysis of human alpha-1-acid glycoprotein (AGP) by capillary liquid chromatography-electrospray mass spectrometry.	GeneRIF	0.0044	65.1	0.0114	0.116	1	1	ORM1
Pubmed	22041182	Control of hypothalamic-pituitary-adrenal stress axis activity by the intermediate conductance calcium-activated potassium channel, SK4.	Pubmed	0.0044	65.1	0.0114	0.116	1	1	KCNN4
Pubmed	7716822	Expression of an endogenous retrovirus (ERV3 HERV-R) in human reproductive and embryonic tissues--evidence for a function for envelope gene products.	Pubmed	0.0044	65.1	0.0114	0.116	1	1	ERV3-1
Pubmed	21520051	Differential regulation of the human CYP11A1 promoter in mouse brain and adrenals.	GeneRIF	0.0044	65.1	0.0114	0.116	1	1	CYP11A1
Pubmed	20628988	Hearing impairment in persons with the hemoglobin SC genotype.	GeneRIF	0.0044	65.1	0.0114	0.116	1	1	HBB

Category	ID	Name	Source	p-value	q-value Bonferroni	q-value FDR B&H	q-value FDR B&Y	Hit Count in Query List	Hit Count in Genome	Hit in Query List
Pubmed	12709368	Rapid screening of multiple beta-globin gene mutations by real-time PCR on the LightCycler: application to carrier screening and prenatal diagnosis of thalassemia syndromes.	GeneRIF	0.0044	65.1	0.0114	0.116	1	1	HBB
Pubmed	5587575	Hemoglobin hasharon (alpha-47 aspartic acid--histidine).	Pubmed	0.0044	65.1	0.0114	0.116	1	1	HBA2
Pubmed	22862814	Two novel mutations in the 3' untranslated region of the beta-globin gene that are associated with the mild phenotype of beta thalassemia.	GeneRIF	0.0044	65.1	0.0114	0.116	1	1	HBB
Pubmed	14689592	p75 neurotrophin receptor functions as a survival receptor in brain-metastatic melanoma cells.	GeneRIF	0.0044	65.1	0.0114	0.116	1	1	NGFR
Pubmed	25312915	Identification of splicing defects caused by mutations in the dysferlin gene.	Pubmed	0.0044	65.1	0.0114	0.116	1	1	DYSF
Pubmed	9399693	Genotypic selection provides experimental confirmation for an alcohol consumption quantitative trait locus in mouse.	Pubmed	0.0044	65.1	0.0114	0.116	1	1	TSPO
Pubmed	24798237	Aberrant methylation and decreased expression of the TGF-ß/Smad target gene FBXO32 in esophageal squamous cell carcinoma.	Pubmed	0.0044	65.1	0.0114	0.116	1	1	FBXO32
Pubmed	11814414	SSeCKS immunolabeling in rat primary sensory neurons.	GeneRIF	0.0044	65.1	0.0114	0.116	1	1	AKAP12
Pubmed	18199717	Hypermethylation of the AKAP12 promoter is a biomarker of Barrett's-associated esophageal neoplastic progression.	GeneRIF	0.0044	65.1	0.0114	0.116	1	1	AKAP12
Pubmed	22686418	Mutation analysis of the STRA6 gene in isolated and non-isolated anophthalmia/microphthalmia.	GeneRIF	0.0044	65.1	0.0114	0.116	1	1	STRA6
Pubmed	12908805	Two new hemoglobin variants with increased oxygen affinity: Hb Nantes [beta34(B16)Val-->Leu] and Hb Vexin [beta116(G18)His-->Leu].	Pubmed	0.0044	65.1	0.0114	0.116	1	1	HBB
Pubmed	18675227	Cellular properties of C-terminal KCNH2 long QT syndrome mutations: description and divergence from clinical phenotypes.	GeneRIF	0.0044	65.1	0.0114	0.116	1	1	KCNH2
Pubmed	2515150	Molecular cloning of a human co-beta-glucosidase cDNA: evidence that four sphingolipid hydrolase activator proteins are encoded by single genes in humans and rats.	Pubmed	0.0044	65.1	0.0114	0.116	1	1	PSAP
Pubmed	24914936	Thyroid hormone receptor a mutation causes a severe and thyroxine-resistant skeletal dysplasia in female mice.	Pubmed	0.0044	65.1	0.0114	0.116	1	1	THRA
Pubmed	6095118	Gene structure of human cardiac hormone precursor, pronatriodilatin.	Pubmed	0.0044	65.1	0.0114	0.116	1	1	NPPA
Pubmed	10599883	Corneodesmosin gene polymorphism demonstrates strong linkage disequilibrium with HLA and association with psoriasis vulgaris.	Pubmed	0.0044	65.1	0.0114	0.116	1	1	CDSN
Pubmed	15910626	The SPINK1 N34S mutation is not associated with Type 2 diabetes mellitus in a population of the USA.	GeneRIF	0.0044	65.1	0.0114	0.116	1	1	SPINK1
Pubmed	14724753	Modulation of calcium-dependent chloride secretion by basolateral SK4-like channels in a human bronchial cell line.	GeneRIF	0.0044	65.1	0.0114	0.116	1	1	KCNN4
Pubmed	15336687	Follow-up of nemaline myopathy in two patients with novel mutations in the skeletal muscle alpha-actin gene (ACTA1).	GeneRIF	0.0044	65.1	0.0114	0.116	1	1	ACTA1
Pubmed	25056659	Human buccal epithelium acquires microbial hyporesponsiveness at birth, a role for secretory leukocyte protease inhibitor.	Pubmed	0.0044	65.1	0.0114	0.116	1	1	SLPI
Pubmed	12419253	Definition of transcriptional promoters in the human beta globin locus control region.	GeneRIF	0.0044	65.1	0.0114	0.116	1	1	HBB
Pubmed	6112263	Glutathione and gamma-glutamyl cycle enzymes in human fetal liver.	Pubmed	0.0044	65.1	0.0114	0.116	1	1	GSS
Pubmed	25120755	Aldo-keto reductase family 1 member B8 is secreted via non-classical pathway.	Pubmed	0.0044	65.1	0.0114	0.116	1	1	AKR1B10
Pubmed	21797700	ß-Thalassemia mutations found during 1 year of prenatal diagnoses in Fars Province, Iran.	GeneRIF	0.0044	65.1	0.0114	0.116	1	1	HBB
Pubmed	11943193	The interferon-inducible gene, Ifi204, acquires malignant transformation capability upon mutation at the Rb-binding sites.	GeneRIF	0.0044	65.1	0.0114	0.116	1	1	IFI16
Pubmed	18398851	Decreased hippocampal cell proliferation correlates with increased expression of BMP4 in the APPswe/PS1DeltaE9 mouse model of Alzheimer's disease.	GeneRIF	0.0044	65.1	0.0114	0.116	1	1	BMP4
Pubmed	9414266	Successful peripheral T-lymphocyte-directed gene transfer for a patient with severe combined immune deficiency caused by adenosine deaminase deficiency.	Pubmed	0.0044	65.1	0.0114	0.116	1	1	ADA
Pubmed	18368131	20-Hydroxyvitamin D3, a product of vitamin D3 hydroxylation by cytochrome P450scc, stimulates keratinocyte differentiation.	GeneRIF	0.0044	65.1	0.0114	0.116	1	1	CYP11A1
Pubmed	7929370	Identification, cloning, and regulation of a novel endothelial cell protein C/activated protein C receptor.	Pubmed	0.0044	65.1	0.0114	0.116	1	1	PROCR
Pubmed	21628317	Sarcolemma instability during mechanical activity in Largemyd cardiac myocytes with loss of dystroglycan extracellular matrix receptor function.	Pubmed	0.0044	65.1	0.0114	0.116	1	1	LARGE
Pubmed	8614797	p75NTR: a receptor after all.	Pubmed	0.0044	65.1	0.0114	0.116	1	1	NGFR
Pubmed	1674946	Expression of the secretory leukoprotease inhibitor gene in epithelial cells.	Pubmed	0.0044	65.1	0.0114	0.116	1	1	SLPI
Pubmed	271989	Molecular evolution of human and rabbit beta-globin mRNAs.	Pubmed	0.0044	65.1	0.0114	0.116	1	1	HBB
Pubmed	21712391	Autocatalytic cleavage of human gamma-glutamyl transpeptidase is highly dependent on N-glycosylation at asparagine 95.	GeneRIF	0.0044	65.1	0.0114	0.116	1	1	GGT1
Pubmed	21418992	[Expression and clinical significance of Trop-2 in human pancreatic cancer].	GeneRIF	0.0044	65.1	0.0114	0.116	1	1	TACSTD2
Pubmed	24802353	Fannin-Lubbock-I [a2ß2^{11}¿(GLY>ASP)], a rare mutation in the beta-globin gene, has been detected for the first time in a Hindu Brahmin family in West Bengal, India.	Pubmed	0.0044	65.1	0.0114	0.116	1	1	HBB
Pubmed	21508100	Endogenic regulation of proliferation and zinc transporters by pigment epithelial cells nonvirally transfected with PEDF.	Pubmed	0.0044	65.1	0.0114	0.116	1	1	SLC39A2
Pubmed	21493782	Calcium-activated potassium channel KCa3.1 in lung dendritic cell migration.	GeneRIF	0.0044	65.1	0.0114	0.116	1	1	KCNN4

Category	ID	Name	Source	p-value	q-value Bonferroni	q-value FDR B&H	q-value FDR B&Y	Hit Count in Query List	Hit Count in Genome	Hit in Query List
Pubmed	10611506	p75(NGFR) and cholinergic neurons in the developing forebrain: a re-examination.	Pubmed	0.0044	65.1	0.0114	0.116	1	1	NGFR
Pubmed	5577460	Haemoglobin Rampa: Alpha 95 Pro--Ser.	Pubmed	0.0044	65.1	0.0114	0.116	1	1	HBA2
Pubmed	23070813	Regulated proteolysis of Trop2 drives epithelial hyperplasia and stem cell self-renewal via ß-catenin signaling.	GeneRIF	0.0044	65.1	0.0114	0.116	1	1	TACSTD2
Pubmed	11358448	Upregulation of p75 neurotrophin receptor after stroke in mice does not contribute to differential vulnerability of striatal neurons.	Pubmed	0.0044	65.1	0.0114	0.116	1	1	NGFR
Pubmed	18385089	Modulation of Nrf2-dependent antioxidant functions in the RPE by Zip2, a zinc transporter protein.	Pubmed	0.0044	65.1	0.0114	0.116	1	1	SLC39A2
Pubmed	17273977	Mutations in STRA6 cause a broad spectrum of malformations including anophthalmia, congenital heart defects, diaphragmatic hernia, alveolar capillary dysplasia, lung hypoplasia, and mental retardation.	GeneRIF	0.0044	65.1	0.0114	0.116	1	1	STRA6
Pubmed	17939176	Missense mutations in the TGM2 gene encoding transglutaminase 2 are found in patients with early-onset type 2 diabetes. Mutation in brief no. 982. Online.	GeneRIF	0.0044	65.1	0.0114	0.116	1	1	TGM2
Pubmed	11077419	Primary olfactory axons form ectopic glomeruli in mice lacking p75NTR.	Pubmed	0.0044	65.1	0.0114	0.116	1	1	NGFR
Pubmed	18075823	Innate host defense functions of secretory leucoprotease inhibitor.	GeneRIF	0.0044	65.1	0.0114	0.116	1	1	SLPI
Pubmed	22575314	Influence of rs5065 atrial natriuretic peptide gene variant on coronary artery disease.	GeneRIF	0.0044	65.1	0.0114	0.116	1	1	NPPA
Pubmed	25100328	Preclinical characterization of recombinant human tissue kallikrein-1 as a novel treatment for type 2 diabetes mellitus.	Pubmed	0.0044	65.1	0.0114	0.116	1	1	KLK1
Pubmed	22329800	Epidermal growth factor induces tumour marker AKR1B10 expression through activator protein-1 signalling in hepatocellular carcinoma cells.	GeneRIF	0.0044	65.1	0.0114	0.116	1	1	AKR1B10
Pubmed	19654318	Control of promatrilysin (MMP7) activation and substrate-specific activity by sulfated glycosaminoglycans.	GeneRIF	0.0044	65.1	0.0114	0.116	1	1	MMP7
Pubmed	18218852	Altered intracellular and extracellular signaling leads to impaired T-cell functions in ADA-SCID patients.	GeneRIF	0.0044	65.1	0.0114	0.116	1	1	ADA
Pubmed	17726294	Endothelial lipase is increased by inflammation and promotes LDL uptake in macrophages.	GeneRIF	0.0044	65.1	0.0114	0.116	1	1	LIPG
Pubmed	15528201	Regional specificity of human ether-a'-go-go-related gene channel activation and inactivation gating.	GeneRIF	0.0044	65.1	0.0114	0.116	1	1	KCNH2
Pubmed	10567372	Sequence, purification, and cloning of an intracellular serine protease, quiescent cell proline dipeptidase.	Pubmed	0.0044	65.1	0.0114	0.116	1	1	DPP7
Pubmed	25040843	Functional analysis of the fractalkine gene promoter in human aortic smooth muscle cells exposed to proinflammatory conditions.	Pubmed	0.0044	65.1	0.0114	0.116	1	1	CX3CL1
Pubmed	15541364	Arachidonic acid activates tissue transglutaminase and stress fiber formation via intracellular reactive oxygen species.	Pubmed	0.0044	65.1	0.0114	0.116	1	1	TGM2
Pubmed	1019344	Nucleotide sequence analysis of coding and noncoding regions of human beta-globin mRNA.	Pubmed	0.0044	65.1	0.0114	0.116	1	1	HBB
Pubmed	24141948	Antigen-specific B-cell receptor sensitizes B cells to infection by influenza virus.	Pubmed	0.0044	65.1	0.0114	0.116	1	1	HLA-DQB1
Pubmed	24954028	Distribution of PCP4 protein in the forebrain of adult mice.	Pubmed	0.0044	65.1	0.0114	0.116	1	1	PCP4
Pubmed	15276679	Lack of association between Connexin 31 (GJB3) alterations and sensorineural deafness in Austria.	GeneRIF	0.0044	65.1	0.0114	0.116	1	1	GJB3
Pubmed	6618888	Hemoglobin Jianghua [beta 120(GH3) LYS leads to ILE]: a new fast-moving variant found in China.	Pubmed	0.0044	65.1	0.0114	0.116	1	1	HBB
Pubmed	24132781	Differential regulation of mouse pancreatic islet insulin secretion and Smad proteins by activin ligands.	Pubmed	0.0044	65.1	0.0114	0.116	1	1	INHBB
Pubmed	12935677	Two STR loci polymorphisms of adenosine deaminase gene in Japanese population.	GeneRIF	0.0044	65.1	0.0114	0.116	1	1	ADA
Pubmed	8358941	Gene cloning and expression of a bone morphogenetic protein derived from a murine osteosarcoma.	Pubmed	0.0044	65.1	0.0114	0.116	1	1	BMP4
Pubmed	19183553	Degradation of transglutaminase 2 by calcium-mediated ubiquitination responding to high oxidative stress.	GeneRIF	0.0044	65.1	0.0114	0.116	1	1	TGM2
Pubmed	16957554	Tissue kallikrein gene polymorphisms induce no change in endothelium-dependent or independent vasodilation in hypertensive and normotensive subjects.	GeneRIF	0.0044	65.1	0.0114	0.116	1	1	KLK1
Pubmed	22889833	Down-regulation and CpG island hypermethylation of CRYAA in age-related nuclear cataract.	GeneRIF	0.0044	65.1	0.0114	0.116	1	1	CRYAA
Pubmed	15753612	Chronic parotitis: not another SPINKosis.	GeneRIF	0.0044	65.1	0.0114	0.116	1	1	SPINK1
Pubmed	16399053	Long QT syndrome caused by a large duplication in the KCNH2 (HERG) gene undetectable by current polymerase chain reaction-based exon-scanning methodologies.	GeneRIF	0.0044	65.1	0.0114	0.116	1	1	KCNH2
Pubmed	15728246	Up-regulation of the interferon-inducible IFI16 gene by oxidative stress triggers p53 transcriptional activity in endothelial cells.	GeneRIF	0.0044	65.1	0.0114	0.116	1	1	IFI16
Pubmed	12365083	[Polymorphism of aldose reductase gene and susceptibility to retinopathy and nephropathy in Caucasians with type 1 diabetes].	GeneRIF	0.0044	65.1	0.0114	0.116	1	1	AKR1B10
Pubmed	16354176	Further evidence of multifunctionality in the keratinocyte: the endothelial protein C receptor.	GeneRIF	0.0044	65.1	0.0114	0.116	1	1	PROCR
Pubmed	7806230	Fibulin-2 (FBLN2): human cDNA sequence, mRNA expression, and mapping of the gene on human and mouse chromosomes.	Pubmed	0.0044	65.1	0.0114	0.116	1	1	FBLN2
Pubmed	24120856	The association of alcohol intake with ¿-glutamyl transferase (GGT) levels: evidence for correlated genetic effects.	Pubmed	0.0044	65.1	0.0114	0.116	1	1	GGT1
Pubmed	19032804	In silico prediction of the chemical block of human ether-a-go-go-related gene (hERG) K+ current.	GeneRIF	0.0044	65.1	0.0114	0.116	1	1	KCNH2
Pubmed	11237463	Connexin31-deficiency in mice causes transient placental dysmorphogenesis but does not impair hearing and skin differentiation.	Pubmed	0.0044	65.1	0.0114	0.116	1	1	GJB3

Category	ID	Name	Source	p-value	q-value Bonferroni	q-value FDR B&H	q-value FDR B&Y	Hit Count in Query List	Hit Count in Genome	Hit in Query List
Pubmed	14587041	Beta-globin gene cluster haplotypes in sickle cell patients from southwest Iran.	GeneRIF	0.0044	65.1	0.0114	0.116	1	1	HBB
Pubmed	23133669	Ion conduction through the hERG potassium channel.	GeneRIF	0.0044	65.1	0.0114	0.116	1	1	KCNH2
Pubmed	24177326	Positive feedback regulation of agonist-stimulated endothelial Ca2+ dynamics by KCa3.1 channels in mouse mesenteric arteries.	Pubmed	0.0044	65.1	0.0114	0.116	1	1	KCNN4
Pubmed	19696402	FVII, FVIIa, and downstream markers of extrinsic pathway activation differ by EPCR Ser219Gly variant in healthy men.	GeneRIF	0.0044	65.1	0.0114	0.116	1	1	PROCR
Pubmed	4422784	Haemoglobin Icaria, a new chain-termination mutant with causes alpha thalassaemia.	Pubmed	0.0044	65.1	0.0114	0.116	1	1	HBA2
Pubmed	11167850	Hereditary non-spherocytic haemolytic anaemia due to red blood cell glutathione synthetase deficiency in four unrelated patients from Spain: clinical and molecular studies.	Pubmed	0.0044	65.1	0.0114	0.116	1	1	GSS
Pubmed	12835487	Correlation of trace mineral concentrations with fructose, gamma-glutamyl transpeptidase, and acid phosphatase in seminal plasma of different categories of infertile men.	GeneRIF	0.0044	65.1	0.0114	0.116	1	1	GGT1
Pubmed	7790890	Processing of procarboxypeptidase E into carboxypeptidase E occurs in secretory vesicles.	Pubmed	0.0044	65.1	0.0114	0.116	1	1	CPE
Pubmed	3384709	Hemoglobin Pierre-Bénite [beta 90(F6)Glu----Asp], a new high affinity variant found in a French family.	Pubmed	0.0044	65.1	0.0114	0.116	1	1	HBB
Pubmed	19685083	Functional analysis of BMP4 mutations identified in pediatric CAKUT patients.	GeneRIF	0.0044	65.1	0.0114	0.116	1	1	BMP4
Pubmed	24650875	MAFbx/Atrogin-1 is required for atrophic remodeling of the unloaded heart.	Pubmed	0.0044	65.1	0.0114	0.116	1	1	FBXO32
Pubmed	23546015	Mechanistic basis for type 2 long QT syndrome caused by KCNH2 mutations that disrupt conserved arginine residues in the voltage sensor.	Pubmed	0.0044	65.1	0.0114	0.116	1	1	KCNH2
Pubmed	20693282	Alternative splicing and polyadenylation contribute to the generation of hERG1 C-terminal isoforms.	GeneRIF	0.0044	65.1	0.0114	0.116	1	1	KCNH2
Pubmed	24533101	Gamma-glutamyltransferase fractions in human plasma and bile: characteristic and biogenesis.	Pubmed	0.0044	65.1	0.0114	0.116	1	1	GGT1
Pubmed	16472284	Functional significance of KCNH2 (HERG) K897T polymorphism for cardiac repolarization assessed by analysis of T-wave morphology.	GeneRIF	0.0044	65.1	0.0114	0.116	1	1	KCNH2
Pubmed	18768862	B7-H3 contributes to the development of pathogenic Th2 cells in a murine model of asthma.	GeneRIF	0.0044	65.1	0.0114	0.116	1	1	CD276
Pubmed	21908602	Molecular coupling in the human ether-a-go-go-related gene-1 (hERG1) K+ channel inactivation pathway.	GeneRIF	0.0044	65.1	0.0114	0.116	1	1	KCNH2
Pubmed	12682247	APCs present A beta(k)-derived peptides that are autoantigenic to type B T cells.	GeneRIF	0.0044	65.1	0.0114	0.116	1	1	HLA-DQB1
Pubmed	21517234	Role of the metalloproteinase-7 (181A>G) polymorphism in gastric cancer susceptibility: a case control study in Kashmir valley.	GeneRIF	0.0044	65.1	0.0114	0.116	1	1	MMP7
Pubmed	22537232	Secretory leukocyte protease inhibitor binds to Neisseria gonorrhoeae outer membrane opacity protein and is bactericidal.	GeneRIF	0.0044	65.1	0.0114	0.116	1	1	SLPI
Pubmed	10657504	Altered biosynthesis and secretion of pro-opiomelanocortin in the intermediate and anterior pituitary of carboxypeptidase E-deficient, Cpe(fat)/Cpe(fat)mice.	Pubmed	0.0044	65.1	0.0114	0.116	1	1	CPE
Pubmed	19309693	Phenotypic spectrum of STRA6 mutations: from Matthew-Wood syndrome to non-lethal anophthalmia.	GeneRIF	0.0044	65.1	0.0114	0.116	1	1	STRA6
Pubmed	24789905	Atrogin-1 deficiency promotes cardiomyopathy and premature death via impaired autophagy.	Pubmed	0.0044	65.1	0.0114	0.116	1	1	FBXO32
Pubmed	21133715	Association of HLA-DQB1 alleles with risk of follicular lymphoma.	GeneRIF	0.0044	65.1	0.0114	0.116	1	1	HLA-DQB1
Pubmed	2334405	Human carboxypeptidase E. Isolation and characterization of the cDNA, sequence conservation, expression and processing in vitro.	Pubmed	0.0044	65.1	0.0114	0.116	1	1	CPE
Pubmed	20492353	Carboxypeptidase E cytoplasmic tail mediates localization of synaptic vesicles to the pre-active zone in hypothalamic pre-synaptic terminals.	GeneRIF	0.0044	65.1	0.0114	0.116	1	1	CPE
Pubmed	23673665	Chondroadherin fragmentation mediated by the protease HTRA1 distinguishes human intervertebral disc degeneration from normal aging.	GeneRIF	0.0044	65.1	0.0114	0.116	1	1	HTRA1
Pubmed	24471736	Detection of Hb anti-Lepore Hong Kong (NG_000007.3: g.63154_70565dup) in Chinese individuals.	Pubmed	0.0044	65.1	0.0114	0.116	1	1	HBA2
Pubmed	10021330	The surface ectoderm is essential for nephric duct formation in intermediate mesoderm.	Pubmed	0.0044	65.1	0.0114	0.116	1	1	BMP4
Pubmed	20600583	Regulation of translocator protein 18 kDa (TSPO) expression in health and disease states.	GeneRIF	0.0044	65.1	0.0114	0.116	1	1	TSPO
Pubmed	10691414	Mutational analysis of the human pancreatic secretory trypsin inhibitor (PSTI) gene in hereditary and sporadic chronic pancreatitis.	Pubmed	0.0044	65.1	0.0114	0.116	1	1	SPINK1
Pubmed	15795541	Plasma levels of tissue factor and soluble E-selectin in sickle cell disease: relationship to genotype and to inflammation.	GeneRIF	0.0044	65.1	0.0114	0.116	1	1	HBB
Pubmed	23685956	P21-activated kinase 5 plays essential roles in the proliferation and tumorigenicity of human hepatocellular carcinoma.	Pubmed	0.0044	65.1	0.0114	0.116	1	1	PAK6
Pubmed	16291573	Atrial natriuretic peptide stimulates lipid mobilization during repeated bouts of endurance exercise.	GeneRIF	0.0044	65.1	0.0114	0.116	1	1	NPPA
Pubmed	22944196	Loss of SPINK1 expression is associated with unfavorable outcomes in urothelial carcinoma of the bladder after radical cystectomy.	Pubmed	0.0044	65.1	0.0114	0.116	1	1	SPINK1
Pubmed	12748861	The SLC39 family of metal ion transporters.	Pubmed	0.0044	65.1	0.0114	0.116	1	1	SLC39A2
Pubmed	19148121	gamma-Glutamyltransferase, obesity, physical activity, and the metabolic syndrome in indigenous Australian adults.	GeneRIF	0.0044	65.1	0.0114	0.116	1	1	GGT1

Category	ID	Name	Source	p-value	q-value Bonferroni	q-value FDR B&H	q-value FDR B&Y	Hit Count in Query List	Hit Count in Genome	Hit in Query List
Pubmed	16132693	Interactions between atrial natriuretic peptide and the renin-angiotensin system during salt-sensitivity exhibited by the proANP gene-disrupted mouse.	GeneRIF	0.0044	65.1	0.0114	0.116	1	1	NPPA
Pubmed	19065337	Two new hemoglobin variants: Hb Sinai-Greenspring [beta34(B16)Val-->Ile, GTC > ATC] and Hb Sinai-Bel Air [beta53(D4)Ala-->Asp, GCT > GAT].	GeneRIF	0.0044	65.1	0.0114	0.116	1	1	HBB
Pubmed	19878047	Movement of the S4 segment in the hERG potassium channel during membrane depolarization.	GeneRIF	0.0044	65.1	0.0114	0.116	1	1	KCNH2
Pubmed	18664495	The role of cathepsin X in the migration and invasiveness of T lymphocytes.	GeneRIF	0.0044	65.1	0.0114	0.116	1	1	CTSK
Pubmed	15886239	Tissue transglutaminase at embryo-maternal interface.	GeneRIF	0.0044	65.1	0.0114	0.116	1	1	TGM2
Pubmed	17570903	Soluble endothelial protein C receptor: influence on arteriovenous fistula thrombosis development in hemodialysis patients.	GeneRIF	0.0044	65.1	0.0114	0.116	1	1	PROCR
Pubmed	21693692	Knockout of the murine cysteine dioxygenase gene results in severe impairment in ability to synthesize taurine and an increased catabolism of cysteine to hydrogen sulfide.	GeneRIF	0.0044	65.1	0.0114	0.116	1	1	CDO1
Pubmed	11287206	Metallocarboxypeptidase Z is dynamically expressed in mouse development.	Pubmed	0.0044	65.1	0.0114	0.116	1	1	CPZ
Pubmed	10762318	Enlarged cholinergic forebrain neurons and improved spatial learning in p75 knockout mice.	Pubmed	0.0044	65.1	0.0114	0.116	1	1	NGFR
Pubmed	16243035	P-Rex1 regulates neutrophil function.	GeneRIF	0.0044	65.1	0.0114	0.116	1	1	PREX1
Pubmed	20113296	Identification of three novel mutations [-41 (A>C), codon 24 (-G), and IVS-I-109 (-T)], in a study of beta-thalassemia alleles in the Isfahan region of Iran.	GeneRIF	0.0044	65.1	0.0114	0.116	1	1	HBB
Pubmed	19934081	Role of CX3C-chemokine CX3C-L/fractalkine expression in a model of slowly progressive renal failure.	GeneRIF	0.0044	65.1	0.0114	0.116	1	1	CX3CL1
Pubmed	17056009	hERG K+ channel blockade by the antipsychotic drug thioridazine: An obligatory role for the S6 helix residue F656.	GeneRIF	0.0044	65.1	0.0114	0.116	1	1	KCNH2
Pubmed	23595988	Ligand-bound thyroid hormone receptor contributes to reprogramming of pancreatic acinar cells into insulin-producing cells.	GeneRIF	0.0044	65.1	0.0114	0.116	1	1	THRA
Pubmed	12562841	Lack of a direct role for macrosialin in oxidized LDL metabolism.	GeneRIF	0.0044	65.1	0.0114	0.116	1	1	CD68
Pubmed	21912055	Association of matrix metalloproteinase-7 (-181A>G) polymorphism with risk of esophageal squamous cell carcinoma in Kashmir Valley.	GeneRIF	0.0044	65.1	0.0114	0.116	1	1	MMP7
Pubmed	11856752	The lysosomal transport of prosaposin requires the conditional interaction of its highly conserved d domain with sphingomyelin.	GeneRIF	0.0044	65.1	0.0114	0.116	1	1	PSAP
Pubmed	8144352	Hb Howick [beta 37(C3)Trp-->Gly]: a new high oxygen affinity variant of the alpha 1 beta 2 contact.	Pubmed	0.0044	65.1	0.0114	0.116	1	1	HBB
Pubmed	20696380	Long-distance control of synapse assembly by target-derived NGF.	GeneRIF	0.0044	65.1	0.0114	0.116	1	1	NGFR
Pubmed	25591676	Clinical heterogeneity and a high proportion of novel mutations in a Chinese cohort of patients with dysferlinopathy.	Pubmed	0.0044	65.1	0.0114	0.116	1	1	DYSF
Pubmed	7633408	The gene for a human microfibril-associated glycoprotein is commonly deleted in Smith-Magenis syndrome patients.	Pubmed	0.0044	65.1	0.0114	0.116	1	1	MFAP4
Pubmed	24073410	Intracellular secretory leukoprotease inhibitor modulates inositol 1,4,5-triphosphate generation and exerts an anti-inflammatory effect on neutrophils of individuals with cystic fibrosis and chronic obstructive pulmonary disease.	Pubmed	0.0044	65.1	0.0114	0.116	1	1	SLPI
Pubmed	16409473	The Ser219-->Gly dimorphism of the endothelial protein C receptor contributes to the higher soluble protein levels observed in individuals with the A3 haplotype.	GeneRIF	0.0044	65.1	0.0114	0.116	1	1	PROCR
Pubmed	21522182	Muscular dystrophy with marked Dysferlin deficiency is consistently caused by primary dysferlin gene mutations.	GeneRIF	0.0044	65.1	0.0114	0.116	1	1	DYSF
Pubmed	16117792	Sustained serine proteases activity by prolonged increase in pH leads to degradation of lipid processing enzymes and profound alterations of barrier function and stratum corneum integrity.	GeneRIF	0.0044	65.1	0.0114	0.116	1	1	KLK7
Pubmed	21538840	Analysis of neurogranin (NRGN) in schizophrenia.	GeneRIF	0.0044	65.1	0.0114	0.116	1	1	NRGN
Pubmed	1247583	Hemoglobin J Cairo: beta 65 (E9) Lys leads to Gln, A new hemoglobin variant discovered in an Egyptian family.	Pubmed	0.0044	65.1	0.0114	0.116	1	1	HBB
Pubmed	9458130	Identification of a new DQB1*0613 allele in a family.	Pubmed	0.0044	65.1	0.0114	0.116	1	1	HLA-DQB1
Pubmed	24608438	The p75 neurotrophin receptor is required for the major loss of sympathetic nerves from islets under autoimmune attack.	Pubmed	0.0044	65.1	0.0114	0.116	1	1	NGFR
Pubmed	22038180	Anti-tissue transglutaminase antibodies activate intracellular tissue transglutaminase by modulating cytosolic Ca2+ homeostasis.	GeneRIF	0.0044	65.1	0.0114	0.116	1	1	TGM2
Pubmed	19208340	Role of transglutaminase 2 in liver injury via cross-linking and silencing of transcription factor Sp1.	Pubmed	0.0044	65.1	0.0114	0.116	1	1	TGM2
Pubmed	21099353	Rb-dependent cellular senescence, multinucleation and susceptibility to oncogenic transformation through PKC scaffolding by SSeCKS/AKAP12.	GeneRIF	0.0044	65.1	0.0114	0.116	1	1	AKAP12
Pubmed	17341691	Electroacupuncture suppresses myostatin gene expression: cell proliferative reaction in mouse skeletal muscle.	Pubmed	0.0044	65.1	0.0114	0.116	1	1	SEZ6L
Pubmed	11801551	Matrix metalloproteinase-7 expression in colorectal cancer liver metastases: evidence for involvement of MMP-7 activation in human cancer metastases.	GeneRIF	0.0044	65.1	0.0114	0.116	1	1	MMP7
Pubmed	11582643	[One year of neonatal screening for sickle-cell disease in Emma Children's Hospital/Academic Medical Center in Amsterdam].	GeneRIF	0.0044	65.1	0.0114	0.116	1	1	HBB

Category	ID	Name	Source	p-value	q-value Bonferroni	q-value FDR B&H	q-value FDR B&Y	Hit Count in Query List	Hit Count in Genome	Hit in Query List
Pubmed	16670323	B cells are required for generation of protective effector and memory CD4 cells in response to Pneumocystis lung infection.	Pubmed	0.0044	65.1	0.0114	0.116	1	1	HLA-DQB1
Pubmed	23440458	Class 3 inhibition of hERG K+ channel by caffeic acid phenethyl ester (CAPE) and curcumin.	Pubmed	0.0044	65.1	0.0114	0.116	1	1	KCNH2
Pubmed	23949222	KCa3.1 channels are involved in the infiltrative behavior of glioblastoma in vivo.	Pubmed	0.0044	65.1	0.0114	0.116	1	1	KCNN4
Pubmed	16887963	Cutting edge: Epstein-Barr virus transactivates the HERV-K18 superantigen by docking to the human complement receptor 2 (CD21) on primary B cells.	Pubmed	0.0044	65.1	0.0114	0.116	1	1	HLA-DQB1
Pubmed	12524687	p75NTR in the spleen: age-dependent changes, effect of NGF and 4-methylcatechol treatment, and structural changes in p75NTR-deficient mice.	Pubmed	0.0044	65.1	0.0114	0.116	1	1	NGFR
Pubmed	4129558	Hemoglobin Rush (beta 101 (g3) glutamine): a new unstable hemoglobin causing mild hemolytic anemia.	Pubmed	0.0044	65.1	0.0114	0.116	1	1	HBB
Pubmed	17476457	Mutation of junctophilin type 2 associated with hypertrophic cardiomyopathy.	GeneRIF	0.0044	65.1	0.0114	0.116	1	1	JPH2
Pubmed	21086180	Plasma lipids and lipoproteins in children and young adults with major ß-thalassemia from western Iran: influence of genotype.	GeneRIF	0.0044	65.1	0.0114	0.116	1	1	HBB
Pubmed	18096416	The molecular heterogeneity of beta-thalassemia in Greece.	GeneRIF	0.0044	65.1	0.0114	0.116	1	1	HBB
Pubmed	15482674	[Clinical phenotype genotype correlation in children with hemoglobin H disease in Zhuhai area of China].	GeneRIF	0.0044	65.1	0.0114	0.116	1	1	HBB
Pubmed	14674677	Characterization of the inhibitory effects of erythromycin and clarithromycin on the HERG potassium channel.	GeneRIF	0.0044	65.1	0.0114	0.116	1	1	KCNH2
Pubmed	12354795	Clinical pathology accreditation: standards for the medical laboratory.	Pubmed	0.0044	65.1	0.0114	0.116	1	1	LARGE
Pubmed	7641802	Expression of gamma-glutamyl transpeptidase in midgestation mouse yolk sac and mouse visceral yolk sac carcinoma cells.	Pubmed	0.0044	65.1	0.0114	0.116	1	1	GGT1
Pubmed	24057343	Congenital long QT syndrome with compound mutations in the KCNH2 gene.	Pubmed	0.0044	65.1	0.0114	0.116	1	1	KCNH2
Pubmed	3494674	DQw1 beta gene from HLA-DR2-Dw12 consists of six exons and expresses multiple DQw1 beta polypeptides through alternative splicing.	Pubmed	0.0044	65.1	0.0114	0.116	1	1	HLA-DQB1
Pubmed	24471655	A new d chain variant, Hb A2-Tunis [d46(CD5)Gly¿¿¿Glu; HBD: c.140G>A], observed in a Tunisian family in association with a compound heterozygosity for Hb C [ß6(A3)Glu¿¿¿Lys; HBB: c.19G>A] ß(0)-thalassemia [IVS-I-1 (ß143, G>A); HBB: c.92+1G>A].	Pubmed	0.0044	65.1	0.0114	0.116	1	1	HBA2
Pubmed	6316358	Exon-intron organization and complete nucleotide sequence of a human major histocompatibility antigen DC beta gene.	Pubmed	0.0044	65.1	0.0114	0.116	1	1	HLA-DQB1
Pubmed	25153388	Kallikrein-related peptidase 7 (KLK7) is a proliferative factor that is aberrantly expressed in human colon cancer.	Pubmed	0.0044	65.1	0.0114	0.116	1	1	KLK7
Pubmed	23397225	TROP2 expression and its correlation with tumor proliferation and angiogenesis in human gliomas.	Pubmed	0.0044	65.1	0.0114	0.116	1	1	TACSTD2
Pubmed	25363454	Polymorphism in IFI16 affects CD4(+) T-cell counts in HIV-1 infection.	Pubmed	0.0044	65.1	0.0114	0.116	1	1	IFI16
Pubmed	12058053	The cytoplasmic tail of invariant chain regulates endosome fusion and morphology.	GeneRIF	0.0044	65.1	0.0114	0.116	1	1	CD74
Pubmed	20018810	Phosphatidylinositol 3,4,5-triphosphate-dependent Rac exchanger 1 (P-Rex-1), a guanine nucleotide exchange factor for Rac, mediates angiogenic responses to stromal cell-derived factor-1/chemokine stromal cell derived factor-1 (SDF-1/CXCL-12) linked to Rac activation, endothelial cell migration, and in vitro angiogenesis.	GeneRIF	0.0044	65.1	0.0114	0.116	1	1	PREX1
Pubmed	15240665	Expression of the B7.1 costimulatory molecule on pancreatic beta cells abrogates the requirement for CD4 T cells in the development of type 1 diabetes.	Pubmed	0.0044	65.1	0.0114	0.116	1	1	HLA-DQB1
Pubmed	24166578	Distinct physiological and developmental properties of hippocampal CA2 subfield revealed by using anti-Purkinje cell protein 4 (PCP4) immunostaining.	Pubmed	0.0044	65.1	0.0114	0.116	1	1	PCP4
Pubmed	21483829	Trafficking defect and proteasomal degradation contribute to the phenotype of a novel KCNH2 long QT syndrome mutation.	GeneRIF	0.0044	65.1	0.0114	0.116	1	1	KCNH2
Pubmed	22563936	Molecular spectrum of ß-thalassemia mutations in the admixed Venezuelan population, and their linkage to ß-globin gene haplotypes.	GeneRIF	0.0044	65.1	0.0114	0.116	1	1	HBB
Pubmed	15572361	IFI16 is an essential mediator of growth inhibition, but not differentiation, induced by the leukemia inhibitory factor/JAK/STAT pathway in medullary thyroid carcinoma cells.	GeneRIF	0.0044	65.1	0.0114	0.116	1	1	IFI16
Pubmed	15041726	Immunohistochemical assessment of the peripheral benzodiazepine receptor in breast cancer and its relationship with survival.	GeneRIF	0.0044	65.1	0.0114	0.116	1	1	TSPO
Pubmed	18301036	HTRA1 polymorphism in dry and wet age-related macular degeneration.	GeneRIF	0.0044	65.1	0.0114	0.116	1	1	HTRA1
Pubmed	17161916	Microglia and macrophages express tumor necrosis factor receptor p75 following middle cerebral artery occlusion in mice.	GeneRIF	0.0044	65.1	0.0114	0.116	1	1	NGFR
Pubmed	12176896	Identification of the genes differentially expressed in human dendritic cell subsets by cDNA subtraction and microarray analysis.	Pubmed	0.0044	65.1	0.0114	0.116	1	1	GPNMB
Pubmed	18156604	The candidate sour taste receptor, PKD2L1, is expressed by type III taste cells in the mouse.	GeneRIF	0.0044	65.1	0.0114	0.116	1	1	PKD2L1
Pubmed	11864985	Mapping the binding site of a human ether-a-go-go-related gene-specific peptide toxin (ErgTx) to the channel's outer vestibule.	GeneRIF	0.0044	65.1	0.0114	0.116	1	1	KCNH2
Pubmed	12165499	Expression and function of transgenic HLA-DQ molecules and lymphocyte development in mice lacking invariant chain.	GeneRIF	0.0044	65.1	0.0114	0.116	1	1	CD74

Category	ID	Name	Source	p-value	q-value Bonferroni	q-value FDR B&H	q-value FDR B&Y	Hit Count in Query List	Hit Count in Genome	Hit in Query List
Pubmed	12917799	Association between diabetes type 1 and DQB1 alleles in a case-control study conducted in Montevideo, Uruguay.	GeneRIF	0.0044	65.1	0.0114	0.116	1	1	HLA-DQB1
Pubmed	23208015	Elevated serum bone morphogenetic protein 4 in patients with chronic kidney disease and coronary artery disease.	GeneRIF	0.0044	65.1	0.0114	0.116	1	1	BMP4
Pubmed	12547158	Peripheral-type benzodiazepine receptor (PBR) gene amplification in MDA-MB-231 aggressive breast cancer cells.	GeneRIF	0.0044	65.1	0.0114	0.116	1	1	TSPO
Pubmed	21077758	Hb S [ß6(A3)Glu¿Val, GAG>GTG] in Mexican Mestizos: frequency and analysis of the 5' ß-globin haplotype.	GeneRIF	0.0044	65.1	0.0114	0.116	1	1	HBB
Pubmed	6206493	Cloning and sequence analysis of the human major histocompatibility complex gene DC-3 beta.	Pubmed	0.0044	65.1	0.0114	0.116	1	1	HLA-DQB1
Pubmed	25211188	DCLK1 facilitates intestinal tumor growth via enhancing pluripotency and epithelial mesenchymal transition.	Pubmed	0.0044	65.1	0.0114	0.116	1	1	DCLK1
Pubmed	19436067	Four additional mouse crosses improve the lipid QTL landscape and identify Lipg as a QTL gene.	Pubmed	0.0044	65.1	0.0114	0.116	1	1	LIPG
Pubmed	12518053	Crystal structure of saposin B reveals a dimeric shell for lipid binding.	GeneRIF	0.0044	65.1	0.0114	0.116	1	1	PSAP
Pubmed	2907503	Nucleotide sequence and expression of the human skeletal alpha-actin gene: evolution of functional regulatory domains.	Pubmed	0.0044	65.1	0.0114	0.116	1	1	ACTA1
Pubmed	21173544	Rapid screening for Japanese dysferlinopathy by fluorescent primer extension.	GeneRIF	0.0044	65.1	0.0114	0.116	1	1	DYSF
Pubmed	22514733	BMP4 was associated with NSCL/P in an Asian population.	GeneRIF	0.0044	65.1	0.0114	0.116	1	1	BMP4
Pubmed	3117885	Recombination between DQ alpha and DQ beta genes generates human histocompatibility leukocyte antigen class II haplotype diversity.	Pubmed	0.0044	65.1	0.0114	0.116	1	1	HLA-DQB1
Pubmed	8018728	A novel serine proteinase-like sequence from human brain.	Pubmed	0.0044	65.1	0.0114	0.116	1	1	KLK15
Pubmed	9647736	Structural characterization and mapping of the normal epithelial cell-specific 1 gene.	Pubmed	0.0044	65.1	0.0114	0.116	1	1	KLK10
Pubmed	11939515	Hb Molfetta [beta126(H4)Val-->Leu, GTG-->CTG]: a new, silent, neutral beta chain variant found in an Italian woman.	Pubmed	0.0044	65.1	0.0114	0.116	1	1	HBB
Pubmed	17171640	Prosaposin is a novel androgen-regulated gene in prostate cancer cell line LNCaP.	GeneRIF	0.0044	65.1	0.0114	0.116	1	1	PSAP
Pubmed	18402547	Delivery of recombinant adeno-associated virus-mediated human tissue kallikrein for therapy of chronic renal failure in rats.	GeneRIF	0.0044	65.1	0.0114	0.116	1	1	KLK1
Pubmed	22732722	Effects of 18-kDa translocator protein knockdown on gene expression of glutamate receptors, transporters, and metabolism, and on cell viability affected by glutamate.	GeneRIF	0.0044	65.1	0.0114	0.116	1	1	TSPO
Pubmed	16823394	Detection of a large genomic deletion in the pancreatic secretory trypsin inhibitor (SPINK1) gene.	Pubmed	0.0044	65.1	0.0114	0.116	1	1	SPINK1
Pubmed	20858281	Trop2 expression contributes to tumor pathogenesis by activating the ERK MAPK pathway.	GeneRIF	0.0044	65.1	0.0114	0.116	1	1	TACSTD2
Pubmed	21802407	Glutathione is essential for early embryogenesis--analysis of a glutathione synthetase knockout mouse.	Pubmed	0.0044	65.1	0.0114	0.116	1	1	GSS
Pubmed	17683065	Cathepsins and osteosarcoma: Expression analysis identifies cathepsin K as an indicator of metastasis.	GeneRIF	0.0044	65.1	0.0114	0.116	1	1	CTSK
Pubmed	11939513	Hb G-San Josè variant levels correlate with alpha-thalassemia genotypes.	GeneRIF	0.0044	65.1	0.0114	0.116	1	1	HBB
Pubmed	16166152	Tryptophan scanning mutagenesis of the HERG K+ channel: the S4 domain is loosely packed and likely to be lipid exposed.	GeneRIF	0.0044	65.1	0.0114	0.116	1	1	KCNH2
Pubmed	15468058	A mouse model of human adaptive immune functions: HLA-A2.1-/HLA-DR1-transgenic H-2 class I-/class II-knockout mice.	Pubmed	0.0044	65.1	0.0114	0.116	1	1	HLA-DQB1
Pubmed	24516604	HERG K+ channel-dependent apoptosis and cell cycle arrest in human glioblastoma cells.	Pubmed	0.0044	65.1	0.0114	0.116	1	1	KCNH2
Pubmed	24696486	Innate nuclear sensor IFI16 translocates into the cytoplasm during the early stage of in vitro human cytomegalovirus infection and is entrapped in the egressing virions during the late stage.	Pubmed	0.0044	65.1	0.0114	0.116	1	1	IFI16
Pubmed	19296056	Soluble endothelial protein C receptor levels in healthy population.	GeneRIF	0.0044	65.1	0.0114	0.116	1	1	PROCR
Pubmed	19937343	Tissue transglutaminase 2 as a biomarker of cervical intraepithelial neoplasia (CIN) and its relationship to p16INK4A and nuclear factor kappaB expression.	GeneRIF	0.0044	65.1	0.0114	0.116	1	1	TGM2
Pubmed	18334539	Matrix metalloproteinase-7 (matrilysin) controls neutrophil egress by generating chemokine gradients.	GeneRIF	0.0044	65.1	0.0114	0.116	1	1	MMP7
Pubmed	10416690	Synthesis of steroids in pancreas: evidence of cytochrome P-450scc activity.	Pubmed	0.0044	65.1	0.0114	0.116	1	1	CYP11A1
Pubmed	10706139	BMP-4 affects the differentiation of metanephric mesenchyme and reveals an early anterior-posterior axis of the embryonic kidney.	Pubmed	0.0044	65.1	0.0114	0.116	1	1	BMP4
Pubmed	8641705	Hb Costa Rica or alpha 2 beta 2 77(EF1)His --> Arg: the first example of a somatic cell mutation in a globin gene.	Pubmed	0.0044	65.1	0.0114	0.116	1	1	HBB
Pubmed	15973412	Abnormal display of PfEMP-1 on erythrocytes carrying haemoglobin C may protect against malaria.	GeneRIF	0.0044	65.1	0.0114	0.116	1	1	HBB
Pubmed	15295654	Mutations in the membrane component, chromosome 1, surface marker 1 (M1S1) gene in gelatinous drop-like corneal dystrophy.	GeneRIF	0.0044	65.1	0.0114	0.116	1	1	TACSTD2
Pubmed	24711452	Overexpression of smooth muscle myosin heavy chain leads to activation of the unfolded protein response and autophagic turnover of thick filament-associated proteins in vascular smooth muscle cells.	Pubmed	0.0044	65.1	0.0114	0.116	1	1	MYH11
Pubmed	2925231	Sequence analysis of HLA class II domains: characterization of the DQw3 family of DQB genes.	Pubmed	0.0044	65.1	0.0114	0.116	1	1	HLA-DQB1
Pubmed	21964441	Endogenous Bmp4 in myoblasts is required for myotube formation in C2C12 cells.	GeneRIF	0.0044	65.1	0.0114	0.116	1	1	BMP4

Category	ID	Name	Source	p-value	q-value Bonferroni	q-value FDR B&H	q-value FDR B&Y	Hit Count in Query List	Hit Count in Genome	Hit in Query List
Pubmed	20931094	Novel characteristics of a trafficking-defective G572R-hERG channel linked to hereditary long QT syndrome.	GeneRIF	0.0044	65.1	0.0114	0.116	1	1	KCNH2
Pubmed	22101894	Induction of acute GVHD by sex-mismatched H-Y antigens in the absence of functional radiosensitive host hematopoietic-derived antigen-presenting cells.	Pubmed	0.0044	65.1	0.0114	0.116	1	1	HLA-DQB1
Pubmed	17321687	The drug binding site of human alpha1-acid glycoprotein: insight from induced circular dichroism and electronic absorption spectra.	GeneRIF	0.0044	65.1	0.0114	0.116	1	1	ORM1
Pubmed	20230523	The novel allele HLA-DQB1*0636 of Caucasian origin has a unique amino acid exchange at position 186 of the beta 2 region.	GeneRIF	0.0044	65.1	0.0114	0.116	1	1	HLA-DQB1
Pubmed	20923576	Association of the LIPG 584C > T polymorphism and serum lipid levels in the Guangxi Bai Ku Yao and Han populations.	GeneRIF	0.0044	65.1	0.0114	0.116	1	1	LIPG
Pubmed	12369832	A positive charge preservation at position 116 of alpha A-crystallin is critical for its structural and functional integrity.	GeneRIF	0.0044	65.1	0.0114	0.116	1	1	CRYAA
Pubmed	16272359	Inhibition of CX3CL1 (fractalkine) improves experimental autoimmune myositis in SJL/J mice.	Pubmed	0.0044	65.1	0.0114	0.116	1	1	CX3CL1
Pubmed	12170471	[Association of HLA-DQB1 alleles and dermatomyositis/ polymyositis].	GeneRIF	0.0044	65.1	0.0114	0.116	1	1	HLA-DQB1
Pubmed	11969404	Cation- and peptide-binding properties of human calmodulin-like skin protein.	GeneRIF	0.0044	65.1	0.0114	0.116	1	1	CALML5
Pubmed	22672614	Thalassemia major phenotypes secondary to the association of ß 5'UTR +20(C ¿ T) allele with ß 39(C ¿ T).	GeneRIF	0.0044	65.1	0.0114	0.116	1	1	HBB
Pubmed	15929988	Proteolytic excision of a repressive loop domain in tartrate-resistant acid phosphatase by cathepsin K in osteoclasts.	GeneRIF	0.0044	65.1	0.0114	0.116	1	1	CTSK
Pubmed	8565332	Expression of SGP-1 mRNA in preimplantation mouse embryos.	Pubmed	0.0044	65.1	0.0114	0.116	1	1	PSAP
Pubmed	19187913	A KCNH2 branch point mutation causing aberrant splicing contributes to an explanation of genotype-negative long QT syndrome.	GeneRIF	0.0044	65.1	0.0114	0.116	1	1	KCNH2
Pubmed	16432067	Most LQT2 mutations reduce Kv11.1 (hERG) current by a class 2 (trafficking-deficient) mechanism.	GeneRIF	0.0044	65.1	0.0114	0.116	1	1	KCNH2
Pubmed	11333380	Nemaline myopathy caused by mutations in the muscle alpha-skeletal-actin gene.	Pubmed	0.0044	65.1	0.0114	0.116	1	1	ACTA1
Pubmed	25268796	A new ß(0) frameshift mutation, HBB: c.44delT (p.Leu14ArgfsX5), identified in an Argentinean family associated with secondary genetic modifiers of ß-thalassemia.	Pubmed	0.0044	65.1	0.0114	0.116	1	1	HBB
Pubmed	909779	Nucleotide sequence of 3' untranslated portion of human alpha globin mRNA.	Pubmed	0.0044	65.1	0.0114	0.116	1	1	HBA2
Pubmed	23168573	Glomerular expression of fractalkine is induced by polyinosinic-polycytidylic acid in human mesangial cells: possible involvement of fractalkine after viral infection.	GeneRIF	0.0044	65.1	0.0114	0.116	1	1	CX3CL1
Pubmed	21852675	Dysregulation of placental endothelial lipase in obese women with gestational diabetes mellitus.	GeneRIF	0.0044	65.1	0.0114	0.116	1	1	LIPG
Pubmed	19057528	Relationship of serum gamma-glutamyltransferase to atherogenic dyslipidemia and glycemic control in type 2 diabetes.	GeneRIF	0.0044	65.1	0.0114	0.116	1	1	GGT1
Pubmed	11023977	A role for p75 neurotrophin receptor in the control of apoptosis-driven hair follicle regression.	Pubmed	0.0044	65.1	0.0114	0.116	1	1	NGFR
Pubmed	17495604	Endothelial lipase and the metabolic syndrome.	GeneRIF	0.0044	65.1	0.0114	0.116	1	1	LIPG
Pubmed	25002588	Interferon ¿-inducible protein (IFI) 16 transcriptionally regulates type i interferons and other interferon-stimulated genes and controls the interferon response to both DNA and RNA viruses.	Pubmed	0.0044	65.1	0.0114	0.116	1	1	IFI16
Pubmed	12042278	Detection of fractalkine in human seminal plasma and its role in infertile patients.	GeneRIF	0.0044	65.1	0.0114	0.116	1	1	CX3CL1
Pubmed	17640799	Bone morphogenetic protein-4 inhibits heat-induced apoptosis by modulating MAPK pathways in human colon cancer HCT116 cells.	GeneRIF	0.0044	65.1	0.0114	0.116	1	1	BMP4
Pubmed	19360854	Enhanced spatial memory and hippocampal long-term potentiation in p75 neurotrophin receptor knockout mice.	GeneRIF	0.0044	65.1	0.0114	0.116	1	1	NGFR
Pubmed	24400717	Gain-of-function KCNH2 mutations in patients with Brugada syndrome.	Pubmed	0.0044	65.1	0.0114	0.116	1	1	KCNH2
Pubmed	16288871	The differential gene expression profiles of proximal and distal muscle groups are altered in pre-pathological dysferlin-deficient mice.	Pubmed	0.0044	65.1	0.0114	0.116	1	1	DYSF
Pubmed	19439805	Comparative effects of the short QT N588K mutation at 37 degrees C on hERG K+ channel current during ventricular, Purkinje fibre and atrial action potentials: an action potential clamp study.	GeneRIF	0.0044	65.1	0.0114	0.116	1	1	KCNH2
Pubmed	24892449	Endothelial CD276 (B7-H3) expression is increased in human malignancies and distinguishes between normal and tumour-derived circulating endothelial cells.	Pubmed	0.0044	65.1	0.0114	0.116	1	1	CD276
Pubmed	24373430	The emerging physiological roles of the glycerophosphodiesterase family.	Pubmed	0.0044	65.1	0.0114	0.116	1	1	GDPD2
Pubmed	24127121	Piperlonguminine downregulates endothelial protein C receptor shedding in vitro and in vivo.	Pubmed	0.0044	65.1	0.0114	0.116	1	1	PROCR
Pubmed	14696968	Identification and characterization of the genes encoding human and mouse osteoactivin.	GeneRIF	0.0044	65.1	0.0114	0.116	1	1	GPNMB
Pubmed	19929564	Matrix metalloproteinase (MMP)-7 in salivary gland cancer.	GeneRIF	0.0044	65.1	0.0114	0.116	1	1	MMP7
Pubmed	10506947	Enzymes involved in purine metabolism--a review of histochemical localization and functional implications.	Pubmed	0.0044	65.1	0.0114	0.116	1	1	ADA
Pubmed	24132234	LARGE glycans on dystroglycan function as a tunable matrix scaffold to prevent dystrophy.	Pubmed	0.0044	65.1	0.0114	0.116	1	1	LARGE
Pubmed	18813308	High expression of TROP2 correlates with poor prognosis in pancreatic cancer.	GeneRIF	0.0044	65.1	0.0114	0.116	1	1	TACSTD2
Pubmed	24040148	Depletion of the p43 mitochondrial T3 receptor increases Sertoli cell proliferation in mice.	Pubmed	0.0044	65.1	0.0114	0.116	1	1	THRA

Category	ID	Name	Source	p-value	q-value Bonferroni	q-value FDR B&H	q-value FDR B&Y	Hit Count in Query List	Hit Count in Genome	Hit in Query List
Pubmed	19533614	Beta-globin gene cluster haplotypes in Afro-Uruguayans from two geographical regions (South and North).	GeneRIF	0.0044	65.1	0.0114	0.116	1	1	HBB
Pubmed	16723379	Merg1a K+ channel induces skeletal muscle atrophy by activating the ubiquitin proteasome pathway.	GeneRIF	0.0044	65.1	0.0114	0.116	1	1	KCNH2
Pubmed	21936829	SLPI and elafin: multifunctional antiproteases of the WFDC family.	Pubmed	0.0044	65.1	0.0114	0.116	1	1	SLPI
Pubmed	15150078	Autoantibodies against EPCR are found in antiphospholipid syndrome and are a risk factor for fetal death.	GeneRIF	0.0044	65.1	0.0114	0.116	1	1	PROCR
Pubmed	7853474	Host response to Sendai virus in mice lacking class II major histocompatibility complex glycoproteins.	Pubmed	0.0044	65.1	0.0114	0.116	1	1	HLA-DQB1
Pubmed	9039268	Secretory leukocyte protease inhibitor: a macrophage product induced by and antagonistic to bacterial lipopolysaccharide.	Pubmed	0.0044	65.1	0.0114	0.116	1	1	SLPI
Pubmed	16355004	Adenoviral E1A suppresses secretory leukoprotease inhibitor and elafin secretion in human alveolar epithelial cells and bronchial epithelial cells.	GeneRIF	0.0044	65.1	0.0114	0.116	1	1	SLPI
Pubmed	15558233	The p75 neurotrophin receptor appears in plasma in diabetic rats-characterisation of a potential early test for neuropathy.	GeneRIF	0.0044	65.1	0.0114	0.116	1	1	NGFR
Pubmed	9425238	The human FXY gene is located within Xp22.3: implications for evolution of the mammalian X chromosome.	Pubmed	0.0044	65.1	0.0114	0.116	1	1	MID1
Pubmed	14716297	A candidate tumor suppressor HtrA1 is downregulated in ovarian cancer.	GeneRIF	0.0044	65.1	0.0114	0.116	1	1	HTRA1
Pubmed	18472023	RPA nucleic acid-binding properties of IFI16-HIN200.	GeneRIF	0.0044	65.1	0.0114	0.116	1	1	IFI16
Pubmed	17925082	Neonatal screening for sickle cell disease in Central Africa: a study of 1825 newborns with a new enzyme-linked immunosorbent assay test.	GeneRIF	0.0044	65.1	0.0114	0.116	1	1	HBB
Pubmed	25190814	Physical and functional interactions between the histone H3K4 demethylase KDM5A and the nucleosome remodeling and deacetylase (NuRD) complex.	Pubmed	0.0044	65.1	0.0114	0.116	1	1	KDM5A
Pubmed	14649315	Hb Buzen [beta138(H16)Ala --> Thr (g.1395 G -->A)]: a new beta chain variant.	GeneRIF	0.0044	65.1	0.0114	0.116	1	1	HBB
Pubmed	12005022	Peptidases play an important role in cataractogenesis: an immunohistochemical study on lenses derived from Shumiya cataract rats.	Pubmed	0.0044	65.1	0.0114	0.116	1	1	DPP7
Pubmed	19850887	Hyperoxia-induced lung injury in gamma-glutamyl transferase deficiency is associated with alterations in nitrosative and nitrative stress.	Pubmed	0.0044	65.1	0.0114	0.116	1	1	GGT1
Pubmed	8864828	Cloning of candidate autoantigen carboxypeptidase H from a human islet library: sequence identity with human brain CPH.	Pubmed	0.0044	65.1	0.0114	0.116	1	1	CPE
Pubmed	9427755	APM-1, a novel human gene, identified by aberrant co-transcription with papillomavirus oncogenes in a cervical carcinoma cell line, encodes a BTB/POZ-zinc finger protein with growth inhibitory activity.	Pubmed	0.0044	65.1	0.0114	0.116	1	1	ZBTB7C
Pubmed	21734253	Functional ADA polymorphism increases sleep depth and reduces vigilant attention in humans.	GeneRIF	0.0044	65.1	0.0114	0.116	1	1	ADA
Pubmed	17487393	Activation of human natural killer cells by recombinant membrane-expressed fractalkine on the surface of tumor cells.	GeneRIF	0.0044	65.1	0.0114	0.116	1	1	CX3CL1
Pubmed	19951916	The glycosaminoglycan-binding domain of PRELP acts as a cell type-specific NF-kappaB inhibitor that impairs osteoclastogenesis.	GeneRIF	0.0044	65.1	0.0114	0.116	1	1	PRELP
Pubmed	21129264	[Single nucleotide polymorphisms of ß-globin gene in ß-thalassaemia patients].	GeneRIF	0.0044	65.1	0.0114	0.116	1	1	HBB
Pubmed	23477808	Elevated plasma fractalkine levels are associated with higher levels of IL-6, Apo-B, LDL-C and insulin, but not with body composition in a large female twin sample.	Pubmed	0.0044	65.1	0.0114	0.116	1	1	CX3CL1
Pubmed	23382562	CD4+ T cell persistence and function after infection are maintained by low-level peptide:MHC class II presentation.	GeneRIF	0.0044	65.1	0.0114	0.116	1	1	HLA-DQB1
Pubmed	12163480	Rabies virus glycoprotein (RVG) is a trimeric ligand for the N-terminal cysteine-rich domain of the mammalian p75 neurotrophin receptor.	GeneRIF	0.0044	65.1	0.0114	0.116	1	1	NGFR
Pubmed	18193088	Suppression of PPN/MG61 attenuates Wnt/beta-catenin signaling pathway and induces apoptosis in human lung cancer.	GeneRIF	0.0044	65.1	0.0114	0.116	1	1	PORCN
Pubmed	21851375	The neurotrophin receptor p75NTR is induced on mature myofibres in inflammatory myopathies and promotes myotube survival to inflammatory stress.	GeneRIF	0.0044	65.1	0.0114	0.116	1	1	NGFR
Pubmed	21211543	Kallikrein 1 is overexpressed by astrocytes in the hippocampus of patients with refractory temporal lobe epilepsy, associated with hippocampal sclerosis.	GeneRIF	0.0044	65.1	0.0114	0.116	1	1	KLK1
Pubmed	23637347	The atrial natriuretic peptide genetic variant rs5068 is associated with a favorable cardiometabolic phenotype in a Mediterranean population.	Pubmed	0.0044	65.1	0.0114	0.116	1	1	NPPA
Pubmed	21300147	Identification and characterization of a copper-binding site in aA-crystallin.	GeneRIF	0.0044	65.1	0.0114	0.116	1	1	CRYAA
Pubmed	22178075	Chromatin structure of the LCR in the human ß-globin locus transcribing the adult d- and ß-globin genes.	GeneRIF	0.0044	65.1	0.0114	0.116	1	1	HBB
Pubmed	23581230	Inhibition of cellular growth and migration by suppression of endothelial protein C receptor (EPCR) in lung carcinoma cells.	GeneRIF	0.0044	65.1	0.0114	0.116	1	1	PROCR
Pubmed	11805156	Tissue and cellular localization of a novel polycystic kidney disease-like gene product, polycystin-L.	Pubmed	0.0044	65.1	0.0114	0.116	1	1	PKD2L1
Pubmed	11864433	[Study on the association of pathologic myopia with HLA-DQB1 gene].	GeneRIF	0.0044	65.1	0.0114	0.116	1	1	HLA-DQB1
Pubmed	1195378	Crystal structure of sickle-cell deoxyhemoglobin at 5 A resolution.	Pubmed	0.0044	65.1	0.0114	0.116	1	1	HBB
Pubmed	10196375	Dysferlin is a plasma membrane protein and is expressed early in human development.	Pubmed	0.0044	65.1	0.0114	0.116	1	1	DYSF

Category	ID	Name	Source	p-value	q-value Bonferroni	q-value FDR B&H	q-value FDR B&Y	Hit Count in Query List	Hit Count in Genome	Hit in Query List
Pubmed	15379570	Peripheral benzodiazepine receptor antisense knockout increases tumorigenicity of MA-10 Leydig cells in vivo and in vitro.	GeneRIF	0.0044	65.1	0.0114	0.116	1	1	TSPO
Pubmed	19756726	Tissue transglutaminase expression in celiac mucosa: an immunohistochemical study.	GeneRIF	0.0044	65.1	0.0114	0.116	1	1	TGM2
Pubmed	20113286	Hb Den Haag [beta45(CD4)Phe-->Tyr]. A new hemoglobin variant observed during early pregnancy diagnostics.	GeneRIF	0.0044	65.1	0.0114	0.116	1	1	HBB
Pubmed	21593382	High levels of adenosine deaminase on dendritic cells promote autoreactive T cell activation and diabetes in nonobese diabetic mice.	GeneRIF	0.0044	65.1	0.0114	0.116	1	1	ADA
Pubmed	8393902	Purification and preliminary characterization of stratum corneum chymotryptic enzyme: a proteinase that may be involved in desquamation.	Pubmed	0.0044	65.1	0.0114	0.116	1	1	KLK7
Pubmed	7756291	Matrilysin-inhibitor complexes: common themes among metalloproteases.	Pubmed	0.0044	65.1	0.0114	0.116	1	1	MMP7
Pubmed	15364333	An intronic mutation causes long QT syndrome.	GeneRIF	0.0044	65.1	0.0114	0.116	1	1	KCNH2
Pubmed	7685034	The structure of human trichohyalin. Potential multiple roles as a functional EF-hand-like calcium-binding protein, a cornified cell envelope precursor, and an intermediate filament-associated (cross-linking) protein.	Pubmed	0.0044	65.1	0.0114	0.116	1	1	TCHH
Pubmed	18838556	Rab-coupling protein coordinates recycling of alpha5beta1 integrin and EGFR1 to promote cell migration in 3D microenvironments.	GeneRIF	0.0044	65.1	0.0114	0.116	1	1	RAB11FIP1
Pubmed	19821083	Effect of Gambogic acid on the regulation of hERG channel in K562 cells in vitro.	GeneRIF	0.0044	65.1	0.0114	0.116	1	1	KCNH2
Pubmed	12694205	Bmp4 gene is expressed at the putative site of fusion in the midfacial region.	GeneRIF	0.0044	65.1	0.0114	0.116	1	1	BMP4
Pubmed	7547694	Transgenic mice expressing MHC class II molecules with truncated A beta cytoplasmic domains reveal signaling-independent defects in antigen presentation.	Pubmed	0.0044	65.1	0.0114	0.116	1	1	HLA-DQB1
Pubmed	19598241	Carboxypeptidase E knockout mice exhibit abnormal dendritic arborization and spine morphology in central nervous system neurons.	GeneRIF	0.0044	65.1	0.0114	0.116	1	1	CPE
Pubmed	23418362	The sorting of proglucagon to secretory granules is mediated by carboxypeptidase E and intrinsic sorting signals.	GeneRIF	0.0044	65.1	0.0114	0.116	1	1	CPE
Pubmed	17508724	Erythroid-specific expression of beta-globin by the sleeping beauty transposon for Sickle cell disease.	GeneRIF	0.0044	65.1	0.0114	0.116	1	1	HBB
Pubmed	11870871	Expression of fractalkine (CX3CL1) and its receptor, CX3CR1, during acute and chronic inflammation in the rodent CNS.	GeneRIF	0.0044	65.1	0.0114	0.116	1	1	CX3CL1
Pubmed	8064228	The CLIP region of invariant chain plays a critical role in regulating major histocompatibility complex class II folding, transport, and peptide occupancy.	Pubmed	0.0044	65.1	0.0114	0.116	1	1	CD74
Pubmed	15327529	Beta-thalassaemia-87 C-->G: relationship of the Hb F modulation and polymorphisms in compound heterozygous patients.	GeneRIF	0.0044	65.1	0.0114	0.116	1	1	HBB
Pubmed	20200508	Adenosine A1 receptors and microglial cells mediate CX3CL1-induced protection of hippocampal neurons against Glu-induced death.	GeneRIF	0.0044	65.1	0.0114	0.116	1	1	CX3CL1
Pubmed	11983918	Gene expression analysis reveals matrilysin as a key regulator of pulmonary fibrosis in mice and humans.	GeneRIF	0.0044	65.1	0.0114	0.116	1	1	MMP7
Pubmed	22905401	Dietary regulation of adenosine deaminase activity in stomach, small intestine and spleen of mice.	GeneRIF	0.0044	65.1	0.0114	0.116	1	1	ADA
Pubmed	11820772	Calmodulin and cyclin D anchoring sites on the Src-suppressed C kinase substrate, SSeCKS.	GeneRIF	0.0044	65.1	0.0114	0.116	1	1	AKAP12
Pubmed	7775425	Identification of a sixth promoter that directs the transcription of gamma-glutamyl transpeptidase type III RNA in mouse.	Pubmed	0.0044	65.1	0.0114	0.116	1	1	GGT1
Pubmed	9405598	Design of potent and selective human cathepsin K inhibitors that span the active site.	Pubmed	0.0044	65.1	0.0114	0.116	1	1	CTSK
Pubmed	12372799	Regulation of arterial tone by smooth muscle myosin type II.	Pubmed	0.0044	65.1	0.0114	0.116	1	1	MYH11
Pubmed	21533074	GWAS of follicular lymphoma reveals allelic heterogeneity at 6p21.32 and suggests shared genetic susceptibility with diffuse large B-cell lymphoma.	Pubmed	0.0044	65.1	0.0114	0.116	1	1	HLA-DQB1
Pubmed	12672029	The p75(NTR) tumor suppressor induces caspase-mediated apoptosis in bladder tumor cells.	GeneRIF	0.0044	65.1	0.0114	0.116	1	1	NGFR
Pubmed	7602333	Phenotypic and pathologic evaluation of the myd mouse. A candidate model for facioscapulohumeral dystrophy.	Pubmed	0.0044	65.1	0.0114	0.116	1	1	LARGE
Pubmed	25705881	X-linked macrocytic dyserythropoietic anemia in females with an ALAS2 mutation.	Pubmed	0.0044	65.1	0.0114	0.116	1	1	ALAS2
Pubmed	21565626	Bone morphogenetic protein-4-induced epithelial-mesenchymal transition and invasiveness through Smad1-mediated signal pathway in squamous cell carcinoma of the head and neck.	GeneRIF	0.0044	65.1	0.0114	0.116	1	1	BMP4
Pubmed	19000448	[Effects of tamoxifen on apoptosis and matrix metalloproteinase-7 expression in estrogen receptor beta-positive colonic cancer cell line HT-29].	GeneRIF	0.0044	65.1	0.0114	0.116	1	1	MMP7
Pubmed	8125256	Activin/inhibin beta B subunit gene disruption leads to defects in eyelid development and female reproduction.	Pubmed	0.0044	65.1	0.0114	0.116	1	1	INHBB
Pubmed	22892349	Increased binding of peripheral benzodiazepine receptor in mild cognitive impairment-dementia converters measured by positron emission tomography with [¹¹C]DAA1106.	GeneRIF	0.0044	65.1	0.0114	0.116	1	1	TSPO
Pubmed	22036600	Familial aggregation of narcolepsy.	GeneRIF	0.0044	65.1	0.0114	0.116	1	1	HLA-DQB1
Pubmed	14751775	Suppression of neurite outgrowth by high-dose nerve growth factor is independent of functional p75NTR receptors.	GeneRIF	0.0044	65.1	0.0114	0.116	1	1	NGFR
Pubmed	16100039	Bone morphogenetic protein 4 promotes pulmonary vascular remodeling in hypoxic pulmonary hypertension.	Pubmed	0.0044	65.1	0.0114	0.116	1	1	BMP4

Category	ID	Name	Source	p-value	q-value Bonferroni	q-value FDR B&H	q-value FDR B&Y	Hit Count in Query List	Hit Count in Genome	Hit in Query List
Pubmed	21810858	Dynamic expression of bone morphogenetic protein 4 in reproductive organs of female mice.	GeneRIF	0.0044	65.1	0.0114	0.116	1	1	BMP4
Pubmed	9806887	Characterization of the human proline/arginine-rich end leucine-rich repeat protein (PRELP) gene promoter and identification of a repressor element.	Pubmed	0.0044	65.1	0.0114	0.116	1	1	PRELP
Pubmed	15013888	Compound heterozygous mutations of M1S1 gene in gelatinous droplike corneal dystrophy.	GeneRIF	0.0044	65.1	0.0114	0.116	1	1	TACSTD2
Pubmed	9478961	Adenosine deaminase-deficient mice generated using a two-stage genetic engineering strategy exhibit a combined immunodeficiency.	Pubmed	0.0044	65.1	0.0114	0.116	1	1	ADA
Pubmed	15221058	[Dysferlinopathy as an extrahepatic cause for the elevation of serum transaminases].	GeneRIF	0.0044	65.1	0.0114	0.116	1	1	DYSF
Pubmed	22952628	Structures of human DPP7 reveal the molecular basis of specific inhibition and the architectural diversity of proline-specific peptidases.	GeneRIF	0.0044	65.1	0.0114	0.116	1	1	DPP7
Pubmed	18397319	AKAP12 in astrocytes induces barrier functions in human endothelial cells through protein kinase Czeta.	GeneRIF	0.0044	65.1	0.0114	0.116	1	1	AKAP12
Pubmed	21653323	ALAS2 acts as a modifier gene in patients with congenital erythropoietic porphyria.	GeneRIF	0.0044	65.1	0.0114	0.116	1	1	ALAS2
Pubmed	9655350	In vivo acetylation identified at lysine 70 of human lens alphaA-crystallin.	Pubmed	0.0044	65.1	0.0114	0.116	1	1	CRYAA
Pubmed	24469951	Matrix metalloproteinase 7 is a useful marker for 5 fluorouracil-based adjuvant chemotherapy in stage II and stage III colorectal cancer patients.	Pubmed	0.0044	65.1	0.0114	0.116	1	1	MMP7
Pubmed	21203347	C57BL/6J, DBA/2J, and DBA/2J.Gpnmb mice have different visual signal processing in the inner retina.	Pubmed	0.0044	65.1	0.0114	0.116	1	1	GPNMB
Pubmed	8105889	Keratinocyte transglutaminase membrane anchorage: analysis of site-directed mutants.	Pubmed	0.0044	65.1	0.0114	0.116	1	1	TGM2
Pubmed	20363888	Regulation of the voltage-insensitive step of HERG activation by extracellular pH.	GeneRIF	0.0044	65.1	0.0114	0.116	1	1	KCNH2
Pubmed	12540484	Pan-neurotrophin receptor p75 contributes to neuronal hyperreactivity and airway inflammation in a murine model of experimental asthma.	Pubmed	0.0044	65.1	0.0114	0.116	1	1	NGFR
Pubmed	20574821	Role of ERG1 isoforms in modulation of ERG1 channel trafficking and function.	GeneRIF	0.0044	65.1	0.0114	0.116	1	1	KCNH2
Pubmed	11345587	Distribution of HERV-LTR elements in the 5'-flanking region of HLA-DQB1 and association with autoimmunity.	GeneRIF	0.0044	65.1	0.0114	0.116	1	1	HLA-DQB1
Pubmed	24376841	RBP2 induces epithelial-mesenchymal transition in non-small cell lung cancer.	Pubmed	0.0044	65.1	0.0114	0.116	1	1	KDM5A
Pubmed	18329694	Expression of matrix metalloproteinase 7 is an unfavorable postoperative prognostic factor in cholangiocarcinoma of the perihilar, hilar, and extrahepatic bile ducts.	GeneRIF	0.0044	65.1	0.0114	0.116	1	1	MMP7
Pubmed	11741928	The binding site for channel blockers that rescue misprocessed human long QT syndrome type 2 ether-a-gogo-related gene (HERG) mutations.	GeneRIF	0.0044	65.1	0.0114	0.116	1	1	KCNH2
Pubmed	20596752	The transglutaminase 2 gene is aberrantly hypermethylated in glioma.	GeneRIF	0.0044	65.1	0.0114	0.116	1	1	TGM2
Pubmed	19577877	HERG1 gene expression as a specific tumor marker in colorectal tissues.	GeneRIF	0.0044	65.1	0.0114	0.116	1	1	KCNH2
Pubmed	17937626	Glucocorticoid receptor-mediated expression of kallikrein 10 in human breast cancer cell lines.	GeneRIF	0.0044	65.1	0.0114	0.116	1	1	KLK10
Pubmed	21792917	Expression and regulation of B7-H3 immunoregulatory receptor, in human mesothelial and mesothelioma cells: immunotherapeutic implications.	GeneRIF	0.0044	65.1	0.0114	0.116	1	1	CD276
Pubmed	24644288	Impaired oxidative endoplasmic reticulum stress response caused by deficiency of thyroid hormone receptor a.	Pubmed	0.0044	65.1	0.0114	0.116	1	1	THRA
Pubmed	12804575	Characterisation of recombinant HERG K+ channel blockade by the Class Ia antiarrhythmic drug procainamide.	GeneRIF	0.0044	65.1	0.0114	0.116	1	1	KCNH2
Pubmed	24998961	Stress sensitivity in patients with atopic dermatitis in relation to the translocator protein 18 kDa (TSPO).	Pubmed	0.0044	65.1	0.0114	0.116	1	1	TSPO
Pubmed	22285440	Screening for mutations of the FOXO4 gene in premature ovarian failure patients.	GeneRIF	0.0044	65.1	0.0114	0.116	1	1	FOXO4
Pubmed	21820334	Polarized secretion of lysosomes at the B cell synapse couples antigen extraction to processing and presentation.	Pubmed	0.0044	65.1	0.0114	0.116	1	1	HLA-DQB1
Pubmed	9010221	Cloning and characterization of AFX, the gene that fuses to MLL in acute leukemias with a t(X;11)(q13;q23).	Pubmed	0.0044	65.1	0.0114	0.116	1	1	FOXO4
Pubmed	11121182	Adenosine deaminase alleles and autistic disorder: case-control and family-based association studies.	GeneRIF	0.0044	65.1	0.0114	0.116	1	1	ADA
Pubmed	25329094	Isoflurane but not sevoflurane or desflurane aggravates injury to neurons in vitro and in vivo via p75NTR-NF-¿B activation.	Pubmed	0.0044	65.1	0.0114	0.116	1	1	NGFR
Pubmed	7680921	Molecular cloning of CD68, a human macrophage marker related to lysosomal glycoproteins.	Pubmed	0.0044	65.1	0.0114	0.116	1	1	CD68
Pubmed	19222470	Lack of association of soluble endothelial protein C receptor and PROCR 6936A/G polymorphism with the risk of venous thromboembolism in a prospective study.	GeneRIF	0.0044	65.1	0.0114	0.116	1	1	PROCR
Pubmed	19688690	Expression pattern alterations of the serine protease HtrA1 in normal human placental tissues and in gestational trophoblastic diseases.	GeneRIF	0.0044	65.1	0.0114	0.116	1	1	HTRA1
Pubmed	19267410	Prosaposin deficiency and saposin B deficiency (activator-deficient metachromatic leukodystrophy): report on two patients detected by analysis of urinary sphingolipids and carrying novel PSAP gene mutations.	GeneRIF	0.0044	65.1	0.0114	0.116	1	1	PSAP
Pubmed	11307949	Expression and properties of recombinant HbA2 (alpha2delta2) and hybrids containing delta-beta sequences.	Pubmed	0.0044	65.1	0.0114	0.116	1	1	HBA2

Category	ID	Name	Source	p-value	q-value Bonferroni	q-value FDR B&H	q-value FDR B&Y	Hit Count in Query List	Hit Count in Genome	Hit in Query List
Pubmed	12882871	Phenotypic heterogeneity and associations of two aldose reductase gene polymorphisms with nephropathy and retinopathy in type 2 diabetes.	GeneRIF	0.0044	65.1	0.0114	0.116	1	1	AKR1B10
Pubmed	22041280	Roles of serine protease inhibitor Kazal type 1 (SPINK1) in pancreatic diseases.	GeneRIF	0.0044	65.1	0.0114	0.116	1	1	SPINK1
Pubmed	14993255	MHC class II expression restricted to CD8alpha+ and CD11b+ dendritic cells is sufficient for control of Leishmania major.	Pubmed	0.0044	65.1	0.0114	0.116	1	1	HLA-DQB1
Pubmed	23409301	X-Linked Protoporphyria	Pubmed	0.0044	65.1	0.0114	0.116	1	1	ALAS2
Pubmed	19954365	Hydroxyurea-induced hematological response in transfusion-independent beta-thalassemia intermedia: case series and review of literature.	GeneRIF	0.0044	65.1	0.0114	0.116	1	1	HBB
Pubmed	9697113	PEP-19 immunohistochemistry defines the basal ganglia and associated structures in the adult human brain, and is dramatically reduced in Huntington's disease.	Pubmed	0.0044	65.1	0.0114	0.116	1	1	PCP4
Pubmed	9731527	A gene related to Caenorhabditis elegans spermatogenesis factor fer-1 is mutated in limb-girdle muscular dystrophy type 2B.	Pubmed	0.0044	65.1	0.0114	0.116	1	1	DYSF
Pubmed	18570888	Mitochondrial reactive oxygen species mediate hypoxic down-regulation of hERG channel protein.	GeneRIF	0.0044	65.1	0.0114	0.116	1	1	KCNH2
Pubmed	1787097	Hb Isehara (or Hb Redondo) [beta 92 (F8) His----Asn]: an unstable variant with a proximal histidine substitution at the heme contact.	Pubmed	0.0044	65.1	0.0114	0.116	1	1	HBB
Pubmed	17027065	EPCR gene A3 haplotype and elevated soluble endothelial protein C receptor (sEPCR) levels in Turkish pediatric stroke patients.	GeneRIF	0.0044	65.1	0.0114	0.116	1	1	PROCR
Pubmed	2372512	Hb Icaria-Hb H disease: identification of the Hb Icaria mutation through analysis of amplified DNA.	Pubmed	0.0044	65.1	0.0114	0.116	1	1	HBA2
Pubmed	25198651	Contribution of dysferlin deficiency to skeletal muscle pathology in asymptomatic and severe dystroglycanopathy models: generation of a new model for Fukuyama congenital muscular dystrophy.	Pubmed	0.0044	65.1	0.0114	0.116	1	1	DYSF
Pubmed	12777209	Presence of atrial natriuretic factor in normal and hyperplastic human prostate and its relationship with oxytocin localisation.	Pubmed	0.0044	65.1	0.0114	0.116	1	1	NPPA
Pubmed	17890909	Transglutaminases: the missing link in nephrogenic systemic fibrosis.	GeneRIF	0.0044	65.1	0.0114	0.116	1	1	TGM2
Pubmed	19301262	Identification of tubulins as substrates of serine protease HtrA1 by mixture-based oriented peptide library screening.	GeneRIF	0.0044	65.1	0.0114	0.116	1	1	HTRA1
Pubmed	2365694	Analysis of the promoter region of the gene encoding mouse cholesterol side-chain cleavage enzyme.	Pubmed	0.0044	65.1	0.0114	0.116	1	1	CYP11A1
Pubmed	12072047	Preliminary evidence that an endogenous retroviral long-terminal repeat (LTR13) at the HLA-DQB1 gene locus confers susceptibility to Addison's disease.	GeneRIF	0.0044	65.1	0.0114	0.116	1	1	HLA-DQB1
Pubmed	18575627	Overexpression of the mitochondrial T3 receptor p43 induces a shift in skeletal muscle fiber types.	Pubmed	0.0044	65.1	0.0114	0.116	1	1	THRA
Pubmed	24339996	Nogo-receptor 1 deficiency has no influence on immune cell repertoire or function during experimental autoimmune encephalomyelitis.	Pubmed	0.0044	65.1	0.0114	0.116	1	1	RTN4R
Pubmed	8104826	The 5' untranslated region of the human gamma-glutamyl transferase mRNA contains a tissue-specific active translational enhancer.	Pubmed	0.0044	65.1	0.0114	0.116	1	1	GGT1
Pubmed	21266828	Refined prognostic role of CD68-positive tumor macrophages in the context of the cellular micromilieu of classical Hodgkin lymphoma.	GeneRIF	0.0044	65.1	0.0114	0.116	1	1	CD68
Pubmed	11800015	In vivo DNA-protein interactions at hypersensitive site 3.5 of the human beta-globin locus control region.	GeneRIF	0.0044	65.1	0.0114	0.116	1	1	HBB
Pubmed	14533779	CX3CL1 and CX3CR1 expression in human brain tissue: noninflammatory control versus multiple sclerosis.	GeneRIF	0.0044	65.1	0.0114	0.116	1	1	CX3CL1
Pubmed	20373357	The PDXK rs2010795 variant is not associated with Parkinson disease in Italy.	GeneRIF	0.0044	65.1	0.0114	0.116	1	1	PDXK
Pubmed	24698628	Ophthalmologic findings in an 18-month-old boy with focal dermal hypoplasia.	Pubmed	0.0044	65.1	0.0114	0.116	1	1	PORCN
Pubmed	16221767	A functional genetic variation of adenosine deaminase affects the duration and intensity of deep sleep in humans.	Pubmed	0.0044	65.1	0.0114	0.116	1	1	ADA
Pubmed	16509868	HLA-DQB1 locus and gastric cancer in Helicobacter pylori infection.	GeneRIF	0.0044	65.1	0.0114	0.116	1	1	HLA-DQB1
Pubmed	17363390	Modulation of hERG potassium currents in HEK-293 cells by protein kinase C. Evidence for direct phosphorylation of pore forming subunits.	GeneRIF	0.0044	65.1	0.0114	0.116	1	1	KCNH2
Pubmed	21029394	Spectral analysis by XANES reveals that GPNMB influences the chemical composition of intact melanosomes.	GeneRIF	0.0044	65.1	0.0114	0.116	1	1	GPNMB
Pubmed	15800067	Human ether-a-go-go-related gene 1 channels are physically linked to beta1 integrins and modulate adhesion-dependent signaling.	GeneRIF	0.0044	65.1	0.0114	0.116	1	1	KCNH2
Pubmed	11912256	Loss-of-function polymorphism of the human kallikrein gene with reduced urinary kallikrein activity.	GeneRIF	0.0044	65.1	0.0114	0.116	1	1	KLK1
Pubmed	11535251	Genetic susceptibility and environmental estrogen-like compounds.	Pubmed	0.0044	65.1	0.0114	0.116	1	1	CYP11A1
Pubmed	19243684	Changes of Nogo-A and receptor NgR in the lumbar spinal cord of ALS model mice.	GeneRIF	0.0044	65.1	0.0114	0.116	1	1	RTN4R
Pubmed	21151024	p75 neurotrophin receptor mediates apoptosis in transit-amplifying cells and its overexpression restores cell death in psoriatic keratinocytes.	GeneRIF	0.0044	65.1	0.0114	0.116	1	1	NGFR
Pubmed	22320917	Regulation of transglutaminase-mediated hepatic cell death in alcoholic steatohepatitis and non-alcoholic steatohepatitis.	GeneRIF	0.0044	65.1	0.0114	0.116	1	1	TGM2
Pubmed	22670777	Mannan-binding lectin (MBL)-associated serine protease-1 (MASP-1), a serine protease associated with humoral pattern-recognition molecules: normal and acute-phase levels in serum and stoichiometry of lectin pathway components.	GeneRIF	0.0044	65.1	0.0114	0.116	1	1	MASP1

Category	ID	Name	Source	p-value	q-value Bonferroni	q-value FDR B&H	q-value FDR B&Y	Hit Count in Query List	Hit Count in Genome	Hit in Query List
Pubmed	19710315	The p75 neurotrophin receptor promotes amyloid-beta(1-42)-induced neuritic dystrophy in vitro and in vivo.	Pubmed	0.0044	65.1	0.0114	0.116	1	1	NGFR
Pubmed	23194051	Napsin A is differentially expressed in sclerosing hemangiomas of the lung.	GeneRIF	0.0044	65.1	0.0114	0.116	1	1	NAPSA
Pubmed	19028477	Cancer biomarker AKR1B10 and carbonyl metabolism.	GeneRIF	0.0044	65.1	0.0114	0.116	1	1	AKR1B10
Pubmed	15161653	Pivotal role of cathepsin K in lung fibrosis.	GeneRIF	0.0044	65.1	0.0114	0.116	1	1	CTSK
Pubmed	15070953	Inhibin B: a potential marker of gonadal activity in patients with anorexia nervosa during weight recovery.	GeneRIF	0.0044	65.1	0.0114	0.116	1	1	INHBB
Pubmed	20047901	Schwann cell p75NTR prevents spontaneous sensory reinnervation of the adult spinal cord.	GeneRIF	0.0044	65.1	0.0114	0.116	1	1	NGFR
Pubmed	22231926	The role of TG2 in ECV304-related vasculogenic mimicry.	GeneRIF	0.0044	65.1	0.0114	0.116	1	1	TGM2
Pubmed	16237120	Increased susceptibility to complement attack due to down-regulation of decay-accelerating factor/CD55 in dysferlin-deficient muscular dystrophy.	Pubmed	0.0044	65.1	0.0114	0.116	1	1	DYSF
Pubmed	22160262	Polymorphism of transglutaminase 2: unusually low frequency of genomic variants with deficient functions.	GeneRIF	0.0044	65.1	0.0114	0.116	1	1	TGM2
Pubmed	15870261	The human beta-globin locus control region can silence as well as activate gene expression.	GeneRIF	0.0044	65.1	0.0114	0.116	1	1	HBB
Pubmed	8642597	Crystal structure of T state haemoglobin with oxygen bound at all four haems.	Pubmed	0.0044	65.1	0.0114	0.116	1	1	HBB
Pubmed	23904164	Functional KCa3.1 channels regulate steroid insensitivity in bronchial smooth muscle cells.	Pubmed	0.0044	65.1	0.0114	0.116	1	1	KCNN4
Pubmed	18465053	Renal tubular function in patients with beta-thalassaemia major in Zahedan, southeast Iran.	Pubmed	0.0044	65.1	0.0114	0.116	1	1	HBB
Pubmed	25478825	Molecular mechanism of the chaperone function of mini-a-crystallin, a 19-residue peptide of human a-crystallin.	Pubmed	0.0044	65.1	0.0114	0.116	1	1	CRYAA
Pubmed	22524255	Genotyping of ß-globin gene mutations in single lymphocytes: a preliminary study for preimplantation genetic diagnosis of monogenic disorders.	GeneRIF	0.0044	65.1	0.0114	0.116	1	1	HBB
Pubmed	17536804	Transition-state variation in human, bovine, and Plasmodium falciparum adenosine deaminases.	GeneRIF	0.0044	65.1	0.0114	0.116	1	1	ADA
Pubmed	10086971	C-terminal HERG mutations: the role of hypokalemia and a KCNQ1-associated mutation in cardiac event occurrence.	Pubmed	0.0044	65.1	0.0114	0.116	1	1	KCNH2
Pubmed	15039364	Helicobacter pylori-mediated gastritis induces local downregulation of secretory leukocyte protease inhibitor in the antrum.	GeneRIF	0.0044	65.1	0.0114	0.116	1	1	SLPI
Pubmed	22419851	Stem cell marker CD271 is expressed by vasculogenic mimicry-forming uveal melanoma cells in three-dimensional cultures.	GeneRIF	0.0044	65.1	0.0114	0.116	1	1	NGFR
Pubmed	11925509	The effect of alpha2,6-linked sialic acid on anti-IgM antibody-induced apoptosis in Ramos cells.	GeneRIF	0.0044	65.1	0.0114	0.116	1	1	ORM1
Pubmed	20570369	CX3CL1-induced modulation at CA1 synapses reveals multiple mechanisms of EPSC modulation involving adenosine receptor subtypes.	GeneRIF	0.0044	65.1	0.0114	0.116	1	1	CX3CL1
Pubmed	19596859	The developmentally regulated osteoblast phosphodiesterase GDE3 is glycerophosphoinositol-specific and modulates cel growth.	GeneRIF	0.0044	65.1	0.0114	0.116	1	1	GDPD2
Pubmed	17484883	Whole-genome analysis and HLA genotyping of enteropathy-type T-cell lymphoma reveals 2 distinct lymphoma subtypes.	GeneRIF	0.0044	65.1	0.0114	0.116	1	1	HLA-DQB1
Pubmed	20190098	Heterogeneous ventricular sympathetic innervation, altered beta-adrenergic receptor expression, and rhythm instability in mice lacking the p75 neurotrophin receptor.	GeneRIF	0.0044	65.1	0.0114	0.116	1	1	NGFR
Pubmed	17297265	HLA-DQB1 allele and hypocretin in Korean narcoleptics with cataplexy.	GeneRIF	0.0044	65.1	0.0114	0.116	1	1	HLA-DQB1
Pubmed	8639768	Early ontogeny of the human marrow from long bones: an immunohistochemical study of hematopoiesis and its microenvironment.	Pubmed	0.0044	65.1	0.0114	0.116	1	1	CD68
Pubmed	23690218	Atrial natriuretic peptide presence in parotid gland of human fetus at 13th week of development and in adult man.	Pubmed	0.0044	65.1	0.0114	0.116	1	1	NPPA
Pubmed	23624156	Dual effects of exercise in dysferlinopathy.	Pubmed	0.0044	65.1	0.0114	0.116	1	1	DYSF
Pubmed	15202000	Selective expression of HERG and Kv2 channels influences proliferation of uterine cancer cells.	GeneRIF	0.0044	65.1	0.0114	0.116	1	1	KCNH2
Pubmed	16329095	Spontaneous development of synovitis and cartilage degeneration in transgenic mice overexpressing cathepsin K.	GeneRIF	0.0044	65.1	0.0114	0.116	1	1	CTSK
Pubmed	10496277	Dysferlin is a surface membrane-associated protein that is absent in Miyoshi myopathy.	Pubmed	0.0044	65.1	0.0114	0.116	1	1	DYSF
Pubmed	20504932	Human cytomegalovirus pUL83 stimulates activity of the viral immediate-early promoter through its interaction with the cellular IFI16 protein.	GeneRIF	0.0044	65.1	0.0114	0.116	1	1	IFI16
Pubmed	19236911	Structure and promoter characterization of aldo-keto reductase family 1 B10 gene.	GeneRIF	0.0044	65.1	0.0114	0.116	1	1	AKR1B10
Pubmed	22382022	The ASB2ß Ubiquitin-interacting motif is involved in its monoubiquitination.	GeneRIF	0.0044	65.1	0.0114	0.116	1	1	ASB2
Pubmed	21818782	Tissue transglutaminase activity is involved in the differentiation of oligodendrocyte precursor cells into myelin-forming oligodendrocytes during CNS remyelination.	Pubmed	0.0044	65.1	0.0114	0.116	1	1	TGM2
Pubmed	11777952	Fractalkine is expressed by smooth muscle cells in response to IFN-gamma and TNF-alpha and is modulated by metalloproteinase activity.	GeneRIF	0.0044	65.1	0.0114	0.116	1	1	CX3CL1
Pubmed	18456010	The action of MBL-associated serine protease 1 (MASP1) on factor XIII and fibrinogen.	GeneRIF	0.0044	65.1	0.0114	0.116	1	1	MASP1
Pubmed	18294055	Dysferlin homozygous mutation G1418D causes limb-girdle type 2B in a Mexican family.	GeneRIF	0.0044	65.1	0.0114	0.116	1	1	DYSF
Pubmed	24875677	KCNH2 polymorphism and methadone dosage interact to enhance QT duration.	Pubmed	0.0044	65.1	0.0114	0.116	1	1	KCNH2
Pubmed	17490901	Genetic relationship of a Mexican Afromestizo population through the analysis of the 3' haplotype of the beta globin gene in betaA chromosomes.	GeneRIF	0.0044	65.1	0.0114	0.116	1	1	HBB

Category	ID	Name	Source	p-value	q-value Bonferroni	q-value FDR B&H	q-value FDR B&Y	Hit Count in Query List	Hit Count in Genome	Hit in Query List
Pubmed	11373325	Immunohistochemical localization and comparison of carboxypeptidases D, E, and Z, alpha-MSH, ACTH, and MIB-1 between human anterior and corticotroph cell "basophil invasion" of the posterior pituitary.	Pubmed	0.0044	65.1	0.0114	0.116	1	1	CPE
Pubmed	18206206	Association of HTRA1 polymorphism and bilaterality in advanced age-related macular degeneration.	GeneRIF	0.0044	65.1	0.0114	0.116	1	1	HTRA1
Pubmed	20092819	Gamma-glutamyltransferase and prognosis in patients with stable coronary heart disease followed over 8 years.	GeneRIF	0.0044	65.1	0.0114	0.116	1	1	GGT1
Pubmed	18387951	An essential ligand-binding domain in the membrane receptor for retinol-binding protein revealed by large-scale mutagenesis and a human polymorphism.	GeneRIF	0.0044	65.1	0.0114	0.116	1	1	STRA6
Pubmed	10894926	Assignment of the endogenous retrovirus HERV-R (ERV3) to human chromosome 7q11.2 by radiation hybrid mapping.	Pubmed	0.0044	65.1	0.0114	0.116	1	1	ERV3-1
Pubmed	11831460	Eukaryotic zinc transporters and their regulation.	Pubmed	0.0044	65.1	0.0114	0.116	1	1	SLC39A2
Pubmed	22505522	The kallikrein 14 gene is down-regulated by androgen receptor signalling and harbours genetic variation that is associated with prostate tumour aggressiveness.	GeneRIF	0.0044	65.1	0.0114	0.116	1	1	KLK14
Pubmed	1526658	A novel gene constitutively expressed in human lymphoid cells is inducible with interferon-gamma in myeloid cells.	Pubmed	0.0044	65.1	0.0114	0.116	1	1	IFI16
Pubmed	20174870	Gender-specific association of ADA genetic polymorphism with human longevity.	GeneRIF	0.0044	65.1	0.0114	0.116	1	1	ADA
Pubmed	20665732	Correlation of the expression of human kallikrein-related peptidases 4 and 7 with the prognosis in oral squamous cell carcinoma.	Pubmed	0.0044	65.1	0.0114	0.116	1	1	KLK7
Pubmed	7673200	Involvement of Ser-451 and Ser-452 in the catalysis of human gamma-glutamyl transpeptidase.	Pubmed	0.0044	65.1	0.0114	0.116	1	1	GGT1
Pubmed	15318348	Developmental and tissue-specific regulation of a novel dysferlin isoform.	GeneRIF	0.0044	65.1	0.0114	0.116	1	1	DYSF
Pubmed	6629823	A new hemoglobin with high oxygen affinity--hemoglobin bunbury: alpha 2 beta 2 [94 (FG1) Asp replaced by Asn].	Pubmed	0.0044	65.1	0.0114	0.116	1	1	HBB
Pubmed	12667665	CSF and serum levels of soluble fractalkine (CX3CL1) in inflammatory diseases of the nervous system.	GeneRIF	0.0044	65.1	0.0114	0.116	1	1	CX3CL1
Pubmed	22738294	Prosaposin, a regulator of estrogen receptor alpha, promotes breast cancer growth.	GeneRIF	0.0044	65.1	0.0114	0.116	1	1	PSAP
Pubmed	15475450	CD74 is expressed by multiple myeloma and is a promising target for therapy.	GeneRIF	0.0044	65.1	0.0114	0.116	1	1	CD74
Pubmed	19535636	Restricted autoantigen recognition associated with deletional and adaptive regulatory mechanisms.	Pubmed	0.0044	65.1	0.0114	0.116	1	1	HLA-DQB1
Pubmed	9662053	Organization of the human carboxypeptidase E gene and molecular scanning for mutations in Japanese subjects with NIDDM or obesity.	Pubmed	0.0044	65.1	0.0114	0.116	1	1	CPE
Pubmed	22065922	Congenital anterior polar cataract associated with a missense mutation in the human alpha crystallin gene CRYAA.	GeneRIF	0.0044	65.1	0.0114	0.116	1	1	CRYAA
Pubmed	24401016	Identification of three new nucleotide substitutions in the ß-globin gene: laboratoristic approach and impact on genetic counselling for beta-thalassaemia.	Pubmed	0.0044	65.1	0.0114	0.116	1	1	HBB
Pubmed	19927780	Are HLA DQB1 alleles correlated with breast cancer histopronostic parameters in Tunisia?	GeneRIF	0.0044	65.1	0.0114	0.116	1	1	HLA-DQB1
Pubmed	26182728	XmnI POLYMORPHISM AND DISEASE SEVERITY IN PATIENTS WITH BETA THALASSEMIA FROM NORTHERN PAKISTAN.	Pubmed	0.0044	65.1	0.0114	0.116	1	1	HBB
Pubmed	21669058	Role of hERG1 K(+) channels in leukemia cells as a positive regulator in SDF-1a-induced proliferation.	GeneRIF	0.0044	65.1	0.0114	0.116	1	1	KCNH2
Pubmed	12419948	Activins A, AB, and B inhibit hepatocyte growth factor synthesis by MRC-5 human lung fibroblasts.	Pubmed	0.0044	65.1	0.0114	0.116	1	1	INHBB
Pubmed	15481883	Hb Suan-Dok [alpha109(G16)Leu-->Arg; CTG-->CGG (alpha2)] described in a patient of African ancestry.	Pubmed	0.0044	65.1	0.0114	0.116	1	1	HBA2
Pubmed	8627329	O-linked oligosaccharide on the 75-kDa neurotrophin receptor.	Pubmed	0.0044	65.1	0.0114	0.116	1	1	NGFR
Pubmed	2457983	Interferons as gene activators: close linkage of two interferon-activatable murine genes.	Pubmed	0.0044	65.1	0.0114	0.116	1	1	IFI16
Pubmed	23243195	The impact of partial and complete loss-of-function mutations in endothelial lipase on high-density lipoprotein levels and functionality in humans.	GeneRIF	0.0044	65.1	0.0114	0.116	1	1	LIPG
Pubmed	23457306	Self-catalytic DNA depurination underlies human ß-globin gene mutations at codon 6 that cause anemias and thalassemias.	GeneRIF	0.0044	65.1	0.0114	0.116	1	1	HBB
Pubmed	10930737	Pyridoxal kinase knockout of Dictyostelium complemented by the human homologue.	Pubmed	0.0044	65.1	0.0114	0.116	1	1	PDXK
Pubmed	15857712	Differential effect of p75 neurotrophin receptor on expression of pro-apoptotic proteins c-jun, p38 and caspase-3 in dorsal root ganglion cells after axotomy in experimental diabetes.	Pubmed	0.0044	65.1	0.0114	0.116	1	1	NGFR
Pubmed	11017088	Mutations in ARHGEF6, encoding a guanine nucleotide exchange factor for Rho GTPases, in patients with X-linked mental retardation.	Pubmed	0.0044	65.1	0.0114	0.116	1	1	ARHGEF6
Pubmed	11038363	Impaired prohormone convertases in Cpe(fat)/Cpe(fat) mice.	Pubmed	0.0044	65.1	0.0114	0.116	1	1	CPE
Pubmed	21057041	Multiple splicing defects caused by hERG splice site mutation 2592+1G>A associated with long QT syndrome.	GeneRIF	0.0044	65.1	0.0114	0.116	1	1	KCNH2
Pubmed	2015347	Ontogeny of adenosine deaminase in the mouse decidua and placenta: immunolocalization and embryo transfer studies.	Pubmed	0.0044	65.1	0.0114	0.116	1	1	ADA
Pubmed	17991484	Development of a new antibody to the human inhibin/activin betaB subunit and its application to improved inhibin B ELISAs.	GeneRIF	0.0044	65.1	0.0114	0.116	1	1	INHBB
Pubmed	18419131	Role of the beta4Thr-beta73Asp hydrogen bond in HbS polymer and domain formation from multinucleate-containing clusters.	GeneRIF	0.0044	65.1	0.0114	0.116	1	1	HBB

Category	ID	Name	Source	p-value	q-value Bonferroni	q-value FDR B&H	q-value FDR B&Y	Hit Count in Query List	Hit Count in Genome	Hit in Query List
Pubmed	10775158	Molecular cloning and expression analysis of the complementary deoxyribonucleic acid for chicken inhibin/activin beta(B) subunit.	Pubmed	0.0044	65.1	0.0114	0.116	1	1	INHBB
Pubmed	22883932	Extracellular adenosine signaling induces CX3CL1 expression in the brain to promote experimental autoimmune encephalomyelitis.	GeneRIF	0.0044	65.1	0.0114	0.116	1	1	CX3CL1
Pubmed	12911608	Identification and characterization of a natural R96C EPCR variant.	Pubmed	0.0044	65.1	0.0114	0.116	1	1	PROCR
Pubmed	15802373	Pituitary resistance to thyroid hormone syndrome is associated with T3 receptor mutants that selectively impair beta2 isoform function.	GeneRIF	0.0044	65.1	0.0114	0.116	1	1	THRA
Pubmed	20353350	Relationship between impaired glycation and the N-terminal structure of the Hb Görwihl [beta5(A2)Pro-->Ala] variant.	GeneRIF	0.0044	65.1	0.0114	0.116	1	1	HBB
Pubmed	17182177	Downregulation and CpG island hypermethylation of NES1/hK10 gene in the pathogenesis of human gastric cancer.	GeneRIF	0.0044	65.1	0.0114	0.116	1	1	KLK10
Pubmed	22357957	Loss of Porcupine impairs convergent extension during gastrulation in zebrafish.	Pubmed	0.0044	65.1	0.0114	0.116	1	1	PORCN
Pubmed	14667806	Molecular cloning of nonsecreted endothelial cell-derived lipase isoforms.	Pubmed	0.0044	65.1	0.0114	0.116	1	1	LIPG
Pubmed	9099727	Human pyridoxal kinase. cDNA cloning, expression, and modulation by ligands of the benzodiazepine receptor.	Pubmed	0.0044	65.1	0.0114	0.116	1	1	PDXK
Pubmed	1670766	Isolation and characterization of cDNA clones to mouse macrophage and human endothelial cell tissue transglutaminases.	Pubmed	0.0044	65.1	0.0114	0.116	1	1	TGM2
Pubmed	24074398	Hb Koln [ß98(FG5) [GTG ¿ ATG, Val ¿ Met]: the first report from India.	Pubmed	0.0044	65.1	0.0114	0.116	1	1	HBB
Pubmed	20926433	Targeted deletion of endothelial lipase increases HDL particles with anti-inflammatory properties both in vitro and in vivo.	GeneRIF	0.0044	65.1	0.0114	0.116	1	1	LIPG
Pubmed	22729558	B7-H3 is expressed in human hepatocellular carcinoma and is associated with tumor aggressiveness and postoperative recurrence.	GeneRIF	0.0044	65.1	0.0114	0.116	1	1	CD276
Pubmed	20797908	T cell vaccination therapy in an induced model of anti-RNP autoimmune glomerulonephritis.	Pubmed	0.0044	65.1	0.0114	0.116	1	1	HLA-DQB1
Pubmed	18614783	Atrial natriuretic peptide frameshift mutation in familial atrial fibrillation.	GeneRIF	0.0044	65.1	0.0114	0.116	1	1	NPPA
Pubmed	18728021	First case of gamma-thalassemia in association with a betaS allele: a pitfall in the neonatal screening for sickle cell disease.	GeneRIF	0.0044	65.1	0.0114	0.116	1	1	HBB
Pubmed	6208479	Structure of adenosine deaminase mRNAs from normal and adenosine deaminase-deficient human cell lines.	Pubmed	0.0044	65.1	0.0114	0.116	1	1	ADA
Pubmed	9753501	T cell-mediated exocrine pancreatic damage in major histocompatibility complex class II-deficient mice.	Pubmed	0.0044	65.1	0.0114	0.116	1	1	HLA-DQB1
Pubmed	20363883	Properties of WT and mutant hERG K(+) channels expressed in neonatal mouse cardiomyocytes.	GeneRIF	0.0044	65.1	0.0114	0.116	1	1	KCNH2
Pubmed	12902341	Structure of the HERG K+ channel S5P extracellular linker: role of an amphipathic alpha-helix in C-type inactivation.	GeneRIF	0.0044	65.1	0.0114	0.116	1	1	KCNH2
Pubmed	11724766	Disruption of antigen-induced airway inflammation and airway hyper-responsiveness in low affinity neurotrophin receptor p75 gene deficient mice.	Pubmed	0.0044	65.1	0.0114	0.116	1	1	NGFR
Pubmed	19681149	Importance of PORCN and Wnt signaling pathways in embryogenesis.	GeneRIF	0.0044	65.1	0.0114	0.116	1	1	PORCN
Pubmed	21919946	Adenosine deaminase activity in fertile and infertile men.	GeneRIF	0.0044	65.1	0.0114	0.116	1	1	ADA
Pubmed	17932560	Adrenomedullin and adrenomedullin binding protein-1 protect endothelium-dependent vascular relaxation in sepsis.	GeneRIF	0.0044	65.1	0.0114	0.116	1	1	ZBTB7C
Pubmed	19794088	Association of a globin gene quadruplication and heterozygous ß thalassemia in patients with thalassemia intermedia.	GeneRIF	0.0044	65.1	0.0114	0.116	1	1	HBA2
Pubmed	10080937	Purification and characterization of human metallocarboxypeptidase Z.	Pubmed	0.0044	65.1	0.0114	0.116	1	1	CPZ
Pubmed	17625111	A role for tissue transglutaminase in stabilization of membrane-cytoskeletal particles shed from the human placenta.	GeneRIF	0.0044	65.1	0.0114	0.116	1	1	TGM2
Pubmed	20225398	Increased tissue kallikrein levels in type 2 diabetes.	GeneRIF	0.0044	65.1	0.0114	0.116	1	1	KLK1
Pubmed	22556410	Detrimental role for human high temperature requirement serine protease A1 (HTRA1) in the pathogenesis of intervertebral disc (IVD) degeneration.	GeneRIF	0.0044	65.1	0.0114	0.116	1	1	HTRA1
Pubmed	21061162	Carboxypeptidase E: elevated expression correlated with tumor growth and metastasis in pheochromocytomas and other cancers.	GeneRIF	0.0044	65.1	0.0114	0.116	1	1	CPE
Pubmed	2512582	Localization of a critical restriction site on the I-A beta chain that determines susceptibility to collagen-induced arthritis in mice.	Pubmed	0.0044	65.1	0.0114	0.116	1	1	HLA-DQB1
Pubmed	24400095	Mesenchymal stromal cells derived from the bone marrow of acute lymphoblastic leukemia patients show altered BMP4 production: correlations with the course of disease.	Pubmed	0.0044	65.1	0.0114	0.116	1	1	BMP4
Pubmed	24779016	BMP-4 genetic variants and protein expression are associated with platinum-based chemotherapy response and prognosis in NSCLC.	Pubmed	0.0044	65.1	0.0114	0.116	1	1	BMP4
Pubmed	6190133	Isolation and characterization of cDNA clones for human skeletal muscle alpha actin.	Pubmed	0.0044	65.1	0.0114	0.116	1	1	ACTA1
Pubmed	16798637	Hb Marineo [beta70(E14)Ala-->Val]: a silent hemoglobin variant with a mutation within the heme pocket.	GeneRIF	0.0044	65.1	0.0114	0.116	1	1	HBB
Pubmed	23963851	A novel mutation of the high-temperature requirement A serine peptidase 1 (HTRA1) gene in a Chinese family with cerebral autosomal recessive arteriopathy with subcortical infarcts and leukoencephalopathy (CARASIL).	Pubmed	0.0044	65.1	0.0114	0.116	1	1	HTRA1
Pubmed	18555227	Serum inhibin B during controlled ovarian hyperstimulation: an additional criterion for deciding whether to proceed with egg retrieval.	GeneRIF	0.0044	65.1	0.0114	0.116	1	1	INHBB
Pubmed	18242218	Megaintestine in claudin-15-deficient mice.	GeneRIF	0.0044	65.1	0.0114	0.116	1	1	CLDN15

Category	ID	Name	Source	p-value	q-value Bonferroni	q-value FDR B&H	q-value FDR B&Y	Hit Count in Query List	Hit Count in Genome	Hit in Query List
Pubmed	17129995	Human disc degeneration is associated with increased MMP 7 expression.	GeneRIF	0.0044	65.1	0.0114	0.116	1	1	MMP7
Pubmed	10100923	Chronic hypertension in ANP knockout mice: contribution of peripheral resistance.	Pubmed	0.0044	65.1	0.0114	0.116	1	1	NPPA
Pubmed	21119217	Reverse engineering gene network identifies new dysferlin-interacting proteins.	GeneRIF	0.0044	65.1	0.0114	0.116	1	1	DYSF
Pubmed	24040176	Serine protease HTRA1 antagonizes transforming growth factor-ß signaling by cleaving its receptors and loss of HTRA1 in vivo enhances bone formation.	Pubmed	0.0044	65.1	0.0114	0.116	1	1	HTRA1
Pubmed	8611149	Identification of a transcription factor that binds to the S box of the I-A beta gene of the major histocompatibility complex.	Pubmed	0.0044	65.1	0.0114	0.116	1	1	HLA-DQB1
Pubmed	8339498	Chromosomal assignment of the human gene for bone morphogenetic protein 4.	Pubmed	0.0044	65.1	0.0114	0.116	1	1	BMP4
Pubmed	3666141	Hemoglobin Grange-Blanche [beta 27(B9) Ala----Val], a new variant with normal expression and increased affinity for oxygen.	Pubmed	0.0044	65.1	0.0114	0.116	1	1	HBB
Pubmed	24384857	The recently suggested intestinal cancer stem cell marker DCLK1 is an epigenetic biomarker for colorectal cancer.	Pubmed	0.0044	65.1	0.0114	0.116	1	1	DCLK1
Pubmed	9456660	Secretory leukocyte protease inhibitor (SLPI) in mucosal fluids inhibits HIV-I.	Pubmed	0.0044	65.1	0.0114	0.116	1	1	SLPI
Pubmed	9252787	Kinetic studies of the effects of K+, Na+ and Li+ on the catalytic activity of human erythrocyte pyridoxal kinase.	Pubmed	0.0044	65.1	0.0114	0.116	1	1	PDXK
Pubmed	23615310	Phenotypic characterization of Ggt1(dwg/dwg) mice,a mouse model for hereditary ¿-glutamyltransferase deficiency.	Pubmed	0.0044	65.1	0.0114	0.116	1	1	GGT1
Pubmed	22468949	Thyroid hormone promotes neuronal differentiation of embryonic neural stem cells by inhibiting STAT3 signaling through TRa1.	GeneRIF	0.0044	65.1	0.0114	0.116	1	1	THRA
Pubmed	15304035	Haplotypes of the EPCR gene, plasma sEPCR levels and the risk of deep venous thrombosis.	GeneRIF	0.0044	65.1	0.0114	0.116	1	1	PROCR
Pubmed	21806934	Blockade of permeation by potassium but normal gating of the G628S nonconducting hERG channel mutant.	GeneRIF	0.0044	65.1	0.0114	0.116	1	1	KCNH2
Pubmed	21691077	Ca2+ activated K+ channel Kca3.1 as a determinant of gastric acid secretion.	GeneRIF	0.0044	65.1	0.0114	0.116	1	1	KCNN4
Pubmed	20001220	The inductive effect of bone morphogenetic protein 4 on chondral-lineage differentiation and in situ cartilage repair.	GeneRIF	0.0044	65.1	0.0114	0.116	1	1	BMP4
Pubmed	18621100	Import of hybrid forms of CYP11A1 into yeast mitochondria.	GeneRIF	0.0044	65.1	0.0114	0.116	1	1	CYP11A1
Pubmed	22086524	The ADA*2 allele of the adenosine deaminase gene (20q13.11) and recurrent spontaneous abortions: an age-dependent association.	GeneRIF	0.0044	65.1	0.0114	0.116	1	1	ADA
Pubmed	22740344	Endothelial lipase is localized to follicular epithelial cells in the thyroid gland and is moderately expressed in adipocytes.	GeneRIF	0.0044	65.1	0.0114	0.116	1	1	LIPG
Pubmed	9154122	Assignment of the gene for human tetranectin (TNA) to chromosome 3p22-->p21.3 by somatic cell hybrid mapping.	Pubmed	0.0044	65.1	0.0114	0.116	1	1	CLEC3B
Pubmed	17671187	Enhanced peritoneal ovarian tumor dissemination by tissue transglutaminase.	GeneRIF	0.0044	65.1	0.0114	0.116	1	1	TGM2
Pubmed	24065476	AKAP12/Gravin gene expression in colorectal cancer: clinical importance and review of the literature.	Pubmed	0.0044	65.1	0.0114	0.116	1	1	AKAP12
Pubmed	16356734	Distinct effects of p75 in mediating actions of neurotrophins on basal forebrain oligodendrocytes.	GeneRIF	0.0044	65.1	0.0114	0.116	1	1	NGFR
Pubmed	6948771	Tryptic peptide comparison of Ia antigen alpha and beta polypeptides from the I-A mutant B6.C-H-2bm12 and its congenic parental strain B6.	Pubmed	0.0044	65.1	0.0114	0.116	1	1	HLA-DQB1
Pubmed	15528150	The potential of electrospray ionization mass spectrometry for the diagnosis of hemoglobin variants found in newborn screening.	GeneRIF	0.0044	65.1	0.0114	0.116	1	1	HBB
Pubmed	16885058	Association of Hb Shelby with Hb S in the south of Brazil.	GeneRIF	0.0044	65.1	0.0114	0.116	1	1	HBB
Pubmed	20096288	Bone morphogenetic protein 4 mediates myocardial ischemic injury through JNK-dependent signaling pathway.	GeneRIF	0.0044	65.1	0.0114	0.116	1	1	BMP4
Pubmed	16776851	Modulation of granulocyte-endothelium interactions by antileukoproteinase: inhibition of anti-type II collagen antibody-induced leukocyte attachment to the synovial endothelium.	GeneRIF	0.0044	65.1	0.0114	0.116	1	1	SLPI
Pubmed	21127709	B7-h3 and its role in antitumor immunity.	GeneRIF	0.0044	65.1	0.0114	0.116	1	1	CD276
Pubmed	15684612	Cloning and characterization of the 5' flanking region of the HGFIN gene indicate a cooperative role among p53 and cytokine-mediated transcription factors: relevance to cell cycle regulation.	GeneRIF	0.0044	65.1	0.0114	0.116	1	1	GPNMB
Pubmed	21941430	Equal force recovery in dysferlin-deficient and wild-type muscles following saponin exposure.	GeneRIF	0.0044	65.1	0.0114	0.116	1	1	DYSF
Pubmed	15528312	A cardioprotective role for the endothelial protein C receptor in lipopolysaccharide-induced endotoxemia in the mouse.	Pubmed	0.0044	65.1	0.0114	0.116	1	1	PROCR
Pubmed	20182795	Parallel inhibition of cell growth and induction of cell migration and invasion in breast cancer cells by bone morphogenetic protein 4.	GeneRIF	0.0044	65.1	0.0114	0.116	1	1	BMP4
Pubmed	16862423	The inflammatory reaction pattern distinguishes primary dysferlinopathies from idiopathic inflammatory myopathies: an important role for the membrane attack complex.	GeneRIF	0.0044	65.1	0.0114	0.116	1	1	DYSF
Pubmed	21683983	Limited smoothelin expression within the muscularis mucosae: validation in bladder diverticula.	GeneRIF	0.0044	65.1	0.0114	0.116	1	1	SMTN
Pubmed	21576365	Impaired locomotor learning and altered cerebellar synaptic plasticity in pep-19/PCP4-null mice.	GeneRIF	0.0044	65.1	0.0114	0.116	1	1	PCP4
Pubmed	23229356	The molecular basis of induction and formation of tunneling nanotubes.	Pubmed	0.0044	65.1	0.0114	0.116	1	1	ACTA1
Pubmed	21297635	Substrate-induced remodeling of the active site regulates human HTRA1 activity.	GeneRIF	0.0044	65.1	0.0114	0.116	1	1	HTRA1
Pubmed	15274039	A missense polymorphism (S205L) of the low-affinity neurotrophin receptor p75NTR gene is associated with depressive disorder and attempted suicide.	GeneRIF	0.0044	65.1	0.0114	0.116	1	1	NGFR

Category	ID	Name	Source	p-value	q-value Bonferroni	q-value FDR B&H	q-value FDR B&Y	Hit Count in Query List	Hit Count in Genome	Hit in Query List
Pubmed	12759346	Hypochlorous acid generated by myeloperoxidase modifies adjacent tryptophan and glycine residues in the catalytic domain of matrix metalloproteinase-7 (matrilysin): an oxidative mechanism for restraining proteolytic activity during inflammation.	GeneRIF	0.0044	65.1	0.0114	0.116	1	1	MMP7
Pubmed	20854095	PORCN mutations and variants identified in patients with focal dermal hypoplasia through diagnostic gene sequencing.	GeneRIF	0.0044	65.1	0.0114	0.116	1	1	PORCN
Pubmed	17273165	Mutation in Mpzl3, a novel [corrected] gene encoding a predicted [corrected] adhesion protein, in the rough coat (rc) mice with severe skin and hair abnormalities.	GeneRIF	0.0044	65.1	0.0114	0.116	1	1	MPZL3
Pubmed	24569898	Arrhythmic potency of human ether-a-go-go-related gene mutations L532P and N588K in a computational model of human atrial myocytes.	Pubmed	0.0044	65.1	0.0114	0.116	1	1	KCNH2
Pubmed	22008728	An 18-kDa translocator protein (TSPO) polymorphism explains differences in binding affinity of the PET radioligand PBR28.	GeneRIF	0.0044	65.1	0.0114	0.116	1	1	TSPO
Pubmed	24042196	Differential expression of the tumor suppressor A-kinase anchor protein 12 in human diffuse and pilocytic astrocytomas is regulated by promoter methylation.	Pubmed	0.0044	65.1	0.0114	0.116	1	1	AKAP12
Pubmed	21479826	TG2 transamidating activity acts as a reostat controlling the interplay between apoptosis and autophagy.	GeneRIF	0.0044	65.1	0.0114	0.116	1	1	TGM2
Pubmed	7536752	IFI 16 gene encodes a nuclear protein whose expression is induced by interferons in human myeloid leukaemia cell lines.	Pubmed	0.0044	65.1	0.0114	0.116	1	1	IFI16
Pubmed	11965279	Protective effects of the sickle cell gene against malaria morbidity and mortality.	GeneRIF	0.0044	65.1	0.0114	0.116	1	1	HBB
Pubmed	23079781	Elevated serine protease HtrA1 inhibits cell proliferation, reduces invasion, and induces apoptosis in esophageal squamous cell carcinoma by blocking the nuclear factor-¿B signaling pathway.	GeneRIF	0.0044	65.1	0.0114	0.116	1	1	HTRA1
Pubmed	20642333	Codon 24 (TAT>TAG) and codon 32 (ATG>AGG) (Hb Rotterdam): two novel alpha2 gene mutations associated with mild alpha-thalassemia found in the same family after newborn screening.	GeneRIF	0.0044	65.1	0.0114	0.116	1	1	HBA2
Pubmed	18989245	The efficacy of human atrial natriuretic peptide in patients with renal dysfunction undergoing cardiac surgery.	GeneRIF	0.0044	65.1	0.0114	0.116	1	1	NPPA
Pubmed	23756599	Transcriptome analysis of MENX-associated rat pituitary adenomas identifies novel molecular mechanisms involved in the pathogenesis of human pituitary gonadotroph adenomas.	Pubmed	0.0044	65.1	0.0114	0.116	1	1	CYP11A1
Pubmed	9600240	Genomic organization and mutational analysis of HERG, a gene responsible for familial long QT syndrome.	Pubmed	0.0044	65.1	0.0114	0.116	1	1	KCNH2
Pubmed	19643728	Aldo-keto reductase family 1 member B10 promotes cell survival by regulating lipid synthesis and eliminating carbonyls.	GeneRIF	0.0044	65.1	0.0114	0.116	1	1	AKR1B10
Pubmed	24284421	Downregulation of STRA6 expression in epidermal keratinocytes leads to hyperproliferation-associated differentiation in both in vitro and in vivo skin models.	Pubmed	0.0044	65.1	0.0114	0.116	1	1	STRA6
Pubmed	12589782	Lack of p75 receptor does not protect photoreceptors from light-induced cell death.	Pubmed	0.0044	65.1	0.0114	0.116	1	1	NGFR
Pubmed	2739657	Characterization and regulation of testicular inhibin beta-subunit mRNA.	Pubmed	0.0044	65.1	0.0114	0.116	1	1	INHBB
Pubmed	22891279	Voltage-sensing domain mode shift is coupled to the activation gate by the N-terminal tail of hERG channels.	GeneRIF	0.0044	65.1	0.0114	0.116	1	1	KCNH2
Pubmed	16767218	Serine protease HtrA1 modulates chemotherapy-induced cytotoxicity.	GeneRIF	0.0044	65.1	0.0114	0.116	1	1	HTRA1
Pubmed	11799143	Induction of Src-suppressed C kinase substrate (SSeCKS) in vascular endothelial cells by bacterial lipopolysaccharide.	GeneRIF	0.0044	65.1	0.0114	0.116	1	1	AKAP12
Pubmed	15979088	The HS2 enhancer of the beta-globin locus control region initiates synthesis of non-coding, polyadenylated RNAs independent of a cis-linked globin promoter.	GeneRIF	0.0044	65.1	0.0114	0.116	1	1	HBB
Pubmed	12184920	Immunohistochemical expression analysis of the human interferon-inducible gene IFI16, a member of the HIN200 family, not restricted to hematopoietic cells.	GeneRIF	0.0044	65.1	0.0114	0.116	1	1	IFI16
Pubmed	17652002	Relationship between gamma-glutamyltransferase and fat mass in a general population of 8-17 years old children. The FLVS II study.	GeneRIF	0.0044	65.1	0.0114	0.116	1	1	GGT1
Pubmed	6809283	Plasma proteins in human cortical bone: enrichment of alpha 2 HS-glycoprotein, alpha 1 acid-glycoprotein, and IgE.	Pubmed	0.0044	65.1	0.0114	0.116	1	1	ORM1
Pubmed	23744352	Potassium channel activation inhibits proliferation of breast cancer cells by activating a senescence program.	Pubmed	0.0044	65.1	0.0114	0.116	1	1	KCNH2
Pubmed	23325523	Prosaposin expression in the regenerated muscles of mdx and cardiotoxin-treated mice.	Pubmed	0.0044	65.1	0.0114	0.116	1	1	PSAP
Pubmed	24080465	Spectrum of ß-thalassemia mutations in Guizhou Province, PR China, including first observation of codon 121 (GAA>TAA) in Chinese population.	Pubmed	0.0044	65.1	0.0114	0.116	1	1	HBB
Pubmed	15111511	Investigation of the human ANP gene in type 1 diabetic nephropathy: case-control and follow-up studies.	GeneRIF	0.0044	65.1	0.0114	0.116	1	1	NPPA
Pubmed	23136156	Mechanism of loss of Kv11.1 K+ current in mutant T421M-Kv11.1-expressing rat ventricular myocytes: interaction of trafficking and gating.	GeneRIF	0.0044	65.1	0.0114	0.116	1	1	KCNH2
Pubmed	16804904	Prognostic significance of matrix metalloproteinase-7 in epithelial ovarian cancer and its relation to beta-catenin expression.	GeneRIF	0.0044	65.1	0.0114	0.116	1	1	MMP7
Pubmed	22791835	PAPSS2 mutations cause autosomal recessive brachyolmia.	GeneRIF	0.0044	65.1	0.0114	0.116	1	1	PAPSS2
Pubmed	22467576	Retinoid content, visual responses, and ocular morphology are compromised in the retinas of mice lacking the retinol-binding protein receptor, STRA6.	Pubmed	0.0044	65.1	0.0114	0.116	1	1	STRA6

Category	ID	Name	Source	p-value	q-value Bonferroni	q-value FDR B&H	q-value FDR B&Y	Hit Count in Query List	Hit Count in Genome	Hit in Query List
Pubmed	8344198	Expression and cellular localization of uterine side-chain cleavage cytochrome P450 messenger ribonucleic acid during early pregnancy in mice.	Pubmed	0.0044	65.1	0.0114	0.116	1	1	CYP11A1
Pubmed	9351627	Bacterial pneumonia causes augmented expression of the secretory leukoprotease inhibitor gene in the murine lung.	Pubmed	0.0044	65.1	0.0114	0.116	1	1	SLPI
Pubmed	3427041	Primary structure of tetranectin, a plasminogen kringle 4 binding plasma protein: homology with asialoglycoprotein receptors and cartilage proteoglycan core protein.	Pubmed	0.0044	65.1	0.0114	0.116	1	1	CLEC3B
Pubmed	2885259	Assignment of the human gamma-glutamyl transferase gene to the long arm of chromosome 22.	Pubmed	0.0044	65.1	0.0114	0.116	1	1	GGT1
Pubmed	23096558	Potential role of adenosine deaminase in the diagnosis of adult-onset Still's disease.	Pubmed	0.0044	65.1	0.0114	0.116	1	1	ADA
Pubmed	7670465	Adenosine-deaminase-deficient mice die perinatally and exhibit liver-cell degeneration, atelectasis and small intestinal cell death.	Pubmed	0.0044	65.1	0.0114	0.116	1	1	ADA
Pubmed	14626449	Expression of peripheral benzodiazepine receptor (PBR) in human tumors: relationship to breast, colorectal, and prostate tumor progression.	GeneRIF	0.0044	65.1	0.0114	0.116	1	1	TSPO
Pubmed	19205975	Hematological and molecular analysis of novel and rare beta-thalassemia mutations in the Indian population.	GeneRIF	0.0044	65.1	0.0114	0.116	1	1	HBB
Pubmed	21968827	Osteoarthritic change is delayed in a Ctsk-knockout mouse model of osteoarthritis.	GeneRIF	0.0044	65.1	0.0114	0.116	1	1	CTSK
Pubmed	12152660	The endothelial protein C receptor (EPCR) 23 bp insert mutation and the risk of venous thrombosis.	GeneRIF	0.0044	65.1	0.0114	0.116	1	1	PROCR
Pubmed	9529353	Paternal uniparental disomy for chromosome 1 revealed by molecular analysis of a patient with pycnodysostosis.	Pubmed	0.0044	65.1	0.0114	0.116	1	1	CTSK
Pubmed	21130771	A novel mutation in the KCNH2 gene associated with short QT syndrome.	GeneRIF	0.0044	65.1	0.0114	0.116	1	1	KCNH2
Pubmed	15983379	CD26, adenosine deaminase, and adenosine receptors mediate costimulatory signals in the immunological synapse.	GeneRIF	0.0044	65.1	0.0114	0.116	1	1	ADA
Pubmed	10510427	Cerebroside sulfate activator protein (Saposin B): chromatographic and electrospray mass spectrometric properties.	Pubmed	0.0044	65.1	0.0114	0.116	1	1	PSAP
Pubmed	19347328	The type 1 diabetes protective HLA DQB1*0602 allele is less frequent in gestational diabetes mellitus.	GeneRIF	0.0044	65.1	0.0114	0.116	1	1	HLA-DQB1
Pubmed	18853459	Analysis of the DYSF mutational spectrum in a large cohort of patients.	GeneRIF	0.0044	65.1	0.0114	0.116	1	1	DYSF
Pubmed	11907108	Reduced functional capacity of CD8+ T cells expanded by post-exposure vaccination of gamma-herpesvirus-infected CD4-deficient mice.	Pubmed	0.0044	65.1	0.0114	0.116	1	1	HLA-DQB1
Pubmed	20450492	Conformational flexibility and allosteric regulation of cathepsin K.	GeneRIF	0.0044	65.1	0.0114	0.116	1	1	CTSK
Pubmed	21115911	Methylation of AKAP12{alpha} promoter in lung cancer.	GeneRIF	0.0044	65.1	0.0114	0.116	1	1	AKAP12
Pubmed	18974585	Renal tubular dysfunction with nephrocalcinosis in a patient with beta thalassemia minor.	Pubmed	0.0044	65.1	0.0114	0.116	1	1	HBB
Pubmed	20869263	Delta chemokine (fractalkine)--a novel mediator of pulmonary arterial hypertension in children undergoing cardiac surgery.	GeneRIF	0.0044	65.1	0.0114	0.116	1	1	CX3CL1
Pubmed	20967228	Tissue transglutaminase promotes drug resistance and invasion by inducing mesenchymal transition in mammary epithelial cells.	GeneRIF	0.0044	65.1	0.0114	0.116	1	1	TGM2
Pubmed	21455340	Autoantibodies against MMP-7 as a novel diagnostic biomarker in esophageal squamous cell carcinoma.	GeneRIF	0.0044	65.1	0.0114	0.116	1	1	MMP7
Pubmed	24626093	Curcumin promotes autophagic survival of a subset of colon cancer stem cells, which are ablated by DCLK1-siRNA.	Pubmed	0.0044	65.1	0.0114	0.116	1	1	DCLK1
Pubmed	15910540	Is inhibin B involved in the toxic effect of lead on male reproduction?	GeneRIF	0.0044	65.1	0.0114	0.116	1	1	INHBB
Pubmed	24589892	GPNMB/OA protein increases the invasiveness of human metastatic prostate cancer cell lines DU145 and PC3 through MMP-2 and MMP-9 activity.	Pubmed	0.0044	65.1	0.0114	0.116	1	1	GPNMB
Pubmed	22578544	Structural and functional analysis of HtrA1 and its subdomains.	GeneRIF	0.0044	65.1	0.0114	0.116	1	1	HTRA1
Pubmed	21340693	BMP4 loss-of-function mutations in developmental eye disorders including SHORT syndrome.	GeneRIF	0.0044	65.1	0.0114	0.116	1	1	BMP4
Pubmed	11004271	Q118X mutation of M1S1 gene caused gelatinous drop-like corneal dystrophy: the P501T of BIGH3 gene found in a family with gelatinous drop-like corneal dystrophy.	Pubmed	0.0044	65.1	0.0114	0.116	1	1	TACSTD2
Pubmed	12424382	The neurotrophin receptor p75NTR as a positive modulator of myelination.	GeneRIF	0.0044	65.1	0.0114	0.116	1	1	NGFR
Pubmed	10594760	Identification of a novel mutation R42P in the gap junction protein beta-3 associated with autosomal dominant erythrokeratoderma variabilis.	Pubmed	0.0044	65.1	0.0114	0.116	1	1	GJB3
Pubmed	24852372	STRA6 is critical for cellular vitamin A uptake and homeostasis.	Pubmed	0.0044	65.1	0.0114	0.116	1	1	STRA6
Pubmed	11089562	Reproductive defects in gamma-glutamyl transpeptidase-deficient mice.	Pubmed	0.0044	65.1	0.0114	0.116	1	1	GGT1
Pubmed	19859662	Regulation of HERG (KCNH2) potassium channel surface expression by diacylglycerol.	GeneRIF	0.0044	65.1	0.0114	0.116	1	1	KCNH2
Pubmed	3384707	Hemoglobin Randwick or beta 15 (A12)Trp----Gly: a new unstable beta-chain hemoglobin variant.	Pubmed	0.0044	65.1	0.0114	0.116	1	1	HBB
Pubmed	15388935	Crystallization and preliminary X-ray diffraction studies of isoform alpha1 of the human thyroid hormone receptor ligand-binding domain.	GeneRIF	0.0044	65.1	0.0114	0.116	1	1	THRA
Pubmed	19277847	Glutamate at position 227 of junctophilin-2 is involved in binding to TRPC3.	Pubmed	0.0044	65.1	0.0114	0.116	1	1	JPH2
Pubmed	9458926	Salt-sensitive hypertension in ANP knockout mice: potential role of abnormal plasma renin activity.	Pubmed	0.0044	65.1	0.0114	0.116	1	1	NPPA

Category	ID	Name	Source	p-value	q-value Bonferroni	q-value FDR B&H	q-value FDR B&Y	Hit Count in Query List	Hit Count in Genome	Hit in Query List
Pubmed	19056122	Expression patterns of two serine protease HtrA1 forms in human placentas complicated by preeclampsia with and without intrauterine growth restriction.	GeneRIF	0.0044	65.1	0.0114	0.116	1	1	HTRA1
Pubmed	21936766	The KCNH2 gene is associated with neurocognition and the risk of schizophrenia.	GeneRIF	0.0044	65.1	0.0114	0.116	1	1	KCNH2
Pubmed	12603091	Hb Santander [beta34(B16)Val --> Asp (GTC --> GAC)]: a new unstable variant found as a de novo mutation in a Spanish patient.	Pubmed	0.0044	65.1	0.0114	0.116	1	1	HBB
Pubmed	16888915	[The "classical" macrophage marker CD68 is strongly expressed in primary human fibroblasts].	GeneRIF	0.0044	65.1	0.0114	0.116	1	1	CD68
Pubmed	17989991	Formation of aberrant TJ strands by overexpression of claudin-15 in MDCK II cells.	Pubmed	0.0044	65.1	0.0114	0.116	1	1	CLDN15
Pubmed	21440677	Mutation and gender-specific risk in type 2 long QT syndrome: implications for risk stratification for life-threatening cardiac events in patients with long QT syndrome.	GeneRIF	0.0044	65.1	0.0114	0.116	1	1	KCNH2
Pubmed	22614014	Macrophage cathepsin K promotes prostate tumor progression in bone.	GeneRIF	0.0044	65.1	0.0114	0.116	1	1	CTSK
Pubmed	24166646	The impact of atrial natriuretic peptide on anxiety, stress and craving in patients with alcohol dependence.	Pubmed	0.0044	65.1	0.0114	0.116	1	1	NPPA
Pubmed	19593686	Contribution of histological, serological, and genetic factors to the clinical heterogeneity of adul onset coeliac disease.	GeneRIF	0.0044	65.1	0.0114	0.116	1	1	HLA-DQB1
Pubmed	19955179	Target promiscuity and heterogeneous effects of tarantula venom peptides affecting Na+ and K+ ion channels.	GeneRIF	0.0044	65.1	0.0114	0.116	1	1	KCNH2
Pubmed	19001151	Dysferlin-deficient muscular dystrophy: gadofluorine M suitability at MR imaging in a mouse model.	GeneRIF	0.0044	65.1	0.0114	0.116	1	1	DYSF
Pubmed	1856451	Presence of adenosine deaminase on the surface of mononuclear blood cells: immunochemical localization using light and electron microscopy.	Pubmed	0.0044	65.1	0.0114	0.116	1	1	ADA
Pubmed	24409072	Overexpression of kallikrein gene 10 is a biomarker for predicting poor prognosis in gastric cancer.	Pubmed	0.0044	65.1	0.0114	0.116	1	1	KLK10
Pubmed	18597230	Nitric oxide level and adenosine deaminase activity in cerebrospinal fluid of patients with subarachnoid hemorrhage.	GeneRIF	0.0044	65.1	0.0114	0.116	1	1	ADA
Pubmed	18778890	The involvement of the fractalkine receptor in the transmigration of neuroblastoma cells through bone-marrow endothelial cells.	GeneRIF	0.0044	65.1	0.0114	0.116	1	1	CX3CL1
Pubmed	17272276	Heteromeric assembly of human ether-à-go-go-related gene (hERG) 1a/1b channels occurs cotranslationally via N-terminal interactions.	GeneRIF	0.0044	65.1	0.0114	0.116	1	1	KCNH2
Pubmed	20351248	Protein folding at the membrane interface, the structure of Nogo-66 requires interactions with a phosphocholine surface.	GeneRIF	0.0044	65.1	0.0114	0.116	1	1	RTN4R
Pubmed	15774461	Recycling of the membrane-anchored chemokine, CX3CL1.	GeneRIF	0.0044	65.1	0.0114	0.116	1	1	CX3CL1
Pubmed	19672025	Role of endothelial lipase in plasma HDL levels in a murine model of hypertriglyceridemia.	GeneRIF	0.0044	65.1	0.0114	0.116	1	1	LIPG
Pubmed	2533389	Interaction of tetranectin with sulphated polysaccharides and trypan blue.	Pubmed	0.0044	65.1	0.0114	0.116	1	1	CLEC3B
Pubmed	22112816	Low plasma level of atrial natriuretic peptide predicts development of diabetes: the prospective Malmo Diet and Cancer study.	GeneRIF	0.0044	65.1	0.0114	0.116	1	1	NPPA
Pubmed	17487834	Serum matrix metalloproteinase 7 levels identifies poor prognosis advanced colorectal cancer patients.	GeneRIF	0.0044	65.1	0.0114	0.116	1	1	MMP7
Pubmed	24824621	Trop-2 is a determinant of breast cancer survival.	Pubmed	0.0044	65.1	0.0114	0.116	1	1	TACSTD2
Pubmed	22052021	Expression of a-crystallin in the retina of human sympathetic ophthalmia.	GeneRIF	0.0044	65.1	0.0114	0.116	1	1	CRYAA
Pubmed	15928807	Phorbol ester up-regulates aldose reductase expression in A549 cells: a potential role for aldose reductase in cell cycle modulation.	GeneRIF	0.0044	65.1	0.0114	0.116	1	1	AKR1B10
Pubmed	19221016	Acetylcholine-induced activation of M3 muscarinic receptors stimulates robust matrix metalloproteinase gene expression in human colon cancer cells.	GeneRIF	0.0044	65.1	0.0114	0.116	1	1	MMP7
Pubmed	19077109	Expression of the translocator protein of 18 kDa by microglia, macrophages and astrocytes based on immunohistochemical localization in abnormal human brain.	GeneRIF	0.0044	65.1	0.0114	0.116	1	1	TSPO
Pubmed	9843210	Mutations in the gene encoding gap junction protein beta-3 associated with autosomal dominant hearing impairment.	Pubmed	0.0044	65.1	0.0114	0.116	1	1	GJB3
Pubmed	17221865	MID1 mutation screening in a large cohort of Opitz G/BBB syndrome patients: twenty-nine novel mutations identified.	GeneRIF	0.0044	65.1	0.0114	0.116	1	1	MID1
Pubmed	18554502	A-kinase anchoring protein 12 regulates the completion of cytokinesis.	GeneRIF	0.0044	65.1	0.0114	0.116	1	1	AKAP12
Pubmed	25713063	Multidomain human peroxidasin 1 is a highly glycosylated and stable homotrimeric high spin ferric peroxidase.	Pubmed	0.0044	65.1	0.0114	0.116	1	1	PXDN
Pubmed	1537303	Isoform-specific thyroid hormone receptor antibodies detect multiple thyroid hormone receptors in rat and human pituitaries.	Pubmed	0.0044	65.1	0.0114	0.116	1	1	THRA
Pubmed	24585434	Serum ANGPTL2 levels reflect clinical features of breast cancer patients: implications for the pathogenesis of breast cancer metastasis.	Pubmed	0.0044	65.1	0.0114	0.116	1	1	ANGPTL2
Pubmed	1517102	A new variant, HB Muscat [alpha 2 beta (2)32(B14)Leu----Val] observed in association with HB S in an Arabian family.	Pubmed	0.0044	65.1	0.0114	0.116	1	1	HBB
Pubmed	11474874	Immunologic and genetic markers in insulin-dependent diabetes mellitus (type 1) in an Argentine population.	GeneRIF	0.0044	65.1	0.0114	0.116	1	1	HLA-DQB1
Pubmed	22260793	NOGO-66 receptor deficient mice show slow acquisition of spatial memory task performance.	Pubmed	0.0044	65.1	0.0114	0.116	1	1	RTN4R
Pubmed	19605482	The carboxy-terminal tail of human cytomegalovirus (HCMV) US28 regulates both chemokine-independent and chemokine-dependent signaling in HCMV-infected cells.	GeneRIF	0.0044	65.1	0.0114	0.116	1	1	CX3CL1

Category	ID	Name	Source	p-value	q-value Bonferroni	q-value FDR B&H	q-value FDR B&Y	Hit Count in Query List	Hit Count in Genome	Hit in Query List
Pubmed	15760896	Degradation of trafficking-defective long QT syndrome type II mutant channels by the ubiquitin-proteasome pathway.	GeneRIF	0.0044	65.1	0.0114	0.116	1	1	KCNH2
Pubmed	11920956	Identification of single nucleotide polymorphisms in the human kallikrein 10 (KLK10) gene and their association with prostate, breast, testicular, and ovarian cancers.	GeneRIF	0.0044	65.1	0.0114	0.116	1	1	KLK10
Pubmed	16883536	[A study on HLA-DQB1 allele associated with genetic susceptibility to duodenal ulcer in Guangdong Hans].	GeneRIF	0.0044	65.1	0.0114	0.116	1	1	HLA-DQB1
Pubmed	19454350	Bone morphogenetic protein 4 signaling regulates epithelial renewal in the urinary tract in response to uropathogenic infection.	GeneRIF	0.0044	65.1	0.0114	0.116	1	1	BMP4
Pubmed	21597388	Non-small cell lung cancer induces an immunosuppressive phenotype of dendritic cells in tumor microenvironment by upregulating B7-H3.	GeneRIF	0.0044	65.1	0.0114	0.116	1	1	CD276
Pubmed	22014576	An antioxidant response phenotype shared between hereditary and sporadic type 2 papillary renal cell carcinoma.	GeneRIF	0.0044	65.1	0.0114	0.116	1	1	AKR1B10
Pubmed	9509262	The molecular genetics of the long QT syndrome: genes causing fainting and sudden death.	Pubmed	0.0044	65.1	0.0114	0.116	1	1	KCNH2
Pubmed	25001942	[Plasma sB7-H3 serves as an assistant diagnostic marker for hepatitis B virus-associated hepatocellular carcinoma].	Pubmed	0.0044	65.1	0.0114	0.116	1	1	CD276
Pubmed	15494491	B7-H3 enhances tumor immunity in vivo by costimulating rapid clonal expansion of antigen-specific CD8+ cytolytic T cells.	GeneRIF	0.0044	65.1	0.0114	0.116	1	1	CD276
Pubmed	17516553	Bone morphogenetic protein 4 induces epithelial-mesenchymal transition through MSX2 induction on pancreatic cancer cell line.	GeneRIF	0.0044	65.1	0.0114	0.116	1	1	BMP4
Pubmed	10603041	Experimental rabies virus infection of p75 neurotrophin receptor-deficient mice.	Pubmed	0.0044	65.1	0.0114	0.116	1	1	NGFR
Pubmed	24687172	Translocator protein 18 kDa negatively regulates inflammation in microglia.	Pubmed	0.0044	65.1	0.0114	0.116	1	1	TSPO
Pubmed	20301712	Focal Dermal Hypoplasia	Pubmed	0.0044	65.1	0.0114	0.116	1	1	PORCN
Pubmed	23683733	Rapid and inexpensive detection of common HBB gene mutations in Tunisian population by high-resolution melting analysis: implication for molecular diagnosis.	Pubmed	0.0044	65.1	0.0114	0.116	1	1	HBB
Pubmed	8003952	Murine prosaposin: expression in the reproductive system of a gene implicated in human genetic disease.	Pubmed	0.0044	65.1	0.0114	0.116	1	1	PSAP
Pubmed	18325492	Inhibiting wild-type and C299S mutant AKR1B10; a homologue of aldose reductase upregulated in cancers.	GeneRIF	0.0044	65.1	0.0114	0.116	1	1	AKR1B10
Pubmed	8877790	Two cardiac natriuretic peptide genes (atrial natriuretic peptide and brain natriuretic peptide) are organized in tandem in the mouse and human genomes.	Pubmed	0.0044	65.1	0.0114	0.116	1	1	NPPA
Pubmed	11556968	Comparative association analysis reveals that corneodesmosin is more closely associated with psoriasis than HLA-Cw*0602-B*5701 in German families.	Pubmed	0.0044	65.1	0.0114	0.116	1	1	CDSN
Pubmed	21726491	Effects of surgery and chronic disease states on the concentrations and phenotype distribution of a1-acid glycoprotein: studies in patients with breast cancer and patients with chronic inflammatory disease.	GeneRIF	0.0044	65.1	0.0114	0.116	1	1	ORM1
Pubmed	17644284	In vivo evidence of role of bone morphogenetic protein-4 in the mouse ovary.	GeneRIF	0.0044	65.1	0.0114	0.116	1	1	BMP4
Pubmed	1457043	The HXY box regulatory element modulates expression of the murine IA antigen-associated invariant chain in L fibroblasts.	Pubmed	0.0044	65.1	0.0114	0.116	1	1	CD74
Pubmed	22798324	Higher plasma fractalkine is associated with better 6-month outcome from ischemic stroke.	GeneRIF	0.0044	65.1	0.0114	0.116	1	1	CX3CL1
Pubmed	8896573	Mutations in the glutathione synthetase gene cause 5-oxoprolinuria.	Pubmed	0.0044	65.1	0.0114	0.116	1	1	GSS
Pubmed	20442402	Adipocytokine orosomucoid integrates inflammatory and metabolic signals to preserve energy homeostasis by resolving immoderate inflammation.	GeneRIF	0.0044	65.1	0.0114	0.116	1	1	ORM1
Pubmed	18663466	Regulation of the murine TRPP3 channel by voltage, pH, and changes in cell volume.	GeneRIF	0.0044	65.1	0.0114	0.116	1	1	PKD2L1
Pubmed	21472892	Mutation update for the PORCN gene.	GeneRIF	0.0044	65.1	0.0114	0.116	1	1	PORCN
Pubmed	12569161	Inhibition of endothelial lipase causes increased HDL cholesterol levels in vivo.	GeneRIF	0.0044	65.1	0.0114	0.116	1	1	LIPG
Pubmed	23493551	Complete and unidirectional conversion of human embryonic stem cells to trophoblast by BMP4.	GeneRIF	0.0044	65.1	0.0114	0.116	1	1	BMP4
Pubmed	8793505	Localization of bone morphogenetic protein-4 messenger RNA in developing mouse cochlea.	Pubmed	0.0044	65.1	0.0114	0.116	1	1	BMP4
Pubmed	11425865	Delineation of the clotrimazole/TRAM-34 binding site on the intermediate conductance calcium-activated potassium channel, IKCa1.	Pubmed	0.0044	65.1	0.0114	0.116	1	1	KCNN4
Pubmed	17803675	Distribution of potassium ion and water permeable channels at perivascular glia in brain and retina of the Large(myd) mouse.	Pubmed	0.0044	65.1	0.0114	0.116	1	1	LARGE
Pubmed	25291260	Endothelial lipase-384A/C polymorphism is associated with acute coronary syndrome and lipid status in elderly Uygur patients in Xinjiang.	Pubmed	0.0044	65.1	0.0114	0.116	1	1	LIPG
Pubmed	18978175	Minigene analysis of intronic variants in common SPINK1 haplotypes associated with chronic pancreatitis.	GeneRIF	0.0044	65.1	0.0114	0.116	1	1	SPINK1
Pubmed	20052409	Transglutaminase 2, a novel regulator of eicosanoid production in asthma revealed by genome-wide expression profiling of distinct asthma phenotypes.	GeneRIF	0.0044	65.1	0.0114	0.116	1	1	TGM2
Pubmed	18760763	C-terminal deletions in the ALAS2 gene lead to gain of function and cause X-linked dominant protoporphyria without anemia or iron overload.	GeneRIF	0.0044	65.1	0.0114	0.116	1	1	ALAS2
Pubmed	18407550	A novel mutation in AlphaA-crystallin (CRYAA) caused autosomal dominant congenital cataract in a large Chinese family.	GeneRIF	0.0044	65.1	0.0114	0.116	1	1	CRYAA
Pubmed	10192395	Identification of the gene responsible for gelatinous drop-like corneal dystrophy.	Pubmed	0.0044	65.1	0.0114	0.116	1	1	TACSTD2

Category	ID	Name	Source	p-value	q-value Bonferroni	q-value FDR B&H	q-value FDR B&Y	Hit Count in Query List	Hit Count in Genome	Hit in Query List
Pubmed	16549784	Properties of human connexin 31, which is implicated in hereditary dermatological disease and deafness.	GeneRIF	0.0044	65.1	0.0114	0.116	1	1	GJB3
Pubmed	3494717	DQ beta sequences in HLA-DR4 haplotypes.	Pubmed	0.0044	65.1	0.0114	0.116	1	1	HLA-DQB1
Pubmed	24491427	IFI16 mis-localization can be a contributing factor to hepatocellular carcinoma progression.	Pubmed	0.0044	65.1	0.0114	0.116	1	1	IFI16
Pubmed	23912959	CX3CL1 expression is associated with poor outcome in breast cancer patients.	Pubmed	0.0044	65.1	0.0114	0.116	1	1	CX3CL1
Pubmed	24312600	Epigenetic Regulation of aA-crystallin in High Myopia-Induced Dark Nuclear Cataract.	Pubmed	0.0044	65.1	0.0114	0.116	1	1	CRYAA
Pubmed	25321086	P75 neurotrophin receptor expression in squamous cell carcinoma.	Pubmed	0.0044	65.1	0.0114	0.116	1	1	NGFR
Pubmed	19617321	Initial evaluation of 11C-DPA-713, a novel TSPO PET ligand, in humans.	GeneRIF	0.0044	65.1	0.0114	0.116	1	1	TSPO
Pubmed	11999355	PCR-based analysis of alpha-thalassemia in Southern Taiwan.	GeneRIF	0.0044	65.1	0.0114	0.116	1	1	HBB
Pubmed	16724628	Adenosine deaminase polymorphism and the relationship of total immunoglobulin E with skin prick test: a study on school children.	GeneRIF	0.0044	65.1	0.0114	0.116	1	1	ADA
Pubmed	19462004	Overexpression of the mitochondrial T3 receptor induces skeletal muscle atrophy during aging.	GeneRIF	0.0044	65.1	0.0114	0.116	1	1	THRA
Pubmed	17681393	Epidemiology and genetic risk of type 1 diabetes among children in Aragon community, Spain.	GeneRIF	0.0044	65.1	0.0114	0.116	1	1	HLA-DQB1
Pubmed	22982576	Vascular peroxidase 1 catalyzes the formation of hypohalous acids: characterization of its substrate specificity and enzymatic properties.	GeneRIF	0.0044	65.1	0.0114	0.116	1	1	PXDN
Pubmed	20975052	DQB1*0602 predicts interindividual differences in physiologic sleep, sleepiness, and fatigue.	GeneRIF	0.0044	65.1	0.0114	0.116	1	1	HLA-DQB1
Pubmed	15538382	FOXO transcription factor activation by oxidative stress mediated by the small GTPase Ral and JNK.	GeneRIF	0.0044	65.1	0.0114	0.116	1	1	FOXO4
Pubmed	15654845	Deafferentation and neurotrophin-mediated intraspinal sprouting: a central role for the p75 neurotrophin receptor.	Pubmed	0.0044	65.1	0.0114	0.116	1	1	NGFR
Pubmed	9126337	The cloning and characterization of a murine secretory leukocyte protease inhibitor cDNA.	Pubmed	0.0044	65.1	0.0114	0.116	1	1	SLPI
Pubmed	20872081	Translocator protein PET imaging for glial activation in multiple sclerosis.	GeneRIF	0.0044	65.1	0.0114	0.116	1	1	TSPO
Pubmed	8522332	Germline mosaicism for an alanine to valine substitution at residue beta 140 in hemoglobin Puttelange, a new variant with high oxygen affinity.	Pubmed	0.0044	65.1	0.0114	0.116	1	1	HBB
Pubmed	21816559	Interactions of the LIPG 584C>T polymorphism and alcohol consumption on serum lipid levels.	GeneRIF	0.0044	65.1	0.0114	0.116	1	1	LIPG
Pubmed	21307873	Protease sensitivity of corneodesmosin variants encoded by the six more common CDSN haplotypes.	Pubmed	0.0044	65.1	0.0114	0.116	1	1	CDSN
Pubmed	25093959	Spectroscopic and computational investigation of iron(III) cysteine dioxygenase: implications for the nature of the putative superoxo-Fe(III) intermediate.	Pubmed	0.0044	65.1	0.0114	0.116	1	1	CDO1
Pubmed	17762646	A coding polymorphism of the kallikrein 1 gene is associated with essential hypertension: a tagging SNP-based association study in a Chinese Han population.	GeneRIF	0.0044	65.1	0.0114	0.116	1	1	KLK1
Pubmed	23838122	proNGF inhibits proliferation and oligodendrogenesis of postnatal hippocampal neural stem/progenitor cells through p75NTR in vitro.	Pubmed	0.0044	65.1	0.0114	0.116	1	1	NGFR
Pubmed	15772937	Computer-based detection of neonatal changes to branching morphogenesis reveals different mechanisms of and predicts prostate enlargement in mice haplo-insufficient for bone morphogenetic protein 4.	Pubmed	0.0044	65.1	0.0114	0.116	1	1	BMP4
Pubmed	2599880	Hemoglobin Windsor or beta 11 (A8)Val----Asp: a new unstable beta-chain hemoglobin variant producing a hemolytic anemia.	Pubmed	0.0044	65.1	0.0114	0.116	1	1	HBB
Pubmed	21205888	Direct evidence for functional smooth muscle myosin II in the 10S self-inhibited monomeric conformation in airway smooth muscle cells.	GeneRIF	0.0044	65.1	0.0114	0.116	1	1	MYH11
Pubmed	7485380	gamma-Glutamyl transpeptidase. What does the organization and expression of a multipromoter gene tell us about its functions?	Pubmed	0.0044	65.1	0.0114	0.116	1	1	GGT1
Pubmed	20868635	Candidate gene analysis identifies a polymorphism in HLA-DQB1 associated with clozapine-induced agranulocytosis.	GeneRIF	0.0044	65.1	0.0114	0.116	1	1	HLA-DQB1
Pubmed	18381244	Identification of a novel frameshift mutation at codon 53 (-T) in the beta-globin gene causing dominantly inherited beta-thalassemia in a Chinese Miao family.	GeneRIF	0.0044	65.1	0.0114	0.116	1	1	HBB
Pubmed	20613781	Effects of protein coding polymorphisms in the kallikrein 1 gene on baseline blood pressure and antihypertensive response to irbesartan in Chinese hypertensive patients.	GeneRIF	0.0044	65.1	0.0114	0.116	1	1	KLK1
Pubmed	23993960	Transglutaminase 2 as a novel activator of LRP6/ß catenin signaling.	Pubmed	0.0044	65.1	0.0114	0.116	1	1	TGM2
Pubmed	24687926	SPINK1 protein expression and prostate cancer progression.	Pubmed	0.0044	65.1	0.0114	0.116	1	1	SPINK1
Pubmed	24213244	Isogenic human pluripotent stem cell pairs reveal the role of a KCNH2 mutation in long-QT syndrome.	Pubmed	0.0044	65.1	0.0114	0.116	1	1	KCNH2
Pubmed	23279037	Atrial natriuretic peptide as a marker of heart failure in children with left ventricular volume overload.	GeneRIF	0.0044	65.1	0.0114	0.116	1	1	NPPA
Pubmed	23940561	Polymorphism of ORM1 is associated with the pharmacokinetics of telmisartan.	Pubmed	0.0044	65.1	0.0114	0.116	1	1	ORM1
Pubmed	20451610	Transglutaminase 2 protects against ischemic stroke.	GeneRIF	0.0044	65.1	0.0114	0.116	1	1	TGM2
Pubmed	21317765	Involvement of the aldo-keto reductase, AKR1B10, in mitomycin-c resistance through reactive oxygen species-dependent mechanisms.	GeneRIF	0.0044	65.1	0.0114	0.116	1	1	AKR1B10
Pubmed	22511307	Junctophilin 2 knockdown interfere with mitochondrium status in ESC-CMs and cardiogenesis of ES cells.	GeneRIF	0.0044	65.1	0.0114	0.116	1	1	JPH2

Category	ID	Name	Source	p-value	q-value Bonferroni	q-value FDR B&H	q-value FDR B&Y	Hit Count in Query List	Hit Count in Genome	Hit in Query List
Pubmed	1307263	Dinucleotide repeat polymorphism in the human thyroid hormone receptor alpha gene (THRA1) on chromosome 17.	Pubmed	0.0044	65.1	0.0114	0.116	1	1	THRA
Pubmed	18473240	A +8 (-->CT) mutation within the 5' untranslated region of beta-globin down-regulates the mRNA transcription.	GeneRIF	0.0044	65.1	0.0114	0.116	1	1	HBB
Pubmed	23386610	Quantitative characterization of the activation steps of mannan-binding lectin (MBL)-associated serine proteases (MASPs) points to the central role of MASP-1 in the initiation of the complement lectin pathway.	GeneRIF	0.0044	65.1	0.0114	0.116	1	1	MASP1
Pubmed	8551230	HLA-DQ8 transgenic mice are highly susceptible to collagen-induced arthritis: a novel model for human polyarthritis.	Pubmed	0.0044	65.1	0.0114	0.116	1	1	HLA-DQB1
Pubmed	19384300	High expression of tumour-associated trypsin inhibitor correlates with liver metastasis and poor prognosis in colorectal cancer.	GeneRIF	0.0044	65.1	0.0114	0.116	1	1	SPINK1
Pubmed	7570001	T lymphocyte-directed gene therapy for ADA-SCID: initial trial results after 4 years.	Pubmed	0.0044	65.1	0.0114	0.116	1	1	ADA
Pubmed	2898474	gamma-Glutamyl transpeptidase: a single copy gene in the rat and a multigene family in the human genome.	Pubmed	0.0044	65.1	0.0114	0.116	1	1	GGT1
Pubmed	14960588	A Kruppel-like factor KLF15 contributes fasting-induced transcriptional activation of mitochondrial acetyl-CoA synthetase gene AceCS2.	GeneRIF	0.0044	65.1	0.0114	0.116	1	1	ACSS1
Pubmed	23348515	Molecular expression and characterization of erythroid-specific 5-aminolevulinate synthase gain-of-function mutations causing X-linked protoporphyria.	GeneRIF	0.0044	65.1	0.0114	0.116	1	1	ALAS2
Pubmed	15970210	Antisense inhibition of transglutaminase 2 affects development of mouse embryo submandibular gland in organ culture.	Pubmed	0.0044	65.1	0.0114	0.116	1	1	TGM2
Pubmed	18391064	Arthritis induced by posttranslationally modified (citrullinated) fibrinogen in DR4-IE transgenic mice.	Pubmed	0.0044	65.1	0.0114	0.116	1	1	HLA-DQB1
Pubmed	19159624	Identification of an ancient Bmp4 mesoderm enhancer located 46 kb from the promoter.	GeneRIF	0.0044	65.1	0.0114	0.116	1	1	BMP4
Pubmed	11238910	Cardiomyopathy in Irx4-deficient mice is preceded by abnormal ventricular gene expression.	Pubmed	0.00454	67.2	0.0118	0.12	2	23	NPPA,ACTA1
Pubmed	24038871	Regulation of mesenchymal-to-epithelial transition by PARAXIS during somitogenesis.	Pubmed	0.00454	67.2	0.0118	0.12	2	23	MID1,ARHGEF6
Pubmed	12805214	Noggin overexpression inhibits eyelid opening by altering epidermal apoptosis and differentiation.	GeneRIF	0.00454	67.2	0.0118	0.12	2	23	BMP4,NGFR
Pubmed	19372376	Upstream open reading frames cause widespread reduction of protein expression and are polymorphic among humans.	GeneRIF	0.00454	67.2	0.0118	0.12	2	23	SPINK1,HBB
Pubmed	20805985	Ablation of Dicer from murine Schwann cells increases their proliferation while blocking myelination.	GeneRIF	0.00454	67.2	0.0118	0.12	2	23	NGFR,CD68
Pubmed	23471215	Conditional mutagenesis of Gata6 in SF1-positive cells causes gonadal-like differentiation in the adrenal cortex of mice.	GeneRIF	0.00454	67.2	0.0118	0.12	2	23	INHBB,CYP11A1
Pubmed	20554972	Vegf regulates embryonic erythroid development through Gata1 modulation.	GeneRIF	0.00454	67.2	0.0118	0.12	2	23	BMP4,ALAS2
Pubmed	18723448	Tbx3 is required for outflow tract development.	GeneRIF	0.00494	73.1	0.0128	0.13	2	24	NPPA,BMP4
Pubmed	21420948	Rbpj regulates development of prosensory cells in the mammalian inner ear.	GeneRIF	0.00494	73.1	0.0128	0.13	2	24	BMP4,NGFR
Pubmed	23274689	Hes1 in the somatic cells of the murine ovary is necessary for oocyte survival and maturation.	GeneRIF	0.00494	73.1	0.0128	0.13	2	24	INHBB,NGFR
Pubmed	21803035	Kruppel-like factor 5 is required for formation and differentiation of the bladder urothelium.	GeneRIF	0.00494	73.1	0.0128	0.13	2	24	SMTN,BMP4
Pubmed	22130515	Tbx2 and Tbx3 induce atrioventricular myocardial development and endocardial cushion formation.	GeneRIF	0.00536	79.2	0.0139	0.141	2	25	NPPA,FBLN2
Pubmed	17698011	Retrograde BMP signaling regulates trigeminal sensory neuron identities and the formation of precise face maps.	GeneRIF	0.00536	79.2	0.0139	0.141	2	25	BMP4,SCN3B
Pubmed	21664901	Redundant and dosage sensitive requirements for Fgf3 and Fgf10 in cardiovascular development.	GeneRIF	0.00536	79.2	0.0139	0.141	2	25	NPPA,BMP4
Pubmed	22485132	Fstl1 antagonizes BMP signaling and regulates ureter development.	GeneRIF	0.00536	79.2	0.0139	0.141	2	25	MYH11,BMP4
Pubmed	16502470	Human colostrum: identification of minor proteins in the aqueous phase by proteomics.	Pubmed	0.00548	81.1	0.0142	0.145	4	160	PSAP,GGT1,ORM1,ORM2
Pubmed	16335952	Human plasma N-glycoproteome analysis by immunoaffinity subtraction, hydrazide chemistry, and mass spectrometry.	Pubmed	0.0056	82.9	0.0145	0.148	5	257	APMAP,ORM1,ORM2,MFAP4,LIPG
Pubmed	6690234	Abstracts of meeting presentations: Human gene mapping 7, Los Angeles Conference (1983), Seventh International Workshop on Human Gene Mapping.	Pubmed	0.00578	85.6	0.0149	0.152	2	26	ADA,THRA
Pubmed	9039502	Prediction of the coding sequences of unidentified human genes. VI. The coding sequences of 80 new genes (KIAA0201-KIAA0280) deduced by analysis of cDNA clones from cell line KG-1 and brain.	Pubmed	0.00606	89.6	0.0149	0.152	3	84	SPOCK2,PXDN,LHFPL2
Pubmed	20012522	Interactions of viral protein U (Vpu) with cellular factors.	Pubmed	0.00623	92.2	0.0149	0.152	2	27	HLA-DQB1,CD74
Pubmed	21983003	Myocardial Tbx20 regulates early atrioventricular canal formation and endocardial epithelial-mesenchymal transition via Bmp2.	GeneRIF	0.00623	92.2	0.0149	0.152	2	27	NPPA,BMP4
Pubmed	15340161	Signal peptide prediction based on analysis of experimentally verified cleavage sites.	GeneRIF	0.00626	92.7	0.0149	0.152	5	264	RTN4R,SPOCK2,AMICA1,MFAP4,MPZL3
Pubmed	17207965	hORFeome v3.1: a resource of human open reading frames representing over 10,000 human genes.	Pubmed	0.00636	94.2	0.0149	0.152	15	1663	KLK15,CDO1,APMAP,CRYAA,BMP4,ORM2,MFAP4,GJB3,ANGPTL2,LIPG,ENPP5,FBXO32,HYI,CTSK,METTL7A
Pubmed	11544199	Construction of long-transcript enriched cDNA libraries from submicrogram amounts of total RNAs by a universal PCR amplification method.	Pubmed	0.00638	94.3	0.0149	0.152	10	899	SLC43A2,PSAP,SPOCK2,IFI16,AKAP12,SMTN,EHD3,MPZL3,DCLK1,ARHGEF6
Pubmed	17951031	Fgfr2b mediated epithelial-mesenchymal interactions coordinate tooth morphogenesis and dental trigeminal axon patterning.	GeneRIF	0.00669	99	0.0149	0.152	2	28	BMP4,NGFR

Category	ID	Name	Source	p-value	q-value Bonferroni	q-value FDR B&H	q-value FDR B&Y	Hit Count in Query List	Hit Count in Genome	Hit in Query List
Pubmed	16140986	Centronuclear myopathy in mice lacking a novel muscle-specific protein kinase transcriptionally regulated by MEF2.	GeneRIF	0.00669	99	0.0149	0.152	2	28	FOXO4,FBXO32
Pubmed	15342556	Sequence comparison of human and mouse genes reveals a homologous block structure in the promoter regions.	Pubmed	0.00687	102	0.0149	0.152	11	1055	DPT,SLC39A2,MRGPRF,SPOCK2,MFAP4,EDEM2,FOXO4,ALAS2,MMP7,KLK1,SCN3B
Pubmed	20201926	Human variation in alcohol response is influenced by variation in neuronal signaling genes.	GeneRIF	0.00691	102	0.0149	0.152	4	171	ACTA1,FBLN2,PRELP,LARGE
Pubmed	19453261	High-density association study of 383 candidate genes for volumetric BMD at the femoral neck and lumbar spine among older men.	GeneRIF	0.00699	103	0.0149	0.152	6	383	CYP11A1,BMP4,THRA,GPNMB,FBXO32,CTSK
Pubmed	15525990	Baf60c is essential for function of BAF chromatin remodelling complexes in heart development.	GeneRIF	0.00716	106	0.0149	0.152	2	29	NPPA,BMP4
Pubmed	15843414	Murine T-box transcription factor Tbx20 acts as a repressor during heart development, and is essential for adult heart integrity, function and adaptation.	GeneRIF	0.00765	113	0.0149	0.152	2	30	NPPA,BMP4
Pubmed	16454711	Modelling thymic HIV-1 Nef effects.	Pubmed	0.00765	113	0.0149	0.152	2	30	HLA-DQB1,CD74
Pubmed	18462699	ER71 acts downstream of BMP, Notch, and Wnt signaling in blood and vessel progenitor specification.	Pubmed	0.00765	113	0.0149	0.152	2	30	MYH11,BMP4
Pubmed	21922073	Misdirection of membrane trafficking by HIV-1 Vpu and Nef: Keys to viral virulence and persistence.	Pubmed	0.00816	121	0.0149	0.152	2	31	HLA-DQB1,CD74
Pubmed	23426374	Sma- and Mad-related protein 7 (Smad7) is required for embryonic eye development in the mouse.	GeneRIF	0.00816	121	0.0149	0.152	2	31	CRYAA,BMP4
Pubmed	24058167	Lung epithelial branching program antagonizes alveolar differentiation.	Pubmed	0.00816	121	0.0149	0.152	2	31	NAPSA,BMP4
Pubmed	24292274	A genome-wide association study identifies multiple susceptibility loci for chronic lymphocytic leukemia.	Pubmed	0.00816	121	0.0149	0.152	2	31	ACTA1,SCN3B
Pubmed	21423176	Analysis of the myosin-II-responsive focal adhesion proteome reveals a role for ß-Pix in negative regulation of focal adhesion maturation.	GeneRIF	0.00857	127	0.0149	0.152	5	285	PROCR,AKAP12,EHD3,TGM2,CAPN5
Pubmed	18927151	BMP signaling negatively regulates bone mass through sclerostin by inhibiting the canonical Wnt pathway.	GeneRIF	0.00868	128	0.0149	0.152	2	32	BMP4,CTSK
Pubmed	22494469	The influence of the HLA-DRB, HLA-DQB and polymorphic positions of the HLA-DRß1 and HLA-DQß1 molecules on risk of Iranian type 1 diabetes mellitus patients.	GeneRIF	0.00875	129	0.0149	0.152	1	2	HLA-DQB1
Pubmed	17372897	Defective antitumor responses in CX3CR1-deficient mice.	Pubmed	0.00875	129	0.0149	0.152	1	2	CX3CL1
Pubmed	19903799	Clinical relevance of thyroid dysfunction in human haematopoiesis: biochemical and molecular studies.	GeneRIF	0.00875	129	0.0149	0.152	1	2	THRA
Pubmed	16956833	Influence of Ggamma-158C --> and beta-(AT)x(T)y globin gene polymorphisms on HbF levels in Italian beta-thalassemia carriers and wild-type subjects.	GeneRIF	0.00875	129	0.0149	0.152	1	2	HBB
Pubmed	11591792	Cockroach allergen-induced eosinophilic airway inflammation in HLA-DQ/human CD4(+) transgenic mice.	Pubmed	0.00875	129	0.0149	0.152	1	2	HLA-DQB1
Pubmed	11124840	HLA class II genes determine the natural variance of hepatitis C viral load.	GeneRIF	0.00875	129	0.0149	0.152	1	2	HLA-DQB1
Pubmed	10777582	Identification and cloning of a new calmodulin-like protein from human epidermis.	Pubmed	0.00875	129	0.0149	0.152	1	2	CALML5
Pubmed	16452636	Cell surface transglutaminase promotes RhoA activation via integrin clustering and suppression of the Src-p190RhoGAP signaling pathway.	GeneRIF	0.00875	129	0.0149	0.152	1	2	TGM2
Pubmed	19609566	Expression of ECM proteins fibulin-1 and -2 in acute and chronic liver disease and in cultured rat liver cells.	GeneRIF	0.00875	129	0.0149	0.152	1	2	FBLN2
Pubmed	15745835	Aldose reductase (AC)n gene polymorphism and susceptibility to diabetic retinopathy in Type 2 diabetes in Caucasians.	GeneRIF	0.00875	129	0.0149	0.152	1	2	AKR1B10
Pubmed	17484621	HLA-DQA1 and -DQB1 allele typing in southern Taiwanese women with breast cancer.	GeneRIF	0.00875	129	0.0149	0.152	1	2	HLA-DQB1
Pubmed	16670303	Peripheral tolerance via the anterior chamber of the eye: role of B cells in MHC class I and II antigen presentation.	Pubmed	0.00875	129	0.0149	0.152	1	2	HLA-DQB1
Pubmed	18312478	Multiple sclerosis in Gypsies from southern Spain: prevalence, mitochondrial DNA haplogroups and HLA class II association.	GeneRIF	0.00875	129	0.0149	0.152	1	2	HLA-DQB1
Pubmed	14634009	Gamma-glutamyltranspeptidase stimulates receptor activator of nuclear factor-kappaB ligand expression independent of its enzymatic activity and serves as a pathological bone-resorbing factor.	GeneRIF	0.00875	129	0.0149	0.152	1	2	GGT1
Pubmed	17717295	Natural killer cells and CD4+ T-cells modulate collateral artery development.	Pubmed	0.00875	129	0.0149	0.152	1	2	HLA-DQB1
Pubmed	19165649	Immunohistological features of hip synovitis in ankylosing spondylitis with advanced hip involvement.	GeneRIF	0.00875	129	0.0149	0.152	1	2	CD68
Pubmed	16232198	Establishment of an immortalized cell line from a precancerous lesion of lung adenocarcinoma, and genes highly expressed in the early stages of lung adenocarcinoma development.	GeneRIF	0.00875	129	0.0149	0.152	1	2	TACSTD2
Pubmed	24294368	MiR-195 inhibits proliferation and growth and induces apoptosis of endometrial stromal cells by targeting FKN.	Pubmed	0.00875	129	0.0149	0.152	1	2	CX3CL1
Pubmed	15057556	Regulation of the MID1 protein function is fine-tuned by a complex pattern of alternative splicing.	Pubmed	0.00875	129	0.0149	0.152	1	2	MID1
Pubmed	20724702	A new role for the muscle repair protein dysferlin in endothelial cell adhesion and angiogenesis.	GeneRIF	0.00875	129	0.0149	0.152	1	2	DYSF
Pubmed	11772392	Regulation of epithelial and lymphocyte cell adhesion by adenosine deaminase-CD26 interaction.	Pubmed	0.00875	129	0.0149	0.152	1	2	ADA
Pubmed	19416966	14-3-3 protein masks the DNA binding interface of forkhead transcription factor FOXO4.	GeneRIF	0.00875	129	0.0149	0.152	1	2	FOXO4

Category	ID	Name	Source	p-value	q-value Bonferroni	q-value FDR B&H	q-value FDR B&Y	Hit Count in Query List	Hit Count in Genome	Hit in Query List
Pubmed	21935919	Plasma hydroxyproline, MMP-7 and collagen I as novel predictive risk markers of hepatobiliary disease-associated cholangiocarcinoma.	GeneRIF	0.00875	129	0.0149	0.152	1	2	MMP7
Pubmed	1281863	Expression of growth-associated protein 43 and nerve growth factor receptor in human skin: a comparative immunohistochemical investigation.	Pubmed	0.00875	129	0.0149	0.152	1	2	NGFR
Pubmed	22584896	The angiogenesis suppressor gene AKAP12 is under the epigenetic control of HDAC7 in endothelial cells.	GeneRIF	0.00875	129	0.0149	0.152	1	2	AKAP12
Pubmed	18414380	Inhibition of the rapid component of the delayed rectifier potassium current in ventricular myocytes by angiotensin II via the AT1 receptor.	GeneRIF	0.00875	129	0.0149	0.152	1	2	KCNH2
Pubmed	16494870	Androgen receptor auto-regulates its expression by a negative feedback loop through upregulation of IFI16 protein.	GeneRIF	0.00875	129	0.0149	0.152	1	2	IFI16
Pubmed	15833172	[Possible association between HLA-HRB1 and DQB1 genes frequency and susceptibility or resistance to Helicobacter pylori infection in Kunming Yi ethnic group children].	GeneRIF	0.00875	129	0.0149	0.152	1	2	HLA-DQB1
Pubmed	22072684	CX3CL1 is neuroprotective in permanent focal cerebral ischemia in rodents.	Pubmed	0.00875	129	0.0149	0.152	1	2	CX3CL1
Pubmed	18006858	Tumor necrosis factor receptor-2 signaling attenuates vein graft neointima formation by promoting endothelial recovery.	GeneRIF	0.00875	129	0.0149	0.152	1	2	NGFR
Pubmed	11556984	HLA-DQ alleles in patients with celiac disease in Turkey.	GeneRIF	0.00875	129	0.0149	0.152	1	2	HLA-DQB1
Pubmed	21947948	Contribution of ß-globin cluster polymorphisms to raise fetal hemoglobin levels in normal adults.	GeneRIF	0.00875	129	0.0149	0.152	1	2	HBB
Pubmed	12687003	The BMP antagonist noggin regulates cranial suture fusion.	GeneRIF	0.00875	129	0.0149	0.152	1	2	BMP4
Pubmed	23473316	Anatomical plasticity of adult brain is titrated by Nogo Receptor 1.	GeneRIF	0.00875	129	0.0149	0.152	1	2	RTN4R
Pubmed	23218978	Major histocompatibility complex class II polymorphisms are associated with the development of anti-resorptive agent-induced osteonecrosis of the jaw.	GeneRIF	0.00875	129	0.0149	0.152	1	2	HLA-DQB1
Pubmed	22750506	Interactions among secretory immunoglobulin A, CD71, and transglutaminase-2 affect permeability of intestinal epithelial cells to gliadin peptides.	GeneRIF	0.00875	129	0.0149	0.152	1	2	TGM2
Pubmed	9442029	Identification of the eukaryotic initiation factor 5A as a retinoic acid-stimulated cellular binding partner for tissue transglutaminase II.	Pubmed	0.00875	129	0.0149	0.152	1	2	TGM2
Pubmed	17129633	VDR polymorphisms influence the immune response in type 1 diabetic children from Santiago, Chile.	GeneRIF	0.00875	129	0.0149	0.152	1	2	HLA-DQB1
Pubmed	19819281	Association of human leukocyte antigen class II alleles with response to immunosuppressive therapy in Korean aplastic anemia patients.	GeneRIF	0.00875	129	0.0149	0.152	1	2	HLA-DQB1
Pubmed	11953202	[Relationship between HLA-DQA1, -DQB1 genes polymorphism and susceptibility to bronchial asthma among Northern Hans].	GeneRIF	0.00875	129	0.0149	0.152	1	2	HLA-DQB1
Pubmed	11258672	The expanded human kallikrein (KLK) gene family: genomic organisation, tissue-specific expression and potential functions.	Pubmed	0.00875	129	0.0149	0.152	1	2	KLK1
Pubmed	16989826	Vaginally administered estroprogestinic decreases serum inhibin A and inhibin B levels and reduces endometrial thickness.	GeneRIF	0.00875	129	0.0149	0.152	1	2	INHBB
Pubmed	24688054	Muscarinic receptor activation increases hERG channel expression through phosphorylation of ubiquitin ligase Nedd4-2.	Pubmed	0.00875	129	0.0149	0.152	1	2	KCNH2
Pubmed	21748758	Biological significance of perineural invasion (PNI) in prostate cancer.	GeneRIF	0.00875	129	0.0149	0.152	1	2	CD74
Pubmed	23836275	Association of breastfeeding with higher serum inhibin B level at adolescence.	Pubmed	0.00875	129	0.0149	0.152	1	2	INHBB
Pubmed	9877162	Napsins: new human aspartic proteinases. Distinction between two closely related genes.	Pubmed	0.00875	129	0.0149	0.152	1	2	NAPSA
Pubmed	3268409	XY follicle cells in ovaries of XX----XY female mouse chimaeras.	Pubmed	0.00875	129	0.0149	0.152	1	2	BMP4
Pubmed	15793791	[Relationship between the microsatellite polymorphism of CYP11 alpha gene and the pathogenesis of hyperandrogenism of polycystic ovary syndrome in Chinese].	GeneRIF	0.00875	129	0.0149	0.152	1	2	CYP11A1
Pubmed	24655482	Microglial Cx3cr1 knockout reduces prion disease incubation time in mice.	Pubmed	0.00875	129	0.0149	0.152	1	2	CX3CL1
Pubmed	18305125	SIX2 and BMP4 mutations associate with anomalous kidney development.	GeneRIF	0.00875	129	0.0149	0.152	1	2	BMP4
Pubmed	2169733	Isolation and functional expression of the human atrial natriuretic peptide clearance receptor cDNA.	Pubmed	0.00875	129	0.0149	0.152	1	2	NPPA
Pubmed	12134251	Polymorphisms of the human leukocyte antigen DRB1 and DQB1 genes and the natural history of human papillomavirus infection.	GeneRIF	0.00875	129	0.0149	0.152	1	2	HLA-DQB1
Pubmed	16237075	CD4 T cell-independent antibody response promotes resolution of primary influenza infection and helps to prevent reinfection.	Pubmed	0.00875	129	0.0149	0.152	1	2	HLA-DQB1
Pubmed	21917268	The association of HLA-DQA1*0401 and DQB1*0604 with thymomatous myasthenia gravis in northern Chinese patients.	GeneRIF	0.00875	129	0.0149	0.152	1	2	HLA-DQB1
Pubmed	19616314	Does the DRB1*1501 allele confer more severe and faster progression in primary progressive multiple sclerosis patients? HLA in primary progressive multiple sclerosis.	GeneRIF	0.00875	129	0.0149	0.152	1	2	HLA-DQB1
Pubmed	12813057	Saposin C is required for normal resistance of acid beta-glucosidase to proteolytic degradation.	GeneRIF	0.00875	129	0.0149	0.152	1	2	PSAP
Pubmed	20552021	Haptoglobin and sickle cell polymorphisms and risk of active trachoma in Gambian children.	GeneRIF	0.00875	129	0.0149	0.152	1	2	HBB
Pubmed	22044760	CD68 and CD163 as prognostic factors for Korean patients with Hodgkin lymphoma.	GeneRIF	0.00875	129	0.0149	0.152	1	2	CD68
Pubmed	22966085	Mannan-binding lectin-associated serine protease (MASP)-1 is crucial for lectin pathway activation in human serum, whereas neither MASP-1 nor MASP-3 is required for alternative pathway function.	GeneRIF	0.00875	129	0.0149	0.152	1	2	MASP1

Category	ID	Name	Source	p-value	q-value Bonferroni	q-value FDR B&H	q-value FDR B&Y	Hit Count in Query List	Hit Count in Genome	Hit in Query List
Pubmed	19508690	The myelin protein MAL affects peripheral nerve myelination: a new player influencing p75 neurotrophin receptor expression.	GeneRIF	0.00875	129	0.0149	0.152	1	2	NGFR
Pubmed	23278646	HLA-DRB1 and HLA-DQB1 genes on susceptibility to and protection from allergic bronchopulmonary aspergillosis in patients with cystic fibrosis.	GeneRIF	0.00875	129	0.0149	0.152	1	2	HLA-DQB1
Pubmed	10633853	Effects of BMP-7 on mouse tooth mesenchyme and chick mandibular mesenchyme.	Pubmed	0.00875	129	0.0149	0.152	1	2	BMP4
Pubmed	11706034	Heparan sulfate proteoglycans retain Noggin at the cell surface: a potential mechanism for shaping bone morphogenetic protein gradients.	Pubmed	0.00875	129	0.0149	0.152	1	2	BMP4
Pubmed	9346914	The interaction of the molecular chaperone, alpha-crystallin, with molten globule states of bovine alpha-lactalbumin.	Pubmed	0.00875	129	0.0149	0.152	1	2	CRYAA
Pubmed	22027433	BMP signaling induces visceral endoderm differentiation of XEN cells and parietal endoderm.	GeneRIF	0.00875	129	0.0149	0.152	1	2	BMP4
Pubmed	19228595	While dysferlin and myoferlin are coexpressed in the human placenta, only dysferlin expression is responsive to trophoblast fusion in model systems.	GeneRIF	0.00875	129	0.0149	0.152	1	2	DYSF
Pubmed	12590979	Intercellular adhesion molecule-1 K469E polymorphism: study of association with multiple sclerosis.	GeneRIF	0.00875	129	0.0149	0.152	1	2	HLA-DQB1
Pubmed	23117657	Arterial and venous endothelia display differential functional fractalkine (CX3CL1) expression by angiotensin-II.	GeneRIF	0.00875	129	0.0149	0.152	1	2	CX3CL1
Pubmed	18650388	Generation of mTert-GFP mice as a model to identify and study tissue progenitor cells.	Pubmed	0.00875	129	0.0149	0.152	1	2	CD276
Pubmed	10220144	Novel KCNQ1 and HERG missense mutations in Dutch long-QT families.	Pubmed	0.00875	129	0.0149	0.152	1	2	KCNH2
Pubmed	9368064	Growth factors stimulate tyrosine dephosphorylation of p75 and its dissociation from the SH2 domain of Grb2.	Pubmed	0.00875	129	0.0149	0.152	1	2	NGFR
Pubmed	24760011	In vivo substrates of the lens molecular chaperones aA-crystallin and aB-crystallin.	Pubmed	0.00875	129	0.0149	0.152	1	2	CRYAA
Pubmed	18987854	Soluble HLA-DQ2 expressed in S2 cells copurifies with a high affinity insect cell derived protein.	GeneRIF	0.00875	129	0.0149	0.152	1	2	HLA-DQB1
Pubmed	16879301	Intravenous synthetic peptide MBP8298 delayed disease progression in an HLA Class II-defined cohort of patients with progressive multiple sclerosis: results of a 24-month double-blind placebo-controlled clinical trial and 5 years of follow-up treatment.	GeneRIF	0.00875	129	0.0149	0.152	1	2	HLA-DQB1
Pubmed	9973415	MHC class II-dependent NK1.1+ gammadelta T cells are induced in mice by Salmonella infection.	Pubmed	0.00875	129	0.0149	0.152	1	2	HLA-DQB1
Pubmed	16766710	The doublecortin and doublecortin-like kinase 1 genes cooperate in murine hippocampal development.	Pubmed	0.00875	129	0.0149	0.152	1	2	DCLK1
Pubmed	11375130	Attenuated processing of proglucagon and glucagon-like peptide-1 in carboxypeptidase E-deficient mice.	Pubmed	0.00875	129	0.0149	0.152	1	2	CPE
Pubmed	15069073	Tissue transglutaminase has intrinsic kinase activity: identification of transglutaminase 2 as an insulin-like growth factor-binding protein-3 kinase.	GeneRIF	0.00875	129	0.0149	0.152	1	2	TGM2
Pubmed	25164659	Plasticity of binocularity and visual acuity are differentially limited by nogo receptor.	Pubmed	0.00875	129	0.0149	0.152	1	2	RTN4R
Pubmed	24599476	Macroglia-microglia interactions via TSPO signaling regulates microglial activation in the mouse retina.	Pubmed	0.00875	129	0.0149	0.152	1	2	TSPO
Pubmed	12475945	Characterization of human alpha-dystrobrevin isoforms in HL-60 human promyelocytic leukemia cells undergoing granulocytic differentiation.	GeneRIF	0.00875	129	0.0149	0.152	1	2	ACTA1
Pubmed	19812317	Mature BDNF, but not proBDNF, reduces excitability of fast-spiking interneurons in mouse dentate gyrus.	Pubmed	0.00875	129	0.0149	0.152	1	2	NGFR
Pubmed	7916668	Human smooth muscle myosin heavy chain isoforms as molecular markers for vascular development and atherosclerosis.	Pubmed	0.00875	129	0.0149	0.152	1	2	MYH11
Pubmed	20049897	Regulation of ATPase activity of transglutaminase 2 by MT1-MMP: implications for mineralization of MC3T3-E1 osteoblast cultures.	GeneRIF	0.00875	129	0.0149	0.152	1	2	TGM2
Pubmed	20875398	The p75 neurotrophin receptor is localized to primary cilia in adult murine hippocampal dentate gyrus granule cells.	GeneRIF	0.00875	129	0.0149	0.152	1	2	NGFR
Pubmed	23224146	Transglutaminase 2 and NF-¿B: an odd couple that shapes breast cancer phenotype.	GeneRIF	0.00875	129	0.0149	0.152	1	2	TGM2
Pubmed	8413626	The daf-4 gene encodes a bone morphogenetic protein receptor controlling C. elegans dauer larva development.	Pubmed	0.00875	129	0.0149	0.152	1	2	BMP4
Pubmed	2892884	Polymorphism of the HLA-D region in American blacks. A DR3 haplotype generated by recombination.	Pubmed	0.00875	129	0.0149	0.152	1	2	HLA-DQB1
Pubmed	17934455	Roles for the pro-neurotrophin receptor sortilin in neuronal development, aging and brain injury.	GeneRIF	0.00875	129	0.0149	0.152	1	2	NGFR
Pubmed	23793622	Cholesterol regulates HERG K+ channel activation by increasing phospholipase C ß1 expression.	Pubmed	0.00875	129	0.0149	0.152	1	2	KCNH2
Pubmed	7929457	Human cathepsin O. Molecular cloning from a breast carcinoma, production of the active enzyme in Escherichia coli, and expression analysis in human tissues.	Pubmed	0.00875	129	0.0149	0.152	1	2	CTSK
Pubmed	12849983	Production of human skeletal alpha-actin proteins by the baculovirus expression system.	Pubmed	0.00875	129	0.0149	0.152	1	2	ACTA1
Pubmed	15555579	Effect of zeta-globin substitution on the O2-transport properties of Hb S in vitro and in vivo.	GeneRIF	0.00875	129	0.0149	0.152	1	2	HBB
Pubmed	24933321	DC-HIL+ CD14+ HLA-DR no/low cells are a potential blood marker and therapeutic target for melanoma.	Pubmed	0.00875	129	0.0149	0.152	1	2	GPNMB
Pubmed	12458204	Oxygen affinity of hemoglobin regulates O2 consumption, metabolism, and physical activity.	Pubmed	0.00875	129	0.0149	0.152	1	2	HBA2

Category	ID	Name	Source	p-value	q-value Bonferroni	q-value FDR B&H	q-value FDR B&Y	Hit Count in Query List	Hit Count in Genome	Hit in Query List
Pubmed	478977	Hemoglobin Suan-Dok (alpha 2 109 (G16) Leu replaced by Arg beta 2): an unstable variant associated with alpha-thalassemia.	Pubmed	0.00875	129	0.0149	0.152	1	2	HBA2
Pubmed	23223298	Distinct roles of Nogo-a and Nogo receptor 1 in the homeostatic regulation of adult neural stem cel function and neuroblast migration.	GeneRIF	0.00875	129	0.0149	0.152	1	2	RTN4R
Pubmed	10188952	A role for p75 receptor in neurotrophin-3 functioning during the development of limb proprioception.	Pubmed	0.00875	129	0.0149	0.152	1	2	NGFR
Pubmed	23798567	Dmdmdx/Largemyd: a new mouse model of neuromuscular diseases useful for studying physiopathological mechanisms and testing therapies.	Pubmed	0.00875	129	0.0149	0.152	1	2	LARGE
Pubmed	15734871	Retrovirus-like long-terminal repeat DQ-LTR13 and genetic susceptibility to type 1 diabetes and autoimmune Addison's disease.	GeneRIF	0.00875	129	0.0149	0.152	1	2	HLA-DQB1
Pubmed	14747189	Expression of fractalkine in the Fallopian tube and of CX3CR1 in sperm.	GeneRIF	0.00875	129	0.0149	0.152	1	2	CX3CL1
Pubmed	11150295	Acetyl-CoA synthetase 2, a mitochondrial matrix enzyme involved in the oxidation of acetate.	Pubmed	0.00875	129	0.0149	0.152	1	2	ACSS1
Pubmed	17943627	Severe mitral regurgitation-relations between magnetic resonance imaging, echocardiography and natriuretic peptides.	GeneRIF	0.00875	129	0.0149	0.152	1	2	NPPA
Pubmed	12135771	Deficiency of triad formation in developing skeletal muscle cells lacking junctophilin type 1.	Pubmed	0.00875	129	0.0149	0.152	1	2	JPH2
Pubmed	23592924	Serine protease HtrA1 accumulates in corneal transforming growth factor beta induced protein (TGFBIp) amyloid deposits.	GeneRIF	0.00875	129	0.0149	0.152	1	2	HTRA1
Pubmed	15529553	HLA-DR B1 and DQ B1 polymorphisms in patients with coronary artery ectasia.	GeneRIF	0.00875	129	0.0149	0.152	1	2	HLA-DQB1
Pubmed	23331206	No association between bronchial asthma and HLA-DRB1, -DQB1 alleles in the Slovak population.	Pubmed	0.00875	129	0.0149	0.152	1	2	HLA-DQB1
Pubmed	18369157	Proline and gamma-carboxylated glutamate residues in matrix Gla protein are critical for binding of bone morphogenetic protein-4.	GeneRIF	0.00875	129	0.0149	0.152	1	2	BMP4
Pubmed	23441109	Pathogenic mutations in two families with congenital cataract identified with whole-exome sequencing.	GeneRIF	0.00875	129	0.0149	0.152	1	2	CRYAA
Pubmed	11145722	Type 1 diabetes-predisposing MHC alleles influence the selection of glutamic acid decarboxylase (GAD) 65-specific T cells in a transgenic model.	Pubmed	0.00875	129	0.0149	0.152	1	2	HLA-DQB1
Pubmed	10769387	The two thyroid hormone receptor genes have opposite effects on estrogen-stimulated sex behaviors.	Pubmed	0.00875	129	0.0149	0.152	1	2	THRA
Pubmed	25030063	Human CD68 promoter GFP transgenic mice allow analysis of monocyte to macrophage differentiation in vivo.	Pubmed	0.00875	129	0.0149	0.152	1	2	CD68
Pubmed	17664274	Small heat shock protein alphaA-crystallin regulates epithelial sodium channel expression.	GeneRIF	0.00875	129	0.0149	0.152	1	2	CRYAA
Pubmed	17588142	Association of HLA DQ B1* and HLA DR B1* alleles with goitrous juvenile autoimmune hypothyroidism--a case control study.	GeneRIF	0.00875	129	0.0149	0.152	1	2	HLA-DQB1
Pubmed	624676	The growth of femur and tibia in three genetically distinct chondrodystrophic mutants of the house mouse.	Pubmed	0.00875	129	0.0149	0.152	1	2	PAPSS2
Pubmed	20085807	Hydroimidazolone modification of human alphaA-crystallin: Effect on the chaperone function and protein refolding ability.	GeneRIF	0.00875	129	0.0149	0.152	1	2	CRYAA
Pubmed	11534018	Adenosine deaminase-acid phosphatase association and the environment: A study in a continental Italian population.	GeneRIF	0.00875	129	0.0149	0.152	1	2	ADA
Pubmed	19244252	Loss of alpha-dystroglycan laminin binding in epithelium-derived cancers is caused by silencing of LARGE.	GeneRIF	0.00875	129	0.0149	0.152	1	2	LARGE
Pubmed	7615398	Hb Anamosa or alpha 2(111)(G18)Ala-->Val beta 2 (alpha 2 mutation) and Hb Mulhacen or alpha 2(123)(H6)Ala-->Ser beta 2 (alpha 1 mutation) are two silent, stable variants detected by sequencing of amplified DNA.	Pubmed	0.00875	129	0.0149	0.152	1	2	HBA2
Pubmed	16781732	Structural rearrangements in the thyroid hormone receptor hinge domain and their putative role in the receptor function.	GeneRIF	0.00875	129	0.0149	0.152	1	2	THRA
Pubmed	18855024	Chromosomal microarray mapping suggests a role for BSX and Neurogranin in neurocognitive and behavioral defects in the 11q terminal deletion disorder (Jacobsen syndrome).	GeneRIF	0.00875	129	0.0149	0.152	1	2	NRGN
Pubmed	19898610	Loss of hepatocyte-nuclear-factor-4alpha affects colonic ion transport and causes chronic inflammation resembling inflammatory bowel disease in mice.	GeneRIF	0.00875	129	0.0149	0.152	1	2	CLDN15
Pubmed	16571082	Regulation of nuclear coactivator and corepressor expression in mouse cerebellum by thyroid hormone.	GeneRIF	0.00875	129	0.0149	0.152	1	2	THRA
Pubmed	2369847	Regional assignment of the mouse alpha A2-crystallin gene (Crya-1) to chromosome 17A3----B by in situ hybridization.	Pubmed	0.00875	129	0.0149	0.152	1	2	CRYAA
Pubmed	22167193	Ex vivo and in vivo effects of isofagomine on acid ß-glucosidase variants and substrate levels in Gaucher disease.	GeneRIF	0.00875	129	0.0149	0.152	1	2	PSAP
Pubmed	21997736	Insulin-like growth factor binding protein-6 interacts with the thyroid hormone receptor a1 and modulates the thyroid hormone-response in osteoblastic differentiation.	GeneRIF	0.00875	129	0.0149	0.152	1	2	THRA
Pubmed	18490834	Inhibitory effects of cholesterol sulfate on progesterone production in human granulosa-like tumor cell line, KGN.	GeneRIF	0.00875	129	0.0149	0.152	1	2	CYP11A1
Pubmed	23893803	Genetic polymorphisms in Matrix Metalloproteinases -1 and -7 and susceptibility to gastric cancer: an association study and meta-analysis.	Pubmed	0.00875	129	0.0149	0.152	1	2	MMP7
Pubmed	9533035	Assignment of protease, serine-like 1 (PRSSL1) to human chromosome 19q13 by in situ hybridization and radiation hybrid mapping.	Pubmed	0.00875	129	0.0149	0.152	1	2	KLK10
Pubmed	15067059	Costimulation of CD8 T cell responses by OX40.	GeneRIF	0.00875	129	0.0149	0.152	1	2	HLA-DQB1

Category	ID	Name	Source	p-value	q-value Bonferroni	q-value FDR B&H	q-value FDR B&Y	Hit Count in Query List	Hit Count in Genome	Hit in Query List
Pubmed	9337402	Allele-specific PCR assays for the tub and cpefat mutations.	Pubmed	0.00875	129	0.0149	0.152	1	2	CPE
Pubmed	17258947	Differential susceptibility of alpha A- and alpha B-crystallin to gamma-ray irradiation.	GeneRIF	0.00875	129	0.0149	0.152	1	2	CRYAA
Pubmed	18650831	MHC loci affecting cervical cancer risk: distinguishing the effects of HLA-DQB1 and non-HLA genes TNF, LTA, TAP1 and TAP2.	GeneRIF	0.00875	129	0.0149	0.152	1	2	HLA-DQB1
Pubmed	23753877	Limiting cardiac ischemic injury by pharmacological augmentation of macrophage migration inhibitory factor-AMP-activated protein kinase signal transduction.	Pubmed	0.00875	129	0.0149	0.152	1	2	CD74
Pubmed	16537808	Real-time monitoring of stress erythropoiesis in vivo using Gata1 and beta-globin LCR luciferase transgenic mice.	GeneRIF	0.00875	129	0.0149	0.152	1	2	HBB
Pubmed	15606898	Tissue transglutaminase contributes to disease progression in the R6/2 Huntington's disease mouse model via aggregate-independent mechanisms.	Pubmed	0.00875	129	0.0149	0.152	1	2	TGM2
Pubmed	22942500	Heritability of performance deficit accumulation during acute sleep deprivation in twins.	GeneRIF	0.00875	129	0.0149	0.152	1	2	ADA
Pubmed	22411813	Cancer-associated epithelial cell adhesion molecule (EpCAM; CD326) enables epidermal Langerhans cell motility and migration in vivo.	GeneRIF	0.00875	129	0.0149	0.152	1	2	HLA-DQB1
Pubmed	23555015	Rab GTPases regulate endothelial cell protein C receptor-mediated endocytosis and trafficking of factor VIIa.	GeneRIF	0.00875	129	0.0149	0.152	1	2	PROCR
Pubmed	20470906	Gene-specific paradoxical QT responses during rapid eye movement sleep in women with congenital long QT syndrome.	GeneRIF	0.00875	129	0.0149	0.152	1	2	KCNH2
Pubmed	10940926	Mast cells migrate, but do not degranulate, in response to fractalkine, a membrane-bound chemokine expressed constitutively in diverse cells of the skin.	Pubmed	0.00875	129	0.0149	0.152	1	2	CX3CL1
Pubmed	14675393	HLA in Czech adult patients with autoimmune diabetes mellitus: comparison with Czech children with type 1 diabetes and patients with type 2 diabetes.	GeneRIF	0.00875	129	0.0149	0.152	1	2	HLA-DQB1
Pubmed	12198244	Differential expression of thyroid hormone recepto isoforms dictates the dominant negative activity of mutant Beta receptor.	GeneRIF	0.00875	129	0.0149	0.152	1	2	THRA
Pubmed	23238728	Gravin orchestrates protein kinase A and ß2-adrenergic receptor signaling critical for synaptic plasticity and memory.	GeneRIF	0.00875	129	0.0149	0.152	1	2	AKAP12
Pubmed	15201952	The influence of matrix metalloproteinase-7 on early mammary tumorigenesis in the multiple intestinal neoplasia mouse.	GeneRIF	0.00875	129	0.0149	0.152	1	2	MMP7
Pubmed	17679948	Association of the HTRA1 -625G>A promoter gene polymorphism with exudative age-related macular degeneration in a Central European population.	GeneRIF	0.00875	129	0.0149	0.152	1	2	HTRA1
Pubmed	21423200	Type I and II interferons enhance dendritic cell maturation and migration capacity by regulating CD38 and CD74 that have synergistic effects with TLR agonists.	GeneRIF	0.00875	129	0.0149	0.152	1	2	CD74
Pubmed	11418606	Acute phase protein alpha 1-acid glycoprotein interacts with plasminogen activator inhibitor type 1 and stabilizes its inhibitory activity.	Pubmed	0.00875	129	0.0149	0.152	1	2	ORM1
Pubmed	20170930	Presence or absence of a known diabetic ketoacidosis precipitant defines distinct syndromes of "A-ß+" ketosis-prone diabetes based on long-term ß-cell function, human leukocyte antigen class II alleles, and sex predilection.	GeneRIF	0.00875	129	0.0149	0.152	1	2	HLA-DQB1
Pubmed	24381092	HLA-DRB1 and HLA-DQB1 allele associations in an Albanian patient population with rheumatoid arthritis: correlations with the specific autoantibody markers and inter-population DRB1 allele frequency variability.	Pubmed	0.00875	129	0.0149	0.152	1	2	HLA-DQB1
Pubmed	22511809	Association of variants in HLA-DQA1-DQB1, PTPN22, INS, and CTLA4 with GAD autoantibodies and insulin secretion in nondiabetic adults of the Botnia Prospective Study.	GeneRIF	0.00875	129	0.0149	0.152	1	2	HLA-DQB1
Pubmed	17052752	Distribution of CD14+ and CD68+ macrophages in the placental bed and basal plate of women with preeclampsia and preterm labor.	GeneRIF	0.00875	129	0.0149	0.152	1	2	CD68
Pubmed	9036956	The nonpolymorphic MHC class II isotype, HLA-DQA2, is expressed on the surface of B lymphoblastoid cells.	Pubmed	0.00875	129	0.0149	0.152	1	2	HLA-DQB1
Pubmed	21931690	Upregulation of hemoglobin expression by oxidative stress in hepatocytes and its implication in nonalcoholic steatohepatitis.	GeneRIF	0.00875	129	0.0149	0.152	1	2	HBB
Pubmed	23741007	Loss of endothelial protein C receptors links coagulation and inflammation to parasite sequestration in cerebral malaria in African children.	Pubmed	0.00875	129	0.0149	0.152	1	2	PROCR
Pubmed	15654974	A proteolytic cascade of kallikreins in the stratum corneum.	GeneRIF	0.00875	129	0.0149	0.152	1	2	KLK14
Pubmed	19940065	Junctophilin damage contributes to early strength deficits and EC coupling failure after eccentric contractions.	GeneRIF	0.00875	129	0.0149	0.152	1	2	JPH2
Pubmed	11067886	Autocrine secretion of interferon gamma negatively regulates homing of immature B cells.	Pubmed	0.00875	129	0.0149	0.152	1	2	CD74
Pubmed	19071118	Disulfide cross-links in the interaction of a cataract linked alphaA-crystallin mutant with betaB1-crystallin.	GeneRIF	0.00875	129	0.0149	0.152	1	2	CRYAA
Pubmed	9060478	Latent transforming growth factor-beta binding protein domains involved in activation and transglutaminase-dependent cross-linking of latent transforming growth factor-beta.	Pubmed	0.00875	129	0.0149	0.152	1	2	TGM2
Pubmed	11481347	Myosin light chain kinase binding to a unique site on F-actin revealed by three-dimensional image reconstruction.	Pubmed	0.00875	129	0.0149	0.152	1	2	ACTA1
Pubmed	10569723	Two new alpha chain variants: Hb Boghé [alpha58(E7)His-->Gln, alpha2], a variant on the distal histidine, and Hb CHarolles [alpha103(G10)His-Tyr, alpha1].	Pubmed	0.00875	129	0.0149	0.152	1	2	HBA2

Category	ID	Name	Source	p-value	q-value Bonferroni	q-value FDR B&H	q-value FDR B&Y	Hit Count in Query List	Hit Count in Genome	Hit in Query List
Pubmed	19473100	Bone morphogenetic protein signaling is involved in human mesenchymal stem cell survival in serum-free medium.	GeneRIF	0.00875	129	0.0149	0.152	1	2	BMP4
Pubmed	24060055	Hypertrophy of the ligament flavum in degenerative lumbar stenosis associated with the increased expression of fractalkine (CX3CL1)/CX3CR1 chemokine.	Pubmed	0.00875	129	0.0149	0.152	1	2	CX3CL1
Pubmed	10666339	The carboxy terminus of AFAP-110 modulates direct interactions with actin filaments and regulates its ability to alter actin filament integrity and induce lamellipodia formation.	Pubmed	0.00875	129	0.0149	0.152	1	2	ACTA1
Pubmed	24699711	A DERL3-associated defect in the degradation of SLC2A1 mediates the Warburg effect.	Pubmed	0.00875	129	0.0149	0.152	1	2	DERL3
Pubmed	18499699	Sulindac treatment alters collagen and matrilysin expression in adenomas of ApcMin/+ mice.	Pubmed	0.00875	129	0.0149	0.152	1	2	MMP7
Pubmed	21951685	In vivo structure/function and expression analysis of the CX3C chemokine fractalkine.	GeneRIF	0.00875	129	0.0149	0.152	1	2	CX3CL1
Pubmed	23863900	Neddylation plays an important role in the regulation of murine and human dendritic cell function.	Pubmed	0.00875	129	0.0149	0.152	1	2	HLA-DQB1
Pubmed	17374730	Tissue transglutaminase inhibits autophagy in pancreatic cancer cells.	GeneRIF	0.00875	129	0.0149	0.152	1	2	TGM2
Pubmed	20934750	Expression and localisation of FoxO3 and FoxO4 in human placenta and fetal membranes.	GeneRIF	0.00875	129	0.0149	0.152	1	2	FOXO4
Pubmed	24140000	Association of T-cell co-regulatory protein expression with clinical outcomes following radical cystectomy for urothelial carcinoma of the bladder.	Pubmed	0.00875	129	0.0149	0.152	1	2	CD276
Pubmed	22220474	Transglutaminase 2: a new paradigm for NF-kappaB involvement in disease.	GeneRIF	0.00875	129	0.0149	0.152	1	2	TGM2
Pubmed	15034071	Adenoviral gene delivery of elafin and secretory leukocyte protease inhibitor attenuates NF-kappa B-dependent inflammatory responses of human endothelial cells and macrophages to atherogenic stimuli.	GeneRIF	0.00875	129	0.0149	0.152	1	2	SLPI
Pubmed	19359777	Microcytic hypochromic anemia patients with thalassemia: genotyping approach.	GeneRIF	0.00875	129	0.0149	0.152	1	2	HBB
Pubmed	21364666	Partial dysferlin reconstitution by adult murine mesoangioblasts is sufficient for full functional recovery in a murine model of dysferlinopathy.	Pubmed	0.00875	129	0.0149	0.152	1	2	DYSF
Pubmed	11098940	Identification by sequencing based typing and complete coding region analysis of three new HLA class II alleles: DRB3*0210, DRB3*0211 and DQB1*0310.	Pubmed	0.00875	129	0.0149	0.152	1	2	HLA-DQB1
Pubmed	939913	Myodystrophy, a new myopathy on chromosome 8 of the mouse.	Pubmed	0.00875	129	0.0149	0.152	1	2	LARGE
Pubmed	7914549	Murine AIDS is an antigen-driven disease: requirements for major histocompatibility complex class II expression and CD4+ T cells.	Pubmed	0.00875	129	0.0149	0.152	1	2	HLA-DQB1
Pubmed	24494193	Methylglyoxal in cells elicits a negative feedback loop entailing transglutaminase 2 and glyoxalase 1.	Pubmed	0.00875	129	0.0149	0.152	1	2	TGM2
Pubmed	18818416	Deficiency and inhibition of cathepsin K reduce body weight gain and increase glucose metabolism in mice.	GeneRIF	0.00875	129	0.0149	0.152	1	2	CTSK
Pubmed	21536683	Protein kinase A activity at the endoplasmic reticulum surface is responsible for augmentation of human ether-a-go-go-related gene product (HERG).	GeneRIF	0.00875	129	0.0149	0.152	1	2	KCNH2
Pubmed	19651604	The eye lens chaperone alpha-crystallin forms defined globular assemblies.	GeneRIF	0.00875	129	0.0149	0.152	1	2	CRYAA
Pubmed	23402770	Novel mutations responsible for a-thalassemia in Iranian families.	GeneRIF	0.00875	129	0.0149	0.152	1	2	HBA2
Pubmed	12787001	The HLA-DRB, -DQB polymorphism and anti-insulin antibody response in Slovenian patients with type 1 diabetes.	GeneRIF	0.00875	129	0.0149	0.152	1	2	HLA-DQB1
Pubmed	25549795	The matrix metalloproteinase-7 and pro-enzyme of metalloproteinase-1 as a potential marker for patients with rectal cancer without distant metastasis.	Pubmed	0.00875	129	0.0149	0.152	1	2	MMP7
Pubmed	21725609	PRL-3 promotes the proliferation of LoVo cells via the upregulation of KCNN4 channels.	GeneRIF	0.00875	129	0.0149	0.152	1	2	KCNN4
Pubmed	25329061	Alpha-1-antitrypsin: a novel human high temperature requirement protease A1 (HTRA1) substrate in human placental tissue.	Pubmed	0.00875	129	0.0149	0.152	1	2	HTRA1
Pubmed	19657146	Identification of a novel HtrA1-susceptible cleavage site in human aggrecan: evidence for the involvement of HtrA1 in aggrecan proteolysis in vivo.	GeneRIF	0.00875	129	0.0149	0.152	1	2	HTRA1
Pubmed	18628975	Histone deacetylases control neurogenesis in embryonic brain by inhibition of BMP2/4 signaling.	GeneRIF	0.00875	129	0.0149	0.152	1	2	BMP4
Pubmed	15301861	Patients with early onset of type 1 diabetes have significantly higher GG genotype at position 49 of the CTLA4 gene.	GeneRIF	0.00875	129	0.0149	0.152	1	2	HLA-DQB1
Pubmed	17967130	Dysregulated BMP signaling and enhanced osteogenic differentiation of connective tissue progenitor cells from patients with fibrodysplasia ossificans progressiva (FOP).	GeneRIF	0.00875	129	0.0149	0.152	1	2	BMP4
Pubmed	8294875	The invariant chain is required for intracellular transport and function of major histocompatibility complex class II molecules.	Pubmed	0.00875	129	0.0149	0.152	1	2	CD74
Pubmed	24581800	Study of the residues involved in the binding of ß1 to ß3 subunits in the sodium channel.	Pubmed	0.00875	129	0.0149	0.152	1	2	SCN3B
Pubmed	19500688	Stratifying risk for celiac disease in a large at-risk United States population by using HLA alleles.	GeneRIF	0.00875	129	0.0149	0.152	1	2	HLA-DQB1
Pubmed	22673022	Left ventricular structure and function in relation to steroid biosynthesis genes in a white population.	GeneRIF	0.00875	129	0.0149	0.152	1	2	CYP11A1
Pubmed	21325036	Novel alleles of HLA-DQ and -DR loci show association with recurrent miscarriages among South Indian women.	GeneRIF	0.00875	129	0.0149	0.152	1	2	HLA-DQB1
Pubmed	22209843	Bone morphogenetic protein (BMP)-4 and BMP-7 suppress granulosa cell apoptosis via different pathways: BMP-4 via PI3K/PDK-1/Akt and BMP-7 via PI3K/PDK-1/PKC.	GeneRIF	0.00875	129	0.0149	0.152	1	2	BMP4

Category	ID	Name	Source	p-value	q-value Bonferroni	q-value FDR B&H	q-value FDR B&Y	Hit Count in Query List	Hit Count in Genome	Hit in Query List
Pubmed	23956503	Fractalkine (CX3CL1) and its receptor CX3CR1 may contribute to increased angiogenesis in diabetic placenta.	Pubmed	0.00875	129	0.0149	0.152	1	2	CX3CL1
Pubmed	11750124	Strain-specific complementation between NRIF1 and NRIF2, two zinc finger proteins sharing structural and biochemical properties.	Pubmed	0.00875	129	0.0149	0.152	1	2	NGFR
Pubmed	23145002	Apoptosis-related gene expression profiles of mouse ESCs and maGSCs: role of Fgf4 and Mnda in pluripotent cell responses to genotoxicity.	GeneRIF	0.00875	129	0.0149	0.152	1	2	IFI16
Pubmed	22815045	Atrogin-1, MuRF-1, and sarcopenia.	GeneRIF	0.00875	129	0.0149	0.152	1	2	FBXO32
Pubmed	24651436	Loss of TACSTD2 contributed to squamous cell carcinoma progression through attenuating TAp63-dependent apoptosis.	Pubmed	0.00875	129	0.0149	0.152	1	2	TACSTD2
Pubmed	23247112	Age-dependent chloride channel expression in skeletal muscle fibres of normal and HSA(LR) myotonic mice.	GeneRIF	0.00875	129	0.0149	0.152	1	2	ACTA1
Pubmed	19933195	Analysis of six genetic risk factors highly associated with AMD in the region surrounding ARMS2 and HTRA1 on chromosome 10, region q26.	GeneRIF	0.00875	129	0.0149	0.152	1	2	HTRA1
Pubmed	17600318	BMP4 induces HO-1 via a Smad-independent, p38MAPK-dependent pathway in pulmonary artery myocytes.	GeneRIF	0.00875	129	0.0149	0.152	1	2	BMP4
Pubmed	21652028	HLA-DRB1/DQB1 susceptibility for autoimmune polyglandular syndrome type II and III in south of Tunisia.	GeneRIF	0.00875	129	0.0149	0.152	1	2	HLA-DQB1
Pubmed	24244600	Decrease in prosaposin in the Dystrophic mdx mouse brain.	Pubmed	0.00875	129	0.0149	0.152	1	2	PSAP
Pubmed	20042692	Bmp2 and Bmp4 exert opposing effects in hypoxic pulmonary hypertension.	GeneRIF	0.00875	129	0.0149	0.152	1	2	BMP4
Pubmed	17155946	Regulated endothelial protein C receptor shedding is mediated by tumor necrosis factor-alpha converting enzyme/ADAM17.	GeneRIF	0.00875	129	0.0149	0.152	1	2	PROCR
Pubmed	7980522	Matrilysin is much more efficient than other matrix metalloproteinases in the proteolytic inactivation o alpha 1-antitrypsin.	Pubmed	0.00875	129	0.0149	0.152	1	2	MMP7
Pubmed	17311920	Role of DNA methylation in stable gene repression.	GeneRIF	0.00875	129	0.0149	0.152	1	2	SLPI
Pubmed	20605793	Involvement of caveolin in low K+-induced endocytic degradation of cell-surface human ether-a-go-go-related gene (hERG) channels.	GeneRIF	0.00875	129	0.0149	0.152	1	2	KCNH2
Pubmed	22131018	Phosphatase of regenerating liver-3 promotes migration and invasion by upregulating matrix metalloproteinases-7 in human colorectal cancer cells.	GeneRIF	0.00875	129	0.0149	0.152	1	2	MMP7
Pubmed	19346663	Effects of long-term intravenous administration of adrenomedullin (AM) plus hANP therapy in acute decompensated heart failure: a pilot study.	GeneRIF	0.00875	129	0.0149	0.152	1	2	NPPA
Pubmed	11861164	Neurodevelopmental control by thyroid hormone receptors.	GeneRIF	0.00875	129	0.0149	0.152	1	2	THRA
Pubmed	20808839	Calcium-activated potassium channels BK and IK1 are functionally expressed in human gliomas but do not regulate cell proliferation.	GeneRIF	0.00875	129	0.0149	0.152	1	2	KCNN4
Pubmed	20231357	Cyclin-dependent kinase 8 positively cooperates with Mediator to promote thyroid hormone receptor-dependent transcriptional activation.	GeneRIF	0.00875	129	0.0149	0.152	1	2	THRA
Pubmed	20540624	Variation in echocardiographic and cardiac hemodynamic effects of PM and ozone inhalation exposure in strains related to Nppa and Npr1 gene knock-out mice.	GeneRIF	0.00875	129	0.0149	0.152	1	2	NPPA
Pubmed	22862152	HLA-DQ association and allele competition in Chinese narcolepsy.	GeneRIF	0.00875	129	0.0149	0.152	1	2	HLA-DQB1
Pubmed	21935404	Drug-tolerant cancer cells show reduced tumor-initiating capacity: depletion of CD44 cells and evidence for epigenetic mechanisms.	GeneRIF	0.00875	129	0.0149	0.152	1	2	KDM5A
Pubmed	15284238	Deamidation affects structural and functional properties of human alphaA-crystallin and its oligomerization with alphaB-crystallin.	GeneRIF	0.00875	129	0.0149	0.152	1	2	CRYAA
Pubmed	12015278	Anterior segment dysgenesis and the developmental glaucomas are complex traits.	Pubmed	0.00875	129	0.0149	0.152	1	2	BMP4
Pubmed	21499941	Diets rich in saturated fat and/or salt differentially modulate atrial natriuretic peptide and renin expression in C57BL/6 mice.	GeneRIF	0.00875	129	0.0149	0.152	1	2	NPPA
Pubmed	20225292	Human leukocyte antigen-DR15, low infant sibling exposure and multiple sclerosis: gene-environment interaction.	GeneRIF	0.00875	129	0.0149	0.152	1	2	HLA-DQB1
Pubmed	16948649	Human leucocyte antigen class II polymorphism in Irish patients with multiple sclerosis.	GeneRIF	0.00875	129	0.0149	0.152	1	2	HLA-DQB1
Pubmed	24449824	hERG1 channels regulate VEGF-A secretion in human gastric cancer: clinicopathological correlations and therapeutical implications.	Pubmed	0.00875	129	0.0149	0.152	1	2	KCNH2
Pubmed	12050583	HLA-DQA1 and HLA-DQB1 genetic markers and clinical presentation in celiac disease.	GeneRIF	0.00875	129	0.0149	0.152	1	2	HLA-DQB1
Pubmed	18692065	Selective Cu2+ binding, redox silencing, and cytoprotective effects of the small heat shock proteins alphaA- and alphaB-crystallin.	GeneRIF	0.00875	129	0.0149	0.152	1	2	CRYAA
Pubmed	15713690	Thyroid hormone regulates tubulin expression in mammalian liver. Effects of deleting thyroid hormone receptor-alpha or -beta.	Pubmed	0.00875	129	0.0149	0.152	1	2	THRA
Pubmed	8896987	Cloning of rat type I receptor cDNA for bone morphogenetic protein-2 and bone morphogenetic protein-4, and the localization compared with that of the ligands.	Pubmed	0.00875	129	0.0149	0.152	1	2	BMP4
Pubmed	16704535	Expression of nerve growth factor receptors is correlated with progression and prognosis of human pancreatic cancer.	GeneRIF	0.00875	129	0.0149	0.152	1	2	NGFR
Pubmed	19789510	Type 1 diabetes: evidence of interaction between ACP1 and ADA1 gene polymorphisms.	GeneRIF	0.00875	129	0.0149	0.152	1	2	ADA
Pubmed	20558822	Impact of combined deficiency of hepatic lipase and endothelial lipase on the metabolism of both high-density lipoproteins and apolipoprotein B-containing lipoproteins.	GeneRIF	0.00875	129	0.0149	0.152	1	2	LIPG
Pubmed	24321989	Exome sequencing and diffusion tensor imaging in developmental disabilities.	Pubmed	0.00875	129	0.0149	0.152	1	2	MID1

Category	ID	Name	Source	p-value	q-value Bonferroni	q-value FDR B&H	q-value FDR B&Y	Hit Count in Query List	Hit Count in Genome	Hit in Query List
Pubmed	18695873	Activin A enhances MMP-7 activity via the transcription factor AP-1 in an esophageal squamous cell carcinoma cell line.	GeneRIF	0.00875	129	0.0149	0.152	1	2	MMP7
Pubmed	19819768	HLA-DQ2 and -DQ8 genotypes in celiac and healthy Libyan children.	GeneRIF	0.00875	129	0.0149	0.152	1	2	HLA-DQB1
Pubmed	15448099	Selective loss of calcitonin gene-related Peptide-expressing primary sensory neurons of the a-cell phenotype in early experimental diabetes.	GeneRIF	0.00875	129	0.0149	0.152	1	2	NGFR
Pubmed	24797634	TRa protects against atherosclerosis in male mice: identification of a novel anti-inflammatory property for TRa in mice.	Pubmed	0.00875	129	0.0149	0.152	1	2	THRA
Pubmed	12682012	PKA phosphorylates the p75 receptor and regulates its localization to lipid rafts.	GeneRIF	0.00875	129	0.0149	0.152	1	2	NGFR
Pubmed	8982156	Lack of neurotrophin-3 results in death of spinal sensory neurons and premature differentiation of their precursors.	Pubmed	0.00875	129	0.0149	0.152	1	2	NGFR
Pubmed	25218469	Down-regulation of ether-a-go-go-related gene potassium channel protein through sustained stimulation of AT1 receptor by angiotensin II.	Pubmed	0.00875	129	0.0149	0.152	1	2	KCNH2
Pubmed	16861620	Conditional expression of human acid beta-glucosidase improves the visceral phenotype in a Gaucher disease mouse model.	GeneRIF	0.00875	129	0.0149	0.152	1	2	PSAP
Pubmed	14694619	[The relationship between silicosis and the polymorphism of HLA-DRB1 *, DQB1 * genes].	GeneRIF	0.00875	129	0.0149	0.152	1	2	HLA-DQB1
Pubmed	19853653	Establishment of yeast reporter assay systems to detect ligands of thyroid hormone receptors alpha and beta.	GeneRIF	0.00875	129	0.0149	0.152	1	2	THRA
Pubmed	20403400	Upregulation of ANP and NPR-C mRNA in the kidney and heart of eNOS knockout mice.	GeneRIF	0.00875	129	0.0149	0.152	1	2	NPPA
Pubmed	15191529	HLA allele frequency and clinical outcome in Italian patients with cutaneous melanoma.	GeneRIF	0.00875	129	0.0149	0.152	1	2	HLA-DQB1
Pubmed	15388327	Distribution of IRAG and cGKI-isoforms in murine tissues.	GeneRIF	0.00875	129	0.0149	0.152	1	2	MRVI1
Pubmed	8587135	A reassessment of mammalian alpha A-crystallin sequences using DNA sequencing: implications for anthropoid affinities of tarsier.	Pubmed	0.00875	129	0.0149	0.152	1	2	CRYAA
Pubmed	22138498	Modulation of TCRß surface expression during TCR revision.	Pubmed	0.00875	129	0.0149	0.152	1	2	HLA-DQB1
Pubmed	9653162	HD mice: a novel mouse mutant with a specific defect in the generation of CD4(+) T cells.	Pubmed	0.00875	129	0.0149	0.152	1	2	HLA-DQB1
Pubmed	20809254	PI3K-dependent host cell actin rearrangements are required for Cronobacter sakazakii invasion of human brain microvascular endothelial cells.	GeneRIF	0.00875	129	0.0149	0.152	1	2	ACTA1
Pubmed	20682466	A new alpha(0)-thalassemia deletion found in a Dutch family (--(AW)).	GeneRIF	0.00875	129	0.0149	0.152	1	2	HBA2
Pubmed	22419550	TROP2 is epigenetically inactivated and modulates IGF-1R signalling in lung adenocarcinoma.	GeneRIF	0.00875	129	0.0149	0.152	1	2	TACSTD2
Pubmed	2731651	A developmental study of the abnormal expression of alpha-cardiac and alpha-skeletal actins in the striated muscle of a mutant mouse.	Pubmed	0.00875	129	0.0149	0.152	1	2	ACTA1
Pubmed	23489213	Expression of neurotrophic factors and their receptors in keratoconic cornea.	Pubmed	0.00875	129	0.0149	0.152	1	2	NGFR
Pubmed	24551305	Smoothelin and caldesmon are reliable markers for distinguishing muscularis propria from desmoplasia: a critical distinction for accurate staging colorectal adenocarcinoma.	Pubmed	0.00875	129	0.0149	0.152	1	2	SMTN
Pubmed	19324423	Expression of ADAM-17, TIMP-3 and fractalkine in the human adult brain endothelial cell line, hCMEC/D3, following pro-inflammatory cytokine treatment.	GeneRIF	0.00875	129	0.0149	0.152	1	2	CX3CL1
Pubmed	21983347	Angiopoietin-like-2 and -3 act through their coiled-coil domains to enhance survival and replating capacity of human cord blood hematopoietic progenitors.	GeneRIF	0.00875	129	0.0149	0.152	1	2	ANGPTL2
Pubmed	14504392	Variable hematopoietic graft rejection and graft-versus-host disease in MHC-matched strains of mice.	Pubmed	0.00875	129	0.0149	0.152	1	2	HLA-DQB1
Pubmed	22566094	p75NTR signal transduction suppressed by BFAR and p75NTR interactions.	GeneRIF	0.00875	129	0.0149	0.152	1	2	NGFR
Pubmed	22968485	Muscle inflammatory signaling in response to 9 days of physical inactivity in young men with low compared with normal birth weight.	GeneRIF	0.00875	129	0.0149	0.152	1	2	CD68
Pubmed	10234043	Enhanced neurotrophin-induced axon growth in myelinated portions of the CNS in mice lacking the p75 neurotrophin receptor.	Pubmed	0.00875	129	0.0149	0.152	1	2	NGFR
Pubmed	17971177	Low-risk HLA genotype in Type 1 diabetes is associated with less destruction of pancreatic B-cells 12 months after diagnosis.	GeneRIF	0.00875	129	0.0149	0.152	1	2	HLA-DQB1
Pubmed	4550395	Multiple alpha chain loci for human haemoglobins: Hb J-Buda and Hb G-Pest.	Pubmed	0.00875	129	0.0149	0.152	1	2	HBA2
Pubmed	22076569	Activation of the BMP4 pathway and early expression of CDX2 characterize non-specialized columnar metaplasia in a human model of Barrett's esophagus.	GeneRIF	0.00875	129	0.0149	0.152	1	2	BMP4
Pubmed	21764478	Overexpression of matrix metalloproteinase-7 and -9 in NSCLC tumor and stromal cells: correlation with a favorable clinical outcome.	GeneRIF	0.00875	129	0.0149	0.152	1	2	MMP7
Pubmed	19893320	Transglutaminase 2 deficiency decreases plaque fibrosis and increases plaque inflammation in apolipoprotein-E-deficient mice.	GeneRIF	0.00875	129	0.0149	0.152	1	2	TGM2
Pubmed	18295595	Icariin induces osteogenic differentiation in vitro in a BMP- and Runx2-dependent manner.	GeneRIF	0.00875	129	0.0149	0.152	1	2	BMP4
Pubmed	17940206	Cbfbeta-SMMHC impairs differentiation of common lymphoid progenitors and reveals an essential role for RUNX in early B-cell development.	GeneRIF	0.00875	129	0.0149	0.152	1	2	MYH11
Pubmed	17607357	Dysferlin-mediated membrane repair protects the heart from stress-induced left ventricular injury.	GeneRIF	0.00875	129	0.0149	0.152	1	2	DYSF
Pubmed	23433854	Ectonucleotide pyrophosphatase/phosphodiesterase (E-NPP) and adenosine deaminase (ADA) activities in prostate cancer patients: influence of Gleason score, treatment and bone metastasis.	Pubmed	0.00875	129	0.0149	0.152	1	2	ADA

Category	ID	Name	Source	p-value	q-value Bonferroni	q-value FDR B&H	q-value FDR B&Y	Hit Count in Query List	Hit Count in Genome	Hit in Query List
Pubmed	15357778	HLA class II genotype and factor VIII inhibitors in mild haemophilia A patients with an Arg593 to Cys mutation.	GeneRIF	0.00875	129	0.0149	0.152	1	2	HLA-DQB1
Pubmed	12871209	Functional characterization and regulation of the taurine transporter and cysteine dioxygenase in human hepatoblastoma HepG2 cells.	Pubmed	0.00875	129	0.0149	0.152	1	2	CDO1
Pubmed	11985790	Association of HLA-DQB1*0301 and HLA-DQB1*0602 with different subtypes of gastric cancer in Taiwan.	GeneRIF	0.00875	129	0.0149	0.152	1	2	HLA-DQB1
Pubmed	8792610	Restricted expression of a novel retinoic acid responsive gene during limb bud dorsoventral patterning and endochondral ossification.	Pubmed	0.00875	129	0.0149	0.152	1	2	STRA6
Pubmed	20214848	Human leukocyte antigen type and progression from onset of symptoms to development of asthma.	GeneRIF	0.00875	129	0.0149	0.152	1	2	HLA-DQB1
Pubmed	18203951	Erythroid defects in TRalpha-/- mice.	GeneRIF	0.00875	129	0.0149	0.152	1	2	THRA
Pubmed	21881600	The p75 receptor mediates axon growth inhibition through an association with PIR-B.	GeneRIF	0.00875	129	0.0149	0.152	1	2	NGFR
Pubmed	24733189	High glucose induces CCL20 in proximal tubular cells via activation of the KCa3.1 channel.	Pubmed	0.00875	129	0.0149	0.152	1	2	KCNN4
Pubmed	11938439	Determination of the relative contribution of three genes-the cystic fibrosis transmembrane conductance regulator gene, the cationic trypsinogen gene, and the pancreatic secretory trypsin inhibitor gene-to the etiology of idiopathic chronic pancreatitis.	GeneRIF	0.00875	129	0.0149	0.152	1	2	SPINK1
Pubmed	21168500	Mesenchymal stem cell transplantation modulates neuroinflammation in focal cerebral ischemia: contribution of fractalkine and IL-5.	GeneRIF	0.00875	129	0.0149	0.152	1	2	CX3CL1
Pubmed	19589860	Inhibin B is a more potent suppressor of rat follicle stimulating hormone release than inhibin a in vitro and in vivo.	GeneRIF	0.00875	129	0.0149	0.152	1	2	INHBB
Pubmed	16897755	PAK4 and alphaPIX determine podosome size and number in macrophages through localized actin regulation.	GeneRIF	0.00875	129	0.0149	0.152	1	2	ARHGEF6
Pubmed	24442343	Increased progesterone receptor A expression in labouring human myometrium is associated with decreased promoter occupancy by the histone demethylase JARID1A.	Pubmed	0.00875	129	0.0149	0.152	1	2	KDM5A
Pubmed	21796887	The investigation of HLA microsatellites influence in predisposition to sarcoidosis among Croatians.	GeneRIF	0.00875	129	0.0149	0.152	1	2	HLA-DQB1
Pubmed	23348224	The endothelial protein C receptor and activated protein C play a limited role in host defense during experimental tuberculosis.	Pubmed	0.00875	129	0.0149	0.152	1	2	PROCR
Pubmed	11819188	Localization and endothelin-3 dependence of stem cells of the enteric nervous system in the embryonic colon.	Pubmed	0.00875	129	0.0149	0.152	1	2	NGFR
Pubmed	18505736	Tissue transglutaminase modulates alpha-synuclein oligomerization.	GeneRIF	0.00875	129	0.0149	0.152	1	2	TGM2
Pubmed	11115400	Novel properties of the protein kinase CK2-site-regulated nuclear- localization sequence of the interferon-induced nuclear factor IFI 16.	Pubmed	0.00875	129	0.0149	0.152	1	2	IFI16
Pubmed	22117603	Association of homozygous sickle cell anemia and glucose-6-phosphate dehydrogenase deficiency.	GeneRIF	0.00875	129	0.0149	0.152	1	2	HBB
Pubmed	15749874	Role of the programmed death-1 pathway in regulation of alloimmune responses in vivo.	GeneRIF	0.00875	129	0.0149	0.152	1	2	HLA-DQB1
Pubmed	15226637	Efficient cardiomyogenic differentiation of embryonic stem cell by fibroblast growth factor 2 and bone morphogenetic protein 2.	GeneRIF	0.00875	129	0.0149	0.152	1	2	BMP4
Pubmed	19577604	Ghrelin receptor agonist, GHRP-2, attenuates burn injury-induced MuRF-1 and MAFbx expression and muscle proteolysis in rats.	GeneRIF	0.00875	129	0.0149	0.152	1	2	FBXO32
Pubmed	11850458	Small proline-rich repeat protein 1A is expressed by axotomized neurons and promotes axonal outgrowth.	GeneRIF	0.00875	129	0.0149	0.152	1	2	ACTA1
Pubmed	12011108	The p75 receptor transduces the signal from myelin-associated glycoprotein to Rho.	Pubmed	0.00875	129	0.0149	0.152	1	2	NGFR
Pubmed	20817870	Selective inhibition of the lectin pathway of complement with phage display selected peptides against mannose-binding lectin-associated serine protease (MASP)-1 and -2: significant contribution of MASP-1 to lectin pathway activation.	GeneRIF	0.00875	129	0.0149	0.152	1	2	MASP1
Pubmed	15120190	Allele and haplotype frequencies for HLA class II (DQA1 and DQB1) loci in patients with celiac disease from Spain.	GeneRIF	0.00875	129	0.0149	0.152	1	2	HLA-DQB1
Pubmed	10559207	Genomic organization of the mouse and human genes encoding the ATP sulfurylase/adenosine 5'-phosphosulfate kinase isoform SK2.	Pubmed	0.00875	129	0.0149	0.152	1	2	PAPSS2
Pubmed	20833965	Pore mutants of HERG and KvLQT1 downregulate the reciprocal currents in stable cell lines.	GeneRIF	0.00875	129	0.0149	0.152	1	2	KCNH2
Pubmed	21686328	Mutational screening of six genes in Chinese patients with congenital cataract and microcornea.	GeneRIF	0.00875	129	0.0149	0.152	1	2	CRYAA
Pubmed	2101836	Hb Davenport or alpha 2(78)(EF7)Asn----His beta 2.	Pubmed	0.00875	129	0.0149	0.152	1	2	HBA2
Pubmed	11552992	Mutations in the thrombomodulin and endothelial protein C receptor genes in women with late fetal loss.	GeneRIF	0.00875	129	0.0149	0.152	1	2	PROCR
Pubmed	24789918	Lysosomal stress in obese adipose tissue macrophages contributes to MITF-dependent Gpnmb induction.	Pubmed	0.00875	129	0.0149	0.152	1	2	GPNMB
Pubmed	7835906	Mapping the gene that encodes phosphatidylinositol-specific phospholipase C-gamma 2 in the human and the mouse.	Pubmed	0.00875	129	0.0149	0.152	1	2	ACTA1
Pubmed	20622017	Analysis of site-specific glycosylation of renal and hepatic ¿-glutamyl transpeptidase from normal human tissue.	GeneRIF	0.00875	129	0.0149	0.152	1	2	GGT1
Pubmed	17141888	The effects of modulation of gamma-glutamyl transpeptidase activity in HepG2 cells on thiol homeostasis and caspase-3-activity.	GeneRIF	0.00875	129	0.0149	0.152	1	2	GGT1
Pubmed	17692272	LOC387715/HTRA1 variants in polypoidal choroidal vasculopathy and age-related macular degeneration in a Japanese population.	GeneRIF	0.00875	129	0.0149	0.152	1	2	HTRA1

Category	ID	Name	Source	p-value	q-value Bonferroni	q-value FDR B&H	q-value FDR B&Y	Hit Count in Query List	Hit Count in Genome	Hit in Query List
Pubmed	19272325	Association between human leukocyte antigen class II alleles and human papillomavirus-mediated cervical cancer in Indian women.	GeneRIF	0.00875	129	0.0149	0.152	1	2	HLA-DQB1
Pubmed	10542261	Microfibril-associated protein 4 is present in lung washings and binds to the collagen region of lung surfactant protein D.	Pubmed	0.00875	129	0.0149	0.152	1	2	MFAP4
Pubmed	22727609	Clinical characteristics of 30 Czech families with long QT syndrome and KCNQ1 and KCNH2 gene mutations: importance of exercise testing.	GeneRIF	0.00875	129	0.0149	0.152	1	2	KCNH2
Pubmed	20054823	The host defense peptide LL-37 activates the tumo suppressing bone morphogenetic protein signaling via inhibition of proteasome in gastric cancer cells.	GeneRIF	0.00875	129	0.0149	0.152	1	2	BMP4
Pubmed	21108609	The relationship between human leukocyte antigens (HLA) and renal cell carcinoma.	GeneRIF	0.00875	129	0.0149	0.152	1	2	HLA-DQB1
Pubmed	20727355	Loss of claudin-15, but not claudin-2, causes Na+ deficiency and glucose malabsorption in mouse small intestine.	GeneRIF	0.00875	129	0.0149	0.152	1	2	CLDN15
Pubmed	23633509	ProNGF promotes neurite growth from a subset of NGF-dependent neurons by a p75NTR-dependent mechanism.	GeneRIF	0.00875	129	0.0149	0.152	1	2	NGFR
Pubmed	17936227	Genetic insights into the disease mechanisms of type II mixed cryoglobulinemia induced by hepatitis C virus.	GeneRIF	0.00875	129	0.0149	0.152	1	2	HLA-DQB1
Pubmed	15634938	Requirement for CD28 may not be absolute for collagen-induced arthritis: study with HLA-DQ8 transgenic mice.	Pubmed	0.00875	129	0.0149	0.152	1	2	HLA-DQB1
Pubmed	16368540	Transglutaminases in mineralized tissues.	GeneRIF	0.00875	129	0.0149	0.152	1	2	TGM2
Pubmed	9565553	Identification and characterization of a novel human aldose reductase-like gene.	Pubmed	0.00875	129	0.0149	0.152	1	2	AKR1B10
Pubmed	17287525	Hypo-osmolar stress induces p75NTR expression by activating Sp1-dependent transcription.	GeneRIF	0.00875	129	0.0149	0.152	1	2	NGFR
Pubmed	23693078	Insulin receptor tyrosine kinase substrate activates EGFR/ERK signalling pathway and promotes cell proliferation of hepatocellular carcinoma.	GeneRIF	0.00875	129	0.0149	0.152	1	2	BAIAP2L1
Pubmed	19926848	Gaining ligand selectivity in thyroid hormone receptors via entropy.	GeneRIF	0.00875	129	0.0149	0.152	1	2	THRA
Pubmed	19269807	Differential CD74 (major histocompatibility complex Class II invariant chain) expression in mouse and human intestinal adenomas.	Pubmed	0.00875	129	0.0149	0.152	1	2	CD74
Pubmed	18343220	The extracellular matrix protein fibronectin is a substrate for kallikrein 7.	GeneRIF	0.00875	129	0.0149	0.152	1	2	KLK7
Pubmed	17610416	Relative predispositional effects of HLA class II DRB1-DQB1 haplotypes and genotypes on type 1 diabetes: a meta-analysis.	GeneRIF	0.00875	129	0.0149	0.152	1	2	HLA-DQB1
Pubmed	23261458	Angiopoietin-like protein 2, a chronic inflammatory mediator, is a new target induced by TGF-ß1 through a Smad3-dependent mechanism.	GeneRIF	0.00875	129	0.0149	0.152	1	2	ANGPTL2
Pubmed	16297186	The association between HLA DR, DQ antigens, and vulval lichen sclerosus in the UK: HLA DRB112 and its associated DRB112/DQB10301/04/09/010 haplotype confers susceptibility to vulval lichen sclerosus, and HLA DRB10301/04 and its associated DRB10301/04/DQB10201/02/03 haplotype protects from vulval lichen sclerosus.	GeneRIF	0.00875	129	0.0149	0.152	1	2	HLA-DQB1
Pubmed	11290790	Relaxed DM requirements during class II peptide loading and CD4+ T cell maturation in BALB/c mice.	Pubmed	0.00875	129	0.0149	0.152	1	2	CD74
Pubmed	22564617	Lipopolysaccharide increases monocyte binding to mesangial cells through fractalkine and its receptor.	GeneRIF	0.00875	129	0.0149	0.152	1	2	CX3CL1
Pubmed	11053637	HLA-DRB1 and DQB1 polymorphisms in southern France and genetic relationships with other Mediterranean populations.	GeneRIF	0.00875	129	0.0149	0.152	1	2	HLA-DQB1
Pubmed	12527373	Normal function of HERG K+ channels expressed in HEK293 cells requires basal protein kinase B activity.	GeneRIF	0.00875	129	0.0149	0.152	1	2	KCNH2
Pubmed	10604301	Mouse genetics: a tool to help unlock the mechanisms of glaucoma.	Pubmed	0.00875	129	0.0149	0.152	1	2	GPNMB
Pubmed	24013014	Sympathetic denervation of peri-infarct myocardium requires the p75 neurotrophin receptor.	Pubmed	0.00875	129	0.0149	0.152	1	2	NGFR
Pubmed	12151090	Pronapsin A and B gene expression in normal and malignant human lung and mononuclear blood cells.	GeneRIF	0.00875	129	0.0149	0.152	1	2	NAPSA
Pubmed	9886271	Confocal laser scanning analysis of the association of fibulin-2 with fibrillin-1 and fibronectin define different stages of skin regeneration.	Pubmed	0.00875	129	0.0149	0.152	1	2	FBLN2
Pubmed	15037989	Genetic prediction of type 1 diabetes in a population with low frequency of HLA risk genotypes and low incidence of the disease (the DIABFIN study).	GeneRIF	0.00875	129	0.0149	0.152	1	2	HLA-DQB1
Pubmed	22556407	Heparan sulfate facilitates FGF and BMP signaling to drive mesoderm differentiation of mouse embryonic stem cells.	GeneRIF	0.00875	129	0.0149	0.152	1	2	BMP4
Pubmed	22913598	HLA-associated phenotypes in youth with autoimmune diabetes.	GeneRIF	0.00875	129	0.0149	0.152	1	2	HLA-DQB1
Pubmed	24327733	PI3K regulates MEK/ERK signaling in breast cancer via the Rac-GEF, P-Rex1.	Pubmed	0.00875	129	0.0149	0.152	1	2	PREX1
Pubmed	19664674	HLA-DRB1 and -DQB1 loci in three west African ethnic groups: genetic relationship with sub-Saharan African and European populations.	GeneRIF	0.00875	129	0.0149	0.152	1	2	HLA-DQB1
Pubmed	21408024	Renal thrombotic microangiopathy in mice with combined deletion of endocytic recycling regulators EHD3 and EHD4.	GeneRIF	0.00875	129	0.0149	0.152	1	2	EHD3
Pubmed	16418402	Membrane-delimited inhibition of maxi-K channel activity by the intermediate conductance Ca2+-activated K channel.	GeneRIF	0.00875	129	0.0149	0.152	1	2	KCNN4
Pubmed	17099142	Novel role of the human alveolar epithelium in regulating intra-alveolar coagulation.	GeneRIF	0.00875	129	0.0149	0.152	1	2	PROCR
Pubmed	18706091	Association of narcolepsy-cataplexy with HLA-DRB1 and DQB1 in Mexican patients: a relationship between HLA and gender is suggested.	GeneRIF	0.00875	129	0.0149	0.152	1	2	HLA-DQB1

Category	ID	Name	Source	p-value	q-value Bonferroni	q-value FDR B&H	q-value FDR B&Y	Hit Count in Query List	Hit Count in Genome	Hit in Query List
Pubmed	20940247	Deregulation of LOXL1 and HTRA1 gene expression in endometriosis.	GeneRIF	0.00875	129	0.0149	0.152	1	2	HTRA1
Pubmed	23272095	A dual reporter mouse model of the human ß-globin locus: applications and limitations.	GeneRIF	0.00875	129	0.0149	0.152	1	2	HBB
Pubmed	12684625	Expression of matrix metalloproteinases 7 and 2 in human renal cell carcinoma.	GeneRIF	0.00875	129	0.0149	0.152	1	2	MMP7
Pubmed	7378053	Haemoglobin binding with haptoglobin. Unequivocal demonstration that the beta-chains of human haemoglobin bind to haptoglobin.	Pubmed	0.00875	129	0.0149	0.152	1	2	HBB
Pubmed	19342667	Protective response to Leishmania major in BALB/c mice requires antigen processing in the absence of DM.	Pubmed	0.00875	129	0.0149	0.152	1	2	CD74
Pubmed	14990915	Association between HLA class II locus and the susceptibility to Artemisia pollen-induced allergic rhinitis in Chinese population.	GeneRIF	0.00875	129	0.0149	0.152	1	2	HLA-DQB1
Pubmed	20408813	Identification of the structural motif responsible fo trimeric assembly of the C-terminal regulatory domains of polycystin channels PKD2L1 and PKD2.	GeneRIF	0.00875	129	0.0149	0.152	1	2	PKD2L1
Pubmed	19561379	Association of HLA-DQA1*0101/2 and DQB1*0502 with myasthenia gravis in southern Iranian patients.	GeneRIF	0.00875	129	0.0149	0.152	1	2	HLA-DQB1
Pubmed	19657147	Increased TG2 expression can result in induction of transforming growth factor beta1, causing increased synthesis and deposition of matrix proteins, which can be regulated by nitric oxide.	GeneRIF	0.00875	129	0.0149	0.152	1	2	TGM2
Pubmed	20301599	Beta-Thalassemia	Pubmed	0.00875	129	0.0149	0.152	1	2	HBB
Pubmed	16767164	Identification of a region on the outer surface of th CBFbeta-SMMHC myeloid oncoprotein assembly competence domain critical for multimerization.	GeneRIF	0.00875	129	0.0149	0.152	1	2	MYH11
Pubmed	16502584	Diagnosis and monitoring of CBFB-MYH11-positive acute myeloid leukemia by qualitative and quantitative RT-PCR.	GeneRIF	0.00875	129	0.0149	0.152	1	2	MYH11
Pubmed	22594481	Distribution of HLA-DRB1 and HLA-DQB1 families of alleles and haplotypes in Vojvodina population.	GeneRIF	0.00875	129	0.0149	0.152	1	2	HLA-DQB1
Pubmed	17065405	Thyroid hormone-stimulated differentiation of primary rib chondrocytes in vitro requires thyroid hormone receptor beta.	Pubmed	0.00875	129	0.0149	0.152	1	2	THRA
Pubmed	12530663	Function of the transcriptional regulating protein of 132 kDa (TReP-132) on human P450scc gene expression.	GeneRIF	0.00875	129	0.0149	0.152	1	2	CYP11A1
Pubmed	2502420	Interferon-gamma activates multiple pathways to regulate the expression of the genes for major histocompatibility class II I-A beta, tumor necrosis factor and complement component C3 in mouse macrophages.	Pubmed	0.00875	129	0.0149	0.152	1	2	HLA-DQB1
Pubmed	22086417	A genome-wide meta-analysis of nodular sclerosing Hodgkin lymphoma identifies risk loci at 6p21.32.	Pubmed	0.00875	129	0.0149	0.152	1	2	HLA-DQB1
Pubmed	19188668	Role of activated protein C and its receptor in inhibition of tumor metastasis.	GeneRIF	0.00875	129	0.0149	0.152	1	2	PROCR
Pubmed	18561316	Genetic alterations of HLA-class II in ovarian cancer.	GeneRIF	0.00875	129	0.0149	0.152	1	2	HLA-DQB1
Pubmed	11777987	Allergic inflammatory response to short ragweed allergenic extract in HLA-DQ transgenic mice lacking CD4 gene.	Pubmed	0.00875	129	0.0149	0.152	1	2	HLA-DQB1
Pubmed	19208355	The HLA alleles DRB1*13 and DQB1*06 are associated to Whipple's disease.	GeneRIF	0.00875	129	0.0149	0.152	1	2	HLA-DQB1
Pubmed	19074585	Thyroid hormone receptor-beta is associated with coronary angiogenesis during pathological cardiac hypertrophy.	Pubmed	0.00875	129	0.0149	0.152	1	2	THRA
Pubmed	9870956	Absence of the p75 neurotrophin receptor alters th pattern of sympathosensory sprouting in the trigeminal ganglia of mice overexpressing nerve growth factor.	Pubmed	0.00875	129	0.0149	0.152	1	2	NGFR
Pubmed	18772456	G6PD deficiency, absence of alpha-thalassemia, and hemolytic rate at baseline are significant independent risk factors for abnormally high cerebral velocities in patients with sickle cell anemia.	GeneRIF	0.00875	129	0.0149	0.152	1	2	HBB
Pubmed	20015957	Specific saposin C deficiency: CNS impairment and acid beta-glucosidase effects in the mouse.	GeneRIF	0.00875	129	0.0149	0.152	1	2	PSAP
Pubmed	23427152	Herpes simplex virus 1 infection induces activatior and subsequent inhibition of the IFI16 and NLRP3 inflammasomes.	GeneRIF	0.00875	129	0.0149	0.152	1	2	IFI16
Pubmed	21930648	Differential glycosylation of a-dystroglycan and proteins other than a-dystroglycan by like-glycosyltransferase.	GeneRIF	0.00875	129	0.0149	0.152	1	2	LARGE
Pubmed	11997516	Conserved CTCF insulator elements flank the mouse and human beta-globin loci.	GeneRIF	0.00875	129	0.0149	0.152	1	2	HBB
Pubmed	11944901	The novel Rab11-FIP/Rip/RCP family of proteins displays extensive homo- and hetero-interacting abilities.	Pubmed	0.00875	129	0.0149	0.152	1	2	RAB11FIP1
Pubmed	14500633	In vivo treatment of class II MHC-deficient mice with anti-TCR antibody restores the generation of circulating CD4 T cells and optimal architecture of thymic medulla.	Pubmed	0.00875	129	0.0149	0.152	1	2	HLA-DQB1
Pubmed	23582991	Genetic influences on the outcome of anti-vascular endothelial growth factor treatment in neovascular age-related macular degeneration.	Pubmed	0.00875	129	0.0149	0.152	1	2	HTRA1
Pubmed	11970984	Aberrant expression of Fas ligand in mice deficien for the MHC class II transactivator.	GeneRIF	0.00875	129	0.0149	0.152	1	2	HLA-DQB1
Pubmed	22491416	Differentiation of exudative age-related macular degeneration and polypoidal choroidal vasculopathy in the ARMS2/HTRA1 locus.	GeneRIF	0.00875	129	0.0149	0.152	1	2	HTRA1
Pubmed	18024075	Hyperglycaemia inhibits Schwann cell proliferation and migration and restricts regeneration of axons and Schwann cells from adult murine DRG.	Pubmed	0.00875	129	0.0149	0.152	1	2	NGFR
Pubmed	12045205	Functional analysis of bone morphogenetic protein type II receptor mutations underlying primary pulmonary hypertension.	Pubmed	0.00875	129	0.0149	0.152	1	2	BMP4
Pubmed	21203384	Transgenic overexpression of LARGE induces a-dystroglycan hyperglycosylation in skeletal and cardiac muscle.	GeneRIF	0.00875	129	0.0149	0.152	1	2	LARGE

Category	ID	Name	Source	p-value	q-value Bonferroni	q-value FDR B&H	q-value FDR B&Y	Hit Count in Query List	Hit Count in Genome	Hit in Query List
Pubmed	2347585	Human delta-aminolevulinate synthase: assignment of the housekeeping gene to 3p21 and the erythroid-specific gene to the X chromosome.	Pubmed	0.00875	129	0.0149	0.152	1	2	ALAS2
Pubmed	16505033	Quantitative correlation of optic nerve pathology with ocular pressure and corneal thickness in the DBA/2 mouse model of glaucoma.	Pubmed	0.00875	129	0.0149	0.152	1	2	GPNMB
Pubmed	11485744	MASP-3 and its association with distinct complexes of the mannan-binding lectin complement activation pathway.	Pubmed	0.00875	129	0.0149	0.152	1	2	MASP1
Pubmed	19404200	Tropical calcific pancreatitis and its association with CTRC and SPINK1 (p.N34S) variants.	GeneRIF	0.00875	129	0.0149	0.152	1	2	SPINK1
Pubmed	18580441	SPINK1 and PRSS1 mutations in benign pancreatic hyperenzymemia.	GeneRIF	0.00875	129	0.0149	0.152	1	2	SPINK1
Pubmed	20445563	Murine lupus susceptibility locus Sle1a requires the expression of two sub-loci to induce inflammatory T cells.	Pubmed	0.00875	129	0.0149	0.152	1	2	HLA-DQB1
Pubmed	15131578	Expression of fractalkine and its receptor, CX3CR1, in atopic dermatitis: possible contribution to skin inflammation.	GeneRIF	0.00875	129	0.0149	0.152	1	2	CX3CL1
Pubmed	22570333	Fundamentally distinct roles of thyroid hormone receptor isoforms in a thyrotroph cell line are due to differential DNA binding.	GeneRIF	0.00875	129	0.0149	0.152	1	2	THRA
Pubmed	19654461	[Pancreatitis-associated genes and development of pancreatic cancer].	GeneRIF	0.00875	129	0.0149	0.152	1	2	SPINK1
Pubmed	18172431	Epigenetic downregulation of atrial natriuretic peptide but not vasopressin mRNA expression in females with eating disorders is related to impulsivity.	GeneRIF	0.00875	129	0.0149	0.152	1	2	NPPA
Pubmed	22404765	The influence of the HLA-DRB1 and HLA-DQB1 allele heterogeneity on disease risk and severity in Iranian patients with multiple sclerosis.	GeneRIF	0.00875	129	0.0149	0.152	1	2	HLA-DQB1
Pubmed	20573231	Bone morphogenetic protein (BMP)-4 and BMP-7 regulate differentially transforming growth factor (TGF)-beta1 in normal human lung fibroblasts (NHLF).	GeneRIF	0.00875	129	0.0149	0.152	1	2	BMP4
Pubmed	23653073	Differential endothelial coverage, response to injury and neointimal integration of CX3CR1/smooth muscle-like cells after carotid or femoral arterial injury.	GeneRIF	0.00875	129	0.0149	0.152	1	2	CX3CL1
Pubmed	22465227	Absence of T and B lymphocytes modulates dystrophic features in dysferlin deficient animal model.	Pubmed	0.00875	129	0.0149	0.152	1	2	DYSF
Pubmed	22883281	Matrix metalloproteinase-7 and matrix metalloproteinase-9 in pediatric multiple sclerosis.	GeneRIF	0.00875	129	0.0149	0.152	1	2	MMP7
Pubmed	12859959	Activation of transglutaminase in mu-calpain null erythrocytes.	GeneRIF	0.00875	129	0.0149	0.152	1	2	TGM2
Pubmed	6317690	Boundaries of gene conversion within the duplicated human alpha-globin genes. Concerted evolution by segmental recombination.	Pubmed	0.00875	129	0.0149	0.152	1	2	HBA2
Pubmed	2780318	Structure of a mouse erythroid 5-aminolevulinate synthase gene and mapping of erythroid-specific DNAse I hypersensitive sites.	Pubmed	0.00875	129	0.0149	0.152	1	2	ALAS2
Pubmed	23459095	A high-density tissue microarray from patients with clinically localized prostate cancer reveals ERG and TATI exclusivity in tumor cells.	Pubmed	0.00875	129	0.0149	0.152	1	2	SPINK1
Pubmed	11485993	Distinct mesodermal signals, including BMPs from the septum transversum mesenchyme, are required in combination for hepatogenesis from the endoderm.	Pubmed	0.00875	129	0.0149	0.152	1	2	BMP4
Pubmed	21036738	Up-regulation of fibronectin and tissue transglutaminase promotes cell invasion involving increased association with integrin and MMP expression in A431 cells.	GeneRIF	0.00875	129	0.0149	0.152	1	2	TGM2
Pubmed	17008454	Atrophy-related ubiquitin ligases atrogin-1 and MuRF-1 are associated with uterine smooth muscle involution in the postpartum period.	GeneRIF	0.00875	129	0.0149	0.152	1	2	FBXO32
Pubmed	11410421	Alpha2(CD31 AGG-->AAG, Arg-->Lys) causing non-deletional alpha-thalassemia in a Chinese family with HbH disease.	Pubmed	0.00875	129	0.0149	0.152	1	2	HBA2
Pubmed	23382862	P-Rex1 cooperates with PDGFRß to drive cellular migration in 3D microenvironments.	GeneRIF	0.00875	129	0.0149	0.152	1	2	PREX1
Pubmed	21927809	Mutation screening of BMP4 and Id2 genes in Chinese patients with congenital ureteropelvic junction obstruction.	GeneRIF	0.00875	129	0.0149	0.152	1	2	BMP4
Pubmed	24798332	Identification of key residues and regions important for porcupine-mediated Wnt acylation.	Pubmed	0.00875	129	0.0149	0.152	1	2	PORCN
Pubmed	2848682	Quantification of P450scc, P450(17) alpha, and iron sulfur protein reductase in Leydig cells and adrenals of inbred strains of mice.	Pubmed	0.00875	129	0.0149	0.152	1	2	CYP11A1
Pubmed	19464260	Acetic acid activates PKD1L3-PKD2L1 channel--a candidate sour taste receptor.	GeneRIF	0.00875	129	0.0149	0.152	1	2	PKD2L1
Pubmed	22123179	CX(3)CR1 drives cytotoxic CD4(+)CD28(-) T cells into the brain of multiple sclerosis patients.	GeneRIF	0.00875	129	0.0149	0.152	1	2	CX3CL1
Pubmed	15337528	c-Cbl binds to tyrosine-phosphorylated neurotrophin receptor p75 and induces its ubiquitination.	GeneRIF	0.00875	129	0.0149	0.152	1	2	NGFR
Pubmed	2483097	A Purkinje cell differentiation marker shows a partial DNA sequence homology to the cellular sis/PDGF2 gene.	Pubmed	0.00875	129	0.0149	0.152	1	2	PCP4
Pubmed	20036214	Sphingosine-1-phosphate receptor S1P1 is regulated by direct interactions with P-Rex1, a Rac guanine nucleotide exchange factor.	GeneRIF	0.00875	129	0.0149	0.152	1	2	PREX1
Pubmed	10377349	Functionally antagonistic interactions between the TrkA and p75 neurotrophin receptors regulate sympathetic neuron growth and target innervation.	Pubmed	0.00875	129	0.0149	0.152	1	2	NGFR
Pubmed	8133077	Localization of activin subunits in early murine development determined by subunit-specific antibodies.	Pubmed	0.00875	129	0.0149	0.152	1	2	INHBB
Pubmed	8258349	Major histocompatibility complex class I molecules are required for the development of insulitis in non-obese diabetic mice.	Pubmed	0.00875	129	0.0149	0.152	1	2	HLA-DQB1

Category	ID	Name	Source	p-value	q-value Bonferroni	q-value FDR B&H	q-value FDR B&Y	Hit Count in Query List	Hit Count in Genome	Hit in Query List
Pubmed	12164779	Endothelial cell-derived lipase mediates uptake and binding of high-density lipoprotein (HDL) particles and the selective uptake of HDL-associated cholesterol esters independent of its enzymic activity.	GeneRIF	0.00875	129	0.0149	0.152	1	2	LIPG
Pubmed	21636783	Structural basis for pregnenolone biosynthesis by the mitochondrial monooxygenase system.	GeneRIF	0.00875	129	0.0149	0.152	1	2	CYP11A1
Pubmed	16231148	Association of HLA-DQA1 and DQB1 alleles with alolpecia areata in Chinese Hans.	GeneRIF	0.00875	129	0.0149	0.152	1	2	HLA-DQB1
Pubmed	6407984	H-2 haplotypes, genes and antigens: second listing. II. The H-2 complex.	Pubmed	0.00875	129	0.0149	0.152	1	2	HLA-DQB1
Pubmed	15985473	Contribution of selective HLA-DRB1/DQB1 alleles and haplotypes to the genetic susceptibility of type 1 diabetes among Lebanese and Bahraini Arabs.	GeneRIF	0.00875	129	0.0149	0.152	1	2	HLA-DQB1
Pubmed	20193456	[Correlation between HLA-DRB1*0901 and 1501, HLA-DQB1*0301 and 0602 alleles and esophageal squamous cell carcinoma of Kazakh in Xinjiang, China].	GeneRIF	0.00875	129	0.0149	0.152	1	2	HLA-DQB1
Pubmed	10493778	Identification of a missense mutation in the alphaA crystallin gene of the lop18 mouse.	Pubmed	0.00875	129	0.0149	0.152	1	2	CRYAA
Pubmed	20337975	Type 1 diabetes patients born to immigrants to Sweden increase their native diabetes risk and differ from Swedish patients in HLA types and islet autoantibodies.	GeneRIF	0.00875	129	0.0149	0.152	1	2	HLA-DQB1
Pubmed	21285453	Inhibin B and anti-Müllerian hormone in spermatic vein of subfertile men with varicocele.	GeneRIF	0.00875	129	0.0149	0.152	1	2	INHBB
Pubmed	23258247	Associations of daily walking activity with biomarkers related to cardiac distress in patients with chronic obstructive pulmonary disease.	GeneRIF	0.00875	129	0.0149	0.152	1	2	NPPA
Pubmed	19193386	Bone marrow stromal cell therapy reduces proNGF and p75 expression in mice with experimental autoimmune encephalomyelitis.	GeneRIF	0.00875	129	0.0149	0.152	1	2	NGFR
Pubmed	20606707	Quantitative biometric phenotype analysis in mouse lenses.	Pubmed	0.00875	129	0.0149	0.152	1	2	CRYAA
Pubmed	23470165	Interaction between CX3CL1 and CX3CR1 regulates vasculitis induced by immune complex deposition.	Pubmed	0.00875	129	0.0149	0.152	1	2	CX3CL1
Pubmed	10220431	CD4(+) T cell-mediated control of a gamma-herpesvirus in B cell-deficient mice is mediated by IFN-gamma.	Pubmed	0.00875	129	0.0149	0.152	1	2	HLA-DQB1
Pubmed	15780976	Increased adipogenesis in bone marrow but decreased bone mineral density in mice devoid of thyroid hormone receptors.	Pubmed	0.00875	129	0.0149	0.152	1	2	THRA
Pubmed	15245369	Molecular basis of predisposition to develop type 1 diabetes mellitus in North Indians.	GeneRIF	0.00875	129	0.0149	0.152	1	2	HLA-DQB1
Pubmed	20603341	Number of autoantibodies and HLA genotype, more than high titers of glutamic acid decarboxylase autoantibodies, predict insulin dependence in latent autoimmune diabetes of adults.	GeneRIF	0.00875	129	0.0149	0.152	1	2	HLA-DQB1
Pubmed	20126398	Differential expression of hERG1 channel isoforms reproduces properties of native I(Kr) and modulates cardiac action potential characteristics.	GeneRIF	0.00875	129	0.0149	0.152	1	2	KCNH2
Pubmed	22924376	Identification of a-globin chain variants: a report from Iran.	GeneRIF	0.00875	129	0.0149	0.152	1	2	HBA2
Pubmed	10449762	Expression pattern of the mouse ortholog of the Pendred's syndrome gene (Pds) suggests a key role for pendrin in the inner ear.	Pubmed	0.00875	129	0.0149	0.152	1	2	BMP4
Pubmed	22524815	Application of MMP-7 and MMP-10 in assisting the diagnosis of malignant pleural effusion.	GeneRIF	0.00875	129	0.0149	0.152	1	2	MMP7
Pubmed	15149334	Matrilysin (MMP-7) expression in renal tubular damage: association with Wnt4.	GeneRIF	0.00875	129	0.0149	0.152	1	2	MMP7
Pubmed	15055351	HLA alleles and type 1 diabetes mellitus in low disease incidence populations of Southern Europe: a comparison of Greeks and Albanians.	GeneRIF	0.00875	129	0.0149	0.152	1	2	HLA-DQB1
Pubmed	1870127	Three-dimensional structure of the complexes between bovine chymotrypsinogen A and two recombinant variants of human pancreatic secretory trypsin inhibitor (Kazal-type).	Pubmed	0.00875	129	0.0149	0.152	1	2	SPINK1
Pubmed	16012168	Alpha-crystallin is a target gene of the farnesoid X-activated receptor in human livers.	GeneRIF	0.00875	129	0.0149	0.152	1	2	CRYAA
Pubmed	22861372	Anti-interleukin-12/23p40 antibody attenuates chronic rejection of cardiac allografts partly via inhibition ¿dT cells.	Pubmed	0.00875	129	0.0149	0.152	1	2	HLA-DQB1
Pubmed	16916661	HLA DQA1*0501 and DQB1*02 in Cuban celiac patients.	GeneRIF	0.00875	129	0.0149	0.152	1	2	HLA-DQB1
Pubmed	16261886	Association between pemphigus vulgaris and human leukocyte antigen in Han nation of northeast China.	GeneRIF	0.00875	129	0.0149	0.152	1	2	HLA-DQB1
Pubmed	12887819	[HLA-DRB and -DQB allele contribution to narcolepsy susceptibility in Chinese patients with narcolepsy].	GeneRIF	0.00875	129	0.0149	0.152	1	2	HLA-DQB1
Pubmed	9886305	A new sickle cell disease phenotype associating Hb S trait, severe pyruvate kinase deficiency (PK Conakry), and an alpha2 globin gene variant (Hb Conakry).	Pubmed	0.00875	129	0.0149	0.152	1	2	HBA2
Pubmed	2395459	Atomic structure of the actin:DNase I complex.	Pubmed	0.00875	129	0.0149	0.152	1	2	ACTA1
Pubmed	17347473	Upregulation of macrophage endothelial lipase by toll-like receptors 4 and 3 modulates macrophage interleukin-10 and -12 production.	Pubmed	0.00875	129	0.0149	0.152	1	2	LIPG
Pubmed	21067613	Genetic variation in the odorant receptors family 13 and the mhc loci influence mate selection in a multiple sclerosis dataset.	GeneRIF	0.00875	129	0.0149	0.152	1	2	HLA-DQB1
Pubmed	2882518	The first external domain of the nonobese diabetic mouse class II I-A beta chain is unique.	Pubmed	0.00875	129	0.0149	0.152	1	2	HLA-DQB1
Pubmed	22228808	Interferon-¿ activates transglutaminase 2 via a phosphatidylinositol-3-kinase-dependent pathway: implications for celiac sprue therapy.	GeneRIF	0.00875	129	0.0149	0.152	1	2	TGM2
Pubmed	15772115	Histone deacetylase HDAC8 associates with smooth muscle alpha-actin and is essential for smooth muscle cell contractility.	GeneRIF	0.00875	129	0.0149	0.152	1	2	ACTA1
Pubmed	14716313	Saposin C is required for lipid presentation by human CD1b.	GeneRIF	0.00875	129	0.0149	0.152	1	2	PSAP

Category	ID	Name	Source	p-value	q-value Bonferroni	q-value FDR B&H	q-value FDR B&Y	Hit Count in Query List	Hit Count in Genome	Hit in Query List
Pubmed	2879243	The c-erb-A gene encodes a thyroid hormone receptor.	Pubmed	0.00875	129	0.0149	0.152	1	2	THRA
Pubmed	12019147	Oncogenic beta-catenin is required for bone morphogenetic protein 4 expression in human cancer cells.	GeneRIF	0.00875	129	0.0149	0.152	1	2	BMP4
Pubmed	24392021	Endocytosis of HERG is clathrin-independent and involves arf6.	Pubmed	0.00875	129	0.0149	0.152	1	2	KCNH2
Pubmed	17996891	Corin is co-expressed with pro-ANP and localized on the cardiomyocyte surface in both zymogen and catalytically active forms.	GeneRIF	0.00875	129	0.0149	0.152	1	2	NPPA
Pubmed	11105903	Targeted disruption of the mouse prosaposin gene affects the development of the prostate gland and other male reproductive organs.	Pubmed	0.00875	129	0.0149	0.152	1	2	PSAP
Pubmed	9250685	The T3R alpha gene encoding a thyroid hormone receptor is essential for post-natal development and thyroid hormone production.	Pubmed	0.00875	129	0.0149	0.152	1	2	THRA
Pubmed	19923894	Interplay between BMP4 and IL-7 in human intrathymic precursor cells.	GeneRIF	0.00875	129	0.0149	0.152	1	2	BMP4
Pubmed	12453977	Association of HLA-DQ genotype in autoantibody-negative and rapid-onset type 1 diabetes.	GeneRIF	0.00875	129	0.0149	0.152	1	2	HLA-DQB1
Pubmed	25917967	Atrial natriuretic peptide and aldosterone synthase gene in essential hypertension: a case-control study.	Pubmed	0.00875	129	0.0149	0.152	1	2	NPPA
Pubmed	21071446	Fractalkine attenuates excito-neurotoxicity via microglial clearance of damaged neurons and antioxidant enzyme heme oxygenase-1 expression.	GeneRIF	0.00875	129	0.0149	0.152	1	2	CX3CL1
Pubmed	11238035	The transmembrane form of the CX3CL1 chemokine fractalkine is expressed predominantly by epithelial cells in vivo.	Pubmed	0.00875	129	0.0149	0.152	1	2	CX3CL1
Pubmed	19643138	Over-expression of integrin beta3 can partially overcome the defect of integrin beta3 signaling in transglutaminase 2 null macrophages.	GeneRIF	0.00875	129	0.0149	0.152	1	2	TGM2
Pubmed	16911023	Interactions between fetal HLA-DQ alleles and maternal smoking influence birthweight.	GeneRIF	0.00875	129	0.0149	0.152	1	2	HLA-DQB1
Pubmed	23341620	SLC4A2-mediated Cl-/HCO3- exchange activity is essential for calpain-dependent regulation of the actin cytoskeleton in osteoclasts.	GeneRIF	0.00875	129	0.0149	0.152	1	2	CTSK
Pubmed	12543794	Association between human leukocyte antigen polymorphism and human papillomavirus 16-positive vulval intraepithelial neoplasia in British women.	GeneRIF	0.00875	129	0.0149	0.152	1	2	HLA-DQB1
Pubmed	21695131	Osteopenia due to enhanced cathepsin K release by BK channel ablation in osteoclasts.	GeneRIF	0.00875	129	0.0149	0.152	1	2	CTSK
Pubmed	19699172	A role for lipid bodies in the cross-presentation of phagocytosed antigens by MHC class I in dendritic cells.	Pubmed	0.00875	129	0.0149	0.152	1	2	HLA-DQB1
Pubmed	18684948	The MHC class II-associated invariant chain interacts with the neonatal Fc gamma receptor and modulates its trafficking to endosomal/lysosomal compartments.	GeneRIF	0.00875	129	0.0149	0.152	1	2	CD74
Pubmed	15941540	[Immunogenetic analysis of human leukocyte antigen DRB1, DQB1 locus among Han ethnic children with Helicobacter pylori infection in Kunming].	GeneRIF	0.00875	129	0.0149	0.152	1	2	HLA-DQB1
Pubmed	19587117	The class II phosphatidylinositol 3 kinase C2beta is required for the activation of the K+ channel KCa3.1 and CD4 T-cells.	GeneRIF	0.00875	129	0.0149	0.152	1	2	KCNN4
Pubmed	17513739	Distinct subsets of FoxP3+ regulatory T cells participate in the control of immune responses.	Pubmed	0.00875	129	0.0149	0.152	1	2	HLA-DQB1
Pubmed	16484124	Gene dose effect of the DQB1*0201 allele contributes to severity of coeliac disease.	GeneRIF	0.00875	129	0.0149	0.152	1	2	HLA-DQB1
Pubmed	21185261	The single pore residue Asp523 in PKD2L1 determines Ca2+ permeation of the PKD1L3/PKD2L1 complex.	GeneRIF	0.00875	129	0.0149	0.152	1	2	PKD2L1
Pubmed	19490633	Nerve growth factor and receptor expression in rheumatoid arthritis and spondyloarthritis.	GeneRIF	0.00875	129	0.0149	0.152	1	2	NGFR
Pubmed	19969308	alpha Pix enhances mutant huntingtin aggregation.	GeneRIF	0.00875	129	0.0149	0.152	1	2	ARHGEF6
Pubmed	10710367	Modulation of mouse cardiac function in vivo by eNOS and ANP.	Pubmed	0.00875	129	0.0149	0.152	1	2	NPPA
Pubmed	16125818	Clock gene polymorphisms and narcolepsy in positive and negative HLA-DQB1*0602 patients.	GeneRIF	0.00875	129	0.0149	0.152	1	2	HLA-DQB1
Pubmed	12694585	Genetic and functional studies in multiple sclerosis patients from Martinique attest for a specific and direct role of the HLA-DR locus in the syndrome.	GeneRIF	0.00875	129	0.0149	0.152	1	2	HLA-DQB1
Pubmed	16327984	Differential expression of trypsinogen and tumor-associated trypsin inhibitor (TATI) in bladder cancer.	GeneRIF	0.00875	129	0.0149	0.152	1	2	SPINK1
Pubmed	24203699	Membrane damage-induced vesicle-vesicle fusion of dysferlin-containing vesicles in muscle cells requires microtubules and kinesin.	Pubmed	0.00875	129	0.0149	0.152	1	2	DYSF
Pubmed	18092954	Atrial natriuretic peptide and CD34 overexpression in human idiopathic dilated cardiomyopathies.	GeneRIF	0.00875	129	0.0149	0.152	1	2	NPPA
Pubmed	23029290	Fibroblast growth factor receptor 1 activation in mammary tumor cells promotes macrophage recruitment in a CX3CL1-dependent manner.	GeneRIF	0.00875	129	0.0149	0.152	1	2	CX3CL1
Pubmed	12034400	A role for matrix metalloproteinases and tumor host interaction in hepatocellular carcinomas.	Pubmed	0.00875	129	0.0149	0.152	1	2	MMP7
Pubmed	12690509	Molecular screening of selected long QT syndrome (LQTS) mutations in 165 consecutive bodies found in water.	GeneRIF	0.00875	129	0.0149	0.152	1	2	KCNH2
Pubmed	7609079	Interactions of thyroid hormone receptor with the human immunodeficiency virus type 1 (HIV-1) long terminal repeat and the HIV-1 Tat transactivator.	Pubmed	0.00875	129	0.0149	0.152	1	2	THRA
Pubmed	16972305	Gene profiling of cathepsin K deficiency in atherogenesis: profibrotic but lipogenic.	Pubmed	0.00875	129	0.0149	0.152	1	2	CTSK
Pubmed	19271144	Overexpression of histamine H(1)-receptor by human amniotic epithelial cells in chorioamnionitis correlates with augmented production of secretory leukocyte protease inhibitor.	GeneRIF	0.00875	129	0.0149	0.152	1	2	SLPI
Pubmed	23590659	Diagnostic testing for a-globin gene disorders in a heterogeneous North American population.	Pubmed	0.00875	129	0.0149	0.152	1	2	HBA2

Category	ID	Name	Source	p-value	q-value Bonferroni	q-value FDR B&H	q-value FDR B&Y	Hit Count in Query List	Hit Count in Genome	Hit in Query List
Pubmed	9226441	Inhibins and activins regulate mammary epithelial cell differentiation through mesenchymal-epithelial interactions.	Pubmed	0.00875	129	0.0149	0.152	1	2	INHBB
Pubmed	519760	The complete sequence of a chromosomal mouse alpha--globin gene reveals elements conserved throughout vertebrate evolution.	Pubmed	0.00875	129	0.0149	0.152	1	2	HBA2
Pubmed	17329206	A role for Nogo receptor in macrophage clearance from injured peripheral nerve.	GeneRIF	0.00875	129	0.0149	0.152	1	2	RTN4R
Pubmed	17571080	Comparative analysis of genes regulated in acute myelomonocytic leukemia with and without inv(16)(p13q22) using microarray techniques, real-time PCR, immunohistochemistry, and flow cytometry immunophenotyping.	GeneRIF	0.00875	129	0.0149	0.152	1	2	MYH11
Pubmed	9745505	Role of CD8+ and CD4+ T cells in the rejection of murine intestinal allografts.	Pubmed	0.00875	129	0.0149	0.152	1	2	HLA-DQB1
Pubmed	10964473	glypican-3 controls cellular responses to Bmp4 in limb patterning and skeletal development.	Pubmed	0.00875	129	0.0149	0.152	1	2	BMP4
Pubmed	2541064	Regional assignment of the loci for adenylate kinase to 9q32 and for alpha 1-acid glycoprotein to 9q31-q32. A locus for Goltz syndrome in region 9q32-qter?	Pubmed	0.00875	129	0.0149	0.152	1	2	ORM1
Pubmed	16208405	Association of HLA-DRB1*0901-DQB1*0303 haplotype with microscopic polyangiitis in Japanese.	GeneRIF	0.00875	129	0.0149	0.152	1	2	HLA-DQB1
Pubmed	9872742	Requirement for diverse, low-abundance peptides in positive selection of T cells.	Pubmed	0.00875	129	0.0149	0.152	1	2	CD74
Pubmed	17409252	Both doublecortin and doublecortin-like kinase play a role in cortical interneuron migration.	GeneRIF	0.00875	129	0.0149	0.152	1	2	DCLK1
Pubmed	19155311	Membrane microvesicles as actors in the establishment of a favorable prostatic tumoral niche: a role for activated fibroblasts and CX3CL1-CX3CR1 axis.	GeneRIF	0.00875	129	0.0149	0.152	1	2	CX3CL1
Pubmed	17651673	Variability in postheparin hepatic lipase activity is associated with plasma adiponectin levels in African Americans.	GeneRIF	0.00875	129	0.0149	0.152	1	2	LIPG
Pubmed	24068396	Immunohistochemical expression of RBP2 and LSD1 in papillary thyroid carcinoma.	Pubmed	0.00875	129	0.0149	0.152	1	2	KDM5A
Pubmed	24498905	Changes in liraglutide-induced body composition are related to modifications in plasma cardiac natriuretic peptides levels in obese type 2 diabetic patients.	Pubmed	0.00875	129	0.0149	0.152	1	2	NPPA
Pubmed	21996251	Utility of frozen cell lines in medium throughput electrophysiology screening of hERG and NaV1.5 blockade.	GeneRIF	0.00875	129	0.0149	0.152	1	2	KCNH2
Pubmed	18062951	Overexpression of pairedless Pax6 in the retina disrupts corneal development and affects lens cell survival.	GeneRIF	0.00875	129	0.0149	0.152	1	2	BMP4
Pubmed	15699500	Closer association of IA-2 humoral autoreactivity with HLA DR3/4 than DQB1*0201/*0302 in Korean T1D patients.	GeneRIF	0.00875	129	0.0149	0.152	1	2	HLA-DQB1
Pubmed	20855658	Transmural differences in myocardial contraction in long-QT syndrome: mechanical consequences of ion channel dysfunction.	GeneRIF	0.00875	129	0.0149	0.152	1	2	KCNH2
Pubmed	9695962	Haemopoietic transformation by the TEL/ABL oncogene.	Pubmed	0.00875	129	0.0149	0.152	1	2	ACTA1
Pubmed	24607897	Autophagy regulation in pancreatic acinar cells is independent of epidermal growth factor receptor signaling.	Pubmed	0.00875	129	0.0149	0.152	1	2	SPINK1
Pubmed	12048248	Crystal structures of the vitamin D-binding protein and its complex with actin: structural basis of the actin-scavenger system.	Pubmed	0.00875	129	0.0149	0.152	1	2	ACTA1
Pubmed	19330007	House dust mite allergen induces asthma via Toll-like receptor 4 triggering of airway structural cells.	GeneRIF	0.00875	129	0.0149	0.152	1	2	HLA-DQB1
Pubmed	23794145	MicroRNA-212 inhibits proliferation of gastric cancer by directly repressing retinoblastoma binding protein 2.	Pubmed	0.00875	129	0.0149	0.152	1	2	KDM5A
Pubmed	22944341	Clinical significance of MMP-7 and PTEN expression in colorectal cancer.	GeneRIF	0.00875	129	0.0149	0.152	1	2	MMP7
Pubmed	18832653	NKT-cell help to B lymphocytes can occur independently of cognate interaction.	Pubmed	0.00875	129	0.0149	0.152	1	2	HLA-DQB1
Pubmed	11756426	Amyloid beta binds trimers as well as monomers of the 75-kDa neurotrophin receptor and activates receptor signaling.	GeneRIF	0.00875	129	0.0149	0.152	1	2	NGFR
Pubmed	19745110	Bone marrow-derived CX3CR1 progenitors contribute to neointimal smooth muscle cells via fractalkine CX3CR1 interaction.	GeneRIF	0.00875	129	0.0149	0.152	1	2	CX3CL1
Pubmed	9512491	Phospholipase C-delta1 and oxytocin receptor signalling: evidence of its role as an effector.	Pubmed	0.00875	129	0.0149	0.152	1	2	TGM2
Pubmed	9341872	AFX1 and p54nrb: fine mapping, genomic structure, and exclusion as candidate genes of X-linked dystonia parkinsonism.	Pubmed	0.00875	129	0.0149	0.152	1	2	FOXO4
Pubmed	11401407	Cooperation of endoderm-derived BMP2 and extraembryonic ectoderm-derived BMP4 in primordial germ cell generation in the mouse.	Pubmed	0.00875	129	0.0149	0.152	1	2	BMP4
Pubmed	11496110	Audiogenic seizure susceptibility in thyroid hormone receptor beta-deficient mice.	Pubmed	0.00875	129	0.0149	0.152	1	2	THRA
Pubmed	18382462	Developmental axon pruning mediated by BDNF-p75NTR-dependent axon degeneration.	Pubmed	0.00875	129	0.0149	0.152	1	2	NGFR
Pubmed	16048795	Abnormal inhibin A and inhibin B secretion in obese women with and without insulin resistance.	GeneRIF	0.00875	129	0.0149	0.152	1	2	INHBB
Pubmed	20956329	T-cell receptor-driven lymphomagenesis in mice derived from a reprogrammed T cell.	Pubmed	0.00875	129	0.0149	0.152	1	2	HLA-DQB1
Pubmed	19824062	Low affinity NGF receptor (p75 neurotrophin receptor) inhibitory antibody reduces pain behavior and CGRP expression in DRG in the mouse sciatic nerve crush model.	GeneRIF	0.00875	129	0.0149	0.152	1	2	NGFR
Pubmed	11788435	Dietary salt supplementation selectively downregulates NPR-C receptor expression in kidney independently of ANP.	Pubmed	0.00875	129	0.0149	0.152	1	2	NPPA
Pubmed	8182124	Molecular cloning of the complementary DNA and gene that encode mouse brain natriuretic peptide and generation of transgenic mice that overexpress the brain natriuretic peptide gene.	Pubmed	0.00875	129	0.0149	0.152	1	2	NPPA
Pubmed	19210988	Gravin dynamics regulates the subcellular distribution of PKA.	GeneRIF	0.00875	129	0.0149	0.152	1	2	AKAP12
Pubmed	23085892	Human leukocyte antigen DQ2.2 and celiac disease.	GeneRIF	0.00875	129	0.0149	0.152	1	2	HLA-DQB1

Category	ID	Name	Source	p-value	q-value Bonferroni	q-value FDR B&H	q-value FDR B&Y	Hit Count in Query List	Hit Count in Genome	Hit in Query List
Pubmed	24323765	Carnosol, rosemary ingredient, induces apoptosis in adult T-cell leukemia/lymphoma cells via glutathione depletion: proteomic approach using fluorescent two-dimensional differential gel electrophoresis.	Pubmed	0.00875	129	0.0149	0.152	1	2	GSS
Pubmed	17327234	Endothelial cell protein C receptor acts as a cellular receptor for factor VIIa on endothelium.	GeneRIF	0.00875	129	0.0149	0.152	1	2	PROCR
Pubmed	14684150	3D structure of the CD26-ADA complex obtained by cryo-EM and single particle analysis.	Pubmed	0.00875	129	0.0149	0.152	1	2	ADA
Pubmed	19760608	Aberrant DNA methylation profile and frequent methylation of KLK10 and OXGR1 genes in hepatocellular carcinoma.	GeneRIF	0.00875	129	0.0149	0.152	1	2	KLK10
Pubmed	12466270	Characterization of the motor activity of mammalian myosin VIIA.	Pubmed	0.00875	129	0.0149	0.152	1	2	ACTA1
Pubmed	17612291	Identification of skeletal muscle mutations in tail snips from neonatal mice using immunohistochemistry.	GeneRIF	0.00875	129	0.0149	0.152	1	2	DYSF
Pubmed	14679080	Ethnic differences in the associations between the HLA-DRB1*04 subtypes and type 1 diabetes.	GeneRIF	0.00875	129	0.0149	0.152	1	2	HLA-DQB1
Pubmed	7503723	Direct binding of F actin to the cytoplasmic domain of the alpha 2 integrin chain in vitro.	Pubmed	0.00875	129	0.0149	0.152	1	2	ACTA1
Pubmed	8101000	Six mRNAs with different 5' ends are encoded by a single gamma-glutamyltransferase gene in mouse.	Pubmed	0.00875	129	0.0149	0.152	1	2	GGT1
Pubmed	19628582	Isoform-specific transcriptional activity of overlapping target genes that respond to thyroid hormone receptors alpha1 and beta1.	GeneRIF	0.00875	129	0.0149	0.152	1	2	THRA
Pubmed	19553558	Autoimmune type 1 diabetes genetic susceptibility encoded by human leukocyte antigen DRB1 and DQB1 genes in Tunisia.	GeneRIF	0.00875	129	0.0149	0.152	1	2	HLA-DQB1
Pubmed	21031025	Genotyping HLA-DRB1 and HLA-DQB1 alleles in Japanese patients with normal tension glaucoma.	GeneRIF	0.00875	129	0.0149	0.152	1	2	HLA-DQB1
Pubmed	21347376	Large induces functional glycans in an O-mannosylation dependent manner and targets GlcNAc terminals on alpha-dystroglycan.	GeneRIF	0.00875	129	0.0149	0.152	1	2	LARGE
Pubmed	22615382	H3K4 demethylation by Jarid1a and Jarid1b contributes to retinoblastoma-mediated gene silencing during cellular senescence.	GeneRIF	0.00875	129	0.0149	0.152	1	2	KDM5A
Pubmed	24823369	¿d T cells are required for pulmonary IL-17A expression after ozone exposure in mice: role of TNFa.	Pubmed	0.00875	129	0.0149	0.152	1	2	CPE
Pubmed	14502572	RNA inhibition of BMP-4 gene expression in postimplantation mouse embryos.	Pubmed	0.00875	129	0.0149	0.152	1	2	BMP4
Pubmed	22711460	Low-amplitude high frequency vibration down-regulates myostatin and atrogin-1 expression, two components of the atrophy pathway in muscle cells.	Pubmed	0.00875	129	0.0149	0.152	1	2	FBXO32
Pubmed	11250046	Polymorphism and distribution of HLA-DR2 alleles in Mexican populations.	GeneRIF	0.00875	129	0.0149	0.152	1	2	HLA-DQB1
Pubmed	7739634	Mouse myodystrophy (myd) mutation: refined mapping in an interval flanked by homology with distal human 4q.	Pubmed	0.00875	129	0.0149	0.152	1	2	LARGE
Pubmed	16538672	Bmp signaling is required for intestinal growth and morphogenesis.	Pubmed	0.00875	129	0.0149	0.152	1	2	BMP4
Pubmed	8873145	Superactivation of transglutaminase type 2 without change in enzyme level occurs during progressive neurodegeneration in the mnd mouse mutant.	Pubmed	0.00875	129	0.0149	0.152	1	2	TGM2
Pubmed	19390203	HLA-DR, -DQB typing of steroid-sensitive idiopathic nephrotic syndrome children in Taiwan.	GeneRIF	0.00875	129	0.0149	0.152	1	2	HLA-DQB1
Pubmed	19363121	Activated protein C protects against ventilator-induced pulmonary capillary leak.	GeneRIF	0.00875	129	0.0149	0.152	1	2	PROCR
Pubmed	20192597	Influence of matrix metalloproteinase gene polymorphisms in healthy North Indians compared to variations in other ethnic groups worldwide.	GeneRIF	0.00875	129	0.0149	0.152	1	2	MMP7
Pubmed	12360463	Tropical calcific pancreatitis: strong association with SPINK1 trypsin inhibitor mutations.	GeneRIF	0.00875	129	0.0149	0.152	1	2	SPINK1
Pubmed	19052207	Genetic variants of Nogo-66 receptor with possible association to schizophrenia block myelin inhibition of axon growth.	GeneRIF	0.00875	129	0.0149	0.152	1	2	RTN4R
Pubmed	23093672	Coordinated repression of cell cycle genes by KDM5A and E2F4 during differentiation.	GeneRIF	0.00875	129	0.0149	0.152	1	2	KDM5A
Pubmed	17681301	Human granulosa-lutein cell in vitro production of progesterone, inhibin A, inhibin B, and activin A are dependent on follicular size and not the presence of the oocyte.	GeneRIF	0.00875	129	0.0149	0.152	1	2	INHBB
Pubmed	20969748	Identification of differentially expressed genes using an annealing control primer system in stage III serous ovarian carcinoma.	Pubmed	0.00875	129	0.0149	0.152	1	2	MRVI1
Pubmed	19519176	Association of polymorphisms in estrogen and thyroid hormone receptors with thyroid cancer risk.	GeneRIF	0.00875	129	0.0149	0.152	1	2	THRA
Pubmed	19015136	Leucocyte cathepsin K affects atherosclerotic lesion composition and bone mineral density in low-density lipoprotein receptor deficient mice.	GeneRIF	0.00875	129	0.0149	0.152	1	2	CTSK
Pubmed	18295738	Meta-analysis of HLA-DRB1 and HLA-DQB1 polymorphisms in Latin American patients with systemic lupus erythematosus.	GeneRIF	0.00875	129	0.0149	0.152	1	2	HLA-DQB1
Pubmed	18818419	Bone morphogenetic proteins 2 and 4 are selectively expressed by late outgrowth endothelial progenitor cells and promote neoangiogenesis.	GeneRIF	0.00875	129	0.0149	0.152	1	2	BMP4
Pubmed	12027934	Does HLA genotype influence the relative risk of Type 1 diabetes conferred by dietary factors?	GeneRIF	0.00875	129	0.0149	0.152	1	2	HLA-DQB1
Pubmed	21366730	Endogenous proBDNF is a negative regulator of migration of cerebellar granule cells in neonatal mice.	GeneRIF	0.00875	129	0.0149	0.152	1	2	NGFR
Pubmed	16200075	Pericellular activation of proMMP-7 (promatrilysin-1) through interaction with CD151.	Pubmed	0.00875	129	0.0149	0.152	1	2	MMP7
Pubmed	18809380	Tissue transglutaminase-induced down-regulation of matrix metalloproteinase-9.	GeneRIF	0.00875	129	0.0149	0.152	1	2	TGM2

Category	ID	Name	Source	p-value	q-value Bonferroni	q-value FDR B&H	q-value FDR B&Y	Hit Count in Query List	Hit Count in Genome	Hit in Query List
Pubmed	20657015	Molecular identification of N-acetylaspartylglutamate synthase and beta-citrylglutamate synthase.	GeneRIF	0.00875	129	0.0149	0.152	1	2	RIMKLB
Pubmed	19433310	Cathepsin X cleaves the C-terminal dipeptide of alpha- and gamma-enolase and impairs survival and neuritogenesis of neuronal cells.	GeneRIF	0.00875	129	0.0149	0.152	1	2	CTSK
Pubmed	18663129	IL-17 produced by Paneth cells drives TNF-induced shock.	Pubmed	0.00875	129	0.0149	0.152	1	2	MMP7
Pubmed	19324884	Tissue transglutaminase regulates matrix metalloproteinase-2 in ovarian cancer by modulating cAMP-response element-binding protein activity.	GeneRIF	0.00875	129	0.0149	0.152	1	2	TGM2
Pubmed	16157380	Glutamic acid decarboxylase autoantibody prevalence and association with HLA genotype in patients with younger-onset type 1 diabetes and proliferative diabetic retinopathy.	GeneRIF	0.00875	129	0.0149	0.152	1	2	HLA-DQB1
Pubmed	24577056	Identifying microRNAs regulating B7-H3 in breast cancer: the clinical impact of microRNA-29c.	Pubmed	0.00875	129	0.0149	0.152	1	2	CD276
Pubmed	23326535	Profilin 1 as a target for cathepsin X activity in tumor cells.	GeneRIF	0.00875	129	0.0149	0.152	1	2	CTSK
Pubmed	18829447	Calpain5 expression is decreased in endometriosis and regulated by HOXA10 in human endometrial cells.	GeneRIF	0.00875	129	0.0149	0.152	1	2	CAPN5
Pubmed	20726222	[Effects of 2,2',4,4'-tetrabrominated diphenyl ethers on the TRalpha1 and TRbeta1 expression in liver in C57BL/6 mice].	GeneRIF	0.00875	129	0.0149	0.152	1	2	THRA
Pubmed	19375646	Extramedullary erythropoiesis in the adult liver requires BMP-4/Smad5-dependent signaling.	GeneRIF	0.00875	129	0.0149	0.152	1	2	BMP4
Pubmed	22374170	Rapid identification of the copy number of a-globin genes by capillary electrophoresis analysis.	GeneRIF	0.00875	129	0.0149	0.152	1	2	HBA2
Pubmed	15927889	Adrenodoxin (Adx) and CYP11A1 (P450scc) induce apoptosis by the generation of reactive oxygen species in mitochondria.	GeneRIF	0.00875	129	0.0149	0.152	1	2	CYP11A1
Pubmed	20937352	Interleukin-17A induces cathepsin K and MMP-9 expression in osteoclasts via celecoxib-blocked prostaglandin E2 in osteoblasts.	GeneRIF	0.00875	129	0.0149	0.152	1	2	CTSK
Pubmed	21199938	Myelination transition zone astrocytes are constitutively phagocytic and have synuclein dependent reactivity in glaucoma.	GeneRIF	0.00875	129	0.0149	0.152	1	2	GPNMB
Pubmed	7092800	The primary structure of genetic variants of mouse hemoglobin.	Pubmed	0.00875	129	0.0149	0.152	1	2	HBA2
Pubmed	10601340	Microtubule actin cross-linking factor (MACF): a hybrid of dystonin and dystrophin that can interact with the actin and microtubule cytoskeletons.	Pubmed	0.00875	129	0.0149	0.152	1	2	ACTA1
Pubmed	8910261	Glycation mediated crosslinking between alpha-crystallin and MP26 in intact lens membranes.	Pubmed	0.00875	129	0.0149	0.152	1	2	CRYAA
Pubmed	24193434	Transglutaminase-2 in cell adhesion: all roads lead to paxillin?	Pubmed	0.00875	129	0.0149	0.152	1	2	TGM2
Pubmed	17099210	Variations in the thrombomodulin and endothelial protein C receptor genes in couples with recurrent miscarriage.	GeneRIF	0.00875	129	0.0149	0.152	1	2	PROCR
Pubmed	3075543	Transcripts of alpha-cardiac and alpha-skeletal actins are early markers for myogenesis in the mouse embryo.	Pubmed	0.00875	129	0.0149	0.152	1	2	ACTA1
Pubmed	19763374	Marrow stromal cell-based cyclooxygenase 2 ex vivo gene-transfer strategy surprisingly lacks bone-regeneration effects and suppresses the bone-regeneration action of bone morphogenetic protein 4 in a mouse critical-sized calvarial defect model.	GeneRIF	0.00875	129	0.0149	0.152	1	2	BMP4
Pubmed	9743527	Antagonist peptide selects thymocytes expressing a class II major histocompatibility complex-restricted T cell receptor into the CD8 lineage.	Pubmed	0.00875	129	0.0149	0.152	1	2	CD74
Pubmed	17965585	Expression and function of transmembrane-4 superfamily (tetraspanin) proteins in osteoclasts: reciprocal roles of Tspan-5 and NET-6 during osteoclastogenesis.	GeneRIF	0.00875	129	0.0149	0.152	1	2	TSPAN13
Pubmed	15203870	HLA class II polymorphism in Egyptian children with lymphomas.	GeneRIF	0.00875	129	0.0149	0.152	1	2	HLA-DQB1
Pubmed	12183536	Streptococcal inhibitor of complement inhibits two additional components of the mucosal innate immune system: secretory leukocyte proteinase inhibitor and lysozyme.	GeneRIF	0.00875	129	0.0149	0.152	1	2	SLPI
Pubmed	18362148	Inhibitory fragment from the p41 form of invariant chain can regulate activity of cysteine cathepsins in antigen presentation.	Pubmed	0.00875	129	0.0149	0.152	1	2	CD74
Pubmed	23555776	The clinical significance of the CD163+ and CD68+ macrophages in patients with hepatocellular carcinoma.	Pubmed	0.00875	129	0.0149	0.152	1	2	CD68
Pubmed	25079347	CBFß and the leukemogenic fusion protein CBFß-SMMHC associate with mitotic chromosomes to epigenetically regulate ribosomal genes.	Pubmed	0.00875	129	0.0149	0.152	1	2	MYH11
Pubmed	21955839	Weak association of anti-sperm antibodies and strong association of familial cryptorchidism/infertility with HLA-DRB1 polymorphisms in prepubertal Ukrainian boys.	GeneRIF	0.00875	129	0.0149	0.152	1	2	HLA-DQB1
Pubmed	2995251	Linkage of the locus encoding the A chain of alpha crystallin (Acry-1) to the major histocompatibility complex in the rat.	Pubmed	0.00875	129	0.0149	0.152	1	2	CRYAA
Pubmed	19717439	Unexpected expression of alpha- and beta-globin in mesencephalic dopaminergic neurons and glial cells.	Pubmed	0.00875	129	0.0149	0.152	1	2	HBA2
Pubmed	19091945	Four-dimensional realistic modeling of pancreatic organogenesis.	GeneRIF	0.00875	129	0.0149	0.152	1	2	BMP4
Pubmed	19587020	Distinct requirements for CD1d intracellular transport for development of V(alpha)14 iNKT cells.	GeneRIF	0.00875	129	0.0149	0.152	1	2	CD74
Pubmed	24700421	Implant wear induced inflammation is mitigated in CX3CR1(-/-) mice.	Pubmed	0.00875	129	0.0149	0.152	1	2	CX3CL1
Pubmed	25337265	Human leukocyte antigen-DRB1*1501 and DQB1*0602 alleles are cervical cancer protective factors among Uighur and Han people in Xinjiang, China.	Pubmed	0.00875	129	0.0149	0.152	1	2	HLA-DQB1

Category	ID	Name	Source	p-value	q-value Bonferroni	q-value FDR B&H	q-value FDR B&Y	Hit Count in Query List	Hit Count in Genome	Hit in Query List
Pubmed	18708743	Kv11.1 (ERG1) K+ channels localize in cholesterol and sphingolipid enriched membranes and are modulated by membrane cholesterol.	GeneRIF	0.00875	129	0.0149	0.152	1	2	KCNH2
Pubmed	11106625	Alteration of tropomyosin function and folding by a nemaline myopathy-causing mutation.	Pubmed	0.00875	129	0.0149	0.152	1	2	ACTA1
Pubmed	16437632	Human leukocyte antigen class II DQB1*0301, DRB1*1101 alleles and spontaneous clearance of hepatitis C virus infection: a meta-analysis.	GeneRIF	0.00875	129	0.0149	0.152	1	2	HLA-DQB1
Pubmed	11532985	The sarcolemmal proteins dysferlin and caveolin-3 interact in skeletal muscle.	Pubmed	0.00875	129	0.0149	0.152	1	2	DYSF
Pubmed	16026545	Gene and protein expression of transforming growth factor beta 2 gene during murine primary palatogenesis.	GeneRIF	0.00875	129	0.0149	0.152	1	2	BMP4
Pubmed	17379640	Regulation of membrane potential and fluid secretion by Ca2+-activated K+ channels in mouse submandibular glands.	GeneRIF	0.00875	129	0.0149	0.152	1	2	KCNN4
Pubmed	11687536	Characterization of a new, dominant V124E mutation in the mouse alphaA-crystallin-encoding gene.	Pubmed	0.00875	129	0.0149	0.152	1	2	CRYAA
Pubmed	15632314	Gene-expression profiling reveals down-regulation of equilibrative nucleoside transporter 1 (ENT1) in Ara-C-resistant CCRF-CEM-derived cells.	GeneRIF	0.00875	129	0.0149	0.152	1	2	ADA
Pubmed	11739587	Retardation of cochlear maturation and impaired hair cell function caused by deletion of all known thyroid hormone receptors.	Pubmed	0.00875	129	0.0149	0.152	1	2	THRA
Pubmed	19896562	HLA class II polymorphism in Latin American patients with multiple sclerosis.	GeneRIF	0.00875	129	0.0149	0.152	1	2	HLA-DQB1
Pubmed	22259222	Chlamydia infection status, genotype, and age-related macular degeneration.	GeneRIF	0.00875	129	0.0149	0.152	1	2	HTRA1
Pubmed	18680227	Association between calcium sensing receptor gene polymorphisms and chronic pancreatitis in a US population: role of serine protease inhibitor Kazal 1type and alcohol.	GeneRIF	0.00875	129	0.0149	0.152	1	2	SPINK1
Pubmed	23857671	The CX3CL1/CX3CR1 reprograms glucose metabolism through HIF-1 pathway in pancreatic adenocarcinoma.	Pubmed	0.00875	129	0.0149	0.152	1	2	CX3CL1
Pubmed	1975428	A truncated human chromosome 16 associated with alpha thalassaemia is stabilized by addition of telomeric repeat (TTAGGG)n.	Pubmed	0.00875	129	0.0149	0.152	1	2	HBA2
Pubmed	17134718	Actin filament severing by cofilin.	GeneRIF	0.00875	129	0.0149	0.152	1	2	ACTA1
Pubmed	15084587	The role of p22 NF-E4 in human globin gene switching.	GeneRIF	0.00875	129	0.0149	0.152	1	2	HBB
Pubmed	22194938	NMR studies of the C-terminus of alpha4 reveal possible mechanism of its interaction with MID1 and protein phosphatase 2A.	GeneRIF	0.00875	129	0.0149	0.152	1	2	MID1
Pubmed	17890443	Influence of ScaI and natriuretic peptide (NP) clearance receptor polymorphisms of the NP System on NP concentration in chronic heart failure.	GeneRIF	0.00875	129	0.0149	0.152	1	2	NPPA
Pubmed	21329684	Human aldo-keto reductases 1B1 and 1B10: a comparative study on their enzyme activity toward electrophilic carbonyl compounds.	GeneRIF	0.00875	129	0.0149	0.152	1	2	AKR1B10
Pubmed	21388954	Syk regulates multiple signaling pathways leading to CX3CL1 chemotaxis in macrophages.	GeneRIF	0.00875	129	0.0149	0.152	1	2	CX3CL1
Pubmed	23233664	Tolerance induction with gene-modified stem cells and immune-preserving conditioning in primed mice: restricting antigen to differentiated antigen-presenting cells permits efficacy.	Pubmed	0.00875	129	0.0149	0.152	1	2	HLA-DQB1
Pubmed	24357671	Syndecan-4 knockout leads to reduced extracellular transglutaminase-2 and protects against tubulointerstitial fibrosis.	Pubmed	0.00875	129	0.0149	0.152	1	2	TGM2
Pubmed	22268124	Allele-specific transactivation of matrix metalloproteinase 7 by FOXA2 and correlation with plasma levels in idiopathic pulmonary fibrosis.	GeneRIF	0.00875	129	0.0149	0.152	1	2	MMP7
Pubmed	20659559	LINGO-1-mediated inhibition of oligodendrocyte differentiation does not require the leucine-rich repeats and is reversed by p75(NTR) antagonists.	GeneRIF	0.00875	129	0.0149	0.152	1	2	NGFR
Pubmed	25417227	Congenital long QT syndrome: a case report of LQT2 and LQT13 in a neonate.	Pubmed	0.00875	129	0.0149	0.152	1	2	KCNH2
Pubmed	11939522	Hb Ube-2 in a Taiwanese subject: an A-->G substitution at codon 68 of the alpha2-globin gene.	Pubmed	0.00875	129	0.0149	0.152	1	2	HBA2
Pubmed	10518533	Interactions of G(h)/transglutaminase with phospholipase Cdelta1 and with GTP.	Pubmed	0.00875	129	0.0149	0.152	1	2	TGM2
Pubmed	15044690	Identification of genes that synergize with Cbfb-MYH11 in the pathogenesis of acute myeloid leukemia.	GeneRIF	0.00875	129	0.0149	0.152	1	2	MYH11
Pubmed	18768081	The genetics of feto-placental development: a study of acid phosphatase locus 1 and adenosine deaminase polymorphisms in a consecutive series of newborn infants.	GeneRIF	0.00875	129	0.0149	0.152	1	2	ADA
Pubmed	20522203	Age- and gender-specific association between ADA (22G>A) and TNF-a (-308G>A) genetic polymorphisms.	GeneRIF	0.00875	129	0.0149	0.152	1	2	ADA
Pubmed	22324317	A novel a(0) -thalassemia deletion in a Greek patient with HbH disease and ß-thalassemia trait.	GeneRIF	0.00875	129	0.0149	0.152	1	2	HBB
Pubmed	14623754	HLA-DQ alleles in white and African American patients with juvenile-onset recurrent respiratory papillomatosis.	GeneRIF	0.00875	129	0.0149	0.152	1	2	HLA-DQB1
Pubmed	20595382	Reduced plasma membrane expression of dysferlin mutants is attributed to accelerated endocytosis via a syntaxin-4-associated pathway.	GeneRIF	0.00875	129	0.0149	0.152	1	2	DYSF
Pubmed	18343237	Cataract mutation P20S of alphaB-crystallin impairs chaperone activity of alphaA-crystallin and induces apoptosis of human lens epithelial cells.	GeneRIF	0.00875	129	0.0149	0.152	1	2	CRYAA
Pubmed	18523306	Antigen presentation by human microvascular endothelial cells triggers ICAM-1-dependent transendothelial protrusion by, and fractalkine-dependent transendothelial migration of, effector memory CD4+ T cells.	GeneRIF	0.00875	129	0.0149	0.152	1	2	CX3CL1

Category	ID	Name	Source	p-value	q-value Bonferroni	q-value FDR B&H	q-value FDR B&Y	Hit Count in Query List	Hit Count in Genome	Hit in Query List
Pubmed	21768337	Large-scale opening of utrophin's tandem calponin homology (CH) domains upon actin binding by an induced-fit mechanism.	GeneRIF	0.00875	129	0.0149	0.152	1	2	ACTA1
Pubmed	1521591	Genetic variability and rodent models of human aging.	Pubmed	0.00875	129	0.0149	0.152	1	2	BMP4
Pubmed	11739519	CD4(+) T cells are required for the development of cytotoxic CD8(+) T cells during Mycobacterium tuberculosis infection.	Pubmed	0.00875	129	0.0149	0.152	1	2	HLA-DQB1
Pubmed	22002247	Identification of P-Rex1 as a novel Rac1-guanine nucleotide exchange factor (GEF) that promotes actin remodeling and GLUT4 protein trafficking in adipocytes.	GeneRIF	0.00875	129	0.0149	0.152	1	2	PREX1
Pubmed	18854880	[Viral genotype and HLA class II alleles influence on extra-hepatic manifestations of chronic HCV infection].	GeneRIF	0.00875	129	0.0149	0.152	1	2	HLA-DQB1
Pubmed	16504290	Granulocytic sarcoma of mesentery in acute myeloid leukemia with CBFB/MYH11 fusion gene but not inv(16) chromosome: case report and review of literature.	GeneRIF	0.00875	129	0.0149	0.152	1	2	MYH11
Pubmed	15634335	Selective modulation of protein C affinity for EPCR and phospholipids by Gla domain mutation.	GeneRIF	0.00875	129	0.0149	0.152	1	2	PROCR
Pubmed	24777581	Lipidomic analyses of female mice lacking hepatic lipase and endothelial lipase indicate selective modulation of plasma lipid species.	Pubmed	0.00875	129	0.0149	0.152	1	2	LIPG
Pubmed	16877778	Endothelial lipase is less effective at influencing HDL metabolism in vivo in mice expressing apoA-II.	GeneRIF	0.00875	129	0.0149	0.152	1	2	LIPG
Pubmed	18988890	Endothelial lipase promotes apolipoprotein AI-mediated cholesterol efflux in THP-1 macrophages.	GeneRIF	0.00875	129	0.0149	0.152	1	2	LIPG
Pubmed	12761217	The forkhead transcription factor FOXO4 induces the down-regulation of hypoxia-inducible factor 1 alpha by a von Hippel-Lindau protein-independent mechanism.	GeneRIF	0.00875	129	0.0149	0.152	1	2	FOXO4
Pubmed	17314516	GPR56 and TG2: possible roles in suppression of tumor growth by the microenvironment.	GeneRIF	0.00875	129	0.0149	0.152	1	2	TGM2
Pubmed	25737953	Genetic determination of the cellular basis of the ghrelin-dependent bone remodeling.	Pubmed	0.00875	129	0.0149	0.152	1	2	CTSK
Pubmed	10906735	Myosin heavy chain gene expression in normal and hyperplastic human prostate tissue.	Pubmed	0.00875	129	0.0149	0.152	1	2	MYH11
Pubmed	10023782	A novel isoform of the smooth muscle cell differentiation marker smoothelin.	Pubmed	0.00875	129	0.0149	0.152	1	2	SMTN
Pubmed	24492129	Rare variants in BMP2 and BMP4 found in otosclerosis patients reduce Smad signaling.	Pubmed	0.00875	129	0.0149	0.152	1	2	BMP4
Pubmed	18270511	The ARID domain of the H3K4 demethylase RBP2 binds to a DNA CCGCCC motif.	GeneRIF	0.00875	129	0.0149	0.152	1	2	KDM5A
Pubmed	9248858	Immunohistochemical localization of fibroblast growth factor-2 in normal and brachymorphic mouse tibial growth plate and articular cartilage.	Pubmed	0.00875	129	0.0149	0.152	1	2	PAPSS2
Pubmed	12037567	Nogo-66 receptor antagonist peptide promotes axonal regeneration.	Pubmed	0.00875	129	0.0149	0.152	1	2	RTN4R
Pubmed	19244534	Deafness in TRbeta mutants is caused by malformation of the tectorial membrane.	Pubmed	0.00875	129	0.0149	0.152	1	2	THRA
Pubmed	19609526	HbA2-Partinico or delta(A2)Pro-->Thr, a new genetic variation in the delta-globin gene in cis to the beta(+) thal IVS-I-110 G>A, and the heterogeneity of delta-globin alleles in double heterozygotes for beta- and delta-globin gene defects.	GeneRIF	0.00875	129	0.0149	0.152	1	2	HBB
Pubmed	9129143	T-cell oncogene rhombotin-2 interacts with retinoblastoma-binding protein 2.	Pubmed	0.00875	129	0.0149	0.152	1	2	KDM5A
Pubmed	24955833	Genetic deletion of transglutaminase 2 does not rescue the phenotypic deficits observed in R6/2 and zQ175 mouse models of Huntington's disease.	Pubmed	0.00875	129	0.0149	0.152	1	2	TGM2
Pubmed	22389502	Hypertrophy in skeletal myotubes induced by junctophilin-2 mutant, Y141H, involves an increase in store-operated Ca2+ entry via Orai1.	GeneRIF	0.00875	129	0.0149	0.152	1	2	JPH2
Pubmed	7805878	Molecular cloning of human cathepsin O, a novel endoproteinase and homologue of rabbit OC2.	Pubmed	0.00875	129	0.0149	0.152	1	2	CTSK
Pubmed	16087715	Characterization of the early stages of thymic NKT cell development.	Pubmed	0.00875	129	0.0149	0.152	1	2	HLA-DQB1
Pubmed	19089815	Lipid-mediated presentation of MHC class II molecules guides thymocytes to the CD4 lineage.	Pubmed	0.00875	129	0.0149	0.152	1	2	HLA-DQB1
Pubmed	23718892	Differential effects of the ß-adrenoceptor blockers carvedilol and metoprolol on SQT1- and SQT2-mutant channels.	Pubmed	0.00875	129	0.0149	0.152	1	2	KCNH2
Pubmed	19005023	Influence of common and specific HLA-DRB1/DQB1 haplotypes on genetic susceptibilities of three distinct Arab populations to type 1 diabetes.	GeneRIF	0.00875	129	0.0149	0.152	1	2	HLA-DQB1
Pubmed	14617034	Two subtypes of hepatitis B virus-associated glomerulonephritis are associated with different HLA-DR2 alleles in Koreans.	GeneRIF	0.00875	129	0.0149	0.152	1	2	HLA-DQB1
Pubmed	17327419	Thyroid hormone excess rather than thyrotropin deficiency induces osteoporosis in hyperthyroidism.	GeneRIF	0.00875	129	0.0149	0.152	1	2	THRA
Pubmed	20179953	Congenital myotonic dystrophy can show congenital fiber type disproportion pathology.	GeneRIF	0.00875	129	0.0149	0.152	1	2	ACTA1
Pubmed	10217147	Direct control of the Forkhead transcription factor AFX by protein kinase B.	Pubmed	0.00875	129	0.0149	0.152	1	2	FOXO4
Pubmed	16180280	Association of extrahepatic manifestations with HLA class II alleles and with virus genotype in HCV infected patients.	GeneRIF	0.00875	129	0.0149	0.152	1	2	HLA-DQB1
Pubmed	16542506	Decrease in expression of bone morphogenetic proteins 4 and 5 in synovial tissue of patients with osteoarthritis and rheumatoid arthritis.	GeneRIF	0.00875	129	0.0149	0.152	1	2	BMP4
Pubmed	21340023	Rapid cytoskeletal response of epithelial cells to force generation by type IV pili.	GeneRIF	0.00875	129	0.0149	0.152	1	2	ACTA1
Pubmed	18259107	Reevaluation of human leukocyte antigen DR-DQ haplotype and genotype in type 1 diabetes in the Japanese population.	GeneRIF	0.00875	129	0.0149	0.152	1	2	HLA-DQB1
Pubmed	22998035	Secretory sorting receptors carboxypeptidase E and secretogranin III in amyloid ß-associated neural degeneration in Alzheimer's disease.	Pubmed	0.00875	129	0.0149	0.152	1	2	CPE

Category	ID	Name	Source	p-value	q-value Bonferroni	q-value FDR B&H	q-value FDR B&Y	Hit Count in Query List	Hit Count in Genome	Hit in Query List
Pubmed	20452593	Association between plasma PCSK9 and gamma-glutamyl transferase levels in diabetic patients.	GeneRIF	0.00875	129	0.0149	0.152	1	2	GGT1
Pubmed	3165083	Genes on chromosomes 1 and 4 in the mouse are associated with repair of radiation-induced chromatin damage.	Pubmed	0.00875	129	0.0149	0.152	1	2	NPPA
Pubmed	25197808	HLA DQB1*06:02 negative narcolepsy with hypocretin/orexin deficiency.	Pubmed	0.00875	129	0.0149	0.152	1	2	HLA-DQB1
Pubmed	12145333	Requirement for thyroid hormone receptor beta in T3 regulation of cholesterol metabolism in mice.	GeneRIF	0.00875	129	0.0149	0.152	1	2	THRA
Pubmed	16878175	HLA-DQ2 and -DQ8 signatures of gluten T cell epitopes in celiac disease.	GeneRIF	0.00875	129	0.0149	0.152	1	2	HLA-DQB1
Pubmed	19158290	Axonal protective effects of the myelin-associated glycoprotein.	GeneRIF	0.00875	129	0.0149	0.152	1	2	RTN4R
Pubmed	16319126	Aberrant dysferlin trafficking in cells lacking caveolin or expressing dystrophy mutants of caveolin-3.	GeneRIF	0.00875	129	0.0149	0.152	1	2	DYSF
Pubmed	20733203	Homeostatic turnover of virus-specific memory CD8 T cells occurs stochastically and is independent of CD4 T cell help.	Pubmed	0.00875	129	0.0149	0.152	1	2	HLA-DQB1
Pubmed	18941249	Up-regulation of vascular endothelial growth factor-D expression in clear cell renal cell carcinoma by CD74: a critical role in cancer cell tumorigenesis.	GeneRIF	0.00875	129	0.0149	0.152	1	2	CD74
Pubmed	11024455	Self-complementary motifs (SCM) in alpha-crystallin small heat shock proteins.	Pubmed	0.00875	129	0.0149	0.152	1	2	CRYAA
Pubmed	1730617	Vertebrate lens alpha-crystallins are modified by O linked N-acetylglucosamine.	Pubmed	0.00875	129	0.0149	0.152	1	2	CRYAA
Pubmed	12788886	A combination of human leukocyte antigen DQB1*02 and the tumor necrosis factor alpha promoter G308A polymorphism predisposes to an insulin-deficient phenotype in patients with type 2 diabetes.	GeneRIF	0.00875	129	0.0149	0.152	1	2	HLA-DQB1
Pubmed	20403137	Polymorphic major histocompatibility complex class II Alu insertions at five loci and their association with HLA-DRB1 and -DQB1 in Japanese and Caucasians.	GeneRIF	0.00875	129	0.0149	0.152	1	2	HLA-DQB1
Pubmed	20627047	[Sequence analysis of GJB3 in Chinese deafness population who carry one heterozygous GJB2 pathogenic mutation].	GeneRIF	0.00875	129	0.0149	0.152	1	2	GJB3
Pubmed	16049696	Differential regulation of AQP2 trafficking in endosomes by microtubules and actin filaments.	GeneRIF	0.00875	129	0.0149	0.152	1	2	RAB11FIP1
Pubmed	10964919	Mutational analysis of affinity and selectivity of kringle-tetranectin interaction. Grafting novel kringle affinity ontp the trtranectin lectin scaffold.	Pubmed	0.00875	129	0.0149	0.152	1	2	CLEC3B
Pubmed	17076705	Characterization of the protective T-cell response generated in CD4-deficient mice by a live attenuated Mycobacterium tuberculosis vaccine.	Pubmed	0.00875	129	0.0149	0.152	1	2	HLA-DQB1
Pubmed	16990558	Critical role of inducible costimulator signaling in the development of arteriosclerosis.	GeneRIF	0.00875	129	0.0149	0.152	1	2	HLA-DQB1
Pubmed	9726990	Identification of CX3CR1. A chemotactic receptor for the human CX3C chemokine fractalkine and a fusion coreceptor for HIV-1.	Pubmed	0.00875	129	0.0149	0.152	1	2	CX3CL1
Pubmed	24755685	The diagnostic importance of matrix metalloproteinase-7 and nestin in gastrointestinal stromal tumors.	Pubmed	0.00875	129	0.0149	0.152	1	2	MMP7
Pubmed	17389012	Low frequency of the disease-associated DRB1*15 DQB1*06 haplotype may contribute to the low prevalence of multiple sclerosis in Sami.	GeneRIF	0.00875	129	0.0149	0.152	1	2	HLA-DQB1
Pubmed	18713751	Alternative splicing of the guanylyl cyclase-A receptor modulates atrial natriuretic peptide signaling.	GeneRIF	0.00875	129	0.0149	0.152	1	2	NPPA
Pubmed	19027490	Some but not all of HLA-II alleles are associated with cervical cancer in Chinese women.	GeneRIF	0.00875	129	0.0149	0.152	1	2	HLA-DQB1
Pubmed	19625333	Multiple sclerosis risk: interaction between human leukocyte antigen and the environment in Sardinian population.	GeneRIF	0.00875	129	0.0149	0.152	1	2	HLA-DQB1
Pubmed	10862973	Reactive-site specificity of human kallistatin toward tissue kallikrein probed by site-directed mutagenesis.	Pubmed	0.00875	129	0.0149	0.152	1	2	KLK1
Pubmed	17728092	Circulating cathepsin K and cystatin C in patients with cancer related bone disease: clinical and therapeutic implications.	GeneRIF	0.00875	129	0.0149	0.152	1	2	CTSK
Pubmed	7559764	Regulation of nerve growth factor receptor gene expression in sympathetic neurons during development.	Pubmed	0.00875	129	0.0149	0.152	1	2	NGFR
Pubmed	12472658	Psoriasis is associated with a SNP haplotype of the corneodesmosin gene (CDSN).	GeneRIF	0.00875	129	0.0149	0.152	1	2	CDSN
Pubmed	10583954	Domain movement in gelsolin: a calcium-activated switch.	Pubmed	0.00875	129	0.0149	0.152	1	2	ACTA1
Pubmed	16858401	Lineage-specific activators affect beta-globin locus chromatin in multipotent hematopoietic progenitors.	GeneRIF	0.00875	129	0.0149	0.152	1	2	HBB
Pubmed	23452376	The PKD domain distinguishes the trafficking and amyloidogenic properties of the pigment cell protein PMEL and its homologue GPNMB.	Pubmed	0.00875	129	0.0149	0.152	1	2	GPNMB
Pubmed	19834056	Glycosaminoglycan-mediated loss of cathepsin K collagenolytic activity in MPS I contributes to osteoclast and growth plate abnormalities.	GeneRIF	0.00875	129	0.0149	0.152	1	2	CTSK
Pubmed	24147040	Prostaglandin E2 induction during mouse adenovirus type 1 respiratory infection regulates inflammatory mediator generation but does not affect viral pathogenesis.	Pubmed	0.00875	129	0.0149	0.152	1	2	HLA-DQB1
Pubmed	23682772	Human GGT2 Does Not Autocleave into a Functional Enzyme: A Cautionary Tale for Interpretation of Microarray Data on Redox Signaling.	Pubmed	0.00875	129	0.0149	0.152	1	2	GGT1
Pubmed	5340470	Hemoglobins of mice: sequence and possible ambiguity at one position of the alpha chain.	Pubmed	0.00875	129	0.0149	0.152	1	2	HBA2
Pubmed	23040355	ENU mutagenesis identifies the first mouse mutants reproducing human ß-thalassemia at the genomic level.	GeneRIF	0.00875	129	0.0149	0.152	1	2	HBB
Pubmed	15637153	Hypertension in mice lacking the proatrial natriuretic peptide convertase corin.	GeneRIF	0.00875	129	0.0149	0.152	1	2	NPPA

Category	ID	Name	Source	p-value	q-value Bonferroni	q-value FDR B&H	q-value FDR B&Y	Hit Count in Query List	Hit Count in Genome	Hit in Query List
Pubmed	15174785	Screening of coeliac disease in north Italian children with type 1 diabetes: limited usefulness of HLA-DQ typing.	GeneRIF	0.00875	129	0.0149	0.152	1	2	HLA-DQB1
Pubmed	21395562	Two novel HLA class II alleles, DRB1*11:96 and DQB1*02:01:03, identified in Koreans.	GeneRIF	0.00875	129	0.0149	0.152	1	2	HLA-DQB1
Pubmed	15277941	Mice with a melanocortin 1 receptor mutation have a slightly greater minimum alveolar concentration than control mice.	GeneRIF	0.00875	129	0.0149	0.152	1	2	HLA-DQB1
Pubmed	23642664	The epidermal Integrin beta-1 and p75NTR positive cells proliferating and migrating during wound healing produce various growth factors, while the expression of p75NTR is decreased in patients with chronic skin ulcers.	Pubmed	0.00875	129	0.0149	0.152	1	2	NGFR
Pubmed	24424083	Dissociation and re-association studies on the interaction domains of mannan-binding lectin (MBL)-associated serine proteases, MASP-1 and MASP-2, provide evidence for heterodimer formation.	Pubmed	0.00875	129	0.0149	0.152	1	2	MASP1
Pubmed	18683889	Endogenous bone morphogenetic proteins mediate 1alpha, 25-dihydroxyvitamin D(3)-induced expression of osteoblast differentiation markers in human dermal fibroblasts.	GeneRIF	0.00875	129	0.0149	0.152	1	2	BMP4
Pubmed	11045836	Human leukocyte antigen class II alleles in white Brazilian patients with celiac disease.	GeneRIF	0.00875	129	0.0149	0.152	1	2	HLA-DQB1
Pubmed	17675479	OX40 costimulation promotes persistence of cytomegalovirus-specific CD8 T Cells: A CD4-dependent mechanism.	Pubmed	0.00875	129	0.0149	0.152	1	2	HLA-DQB1
Pubmed	17693975	A comparative study of plasma renin activity, aldosterone and atrial natriuretic hormone between normotensive and hypertensive people living at below sea level altitude (Jordan Valley) and those residing at moderate altitude above.	GeneRIF	0.00875	129	0.0149	0.152	1	2	NPPA
Pubmed	22107967	The use of Bcl-2 over-expression to stabilize hybridomas specific to the HERG potassium channel.	GeneRIF	0.00875	129	0.0149	0.152	1	2	KCNH2
Pubmed	19213939	Signaling hierarchy regulating human endothelial cell development.	GeneRIF	0.00875	129	0.0149	0.152	1	2	BMP4
Pubmed	21393506	Human CD271-positive melanoma stem cells associated with metastasis establish tumor heterogeneity and long-term growth.	GeneRIF	0.00875	129	0.0149	0.152	1	2	NGFR
Pubmed	2606724	Hb Evans or alpha 262(E11)Val----Met beta 2; an unstable hemoglobin causing a mild hemolytic anemia.	Pubmed	0.00875	129	0.0149	0.152	1	2	HBA2
Pubmed	11866271	Mutational screening of patients with nonalcoholic chronic pancreatitis: identification of further trypsinogen variants.	GeneRIF	0.00875	129	0.0149	0.152	1	2	SPINK1
Pubmed	12646641	DM loss in k haplotype mice reveals isotype-specific chaperone requirements.	Pubmed	0.00875	129	0.0149	0.152	1	2	CD74
Pubmed	11090452	Actin-binding properties and colocalization with actin during spermiogenesis of mammalian sperm calicin.	Pubmed	0.00875	129	0.0149	0.152	1	2	ACTA1
Pubmed	20188090	In vivo lens deficiency of the R49C alphaA-crystallin mutant.	Pubmed	0.00875	129	0.0149	0.152	1	2	CRYAA
Pubmed	18200971	[Association between HLA-DQA1, HLA-DQB1 alleles and risk of early pregnancy loss].	GeneRIF	0.00875	129	0.0149	0.152	1	2	HLA-DQB1
Pubmed	24349085	Transglutaminase 2 contributes to apoptosis induction in Jurkat T cells by modulating Ca2+ homeostasis via cross-linking RAP1GDS1.	Pubmed	0.00875	129	0.0149	0.152	1	2	TGM2
Pubmed	17256150	Analysis of extended HLA haplotypes in multiple sclerosis and narcolepsy families confirms a predisposing effect for the class I region in Tasmanian MS patients.	GeneRIF	0.00875	129	0.0149	0.152	1	2	HLA-DQB1
Pubmed	10987280	Matrilysin-2, a new matrix metalloproteinase expressed in human tumors and showing the minimal domain organization required for secretion, latency, and activity.	Pubmed	0.00875	129	0.0149	0.152	1	2	MMP7
Pubmed	22999927	Altered atrial neurotransmitter release in transgenic p75(-/-) and gp130 KO mice.	Pubmed	0.00875	129	0.0149	0.152	1	2	NGFR
Pubmed	17394396	Molecular analysis of alpha/beta-thalassemia in a southern Chinese population.	GeneRIF	0.00875	129	0.0149	0.152	1	2	HBB
Pubmed	11923913	Genetic dissection of the human leukocyte antigen region by use of haplotypes of Tasmanians with multiple sclerosis.	GeneRIF	0.00875	129	0.0149	0.152	1	2	HLA-DQB1
Pubmed	12721936	Reduced frequency of HLA DRB1*03-DQB1*02 in children with type 1 diabetes associated with enterovirus RNA.	GeneRIF	0.00875	129	0.0149	0.152	1	2	HLA-DQB1
Pubmed	23243277	A novel role of BMP4 in adult hematopoietic stem and progenitor cell homing via Smad independent regulation of integrin-a4 expression.	GeneRIF	0.00875	129	0.0149	0.152	1	2	BMP4
Pubmed	19091121	Desmoglein 2 is a substrate of kallikrein 7 in pancreatic cancer.	GeneRIF	0.00875	129	0.0149	0.152	1	2	KLK7
Pubmed	15462607	Human genetics and responses to influenza vaccination: clinical implications.	GeneRIF	0.00875	129	0.0149	0.152	1	2	HLA-DQB1
Pubmed	23685348	Hemoglobin is present as a canonical a2ß2 tetramer in dopaminergic neurons.	Pubmed	0.00875	129	0.0149	0.152	1	2	HBA2
Pubmed	8463322	Covalently immobilized laminin peptide Tyr-Ile-Gly-Ser-Arg (YIGSR) supports cell spreading and co-localization of the 67-kilodalton laminin receptor with alpha-actinin and vinculin.	Pubmed	0.00875	129	0.0149	0.152	1	2	ACTA1
Pubmed	19814781	Retinoic acid enhances skeletal muscle progenitor formation and bypasses inhibition by bone morphogenetic protein 4 but not dominant negative beta-catenin.	GeneRIF	0.00875	129	0.0149	0.152	1	2	BMP4
Pubmed	18313864	Microphthalmia transcription factor regulates the expression of the novel osteoclast factor GPNMB.	GeneRIF	0.00875	129	0.0149	0.152	1	2	GPNMB
Pubmed	21840312	Src-stimulated IRTKS phosphorylation enhances cell migration.	GeneRIF	0.00875	129	0.0149	0.152	1	2	BAIAP2L1
Pubmed	17947706	Nerve growth factor promotes TLR4 signaling-induced maturation of human dendritic cells in vitro through inducible p75NTR 1.	GeneRIF	0.00875	129	0.0149	0.152	1	2	NGFR
Pubmed	21645550	Missense mutation of the reticulon-4 receptor alters spatial memory and social interaction in mice.	GeneRIF	0.00875	129	0.0149	0.152	1	2	RTN4R
Pubmed	15726664	Fractalkine and CX3CR1 are involved in the recruitment of intraepithelial lymphocytes of intrahepatic bile ducts.	GeneRIF	0.00875	129	0.0149	0.152	1	2	CX3CL1

Category	ID	Name	Source	p-value	q-value Bonferroni	q-value FDR B&H	q-value FDR B&Y	Hit Count in Query List	Hit Count in Genome	Hit in Query List
Pubmed	22109529	P-Rex1 is required for efficient melanoblast migration and melanoma metastasis.	GeneRIF	0.00875	129	0.0149	0.152	1	2	PREX1
Pubmed	17325942	Generic human leukocyte antigen class II (DRB1 and DQB1) alleles in patients with paracoccidioidomycosis.	GeneRIF	0.00875	129	0.0149	0.152	1	2	HLA-DQB1
Pubmed	12555809	High-mobility group protein 2 may be involved in the locus control region regulation of the beta-globin gene cluster.	GeneRIF	0.00875	129	0.0149	0.152	1	2	HBB
Pubmed	21318258	Respiratory regulation in narcolepsy.	GeneRIF	0.00875	129	0.0149	0.152	1	2	HLA-DQB1
Pubmed	15194331	Development of donor-specific microchimerism in liver transplant recipient with HLA-DRB1 and -DQB1 mismatch related to rejection episodes.	GeneRIF	0.00875	129	0.0149	0.152	1	2	HLA-DQB1
Pubmed	24477565	Downregulation of a putative tumor suppressor BMP4 by SOX2 promotes growth of lung squamous cell carcinoma.	Pubmed	0.00875	129	0.0149	0.152	1	2	BMP4
Pubmed	25320221	Role of fractalkine in the pathogenesis of primary Sjögren syndrome: increased serum levels of fractalkine, its expression in labial salivary glands, and the association with clinical manifestations.	Pubmed	0.00875	129	0.0149	0.152	1	2	CX3CL1
Pubmed	9864144	Murine T lymphocytes incapable of producing macrophage inhibitory protein-1 are impaired in causing graft-versus-host disease across a class I but not class II major histocompatibility complex barrier.	Pubmed	0.00875	129	0.0149	0.152	1	2	HLA-DQB1
Pubmed	9247966	Cell surface adenosine deaminase: much more than an ectoenzyme.	Pubmed	0.00875	129	0.0149	0.152	1	2	ADA
Pubmed	14583444	Molecular interaction of connexin 30.3 and connexin 31 suggests a dominant-negative mechanism associated with erythrokeratodermia variabilis.	GeneRIF	0.00875	129	0.0149	0.152	1	2	GJB3
Pubmed	21292823	Thyroid hormone receptor a and regulation of type 3 deiodinase.	GeneRIF	0.00875	129	0.0149	0.152	1	2	THRA
Pubmed	21535077	HLA class II alleles and chronic hepatitis C virus infection.	GeneRIF	0.00875	129	0.0149	0.152	1	2	HLA-DQB1
Pubmed	17446259	The connexin31 F137L mutant mouse as a model for the human skin disease erythrokeratodermia variabilis (EKV).	GeneRIF	0.00875	129	0.0149	0.152	1	2	GJB3
Pubmed	24258150	Angiopoietin-like protein 2 accelerates carcinogenesis by activating chronic inflammation and oxidative stress.	Pubmed	0.00875	129	0.0149	0.152	1	2	ANGPTL2
Pubmed	10681521	Mechanisms by which soluble endothelial cell protein C receptor modulates protein C and activated protein C function.	Pubmed	0.00875	129	0.0149	0.152	1	2	PROCR
Pubmed	11229460	Rheumatoid arthritis in southern Spain: toward elucidation of a unifying role of the HLA class II region in disease predisposition.	GeneRIF	0.00875	129	0.0149	0.152	1	2	HLA-DQB1
Pubmed	21727005	Intragenic rearrangements in LARGE and POMGNT1 genes in severe dystroglycanopathies.	GeneRIF	0.00875	129	0.0149	0.152	1	2	LARGE
Pubmed	20407391	Association of common variants in NPPA and NPPB with blood pressure does not translate into kidney damage in a general population study.	GeneRIF	0.00875	129	0.0149	0.152	1	2	NPPA
Pubmed	20955473	Neurotrophin receptors TrkB.T1 and p75NTR cooperate in modulating both functional and structural plasticity in mature hippocampal neurons.	GeneRIF	0.00875	129	0.0149	0.152	1	2	NGFR
Pubmed	17023579	Effects of membrane and soluble EPCR on the hemostatic balance and endotoxemia in mice.	GeneRIF	0.00875	129	0.0149	0.152	1	2	PROCR
Pubmed	19635983	Biomarkers of left atrial volume: a longitudinal study in patients with end stage renal disease.	GeneRIF	0.00875	129	0.0149	0.152	1	2	NPPA
Pubmed	12083821	Developmental expression of HERV-R (ERV3) and HERV-K in human tissue.	Pubmed	0.00875	129	0.0149	0.152	1	2	ERV3-1
Pubmed	21418452	HLA-DQ haplotypes differ by ethnicity in patients with childhood-onset diabetes.	GeneRIF	0.00875	129	0.0149	0.152	1	2	HLA-DQB1
Pubmed	17227822	Signaling requirements for translocation of P-Rex1, a key Rac2 exchange factor involved in chemoattractant-stimulated human neutrophil function.	GeneRIF	0.00875	129	0.0149	0.152	1	2	PREX1
Pubmed	6086309	The nucleotide sequence of the murine I-E beta b immune response gene: evidence for gene conversion events in class II genes of the major histocompatibility complex.	Pubmed	0.00875	129	0.0149	0.152	1	2	HLA-DQB1
Pubmed	16891216	Subtle differences in HLA DQ haplotype-associated presentation of AChR alpha-chain peptides may suffice to mediate myasthenia gravis.	GeneRIF	0.00875	129	0.0149	0.152	1	2	HLA-DQB1
Pubmed	12089669	Human leukocyte antigen class II alleles may contribute to the severity of hepatitis C virus-related liver disease.	GeneRIF	0.00875	129	0.0149	0.152	1	2	HLA-DQB1
Pubmed	20042595	Differentiation of embryonic stem cells 1 (Dies1) is a component of bone morphogenetic protein 4 (BMP4) signaling pathway required for proper differentiation of mouse embryonic stem cells.	GeneRIF	0.00875	129	0.0149	0.152	1	2	BMP4
Pubmed	23593151	Interaction between HLA-DRB1-DQB1 haplotypes in Sardinian multiple sclerosis population.	Pubmed	0.00875	129	0.0149	0.152	1	2	HLA-DQB1
Pubmed	16707473	Intratumor CD4 T-cell accumulation requires stronger priming than for expansion and lymphokine secretion.	Pubmed	0.00875	129	0.0149	0.152	1	2	HLA-DQB1
Pubmed	16301118	Tissue transglutaminase interacts with protein kinase A anchor protein 13 in prostate cancer.	GeneRIF	0.00875	129	0.0149	0.152	1	2	TGM2
Pubmed	23330251	Genetic defects in pregnenolone synthesis.	GeneRIF	0.00875	129	0.0149	0.152	1	2	CYP11A1
Pubmed	6422542	Signal sequences distinguish class II histocompatibility antigen beta chains of different loci.	Pubmed	0.00875	129	0.0149	0.152	1	2	HLA-DQB1
Pubmed	24533866	Glutathione-conjugated sulfanylalkanols are substrates for ABCC11 and ¿-glutamyl transferase 1: a potential new pathway for the formation of odorant precursors in the apocrine sweat gland.	Pubmed	0.00875	129	0.0149	0.152	1	2	GGT1
Pubmed	25300013	Mechanisms and energetics of potassium channel block by local anesthetics and antifungal agents.	Pubmed	0.00875	129	0.0149	0.152	1	2	KCNN4
Pubmed	15128815	Cellular and humoral immunity against vaccinia virus infection of mice.	Pubmed	0.00875	129	0.0149	0.152	1	2	HLA-DQB1
Pubmed	12825076	Mutations in the SPINK1 gene in idiopathic pancreatitis Italian patients.	GeneRIF	0.00875	129	0.0149	0.152	1	2	SPINK1

Category	ID	Name	Source	p-value	q-value Bonferroni	q-value FDR B&H	q-value FDR B&Y	Hit Count in Query List	Hit Count in Genome	Hit in Query List
Pubmed	11726557	Suppression of the deafness and thyroid dysfunction in Thrb-null mice by an independent mutation in the Thra thyroid hormone receptor alpha gene.	GeneRIF	0.00875	129	0.0149	0.152	1	2	THRA
Pubmed	18326745	Elevated retina-specific expression of the small heat shock protein, alphaA-crystallin, is associated with photoreceptor protection in experimental uveitis.	Pubmed	0.00875	129	0.0149	0.152	1	2	CRYAA
Pubmed	12493718	Immunolocalization of inhibin and activin alpha and betaB subunits and expression of corresponding messenger RNAs in the human adult testis.	GeneRIF	0.00875	129	0.0149	0.152	1	2	INHBB
Pubmed	24324211	CX3CL1 and CX3CR1 expression in tertiary lymphoid structures in salivary gland infiltrates: fractalkine contribution to lymphoid neogenesis in Sjogren's syndrome.	Pubmed	0.00875	129	0.0149	0.152	1	2	CX3CL1
Pubmed	18657169	Bone morphogenetic protein 4 signaling regulates development of the anterior visceral endoderm in the mouse embryo.	GeneRIF	0.00875	129	0.0149	0.152	1	2	BMP4
Pubmed	21118624	Frequency of HLA-DQA1*0501 and DQB1*0201 alleles in patients with coeliac disease, their first-degree relatives and controls in Jordan.	GeneRIF	0.00875	129	0.0149	0.152	1	2	HLA-DQB1
Pubmed	17609255	Smoking impairs muscle protein synthesis and increases the expression of myostatin and MAFbx in muscle.	GeneRIF	0.00875	129	0.0149	0.152	1	2	FBXO32
Pubmed	17714036	HLA-DQ haplotypes in Spanish and German families with Graves' disease: contribution to DQA1*0501-DQB1*0301 mediated genetic susceptibility from fathers.	GeneRIF	0.00875	129	0.0149	0.152	1	2	HLA-DQB1
Pubmed	20090848	Mice lacking thyroid hormone receptor Beta show enhanced apoptosis and delayed liver commitment for proliferation after partial hepatectomy.	Pubmed	0.00875	129	0.0149	0.152	1	2	THRA
Pubmed	12124873	Differences in idiopathic inflammatory myopathy phenotypes and genotypes between Mesoamerican Mestizos and North American Caucasians: ethnogeographic influences in the genetics and clinical expression of myositis.	GeneRIF	0.00875	129	0.0149	0.152	1	2	HLA-DQB1
Pubmed	24472859	Serum MASP-1 in complex with MBL activates endothelial cells.	Pubmed	0.00875	129	0.0149	0.152	1	2	MASP1
Pubmed	24732400	Loss of collagen VII is associated with reduced transglutaminase 2 abundance and activity.	Pubmed	0.00875	129	0.0149	0.152	1	2	TGM2
Pubmed	12659864	Crystallization and preliminary X-ray structural studies of hemoglobin A2 and hemoglobin E, isolated from the blood samples of beta-thalassemic patients.	GeneRIF	0.00875	129	0.0149	0.152	1	2	HBA2
Pubmed	15286785	Donor APCs are required for maximal GVHD but not for GVL.	Pubmed	0.00875	129	0.0149	0.152	1	2	HLA-DQB1
Pubmed	8702639	Fibrillin-1 and fibulin-2 interact and are colocalized in some tissues.	Pubmed	0.00875	129	0.0149	0.152	1	2	FBLN2
Pubmed	18616983	Branched-chain amino acids and arginine suppress MaFbx/atrogin-1 mRNA expression via mTOR pathway in C2C12 cell line.	GeneRIF	0.00875	129	0.0149	0.152	1	2	FBXO32
Pubmed	20739468	Microarray analysis of iris gene expression in mice with mutations influencing pigmentation.	Pubmed	0.00875	129	0.0149	0.152	1	2	GPNMB
Pubmed	22528235	Expression of IRF-4 and IBP in skin lesions of patients with psoriasis vulgaris.	Pubmed	0.00875	129	0.0149	0.152	1	2	TSPO
Pubmed	23267013	The intramembrane protease Sppl2a is required for B cell and DC development and survival via cleavage of the invariant chain.	GeneRIF	0.00875	129	0.0149	0.152	1	2	CD74
Pubmed	16573562	HLA-DRB1, -DQB1 alleles in head and neck carcinoma patients.	GeneRIF	0.00875	129	0.0149	0.152	1	2	HLA-DQB1
Pubmed	10079113	In vivo blood flow abnormalities in the transgenic knockout sickle cell mouse.	Pubmed	0.00875	129	0.0149	0.152	1	2	HBA2
Pubmed	24531688	Loss of Mpzl3 function causes various skin abnormalities and greatly reduced adipose depots.	Pubmed	0.00875	129	0.0149	0.152	1	2	MPZL3
Pubmed	12712614	[The role of enzymatic features of hypoxia in ulcers].	GeneRIF	0.00875	129	0.0149	0.152	1	2	ADA
Pubmed	22611320	CD74 and macrophage migration inhibitory factor as therapeutic targets in gastric cancer.	GeneRIF	0.00875	129	0.0149	0.152	1	2	CD74
Pubmed	8506363	Positional specification of ventricular myosin light chain 2 expression in the primitive murine heart tube.	Pubmed	0.00875	129	0.0149	0.152	1	2	NPPA
Pubmed	19840940	Transglutaminase 2 regulates the GTPase-activating activity of Bcr.	GeneRIF	0.00875	129	0.0149	0.152	1	2	TGM2
Pubmed	15238770	The role of CFTR and SPINK-1 mutations in pancreatic disorders in HIV-positive patients: a case-control study.	GeneRIF	0.00875	129	0.0149	0.152	1	2	SPINK1
Pubmed	12606540	Distinct transglutaminase 2-independent and transglutaminase 2-dependent pathways mediate articular chondrocyte hypertrophy.	GeneRIF	0.00875	129	0.0149	0.152	1	2	TGM2
Pubmed	21277289	Regulation of aldo-keto reductase AKR1B10 gene expression: involvement of transcription factor Nrf2.	GeneRIF	0.00875	129	0.0149	0.152	1	2	AKR1B10
Pubmed	18769865	Temporal trends of HLA genotype frequencies of type 1 diabetes patients in Sweden from 1986 to 2005 suggest altered risk.	GeneRIF	0.00875	129	0.0149	0.152	1	2	HLA-DQB1
Pubmed	24821910	CX3CL1 (fractalkine) and its receptor CX3CR1 regulate atopic dermatitis by controlling effector T cell retention in inflamed skin.	Pubmed	0.00875	129	0.0149	0.152	1	2	CX3CL1
Pubmed	20199803	Genetic variation in CYP11A1 and StAR in relation to endometrial cancer risk.	GeneRIF	0.00875	129	0.0149	0.152	1	2	CYP11A1
Pubmed	12794112	Atrial natriuretic peptide polarizes human dendritic cells toward a Th2-promoting phenotype through its receptor guanylyl cyclase-coupled receptor A.	GeneRIF	0.00875	129	0.0149	0.152	1	2	NPPA
Pubmed	20100475	Regulation of cellular actin architecture by S100A10.	GeneRIF	0.00875	129	0.0149	0.152	1	2	ACTA1
Pubmed	24935307	Serum fractalkine and interferon-gamma inducible protein-10 concentrations are early detection markers for acute renal allograft rejection.	Pubmed	0.00875	129	0.0149	0.152	1	2	CX3CL1
Pubmed	9857240	Regulation of human prostate-specific antigen gene expression in transgenic mice: evidence for an enhancer between the PSA and human glandular kallikrein-1 genes.	GeneRIF	0.00875	129	0.0149	0.152	1	2	KLK1

Category	ID	Name	Source	p-value	q-value Bonferroni	q-value FDR B&H	q-value FDR B&Y	Hit Count in Query List	Hit Count in Genome	Hit in Query List
Pubmed	23951013	The MRC1/CD68 ratio is positively associated with adipose tissue lipogenesis and with muscle mitochondrial gene expression in humans.	Pubmed	0.00875	129	0.0149	0.152	1	2	CD68
Pubmed	20810110	Role of transglutaminases in cuff-induced atherosclerotic lesion formation in femoral arteries of ApoE3 Leiden mice.	GeneRIF	0.00875	129	0.0149	0.152	1	2	TGM2
Pubmed	22142843	Overexpression of phospho mutant forms of transglutaminase 2 downregulates epidermal growth factor receptor.	GeneRIF	0.00875	129	0.0149	0.152	1	2	TGM2
Pubmed	939824	A chondroplastic, brachymorphic and stubby chondrodystophies in mice.	Pubmed	0.00875	129	0.0149	0.152	1	2	PAPSS2
Pubmed	11535622	Deficiency of triad junction and contraction in mutant skeletal muscle lacking junctophilin type 1.	Pubmed	0.00875	129	0.0149	0.152	1	2	JPH2
Pubmed	22498810	Using bioinformatics and systems genetics to dissect HDL-cholesterol genetics in an MRL/MpJ x SM/J intercross.	Pubmed	0.00875	129	0.0149	0.152	1	2	LIPG
Pubmed	21627832	The effect of cathepsin K deficiency on airway development and TGF-ß1 degradation.	GeneRIF	0.00875	129	0.0149	0.152	1	2	CTSK
Pubmed	17762854	Transglutaminase autoantibodies in dermatitis herpetiformis and celiac sprue.	GeneRIF	0.00875	129	0.0149	0.152	1	2	TGM2
Pubmed	15603876	HLA-DRB1 and -DQB1 alleles in mestizo patients with Vogt-Koyanagi-Harada's disease in Southern California.	GeneRIF	0.00875	129	0.0149	0.152	1	2	HLA-DQB1
Pubmed	19740516	Napsin A and thyroid transcription factor-1 expression in carcinomas of the lung, breast, pancreas, colon, kidney, thyroid, and malignant mesothelioma.	GeneRIF	0.00875	129	0.0149	0.152	1	2	NAPSA
Pubmed	16253915	Functional assessment of compound mutations in the KCNQ1 and KCNH2 genes associated with long QT syndrome.	GeneRIF	0.00875	129	0.0149	0.152	1	2	KCNH2
Pubmed	3500915	Comparative sequence analysis of cDNA clones encoding I-A molecules of the CH12 B cell lymphoma: nucleotide differences do not account for their "defective" function in B cell stimulation.	Pubmed	0.00875	129	0.0149	0.152	1	2	HLA-DQB1
Pubmed	11060316	THIK-1 and THIK-2, a novel subfamily of tandem pore domain K+ channels.	Pubmed	0.00875	129	0.0149	0.152	1	2	KCNK12
Pubmed	9537446	Contribution of the murine mdr1a P-glycoprotein to hepatobiliary and intestinal elimination of cationic drugs as measured in mice with an mdr1a gene disruption.	Pubmed	0.00875	129	0.0149	0.152	1	2	CTSK
Pubmed	9382920	Histocompatibility gene mutation rates in the mouse: a 25-year review.	Pubmed	0.00875	129	0.0149	0.152	1	2	HLA-DQB1
Pubmed	17724704	Mutations and polymorphisms in hemoglobin genes and the risk of pulmonary hypertension and death in sickle cell disease.	GeneRIF	0.00875	129	0.0149	0.152	1	2	HBB
Pubmed	16645504	T280M variation of the CX3C receptor gene is associated with increased risk for severe respiratory syncytial virus bronchiolitis.	GeneRIF	0.00875	129	0.0149	0.152	1	2	CX3CL1
Pubmed	21392254	High levels of protein C are determined by PROCR haplotype 3.	GeneRIF	0.00875	129	0.0149	0.152	1	2	PROCR
Pubmed	16691213	ATP-binding cassette transporter 1 and transglutaminase 2 act on the same genetic pathway in the apoptotic cell clearance.	GeneRIF	0.00875	129	0.0149	0.152	1	2	TGM2
Pubmed	11285306	The regulatory role of DR4 in a spontaneous diabetes DQ8 transgenic model.	Pubmed	0.00875	129	0.0149	0.152	1	2	HLA-DQB1
Pubmed	12530640	Regulation of steroid hormone biosynthesis in R2C and MA-10 Leydig tumor cells: role of the cholesterol transfer proteins StAR and PBR.	Pubmed	0.00875	129	0.0149	0.152	1	2	TSPO
Pubmed	20861181	Different HLA class II (DRB1 and DQB1) alleles determine either susceptibility or resistance to NMO and multiple sclerosis among the French Afro-Caribbean population.	GeneRIF	0.00875	129	0.0149	0.152	1	2	HLA-DQB1
Pubmed	17396032	Association of extracellular cleavage of E-cadherin mediated by MMP-7 with HGF-induced in vitro invasion in human stomach cancer cells.	GeneRIF	0.00875	129	0.0149	0.152	1	2	MMP7
Pubmed	23303949	Doublecortin (Dcx) family proteins regulate filamentous actin structure in developing neurons.	GeneRIF	0.00875	129	0.0149	0.152	1	2	DCLK1
Pubmed	20052289	Interferon-inducible IFI16, a negative regulator of cell growth, down-regulates expression of human telomerase reverse transcriptase (hTERT) gene.	GeneRIF	0.00875	129	0.0149	0.152	1	2	IFI16
Pubmed	12513693	Identification and cloning of two isoforms of human high-temperature requirement factor A3 (HtrA3), characterization of its genomic structure and comparison of its tissue distribution with HtrA1 and HtrA2.	GeneRIF	0.00875	129	0.0149	0.152	1	2	HTRA1
Pubmed	11802942	[Analysis of the association between HLA-DRB(1), DQB(1) gene and pulmonary tuberculosis complicated with diabetes mellitus].	GeneRIF	0.00875	129	0.0149	0.152	1	2	HLA-DQB1
Pubmed	23383068	Overexpression of MyrAkt1 in endothelial cells leads to erythropoietin- and BMP4-independent splenic erythropoiesis in mice.	GeneRIF	0.00875	129	0.0149	0.152	1	2	BMP4
Pubmed	11120878	Increased sensitivity to thyroid hormone in mice with complete deficiency of thyroid hormone receptor alpha.	Pubmed	0.00875	129	0.0149	0.152	1	2	THRA
Pubmed	22897138	Fractalkine and its receptor (CX3CR1) in patients with stable coronary artery disease and diabetes mellitus.	GeneRIF	0.00875	129	0.0149	0.152	1	2	CX3CL1
Pubmed	17845309	Association of polymorphisms of human leucocyte antigen-DQA1 and DQB1 alleles with chronic hepatitis B virus infection, liver cirrhosis and hepatocellular carcinoma in Chinese.	GeneRIF	0.00875	129	0.0149	0.152	1	2	HLA-DQB1
Pubmed	8464058	Protein-DNA interactions of the mouse alpha A-crystallin control regions. Differences between expressing and non-expressing cells.	Pubmed	0.00875	129	0.0149	0.152	1	2	CRYAA
Pubmed	6434690	Gene conversion between murine class II major histocompatibility complex loci. Functional and molecular evidence from the bm 12 mutant.	Pubmed	0.00875	129	0.0149	0.152	1	2	HLA-DQB1
Pubmed	21466834	Characterization of the effect of TIMAP phosphorylation on its interaction with protein phosphatase 1.	GeneRIF	0.00875	129	0.0149	0.152	1	2	PPP1R16B
Pubmed	25206283	PRSS1 and SPINK1 mutations in idiopathic chronic and recurrent acute pancreatitis.	Pubmed	0.00875	129	0.0149	0.152	1	2	SPINK1

Category	ID	Name	Source	p-value	q-value Bonferroni	q-value FDR B&H	q-value FDR B&Y	Hit Count in Query List	Hit Count in Genome	Hit in Query List
Pubmed	25519903	HTRA1 (high temperature requirement A serine peptidase 1) gene is transcriptionally regulated by insertion/deletion nucleotides located at the 3' end of the ARMS2 (age-related maculopathy susceptibility 2) gene in patients with age-related macular degeneration.	Pubmed	0.00875	129	0.0149	0.152	1	2	HTRA1
Pubmed	23671666	Differential distribution of the Ca (2+) regulator Pcp4 in the branchial arches is regulated by Hoxa2.	Pubmed	0.00875	129	0.0149	0.152	1	2	PCP4
Pubmed	22308398	Stage-specific functions of leukemia/lymphoma-related factor (LRF) in the transcriptional control of osteoclast development.	GeneRIF	0.00875	129	0.0149	0.152	1	2	CTSK
Pubmed	16847316	Shared stabilization functions of pyrimidine-rich determinants in the erythroid 15-lipoxygenase and alpha-globin mRNAs.	GeneRIF	0.00875	129	0.0149	0.152	1	2	HBA2
Pubmed	23644699	Association between promoters polymorphisms of matrix metalloproteinases and risk of digestive cancers: a meta-analysis.	Pubmed	0.00875	129	0.0149	0.152	1	2	MMP7
Pubmed	25413104	HLA-DQ2/DQ8 and HLA-DQB1*02 homozygosity typing by real-time polymerase chain reaction for the assessment of celiac disease genetic risk: evaluation of a Spanish celiac population.	Pubmed	0.00875	129	0.0149	0.152	1	2	HLA-DQB1
Pubmed	23333309	Redox-dependent control of FOXO/DAF-16 by transportin-1.	GeneRIF	0.00875	129	0.0149	0.152	1	2	FOXO4
Pubmed	11137199	Gender differences in genetic susceptibility for lung cancer.	GeneRIF	0.00875	129	0.0149	0.152	1	2	CYP11A1
Pubmed	19117950	Atrogin-1/MAFbx enhances simulated ischemia/reperfusion-induced apoptosis in cardiomyocytes through degradation of MAPK phosphatase-1 and sustained JNK activation.	Pubmed	0.00875	129	0.0149	0.152	1	2	FBXO32
Pubmed	17505143	CX3CR1 polymorphisms are associated with atopy but not asthma in German children.	GeneRIF	0.00875	129	0.0149	0.152	1	2	CX3CL1
Pubmed	22227097	Features of the retinal environment which affect the activities and product profile of cholesterol-metabolizing cytochromes P450 CYP27A1 and CYP11A1.	GeneRIF	0.00875	129	0.0149	0.152	1	2	CYP11A1
Pubmed	17785916	New HLA DRB1 and DQB1 haplotypes in a pedigree of familial Graves' disease in Japan.	GeneRIF	0.00875	129	0.0149	0.152	1	2	HLA-DQB1
Pubmed	16114898	14-3-3 Protein interacts with nuclear localization sequence of forkhead transcription factor FoxO4.	GeneRIF	0.00875	129	0.0149	0.152	1	2	FOXO4
Pubmed	21105874	Stress-induced expression of the p75 neurotrophin receptor is regulated by O-GlcNAcylation of the Sp1 transcription factor.	GeneRIF	0.00875	129	0.0149	0.152	1	2	NGFR
Pubmed	19563789	Fractalkine and CX3CR1 are involved in the migration of intravenously grafted human bone marrow stromal cells toward ischemic brain lesion in rats.	GeneRIF	0.00875	129	0.0149	0.152	1	2	CX3CL1
Pubmed	19690958	Elevated serum matrix metalloproteinase-3 and -7 in H. pylori-related gastric cancer can be biomarkers correlating with a poor survival.	GeneRIF	0.00875	129	0.0149	0.152	1	2	MMP7
Pubmed	21625219	Cancer cells promote survival through depletion of the von Hippel-Lindau tumor suppressor by protein crosslinking.	GeneRIF	0.00875	129	0.0149	0.152	1	2	TGM2
Pubmed	16045809	Overexpression of cathepsin K during silica-induced lung fibrosis and control by TGF-beta.	GeneRIF	0.00875	129	0.0149	0.152	1	2	CTSK
Pubmed	15304375	Cardiac glucose utilization in mice with mutated alpha- and beta-thyroid hormone receptors.	Pubmed	0.00875	129	0.0149	0.152	1	2	THRA
Pubmed	23178147	Interleukin-33 overexpression is associated with gamma-glutamyl transferase in biliary atresia.	GeneRIF	0.00875	129	0.0149	0.152	1	2	GGT1
Pubmed	11909868	CX3CR1 tyrosine sulfation enhances fractalkine-induced cell adhesion.	GeneRIF	0.00875	129	0.0149	0.152	1	2	CX3CL1
Pubmed	18855222	Serum levels of natriuretic peptides in children with various types of loading conditions.	GeneRIF	0.00875	129	0.0149	0.152	1	2	NPPA
Pubmed	18316707	HTRA1 variants in exudative age-related macular degeneration and interactions with smoking and CFH.	GeneRIF	0.00875	129	0.0149	0.152	1	2	HTRA1
Pubmed	24727450	Secreted type of amyloid precursor protein induces glial differentiation by stimulating the BMP/Smad signaling pathway.	Pubmed	0.00875	129	0.0149	0.152	1	2	BMP4
Pubmed	18095658	Zn2+ enhances the molecular chaperone function and stability of alpha-crystallin.	GeneRIF	0.00875	129	0.0149	0.152	1	2	CRYAA
Pubmed	16127153	Activin controls skin morphogenesis and wound repair predominantly via stromal cells and in a concentration-dependent manner via keratinocytes.	GeneRIF	0.00875	129	0.0149	0.152	1	2	INHBB
Pubmed	22952837	Macrophage migration inhibitory factor inhibits the migration of cartilage end plate-derived stem cells by reacting with CD74.	GeneRIF	0.00875	129	0.0149	0.152	1	2	CD74
Pubmed	20809186	Expressions of matrix metalloproteinase-7 and matrix metalloproteinase-14 associated with the activation of extracellular signal-regulated kinase1/2 in human brain gliomas of different pathological grades.	GeneRIF	0.00875	129	0.0149	0.152	1	2	MMP7
Pubmed	24758334	The association of HLA-DRB1 and HLA-DQB1 alleles with the development of factor VIII inhibitors in severe haemophilia A patients in India.	Pubmed	0.00875	129	0.0149	0.152	1	2	HLA-DQB1
Pubmed	8651889	Bone morphogenetic protein-2 and -4 expression during murine orofacial development.	Pubmed	0.00875	129	0.0149	0.152	1	2	BMP4
Pubmed	15004872	Accumulation and aberrant modifications of alpha-crystallins in anterior polar cataracts.	GeneRIF	0.00875	129	0.0149	0.152	1	2	CRYAA
Pubmed	12819058	Regulation of pulmonary and systemic bacterial lipopolysaccharide responses in transgenic mice expressing human elafin.	GeneRIF	0.00875	129	0.0149	0.152	1	2	SLPI
Pubmed	1353685	Identification of transglutaminase substrates in HT29 colon cancer cells: use of 5-(biotinamido)pentylamine as a transglutaminase-specific probe.	Pubmed	0.00875	129	0.0149	0.152	1	2	TGM2
Pubmed	23113638	The DC-HIL ligand syndecan-4 is a negative regulator of T-cell allo-reactivity responsible for graft-versus-host disease.	GeneRIF	0.00875	129	0.0149	0.152	1	2	GPNMB
Pubmed	22621787	Modulation of endothelial SK3 channel activity by Ca²+dependent caveolar trafficking.	GeneRIF	0.00875	129	0.0149	0.152	1	2	KCNN4

Category	ID	Name	Source	p-value	q-value Bonferroni	q-value FDR B&H	q-value FDR B&Y	Hit Count in Query List	Hit Count in Genome	Hit in Query List
Pubmed	10888604	A candidate gene for psoriasis near HLA-C, HCR (Pg8), is highly polymorphic with a disease-associated susceptibility allele.	Pubmed	0.00875	129	0.0149	0.152	1	2	CDSN
Pubmed	8058073	Developmental expression of mouse steroidogenic factor-1, an essential regulator of the steroid hydroxylases.	Pubmed	0.00875	129	0.0149	0.152	1	2	CYP11A1
Pubmed	23493375	Role of thyroid hormone receptor subtypes a and ß on gene expression in the cerebral cortex and striatum of postnatal mice.	GeneRIF	0.00875	129	0.0149	0.152	1	2	THRA
Pubmed	14991600	MHC class II-independent CD25+ CD4+ CD8alpha beta+ alpha beta T cells attenuate CD4+ T cell-induced transfer colitis.	Pubmed	0.00875	129	0.0149	0.152	1	2	HLA-DQB1
Pubmed	18378617	Leukocyte integrin Mac-1 promotes acute cardiac allograft rejection.	GeneRIF	0.00875	129	0.0149	0.152	1	2	HLA-DQB1
Pubmed	18450852	Human leukocyte antigen and cytokine receptor gene polymorphisms associated with heterogeneous immune responses to mumps viral vaccine.	GeneRIF	0.00875	129	0.0149	0.152	1	2	HLA-DQB1
Pubmed	14702176	Overexpression of iNOS and down-regulation of BMPs-2, 4 and 7 in retinoic acid induced cleft palate formation.	GeneRIF	0.00875	129	0.0149	0.152	1	2	BMP4
Pubmed	15120192	HLA class II typing in newborns reveals a low frequency of the DRB1*04 allele and a high frequency of DRB1*11 allele in three regions of continental Italy.	GeneRIF	0.00875	129	0.0149	0.152	1	2	HLA-DQB1
Pubmed	19321257	Overexpression of bone morphogenetic protein 4 enhances the invasiveness of Smad4-deficient human colorectal cancer cells.	GeneRIF	0.00875	129	0.0149	0.152	1	2	BMP4
Pubmed	12138181	Involvement of histone methylation and phosphorylation in regulation of transcription by thyroid hormone receptor.	Pubmed	0.00875	129	0.0149	0.152	1	2	THRA
Pubmed	16402023	Effect of p75NTR on the regulation of photoreceptor apoptosis in the rd mouse.	GeneRIF	0.00875	129	0.0149	0.152	1	2	NGFR
Pubmed	14551263	Activins are critical modulators of growth and survival.	GeneRIF	0.00875	129	0.0149	0.152	1	2	INHBB
Pubmed	8781443	Inactivation of mouse alpha-globin gene by homologous recombination: mouse model of hemoglobin H disease.	Pubmed	0.00875	129	0.0149	0.152	1	2	HBA2
Pubmed	18316031	RBP4 disrupts vitamin A uptake homeostasis in a STRA6-deficient animal model for Matthew-Wood syndrome.	GeneRIF	0.00875	129	0.0149	0.152	1	2	STRA6
Pubmed	20874003	Clinical significance of GPR56, transglutaminase 2, and NF-¿B in esophageal squamous cell carcinoma.	GeneRIF	0.00875	129	0.0149	0.152	1	2	TGM2
Pubmed	7719943	Asymmetric signaling requirements for thymocyte commitment to the CD4+ versus CD8+ T cell lineages: a new perspective on thymic commitment and selection.	Pubmed	0.00875	129	0.0149	0.152	1	2	HLA-DQB1
Pubmed	19850045	The histone demethylase RBP2 Is overexpressed in gastric cancer and its inhibition triggers senescence of cancer cells.	GeneRIF	0.00875	129	0.0149	0.152	1	2	KDM5A
Pubmed	15885635	HLA class II involvement in HIV-associated Toxoplasmic encephalitis development.	GeneRIF	0.00875	129	0.0149	0.152	1	2	HLA-DQB1
Pubmed	25146988	Molecular mechanism of formation of cortical opacity in CRYAAN101D transgenic mice.	Pubmed	0.00875	129	0.0149	0.152	1	2	CRYAA
Pubmed	23539451	Endothelial cell protein C receptor opposes mesothelioma growth driven by tissue factor.	GeneRIF	0.00875	129	0.0149	0.152	1	2	PROCR
Pubmed	19350579	The DC-HIL/syndecan-4 pathway inhibits human allogeneic T-cell responses.	GeneRIF	0.00875	129	0.0149	0.152	1	2	GPNMB
Pubmed	25862418	MBL-associated serine proteases (MASPs) and infectious diseases.	Pubmed	0.00875	129	0.0149	0.152	1	2	MASP1
Pubmed	9747029	Expression and chromosomal localization of KIAA0369, a putative kinase structurally related to Doublecortin.	Pubmed	0.00875	129	0.0149	0.152	1	2	DCLK1
Pubmed	17823308	The ligand occupancy of endothelial protein C receptor switches the protease-activated receptor 1-dependent signaling specificity of thrombin from a permeability-enhancing to a barrier-protective response in endothelial cells.	GeneRIF	0.00875	129	0.0149	0.152	1	2	PROCR
Pubmed	16410722	Connecting p63 to cellular proliferation: the example of the adenosine deaminase target gene.	GeneRIF	0.00875	129	0.0149	0.152	1	2	ADA
Pubmed	17406854	Relationship between increased relative birthweight and infections during pregnancy in children with a high-risk diabetes HLA genotype.	GeneRIF	0.00875	129	0.0149	0.152	1	2	HLA-DQB1
Pubmed	3857584	Lens-specific expression of the chloramphenicol acetyltransferase gene promoted by 5' flanking sequences of the murine alpha A-crystallin gene in explanted chicken lens epithelia.	Pubmed	0.00875	129	0.0149	0.152	1	2	CRYAA
Pubmed	21896629	Transmembrane protein 2 (Tmem2) is required to regionally restrict atrioventricular canal boundary and endocardial cushion development.	GeneRIF	0.00875	129	0.0149	0.152	1	2	BMP4
Pubmed	24717731	Natriuretic peptides buffer renin-dependent hypertension.	Pubmed	0.00875	129	0.0149	0.152	1	2	NPPA
Pubmed	24200403	The role of EDEM2 compared with EDEM1 in ricin transport from the endoplasmic reticulum to the cytosol.	Pubmed	0.00875	129	0.0149	0.152	1	2	EDEM2
Pubmed	11872237	Infectious complications in sickle cell disease are influenced by HLA class II alleles.	GeneRIF	0.00875	129	0.0149	0.152	1	2	HLA-DQB1
Pubmed	17054378	Molecular dynamics calculations of wild type vs. mutant protein C: relationship between binding affinity to endothelial cell protein C receptor and hereditary disease.	GeneRIF	0.00875	129	0.0149	0.152	1	2	PROCR
Pubmed	18373618	Tumor necrosis factor p55 and p75 receptors are involved in chemical-induced apoptosis of dentate granule neurons.	GeneRIF	0.00875	129	0.0149	0.152	1	2	NGFR
Pubmed	11094058	ProSAAS processing in mouse brain and pituitary.	Pubmed	0.00875	129	0.0149	0.152	1	2	CPE
Pubmed	24401612	Vascular expression of the chemokine CX3CL1 promotes osteoclast recruitment and exacerbates bone resorption in an irradiated murine model.	Pubmed	0.00875	129	0.0149	0.152	1	2	CX3CL1
Pubmed	22227189	The matrix metalloproteinase-7 regulates the extracellular shedding of syndecan-2 from colon cancer cells.	GeneRIF	0.00875	129	0.0149	0.152	1	2	MMP7
Pubmed	4212045	Haemoglobin inkster (alpha2 85aspartic acid leads to valine beta2) coexisting with beta-thalassaemia in a Caucasian family.	Pubmed	0.00875	129	0.0149	0.152	1	2	HBA2

Category	ID	Name	Source	p-value	q-value Bonferroni	q-value FDR B&H	q-value FDR B&Y	Hit Count in Query List	Hit Count in Genome	Hit in Query List
Pubmed	12595234	Regional expression of p75NTR contributes to neurotrophin regulation of cerebellar patterning.	GeneRIF	0.00875	129	0.0149	0.152	1	2	NGFR
Pubmed	19671703	Absence of direct cyclic nucleotide modulation of mEAG1 and hERG1 channels revealed with fluorescence and electrophysiological methods.	GeneRIF	0.00875	129	0.0149	0.152	1	2	KCNH2
Pubmed	10504457	Accumulation of matrilysin (MMP-7) and macrophage metalloelastase (MMP-12) in actinic damage.	Pubmed	0.00875	129	0.0149	0.152	1	2	MMP7
Pubmed	17207713	DQB1*0301 and DQB1*0601 modulate narcolepsy susceptibility in Koreans.	GeneRIF	0.00875	129	0.0149	0.152	1	2	HLA-DQB1
Pubmed	17893198	Endotoxemia and sepsis mortality reduction by non-anticoagulant activated protein C.	Pubmed	0.00875	129	0.0149	0.152	1	2	PROCR
Pubmed	14532291	Role of the conserved SRLFDQFFG region of alpha-crystallin, a small heat shock protein. Effect on oligomeric size, subunit exchange, and chaperone-like activity.	GeneRIF	0.00875	129	0.0149	0.152	1	2	CRYAA
Pubmed	7738119	Expression and localization of annexin VII (synexin) in muscle cells.	Pubmed	0.00875	129	0.0149	0.152	1	2	CLEC3B
Pubmed	15096192	Possession of human leucocyte antigen DQ6 alleles and the rate of CD4 T-cell decline in human immunodeficiency virus-1 infection.	GeneRIF	0.00875	129	0.0149	0.152	1	2	HLA-DQB1
Pubmed	16836882	[Possible association between polymorphisms of human leukocyte antigen-DQ genes and susceptibility to trichloroethylene-induced severe generalized dermatitis].	GeneRIF	0.00875	129	0.0149	0.152	1	2	HLA-DQB1
Pubmed	16210606	Tight regulation of IFN-gamma transcription and secretion in immature and mature B cells by the inhibitory MHC class I receptor, Ly49G2.	Pubmed	0.00875	129	0.0149	0.152	1	2	CD74
Pubmed	21903576	Control of protein kinase C activity, phorbol ester-induced cytoskeletal remodeling, and cell survival signals by the scaffolding protein SSeCKS/GRAVIN/AKAP12.	GeneRIF	0.00875	129	0.0149	0.152	1	2	AKAP12
Pubmed	19433603	Association and differential role of PRSS1 and SPINK1 mutation in early-onset and late-onset idiopathic chronic pancreatitis in Chinese subjects.	GeneRIF	0.00875	129	0.0149	0.152	1	2	SPINK1
Pubmed	24789788	The nucleocapsid domain of Gag is dispensable for actin incorporation into HIV-1 and for association of viral budding sites with cortical F-actin.	Pubmed	0.00875	129	0.0149	0.152	1	2	ACTA1
Pubmed	20691532	HLA class II alleles and multiple sclerosis in Tunisian patients.	GeneRIF	0.00875	129	0.0149	0.152	1	2	HLA-DQB1
Pubmed	21885613	Factor X binding to endothelial cell protein C receptor: comparison with factor VIIa and activated protein C.	GeneRIF	0.00875	129	0.0149	0.152	1	2	PROCR
Pubmed	22616995	Peripheral benzodiazepine receptor regulates vascular endothelial activations via suppression of the voltage-dependent anion channel-1.	GeneRIF	0.00875	129	0.0149	0.152	1	2	TSPO
Pubmed	23269702	Antagonism of inhibitor of apoptosis proteins increases bone metastasis via unexpected osteoclast activation.	GeneRIF	0.00875	129	0.0149	0.152	1	2	CTSK
Pubmed	14645507	Human beta-globin locus control region HS5 contains CTCF- and developmental stage-dependent enhancer-blocking activity in erythroid cells.	GeneRIF	0.00875	129	0.0149	0.152	1	2	HBB
Pubmed	15507397	Analysis of children with type 1 diabetes in Korea: high prevalence of specific anti-islet autoantibodies, immunogenetic similarities to Western populations with "unique" haplotypes, and lack of discrimination by aspartic acid at position 57 of DQB.	GeneRIF	0.00875	129	0.0149	0.152	1	2	HLA-DQB1
Pubmed	9582364	Role of Sp proteins and RORalpha in transcription regulation of murine prosaposin.	Pubmed	0.00875	129	0.0149	0.152	1	2	PSAP
Pubmed	12399446	The gene locus encoding iodothyronine deiodinase type 3 (Dio3) is imprinted in the fetus and expresses antisense transcripts.	GeneRIF	0.00875	129	0.0149	0.152	1	2	KLK14
Pubmed	24833109	Matrilysin/matrix metalloproteinase-7(MMP7) cleavage of perlecan/HSPG2 creates a molecular switch to alter prostate cancer cell behavior.	Pubmed	0.00875	129	0.0149	0.152	1	2	MMP7
Pubmed	15272014	Augmentation of tissue transglutaminase expression and activation by epidermal growth factor inhibit doxorubicin-induced apoptosis in human breast cancer cells.	GeneRIF	0.00875	129	0.0149	0.152	1	2	TGM2
Pubmed	9931005	Solution structure and dynamics of the CX3C chemokine domain of fractalkine and its interaction with an N-terminal fragment of CX3CR1.	Pubmed	0.00875	129	0.0149	0.152	1	2	CX3CL1
Pubmed	11686302	Cell-surface transglutaminase promotes fibronectin assembly via interaction with the gelatin-binding domain of fibronectin: a role in TGFbeta-dependent matrix deposition.	Pubmed	0.00875	129	0.0149	0.152	1	2	TGM2
Pubmed	24086375	SMG6 cleavage generates metastable decay intermediates from nonsense-containing ß-globin mRNA.	Pubmed	0.00875	129	0.0149	0.152	1	2	HBB
Pubmed	8567732	Identification and localization of an actin-binding motif that is unique to the epsilon isoform of protein kinase C and participates in the regulation of synaptic function.	Pubmed	0.00875	129	0.0149	0.152	1	2	ACTA1
Pubmed	21757696	Reactivity of the N-terminal region of fibronectin protein to transglutaminase 2 and factor XIIIA.	GeneRIF	0.00875	129	0.0149	0.152	1	2	TGM2
Pubmed	17625593	Activation of proteinase-activated receptor-2 by human kallikrein-related peptidases.	GeneRIF	0.00875	129	0.0149	0.152	1	2	KLK14
Pubmed	16514669	Bone morphogenetic protein-4 enhances vascular endothelial growth factor secretion by human retinal pigment epithelial cells.	GeneRIF	0.00875	129	0.0149	0.152	1	2	BMP4
Pubmed	8631316	A novel dystrophin/utrophin-associated protein is an enzymatically inactive member of the phosphoglucomutase superfamily.	Pubmed	0.00875	129	0.0149	0.152	1	2	PGM5
Pubmed	11771659	Large-scale screening for candidate genes of ossification of the posterior longitudinal ligament of the spine.	GeneRIF	0.00875	129	0.0149	0.152	1	2	BMP4
Pubmed	11839310	Solution structure of the calponin CH domain and fitting to the 3D-helical reconstruction of F-actin:calponin.	Pubmed	0.00875	129	0.0149	0.152	1	2	ACTA1
Pubmed	17341477	Expression and localization of plasma transglutaminase factor XIIIA in bone.	GeneRIF	0.00875	129	0.0149	0.152	1	2	TGM2

Category	ID	Name	Source	p-value	q-value Bonferroni	q-value FDR B&H	q-value FDR B&Y	Hit Count in Query List	Hit Count in Genome	Hit in Query List
Pubmed	20716950	BCL2 interaction with actin in vitro may inhibit cell motility by enhancing actin polymerization.	GeneRIF	0.00875	129	0.0149	0.152	1	2	ACTA1
Pubmed	17964057	Multifaceted roles of human elafin and secretory leukocyte proteinase inhibitor (SLPI), two serine protease inhibitors of the chelonianin family.	GeneRIF	0.00875	129	0.0149	0.152	1	2	SLPI
Pubmed	2752146	Locus assignment of two alpha-globin structural mutants from the Caribbean basin: alpha Fort de France (alpha 45 Arg) and alpha Spanish Town (alpha 27 Val).	Pubmed	0.00875	129	0.0149	0.152	1	2	HBA2
Pubmed	24936834	DC-HIL-expressing myelomonocytic cells are critical promoters of melanoma growth.	Pubmed	0.00875	129	0.0149	0.152	1	2	GPNMB
Pubmed	17213321	The SCL transcriptional network and BMP signaling pathway interact to regulate RUNX1 activity.	GeneRIF	0.00875	129	0.0149	0.152	1	2	BMP4
Pubmed	20494937	Cathepsin X is secreted by human osteoblasts, digests CXCL-12 and impairs adhesion of hematopoietic stem and progenitor cells to osteoblasts.	GeneRIF	0.00875	129	0.0149	0.152	1	2	CTSK
Pubmed	20233579	Genetic modification of the inner ear lateral semicircular canal phenotype of the Bmp4 haplo-insufficient mouse.	GeneRIF	0.00875	129	0.0149	0.152	1	2	BMP4
Pubmed	23664458	Dehydroepiandrosterone supplementation improves predictive markers for diminished ovarian reserve: serum AMH, inhibin B and antral follicle count.	Pubmed	0.00875	129	0.0149	0.152	1	2	INHBB
Pubmed	20002609	HLA DR-DQ combination associated with the increased risk of developing human HCV positive non-Hodgkin's lymphoma is related to the type II mixed cryoglobulinemia.	GeneRIF	0.00875	129	0.0149	0.152	1	2	HLA-DQB1
Pubmed	17170365	Human vascular smooth muscle cells express functionally active endothelial cell protein C receptor.	GeneRIF	0.00875	129	0.0149	0.152	1	2	PROCR
Pubmed	23496815	Airway epithelial cells initiate the allergen response through transglutaminase 2 by inducing IL-33 expression and a subsequent Th2 response.	GeneRIF	0.00875	129	0.0149	0.152	1	2	TGM2
Pubmed	21280221	In silico discovery and experimental validation of new protein-protein interactions.	GeneRIF	0.00875	129	0.0149	0.152	1	2	DYSF
Pubmed	22370814	Factor VIIa binding to endothelial cell protein C receptor: differences between mouse and human systems.	GeneRIF	0.00875	129	0.0149	0.152	1	2	PROCR
Pubmed	6447833	Characterization of ATPase in sarcoplasmic reticulum from two strains of dystrophic mice.	Pubmed	0.00875	129	0.0149	0.152	1	2	LARGE
Pubmed	18756979	[Use of ABC typing to redefine subtypes of acute-onset type 1 diabetes mellitus: study of 308 patients].	GeneRIF	0.00875	129	0.0149	0.152	1	2	HLA-DQB1
Pubmed	20639122	Protein oxidation in Huntington disease affects energy production and vitamin B6 metabolism.	GeneRIF	0.00875	129	0.0149	0.152	1	2	PDXK
Pubmed	24376798	HLA-DQB1*03 confers susceptibility to chronic hepatitis C in Japanese: a genome-wide association study.	Pubmed	0.00875	129	0.0149	0.152	1	2	HLA-DQB1
Pubmed	19671135	Natural antisense transcript of natriuretic peptide precursor A (NPPA): structural organization and modulation of NPPA expression.	GeneRIF	0.00875	129	0.0149	0.152	1	2	NPPA
Pubmed	17105769	Sequential implication of the mental retardation proteins ARHGEF6 and PAK3 in spine morphogenesis.	GeneRIF	0.00875	129	0.0149	0.152	1	2	ARHGEF6
Pubmed	11499613	Characterization of glycated hemoglobin in diabetic patients: usefulness of electrospray mass spectrometry in monitoring the extent and distribution of glycation.	Pubmed	0.00875	129	0.0149	0.152	1	2	HBB
Pubmed	18367676	CX3CL1 and CCL14 regulate extracellular matrix and adhesion molecules in the trophoblast: potential roles in human embryo implantation.	GeneRIF	0.00875	129	0.0149	0.152	1	2	CX3CL1
Pubmed	10837251	Cyclic AMP regulates the HERG K(+) channel by dual pathways.	Pubmed	0.00875	129	0.0149	0.152	1	2	KCNH2
Pubmed	12196893	HLA haplotypes and class II molecular alleles in Argentinian patients with pemphigus vulgaris.	GeneRIF	0.00875	129	0.0149	0.152	1	2	HLA-DQB1
Pubmed	18579430	Overexpression of A-kinase anchoring protein 12A activates sterol regulatory element binding protein-2 and enhances cholesterol efflux in hepatic cells.	GeneRIF	0.00875	129	0.0149	0.152	1	2	AKAP12
Pubmed	10727444	Interaction between succinyl CoA synthetase and the heme-biosynthetic enzyme ALAS-E is disrupted in sideroblastic anemia.	Pubmed	0.00875	129	0.0149	0.152	1	2	ALAS2
Pubmed	20041188	CX3CR1 is expressed by human B lymphocytes and mediates [corrected] CX3CL1 driven chemotaxis of tonsil centrocytes.	GeneRIF	0.00875	129	0.0149	0.152	1	2	CX3CL1
Pubmed	21525355	The human T-cell leukemia virus type 1 oncoprotein tax controls forkhead box O4 activity through degradation by the proteasome.	GeneRIF	0.00875	129	0.0149	0.152	1	2	FOXO4
Pubmed	6547932	The characterization of hemoglobin Manitoba or alpha (2)102(G9)Ser----Arg beta 2 and hemoglobin Contaldo or alpha (2)103(G10)His----Arg beta 2 by high performance liquid chromatography.	Pubmed	0.00875	129	0.0149	0.152	1	2	HBA2
Pubmed	17601796	Genetic removal of the A2A adenosine receptor enhances pulmonary inflammation, mucin production, and angiogenesis in adenosine deaminase-deficient mice.	GeneRIF	0.00875	129	0.0149	0.152	1	2	ADA
Pubmed	15310753	Basic fibroblast growth factor-induced cell death is effected through sustained activation of p38MAPK and up-regulation of the death receptor p75NTR.	GeneRIF	0.00875	129	0.0149	0.152	1	2	NGFR
Pubmed	9372952	Constitutive activation of gene expression by thyroid hormone receptor results from reversal of p53-mediated repression.	Pubmed	0.00875	129	0.0149	0.152	1	2	THRA
Pubmed	18652916	HLA-DQB1 and -DPB1 allele profile in HIV infected patients with and without pulmonary tuberculosis of south India.	GeneRIF	0.00875	129	0.0149	0.152	1	2	HLA-DQB1
Pubmed	16187186	Mutation analysis of SPINK1 and CFTR gene in Korean patients with alcoholic chronic pancreatitis.	GeneRIF	0.00875	129	0.0149	0.152	1	2	SPINK1

Category	ID	Name	Source	p-value	q-value Bonferroni	q-value FDR B&H	q-value FDR B&Y	Hit Count in Query List	Hit Count in Genome	Hit in Query List
Pubmed	18036312	[Relationship between autoantibodies and HLA-DQ genotypes in patients with type 1 diabetes mellitus].	GeneRIF	0.00875	129	0.0149	0.152	1	2	HLA-DQB1
Pubmed	17122078	A mutant thyroid hormone receptor alpha antagonizes peroxisome proliferator-activated receptor alpha signaling in vivo and impairs fatty acid oxidation.	GeneRIF	0.00875	129	0.0149	0.152	1	2	THRA
Pubmed	17460430	Investigation of the association between normal-tension glaucoma and single nucleotide polymorphisms in natriuretic peptide gene.	GeneRIF	0.00875	129	0.0149	0.152	1	2	NPPA
Pubmed	23721480	Trafficking defects in PAS domain mutant Kv11.1 channels: roles of reduced domain stability and altered domain-domain interactions.	Pubmed	0.00875	129	0.0149	0.152	1	2	KCNH2
Pubmed	8449404	Cloning of the mouse agouti gene predicts a secreted protein ubiquitously expressed in mice carrying the lethal yellow mutation.	Pubmed	0.00875	129	0.0149	0.152	1	2	ADA
Pubmed	18796614	Protein histidine phosphatase 1 negatively regulates CD4 T cells by inhibiting the K+ channel KCa3.1.	GeneRIF	0.00875	129	0.0149	0.152	1	2	KCNN4
Pubmed	20694145	Control of cerebellar long-term potentiation by P-Rex-family guanine-nucleotide exchange factors and phosphoinositide 3-kinase.	GeneRIF	0.00875	129	0.0149	0.152	1	2	PREX1
Pubmed	17503480	Genetic susceptibility to environmental toxicants in ALS.	GeneRIF	0.00875	129	0.0149	0.152	1	2	GSS
Pubmed	15645749	Systemic lupus erythematosus in southern Spain: a comparative clinical and genetic study between Caucasian and Gypsy patients.	GeneRIF	0.00875	129	0.0149	0.152	1	2	HLA-DQB1
Pubmed	23899556	Phosphorylation of P-Rex1 at serine 1169 participates in IGF-1R signaling in breast cancer cells.	Pubmed	0.00875	129	0.0149	0.152	1	2	PREX1
Pubmed	17260942	Paradoxical effects of substitution and deletion mutation of Arg56 on the structure and chaperone function of human alphaB-crystallin.	GeneRIF	0.00875	129	0.0149	0.152	1	2	CRYAA
Pubmed	1643309	Localization of the interleukin-3 gene to rat chromosome 10 by linkage analyses with a variable number of tandem repeats (VNTR).	Pubmed	0.00875	129	0.0149	0.152	1	2	NGFR
Pubmed	1670773	Calcium-induced intracellular cross-linking of lipocortin I by tissue transglutaminase in A431 cells. Augmentation by membrane phospholipids.	Pubmed	0.00875	129	0.0149	0.152	1	2	TGM2
Pubmed	15350494	Distribution of HLA class II (DRB1/DQB1) alleles and haplotypes among Bahraini and Lebanese Arabs.	GeneRIF	0.00875	129	0.0149	0.152	1	2	HLA-DQB1
Pubmed	15538648	The role of HFE mutations on iron metabolism in beta-thalassemia carriers.	GeneRIF	0.00875	129	0.0149	0.152	1	2	HBB
Pubmed	11483509	The p41 isoform of invariant chain is a chaperone for cathepsin L.	Pubmed	0.00875	129	0.0149	0.152	1	2	CD74
Pubmed	25200477	Co-localization of gluten consumption and HLA-DQ2 and -DQ8 genotypes, a clue to the history of celiac disease.	Pubmed	0.00875	129	0.0149	0.152	1	2	HLA-DQB1
Pubmed	11807035	Signalling between the hindbrain and paraxial tissues dictates neural crest migration pathways.	Pubmed	0.00875	129	0.0149	0.152	1	2	BMP4
Pubmed	23166301	TAK1 is essential for osteoclast differentiation and is an important modulator of cell death by apoptosis and necroptosis.	Pubmed	0.00875	129	0.0149	0.152	1	2	CTSK
Pubmed	16354200	Endothelial protein C receptor and protease-activated receptor-1 mediate induction of a wound-healing phenotype in human keratinocytes by activated protein C.	GeneRIF	0.00875	129	0.0149	0.152	1	2	PROCR
Pubmed	5345070	Studies on the mouse hemoglobin loci. 8. A fourth alpha-chain phenotype.	Pubmed	0.00875	129	0.0149	0.152	1	2	HBA2
Pubmed	22019704	BMP4 increases expression of HMGA2 in mesenchymal stem cells.	GeneRIF	0.00875	129	0.0149	0.152	1	2	BMP4
Pubmed	21501655	Tumor necrosis factor-a increases angiopoietin-like protein 2 gene expression by activating Foxo1 in 3T3-L1 adipocytes.	GeneRIF	0.00875	129	0.0149	0.152	1	2	ANGPTL2
Pubmed	19433624	Deletion of the secretory vesicle proteins IA-2 and IA-2beta disrupts circadian rhythms of cardiovascular and physical activity.	GeneRIF	0.00875	129	0.0149	0.152	1	2	HLA-DQB1
Pubmed	15097871	Differential expression of the trypsin inhibitor SPINK3 mRNA and the mouse ortholog of secretory granule protein ZG-16p mRNA in the mouse pancreas after repetitive injury.	GeneRIF	0.00875	129	0.0149	0.152	1	2	SPINK1
Pubmed	21128211	Lack of effect of bone morphogenetic protein 2 and 4 gene polymorphisms on bone density in postmenopausal Turkish women.	GeneRIF	0.00875	129	0.0149	0.152	1	2	BMP4
Pubmed	16873365	Molecular identification and physiological roles of parotid acinar cell maxi-K channels.	GeneRIF	0.00875	129	0.0149	0.152	1	2	KCNN4
Pubmed	22972301	Increased proteasome activity in human embryonic stem cells is regulated by PSMD11.	GeneRIF	0.00875	129	0.0149	0.152	1	2	FOXO4
Pubmed	19940266	Roles for endoplasmic reticulum-associated degradation and the novel endoplasmic reticulum stress response gene Derlin-3 in the ischemic heart.	GeneRIF	0.00875	129	0.0149	0.152	1	2	DERL3
Pubmed	23896968	Variations in both TG1 and TG2 isozyme-specific in situ activities and protein expressions during mouse embryonic development.	Pubmed	0.00875	129	0.0149	0.152	1	2	TGM2
Pubmed	11860449	Molecular characterization of (deltabeta)(0)/beta(0)-thalassemia and (deltabeta)(0)-thalassemia/hemoglobin E in Thai patients.	GeneRIF	0.00875	129	0.0149	0.152	1	2	HBB
Pubmed	20138312	[Outcome differences in pediatric patients with type 1 diabetes mellitus depending on their HLA-DQ genotypes].	GeneRIF	0.00875	129	0.0149	0.152	1	2	HLA-DQB1
Pubmed	18538735	The role of SPINK1 in ETS rearrangement-negative prostate cancers.	GeneRIF	0.00875	129	0.0149	0.152	1	2	SPINK1
Pubmed	20979817	[Change of matrix metalloproteinase-1 and matrix metalloproteinase-7 in serum and bronchoalveolar lavage fluid of patients with idiopathic pulmonary fibrosis and sarcoidosis].	GeneRIF	0.00875	129	0.0149	0.152	1	2	MMP7
Pubmed	21456062	Regulation of phosphatase homologue of tensin protein expression by bone morphogenetic proteins in prostate epithelial cells.	GeneRIF	0.00875	129	0.0149	0.152	1	2	BMP4
Pubmed	14500695	Distribution of the serine protease HtrA1 in normal human tissues.	Pubmed	0.00875	129	0.0149	0.152	1	2	HTRA1

Category	ID	Name	Source	p-value	q-value Bonferroni	q-value FDR B&H	q-value FDR B&Y	Hit Count in Query List	Hit Count in Genome	Hit in Query List
Pubmed	22891348	Thyroid hormone triggers the developmental loss of axonal regenerative capacity via thyroid hormone receptor a1 and krüppel-like factor 9 in Purkinje cells.	GeneRIF	0.00875	129	0.0149	0.152	1	2	THRA
Pubmed	16966600	Extreme genetic risk for type 1A diabetes.	GeneRIF	0.00875	129	0.0149	0.152	1	2	HLA-DQB1
Pubmed	10438535	The EVH2 domain of the vasodilator-stimulated phosphoprotein mediates tetramerization, F-actin binding, and actin bundle formation.	Pubmed	0.00875	129	0.0149	0.152	1	2	ACTA1
Pubmed	25745807	MASP-1 of the complement system promotes clotting via prothrombin activation.	Pubmed	0.00875	129	0.0149	0.152	1	2	MASP1
Pubmed	11472890	Comparison in localization between cystatin C and cathepsin K in osteoclasts and other cells in mouse tibia epiphysis by immunolight and immunoelectron microscopy.	Pubmed	0.00875	129	0.0149	0.152	1	2	CTSK
Pubmed	7951235	The Friedreich ataxia region: characterization of two novel genes and reduction of the critical region to 300 kb.	Pubmed	0.00875	129	0.0149	0.152	1	2	FAM189A2
Pubmed	21606508	T cell receptor signal strength in Treg and iNKT cell development demonstrated by a novel fluorescent reporter mouse.	Pubmed	0.00875	129	0.0149	0.152	1	2	HLA-DQB1
Pubmed	24270662	Gene expression profiling of the leading edge of cutaneous squamous cell carcinoma: IL-24-driven MMP-7.	Pubmed	0.00875	129	0.0149	0.152	1	2	MMP7
Pubmed	24704993	MMP-7 is upregulated by COX-2 and promotes proliferation and invasion of lung adenocarcinoma cells.	Pubmed	0.00875	129	0.0149	0.152	1	2	MMP7
Pubmed	17938215	L-Ficolin/mannose-binding lectin-associated serine protease complexes bind to group B streptococci primarily through N-acetylneuraminic acid of capsular polysaccharide and activate the complement pathway.	GeneRIF	0.00875	129	0.0149	0.152	1	2	MASP1
Pubmed	23628390	No association of the HLA-DQ alleles with myasthenia gravis in Cuban patients.	Pubmed	0.00875	129	0.0149	0.152	1	2	HLA-DQB1
Pubmed	19478349	Interplay between human leukocyte antigen genes and the microbial colonization process of the newborn intestine.	GeneRIF	0.00875	129	0.0149	0.152	1	2	HLA-DQB1
Pubmed	9099699	Cloning and expression of human carboxypeptidase Z, a novel metallocarboxypeptidase.	Pubmed	0.00875	129	0.0149	0.152	1	2	CPZ
Pubmed	11745667	The neurotrophin receptors, trkB and p75, differentially regulate motor axonal regeneration.	Pubmed	0.00875	129	0.0149	0.152	1	2	NGFR
Pubmed	9299436	Adenosine deaminase is a specific partner for the Grb2 isoform Grb3-3.	Pubmed	0.00875	129	0.0149	0.152	1	2	ADA
Pubmed	23880664	Mutual interaction of kisspeptin, estrogen and bone morphogenetic protein-4 activity in GnRH regulation by GT1-7 cells.	Pubmed	0.00875	129	0.0149	0.152	1	2	BMP4
Pubmed	19608980	Nitric oxide increases cardiac IK1 by nitrosylation of cysteine 76 of Kir2.1 channels.	GeneRIF	0.00875	129	0.0149	0.152	1	2	KCNN4
Pubmed	21677668	Identification of TROP2 (TACSTD2), an EpCAM-like molecule, as a specific marker for TGF-ß1-dependent human epidermal Langerhans cells.	GeneRIF	0.00875	129	0.0149	0.152	1	2	TACSTD2
Pubmed	24451386	Endothelial mineralocorticoid receptor activation enhances endothelial protein C receptor and decreases vascular thrombosis in mice.	Pubmed	0.00875	129	0.0149	0.152	1	2	PROCR
Pubmed	23982337	RNA interference suppression of Nogo-66 receptor prevents Nogo-66-mediated inhibition of invasion and adhesion and simultaneously increases cell apoptosis in C6 cells.	Pubmed	0.00875	129	0.0149	0.152	1	2	RTN4R
Pubmed	16148166	Major histocompatibility complex class II (HLA-DRB and -DQB) allele frequencies in Botswana: association with human immunodeficiency virus type 1 infection.	GeneRIF	0.00875	129	0.0149	0.152	1	2	HLA-DQB1
Pubmed	21603979	ZIP2 and ZIP4 mediate age-related zinc fluxes across the retinal pigment epithelium.	GeneRIF	0.00875	129	0.0149	0.152	1	2	SLC39A2
Pubmed	9861043	Thyroid hormone receptor beta-dependent expression of a potassium conductance in inner hair cells at the onset of hearing.	Pubmed	0.00875	129	0.0149	0.152	1	2	THRA
Pubmed	11086052	On the self-referential nature of naive MHC class II-restricted T cells.	Pubmed	0.00875	129	0.0149	0.152	1	2	HLA-DQB1
Pubmed	18555711	Sodium butyrate activates erythroid-specific 5-aminolevulinate synthase gene through Sp1 elements at its promoter.	GeneRIF	0.00875	129	0.0149	0.152	1	2	ALAS2
Pubmed	14764740	A spontaneous model for autoimmune myocarditis using the human MHC molecule HLA-DQ8.	Pubmed	0.00875	129	0.0149	0.152	1	2	HLA-DQB1
Pubmed	17392350	Polymorphism of immunoglobulin enhancer element HS1,2A: allele *2 associates with systemic sclerosis. Comparison with HLA-DR and DQ allele frequency.	GeneRIF	0.00875	129	0.0149	0.152	1	2	HLA-DQB1
Pubmed	20510319	Effect of human leukocyte antigen class II genes on acute-onset and slow-onset type 1 diabetes in the Japanese population.	GeneRIF	0.00875	129	0.0149	0.152	1	2	HLA-DQB1
Pubmed	22126332	Antifibrotic effects of curcumin are associated with overexpression of cathepsins K and L in bleomycin treated mice and human fibroblasts.	GeneRIF	0.00875	129	0.0149	0.152	1	2	CTSK
Pubmed	17900714	Associations between plasma natriuretic peptides and echocardiographic abnormalities in geriatric outpatients.	GeneRIF	0.00875	129	0.0149	0.152	1	2	NPPA
Pubmed	24937480	Diminished hERG K+ channel activity facilitates strong human labour contractions but is dysregulated in obese women.	Pubmed	0.00875	129	0.0149	0.152	1	2	KCNH2
Pubmed	18627303	Genetic deficiency in tissue kallikrein activity in mouse and man: effect on arteries, heart and kidney.	GeneRIF	0.00875	129	0.0149	0.152	1	2	KLK1
Pubmed	16799707	HLA class II polymorphism: protective or risk factors to breast cancer in Tunisia?	GeneRIF	0.00875	129	0.0149	0.152	1	2	HLA-DQB1
Pubmed	23740247	The MID1 E3 ligase catalyzes the polyubiquitination of Alpha4 (a4), a regulatory subunit of protein phosphatase 2A (PP2A): novel insights into MID1-mediated regulation of PP2A.	Pubmed	0.00875	129	0.0149	0.152	1	2	MID1
Pubmed	20977904	Combined bicarbonate conductance-impairing variants in CFTR and SPINK1 variants are associated with chronic pancreatitis in patients without cystic fibrosis.	GeneRIF	0.00875	129	0.0149	0.152	1	2	SPINK1

Category	ID	Name	Source	p-value	q-value Bonferroni	q-value FDR B&H	q-value FDR B&Y	Hit Count in Query List	Hit Count in Genome	Hit in Query List
Pubmed	17901852	The concentration of atrial and brain natriuretic peptide in patients with idiopathic hypertension.	GeneRIF	0.00875	129	0.0149	0.152	1	2	NPPA
Pubmed	15696102	Antigen presentation of the immunodominant T-cell epitope of the major mugwort pollen allergen, Art v 1, is associated with the expression of HLA-DRB1 *01.	GeneRIF	0.00875	129	0.0149	0.152	1	2	HLA-DQB1
Pubmed	22308705	[Distribution of HLA specificity frequencies in patients with cystic echinococcosis].	GeneRIF	0.00875	129	0.0149	0.152	1	2	HLA-DQB1
Pubmed	9719152	Glomerular deposition of mannose-binding lectin (MBL) indicates a novel mechanism of complement activation in IgA nephropathy.	Pubmed	0.00875	129	0.0149	0.152	1	2	MASP1
Pubmed	21858227	The thyroid hormone receptors modulate the skin response to retinoids.	Pubmed	0.00875	129	0.0149	0.152	1	2	THRA
Pubmed	16441489	HLA-DRB1*0826 and HLA-DQB1*0627, two novel class II alleles identified in blood stem cell donors of Caucasian origin.	GeneRIF	0.00875	129	0.0149	0.152	1	2	HLA-DQB1
Pubmed	18641458	Aquaporin-4 expression is severely reduced in human sarcoglycanopathies and dysferlinopathies.	GeneRIF	0.00875	129	0.0149	0.152	1	2	DYSF
Pubmed	20624652	IL-13 and TH2 cytokine exposure triggers matrix metalloproteinase 7-mediated Fas ligand cleavage from bronchial epithelial cells.	GeneRIF	0.00875	129	0.0149	0.152	1	2	MMP7
Pubmed	20132408	Characterization of smooth muscle differentiation of purified human skeletal muscle-derived cells.	GeneRIF	0.00875	129	0.0149	0.152	1	2	MYH11
Pubmed	21063088	Modulation of human ether a gogo related channels by CASQ2 contributes to etiology of catecholaminergic polymorphic ventricular tachycardia (CPVT).	GeneRIF	0.00875	129	0.0149	0.152	1	2	KCNH2
Pubmed	24624896	Matrilysin (MMP-7) catalytic activity regulates ß-catenin localization and signaling activation in lung epithelial cells.	Pubmed	0.00875	129	0.0149	0.152	1	2	MMP7
Pubmed	18465753	p21 activated kinase 5 activates Raf-1 and targets it to mitochondria.	GeneRIF	0.00875	129	0.0149	0.152	1	2	PAK6
Pubmed	6436017	The Eb beta gene may have acted as the donor gene in a gene conversion-like event generating the Abm 12 beta mutant.	Pubmed	0.00875	129	0.0149	0.152	1	2	HLA-DQB1
Pubmed	15613430	The orphan nuclear receptor, liver receptor homolog-1, regulates cholesterol side-chain cleavage cytochrome p450 enzyme in human granulosa cells.	GeneRIF	0.00875	129	0.0149	0.152	1	2	CYP11A1
Pubmed	18287507	Locomotor deficiencies and aberrant development of subtype-specific GABAergic interneurons caused by an unliganded thyroid hormone receptor alpha1.	GeneRIF	0.00875	129	0.0149	0.152	1	2	THRA
Pubmed	23079991	Phenotype characterization and sequence analysis of BMP2 and BMP4 variants in two Mexican families with oligodontia.	GeneRIF	0.00875	129	0.0149	0.152	1	2	BMP4
Pubmed	20333725	Molecular signaling operated by a diet-compatible mixture of oxysterols in up-regulating CD36 receptor in CD68 positive cells.	GeneRIF	0.00875	129	0.0149	0.152	1	2	CD68
Pubmed	18427198	Human leukocyte antigen-DQ alleles and haplotypes and their associations with resistance and susceptibility to HIV-1 infection.	GeneRIF	0.00875	129	0.0149	0.152	1	2	HLA-DQB1
Pubmed	23610395	Nuclear receptor corepressor (NCOR1) regulates in vivo actions of a mutated thyroid hormone receptor a.	GeneRIF	0.00875	129	0.0149	0.152	1	2	THRA
Pubmed	11747442	The role of beta chains in the control of the hemoglobin oxygen binding function: chimeric human/mouse proteins, structure, and function.	GeneRIF	0.00875	129	0.0149	0.152	1	2	HBB
Pubmed	23630275	Steroidogenic enzyme Cyp11a1 regulates Type 2 CD8+ T cell skewing in allergic lung disease.	GeneRIF	0.00875	129	0.0149	0.152	1	2	CYP11A1
Pubmed	18490777	The Mer receptor tyrosine kinase is required for the loss of B cell tolerance in the chronic graft-versus-host disease model of systemic lupus erythematosus.	GeneRIF	0.00875	129	0.0149	0.152	1	2	HLA-DQB1
Pubmed	15666831	Study of the function of proximal SF-1 binding sites on Cyp11A1 promoter.	GeneRIF	0.00875	129	0.0149	0.152	1	2	CYP11A1
Pubmed	17218410	Activating protein-1 cooperates with steroidogenic factor-1 to regulate 3',5'-cyclic adenosine 5'-monophosphate-dependent human CYP11A1 transcription in vitro and in vivo.	GeneRIF	0.00875	129	0.0149	0.152	1	2	CYP11A1
Pubmed	20570888	DC-HIL/glycoprotein Nmb promotes growth of melanoma in mice by inhibiting the activation of tumor-reactive T cells.	GeneRIF	0.00875	129	0.0149	0.152	1	2	GPNMB
Pubmed	21799929	Interleukin-6 contributes to inflammation and remodeling in a model of adenosine mediated lung injury.	GeneRIF	0.00875	129	0.0149	0.152	1	2	ADA
Pubmed	19505922	Fecal cyclooxygenase 2 plus matrix metalloproteinase 7 mRNA assays as a marker for colorectal cancer screening.	GeneRIF	0.00875	129	0.0149	0.152	1	2	MMP7
Pubmed	22558167	Experimental verification of a predicted intronic microRNA in human NGFR gene with a potential pro-apoptotic function.	GeneRIF	0.00875	129	0.0149	0.152	1	2	NGFR
Pubmed	9344615	Cleavage of beta 4 integrin by matrilysin.	Pubmed	0.00875	129	0.0149	0.152	1	2	MMP7
Pubmed	11894970	Cytotoxic T lymphocyte antigen 4 gene polymorphism confers susceptibility to type 1 diabetes in Japanese children: analysis of association with HLA genotypes and autoantibodies.	GeneRIF	0.00875	129	0.0149	0.152	1	2	HLA-DQB1
Pubmed	9252372	Identification of cytoplasmic actin as an abundant glutaminyl substrate for tissue transglutaminase in HL-60 and U937 cells undergoing apoptosis.	Pubmed	0.00875	129	0.0149	0.152	1	2	TGM2
Pubmed	24578002	In-depth comparative characterization of hemoglobin glycation in normal and diabetic bloods by LC-MSMS.	Pubmed	0.00875	129	0.0149	0.152	1	2	HBB
Pubmed	23844868	MuRF-1 and atrogin-1 protein expression and quadriceps fiber size and muscle mass in stable patients with COPD.	Pubmed	0.00875	129	0.0149	0.152	1	2	FBXO32
Pubmed	22095947	Matrix metalloproteinase-7 as a surrogate marker predicts renal Wnt/ß-catenin activity in CKD.	GeneRIF	0.00875	129	0.0149	0.152	1	2	MMP7
Pubmed	10984435	Myosin-X, a novel myosin with pleckstrin homology domains, associates with regions of dynamic actin.	Pubmed	0.00875	129	0.0149	0.152	1	2	ACTA1
Pubmed	12529175	Catalytic properties and inhibition of proline-specific dipeptidyl peptidases II, IV and VII.	GeneRIF	0.00875	129	0.0149	0.152	1	2	DPP7

Category	ID	Name	Source	p-value	q-value Bonferroni	q-value FDR B&H	q-value FDR B&Y	Hit Count in Query List	Hit Count in Genome	Hit in Query List
Pubmed	16650464	Serine proteases HTRA1 and HTRA3 are down-regulated with increasing grades of human endometrial cancer.	GeneRIF	0.00875	129	0.0149	0.152	1	2	HTRA1
Pubmed	17592398	HLA-DRB1*1101 allele may be associated with bilateral Méniére's disease in southern European population.	GeneRIF	0.00875	129	0.0149	0.152	1	2	HLA-DQB1
Pubmed	24162265	The pattern of expression and role of triiodothyronine (T3) receptors and type I 5'-deiodinase in breast carcinomas, benign breast diseases, lactational change, and normal breast epithelium.	Pubmed	0.00875	129	0.0149	0.152	1	2	THRA
Pubmed	12045257	Matrix metalloproteinase-7 is expressed by pancreatic cancer precursors and regulates acinar-to-ductal metaplasia in exocrine pancreas.	GeneRIF	0.00875	129	0.0149	0.152	1	2	MMP7
Pubmed	21192298	The expression of tumor necrosis factor-a and CD68 in high-intensity zone of lumbar intervertebral disc on magnetic resonance image in the patients with low back pain.	GeneRIF	0.00875	129	0.0149	0.152	1	2	CD68
Pubmed	24630518	Overexpression of B7-H3 in CD133+ colorectal cancer cells is associated with cancer progression and survival in human patients.	Pubmed	0.00875	129	0.0149	0.152	1	2	CD276
Pubmed	12150008	Association of HLA-DQ and HLA-DR alleles with susceptibility or resistance to HIV-1 infection among the population of Chaco Province, Argentina.	GeneRIF	0.00875	129	0.0149	0.152	1	2	HLA-DQB1
Pubmed	21678409	Interplay between membrane lipid peroxidation, transglutaminase activity, and cyclooxygenase 2 expression in the tissue adjoining to breast cancer.	GeneRIF	0.00875	129	0.0149	0.152	1	2	TGM2
Pubmed	11934390	HLA polymorphism in type 1 diabetes Tunisians.	GeneRIF	0.00875	129	0.0149	0.152	1	2	HLA-DQB1
Pubmed	20541041	Long QT syndrome with compound mutations is associated with a more severe phenotype: a Japanese multicenter study.	GeneRIF	0.00875	129	0.0149	0.152	1	2	KCNH2
Pubmed	19732768	The inactivation of the sortilin gene leads to a partial disruption of prosaposin trafficking to the lysosomes.	GeneRIF	0.00875	129	0.0149	0.152	1	2	PSAP
Pubmed	22049084	High temperature requirement factor A1 (HTRA1) gene regulates angiogenesis through transforming growth factor-ß family member growth differentiation factor 6.	GeneRIF	0.00875	129	0.0149	0.152	1	2	HTRA1
Pubmed	12789270	Neurotrophin-induced melanoma cell migration is mediated through the actin-bundling protein fascin.	Pubmed	0.00875	129	0.0149	0.152	1	2	NGFR
Pubmed	12738725	Differential splicing of KLK5 and KLK7 in epithelial ovarian cancer produces novel variants with potential as cancer biomarkers.	GeneRIF	0.00875	129	0.0149	0.152	1	2	KLK7
Pubmed	16286510	Growth factor-induced shedding of syndecan-1 confers glypican-1 dependence on mitogenic responses of cancer cells.	GeneRIF	0.00875	129	0.0149	0.152	1	2	MMP7
Pubmed	15749233	The impact of cystic fibrosis and PSTI/SPINK1 gene mutations on susceptibility to chronic pancreatitis.	GeneRIF	0.00875	129	0.0149	0.152	1	2	SPINK1
Pubmed	1783409	Localization of human glandular kallikrein-1 gene to chromosome 19q13.3-13.4 by in situ hybridization.	Pubmed	0.00875	129	0.0149	0.152	1	2	KLK1
Pubmed	20858521	Genetic and diabetic auto-antibody markers in Saudi children with type 1 diabetes.	GeneRIF	0.00875	129	0.0149	0.152	1	2	HLA-DQB1
Pubmed	15993720	Association of HLA-DR and -DQ genes with Graves disease in Koreans.	GeneRIF	0.00875	129	0.0149	0.152	1	2	HLA-DQB1
Pubmed	18938112	Chemokines and common variable immunodeficiency; possible contribution of the fractalkine system (CX3CL1/CX3CR1) to chronic inflammation.	GeneRIF	0.00875	129	0.0149	0.152	1	2	CX3CL1
Pubmed	12974555	Incidence rate of type 1 diabetes in Santiago (Chile) by HLA-DQA1 and DQB1 genotypes.	GeneRIF	0.00875	129	0.0149	0.152	1	2	HLA-DQB1
Pubmed	22948049	Tumor-associated macrophages predict inferior outcomes in classic Hodgkin lymphoma: a correlative study from the E2496 Intergroup trial.	GeneRIF	0.00875	129	0.0149	0.152	1	2	CD68
Pubmed	18803847	Transcriptional regulation of mouse alpha A-crystallin gene in a 148kb Cryaa BAC and its derivates.	GeneRIF	0.00875	129	0.0149	0.152	1	2	CRYAA
Pubmed	7816144	F-actin binding site masked by the intramolecular association of vinculin head and tail domains.	Pubmed	0.00875	129	0.0149	0.152	1	2	ACTA1
Pubmed	21828287	Role of ubiquitylation and USP8-dependent deubiquitylation in the endocytosis and lysosomal targeting of plasma membrane KCa3.1.	Pubmed	0.00875	129	0.0149	0.152	1	2	KCNN4
Pubmed	21307265	p75NTR regulates Abeta deposition by increasing Abeta production but inhibiting Abeta aggregation with its extracellular domain.	GeneRIF	0.00875	129	0.0149	0.152	1	2	NGFR
Pubmed	16863914	Class II transactivator-mediated regulation of major histocompatibility complex class II antigen expression is important for hematopoietic progenitor cell suppression by chemokines and iron-binding proteins.	GeneRIF	0.00875	129	0.0149	0.152	1	2	HLA-DQB1
Pubmed	20570964	The DNA damage repair protein Ku70 interacts with FOXO4 to coordinate a conserved cellular stress response.	GeneRIF	0.00875	129	0.0149	0.152	1	2	FOXO4
Pubmed	12402336	DHPLC analysis of potassium ion channel genes in congenital long QT syndrome.	GeneRIF	0.00875	129	0.0149	0.152	1	2	KCNH2
Pubmed	20062572	Association of HLA-DR and -DQ Genes with Familial Moyamoya Disease in Koreans.	GeneRIF	0.00875	129	0.0149	0.152	1	2	HLA-DQB1
Pubmed	15476152	Relationship of HLA-DQ8 and severity of celiac disease: comparison of New York and Parisian cohorts.	GeneRIF	0.00875	129	0.0149	0.152	1	2	HLA-DQB1
Pubmed	22916220	aA crystallin may protect against geographic atrophy-meta-analysis of cataract vs. cataract surgery for geographic atrophy and experimental studies.	GeneRIF	0.00875	129	0.0149	0.152	1	2	CRYAA
Pubmed	21750038	A novel role for MuSK and non-canonical Wnt signaling during segmental neural crest cell migration.	Pubmed	0.00875	129	0.0149	0.152	1	2	NGFR
Pubmed	17110602	Angiopoietin-like protein3 regulates plasma HDL cholesterol through suppression of endothelial lipase.	GeneRIF	0.00875	129	0.0149	0.152	1	2	LIPG

Category	ID	Name	Source	p-value	q-value Bonferroni	q-value FDR B&H	q-value FDR B&Y	Hit Count in Query List	Hit Count in Genome	Hit in Query List
Pubmed	12571168	Serum anti-Müllerian hormone is more strongly related to ovarian follicular status than serum inhibin B, estradiol, FSH and LH on day 3.	GeneRIF	0.00875	129	0.0149	0.152	1	2	INHBB
Pubmed	23076342	ETS variant 1 regulates matrix metalloproteinase-7 transcription in LNCaP prostate cancer cells.	Pubmed	0.00875	129	0.0149	0.152	1	2	MMP7
Pubmed	18554163	The polymorphism of HLA-DR and -DQ allelic genes associated with intrahepatic cholestasis of pregnancy.	GeneRIF	0.00875	129	0.0149	0.152	1	2	HLA-DQB1
Pubmed	23953525	Identification of the effects of aging-related gene-matrix metalloproteinase on allograft outcomes in kidney transplantation.	Pubmed	0.00875	129	0.0149	0.152	1	2	MMP7
Pubmed	17667983	Advanced glycation endproducts induce podocyte apoptosis by activation of the FOXO4 transcription factor.	GeneRIF	0.00875	129	0.0149	0.152	1	2	FOXO4
Pubmed	16409268	Association of HLA-DQA1 and DQB1 genes with vitiligo in Chinese Hans.	GeneRIF	0.00875	129	0.0149	0.152	1	2	HLA-DQB1
Pubmed	19173304	Matrilysin (MMP-7) cleaves C-type lectin domain family 3 member A (CLEC3A) on tumor cell surface and modulates its cell adhesion activity.	GeneRIF	0.00875	129	0.0149	0.152	1	2	MMP7
Pubmed	12388540	Corepressor SMRT functions as a coactivator for thyroid hormone receptor T3Ralpha from a negative hormone response element.	GeneRIF	0.00875	129	0.0149	0.152	1	2	THRA
Pubmed	24570486	mTORC1 promotes denervation-induced muscle atrophy through a mechanism involving the activation of FoxO and E3 ubiquitin ligases.	Pubmed	0.00875	129	0.0149	0.152	1	2	FBXO32
Pubmed	20378727	17Beta-estradiol inhibits IL-8 in cystic fibrosis by up-regulating secretory leucoprotease inhibitor.	GeneRIF	0.00875	129	0.0149	0.152	1	2	SLPI
Pubmed	18073310	Serological evidence of thyroid autoimmunity among schoolchildren in two different socioeconomic environments.	GeneRIF	0.00875	129	0.0149	0.152	1	2	HLA-DQB1
Pubmed	21933672	Localization and phenotype-specific expression of ryanodine calcium release channels in C57BL6 and DBA/2J mouse strains.	GeneRIF	0.00875	129	0.0149	0.152	1	2	GPNMB
Pubmed	21164105	Tissue kallikrein is essential for invasive capacity of circulating proangiogenic cells.	GeneRIF	0.00875	129	0.0149	0.152	1	2	KLK1
Pubmed	12857743	Differential and regulated binding of cAMP-dependent protein kinase and protein kinase C isoenzymes to gravin in human model neurons: Evidence that gravin provides a dynamic platform for the localization for kinases during neuronal development.	GeneRIF	0.00875	129	0.0149	0.152	1	2	AKAP12
Pubmed	25266722	A direct interaction between the sigma-1 receptor and the hERG voltage-gated K+ channel revealed by atomic force microscopy and homogeneous tim resolved fluorescence (HTRF®).	Pubmed	0.00875	129	0.0149	0.152	1	2	KCNH2
Pubmed	21689370	Aminoterminal natriuretic peptide as a determinant of PAI-1 levels in a sample of the adult male Italian population.	GeneRIF	0.00875	129	0.0149	0.152	1	2	NPPA
Pubmed	1544421	Binding sites involved in the interaction of actin with the N-terminal region of dystrophin.	Pubmed	0.00875	129	0.0149	0.152	1	2	ACTA1
Pubmed	15611136	AlphaPIX associates with calpain 4, the small subunit of calpain, and has a dual role in integrin-mediated cell spreading.	GeneRIF	0.00875	129	0.0149	0.152	1	2	ARHGEF6
Pubmed	20203298	Cholate-containing high-fat diet induces the formation of multinucleated giant cells in atherosclerotic plaques of apolipoprotein E-/- mice.	GeneRIF	0.00875	129	0.0149	0.152	1	2	CTSK
Pubmed	17608931	Absence of glaucoma in DBA/2J mice homozygous for wild-type versions of Gpnmb and Tyrp1.	GeneRIF	0.00875	129	0.0149	0.152	1	2	GPNMB
Pubmed	23863940	Ptpn11 deletion in a novel progenitor causes metachondromatosis by inducing hedgehog signalling.	GeneRIF	0.00875	129	0.0149	0.152	1	2	CTSK
Pubmed	20128681	Hypoxia and kinase activity regulate lung epithelial cell glutathione.	GeneRIF	0.00875	129	0.0149	0.152	1	2	GSS
Pubmed	12750454	Compensatory role of thyroid hormone receptor (TR) alpha 1 in resistance to thyroid hormone: study in mice with a targeted mutation in the TR beta gene and deficient in TR alpha 1.	Pubmed	0.00875	129	0.0149	0.152	1	2	THRA
Pubmed	17372201	Saposin B is the dominant saposin that facilitates lipid binding to human CD1d molecules.	GeneRIF	0.00875	129	0.0149	0.152	1	2	PSAP
Pubmed	11724418	Correlations between the incidence of childhood-onset type I diabetes in Europe and HLA genotypes.	GeneRIF	0.00875	129	0.0149	0.152	1	2	HLA-DQB1
Pubmed	19290000	Prediction of duration and success rate of unrelated hematopoietic stem cell donor searches based on the patient's HLA-DRB1 allele and DRB1-DQB1 haplotype frequencies.	GeneRIF	0.00875	129	0.0149	0.152	1	2	HLA-DQB1
Pubmed	9164977	Up-regulation of cytokine mRNA, adhesion molecule proteins, and MHC class II proteins in salivary glands of TGF-beta1 knockout mice: MHC class II is a factor in the pathogenesis of TGF-beta1 knockout mice.	Pubmed	0.00875	129	0.0149	0.152	1	2	HLA-DQB1
Pubmed	15845476	Helicobacter pylori binds to CD74 on gastric epithelial cells and stimulates interleukin-8 production.	GeneRIF	0.00875	129	0.0149	0.152	1	2	CD74
Pubmed	23056244	Overexpression of HTRA1 leads to down-regulation of fibronectin and functional changes in RF/6A cells and HUVECs.	GeneRIF	0.00875	129	0.0149	0.152	1	2	HTRA1
Pubmed	8188281	Localization of the delta-opioid receptor gene to mouse chromosome 4 by linkage analysis.	Pubmed	0.00875	129	0.0149	0.152	1	2	NPPA
Pubmed	16893414	Does the p75 neurotrophin receptor mediate Abeta-induced toxicity in Alzheimer's disease?	GeneRIF	0.00875	129	0.0149	0.152	1	2	NGFR
Pubmed	18995834	A role for mammalian Sin3 in permanent gene silencing.	Pubmed	0.00875	129	0.0149	0.152	1	2	KDM5A
Pubmed	19625650	SUMOylation of tissue transglutaminase as link between oxidative stress and inflammation.	GeneRIF	0.00875	129	0.0149	0.152	1	2	TGM2
Pubmed	8414517	Characterization of the retinoblastoma binding proteins RBP1 and RBP2.	Pubmed	0.00875	129	0.0149	0.152	1	2	KDM5A
Pubmed	9680361	Effect of steroid hormones and retinoids on the formation of capillary-like tubular structures of human microvascular endothelial cells in fibrin matrices is related to urokinase expression.	Pubmed	0.00875	129	0.0149	0.152	1	2	THRA

Category	ID	Name	Source	p-value	q-value Bonferroni	q-value FDR B&H	q-value FDR B&Y	Hit Count in Query List	Hit Count in Genome	Hit in Query List
Pubmed	8343954	The chondroitin sulfate form of invariant chain can enhance stimulation of T cell responses through interaction with CD44.	Pubmed	0.00875	129	0.0149	0.152	1	2	CD74
Pubmed	18490773	Tissue transglutaminase activation modulates inflammation in cystic fibrosis via PPARgamma down-regulation.	GeneRIF	0.00875	129	0.0149	0.152	1	2	TGM2
Pubmed	22320325	Overexpression of the DNA sensor proteins, absent in melanoma 2 and interferon-inducible 16, contributes to tumorigenesis of oral squamous cell carcinoma with p53 inactivation.	GeneRIF	0.00875	129	0.0149	0.152	1	2	IFI16
Pubmed	18345414	Prevalence of HLA DQB1*0602 allele in patients with migraine.	GeneRIF	0.00875	129	0.0149	0.152	1	2	HLA-DQB1
Pubmed	24134754	Childhood-onset hereditary pancreatitis with mutations in the CT gene and SPINK1 gene.	Pubmed	0.00875	129	0.0149	0.152	1	2	SPINK1
Pubmed	11555635	Characterization of the TBX5 binding site and analysis of mutations that cause Holt-Oram syndrome.	Pubmed	0.00875	129	0.0149	0.152	1	2	NPPA
Pubmed	22874102	Quantitative DNA methylation analysis of genes coding for kallikrein-related peptidases 6 and 10 as biomarkers for prostate cancer.	GeneRIF	0.00875	129	0.0149	0.152	1	2	KLK10
Pubmed	11472891	Characterization of human bone morphogenetic protein (BMP)-4 and -7 gene promoters: activation of BMP promoters by Gli, a sonic hedgehog mediator.	Pubmed	0.00875	129	0.0149	0.152	1	2	BMP4
Pubmed	19065273	Sequence variants in HTRA1 and LOC387715/ARMS2 and phenotype and response to photodynamic therapy in neovascular age-related macular degeneration in populations from Israel.	GeneRIF	0.00875	129	0.0149	0.152	1	2	HTRA1
Pubmed	15466465	Thyroxine-thyroid hormone receptor interactions.	Pubmed	0.00875	129	0.0149	0.152	1	2	THRA
Pubmed	19553474	{alpha}-synuclein and its A30P mutant affect actin cytoskeletal structure and dynamics.	GeneRIF	0.00875	129	0.0149	0.152	1	2	ACTA1
Pubmed	14688093	Delayed clearance of Ehrlichia chaffeensis infection in CD4+ T-cell knockout mice.	Pubmed	0.00875	129	0.0149	0.152	1	2	HLA-DQB1
Pubmed	21194254	First report on the co-inheritance of beta-globin IVS-I-5 (G-->C) thalassemia with delta globin CD12 {Asn-->Lys (AAT-->AAA)}HbA2-NYU in Iran.	GeneRIF	0.00875	129	0.0149	0.152	1	2	HBB
Pubmed	20108119	Incidence, prevalence, etiology, and prognosis of first-time chronic pancreatitis in young patients: a nationwide cohort study.	GeneRIF	0.00875	129	0.0149	0.152	1	2	SPINK1
Pubmed	20939879	SERPINB5 and AKAP12 - expression and promoter methylation of metastasis suppressor genes in pancreatic ductal adenocarcinoma.	GeneRIF	0.00875	129	0.0149	0.152	1	2	AKAP12
Pubmed	15382236	Relationship between chaperone activity and oligomeric size of recombinant human alphaA- and alphaB-crystallin: a tryptic digestion study.	GeneRIF	0.00875	129	0.0149	0.152	1	2	CRYAA
Pubmed	11241215	Identification of the ligand-binding site of the BMP type IA receptor for BMP-4.	Pubmed	0.00875	129	0.0149	0.152	1	2	BMP4
Pubmed	8914602	Molecular genetic characterization and comparative mapping of the human PCP4 gene.	Pubmed	0.00875	129	0.0149	0.152	1	2	PCP4
Pubmed	19619312	AlphaA-crystallin R49Cneo mutation influences the architecture of lens fiber cell membranes and causes posterior and nuclear cataracts in mice.	GeneRIF	0.00875	129	0.0149	0.152	1	2	CRYAA
Pubmed	12054662	Esterification of the propionate groups promotes alpha/beta hemoglobin chain homogeneity of CN-hemin binding.	GeneRIF	0.00875	129	0.0149	0.152	1	2	HBB
Pubmed	17049585	Endothelial protein C receptor plasma levels increase in chronic liver disease, while thrombomodulin plasma levels increase only in hepatocellular carcinoma.	GeneRIF	0.00875	129	0.0149	0.152	1	2	PROCR
Pubmed	12124219	Ventricular expression of natriuretic peptides in Npr1(-/-) mice with cardiac hypertrophy and fibrosis.	Pubmed	0.00875	129	0.0149	0.152	1	2	NPPA
Pubmed	16757564	GPR56, an atypical G protein-coupled receptor, binds tissue transglutaminase, TG2, and inhibits melanoma tumor growth and metastasis.	GeneRIF	0.00875	129	0.0149	0.152	1	2	TGM2
Pubmed	22974215	MMP7 is a target of the tumour-associated antigen EpCAM.	GeneRIF	0.00875	129	0.0149	0.152	1	2	MMP7
Pubmed	18359858	Substrate specificity of human kallikreins 1 and 6 determined by phage display.	GeneRIF	0.00875	129	0.0149	0.152	1	2	KLK1
Pubmed	1973687	Molecular cloning and expression of murine guanylate cyclase/atrial natriuretic factor receptor cDNA.	Pubmed	0.00875	129	0.0149	0.152	1	2	NPPA
Pubmed	23389822	Evolution of crystallins for a role in the vertebrate eye lens.	Pubmed	0.00875	129	0.0149	0.152	1	2	CRYAA
Pubmed	18842989	The MIF receptor CD74 in diabetic podocyte injury.	GeneRIF	0.00875	129	0.0149	0.152	1	2	CD74
Pubmed	22910694	A review of B7-H3 and B7-H4 immune molecules and their role in ovarian cancer.	GeneRIF	0.00875	129	0.0149	0.152	1	2	CD276
Pubmed	3023046	A 5' duplication of the alpha-cardiac actin gene in BALB/c mice is associated with abnormal levels of alpha-cardiac and alpha-skeletal actin mRNAs in adult cardiac tissue.	Pubmed	0.00875	129	0.0149	0.152	1	2	ACTA1
Pubmed	19937591	Genotype and phenotype correlations in diabetic patients in Uruguay.	GeneRIF	0.00875	129	0.0149	0.152	1	2	HLA-DQB1
Pubmed	15781459	The FOXO3a transcription factor regulates cardiac myocyte size downstream of AKT signaling.	GeneRIF	0.00875	129	0.0149	0.152	1	2	FBXO32
Pubmed	22738435	Identification and characterization of two novel and differentially expressed isoforms of human a2- and a1-globin genes.	GeneRIF	0.00875	129	0.0149	0.152	1	2	HBA2
Pubmed	7665234	Cloning of the gene encoding Trop-2, a cell-surface glycoprotein expressed by human carcinomas.	Pubmed	0.00875	129	0.0149	0.152	1	2	TACSTD2
Pubmed	17904861	Photic injury promotes cleavage of p75NTR by TACE and nuclear trafficking of the p75 intracellular domain.	GeneRIF	0.00875	129	0.0149	0.152	1	2	NGFR
Pubmed	23271024	Genetic features of thyroid hormone receptors.	Pubmed	0.00875	129	0.0149	0.152	1	2	THRA
Pubmed	24257613	Adenosine deaminase acts as a natural antagonist for dipeptidyl peptidase 4-mediated entry of the Middle East respiratory syndrome coronavirus.	Pubmed	0.00875	129	0.0149	0.152	1	2	ADA
Pubmed	21680736	Sig1R protein regulates hERG channel expression through a post-translational mechanism in leukemic cells.	GeneRIF	0.00875	129	0.0149	0.152	1	2	KCNH2

Category	ID	Name	Source	p-value	q-value Bonferroni	q-value FDR B&H	q-value FDR B&Y	Hit Count in Query List	Hit Count in Genome	Hit in Query List
Pubmed	10395662	Activation of the extracellular signal-related kinase/mitogen-activated protein kinase pathway discriminates CD4 versus CD8 lineage commitment in the thymus.	Pubmed	0.00875	129	0.0149	0.152	1	2	HLA-DQB1
Pubmed	9500516	Salmonella infections in the absence of the major histocompatibility complex II.	Pubmed	0.00875	129	0.0149	0.152	1	2	HLA-DQB1
Pubmed	12479974	Biosynthesis and processing of pro CCK: recent progress and future challenges.	Pubmed	0.00875	129	0.0149	0.152	1	2	CPE
Pubmed	18296082	Characterisation of fractalkine/CX3CL1 and fractalkine receptor (CX3CR1) expression in abdominal aortic aneurysm disease.	GeneRIF	0.00875	129	0.0149	0.152	1	2	CX3CL1
Pubmed	22820375	Modulation of glutamine metabolism by the PI(3)K-PKB-FOXO network regulates autophagy.	Pubmed	0.00875	129	0.0149	0.152	1	2	FOXO4
Pubmed	16297190	Clinical and genetic heterogeneity of erythrokeratoderma variabilis.	GeneRIF	0.00875	129	0.0149	0.152	1	2	GJB3
Pubmed	15476181	[Beta-thalassemia mutations and single nucleotide polymorphism at -158 of Ggamma-globin gene associated with altered levels of Hb F in beta-thalassemia heterozygotes].	GeneRIF	0.00875	129	0.0149	0.152	1	2	HBB
Pubmed	19380617	Genetic deficit of SK3 and IK1 channels disrupts the endothelium-derived hyperpolarizing factor vasodilator pathway and causes hypertension.	GeneRIF	0.00875	129	0.0149	0.152	1	2	KCNN4
Pubmed	12692530	The DCX-domain tandems of doublecortin and doublecortin-like kinase.	GeneRIF	0.00875	129	0.0149	0.152	1	2	DCLK1
Pubmed	16758344	Luteal-phase inhibin A and follicular-phase inhibin B levels are not characteristic of patients with an elevated LH-to-FSH ratio.	GeneRIF	0.00875	129	0.0149	0.152	1	2	INHBB
Pubmed	2936753	Ultrastructure of the intact skeleton of the human erythrocyte membrane.	Pubmed	0.00875	129	0.0149	0.152	1	2	ACTA1
Pubmed	18596037	Combinatorial signals of activin/nodal and bone morphogenic protein regulate the early lineage segregation of human embryonic stem cells.	GeneRIF	0.00875	129	0.0149	0.152	1	2	BMP4
Pubmed	24956133	Structural features of the Nogo receptor signaling complexes at the neuron/myelin interface.	Pubmed	0.00875	129	0.0149	0.152	1	2	RTN4R
Pubmed	9725218	Maturation of B cell precursors is impaired in thymic-deprived nude and old mice.	Pubmed	0.00875	129	0.0149	0.152	1	2	HLA-DQB1
Pubmed	18713009	Expression of tissue and plasma kallikreins and kinin B1 and B2 receptors in lung cancer.	GeneRIF	0.00875	129	0.0149	0.152	1	2	KLK1
Pubmed	7751707	Class II major histocompatibility complex-deficient mice initially control an infection with Leishmania major but succumb to the disease.	Pubmed	0.00875	129	0.0149	0.152	1	2	HLA-DQB1
Pubmed	24218563	TREM2 governs Kupffer cell activation and explains belr1 genetic resistance to malaria liver stage infection.	Pubmed	0.00875	129	0.0149	0.152	1	2	HLA-DQB1
Pubmed	16122729	Noggin antagonism of BMP4 signaling controls development of the axial skeleton in the mouse.	GeneRIF	0.00875	129	0.0149	0.152	1	2	BMP4
Pubmed	20598923	Alpha-thalassemia is associated with a decreased occurrence and a delayed age-at-onset of albuminuria in sickle cell anemia patients.	GeneRIF	0.00875	129	0.0149	0.152	1	2	HBB
Pubmed	17628551	Increased intrafollicular androgen levels affect human granulosa cell secretion of anti-Müllerian hormone and inhibin-B.	GeneRIF	0.00875	129	0.0149	0.152	1	2	INHBB
Pubmed	12039959	Thyroid hormone receptor alpha 1 regulates expression of the Na+/H+ exchanger (NHE1).	GeneRIF	0.00875	129	0.0149	0.152	1	2	THRA
Pubmed	15786423	Residual insulin secretion at diagnosis of type 1 diabetes is independently associated with both, age of onset and HLA genotype.	GeneRIF	0.00875	129	0.0149	0.152	1	2	HLA-DQB1
Pubmed	25220258	Carboxypeptidase E is a novel modulator of RANKL-induced osteoclast differentiation.	Pubmed	0.00875	129	0.0149	0.152	1	2	CPE
Pubmed	11929591	The distribution of DQ genes in the Saharawi population provides only a partial explanation for the high celiac disease prevalence.	GeneRIF	0.00875	129	0.0149	0.152	1	2	HLA-DQB1
Pubmed	22535953	Human high temperature requirement serine protease A1 (HTRA1) degrades tau protein aggregates.	GeneRIF	0.00875	129	0.0149	0.152	1	2	HTRA1
Pubmed	17174123	Dendritic cells regulate exposure of MHC class II at their plasma membrane by oligoubiquitination.	Pubmed	0.00875	129	0.0149	0.152	1	2	HLA-DQB1
Pubmed	25222045	Characterization of two unique a-globin gene cluster deletions causing a-thalassemia in Israeli Arabs.	Pubmed	0.00875	129	0.0149	0.152	1	2	HBA2
Pubmed	13129928	Involvement of napsin A in the C- and N-terminal processing of surfactant protein B in type-II pneumocytes of the human lung.	GeneRIF	0.00875	129	0.0149	0.152	1	2	NAPSA
Pubmed	8676513	Infection of class II-deficient mice by the DA strain of Theiler's virus.	Pubmed	0.00875	129	0.0149	0.152	1	2	HLA-DQB1
Pubmed	18436581	Thrombin-induced expression of endothelial CX3CL1 potentiates monocyte CCL2 production and transendothelial migration.	GeneRIF	0.00875	129	0.0149	0.152	1	2	CX3CL1
Pubmed	12805224	The thyroid hormone receptor antagonizes CREB-mediated transcription.	Pubmed	0.00875	129	0.0149	0.152	1	2	THRA
Pubmed	22007938	Higher intratumoral expression of CD1a, tryptase, and CD68 in a follicular variant of papillary thyroid carcinoma compared to adenomas: correlation with clinical and pathological parameters.	GeneRIF	0.00875	129	0.0149	0.152	1	2	CD68
Pubmed	24599937	Tissue kallikrein deficiency, insulin resistance, and diabetes in mouse and man.	Pubmed	0.00875	129	0.0149	0.152	1	2	KLK1
Pubmed	18715618	Correlation between beta-catenin widespread nuclear expression and matrix metalloproteinase-7 overexpression in sporadic desmoid tumors.	GeneRIF	0.00875	129	0.0149	0.152	1	2	MMP7
Pubmed	24013228	BMP4/Thrombospondin-1 loop paracrinically inhibits tumor angiogenesis and suppresses the growth of solid tumors.	Pubmed	0.00875	129	0.0149	0.152	1	2	BMP4
Pubmed	23232839	Endothelial dysfunction in diabetes and hypertension: cross talk in RAS, BMP4, and ROS-dependent COX-2-derived prostanoids.	GeneRIF	0.00875	129	0.0149	0.152	1	2	BMP4
Pubmed	11347740	Concordance for type 1 diabetes in identical twins is affected by insulin genotype.	GeneRIF	0.00875	129	0.0149	0.152	1	2	HLA-DQB1
Pubmed	24480136	THRA and DIO2 mutations are unlikely to be a common cause of increased bone mineral density in euthyroid post-menopausal women.	Pubmed	0.00875	129	0.0149	0.152	1	2	THRA
Pubmed	7407925	The structure of a human alpha-globin pseudogene and its relationship to alpha-globin gene duplication.	Pubmed	0.00875	129	0.0149	0.152	1	2	HBA2

Category	ID	Name	Source	p-value	q-value Bonferroni	q-value FDR B&H	q-value FDR B&Y	Hit Count in Query List	Hit Count in Genome	Hit in Query List
Pubmed	22465406	aA-crystallin in the pathogenesis and intervention of experimental murine corneal neovascularization.	GeneRIF	0.00875	129	0.0149	0.152	1	2	CRYAA
Pubmed	15972644	Helicobacter pylori-induced IL-8 production by gastric epithelial cells up-regulates CD74 expression.	GeneRIF	0.00875	129	0.0149	0.152	1	2	CD74
Pubmed	21346800	Development of axon-target specificity of ponto-cerebellar afferents.	Pubmed	0.00875	129	0.0149	0.152	1	2	BMP4
Pubmed	12209609	Risk of invasive cervical cancer associated with polymorphic HLA DR/DQ haplotypes.	GeneRIF	0.00875	129	0.0149	0.152	1	2	HLA-DQB1
Pubmed	19066423	Multi-organ iron overload in an African-American man with ALAS2 R452S and SLC40A1 R561G.	GeneRIF	0.00875	129	0.0149	0.152	1	2	ALAS2
Pubmed	22242915	The guanine-nucleotide-exchange factor P-Rex1 is activated by protein phosphatase 1a.	GeneRIF	0.00875	129	0.0149	0.152	1	2	PREX1
Pubmed	18599551	Stimulation of N-terminal truncated isoform of androgen receptor stabilizes human ether-á-go-go-related gene-encoded potassium channel protein via activation of extracellular signal regulated kinase 1/2.	GeneRIF	0.00875	129	0.0149	0.152	1	2	KCNH2
Pubmed	12239155	Role of Cbfb in hematopoiesis and perturbations resulting from expression of the leukemogenic fusion gene Cbfb-MYH11.	GeneRIF	0.00875	129	0.0149	0.152	1	2	MYH11
Pubmed	17301827	Different HLA class II associations in ulcerative colitis patients with and without primary sclerosing cholangitis.	GeneRIF	0.00875	129	0.0149	0.152	1	2	HLA-DQB1
Pubmed	15116308	HLA-DRB1 and -DQB1 alleles and haplotypes in Zambian couples and their associations with heterosexual transmission of HIV type 1.	GeneRIF	0.00875	129	0.0149	0.152	1	2	HLA-DQB1
Pubmed	19286669	Attenuated muscle regeneration is a key factor in dysferlin-deficient muscular dystrophy.	GeneRIF	0.00875	129	0.0149	0.152	1	2	DYSF
Pubmed	24327965	In vivo analysis of the contribution of bone resorption to the control of glucose metabolism in mice.	Pubmed	0.00875	129	0.0149	0.152	1	2	CTSK
Pubmed	15664787	Does genotype mask the relationship between psychological factors and immune function?	GeneRIF	0.00875	129	0.0149	0.152	1	2	HLA-DQB1
Pubmed	17038551	Estrogen decrease in tight junctional resistance involves matrix-metalloproteinase-7-mediated remodeling of occludin.	GeneRIF	0.00875	129	0.0149	0.152	1	2	MMP7
Pubmed	11776160	[Study on human leucocyte antigen-DQ region gene polymorphism in cases of habitual abortion with anticardiolipin antibody].	GeneRIF	0.00875	129	0.0149	0.152	1	2	HLA-DQB1
Pubmed	24130874	Thioredoxin is involved in endothelial cell extracellular transglutaminase 2 activation mediated by celiac disease patient IgA.	Pubmed	0.00875	129	0.0149	0.152	1	2	TGM2
Pubmed	21269755	Adenosine deaminase activity during menses, follicular and luteal phases of the menstrual cycle.	GeneRIF	0.00875	129	0.0149	0.152	1	2	ADA
Pubmed	22191848	TGFB3 and BMP4 polymorphism are associated with isolated tooth agenesis.	GeneRIF	0.00875	129	0.0149	0.152	1	2	BMP4
Pubmed	25326709	Effect of atorvastatin on the expression of gamma-glutamyl transferase in aortic atherosclerotic plaques of apolipoprotein E-knockout mice.	Pubmed	0.00875	129	0.0149	0.152	1	2	GGT1
Pubmed	19409876	ADP-ribosylation factor like 7 (ARL7) interacts with alpha-tubulin and modulates intracellular vesicular transport.	GeneRIF	0.00875	129	0.0149	0.152	1	2	ARL4C
Pubmed	21320870	Cerebral small-vessel disease protein HTRA1 controls the amount of TGF-ß1 via cleavage of proTGF-ß1.	GeneRIF	0.00875	129	0.0149	0.152	1	2	HTRA1
Pubmed	17618464	Evidence for expansion-based temporal BMP4/NOGGIN interactions in specifying periodontium morphogenesis.	GeneRIF	0.00875	129	0.0149	0.152	1	2	BMP4
Pubmed	10491393	Drosophila APC2 is a cytoskeletally-associated protein that regulates wingless signaling in the embryonic epidermis.	Pubmed	0.00875	129	0.0149	0.152	1	2	ACTA1
Pubmed	17227950	Enhanced CXCL1 production and angiogenesis in adenosine-mediated lung disease.	GeneRIF	0.00875	129	0.0149	0.152	1	2	ADA
Pubmed	25201980	Aberrant calcium signaling by transglutaminase-mediated posttranslational modification of inositol 1,4,5-trisphosphate receptors.	Pubmed	0.00875	129	0.0149	0.152	1	2	TGM2
Pubmed	12835080	HLA class II allele frequencies in the Lebanese population.	GeneRIF	0.00875	129	0.0149	0.152	1	2	HLA-DQB1
Pubmed	11701614	Interaction between PEVK-titin and actin filaments: origin of a viscous force component in cardiac myofibrils.	Pubmed	0.00875	129	0.0149	0.152	1	2	ACTA1
Pubmed	23807291	An ex vivo study on immunohistochemical localization of MMP-7 and MMP-9 in temporomandibular joint discs with internal derangement.	Pubmed	0.00875	129	0.0149	0.152	1	2	MMP7
Pubmed	16219686	Interaction between secretogranin III and carboxypeptidase E facilitates prohormone sorting within secretory granules.	GeneRIF	0.00875	129	0.0149	0.152	1	2	CPE
Pubmed	20372795	MMP-7 and SGCE as distinctive molecular factors in sporadic colorectal cancers from the mutator phenotype pathway.	GeneRIF	0.00875	129	0.0149	0.152	1	2	MMP7
Pubmed	15476187	[Analysis of HLA-DRB1,DQB1 allele polymorphism in the Kunming Yi nationality population].	GeneRIF	0.00875	129	0.0149	0.152	1	2	HLA-DQB1
Pubmed	23152542	The C-terminus of CBFß-SMMHC is required to induce embryonic hematopoietic defects and leukemogenesis.	GeneRIF	0.00875	129	0.0149	0.152	1	2	MYH11
Pubmed	16470178	Delayed cutaneous wound healing in mice lacking solute carrier 11a1 (formerly Nramp1): correlation with decreased expression of secretory leukocyte protease inhibitor.	GeneRIF	0.00875	129	0.0149	0.152	1	2	SLPI
Pubmed	12125082	Fractalkine and fractalkine receptors in human neurons and glial cells.	GeneRIF	0.00875	129	0.0149	0.152	1	2	CX3CL1
Pubmed	15336778	HLA alleles, IFN-gamma responses to HPV-11 E6, and disease severity in patients with recurrent respiratory papillomatosis.	GeneRIF	0.00875	129	0.0149	0.152	1	2	HLA-DQB1
Pubmed	10966857	Translational regulation of proinsulin biosynthesis and proinsulin conversion in the pancreatic beta-cell.	Pubmed	0.00875	129	0.0149	0.152	1	2	CPE
Pubmed	21901231	Common variants in the haemostatic gene pathway contribute to risk of early-onset myocardial infarction in the Italian population.	GeneRIF	0.00875	129	0.0149	0.152	1	2	PROCR
Pubmed	20301407	Genetic Prion Diseases	Pubmed	0.00875	129	0.0149	0.152	1	2	HLA-DQB1

Category	ID	Name	Source	p-value	q-value Bonferroni	q-value FDR B&H	q-value FDR B&Y	Hit Count in Query List	Hit Count in Genome	Hit in Query List
Pubmed	15374821	Aberrant expression of myosin isoforms in skeletal muscles from mice lacking the rev-erbAalpha orphan receptor gene.	GeneRIF	0.00875	129	0.0149	0.152	1	2	THRA
Pubmed	19166515	Contactin-associated protein (Caspr) 2 interacts with carboxypeptidase E in the CNS.	GeneRIF	0.00875	129	0.0149	0.152	1	2	CPE
Pubmed	24371137	GPR37 protein trafficking to the plasma membrane regulated by prosaposin and GM1 gangliosides promotes cell viability.	Pubmed	0.00875	129	0.0149	0.152	1	2	PSAP
Pubmed	18792407	Engagement of the EP2 prostanoid receptor closes the K+ channel KCa3.1 in human lung mast cells and attenuates their migration.	GeneRIF	0.00875	129	0.0149	0.152	1	2	KCNN4
Pubmed	648524	Interaction of adenylosuccinate synthetase with F-actin.	Pubmed	0.00875	129	0.0149	0.152	1	2	ACTA1
Pubmed	21251479	HLA class II alleles as markers of tuberculosis susceptibility and resistance.	GeneRIF	0.00875	129	0.0149	0.152	1	2	HLA-DQB1
Pubmed	22046441	IFI16 protein mediates the anti-inflammatory actions of the type-I interferons through suppression of activation of caspase-1 by inflammasomes.	GeneRIF	0.00875	129	0.0149	0.152	1	2	IFI16
Pubmed	24907345	The inhibitory effect of IFN-¿ on protease HTRA1 expression in rheumatoid arthritis.	Pubmed	0.00875	129	0.0149	0.152	1	2	HTRA1
Pubmed	12547834	Natriuretic peptide receptor A activation stabilizes a membrane-distal dimer interface.	GeneRIF	0.00875	129	0.0149	0.152	1	2	NPPA
Pubmed	22379027	Follicular helper NKT cells induce limited B cell responses and germinal center formation in the absence of CD4(+) T cell help.	Pubmed	0.00875	129	0.0149	0.152	1	2	HLA-DQB1
Pubmed	16237774	Relationship of human leukocyte antigen class II genes with the susceptibility to hepatitis B virus infection and the response to interferon in HBV-infected patients.	GeneRIF	0.00875	129	0.0149	0.152	1	2	HLA-DQB1
Pubmed	22539677	Thyroid hormone limits postnatal Sertoli cell proliferation in vivo by activation of its alpha1 isoform receptor (TRalpha1) present in these cells and by regulation of Cdk4/JunD/c-myc mRNA levels in mice.	Pubmed	0.00875	129	0.0149	0.152	1	2	THRA
Pubmed	23318129	The DQB1 *03:02 HLA haplotype is associated with increased risk of chronic pain after inguinal hernia surgery and lumbar disc herniation.	GeneRIF	0.00875	129	0.0149	0.152	1	2	HLA-DQB1
Pubmed	23417432	Urine of patients with early prostate cancer contains lower levels of light chain fragments of inter-alpha-trypsin inhibitor and saposin B but increased expression of an inter-alpha-trypsin inhibitor heavy chain 4 fragment.	Pubmed	0.00875	129	0.0149	0.152	1	2	PSAP
Pubmed	15059369	[Subclassification of seronegative type 1 diabetic subjects with HLA-DQ genotypes].	GeneRIF	0.00875	129	0.0149	0.152	1	2	HLA-DQB1
Pubmed	7592651	Repression of I-A beta gene expression by the transcription factor PU.1.	Pubmed	0.00875	129	0.0149	0.152	1	2	HLA-DQB1
Pubmed	16195413	Follicle size class contributes to distinct secretion patterns of inhibin isoforms during the rat estrous cycle.	GeneRIF	0.00875	129	0.0149	0.152	1	2	INHBB
Pubmed	19841542	Activated protein C therapy slows ALS-like disease in mice by transcriptionally inhibiting SOD1 in motor neurons and microglia cells.	Pubmed	0.00875	129	0.0149	0.152	1	2	PROCR
Pubmed	22218223	CIITA promoter I CARD-deficient mice express functional MHC class II genes in myeloid and lymphoid compartments.	GeneRIF	0.00875	129	0.0149	0.152	1	2	HLA-DQB1
Pubmed	15301863	The rare HLA-DQA1*03-DQB1*02 haplotype confers susceptibility to type 1 diabetes in whites and is preferentially associated with early clinical disease onset in male subjects.	GeneRIF	0.00875	129	0.0149	0.152	1	2	HLA-DQB1
Pubmed	22761382	Hepatitis C virus-mediated inhibition of cathepsin S increases invariant-chain expression on hepatocyte surface.	GeneRIF	0.00875	129	0.0149	0.152	1	2	CD74
Pubmed	23388725	Methyltransferase PRMT1 is a binding partner of HBx and a negative regulator of hepatitis B virus transcription.	GeneRIF	0.00875	129	0.0149	0.152	1	2	HLA-DQB1
Pubmed	19367338	Inhibitory activity of myelin-associated glycoprotein on sensory neurons is largely independent of NgR1 and NgR2 and resides within Ig-Like domains 4 and 5.	GeneRIF	0.00875	129	0.0149	0.152	1	2	RTN4R
Pubmed	24576301	Host cell virus entry mediated by Australian bat lyssavirus G envelope glycoprotein occurs through a clathrin-mediated endocytic pathway that requires actin and Rab5.	Pubmed	0.00875	129	0.0149	0.152	1	2	ACTA1
Pubmed	25354456	Expression and evolution of the Tiki1 and Tiki2 genes in vertebrates.	Pubmed	0.00875	129	0.0149	0.152	1	2	TRABD2B
Pubmed	23027953	Nuclear IFI16 induction of IRF-3 signaling during herpesviral infection and degradation of IFI16 by the viral ICP0 protein.	GeneRIF	0.00875	129	0.0149	0.152	1	2	IFI16
Pubmed	10731151	Rapid progression to AIDS in HIV+ individuals with a structural variant of the chemokine receptor CX3CR1.	Pubmed	0.00875	129	0.0149	0.152	1	2	CX3CL1
Pubmed	19409928	Potassium channel expression in human CD4+ regulatory and naïve T cells from healthy subjects and multiple sclerosis patients.	GeneRIF	0.00875	129	0.0149	0.152	1	2	KCNN4
Pubmed	25092262	Identification of fibronectin binding sites in dermatopontin and their biological function.	Pubmed	0.00875	129	0.0149	0.152	1	2	DPT
Pubmed	24676787	High coexpression of CCL2 and CX3CL1 is gender-specifically associated with good prognosis in soft tissue sarcoma patients.	Pubmed	0.00875	129	0.0149	0.152	1	2	CX3CL1
Pubmed	15827562	Protein studies in dysferlinopathy patients using llama-derived antibody fragments selected by phage display.	Pubmed	0.00875	129	0.0149	0.152	1	2	DYSF
Pubmed	19254255	HLA DRB1*130101-DQB1*060101 haplotype is associated with acute chest syndrome in sickle cell anemia patients.	GeneRIF	0.00875	129	0.0149	0.152	1	2	HLA-DQB1
Pubmed	16456532	CD8+ T cells are effector cells of contact dermatitis to common skin allergens in mice.	Pubmed	0.00875	129	0.0149	0.152	1	2	HLA-DQB1
Pubmed	17343268	Exclusion of OGDH and BMP4 as candidate genes in two siblings with autosomal recessive DOOR syndrome.	GeneRIF	0.00875	129	0.0149	0.152	1	2	BMP4
Pubmed	16338472	Regulation of murine Ada gene expression in the placenta by transcription factor RUNX1.	GeneRIF	0.00875	129	0.0149	0.152	1	2	ADA
Pubmed	22938381	HLA class II polymorphisms and recurrent spontaneous abortion in a Southern Brazilian cohort.	Pubmed	0.00875	129	0.0149	0.152	1	2	HLA-DQB1

Category	ID	Name	Source	p-value	q-value Bonferroni	q-value FDR B&H	q-value FDR B&Y	Hit Count in Query List	Hit Count in Genome	Hit in Query List
Pubmed	19486004	Tunisian endemic pemphigus foliaceus is associated with the HLA-DR3 gene: anti-desmoglein 1 antibody-positive healthy subjects bear protective alleles.	GeneRIF	0.00875	129	0.0149	0.152	1	2	HLA-DQB1
Pubmed	1525182	Site-specific glycation of lens crystallins by ascorbic acid.	Pubmed	0.00875	129	0.0149	0.152	1	2	CRYAA
Pubmed	7477119	Brief report: impaired processing of prohormones associated with abnormalities of glucose homeostasis and adrenal function.	Pubmed	0.00875	129	0.0149	0.152	1	2	CPE
Pubmed	21320466	hERG channel blockade by externally applied quaternary ammonium derivatives.	GeneRIF	0.00875	129	0.0149	0.152	1	2	KCNH2
Pubmed	24284793	BMP4 and BMP7 induce the white-to-brown transition of primary human adipose stem cells.	Pubmed	0.00875	129	0.0149	0.152	1	2	BMP4
Pubmed	11159348	Mesenchymal cell precursors of peritubular smooth muscle cells of the mouse testis can be identified by the presence of the p75 neurotrophin receptor.	Pubmed	0.00875	129	0.0149	0.152	1	2	NGFR
Pubmed	22174839	Dysferlin interacts with histone deacetylase 6 and increases alpha-tubulin acetylation.	GeneRIF	0.00875	129	0.0149	0.152	1	2	DYSF
Pubmed	20080985	Adipogenesis is differentially impaired by thyroid hormone receptor mutant isoforms.	GeneRIF	0.00875	129	0.0149	0.152	1	2	THRA
Pubmed	15774622	CCR6 regulates CD4+ T-cell-mediated acute graft-versus-host disease responses.	GeneRIF	0.00875	129	0.0149	0.152	1	2	HLA-DQB1
Pubmed	17234469	Expression of glutathione S-transferase pi and glutathione synthase correlates with survival in early stage non-small cell carcinomas of the lung.	GeneRIF	0.00875	129	0.0149	0.152	1	2	GSS
Pubmed	23793423	Association of HLA-DRB1*15 and HLADQB1*06 with SLE in Saudis.	Pubmed	0.00875	129	0.0149	0.152	1	2	HLA-DQB1
Pubmed	23867623	Atrial natriuretic peptide is negatively regulated by microRNA-425.	Pubmed	0.00875	129	0.0149	0.152	1	2	NPPA
Pubmed	20512991	CX(3)CR1 and vascular adhesion protein-1-dependent recruitment of CD16(+) monocytes across human liver sinusoidal endothelium.	GeneRIF	0.00875	129	0.0149	0.152	1	2	CX3CL1
Pubmed	25139985	Experimental basis for a new allosteric model for multisubunit proteins.	Pubmed	0.00875	129	0.0149	0.152	1	2	HBB
Pubmed	16356170	Rapid screening for glucose-6-phosphate dehydrogenase deficiency and haemoglobin polymorphisms in Africa by a simple high-throughput SSOP-ELISA method.	GeneRIF	0.00875	129	0.0149	0.152	1	2	HBB
Pubmed	23174700	Lubricin and smooth muscle a-actin-containing myofibroblasts in the pseudomembranes around loose hip and knee prostheses.	GeneRIF	0.00875	129	0.0149	0.152	1	2	ACTA1
Pubmed	19923448	Role of CX3CL1/fractalkine in osteoclast differentiation and bone resorption.	GeneRIF	0.00875	129	0.0149	0.152	1	2	CX3CL1
Pubmed	20488791	TLR9-dependent induction of intestinal alpha-defensins by Toxoplasma gondii.	GeneRIF	0.00875	129	0.0149	0.152	1	2	MMP7
Pubmed	11931637	Characterization and expression of human bifunctional 3'-phosphoadenosine 5'-phosphosulphate synthase isoforms.	GeneRIF	0.00875	129	0.0149	0.152	1	2	PAPSS2
Pubmed	11178107	Genetic modification of glaucoma associated phenotypes between AKXD-28/Ty and DBA/2J mice.	Pubmed	0.00875	129	0.0149	0.152	1	2	GPNMB
Pubmed	19884273	Association of HLA class II genes with systemic sclerosis in Spanish patients.	GeneRIF	0.00875	129	0.0149	0.152	1	2	HLA-DQB1
Pubmed	19403809	p75 and TrkA signaling regulates sympathetic neuronal firing patterns via differential modulation of voltage-gated currents.	GeneRIF	0.00875	129	0.0149	0.152	1	2	NGFR
Pubmed	10191308	Functional analysis of a mouse brain Elk-type K+ channel.	Pubmed	0.00875	129	0.0149	0.152	1	2	KCNH2
Pubmed	11259305	Binding of dystrophin's tandem calponin homology domain to F-actin is modulated by actin's structure.	Pubmed	0.00875	129	0.0149	0.152	1	2	ACTA1
Pubmed	19214751	WIN55,212-2 inhibits production of CX3CL1 by human astrocytes: involvement of p38 MAP kinase.	GeneRIF	0.00875	129	0.0149	0.152	1	2	CX3CL1
Pubmed	11016932	Identification of a major heparin-binding site in kallistatin.	Pubmed	0.00875	129	0.0149	0.152	1	2	KLK1
Pubmed	22645147	The forkhead box transcription factor FOXC1 promotes breast cancer invasion by inducing matrix metalloprotease 7 (MMP7) expression.	GeneRIF	0.00875	129	0.0149	0.152	1	2	MMP7
Pubmed	16428780	Biogenesis of Leishmania major-harboring vacuoles in murine dendritic cells.	Pubmed	0.00875	129	0.0149	0.152	1	2	CD68
Pubmed	22529380	Subcellular distribution of Lck during CD4 T-cell maturation in the thymic medulla regulates the T-cell activation threshold.	GeneRIF	0.00875	129	0.0149	0.152	1	2	HLA-DQB1
Pubmed	11773436	Congenital hypothyroid Pax8(-/-) mutant mice can be rescued by inactivating the TRalpha gene.	GeneRIF	0.00875	129	0.0149	0.152	1	2	THRA
Pubmed	19112531	Identification of STRA6 and SKI sequence variants in patients with anophthalmia/microphthalmia.	GeneRIF	0.00875	129	0.0149	0.152	1	2	STRA6
Pubmed	24768947	Interleukin-10 gene promoter polymorphisms in celiac patients from north-eastern Italy.	Pubmed	0.00875	129	0.0149	0.152	1	2	HLA-DQB1
Pubmed	10835640	Mutations in the gene encoding the serine protease inhibitor, Kazal type 1 are associated with chronic pancreatitis.	Pubmed	0.00875	129	0.0149	0.152	1	2	SPINK1
Pubmed	7769089	Expression and differential regulation of natriuretic peptides in mouse macrophages.	Pubmed	0.00875	129	0.0149	0.152	1	2	NPPA
Pubmed	11978563	HLA class II alleles in patients with alopecia areata.	GeneRIF	0.00875	129	0.0149	0.152	1	2	HLA-DQB1
Pubmed	1893873	Involvement of Bone Morphogenetic Protein-4 (BMP-4) and Vgr-1 in morphogenesis and neurogenesis in the mouse.	Pubmed	0.00875	129	0.0149	0.152	1	2	BMP4
Pubmed	25357219	Observation of microvessels and invasion in early colorectal neoplasms on narrow band imaging: combination with CD34 and matrix metalloproteinase-7 expression.	Pubmed	0.00875	129	0.0149	0.152	1	2	MMP7
Pubmed	21960143	Depletion of cathepsin D by transglutaminase 2 through protein cross-linking promotes cell survival.	GeneRIF	0.00875	129	0.0149	0.152	1	2	TGM2
Pubmed	19996413	Impaired clot retraction in factor XIII A subunit-deficient mice.	GeneRIF	0.00875	129	0.0149	0.152	1	2	TGM2
Pubmed	16497624	Mutations in the calcium-sensing receptor: a new genetic risk factor for chronic pancreatitis?	GeneRIF	0.00875	129	0.0149	0.152	1	2	SPINK1
Pubmed	17212706	Childhood asthma and human leukocyte antigen type.	GeneRIF	0.00875	129	0.0149	0.152	1	2	HLA-DQB1
Pubmed	12210873	ACT and UCH-L1 polymorphisms in Parkinson's disease and age of onset.	GeneRIF	0.00875	129	0.0149	0.152	1	2	ACTA1

Category	ID	Name	Source	p-value	q-value Bonferroni	q-value FDR B&H	q-value FDR B&Y	Hit Count in Query List	Hit Count in Genome	Hit in Query List
Pubmed	20047948	Neuronopathic Gaucher disease in the mouse: viable combined selective saposin C deficiency and mutant glucocerebrosidase (V394L) mice with glucosylsphingosine and glucosylceramide accumulation and progressive neurological deficits.	GeneRIF	0.00875	129	0.0149	0.152	1	2	PSAP
Pubmed	16883543	[The genetic polymorphism of HLA-DQA1 and HLA-DQB1 genes of Chinese Han population in Jiangsu area is studied by PCR-sequence-based typing].	GeneRIF	0.00875	129	0.0149	0.152	1	2	HLA-DQB1
Pubmed	22209233	Casein kinase 2 regulates vaccinia virus actin tail formation.	GeneRIF	0.00875	129	0.0149	0.152	1	2	ACTA1
Pubmed	11955434	P-Rex1, a PtdIns(3,4,5)P3- and Gbetagamma-regulated guanine-nucleotide exchange factor for Rac.	GeneRIF	0.00875	129	0.0149	0.152	1	2	PREX1
Pubmed	11442349	Tissue transglutaminase selectively modifies proteins associated with truncated mutant huntingtin in intact cells.	Pubmed	0.00875	129	0.0149	0.152	1	2	TGM2
Pubmed	25209151	Resolution of HLA-B*44:02:01G, -DRB1*14:01:01G and -DQB1*03:01:01G reveals a high allelic variability among 12 European populations.	Pubmed	0.00875	129	0.0149	0.152	1	2	HLA-DQB1
Pubmed	20194586	Antigen-presenting dendritic cells rescue CD4-depleted CCR2-/- mice from lethal Histoplasma capsulatum infection.	Pubmed	0.00875	129	0.0149	0.152	1	2	HLA-DQB1
Pubmed	18955791	Human leukocyte antigen class II association with spontaneous recovery from hepatitis B virus infection in Koreans: analysis at the haplotype level.	GeneRIF	0.00875	129	0.0149	0.152	1	2	HLA-DQB1
Pubmed	546546	[Up-dating the classification of malignant conjunctival melanomas].	Pubmed	0.00875	129	0.0149	0.152	1	2	ALAS2
Pubmed	8635221	Removal of Arg141 from the alpha chain of human hemoglobin by carboxypeptidases N and M.	Pubmed	0.00875	129	0.0149	0.152	1	2	HBA2
Pubmed	10912470	Altered expression of natriuretic peptide receptors in proANP gene disrupted mice.	Pubmed	0.00875	129	0.0149	0.152	1	2	NPPA
Pubmed	21397192	Interferon-inducible protein 16: insight into the interaction with tumor suppressor p53.	GeneRIF	0.00875	129	0.0149	0.152	1	2	IFI16
Pubmed	15304490	Endothelial lipase modulates susceptibility to atherosclerosis in apolipoprotein-E-deficient mice.	GeneRIF	0.00875	129	0.0149	0.152	1	2	LIPG
Pubmed	25533914	Plasma levels of mannan-binding lectin-associated serine proteases MASP-1 and MASP-2 are elevated in type 1 diabetes and correlate with glycaemic control.	Pubmed	0.00875	129	0.0149	0.152	1	2	MASP1
Pubmed	17991705	Decline in intestinal mucosal IL-10 expression and decreased intestinal barrier function in a mouse model of total parenteral nutrition.	GeneRIF	0.00875	129	0.0149	0.152	1	2	CLDN15
Pubmed	12493453	DQA1 and DQB1 genes polymorphism on acute rejection development in liver transplantation.	GeneRIF	0.00875	129	0.0149	0.152	1	2	HLA-DQB1
Pubmed	16898315	Screening of concurrent alpha-thalassaemia 1 in beta-thalassaemia carriers.	GeneRIF	0.00875	129	0.0149	0.152	1	2	HBB
Pubmed	17406641	Specific combinations of HLA-DR2 and DR3 class II haplotypes contribute graded risk for disease susceptibility and autoantibodies in human SLE.	GeneRIF	0.00875	129	0.0149	0.152	1	2	HLA-DQB1
Pubmed	16800740	Expression of the human kallikrein genes 10 (KLK10) and 11 (KLK11) in cancerous and non-cancerous lung tissues.	GeneRIF	0.00875	129	0.0149	0.152	1	2	KLK10
Pubmed	7759870	Evidence for lineage commitment and initiation of positive selection by thymocytes with intermediate surface phenotypes.	Pubmed	0.00875	129	0.0149	0.152	1	2	HLA-DQB1
Pubmed	15082592	Absence of PRSS1 mutations and association of SPINK1 trypsin inhibitor mutations in hereditary and non-hereditary chronic pancreatitis.	GeneRIF	0.00875	129	0.0149	0.152	1	2	SPINK1
Pubmed	12417403	Mutation of Large, which encodes a putative glycosyltransferase, in an animal model of muscular dystrophy.	Pubmed	0.00875	129	0.0149	0.152	1	2	LARGE
Pubmed	24080013	BMP4 is required for the initial expression of MITF in melanocyte precursor differentiation from embryonic stem cells.	Pubmed	0.00875	129	0.0149	0.152	1	2	BMP4
Pubmed	23328279	Hepatic lipase- and endothelial lipase-deficiency in mice promotes macrophage-to-feces RCT and HDL antioxidant properties.	GeneRIF	0.00875	129	0.0149	0.152	1	2	LIPG
Pubmed	14634076	Requirements for T cell-polarized tubulation of class II+ compartments in dendritic cells.	GeneRIF	0.00875	129	0.0149	0.152	1	2	HLA-DQB1
Pubmed	23261940	NADPH oxidase 2-derived superoxide downregulates endothelial KCa3.1 in preeclampsia.	GeneRIF	0.00875	129	0.0149	0.152	1	2	KCNN4
Pubmed	2537655	Tissue-differential expression of two distinct genes for phosphoribosyl pyrophosphate synthetase and existence of the testis-specific transcript.	Pubmed	0.00875	129	0.0149	0.152	1	2	PRPS2
Pubmed	19219041	Association of common variants in NPPA and NPPB with circulating natriuretic peptides and blood pressure.	GeneRIF	0.00875	129	0.0149	0.152	1	2	NPPA
Pubmed	20450916	Inhibition of tissue transglutaminase sensitizes TRAIL-resistant lung cancer cells through upregulation of death receptor 5.	GeneRIF	0.00875	129	0.0149	0.152	1	2	TGM2
Pubmed	3584986	Complete sequence of the HLA DQ alpha and DQ beta cDNA from a DR5/DQw3 cell line.	Pubmed	0.00875	129	0.0149	0.152	1	2	HLA-DQB1
Pubmed	22740690	X-linked sideroblastic anemia due to carboxyl-terminal ALAS2 mutations that cause loss of binding to the ß-subunit of succinyl-CoA synthetase (SUCLA2).	GeneRIF	0.00875	129	0.0149	0.152	1	2	ALAS2
Pubmed	21411731	Macrophage migration inhibitory factor and CD74 regulate macrophage chemotactic responses via MAPK and Rho GTPase.	GeneRIF	0.00875	129	0.0149	0.152	1	2	CD74
Pubmed	7358733	Sites of nonenzymatic glycosylation of human hemoglobin A.	Pubmed	0.00875	129	0.0149	0.152	1	2	HBB
Pubmed	8111631	Neurosteroid biosynthesis: genes for adrenal steroidogenic enzymes are expressed in the brain.	Pubmed	0.00875	129	0.0149	0.152	1	2	CYP11A1
Pubmed	10382755	Fractalkine and macrophage-derived chemokine: T cell-attracting chemokines expressed in T cell area dendritic cells.	Pubmed	0.00875	129	0.0149	0.152	1	2	CX3CL1
Pubmed	10644413	Bmp-4 requires the presence of the digits to initiate programmed cell death in limb interdigital tissues.	Pubmed	0.00875	129	0.0149	0.152	1	2	BMP4

Category	ID	Name	Source	p-value	q-value Bonferroni	q-value FDR B&H	q-value FDR B&Y	Hit Count in Query List	Hit Count in Genome	Hit in Query List
Pubmed	19767734	The obesity susceptibility gene Cpe links FoxO1 signaling in hypothalamic pro-opiomelanocortin neurons with regulation of food intake.	GeneRIF	0.00875	129	0.0149	0.152	1	2	CPE
Pubmed	9618718	Investigation of the 'fines' hypothesis of primary open-angle glaucoma: the possible role of alpha-crystallin.	Pubmed	0.00875	129	0.0149	0.152	1	2	CRYAA
Pubmed	17287858	Rare CBFB-MYH11 fusion transcripts in AML with inv(16)/t(16;16) are associated with therapy-related AML M4eo, atypical cytomorphology, atypical immunophenotype, atypical additional chromosomal rearrangements and low white blood cell count: a study on 162 patients.	GeneRIF	0.00875	129	0.0149	0.152	1	2	MYH11
Pubmed	22289504	CD163 versus CD68 in tumor associated macrophages of classical Hodgkin lymphoma.	GeneRIF	0.00875	129	0.0149	0.152	1	2	CD68
Pubmed	18163887	Tissue-specific promoter utilisation of the kallikrein-related peptidase genes, KLK5 and KLK7, and cellular localisation of the encoded proteins suggest roles in exocrine pancreatic function.	GeneRIF	0.00875	129	0.0149	0.152	1	2	KLK7
Pubmed	19041429	HLA class II association with autoimmune hepatitis in Latin America: a meta-analysis.	GeneRIF	0.00875	129	0.0149	0.152	1	2	HLA-DQB1
Pubmed	17909943	C-Terminal truncation affects subunit exchange of human alphaA-crystallin with alphaB-crystallin.	GeneRIF	0.00875	129	0.0149	0.152	1	2	CRYAA
Pubmed	25153389	Prognostic significance of human tissue kallikrein-related peptidases 6 and 10 in gastric cancer.	Pubmed	0.00875	129	0.0149	0.152	1	2	KLK10
Pubmed	19680746	Celiac disease IgA modulates vascular permeability in vitro through the activity of transglutaminase 2 and RhoA.	GeneRIF	0.00875	129	0.0149	0.152	1	2	TGM2
Pubmed	22662777	HLA-DQ typing in the diagnostic algorithm of celiac disease.	GeneRIF	0.00875	129	0.0149	0.152	1	2	HLA-DQB1
Pubmed	22898764	HLA class II is a factor in cardiovascular morbidity and mortality rates in patients with type 1 diabetes.	GeneRIF	0.00875	129	0.0149	0.152	1	2	HLA-DQB1
Pubmed	2911574	Sequence investigation of the major gastrointestinal tumor-associated antigen gene family, GA733.	Pubmed	0.00875	129	0.0149	0.152	1	2	TACSTD2
Pubmed	647395	Naked axons in myodystrophic mice.	Pubmed	0.00875	129	0.0149	0.152	1	2	LARGE
Pubmed	11725045	New Insights into the Structure and Function of the Thyroid Hormone Receptor.	Pubmed	0.00875	129	0.0149	0.152	1	2	THRA
Pubmed	11818506	Nerve growth factor regulates dopamine D(2) receptor expression in prolactinoma cell lines via p75(NGFR)-mediated activation of nuclear factor-kappaB.	GeneRIF	0.00875	129	0.0149	0.152	1	2	NGFR
Pubmed	16880207	BMP4 activation and secretion are negatively regulated by an intracellular gremlin-BMP4 interaction.	GeneRIF	0.00875	129	0.0149	0.152	1	2	BMP4
Pubmed	16732273	Control of microglial neurotoxicity by the fractalkine receptor.	Pubmed	0.00875	129	0.0149	0.152	1	2	CX3CL1
Pubmed	24352566	A PAK6-IQGAP1 complex promotes disassembly of cell-cell adhesions.	Pubmed	0.00875	129	0.0149	0.152	1	2	PAK6
Pubmed	18569569	Lack of association of delta-aminolevulinate dehydratase polymorphisms with blood lead levels and hemoglobin in Romanian women from a lead-contaminated region.	GeneRIF	0.00875	129	0.0149	0.152	1	2	ALAS2
Pubmed	21821711	KLF1 gene mutations cause borderline HbA(2).	GeneRIF	0.00875	129	0.0149	0.152	1	2	HBA2
Pubmed	15688004	Activation of FoxO transcription factors contributes to the antiproliferative effect of cAMP.	GeneRIF	0.00875	129	0.0149	0.152	1	2	FOXO4
Pubmed	19132243	High glucose conditions induce upregulation of fractalkine and monocyte chemotactic protein-1 in human smooth muscle cells.	GeneRIF	0.00875	129	0.0149	0.152	1	2	CX3CL1
Pubmed	22710983	Distinctive contact between CD34+ hematopoietic progenitors and CXCL12+ CD271+ mesenchymal stromal cells in benign and myelodysplastic bone marrow.	GeneRIF	0.00875	129	0.0149	0.152	1	2	NGFR
Pubmed	18639655	Study of subunit interactions of alpha A- and alpha B-crystallins and the effects of gamma-irradiation on their interactions by surface plasmon resonance.	GeneRIF	0.00875	129	0.0149	0.152	1	2	CRYAA
Pubmed	17762175	Enhancement of P2Y6-induced Cl- secretion by IL-13 and modulation of SK4 channels activity in human bronchial cells.	GeneRIF	0.00875	129	0.0149	0.152	1	2	KCNN4
Pubmed	11802952	[Association between asthma and the polymorphism of HLA-DQ genes].	GeneRIF	0.00875	129	0.0149	0.152	1	2	HLA-DQB1
Pubmed	17576097	Screening the human prepro-orexin gene in a single-centre narcolepsy cohort.	GeneRIF	0.00875	129	0.0149	0.152	1	2	HLA-DQB1
Pubmed	18334636	P-Rex2 regulates Purkinje cell dendrite morphology and motor coordination.	GeneRIF	0.00875	129	0.0149	0.152	1	2	PREX1
Pubmed	21898398	CCN3 increases BMP-4 expression and bone mineralization in osteoblasts.	GeneRIF	0.00875	129	0.0149	0.152	1	2	BMP4
Pubmed	15987793	Complete cystic fibrosis transmembrane conductance regulator gene sequencing in patients with idiopathic chronic pancreatitis and controls.	GeneRIF	0.00875	129	0.0149	0.152	1	2	SPINK1
Pubmed	18586828	The role of transcriptional activator GATA-1 at human beta-globin HS2.	GeneRIF	0.00875	129	0.0149	0.152	1	2	HBB
Pubmed	21525510	Fractalkine is a novel human adipochemokine associated with type 2 diabetes.	GeneRIF	0.00875	129	0.0149	0.152	1	2	CX3CL1
Pubmed	23673366	BMP4 is a novel transcriptional target and mediator of mammary cell migration downstream of the Hippo pathway component TAZ.	Pubmed	0.00875	129	0.0149	0.152	1	2	BMP4
Pubmed	25321193	Shed Syndecan-1 is involved in chemotherapy resistance via the EGFR pathway in colorectal cancer.	Pubmed	0.00875	129	0.0149	0.152	1	2	MMP7
Pubmed	2972276	Hydrolysis of alpha-human atrial natriuretic peptide in vitro by human kidney membranes and purified endopeptidase-24.11. Evidence for a novel cleavage site.	Pubmed	0.00875	129	0.0149	0.152	1	2	NPPA
Pubmed	22635104	Involvement of insulin-like growth factor 1 receptor signaling in the amyloid-ß peptide oligomers-induced p75 neurotrophin receptor protein expression in mouse hippocampus.	GeneRIF	0.00875	129	0.0149	0.152	1	2	NGFR

Category	ID	Name	Source	p-value	q-value Bonferroni	q-value FDR B&H	q-value FDR B&Y	Hit Count in Query List	Hit Count in Genome	Hit in Query List
Pubmed	14684598	Insulin-like growth factor-induced transcriptional activity of the skeletal alpha-actin gene is regulated by signaling mechanisms linked to voltage-gated calcium channels during myoblast differentiation.	GeneRIF	0.00875	129	0.0149	0.152	1	2	ACTA1
Pubmed	24052262	ASB2a, an E3 Ubiquitin Ligase Specificity Subunit, Regulates Cell Spreading and Triggers Proteasomal Degradation of Filamins by Targeting the Filamin Calponin Homology 1 Domain.	Pubmed	0.00875	129	0.0149	0.152	1	2	ASB2
Pubmed	23697277	Gamma-glutamyltransferase and C-reactive protein in stable chronic obstructive pulmonary disease.	GeneRIF	0.00875	129	0.0149	0.152	1	2	GGT1
Pubmed	20095964	S165F mutation of junctophilin 2 affects Ca2+ signalling in skeletal muscle.	GeneRIF	0.00875	129	0.0149	0.152	1	2	JPH2
Pubmed	22118688	Analysis of BMP4 and BMP7 signaling in breast cancer cells unveils time-dependent transcription patterns and highlights a common synexpression group of genes.	GeneRIF	0.00875	129	0.0149	0.152	1	2	BMP4
Pubmed	7510692	A delayed-early gene activated by fibroblast growth factor-1 encodes a protein related to aldose reductase.	Pubmed	0.00875	129	0.0149	0.152	1	2	AKR1B10
Pubmed	18765527	Epithelial and stromal cathepsin K and CXCL14 expression in breast tumor progression.	GeneRIF	0.00875	129	0.0149	0.152	1	2	CTSK
Pubmed	9325059	The visual assignment of genes by fiber-fish: BTF3 protein homologue gene (BTF3) and a novel pseudogene of human RNA helicase A (DDX9P) on 13q22.	Pubmed	0.00875	129	0.0149	0.152	1	2	BTF3P11
Pubmed	14657873	The CX3C chemokine fractalkine in allergic asthma and rhinitis.	GeneRIF	0.00875	129	0.0149	0.152	1	2	CX3CL1
Pubmed	19758188	NGF and its receptor system: a new dimension in the pathogenesis of psoriasis and psoriatic arthritis.	GeneRIF	0.00875	129	0.0149	0.152	1	2	NGFR
Pubmed	22505523	Kallikrein-related peptidase signaling in colon carcinoma cells: targeting proteinase-activated receptors.	GeneRIF	0.00875	129	0.0149	0.152	1	2	KLK14
Pubmed	1225760	Linkage relationships of markers on chromosome 17 of the house mouse.	Pubmed	0.00875	129	0.0149	0.152	1	2	THRA
Pubmed	19859778	Depressed expression of MuRF1 and MAFbx in areas remote of recent myocardial infarction: a mechanism contributing to myocardial remodeling?	GeneRIF	0.00875	129	0.0149	0.152	1	2	FBXO32
Pubmed	23981568	Fractalkine (CX3CL1) enhances hippocampal N-methyl-D-aspartate receptor (NMDAR) function via D-serine and adenosine receptor type A2 (A2AR) activity.	Pubmed	0.00875	129	0.0149	0.152	1	2	CX3CL1
Pubmed	17804692	CD83 influences cell-surface MHC class II expression on B cells and other antigen-presenting cells.	GeneRIF	0.00875	129	0.0149	0.152	1	2	HLA-DQB1
Pubmed	22825846	Kallikrein-related peptidase-7 regulates caspase-14 maturation during keratinocyte terminal differentiation by generating an intermediate form.	GeneRIF	0.00875	129	0.0149	0.152	1	2	KLK7
Pubmed	19759519	The antiapoptotic protein Bcl-xL negatively regulates the bone-resorbing activity of osteoclasts in mice.	Pubmed	0.00875	129	0.0149	0.152	1	2	CTSK
Pubmed	24569872	Migration inhibitory factor enhances inflammation via CD74 in cartilage end plates with Modic type 1 changes on MRI.	Pubmed	0.00875	129	0.0149	0.152	1	2	CD74
Pubmed	18780836	hem6: an ENU-induced recessive hypochromic microcytic anemia mutation in the mouse.	GeneRIF	0.00875	129	0.0149	0.152	1	2	ALAS2
Pubmed	17161429	MHC-class-II-deficiency impairs wound healing.	Pubmed	0.00875	129	0.0149	0.152	1	2	HLA-DQB1
Pubmed	12083803	Procathepsin D interacts with prosaposin in cancer cells but its internalization is not mediated by LDL receptor-related protein.	GeneRIF	0.00875	129	0.0149	0.152	1	2	PSAP
Pubmed	11159823	Cardiac ion channel expression and contractile function in mice with deletion of thyroid hormone receptor alpha or beta.	Pubmed	0.00875	129	0.0149	0.152	1	2	THRA
Pubmed	7594462	Expression of ecto-adenosine deaminase and CD26 in human T cells triggered by the TCR-CD3 complex. Possible role of adenosine deaminase as costimulatory molecule.	Pubmed	0.00875	129	0.0149	0.152	1	2	ADA
Pubmed	3692165	Nucleotide sequence analysis of class II genes borne by mouse t chromosomes.	Pubmed	0.00875	129	0.0149	0.152	1	2	HLA-DQB1
Pubmed	18312480	Evidence for HLA class II susceptible and protective haplotypes for osteomyelitis in pediatric patients with sickle cell anemia.	GeneRIF	0.00875	129	0.0149	0.152	1	2	HLA-DQB1
Pubmed	19758197	Human leukocyte antigen-DRB1 and -DQB1 genotyping in lupus patients with and without antiphospholipid syndrome.	GeneRIF	0.00875	129	0.0149	0.152	1	2	HLA-DQB1
Pubmed	24782191	The role of transforming growth factor ß signaling in fibroblast-like synoviocytes from patients with oligoarticular juvenile idiopathic arthritis: dysregulation of transforming growth factor ß signaling, including overexpression of bone morphogenetic protein 4, may lead to a chondrocyte phenotype and may contribute to bony hypertrophy.	Pubmed	0.00875	129	0.0149	0.152	1	2	BMP4
Pubmed	15388638	Bone morphogenic protein 4 produced in endothelial cells by oscillatory shear stress induces monocyte adhesion by stimulating reactive oxygen species production from a nox1-based NADPH oxidase.	GeneRIF	0.00875	129	0.0149	0.152	1	2	BMP4
Pubmed	19136670	Scavenger receptor BI-mediated selective uptake is required for the remodeling of high density lipoprotein by endothelial lipase.	GeneRIF	0.00875	129	0.0149	0.152	1	2	LIPG
Pubmed	21906273	Nogo receptor is involved in the adhesion of dendritic cells to myelin.	GeneRIF	0.00875	129	0.0149	0.152	1	2	RTN4R
Pubmed	25482559	Regulatory T cells occupy an isolated niche in the intestine that is antigen independent.	Pubmed	0.00875	129	0.0149	0.152	1	2	HLA-DQB1
Pubmed	16571783	Distal colonic K(+) secretion occurs via BK channels.	Pubmed	0.00875	129	0.0149	0.152	1	2	KCNN4
Pubmed	22062893	Mononuclear cell adenosine deaminase and CD26/dipeptidylpeptidase-IV activities are sensitive markers of reperfusion during percutaneous transluminal angioplasty.	Pubmed	0.00875	129	0.0149	0.152	1	2	ADA
Pubmed	2039600	Purification and characterization of elastase-specific inhibitor. Sequence homology with mucus proteinase inhibitor.	Pubmed	0.00875	129	0.0149	0.152	1	2	SLPI

Category	ID	Name	Source	p-value	q-value Bonferroni	q-value FDR B&H	q-value FDR B&Y	Hit Count in Query List	Hit Count in Genome	Hit in Query List
Pubmed	20567233	Functional osteoclast attachment requires inositol-1,4,5-trisphosphate receptor-associated cGMP-dependent kinase substrate.	GeneRIF	0.00875	129	0.0149	0.152	1	2	MRVI1
Pubmed	17124999	HLa-class II (DRB & DQB1) in Thai sudden unexplained death syndrome (Thai SUDS) families (Lai-Tai families).	GeneRIF	0.00875	129	0.0149	0.152	1	2	HLA-DQB1
Pubmed	14551202	A new role for IQ motif proteins in regulating calmodulin function.	GeneRIF	0.00875	129	0.0149	0.152	1	2	PCP4
Pubmed	23055188	Different effects of ERß and TROP2 expression in Chinese patients with early-stage colon cancer.	GeneRIF	0.00875	129	0.0149	0.152	1	2	TACSTD2
Pubmed	2209618	The complete amino-acid sequences of human ganglioside GM2 activator protein and cerebroside sulfate activator protein.	Pubmed	0.00875	129	0.0149	0.152	1	2	PSAP
Pubmed	12925852	The B7 family member B7-H3 preferentially down-regulates T helper type 1-mediated immune responses.	GeneRIF	0.00875	129	0.0149	0.152	1	2	CD276
Pubmed	11909945	BP1, a homeodomain-containing isoform of DLX4, represses the beta-globin gene.	GeneRIF	0.00875	129	0.0149	0.152	1	2	HBB
Pubmed	12235106	Synergistic enhancement of bone formation and healing by stem cell-expressed VEGF and bone morphogenetic protein-4.	GeneRIF	0.00875	129	0.0149	0.152	1	2	BMP4
Pubmed	16300411	Elafin and its precursor trappin-2 still inhibit neutrophil serine proteinases when they are covalently bound to extracellular matrix proteins by tissue transglutaminase.	GeneRIF	0.00875	129	0.0149	0.152	1	2	TGM2
Pubmed	15548533	Genomic organization and functional analysis of murine PKD2L1.	GeneRIF	0.00875	129	0.0149	0.152	1	2	PKD2L1
Pubmed	21031019	LOC387715/HTRA1 gene polymorphisms and susceptibility to age-related macular degeneration: A HuGE review and meta-analysis.	GeneRIF	0.00875	129	0.0149	0.152	1	2	HTRA1
Pubmed	21091360	Association analysis about HLA-DRB1, -DQB1 polymorphism and auto-antibodies against a(1)-adrenergic receptors in Chinese patients with essential hypertension.	GeneRIF	0.00875	129	0.0149	0.152	1	2	HLA-DQB1
Pubmed	11724419	Low frequency of HLA DRB1*03 - DQB1*02 and DQB1*0302 haplotypes in Romania is consistent with the country's low incidence of Type I diabetes.	GeneRIF	0.00875	129	0.0149	0.152	1	2	HLA-DQB1
Pubmed	16267776	Association between human leukocyte antigen class II alleles and genotype of Borrelia burgdorferi in patients with early lyme disease.	GeneRIF	0.00875	129	0.0149	0.152	1	2	HLA-DQB1
Pubmed	20719952	HIV controllers with HLA-DRB1*13 and HLA-DQB1*06 alleles have strong, polyfunctional mucosal CD4+ T-cell responses.	GeneRIF	0.00875	129	0.0149	0.152	1	2	HLA-DQB1
Pubmed	18939352	Correlation between CFH Y402H and HTRA1 rs11200638 genotype to typical exudative age-related macular degeneration and polypoidal choroidal vasculopathy phenotype in the Japanese population.	GeneRIF	0.00875	129	0.0149	0.152	1	2	HTRA1
Pubmed	17989347	Functional characterization of bone morphogenetic protein binding sites and Smad1/5 activation in human vascular cells.	GeneRIF	0.00875	129	0.0149	0.152	1	2	BMP4
Pubmed	17395831	Regulation of hepatic stellate cell differentiation by the neurotrophin receptor p75NTR.	GeneRIF	0.00875	129	0.0149	0.152	1	2	NGFR
Pubmed	3007037	Chromosomal mapping of enzyme loci in the domestic cat: GSR to C2, ADA and ITPA to A3, and LDHA-ACP2 to D1.	Pubmed	0.00875	129	0.0149	0.152	1	2	ADA
Pubmed	17406941	Association of HLA class II genes with idiopathic pulmonary arterial hypertension in Koreans.	GeneRIF	0.00875	129	0.0149	0.152	1	2	HLA-DQB1
Pubmed	15661757	Localization and functional analysis of the LARGE family of glycosyltransferases: significance for muscular dystrophy.	Pubmed	0.00875	129	0.0149	0.152	1	2	LARGE
Pubmed	18644839	MMP-7 cleaves the NR1 NMDA receptor subunit and modifies NMDA receptor function.	GeneRIF	0.00875	129	0.0149	0.152	1	2	MMP7
Pubmed	9298982	Localization of p21-activated kinase 1 (PAK1) to pinocytic vesicles and cortical actin structures in stimulated cells.	Pubmed	0.00875	129	0.0149	0.152	1	2	ACTA1
Pubmed	20668227	ATP secreted by endothelial cells blocks CX3CL 1 elicited natural killer cell chemotaxis and cytotoxicity via P2Y11 receptor activation.	GeneRIF	0.00875	129	0.0149	0.152	1	2	CX3CL1
Pubmed	14669136	Mapping multiple sclerosis susceptibility to the HLA-DR locus in African Americans.	GeneRIF	0.00875	129	0.0149	0.152	1	2	HLA-DQB1
Pubmed	15858214	Regulation of neonatal Sertoli cell development by thyroid hormone receptor alpha1.	Pubmed	0.00875	129	0.0149	0.152	1	2	THRA
Pubmed	17906106	Atherogenic vascular and lipid phenotypes in young patients with Type 1 diabetes are associated with diabetes high-risk HLA genotype.	GeneRIF	0.00875	129	0.0149	0.152	1	2	HLA-DQB1
Pubmed	9390561	Identification and molecular characterization of fractalkine receptor CX3CR1, which mediates both leukocyte migration and adhesion.	Pubmed	0.00875	129	0.0149	0.152	1	2	CX3CL1
Pubmed	16988007	Association of selective HLA class II susceptibility conferring and protective haplotypes with type 2 diabetes in patients from Bahrain and Lebanon.	GeneRIF	0.00875	129	0.0149	0.152	1	2	HLA-DQB1
Pubmed	11829448	Involvement of tissue transglutaminase in the stabilisation of biomaterial/tissue interfaces important in medical devices.	Pubmed	0.00875	129	0.0149	0.152	1	2	TGM2
Pubmed	19114539	The skeletal phenotypes of TRalpha and TRbeta mutant mice.	GeneRIF	0.00875	129	0.0149	0.152	1	2	THRA
Pubmed	22639377	High expression of CX3CL1/CX3CR1 axis predicts a poor prognosis of pancreatic ductal adenocarcinoma.	GeneRIF	0.00875	129	0.0149	0.152	1	2	CX3CL1
Pubmed	20826790	Vasodilator-stimulated phosphoprotein (VASP) induces actin assembly in dendritic spines to promote their development and potentiate synaptic strength.	GeneRIF	0.00875	129	0.0149	0.152	1	2	ACTA1
Pubmed	18095157	Decreased fractalkine and increased IP-10 expression in aged brain of APP(swe) transgenic mice.	GeneRIF	0.00875	129	0.0149	0.152	1	2	CX3CL1
Pubmed	14607920	Antigenic epitopes fused to cationic peptide bound to oligonucleotides facilitate Toll-like receptor 9-dependent, but CD4+ T cell help-independent, priming of CD8+ T cells.	Pubmed	0.00875	129	0.0149	0.152	1	2	HLA-DQB1

Category	ID	Name	Source	p-value	q-value Bonferroni	q-value FDR B&H	q-value FDR B&Y	Hit Count in Query List	Hit Count in Genome	Hit in Query List
Pubmed	11812768	Asian-specific HLA haplotypes reveal heterogeneity of the contribution of HLA-DR and -DQ haplotypes to susceptibility to type 1 diabetes.	GeneRIF	0.00875	129	0.0149	0.152	1	2	HLA-DQB1
Pubmed	20697023	Deficiency of antigen-presenting cell invariant chain reduces atherosclerosis in mice.	Pubmed	0.00875	129	0.0149	0.152	1	2	CD74
Pubmed	19224915	Saposin B-dependent reconstitution of arylsulfatase A activity in vitro and in cell culture models of metachromatic leukodystrophy.	GeneRIF	0.00875	129	0.0149	0.152	1	2	PSAP
Pubmed	24977291	Factor VIIa binding to endothelial cell protein C receptor protects vascular barrier integrity in vivo.	Pubmed	0.00875	129	0.0149	0.152	1	2	PROCR
Pubmed	21127009	Impaired muscle growth and response to insulin-like growth factor 1 in dysferlin-mediated muscular dystrophy.	Pubmed	0.00875	129	0.0149	0.152	1	2	DYSF
Pubmed	12586611	Activated protein C alters cytosolic calcium flux in human brain endothelium via binding to endothelial protein C receptor and activation of protease activated receptor-1.	GeneRIF	0.00875	129	0.0149	0.152	1	2	PROCR
Pubmed	16158194	Population frequency of HLA haplotypes contributes to the prevalence difference of multiple sclerosis in Ireland.	GeneRIF	0.00875	129	0.0149	0.152	1	2	HLA-DQB1
Pubmed	9268494	Allospecific cytotoxic T cells generated from beta 2m-/- mice in primary MLC: analysis of activation requirements, specificity, and phenotype.	Pubmed	0.00875	129	0.0149	0.152	1	2	HLA-DQB1
Pubmed	18680509	The four and a half LIM domain protein 2 interacts with and regulates the HERG channel.	GeneRIF	0.00875	129	0.0149	0.152	1	2	KCNH2
Pubmed	18951049	Molecular, hematological and clinical aspects of thalassemia major and thalassemia intermedia associated with Hb E-beta-thalassemia in Northeast Thailand.	GeneRIF	0.00875	129	0.0149	0.152	1	2	HBB
Pubmed	15579768	Coexpression of fractalkine and its receptor in normal human endometrium and in endometrium from users of progestin-only contraception supports a role for fractalkine in leukocyte recruitment and endometrial remodeling.	GeneRIF	0.00875	129	0.0149	0.152	1	2	CX3CL1
Pubmed	18587495	Chordin-like 1, a bone morphogenetic protein-4 antagonist, is upregulated by hypoxia in human retinal pericytes and plays a role in regulating angiogenesis.	GeneRIF	0.00875	129	0.0149	0.152	1	2	BMP4
Pubmed	18056116	Aldo-keto reductase family 1 B10 affects fatty acid synthesis by regulating the stability of acetyl-CoA carboxylase-alpha in breast cancer cells.	GeneRIF	0.00875	129	0.0149	0.152	1	2	AKR1B10
Pubmed	23289476	Expression of M2-polarized macrophages is associated with poor prognosis for advanced epithelial ovarian cancer.	Pubmed	0.00875	129	0.0149	0.152	1	2	CD68
Pubmed	20042427	Loss-of-function mutation of the SCN3B-encoded sodium channel {beta}3 subunit associated with a case of idiopathic ventricular fibrillation.	GeneRIF	0.00875	129	0.0149	0.152	1	2	SCN3B
Pubmed	23873798	Increased tissue transglutaminase activity contributes to central vascular stiffness in eNOS knockout mice.	Pubmed	0.00875	129	0.0149	0.152	1	2	TGM2
Pubmed	25096180	Skeletal muscle atrophy and the E3 ubiquitin ligases MuRF1 and MAFbx/atrogin-1.	Pubmed	0.00875	129	0.0149	0.152	1	2	FBXO32
Pubmed	20405035	Dysferlin interacts with tubulin and microtubules in mouse skeletal muscle.	GeneRIF	0.00875	129	0.0149	0.152	1	2	DYSF
Pubmed	18815226	Regulation of cell proliferation by intermediate-conductance Ca2+-activated potassium and volume-sensitive chloride channels in mouse mesenchymal stem cells.	GeneRIF	0.00875	129	0.0149	0.152	1	2	KCNN4
Pubmed	12770797	HLA class II and TNF genes in African Americans from the Southeastern United States: regional differences in allele frequencies.	GeneRIF	0.00875	129	0.0149	0.152	1	2	HLA-DQB1
Pubmed	9275097	Cholecystokinin (CCK) levels are greatly reduced in the brains but not the duodenums of Cpe(fat)/Cpe(fat) mice: a regional difference in the involvement of carboxypeptidase E (Cpe) in pro-CCK processing.	Pubmed	0.00875	129	0.0149	0.152	1	2	CPE
Pubmed	21199870	Single particle tracking confirms that multivalent Tat protein transduction domain-induced heparan sulfate proteoglycan cross-linkage activates Rac1 for internalization.	GeneRIF	0.00875	129	0.0149	0.152	1	2	ACTA1
Pubmed	17123807	The consequence of PRELP overexpression on skin.	GeneRIF	0.00875	129	0.0149	0.152	1	2	PRELP
Pubmed	7737291	Intrathymic T cell receptor (TcR) targeting in mice lacking CD4 or major histocompatibility complex (MHC) class II: rescue of CD4 T cell lineage without co-engagement of TcR/CD4 by MHC class II.	Pubmed	0.00875	129	0.0149	0.152	1	2	HLA-DQB1
Pubmed	21164565	Overlapping LQT1 and LQT2 phenotype in a patient with long QT syndrome associated with loss-of-function variations in KCNQ1 and KCNH2.	GeneRIF	0.00875	129	0.0149	0.152	1	2	KCNH2
Pubmed	12144623	HLA-DRB1 and HLA-DQB1 polymorphisms in Pacific Islands populations.	GeneRIF	0.00875	129	0.0149	0.152	1	2	HLA-DQB1
Pubmed	15173170	Binding and kinetic mechanisms of the Zeta class glutathione transferase.	Pubmed	0.00875	129	0.0149	0.152	1	2	GSS
Pubmed	12890388	Association between HLA-DQA1, -DQB1 gene polymorphisms and susceptibility to asthma in northern Chinese subjects.	GeneRIF	0.00875	129	0.0149	0.152	1	2	HLA-DQB1
Pubmed	12235085	Histological features and HLA class II alleles in hepatitis C virus chronically infected patients with persistently normal alanine aminotransferase levels.	GeneRIF	0.00875	129	0.0149	0.152	1	2	HLA-DQB1
Pubmed	15248208	Association between HLA class II genes and autoantibodies to cyclic citrullinated peptides (CCPs) influences the severity of rheumatoid arthritis.	GeneRIF	0.00875	129	0.0149	0.152	1	2	HLA-DQB1
Pubmed	10932194	Insertion of Inhbb into the Inhba locus rescues the Inhba-null phenotype and reveals new activin functions.	Pubmed	0.00875	129	0.0149	0.152	1	2	INHBB
Pubmed	9529148	Crystal structure of I-Ak in complex with a dominant epitope of lysozyme.	Pubmed	0.00875	129	0.0149	0.152	1	2	HLA-DQB1

Category	ID	Name	Source	p-value	q-value Bonferroni	q-value FDR B&H	q-value FDR B&Y	Hit Count in Query List	Hit Count in Genome	Hit in Query List
Pubmed	18535624	Off-response property of an acid-activated cation channel complex PKD1L3-PKD2L1.	GeneRIF	0.00875	129	0.0149	0.152	1	2	PKD2L1
Pubmed	18272668	HLA class II haplotypes distinctly associated with vaso-occlusion in children with sickle cell disease.	GeneRIF	0.00875	129	0.0149	0.152	1	2	HLA-DQB1
Pubmed	18722491	Human leukocyte antigen class II allele association to disease progression in Iranian patients with chronic lymphocytic leukemia.	GeneRIF	0.00875	129	0.0149	0.152	1	2	HLA-DQB1
Pubmed	18923542	The regulation of the cardiac potassium channel (HERG) by caveolin-1.	GeneRIF	0.00875	129	0.0149	0.152	1	2	KCNH2
Pubmed	17017992	Matrix metalloproteinase (MMP)-7 activates MMP-8 but not MMP-13.	GeneRIF	0.00875	129	0.0149	0.152	1	2	MMP7
Pubmed	2531377	Endopeptidase-24.11 in human plasma degrades atrial natriuretic factor (ANF) to ANF(99-105/106-126).	Pubmed	0.00875	129	0.0149	0.152	1	2	NPPA
Pubmed	22860046	Cathepsin K-Cre causes unexpected germline deletion of genes in mice.	GeneRIF	0.00875	129	0.0149	0.152	1	2	CTSK
Pubmed	12192052	MLL-AFX requires the transcriptional effector domains of AFX to transform myeloid progenitors and transdominantly interfere with forkhead protein function.	GeneRIF	0.00875	129	0.0149	0.152	1	2	FOXO4
Pubmed	10473130	Dynamic expression of TSC-22 at sites of epithelial-mesenchymal interactions during mouse development.	Pubmed	0.00875	129	0.0149	0.152	1	2	BMP4
Pubmed	9774466	Induction of thymocyte apoptosis by Ca2+-independent protein kinase C (nPKC) activation and its regulation by calcineurin activation.	Pubmed	0.00875	129	0.0149	0.152	1	2	HLA-DQB1
Pubmed	15681836	Pro-NGF isolated from the human brain affected by Alzheimer's disease induces neuronal apoptosis mediated by p75NTR.	GeneRIF	0.00875	129	0.0149	0.152	1	2	NGFR
Pubmed	17166701	Retinoic acid regulation of eye and testis-specific transcripts within a complex locus.	Pubmed	0.00875	129	0.0149	0.152	1	2	ZBTB7C
Pubmed	16011982	Analysis of B- and T-cell clonality and HLA class II alleles in patients with idiopathic thrombocytopenic purpura: correlation with Helicobacter pylori infection and response to eradication treatment.	GeneRIF	0.00875	129	0.0149	0.152	1	2	HLA-DQB1
Pubmed	22895561	Glycosaminoglycan chains of biglycan promote bone morphogenetic protein-4-induced osteoblast differentiation.	GeneRIF	0.00875	129	0.0149	0.152	1	2	BMP4
Pubmed	20456446	LOC387715/HTRA1 polymorphisms, smoking and combined effects on exudative age-related macular degeneration in a Korean population.	GeneRIF	0.00875	129	0.0149	0.152	1	2	HTRA1
Pubmed	7558133	Increased number of CD4-CD8+ MHC class II-specific T cells in MHC class II-deficient mice.	Pubmed	0.00875	129	0.0149	0.152	1	2	HLA-DQB1
Pubmed	10798362	The spectrum of mutations in erythrokeratodermias -novel and de novo mutations in GJB3.	Pubmed	0.00875	129	0.0149	0.152	1	2	GJB3
Pubmed	24913494	A hormone-dependent feedback-loop controls androgen receptor levels by limiting MID1, a novel translation enhancer and promoter of oncogenic signaling.	Pubmed	0.00875	129	0.0149	0.152	1	2	MID1
Pubmed	15835269	Dysferlin interacts with affixin (beta-parvin) at the sarcolemma.	GeneRIF	0.00875	129	0.0149	0.152	1	2	DYSF
Pubmed	7613473	Identification of the binding and activating sites of the sphingolipid activator protein, saposin C, with glucocerebrosidase.	Pubmed	0.00875	129	0.0149	0.152	1	2	PSAP
Pubmed	18700785	Mechanism of insolubilization by a single-point mutation in alphaA-crystallin linked with hereditary human cataracts.	GeneRIF	0.00875	129	0.0149	0.152	1	2	CRYAA
Pubmed	24469108	CD74-NRG1 Fusions in Lung Adenocarcinoma.	Pubmed	0.00875	129	0.0149	0.152	1	2	CD74
Pubmed	25180603	Neurotrophin receptor p75(NTR) mediates Huntington's disease-associated synaptic and memory dysfunction.	Pubmed	0.00875	129	0.0149	0.152	1	2	NGFR
Pubmed	18523305	Transamidation by transglutaminase 2 transforms S100A11 calgranulin into a procatabolic cytokine for chondrocytes.	GeneRIF	0.00875	129	0.0149	0.152	1	2	TGM2
Pubmed	15653504	CX3CR1-mediated dendritic cell access to the intestinal lumen and bacterial clearance.	GeneRIF	0.00875	129	0.0149	0.152	1	2	HLA-DQB1
Pubmed	9251807	Actin-titin interaction in cardiac myofibrils: probing a physiological role.	Pubmed	0.00875	129	0.0149	0.152	1	2	ACTA1
Pubmed	11049973	T-lymphocyte production of macrophage inflammatory protein-1alpha is critical to the recruitment of CD8(+) T cells to the liver, lung, and spleen during graft-versus-host disease.	Pubmed	0.00875	129	0.0149	0.152	1	2	HLA-DQB1
Pubmed	22372829	Influence of endothelial cell protein C receptor on plasma clearance of factor VIIa.	GeneRIF	0.00875	129	0.0149	0.152	1	2	PROCR
Pubmed	15131262	Aberrant dynamics of histone deacetylation at the thyrotropin-releasing hormone gene in resistance to thyroid hormone.	Pubmed	0.00875	129	0.0149	0.152	1	2	THRA
Pubmed	17709382	Histone deacetylase inhibitors reduce steroidogenesis through SCF-mediated ubiquitination and degradation of steroidogenic factor 1 (NR5A1).	GeneRIF	0.00875	129	0.0149	0.152	1	2	CYP11A1
Pubmed	14690244	Identification of gp17 glycoprotein and characterization of prostatic acid phosphatase (PAP) and carboxypeptidase E (CPE) fragments in a human seminal plasma fraction interacting with concanavalin A.	GeneRIF	0.00875	129	0.0149	0.152	1	2	CPE
Pubmed	15322189	Dissecting MHC class II export, B cell maturation, and DM stability defects in invariant chain mutant mice.	Pubmed	0.00875	129	0.0149	0.152	1	2	CD74
Pubmed	7373648	The structure of human carbonmonoxy haemoglobin at 2.7 A resolution.	Pubmed	0.00875	129	0.0149	0.152	1	2	HBB
Pubmed	12213803	Deciphering vascular endothelial cell growth factor/vascular permeability factor signaling to vascular permeability. Inhibition by atrial natriuretic peptide.	GeneRIF	0.00875	129	0.0149	0.152	1	2	NPPA
Pubmed	16604076	The generation of protective memory-like CD8+ T cells during homeostatic proliferation requires CD4+ T cells.	Pubmed	0.00875	129	0.0149	0.152	1	2	HLA-DQB1
Pubmed	20031640	Single nucleotide polymorphisms in homocysteine metabolism pathway genes: association of CHDH A119C and MTHFR C677T with hyperhomocysteinemia.	GeneRIF	0.00875	129	0.0149	0.152	1	2	CHDH

Category	ID	Name	Source	p-value	q-value Bonferroni	q-value FDR B&H	q-value FDR B&Y	Hit Count in Query List	Hit Count in Genome	Hit in Query List
Pubmed	21868565	Evaluation of kallikrein-related peptidase 5 expression and its significance for breast cancer patients: association with kallikrein-related peptidase 7 expression.	GeneRIF	0.00875	129	0.0149	0.152	1	2	KLK7
Pubmed	19780863	Lipoprotein lipase and endothelial lipase in human testis and in germ cell neoplasms.	GeneRIF	0.00875	129	0.0149	0.152	1	2	LIPG
Pubmed	22799313	Association between HLA-DQ genotypes and haplotypes vs Helicobacter pylori infection in an Indonesian population.	GeneRIF	0.00875	129	0.0149	0.152	1	2	HLA-DQB1
Pubmed	20680712	Prognostic impact of ERß and MMP7 expression on overall survival in colon cancer.	GeneRIF	0.00875	129	0.0149	0.152	1	2	MMP7
Pubmed	18480258	Activin B receptor ALK7 is a negative regulator of pancreatic beta-cell function.	GeneRIF	0.00875	129	0.0149	0.152	1	2	INHBB
Pubmed	18854834	Co-expression of KLK6 and KLK10 as prognostic factors for survival in pancreatic ductal adenocarcinoma.	GeneRIF	0.00875	129	0.0149	0.152	1	2	KLK10
Pubmed	17962403	Structural and functional analysis of the PDZ domains of human HtrA1 and HtrA3.	GeneRIF	0.00875	129	0.0149	0.152	1	2	HTRA1
Pubmed	20231279	Dual roles of smad proteins in the conversion from myoblasts to osteoblastic cells by bone morphogenetic proteins.	GeneRIF	0.00875	129	0.0149	0.152	1	2	BMP4
Pubmed	11891182	Up-regulation of MHC class I expression accompanies but is not required for spontaneous myopathy in dysferlin-deficient SJL/J mice.	GeneRIF	0.00875	129	0.0149	0.152	1	2	DYSF
Pubmed	17890485	Expression and secretion of the atrial natriuretic peptide in human adipose tissue and preadipocytes.	GeneRIF	0.00875	129	0.0149	0.152	1	2	NPPA
Pubmed	11463821	Disruption of gamma-glutamyl leukotrienase results in disruption of leukotriene D(4) synthesis in vivo and attenuation of the acute inflammatory response.	Pubmed	0.00875	129	0.0149	0.152	1	2	GGT1
Pubmed	15602651	High frequency of abnormal glucose tolerance in DQA1*0102/DQB1*0602 relatives identified as part of the Diabetes Prevention Trial--Type 1 Diabetes.	GeneRIF	0.00875	129	0.0149	0.152	1	2	HLA-DQB1
Pubmed	24719048	Pro-atherogenic shear stress and HIV proteins synergistically upregulate cathepsin K in endothelial cells.	Pubmed	0.00875	129	0.0149	0.152	1	2	CTSK
Pubmed	18003857	Fractalkine receptor (CX3CR1) inhibition is protective against ischemic acute renal failure in mice.	GeneRIF	0.00875	129	0.0149	0.152	1	2	CX3CL1
Pubmed	18195387	Ca2+-activated K+ channels in murine endothelial cells: block by intracellular calcium and magnesium.	GeneRIF	0.00875	129	0.0149	0.152	1	2	KCNN4
Pubmed	17956579	Autoantibody epitopes to the smaller isoform of glutamate decarboxylase do not differ in Swedish and Japanese type 1 diabetes patients and may be associated with high-risk human leucocyte antigen class II alleles.	GeneRIF	0.00875	129	0.0149	0.152	1	2	HLA-DQB1
Pubmed	7357610	Secondary structure of mouse and rabbit alpha- and beta-globin mRNAs: differential accessibility of alpha and beta initiator AUG codons towards nucleases.	Pubmed	0.00875	129	0.0149	0.152	1	2	HBA2
Pubmed	23277474	Induced pluripotent stem cells used to reveal drug actions in a long QT syndrome family with complex genetics.	GeneRIF	0.00875	129	0.0149	0.152	1	2	KCNH2
Pubmed	16864790	Regulation of T cell activation and tolerance by PDL2.	GeneRIF	0.00875	129	0.0149	0.152	1	2	CD276
Pubmed	22508689	Upregulation of proteasome activity in muscle RING finger 1-null mice following denervation.	GeneRIF	0.00875	129	0.0149	0.152	1	2	FBXO32
Pubmed	21555591	Control of mTORC1 signaling by the Opitz syndrome protein MID1.	GeneRIF	0.00875	129	0.0149	0.152	1	2	MID1
Pubmed	7842012	Two long QT syndrome loci map to chromosomes 3 and 7 with evidence for further heterogeneity.	Pubmed	0.00875	129	0.0149	0.152	1	2	KCNH2
Pubmed	19840952	Fractalkine has anti-apoptotic and proliferative effects on human vascular smooth muscle cells via epidermal growth factor receptor signalling.	GeneRIF	0.00875	129	0.0149	0.152	1	2	CX3CL1
Pubmed	12782713	MIF signal transduction initiated by binding to CD74.	GeneRIF	0.00875	129	0.0149	0.152	1	2	CD74
Pubmed	18772344	CX3CL1 up-regulation is associated with recruitment of CX3CR1+ mononuclear phagocytes and T lymphocytes in the lungs during cigarette smoke-induced emphysema.	GeneRIF	0.00875	129	0.0149	0.152	1	2	CX3CL1
Pubmed	18590565	Evolutionary analyses of KCNQ1 and HERG voltage-gated potassium channel sequences reveal location-specific susceptibility and augmented chemical severities of arrhythmogenic mutations.	GeneRIF	0.00875	129	0.0149	0.152	1	2	KCNH2
Pubmed	8929537	Failure of embryonic hematopoiesis and lethal hemorrhages in mouse embryos heterozygous for a knocked-in leukemia gene CBFB-MYH11.	Pubmed	0.00875	129	0.0149	0.152	1	2	MYH11
Pubmed	24051141	Targeted gene sequencing identifies variants in the protein C and endothelial protein C receptor genes in patients with unprovoked venous thromboembolism.	Pubmed	0.00875	129	0.0149	0.152	1	2	PROCR
Pubmed	11884416	Processing of pro-atrial natriuretic peptide by corin in cardiac myocytes.	GeneRIF	0.00875	129	0.0149	0.152	1	2	NPPA
Pubmed	8663190	Four distinct membrane-bound dipeptidase RNAs are differentially expressed and show discordant regulation with gamma-glutamyl transpeptidase.	Pubmed	0.00875	129	0.0149	0.152	1	2	GGT1
Pubmed	25280803	Association between serum ¿-glutamyltranspeptidase and atherosclerosis: a population-based cross-sectional study.	Pubmed	0.00875	129	0.0149	0.152	1	2	GGT1
Pubmed	24673558	The ability of thyroid hormone receptors to sense t4 as an agonist depends on receptor isoform and on cellular cofactors.	Pubmed	0.00875	129	0.0149	0.152	1	2	THRA
Pubmed	20685690	Sarcoidosis HLA class II genotyping distinguishes differences of clinical phenotype across ethnic groups.	GeneRIF	0.00875	129	0.0149	0.152	1	2	HLA-DQB1
Pubmed	7673243	Identification of a human type II receptor for bone morphogenetic protein-4 that forms differential heteromeric complexes with bone morphogenetic protein type I receptors.	Pubmed	0.00875	129	0.0149	0.152	1	2	BMP4
Pubmed	16234023	HLA-DQA1*0103-DQB1*0601 haplotype and Helicobacter pylori-positive gastric mucosa-associated lymphoid tissue lymphoma.	GeneRIF	0.00875	129	0.0149	0.152	1	2	HLA-DQB1

Category	ID	Name	Source	p-value	q-value Bonferroni	q-value FDR B&H	q-value FDR B&Y	Hit Count in Query List	Hit Count in Genome	Hit in Query List
Pubmed	15073633	C677T polymorphism of the MTHFR gene and variant hemoglobins: a study in newborns from Salvador, Bahia, Brazil.	GeneRIF	0.00875	129	0.0149	0.152	1	2	HBB
Pubmed	23031786	Significantly upregulated TACSTD2 and Cyclin D1 correlate with poor prognosis of invasive ducta breast cancer.	GeneRIF	0.00875	129	0.0149	0.152	1	2	TACSTD2
Pubmed	23454123	Repression of bone morphogenetic protein 4 by let-7i attenuates mesenchymal migration of head and neck cancer cells.	GeneRIF	0.00875	129	0.0149	0.152	1	2	BMP4
Pubmed	24236063	Identification and characterization of angiogenesis targets through proteomic profiling of endothelial cells in human cancer tissues.	Pubmed	0.00875	129	0.0149	0.152	1	2	CD276
Pubmed	24336288	Primary cilia are specialized calcium signalling organelles.	Pubmed	0.00875	129	0.0149	0.152	1	2	PKD2L1
Pubmed	22127230	Intake of branched-chain amino acids influences the levels of MAFbx mRNA and MuRF-1 total protein in resting and exercising human muscle.	GeneRIF	0.00875	129	0.0149	0.152	1	2	FBXO32
Pubmed	15482472	Progressive hair loss and myocardial degeneration in rough coat mice: reduced lysyl oxidase-like (LOXL) in the skin and heart.	GeneRIF	0.00875	129	0.0149	0.152	1	2	MPZL3
Pubmed	6452630	Cloning and complete nucleotide sequence of human 5'-alpha-globin gene.	Pubmed	0.00875	129	0.0149	0.152	1	2	HBA2
Pubmed	19439611	Identification of BLyS (B lymphocyte stimulator), a non-myelin-associated protein, as a functional ligand for Nogo-66 receptor.	GeneRIF	0.00875	129	0.0149	0.152	1	2	RTN4R
Pubmed	24789789	Investigating the role of F-actin in human immunodeficiency virus assembly by live-cell microscopy.	Pubmed	0.00875	129	0.0149	0.152	1	2	ACTA1
Pubmed	15630442	A protective role for the A1 adenosine receptor in adenosine-dependent pulmonary injury.	GeneRIF	0.00875	129	0.0149	0.152	1	2	ADA
Pubmed	25287946	Angiopoietin-like protein 2 renders colorectal cancer cells resistant to chemotherapy by activating spleen tyrosine kinase-phosphoinositide 3-kinase-dependent anti-apoptotic signaling.	Pubmed	0.00875	129	0.0149	0.152	1	2	ANGPTL2
Pubmed	10022822	Crystal structure of MHC class II-associated p41 Ii fragment bound to cathepsin L reveals the structural basis for differentiation between cathepsins L and S.	Pubmed	0.00875	129	0.0149	0.152	1	2	CD74
Pubmed	11099506	Identification of the protein C/activated protein C binding sites on the endothelial cell protein C receptor. Implications for a novel mode of ligand recognition by a major histocompatibility complex class 1-type receptor.	Pubmed	0.00875	129	0.0149	0.152	1	2	PROCR
Pubmed	15630456	Endothelial lipase provides an alternative pathway for FFA uptake in lipoprotein lipase-deficient mouse adipose tissue.	GeneRIF	0.00875	129	0.0149	0.152	1	2	LIPG
Pubmed	16798289	Ovarian enzyme activities in women with polycystic ovary syndrome.	GeneRIF	0.00875	129	0.0149	0.152	1	2	CYP11A1
Pubmed	12083823	Role of G protein beta3 subunit C825T and HLA class II polymorphisms in the immune response after HBV vaccination.	GeneRIF	0.00875	129	0.0149	0.152	1	2	HLA-DQB1
Pubmed	7541633	Differential role of the low affinity neurotrophin receptor (p75) in retrograde axonal transport of the neurotrophins.	Pubmed	0.00875	129	0.0149	0.152	1	2	NGFR
Pubmed	25075639	A Rac1-independent role for P-Rex1 in melanoblasts.	Pubmed	0.00875	129	0.0149	0.152	1	2	PREX1
Pubmed	16528383	Tissue transglutaminase (TG2) protects cardiomyocytes against ischemia/reperfusion injury by regulating ATP synthesis.	GeneRIF	0.00875	129	0.0149	0.152	1	2	TGM2
Pubmed	18774102	Identification of large gene deletions and duplications in KCNQ1 and KCNH2 in patients with long QT syndrome.	GeneRIF	0.00875	129	0.0149	0.152	1	2	KCNH2
Pubmed	24657443	Identification of prosaposin and transgelin as potential biomarkers for gallbladder cancer using quantitative proteomics.	Pubmed	0.00875	129	0.0149	0.152	1	2	PSAP
Pubmed	22383492	Oncogenic KRAS regulates BMP4 expression in colon cancer cell lines.	GeneRIF	0.00875	129	0.0149	0.152	1	2	BMP4
Pubmed	15109436	Association of HLA-DQB1 coding region with unexplained recurrent spontaneous abortion.	GeneRIF	0.00875	129	0.0149	0.152	1	2	HLA-DQB1
Pubmed	19893004	Fibulin-2 and fibulin-5 cooperatively function to form the internal elastic lamina and protect from vascular injury.	GeneRIF	0.00875	129	0.0149	0.152	1	2	FBLN2
Pubmed	19360357	Three-dimensional culture using a radial flow bioreactor induces matrix metalloprotease 7-mediated EMT-like process in tumor cells via TGFbeta1/Smad pathway.	GeneRIF	0.00875	129	0.0149	0.152	1	2	MMP7
Pubmed	9668039	Frabin, a novel FGD1-related actin filament-binding protein capable of changing cell shape and activating c-Jun N-terminal kinase.	Pubmed	0.00875	129	0.0149	0.152	1	2	ACTA1
Pubmed	9695791	Gene sharing in lens and cornea: facts and implications.	Pubmed	0.00875	129	0.0149	0.152	1	2	CRYAA
Pubmed	2493384	The expression of I-A correlates with the uptake of interferon-gamma by macrophages.	Pubmed	0.00875	129	0.0149	0.152	1	2	HLA-DQB1
Pubmed	17971512	Repeated resistance exercise training induces different changes in mRNA expression of MAFbx and MuRF-1 in human skeletal muscle.	GeneRIF	0.00875	129	0.0149	0.152	1	2	FBXO32
Pubmed	21387310	HtrA1 sensitizes ovarian cancer cells to cisplatin-induced cytotoxicity by targeting XIAP for degradation.	GeneRIF	0.00875	129	0.0149	0.152	1	2	HTRA1
Pubmed	21814482	Dimerumic acid inhibits SW620 cell invasion by attenuating H2O2-mediated MMP-7 expression via JNK/C-Jun and ERK/C-Fos activation in an AP-1-dependent manner.	GeneRIF	0.00875	129	0.0149	0.152	1	2	MMP7
Pubmed	16928883	Comparison of human immunodeficiency virus type 1-specific inhibitory activities in saliva and other human mucosal fluids.	Pubmed	0.00875	129	0.0149	0.152	1	2	SLPI
Pubmed	25651370	Association of HLA-DR/DQ polymorphism with Alzheimer's disease.	Pubmed	0.00875	129	0.0149	0.152	1	2	HLA-DQB1
Pubmed	20498017	The role of UDP-Glc:glycoprotein glucosyltransferase 1 in the maturation of an obligate substrate prosaposin.	GeneRIF	0.00875	129	0.0149	0.152	1	2	PSAP
Pubmed	11130770	Altered frequency of a promoter polymorphism of the kinin B2 receptor gene in hypertensive African-Americans.	GeneRIF	0.00875	129	0.0149	0.152	1	2	KLK1

Category	ID	Name	Source	p-value	q-value Bonferroni	q-value FDR B&H	q-value FDR B&Y	Hit Count in Query List	Hit Count in Genome	Hit in Query List
Pubmed	20051322	Molecular identification of the HLA-DRB1-DQB1 for diagnosis and follow-up of acute leukemias.	GeneRIF	0.00875	129	0.0149	0.152	1	2	HLA-DQB1
Pubmed	24081251	Clinical presentation and molecular identification of four uncommon alpha globin variants in Thailand. Initiation codon mutation of a2-globin Gene (HBA2:c.1delA), donor splice site mutation of a1-globin gene (IVSI-1, HBA1:c.95 + 1G>A), hemoglobin Queens Park/Chao Pra Ya (HBA1:c.98T>A) and hemoglobin Westmead (HBA2:c.369C>G).	Pubmed	0.00875	129	0.0149	0.152	1	2	HBA2
Pubmed	15536412	Genetic basis of the latex-fruit syndrome: association with HLA class II alleles in a Spanish population.	GeneRIF	0.00875	129	0.0149	0.152	1	2	HLA-DQB1
Pubmed	12617848	Expression profiles of the connexin genes, Gjb1 and Gjb3, in the developing mouse cochlea.	GeneRIF	0.00875	129	0.0149	0.152	1	2	GJB3
Pubmed	571871	Nerve growth factor receptors. Characterization of two distinct classes of binding sites on chick embryo sensory ganglia cells.	Pubmed	0.00875	129	0.0149	0.152	1	2	NGFR
Pubmed	12464266	Identification of novel matrix metalloproteinase-7 (matrilysin) cleavage sites in murine and human Fas ligand.	GeneRIF	0.00875	129	0.0149	0.152	1	2	MMP7
Pubmed	20876521	In situ detection of active transglutaminases for keratinocyte type (TGase 1) and tissue type (TGase 2) using fluorescence-labeled highly reactive substrate peptides.	Pubmed	0.00875	129	0.0149	0.152	1	2	TGM2
Pubmed	11602620	Requirement for endocytic antigen processing and influence of invariant chain and H-2M deficiencies in CNS autoimmunity.	Pubmed	0.00875	129	0.0149	0.152	1	2	CD74
Pubmed	11679920	Human leukocyte antigen class I and II alleles and risk of cervical neoplasia: results from a population-based study in Costa Rica.	GeneRIF	0.00875	129	0.0149	0.152	1	2	HLA-DQB1
Pubmed	15181150	The RCP-Rab11 complex regulates endocytic protein sorting.	GeneRIF	0.00875	129	0.0149	0.152	1	2	RAB11FIP1
Pubmed	22691502	Revised mechanism of complement lectin-pathway activation revealing the role of serine protease MASP-1 as the exclusive activator of MASP-2.	GeneRIF	0.00875	129	0.0149	0.152	1	2	MASP1
Pubmed	10702419	Anti-inflammatory effects of mutant forms of secretory leukocyte protease inhibitor.	Pubmed	0.00875	129	0.0149	0.152	1	2	SLPI
Pubmed	11731621	Identification, localization, and function in steroidogenesis of PAP7: a peripheral-type benzodiazepine receptor- and PKA (RIalpha)-associated protein.	Pubmed	0.00875	129	0.0149	0.152	1	2	TSPO
Pubmed	22490543	[Analysis of the binding domain of hydroxypyruvate isomerase homologues in hypertrophic scar fibroblasts].	GeneRIF	0.00875	129	0.0149	0.152	1	2	HYI
Pubmed	12447731	Human leukocyte antigen class II and cervical cancer risk: a population-based study.	GeneRIF	0.00875	129	0.0149	0.152	1	2	HLA-DQB1
Pubmed	8643655	Biosynthesis of major histocompatibility complex molecules and generation of T cells in Ii TAP1 double-mutant mice.	Pubmed	0.00875	129	0.0149	0.152	1	2	CD74
Pubmed	17965779	Atrogin-1 inhibits Akt-dependent cardiac hypertrophy in mice via ubiquitin-dependent coactivation of Forkhead proteins.	GeneRIF	0.00875	129	0.0149	0.152	1	2	FBXO32
Pubmed	12890912	Differential increase in natriuretic peptides in elite dynamic and static athletes.	GeneRIF	0.00875	129	0.0149	0.152	1	2	NPPA
Pubmed	17028893	Modulation of cell proliferation during palatogenesis by the interplay between Tbx3 and Bmp4.	GeneRIF	0.00875	129	0.0149	0.152	1	2	BMP4
Pubmed	20659433	Over-expression of the DCLK gene transcript CARP decreases CA3/CA1 network excitability.	GeneRIF	0.00875	129	0.0149	0.152	1	2	DCLK1
Pubmed	18215522	IRSp53: crossing the road of membrane and actin dynamics in the formation of membrane protrusions.	Pubmed	0.00875	129	0.0149	0.152	1	2	BAIAP2L1
Pubmed	21123999	Inactivation of Bmp4 from the Tbx1 expression domain causes abnormal pharyngeal arch artery and cardiac outflow tract remodeling.	GeneRIF	0.00875	129	0.0149	0.152	1	2	BMP4
Pubmed	8636144	Expression of the smooth muscle cell calponin gene marks the early cardiac and smooth muscle cell lineages during mouse embryogenesis.	Pubmed	0.00875	129	0.0149	0.152	1	2	MYH11
Pubmed	18400893	Beta-amyloid(1-42) induces neuronal death through the p75 neurotrophin receptor.	GeneRIF	0.00875	129	0.0149	0.152	1	2	NGFR
Pubmed	14649316	Hb Setif [alpha94(G1)Asp --> Tyr (alpha2)] detected in a Turkish family.	GeneRIF	0.00875	129	0.0149	0.152	1	2	HBA2
Pubmed	15131306	Structure of nerve growth factor complexed with the shared neurotrophin receptor p75.	GeneRIF	0.00875	129	0.0149	0.152	1	2	NGFR
Pubmed	12414903	Production of activins by the human endosalpinx.	GeneRIF	0.00875	129	0.0149	0.152	1	2	INHBB
Pubmed	19050930	Digenic inheritance of non-syndromic deafness caused by mutations at the gap junction proteins Cx26 and Cx31.	GeneRIF	0.00875	129	0.0149	0.152	1	2	GJB3
Pubmed	20549701	p75(NTR) induces apoptosis in medulloblastoma cells.	GeneRIF	0.00875	129	0.0149	0.152	1	2	NGFR
Pubmed	20491537	Matrilysins may not predict the metastatic potential in squamous cell carcinoma of the tongue.	GeneRIF	0.00875	129	0.0149	0.152	1	2	MMP7
Pubmed	19338980	Role of the interferon-inducible IFI16 gene in the induction of ICAM-1 by TNF-alpha.	GeneRIF	0.00875	129	0.0149	0.152	1	2	IFI16
Pubmed	12233873	HLA-DR/DQ haplotype in rheumatoid arthritis: novel allelic associations in UK Caucasians.	GeneRIF	0.00875	129	0.0149	0.152	1	2	HLA-DQB1
Pubmed	15550245	Molecular mechanism of AHSP-mediated stabilization of alpha-hemoglobin.	Pubmed	0.00875	129	0.0149	0.152	1	2	HBA2
Pubmed	21362572	FGF2 sustains NANOG and switches the outcome of BMP4-induced human embryonic stem cell differentiation.	GeneRIF	0.00875	129	0.0149	0.152	1	2	BMP4
Pubmed	11441185	Ventroptin: a BMP-4 antagonist expressed in a double-gradient pattern in the retina.	GeneRIF	0.00875	129	0.0149	0.152	1	2	BMP4
Pubmed	17512924	Molecular assay of -alpha(3.7) and -alpha(4.2) deletions causing alpha-thalassemia by denaturing high-performance liquid chromatography.	Pubmed	0.00875	129	0.0149	0.152	1	2	HBA2
Pubmed	12584250	Reduced survival of lens epithelial cells in the alphaA-crystallin-knockout mouse.	GeneRIF	0.00875	129	0.0149	0.152	1	2	CRYAA

Category	ID	Name	Source	p-value	q-value Bonferroni	q-value FDR B&H	q-value FDR B&Y	Hit Count in Query List	Hit Count in Genome	Hit in Query List
Pubmed	8097430	Another view of the selective model of thymocyte selection.	Pubmed	0.00875	129	0.0149	0.152	1	2	HLA-DQB1
Pubmed	17163998	Lack of significant association of an insertion/deletion polymorphism in the angiotensin converting enzyme (ACE) gene with tropical calcific pancreatitis.	GeneRIF	0.00875	129	0.0149	0.152	1	2	SPINK1
Pubmed	11808628	Chromosomal mapping of calmodulin 1 (CALM1) and alpha-globin 1 genes (HBA1) in the bovine.	Pubmed	0.00875	129	0.0149	0.152	1	2	HBA2
Pubmed	11833853	Variable severity of beta-thalassemia patients of eastern India: effect of alpha-thalassemia and xmnI polymorphism.	GeneRIF	0.00875	129	0.0149	0.152	1	2	HBB
Pubmed	22178447	Stat1 and CD74 overexpression is co-dependent and linked to increased invasion and lymph node metastasis in triple-negative breast cancer.	GeneRIF	0.00875	129	0.0149	0.152	1	2	CD74
Pubmed	16464953	Specific human leukocyte antigen DQ influence on expression of antiislet autoantibodies and progression to type 1 diabetes.	GeneRIF	0.00875	129	0.0149	0.152	1	2	HLA-DQB1
Pubmed	21073007	Presence and interaction in tissues of atrial natriuretic peptide, oxytocin and vasopressin: new insights.	GeneRIF	0.00875	129	0.0149	0.152	1	2	NPPA
Pubmed	23421483	The DR7-DQ2 haplotype in a native Norwegian population.	GeneRIF	0.00875	129	0.0149	0.152	1	2	HLA-DQB1
Pubmed	17711877	Cell-surface transglutaminase undergoes internalization and lysosomal degradation: an essential role for LRP1.	GeneRIF	0.00875	129	0.0149	0.152	1	2	TGM2
Pubmed	24606882	The impact of IFN-¿ receptor on SLPI expression in active tuberculosis: association with disease severity.	Pubmed	0.00875	129	0.0149	0.152	1	2	SLPI
Pubmed	18022136	Interethnic differences in carriage of haemoglobin AS and Fcgamma receptor IIa (CD32) genotypes in children living in eastern Sudan.	GeneRIF	0.00875	129	0.0149	0.152	1	2	HBB
Pubmed	14988406	Role of hyaluronan and reactive oxygen species in tissue kallikrein-mediated epidermal growth factor receptor activation in human airways.	GeneRIF	0.00875	129	0.0149	0.152	1	2	KLK1
Pubmed	12538710	MHC class II-associated invariant chain isoforms regulate pulmonary immune responses.	GeneRIF	0.00875	129	0.0149	0.152	1	2	CD74
Pubmed	11195230	HLA-DQA1*05-DQB1*0201 positivity predisposes to coeliac disease in Czech diabetic children.	GeneRIF	0.00875	129	0.0149	0.152	1	2	HLA-DQB1
Pubmed	16642438	Sequence and haplotype analysis supports HLA-C as the psoriasis susceptibility 1 gene.	GeneRIF	0.00875	129	0.0149	0.152	1	2	CDSN
Pubmed	23283983	Rab11-family interacting proteins define spatially and temporally distinct regions within the dynamic Rab11a-dependent recycling system.	GeneRIF	0.00875	129	0.0149	0.152	1	2	RAB11FIP1
Pubmed	24967968	Dysferlin regulates cell membrane repair by facilitating injury-triggered acid sphingomyelinase secretion.	Pubmed	0.00875	129	0.0149	0.152	1	2	DYSF
Pubmed	17082664	Modulation of autoimmunity by TLR9 in the chronic graft-vs-host model of systemic lupus erythematosus.	GeneRIF	0.00875	129	0.0149	0.152	1	2	HLA-DQB1
Pubmed	18544639	Factor XIIIA mobilizes transglutaminase 2 to induce chondrocyte hypertrophic differentiation.	GeneRIF	0.00875	129	0.0149	0.152	1	2	TGM2
Pubmed	11867729	Persistence of oligodendrocyte precursor cells and altered myelination in optic nerve associated to retina degeneration in mice devoid of all thyroid hormone receptors.	GeneRIF	0.00875	129	0.0149	0.152	1	2	THRA
Pubmed	19398663	Matrix metalloproteinase-7 and ADAM-12 (a disintegrin and metalloproteinase-12) define a signaling axis in agonist-induced hypertension and cardiac hypertrophy.	GeneRIF	0.00875	129	0.0149	0.152	1	2	MMP7
Pubmed	2539258	Two erbA homologs encoding proteins with different T3 binding capacities are transcribed from opposite DNA strands of the same genetic locus.	Pubmed	0.00875	129	0.0149	0.152	1	2	THRA
Pubmed	20736347	Thyroid hormone receptors are down-regulated in skeletal muscle of patients with non-thyroidal illness syndrome secondary to non-septic shock.	GeneRIF	0.00875	129	0.0149	0.152	1	2	THRA
Pubmed	16361248	Mechanisms of pharmacological rescue of trafficking-defective hERG mutant channels in human long QT syndrome.	Pubmed	0.00875	129	0.0149	0.152	1	2	KCNH2
Pubmed	16940551	Placental triglyceride accumulation in maternal type 1 diabetes is associated with increased lipase gene expression.	GeneRIF	0.00875	129	0.0149	0.152	1	2	LIPG
Pubmed	23936059	Long QT interval in Turner syndrome--a high prevalence of LQTS gene mutations.	Pubmed	0.00875	129	0.0149	0.152	1	2	KCNH2
Pubmed	24283384	MicroRNA-148a is downregulated in gastric cancer, targets MMP7, and indicates tumor invasiveness and poor prognosis.	Pubmed	0.00875	129	0.0149	0.152	1	2	MMP7
Pubmed	21691712	Over-expression of Nkx2.5 and/or cardiac a-actin inhibit the contraction ability of ADSCs-derived cardiomyocytes.	GeneRIF	0.00875	129	0.0149	0.152	1	2	ACTA1
Pubmed	20214740	Delta-like 1 contributes to cell growth by increasing the interferon-inducible protein 16 expression in hepatocellular carcinoma.	GeneRIF	0.00875	129	0.0149	0.152	1	2	IFI16
Pubmed	22739106	Activin B regulates islet composition and islet mass but not whole body glucose homeostasis or insulin sensitivity.	GeneRIF	0.00875	129	0.0149	0.152	1	2	INHBB
Pubmed	7156978	alpha A-crystallin messenger RNA of the mouse lens: more noncoding than coding sequences.	Pubmed	0.00875	129	0.0149	0.152	1	2	CRYAA
Pubmed	23772037	Cutting edge: Conditional MHC class II expression reveals a limited role for B cell antigen presentation in primary and secondary CD4 T cell responses.	Pubmed	0.00875	129	0.0149	0.152	1	2	HLA-DQB1
Pubmed	16868939	Ubiquitin ligase gene expression in healthy volunteers with 20-day bedrest.	GeneRIF	0.00875	129	0.0149	0.152	1	2	FBXO32
Pubmed	25007873	TIMAP promotes angiogenesis by suppressing PTEN-mediated Akt inhibition in human glomerular endothelial cells.	Pubmed	0.00875	129	0.0149	0.152	1	2	PPP1R16B
Pubmed	17681148	Hepatic proprotein convertases modulate HDL metabolism.	Pubmed	0.00875	129	0.0149	0.152	1	2	LIPG

Category	ID	Name	Source	p-value	q-value Bonferroni	q-value FDR B&H	q-value FDR B&Y	Hit Count in Query List	Hit Count in Genome	Hit in Query List
Pubmed	20665636	Inhibition of transglutaminase 2 mitigates transcriptional dysregulation in models of Huntington disease.	Pubmed	0.00875	129	0.0149	0.152	1	2	TGM2
Pubmed	23959645	The Effect of ACP1-ADA1 Genetic Interaction on Human Life Span.	Pubmed	0.00875	129	0.0149	0.152	1	2	ADA
Pubmed	24068281	Crosstalk between monocytes and renal mesangial cells via interaction of metalloproteinases and fractalkine in diabetic nephropathy.	Pubmed	0.00875	129	0.0149	0.152	1	2	CX3CL1
Pubmed	1748486	Structure function analysis of the H-2 Abp gene.	Pubmed	0.00875	129	0.0149	0.152	1	2	HLA-DQB1
Pubmed	18852684	Analysis of PRSS1 and SPINK1 mutations in Korean patients with idiopathic and familial pancreatitis.	GeneRIF	0.00875	129	0.0149	0.152	1	2	SPINK1
Pubmed	17363454	Overexpression of gamma-glutamyltransferase in transgenic mice accelerates bone resorption and causes osteoporosis.	GeneRIF	0.00875	129	0.0149	0.152	1	2	GGT1
Pubmed	22281491	The carboxyl-terminal region of Crtac1B/LOTUS acts as a functional domain in endogenous antagonism to Nogo receptor-1.	GeneRIF	0.00875	129	0.0149	0.152	1	2	RTN4R
Pubmed	25035924	Signal-peptide-peptidase-like 2a is required for CD74 intramembrane proteolysis in human B cells.	Pubmed	0.00875	129	0.0149	0.152	1	2	CD74
Pubmed	23656889	Blockade of KCa3.1 ameliorates renal fibrosis through the TGF-ß1/Smad pathway in diabetic mice.	Pubmed	0.00875	129	0.0149	0.152	1	2	KCNN4
Pubmed	19413900	Macrophage CD74 contributes to MIF-induced pulmonary inflammation.	GeneRIF	0.00875	129	0.0149	0.152	1	2	CD74
Pubmed	18036100	Evolution of hepatitis C virus infection under host factor influence in an ethnically complex population.	GeneRIF	0.00875	129	0.0149	0.152	1	2	HLA-DQB1
Pubmed	8737292	The extracellular matrix proteins fibulin-1 and fibulin-2 in the early human embryo.	Pubmed	0.00875	129	0.0149	0.152	1	2	FBLN2
Pubmed	15365991	Sequence variations of the alpha-globin genes: scanning of high CG content genes with DHPLC and DG-DGGE.	GeneRIF	0.00875	129	0.0149	0.152	1	2	HBA2
Pubmed	22751012	A regulatory loop involving Dies1 and miR-125a controls BMP4 signaling in mouse embryonic stem cells.	GeneRIF	0.00875	129	0.0149	0.152	1	2	BMP4
Pubmed	16934466	Muscle protein analysis in the detection of heterozygotes for recessive limb girdle muscular dystrophy type 2B and 2E.	GeneRIF	0.00875	129	0.0149	0.152	1	2	DYSF
Pubmed	11146555	Functional interaction between p53 and the interferon-inducible nucleoprotein IFI 16.	Pubmed	0.00875	129	0.0149	0.152	1	2	IFI16
Pubmed	10934155	Gelatinase B is required for alveolar bronchiolization after intratracheal bleomycin.	Pubmed	0.00875	129	0.0149	0.152	1	2	MMP7
Pubmed	23377316	Soluble endothelial protein C receptor levels and protein C activity in patients with acute ST-segment elevation myocardial infarction.	Pubmed	0.00875	129	0.0149	0.152	1	2	PROCR
Pubmed	17284534	BMP4, SCF, and hypoxia cooperatively regulate the expansion of murine stress erythroid progenitors.	GeneRIF	0.00875	129	0.0149	0.152	1	2	BMP4
Pubmed	22217834	Hormonal regulation of steroidogenic enzyme gene expression in Leydig cells.	GeneRIF	0.00875	129	0.0149	0.152	1	2	CYP11A1
Pubmed	21930711	Protein phosphatase 2A (PP2A)-specific ubiquitin ligase MID1 is a sequence-dependent regulator of translation efficiency controlling 3-phosphoinositide-dependent protein kinase-1 (PDPK-1).	GeneRIF	0.00875	129	0.0149	0.152	1	2	MID1
Pubmed	18408266	Comparison of allele frequency for HLA-DR and HLA-DQ between patients with ECC and caries-free children.	GeneRIF	0.00875	129	0.0149	0.152	1	2	HLA-DQB1
Pubmed	24325866	The two major isoforms of thyroid hormone receptor, TRa1 and TRß1, preferentially partner with distinct panels of auxiliary proteins.	Pubmed	0.00875	129	0.0149	0.152	1	2	THRA
Pubmed	25123163	Itch-associated peptides: RNA-Seq and bioinformatic analysis of natriuretic precursor peptide B and gastrin releasing peptide in dorsal root and trigeminal ganglia, and the spinal cord.	Pubmed	0.00875	129	0.0149	0.152	1	2	NPPA
Pubmed	22955918	Gene expression profiling of microdissected Hodgkin Reed-Sternberg cells correlates with treatment outcome in classical Hodgkin lymphoma.	GeneRIF	0.00875	129	0.0149	0.152	1	2	CD68
Pubmed	16469487	Transglutaminase activity regulates osteoblast differentiation and matrix mineralization in MC3T3-E1 osteoblast cultures.	GeneRIF	0.00875	129	0.0149	0.152	1	2	TGM2
Pubmed	12455817	HLA haplotypes and susceptibility to rheumatoid arthritis. More than class II genes.	GeneRIF	0.00875	129	0.0149	0.152	1	2	HLA-DQB1
Pubmed	25831530	cGAS-mediated stabilization of IFI16 promotes innate signaling during herpes simplex virus infection.	Pubmed	0.00875	129	0.0149	0.152	1	2	IFI16
Pubmed	16407455	Regeneration of lesioned entorhino-hippocampal axons in vitro by combined degradation of inhibitory proteoglycans and blockade of Nogo-66/NgR signaling.	GeneRIF	0.00875	129	0.0149	0.152	1	2	RTN4R
Pubmed	19372452	Fractalkine receptor/ligand genetic variants and carotid intima-media thickness.	GeneRIF	0.00875	129	0.0149	0.152	1	2	CX3CL1
Pubmed	24441872	Macrophage migration inhibitory factor deficiency in chronic obstructive pulmonary disease.	Pubmed	0.00875	129	0.0149	0.152	1	2	CD74
Pubmed	25253723	Cytoskeletal confinement of CX3CL1 limits its susceptibility to proteolytic cleavage by ADAM10.	Pubmed	0.00875	129	0.0149	0.152	1	2	CX3CL1
Pubmed	21959923	Association of ARMS2/HTRA1 variants with polypoidal choroidal vasculopathy phenotype in a Korean population.	GeneRIF	0.00875	129	0.0149	0.152	1	2	HTRA1
Pubmed	23785123	Co-complexes of MASP-1 and MASP-2 associated with the soluble pattern-recognition molecules drive lectin pathway activation in a manner inhibitable by MAp44.	Pubmed	0.00875	129	0.0149	0.152	1	2	MASP1
Pubmed	15116250	Contribution of polymorphisms in the endothelial protein C receptor gene to soluble endothelial protein C receptor and circulating activated protein C levels, and thrombotic risk.	GeneRIF	0.00875	129	0.0149	0.152	1	2	PROCR
Pubmed	15157381	[Diagnosis of childhood narcolepsy and significance of HLA in its diagnosis].	GeneRIF	0.00875	129	0.0149	0.152	1	2	HLA-DQB1
Pubmed	22964785	Time course and spatial profile of Nogo-A expression in experimental autoimmune encephalomyelitis in C57BL/6 mice.	GeneRIF	0.00875	129	0.0149	0.152	1	2	RTN4R

Category	ID	Name	Source	p-value	q-value Bonferroni	q-value FDR B&H	q-value FDR B&Y	Hit Count in Query List	Hit Count in Genome	Hit in Query List
Pubmed	23278608	Human leukocyte antigens class I and class II in patients with pemphigus in southern Turkey.	GeneRIF	0.00875	129	0.0149	0.152	1	2	HLA-DQB1
Pubmed	20507388	Association of human leukocyte antigen class II alleles with pemphigus vulgaris in a Turkish population.	GeneRIF	0.00875	129	0.0149	0.152	1	2	HLA-DQB1
Pubmed	21168856	Inhibin B and anti-Müllerian hormone as markers of gonadal function after treatment for medulloblastoma or posterior fossa ependymoma during childhood.	GeneRIF	0.00875	129	0.0149	0.152	1	2	INHBB
Pubmed	7588370	Perspectives in steroid hydroxylase gene expression: novel sites of expression during embryonic development.	Pubmed	0.00875	129	0.0149	0.152	1	2	CYP11A1
Pubmed	22132688	Dystrophin deficiency exacerbates skeletal muscle pathology in dysferlin-null mice.	Pubmed	0.00875	129	0.0149	0.152	1	2	DYSF
Pubmed	12911285	Polymorphisms of HLA class II predispose children and adolescents with type 1 diabetes mellitus to autoimmune thyroid disease.	GeneRIF	0.00875	129	0.0149	0.152	1	2	HLA-DQB1
Pubmed	18821214	HLA-class II alleles in Egyptian patients with hepatocellular carcinoma.	GeneRIF	0.00875	129	0.0149	0.152	1	2	HLA-DQB1
Pubmed	22903729	Free fatty acids inhibit TM-EPCR expression through JNK pathway: an implication for the development of the prothrombotic state in metabolic syndrome.	GeneRIF	0.00875	129	0.0149	0.152	1	2	PROCR
Pubmed	15185301	HLA class II genotype influences the type of liver injury in drug-induced idiosyncratic liver disease.	GeneRIF	0.00875	129	0.0149	0.152	1	2	HLA-DQB1
Pubmed	10691312	Functions of thyroid hormone receptors in mice.	Pubmed	0.00875	129	0.0149	0.152	1	2	THRA
Pubmed	16751613	Fluorescence resonance energy transfer study of subunit exchange in human lens crystallins and congenital cataract crystallin mutants.	GeneRIF	0.00875	129	0.0149	0.152	1	2	CRYAA
Pubmed	18230685	Glucocorticoid suppression of CX3CL1 (fractalkine) by reduced gene promoter recruitment of NF-kappaB.	GeneRIF	0.00875	129	0.0149	0.152	1	2	CX3CL1
Pubmed	23333608	Structural and phylogenetic comparison of napsin genes: the duplication, loss of function and human-specific pseudogenization of napsin B.	GeneRIF	0.00875	129	0.0149	0.152	1	2	NAPSA
Pubmed	7857923	Stoichiometry of the atrial natriuretic factor-R1 receptor complex in the bovine zona glomerulosa.	Pubmed	0.00875	129	0.0149	0.152	1	2	NPPA
Pubmed	10642592	Matrix metalloproteinase-7-dependent release of tumor necrosis factor-alpha in a model of herniated disc resorption.	Pubmed	0.00875	129	0.0149	0.152	1	2	MMP7
Pubmed	23572150	KCNN4 channels participate in the EMT induced by PRL-3 in colorectal cancer.	Pubmed	0.00875	129	0.0149	0.152	1	2	KCNN4
Pubmed	12204258	Expression analysis of a novel p75(NTR) signaling protein, which regulates cell cycle progression and apoptosis.	GeneRIF	0.00875	129	0.0149	0.152	1	2	NGFR
Pubmed	14698679	Modulation of sympathetic activity by corticotropin-releasing hormone and atrial natriuretic peptide.	GeneRIF	0.00875	129	0.0149	0.152	1	2	NPPA
Pubmed	10958653	Myotilin is mutated in limb girdle muscular dystrophy 1A.	Pubmed	0.00875	129	0.0149	0.152	1	2	ACTA1
Pubmed	25092331	Impaired selectin-dependent leukocyte recruitment induces T-cell exhaustion and prevents chronic allograft vasculopathy and rejection.	Pubmed	0.00875	129	0.0149	0.152	1	2	HLA-DQB1
Pubmed	16277691	The impact of HLA-DRB1 genes on extra-articular disease manifestations in rheumatoid arthritis.	GeneRIF	0.00875	129	0.0149	0.152	1	2	HLA-DQB1
Pubmed	17911173	Lack of mutations in the thyroid hormone receptor (TR) alpha and beta genes but frequent hypermethylation of the TRbeta gene in differentiated thyroid tumors.	GeneRIF	0.00875	129	0.0149	0.152	1	2	THRA
Pubmed	24344200	Immunosuppression by N-methyl-D-aspartate receptor antagonists is mediated through inhibition of Kv1.3 and KCa3.1 channels in T cells.	Pubmed	0.00875	129	0.0149	0.152	1	2	KCNN4
Pubmed	12682910	Critical role of the transcription factor AP-1 for the constitutive and interferon-induced expression of IFI 16.	GeneRIF	0.00875	129	0.0149	0.152	1	2	IFI16
Pubmed	25148687	Tandem pore domain halothane-inhibited K+ channel subunits THIK1 and THIK2 assemble and form active channels.	Pubmed	0.00875	129	0.0149	0.152	1	2	KCNK12
Pubmed	20854119	Spectrum of a-globin gene mutations in the Kerman Province of Iran.	GeneRIF	0.00875	129	0.0149	0.152	1	2	HBA2
Pubmed	16709807	Conditional ablation of MHC-II suggests an indirect role for MHC-II in regulatory CD4 T cell maintenance.	Pubmed	0.00875	129	0.0149	0.152	1	2	HLA-DQB1
Pubmed	15298337	Seasonality in clinical onset of type 1 diabetes in belgian patients above the age of 10 is restricted to HLA-DQ2/DQ8-negative males, which explains the male to female excess in incidence.	GeneRIF	0.00875	129	0.0149	0.152	1	2	HLA-DQB1
Pubmed	22617145	Evaluation of the pathogenicity of GJB3 and GJB6 variants associated with nonsyndromic hearing loss.	GeneRIF	0.00875	129	0.0149	0.152	1	2	GJB3
Pubmed	12140558	Post-translational disruption of dystroglycan-ligand interactions in congenital muscular dystrophies.	GeneRIF	0.00875	129	0.0149	0.152	1	2	LARGE
Pubmed	24352739	Neuron-glia crosstalk in health and disease: fractalkine and CX3CR1 take centre stage.	Pubmed	0.00875	129	0.0149	0.152	1	2	CX3CL1
Pubmed	24938130	Aryl hydrocarbon receptor catabolic activity in bone metabolism is osteoclast dependent in vivo.	Pubmed	0.00875	129	0.0149	0.152	1	2	CTSK
Pubmed	11754358	Hypereosinophilic syndrome induced by neonatal immunization against MHC class II alloantigen: critical role of IL-4.	Pubmed	0.00875	129	0.0149	0.152	1	2	HLA-DQB1
Pubmed	19409379	The role of hERG1 K+ channels and a functional link between hERG1 K+ channels and SDF-1 in acute leukemic cell migration.	GeneRIF	0.00875	129	0.0149	0.152	1	2	KCNH2
Pubmed	22080209	Identification of human salivary transglutaminases.	GeneRIF	0.00875	129	0.0149	0.152	1	2	TGM2
Pubmed	18156628	HtrA1 inhibits mineral deposition by osteoblasts: requirement for the protease and PDZ domains.	GeneRIF	0.00875	129	0.0149	0.152	1	2	HTRA1
Pubmed	22355679	Essential role of microfibrillar-associated protein 4 in human cutaneous homeostasis and in its photoprotection.	GeneRIF	0.00875	129	0.0149	0.152	1	2	MFAP4

Category	ID	Name	Source	p-value	q-value Bonferroni	q-value FDR B&H	q-value FDR B&Y	Hit Count in Query List	Hit Count in Genome	Hit in Query List
Pubmed	18699806	Altered expression of antimicrobial molecules in cigarette smoke-exposed emphysematous mice lungs.	GeneRIF	0.00875	129	0.0149	0.152	1	2	SLPI
Pubmed	18675265	Targeted disruption of the Pak5 and Pak6 genes in mice leads to deficits in learning and locomotion.	GeneRIF	0.00875	129	0.0149	0.152	1	2	PAK6
Pubmed	14649314	Three new hemoglobin variants with abnormal oxygen affinity: Hb Saratoga Springs [alpha40(C5)Lys --> Asn (alpha1)], Hb Santa Clara [beta97(FG4)His --> Asn], and Hb Sparta [beta103(G5)Phe --> Val].	GeneRIF	0.00875	129	0.0149	0.152	1	2	HBB
Pubmed	20836749	Correlations between islet autoantibody specificity and the SLC30A8 genotype with HLA-DQB1 and metabolic control in new onset type 1 diabetes.	GeneRIF	0.00875	129	0.0149	0.152	1	2	HLA-DQB1
Pubmed	16413472	Cbf beta-SMMHC induces distinct abnormal myeloid progenitors able to develop acute myeloid leukemia.	Pubmed	0.00875	129	0.0149	0.152	1	2	MYH11
Pubmed	20405713	[Immune polymorphism analysis of HLA class II antigens in ulcer diseases associated with Helicobacter pylori in children].	GeneRIF	0.00875	129	0.0149	0.152	1	2	HLA-DQB1
Pubmed	22730330	Cathepsin S cannibalism of cathepsin K as a mechanism to reduce type I collagen degradation.	GeneRIF	0.00875	129	0.0149	0.152	1	2	CTSK
Pubmed	15118286	Neurohumoral profiles in patients with hypertrophic cardiomyopathy: differences to hypertensive left ventricular hypertrophy.	GeneRIF	0.00875	129	0.0149	0.152	1	2	NPPA
Pubmed	16586073	Increased nerve growth factor and its receptors in atopic dermatitis: an immunohistochemical study.	GeneRIF	0.00875	129	0.0149	0.152	1	2	NGFR
Pubmed	21252205	Risk- and non-risk-associated variants at the 10q26 AMD locus influence ARMS2 mRNA expression but exclude pathogenic effects due to protein deficiency.	GeneRIF	0.00875	129	0.0149	0.152	1	2	HTRA1
Pubmed	8786845	Matrix metalloproteinases degrade myelin basic protein.	Pubmed	0.00875	129	0.0149	0.152	1	2	MMP7
Pubmed	16365196	Disruption of the cathepsin K gene reduces atherosclerosis progression and induces plaque fibrosis but accelerates macrophage foam cell formation.	GeneRIF	0.00875	129	0.0149	0.152	1	2	CTSK
Pubmed	22641693	Bone morphogenetic proteins differentially regulate pigmentation in human skin cells.	GeneRIF	0.00875	129	0.0149	0.152	1	2	BMP4
Pubmed	19544488	B7-H3 is a potent inhibitor of human T-cell activation: No evidence for B7-H3 and TREML2 interaction.	GeneRIF	0.00875	129	0.0149	0.152	1	2	CD276
Pubmed	21029659	[Polymorphism of HLA-DRB1 and -DQB1 alleles among young women with cervical squamous cell carcinoma and its relation with human papillomavirus type 16 infection].	GeneRIF	0.00875	129	0.0149	0.152	1	2	HLA-DQB1
Pubmed	12109964	Human leukocyte antigen alleles in patients with bipolar disorder in the Korean population.	GeneRIF	0.00875	129	0.0149	0.152	1	2	HLA-DQB1
Pubmed	11588129	HLA class II DR-DQ and increased risk of cervical cancer among Senegalese women.	GeneRIF	0.00875	129	0.0149	0.152	1	2	HLA-DQB1
Pubmed	20004474	Transglutaminase II interacts with rac1, regulates production of reactive oxygen species, expression of snail, secretion of Th2 cytokines and mediates in vitro and in vivo allergic inflammation.	GeneRIF	0.00875	129	0.0149	0.152	1	2	TGM2
Pubmed	18021008	Association of bone morphogenetic proteins with otosclerosis.	GeneRIF	0.00875	129	0.0149	0.152	1	2	BMP4
Pubmed	16841096	Role of A2B adenosine receptor signaling in adenosine-dependent pulmonary inflammation and injury.	GeneRIF	0.00875	129	0.0149	0.152	1	2	ADA
Pubmed	23702483	The role of focal adhesion kinase in BMP4 induction of mesenchymal stem cell adipogenesis.	GeneRIF	0.00875	129	0.0149	0.152	1	2	BMP4
Pubmed	23648579	Tetraspanin-13 modulates voltage-gated CaV2.2 Ca2+ channels.	Pubmed	0.00875	129	0.0149	0.152	1	2	TSPAN13
Pubmed	19834057	Genetic manipulation of dysferlin expression in skeletal muscle: novel insights into muscular dystrophy.	GeneRIF	0.00875	129	0.0149	0.152	1	2	DYSF
Pubmed	21943816	Association between HLA-DQA1, HLA-DQB1 and oral cancer.	GeneRIF	0.00875	129	0.0149	0.152	1	2	HLA-DQB1
Pubmed	18511946	Age-related macular degeneration is associated with an unstable ARMS2 (LOC387715) mRNA.	GeneRIF	0.00875	129	0.0149	0.152	1	2	HTRA1
Pubmed	17804733	Kallikrein 6 induces E-cadherin shedding and promotes cell proliferation, migration, and invasion.	GeneRIF	0.00875	129	0.0149	0.152	1	2	KLK1
Pubmed	20813955	The molecular interaction of CAR and JAML recruits the central cell signal transducer PI3K.	GeneRIF	0.00875	129	0.0149	0.152	1	2	AMICA1
Pubmed	19502653	Common SPINK-1 mutations do not predispose to the development of non-alcoholic fatty liver disease.	GeneRIF	0.00875	129	0.0149	0.152	1	2	SPINK1
Pubmed	24284363	Molecular analysis of potassium ion channel genes in sudden death cases among patients administered psychotropic drug therapy: are polymorphisms in LQT genes a potential risk factor?	Pubmed	0.00875	129	0.0149	0.152	1	2	KCNH2
Pubmed	15240734	A3 adenosine receptor signaling contributes to airway inflammation and mucus production in adenosine deaminase-deficient mice.	Pubmed	0.00875	129	0.0149	0.152	1	2	ADA
Pubmed	20463743	Opposing effects of the HLA-DRB1*0301-DQB1*0201 haplotype on the risk for multiple sclerosis in diverse Arab populations in Israel.	GeneRIF	0.00875	129	0.0149	0.152	1	2	HLA-DQB1
Pubmed	18725155	CYP11A1 and CYP17 promoter polymorphisms associate with hyperandrogenemia in polycystic ovary syndrome.	GeneRIF	0.00875	129	0.0149	0.152	1	2	CYP11A1
Pubmed	22511776	Monospecific inhibitors show that both mannan-binding lectin-associated serine protease-1 (MASP-1) and -2 Are essential for lectin pathway activation and reveal structural plasticity of MASP-2.	GeneRIF	0.00875	129	0.0149	0.152	1	2	MASP1
Pubmed	21252093	Sézary syndrome cells overexpress syndecan-4 bearing distinct heparan sulfate moieties that suppress T-cell activation by binding DC-HIL and trapping TGF-beta on the cell surface.	GeneRIF	0.00875	129	0.0149	0.152	1	2	GPNMB

Category	ID	Name	Source	p-value	q-value Bonferroni	q-value FDR B&H	q-value FDR B&Y	Hit Count in Query List	Hit Count in Genome	Hit in Query List
Pubmed	18950703	G-protein-coupled receptor-associated A-kinase anchoring proteins AKAP5 and AKAP12: differential trafficking and distribution.	GeneRIF	0.00875	129	0.0149	0.152	1	2	AKAP12
Pubmed	14995006	Is HLA class II susceptibility to primary antiphospholipid syndrome different from susceptibility to secondary antiphospholipid syndrome?	GeneRIF	0.00875	129	0.0149	0.152	1	2	HLA-DQB1
Pubmed	14507909	Human mesotrypsin is a unique digestive protease specialized for the degradation of trypsin inhibitors.	GeneRIF	0.00875	129	0.0149	0.152	1	2	SPINK1
Pubmed	7685774	Antigen nonspecific effect of major histocompatibility complex haplotype on autoantibody levels in systemic lupus erythematosus-prone lpr mice.	Pubmed	0.00875	129	0.0149	0.152	1	2	HLA-DQB1
Pubmed	19799789	Hypertrophy is induced during the in vitro chondrogenic differentiation of human mesenchymal stem cells by bone morphogenetic protein-2 and bone morphogenetic protein-4 gene transfer.	GeneRIF	0.00875	129	0.0149	0.152	1	2	BMP4
Pubmed	17923522	Individual matrix metalloproteinases control distinct transcriptional responses in airway epithelial cells infected with Pseudomonas aeruginosa.	Pubmed	0.00875	129	0.0149	0.152	1	2	MMP7
Pubmed	18808722	Protective effect of KCNH2 single nucleotide polymorphism K897T in LQTS families and identification of novel KCNQ1 and KCNH2 mutations.	GeneRIF	0.00875	129	0.0149	0.152	1	2	KCNH2
Pubmed	24673545	Serum levels of fractalkine are associated with markers of insulin resistance in gestational diabetes.	Pubmed	0.00875	129	0.0149	0.152	1	2	CX3CL1
Pubmed	23974513	Chemokines and atherosclerosis: focus on the CX3CL1/CX3CR1 pathway.	Pubmed	0.00875	129	0.0149	0.152	1	2	CX3CL1
Pubmed	21344381	Cooperative nongenomic and genomic actions on thyroid hormone mediated-modulation of T cell proliferation involve up-regulation of thyroid hormone receptor and inducible nitric oxide synthase expression.	GeneRIF	0.00875	129	0.0149	0.152	1	2	THRA
Pubmed	9268129	Dental innervation and CGRP in adult p75-deficient mice.	Pubmed	0.00875	129	0.0149	0.152	1	2	NGFR
Pubmed	20850938	[Inheritance in erythropoietic protoporphyria].	GeneRIF	0.00875	129	0.0149	0.152	1	2	ALAS2
Pubmed	6469697	Hb J Oxford [alpha 15 (A13) Gly----Asp] in Japan.	Pubmed	0.00875	129	0.0149	0.152	1	2	HBA2
Pubmed	8088790	Cloning of genomic loci and chromosomal localization of the human PCTAIRE-1 and -3 protein kinase genes.	Pubmed	0.00875	129	0.0149	0.152	1	2	CDK18
Pubmed	14688470	Polymorphisms of SPINK1 N34S and CFTR in patients with sporadic and familial pancreatic cancer.	GeneRIF	0.00875	129	0.0149	0.152	1	2	SPINK1
Pubmed	9503024	Genomic organization of mouse Capn5 and Capn6 genes confirms that they are a distinct calpain subfamily.	Pubmed	0.00875	129	0.0149	0.152	1	2	CAPN5
Pubmed	24177035	Dysferlin and myoferlin regulate transverse tubule formation and glycerol sensitivity.	Pubmed	0.00875	129	0.0149	0.152	1	2	DYSF
Pubmed	25063248	Matrix metalloproteinase-7 A-181G and its interaction with matrix metalloproteinase-9 C-1562T polymorphism in preeclamptic patients: association with malondialdehyde level and severe preeclampsia.	Pubmed	0.00875	129	0.0149	0.152	1	2	MMP7
Pubmed	17196618	Expression of GATA-1 in a non-hematopoietic cel line induces beta-globin locus control region chromatin structure remodeling and an erythroid pattern of gene expression.	GeneRIF	0.00875	129	0.0149	0.152	1	2	HBB
Pubmed	22174188	SNX10 is required for osteoclast formation and resorption activity.	GeneRIF	0.00875	129	0.0149	0.152	1	2	CTSK
Pubmed	20820776	Alternative Ii-independent antigen-processing pathway in leukemic blasts involves TAP-dependent peptide loading of HLA class II complexes.	GeneRIF	0.00875	129	0.0149	0.152	1	2	CD74
Pubmed	22093090	Fractalkine/CX3CL1 protects striatal neurons from synergistic morphine and HIV-1 Tat-induced dendritic losses and death.	GeneRIF	0.00875	129	0.0149	0.152	1	2	CX3CL1
Pubmed	19463786	Noggin and BMP4 co-modulate adult hippocampal neurogenesis in the APP(swe)/PS1(DeltaE9) transgenic mouse model of Alzheimer's disease.	GeneRIF	0.00875	129	0.0149	0.152	1	2	BMP4
Pubmed	21296087	Detection and characterization of the in vitro e3 ligase activity of the human MID1 protein.	GeneRIF	0.00875	129	0.0149	0.152	1	2	MID1
Pubmed	2888727	Allelic forms of the alpha- and beta-chain genes encoding DQw1-positive heterodimers.	Pubmed	0.00875	129	0.0149	0.152	1	2	HLA-DQB1
Pubmed	8841408	Different susceptibilities of fibulin-1 and fibulin-2 to cleavage by matrix metalloproteinases and other tissue proteases.	Pubmed	0.00875	129	0.0149	0.152	1	2	FBLN2
Pubmed	24826793	Novel point mutation of the a2-globin gene (HBA2) and a rare 2.4¿kb deletion of the a1-globin gene (HBA1), identified in two chinese patients with Hb H disease.	Pubmed	0.00875	129	0.0149	0.152	1	2	HBA2
Pubmed	20462916	Association of the human leucocyte antigen region with susceptibility to Parkinson's disease.	GeneRIF	0.00875	129	0.0149	0.152	1	2	HLA-DQB1
Pubmed	8294199	Hb Melusine [alpha 114(GH2)Pro-->Ser]: a new neutral hemoglobin variant.	Pubmed	0.00875	129	0.0149	0.152	1	2	HBA2
Pubmed	3013550	Isolation and characterization of cDNA clones from mouse skeletal muscle actin mRNA.	Pubmed	0.00875	129	0.0149	0.152	1	2	ACTA1
Pubmed	7959780	Protective protein for beta-galactosidase, Ppgb, maps to the distal imprinting region of mouse chromosome 2 but is not imprinted.	Pubmed	0.00875	129	0.0149	0.152	1	2	ADA
Pubmed	9927422	Different functions for the thyroid hormone receptors TRalpha and TRbeta in the control of thyroid hormone production and post-natal development.	Pubmed	0.00875	129	0.0149	0.152	1	2	THRA
Pubmed	23916610	MicroRNA-187, down-regulated in clear cell renal cell carcinoma and associated with lower survival, inhibits cell growth and migration though targeting B7-H3.	Pubmed	0.00875	129	0.0149	0.152	1	2	CD276
Pubmed	18759454	Apoptosis signal-regulating kinase 1 attenuates atrial natriuretic peptide secretion.	GeneRIF	0.00875	129	0.0149	0.152	1	2	NPPA
Pubmed	15618358	Role of carboxypeptidase E in processing of pro-islet amyloid polypeptide in {beta}-cells.	GeneRIF	0.00875	129	0.0149	0.152	1	2	CPE

Category	ID	Name	Source	p-value	q-value Bonferroni	q-value FDR B&H	q-value FDR B&Y	Hit Count in Query List	Hit Count in Genome	Hit in Query List
Pubmed	16670820	Expression of inhibin/activin subunits alpha (-alpha), beta A (-beta (A)) and beta B (-beta (B)) in placental tissue of normal and intrauterine growth restricted (IUGR) pregnancies.	GeneRIF	0.00875	129	0.0149	0.152	1	2	INHBB
Pubmed	22284614	Influence of HLA alleles in response to treatment with pegylated interferon-alpha and ribavirin in patients with chronic hepatitis C.	GeneRIF	0.00875	129	0.0149	0.152	1	2	HLA-DQB1
Pubmed	22454509	Macrophage migration inhibitory factor (MIF) promotes cell survival and proliferation of neural stem/progenitor cells.	GeneRIF	0.00875	129	0.0149	0.152	1	2	CD74
Pubmed	25338725	Overexpression of Reg4, alone or combined with MMP-7 overexpression, is predictive of poor prognosis in colorectal cancer.	Pubmed	0.00875	129	0.0149	0.152	1	2	MMP7
Pubmed	21694611	Influence of HLA-DQ2 and DQ8 on severity in celiac Disease.	GeneRIF	0.00875	129	0.0149	0.152	1	2	HLA-DQB1
Pubmed	21599637	Canonical Wnt signalling requires the BMP pathway to inhibit oligodendrocyte maturation.	GeneRIF	0.00875	129	0.0149	0.152	1	2	BMP4
Pubmed	18562179	Association of HLA-DQA1 and DQB1 alleles with keloids in Chinese Hans.	GeneRIF	0.00875	129	0.0149	0.152	1	2	HLA-DQB1
Pubmed	1322300	Characterization of gap junction genes expressed in F9 embryonic carcinoma cells: molecular cloning of mouse connexin31 and -45 cDNAs.	Pubmed	0.00875	129	0.0149	0.152	1	2	GJB3
Pubmed	15173106	The small leucine-rich proteoglycan biglycan modulates BMP-4-induced osteoblast differentiation.	GeneRIF	0.00875	129	0.0149	0.152	1	2	BMP4
Pubmed	12076860	Up-regulated expression of fractalkine and its receptor CX3CR1 during liver injury in humans.	GeneRIF	0.00875	129	0.0149	0.152	1	2	CX3CL1
Pubmed	22034513	Transglutaminase 2-mediated activation of ß-catenin signaling has a critical role in warfarin-induced vascular calcification.	GeneRIF	0.00875	129	0.0149	0.152	1	2	TGM2
Pubmed	22138126	Localization of nerve growth factor (NGF) receptors in the mitochondrial compartment: characterization and putative role.	GeneRIF	0.00875	129	0.0149	0.152	1	2	NGFR
Pubmed	4085253	Assignment of the mouse alpha A-crystallin structural gene to chromosome 17.	Pubmed	0.00875	129	0.0149	0.152	1	2	CRYAA
Pubmed	7866401	Molecular genetics of metachromatic leukodystrophy.	Pubmed	0.00875	129	0.0149	0.152	1	2	PSAP
Pubmed	16117728	Increased plasma levels of natriuretic peptide type B and A in children with congenital heart defects with left compared with right ventricular volume overload or pressure overload.	GeneRIF	0.00875	129	0.0149	0.152	1	2	NPPA
Pubmed	15388265	Diabetes mellitus in adults: association of HLA DRB1 and DQB1 diabetes risk alleles with GADab presence and C-peptide secretion.	GeneRIF	0.00875	129	0.0149	0.152	1	2	HLA-DQB1
Pubmed	12145745	Visualizing human leukocyte antigen class II risk haplotypes in human systemic lupus erythematosus.	GeneRIF	0.00875	129	0.0149	0.152	1	2	HLA-DQB1
Pubmed	12270925	Interaction with GM130 during HERG ion channel trafficking. Disruption by type 2 congenital long QT syndrome mutations. Human Ether-à-go-go-Related Gene.	GeneRIF	0.00875	129	0.0149	0.152	1	2	KCNH2
Pubmed	9531264	Distinct peptide loading pathways for MHC class II molecules associated with alternative Ii chain isoforms.	Pubmed	0.00875	129	0.0149	0.152	1	2	CD74
Pubmed	18375543	Transglutaminase 2 protects against ischemic insult, interacts with HIF1beta, and attenuates HIF1 signaling.	GeneRIF	0.00875	129	0.0149	0.152	1	2	TGM2
Pubmed	15691311	Association of Helicobacter pylori-related distal gastric cancer with the HLA class II gene DQB10602 and cagA strains in a southern European population.	GeneRIF	0.00875	129	0.0149	0.152	1	2	HLA-DQB1
Pubmed	16112030	Severe pulmonary sarcoidosis is strongly associated with the haplotype HLA-DQB1*0602-DRB1*150101.	GeneRIF	0.00875	129	0.0149	0.152	1	2	HLA-DQB1
Pubmed	18085741	Associations of erosive arthritis with anti-cyclic citrullinated peptide antibodies and MHC Class II alleles in systemic lupus erythematosus.	GeneRIF	0.00875	129	0.0149	0.152	1	2	HLA-DQB1
Pubmed	23572149	High CD74 expression correlates with ZAP70 expression in B cell chronic lymphocytic leukemia patients.	Pubmed	0.00875	129	0.0149	0.152	1	2	CD74
Pubmed	9645951	Distinct subcellular localization of calcium binding S100 proteins in human smooth muscle cells and their relocation in response to rises in intracellular calcium.	Pubmed	0.00875	129	0.0149	0.152	1	2	ACTA1
Pubmed	7750493	Expression of the steroidogenic enzyme P450scc in the central and peripheral nervous systems during rodent embryogenesis.	Pubmed	0.00875	129	0.0149	0.152	1	2	CYP11A1
Pubmed	20231316	Histone demethylase KDM5A is an integral part of the core Notch-RBP-J repressor complex.	GeneRIF	0.00875	129	0.0149	0.152	1	2	KDM5A
Pubmed	18356750	Cutaneous microvascular dysfunction is associated with human leukocyte antigen-DQ in youths with type 1 diabetes.	GeneRIF	0.00875	129	0.0149	0.152	1	2	HLA-DQB1
Pubmed	16914140	Complex formation between tissue transglutaminase II (tTG) and vascular endothelial growth factor receptor 2 (VEGFR-2): proposed mechanism for modulation of endothelial cell response to VEGF.	GeneRIF	0.00875	129	0.0149	0.152	1	2	TGM2
Pubmed	21075281	Protein quality control during erythropoiesis and hemoglobin synthesis.	GeneRIF	0.00875	129	0.0149	0.152	1	2	HBB
Pubmed	11821948	The interaction of p53 with the nuclear matrix is mediated by F-actin and modulated by DNA damage.	Pubmed	0.00875	129	0.0149	0.152	1	2	ACTA1
Pubmed	23707940	miR-421 induces cell proliferation and apoptosis resistance in human nasopharyngeal carcinoma via downregulation of FOXO4.	GeneRIF	0.00875	129	0.0149	0.152	1	2	FOXO4
Pubmed	22865667	Human serine protease HTRA1 positively regulates osteogenesis of human bone marrow-derived mesenchymal stem cells and mineralization of differentiating bone-forming cells through the modulation of extracellular matrix protein.	GeneRIF	0.00875	129	0.0149	0.152	1	2	HTRA1

Category	ID	Name	Source	p-value	q-value Bonferroni	q-value FDR B&H	q-value FDR B&Y	Hit Count in Query List	Hit Count in Genome	Hit in Query List
Pubmed	18848812	In utero onset of long QT syndrome with atrioventricular block and spontaneous or lidocaine induced ventricular tachycardia: compound effects of hERG pore region mutation and SCN5A N-terminus variant.	GeneRIF	0.00875	129	0.0149	0.152	1	2	KCNH2
Pubmed	6897014	A fetal skeletal muscle actin mRNA in the mouse and its identity with cardiac actin mRNA.	Pubmed	0.00875	129	0.0149	0.152	1	2	ACTA1
Pubmed	10556977	Tissue transglutaminase is a caspase substrate during apoptosis. Cleavage causes loss of transamidating function and is a biochemical marker of caspase 3 activation.	Pubmed	0.00875	129	0.0149	0.152	1	2	TGM2
Pubmed	25778399	RhoGDIß Inhibits Bone Morphogenetic Protein 4 (BMP4)-induced Adipocyte Lineage Commitment and Favors Smooth Muscle-like Cell Differentiation.	Pubmed	0.00875	129	0.0149	0.152	1	2	BMP4
Pubmed	9843985	Transgenic knockout mice exclusively expressing human hemoglobin S after transfer of a 240-kb betas-globin yeast artificial chromosome: A mouse model of sickle cell anemia.	Pubmed	0.00875	129	0.0149	0.152	1	2	HBA2
Pubmed	15551748	Differential regulation of inhibin subunits by germ cells in human testes.	GeneRIF	0.00875	129	0.0149	0.152	1	2	INHBB
Pubmed	25383971	Variation at HLA-DRB1 is associated with resistance to enteric fever.	Pubmed	0.00875	129	0.0149	0.152	1	2	HLA-DQB1
Pubmed	11592043	[Sequencing-based typing of HLA-DPB1 and DQB1 exon 2 in patients with type 1 diabetes mellitus].	GeneRIF	0.00875	129	0.0149	0.152	1	2	HLA-DQB1
Pubmed	10880574	Corin, a transmembrane cardiac serine protease, acts as a pro-atrial natriuretic peptide-converting enzyme.	Pubmed	0.00875	129	0.0149	0.152	1	2	NPPA
Pubmed	15855271	Implications of the serine protease HtrA1 in amyloid precursor protein processing.	GeneRIF	0.00875	129	0.0149	0.152	1	2	HTRA1
Pubmed	15136557	MHC class II-independent and -dependent T cell expansion and B cell hyperactivity in vivo in mice deficient in CD152 (CTLA-4).	Pubmed	0.00875	129	0.0149	0.152	1	2	HLA-DQB1
Pubmed	19207936	Differential expression of HLA-DQ alleles in peripheral blood mononuclear cells: alleles associated with susceptibility to and protection from autoimmune type 1 diabetes.	GeneRIF	0.00875	129	0.0149	0.152	1	2	HLA-DQB1
Pubmed	8382772	Retroposition in a family of carcinoma-associated antigen genes.	Pubmed	0.00875	129	0.0149	0.152	1	2	TACSTD2
Pubmed	24204699	Concomitant tumor expression of EGFR and TATI/SPINK1 associates with better prognosis in colorectal cancer.	Pubmed	0.00875	129	0.0149	0.152	1	2	SPINK1
Pubmed	23241319	Interactions between hERG and KCNQ1 a-subunits are mediated by their COOH termini and modulated by cAMP.	GeneRIF	0.00875	129	0.0149	0.152	1	2	KCNH2
Pubmed	10845921	A(2A) receptor dependent and A(2A) receptor independent effects of extracellular adenosine on murine thymocytes in conditions of adenosine deaminase deficiency.	Pubmed	0.00875	129	0.0149	0.152	1	2	ADA
Pubmed	21809340	Gradients of natriuretic peptide precursor A (NPPA) in oviduct and of natriuretic peptide receptor 1 (NPR1) in spermatozoon are involved in mouse sperm chemotaxis and fertilization.	GeneRIF	0.00875	129	0.0149	0.152	1	2	NPPA
Pubmed	12135603	Induction of IkappaB: atrial natriuretic peptide as a regulator of the NF-kappaB pathway.	GeneRIF	0.00875	129	0.0149	0.152	1	2	NPPA
Pubmed	11095601	Corneal epithelial tight junctions and their response to lipopolysaccharide challenge.	Pubmed	0.00875	129	0.0149	0.152	1	2	CLDN15
Pubmed	24143235	Differential activation of Wnt-ß-catenin pathway in triple negative breast cancer increases MMP7 in a PTEN dependent manner.	Pubmed	0.00875	129	0.0149	0.152	1	2	MMP7
Pubmed	11220743	HIV-1 Tat neurotoxicity is prevented by matrix metalloproteinase inhibitors.	Pubmed	0.00875	129	0.0149	0.152	1	2	MMP7
Pubmed	20711474	ADAM10 releases a soluble form of the GPNMB/Osteoactivin extracellular domain with angiogenic properties.	GeneRIF	0.00875	129	0.0149	0.152	1	2	GPNMB
Pubmed	15882059	A biophysical investigation of recombinant hemoglobins with aromatic B10 mutations in the distal heme pockets.	GeneRIF	0.00875	129	0.0149	0.152	1	2	HBB
Pubmed	19552401	Targeting and insertion of the cholesterol-binding translocator protein into the outer mitochondrial membrane.	Pubmed	0.00875	129	0.0149	0.152	1	2	TSPO
Pubmed	15281007	Type 2 diabetes and the genetics of signal transduction: a study of interaction between adenosine deaminase and acid phosphatase locus 1 polymorphisms.	GeneRIF	0.00875	129	0.0149	0.152	1	2	ADA
Pubmed	11427739	Induction of primordial germ cells from murine epiblasts by synergistic action of BMP4 and BMP8B signaling pathways.	Pubmed	0.00875	129	0.0149	0.152	1	2	BMP4
Pubmed	23730214	CD74-dependent deregulation of the tumor suppressor scribble in human epithelial and breast cancer cells.	Pubmed	0.00875	129	0.0149	0.152	1	2	CD74
Pubmed	16051666	Contrasting skeletal phenotypes in mice with an identical mutation targeted to thyroid hormone receptor alpha1 or beta.	GeneRIF	0.00875	129	0.0149	0.152	1	2	THRA
Pubmed	19187226	Hypoxia induces erythroid-specific 5-aminolevulinate synthase expression in human erythroid cells through transforming growth factor-beta signaling.	GeneRIF	0.00875	129	0.0149	0.152	1	2	ALAS2
Pubmed	20676769	The prevalence of cationic trypsinogen (PRSS1) and serine protease inhibitor, Kazal type 1 (SPINK1) gene mutations in Polish patients with alcoholic and idiopathic chronic pancreatitis.	GeneRIF	0.00875	129	0.0149	0.152	1	2	SPINK1
Pubmed	21335487	ß2-adrenergic receptor activation prevents rodent dopaminergic neurotoxicity by inhibiting microglia via a novel signaling pathway.	Pubmed	0.00875	129	0.0149	0.152	1	2	SLPI
Pubmed	15336786	HLA DRB1* and DQB1* allelic diversity among Nadars: a primitive south Indian Dravidian caste group.	GeneRIF	0.00875	129	0.0149	0.152	1	2	HLA-DQB1
Pubmed	18708042	Alanine aminotransferase and gamma-glutamyl transferase are associated with the metabolic syndrome but not with angiographically determined coronary atherosclerosis.	GeneRIF	0.00875	129	0.0149	0.152	1	2	GGT1

Category	ID	Name	Source	p-value	q-value Bonferroni	q-value FDR B&H	q-value FDR B&Y	Hit Count in Query List	Hit Count in Genome	Hit in Query List
Pubmed	21652727	Type-2 iodothyronine 5'deiodinase (D2) in skeletal muscle of C57Bl/6 mice. II. Evidence for a role of D2 in the hypermetabolism of thyroid hormone receptor alpha-deficient mice.	GeneRIF	0.00875	129	0.0149	0.152	1	2	THRA
Pubmed	6187470	Alternative RNA splicing of the murine alpha A-crystallin gene: protein-coding information within an intron.	Pubmed	0.00875	129	0.0149	0.152	1	2	CRYAA
Pubmed	8227090	In vitro chamber specification during embryonic stem cell cardiogenesis. Expression of the ventricular myosin light chain-2 gene is independent of heart tube formation.	Pubmed	0.00875	129	0.0149	0.152	1	2	NPPA
Pubmed	20141582	Ontogenetic profile of the expression of thyroid hormone receptors in rat and human corpora cavernosa of the penis.	GeneRIF	0.00875	129	0.0149	0.152	1	2	THRA
Pubmed	23135544	LARGE2 generates the same xylose- and glucuronic acid-containing glycan structures as LARGE.	Pubmed	0.00875	129	0.0149	0.152	1	2	LARGE
Pubmed	19696993	Genotype-phenotype correlation in 9 patients with tropical pancreatitis and identified gene mutations.	GeneRIF	0.00875	129	0.0149	0.152	1	2	SPINK1
Pubmed	16650414	Follicular fluid levels of inhibin A, inhibin B, and activin A levels reflect changes in follicle size but are not independent markers of the oocyte's ability to fertilize.	GeneRIF	0.00875	129	0.0149	0.152	1	2	INHBB
Pubmed	14581805	Assessment of the DQ heterodimer test in the diagnosis of celiac disease in the Canary Islands (Spain).	GeneRIF	0.00875	129	0.0149	0.152	1	2	HLA-DQB1
Pubmed	12137805	Placental cytochrome P450scc (CYP11A1): comparison of catalytic properties between conditions of limiting and saturating adrenodoxin reductase.	GeneRIF	0.00875	129	0.0149	0.152	1	2	CYP11A1
Pubmed	21408188	The association of mid-regional pro-adrenomedullin and mid-regional pro-atrial natriuretic peptide with mortality in an incident dialysis cohort.	GeneRIF	0.00875	129	0.0149	0.152	1	2	NPPA
Pubmed	12666382	[Prevalence and predictive value of GAD65 autoantibodies and their correlation with HLA DR-DQ genotypes in children with type-1 diabetes].	GeneRIF	0.00875	129	0.0149	0.152	1	2	HLA-DQB1
Pubmed	21566120	The thyroid hormone receptors as modulators of skin proliferation and inflammation.	Pubmed	0.00875	129	0.0149	0.152	1	2	THRA
Pubmed	20836851	Hemoglobin a and ß are ubiquitous in the human lung, decline in idiopathic pulmonary fibrosis but not in COPD.	GeneRIF	0.00875	129	0.0149	0.152	1	2	HBB
Pubmed	18337405	Synaptic function for the Nogo-66 receptor NgR1: regulation of dendritic spine morphology and activity-dependent synaptic strength.	GeneRIF	0.00875	129	0.0149	0.152	1	2	RTN4R
Pubmed	16923798	C-terminal HERG (LQT2) mutations disrupt IKr channel regulation through 14-3-3epsilon.	GeneRIF	0.00875	129	0.0149	0.152	1	2	KCNH2
Pubmed	21570397	Susceptibility to amoxicillin-clavulanate-induced liver injury is influenced by multiple HLA class I and II alleles.	Pubmed	0.00875	129	0.0149	0.152	1	2	HLA-DQB1
Pubmed	11891768	Nogos and the Nogo-66 receptor: factors inhibiting CNS neuron regeneration.	GeneRIF	0.00875	129	0.0149	0.152	1	2	RTN4R
Pubmed	10611475	Thymosin beta(4) serves as a glutaminyl substrate of transglutaminase. Labeling with fluorescent dansylcadaverine does not abolish interaction with G-actin.	Pubmed	0.00875	129	0.0149	0.152	1	2	TGM2
Pubmed	17568789	Distinctive gene expression signatures in rheumatoid arthritis synovial tissue fibroblast cells: correlates with disease activity.	Pubmed	0.00875	129	0.0149	0.152	1	2	BAIAP2L1
Pubmed	24615104	Association of MMP7-181A/G and MMP13-77A/G polymorphisms with colorectal cancer in a Mexican population.	Pubmed	0.00875	129	0.0149	0.152	1	2	MMP7
Pubmed	21628999	Differential regulation and localization of carboxypeptidase D and carboxypeptidase E in human and mouse ß-cells.	GeneRIF	0.00875	129	0.0149	0.152	1	2	CPE
Pubmed	19673046	Ion transport and barrier function in a telomerase-immortalized human nondysplastic, Barrett's cell line (BAR-T).	Pubmed	0.00875	129	0.0149	0.152	1	2	CLDN15
Pubmed	24492704	A transgenic mouse model for early prostate metastasis to lymph nodes.	Pubmed	0.00875	129	0.0149	0.152	1	2	AKAP12
Pubmed	23345219	Bradykinin-induced chemotaxis of human gliomas requires the activation of KCa3.1 and ClC-3.	GeneRIF	0.00875	129	0.0149	0.152	1	2	KCNN4
Pubmed	18682600	Dendritic cell and natural killer cell cross-talk: a pivotal role of CX3CL1 in NK cytoskeleton organization and activation.	GeneRIF	0.00875	129	0.0149	0.152	1	2	CX3CL1
Pubmed	24674776	Upregulation of KCa3.1 K(+) channel in mesenteric lymph node CD4(+) T lymphocytes from a mouse model of dextran sodium sulfate-induced inflammatory bowel disease.	Pubmed	0.00875	129	0.0149	0.152	1	2	KCNN4
Pubmed	8929711	Different contribution of HLA-DR and -DQ genes in susceptibility and resistance to insulin-dependent diabetes mellitus (IDDM).	Pubmed	0.00875	129	0.0149	0.152	1	2	HLA-DQB1
Pubmed	22096481	Nogo-receptors NgR1 and NgR2 do not mediate regulation of CD4 T helper responses and CNS repair in experimental autoimmune encephalomyelitis.	GeneRIF	0.00875	129	0.0149	0.152	1	2	RTN4R
Pubmed	11429692	Cooperation of Cdc42 small G protein-activating and actin filament-binding activities of frabin in microspike formation.	Pubmed	0.00875	129	0.0149	0.152	1	2	ACTA1
Pubmed	22786832	Does HLA Class II haplotype play a role in adult acute disseminated encephalomyelitis? Preliminary findings from a Southern Italy hospital-based study.	GeneRIF	0.00875	129	0.0149	0.152	1	2	HLA-DQB1
Pubmed	17722421	Major histocompatibility complex class II polymorphisms and risk of cervical cancer in Puerto Rican women.	GeneRIF	0.00875	129	0.0149	0.152	1	2	HLA-DQB1
Pubmed	11845225	Progression to insulin-requiring diabetes in seronegative prediabetic subjects: the role of two HLA-DQ high-risk haplotypes.	GeneRIF	0.00875	129	0.0149	0.152	1	2	HLA-DQB1
Pubmed	18539144	Association of endothelial protein C receptor haplotypes, factor V Leiden and recurrent first trimester pregnancy loss.	GeneRIF	0.00875	129	0.0149	0.152	1	2	PROCR
Pubmed	17613931	Screening for human cationic trypsinogen (PRSS1) and trypsinogen inhibitor gene (SPINK1) mutations in a Finnish family with hereditary pancreatitis.	GeneRIF	0.00875	129	0.0149	0.152	1	2	SPINK1

Category	ID	Name	Source	p-value	q-value Bonferroni	q-value FDR B&H	q-value FDR B&Y	Hit Count in Query List	Hit Count in Genome	Hit in Query List
Pubmed	23628399	Associations of HLA-DRB1 and -DQB1 alleles with severe recurrent respiratory papillomatosis in Korean patients.	Pubmed	0.00875	129	0.0149	0.152	1	2	HLA-DQB1
Pubmed	17142775	Inhibition of macrophage migration inhibitory factor or its receptor (CD74) attenuates growth and invasion of DU-145 prostate cancer cells.	GeneRIF	0.00875	129	0.0149	0.152	1	2	CD74
Pubmed	8344468	Tracing the incorporation of the sperm tail in the mouse zygote and early embryo using an anti-testicular alpha-tubulin antibody.	Pubmed	0.00875	129	0.0149	0.152	1	2	NGFR
Pubmed	19850645	Rosiglitazone activation of PPARgamma suppresses fractalkine signaling.	GeneRIF	0.00875	129	0.0149	0.152	1	2	CX3CL1
Pubmed	23161572	BMP2/BMP4 colorectal cancer susceptibility loci in northern and southern European populations.	GeneRIF	0.00875	129	0.0149	0.152	1	2	BMP4
Pubmed	25463304	Down-regulation of aldo-keto reductase AKR1B10 gene expression by a phorbol ester via the ERK/c-Jun signaling pathway.	Pubmed	0.00875	129	0.0149	0.152	1	2	AKR1B10
Pubmed	16799046	Arginine 54 and Tyrosine 118 residues of {alpha}A-crystallin are crucial for lens formation and transparency.	Pubmed	0.00875	129	0.0149	0.152	1	2	CRYAA
Pubmed	15290897	Comparative clinico-pathological study of saposin-A-deficient (SAP-A-/-) and Twitcher mice.	Pubmed	0.00875	129	0.0149	0.152	1	2	PSAP
Pubmed	19428990	PC2/CPE-mediated pro-protein processing in tumor cells and its differentiated cells or tissues.	GeneRIF	0.00875	129	0.0149	0.152	1	2	CPE
Pubmed	20581655	The interaction of ACP1, ADA1, diabetes and gender in coronary artery disease.	GeneRIF	0.00875	129	0.0149	0.152	1	2	ADA
Pubmed	12071546	HLA and hypocretin studies in Korean patients with narcolepsy.	GeneRIF	0.00875	129	0.0149	0.152	1	2	HLA-DQB1
Pubmed	15034026	Effect of decreasing the affinity of the class II-associated invariant chain peptide on the MHC class II peptide repertoire in the presence or absence of H-2M.	Pubmed	0.00875	129	0.0149	0.152	1	2	CD74
Pubmed	17672933	[Correlations of polymorphisms in matrix metalloproteinase-3 and -7 promoters to susceptibility to brain astrocytoma].	GeneRIF	0.00875	129	0.0149	0.152	1	2	MMP7
Pubmed	19221194	A concentration-dependent endocytic trap and sink mechanism converts Bmper from an activator to an inhibitor of Bmp signaling.	GeneRIF	0.00875	129	0.0149	0.152	1	2	BMP4
Pubmed	11322334	Expression of neurotrophin receptors in the developing and adult testis.	Pubmed	0.00875	129	0.0149	0.152	1	2	NGFR
Pubmed	20552250	Reconstruction of gene association network reveals a transmembrane protein required for adipogenesis and targeted by PPAR¿.	GeneRIF	0.00875	129	0.0149	0.152	1	2	APMAP
Pubmed	25420917	Involvement of the CX3CL1 (fractalkine)/CX3CR1 pathway in the pathogenesis of acute graft-versus-host disease.	Pubmed	0.00875	129	0.0149	0.152	1	2	CX3CL1
Pubmed	15817465	Insights into hydrophobicity and the chaperone-like function of alphaA- and alphaB-crystallins: an isothermal titration calorimetric study.	GeneRIF	0.00875	129	0.0149	0.152	1	2	CRYAA
Pubmed	17550612	hZip2 and hZip3 zinc transporters are down regulated in human prostate adenocarcinomatous glands.	GeneRIF	0.00875	129	0.0149	0.152	1	2	SLC39A2
Pubmed	21882634	[Influence of genetic mutations on clinical presentation of subretinal neovascularization. Report 2: The impact of HTRA and VEGF genes polymorphism].	GeneRIF	0.00875	129	0.0149	0.152	1	2	HTRA1
Pubmed	24378873	Elevated expression of activins promotes muscle wasting and cachexia.	Pubmed	0.00875	129	0.0149	0.152	1	2	INHBB
Pubmed	15220331	Alpha1B-adrenoceptor signaling and cell motility: GTPase function of Gh/transglutaminase 2 inhibits cell migration through interaction with cytoplasmic tail of integrin alpha subunits.	GeneRIF	0.00875	129	0.0149	0.152	1	2	TGM2
Pubmed	7730416	Transglutaminase-catalyzed matrix cross-linking in differentiating cartilage: identification of osteonectin as a major glutaminyl substrate.	Pubmed	0.00875	129	0.0149	0.152	1	2	TGM2
Pubmed	18178838	Regulation of CD1a surface expression and antigen presentation by invariant chain and lipid rafts.	GeneRIF	0.00875	129	0.0149	0.152	1	2	CD74
Pubmed	6585297	Both alpha and beta chains of HLA-DC class II histocompatibility antigens display extensive polymorphism in their amino-terminal domains.	Pubmed	0.00875	129	0.0149	0.152	1	2	HLA-DQB1
Pubmed	21676452	Paracrine SLPI secretion upregulates MMP-9 transcription and secretion in ovarian cancer cells.	GeneRIF	0.00875	129	0.0149	0.152	1	2	SLPI
Pubmed	10075846	A BMP-inducible gene, dlx5, regulates osteoblast differentiation and mesoderm induction.	Pubmed	0.00875	129	0.0149	0.152	1	2	BMP4
Pubmed	25310975	An MHC II-dependent activation loop between adipose tissue macrophages and CD4+ T cells controls obesity-induced inflammation.	Pubmed	0.00875	129	0.0149	0.152	1	2	HLA-DQB1
Pubmed	15239678	Matrilysin-1 (MMP-7) and MMP-19 are expressed by Paget's cells in extramammary Paget's disease.	GeneRIF	0.00875	129	0.0149	0.152	1	2	MMP7
Pubmed	17222801	Wnt/beta-catenin signaling regulates expression of PRDC, an antagonist of the BMP-4 signaling pathway.	GeneRIF	0.00875	129	0.0149	0.152	1	2	BMP4
Pubmed	16455621	Polymorphisms in the promoter regions of the matrix metalloproteinases-7, -9 and the risk of endometriosis and adenomyosis in China.	GeneRIF	0.00875	129	0.0149	0.152	1	2	MMP7
Pubmed	20157352	Genetic analysis of typical wet-type age-related macular degeneration and polypoidal choroidal vasculopathy in Japanese population.	GeneRIF	0.00875	129	0.0149	0.152	1	2	HTRA1
Pubmed	19592628	Maternal BMI before pregnancy, maternal weight gain during pregnancy, and risk of persistent positivity for multiple diabetes-associated autoantibodies in children with the high-risk HLA genotype: the MIDIA study.	GeneRIF	0.00875	129	0.0149	0.152	1	2	HLA-DQB1
Pubmed	25649006	Evaluation of human tissue kallikrein-related peptidases 6 and 10 expression in early gastroesophageal adenocarcinoma.	Pubmed	0.00875	129	0.0149	0.152	1	2	KLK10
Pubmed	22285803	Heterogeneous nuclear ribonucleoprotein A1 is a novel cellular target of atrial natriuretic peptide signaling in renal epithelial cells.	GeneRIF	0.00875	129	0.0149	0.152	1	2	NPPA

Category	ID	Name	Source	p-value	q-value Bonferroni	q-value FDR B&H	q-value FDR B&Y	Hit Count in Query List	Hit Count in Genome	Hit in Query List
Pubmed	11481455	Brain glucose utilization in mice with a targeted mutation in the thyroid hormone alpha or beta receptor gene.	Pubmed	0.00875	129	0.0149	0.152	1	2	THRA
Pubmed	21258343	Mutations in lectin complement pathway genes COLEC11 and MASP1 cause 3MC syndrome.	GeneRIF	0.00875	129	0.0149	0.152	1	2	MASP1
Pubmed	15166166	Nucleotide variation at Msn and Alas2, two genes flanking the centromere of the X chromosome in humans.	GeneRIF	0.00875	129	0.0149	0.152	1	2	ALAS2
Pubmed	17461431	Insulin VNTR I/III genotype is associated with autoantibodies against glutamic acid decarboxylase in newly diagnosed type 1 diabetes.	GeneRIF	0.00875	129	0.0149	0.152	1	2	HLA-DQB1
Pubmed	9815277	Immunohistochemical localization of carboxypeptidases E and D in the human placenta and umbilical cord.	Pubmed	0.00875	129	0.0149	0.152	1	2	CPE
Pubmed	16769963	Strong association of MuSK antibody-positive myasthenia gravis and HLA-DR14-DQ5.	GeneRIF	0.00875	129	0.0149	0.152	1	2	HLA-DQB1
Pubmed	24321473	HLA-DR, DQ class II DNA typing in pediatric panuveitis and tubulointerstitial nephritis and uveitis.	Pubmed	0.00875	129	0.0149	0.152	1	2	HLA-DQB1
Pubmed	11326276	Beta-catenin-sensitive isoforms of lymphoid enhancer factor-1 are selectively expressed in colon cancer.	Pubmed	0.00875	129	0.0149	0.152	1	2	MMP7
Pubmed	14614561	IA-2 is not required for the development of diabetes in NOD mice.	GeneRIF	0.00875	129	0.0149	0.152	1	2	HLA-DQB1
Pubmed	21464700	Combination of napsin A and TTF-1 immunohistochemistry helps in differentiating primary lung adenocarcinoma from metastatic carcinoma in the lung.	GeneRIF	0.00875	129	0.0149	0.152	1	2	NAPSA
Pubmed	24305782	Assessment of adenosine deaminase (ADA) activity and oxidative stress in patients with chronic tonsillitis.	Pubmed	0.00875	129	0.0149	0.152	1	2	ADA
Pubmed	10512193	Retinoic acid teratogenicity: the role of goosecoid and BMP-4.	Pubmed	0.00875	129	0.0149	0.152	1	2	BMP4
Pubmed	19403524	Tissue transglutaminase is an essential participant in the epidermal growth factor-stimulated signaling pathway leading to cancer cell migration and invasion.	GeneRIF	0.00875	129	0.0149	0.152	1	2	TGM2
Pubmed	23857504	The effects of MyD88 deficiency on disease phenotype in dysferlin-deficient A/J mice: role of endogenous TLR ligands.	Pubmed	0.00875	129	0.0149	0.152	1	2	DYSF
Pubmed	2997193	Sequence of gene and cDNA encoding murine major histocompatibility complex class II gene A beta 2.	Pubmed	0.00875	129	0.0149	0.152	1	2	HLA-DQB1
Pubmed	22155912	IL28B CC-genotype association with HLA-DQB1*0301 allele increases the prediction of spontaneous HCV RNA clearance in thalassaemic HCV-infected patients.	GeneRIF	0.00875	129	0.0149	0.152	1	2	HLA-DQB1
Pubmed	18573344	A bi-directional carboxypeptidase E-driven transport mechanism controls BDNF vesicle homeostasis in hippocampal neurons.	GeneRIF	0.00875	129	0.0149	0.152	1	2	CPE
Pubmed	20332100	The Nance-Horan syndrome protein encodes a functional WAVE homology domain (WHD) and is important for co-ordinating actin remodelling and maintaining cell morphology.	GeneRIF	0.00875	129	0.0149	0.152	1	2	ACTA1
Pubmed	14581400	Smooth muscle cells in human atherosclerotic plaques express the fractalkine receptor CX3CR1 and undergo chemotaxis to the CX3C chemokine fractalkine (CX3CL1).	GeneRIF	0.00875	129	0.0149	0.152	1	2	CX3CL1
Pubmed	20522589	Atrogin-1 affects muscle protein synthesis and degradation when energy metabolism is impaired by the antidiabetes drug berberine.	GeneRIF	0.00875	129	0.0149	0.152	1	2	FBXO32
Pubmed	17146758	Differential gene expression in skeletal muscle cells after membrane depolarization.	GeneRIF	0.00875	129	0.0149	0.152	1	2	ACTA1
Pubmed	7680654	Cortactin, an 80/85-kilodalton pp60src substrate, is a filamentous actin-binding protein enriched in the cell cortex.	Pubmed	0.00875	129	0.0149	0.152	1	2	ACTA1
Pubmed	6576979	Primary structure of human class II histocompatibility antigens 3rd communication. Amino acid sequence comparison between DR and DC subclass antigens derived from a lymphoblastoid B cell line homozygous at the HLA loci (HLA-A3,3; B7,7; Dw2,2; DR2,2: MT1,1; Dc1,1: MB1,1).	Pubmed	0.00875	129	0.0149	0.152	1	2	HLA-DQB1
Pubmed	21179475	A key role for E-cadherin in intestinal homeostasis and Paneth cell maturation.	GeneRIF	0.00875	129	0.0149	0.152	1	2	PREX1
Pubmed	20359117	[Expression and clinical significance of Ang-2 and MMP-7 in laryngeal carcinoma].	GeneRIF	0.00875	129	0.0149	0.152	1	2	MMP7
Pubmed	9244347	I-A beta gene expression regulation in macrophages derived from mice susceptible or resistant to infection with M. bovis BCG.	Pubmed	0.00875	129	0.0149	0.152	1	2	HLA-DQB1
Pubmed	15919833	Differential expression of the peripheral benzodiazepine receptor and gremlin during adipogenesis.	GeneRIF	0.00875	129	0.0149	0.152	1	2	TSPO
Pubmed	23200849	Ga(h)/transglutaminase-2 activity is required for maximal activation of adenylylcyclase 8 in human and rat glioma cells.	GeneRIF	0.00875	129	0.0149	0.152	1	2	TGM2
Pubmed	12925572	Glycosylation defects: a new mechanism for muscular dystrophy?	Pubmed	0.00875	129	0.0149	0.152	1	2	LARGE
Pubmed	20664794	Age-related macular degeneration-associated variants at chromosome 10q26 do not significantly alter ARMS2 and HTRA1 transcript levels in the human retina.	GeneRIF	0.00875	129	0.0149	0.152	1	2	HTRA1
Pubmed	19160088	High prevalence of four long QT syndrome founder mutations in the Finnish population.	GeneRIF	0.00875	129	0.0149	0.152	1	2	KCNH2
Pubmed	17539903	Gender, genotype, and phenotype differences in Smith-Magenis syndrome: a meta-analysis of 105 cases.	GeneRIF	0.00875	129	0.0149	0.152	1	2	MFAP4
Pubmed	12429719	The presence of stomatin in detergent-insoluble domains of neutrophil granule membranes.	GeneRIF	0.00875	129	0.0149	0.152	1	2	CD68
Pubmed	24716659	Critical role of histone demethylase RBP2 in human gastric cancer angiogenesis.	Pubmed	0.00875	129	0.0149	0.152	1	2	KDM5A
Pubmed	19188446	A tautomerase-null macrophage migration-inhibitory factor (MIF) gene knock-in mouse model reveals that protein interactions and not enzymatic activity mediate MIF-dependent growth regulation.	GeneRIF	0.00875	129	0.0149	0.152	1	2	CD74

Category	ID	Name	Source	p-value	q-value Bonferroni	q-value FDR B&H	q-value FDR B&Y	Hit Count in Query List	Hit Count in Genome	Hit in Query List
Pubmed	15492986	Identification of carboxypeptidase E and gamma-glutamyl hydrolase as biomarkers for pulmonary neuroendocrine tumors by cDNA microarray.	GeneRIF	0.00875	129	0.0149	0.152	1	2	CPE
Pubmed	15539616	Differential additive effects of endothelial lipase and scavenger receptor-class B type I on high-density lipoprotein metabolism in knockout mouse models.	Pubmed	0.00875	129	0.0149	0.152	1	2	LIPG
Pubmed	17947664	Posttranslational regulation of I-Ed by affinity for CLIP.	GeneRIF	0.00875	129	0.0149	0.152	1	2	CD74
Pubmed	16626672	Effects of adenosine deaminase and A1 receptor deficiency in normoxic and ischaemic mouse hearts.	Pubmed	0.00875	129	0.0149	0.152	1	2	ADA
Pubmed	16188098	[Relationship between pneumoconiosis and the polymorphisms of HLA-DRB1*, DQB1* genes].	GeneRIF	0.00875	129	0.0149	0.152	1	2	HLA-DQB1
Pubmed	22483801	Structures of the HIN domain:DNA complexes reveal ligand binding and activation mechanisms of the AIM2 inflammasome and IFI16 receptor.	GeneRIF	0.00875	129	0.0149	0.152	1	2	IFI16
Pubmed	6323162	Chromosomal localisation of the human homologues to the oncogenes erbA and B.	Pubmed	0.00875	129	0.0149	0.152	1	2	THRA
Pubmed	24275510	Alpha-crystallin-mediated protection of lens cells against heat and oxidative stress-induced cell death.	Pubmed	0.00875	129	0.0149	0.152	1	2	CRYAA
Pubmed	18192214	Genomic structure, transcriptional control, and tissue distribution of HERG1 and KCNQ1 genes.	GeneRIF	0.00875	129	0.0149	0.152	1	2	KCNH2
Pubmed	23645563	Expression of the chemokines CXCL12 and CX3CL1 and their receptors in human nerve sheath tumors.	Pubmed	0.00875	129	0.0149	0.152	1	2	CX3CL1
Pubmed	9915832	Cloning and characterization of PRAX-1. A new protein that specifically interacts with the peripheral benzodiazepine receptor.	Pubmed	0.00875	129	0.0149	0.152	1	2	TSPO
Pubmed	20538909	Interaction between PKD1L3 and PKD2L1 through their transmembrane domains is required for localization of PKD2L1 at taste pores in taste cells of circumvallate and foliate papillae.	GeneRIF	0.00875	129	0.0149	0.152	1	2	PKD2L1
Pubmed	21892902	Natriuretic peptide levels in intrauterine growth-restricted fetuses with absent and reversed end-diastolic flow of the umbilical artery in relation to ductus venosus flow velocities.	GeneRIF	0.00875	129	0.0149	0.152	1	2	NPPA
Pubmed	19432819	Temporal and contextual orchestration of cardiac fate by WNT-BMP synergy and threshold.	GeneRIF	0.00875	129	0.0149	0.152	1	2	BMP4
Pubmed	9037065	Intestinal tumorigenesis is suppressed in mice lacking the metalloproteinase matrilysin.	Pubmed	0.00875	129	0.0149	0.152	1	2	MMP7
Pubmed	12571085	High-level activation by a duodenum-specific enhancer requires functional GATA binding sites.	GeneRIF	0.00875	129	0.0149	0.152	1	2	ADA
Pubmed	22886282	Association of BMPR1A polymorphism, but not BMP4, with kidney size in full-term newborns.	GeneRIF	0.00875	129	0.0149	0.152	1	2	BMP4
Pubmed	10372556	Complete genomic structure of the mouse cathepsin K gene (Ctsk) and its localization next to the Arnt gene on mouse chromosome 3.	Pubmed	0.00875	129	0.0149	0.152	1	2	CTSK
Pubmed	24375405	Transglutaminase 2-dependent deamidation of glyceraldehyde-3-phosphate dehydrogenase promotes trophoblastic cell fusion.	Pubmed	0.00875	129	0.0149	0.152	1	2	TGM2
Pubmed	15944260	The role of host CD4 T cells in the pathogenesis of the chronic graft-versus-host model of systemic lupus erythematosus.	Pubmed	0.00875	129	0.0149	0.152	1	2	HLA-DQB1
Pubmed	12021143	The combination of several polymorphic amino acid residues in the DQalpha and DQbeta chains forms a domain structure pattern and is associated with insulin-dependent diabetes mellitus.	GeneRIF	0.00875	129	0.0149	0.152	1	2	HLA-DQB1
Pubmed	16455968	Role of MHC class II on memory B cells in post-germinal center B cell homeostasis and memory response.	Pubmed	0.00875	129	0.0149	0.152	1	2	HLA-DQB1
Pubmed	22588649	HLA-DRB1*1501 and HLA-DQB1*0301 alleles are positively associated with HPV16 infection-related Kazakh esophageal squamous cell carcinoma in Xinjiang China.	GeneRIF	0.00875	129	0.0149	0.152	1	2	HLA-DQB1
Pubmed	22224817	A large-scale population-based analysis of common genetic variation in the thyroid hormone receptor alpha locus and bone.	GeneRIF	0.00875	129	0.0149	0.152	1	2	THRA
Pubmed	24224958	Involvement of NT3 and P75(NTR) in photoreceptor degeneration following selective Müller cell ablation.	Pubmed	0.00875	129	0.0149	0.152	1	2	NGFR
Pubmed	22824791	Carboxypeptidase E: a negative regulator of the canonical Wnt signaling pathway.	GeneRIF	0.00875	129	0.0149	0.152	1	2	CPE
Pubmed	11278781	Analysis of the cyclic nucleotide binding domain of the HERG potassium channel and interactions with KCNE2.	Pubmed	0.00875	129	0.0149	0.152	1	2	KCNH2
Pubmed	5639009	Hemoglobin-Bibba or alpha-2-136Pro-beta 2, an unstable alpha chain abnormal hemoglobin.	Pubmed	0.00875	129	0.0149	0.152	1	2	HBA2
Pubmed	12529713	Cationic trypsinogen and pancreatic secretory trypsin inhibitor gene mutations in neonatal hypertrypsinaemia.	GeneRIF	0.00875	129	0.0149	0.152	1	2	SPINK1
Pubmed	11884591	Control of cell cycle exit and entry by protein kinase B-regulated forkhead transcription factors.	GeneRIF	0.00875	129	0.0149	0.152	1	2	FOXO4
Pubmed	18356404	Trends in high-risk HLA susceptibility genes among Colorado youth with type 1 diabetes.	GeneRIF	0.00875	129	0.0149	0.152	1	2	HLA-DQB1
Pubmed	18596036	Crystal structure of the CUB1-EGF-CUB2 domain of human MASP-1/3 and identification of its interaction sites with mannan-binding lectin and ficolins.	GeneRIF	0.00875	129	0.0149	0.152	1	2	MASP1
Pubmed	21059907	Ubiquitination regulates MHC class II-peptide complex retention and degradation in dendritic cells.	GeneRIF	0.00875	129	0.0149	0.152	1	2	HLA-DQB1
Pubmed	11937527	Activating immunity in the liver. II. IFN-beta attenuates NK cell-dependent liver injury triggered by liver NKT cell activation.	GeneRIF	0.00875	129	0.0149	0.152	1	2	HLA-DQB1
Pubmed	18829570	A bicistronic CYCLIN D1-TROP2 mRNA chimera demonstrates a novel oncogenic mechanism in human cancer.	GeneRIF	0.00875	129	0.0149	0.152	1	2	TACSTD2

Category	ID	Name	Source	p-value	q-value Bonferroni	q-value FDR B&H	q-value FDR B&Y	Hit Count in Query List	Hit Count in Genome	Hit in Query List
Pubmed	24909373	Dermatopontin is a novel regulator of the CdCl2-induced decrease in claudin-11 expression.	Pubmed	0.00875	129	0.0149	0.152	1	2	DPT
Pubmed	23454274	Protein kinase C delta is a substrate of tissue transglutaminase and a novel autoantigen in coeliac disease.	GeneRIF	0.00875	129	0.0149	0.152	1	2	TGM2
Pubmed	21970857	Loss of Trop2 promotes carcinogenesis and features of epithelial to mesenchymal transition in squamous cell carcinoma.	GeneRIF	0.00875	129	0.0149	0.152	1	2	TACSTD2
Pubmed	15824087	FOXO-dependent expression of the proapoptotic protein Bim: pivotal role for apoptosis signaling in endothelial progenitor cells.	GeneRIF	0.00875	129	0.0149	0.152	1	2	FOXO4
Pubmed	18032673	Modulation of semaphorin3A activity by p75 neurotrophin receptor influences peripheral axon patterning.	GeneRIF	0.00875	129	0.0149	0.152	1	2	NGFR
Pubmed	9620453	The early expression of Rev-erbbeta occurs in the developing nervous system of mouse embryo.	Pubmed	0.00875	129	0.0149	0.152	1	2	THRA
Pubmed	7572345	Mammalian phosphoribosyl-pyrophosphate synthetase.	Pubmed	0.00875	129	0.0149	0.152	1	2	PRPS2
Pubmed	21817962	LOC387715/HTRA1 variants and the response to combined photodynamic therapy with intravitreal bevacizumab for polypoidal choroidal vasculopathy.	GeneRIF	0.00875	129	0.0149	0.152	1	2	HTRA1
Pubmed	12579512	[Association between HLA-DRB1, DQB1 genes and pemphigus vulgaris in Chinese Hans].	GeneRIF	0.00875	129	0.0149	0.152	1	2	HLA-DQB1
Pubmed	23100431	Deficiency of the microglial receptor CX3CR1 impairs postnatal functional development of thalamocortical synapses in the barrel cortex.	GeneRIF	0.00875	129	0.0149	0.152	1	2	CX3CL1
Pubmed	22443068	[Effect of fluoride on the expression of StAR mRNA and P450scc mRNA in the progesterone synthesis of mouse Leydig tumor cells].	GeneRIF	0.00875	129	0.0149	0.152	1	2	CYP11A1
Pubmed	23899066	TTF-1 and napsin A do not differentiate metastatic lung adenocarcinomas from primary esophageal adenocarcinomas: proposal of a novel staining panel.	Pubmed	0.00875	129	0.0149	0.152	1	2	NAPSA
Pubmed	11443354	The evolution of an alternatively spliced exon in the alphaA-crystallin gene.	Pubmed	0.00875	129	0.0149	0.152	1	2	CRYAA
Pubmed	21602817	Differential expression of cathepsin K in neoplasms harboring TFE3 gene fusions.	GeneRIF	0.00875	129	0.0149	0.152	1	2	CTSK
Pubmed	24713697	Neurogranin alters the structure and calcium binding properties of calmodulin.	Pubmed	0.00875	129	0.0149	0.152	1	2	NRGN
Pubmed	17389015	HLA-DRB1*0402 haplotypes without DQB1*0302 in Venezuelan patients with pemphigus vulgaris.	GeneRIF	0.00875	129	0.0149	0.152	1	2	HLA-DQB1
Pubmed	24205408	Intracellular modulation, extracellular disposal and serum increase of MiR-150 mark lymphocyte activation.	Pubmed	0.00875	129	0.0149	0.152	1	2	HLA-DQB1
Pubmed	23559013	A DR6/p75(NTR) complex is responsible for ß-amyloid-induced cortical neuron death.	GeneRIF	0.00875	129	0.0149	0.152	1	2	NGFR
Pubmed	18801688	HLA polymorphism and early rheumatoid arthritis in the Moroccan population.	GeneRIF	0.00875	129	0.0149	0.152	1	2	HLA-DQB1
Pubmed	18316565	Prognostic significance of macrophage infiltration in leiomyosarcomas.	GeneRIF	0.00875	129	0.0149	0.152	1	2	CD68
Pubmed	11920200	Molecular basis of haemoglobinopathies and G6PD deficiency in the Comorian population.	GeneRIF	0.00875	129	0.0149	0.152	1	2	HBB
Pubmed	16009967	Nerve growth factor and its precursor differentially regulate hair cycle progression in mice.	Pubmed	0.00875	129	0.0149	0.152	1	2	NGFR
Pubmed	15939815	Calcineurin-nuclear factor of activated T cells pathway-dependent cardiac remodeling in mice deficient in guanylyl cyclase A, a receptor for atrial and brain natriuretic peptides.	GeneRIF	0.00875	129	0.0149	0.152	1	2	NPPA
Pubmed	15498831	Peripheral-type benzodiazepine receptor-mediated action of steroidogenic acute regulatory protein on cholesterol entry into leydig cell mitochondria.	GeneRIF	0.00875	129	0.0149	0.152	1	2	TSPO
Pubmed	18949065	Isolation and characterization of betaA3-crystallin associated proteinase from alpha-crystallin fraction of human lenses.	GeneRIF	0.00875	129	0.0149	0.152	1	2	CRYAA
Pubmed	11160278	Diverse repertoire of the MHC class II-peptide complexes is required for presentation of viral superantigens.	Pubmed	0.00875	129	0.0149	0.152	1	2	CD74
Pubmed	25637537	Cysteine dioxygenase type 1 promotes adipogenesis via interaction with peroxisome proliferator-activated receptor gamma.	Pubmed	0.00875	129	0.0149	0.152	1	2	CDO1
Pubmed	17895402	Endothelial damage from cytomegalovirus-specific host immune response can be prevented by targeted disruption of fractalkine-CX3CR1 interaction.	GeneRIF	0.00875	129	0.0149	0.152	1	2	CX3CL1
Pubmed	15120191	HLA-DRB1 alleles and HLA-DRB1 shared epitopes are markers for juvenile rheumatoid arthritis subgroups in Colombian mestizos.	GeneRIF	0.00875	129	0.0149	0.152	1	2	HLA-DQB1
Pubmed	17403029	Type 2 transglutaminase differentially modulates striatal cell death in the presence of wild type or mutant huntingtin.	GeneRIF	0.00875	129	0.0149	0.152	1	2	TGM2
Pubmed	17364902	Molecular genetic studies on relationships among longevity, diseases, and HLA-DRB1/DQB1 allelic polymorphism.	GeneRIF	0.00875	129	0.0149	0.152	1	2	HLA-DQB1
Pubmed	7635525	Presensitization by skin grafting from major histocompatibility complex class I or major histocompatibility complex class II deficient mice identifies class I antigens as inducers of allosensitization.	Pubmed	0.00875	129	0.0149	0.152	1	2	HLA-DQB1
Pubmed	9685215	Prominent sex steroid metabolism in human lymphocytes.	Pubmed	0.00875	129	0.0149	0.152	1	2	CYP11A1
Pubmed	21389256	Conventional dendritic cells are required for the activation of helper-dependent CD8 T cell responses to a model antigen after cutaneous vaccination with lentiviral vectors.	Pubmed	0.00875	129	0.0149	0.152	1	2	HLA-DQB1
Pubmed	21912971	The effect of inflammation on the expression and distribution of the MAS-related gene receptors MrgE and MrgF in the murine ileum.	GeneRIF	0.00875	129	0.0149	0.152	1	2	MRGPRF
Pubmed	23593460	Functional deficits in PAK5, PAK6 and PAK5/PAK6 knockout mice.	Pubmed	0.00875	129	0.0149	0.152	1	2	PAK6
Pubmed	22682622	Dystrophin and dysferlin double mutant mice: a novel model for rhabdomyosarcoma.	GeneRIF	0.00875	129	0.0149	0.152	1	2	DYSF

Category	ID	Name	Source	p-value	q-value Bonferroni	q-value FDR B&H	q-value FDR B&Y	Hit Count in Query List	Hit Count in Genome	Hit in Query List
Pubmed	24858807	Pulmonary hypertension in wild type mice and animals with genetic deficit in KCa2.3 and KCa3.1 channels.	Pubmed	0.00875	129	0.0149	0.152	1	2	KCNN4
Pubmed	23603386	Klotho sensitivity of the hERG channel.	GeneRIF	0.00875	129	0.0149	0.152	1	2	KCNH2
Pubmed	15334053	Identification of SCN3B as a novel p53-inducible proapoptotic gene.	GeneRIF	0.00875	129	0.0149	0.152	1	2	SCN3B
Pubmed	23852617	Common polymorphisms in the CYP1A1 and CYP11A1 genes and polycystic ovary syndrome risk: a meta-analysis and meta-regression.	Pubmed	0.00875	129	0.0149	0.152	1	2	CYP11A1
Pubmed	25594584	Distinctive expression patterns of glycoprotein non metastatic B and folliculin in renal tumors in patients with Birt-Hogg-Dubé syndrome.	Pubmed	0.00875	129	0.0149	0.152	1	2	GPNMB
Pubmed	9786936	The plasminogen binding site of the C-type lectin tetranectin is located in the carbohydrate recognition domain, and binding is sensitive to both calcium and lysine.	Pubmed	0.00875	129	0.0149	0.152	1	2	CLEC3B
Pubmed	16639013	A transgenic mouse model for human autosomal dominant cataract.	Pubmed	0.00875	129	0.0149	0.152	1	2	CRYAA
Pubmed	8082810	Tyrosine-rich acidic matrix protein (TRAMP) is a tyrosine-sulphated and widely distributed protein of the extracellular matrix.	Pubmed	0.00875	129	0.0149	0.152	1	2	DPT
Pubmed	18049479	Rap1 is involved in the signal transduction of myelin-associated glycoprotein.	GeneRIF	0.00875	129	0.0149	0.152	1	2	NGFR
Pubmed	24788416	Insulin resistance aggravates atherosclerosis by reducing vascular smooth muscle cell survival and increasing CX3CL1/CX3CR1 axis.	Pubmed	0.00875	129	0.0149	0.152	1	2	CX3CL1
Pubmed	18191841	Levels of steroidogenic acute regulatory protein and mitochondrial membrane potential in granulosa cells of older poor-responder women.	GeneRIF	0.00875	129	0.0149	0.152	1	2	CYP11A1
Pubmed	23158025	Distinguishing deficiencies in the steroidogenic acute regulatory protein and the cholesterol side chain cleavage enzyme causing neonatal adrenal failure.	GeneRIF	0.00875	129	0.0149	0.152	1	2	CYP11A1
Pubmed	16720210	The relationship between HLA class II polymorphisms and somatic deletions in testicular B cell lymphomas of Dutch patients.	GeneRIF	0.00875	129	0.0149	0.152	1	2	HLA-DQB1
Pubmed	21533062	LARGE expression augments the glycosylation of glycoproteins in addition to a-dystroglycan conferring laminin binding.	GeneRIF	0.00875	129	0.0149	0.152	1	2	LARGE
Pubmed	17662998	Characterisation of amyloid fibril formation by small heat-shock chaperone proteins human alphaA-, alphaB- and R120G alphaB-crystallins.	GeneRIF	0.00875	129	0.0149	0.152	1	2	CRYAA
Pubmed	9230439	A minK-HERG complex regulates the cardiac potassium current I(Kr).	Pubmed	0.00875	129	0.0149	0.152	1	2	KCNH2
Pubmed	19389928	Monokine induced by interferon-gamma (MIG/CXCL9) is derived from both donor and recipient sources during rejection of class II major histocompatibility complex disparate skin allografts.	Pubmed	0.00875	129	0.0149	0.152	1	2	HLA-DQB1
Pubmed	21959449	Isolation of epithelial cells in the developing primary lip and palate.	GeneRIF	0.00875	129	0.0149	0.152	1	2	BMP4
Pubmed	12442276	KCNQ1 and KCNH2 mutations associated with long QT syndrome in a Chinese population.	GeneRIF	0.00875	129	0.0149	0.152	1	2	KCNH2
Pubmed	15817638	Beta2-adrenoceptor regulation of the K+ channel iKCa1 in human mast cells.	GeneRIF	0.00875	129	0.0149	0.152	1	2	KCNN4
Pubmed	22569264	Clusterin is a specific stabilizer and liberator of extracellular cathepsin K.	GeneRIF	0.00875	129	0.0149	0.152	1	2	CTSK
Pubmed	22048274	Fucose and sialic acid expressions in human seminal fibronectin and a{1} -acid glycoprotein associated with leukocytospermia of infertile men.	GeneRIF	0.00875	129	0.0149	0.152	1	2	ORM1
Pubmed	12163017	Expression and biological activity of Baculovirus generated wild-type human slow alpha tropomyosin and the Met9Arg mutant responsible for a dominant form of nemaline myopathy.	Pubmed	0.00875	129	0.0149	0.152	1	2	ACTA1
Pubmed	20374310	Histological analysis of synovium by treatment of etanercept for rheumatoid arthritis.	GeneRIF	0.00875	129	0.0149	0.152	1	2	CD68
Pubmed	16771953	Gastrointestinal symptoms in families of patients with an SCN5A-encoded cardiac channelopathy: evidence of an intestinal channelopathy.	GeneRIF	0.00875	129	0.0149	0.152	1	2	KCNH2
Pubmed	10348820	Involvement of T3Ralpha- and beta-receptor subtypes in mediation of T3 functions during postnatal murine intestinal development.	Pubmed	0.00875	129	0.0149	0.152	1	2	THRA
Pubmed	16061944	Gaucher disease mouse models: point mutations at the acid beta-glucosidase locus combined with low level prosaposin expression lead to disease variants.	Pubmed	0.00875	129	0.0149	0.152	1	2	PSAP
Pubmed	21172909	Endothelium-dependent vasodilation in human mesenteric artery is primarily mediated by myoendothelial gap junctions intermediate conductance calcium-activated K+ channel and nitric oxide.	GeneRIF	0.00875	129	0.0149	0.152	1	2	KCNN4
Pubmed	19812988	Differential association of HLA with three subtypes of type 1 diabetes: fulminant, slowly progressive and acute-onset.	GeneRIF	0.00875	129	0.0149	0.152	1	2	HLA-DQB1
Pubmed	19288145	Linking conformation change to hemoglobin activation via chain-selective time-resolved resonance Raman spectroscopy of protoheme/mesoheme hybrids.	GeneRIF	0.00875	129	0.0149	0.152	1	2	HBB
Pubmed	20826138	Presence and functional role of the rapidly activating delayed rectifier K(+) current in left and right atria of adult mice.	GeneRIF	0.00875	129	0.0149	0.152	1	2	KCNH2
Pubmed	15767343	Thyroid cancer susceptibility and THRA1 and BAT-40 repeats polymorphisms.	GeneRIF	0.00875	129	0.0149	0.152	1	2	THRA
Pubmed	20431619	Autonomous role of medullary thymic epithelial cells in central CD4(+) T cell tolerance.	Pubmed	0.00875	129	0.0149	0.152	1	2	HLA-DQB1
Pubmed	20851114	Development of a high resolution melting method for the detection of genetic variations in Long QT Syndrome.	GeneRIF	0.00875	129	0.0149	0.152	1	2	KCNH2
Pubmed	15800062	Neutrophil migration across tight junctions is mediated by adhesive interactions between epithelial coxsackie and adenovirus receptor and a junctional adhesion molecule-like protein on neutrophils.	Pubmed	0.00875	129	0.0149	0.152	1	2	AMICA1

Category	ID	Name	Source	p-value	q-value Bonferroni	q-value FDR B&H	q-value FDR B&Y	Hit Count in Query List	Hit Count in Genome	Hit in Query List
Pubmed	18599937	HLA-DQB1*05 association with Hashimoto's thyroiditis in children of Northern Greek origin.	GeneRIF	0.00875	129	0.0149	0.152	1	2	HLA-DQB1
Pubmed	11132230	Few reports of hemoglobin E/beta-thalassemia in Northeast India: underdiagnosis or complete exclusion of beta-thalassemia by hemoglobin E.	GeneRIF	0.00875	129	0.0149	0.152	1	2	HBB
Pubmed	15871822	[Serological and genetic markers in the diagnosis and follow-up of coeliac disease].	GeneRIF	0.00875	129	0.0149	0.152	1	2	HLA-DQB1
Pubmed	3371836	[Structural analysis of human major histocompatibility complex class II genes].	Pubmed	0.00875	129	0.0149	0.152	1	2	HLA-DQB1
Pubmed	15749087	Expression pattern of NOGO and NgR genes during human development.	GeneRIF	0.00875	129	0.0149	0.152	1	2	RTN4R
Pubmed	16891422	Transient receptor potential family members PKD1L3 and PKD2L1 form a candidate sour taste receptor.	GeneRIF	0.00875	129	0.0149	0.152	1	2	PKD2L1
Pubmed	20813954	The junctional adhesion molecule JAML is a costimulatory receptor for epithelial gammadelta T cell activation.	GeneRIF	0.00875	129	0.0149	0.152	1	2	AMICA1
Pubmed	9790779	The linked human elongation initiation factor 4A1 (EIF4A1) and CD68 genes map to chromosome 17p13.	Pubmed	0.00875	129	0.0149	0.152	1	2	CD68
Pubmed	15126315	Expression of the chemokine receptor CCR2 on immature B cells negatively regulates their cytoskeletal rearrangement and migration.	GeneRIF	0.00875	129	0.0149	0.152	1	2	CD74
Pubmed	8055537	Expression of mRNA of murine bone-related proteins in ectopic bone induced by murine bone morphogenetic protein-4.	Pubmed	0.00875	129	0.0149	0.152	1	2	BMP4
Pubmed	17126830	MHC class II genes in Mexican patients with idiopathic dilated cardiomyopathy.	GeneRIF	0.00875	129	0.0149	0.152	1	2	HLA-DQB1
Pubmed	10657990	Nuclear localization of PAPS synthetase 1: a sulfate activation pathway in the nucleus of eukaryotic cells.	Pubmed	0.00875	129	0.0149	0.152	1	2	PAPSS2
Pubmed	18033314	Sézary syndrome is a unique cutaneous T-cell lymphoma as identified by an expanded gene signature including diagnostic marker molecules CDO1 and DNM3.	GeneRIF	0.00875	129	0.0149	0.152	1	2	CDO1
Pubmed	19728912	[Expression and clinical significance of survivin and matrix metalloproteinase-7 in colon cancer].	GeneRIF	0.00875	129	0.0149	0.152	1	2	MMP7
Pubmed	16127462	Human aldose reductase expression accelerates diabetic atherosclerosis in transgenic mice.	GeneRIF	0.00875	129	0.0149	0.152	1	2	AKR1B10
Pubmed	17471309	Recruitment of CD16+ monocytes into synovial tissues is mediated by fractalkine and CX3CR1 in rheumatoid arthritis patients.	GeneRIF	0.00875	129	0.0149	0.152	1	2	CX3CL1
Pubmed	19729679	Translocator protein 2 is involved in cholesterol redistribution during erythropoiesis.	GeneRIF	0.00875	129	0.0149	0.152	1	2	TSPO
Pubmed	11566198	The solution structure and heme binding of the presequence of murine 5-aminolevulinate synthase.	Pubmed	0.00875	129	0.0149	0.152	1	2	ALAS2
Pubmed	18718667	The HtrA1 promoter polymorphism, smoking, and age-related macular degeneration in multiple case-control samples.	GeneRIF	0.00875	129	0.0149	0.152	1	2	HTRA1
Pubmed	16301320	Phosphorylation of P-Rex1 by the cyclic AMP-dependent protein kinase inhibits the phosphatidylinositiol (3,4,5)-trisphosphate and Gbetagamma-mediated regulation of its activity.	GeneRIF	0.00875	129	0.0149	0.152	1	2	PREX1
Pubmed	10430610	Divergent roles for thyroid hormone receptor beta isoforms in the endocrine axis and auditory system.	Pubmed	0.00875	129	0.0149	0.152	1	2	THRA
Pubmed	17182967	Cooperation between MASP-1 and MASP-2 in the generation of C3 convertase through the MBL pathway.	Pubmed	0.00875	129	0.0149	0.152	1	2	MASP1
Pubmed	22562303	Tumor necrosis factor alpha stimulates cathepsin K and V activity via juxtacrine monocyte-endothelial cell signaling and JNK activation.	GeneRIF	0.00875	129	0.0149	0.152	1	2	CTSK
Pubmed	24794251	Infiltration of CD3¿ and CD68¿ cells in bladder cancer is subtype specific and affects the outcome of patients with muscle-invasive tumors.	Pubmed	0.00875	129	0.0149	0.152	1	2	CD68
Pubmed	17050061	Tissue kallikrein and kininogen levels in fetoplacental tissues from normotensive pregnant women and women with pregnancy-induced hypertension.	GeneRIF	0.00875	129	0.0149	0.152	1	2	KLK1
Pubmed	21411747	Transcriptional regulation of bone morphogenetic protein 4 by tumor necrosis factor and its relationship with age-related macular degeneration.	GeneRIF	0.00875	129	0.0149	0.152	1	2	BMP4
Pubmed	20971723	Globotriaosylceramide leads to K(Ca)3.1 channel dysfunction: a new insight into endothelial dysfunction in Fabry disease.	GeneRIF	0.00875	129	0.0149	0.152	1	2	KCNN4
Pubmed	277329	Characterization and kinetics of synthesis of 15S beta-globin RNA, a putative precursor of beta-globin mRNA.	Pubmed	0.00875	129	0.0149	0.152	1	2	HBA2
Pubmed	20354171	Bone marrow graft-versus-host disease: early destruction of hematopoietic niche after MHC-mismatched hematopoietic stem cell transplantation.	Pubmed	0.00875	129	0.0149	0.152	1	2	HLA-DQB1
Pubmed	12039527	HLA DQA1-DQB1 genotypes in Bedouin families with celiac disease.	GeneRIF	0.00875	129	0.0149	0.152	1	2	HLA-DQB1
Pubmed	25253694	p75 regulates Purkinje cell firing by modulating SK channel activity through Rac1.	Pubmed	0.00875	129	0.0149	0.152	1	2	NGFR
Pubmed	15766562	KLK5 and KLK7, two members of the human tissue kallikrein family, are differentially expressed in lung cancer.	GeneRIF	0.00875	129	0.0149	0.152	1	2	KLK7
Pubmed	24226422	CD271 on melanoma cell is an IFN-¿-inducible immunosuppressive factor that mediates downregulation of melanoma antigens.	Pubmed	0.00875	129	0.0149	0.152	1	2	NGFR
Pubmed	16102832	Heterogeneity of MBL-MASP complexes.	GeneRIF	0.00875	129	0.0149	0.152	1	2	MASP1
Pubmed	20643647	Molecular characterization of N-acetylaspartylglutamate synthetase.	Pubmed	0.00875	129	0.0149	0.152	1	2	RIMKLB
Pubmed	18055444	Identification of a novel putative gastrointestinal stem cell and adenoma stem cell marker, doublecortin and CaM kinase-like-1, following radiation injury and in adenomatous polyposis coli/multiple intestinal neoplasia mice.	GeneRIF	0.00875	129	0.0149	0.152	1	2	DCLK1

Category	ID	Name	Source	p-value	q-value Bonferroni	q-value FDR B&H	q-value FDR B&Y	Hit Count in Query List	Hit Count in Genome	Hit in Query List
Pubmed	19794069	Binding of DC-HIL to dermatophytic fungi induces tyrosine phosphorylation and potentiates antigen presenting cell function.	GeneRIF	0.00875	129	0.0149	0.152	1	2	GPNMB
Pubmed	11959863	Calcium-sensitive phospholipid binding properties of normal and mutant ferlin C2 domains.	GeneRIF	0.00875	129	0.0149	0.152	1	2	DYSF
Pubmed	24210685	PSORS1C1/CDSN is associated with ankylosing spondylitis.	Pubmed	0.00875	129	0.0149	0.152	1	2	CDSN
Pubmed	18291005	Haptoglobin genotype, anaemia and malaria in Gambian children.	GeneRIF	0.00875	129	0.0149	0.152	1	2	HBB
Pubmed	18209473	Cellular localization of THIK-1 (K(2P)13.1) and THIK-2 (K(2P)12.1) K channels in the mammalian kidney.	GeneRIF	0.00875	129	0.0149	0.152	1	2	KCNK12
Pubmed	7527351	Development of cytochrome P-450 side chain cleavage mRNA levels in neonatal ovaries of normal and hypogonadal (hpg) mice.	Pubmed	0.00875	129	0.0149	0.152	1	2	CYP11A1
Pubmed	16225776	Association of HLA-DQ with idiopathic dilated cardiomyopathy in a northern Chinese Han population.	GeneRIF	0.00875	129	0.0149	0.152	1	2	HLA-DQB1
Pubmed	11667971	The role of secretory leukocyte proteinase inhibitor and elafin (elastase-specific inhibitor/skin-derived antileukoprotease) as alarm antiproteinases in inflammatory lung disease.	GeneRIF	0.00875	129	0.0149	0.152	1	2	SLPI
Pubmed	22158624	Bone morphogenetic protein 4 promotes vascular smooth muscle contractility by activating microRNA-21 (miR-21), which down-regulates expression of family of dedicator of cytokinesis (DOCK) proteins.	GeneRIF	0.00875	129	0.0149	0.152	1	2	BMP4
Pubmed	16453294	A murine model of mixed connective tissue disease induced with U1 small nuclear RNP autoantigen.	Pubmed	0.00875	129	0.0149	0.152	1	2	HLA-DQB1
Pubmed	21075156	High frequency of low-risk human leukocyte antigen class II genotypes in latent celiac disease.	GeneRIF	0.00875	129	0.0149	0.152	1	2	HLA-DQB1
Pubmed	22509268	HLA-DRB1-DQB1 haplotypes confer susceptibility and resistance to multiple sclerosis in Sardinia.	GeneRIF	0.00875	129	0.0149	0.152	1	2	HLA-DQB1
Pubmed	19028686	Cathepsin K activity-dependent regulation of osteoclast actin ring formation and bone resorption.	GeneRIF	0.00875	129	0.0149	0.152	1	2	CTSK
Pubmed	16113830	Influence of the 4600A/G and 4678G/C polymorphisms in the endothelial protein C receptor (EPCR) gene on the risk of venous thromboembolism in carriers of factor V Leiden.	GeneRIF	0.00875	129	0.0149	0.152	1	2	PROCR
Pubmed	19095674	Expression of secretory leukocyte protease inhibitor and elafin in human fallopian tube and in an in-vitro model of Chlamydia trachomatis infection.	GeneRIF	0.00875	129	0.0149	0.152	1	2	SLPI
Pubmed	12776178	Effects of ligand and thyroid hormone receptor isoforms on hepatic gene expression profiles of thyroid hormone receptor knockout mice.	GeneRIF	0.00875	129	0.0149	0.152	1	2	THRA
Pubmed	11868825	Secretory leukocyte proteinase inhibitor and elafin are resistant to degradation by MMP-8.	GeneRIF	0.00875	129	0.0149	0.152	1	2	SLPI
Pubmed	14630998	Depressed thermogenesis but competent brown adipose tissue recruitment in mice devoid of all hormone-binding thyroid hormone receptors.	Pubmed	0.00875	129	0.0149	0.152	1	2	THRA
Pubmed	21901639	Differential gene expression of muscle-specific ubiquitin ligase MAFbx/Atrogin-1 and MuRF1 in response to immobilization-induced atrophy of slow-twitch and fast-twitch muscles.	GeneRIF	0.00875	129	0.0149	0.152	1	2	FBXO32
Pubmed	12878360	HLA class II genotypic frequencies in atopic asthma: association of DRB1*01-DQB1*0501 genotype with Artemisia vulgaris allergic asthma.	GeneRIF	0.00875	129	0.0149	0.152	1	2	HLA-DQB1
Pubmed	25609432	Endothelial CD74 mediates macrophage migration inhibitory factor protection in hyperoxic lung injury.	Pubmed	0.00875	129	0.0149	0.152	1	2	CD74
Pubmed	24381310	A histone deacetylase inhibitor improves hypothyroidism caused by a TRa1 mutant.	Pubmed	0.00875	129	0.0149	0.152	1	2	THRA
Pubmed	18844476	Identification and consequences of polymorphisms in the thyroid hormone receptor alpha and beta genes.	GeneRIF	0.00875	129	0.0149	0.152	1	2	THRA
Pubmed	23402018	The role of MASP-1/3 in complement activation.	GeneRIF	0.00875	129	0.0149	0.152	1	2	MASP1
Pubmed	21952138	Interaction between a novel intronic IVS3+172 variant and N29I mutation in PRSS1 gene is associated with pancreatitis in a Malaysian Chinese family.	GeneRIF	0.00875	129	0.0149	0.152	1	2	SPINK1
Pubmed	19282388	Interference of a mutant thyroid hormone receptor alpha1 with hepatic glucose metabolism.	GeneRIF	0.00875	129	0.0149	0.152	1	2	THRA
Pubmed	19500772	Genomic and genic deletions of the FOX gene cluster on 16q24.1 and inactivating mutations of FOXF1 cause alveolar capillary dysplasia and other malformations.	GeneRIF	0.00875	129	0.0149	0.152	1	2	STRA6
Pubmed	15985430	Involvement of inflammation, degradation, and apoptosis in a mouse model of glaucoma.	Pubmed	0.00875	129	0.0149	0.152	1	2	GPNMB
Pubmed	20141543	Enhancement of effector CD8+ T-cell function by tumour-associated B7-H3 and modulation of its counter-receptor triggering receptor expressed on myeloid cell-like transcript 2 at tumour sites.	GeneRIF	0.00875	129	0.0149	0.152	1	2	CD276
Pubmed	14998624	Long QT syndrome in neonates: conduction disorders associated with HERG mutations and sinus bradycardia with KCNQ1 mutations.	GeneRIF	0.00875	129	0.0149	0.152	1	2	KCNH2
Pubmed	16914520	Association of 14-3-3 proteins to beta1-adrenergic receptors modulates Kv11.1 K+ channel activity in recombinant systems.	GeneRIF	0.00875	129	0.0149	0.152	1	2	KCNH2
Pubmed	11067867	Metabolites from apoptotic thymocytes inhibit thymopoiesis in adenosine deaminase-deficient fetal thymic organ cultures.	Pubmed	0.00875	129	0.0149	0.152	1	2	ADA
Pubmed	18353867	TGFbeta mediates activation of transglutaminase 2 in response to oxidative stress that leads to protein aggregation.	GeneRIF	0.00875	129	0.0149	0.152	1	2	TGM2

Category	ID	Name	Source	p-value	q-value Bonferroni	q-value FDR B&H	q-value FDR B&Y	Hit Count in Query List	Hit Count in Genome	Hit in Query List
Pubmed	10559302	Activation of the PAK-related kinase by human immunodeficiency virus type 1 Nef in primary human peripheral blood lymphocytes and macrophages leads to phosphorylation of a PIX-p95 complex.	Pubmed	0.00875	129	0.0149	0.152	1	2	ARHGEF6
Pubmed	7626442	Role of the peripheral-type benzodiazepine receptor and the polypeptide diazepam binding inhibitor in steroidogenesis.	Pubmed	0.00875	129	0.0149	0.152	1	2	TSPO
Pubmed	12673836	Effect of alpha-globin genotype on the pathophysiology of sickle cell disease.	GeneRIF	0.00875	129	0.0149	0.152	1	2	HBA2
Pubmed	18953252	Expression of kallikrein 7 diminishes pancreatic cancer cell adhesion to vitronectin and enhances urokinase-type plasminogen activator receptor shedding.	GeneRIF	0.00875	129	0.0149	0.152	1	2	KLK7
Pubmed	15388868	The reaction of alpha-crystallin with the cross-linker 3,3'-dithiobis(sulfosuccinimidyl propionate) demonstrates close proximity of the C termini of alphaA and alphaB in the native assembly.	GeneRIF	0.00875	129	0.0149	0.152	1	2	CRYAA
Pubmed	16760894	The interaction between alphaA- and alphaB-crystallin is sequence-specific.	GeneRIF	0.00875	129	0.0149	0.152	1	2	CRYAA
Pubmed	13872627	[The structure of normal adult human hemoglobins].	Pubmed	0.00875	129	0.0149	0.152	1	2	HBB
Pubmed	20722389	Trichohyalin is a potential major autoantigen in human alopecia areata.	GeneRIF	0.00875	129	0.0149	0.152	1	2	TCHH
Pubmed	21975552	The filopodium: a stable structure with highly regulated repetitive cycles of elongation and persistence depending on the actin cross-linker fascin.	GeneRIF	0.00875	129	0.0149	0.152	1	2	ACTA1
Pubmed	22069105	Increased risk of type 1 diabetes in Polish children association with INS-IGF2 5'VNTR and lack of association with HLA haplotype.	GeneRIF	0.00875	129	0.0149	0.152	1	2	CD74
Pubmed	2263504	Two different genes encode delta-aminolevulinate synthase in humans: nucleotide sequences of cDNAs for the housekeeping and erythroid genes.	Pubmed	0.00875	129	0.0149	0.152	1	2	ALAS2
Pubmed	21451526	Fractalkine and its receptor, CX3CR1, promote hypertensive interstitial fibrosis in the kidney.	GeneRIF	0.00875	129	0.0149	0.152	1	2	CX3CL1
Pubmed	24853027	Antigenic peptide molecular recognition by the DRB1-DQB1 haplotype modulates multiple sclerosis susceptibility.	Pubmed	0.00875	129	0.0149	0.152	1	2	HLA-DQB1
Pubmed	8395021	Structure of gelsolin segment 1-actin complex and the mechanism of filament severing.	Pubmed	0.00875	129	0.0149	0.152	1	2	ACTA1
Pubmed	1383230	The EGF receptor is an actin-binding protein.	Pubmed	0.00875	129	0.0149	0.152	1	2	ACTA1
Pubmed	18974396	Expression of an IFN-inducible cellular senescence gene, IFI16, is up-regulated by p53.	GeneRIF	0.00875	129	0.0149	0.152	1	2	IFI16
Pubmed	19351602	Resistin up-regulates fractalkine expression in human endothelial cells: lack of additive effect with TNF-alpha.	GeneRIF	0.00875	129	0.0149	0.152	1	2	CX3CL1
Pubmed	11959145	The calcium-binding EF-hand in polycystin-L is not a domain for channel activation and ensuing inactivation.	GeneRIF	0.00875	129	0.0149	0.152	1	2	PKD2L1
Pubmed	20959647	Expression of the vasoactive proteins AT1, AT2, and ANP by pregnancy-induced mouse uterine natural killer cells.	GeneRIF	0.00875	129	0.0149	0.152	1	2	NPPA
Pubmed	7663508	Hyperproinsulinaemia in obese fat/fat mice associated with a carboxypeptidase E mutation which reduces enzyme activity.	Pubmed	0.00875	129	0.0149	0.152	1	2	CPE
Pubmed	21336735	Correlation of polymorphism of IL-8 and MMP-7 with occurrence and lymph node metastasis of early stage cervical cancer.	GeneRIF	0.00875	129	0.0149	0.152	1	2	MMP7
Pubmed	18403391	Haplotypes of the EPCR gene, prothrombin levels, and the risk of venous thrombosis in carriers of the prothrombin G20210A mutation.	GeneRIF	0.00875	129	0.0149	0.152	1	2	PROCR
Pubmed	21198750	Soluble CD26¿/¿dipeptidyl peptidase IV enhances human lymphocyte proliferation in vitro independent of dipeptidyl peptidase enzyme activity and adenosine deaminase binding.	GeneRIF	0.00875	129	0.0149	0.152	1	2	ADA
Pubmed	11160274	Activated p56lck directs maturation of both CD4 and CD8 single-positive thymocytes.	Pubmed	0.00875	129	0.0149	0.152	1	2	HLA-DQB1
Pubmed	20453107	Human adenosine deaminase 2 induces differentiation of monocytes into macrophages and stimulates proliferation of T helper cells and macrophages.	GeneRIF	0.00875	129	0.0149	0.152	1	2	ADA
Pubmed	6244294	Nucleotide sequence of the coding portion of human alpha globin messenger RNA.	Pubmed	0.00875	129	0.0149	0.152	1	2	HBA2
Pubmed	12675131	Interactions between p75 and TrkA receptors in differentiation and vulnerability of SN56 cholinergic cells to beta-amyloid.	GeneRIF	0.00875	129	0.0149	0.152	1	2	NGFR
Pubmed	9498238	Expression of cytochrome P450 genes encoding enzymes active in the metabolism of tamoxifen in human uterine endometrium.	Pubmed	0.00875	129	0.0149	0.152	1	2	CYP11A1
Pubmed	22262657	Natural IgM anti-leukocyte autoantibodies attenuate excess inflammation mediated by innate and adaptive immune mechanisms involving Th-17.	Pubmed	0.00875	129	0.0149	0.152	1	2	HLA-DQB1
Pubmed	22163030	Statins promote the regression of atherosclerosis via activation of the CCR7-dependent emigration pathway in macrophages.	GeneRIF	0.00875	129	0.0149	0.152	1	2	CD68
Pubmed	1511986	Two new human hemoglobin variants caused by unusual mutational events: Hb Zaïre contains a five residue repetition within the alpha-chain and Hb Duino has two residues substituted in the beta-chain.	Pubmed	0.00875	129	0.0149	0.152	1	2	HBB
Pubmed	23536555	Trop-2 promotes prostate cancer metastasis by modulating ß(1) integrin functions.	GeneRIF	0.00875	129	0.0149	0.152	1	2	TACSTD2
Pubmed	21122828	ARMS2/HTRA1 locus can confer differential susceptibility to the advanced subtypes of age-related macular degeneration.	GeneRIF	0.00875	129	0.0149	0.152	1	2	HTRA1
Pubmed	20624944	Primed antigen-specific CD4+ T cells are required for NK cell activation in vivo upon Leishmania major infection.	Pubmed	0.00875	129	0.0149	0.152	1	2	HLA-DQB1
Pubmed	19215610	Hypocholesterolemia in adult patients with thalassemia: a link with the severity of genotype in thalassemia intermedia patients.	GeneRIF	0.00875	129	0.0149	0.152	1	2	HBB
Pubmed	19002257	Plasma gelsolin depletion and circulating actin in sepsis: a pilot study.	GeneRIF	0.00875	129	0.0149	0.152	1	2	ACTA1

Category	ID	Name	Source	p-value	q-value Bonferroni	q-value FDR B&H	q-value FDR B&Y	Hit Count in Query List	Hit Count in Genome	Hit in Query List
Pubmed	22495379	Desmoglein-3 and Napsin A double stain, a useful immunohistochemical marker for differentiation of lung squamous cell carcinoma and adenocarcinoma from other subtypes.	GeneRIF	0.00875	129	0.0149	0.152	1	2	NAPSA
Pubmed	21950330	Mutation analysis of GJB3 and GJB4 in Chinese patients with erythrokeratodermia variabilis.	GeneRIF	0.00875	129	0.0149	0.152	1	2	GJB3
Pubmed	21895525	Multiple arrhythmic syndromes in a newborn, owing to a novel mutation in SCN5A.	Pubmed	0.00875	129	0.0149	0.152	1	2	SCN3B
Pubmed	24375867	Down-regulation of miR-96 by bone morphogenetic protein signaling is critical for vascular smooth muscle cell phenotype modulation.	Pubmed	0.00875	129	0.0149	0.152	1	2	BMP4
Pubmed	16720217	HLA-DQ polymorphism in Turkish patients with myasthenia gravis.	GeneRIF	0.00875	129	0.0149	0.152	1	2	HLA-DQB1
Pubmed	21529443	Insulin-like growth factor binding protein-3 interacts with the thyroid hormone receptor a1 and modulates transcription of thyroid hormone responsive gene.	GeneRIF	0.00875	129	0.0149	0.152	1	2	THRA
Pubmed	23520473	Substrate compositional variation with tissue/region and Gba1 mutations in mouse models--implications for Gaucher disease.	GeneRIF	0.00875	129	0.0149	0.152	1	2	PSAP
Pubmed	15107633	Associations among human leukocyte antigen (HLA) class II DQ variants, bacterial sexually transmitted diseases, endometritis, and fertility among women with clinical pelvic inflammatory disease.	GeneRIF	0.00875	129	0.0149	0.152	1	2	HLA-DQB1
Pubmed	18362109	Genotype-phenotype correlations for exudative age-related macular degeneration associated with homozygous HTRA1 and CFH genotypes.	GeneRIF	0.00875	129	0.0149	0.152	1	2	HTRA1
Pubmed	11901152	Clustered charged amino acids of human adenosine deaminase comprise a functional epitope for binding the adenosine deaminase complexing protein CD26/dipeptidyl peptidase IV.	GeneRIF	0.00875	129	0.0149	0.152	1	2	ADA
Pubmed	23922798	Histone demethylase retinoblastoma binding protein 2 is overexpressed in hepatocellular carcinoma and negatively regulated by hsa-miR-212.	Pubmed	0.00875	129	0.0149	0.152	1	2	KDM5A
Pubmed	9693036	Genomic structure of three long QT syndrome genes: KVLQT1, HERG, and KCNE1.	Pubmed	0.00875	129	0.0149	0.152	1	2	KCNH2
Pubmed	20646540	[Expression of macrophage migration inhibitory factor and CD74 in preeclamptic placenta and its correlation with preeclampsia].	GeneRIF	0.00875	129	0.0149	0.152	1	2	CD74
Pubmed	11692102	Requirements for allergen-induced airway inflammation and hyperreactivity in CD4-deficient and CD4-sufficient HLA-DQ transgenic mice.	Pubmed	0.00875	129	0.0149	0.152	1	2	HLA-DQB1
Pubmed	21908620	Activation of extracellular transglutaminase 2 by thioredoxin.	GeneRIF	0.00875	129	0.0149	0.152	1	2	TGM2
Pubmed	20410215	Fractalkine expression and CD16+ monocyte accumulation in glomerular lesions: association with their severity and diversity in lupus models.	GeneRIF	0.00875	129	0.0149	0.152	1	2	CX3CL1
Pubmed	16247030	Retinal ganglion cell degeneration is topological but not cell type specific in DBA/2J mice.	Pubmed	0.00875	129	0.0149	0.152	1	2	GPNMB
Pubmed	19255754	Cost-effective HLA typing with tagging SNPs predicts celiac disease risk haplotypes in the Finnish, Hungarian, and Italian populations.	GeneRIF	0.00875	129	0.0149	0.152	1	2	HLA-DQB1
Pubmed	12021150	HLA class I and class II association with ankylosing spondylitis in a southern Indian population.	GeneRIF	0.00875	129	0.0149	0.152	1	2	HLA-DQB1
Pubmed	12622778	An additional susceptibility gene for juvenile idiopathic arthritis in the HLA class I region on several DR-DQ haplotypes.	GeneRIF	0.00875	129	0.0149	0.152	1	2	HLA-DQB1
Pubmed	18849643	Application of a global proteomic approach to archival precursor lesions: deleted in malignant brain tumors 1 and tissue transglutaminase 2 are upregulated in pancreatic cancer precursors.	GeneRIF	0.00875	129	0.0149	0.152	1	2	TGM2
Pubmed	21422172	A novel isoform of the Ly108 gene ameliorates murine lupus.	GeneRIF	0.00875	129	0.0149	0.152	1	2	HLA-DQB1
Pubmed	18279642	[Study of celiac disease in the pediatric population of Cantabria (Spain) and first-degree relatives].	GeneRIF	0.00875	129	0.0149	0.152	1	2	HLA-DQB1
Pubmed	18222177	Ectonucleotide pyrophosphatase/phosphodiesterase (E-NPP) and adenosine deaminase (ADA) activities in patients with uterine cervix neoplasia.	GeneRIF	0.00875	129	0.0149	0.152	1	2	ADA
Pubmed	22330344	Gluten induces coeliac-like disease in sensitised mice involving IgA, CD71 and transglutaminase 2 interactions that are prevented by probiotics.	GeneRIF	0.00875	129	0.0149	0.152	1	2	TGM2
Pubmed	19491722	Analyses of single nucleotide polymorphisms and haplotype linkage of LOC387715 and the HTRA1 gene in exudative age-related macular degeneration in a Chinese cohort.	GeneRIF	0.00875	129	0.0149	0.152	1	2	HTRA1
Pubmed	22661093	B7-H3 participates in the development of experimental pneumococcal meningitis by augmentation of the inflammatory response via a TLR2-dependent mechanism.	GeneRIF	0.00875	129	0.0149	0.152	1	2	CD276
Pubmed	19255159	Localization of age-related macular degeneration-associated ARMS2 in cytosol, not mitochondria.	GeneRIF	0.00875	129	0.0149	0.152	1	2	HTRA1
Pubmed	22081313	Isolation of galectin-1 from human platelets: its interaction with actin.	GeneRIF	0.00875	129	0.0149	0.152	1	2	ACTA1
Pubmed	21963846	Tissue transglutaminase links TGF-ß, epithelial to mesenchymal transition and a stem cell phenotype in ovarian cancer.	GeneRIF	0.00875	129	0.0149	0.152	1	2	TGM2
Pubmed	22322858	Type 2 transglutaminase is involved in the autophagy-dependent clearance of ubiquitinated proteins.	GeneRIF	0.00875	129	0.0149	0.152	1	2	TGM2
Pubmed	21461577	Inhibition of endothelial cell migration through the down-regulation of MMP-9 by A-kinase anchoring protein 12.	GeneRIF	0.00875	129	0.0149	0.152	1	2	AKAP12
Pubmed	19885868	Inducible activation of IFI 16 results in suppression of telomerase activity, growth suppression and induction of cellular senescence.	GeneRIF	0.00875	129	0.0149	0.152	1	2	IFI16

Category	ID	Name	Source	p-value	q-value Bonferroni	q-value FDR B&H	q-value FDR B&Y	Hit Count in Query List	Hit Count in Genome	Hit in Query List
Pubmed	20332189	Activin plays a key role in the maintenance of long term memory and late-LTP.	Pubmed	0.00875	129	0.0149	0.152	1	2	INHBB
Pubmed	16601134	Inhibins differentially antagonize activin and bone morphogenetic protein action in a mouse adrenocortical cell line.	GeneRIF	0.00875	129	0.0149	0.152	1	2	INHBB
Pubmed	19453546	Parallel underexpression of kallikrein 5 and kallikrein 7 mRNA in breast malignancies.	GeneRIF	0.00875	129	0.0149	0.152	1	2	KLK7
Pubmed	11132233	Genetic factors affecting clinical severity in beta-thalassemia syndromes.	GeneRIF	0.00875	129	0.0149	0.152	1	2	HBB
Pubmed	21723825	Cofilin-linked changes in actin filament flexibility promote severing.	GeneRIF	0.00875	129	0.0149	0.152	1	2	ACTA1
Pubmed	20736794	Matrix metalloproteinases that associate with and cleave bone morphogenetic protein-2 in vitro are elevated in hypertrophic fracture nonunion tissue.	GeneRIF	0.00875	129	0.0149	0.152	1	2	MMP7
Pubmed	2844164	The collagenase gene family in humans consists of at least four members.	Pubmed	0.00875	129	0.0149	0.152	1	2	MMP7
Pubmed	17445091	Non-hematopoietic EPCR regulates the coagulation and inflammatory responses during endotoxemia.	Pubmed	0.00875	129	0.0149	0.152	1	2	PROCR
Pubmed	19342447	The osteogenic transcription factor runx2 controls genes involved in sterol/steroid metabolism, including CYP11A1 in osteoblasts.	GeneRIF	0.00875	129	0.0149	0.152	1	2	CYP11A1
Pubmed	18216162	Apical maxi-K (KCa1.1) channels mediate K+ secretion by the mouse submandibular exocrine gland.	GeneRIF	0.00875	129	0.0149	0.152	1	2	KCNN4
Pubmed	16774752	Carboxypeptidase cathepsin X mediates beta2-integrin-dependent adhesion of differentiated U-937 cells.	GeneRIF	0.00875	129	0.0149	0.152	1	2	CTSK
Pubmed	16802776	Lack of association of HLA class II alleles with peanut allergy.	GeneRIF	0.00875	129	0.0149	0.152	1	2	HLA-DQB1
Pubmed	18633189	Arterial stiffness, physical activity, and atrial natriuretic Peptide gene polymorphism in older subjects.	GeneRIF	0.00875	129	0.0149	0.152	1	2	NPPA
Pubmed	1808202	Epidermal growth factor induces rapid reorganization of the actin microfilament system in human A431 cells.	Pubmed	0.00875	129	0.0149	0.152	1	2	ACTA1
Pubmed	10329630	The interferon-inducible nucleolar p204 protein binds the ribosomal RNA-specific UBF1 transcription factor and inhibits ribosomal RNA transcription.	Pubmed	0.00875	129	0.0149	0.152	1	2	IFI16
Pubmed	11969288	Dendrite-like process formation and cytoskeletal remodeling regulated by delta-catenin expression.	Pubmed	0.00875	129	0.0149	0.152	1	2	ACTA1
Pubmed	9369230	Disturbed progastrin processing in carboxypeptidase E-deficient fat mice.	Pubmed	0.00875	129	0.0149	0.152	1	2	CPE
Pubmed	11409167	Assessment of protein gene product 9.5 as a marker of neural crest-derived precursor cells in the developing enteric nervous system.	Pubmed	0.00875	129	0.0149	0.152	1	2	NGFR
Pubmed	12759241	Matrilysin (matrix metalloproteinase-7) mediates E-cadherin ectodomain shedding in injured lung epithelium.	GeneRIF	0.00875	129	0.0149	0.152	1	2	MMP7
Pubmed	15309343	No allelic variation in genes with high gliadin homology in patients with celiac disease and type 1 diabetes.	GeneRIF	0.00875	129	0.0149	0.152	1	2	HLA-DQB1
Pubmed	21807613	Muscle sparing in muscle RING finger 1 null mice: response to synthetic glucocorticoids.	GeneRIF	0.00875	129	0.0149	0.152	1	2	FBXO32
Pubmed	21130993	Endothelial lipase (EL) and EL-generated lysophosphatidylcholines promote IL-8 expression in endothelial cells.	GeneRIF	0.00875	129	0.0149	0.152	1	2	LIPG
Pubmed	15013978	Specific HLA-DRB and -DQB alleles and haplotypes confer disease susceptibility or resistance in Bahraini type 1 diabetes patients.	GeneRIF	0.00875	129	0.0149	0.152	1	2	HLA-DQB1
Pubmed	8622706	A C3H strain-specific allele (alpha va126) of the murine alpha-globin gene newly detected by UT-PAGE and RT-PCR-SSCP analysis.	Pubmed	0.00875	129	0.0149	0.152	1	2	HBA2
Pubmed	1652217	Natriuretic peptides inhibit rat astroglial proliferation: mediation by C receptor.	Pubmed	0.00875	129	0.0149	0.152	1	2	NPPA
Pubmed	11371512	A mutation in the saposin A domain of the sphingolipid activator protein (prosaposin) gene results in a late-onset, chronic form of globoid cell leukodystrophy in the mouse.	Pubmed	0.00875	129	0.0149	0.152	1	2	PSAP
Pubmed	18523267	Blunted IgE-mediated activation of mast cells in mice lacking the Ca2+-activated K+ channel KCa3.1.	GeneRIF	0.00875	129	0.0149	0.152	1	2	KCNN4
Pubmed	19700761	Bone marrow-derived cathepsin K cleaves SPARC in bone metastasis.	GeneRIF	0.00875	129	0.0149	0.152	1	2	CTSK
Pubmed	17584585	Immunogenetics of HLA class II in Israeli patients with adult-onset type 1 diabetes mellitus.	GeneRIF	0.00875	129	0.0149	0.152	1	2	HLA-DQB1
Pubmed	12192002	Biophysical characterization of the alpha-globin binding protein alpha-hemoglobin stabilizing protein.	GeneRIF	0.00875	129	0.0149	0.152	1	2	HBA2
Pubmed	11075809	Thyroid hormone receptor beta-deficient mice show complete loss of the normal cholesterol 7alpha-hydroxylase (CYP7A) response to thyroid hormone but display enhanced resistance to dietary cholesterol.	Pubmed	0.00875	129	0.0149	0.152	1	2	THRA
Pubmed	14749532	Dysferlin in a hyperCKaemic patient with caveolin 3 mutation and in C2C12 cells after p38 MAP kinase inhibition.	GeneRIF	0.00875	129	0.0149	0.152	1	2	DYSF
Pubmed	19131967	Sall2 is a novel p75NTR-interacting protein that links NGF signalling to cell cycle progression and neurite outgrowth.	GeneRIF	0.00875	129	0.0149	0.152	1	2	NGFR
Pubmed	23936109	Fractalkine depresses cardiomyocyte contractility.	Pubmed	0.00875	129	0.0149	0.152	1	2	CX3CL1
Pubmed	23257389	Novel SCN3B mutation associated with brugada syndrome affects intracellular trafficking and function of Nav1.5.	GeneRIF	0.00875	129	0.0149	0.152	1	2	SCN3B
Pubmed	19346456	Endogenous cardiac natriuretic peptides protect the heart in a mouse model of dilated cardiomyopathy and sudden death.	GeneRIF	0.00875	129	0.0149	0.152	1	2	NPPA
Pubmed	12560236	Regulation of the human endothelial cell protein C receptor gene promoter by multiple Sp1 binding sites.	GeneRIF	0.00875	129	0.0149	0.152	1	2	PROCR
Pubmed	22283518	A puzzle over several decades: eye anomalies with FRAS1 and STRA6 mutations in the same family.	Pubmed	0.00875	129	0.0149	0.152	1	2	STRA6
Pubmed	1177322	Three-dimensional fourier synthesis of human deoxyhaemoglobin at 2-5 A resolution: refinement of the atomic model.	Pubmed	0.00875	129	0.0149	0.152	1	2	HBB

Category	ID	Name	Source	p-value	q-value Bonferroni	q-value FDR B&H	q-value FDR B&Y	Hit Count in Query List	Hit Count in Genome	Hit in Query List
Pubmed	22607836	MASP interactions with plasma-derived MBL.	GeneRIF	0.00875	129	0.0149	0.152	1	2	MASP1
Pubmed	20813660	[Protein kinase D3 is involved in negative regulation of MMP-7 in prostate cancer cells].	GeneRIF	0.00875	129	0.0149	0.152	1	2	MMP7
Pubmed	20614846	Anti-Tribbles homolog 2 (TRIB2) autoantibodies in narcolepsy are associated with recent onset of cataplexy.	GeneRIF	0.00875	129	0.0149	0.152	1	2	HLA-DQB1
Pubmed	3793760	Tropomyosin distinguishes between the two actin-binding sites of villin and affects actin-binding properties of other brush border proteins.	Pubmed	0.00875	129	0.0149	0.152	1	2	ACTA1
Pubmed	8586438	A novel human phosphoglucomutase (PGM5) maps to the centromeric region of chromosome 9.	Pubmed	0.00875	129	0.0149	0.152	1	2	PGM5
Pubmed	21934092	MicroRNA-499-5p promotes cellular invasion and tumor metastasis in colorectal cancer by targeting FOXO4 and PDCD4.	GeneRIF	0.00875	129	0.0149	0.152	1	2	FOXO4
Pubmed	10400985	Functional characterization of the Opitz syndrome gene product (midin): evidence for homodimerization and association with microtubules throughout the cell cycle.	Pubmed	0.00875	129	0.0149	0.152	1	2	MID1
Pubmed	22709495	Fractalkine stimulates cell growth and increases its expression via NF-¿B pathway in RA-FLS.	GeneRIF	0.00875	129	0.0149	0.152	1	2	CX3CL1
Pubmed	23471969	An intracellular domain fragment of the p75 neurotrophin receptor (p75(NTR)) enhances tropomyosin receptor kinase A (TrkA) receptor function.	GeneRIF	0.00875	129	0.0149	0.152	1	2	NGFR
Pubmed	22573678	Transglutaminase inhibition protects against oxidative stress-induced neuronal death downstream of pathological ERK activation.	GeneRIF	0.00875	129	0.0149	0.152	1	2	TGM2
Pubmed	17009258	Tetraspanin CD151 is expressed in osteoarthritic cartilage and is involved in pericellular activation of pro-matrix metalloproteinase 7 in osteoarthritic chondrocytes.	GeneRIF	0.00875	129	0.0149	0.152	1	2	MMP7
Pubmed	9140816	Altered expression of atrial natriuretic peptide and contractile protein genes in hypertrophied ventricle of JVS mice with systemic carnitine deficiency.	Pubmed	0.00875	129	0.0149	0.152	1	2	NPPA
Pubmed	22291595	The intracellular DNA sensor IFI16 gene acts as restriction factor for human cytomegalovirus replication.	GeneRIF	0.00875	129	0.0149	0.152	1	2	IFI16
Pubmed	8626785	Identification and characterization of a versatile retinoid response element (retinoic acid receptor response element-retinoid X receptor response element) in the mouse tissue transglutaminase gene promoter.	Pubmed	0.00875	129	0.0149	0.152	1	2	TGM2
Pubmed	10679223	Human 3'-phosphoadenosine 5'-phosphosulfate synthetase 1 (PAPSS1) and PAPSS2: gene cloning, characterization and chromosomal localization.	Pubmed	0.00875	129	0.0149	0.152	1	2	PAPSS2
Pubmed	12721173	Associations between patients with endometriosis and HLA class II; the analysis of HLA-DQB1 and HLA-DPB1 genotypes.	GeneRIF	0.00875	129	0.0149	0.152	1	2	HLA-DQB1
Pubmed	10217480	Alpha-crystallin as a molecular chaperone.	Pubmed	0.00875	129	0.0149	0.152	1	2	CRYAA
Pubmed	19491393	Blockade of interleukin-6 signaling augments regulatory T-cell reconstitution and attenuates the severity of graft-versus-host disease.	Pubmed	0.00875	129	0.0149	0.152	1	2	HLA-DQB1
Pubmed	14643893	The major splice variant of human 5-aminolevulinate synthase-2 contributes significantly to erythroid heme biosynthesis.	GeneRIF	0.00875	129	0.0149	0.152	1	2	ALAS2
Pubmed	16108039	Tissue transglutaminase (TG2) acting as G protein protects hepatocytes against Fas-mediated cell death in mice.	GeneRIF	0.00875	129	0.0149	0.152	1	2	TGM2
Pubmed	21255765	Immunohistochemical expression of matrix metalloproteinases in squamous cell carcinoma of the tongue and lower lip.	GeneRIF	0.00875	129	0.0149	0.152	1	2	MMP7
Pubmed	18273032	Cord blood islet autoantibodies and seasonal association with the type 1 diabetes high-risk genotype.	GeneRIF	0.00875	129	0.0149	0.152	1	2	HLA-DQB1
Pubmed	20226272	Utility of the recovery electrocardiogram after exercise: a novel indicator for the diagnosis and genotyping of long QT syndrome?	GeneRIF	0.00875	129	0.0149	0.152	1	2	KCNH2
Pubmed	15795176	The influences of p75 neurotrophin receptor and brain-derived neurotrophic factor in the sympathetic innervation of target tissues during murine postnatal development.	Pubmed	0.00875	129	0.0149	0.152	1	2	NGFR
Pubmed	8603590	The sites of gene expression of atrial, brain, and C-type natriuretic peptides in mouse fetal development: temporal changes in embryos and placenta.	Pubmed	0.00875	129	0.0149	0.152	1	2	NPPA
Pubmed	23567476	Distinct behavioral phenotypes in male mice lacking the thyroid hormone receptor a1 or ß isoforms.	Pubmed	0.00875	129	0.0149	0.152	1	2	THRA
Pubmed	19021035	BMP-2 vs. BMP-4 expression and activity in glucocorticoid-arrested MC3T3-E1 osteoblasts: Smad signaling, not alkaline phosphatase activity, predicts rescue of mineralization.	GeneRIF	0.00875	129	0.0149	0.152	1	2	BMP4
Pubmed	18231640	A pathologic link between Wilms tumor suppressor gene, WT1, and IFI16.	GeneRIF	0.00875	129	0.0149	0.152	1	2	IFI16
Pubmed	19617323	The importance of acetyl coenzyme A synthetase for 11C-acetate uptake and cell survival in hepatocellular carcinoma.	GeneRIF	0.00875	129	0.0149	0.152	1	2	ACSS1
Pubmed	18958543	Polymorphisms of matrix metalloproteinase-7 and chymase are associated with susceptibility to and progression of gastric cancer in Japan.	GeneRIF	0.00875	129	0.0149	0.152	1	2	MMP7
Pubmed	12875970	Identification of genes up-regulated in urothelial tumors: the 67-kd laminin receptor and tumor-associated trypsin inhibitor.	GeneRIF	0.00875	129	0.0149	0.152	1	2	SPINK1
Pubmed	21880134	Constitutive expression of cathepsin K in the human intervertebral disc: new insight into disc extracellular matrix remodeling via cathepsin K and receptor activator of nuclear factor-¿B ligand.	GeneRIF	0.00875	129	0.0149	0.152	1	2	CTSK
Pubmed	1634363	A new alpha chain variant, Hb Hanamaki or alpha 2(139)(HC1)Lys----Glu beta 2, found in a Japanese family.	Pubmed	0.00875	129	0.0149	0.152	1	2	HBA2
Pubmed	19369399	Sulfation of chondroitin sulfate proteoglycans is necessary for proper Indian hedgehog signaling in the developing growth plate.	GeneRIF	0.00875	129	0.0149	0.152	1	2	PAPSS2

Category	ID	Name	Source	p-value	q-value Bonferroni	q-value FDR B&H	q-value FDR B&Y	Hit Count in Query List	Hit Count in Genome	Hit in Query List
Pubmed	19635103	Expression of the interferon-inducible proteins MxA and IFI16 in liver allografts.	GeneRIF	0.00875	129	0.0149	0.152	1	2	IFI16
Pubmed	22024063	Immunohistochemical study of vascular endothelial growth factor (VEGF) and matrilysin (MMP-7) in T1 adenocarcinoma of the colon and rectum to predict lymph node metastases or distant metastases.	GeneRIF	0.00875	129	0.0149	0.152	1	2	MMP7
Pubmed	14990579	Requirement of IFI16 for the maximal activation o p53 induced by ionizing radiation.	GeneRIF	0.00875	129	0.0149	0.152	1	2	IFI16
Pubmed	18509540	Effective detection of human leukocyte antigen ris alleles in celiac disease using tag single nucleotide polymorphisms.	GeneRIF	0.00875	129	0.0149	0.152	1	2	HLA-DQB1
Pubmed	20884616	Nucleoside diphosphate kinase B knock-out mice have impaired activation of the K+ channel KCa3.1, resulting in defective T cell activation.	GeneRIF	0.00875	129	0.0149	0.152	1	2	KCNN4
Pubmed	25205867	Overexpression of HtrA1 and exposure to mainstream cigarette smoke leads to choroidal neovascularization and subretinal deposits in aged mice.	Pubmed	0.00875	129	0.0149	0.152	1	2	HTRA1
Pubmed	12808095	Transcriptional program of apoptosis induction following interleukin 2 deprivation: identification of RC3, a calcium/calmodulin binding protein, as a novel proapoptotic factor.	GeneRIF	0.00875	129	0.0149	0.152	1	2	NRGN
Pubmed	7908320	Prevention of nephritis in major histocompatibility complex class II-deficient MRL-lpr mice.	Pubmed	0.00875	129	0.0149	0.152	1	2	HLA-DQB1
Pubmed	18447576	MMP1 and MMP7 as potential peripheral blood biomarkers in idiopathic pulmonary fibrosis.	GeneRIF	0.00875	129	0.0149	0.152	1	2	MMP7
Pubmed	16508016	A nucleolin-binding 3' untranslated region element stabilizes beta-globin mRNA in vivo.	GeneRIF	0.00875	129	0.0149	0.152	1	2	HBB
Pubmed	22387881	Phosphatidylethanolamine N-methyltransferase and choline dehydrogenase gene polymorphisms are associated with human sperm concentration.	GeneRIF	0.00875	129	0.0149	0.152	1	2	CHDH
Pubmed	8018603	Molecular characterization of a novel serine protease involved in activation of the complement system by mannose-binding protein.	Pubmed	0.00875	129	0.0149	0.152	1	2	MASP1
Pubmed	22242126	Ablation of Mrds1/Ofcc1 induces hyper-¿-glutamyl transpeptidasemia without abnormal head development and schizophrenia-relevant behaviors in mice.	GeneRIF	0.00875	129	0.0149	0.152	1	2	GGT1
Pubmed	21308979	Polymorphisms in CHDH gene and the risk of tooth agenesis.	GeneRIF	0.00875	129	0.0149	0.152	1	2	CHDH
Pubmed	1517709	Distribution of the nuclear thyroid-hormone receptor in extraocular and skeletal muscles.	Pubmed	0.00875	129	0.0149	0.152	1	2	THRA
Pubmed	21855631	Activin, BMP and FGF pathways cooperate to promote endoderm and pancreatic lineage cell differentiation from human embryonic stem cells.	GeneRIF	0.00875	129	0.0149	0.152	1	2	BMP4
Pubmed	22521184	Association of HLA-DR/DQ polymorphism with myasthenia gravis in Tunisian patients.	GeneRIF	0.00875	129	0.0149	0.152	1	2	HLA-DQB1
Pubmed	19366662	Insulin receptor tyrosine kinase substrate links the E. coli O157:H7 actin assembly effectors Tir and EspF(U) during pedestal formation.	GeneRIF	0.00875	129	0.0149	0.152	1	2	BAIAP2L1
Pubmed	9328125	Hypogammaglobulinaemia occurs in Fas-deficient MRL-lpr mice following deletion of MHC class II molecules.	Pubmed	0.00875	129	0.0149	0.152	1	2	HLA-DQB1
Pubmed	22641052	siRNA knockdown of ADAM-10, but not ADAM-17, significantly reduces fractalkine shedding following pro-inflammatory cytokine treatment in a human adult brain endothelial cell line.	GeneRIF	0.00875	129	0.0149	0.152	1	2	CX3CL1
Pubmed	17429723	Carrageenan reduces bone morphogenetic protein-4 (BMP4) and activates the Wnt/beta-catenin pathway in normal human colonocytes.	GeneRIF	0.00875	129	0.0149	0.152	1	2	BMP4
Pubmed	22155582	Composition and proteolytic processing of corneal deposits associated with mutations in the TGFBI gene.	GeneRIF	0.00875	129	0.0149	0.152	1	2	HTRA1
Pubmed	18587492	Crystallin gene mutations in Indian families with inherited pediatric cataract.	Pubmed	0.00875	129	0.0149	0.152	1	2	CRYAA
Pubmed	1893870	alpha-Actin proteins and gene transcripts are colocalized in embryonic mouse muscle.	Pubmed	0.00875	129	0.0149	0.152	1	2	ACTA1
Pubmed	23825125	Carboxypeptidase E protects hippocampal neurons during stress in male mice by up-regulating prosurvival BCL2 protein expression.	Pubmed	0.00875	129	0.0149	0.152	1	2	CPE
Pubmed	23584220	Immunohistohemical expression of the chemokine fractalkine and its receptor in the human brain cortex after severe traumatic brain injury and brain hemorrhage.	GeneRIF	0.00875	129	0.0149	0.152	1	2	CX3CL1
Pubmed	18603555	Significance of borderline hemoglobin A2 values in an Italian population with a high prevalence of beta-thalassemia.	GeneRIF	0.00875	129	0.0149	0.152	1	2	HBB
Pubmed	19052260	Inhibition of the KCa3.1 channels by AMP-activated protein kinase in human airway epithelial cells.	GeneRIF	0.00875	129	0.0149	0.152	1	2	KCNN4
Pubmed	20501942	Cdc42 regulates bone modeling and remodeling in mice by modulating RANKL/M-CSF signaling and osteoclast polarization.	Pubmed	0.00875	129	0.0149	0.152	1	2	CTSK
Pubmed	22590725	Foxo/atrogin induction in human and experimental myositis.	GeneRIF	0.00875	129	0.0149	0.152	1	2	FBXO32
Pubmed	18602709	p75(NTR) activation of NF-kappaB is involved in PrP106-126-induced apoptosis in mouse neuroblastoma cells.	GeneRIF	0.00875	129	0.0149	0.152	1	2	NGFR
Pubmed	22199361	Death-associated protein 6 (Daxx) mediates cAMP dependent stimulation of Cyp11a1 (P450scc) transcription.	GeneRIF	0.00875	129	0.0149	0.152	1	2	CYP11A1
Pubmed	21600847	Comparison of atrial and brain natriuretic peptide for the assessment of mitral stenosis.	GeneRIF	0.00875	129	0.0149	0.152	1	2	NPPA
Pubmed	20922190	Effects of pro-inflammatory cytokines on the production of soluble fractalkine and ADAM17 by HepG2 cells.	GeneRIF	0.00875	129	0.0149	0.152	1	2	CX3CL1
Pubmed	18243818	Dysregulation of pulsatility in aging IV. Pulsatile signaling and cardiovascular aging: functions and regulation of natriuretic peptide signaling.	GeneRIF	0.00875	129	0.0149	0.152	1	2	NPPA

Category	ID	Name	Source	p-value	q-value Bonferroni	q-value FDR B&H	q-value FDR B&Y	Hit Count in Query List	Hit Count in Genome	Hit in Query List
Pubmed	14516315	Role of PTEN and MMP-7 expression in growth, invasion, metastasis and angiogenesis of gastric carcinoma.	GeneRIF	0.00875	129	0.0149	0.152	1	2	MMP7
Pubmed	11113349	Expression of peptides and other neurochemical markers in hypothalamus and olfactory bulb of mice devoid of all known thyroid hormone receptors.	Pubmed	0.00875	129	0.0149	0.152	1	2	THRA
Pubmed	15958417	Characterization of the LARGE family of putative glycosyltransferases associated with dystroglycanopathies.	Pubmed	0.00875	129	0.0149	0.152	1	2	LARGE
Pubmed	15009387	HLA-DQA1 and DQB1 alleles are associated with genetic susceptibility to psoriasis vulgaris in Chinese Han.	GeneRIF	0.00875	129	0.0149	0.152	1	2	HLA-DQB1
Pubmed	12793199	The role of HLA-DRB1*04 alleles and their association with HLA-DQB genes in genetic susceptibility to rheumatoid arthritis in Hungarian patients.	GeneRIF	0.00875	129	0.0149	0.152	1	2	HLA-DQB1
Pubmed	11306819	Assignment of TACSTD1 (alias TROP1, M4S1) to human chromosome 2p21 and refinement of mapping of TACSTD2 (alias TROP2, M1S1) to human chromosome 1p32 by in situ hybridization.	Pubmed	0.00875	129	0.0149	0.152	1	2	TACSTD2
Pubmed	23809616	Human leucocyte antigen alleles and haplotypes and their associations with antinuclear antibodies features in Chinese patients with primary biliary cirrhosis.	Pubmed	0.00875	129	0.0149	0.152	1	2	HLA-DQB1
Pubmed	20861306	Heparan sulfate acts as a bone morphogenetic protein coreceptor by facilitating ligand-induced receptor hetero-oligomerization.	Pubmed	0.00875	129	0.0149	0.152	1	2	BMP4
Pubmed	16224053	Expression of fractalkine (CX3CL1) and its receptor, CX3CR1, is elevated in coronary artery disease and is reduced during statin therapy.	GeneRIF	0.00875	129	0.0149	0.152	1	2	CX3CL1
Pubmed	3795040	Interaction between human serum albumin and alpha 1-acid glycoprotein in the binding of lidocaine to purified protein fractions and sera.	Pubmed	0.00875	129	0.0149	0.152	1	2	ORM1
Pubmed	18981094	Adaptable TCR avidity thresholds for negative selection.	Pubmed	0.00875	129	0.0149	0.152	1	2	HLA-DQB1
Pubmed	20683935	CX3CL1-CX3CR1 interaction prevents carbon tetrachloride-induced liver inflammation and fibrosis in mice.	GeneRIF	0.00875	129	0.0149	0.152	1	2	CX3CL1
Pubmed	2536962	Localization of human phosphoribosylpyrophosphate synthetase subunit I and II genes (PRPS1 and PRPS2) to different regions of the X chromosome and assignment of two PRPS1-related genes to autosomes.	Pubmed	0.00875	129	0.0149	0.152	1	2	PRPS2
Pubmed	9199170	IQGAP1, a Rac- and Cdc42-binding protein, directly binds and cross-links microfilaments.	Pubmed	0.00875	129	0.0149	0.152	1	2	ACTA1
Pubmed	18495154	Solution structure of the inner DysF domain of myoferlin and implications for limb girdle muscular dystrophy type 2b.	GeneRIF	0.00875	129	0.0149	0.152	1	2	DYSF
Pubmed	21167183	Myocardial dysfunction in an animal model of cancer cachexia.	GeneRIF	0.00875	129	0.0149	0.152	1	2	FBXO32
Pubmed	15128847	CD4-positive T cell-mediated neuroprotection requires dual compartment antigen presentation.	Pubmed	0.00875	129	0.0149	0.152	1	2	HLA-DQB1
Pubmed	18352820	The role of major histocompatibility complex alleles in the susceptibility of Brazilian patients to develop the myogenic type of Graves' orbitopathy.	GeneRIF	0.00875	129	0.0149	0.152	1	2	HLA-DQB1
Pubmed	14638693	Evidence for a critical role of the tumor necrosis factor alpha convertase (TACE) in ectodomain shedding of the p75 neurotrophin receptor (p75NTR).	GeneRIF	0.00875	129	0.0149	0.152	1	2	NGFR
Pubmed	11827978	Assembly of human hemoglobin (Hb) beta- and gamma-globin chains expressed in a cell-free system with alpha-globin chains to form Hb A and Hb F.	GeneRIF	0.00875	129	0.0149	0.152	1	2	HBB
Pubmed	19129726	Heritability of plasma concentrations of activated protein C in a Spanish population.	GeneRIF	0.00875	129	0.0149	0.152	1	2	PROCR
Pubmed	22425255	A genome-wide search for genetic influences and biological pathways related to the brain's white matter integrity.	Pubmed	0.00875	129	0.0149	0.152	1	2	TRABD2B
Pubmed	16407206	Chronic inhibition of cardiac Kir2.1 and HERG potassium channels by celastrol with dual effects on both ion conductivity and protein trafficking.	GeneRIF	0.00875	129	0.0149	0.152	1	2	KCNH2
Pubmed	10848522	Effects of thyroid hormone receptor gene disruption on myosin isoform expression in mouse skeletal muscles.	Pubmed	0.00875	129	0.0149	0.152	1	2	THRA
Pubmed	9811821	Impaired osteoclastic bone resorption leads to osteopetrosis in cathepsin-K-deficient mice.	Pubmed	0.00875	129	0.0149	0.152	1	2	CTSK
Pubmed	15752776	LARGE2 facilitates the maturation of alpha-dystroglycan more effectively than LARGE.	GeneRIF	0.00875	129	0.0149	0.152	1	2	LARGE
Pubmed	22987923	Salt-sensitive hypertension and cardiac hypertrophy in transgenic mice expressing a corin variant identified in blacks.	GeneRIF	0.00875	129	0.0149	0.152	1	2	NPPA
Pubmed	24052076	A novel extracellular role for tissue transglutaminase in matrix-bound VEGF-mediated angiogenesis.	Pubmed	0.00875	129	0.0149	0.152	1	2	TGM2
Pubmed	21792010	Expression of macrophage migration inhibitory factor and CD74 in cervical squamous cell carcinoma.	GeneRIF	0.00875	129	0.0149	0.152	1	2	CD74
Pubmed	22950951	Microarray analysis of mRNA from cumulus cells following in vivo or in vitro maturation of mouse cumulus-oocyte complexes.	GeneRIF	0.00875	129	0.0149	0.152	1	2	HBA2
Pubmed	20062085	Thyroid hormone receptors are tumor suppressors in a mouse model of metastatic follicular thyroid carcinoma.	GeneRIF	0.00875	129	0.0149	0.152	1	2	THRA
Pubmed	15507486	Differential actin binding along the PEVK domain of skeletal muscle titin.	GeneRIF	0.00875	129	0.0149	0.152	1	2	ACTA1
Pubmed	21912905	Increase of a-SMA(+) and CK (+) cells as an early sign of epithelial-mesenchymal transition during colorectal carcinogenesis.	GeneRIF	0.00875	129	0.0149	0.152	1	2	ACTA1
Pubmed	11740498	Homeostasis and anergy of CD4(+)CD25(+) suppressor T cells in vivo.	Pubmed	0.00875	129	0.0149	0.152	1	2	HLA-DQB1
Pubmed	19709758	LTP impairment by fractalkine/CX3CL1 in mouse hippocampus is mediated through the activity of adenosine receptor type 3 (A3R).	GeneRIF	0.00875	129	0.0149	0.152	1	2	CX3CL1

Category	ID	Name	Source	p-value	q-value Bonferroni	q-value FDR B&H	q-value FDR B&Y	Hit Count in Query List	Hit Count in Genome	Hit in Query List
Pubmed	20551465	Idiopathic chronic pancreatitis in India: phenotypic characterisation and strong genetic susceptibility due to SPINK1 and CFTR gene mutations.	GeneRIF	0.00875	129	0.0149	0.152	1	2	SPINK1
Pubmed	20697954	Dyslexia-associated kiaa0319-like protein interacts with axon guidance receptor nogo receptor 1.	GeneRIF	0.00875	129	0.0149	0.152	1	2	RTN4R
Pubmed	8159766	A family of potassium channel genes related to eag in Drosophila and mammals.	Pubmed	0.00875	129	0.0149	0.152	1	2	KCNH2
Pubmed	12362498	[Frequency of DRB1* and DQB1* alleles in Polish patients with dilated cardiomyopathy].	GeneRIF	0.00875	129	0.0149	0.152	1	2	HLA-DQB1
Pubmed	12656131	A population- and family-based study of Canadian families reveals association of HLA DRB1*0103 with colonic involvement in inflammatory bowel disease.	GeneRIF	0.00875	129	0.0149	0.152	1	2	HLA-DQB1
Pubmed	11472060	Fractalkine shares signal sequence with TARC: gene structures and expression profiles of two chemokine genes.	Pubmed	0.00875	129	0.0149	0.152	1	2	CX3CL1
Pubmed	18680557	Human adenosine deaminase as an allosteric modulator of human A(1) adenosine receptor: abolishment of negative cooperativity for [H](R)-pia binding to the caudate nucleus.	GeneRIF	0.00875	129	0.0149	0.152	1	2	ADA
Pubmed	10920242	A linkage map of distal mouse chromosome 4 in the vicinity of Ssm1, a strain-specific modifier of methylation.	Pubmed	0.00875	129	0.0149	0.152	1	2	NPPA
Pubmed	9239401	Interferon-gamma deficiency prevents coronary arteriosclerosis but not myocardial rejection in transplanted mouse hearts.	Pubmed	0.00875	129	0.0149	0.152	1	2	HLA-DQB1
Pubmed	14586015	Sonic hedgehog and bone morphogenetic protein regulate interneuron development from dorsal telencephalic progenitors in vitro.	GeneRIF	0.00875	129	0.0149	0.152	1	2	BMP4
Pubmed	18329042	Crystal structure of human epidermal kallikrein 7 (hK7) synthesized directly in its native state in E. coli: insights into the atomic basis of its inhibition by LEKTI domain 6 (LD6).	GeneRIF	0.00875	129	0.0149	0.152	1	2	KLK7
Pubmed	20837372	Serum cathepsin levels in coronary artery ectasia.	GeneRIF	0.00875	129	0.0149	0.152	1	2	CTSK
Pubmed	17093291	Suppression of endothelial or lipoprotein lipase in THP-1 macrophages attenuates proinflammatory cytokine secretion.	GeneRIF	0.00875	129	0.0149	0.152	1	2	LIPG
Pubmed	23783374	Expression of cilium-associated genes defines novel molecular subtypes of idiopathic pulmonary fibrosis.	Pubmed	0.00875	129	0.0149	0.152	1	2	MMP7
Pubmed	21967801	Transglutaminase 2 facilitates the distant hematogenous metastasis of breast cancer by modulating interleukin-6 in cancer cells.	GeneRIF	0.00875	129	0.0149	0.152	1	2	TGM2
Pubmed	22554505	The hERG channel is dependent upon the Hsp90a isoform for maturation and trafficking.	GeneRIF	0.00875	129	0.0149	0.152	1	2	KCNH2
Pubmed	22954591	Neuroendocrine control of female reproductive function by the activin receptor ALK7.	GeneRIF	0.00875	129	0.0149	0.152	1	2	INHBB
Pubmed	12445316	HLA-DRB1 and DQB1 allele distribution in the Muong population exposed to malaria in Vietnam.	GeneRIF	0.00875	129	0.0149	0.152	1	2	HLA-DQB1
Pubmed	10577503	Macrophage scavenger receptors and foam cell formation.	Pubmed	0.00875	129	0.0149	0.152	1	2	CD68
Pubmed	23420843	A periodic pattern of evolutionarily conserved basic and acidic residues constitutes the binding interface of actin-tropomyosin.	GeneRIF	0.00875	129	0.0149	0.152	1	2	ACTA1
Pubmed	16142923	Recognition sequence 2 (residues 60-71) plays a role in oligomerization and exchange dynamics of alphaB-crystallin.	GeneRIF	0.00875	129	0.0149	0.152	1	2	CRYAA
Pubmed	12857425	Inhibin A, inhibin B and activin A concentrations in umbilical cord artery and vein.	GeneRIF	0.00875	129	0.0149	0.152	1	2	INHBB
Pubmed	15556690	Hepatitis C virus-related hepatocellular carcinoma and B-cell lymphoma patients show a different profile of major histocompatibility complex class I alleles.	GeneRIF	0.00875	129	0.0149	0.152	1	2	HLA-DQB1
Pubmed	20823837	HLA-G 14 bp deletion/insertion polymorphism in celiac disease.	GeneRIF	0.00875	129	0.0149	0.152	1	2	HLA-DQB1
Pubmed	11278661	Androgen receptor specifically interacts with a novel p21-activated kinase, PAK6.	Pubmed	0.00875	129	0.0149	0.152	1	2	PAK6
Pubmed	10971749	Demyelination and axonal dystrophy in alpha A-crystallin transgenic mice.	Pubmed	0.00875	129	0.0149	0.152	1	2	CRYAA
Pubmed	21987822	Like-acetylglucosaminyltransferase (LARGE)-dependent modification of dystroglycan at Thr-317/319 is required for laminin binding and arenavirus infection.	GeneRIF	0.00875	129	0.0149	0.152	1	2	LARGE
Pubmed	21207956	Hypoxic induction of human erythroid-specific d-aminolevulinate synthase mediated by hypoxia-inducible factor 1.	GeneRIF	0.00875	129	0.0149	0.152	1	2	ALAS2
Pubmed	15259342	[Association of the HLA-DQB*0501, allele of the major histocompatibility complex with gastric cancer in Mexico].	GeneRIF	0.00875	129	0.0149	0.152	1	2	HLA-DQB1
Pubmed	17143607	An association analysis of the HLA gene region in latent autoimmune diabetes in adults.	GeneRIF	0.00875	129	0.0149	0.152	1	2	HLA-DQB1
Pubmed	17531524	Nerve growth factor and tissue repair remodeling: trkA(NGFR) and p75(NTR), two receptors one fate.	GeneRIF	0.00875	129	0.0149	0.152	1	2	NGFR
Pubmed	15953084	Corneodesmosin (CDSN) gene association with psoriasis vulgaris in Caucasian but not in Japanese populations.	GeneRIF	0.00875	129	0.0149	0.152	1	2	CDSN
Pubmed	21865054	Age- and gender-specific epistasis between ADA and TNF-a influences human life-expectancy.	GeneRIF	0.00875	129	0.0149	0.152	1	2	ADA
Pubmed	20833718	Distinct MHC class II molecules are associated on the dendritic cell surface in cholesterol-dependent membrane microdomains.	GeneRIF	0.00875	129	0.0149	0.152	1	2	HLA-DQB1
Pubmed	25071021	HERG1 functions as an oncogene in pancreatic cancer and is downregulated by miR-96.	Pubmed	0.00875	129	0.0149	0.152	1	2	KCNH2
Pubmed	20926141	The functional significance of the TGM2 gene in schizophrenia: a correlation of SNPs and circulating IL-2 levels.	GeneRIF	0.00875	129	0.0149	0.152	1	2	TGM2
Pubmed	10869562	Identification of a novel secretory leukocyte protease inhibitor-binding protein involved in membrane phospholipid movement.	Pubmed	0.00875	129	0.0149	0.152	1	2	SLPI
Pubmed	15322286	Interactions and chaperone function of alphaA-crystallin with T5P gammaC-crystallin mutant.	GeneRIF	0.00875	129	0.0149	0.152	1	2	CRYAA

Category	ID	Name	Source	p-value	q-value Bonferroni	q-value FDR B&H	q-value FDR B&Y	Hit Count in Query List	Hit Count in Genome	Hit in Query List
Pubmed	9315663	Tissue transglutaminase-dependent posttranslational modification of the retinoblastoma gene product in promonocytic cells undergoing apoptosis.	Pubmed	0.00875	129	0.0149	0.152	1	2	TGM2
Pubmed	8483910	Mice lacking major histocompatibility complex class I and class II molecules.	Pubmed	0.00875	129	0.0149	0.152	1	2	HLA-DQB1
Pubmed	20198348	Porcupine expression is associated with the expression of S100P and other cancer-related molecules in non-small cell lung carcinoma.	GeneRIF	0.00875	129	0.0149	0.152	1	2	PORCN
Pubmed	21543057	Invariant chain processing is independent of cathepsin variation between primary human B cells/dendritic cells and B-lymphoblastoid cells.	GeneRIF	0.00875	129	0.0149	0.152	1	2	CD74
Pubmed	9271631	HLA class II haplotype and sequence analysis support a role for DQ in narcolepsy.	Pubmed	0.00875	129	0.0149	0.152	1	2	HLA-DQB1
Pubmed	15878242	Co-expression of the P75 neurotrophin receptor and neurotrophin receptor-interacting melanoma antigen homolog in the mature rat brain.	Pubmed	0.00875	129	0.0149	0.152	1	2	NGFR
Pubmed	21471287	Differential roles for the interferon-inducible IFI16 and AIM2 innate immune sensors for cytosolic DNA in cellular senescence of human fibroblasts.	GeneRIF	0.00875	129	0.0149	0.152	1	2	IFI16
Pubmed	25902523	The prodomain of BMP4 is necessary and sufficient to generate stable BMP4/7 heterodimers with enhanced bioactivity in vivo.	Pubmed	0.00875	129	0.0149	0.152	1	2	BMP4
Pubmed	18955792	Association of specific IgE to staphylococcal superantigens with the phenotype of chronic urticaria.	GeneRIF	0.00875	129	0.0149	0.152	1	2	HLA-DQB1
Pubmed	18344018	Kallikrein-related peptidases.	Pubmed	0.00875	129	0.0149	0.152	1	2	KLK1
Pubmed	15617864	Gene, gut and schizophrenia: the meeting point for the gene-environment interaction in developing schizophrenia.	GeneRIF	0.00875	129	0.0149	0.152	1	2	HLA-DQB1
Pubmed	15996167	HLA-DQA1, -DQB1 polymorphism and genetic susceptibility to idiopathic dilated cardiomyopathy in Hans of northern China.	GeneRIF	0.00875	129	0.0149	0.152	1	2	HLA-DQB1
Pubmed	15081428	Modifiers of the jumonji mutation downregulate cyclin D1 expression and cardiac cell proliferation.	GeneRIF	0.00875	129	0.0149	0.152	1	2	KDM5A
Pubmed	20670354	Polymorphism of HLA class II genes in Berbers from Southern Tunisia.	GeneRIF	0.00875	129	0.0149	0.152	1	2	HLA-DQB1
Pubmed	22157634	Germline mosaic transmission of a novel duplication of PXDN and MYT1L to two male half-siblings with autism.	GeneRIF	0.00875	129	0.0149	0.152	1	2	PXDN
Pubmed	20039311	Osteoclast-specific Dicer gene deficiency suppresses osteoclastic bone resorption.	GeneRIF	0.00875	129	0.0149	0.152	1	2	CTSK
Pubmed	19468071	Rescue of skeletal muscle alpha-actin-null mice by cardiac (fetal) alpha-actin.	Pubmed	0.00875	129	0.0149	0.152	1	2	ACTA1
Pubmed	21191724	Haplotype analysis of the ARMS2/HTRA1 region in Japanese patients with typical neovascular age-related macular degeneration or polypoidal choroidal vasculopathy.	GeneRIF	0.00875	129	0.0149	0.152	1	2	HTRA1
Pubmed	15958261	Frequency of DRB1-DQB1 two-locus haplotypes in tuberculosis: preliminary report.	GeneRIF	0.00875	129	0.0149	0.152	1	2	HLA-DQB1
Pubmed	20208058	Multimodal CARS microscopy determination of the impact of diet on macrophage infiltration and lipid accumulation on plaque formation in ApoE-deficient mice.	Pubmed	0.00875	129	0.0149	0.152	1	2	CD68
Pubmed	19179430	Chronic endothelium-dependent regulation of arterial blood pressure by atrial natriuretic peptide: role of nitric oxide and endothelin-1.	GeneRIF	0.00875	129	0.0149	0.152	1	2	NPPA
Pubmed	11889175	Functionally impaired TR mutants are present in thyroid papillary cancer.	GeneRIF	0.00875	129	0.0149	0.152	1	2	THRA
Pubmed	25034269	LOTUS suppresses axon growth inhibition by blocking interaction between Nogo receptor-1 and all four types of its ligand.	Pubmed	0.00875	129	0.0149	0.152	1	2	RTN4R
Pubmed	25710914	cGAS and Ifi204 cooperate to produce type I IFNs in response to Francisella infection.	Pubmed	0.00875	129	0.0149	0.152	1	2	IFI16
Pubmed	20671064	The serine protease HtrA1 specifically interacts and degrades the tuberous sclerosis complex 2 protein.	GeneRIF	0.00875	129	0.0149	0.152	1	2	HTRA1
Pubmed	17992259	The muscle-specific ubiquitin ligase atrogin-1/MAFbx mediates statin-induced muscle toxicity.	GeneRIF	0.00875	129	0.0149	0.152	1	2	FBXO32
Pubmed	7902569	Thymocyte development in major histocompatibility complex-deficient mice: evidence for stochastic commitment to the CD4 and CD8 lineages.	Pubmed	0.00875	129	0.0149	0.152	1	2	HLA-DQB1
Pubmed	22418443	Transglutaminase 2 promotes both caspase-dependent and caspase-independent apoptotic cell death via the calpain/Bax protein signaling pathway.	GeneRIF	0.00875	129	0.0149	0.152	1	2	TGM2
Pubmed	17638887	Matrix metalloproteinase 7 mediates mammary epithelial cell tumorigenesis through the ErbB4 receptor.	GeneRIF	0.00875	129	0.0149	0.152	1	2	MMP7
Pubmed	8500908	Influence of genes from the major histocompatibility complex on the antibody repertoire against culture filtrate antigens in mice infected with live Mycobacterium bovis BCG.	Pubmed	0.00875	129	0.0149	0.152	1	2	HLA-DQB1
Pubmed	16313886	The p53 oncoprotein is a substrate for tissue transglutaminase kinase activity.	GeneRIF	0.00875	129	0.0149	0.152	1	2	TGM2
Pubmed	20378355	Is there an association of regulatory region polymorphism in the alpha-1-antichymotrypsin gene with sporadic Alzheimer's disease in the northern Han-Chinese population?	GeneRIF	0.00875	129	0.0149	0.152	1	2	ACTA1
Pubmed	2777341	Complete sequences of DQA1 and DQB1 cDNA clones corresponding to the DQw4 specificity.	Pubmed	0.00875	129	0.0149	0.152	1	2	HLA-DQB1
Pubmed	12562961	Isometric force and endurance in skeletal muscle of mice devoid of all known thyroid hormone receptors.	Pubmed	0.00875	129	0.0149	0.152	1	2	THRA
Pubmed	23939983	Polymorphisms in BMP4 and FGFR1 genes are associated with fracture non-union.	Pubmed	0.00875	129	0.0149	0.152	1	2	BMP4
Pubmed	16965413	Psoriasis vulgaris in Chinese individuals is associated with PSORS1C3 and CDSN genes.	GeneRIF	0.00875	129	0.0149	0.152	1	2	CDSN
Pubmed	6928693	Construction of recombinant plasmids containing rat muscle actin and myosin light chain DNA sequences.	Pubmed	0.00875	129	0.0149	0.152	1	2	ACTA1
Pubmed	14644808	Some new thoughts on the pathophysiology and genetics of polycystic ovary syndrome.	GeneRIF	0.00875	129	0.0149	0.152	1	2	CYP11A1

Category	ID	Name	Source	p-value	q-value Bonferroni	q-value FDR B&H	q-value FDR B&Y	Hit Count in Query List	Hit Count in Genome	Hit in Query List
Pubmed	11783381	[The study on human leucocyte antigen DQ region genes polymorphism in unexplained habitual abortion patients].	GeneRIF	0.00875	129	0.0149	0.152	1	2	HLA-DQB1
Pubmed	20860824	Irresponsiveness of two retinoblastoma cases to conservative therapy correlates with up- regulation of hERG1 channels and of the VEGF-A pathway.	GeneRIF	0.00875	129	0.0149	0.152	1	2	KCNH2
Pubmed	12676954	Multiple Ets factors and interferon regulatory factor-4 modulate CD68 expression in a cell type-specific manner.	GeneRIF	0.00875	129	0.0149	0.152	1	2	CD68
Pubmed	17349981	Familial Alzheimer's disease presenilin 1 mutation M146V increases gamma secretase cutting of p75NTR in vitro.	GeneRIF	0.00875	129	0.0149	0.152	1	2	NGFR
Pubmed	12095619	Suppression of DTT-induced aggregation of abrin by alphaA- and alphaB-crystallins: a model aggregation assay for alpha-crystallin chaperone activity in vitro.	GeneRIF	0.00875	129	0.0149	0.152	1	2	CRYAA
Pubmed	18511876	Association of E1AF mRNA expression with tumor progression and matrilysin in human rectal cancer.	GeneRIF	0.00875	129	0.0149	0.152	1	2	MMP7
Pubmed	18285402	The secretory leukocyte protease inhibitor (SLPI) and the secondary granule protein lactoferrin are synthesized in myelocytes, colocalize in subcellular fractions of neutrophils, and are coreleased by activated neutrophils.	GeneRIF	0.00875	129	0.0149	0.152	1	2	SLPI
Pubmed	16608842	Absence of dysferlin alters myogenin expression and delays human muscle differentiation "in vitro".	GeneRIF	0.00875	129	0.0149	0.152	1	2	DYSF
Pubmed	19407504	Differential gene expression in models of pituitary prolactin-producing tumoral cells.	GeneRIF	0.00875	129	0.0149	0.152	1	2	BMP4
Pubmed	17153464	Expression of MMP-7 and MT1-MMP in peritoneal dissemination of gastric cancer.	GeneRIF	0.00875	129	0.0149	0.152	1	2	MMP7
Pubmed	16803690	Effect of HLA DQ2, dietary exposure and coeliac disease on the development of antibody response to gliadin in children.	GeneRIF	0.00875	129	0.0149	0.152	1	2	HLA-DQB1
Pubmed	24764683	Preliminary study correlating CX3CL1/CX3CR1 expression with gastric carcinoma and gastric carcinoma perineural invasion.	Pubmed	0.00875	129	0.0149	0.152	1	2	CX3CL1
Pubmed	24023032	Human epididymis protein 4 and secretory leukocyte protease inhibitor in vaginal fluid: relation to vaginal components and bacterial composition.	Pubmed	0.00875	129	0.0149	0.152	1	2	SLPI
Pubmed	1944303	Isolation and characterization of mouse complementary DNAs encoding alpha and beta thyroid hormone receptors from thyrotrope cells: the mouse pituitary-specific beta 2 isoform differs at the amino terminus from the corresponding species from rat pituitary tumor cells.	Pubmed	0.00875	129	0.0149	0.152	1	2	THRA
Pubmed	12064918	Inhibition of Smad5 in human hematopoietic progenitors blocks erythroid differentiation induced by BMP4.	GeneRIF	0.00875	129	0.0149	0.152	1	2	BMP4
Pubmed	21199427	Cross-sectional and longitudinal association of serum alanine aminotransaminase and ¿-glutamyltransferase with metabolic syndrome in middle-aged and elderly Chinese people.	GeneRIF	0.00875	129	0.0149	0.152	1	2	GGT1
Pubmed	24026251	Ethanol induces cell cycle arrest and triggers apoptosis via Sp1-dependent p75NTR expression in human neuroblastoma cells.	Pubmed	0.00875	129	0.0149	0.152	1	2	NGFR
Pubmed	15703394	Nuclear translocation of the p75 neurotrophin receptor cytoplasmic domain in response to neurotrophin binding.	Pubmed	0.00875	129	0.0149	0.152	1	2	NGFR
Pubmed	12501218	Alpha-crystallin regions affected by adenosine 5'-triphosphate identified by hydrogen-deuterium exchange.	GeneRIF	0.00875	129	0.0149	0.152	1	2	CRYAA
Pubmed	21704030	Bone morphogenetic protein -4 and -5 in pancreatic cancer--novel bidirectional players.	GeneRIF	0.00875	129	0.0149	0.152	1	2	BMP4
Pubmed	17446207	Protein kinase C delta stimulates antigen presentation by Class II MHC in murine dendritic cells.	GeneRIF	0.00875	129	0.0149	0.152	1	2	HLA-DQB1
Pubmed	10679077	NK1.1+ T cells in the liver arise in the thymus and are selected by interactions with class I molecules on CD4+CD8+ cells.	Pubmed	0.00875	129	0.0149	0.152	1	2	HLA-DQB1
Pubmed	18339073	Celiac disease autoantibodies in severe autoimmune liver disease and the effect of liver transplantation.	GeneRIF	0.00875	129	0.0149	0.152	1	2	HLA-DQB1
Pubmed	18725990	Misfolded proteins activate factor XII in humans, leading to kallikrein formation without initiating coagulation.	GeneRIF	0.00875	129	0.0149	0.152	1	2	KLK1
Pubmed	16107953	A case-control study to examine HLA haplotype associations in patients with posttreatment chronic Lyme disease.	GeneRIF	0.00875	129	0.0149	0.152	1	2	HLA-DQB1
Pubmed	24462663	Mutation in osteoactivin decreases bone formation in vivo and osteoblast differentiation in vitro.	Pubmed	0.00875	129	0.0149	0.152	1	2	GPNMB
Pubmed	23125099	Xylosyl- and glucuronyltransferase functions of LARGE in a-dystroglycan modification are conserved in LARGE2.	Pubmed	0.00875	129	0.0149	0.152	1	2	LARGE
Pubmed	20923326	HLA-class II genes in Mexican Amerindian Mayas: relatedness with Guatemalan Mayans and other populations.	GeneRIF	0.00875	129	0.0149	0.152	1	2	HLA-DQB1
Pubmed	24112685	Cardiac channelopathies associated with infantile fatal ventricular arrhythmias: from the cradle to the bench.	Pubmed	0.00875	129	0.0149	0.152	1	2	KCNH2
Pubmed	12948297	HLA-DQ polymorphisms modify the risk of thyroid autoimmunity in children with type 1 diabetes mellitus.	GeneRIF	0.00875	129	0.0149	0.152	1	2	HLA-DQB1
Pubmed	20486920	Mold-sensitivity in children with moderate-severe asthma is associated with HLA-DR and HLA-DQ.	GeneRIF	0.00875	129	0.0149	0.152	1	2	HLA-DQB1
Pubmed	15070402	Mig12, a novel Opitz syndrome gene product partner, is expressed in the embryonic ventral midline and co-operates with Mid1 to bundle and stabilize microtubules.	GeneRIF	0.00875	129	0.0149	0.152	1	2	MID1
Pubmed	9438424	Developmental origin and Kit-dependent development of the interstitial cells of cajal in the mammalian small intestine.	Pubmed	0.00875	129	0.0149	0.152	1	2	MYH11

Category	ID	Name	Source	p-value	q-value Bonferroni	q-value FDR B&H	q-value FDR B&Y	Hit Count in Query List	Hit Count in Genome	Hit in Query List
Pubmed	19172437	Relationship between erythrocyte catalase and serum adenosine deaminase activities in eclampsia.	GeneRIF	0.00875	129	0.0149	0.152	1	2	ADA
Pubmed	15452304	Bias in parental transmission of the HLA-DR3 allele in Sardinian multiple sclerosis.	GeneRIF	0.00875	129	0.0149	0.152	1	2	HLA-DQB1
Pubmed	25174582	Angiopoietin-like protein 2 negatively regulated by microRNA-25 contributes to the malignant progression of colorectal cancer.	Pubmed	0.00875	129	0.0149	0.152	1	2	ANGPTL2
Pubmed	17429906	A mitochondrial DNA polymorphism associated with cardiac arrhythmia investigated in sudden infant death syndrome.	GeneRIF	0.00875	129	0.0149	0.152	1	2	KCNH2
Pubmed	16762005	Activated hepatic stellate cells overexpress p75NTR after partial hepatectomy and undergo apoptosis on nerve growth factor stimulation.	GeneRIF	0.00875	129	0.0149	0.152	1	2	NGFR
Pubmed	19027000	Dependence of myoblast fusion on a cortical actin wall and nonmuscle myosin IIA.	Pubmed	0.00875	129	0.0149	0.152	1	2	ACTA1
Pubmed	19939495	MBL-associated serine protease-3 circulates in high serum concentrations predominantly in complex with Ficolin-3 and regulates Ficolin-3 mediated complement activation.	GeneRIF	0.00875	129	0.0149	0.152	1	2	MASP1
Pubmed	12383268	The inhibitory region of troponin-I alters the ability of F-actin to interact with different segments of myosin.	GeneRIF	0.00875	129	0.0149	0.152	1	2	ACTA1
Pubmed	16382148	Tissue transglutaminase serves as an inhibitor of apoptosis by cross-linking caspase 3 in thapsigargin-treated cells.	GeneRIF	0.00875	129	0.0149	0.152	1	2	TGM2
Pubmed	23063527	Gravin is a transitory effector of polo-like kinase 1 during cell division.	GeneRIF	0.00875	129	0.0149	0.152	1	2	AKAP12
Pubmed	10024608	Update on interconversions of vitamin B-6 with its coenzyme.	Pubmed	0.00875	129	0.0149	0.152	1	2	PDXK
Pubmed	18223170	CD8+ but not CD4+ T cells require cognate interactions with target tissues to mediate GVHD across only minor H antigens, whereas both CD4+ and CD8+ T cells require direct leukemic contact to mediate GVL.	Pubmed	0.00875	129	0.0149	0.152	1	2	HLA-DQB1
Pubmed	22666441	Lack of correlation between outcomes of membrane repair assay and correction of dystrophic changes in experimental therapeutic strategy in dysferlinopathy.	Pubmed	0.00875	129	0.0149	0.152	1	2	DYSF
Pubmed	15802493	Athyroid Pax8-/- mice cannot be rescued by the inactivation of thyroid hormone receptor alpha1.	GeneRIF	0.00875	129	0.0149	0.152	1	2	THRA
Pubmed	23138098	Alpha thalassaemia and extended alpha globin genes in Sri Lanka.	GeneRIF	0.00875	129	0.0149	0.152	1	2	HBA2
Pubmed	8127707	Activation of myoD gene transcription by 3,5,3'-triiodo-L-thyronine: a direct role for the thyroid hormone and retinoid X receptors.	Pubmed	0.00875	129	0.0149	0.152	1	2	THRA
Pubmed	22337703	A study of the combined effects of the EHD3 and FREM3 genes in patients with major depressive disorder.	GeneRIF	0.00875	129	0.0149	0.152	1	2	EHD3
Pubmed	7589238	Localization of utrophin and aciculin at sites of cell matrix and cell-cell adhesion in cultured cells.	Pubmed	0.00875	129	0.0149	0.152	1	2	PGM5
Pubmed	17474152	Adenosine closes the K+ channel KCa3.1 in human lung mast cells and inhibits their migration via the adenosine A2A receptor.	GeneRIF	0.00875	129	0.0149	0.152	1	2	KCNN4
Pubmed	24970844	Listeria monocytogenes induces IFNß expression through an IFI16-, cGAS- and STING-dependent pathway.	Pubmed	0.00875	129	0.0149	0.152	1	2	IFI16
Pubmed	12404109	Evaluation of BMP4 and its specific inhibitor NOG as candidates in human neural tube defects (NTDs).	GeneRIF	0.00875	129	0.0149	0.152	1	2	BMP4
Pubmed	19667088	Complement protease MASP-1 activates human endothelial cells: PAR4 activation is a link between complement and endothelial function.	GeneRIF	0.00875	129	0.0149	0.152	1	2	MASP1
Pubmed	12083796	Mitogen-induced, FAK-dependent tyrosine phosphorylation of the SSeCKS scaffolding protein.	GeneRIF	0.00875	129	0.0149	0.152	1	2	AKAP12
Pubmed	16272144	Regulation of intracellular localization and transcriptional activity of FOXO4 by protein kinase B through phosphorylation at the motif sites conserved among the FOXO family.	Pubmed	0.00875	129	0.0149	0.152	1	2	FOXO4
Pubmed	22427585	Oligomerization with wt aA- and aB-crystallins reduces proteasome-mediated degradation of C-terminally truncated aA-crystallin.	Pubmed	0.00875	129	0.0149	0.152	1	2	CRYAA
Pubmed	11064106	L-selectin gene T668C mutation in type 1 diabetes patients and their first degree relatives.	GeneRIF	0.00875	129	0.0149	0.152	1	2	HLA-DQB1
Pubmed	25574047	Knockout of aA-crystallin inhibits ocular neovascularization.	Pubmed	0.00875	129	0.0149	0.152	1	2	CRYAA
Pubmed	15307413	Frequencies of sickle cell trait and glucose-6-phosphate dehydrogenase deficiency differ in highland and nearby lowland malaria-endemic areas of Kenya.	GeneRIF	0.00875	129	0.0149	0.152	1	2	HBB
Pubmed	12452372	Germline mutations in CFTR and PSTI genes in chronic pancreatitis patients.	GeneRIF	0.00875	129	0.0149	0.152	1	2	SPINK1
Pubmed	16905561	Heterogeneity at the HLA-DRB1 locus and risk for multiple sclerosis.	GeneRIF	0.00875	129	0.0149	0.152	1	2	HLA-DQB1
Pubmed	12370377	Enhancement of complement activation and opsonophagocytosis by complexes of mannose-binding lectin with mannose-binding lectin-associated serine protease after binding to Staphylococcus aureus.	GeneRIF	0.00875	129	0.0149	0.152	1	2	MASP1
Pubmed	14680508	HLA class II associations with rheumatic heart disease among clinically homogeneous patients in children in Latvia.	GeneRIF	0.00875	129	0.0149	0.152	1	2	HLA-DQB1
Pubmed	21616098	Proteolytic action of kallikrein-related peptidase 7 produces unique active matrix metalloproteinase-9 lacking the C-terminal hemopexin domains.	GeneRIF	0.00875	129	0.0149	0.152	1	2	KLK7
Pubmed	21844197	Interaction between the cardiac rapidly (IKr) and slowly (IKs) activating delayed rectifier potassium channels revealed by low K+-induced hERG endocytic degradation.	GeneRIF	0.00875	129	0.0149	0.152	1	2	KCNH2
Pubmed	19094093	Thyroid hormone receptor alpha plays an essential role in the normalisation of adult-onset hypothyroidism-related hypoexpression of synaptic plasticity target genes in striatum.	GeneRIF	0.00875	129	0.0149	0.152	1	2	THRA

Category	ID	Name	Source	p-value	q-value Bonferroni	q-value FDR B&H	q-value FDR B&Y	Hit Count in Query List	Hit Count in Genome	Hit in Query List
Pubmed	25450678	c-Cbl regulates aPix-mediated cell migration and invasion.	Pubmed	0.00875	129	0.0149	0.152	1	2	ARHGEF6
Pubmed	17967566	The MYO9B gene is a strong risk factor for developing refractory celiac disease.	GeneRIF	0.00875	129	0.0149	0.152	1	2	HLA-DQB1
Pubmed	14530329	T cells induce extended class II MHC compartments in dendritic cells in a Toll-like receptor-dependent manner.	Pubmed	0.00875	129	0.0149	0.152	1	2	HLA-DQB1
Pubmed	21768085	Membrane-type matrix metalloproteinase-3 regulates neuronal responsiveness to myelin through Nogo-66 receptor 1 cleavage.	GeneRIF	0.00875	129	0.0149	0.152	1	2	RTN4R
Pubmed	20156196	Transglutaminase-2: a new endostatin partner in the extracellular matrix of endothelial cells.	GeneRIF	0.00875	129	0.0149	0.152	1	2	TGM2
Pubmed	21454605	A multi-domain fragment of Nogo-A protein is a potent inhibitor of cortical axon regeneration via Nogo receptor 1.	GeneRIF	0.00875	129	0.0149	0.152	1	2	RTN4R
Pubmed	9771708	Mutations in orthologous genes in human spondyloepimetaphyseal dysplasia and the brachymorphic mouse.	Pubmed	0.00875	129	0.0149	0.152	1	2	PAPSS2
Pubmed	17960174	BMP-4 upregulates Kit expression in mouse melanoblasts prior to the Kit-dependent cycle of melanogenesis.	GeneRIF	0.00875	129	0.0149	0.152	1	2	BMP4
Pubmed	17712477	Ectopic expression of neurotrophic peptide derived from saposin C increases proliferation and upregulates androgen receptor expression and transcriptional activity in human prostate cancer cells.	GeneRIF	0.00875	129	0.0149	0.152	1	2	PSAP
Pubmed	16452242	An essential role of antigen-presenting cell/T-helper type 1 cell-cell interactions in draining lymph node during complete eradication of class II negative tumor tissue by T-helper type 1 cell therapy.	Pubmed	0.00875	129	0.0149	0.152	1	2	HLA-DQB1
Pubmed	15144476	Association of HLA-DQ5 and HLA-DR1 with sensitization to organic acid anhydrides.	GeneRIF	0.00875	129	0.0149	0.152	1	2	HLA-DQB1
Pubmed	22314623	Low macrophage accumulation in skeletal muscle of obese type 2 diabetics and elderly subjects.	GeneRIF	0.00875	129	0.0149	0.152	1	2	CD68
Pubmed	23273913	Down-regulation of CD74 inhibits growth and invasion in clear cell renal cell carcinoma through HIF-1a pathway.	Pubmed	0.00875	129	0.0149	0.152	1	2	CD74
Pubmed	18820539	Association of DRB1 and DQB1 HLA class II polymorphisms in high-grade and neoplastic cervical lesions of women from Argentina.	GeneRIF	0.00875	129	0.0149	0.152	1	2	HLA-DQB1
Pubmed	12055230	Dual functions of fractalkine/CX3C ligand 1 in trafficking of perforin+/granzyme B+ cytotoxic effector lymphocytes that are defined by CX3CR1 expression.	GeneRIF	0.00875	129	0.0149	0.152	1	2	CX3CL1
Pubmed	22962310	17ß-Estradiol-induced interaction of ERa with NPPA regulates gene expression in cardiomyocytes.	GeneRIF	0.00875	129	0.0149	0.152	1	2	NPPA
Pubmed	10346821	Mice devoid of all known thyroid hormone receptors are viable but exhibit disorders of the pituitary-thyroid axis, growth, and bone maturation.	Pubmed	0.00875	129	0.0149	0.152	1	2	THRA
Pubmed	17947650	Syndecan-4 mediates the coinhibitory function of DC-HIL on T cell activation.	GeneRIF	0.00875	129	0.0149	0.152	1	2	GPNMB
Pubmed	20826916	Bladder cancer risk associated with genotypic polymorphism of the matrix metalloproteinase-1 and 7 in North Indian population.	GeneRIF	0.00875	129	0.0149	0.152	1	2	MMP7
Pubmed	1850510	Genomic organization of the human thyroid hormone receptor alpha (c-erbA-1) gene.	Pubmed	0.00875	129	0.0149	0.152	1	2	THRA
Pubmed	15574430	Interleukin-18 is a pro-hypertrophic cytokine that acts through a phosphatidylinositol 3-kinase-phosphoinositide-dependent kinase-1-Akt-GATA4 signaling pathway in cardiomyocytes.	GeneRIF	0.00875	129	0.0149	0.152	1	2	NPPA
Pubmed	15861393	Serine protease HtrA1 is developmentally regulated in trophoblast and uterine decidual cells during placental formation in the mouse.	GeneRIF	0.00875	129	0.0149	0.152	1	2	HTRA1
Pubmed	19398782	Heparan sulfate proteoglycans are receptors for the cell-surface trafficking and biological activity of transglutaminase-2.	GeneRIF	0.00875	129	0.0149	0.152	1	2	TGM2
Pubmed	19477936	Analysis of hypertrophic thyrotrophs in pituitaries of athyroid Pax8-/- mice.	Pubmed	0.00875	129	0.0149	0.152	1	2	THRA
Pubmed	1845803	IFN-gamma induces the expression of the genes for MHC class II I-A beta and tumor necrosis factor through a protein kinase C-independent pathway.	Pubmed	0.00875	129	0.0149	0.152	1	2	HLA-DQB1
Pubmed	19581582	Prosaposin inhibits tumor metastasis via paracrine and endocrine stimulation of stromal p53 and Tsp-1.	GeneRIF	0.00875	129	0.0149	0.152	1	2	PSAP
Pubmed	11916169	Relative and absolute HLA-DQA1-DQB1 linked risk for developing type I diabetes before 40 years of age in the Belgian population: implications for future prevention studies.	GeneRIF	0.00875	129	0.0149	0.152	1	2	HLA-DQB1
Pubmed	8389625	Genetic mapping of the mouse topoisomerase II alpha gene to chromosome 11.	Pubmed	0.00875	129	0.0149	0.152	1	2	THRA
Pubmed	22605579	Doublecortin and doublecortin-like are expressed in overlapping and non-overlapping neuronal cell population: implications for neurogenesis.	GeneRIF	0.00875	129	0.0149	0.152	1	2	DCLK1
Pubmed	19144825	Association of v-ErbA with Smad4 disrupts TGF-beta signaling.	GeneRIF	0.00875	129	0.0149	0.152	1	2	THRA
Pubmed	17611763	Polymorphisms of Fractalkine receptor CX3CR1 and plasma levels of its ligand CX3CL1 in colorectal cancer patients.	GeneRIF	0.00875	129	0.0149	0.152	1	2	CX3CL1
Pubmed	16386650	Association of HLA-DR and HLA-DQ genes with susceptibility to pulmonary tuberculosis in Koreans: preliminary evidence of associations with drug resistance, disease severity, and disease recurrence.	GeneRIF	0.00875	129	0.0149	0.152	1	2	HLA-DQB1
Pubmed	22289203	Lack of significant influence for Fc¿RIIa-RH131 or hemoglobin AA/AS polymorphisms on immunity and susceptibility to uncomplicated malaria and existence of marked linkage between the two polymorphisms in Daraweesh.	GeneRIF	0.00875	129	0.0149	0.152	1	2	HBB
Pubmed	23300972	Gene specific actions of thyroid hormone receptor subtypes.	GeneRIF	0.00875	129	0.0149	0.152	1	2	THRA

Category	ID	Name	Source	p-value	q-value Bonferroni	q-value FDR B&H	q-value FDR B&Y	Hit Count in Query List	Hit Count in Genome	Hit in Query List
Pubmed	15826953	Main chain hydrogen bond interactions in the binding of proline-rich gluten peptides to the celiac disease-associated HLA-DQ2 molecule.	GeneRIF	0.00875	129	0.0149	0.152	1	2	HLA-DQB1
Pubmed	22699143	Screening of R122H and N29I mutations in the PRSS1 gene and N34S mutation in the SPINK1 gene in Mexican pediatric patients with acute and recurrent pancreatitis.	GeneRIF	0.00875	129	0.0149	0.152	1	2	SPINK1
Pubmed	10975810	TCR signaling for initiation and completion of thymocyte positive selection has distinct requirements for ligand quality and presenting cell type.	Pubmed	0.00875	129	0.0149	0.152	1	2	HLA-DQB1
Pubmed	22210872	Expression of BMPRIA on human thymic NK cell precursors: role of BMP signaling in intrathymic NK cell development.	GeneRIF	0.00875	129	0.0149	0.152	1	2	BMP4
Pubmed	22085609	Temperature-dependent structural and functional properties of a mutant (F71L) aA-crystallin: molecular basis for early onset of age-related cataract.	GeneRIF	0.00875	129	0.0149	0.152	1	2	CRYAA
Pubmed	20726994	ANP, BNP and D-dimer predict right ventricular dysfunction in patients with acute pulmonary embolism.	GeneRIF	0.00875	129	0.0149	0.152	1	2	NPPA
Pubmed	15187169	Development of antigen-specific CD8+ CTL in MHC class I-deficient mice through CD4 to CD8 conversion.	Pubmed	0.00875	129	0.0149	0.152	1	2	HLA-DQB1
Pubmed	12417635	Invariant chain controls the activity of extracellular cathepsin L.	GeneRIF	0.00875	129	0.0149	0.152	1	2	CD74
Pubmed	23549871	Prespecified candidate biomarkers identify follicular lymphoma patients who achieved longer progression-free survival with bortezomib-rituximab versus rituximab.	Pubmed	0.00875	129	0.0149	0.152	1	2	CD68
Pubmed	19855381	Enteric defensins are essential regulators of intestinal microbial ecology.	GeneRIF	0.00875	129	0.0149	0.152	1	2	MMP7
Pubmed	11350761	Leiomodin and tropomodulin in smooth muscle.	GeneRIF	0.00875	129	0.0149	0.152	1	2	LMOD1
Pubmed	16215672	Modulation of Fas-induced apoptosis by p75 neurotrophin receptor in a human neuroblastoma cell line.	GeneRIF	0.00875	129	0.0149	0.152	1	2	NGFR
Pubmed	18481581	Human leukocyte antigen class II alleles and dental caries in a child population.	GeneRIF	0.00875	129	0.0149	0.152	1	2	HLA-DQB1
Pubmed	9723702	An altered invariant chain protein with an antigenic peptide in place of CLIP forms SDS-stable complexes with class II alphabeta dimers and facilitates highly efficient peptide loading.	Pubmed	0.00875	129	0.0149	0.152	1	2	CD74
Pubmed	18485588	Human leukocyte elastase counteracts matrix metalloproteinase-7 induced apoptosis resistance of tumor cells.	GeneRIF	0.00875	129	0.0149	0.152	1	2	MMP7
Pubmed	21216831	Decompensated heart failure is associated with reduced corin levels and decreased cleavage of pro-atrial natriuretic peptide.	GeneRIF	0.00875	129	0.0149	0.152	1	2	NPPA
Pubmed	1074709	My franchise was a teen-age lion's den.	Pubmed	0.00875	129	0.0149	0.152	1	2	ACTA1
Pubmed	24404170	Targeting of GFP-Cre to the mouse Cyp11a1 locus both drives cre recombinase expression in steroidogenic cells and permits generation of Cyp11a1 knock out mice.	Pubmed	0.00875	129	0.0149	0.152	1	2	CYP11A1
Pubmed	24174323	Translocator protein/peripheral benzodiazepine receptor is not required for steroid hormone biosynthesis.	Pubmed	0.00875	129	0.0149	0.152	1	2	TSPO
Pubmed	19644512	The adenine nucleotide translocator 1 acts as a type 2 transglutaminase substrate: implications for mitochondrial-dependent apoptosis.	GeneRIF	0.00875	129	0.0149	0.152	1	2	TGM2
Pubmed	21257328	Accumulation and distribution of a-synuclein and ubiquitin in the CNS of Gaucher disease mouse models.	Pubmed	0.00875	129	0.0149	0.152	1	2	PSAP
Pubmed	18409517	[Increased activity of PRPP synthetase].	GeneRIF	0.00875	129	0.0149	0.152	1	2	PRPS2
Pubmed	17381426	Substrate specificity and catalytic efficiency of aldo-keto reductases with phospholipid aldehydes.	Pubmed	0.00875	129	0.0149	0.152	1	2	AKR1B10
Pubmed	16670267	Endothelial catabolism of extracellular adenosine during hypoxia: the role of surface adenosine deaminase and CD26.	GeneRIF	0.00875	129	0.0149	0.152	1	2	ADA
Pubmed	21797699	The carrier frequency of a-globin gene triplication in an Iranian population with normal or borderline hematological parameters.	GeneRIF	0.00875	129	0.0149	0.152	1	2	HBA2
Pubmed	17977773	Ubiquitin ligases MuRF1 and MAFbx in human skeletal muscle atrophy.	GeneRIF	0.00875	129	0.0149	0.152	1	2	FBXO32
Pubmed	8006596	Class I dependence of the development of CD4+ CD8- NK1.1+ thymocytes.	Pubmed	0.00875	129	0.0149	0.152	1	2	HLA-DQB1
Pubmed	21750192	The E3 ubiquitin ligase specificity subunit ASB2a targets filamins for proteasomal degradation by interacting with the filamin actin-binding domain.	Pubmed	0.00875	129	0.0149	0.152	1	2	ASB2
Pubmed	15329520	[Mutations of SPINK1 and PRSS1 gene in Korean patients with chronic pancreatitis].	GeneRIF	0.00875	129	0.0149	0.152	1	2	SPINK1
Pubmed	15761857	Adaptation to past malarial endemia and susceptibility to common diseases in modern populations: a study of adenosine deaminase and MN blood group genetic polymorphisms.	GeneRIF	0.00875	129	0.0149	0.152	1	2	ADA
Pubmed	7881131	Temporally and spatially restricted expression of apolipoprotein J in the developing heart defines discrete stages of valve morphogenesis.	Pubmed	0.00875	129	0.0149	0.152	1	2	NPPA
Pubmed	17569791	Malignant transformation of multipotent muscle-derived cells by concurrent differentiation signals.	GeneRIF	0.00875	129	0.0149	0.152	1	2	BMP4
Pubmed	19232384	Abundant expression of Kallikrein 1 gene in human keratinocytes was mediated by GATA3.	GeneRIF	0.00875	129	0.0149	0.152	1	2	KLK1
Pubmed	20068219	H4R3 methylation facilitates beta-globin transcription by regulating histone acetyltransferase binding and H3 acetylation.	GeneRIF	0.00875	129	0.0149	0.152	1	2	HBB
Pubmed	9069129	Localization of the mitochondrial carbonic anhydrase V gene, Car5, on mouse chromosome 8.	Pubmed	0.00875	129	0.0149	0.152	1	2	ACTA1
Pubmed	24130878	Direct interaction between AR and PAK6 in androgen-stimulated PAK6 activation.	Pubmed	0.00875	129	0.0149	0.152	1	2	PAK6
Pubmed	19359372	Ether-à-go-go-related gene K+ channels contribute to threshold excitability of mouse auditory brainstem neurons.	GeneRIF	0.00875	129	0.0149	0.152	1	2	KCNH2
Pubmed	23416980	Loss of miR-125b-1 contributes to head and neck cancer development by dysregulating TACSTD2 and MAPK pathway.	Pubmed	0.00875	129	0.0149	0.152	1	2	TACSTD2

Category	ID	Name	Source	p-value	q-value Bonferroni	q-value FDR B&H	q-value FDR B&Y	Hit Count in Query List	Hit Count in Genome	Hit in Query List
Pubmed	16303674	Human leukocyte antigen-DQB1 and -DRB1 associations in patients with idiopathic sudden sensorineural hearing loss from a defined population of Northwest Spain.	GeneRIF	0.00875	129	0.0149	0.152	1	2	HLA-DQB1
Pubmed	17122049	The Nogo-Nogo receptor pathway limits a spectrum of adult CNS axonal growth.	Pubmed	0.00875	129	0.0149	0.152	1	2	RTN4R
Pubmed	17919990	HLA DQ gene dosage and risk and severity of celiac disease.	GeneRIF	0.00875	129	0.0149	0.152	1	2	HLA-DQB1
Pubmed	15471889	The co-inheritance of type 1 diabetes and multiple sclerosis in Sardinia cannot be explained by genotype variation in the HLA region alone.	GeneRIF	0.00875	129	0.0149	0.152	1	2	HLA-DQB1
Pubmed	9254699	Peg5/Neuronatin is an imprinted gene located on sub-distal chromosome 2 in the mouse.	Pubmed	0.00875	129	0.0149	0.152	1	2	ADA
Pubmed	8398147	p75-deficient trigeminal sensory neurons have an altered response to NGF but not to other neurotrophins.	Pubmed	0.00875	129	0.0149	0.152	1	2	NGFR
Pubmed	17610817	Polarized signaling endosomes coordinate BDNF-induced chemotaxis of cerebellar precursors.	Pubmed	0.00875	129	0.0149	0.152	1	2	NGFR
Pubmed	19317620	Transforming growth factor-B1 and matrix metalloproteinase-7 promoter variants induce risk for Helicobacter pylori-associated gastric precancerous lesions.	GeneRIF	0.00875	129	0.0149	0.152	1	2	MMP7
Pubmed	11309382	Inhibition of c-Abl tyrosine kinase activity by filamentous actin.	Pubmed	0.00875	129	0.0149	0.152	1	2	ACTA1
Pubmed	24352660	Control of vascular permeability by atrial natriuretic peptide via a GEF-H1-dependent mechanism.	Pubmed	0.00875	129	0.0149	0.152	1	2	NPPA
Pubmed	20566851	Dissecting the involvement of tropomyosin-related kinase A and p75 neurotrophin receptor signaling in NGF deficit-induced neurodegeneration.	GeneRIF	0.00875	129	0.0149	0.152	1	2	NGFR
Pubmed	12975523	A1 adenosine receptors mediate hypoxia-induced ventriculomegaly.	Pubmed	0.00875	129	0.0149	0.152	1	2	ADA
Pubmed	21415673	High incidence of PRSS1 and SPINK1 mutations in Korean children with acute recurrent and chronic pancreatitis.	GeneRIF	0.00875	129	0.0149	0.152	1	2	SPINK1
Pubmed	23727038	Rhinovirus colocalizes with CD68- and CD11b-positive macrophages following experimental infection in humans.	Pubmed	0.00875	129	0.0149	0.152	1	2	CD68
Pubmed	18804908	Glucosamine is an effective chemo-sensitizer via transglutaminase 2 inhibition.	GeneRIF	0.00875	129	0.0149	0.152	1	2	TGM2
Pubmed	16148119	Syk is required for monocyte/macrophage chemotaxis to CX3CL1 (Fractalkine).	GeneRIF	0.00875	129	0.0149	0.152	1	2	CX3CL1
Pubmed	17180363	Is typing for HLA class II alleles beneficial in Indian children with idiopathic nephrotic syndrome?	GeneRIF	0.00875	129	0.0149	0.152	1	2	HLA-DQB1
Pubmed	18709654	The p75 receptor is associated with inflammatory thermal hypersensitivity.	GeneRIF	0.00875	129	0.0149	0.152	1	2	NGFR
Pubmed	15740708	Transglutaminase 2 expression in the salivary myoepithelial cells of mouse embryo.	Pubmed	0.00875	129	0.0149	0.152	1	2	TGM2
Pubmed	15756536	Combined positivity for HLA DQ2/DQ8 and IA-2 antibodies defines population at high risk of developing type 1 diabetes.	GeneRIF	0.00875	129	0.0149	0.152	1	2	HLA-DQB1
Pubmed	16453125	Congenital cataract and macular hypoplasia in humans associated with a de novo mutation in CRYAA and compound heterozygous mutations in P.	GeneRIF	0.00875	129	0.0149	0.152	1	2	CRYAA
Pubmed	16320082	D6S265*15 marks a DRB1*15, DQB1*0602 haplotype associated with attenuated protection from type 1 diabetes mellitus.	GeneRIF	0.00875	129	0.0149	0.152	1	2	HLA-DQB1
Pubmed	1360280	Isolation and mapping of four new DNA markers from mouse chromosome 4.	Pubmed	0.00875	129	0.0149	0.152	1	2	NPPA
Pubmed	11750125	Genomic structure, alternative splice forms and normal and mutant alleles of cadherin 23 (Cdh23).	Pubmed	0.00875	129	0.0149	0.152	1	2	PSAP
Pubmed	20219632	Distinct signaling pathways of precursor BDNF and mature BDNF in cultured cerebellar granule neurons.	GeneRIF	0.00875	129	0.0149	0.152	1	2	NGFR
Pubmed	25400811	Association of natriuretic peptide polymorphisms with left ventricular dysfunction in southern Han Chinese coronary artery disease patients.	Pubmed	0.00875	129	0.0149	0.152	1	2	NPPA
Pubmed	15188059	Human recombinant B7-H3 expressed in E. coli enhances T lymphocyte proliferation and IL-10 secretion in vitro.	GeneRIF	0.00875	129	0.0149	0.152	1	2	CD276
Pubmed	19845278	ANP and insulin presence in human foetal pancreas.	GeneRIF	0.00875	129	0.0149	0.152	1	2	NPPA
Pubmed	16083868	Activated mouse T-cells synthesize MHC class II, process, and present morbillivirus nucleocapsid protein to primed T-cells.	Pubmed	0.00875	129	0.0149	0.152	1	2	HLA-DQB1
Pubmed	15305234	HLA-DRB1*04 and HLA-DQB1*03 association with the atrophic but not with the goitrous form of chronic autoimmune thyroiditis in a Brazilian population.	GeneRIF	0.00875	129	0.0149	0.152	1	2	HLA-DQB1
Pubmed	22164220	The GATA1-HS2 enhancer allows persistent and position-independent expression of a ß-globin transgene.	GeneRIF	0.00875	129	0.0149	0.152	1	2	HBB
Pubmed	7010180	Homology and concerted evolution at the alpha 1 and alpha 2 loci of human alpha-globin.	Pubmed	0.00875	129	0.0149	0.152	1	2	HBA2
Pubmed	20044576	O-mannosyl phosphorylation of alpha-dystroglycan is required for laminin binding.	GeneRIF	0.00875	129	0.0149	0.152	1	2	LARGE
Pubmed	19073770	Gene silencing of phogrin unveils its essential role in glucose-responsive pancreatic beta-cell growth.	GeneRIF	0.00875	129	0.0149	0.152	1	2	HLA-DQB1
Pubmed	24763718	JC polyomavirus infection is strongly controlled by human leucocyte antigen class II variants.	Pubmed	0.00875	129	0.0149	0.152	1	2	HLA-DQB1
Pubmed	15242955	HLA class II profile and distribution of HLA-DRB1 and HLA-DQB1 alleles and haplotypes among Lebanese and Bahraini Arabs.	GeneRIF	0.00875	129	0.0149	0.152	1	2	HLA-DQB1
Pubmed	17002902	Human leukocyte antigen non-class II determinants for type 1 diabetes in the Finnish population.	GeneRIF	0.00875	129	0.0149	0.152	1	2	HLA-DQB1
Pubmed	15378608	Ternary complex with Trk, p75, and an ankyrin-rich membrane spanning protein.	Pubmed	0.00875	129	0.0149	0.152	1	2	NGFR
Pubmed	25172978	HLA genotyping in pediatric celiac disease patients.	Pubmed	0.00875	129	0.0149	0.152	1	2	HLA-DQB1
Pubmed	7518430	Functional alterations in adult and fetal hemoglobin Sassari Asp-alpha 126(H9)-->His. The role of alpha 1 alpha 2 contact.	Pubmed	0.00875	129	0.0149	0.152	1	2	HBA2

Category	ID	Name	Source	p-value	q-value Bonferroni	q-value FDR B&H	q-value FDR B&Y	Hit Count in Query List	Hit Count in Genome	Hit in Query List
Pubmed	20101097	Estimation of genetic risk for Type 1 diabetes mellitus in newborns on dried blood spot.	GeneRIF	0.00875	129	0.0149	0.152	1	2	HLA-DQB1
Pubmed	2901712	Duplication of the bcr and gamma-glutamyl transpeptidase genes.	Pubmed	0.00875	129	0.0149	0.152	1	2	GGT1
Pubmed	22242175	Human PAPS synthase isoforms are dynamically regulated enzymes with access to nucleus and cytoplasm.	GeneRIF	0.00875	129	0.0149	0.152	1	2	PAPSS2
Pubmed	3137158	Signaling to a B-cell clone by Ek, but not Ak, does not reflect alteration of Ak genes.	Pubmed	0.00875	129	0.0149	0.152	1	2	HLA-DQB1
Pubmed	23710594	Differential sensitivity of Pak5, Pak6, and Pak5/Pak6 double-knockout mice to the stimulant effects of amphetamine and exercise-induced alterations in body weight.	Pubmed	0.00875	129	0.0149	0.152	1	2	PAK6
Pubmed	10523848	alphaPIX nucleotide exchange factor is activated by interaction with phosphatidylinositol 3-kinase.	Pubmed	0.00875	129	0.0149	0.152	1	2	ARHGEF6
Pubmed	15673572	The primitive streak gene Mixl1 is required for efficient haematopoiesis and BMP4-induced ventral mesoderm patterning in differentiating ES cells.	GeneRIF	0.00875	129	0.0149	0.152	1	2	BMP4
Pubmed	7547576	International Cell Exchange, 1994.	Pubmed	0.00875	129	0.0149	0.152	1	2	HLA-DQB1
Pubmed	12072400	A targeted thyroid hormone receptor alpha gene dominant-negative mutation (P398H) selectively impairs gene expression in differentiated embryonic stem cells.	GeneRIF	0.00875	129	0.0149	0.152	1	2	THRA
Pubmed	7556925	NGF receptor expression in sensory neurons develops normally in embryos lacking NGF.	Pubmed	0.00875	129	0.0149	0.152	1	2	NGFR
Pubmed	19446361	Cathepsin X prevents an effective immune response against Helicobacter pylori infection.	GeneRIF	0.00875	129	0.0149	0.152	1	2	CTSK
Pubmed	20169155	Quantitative kinetic study of the actin-bundling protein L-plastin and of its impact on actin turn-over.	GeneRIF	0.00875	129	0.0149	0.152	1	2	ACTA1
Pubmed	23146748	Decreased levels of AKR1B1 and AKR1B10 in cancerous endometrium compared to adjacent non-cancerous tissue.	GeneRIF	0.00875	129	0.0149	0.152	1	2	AKR1B10
Pubmed	17675532	The acute-phase protein alpha 1-acid glycoprotein (AGP) induces rises in cytosolic Ca2+ in neutrophil granulocytes via sialic acid binding immunoglobulin-like lectins (siglecs).	GeneRIF	0.00875	129	0.0149	0.152	1	2	ORM1
Pubmed	17177860	Carboxypeptidase E in the mouse placenta.	GeneRIF	0.00875	129	0.0149	0.152	1	2	CPE
Pubmed	18593767	Anti-mullerian hormone and inhibin B in the definition of ovarian aging and the menopause transition.	GeneRIF	0.00875	129	0.0149	0.152	1	2	INHBB
Pubmed	22665496	Cross talk between signaling and vitamin A transport by the retinol-binding protein receptor STRA6.	GeneRIF	0.00875	129	0.0149	0.152	1	2	STRA6
Pubmed	9024139	Four novel KVLQT1 and four novel HERG mutations in familial long-QT syndrome.	Pubmed	0.00875	129	0.0149	0.152	1	2	KCNH2
Pubmed	8524305	Unliganded thyroid hormone receptor alpha can target TATA-binding protein for transcriptional repression.	Pubmed	0.00875	129	0.0149	0.152	1	2	THRA
Pubmed	7540943	In the absence of a CD40 signal, B cells are tolerogenic.	Pubmed	0.00875	129	0.0149	0.152	1	2	HLA-DQB1
Pubmed	22738610	Hb E/ß-thalassemia: the second most common cause of transfusion-dependent thalassemia in the Gwalior-Chambal region of Central India.	GeneRIF	0.00875	129	0.0149	0.152	1	2	HBB
Pubmed	12011451	Positive selection of self-MHC-reactive T cells by individual peptide-MHC class II complexes.	Pubmed	0.00875	129	0.0149	0.152	1	2	CD74
Pubmed	18385523	Tissue inhibitor of metalloproteinase-1 moderates airway re-epithelialization by regulating matrilysin activity.	GeneRIF	0.00875	129	0.0149	0.152	1	2	MMP7
Pubmed	20345872	Human leucocyte antigen class II DRB1 and DQB1 associations in human immunodeficiency virus-infected patients of Mumbai, India.	GeneRIF	0.00875	129	0.0149	0.152	1	2	HLA-DQB1
Pubmed	25575539	Intraperitoneal administration of activated protein C prevents postsurgical adhesion band formation.	Pubmed	0.00875	129	0.0149	0.152	1	2	PROCR
Pubmed	10757647	Connexin mutations associated with palmoplantar keratoderma and profound deafness in a single family.	Pubmed	0.00875	129	0.0149	0.152	1	2	GJB3
Pubmed	12933686	Functional activation of cerebral metabolism in mice with mutated thyroid hormone nuclear receptors.	Pubmed	0.00875	129	0.0149	0.152	1	2	THRA
Pubmed	18636074	Cbfa1-dependent expression of an interferon-inducible p204 protein is required for chondrocyte differentiation.	GeneRIF	0.00875	129	0.0149	0.152	1	2	IFI16
Pubmed	15017020	Atrial natriuretic peptide gene polymorphisms and risk of ischemic stroke in humans.	GeneRIF	0.00875	129	0.0149	0.152	1	2	NPPA
Pubmed	21575908	IFI16 acts as a nuclear pathogen sensor to induce the inflammasome in response to Kaposi Sarcoma-associated herpesvirus infection.	GeneRIF	0.00875	129	0.0149	0.152	1	2	IFI16
Pubmed	7539415	Secretory leucocyte protease inhibitor in the male genital tract: PSA-induced proteolytic processing in human semen and tissue localization.	Pubmed	0.00875	129	0.0149	0.152	1	2	SLPI
Pubmed	23344953	BMP induces cochlin expression to facilitate self-renewal and suppress neural differentiation of mouse embryonic stem cells.	GeneRIF	0.00875	129	0.0149	0.152	1	2	BMP4
Pubmed	25249603	Upregulation of bone morphogenetic protein-1/mammalian tolloid and procollagen C-proteinase enhancer-1 in corneal scarring.	Pubmed	0.00875	129	0.0149	0.152	1	2	BMP4
Pubmed	20392899	Patient HLA-DRB1* and -DQB1* allele and haplotype association with hepatitis C virus persistence and clearance.	GeneRIF	0.00875	129	0.0149	0.152	1	2	HLA-DQB1
Pubmed	10362681	Cardiovascular phenotype and temperature control in mice lacking thyroid hormone receptor-beta or both alpha1 and beta.	Pubmed	0.00875	129	0.0149	0.152	1	2	THRA
Pubmed	7875199	Immune response to Mycobacterium bovis bacille Calmette Guérin infection in major histocompatibility complex class I- and II-deficient knock-out mice: contribution of CD4 and CD8 T cells to acquired resistance.	Pubmed	0.00875	129	0.0149	0.152	1	2	HLA-DQB1
Pubmed	25009391	Association of human leukocyte antigen DQB1 and DRB1 alleles with chronic hepatitis B.	Pubmed	0.00875	129	0.0149	0.152	1	2	HLA-DQB1
Pubmed	10485890	Identification of a zinc finger protein whose subcellular distribution is regulated by serum and nerve growth factor.	Pubmed	0.00875	129	0.0149	0.152	1	2	NGFR

Category	ID	Name	Source	p-value	q-value Bonferroni	q-value FDR B&H	q-value FDR B&Y	Hit Count in Query List	Hit Count in Genome	Hit in Query List
Pubmed	21520985	Immunohistochemical localization and analysis of kallikrein-related peptidase 7 and 11 expression in paired cancer and benign foci in prostate cancer patients.	GeneRIF	0.00875	129	0.0149	0.152	1	2	KLK7
Pubmed	19917609	Serine 254 enhances an induced fit mechanism in murine 5-aminolevulinate synthase.	GeneRIF	0.00875	129	0.0149	0.152	1	2	ALAS2
Pubmed	25256355	New susceptibility variants to narcolepsy identified in HLA class II region.	Pubmed	0.00875	129	0.0149	0.152	1	2	HLA-DQB1
Pubmed	17241867	Enhanced recruitment of CX3CR1+ T cells by mucosal endothelial cell-derived fractalkine in inflammatory bowel disease.	GeneRIF	0.00875	129	0.0149	0.152	1	2	CX3CL1
Pubmed	18688283	The intermediate-conductance calcium-activated potassium channel KCa3.1 contributes to atherogenesis in mice and humans.	GeneRIF	0.00875	129	0.0149	0.152	1	2	KCNN4
Pubmed	10559237	Molecular cloning and characterization of human trabeculin-alpha, a giant protein defining a new family of actin-binding proteins.	Pubmed	0.00875	129	0.0149	0.152	1	2	ACTA1
Pubmed	8573092	Inhibition of human pancreatic proteinases by mucus proteinase inhibitor, eglin c and aprotinin.	Pubmed	0.00875	129	0.0149	0.152	1	2	SLPI
Pubmed	23241402	Role of proinflammatory CD68(+) mannose receptor(-) macrophages in peroxiredoxin-1 expression and in abdominal aortic aneurysms in humans.	GeneRIF	0.00875	129	0.0149	0.152	1	2	CD68
Pubmed	9268132	Expression of multiple thyroid hormone receptor mRNAs in human oocytes, cumulus cells, and granulosa cells.	Pubmed	0.00875	129	0.0149	0.152	1	2	THRA
Pubmed	19151380	Mechanism of cataract formation in alphaA-crystallin Y118D mutation.	Pubmed	0.00875	129	0.0149	0.152	1	2	CRYAA
Pubmed	20688330	Sulforaphane inhibits endothelial lipase expression through NF-¿B in endothelial cells.	GeneRIF	0.00875	129	0.0149	0.152	1	2	LIPG
Pubmed	12781692	Heparan sulfate-FGF10 interactions during lung morphogenesis.	GeneRIF	0.00875	129	0.0149	0.152	1	2	BMP4
Pubmed	17015616	Regulation of myogenic progenitor proliferation in human fetal skeletal muscle by BMP4 and its antagonist Gremlin.	GeneRIF	0.00875	129	0.0149	0.152	1	2	BMP4
Pubmed	12028537	Association of HLA-DRB1*1502-DQB1*0501 haplotype with susceptibility to systemic lupus erythematosus in Thais.	GeneRIF	0.00875	129	0.0149	0.152	1	2	HLA-DQB1
Pubmed	15111313	Tumor necrosis factor-alpha induces fractalkine expression preferentially in arterial endothelial cells and mithramycin A suppresses TNF-alpha-induced fractalkine expression.	GeneRIF	0.00875	129	0.0149	0.152	1	2	CX3CL1
Pubmed	15719367	Thymic cortical epithelium induces self tolerance.	Pubmed	0.00875	129	0.0149	0.152	1	2	HLA-DQB1
Pubmed	9326226	Involvement of transcription factor encoded by the mouse mi locus (MITF) in expression of p75 receptor of nerve growth factor in cultured mast cells of mice.	Pubmed	0.00875	129	0.0149	0.152	1	2	NGFR
Pubmed	11309393	Molecular determinants of the interaction between the inositol 1,4,5-trisphosphate receptor-associated cGMP kinase substrate (IRAG) and cGMP kinase Ibeta.	Pubmed	0.00875	129	0.0149	0.152	1	2	MRVI1
Pubmed	18673368	Transglutaminases and transglutaminase-catalyzed cross-links colocalize with the pathological lesions in Alzheimer's disease brain.	GeneRIF	0.00875	129	0.0149	0.152	1	2	TGM2
Pubmed	11877456	CHO1, a mammalian kinesin-like protein, interacts with F-actin and is involved in the terminal phase of cytokinesis.	GeneRIF	0.00875	129	0.0149	0.152	1	2	ACTA1
Pubmed	18433881	Parental non-inherited HLA resistance alleles do not confer protection against multiple sclerosis.	GeneRIF	0.00875	129	0.0149	0.152	1	2	HLA-DQB1
Pubmed	24140838	AKR1B10, a transcriptional target of p53, is downregulated in colorectal cancers associated with poor prognosis.	Pubmed	0.00875	129	0.0149	0.152	1	2	AKR1B10
Pubmed	18239127	Cathepsin K-dependent toll-like receptor 9 signaling revealed in experimental arthritis.	GeneRIF	0.00875	129	0.0149	0.152	1	2	CTSK
Pubmed	23364466	Loss-of-function ferrochelatase and gain-of-function erythroid-specific 5-aminolevulinate synthase mutations causing erythropoietic protoporphyria and x-linked protoporphyria in North American patients reveal novel mutations and a high prevalence of X-linked protoporphyria.	Pubmed	0.00875	129	0.0149	0.152	1	2	ALAS2
Pubmed	18537141	Bone morphogenetic protein regulation of enteric neuronal phenotypic diversity: relationship to timing of cell cycle exit.	GeneRIF	0.00875	129	0.0149	0.152	1	2	BMP4
Pubmed	21508093	The oligomeric state of thyroid receptor regulates hormone binding kinetics.	GeneRIF	0.00875	129	0.0149	0.152	1	2	THRA
Pubmed	15223613	Involvement of fractalkine and macrophage inflammatory protein-1 alpha in moderate-severe depression.	GeneRIF	0.00875	129	0.0149	0.152	1	2	CX3CL1
Pubmed	11756990	Associations between human leukocyte antigens and nonresponsiveness to influenza vaccine.	GeneRIF	0.00875	129	0.0149	0.152	1	2	HLA-DQB1
Pubmed	20709056	aA-Crystallin associates with a6 integrin receptor complexes and regulates cellular signaling.	Pubmed	0.00875	129	0.0149	0.152	1	2	CRYAA
Pubmed	22652599	Fractalkine activates a signal transduction pathway similar to P2Y12 and is associated with impaired clopidogrel responsiveness.	GeneRIF	0.00875	129	0.0149	0.152	1	2	CX3CL1
Pubmed	16796128	HLA class II allele frequencies in type 1A diabetes mellitus Slovak patients.	GeneRIF	0.00875	129	0.0149	0.152	1	2	HLA-DQB1
Pubmed	17932484	Hypermetabolism in mice caused by the central action of an unliganded thyroid hormone receptor alpha1.	GeneRIF	0.00875	129	0.0149	0.152	1	2	THRA
Pubmed	19244160	Hyperantithrombotic, noncytoprotective Glu149Ala-activated protein C mutant.	GeneRIF	0.00875	129	0.0149	0.152	1	2	PROCR
Pubmed	15979995	Presence of membrane and soluble forms of Fas ligand and of matrilysin (MMP-7) activity in normal and abnormal human semen.	GeneRIF	0.00875	129	0.0149	0.152	1	2	MMP7
Pubmed	20019415	Upregulation of fractalkine and its receptor, CX3CR1, is associated with coronary plaque rupture in patients with unstable angina pectoris.	GeneRIF	0.00875	129	0.0149	0.152	1	2	CX3CL1
Pubmed	23155424	Adiponectin mediated MHC class II mismatched cardiac graft rejection in mice is IL-4 dependent.	Pubmed	0.00875	129	0.0149	0.152	1	2	HLA-DQB1

Category	ID	Name	Source	p-value	q-value Bonferroni	q-value FDR B&H	q-value FDR B&Y	Hit Count in Query List	Hit Count in Genome	Hit in Query List
Pubmed	19030200	Fractalkine and TGF-beta1 levels reflect the severity of chronic pancreatitis in humans.	GeneRIF	0.00875	129	0.0149	0.152	1	2	CX3CL1
Pubmed	22174419	Receptor for activated C kinase 1 (RACK1) inhibits function of transient receptor potential (TRP)-type channel Pkd2L1 through physical interaction.	GeneRIF	0.00875	129	0.0149	0.152	1	2	PKD2L1
Pubmed	20194731	Thyroid hormone receptor {alpha} modulates lipopolysaccharide-induced changes in peripheral thyroid hormone metabolism.	Pubmed	0.00875	129	0.0149	0.152	1	2	THRA
Pubmed	17531857	HLA-DRB1*1501, -DQB1*0301, -DQB1*0302, -DQB1*0602, and -DQB1*0603 alleles are associated with more severe disease outcome on MRI in patients with multiple sclerosis.	GeneRIF	0.00875	129	0.0149	0.152	1	2	HLA-DQB1
Pubmed	8039701	Identification of negative-acting and protein-binding elements in the mouse alpha A-crystallin -1556/-1165 region.	Pubmed	0.00875	129	0.0149	0.152	1	2	CRYAA
Pubmed	15906352	HLA class II allele control of HPV load in carcinoma in situ of the cervix uteri.	GeneRIF	0.00875	129	0.0149	0.152	1	2	HLA-DQB1
Pubmed	23592992	Rab11-FIP1C and Rab14 direct plasma membrane sorting and particle incorporation of the HIV-1 envelope glycoprotein complex.	Pubmed	0.00875	129	0.0149	0.152	1	2	RAB11FIP1
Pubmed	20071715	Utility of treadmill testing in identification and genotype prediction in long-QT syndrome.	GeneRIF	0.00875	129	0.0149	0.152	1	2	KCNH2
Pubmed	11218373	HLA-DQ locus of the human leukocyte antigen complex and type 1 diabetes mellitus: a HuGE review.	GeneRIF	0.00875	129	0.0149	0.152	1	2	HLA-DQB1
Pubmed	21856036	Molecular mechanism and structural basis of interactions of dipeptidyl peptidase IV with adenosine deaminase and human immunodeficiency virus type-1 transcription transactivator.	GeneRIF	0.00875	129	0.0149	0.152	1	2	ADA
Pubmed	12163459	Adenosine kinase inhibition promotes survival of fetal adenosine deaminase-deficient thymocytes by blocking dATP accumulation.	GeneRIF	0.00875	129	0.0149	0.152	1	2	ADA
Pubmed	15562017	Relationship of estradiol and inhibin to the follicle-stimulating hormone variability in hypergonadotropic hypogonadism or premature ovarian failure.	GeneRIF	0.00875	129	0.0149	0.152	1	2	INHBB
Pubmed	18719072	Identification of the invariant chain (CD74) as an angiotensin AGTR1-interacting protein.	GeneRIF	0.00875	129	0.0149	0.152	1	2	CD74
Pubmed	16547003	BMP4 regulates pancreatic progenitor cell expansion through Id2.	GeneRIF	0.00875	129	0.0149	0.152	1	2	BMP4
Pubmed	19932102	O-GlcNAcylation enhances FOXO4 transcriptional regulation in response to stress.	GeneRIF	0.00875	129	0.0149	0.152	1	2	FOXO4
Pubmed	10075657	Interactions between neurogranin and calmodulin in vivo.	Pubmed	0.00875	129	0.0149	0.152	1	2	NRGN
Pubmed	24135912	Induction of high temperature requirement A1, a serine protease, by TGF-beta1 in articular chondrocytes of mouse models of OA.	Pubmed	0.00875	129	0.0149	0.152	1	2	HTRA1
Pubmed	7806273	The closely linked genes encoding the myeloid nuclear differentiation antigen (MNDA) and IFI16 exhibit contrasting haemopoietic expression.	Pubmed	0.00875	129	0.0149	0.152	1	2	IFI16
Pubmed	24899623	A motif in LILRB2 critical for Angptl2 binding and activation.	Pubmed	0.00875	129	0.0149	0.152	1	2	ANGPTL2
Pubmed	15471861	Transglutaminase 2 induces nuclear factor-kappaB activation via a novel pathway in BV-2 microglia.	GeneRIF	0.00875	129	0.0149	0.152	1	2	TGM2
Pubmed	11222741	Hormone selectivity in thyroid hormone receptors.	Pubmed	0.00875	129	0.0149	0.152	1	2	THRA
Pubmed	22295097	The 18 kDa translocator protein (peripheral benzodiazepine receptor) expression in the bone of normal, osteoprotegerin or low calcium diet treated mice.	GeneRIF	0.00875	129	0.0149	0.152	1	2	TSPO
Pubmed	22447857	The WNT inhibitor Dickkopf 1 and bone morphogenetic protein 4 rescue adipogenesis in hypertrophic obesity in humans.	GeneRIF	0.00875	129	0.0149	0.152	1	2	BMP4
Pubmed	7726898	Studies with MHC-deficient knock-out mice reveal impact of both MHC I- and MHC II-dependent T cell responses on Listeria monocytogenes infection.	Pubmed	0.00875	129	0.0149	0.152	1	2	HLA-DQB1
Pubmed	22563848	Hb A2/E levels found in co-inheritance with the a-thalassemia-1 - -(SEA)/type deletion and either Hb E or ß-thalassemia.	GeneRIF	0.00875	129	0.0149	0.152	1	2	HBA2
Pubmed	22572859	Activated innate immunity and the involvement of CX3CR1-fractalkine in promoting hematuria in patients with IgA nephropathy.	GeneRIF	0.00875	129	0.0149	0.152	1	2	CX3CL1
Pubmed	16449193	Salamander limb regeneration involves the activation of a multipotent skeletal muscle satellite cell population.	Pubmed	0.00875	129	0.0149	0.152	1	2	DERL3
Pubmed	21622534	Mutant thyroid hormone receptors (TRs) isolated from distinct cancer types display distinct target gene specificities: a unique regulatory repertoire associated with two renal clear cell carcinomas.	GeneRIF	0.00875	129	0.0149	0.152	1	2	THRA
Pubmed	18292516	Involvement of secretory and endosomal compartments in presentation of an exogenous self-glycolipid to type II NKT cells.	Pubmed	0.00875	129	0.0149	0.152	1	2	PSAP
Pubmed	15449022	HLA-DRB1 and -DQB1 alleles and gene polymorphisms of selected cytokines in systemic lupus erythematosus.	GeneRIF	0.00875	129	0.0149	0.152	1	2	HLA-DQB1
Pubmed	18280250	N-terminal pro A-type natriuretic peptide but not N-terminal pro C-type natriuretic peptide concentrations are related to cardiac diseases in infants.	GeneRIF	0.00875	129	0.0149	0.152	1	2	NPPA
Pubmed	6411350	Structure of the murine immune response I-A beta locus: sequence of the I-A beta gene and an adjacent beta-chain second domain exon.	Pubmed	0.00875	129	0.0149	0.152	1	2	HLA-DQB1
Pubmed	23542086	NT-proANP and NT-proBNP as prognostic markers in patients with acute decompensated heart failure of different etiologies.	Pubmed	0.00875	129	0.0149	0.152	1	2	NPPA
Pubmed	25372290	Impact of the superoxide dismutase 2 Val16Ala polymorphism on the relationship between valproic acid exposure and elevation of ¿-glutamyltransferase in patients with epilepsy: a population pharmacokinetic-pharmacodynamic analysis.	Pubmed	0.00875	129	0.0149	0.152	1	2	GGT1
Pubmed	12023345	Id2 negatively regulates B cell differentiation in the spleen.	Pubmed	0.00875	129	0.0149	0.152	1	2	CD74

Category	ID	Name	Source	p-value	q-value Bonferroni	q-value FDR B&H	q-value FDR B&Y	Hit Count in Query List	Hit Count in Genome	Hit in Query List
Pubmed	16670300	Further differentiation of murine double-positive thymocytes is inhibited in adenosine deaminase-deficient murine fetal thymic organ culture.	Pubmed	0.00875	129	0.0149	0.152	1	2	ADA
Pubmed	14690436	Two 14-3-3 binding motifs are required for stable association of Forkhead transcription factor FOXO4 with 14-3-3 proteins and inhibition of DNA binding.	GeneRIF	0.00875	129	0.0149	0.152	1	2	FOXO4
Pubmed	12530526	Carboxypeptidase E and thrombospondin-1 are differently expressed in subcutaneous and visceral fat of obese subjects.	GeneRIF	0.00875	129	0.0149	0.152	1	2	CPE
Pubmed	16092935	Why do Nogo/Nogo-66 receptor gene knockouts result in inferior regeneration compared to treatment with neutralizing agents?	GeneRIF	0.00875	129	0.0149	0.152	1	2	RTN4R
Pubmed	19131509	Distinct dysregulation of lipid metabolism by unliganded thyroid hormone receptor isoforms.	GeneRIF	0.00875	129	0.0149	0.152	1	2	THRA
Pubmed	21299850	Serotonin transporter (SERT) and translocator protein (TSPO) expression in the obese ob/ob mouse.	GeneRIF	0.00875	129	0.0149	0.152	1	2	TSPO
Pubmed	17672826	Atrial natriuretic peptide gene promoter polymorphism is associated with left ventricular hypertrophy in hypertension.	GeneRIF	0.00875	129	0.0149	0.152	1	2	NPPA
Pubmed	17114422	CCR4 is a key modulator of innate immune responses.	Pubmed	0.00875	129	0.0149	0.152	1	2	CD74
Pubmed	14525595	Development of a novel transgenic mouse for the study of interactions between CD4 and CD8 T cells during graft rejection.	Pubmed	0.00875	129	0.0149	0.152	1	2	HLA-DQB1
Pubmed	14555305	Thalassemia minor, the Gilbert mutation, and the risk of gallstones.	GeneRIF	0.00875	129	0.0149	0.152	1	2	HBB
Pubmed	19369458	Correlation of CX3CL1 and CX3CR1 levels with response to infliximab therapy in patients with rheumatoid arthritis.	GeneRIF	0.00875	129	0.0149	0.152	1	2	CX3CL1
Pubmed	19117368	Transfer of the shared epitope through microchimerism in women with rheumatoid arthritis.	GeneRIF	0.00875	129	0.0149	0.152	1	2	HLA-DQB1
Pubmed	22653970	Long QT2 mutation on the Kv11.1 ion channel inhibits current activity by ablating a protein kinase Ca consensus site.	GeneRIF	0.00875	129	0.0149	0.152	1	2	KCNH2
Pubmed	17634957	Induction of potent CD4+ T cell-mediated antitumor responses by a helper HER-2/neu peptide linked to the Ii-Key moiety of the invariant chain.	GeneRIF	0.00875	129	0.0149	0.152	1	2	CD74
Pubmed	17144392	Polymorphism of HLA-DR and HLA-DQ in rheumatoid arthritis patients and clinical response to methotrexate--a hospital-based study.	GeneRIF	0.00875	129	0.0149	0.152	1	2	HLA-DQB1
Pubmed	12849668	Modulating effects of age and gender on the clinical course of long QT syndrome by genotype.	GeneRIF	0.00875	129	0.0149	0.152	1	2	KCNH2
Pubmed	23776208	MIF intersubunit disulfide mutant antagonist supports activation of CD74 by endogenous MIF trimer at physiologic concentrations.	GeneRIF	0.00875	129	0.0149	0.152	1	2	CD74
Pubmed	10712278	Isometric force and endurance in soleus muscle of thyroid hormone receptor-alpha(1)- or -beta-deficient mice.	Pubmed	0.00875	129	0.0149	0.152	1	2	THRA
Pubmed	24331839	Immunohistochemical study of 36 cases of pulmonary sarcomatoid carcinoma--sensitivity of TTF-1 is superior to napsin.	Pubmed	0.00875	129	0.0149	0.152	1	2	NAPSA
Pubmed	20443243	[Association analysis between candidate genes for multiple sclerosis and brain MRI parameters].	GeneRIF	0.00875	129	0.0149	0.152	1	2	HLA-DQB1
Pubmed	19407813	NRH2 is a trafficking switch to regulate sortilin localization and permit proneurotrophin-induced cell death.	GeneRIF	0.00875	129	0.0149	0.152	1	2	NGFR
Pubmed	19120276	From Type 1, through LADA, to type 2 diabetes: a continuous spectrum?	GeneRIF	0.00875	129	0.0149	0.152	1	2	HLA-DQB1
Pubmed	8413665	The structure of crystalline profilin-beta-actin.	Pubmed	0.00875	129	0.0149	0.152	1	2	ACTA1
Pubmed	23576602	ProBDNF and its receptors are upregulated in glioma and inhibit the growth of glioma cells in vitro.	Pubmed	0.00875	129	0.0149	0.152	1	2	NGFR
Pubmed	18481277	Prosaposin is an AR-target gene and its neurotrophic domain upregulates AR expression and activity in prostate stromal cells.	GeneRIF	0.00875	129	0.0149	0.152	1	2	PSAP
Pubmed	18392554	Early modification of sickle cell disease clinical course by UDP-glucuronosyltransferase 1A1 gene promoter polymorphism.	GeneRIF	0.00875	129	0.0149	0.152	1	2	HBB
Pubmed	12963025	RanBPM is a novel binding protein for p75NTR.	GeneRIF	0.00875	129	0.0149	0.152	1	2	NGFR
Pubmed	1429635	Identification by site-directed mutagenesis of two lysine residues in cholesterol side chain cleavage cytochrome P450 that are essential for adrenodoxin binding.	Pubmed	0.00875	129	0.0149	0.152	1	2	CYP11A1
Pubmed	23385585	Doublecortin-like kinase enhances dendritic remodelling and negatively regulates synapse maturation.	GeneRIF	0.00875	129	0.0149	0.152	1	2	DCLK1
Pubmed	15851575	Association of HLA class I and class II polymorphisms with age-related macular degeneration.	GeneRIF	0.00875	129	0.0149	0.152	1	2	HLA-DQB1
Pubmed	24100137	Inhibitor selectivity between aldo-keto reductase superfamily members AKR1B10 and AKR1B1: role of Trp112 (Trp111).	Pubmed	0.00875	129	0.0149	0.152	1	2	AKR1B10
Pubmed	17356047	Dysregulation of placental endothelial lipase and lipoprotein lipase in intrauterine growth-restricted pregnancies.	GeneRIF	0.00875	129	0.0149	0.152	1	2	LIPG
Pubmed	10945474	Genomic construct and mapping of the gene for CMAP (leukocystatin/cystatin F, CST7) and identification of a proximal novel gene, BSCv (C20orf3).	Pubmed	0.00875	129	0.0149	0.152	1	2	APMAP
Pubmed	12397372	Cellular distribution and contribution of cyclooxygenase COX-2 to diabetogenesis in NOD mouse.	GeneRIF	0.00875	129	0.0149	0.152	1	2	CD68
Pubmed	19906950	ANP-mediated inhibition of distal nephron fractional sodium reabsorption in wild-type and mice overexpressing natriuretic peptide receptor.	GeneRIF	0.00875	129	0.0149	0.152	1	2	NPPA
Pubmed	11476906	Heat-shock protein 70-1 and HLA class II gene polymorphisms associated with celiac disease susceptibility in Navarra (Spain).	GeneRIF	0.00875	129	0.0149	0.152	1	2	HLA-DQB1

Category	ID	Name	Source	p-value	q-value Bonferroni	q-value FDR B&H	q-value FDR B&Y	Hit Count in Query List	Hit Count in Genome	Hit in Query List
Pubmed	20680101	NGF and NGF-receptor expression of cultured immortalized human corneal endothelial cells.	GeneRIF	0.00875	129	0.0149	0.152	1	2	NGFR
Pubmed	8647860	Proteolytic activity of human osteoclast cathepsin K. Expression, purification, activation, and substrate identification.	Pubmed	0.00875	129	0.0149	0.152	1	2	CTSK
Pubmed	9787139	Reversal of lethal alpha- and beta-thalassemias in mice by expression of human embryonic globins.	Pubmed	0.00875	129	0.0149	0.152	1	2	HBA2
Pubmed	23152410	IGF-I receptor phosphorylation is impaired in cathepsin X-deficient prostate cancer cells.	GeneRIF	0.00875	129	0.0149	0.152	1	2	CTSK
Pubmed	11165491	Bambi is coexpressed with Bmp-4 during mouse embryogenesis.	Pubmed	0.00875	129	0.0149	0.152	1	2	BMP4
Pubmed	16122986	Molecular genetic studies on the compatibility of HLA class II alleles in patients with unexplained recurrent miscarriage in the Japanese population.	GeneRIF	0.00875	129	0.0149	0.152	1	2	HLA-DQB1
Pubmed	15034572	Antisickling effects of an endogenous human alpha-like globin.	Pubmed	0.00875	129	0.0149	0.152	1	2	HBA2
Pubmed	24683185	HLA-DRa1 constructs block CD74 expression and MIF effects in experimental autoimmune encephalomyelitis.	Pubmed	0.00875	129	0.0149	0.152	1	2	CD74
Pubmed	25426952	carboxypeptidase E-¿N, a neuroprotein transiently expressed during development protects embryonic neurons against glutamate neurotoxicity.	Pubmed	0.00875	129	0.0149	0.152	1	2	CPE
Pubmed	11454903	Adenosine-mediated mast cell degranulation in adenosine deaminase-deficient mice.	Pubmed	0.00875	129	0.0149	0.152	1	2	ADA
Pubmed	23322532	Overexpression of nerve growth factor by murine smooth muscle cells: role of the p75 neurotrophin receptor on sympathetic and sensory sprouting.	Pubmed	0.00875	129	0.0149	0.152	1	2	NGFR
Pubmed	17922479	Understanding the accuracy of statistical haplotype inference with sequence data of known phase.	Pubmed	0.00875	129	0.0149	0.152	1	2	KLK14
Pubmed	3620470	The beta-delta crossover leading to the beta delta hybrid gene of hemoglobin P-Nilotic is located within 54 base-pairs of the 5' end of exon 2 or between codons 31 and 50.	Pubmed	0.00875	129	0.0149	0.152	1	2	HBB
Pubmed	8916035	Unexpected behavior of a gene trap vector comprising a fusion between the Sh ble and the lacZ genes.	Pubmed	0.00875	129	0.0149	0.152	1	2	AKAP12
Pubmed	21557918	Abnormal expression of uncoupling protein-2 correlates with CYP11A1 expression in polycystic ovary syndrome.	GeneRIF	0.00875	129	0.0149	0.152	1	2	CYP11A1
Pubmed	17919266	Islet autoantibodies are associated with HLA-DQ genotypes in Han Chinese patients with type 1 diabetes and their relatives.	GeneRIF	0.00875	129	0.0149	0.152	1	2	HLA-DQB1
Pubmed	17718385	[Characterization of the HtrA family of proteins].	GeneRIF	0.00875	129	0.0149	0.152	1	2	HTRA1
Pubmed	16201295	Association of HLA-DRB1, DQB1 alleles with chronic urticaria.	GeneRIF	0.00875	129	0.0149	0.152	1	2	HLA-DQB1
Pubmed	11672428	Unfolding and refolding of a quinone oxidoreductase: alpha-crystallin, a molecular chaperone, assists its reactivation.	Pubmed	0.00875	129	0.0149	0.152	1	2	CRYAA
Pubmed	18722178	Genome-wide analysis of the H3K4 histone demethylase RBP2 reveals a transcriptional program controlling differentiation.	GeneRIF	0.00875	129	0.0149	0.152	1	2	KDM5A
Pubmed	8593698	Preferential differentiation of P19 mouse embryonal carcinoma cells into smooth muscle cells. Use of retinoic acid and antisense against the central nervous system-specific POU transcription factor Brn-2.	Pubmed	0.00875	129	0.0149	0.152	1	2	MYH11
Pubmed	19535340	Crystal structures of human SIRT3 displaying substrate-induced conformational changes.	GeneRIF	0.00875	129	0.0149	0.152	1	2	ACSS1
Pubmed	12169435	Thyroid hormone and cardiac function in mice deficient in thyroid hormone receptor-alpha or -beta: an echocardiograph study.	Pubmed	0.00875	129	0.0149	0.152	1	2	THRA
Pubmed	25157096	Proinsulin-specific, HLA-DQ8, and HLA-DQ8-transdimer-restricted CD4+ T cells infiltrate islets in type 1 diabetes.	Pubmed	0.00875	129	0.0149	0.152	1	2	HLA-DQB1
Pubmed	10473614	Molecular cloning, expression, and characterization of angiopoietin-related protein. angiopoietin-related protein induces endothelial cell sprouting.	Pubmed	0.00875	129	0.0149	0.152	1	2	ANGPTL2
Pubmed	21315773	Human T-cell leukemia virus type 1 tax transactivates the matrix metalloproteinase 7 gene via JunD/AP-1 signaling.	GeneRIF	0.00875	129	0.0149	0.152	1	2	MMP7
Pubmed	1316918	Functional nerve growth factor receptors on human B lymphocytes. Interaction with IL-2.	Pubmed	0.00875	129	0.0149	0.152	1	2	NGFR
Pubmed	18774995	Annual screening detects celiac disease in children with type 1 diabetes.	GeneRIF	0.00875	129	0.0149	0.152	1	2	HLA-DQB1
Pubmed	20610657	FIP1/RCP binding to Golgin-97 regulates retrograde transport from recycling endosomes to the trans-Golgi network.	GeneRIF	0.00875	129	0.0149	0.152	1	2	RAB11FIP1
Pubmed	18165224	p204 protein is a novel modulator of ras activity.	GeneRIF	0.00875	129	0.0149	0.152	1	2	IFI16
Pubmed	11972882	HLA-DQA1 and -DQB1 genes and Helicobacter pylori infection in Italian patients with gastric adenocarcinoma.	GeneRIF	0.00875	129	0.0149	0.152	1	2	HLA-DQB1
Pubmed	24553827	Branched-chain amino acids inhibit the TGF-beta-induced down-regulation of taurine biosynthetic enzyme cysteine dioxygenase in HepG2 cells.	Pubmed	0.00875	129	0.0149	0.152	1	2	CDO1
Pubmed	22982407	The combined effect of acetylation and glycation on the chaperone and anti-apoptotic functions of human a-crystallin.	GeneRIF	0.00875	129	0.0149	0.152	1	2	CRYAA
Pubmed	21999204	Mesothelin enhances invasion of ovarian cancer by inducing MMP-7 through MAPK/ERK and JNK pathways.	GeneRIF	0.00875	129	0.0149	0.152	1	2	MMP7
Pubmed	23613523	TCR triggering modulates the responsiveness and homeostatic proliferation of CD4+ thymic emigrants to IL-7 therapy.	Pubmed	0.00875	129	0.0149	0.152	1	2	HLA-DQB1
Pubmed	24058627	Enhanced cardiac function in Gravin mutant mice involves alterations in the ß-adrenergic receptor signaling cascade.	Pubmed	0.00875	129	0.0149	0.152	1	2	AKAP12
Pubmed	19931242	The interaction of angiocidin with tissue transglutaminase.	GeneRIF	0.00875	129	0.0149	0.152	1	2	TGM2
Pubmed	20943663	Chronic and acute models of retinal neurodegeneration TrkA activity are neuroprotective whereas p75NTR activity is neurotoxic through a paracrine mechanism.	GeneRIF	0.00875	129	0.0149	0.152	1	2	NGFR

Category	ID	Name	Source	p-value	q-value Bonferroni	q-value FDR B&H	q-value FDR B&Y	Hit Count in Query List	Hit Count in Genome	Hit in Query List
Pubmed	17257313	Insulin gene VNTR, CTLA-4 +49A/G and HLA-DQB1 alleles distinguish latent autoimmune diabetes in adults from type 1 diabetes and from type 2 diabetes group.	GeneRIF	0.00875	129	0.0149	0.152	1	2	HLA-DQB1
Pubmed	7532679	Breakdown of B cell tolerance in a mouse model o systemic lupus erythematosus.	Pubmed	0.00875	129	0.0149	0.152	1	2	ASB2
Pubmed	23065739	Serum CA19-9, cathepsin D, and matrix metalloproteinase-7 as a diagnostic panel for pancreatic ductal adenocarcinoma.	GeneRIF	0.00875	129	0.0149	0.152	1	2	MMP7
Pubmed	16873714	Impaired endothelium-derived hyperpolarizing factor-mediated dilations and increased blood pressure in mice deficient of the intermediate-conductance Ca2+-activated K+ channel.	Pubmed	0.00875	129	0.0149	0.152	1	2	KCNN4
Pubmed	10215625	Schwann cell hyperplasia and tumors in transgenic mice expressing a naturally occurring mutant NF2 protein.	Pubmed	0.00875	129	0.0149	0.152	1	2	NGFR
Pubmed	22147846	Urinary monocyte chemoattractant protein 1 and alpha 1 acid glycoprotein as biomarkers of renal disease activity in juvenile-onset systemic lupus erythematosus.	GeneRIF	0.00875	129	0.0149	0.152	1	2	ORM1
Pubmed	25007147	A novel CIC-FOXO4 gene fusion in undifferentiated small round cell sarcoma: a genetically distinct variant of Ewing-like sarcoma.	Pubmed	0.00875	129	0.0149	0.152	1	2	FOXO4
Pubmed	11731612	TRs have common and isoform-specific functions in regulation of the cardiac myosin heavy chain genes.	Pubmed	0.00875	129	0.0149	0.152	1	2	THRA
Pubmed	18065200	The MHC type 1 diabetes susceptibility gene is centromeric to HLA-DQB1.	GeneRIF	0.00875	129	0.0149	0.152	1	2	HLA-DQB1
Pubmed	18785031	Common variation in NOS1AP and KCNH2 genes and QT interval duration in young adults. The Cardiovascular Risk in Young Finns Study.	GeneRIF	0.00875	129	0.0149	0.152	1	2	KCNH2
Pubmed	11679633	Identification of ubiquitin ligases required for skeletal muscle atrophy.	Pubmed	0.00875	129	0.0149	0.152	1	2	FBXO32
Pubmed	9824491	The Ii41 isoform of invariant chain mediates both positive and negative selection events in T-cell receptor transgenic mice.	Pubmed	0.00875	129	0.0149	0.152	1	2	CD74
Pubmed	3474517	Interaction between two different regulatory elements activates the murine alpha A-crystallin gene promoter in explanted lens epithelia.	Pubmed	0.00875	129	0.0149	0.152	1	2	CRYAA
Pubmed	19204159	Modification of Multiple Sclerosis Phenotypes by African Ancestry at HLA.	GeneRIF	0.00875	129	0.0149	0.152	1	2	HLA-DQB1
Pubmed	12484513	Plasma levels of adrenomedullin and atrial and brain natriuretic peptides in the general population: their relations to age and pulse pressure.	GeneRIF	0.00875	129	0.0149	0.152	1	2	NPPA
Pubmed	7030925	Polymorphism of the mouse H-2 loci.	Pubmed	0.00875	129	0.0149	0.152	1	2	HLA-DQB1
Pubmed	11689711	Regulation of the Forkhead transcription factor AFX by Ral-dependent phosphorylation of threonines 447 and 451.	Pubmed	0.00875	129	0.0149	0.152	1	2	FOXO4
Pubmed	24012852	Autonomous functions of murine thyroid hormone receptor TRa and TRß in cochlear hair cells.	Pubmed	0.00875	129	0.0149	0.152	1	2	THRA
Pubmed	15086490	Significance of the tissue kallikrein promoter and transforming growth factor-beta1 polymorphisms with renal progression in children with vesicoureteral reflux.	GeneRIF	0.00875	129	0.0149	0.152	1	2	KLK1
Pubmed	18799722	CX3CL1/fractalkine is released from apoptotic lymphocytes to stimulate macrophage chemotaxis.	GeneRIF	0.00875	129	0.0149	0.152	1	2	CX3CL1
Pubmed	18499961	[Polymorphism in CYP11alpha and CYP17 genes and the etiology of hyperandrogenism in patients with polycystic ovary syndrome].	GeneRIF	0.00875	129	0.0149	0.152	1	2	CYP11A1
Pubmed	22184118	Type 1 diabetes-associated HLA-DQ8 transdimer accommodates a unique peptide repertoire.	GeneRIF	0.00875	129	0.0149	0.152	1	2	HLA-DQB1
Pubmed	23344661	Integrins a4 and aM, collagen1A1, and matrix metalloproteinase 7 are upregulated in acute Kawasaki disease vasculopathy.	GeneRIF	0.00875	129	0.0149	0.152	1	2	MMP7
Pubmed	22504665	microRNA-145 in Barrett's oesophagus: regulating BMP4 signalling via GATA6.	GeneRIF	0.00875	129	0.0149	0.152	1	2	BMP4
Pubmed	20080707	EGF potentiated oncogenesis requires a tissue transglutaminase-dependent signaling pathway leading to Src activation.	GeneRIF	0.00875	129	0.0149	0.152	1	2	TGM2
Pubmed	17002687	The CX3CL1-CX3CR1 system and psoriasis.	GeneRIF	0.00875	129	0.0149	0.152	1	2	CX3CL1
Pubmed	10192392	Interacting loci cause severe iris atrophy and glaucoma in DBA/2J mice.	Pubmed	0.00875	129	0.0149	0.152	1	2	GPNMB
Pubmed	7691725	Differential regulation of B7 mRNA in enterocytes and lymphoid cells.	Pubmed	0.00875	129	0.0149	0.152	1	2	HLA-DQB1
Pubmed	22101769	A mechanism for glycoconjugate vaccine activation of the adaptive immune system and its implications for vaccine design.	Pubmed	0.00875	129	0.0149	0.152	1	2	HLA-DQB1
Pubmed	8850569	Fibulin-1 and fibulin-2 expression during organogenesis in the developing mouse embryo.	Pubmed	0.00875	129	0.0149	0.152	1	2	FBLN2
Pubmed	23303826	Human cytomegalovirus chemokine receptor US28 induces migration of cells on a CX3CL1-presenting surface.	GeneRIF	0.00875	129	0.0149	0.152	1	2	CX3CL1
Pubmed	16316347	Constriction velocities of renal afferent and efferent arterioles of mice are not related to SMB expression.	GeneRIF	0.00875	129	0.0149	0.152	1	2	MYH11
Pubmed	18036860	Pemphigus and HLA in Morocco.	GeneRIF	0.00875	129	0.0149	0.152	1	2	HLA-DQB1
Pubmed	16565492	Kupffer cell-dependent hepatitis occurs during influenza infection.	Pubmed	0.00875	129	0.0149	0.152	1	2	HLA-DQB1
Pubmed	22322976	Thyroid hormone receptor-a and vascular function.	GeneRIF	0.00875	129	0.0149	0.152	1	2	THRA
Pubmed	24190996	TAp73 knockout mice show morphological and functional nervous system defects associated with loss of p75 neurotrophin receptor.	Pubmed	0.00875	129	0.0149	0.152	1	2	NGFR
Pubmed	20301720	Celiac Disease	Pubmed	0.00875	129	0.0149	0.152	1	2	HLA-DQB1
Pubmed	20830690	The utility of napsin-A in the identification of primary and metastatic lung adenocarcinoma among cytologically poorly differentiated carcinomas.	GeneRIF	0.00875	129	0.0149	0.152	1	2	NAPSA
Pubmed	16195464	Experience-driven plasticity of visual cortex limited by myelin and Nogo receptor.	GeneRIF	0.00875	129	0.0149	0.152	1	2	RTN4R
Pubmed	12048180	FOXO forkhead transcription factors induce G(2)-M checkpoint in response to oxidative stress.	GeneRIF	0.00875	129	0.0149	0.152	1	2	FOXO4

Category	ID	Name	Source	p-value	q-value Bonferroni	q-value FDR B&H	q-value FDR B&Y	Hit Count in Query List	Hit Count in Genome	Hit in Query List
Pubmed	282635	Comparison of cloned mouse alpha- and beta-globin genes: conservation of intervening sequence locations and extragenic homology.	Pubmed	0.00875	129	0.0149	0.152	1	2	HBA2
Pubmed	17203524	An algorithm for family screening for coeliac disease.	GeneRIF	0.00875	129	0.0149	0.152	1	2	HLA-DQB1
Pubmed	24040265	Developmental expression of translocator protein/peripheral benzodiazepine receptor in reproductive tissues.	Pubmed	0.00875	129	0.0149	0.152	1	2	TSPO
Pubmed	24021552	The role of CAV3 in long-QT syndrome: clinical and functional assessment of a caveolin-3/Kv11.1 double heterozygote versus caveolin-3 single heterozygote.	Pubmed	0.00875	129	0.0149	0.152	1	2	KCNH2
Pubmed	24260329	Translocator protein 18 kDa (TSPO) is regulated in white and brown adipose tissue by obesity.	Pubmed	0.00875	129	0.0149	0.152	1	2	TSPO
Pubmed	12113294	Serum adenosine deaminase and cytidine deaminase activities in patients with systemic lupus erythematosus.	GeneRIF	0.00875	129	0.0149	0.152	1	2	ADA
Pubmed	9143502	Structure and chromosomal assignment of the human cathepsin K gene.	Pubmed	0.00875	129	0.0149	0.152	1	2	CTSK
Pubmed	17227789	The common non-synonymous variant G38S of the KCNE1-(minK)-gene is not associated to QT interval in Central European Caucasians: results from the KORA study.	GeneRIF	0.00875	129	0.0149	0.152	1	2	KCNH2
Pubmed	24648278	Bone morphogenetic protein-2 antagonizes bone morphogenetic protein-4 induced cardiomyocyte hypertrophy and apoptosis.	Pubmed	0.00875	129	0.0149	0.152	1	2	BMP4
Pubmed	19494329	Enhanced airway inflammation and remodeling in adenosine deaminase-deficient mice lacking the A2B adenosine receptor.	GeneRIF	0.00875	129	0.0149	0.152	1	2	ADA
Pubmed	18627302	Quantitative RT-PCR analysis and immunohistochemical localization of the kallikrein related peptidases 13 and 14 in lung.	GeneRIF	0.00875	129	0.0149	0.152	1	2	KLK14
Pubmed	20039448	Atrial natriuretic peptide signal pathway upregulated in stomach of streptozotocin-induced diabetic mice.	GeneRIF	0.00875	129	0.0149	0.152	1	2	NPPA
Pubmed	21527754	Amyloid beta enhances migration of endothelial progenitor cells by upregulating CX3CR1 in response to fractalkine, which may be associated with development of choroidal neovascularization.	GeneRIF	0.00875	129	0.0149	0.152	1	2	CX3CL1
Pubmed	25681747	Potentially therapeutic levels of anti-sickling globin gene expression following lentivirus-mediated gene transfer in sickle cell disease bone marrow CD34+ cells.	Pubmed	0.00875	129	0.0149	0.152	1	2	HBB
Pubmed	12356724	Retardation of post-natal development caused by a negatively acting thyroid hormone receptor alpha1.	GeneRIF	0.00875	129	0.0149	0.152	1	2	THRA
Pubmed	10922377	The mixed lineage kinase DLK is oligomerized by tissue transglutaminase during apoptosis.	Pubmed	0.00875	129	0.0149	0.152	1	2	TGM2
Pubmed	11207297	Organ-specific regulation of the CD8 T cell response to Listeria monocytogenes infection.	Pubmed	0.00875	129	0.0149	0.152	1	2	HLA-DQB1
Pubmed	21475087	Immunolabeling of the inhibin-ßA and -ßB subunit in normal and malignant human cervical tissue and cervical cancer cell lines.	GeneRIF	0.00875	129	0.0149	0.152	1	2	INHBB
Pubmed	17488972	Thyroid status during skeletal development determines adult bone structure and mineralization.	Pubmed	0.00875	129	0.0149	0.152	1	2	THRA
Pubmed	15155685	Secretory leucoprotease inhibitor impairs Toll-like receptor 2- and 4-mediated responses in monocytic cells.	GeneRIF	0.00875	129	0.0149	0.152	1	2	SLPI
Pubmed	23402837	Association of HLA-DR-DQ polymorphisms with diabetes in Tunisian patients.	Pubmed	0.00875	129	0.0149	0.152	1	2	HLA-DQB1
Pubmed	728996	The isolation and characterization of linked delta- and beta-globin genes from a cloned library of human DNA.	Pubmed	0.00875	129	0.0149	0.152	1	2	HBB
Pubmed	15663743	New strategies for efficient typing of HLA class-II loci DQB1 and DRB1 by using Pyrosequencing.	GeneRIF	0.00875	129	0.0149	0.152	1	2	HLA-DQB1
Pubmed	17196528	Structural and mechanistic insights into nerve growth factor interactions with the TrkA and p75 receptors.	GeneRIF	0.00875	129	0.0149	0.152	1	2	NGFR
Pubmed	21546901	A novel role of the CX3CR1/CX3CL1 system in the cross-talk between chronic lymphocytic leukemia cells and tumor microenvironment.	GeneRIF	0.00875	129	0.0149	0.152	1	2	CX3CL1
Pubmed	11558903	Identification of sequence polymorphisms in two sulfation-related genes, PAPSS2 and SLC26A2, and an association analysis with knee osteoarthritis.	GeneRIF	0.00875	129	0.0149	0.152	1	2	PAPSS2
Pubmed	18400943	Cross-talk between fibroblast growth factor and bone morphogenetic proteins regulates gap junction-mediated intercellular communication in lens cells.	GeneRIF	0.00875	129	0.0149	0.152	1	2	BMP4
Pubmed	22442151	Transglutaminase-2 interaction with heparin: identification of a heparin binding site that regulates cell adhesion to fibronectin-transglutaminase-2 matrix.	GeneRIF	0.00875	129	0.0149	0.152	1	2	TGM2
Pubmed	21187079	Roles of rat and human aldo-keto reductases in metabolism of farnesol and geranylgeraniol.	Pubmed	0.00875	129	0.0149	0.152	1	2	AKR1B10
Pubmed	22458929	Expression TGM2 and BNIP3 have prognostic significance in laryngeal cancer patients receiving surgery and postoperative radiotherapy: a retrospective study.	GeneRIF	0.00875	129	0.0149	0.152	1	2	TGM2
Pubmed	23897050	Role of ADAM-17, p38 MAPK, cathepsins, and the proteasome pathway in the synthesis and shedding of fractalkine/CX3 CL1 in rheumatoid arthritis.	Pubmed	0.00875	129	0.0149	0.152	1	2	CX3CL1
Pubmed	17200117	Src docks to A-kinase anchoring protein gravin, regulating beta2-adrenergic receptor resensitization and recycling.	GeneRIF	0.00875	129	0.0149	0.152	1	2	AKAP12
Pubmed	12964026	Negative regulation of forkhead transcription factor AFX (Foxo4) by CBP-induced acetylation.	GeneRIF	0.00875	129	0.0149	0.152	1	2	FOXO4
Pubmed	16542748	Re-routing of the invariant chain to the direct sorting pathway by introduction of an AP3-binding motif from LIMP II.	GeneRIF	0.00875	129	0.0149	0.152	1	2	CD74
Pubmed	19491194	Inhibin betaB expression in murine adipose tissue and its regulation by leptin, insulin and dexamethasone.	Pubmed	0.00875	129	0.0149	0.152	1	2	INHBB

Category	ID	Name	Source	p-value	q-value Bonferroni	q-value FDR B&H	q-value FDR B&Y	Hit Count in Query List	Hit Count in Genome	Hit in Query List
Pubmed	8663040	Metabolic and immunologic consequences of limited adenosine deaminase expression in mice.	Pubmed	0.00875	129	0.0149	0.152	1	2	ADA
Pubmed	21750716	Identification of the chemokine CX3CL1 as a new regulator of malignant cell proliferation in epithelial ovarian cancer.	GeneRIF	0.00875	129	0.0149	0.152	1	2	CX3CL1
Pubmed	12270926	Deficiencies in pro-thyrotropin-releasing hormone processing and abnormalities in thermoregulation in Cpefat/fat mice.	GeneRIF	0.00875	129	0.0149	0.152	1	2	CPE
Pubmed	15537790	Human EDEM2, a novel homolog of family 47 glycosidases, is involved in ER-associated degradation of glycoproteins.	GeneRIF	0.00875	129	0.0149	0.152	1	2	EDEM2
Pubmed	17344279	HLA related genetic risk for coeliac disease.	GeneRIF	0.00875	129	0.0149	0.152	1	2	HLA-DQB1
Pubmed	24939415	Involvement of macrophage migration inhibitory factor and its receptor (CD74) in human breast cancer.	Pubmed	0.00875	129	0.0149	0.152	1	2	CD74
Pubmed	17875181	HLA-DQB1 0602 allele is associated with splenomegaly in Japanese sarcoidosis.	GeneRIF	0.00875	129	0.0149	0.152	1	2	HLA-DQB1
Pubmed	11159543	Expression, purification, and characterization of human hemoglobins Gower-1 (zeta(2)epsilon(2)), Gower-2 (alpha(2)epsilon(2)), and Portland-2 (zeta(2)beta(2)) assembled in complex transgenic-knockout mice.	Pubmed	0.00875	129	0.0149	0.152	1	2	HBB
Pubmed	16420246	HLA class II haplotype DRB1*04-DQB1*0301 contributes to risk of familial generalized vitiligo and early disease onset.	GeneRIF	0.00875	129	0.0149	0.152	1	2	HLA-DQB1
Pubmed	18502673	Lack of a significant role of P-Rex1, a major regulator of macrophage Rac1 activation and chemotaxis, in atherogenesis.	GeneRIF	0.00875	129	0.0149	0.152	1	2	PREX1
Pubmed	8195005	Hb Kurdistan [alpha 47(CE5)Asp-->Tyr], a new alpha chain variant in combination with beta (0)-thalassemia.	Pubmed	0.00875	129	0.0149	0.152	1	2	HBA2
Pubmed	11024583	Genetic epidemiology of type 1 diabetes mellitus in Taiwan.	GeneRIF	0.00875	129	0.0149	0.152	1	2	HLA-DQB1
Pubmed	7550311	Lethal alpha-thalassaemia created by gene targeting in mice and its genetic rescue.	Pubmed	0.00875	129	0.0149	0.152	1	2	HBA2
Pubmed	20482314	Tissue kallikrein promotes prostate cancer cell migration and invasion via a protease-activated receptor-1-dependent signaling pathway.	GeneRIF	0.00875	129	0.0149	0.152	1	2	KLK1
Pubmed	12591179	Thyroid hormone receptors: lessons from knockout and knock-in mutant mice.	GeneRIF	0.00875	129	0.0149	0.152	1	2	THRA
Pubmed	9832551	Nexilin: a novel actin filament-binding protein localized at cell-matrix adherens junction.	Pubmed	0.00875	129	0.0149	0.152	1	2	ACTA1
Pubmed	20709014	Regulation of cell proliferation and apoptosis through fibrocystin-prosaposin interaction.	Pubmed	0.00875	129	0.0149	0.152	1	2	PSAP
Pubmed	24158116	Three polymorphisms in promoter of protein C gene with endothelial protein c receptor gene and risk of venous thrombosis.	Pubmed	0.00875	129	0.0149	0.152	1	2	PROCR
Pubmed	20140183	Genetic and functional dissection of HTRA1 and LOC387715 in age-related macular degeneration.	GeneRIF	0.00875	129	0.0149	0.152	1	2	HTRA1
Pubmed	18988857	PirB is a functional receptor for myelin inhibitors of axonal regeneration.	GeneRIF	0.00875	129	0.0149	0.152	1	2	NGFR
Pubmed	21991388	STAT3 knockdown reduces pancreatic cancer cell invasiveness and matrix metalloproteinase-7 expression in nude mice.	GeneRIF	0.00875	129	0.0149	0.152	1	2	MMP7
Pubmed	21555577	CD4+ T cells support cytotoxic T lymphocyte priming by controlling lymph node input.	Pubmed	0.00875	129	0.0149	0.152	1	2	CD74
Pubmed	3032738	Polymorphism and gene conversion in mouse alpha-globin haplotypes.	Pubmed	0.00875	129	0.0149	0.152	1	2	HBA2
Pubmed	20080971	Distal histidine stabilizes bound O2 and acts as a gate for ligand entry in both subunits of adult human hemoglobin.	GeneRIF	0.00875	129	0.0149	0.152	1	2	HBB
Pubmed	20833134	Activation of the unfolded protein response by a cataract-associated aA-crystallin mutation.	GeneRIF	0.00875	129	0.0149	0.152	1	2	CRYAA
Pubmed	19389844	Plasma gelsolin and circulating actin correlate with hemodialysis mortality.	GeneRIF	0.00875	129	0.0149	0.152	1	2	ACTA1
Pubmed	11408267	Neural change in Trichinella-infected mice is MHC II independent and involves M-CSF-derived macrophages.	Pubmed	0.00875	129	0.0149	0.152	1	2	HLA-DQB1
Pubmed	15361546	Viral macrophage inflammatory protein-II and fractalkine (CX3CL1) chimeras identify molecular determinants of affinity, efficacy, and selectivity at CX3CR1.	GeneRIF	0.00875	129	0.0149	0.152	1	2	CX3CL1
Pubmed	19549593	Novel non-genomic signaling of thyroid hormone receptors in thyroid carcinogenesis.	Pubmed	0.00875	129	0.0149	0.152	1	2	THRA
Pubmed	10581178	Functional genomic analysis reveals the utility of the I/LWEQ module as a predictor of protein:actin interaction.	Pubmed	0.00875	129	0.0149	0.152	1	2	ACTA1
Pubmed	19074353	Regulation of dendritic cell migration by CD74, the MHC class II-associated invariant chain.	GeneRIF	0.00875	129	0.0149	0.152	1	2	CD74
Pubmed	9671738	A member of a family of sulfate-activating enzymes causes murine brachymorphism.	Pubmed	0.00875	129	0.0149	0.152	1	2	PAPSS2
Pubmed	18424556	Deletion of G protein-coupled receptor 48 leads to ocular anterior segment dysgenesis (ASD) through down-regulation of Pitx2.	GeneRIF	0.00921	136	0.0157	0.16	2	33	CRYAA,BMP4
Pubmed	22703881	Genetic associations for activated partial thromboplastin time and prothrombin time, their gene expression profiles, and risk of coronary artery disease.	Pubmed	0.00921	136	0.0157	0.16	2	33	PROCR,EDEM2
Pubmed	25064009	Large-scale meta-analysis of genome-wide association data identifies six new risk loci for Parkinson's disease.	Pubmed	0.00921	136	0.0157	0.16	2	33	HLA-DQB1,GPNMB
Pubmed	21738480	Multiple loci are associated with white blood cell phenotypes.	Pubmed	0.00921	136	0.0157	0.16	2	33	CDSN,THRA
Pubmed	19834535	Sequential use of transcriptional profiling, expression quantitative trait mapping, and gene association implicates MMP20 in human kidney aging.	GeneRIF	0.00951	141	0.0162	0.165	3	99	FBLN2,GPNMB,MMP7
Pubmed	20585627	Web-based, participant-driven studies yield novel genetic associations for common traits.	GeneRIF	0.00976	144	0.0166	0.169	2	34	TCHH,TCHHL1
Pubmed	19238444	The genomic basis of cerebral palsy: a HuGE systematic literature review.	GeneRIF	0.00976	144	0.0166	0.169	2	34	NPPA,PROCR
Pubmed	25505242	Microtubule-associated proteins 1 (MAP1) promote human immunodeficiency virus type I (HIV-1) intracytoplasmic routing to the nucleus.	Pubmed	0.00976	144	0.0166	0.169	2	34	ANKRD35,TSPAN13
Pubmed	21800051	The interaction between ubiquitin C-terminal hydrolase 37 and glucose-regulated protein 78 in hepatocellular carcinoma.	GeneRIF	0.00977	145	0.0166	0.169	3	100	CDSN,HBA2,HBB

Category	ID	Name	Source	p-value	q-value Bonferroni	q-value FDR B&H	q-value FDR B&Y	Hit Count in Query List	Hit Count in Genome	Hit in Query List
Pubmed	24816252	An atlas of genetic influences on human blood metabolites.	Pubmed	0.0101	149	0.0172	0.175	4	191	ASPG,PKD2L1,GGT1,LIPG
Pubmed	16707424	Identification of candidate alkylator-induced cancer susceptibility genes by whole genome scanning in mice.	Pubmed	0.0103	153	0.0175	0.179	2	35	STRA6,CD276
Pubmed	23867815	HIV gp120 induced gene expression signatures in vaginal epithelial cells.	Pubmed	0.0103	153	0.0175	0.179	2	35	ORM2,MMP7
Pubmed	17573780	RBP2 is an MRG15 complex component and down-regulates intragenic histone H3 lysine 4 methylation.	GeneRIF	0.0109	161	0.0185	0.189	2	36	KDM5A,ACTA1
Pubmed	24680892	AcvR1-mediated BMP signaling in second heart field is required for arterial pole development: implications for myocardial differentiation and regional identity.	Pubmed	0.0115	170	0.0187	0.191	2	37	NPPA,BMP4
Pubmed	1302014	Evolution of the mammalian G protein alpha subunit multigene family.	Pubmed	0.0115	170	0.0187	0.191	2	37	ADA,THRA
Pubmed	22087225	Biochemical and computational analysis of LNX1 interacting proteins.	GeneRIF	0.0121	179	0.0187	0.191	2	38	MUSTN1,PAK6
Pubmed	19574343	Quantitative trait loci predicting circulating sex steroid hormones in men from the NCI-Breast and Prostate Cancer Cohort Consortium (BPC3).	GeneRIF	0.0121	179	0.0187	0.191	2	38	INHBB,CYP11A1
Pubmed	19911011	Mutation of ARHGAP9 in patients with coronary spastic angina.	GeneRIF	0.0121	179	0.0187	0.191	2	38	PREX1,ARHGEF6
Pubmed	14614146	A comprehensive analysis of 22q11 gene expression in the developing and adult brain.	Pubmed	0.0121	179	0.0187	0.191	2	38	RTN4R,GGT1
Pubmed	24997765	Gene co-regulation by Fezf2 selects neurotransmitter identity and connectivity of corticospinal neurons.	Pubmed	0.0127	188	0.0187	0.191	2	39	AKAP12,LIPG
Pubmed	23864651	The identification of novel proteins that interact with the GLP-1 receptor and restrain its activity.	Pubmed	0.0127	188	0.0187	0.191	2	39	NRGN,TSPAN13
Pubmed	21838611	Diagnostic utility of PAX8, TTF-1 and napsin A for discriminating metastatic carcinoma from primary adenocarcinoma of the lung.	GeneRIF	0.0131	194	0.0187	0.191	1	3	NAPSA
Pubmed	21625513	Sour taste responses in mice lacking PKD channels.	GeneRIF	0.0131	194	0.0187	0.191	1	3	PKD2L1
Pubmed	23638949	Novel mutations of SLC26A4 in Chinese patients with nonsyndromic hearing loss.	Pubmed	0.0131	194	0.0187	0.191	1	3	GJB3
Pubmed	21595780	Morphology of the developing muscularis externa in the mouse esophagus.	Pubmed	0.0131	194	0.0187	0.191	1	3	ACTA1
Pubmed	20445565	The association between the PTPN22 1858C>T variant and type 1 diabetes depends on HLA risk and GAD65 autoantibodies.	GeneRIF	0.0131	194	0.0187	0.191	1	3	HLA-DQB1
Pubmed	21252088	Factor VIIa bound to endothelial cell protein C receptor activates protease activated receptor-1 and mediates cell signaling and barrier protection.	GeneRIF	0.0131	194	0.0187	0.191	1	3	PROCR
Pubmed	1863359	Regional mapping of genes encoding human steroidogenic enzymes: P450scc to 15q23-q24, adrenodoxin to 11q22; adrenodoxin reductase to 17q24-q25; and P450c17 to 10q24-q25.	Pubmed	0.0131	194	0.0187	0.191	1	3	CYP11A1
Pubmed	23687431	No association of age-related maculopathy susceptibility protein 2/HtrA serine peptidase 1 or complement factor H polymorphisms with early age-related maculopathy in a Chinese cohort.	GeneRIF	0.0131	194	0.0187	0.191	1	3	HTRA1
Pubmed	11393660	Class II HLA associations with autoantibodies in scleroderma: a highly significant role for HLA-DP	GeneRIF	0.0131	194	0.0187	0.191	1	3	HLA-DQB1
Pubmed	16751419	CD4 T cells play major effector role and CD8 T cells initiating role in spontaneous autoimmune myocarditis of HLA-DQ8 transgenic IAb knockout nonobese diabetic mice.	Pubmed	0.0131	194	0.0187	0.191	1	3	HLA-DQB1
Pubmed	11377425	Interaction of human recombinant alphaA- and alphaB-crystallins with early and late unfolding intermediates of citrate synthase on its thermal denaturation.	Pubmed	0.0131	194	0.0187	0.191	1	3	CRYAA
Pubmed	22974233	Coronin 3 promotes gastric cancer metastasis via the up-regulation of MMP-9 and cathepsin K.	GeneRIF	0.0131	194	0.0187	0.191	1	3	CTSK
Pubmed	11316952	Intracellular distribution of gravin, a PKA and PKC binding protein, in vascular endothelial cells.	Pubmed	0.0131	194	0.0187	0.191	1	3	AKAP12
Pubmed	15180993	An RNA-binding domain in the thyroid hormone receptor enhances transcriptional activation.	GeneRIF	0.0131	194	0.0187	0.191	1	3	THRA
Pubmed	25587035	Elevated adenosine signaling via adenosine A2B receptor induces normal and sickle erythrocyte sphingosine kinase 1 activity.	Pubmed	0.0131	194	0.0187	0.191	1	3	ADA
Pubmed	23243276	Anti-IFN-¿ autoantibodies in adults with disseminated nontuberculous mycobacterial infections are associated with HLA-DRB1*16:02 and HLA-DQB1*05:02 and the reactivation of latent varicella-zoster virus infection.	GeneRIF	0.0131	194	0.0187	0.191	1	3	HLA-DQB1
Pubmed	17884985	A variant of mitochondrial protein LOC387715/ARMS2, not HTRA1, is strongly associated with age-related macular degeneration.	GeneRIF	0.0131	194	0.0187	0.191	1	3	HTRA1
Pubmed	21700325	Gaucher disease glucocerebrosidase and a-synuclein form a bidirectional pathogenic loop in synucleinopathies.	GeneRIF	0.0131	194	0.0187	0.191	1	3	PSAP
Pubmed	19858092	Increased adenosine contributes to penile fibrosis, a dangerous feature of priapism, via A2B adenosine receptor signaling.	GeneRIF	0.0131	194	0.0187	0.191	1	3	ADA
Pubmed	8612804	Human mineralocorticoid receptor interacts with actin under mineralocorticoid ligand modulation.	Pubmed	0.0131	194	0.0187	0.191	1	3	ACTA1
Pubmed	16373339	OAZ regulates bone morphogenetic protein signaling through Smad6 activation.	GeneRIF	0.0131	194	0.0187	0.191	1	3	BMP4
Pubmed	18256758	Expression of peripheral benzodiazepine receptor, PCNA, and caspase-3 in cells of skin melanoma and squamous cell carcinoma.	GeneRIF	0.0131	194	0.0187	0.191	1	3	TSPO
Pubmed	21282512	Induction and maintenance of IL-4 expression are regulated differently by the 3' enhancer in CD4 T cells.	GeneRIF	0.0131	194	0.0187	0.191	1	3	HLA-DQB1
Pubmed	10815801	Evi27 encodes a novel membrane protein with homology to the IL17 receptor.	Pubmed	0.0131	194	0.0187	0.191	1	3	CHDH

Category	ID	Name	Source	p-value	q-value Bonferroni	q-value FDR B&H	q-value FDR B&Y	Hit Count in Query List	Hit Count in Genome	Hit in Query List
Pubmed	18161619	LOC387715/HTRA1 and complement factor H variants in patients with age-related macular degeneration seen at the mayo clinic.	GeneRIF	0.0131	194	0.0187	0.191	1	3	HTRA1
Pubmed	12525172	Polycystin-2 interacts with troponin I, an angiogenesis inhibitor.	GeneRIF	0.0131	194	0.0187	0.191	1	3	PKD2L1
Pubmed	17468179	Identification of a novel role of ZIC3 in regulating cardiac development.	GeneRIF	0.0131	194	0.0187	0.191	1	3	NPPA
Pubmed	15149849	Role of the p70(S6K) pathway in regulating the actin cytoskeleton and cell migration.	GeneRIF	0.0131	194	0.0187	0.191	1	3	ACTA1
Pubmed	19900269	Insights in to the pathogenesis of axial spondyloarthropathy based on gene expression profiles.	GeneRIF	0.0131	194	0.0187	0.191	1	3	SLPI
Pubmed	11136691	Genotype-phenotype correlation in the long-QT syndrome: gene-specific triggers for life-threatening arrhythmias.	GeneRIF	0.0131	194	0.0187	0.191	1	3	KCNH2
Pubmed	24795058	Relationship between AHSP gene expression, ß/a globin mRNA ratio, and clinical severity of the ß-thalassemia patients.	Pubmed	0.0131	194	0.0187	0.191	1	3	HBB
Pubmed	21187074	Brain infarction correlates more closely with acrolein than with reactive oxygen species.	Pubmed	0.0131	194	0.0187	0.191	1	3	TGM2
Pubmed	19389705	Biological activity of nerve growth factor precurso is dependent upon relative levels of its receptors.	GeneRIF	0.0131	194	0.0187	0.191	1	3	NGFR
Pubmed	9285405	Deficient positive selection of CD4 T cells in mice displaying altered repertoires of MHC class II-bound self-peptides.	Pubmed	0.0131	194	0.0187	0.191	1	3	CD74
Pubmed	11672906	Identification of an association between HLA class II alleles and low antibody levels after measles immunization.	GeneRIF	0.0131	194	0.0187	0.191	1	3	HLA-DQB1
Pubmed	22521249	TH2 cytokines increase kallikrein 7 expression and function in patients with atopic dermatitis.	GeneRIF	0.0131	194	0.0187	0.191	1	3	KLK7
Pubmed	10783894	AFX-like Forkhead transcription factors mediate cell-cycle regulation by Ras and PKB through p27kip1.	Pubmed	0.0131	194	0.0187	0.191	1	3	FOXO4
Pubmed	18177426	Patterning of mouse embryonic stem cell-derived pan-mesoderm by Activin A/Nodal and Bmp4 signaling requires Fibroblast Growth Factor activity.	GeneRIF	0.0131	194	0.0187	0.191	1	3	BMP4
Pubmed	16579847	Interleukin-10 haplotypes in Celiac Disease in the Spanish population.	GeneRIF	0.0131	194	0.0187	0.191	1	3	HLA-DQB1
Pubmed	22957785	REG4 contributes to the invasiveness of pancreatic cancer by upregulating MMP-7 and MMP-9.	GeneRIF	0.0131	194	0.0187	0.191	1	3	MMP7
Pubmed	22227562	Increased hyperoxia-induced lung injury in nitric oxide synthase 2 null mice is mediated via angiopoietin 2.	GeneRIF	0.0131	194	0.0187	0.191	1	3	ANGPTL2
Pubmed	17209789	Gingival crevicular fluid matrix metalloproteinase (MMP)-7, extracellular MMP inducer, and tissue inhibitor of MMP-1 levels in periodontal disease.	GeneRIF	0.0131	194	0.0187	0.191	1	3	MMP7
Pubmed	8918254	Molecular cloning of an avian anti-Müllerian hormone homologue.	Pubmed	0.0131	194	0.0187	0.191	1	3	INHBB
Pubmed	17696452	Adenosine deaminase 1 and concentrative nucleoside transporters 2 and 3 regulate adenosine on the apical surface of human airway epithelia: implications for inflammatory lung diseases.	GeneRIF	0.0131	194	0.0187	0.191	1	3	ADA
Pubmed	18165355	Fractalkine deficiency markedly reduces macrophage accumulation and atherosclerotic lesion formation in CCR2-/- mice: evidence for independent chemokine functions in atherogenesis.	GeneRIF	0.0131	194	0.0187	0.191	1	3	CX3CL1
Pubmed	17901247	IL-15 regulates immature B-cell homing in an Ly49D-, IL-12 , and IL-18 dependent manner.	Pubmed	0.0131	194	0.0187	0.191	1	3	CD74
Pubmed	25277308	FPR1 interacts with CFH, HTRA1 and smoking in exudative age-related macular degeneration and polypoidal choroidal vasculopathy.	Pubmed	0.0131	194	0.0187	0.191	1	3	HTRA1
Pubmed	16651263	Functional consequences of interactions between Pax9 and Msx1 genes in normal and abnormal tooth development.	GeneRIF	0.0131	194	0.0187	0.191	1	3	BMP4
Pubmed	10490975	IL-5 mediates eosinophilic rejection of MHC class II-disparate skin allografts in mice.	Pubmed	0.0131	194	0.0187	0.191	1	3	HLA-DQB1
Pubmed	18084828	[The distribution of HLA genes in Russian patients with germ cell tumors].	GeneRIF	0.0131	194	0.0187	0.191	1	3	HLA-DQB1
Pubmed	19635823	Actin dynamics and Rho GTPases regulate the size and formation of parasitophorous vacuoles containing Coxiella burnetii.	GeneRIF	0.0131	194	0.0187	0.191	1	3	ACTA1
Pubmed	21741664	HLA associations with multiple sclerosis in Greece.	GeneRIF	0.0131	194	0.0187	0.191	1	3	HLA-DQB1
Pubmed	3084797	Comparison of three actin-coding sequences in the mouse; evolutionary relationships between the actin genes of warm-blooded vertebrates.	Pubmed	0.0131	194	0.0187	0.191	1	3	ACTA1
Pubmed	16939398	Osteoclastic bone degradation and the role of different cysteine proteinases and matrix metalloproteinases: differences between calvaria and long bone.	Pubmed	0.0131	194	0.0187	0.191	1	3	CTSK
Pubmed	19539001	IFIH1 gene polymorphisms in type 1 diabetes: genetic association analysis and genotype-phenotype correlation in the Belgian population.	GeneRIF	0.0131	194	0.0187	0.191	1	3	HLA-DQB1
Pubmed	15014429	HLA-DQ6 and ingestion of contaminated water: possible gene-environment interaction in an outbreak of Leptospirosis.	GeneRIF	0.0131	194	0.0187	0.191	1	3	HLA-DQB1
Pubmed	15789899	DRB1, DQA1, DQB1 genes in Turkish children with rheumatic fever.	GeneRIF	0.0131	194	0.0187	0.191	1	3	HLA-DQB1
Pubmed	12068310	Oligodendrocyte-myelin glycoprotein is a Nogo receptor ligand that inhibits neurite outgrowth.	GeneRIF	0.0131	194	0.0187	0.191	1	3	RTN4R
Pubmed	19022775	RECK forms cowbell-shaped dimers and inhibits matrix metalloproteinase-catalyzed cleavage of fibronectin.	GeneRIF	0.0131	194	0.0187	0.191	1	3	MMP7
Pubmed	23220946	Collectin-11/MASP complex formation triggers activation of the lectin complement pathway--the fifth lectin pathway initiation complex.	Pubmed	0.0131	194	0.0187	0.191	1	3	MASP1
Pubmed	11376336	Structure of a human insulin peptide-HLA-DQ8 complex and susceptibility to type 1 diabetes.	Pubmed	0.0131	194	0.0187	0.191	1	3	HLA-DQB1
[truncated: 1,096,334 more chars]
